# Ring finger protein 126 promotes breast cancer metastasis and serves as a potential target to improve the therapeutic sensitivity of ATR inhibitors

**DOI:** 10.1186/s13058-022-01586-0

**Published:** 2022-12-20

**Authors:** You Pan, Yuchao Yang, Rong Huang, Huawei Yang, Qinghua Huang, Yinan Ji, Jingxing Dai, Kun Qiao, Wei Tang, Longgui Xie, Ming Yin, Jun Ouyang, Shipeng Ning, Danke Su

**Affiliations:** 1 Department of Breast Surgery, Key Laboratory of Breast Cancer Diagnosis and Treatment Research of Guangxi Department of Education, Guangxi Medical University Cancer Hospital, Nanning, 530000 China; 2 Guangdong Provincial Key Laboratory of Medical Biomechanics & Nation Key Discipline of Human Anatomy, School of Basic Medical Science, Southern Medical University, Guangzhou, 510515 China; 3 Department of Radiology, Guangxi Medical University Cancer Hospital, Nanning, 530000 China; 4 Department of Imaging, Nanfang Hospital, Southern Medical University, Guangzhou, 510515 China; 5 Department of Breast Surgery, Harbin Medical University Cancer Hospital, Harbin, 150000 China

**Keywords:** Breast cancer, RNF126, Metastasis, CDK2-mediated, ATR inhibitor

## Abstract

**Background/aims:**

This study explores the relationship between the E3 ubiquitin ligase Ring finger protein 126 (RNF126) and early breast cancer metastasis and tests the hypothesis that RNF126 determines the efficacy of inhibitors targeting Ataxia telangiectasia mutated and Rad3-related kinase (ATR).

**Methods:**

Various metastasis-related genes were identified by univariable Cox proportional hazards regression analysis based on the GSE11121 dataset. The RNF126-related network modules were identified by WGCNA, whereas cell viability, invasion, and migration assays were performed to evaluate the biological characteristics of breast cancer cells with or without RNF126 knockdown. MTT, immunoblotting, immunofluorescence, and DNA fiber assays were conducted to determine the efficiency of ATR inhibitor in cells with or without RNF126 knockdown.

**Results:**

RNF126 was associated with early breast cancer metastasis. RNF126 promoted breast cancer cell proliferation, growth, migration, and invasion. ATR inhibitors were more effective at killing breast cancer cells with intact RNF126 due to replication stress compared with the corresponding cells with RNF126 knockdown. Cyclin-dependent kinase 2 (CDK2) was involved in regulating replication stress in breast cancer cells with intact RNF126.

**Conclusion:**

A high level of expression of RNF126 in early breast cancer patients without lymph node metastases may indicate a high-risk type of metastatic disease, possibly due to RNF126, which may increase breast cancer cell proliferation and invasion. RNF126-expressing breast cancer cells exhibit CDK2-mediated replication stress that makes them potential targets for ATR inhibitors.

**Supplementary Information:**

The online version contains supplementary material available at 10.1186/s13058-022-01586-0.

## Introduction

Breast cancer is the most common cancer and the leading cause of cancer mortality among women [1]. With improved screening, more and more breast cancer patients are being detected early. Although there are many strategies for early breast cancer, recurrence and metastasis are still the primary causes of breast cancer treatment failure. Metastasis is no longer believed to involve a linear cascade of events, but rather multiple signaling pathways, such as those related to the cell cycle [2] and tumor microenvironment [3]. According to clinical guidelines, ionizing radiation and chemotherapies are the major choices for postoperative adjuvant therapies in early breast cancer. Their main mechanism involves DNA damage [4]. However, partial breast cancer therapy may result in endogenous factors antagonizing of the DNA damage caused by ionizing radiation or chemotherapy due to an upregulation of the DNA damage response, which comprises cell cycle checkpoints and DNA repair [5]. Cell cycle checkpoint kinase inhibitors can sensitize breast cancer cells to radiotherapy or chemotherapy. Unfortunately, it seems that these therapies cannot effectively kill breast cancer cells by themselves, and there is an absence of appropriate biomarkers [6, 7].

Ring finger protein 126 (RNF126) is an E3 ubiquitin ligase that is involved in diverse biological processes, including cell proliferation [8], DNA repair [9], and the cell cycle [10]. Overexpression of RNF126 can promote proliferation in the tongue [11] and gastric cancer [8] cells. In addition, RNF126 facilitates DNA double-stranded break repair by promoting homologous recombination [12] and nonhomologous end joining [13]. However, the proliferation regulatory ability of RNF126 in breast cancer metastasis has not yet been evaluated.

Ataxia telangiectasia mutated and Rad3-related kinase (ATR), a cell cycle checkpoint kinase, can be activated in response to replication protein A-coated single-stranded DNA caused by endogenous or exogenous factors [14]. ATR-phosphorylated CHEK1 results in cell cycle arrest at the G2/M checkpoint, allowing for DNA repair. ATR plays a major role in preventing cells with incomplete DNA replication from undergoing mitosis after exposure to DNA-damaging agents, such as chemotherapeutic drugs or ionizing radiation [15]. Suppression of ATR signaling increases firing of dormant origins, leading to massive fork collapse and DNA breakage [16]. Accordingly, ATR inhibitors have been developed and are currently being used in preclinical and clinical studies. In addition, ATR inhibitors paired with genotoxic chemotherapeutics or radiotherapy have synergistic activity in numerous cancer cells [17–19]. However, ATR inhibitor monotherapy in cancer cells has rarely been reported. Moreover, ATR activity itself may not be sufficient as an effective target for ATR inhibitors [20, 21].

Here, we identify a specific metastasis-related gene expression signature from a microarray dataset comprising early breast cancer patients without lymphatic metastasis (GSE11121) in the Gene Expression Omnibus (GEO) [22]. The dataset showed that RNF126 is associated with breast cancer metastasis. We applied the signature to The Cancer Genome Atlas (TCGA) to determine RNF126 was highly expressed in the tumor tissues and constructed RNF126-related network modules through weighted gene coexpression network analysis (WGCNA). Gene Set Enrichment Analysis (GSEA) also implied that the cell cycle pathway is enriched in patients overexpressing RNF126. Additionally, we characterized the molecular function of RNF126, examining its proliferation ability in both in vitro and in vivo experiments. Treatment with ATR inhibitors increased cell death by triggering abnormal origin firing in breast cancer cells with higher RNF126 expression. CDK2 may mediate the killing effect of ATR inhibitors on RNF126 high-expression breast cancer cells. In brief, our results suggest that higher expression of RNF126 may accelerate breast cancer metastasis and that RNF126 may be an effective biological target for ATR inhibitors.

## Materials and methods

### Patients and datasets

Microarray data of 200 breast cancer samples (GSE11121) and the survival information were downloaded from the GEO database (https://www.ncbi.nlm.nih.gov/geo/). RNA-seq data of 88 breast cancer samples without lymph node metastasis, including 44 cancer samples and 44 para-cancer samples, and their clinicopathological information were downloaded from the TCGA database (https://portal.gdc.cancer.gov/projects/TCGA-BRCA).

### Identification of metastasis-related genes

The GSE11121 samples were processed using the R package WGCNA for removing outliers. After clustering, 199 breast cancer samples were included, GSM282518 was excluded. A univariable Cox proportional hazards regression model was used to select metastasis-related genes with |coefficient|> 0.50, *P* value < 0.05. Metastasis-related differentially expressed genes were analyzed by using Gene Ontology (GO), Kyoto Encyclopedia of Genes and Genomes (KEGG) analyses, and the GSEA with the clusterProfiler package of R. The KEGG pathways and GO terms regarding cellular component, molecular function, and biological process with *P* values and false discovery rates less than 0.05 were considered statistically significant.

Then, WGCNA was performed to identify RNF126-related signal pathways. The similarity matrix was transformed into an adjacency matrix with a network type of signed and a soft threshold of *β* = 5 and then transformed into a topological matrix with the topological overlap measure (TOM) describing the degree of association between genes. 1-TOM was used as the distance to cluster the genes, and then the dynamic pruning tree was built to identify the modules. We identified 5 modules by setting the merging threshold function at 0.20. The enrichment *P* values for the GSEA were based on 1000 permutations and adjusted by calculating the false discovery rates. The GSEA results were visualized using the R package enrichplot.

### Cell culture and transfection

Human breast cancer cell lines (MCF7 and MDA-MB-231) were cultured in DMEM medium supplemented with 10% fetal bovine serum (FBS), and maintained at 37 °C in a humidified incubator with 5% CO_2_. All DNA-plasmid transfections were performed using Lipofectamine 2000 according to the manufacturer's recommendations (Invitrogen). The sequences of shRNA used were as follows: shRNF126#1 5′-TGCATGGTTTGTGGCGGAAGA-3′; shRNF126#2 5′-CAACGAGAACGCCACATGGTC-3′.

### Quantitative real-time polymerase chain reaction (qRT-PCR)

The total RNA was isolated with the TRIzol reagent (Invitrogen). Novogene (Beijing, China) completed the cDNA library construction. Experiments were carried out in triplicate for each data point. Reactions were performed using SYBR Green mix (Roche) and a MyiQ real-time PCR detection system (Bio-Rad). Relative mRNA levels were calculated using the comparative Ct method (ΔCt).

GAPDH forward primers: 5′-CTCTGCTCCTCCTGTTCGAC-3′; reverse primers: 5′-TTAAAAGCAGCCCTGGTGAC-3′.

RNF126 forward primers: 5′-TATCGAGGAGCTTCCGGAAGAGA-3′; reverse primers: 5′-AAAGCAAACTGTCCGTAGCCCT-3′.

CDK1 forward primers:5′-GGATGTGCTTATGCAGGATTCC-3′; reverse primers: 5′-CATGTACTGACCAGGAGGGATAG-3′.

CDK2 forward primers: 5′-TATTAACACAGAGGGGGCCA -3′; reverse primers: 5′-AAAGATCCGGAAGAGCTGGT-3′.

CDK5 forward primers:5′-GGAAGGCACCTACGGAACTG-3′; reverse primers: 5′-GGCACACCCTCATCATCGT-3′.

### Scratch wound assay

The cells were inoculated in a 6-well plate, scraped through each hole with the tip of a sterile 10 μL pipette and washed with phosphate-buffered saline to remove any debris. After 24 h, the cells migrated to the empty space.

### Cell invasion and migration assays

Approximately 2 × 10^4^ cells in 300 μL DMEM medium without FBS were seeded into upper transwell chamber (8 μm pore size) to evaluate cell migration. The lower chamber was filled with 800 μL DMEM medium supplemented with 10% FBS. After 24 h, the cells attached to the lower surface of the membrane were fixed with 4% formaldehyde, stained with 0.5% crystal violet, and then counted under a microscope in five random fields. Invasion assays were performed under the same conditions as the migration assays, but in matrigel-coated transwell (Corning, NY, USA) inserts.

### Cell viability and calculation of half-maximal inhibitory concentration (IC50)

The cells were seeded in 96-well plates in 100 μL DMEM medium containing 10% FBS, at a density of 2 × 10^3^ cells per well. The cells were exposed to various doses of inhibitors and assayed for viability at indicated times, using the MTT according to the manufacturer's instructions. In brief, MTT (20 mL of 5 mg/mL) was added to each well and cells were incubated for a further 3.5 h in an incubator. MTT solvent was added after removing the medium and the cells in plates were agitated on an orbital shaker for 15 min. The absorbance was read at 590 nm with a reference filter of 620 nm. The absorbance values were normalized with respect to those of untreated control cells. The IC50 was calculated using nonlinear regression analysis in GraphPad Prism 6.0.

### In vivo studies

MCF7 and MDA-MB-231 cells (1 × 10^7^) in 150 μL PBS were subcutaneously injected into the right flank of female nude mice. Tumor volume was measured by caliper and calculated as length × width^2^/2. When tumor volume grew up to 50–100 mm^3^, the mice were randomly divided into two groups (five mice per group), and then treated with PBS daily, AZD6738 (50 mg/kg, oral, daily). Tumor volume was measured every 3 days. In assays to measure formation of metastases, 10^7^ breast cancer cells were injected into tail veins of mice. The number of metastases was assessed in 3 or 6 weeks, respectively. All the animal experiments were carried out with the approval of the guidelines of Guangxi Medical University Cancer Hospital.

### Immunohistochemistry

The formalin-fixed mouse tumor, liver, or lung tissues were embedded with paraffin. The treated tissues were sectioned (3 μm) and stained with the hematoxylin and eosin (H&E). H&E-stained liver or lung sections were imaged using a microscope (Olympus). For immunostaining, slides were heated to 60 °C and then deparaffinized in xylene. The slides were rehydrated in descending alcohol concentrations. Antigen retrieval was performed by incubating slides in a retrieval solution of citrate buffer. Hydrogen peroxide was added to block endogenous peroxidase activity to decrease unwanted background staining. Primary antibody (RNF126, ab234812, Abcam) was added at 1:100 dilution. The substitution of primary antibody performed negative controls with phosphate-buffered saline (PBS). To guarantee consistent IHC evaluation, slides from a reference tumor previously determined as positive were included in each staining procedure. Evaluations of staining reactions were performed in accordance with the immunoreactive score (IRS) proposed by Remmele and Stegner: IRS = staining intensity (SI) X percentage of positive cells (PP). Staining intensity was marked as nongranulated (0); low grade (light yellow; 1); moderate (brownish yellow; 2); or strong (reddish brown; 3). The PP was scored as negative (< 5%; 0); weak (5–10%; 1); moderate (11–50%; 2); strong (51–80%; 3); or very strong (> 81%; 4). Specimens scoring beyond 3 were considered positive overexpression. Slides were studied with the microscopic (Olympus).

### Immunoblotting

Cellular extracts were prepared by resuspending cells in radio immunoprecipitation assay (RIPA) buffer. After protein samples were separated by 5%, 12%, or 15% SDS-PAGE, they were transferred onto polyvinylidene fluoride (PVDF) membranes (Millipore, Burlington, MA). Around 5% BSA was used to incubate the PVDF membrane for 1 h at room temperature, and then at 4 °C overnight with antibodies specific to glyceraldehyde-3-phosphate dehydrogenase (GAPDH) (AP0066, 1:10,000; Bioworld), RNF126 (C-1 SC-376005, 1:100; Santa Cruz Biotechnology); ATR (C-1 SC-515173, 1:100; Santa Cruz Biotechnology); p-ATR (S428 AB178407, 1:100; Abcam); γ-H2AX (Ser139 SC-517348, 1:100; Santa Cruz Biotechnology); p-RPA2 (S4/S8; rabbit polyclonal, BL647, 1: 1000; Bethyl Laboratories); CHEK1 (AB32531, 1:100; Abcam); CDK1 (AB265590, 1:500; Abcam); CDK2 (610,146, 1:200; BD Biosciences); CDK5 (AB40773, 1:500; Abcam); Cleaved PARP (AB4830, 1:200; Abcam). After washing in Tris-buffered saline with 0.1% Tween 20 (TBST) for three times (10 min each time), the PVDF membranes were incubated with secondary antibodies (Goat anti-mouse IgG-horseradish peroxidase (HRP)–conjugated (#7076S, 1:1,000; Cell Signaling Technology), goat anti-rabbit IgG-HRP–conjugated (#7074S, 1:1000; Cell Signaling Technology), and donkey anti-goat IgG-HRP–conjugated (A2216, 1:1,000; Santa Cruz Biotechnology)) for 1 h at room temperature. By using enhanced chemiluminescence blotting reagents, proteins were detected after three TBST washes (FUDE Biological, Hangzhou, China). Signal intensity was assessed by using a Tanon-5500 chemiluminescence detection system (Tanon Science & Technology Ltd, Shanghai, China).

### Immunofluorescence analysis

Cells growing on slides were fixed directly in 3–4% paraformaldehyde. Cells were extracted for 5 min on ice with 0.5% Triton X-100 in cytoskeletal (CSK) buffer (10 mmol/L PIPES, 300 mmol/L sucrose, 100 mmol/L NaCl, 3 mmol/L MgCl_2_; pH = 6.8) supplemented with 1 mmol/L phenylmethylsulfonyl fluoride, 0.5 mmol/L sodium vanadate, and proteasome inhibitor for 10 min at 4 °C. Then, extracted cells were fixed with 3–4% paraformaldehyde. The cells were permeabilized for 10 min with PBS containing 0.5% Triton X-100 for 15 min at room temperature, followed by blocking with 1% BSA, and then incubated with primary antibodies specific to CDC45 (H-300 clone, SC20685, 1:50; Santa Cruz Biotechnology). The bound antibodies were revealed with chicken anti-rabbit IgG Alexa Fluor 488. Slides were viewed at 1,000 magnifications with a NIKON 90i fluorescence microscope (photometric cooled mono CCD camera, NIKON, Tokyo, Japan).

### DNA fiber assays

DNA fiber assays were performed as published with some modifications [23]. Cells were pulse-labeled with 50 mmol/L IdU (Sigma-Aldrich #I7125) for 40 min and then pulse-labeled with 200 mmol/L CldU (Sigma–Aldrich #C6891) for 40 min in the presence or absence of ATR inhibitor. At the end of the CldU pulse, cell suspensions (2.5 mL) were mixed with 7.5 mL of lysis buffer (0.5% SDS, 200 mmol/L Tris–HCl (pH 7.4), 50 mmol/L EDTA). Each mixture was dropped on the top of an uncoated regular glass slide. Slides were inclined at 25° to spread the suspension on the glass. Once dried, DNA spreads were fixed by incubation for 10 min in a 3:1 solution of methanol-acetic acid. The slides were dried and placed in precooled 70% ethanol at 4 °C for at least 1 h. DNA was denatured with 2.5 mol/L HCl for 30 min at 37 °C. The slides were blocked in 1% BSA in PBS for 30 min at room temperature and then incubated with mouse anti-BrdU antibody (BD Biosciences #347,580) at a 1:200 dilution and rat anti-CldU antibody (Abcam #ab6326) at a 1:400 dilution. The slides were incubated with secondary fluorescent antibodies [goat anti-mouse IgG (H + L) Alexa Fluor 594 secondary antibody (A-11032, 1:400; Thermo Fisher Scientific); or chicken anti-rabbit IgG (H + L) Alexa Fluor 488 secondary antibody (A-21441, 1:400); Thermo Fisher Scientific]. Replication fibers were viewed at 1000 magnifications on a NIKON 90i fluorescence microscope (photometric cooled mono CCD camera, NIKON, Tokyo, Japan). Signals were measured using ImageJ software (NCI/NIH), with some modifications made specifically to measure DNA fibers.

### Statistical analysis

Statistical analyses were undertaken using the statistical software package, R version 4.0.4. Univariate Cox regression analysis was performed to select genes with *P* < 0.05 for metastasis. Univariate survival analysis was performed by Kaplan–Meier survival analysis with the log-rank test. Paired *t*-test, one-way or two-way ANOVA analysis of variance were used to determine the significance of differences between groups. Continuous variables were expressed as mean ± SD. In all the statistical analyses, *P* < 0.05 was considered as statistically significant.

## Results

### RNF126 expression is related to breast cancer metastasis and the cell cycle pathway

In the GSE11121, 1568 genes were identified by univariable Cox proportional hazards regression analysis: 699 genes were negatively, and 869 genes were positively associated with a statistically significant metastasis-free survival benefit (Additional files 1 and 2: Fig. S1 and Table S1). Gene Ontology (GO) and Kyoto Encyclopedia of Genes and Genomes (KEGG) functional enrichment analysis showed that 1568 differentially expressed genes were significantly associated with 908 GO terms and 32 KEGG pathways (Details in Additional file 2: Tables S2 and S3). The top 10 GO terms and KEGG pathways are shown in Additional file 1: Figs. S2 and S3.

**Fig. 1:**
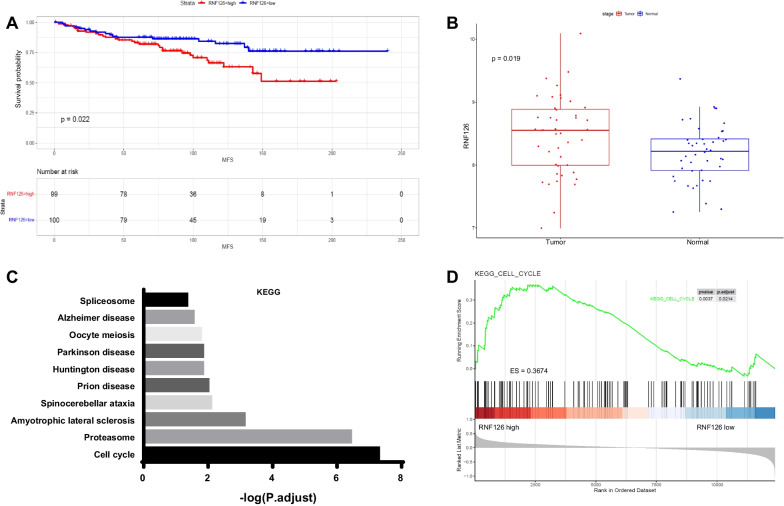
Expression of RNF126 related to breast cancer metastasis and cell cycle pathway. **A** Metastasis free survival analysis in patients with high RNF126 expression and low RNF126 expression in the GSE11121 cohorts (*n* = 199, *P* = 0.022). **B** RNF126 gene expression between tumor and paired normal tissues of 44 early-stage (T1–T3, N0, M0) breast cancer patients in TCGA database (*P* = 0.019). **C** KEGG analysis for the genes in the turquoise modules. **D** Gene set enrichment analysis of cell cycle pathway between RNF126-high and RNF126-low subgroups in the GSE11121 (*P* = 0.0037, ES = 0.3674)

RNF126 is a cell proliferation gene, and we found that cohort patients with a higher level of RNF126 had shorter metastasis-free survival (Fig. 1A). Given the lack of normal paired tissues in GSE11121 dataset, we analyzed the expression of RNF126 in 44 early-stage (T1–T3, N0, M0) breast cancer patients in TCGA to ascertain whether RNF126 was higher expression in tumor tissues. The results showed that RNF126 was upregulated in tumor samples compared with normal samples (Fig. 1B).

To obtain the RNF126-related signaling pathways, WGCNA analysis was applied to the remaining genes (*n* = 1567). The log10(*k*) of the node with *K* was negatively correlated with the log10(*P*(*k*)), and the correlation coefficient was greater than 0.85. The optimal soft-thresholding power was 5 based on the scale-free network. Five modules were identified based on the optimal soft-thresholding power. According to the Pearson correlation coefficient between RNF126 expression and each module, turquoise and gray modules were positively correlated with a higher RNF126 level, whereas yellow, blue, and brown modules were positively correlated with a lower RNF126 level; the genes in all of these modules were selected for further analysis (Additional file 1: Fig. S4). The top significantly enriched GO and KEGG pathways for the genes of the turquoise modules are shown in Additional file 1: Fig. S5 and Fig. 1C, respectively (Details in Additional file 2: Tables S4 and S5). The gray modules could not enrich GO and KEGG pathways. The enriched GO terms of the blue, brown, and yellow modules are shown in Additional file 2: Tables S6, S7, and S8; among these three modules, only yellow modules could enrich KEGG pathways (Details in Additional file 2: Table S9). Next, GSEA showed that samples with higher RNF126 levels were enriched in cell cycle pathways (Fig. 1D). Thus, high RNF126 expression appears to be associated with breast cancer metastasis and is enriched in cell cycle pathways.

### RNF126 promotes breast cancer cell proliferation, cell growth, migration, and invasion

**Fig. 2:**
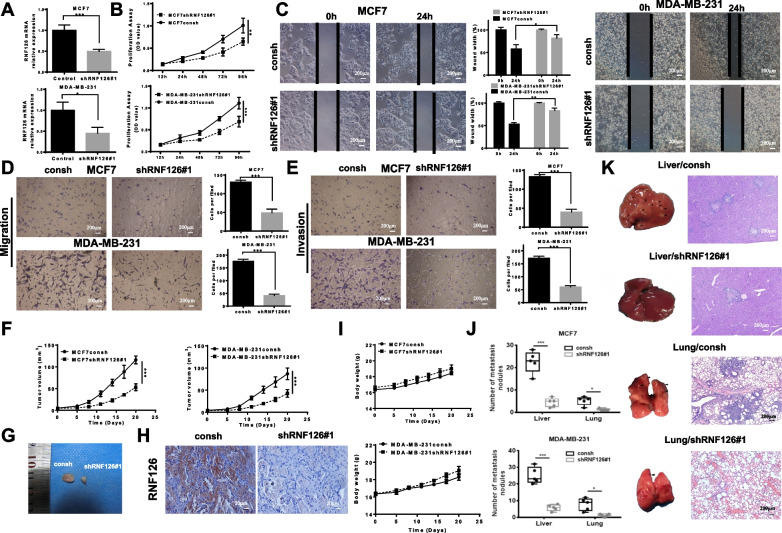
RNF126 promotes cell proliferation, cell growth, migration, and invasion of breast cancer cells. **A** The relative expression levels of RNF126 mRNA in MCF7 (upper panel) and MDA-MB-231 (down panel) cells with or without RNF126 knockdown (Paired *t*-test). **B** Cell proliferation assay shows the effects of RNF126 on MCF7 (upper panel) and MDA-MB-231 (down panel) cells at indicated time points (Two-way ANOVA). **C** Wound healing assay was performed to measure the effect of RNF126 knockdown on the migration of MCF7 (left panel) and MDA-MB-231 (right panel) cells (Paired *t*-test, scale bar, 200 μm). The migration **D** and invasion **E** ability of MCF7 (upper panel) and MDA-MB-231 (down panel) cells with or without RNF126 knockdown was measured by transwell assay (Paired *t*-test, scale bar, 200 μm). **F** Tumor growth curves of the mice (*n* = 5 per group) with or without RNF126 knockdown (MCF7, left panel; MDA-MB-231, right panel). The tumor volume was monitored every 3 days (Two-way ANOVA). **G** Diagram of the tumor on day 21. **H** Schematic representation of tumor with or without RNF126 knockdown by IHC. **I** Weight of tumors derived from mice in each group (MCF7, upper panel; MDA-MB-231, down panel). **J** Number of metastases of liver and lung in MCF7 (upper panel) and MDA-MB-231 (down panel) with or without RNF126 knockdown in mice (Two-way ANOVA). **K** Schematic diagram of metastatic nodules. Histological observation of metastatic organs includes of lung, liver visualized using H&E staining. Black arrow: metastasis nodules. Data are presented as mean ± SD. *P < 0.05, **P < 0.01, and ***P < 0.001. All presented results are from three independent experiments

RNF126 can promote the cell proliferation of tongue cancer and gastric cancer cells [8, 11]. To investigate the functional effects of RNF126 on breast cancer cells, RNF126 expression in MCF7 and MDA-MB-231 cells was suppressed by two RNF126 shRNAs. The efficient knockdown of RNF126 was confirmed by qRT-PCR (Fig. 2A). Cell growth was significantly reduced in both RNF126 knockdown cell lines compared to control cells (Fig. 2B and Additional file 1: Fig. S6). Subsequently, we used a wound-healing assay to investigate the effect of RNF126 knockdown on the migration capabilities of MCF7 and MDA-MB-231 cells. Compared with their respective control cells, shRNF126 cells displayed lower migration abilities (Fig. 2C). A transwell migration assay obtained the same results (Fig. 2D). Next, we examined the effect of RNF126 on the invasion abilities of MCF7 and MDA-MB-231 cells. Both shRNF126 cell lines exhibited decreased invasion compared with their respective control cells (Fig. 2E). Mice bearing a subcutaneous xenograft derived from MCF7 or MDA-MB-231 cells with or without RNF126 knockdown showed significantly reduced tumor growth after RNF126 knockdown (Fig. 2F–H). However, two groups had no significant difference in body weight (Fig. 2I). In assays to measure formation of metastases, MCF7 or MDA-MB-231 cells with or without RNF126 knockdown were injected into the tail veins of mice, and we measured formation of liver and pulmonary metastases. The results showed knockdown of RNF126 reduced the ability of these cells to form liver and lung metastases in mice (Fig. 2J and K). Thus, we conclude that RNF126 promotes breast cancer cell proliferation, cell growth, migration, and invasion.

### ATR inhibitors are more effective at killing breast cancer cells with intact RNF126 than the corresponding cells with RNF126 knockdown

**Fig. 3:**
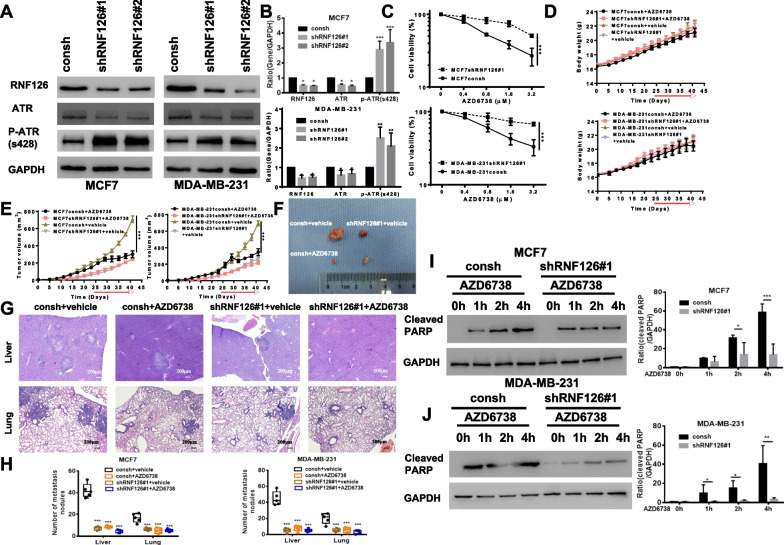
ATR inhibitors are more effective in killing breast cancer cells with completed RNF126 rather than corresponding cells with RNF126 knockdown. **A** Western blot analysis showed that RNF126 knockdown promoted the expression of p-ATR (S428). GAPDH was used as a loading control. **B** Band intensities were quantified and are presented as bar graphs (Two-way ANOVA, MCF7, upper panel; MDA-MB-231, down panel). **C** MTT assay showed the effect of the ATR inhibitor (AZD6788) on MCF7 (upper panel) and MDA-MB-231 (down panel) cell viability. Cell viability was expressed as the percentage of viable cells in treated wells relative to the percentage of viable cells in control wells (100% viability). Cells were treated with various concentrations of AZD6788 for 72 h (*n* = 3, Two-way ANOVA). **D** Tumor bearing mice were treated with PBS, AZD6738 (50 mg/kg, daily) for 21 days, respectively, Body weight curves of the mice (*n* = 5 each group). **E** Tumor growth curves of the mice during the treatment period (*n* = 5 each group, Two-way ANOVA). **F** Schematic diagram of tumor on day 42. **G** Histological observation of metastatic organs includes of lung, liver visualized using H&E staining. **H** Number of metastases of liver and lung in each group (Two-way ANOVA, MCF7, left panel; MDA-MB-231, right panel). **I**, **J** Western blot showed cleaved PARP expression in MCF7 (**I**) and MDA-MB-231 (**J**) cells with or without RNF126 depletion. Cells with or without RNF126 shRNA#1 infection were treated with AZD6738 (1 μM) at indicated time points. Band intensities of cleaved PARP were quantified and are presented as bar graphs (Two-way ANOVA). Data are presented as mean ± SD. *P < 0.05, **P < 0.01, and ***P < 0.001. All presented results are from three independent experiments

ATR is an important regulator of the cell cycle. RNF126 knockdown decreased the expression of CHEK1, a downstream factor of ATR [24]. We confirmed these results (Additional file 1: Fig. S7). Moreover, we observed that RNF126 knockdown increased the expression of p-ATR (S428), a biomarker of ATR function [25] (Fig. 3A and B). Thus, we hypothesized that breast cancer cells with RNF126 knockdown could be more sensitive to ATR inhibitors. Interestingly, our results showed that breast cancer cells with RNF126 knockdown were less sensitive to ATR inhibitors than the cells with intact RNF126 (Fig. 3C). We investigated whether AZD6738 would exhibit anti-tumor efficacy in xenografts with or without RNF126 knockdown model. The mice were treated with AZD6738 (50 mg/Kg, oral, daily) and PBS for 21 days. No significant difference in body weight was observed between PBS group and AZD6738 group (Fig. 3D). However, as shown in Fig. 3E and F, the tumor growth of AZD6738 treated group was significantly inhibited compared with PBS groups, when RNF126 was intact. In addition, AZD6738 reduced the number of liver and lung metastases nodules in these cells with RNF126 intact (Fig. 3G, H). To determine whether ATR inhibitors could increase cell apoptosis, we measured caspase 3-mediated cleavage of PARP (Cleaved PARP), a biomarker of apoptosis. Cleaved PARP was increased in ATR inhibitor-treated MCF7 (Fig. 3I) and MDA-MB-231 (Fig. 3J) cells, but a significant increase was not found under the same conditions in the corresponding cells with RNF126 depletion. These results suggest that ATR inhibition suppresses the proliferation of breast cancer cells expressing higher levels of RNF126 rather than the corresponding cells with lower levels of RNF126. However, CHEK1 and ATR inhibitors have been reported to have synergistic lethal effects [26], which were also confirmed (Additional file 1: Figs. S8A and S9A). Nonetheless, our results showed that RNF126-depleted breast cancer cells were not sensitive to CHEK1 and ATR inhibitors (Additional file 1: Figs. S8B and S9B). Accordingly, it seems that another factor may influence the sensitivity of breast cells with intact RNF126 to these inhibitors.

### Knockdown of RNF126 diminished replication stress

**Fig. 4:**
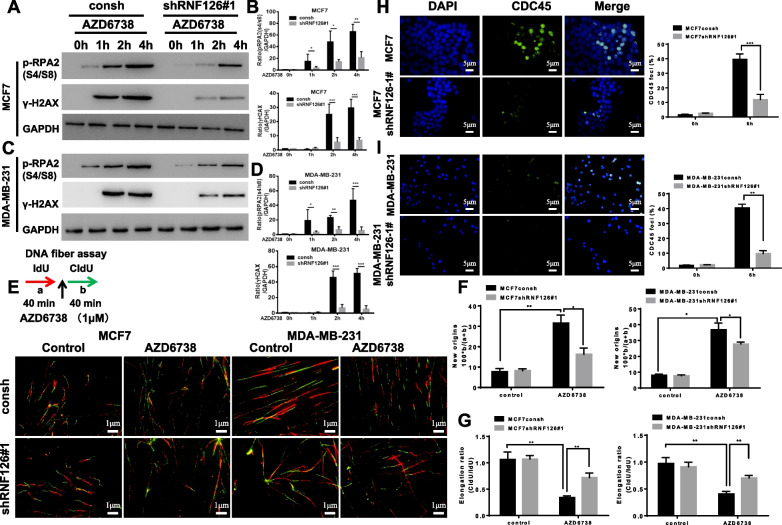
Knockdown of RNF126 leads to decreased replication stress. **A**, **C** Western blot analysis showed AZD6738 (1 μM) led to a greater increase in levels of p-RPA2 and γ-H2AX in parental cells, compared to cells with RNF126 knockdown by RNF126 shRNA#1 (**A**, MCF7 cells; **C**, MDA-MB-231). **B**, **D** Band intensities were quantified and are presented as bar graphs (Two-way ANOVA, p-RPA2, upper panel; γ-H2AX, down panel). **E** Schematic of DNA fiber analysis. a, red tracks, IdU; b, green tracks, CldU, scale bar,1 μm. **F** AZD6738 (1 μM) increased the rate of replication initiation, particularly in cells with intact RNF126, compared with cells depleted of RNF126. The frequency of new origins was calculated as the number of green signals (b) divided by the total of green plus red signals (a + b) (One-way ANOVA, MCF7, left panel; MDA-MB-231, right panel) **G** AZD6738 induced a greater decrease in replication fork speeds in MCF7 (left panel) and MDA-MB-231 (right panel) cells compared with corresponding cells with RNF126 knockdown. The CIdu/Idu ratio was used to determine elongation (One-way ANOVA). **H**, **I** The proportion of cells with foci of CDC45 in MCF7 (**H**) and MDA-MB-231 (**I**) cells with or without RNF126 knockdown. Cells were treated with AZD6738 (1 μM) for the indicated times and then subjected to immunofluorescence staining. Representative foci of CDC45 are indicated (Paired *t*-test, scale bar, 5 μm). Data are presented as mean ± SD. *P < 0.05, **P < 0.01, and ***P < 0.001. All presented results are from three independent experiments

Both phosphorylated RPA2 (p-RPA2) and γ-H2AX are major markers of replication stress [27, 28]. In order to identify the cause of RNF126 knockdown cells' insensitivity to ATR inhibitors, we measured the range of replication stress following ATR inhibition in cells with and without RNF126 knockdown. AZD6738 treatment led to a greater increase in p-RPA2 and γ-H2AX protein levels in parental MCF7 cells compared with MCF7 cells with RNF126 knockdown (Fig. 4A, B). Similar results were observed in MDA-MB-231 cells (Fig. 4C, D) and for the second RNF126, shRNA#2. RNF126 knockdown by RNF126 shRNA#2 abrogated ATR inhibition-induced replication stress in both MCF7 and MDA-MB-231 cells (Additional file 1: Fig. S10). Thus, ATR helps to suppress oncogene-induced replication stress by inhibiting excess origin firing [29], and inhibition of ATR slows down replication fork progression [30].

We next used a DNA fiber assay to determine whether ATR inhibitor-treated cells with or without RNF126 knockdown treatment altered the initiation of DNA replication (Fig. 4E). The percentage of new origins increased when cells were treated with AZD6738 in both parental MCF7 cells and MCF7 cells with RNF126 knockdown (Fig. 4F), whereas the magnitude of the increase was greater in parental cells than in cells with RNF126 knockdown. A similar result was seen in MDA-MB-231 cells (Fig. 4F). In addition, the elongation ratio of the replication fork, an alteration resulting in replication initiation, was decreased when ATR activity was inhibited, particularly in MCF7 and MDA-MB-231 cells without RNF126 knockdown (Fig. 4G). CDC45 is an essential protein for controlling replication initiation that limits the initial firing of replication origins rather than elongation processes [31]. We used immunofluorescence staining to analyze foci of CDC45 in response to AZD6738. A more marked increase in the proportion of cells with CDC45 foci was observed in MCF7 (Fig. 4H) and MDA-MB-231 (Fig. 4I) cells than in their corresponding cells depleted of RNF126. Thus, these results suggest that RNF126 also contributes to oncogene-induced replication stress and that knockdown of RNF126 reduces the sensitivity of ATR inhibitors by lowering replication stress.

### Replication stress in breast cancer cells with a higher level of RNF126 is mediated by CDK2

**Fig. 5:**
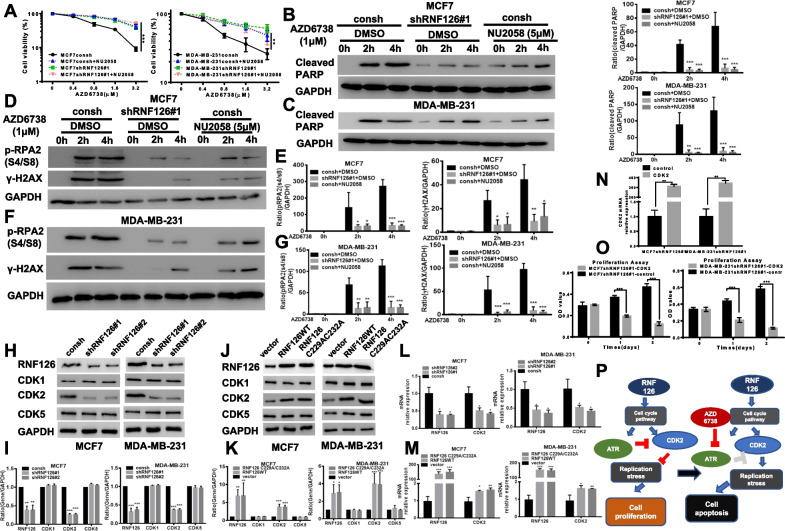
Replication stress in breast cancer cells with a higher level of RNF126 is mediated by CDK2. **A** MTT assay showed the effect of co-treatment NU2058 (5 μM) and various concentrations AZD6738 on MCF7 (left panel) and MDA-MB-231 (right panel) cell proliferation (Two-way ANOVA). **B**, **C** The expression of cleave-PARP in MCF7 (**B**) and MDA-MB-231 (**C**) cells with or without RNF126 depletion. Cells treated with AZD6738 alone or combined with NU2058 (5 μM) at indicated time points. The indicated proteins were analyzed by western blot. Band intensities were quantified and are presented as bar graphs (Two-way ANOVA). **D**, **F** Western blot analysis showed NU2058 (5 μM) decreased the levels of p-RPA2 and γ-H2AX in parental cells caused by AZD6738 (**D**, MCF7; **F**, MDA-MB-231). **E**, **G** Band intensities of p-RPA2 (left panel) and γ-H2AX (right panel) were quantified and are presented as bar graphs (Two-way ANOVA). **H** RNF126 knockdown by shRNAs led to decreased expression of CDK2 protein in MCF7 (left panel) and MDA-MB-231 cells (right panel). **I** Band intensities were quantified and are presented as bar graphs (Two-way ANOVA, left, MCF7; right, MDA-MB-231). **J** The expression of an E3 ligase mutant of RNF126 did not affect CDK2 protein expression. MCF7 (left panel) or MDA-MB-231 (right panel) cells were transfected with control vector, RNF126WT, or E3 ligase-deficient RNF126 (RNF126 C229A/C232A) plasmids and levels of CDK1/CDK2/CDK5 proteins were then detected by Western blotting. **K** Band intensities were quantified and are presented as bar graphs (Two-way ANOVA, left, MCF7; right, MDA-MB-231). **L** RNF126 and CDK2 mRNA levels in MCF7 (left panel) or MDA-MB-231 (right panel) cells, with or without RNF126 knockdown by shRNAs (One-way ANOVA). **M** The level of CDK2 mRNA expression in MCF7 (left panel) or MDA-MB-231 (right panel) cells, with RNF126WT/RNF126 C229A/C232A overexpression (One-way ANOVA). **N** The CDK2 mRNA relative expression levels in MCF7 and MDA-MB-231 without RNF126 (not CDK2) expression (Paired *t*-test). **O** Cell proliferation assay showed the effects of CDK2 overexpression in MCF7shRNF126#1 (left panel) and MDA-MB-231shRNF126#1 (right panel) cell at indicated time points (Two-way ANOVA). **P** Model for how RNF126 promotes breast cancer cell proliferation and as a biomarker of ATR inhibition. Data are presented as mean ± SD. *P < 0.05, **P < 0.01, and ***P < 0.001. All presented results are from three independent experiments

ATR prevents unscheduled origin firing and replication-related DNA damage [32]. ATR inhibition causes overactivation of CDKs-mediated excess origin firing and subsequently increases replication stress. To determine whether CDKs mediate endogenous replication stress in breast cancer cells with intact RNF126, we next measured the viability of breast cancer cells with or without RNF126 after co-treatment with AZD6738 and NU2058, a CDKs inhibitor. NU2058 pretreatment abrogated the cytotoxic effect of AZD6738 in MCF7 and MDA-MB-231 with RNF126 expression (Fig. 5A). Another CDKs inhibitor, Roscovitine, obtained the same results (Additional file 1: Fig. S11). NU2058 reduced the cell apoptosis of both MCF7 (Fig. 5B) and MDA-MB-231 (Fig. 5C) cells with higher expression of RNF126 mediated by treatment with AZD6738. Furthermore, NU2058 significantly alleviated replication stress in MCF7 and MDA-MB-231 cells with intact RNF126 treated with AZD6738 compared with MCF7 (Fig. 5D, E) and MDA-MB-231 (Fig. 5F, G) cells with RNF126 knockdown. Since NU2058 and Roscovitine have certain cross-linking effect on CDK1/CDK2/CDK5. To further clarify the relationship between RNF126 and CDKs, we detected whether the knockdown of RNF126 caused changes of CDK1/CDK2/CDK5 in protein or mRNA levels by western blot and qRT-PCR. The results showed that the knockdown of RNF126 could reduce the expression levels of CDK2 protein and mRNA, but not CDK1 or CDK5. Overexpression of RNF126 increased the protein and mRNA expression levels of CDK2, nor CDK1 or CDK5 (Fig. 5H–M, Additional file 1: Fig. S12). Considering that CDK2 may mediate the killing effect of ATR inhibitors on cells with high RNF126 expression, we performed CDK2 overexpression assay on RNF126 knockdown cells to verify (Fig. 5N), it is shown that overexpression of CDK2 in RNF126 knockdown cells accelerated the cell-killing effect (Fig. 5O). Taken together, these data suggest a mechanism of action in which inhibition of ATR in intact RNF126 cells induces an increase in CDK2-mediated replication stress in breast cancer cells, ultimately leading to DNA damage, replication fork collapse, cell apoptosis, and cell death (Fig. 5P).

## Discussion

Recurrence and metastasis are the main reasons for treatment failure in early breast cancer, however, the mechanism is still unclear. RNF126 promotes cell proliferation in a variety of cancer cells [8, 11], but the biological function of RNF126 in promoting breast cancer metastasis in early breast cancer is lacking. A reanalysis of the GSE11121 dataset of early breast cancer patients without lymph node metastasis found that RNF126 could promote breast cancer metastasis (Fig. 1A), and we verified this result in vitro and in vivo experiments (Fig. 2). Furthermore, analysis of 44 cases of early breast cancer patients without lymph node metastasis in TCGA found that RNF126 was highly expressed in tumor samples compared with normal breast tissues (Fig. 1B). These results are close to our previously reported. We have reported that RNF126 may be highly expressed in invasive breast cancer and that its expression is associated with a poor prognosis [24]. However, both datasets contained only a few samples. Thus, further study with a larger number of early breast cancer cases without lymph node metastasis is required.

Regardless, these results still suggested that RNF126 might be a potential therapeutic biomarker for breast cancer, even though the relationship between RNF126 protein expression and metastasis was still unclear. Using WGCNA, we found some signaling pathways related to the high expression of RNF126, such as cell cycle, proteasome, spliceosome pathway (Fig. 1C and D). It is well established that cell cycle machinery is controlled by such mechanisms, including feedback loops of genes and protein products that display periodic activation and repression, which are associated with cell proliferation and apoptosis [33]. We postulated that RNF126 may activate the cell cycle pathway and thereby promoted thereby promote breast cancer's malignant evolution. ATR is an important protein in the cell cycle signaling pathway. Targeting of the ATR is a promising strategy for the treatment of cancer. However, there is currently a lack of effective correlates with guiding the use of ATR inhibitors, and the expression level of ATR cannot effectively predict the effectiveness of ATR inhibitors. Moreover, we also found that ATR's expression level cannot predict breast cancer cells' metastasis (Additional file 1: Fig. S13). RNF126 can regulate the expression of CHEK1 protein and can be used as a predictive biomarker of CHEK1 inhibitors. ATR inhibitors are reported to be similar to CHEK1 inhibitors and we speculated that RNF126 might be a determinant of ATR inhibitors. In our model, we determined that breast cancer cells with a higher level of RNF126 are more sensitive to ATR inhibitors than breast cancer cells with a lower level of RNF126.

However, there is considerable crosstalk in the DNA damage response network, and CHEK1 is also activated by claspin after replication stress, independently of ATR [34]. There is also an ATR-dependent, CHEK1-independent, intra-S phase checkpoint that suppresses origin firing [35]. Moreover, ATR and CHEK1 have distinct functions and may not act linearly in the kinase cascade [36]. In addition, the combination of ATR and CHEK1 inhibitors has a synergistic lethal effect, and our results also supported this conclusion (Additional file 1: Figs. S8A, S9A). Interestingly, although we have previously reported that RNF126 knockdown reduces CHEK1 expression (24), we did not observe a synergistic lethal effect of ATR inhibitors on breast cancer cells with knockdown of RNF126 (Additional file 1: Figs. S8B and S9B). p-ATR can represent the functional status of ATR protein [25], that was examined p-ATR expression by western blot. The results showed that RNF126 knockdown increased p-ATR expression, which meant that the function of ATR protein had been activated, but ATR inhibitors could not effectively kill breast cancer cells with RNF126 knockdown (Fig. 3). These results implied that RNF126 may be a biomarker of ATR inhibitors.

It has been reported that ATR suppresses oncogene-induced replication stress and that ATR inhibitor monotherapy can effectively damage cells with high replication stress [37]. We hypothesized that the lower efficiency of ATR inhibitors in breast cancer cells with RNF126 knockdown might be explained by the relative reduction in replication stress [38, 39]. Activation of oncogenes leads to replication stress, as an excess of ongoing replication forks will consume the limited dNTP pool and causes fork stalling. We demonstrated that breast cancer cells with higher RNF126 expression could stall the replication fork and trigger abnormal replication initiation after ATR inhibitor application. However, the replication process of breast cancer cells with RNF126 knockdown was only mildly affected, with only mild changes in the replication elongation rate and new replication initiation rate (Fig. 4). These results confirmed our hypothesis that their own replication stress causes the ATR inhibitor sensitivity of breast cancer cells with higher RNF126 expression.

Wee1 deactivation leads to increased dNTP demand and replication stress through CDKs-induced firing of dormant replication origins [40]. Oncogene-deregulated CDKs activity is required to manifest the synthetic lethality of ATR and CHEK1 inhibitors [26]. Thus, we presumed that CDKs induce endogenous replication stress in breast cancer cells with RNF126. After using CDKs inhibitors to pretreat breast cancer cells with a higher level of RNF126, we observed that the cell-killing effect of ATR inhibitors could be counteracted by the inhibitors, in conjunction with an alleviates replication stress. Given that both NU2058 and Roscovitine have some interaction with CDK1/CDK2/CDK5, we detected the protein and mRNA levels of CDK1/CDK2/CDK5 by knockdown or overexpression of RNF126, we found that the expression level of RNF126 could affect the expression of CDK2, but not CDK1 or CDK5. In addition, overexpression of RNF126 C229A/C232A (the ubiquitination function of RNF126 was inactivated) [41] could not affect the expression of CDK2. These results showed that RNF126 might regulate the expression of CDK2 through transcription rather than ubiquitination (Fig. 5). E2F1 regulated expression of CDK2 by binding the promoter region of CDK2 [42]. RNF126 also binds to E2F1 [12], and the ability of RNF126 to regulate CDK2 might be mediated by E2F1. This hypothesis needs to be further verified. Moreover, overexpression of CDK2 in RNF126 knockdown cells accelerated the cell-killing effect. Thus, CDK2-mediated replication stress in breast cancer with a high level of RNF126 expression might be one of the reasons for the sensitivity of these breast cancer cells to ATR inhibitors.

Notably, despite the use of ATR inhibition as a monotherapy strategy to target chronic lymphocytic leukemia cells with TP53 defects [43], our results showed that breast cancer cells with high expression of RNF126 had enough endogenous replication stress via CDK2 to mediate and affect the sensitivity to ATR inhibitors in breast cancer cells with or without wild-type TP53 (Fig. 5P). This was consistent with published data reporting that ATR inhibition can target cancer cells as single agents irrespective of TP53 status [44]. Thus, acting as single agents, the mechanisms by which ATR inhibitors lead to cell death might be distinct. Overall, RNF126 has a greater advantage than ATR, or p-ATR expression alone, as a biomarker of ATR inhibitors.

## Conclusion

In conclusion, our model demonstrates that the high expression of RNF126 in early breast cancer patients without lymph node metastasis indicates a high-risk type of metastasis. This conclusion is validated using both in vitro and in vivo assays. Breast cancer cells with higher RNF126 expression have CDK2-mediated replication stress, which makes them potential targets for ATR inhibitors. Our results provide a proof-of-concept in preclinical models of a new paradigm for treating breast cancer with high expression of RNF126 via ATR inhibitors alone.

## Supplementary Information

**Additional file 1: Fig. S1.** The univariable cox proportional hazards regression analysis results in the GSE11121. The volcano plot showed the gene information between the positive and negative metastases-related genes. **Fig. S2.** GO analysis of all metastases-related genes in the GSE11121. GO analyses showed that information of positive (**A**) and negative (**B**) metastases-related genes have high enrichment in biological processes (BP), cellular components (CC), and molecular functions (MF). **Fig. S3.** KEGG analysis of all metastases-related genes in the GSE11121. KEGG analyses showed that information of positive (**A**) and negative (**B**) metastases-related genes has high enrichment in biological processes. **Fig. S4.** Weighted gene coexpression network analysis of RNF126 in the GSE11121. **A** Determination of soft-threshold power in the WGCNA. Analysis of the scale-free index for various soft-threshold powers (*β* = 5). **B** Analysis of the mean connectivity for various soft-threshold powers. **C** Checking the scale-free topology when *β* = 5. The x-axis demonstrates the logarithm of whole network connectivity, while the y-axis shows the logarithm of the corresponding frequency distribution. **D** On this plot, the distribution follows an approximately straight line, called approximately scale-free topology. **E** Dendrogram of all differentially expressed genes clustered based on dissimilarity measurement (1-TOM). The color band shows the results obtained from the automatic single-block analysis. **F** Matrix plot showed the degree of association of high RNF126 expression class and low RNF126 expression class with gene modules. **Fig. S5.** GO analysis of genes in the turquoise module. GO analyses showed the turquoise module enrichment information in BP, CC, and MF. **Fig. S6.** RNF126 promotes cell proliferation in MCF7 and MDA-MB-231 cells. **A**, **C** The RNF126 mRNA relative expression levels in MCF7 and MDA-MB-231 cells with or without RNF126 knockdown by shRNF126#2 (Paired *t*-test). **B**, **D** Cell proliferation assay showed the effects of depleted RNF126 by shRNF126#2 on MCF7 and MDA-MB-231 cells at indicated time points (Two-way ANOVA). Data are presented as mean ± SD. *P < 0.05, **P < 0.01, and ***P < 0.001. All presented results are from three independent experiments. **Fig. S7.** **A** RNF126 knockdown by shRNAs led to decreased expression of CHEK1 protein in MCF7 (left panel) and MDA-MB-231 cells (right panel). **B** Band intensities were quantified and are presented as bar graphs (Two-way ANOVA, left, MCF7; right, MDA-MB-231). **Fig. S8.** The effect of MCF7 cells with or without RNF126 knockdown treated with AZD6738 and AZD7762 individually or in combination. **A**, **B** MCF7 cells with or without RNF126 knockdown cultures treated with AZD6738 and AZD7762 individually or in combination at indicated concentrations for 72 h. (Upper panel) Cell viability was measured and normalized to DMSO control values. (Down panel) CI was calculated by using CalcuSyn software. CI less than 1 demonstrates the synergy between two drugs (*n* = 3). **Fig. S9.** The effect of MDA-MB-231 cells with or without RNF126 knockdown treated with AZD6738 and AZD7762 individually or in combination. **A**, **B** MDA-MB-231 cells with or without RNF126-depleted cultures treated with AZD6738 and AZD7762 individually or in combination at indicated concentrations for 72 h. (Upper panel) Cell viability was measured and normalized to DMSO control values. (Down panel) CI was calculated by using CalcuSyn software. CI less than 1 demonstrates the synergy between two drugs (*n* = 3). **Fig. S10.** AZD6738 increased replication stress in parental cells compared to cells depleted of RNF126 by shRNF126#2. Western blot analyses showed AZD6738 (1 μM) led to a greater increase in levels of p-RPA2 and γ-H2AX in parental cells than in cells with RNF126 knockdown by RNF126 shRNA#2. (**A**: MCF7 cells; **D**: MDA-MB-231 cells). Band intensities of p-RPA2 and γ-H2AX in MCF7 (**B**, **C**) and MDA-MB-231 (**E**, **F**) were quantified and are presented as bar graphs (Two-way ANOVA). Data are presented as mean ± SD. *P < 0.05, **P < 0.01, and ***P < 0.001. All presented results are from three independent experiments. **Fig. S11.** The effect of co-treatment Roscovitine and AZD6738 on MCF7 and MDA-MB-231 cells with or without RNF126 knockdown. MCF7 (**A**) and MDA-MB-231 (**B**) cells with or without RNF126 knockdown cultures treated with Roscovitine (5 μM) and various concentrations of AZD6738 for 72 h (Two-way ANOVA). Data are presented as mean ± SD. *P < 0.05, **P < 0.01, and ***P < 0.001. All presented results are from three independent experiments. **Fig. S12.** **A**, **B** RNF126 and CDK1/CDK5 mRNA levels in MCF7 (**A**) or MDA-MB-231 (**B**) cells, with or without RNF126 knockdown by shRNAs (One-way ANOVA). **C**, **D** The level of CDK2 mRNA expression in MCF7 (**C**) or MDA-MB-231 (**D**) cells with RNF126WT/RNF126 C229A/C232A overexpression (One-way ANOVA). Data are presented as mean ± SD. *P < 0.05, **P < 0.01, and ***P < 0.001. All presented results are from three independent experiments. **Fig. S13.** Metastases free survival analysis in patients between high ATR and low ATR expression in the GSE11121 cohorts. No statistical differences in metastases-free survival prognosis between breast cancer patients with higher or lower ATR expression (*n* = 199).

**Additional file 2: Table S1.** The univariable cox proportional hazards regression analysis results in GSE11121. **Table S2.** Gene Ontology Pathway Enrichment Analysis of all metastases-related genes. **Table S3.** Kyoto encyclopedia of genes and genomes pathway enrichment analysis of all metastases-related genes in GSE11121. **Table S4.** GO analysis of genes in the turquoise module. **Table S5.** KEGG analysis of genes in the turquoise module. **Table S6.** GO analysis of genes in the blue module. **Table S7.** GO analysis of genes in the brown module. **Table S8.** GO analysis of genes in the yellow module. **Table S9.** KEGG analysis of genes in the yellow module.

## Data Availability

All data generated for this study are included in the article.
