# Supplementary material for: Ring finger protein 126 promotes breast cancer metastasis and serves as a potential target to improve the therapeutic sensitivity of ATR inhibitors
Source: Breast Cancer Res. 2022 Dec 20;24:92. doi: 10.1186/s13058-022-01586-0 (PMC9764525; doi:10.1186/s13058-022-01586-0)
Supplement: Supplementary file 1 — Additional file 1: Fig. S1. The univariable cox proportional hazards regression analysis results in the GSE11121. The volcano plot showed the gene information between the positive and negative metastases-related genes. Fig. S2. GO analysis of all metastases-related genes in the GSE11121. GO analyses showed that information of positive (A) and negative (B) metastases-related genes have high enrichment in biological processes (BP), cellular components (CC), and molecular functions (MF). Fig. S3. KEGG analysis of all metastases-related genes in the GSE11121. KEGG analyses showed that information of positive (A) and negative (B) metastases-related genes has high enrichment in biological processes. Fig. S4. Weighted gene coexpression network analysis of RNF126 in the GSE11121. A Determination of soft-threshold power in the WGCNA. Analysis of the scale-free index for various soft-threshold powers (β = 5). B Analysis of the mean connectivity for various soft-threshold powers. C Checking the scale-free topology when β = 5. The x-axis demonstrates the logarithm of whole network connectivity, while the y-axis shows the logarithm of the corresponding frequency distribution. D On this plot, the distribution follows an approximately straight line, called approximately scale-free topology. E Dendrogram of all differentially expressed genes clustered based on dissimilarity measurement (1-TOM). The color band shows the results obtained from the automatic single-block analysis. F Matrix plot showed the degree of association of high RNF126 expression class and low RNF126 expression class with gene modules. Fig. S5. GO analysis of genes in the turquoise module. GO analyses showed the turquoise module enrichment information in BP, CC, and MF. Fig. S6. RNF126 promotes cell proliferation in MCF7 and MDA-MB-231 cells. A, C The RNF126 mRNA relative expression levels in MCF7 and MDA-MB-231 cells with or without RNF126 knockdown by shRNF126#2 (Paired t-test). B, D Cell proliferation assay sh [file 13058_2022_1586_MOESM1_ESM.docx]

**
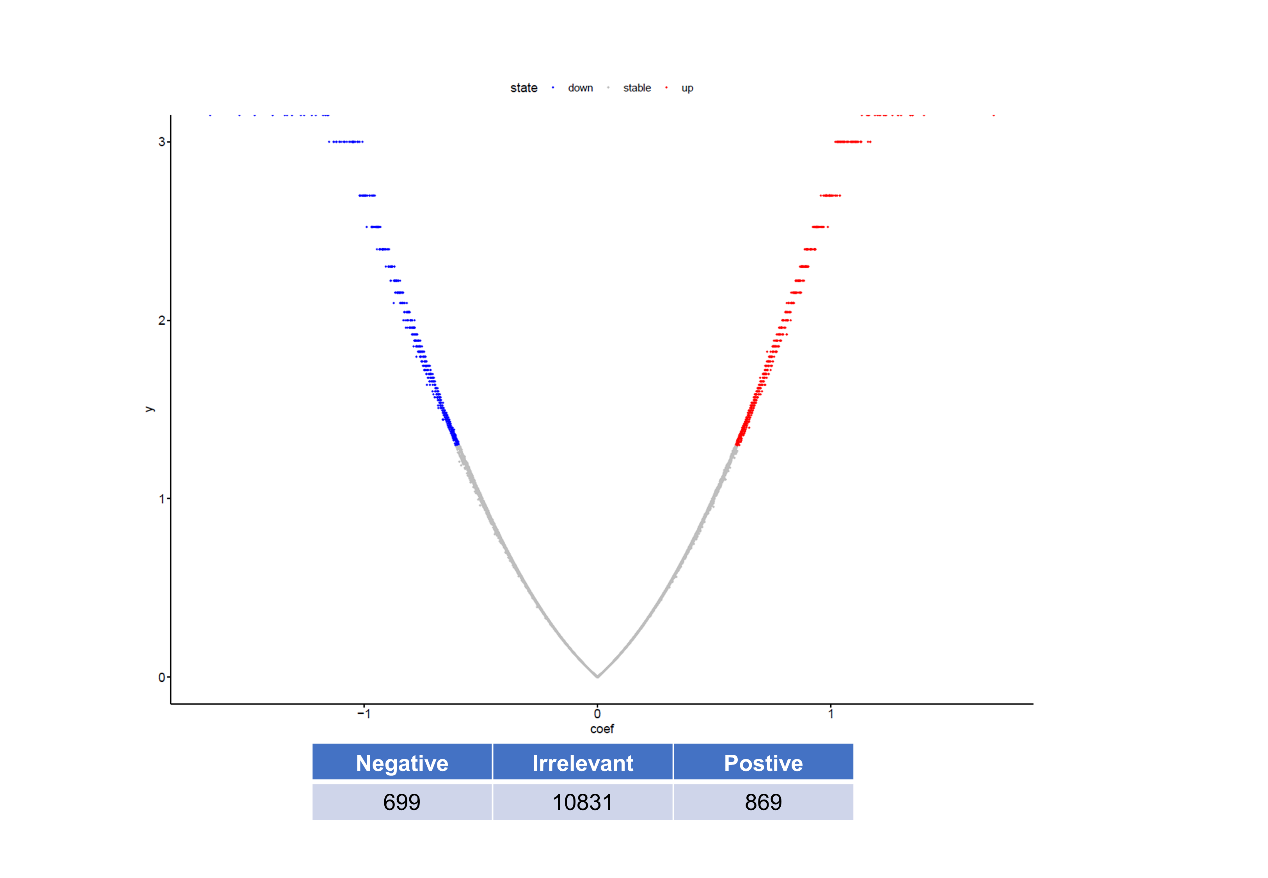
**

**Supplementary Figure 1.** The univariable cox proportional hazards regression analysis results in the GSE11121. The volcano plot showed the gene information between the positive and negative metastases-related genes.


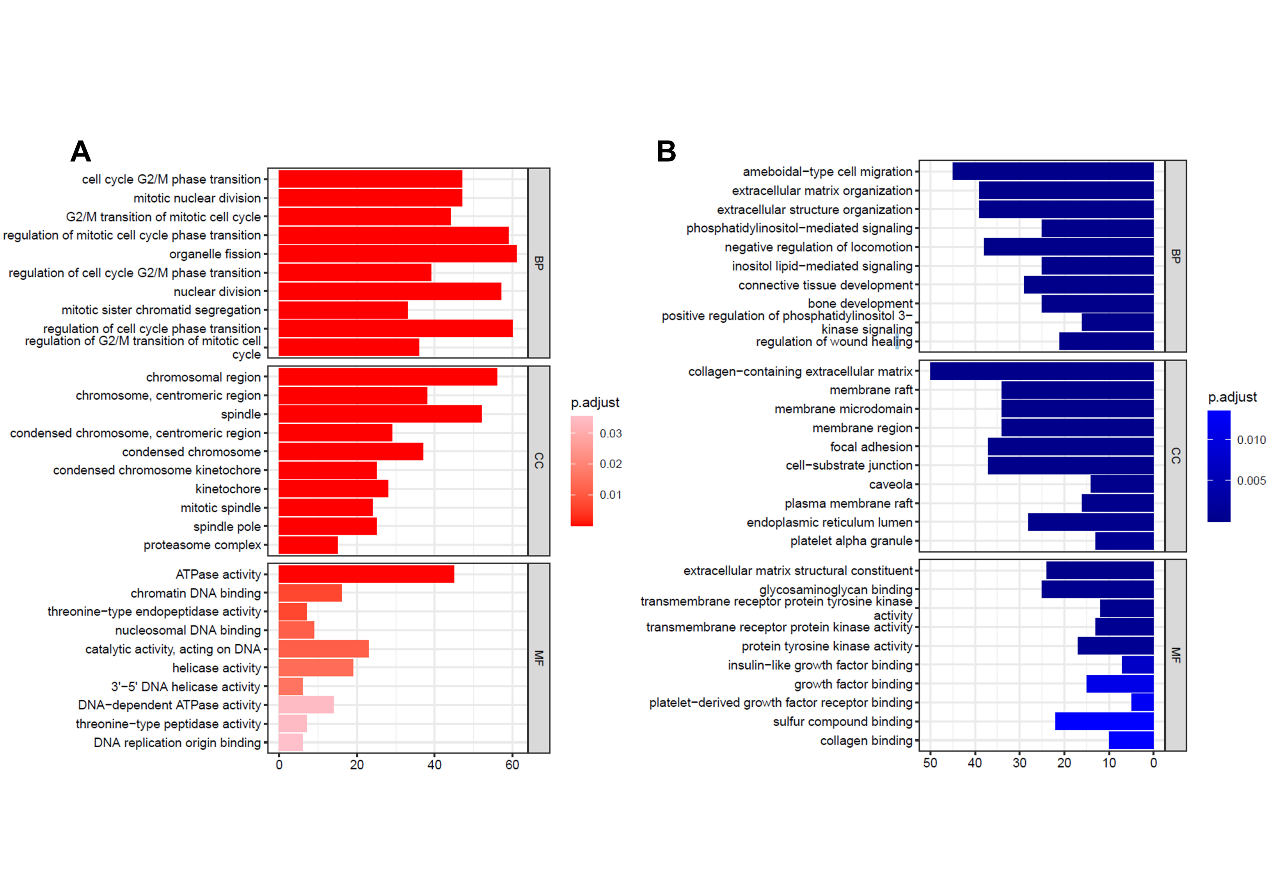


**Supplementary Figure 2.** GO analysis of all metastases-related genes in the GSE11121. GO analyses showed that information of positive (A) and negative (B) metastases-related genes have high enrichment in biological processes (BP), cellular components (CC), and molecular functions (MF).


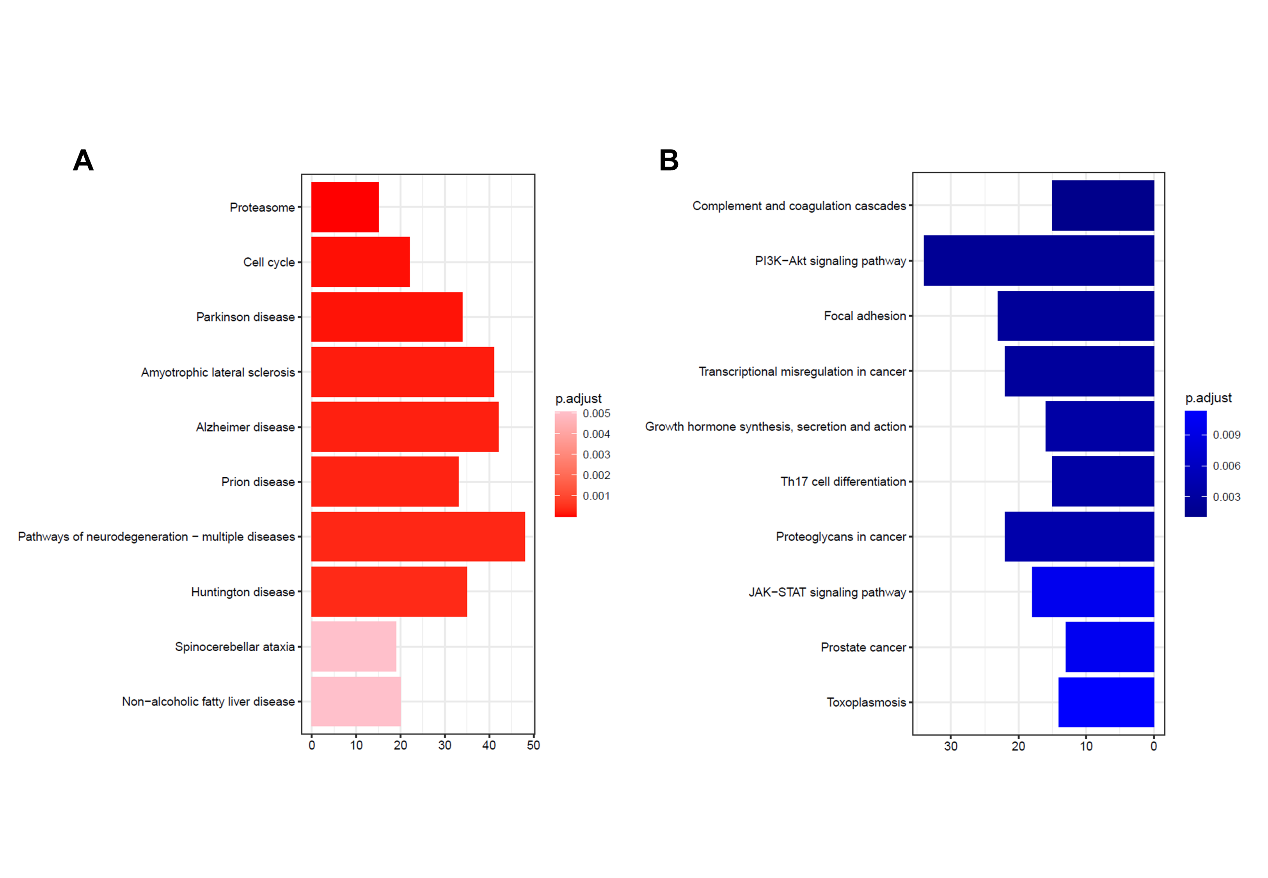


**Supplementary Figure 3.** KEGG analysis of all metastases-related genes in the GSE11121. KEGG analyses showed that information of positive (A) and negative (B) metastases-related genes has high enrichment in biological processes.


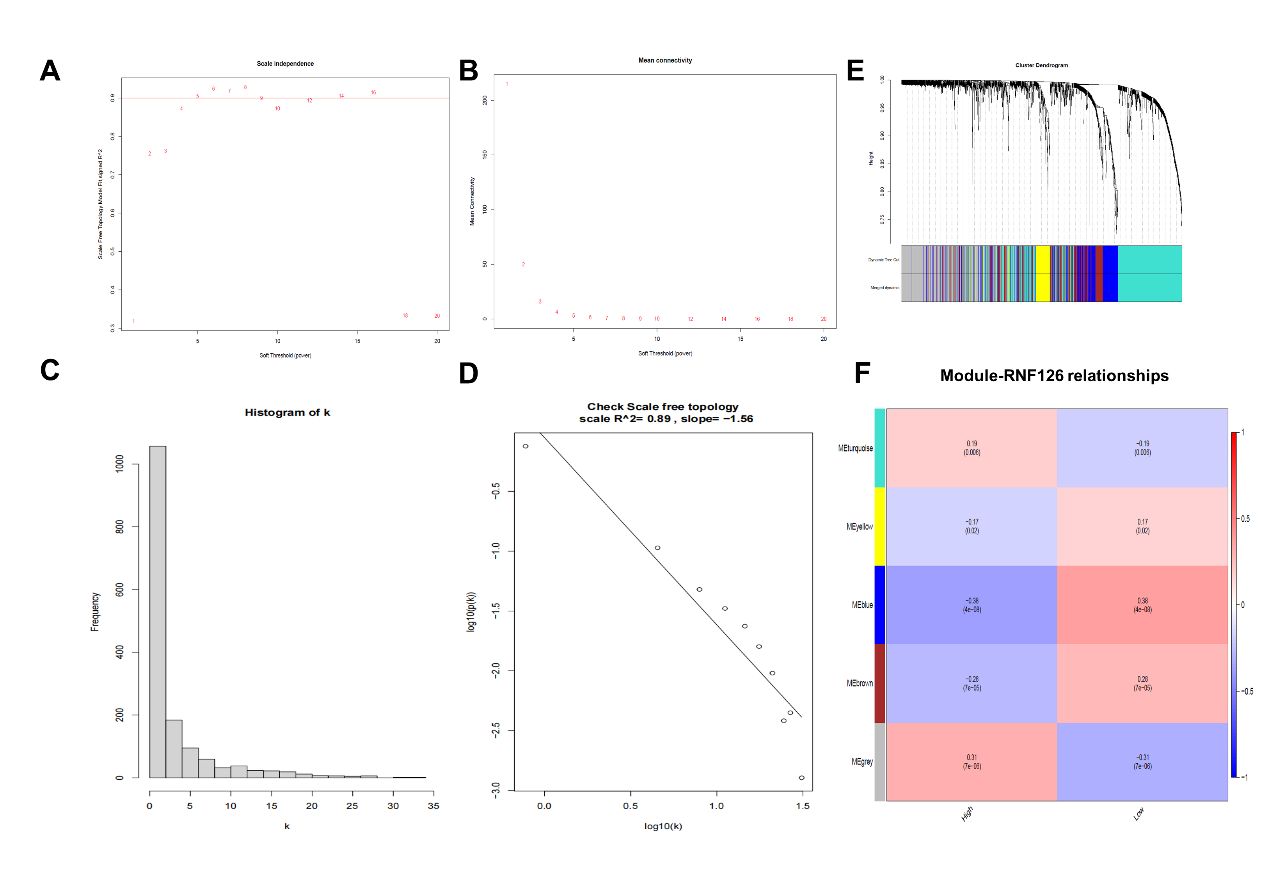


**Supplementary Figure 4.** Weighted gene co-expression network analysis of RNF126 in the GSE11121. (A) Determination of soft-threshold power in the WGCNA. Analysis of the scale-free index for various soft-threshold powers (β = 5). (B) Analysis of the mean connectivity for various soft-threshold powers. (C) Checking the scale free topology when β = 5. The x-axis demonstrates the logarithm of whole network connectivity, while the y-axis shows the logarithm of the corresponding frequency distribution. (D) On this plot, the distribution follows an approximately straight line, called approximately scale-free topology. (E) Dendrogram of all differentially expressed genes clustered based on dissimilarity measurement (1-TOM). The color band shows the results obtained from the automatic single-block analysis. (F) Matrix plot showed the degree of association of high RNF126 expression class and low RNF126 expression class with gene modules.


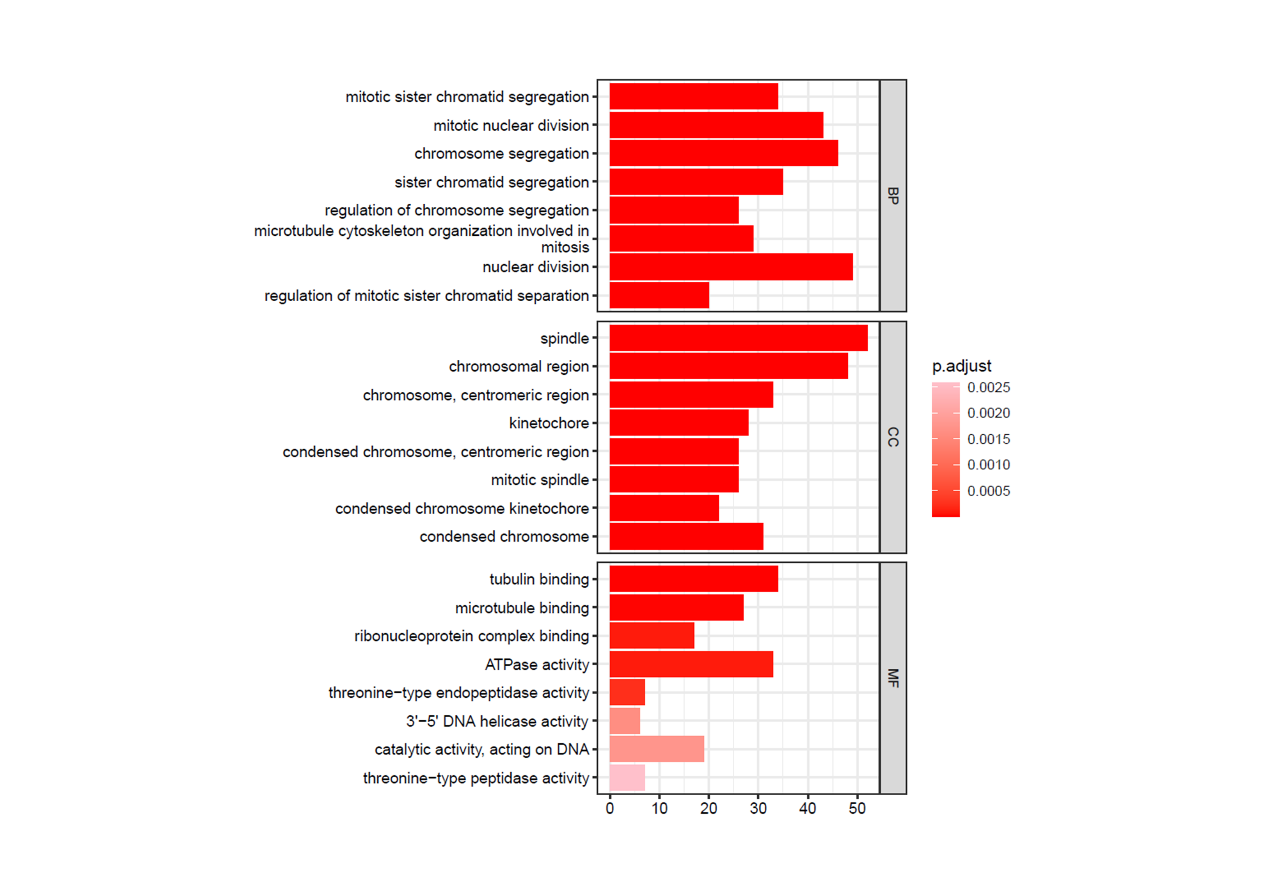


**Supplementary Figure 5.** GO analysis of genes in the turquoise module. GO analyses showed the turquoise module enrichment information in BP, CC, and MF.


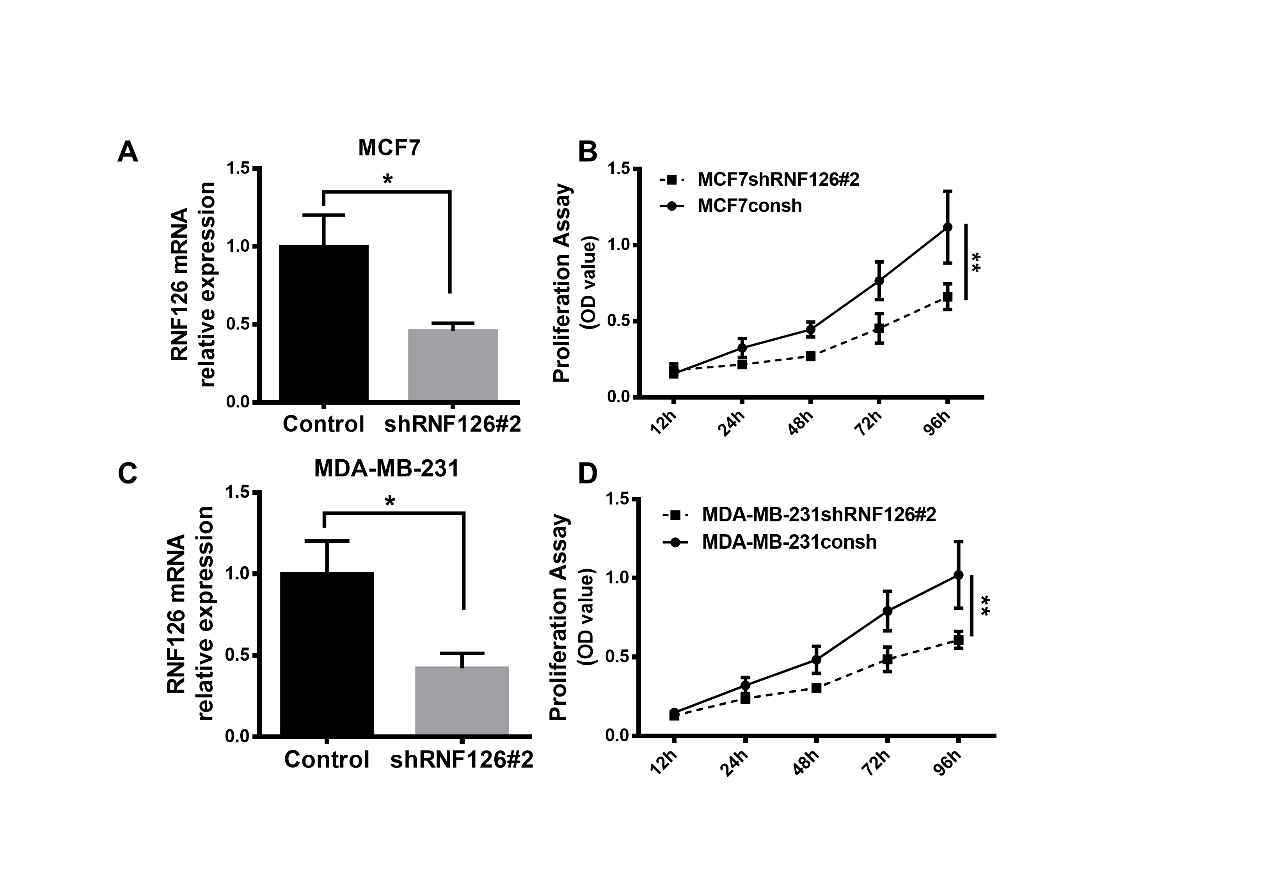


**Supplementary Figure 6.** RNF126 promotes cell proliferation in MCF7 and MDA-MB-231 cells. (A, C) The RNF126 mRNA relative expression levels in MCF7 and MDA-MB-231 cells with or without RNF126 knockdown by shRNF126#2 (Paired t-test). (B, D) Cell proliferation assay showed the effects of depleted RNF126 by shRNF126#2 on MCF7 and MDA-MB-231 cells at indicated time points (Two-way ANOVA). Data are presented as mean ± SD. *P < 0.05, **P < 0.01, and ***P < 0.001. All presented results are from three independent experiments.


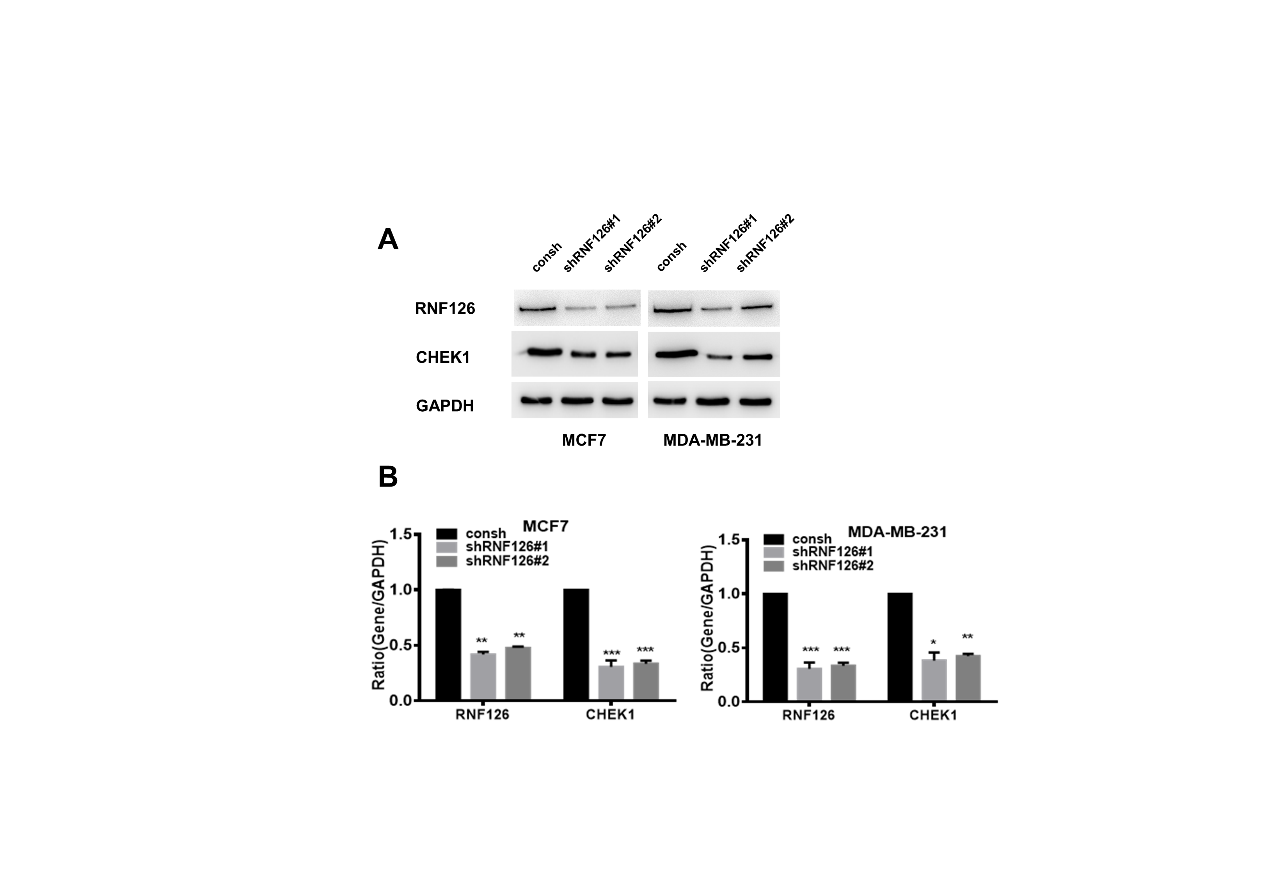


**Supplement Figure 7.** (A) RNF126 knockdown by shRNAs led to decreased expression of CHEK1 protein in MCF7 (left panel) and MDA-MB-231 cells (right panel). (B) Band intensities were quantified and are presented as bar graphs (Two-way ANOVA, left, MCF7; right, MDA-MB-231).


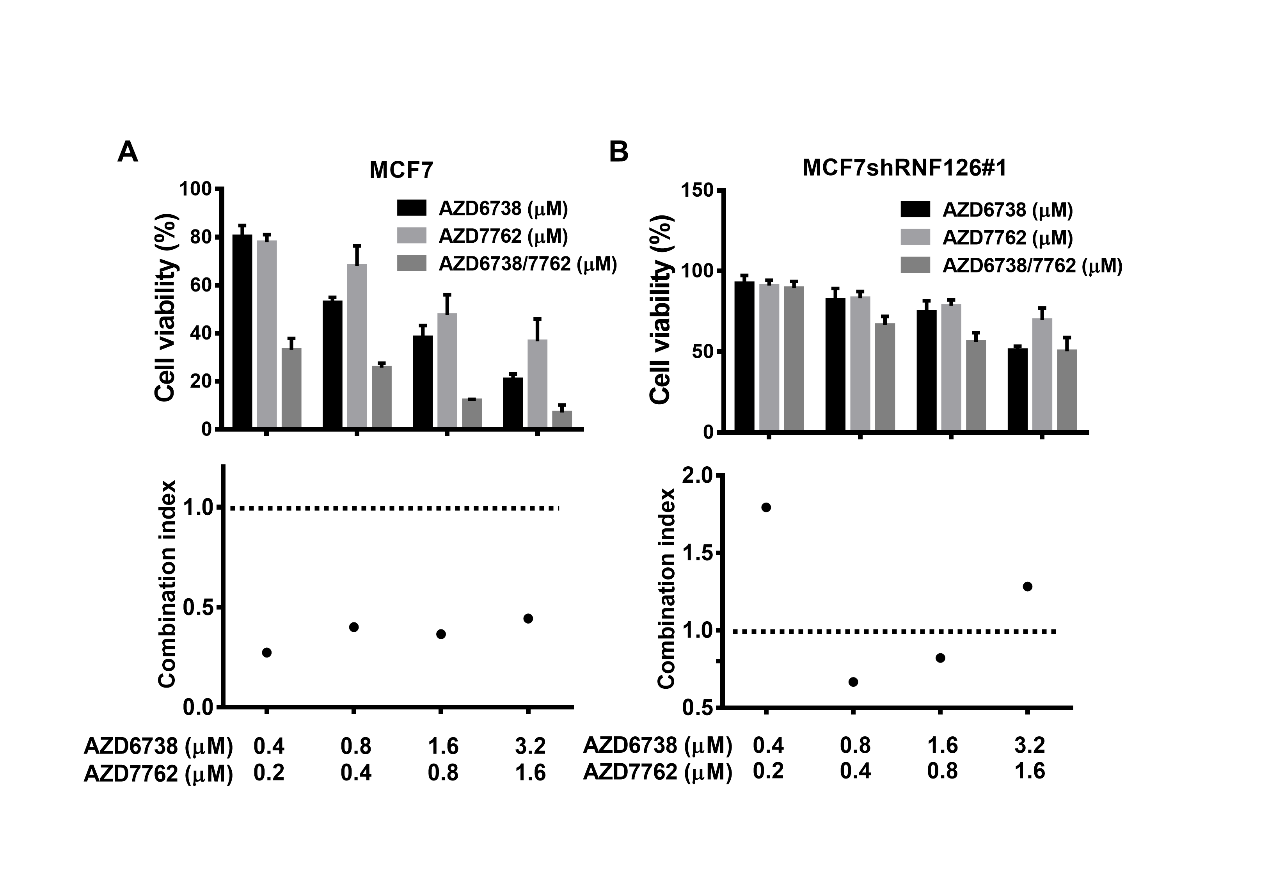


**Supplementary Figure 8.** The effect of MCF7 cells with or without RNF126 knockdown treated with AZD6738 and AZD7762 individually or in combination. (A, B) MCF7 cells with or without RNF126 knockdown cultures treated with AZD6738 and AZD7762 individually or in combination at indicated concentrations for 72 h. (Upper panel) Cell viability were measured and normalized to DMSO control values. (Down panel) CI was calculated by using CalcuSyn software. CI less than 1 demonstrates the synergy between two drugs (n = 3).


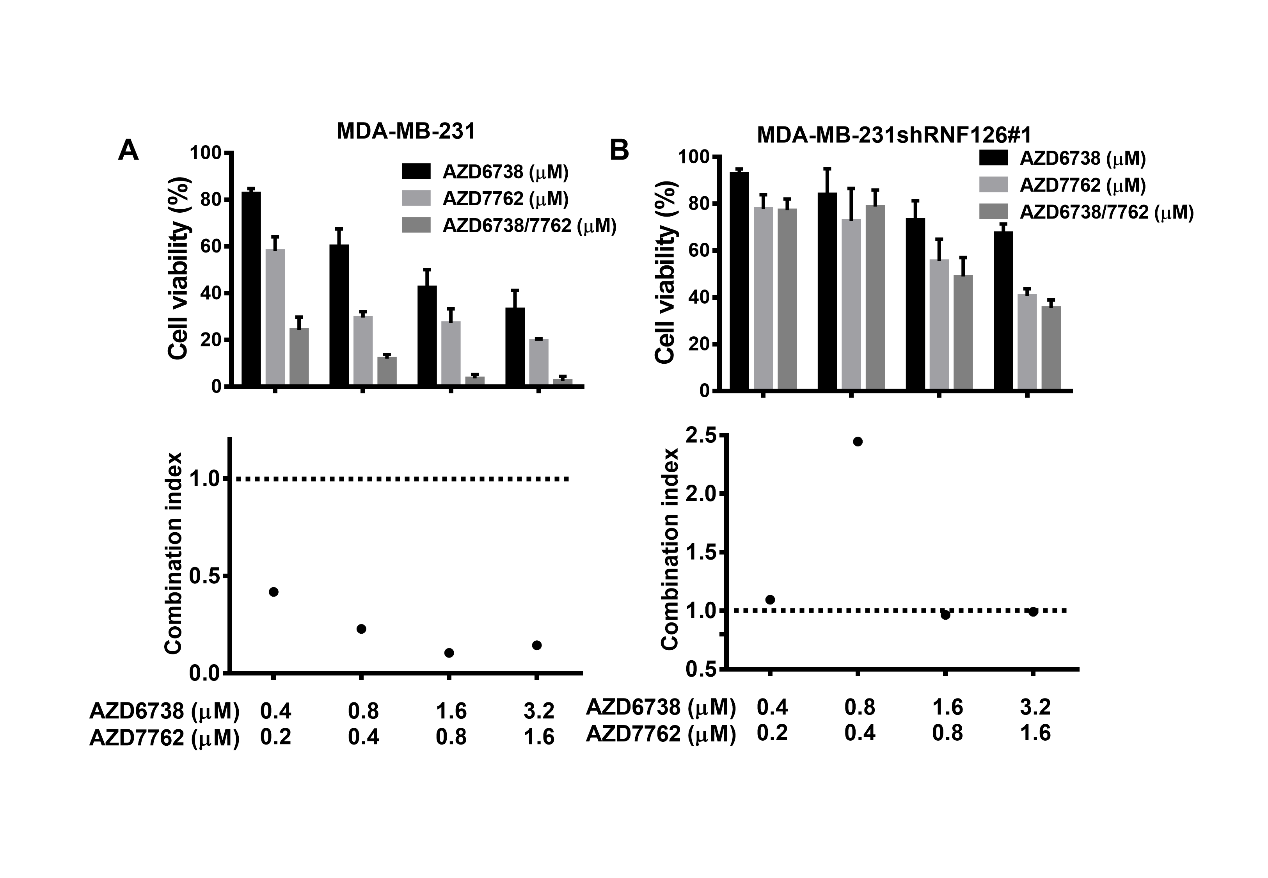


**Supplementary Figure 9.** The effect of MDA-MB-231 cells with or without RNF126 knockdown treated with AZD6738 and AZD7762 individually or in combination. (A, B) MDA-MB-231 cells with or without RNF126 depleted cultures treated with AZD6738 and AZD7762 individually or in combination at indicated concentrations for 72 h. (Upper panel) Cell viability were measured and normalized to DMSO control values. (Down panel) CI was calculated by using CalcuSyn software. CI less than 1 demonstrates the synergy between two drugs (n = 3).


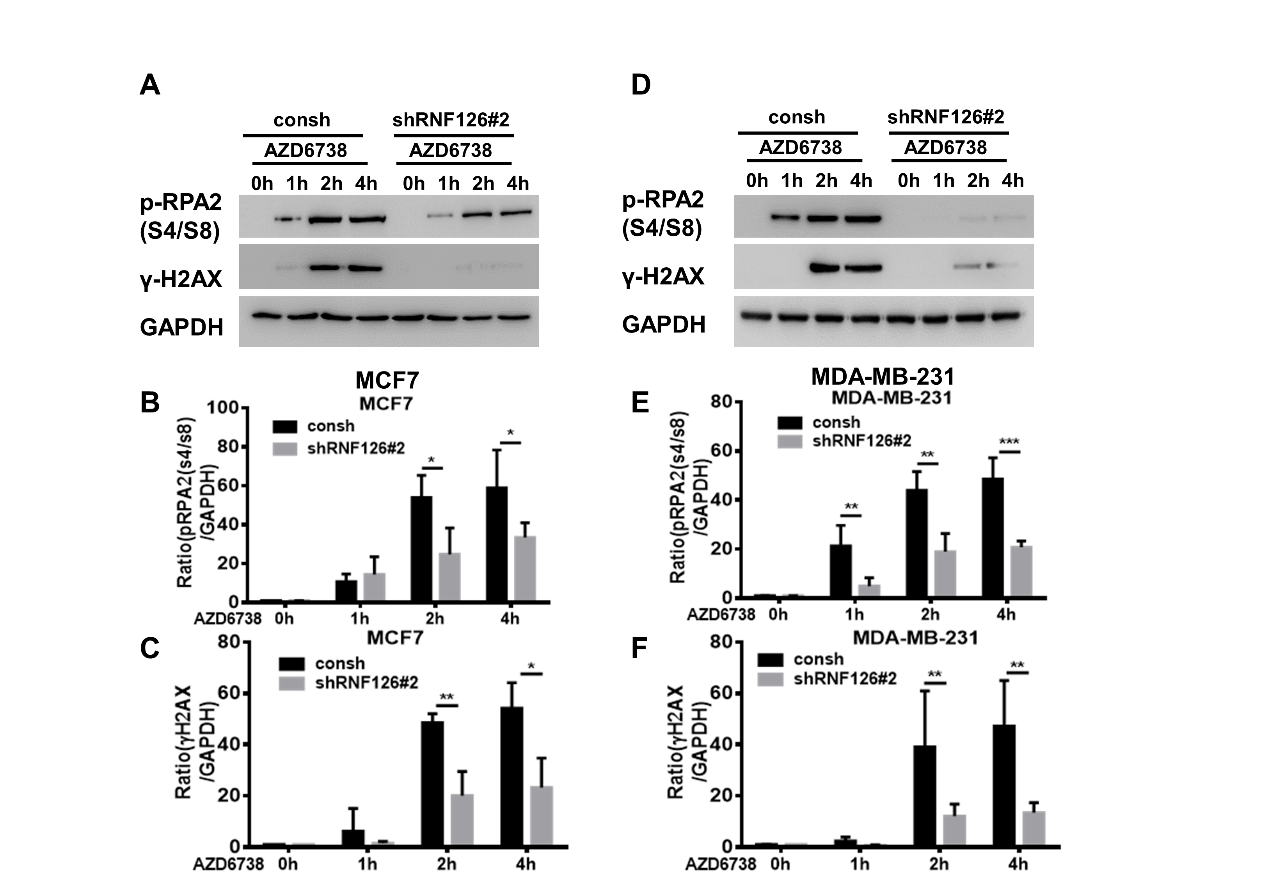


**Supplementary Figure 10.** AZD6738 increased replication stress in parental cells compared to cells depleted of RNF126 by shRNF126#2. Western blot analyses showed AZD6738 (1 μM) led to a greater increase in levels of p-RPA2 and γ-H2AX in parental cells than in cells with RNF126 knockdown by RNF126 shRNA#2. (A: MCF7 cells; D: MDA-MB-231 cells). Band intensities of p-RPA2 and γ-H2AX in MCF7 (B, C) and MDA-MB-231 (E, F) were quantified and are presented as bar graphs (Two-way ANOVA). Data are presented as mean ± SD. *P < 0.05, **P < 0.01, and ***P < 0.001. All presented results are from three independent experiments.


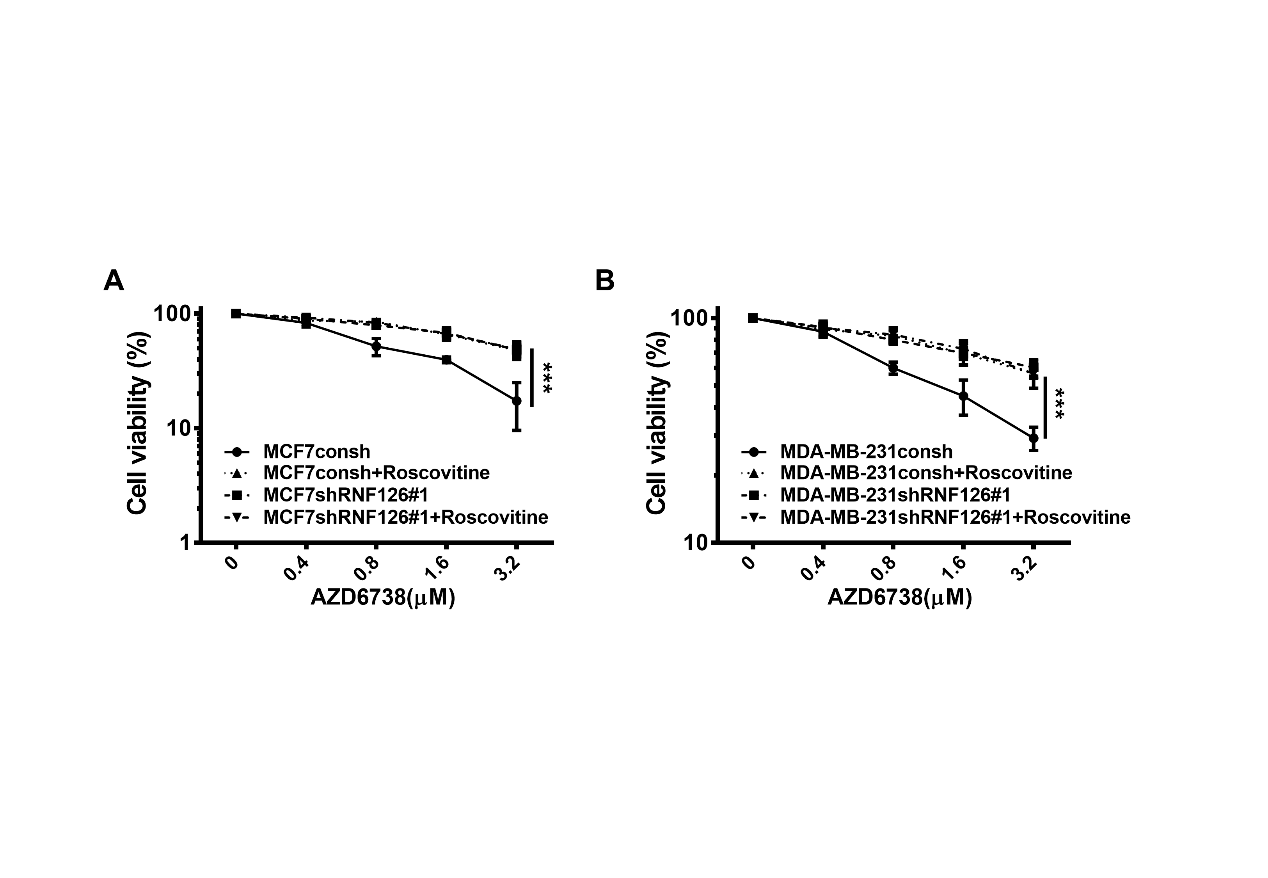


**Supplementary Figure 11.** The effect of co-treatment Roscovitine and AZD6738 on MCF7 and MDA-MB-231 cells with or without RNF126 knockdown. MCF7 (A) and MDA-MB-231 (B) cells with or without RNF126 knockdown cultures treated with Roscovitine (5 μM) and various concentrations of AZD6738 for 72h (Two-way ANOVA). Data are presented as mean ± SD. *P < 0.05, **P < 0.01, and ***P < 0.001. All presented results are from three independent experiments.


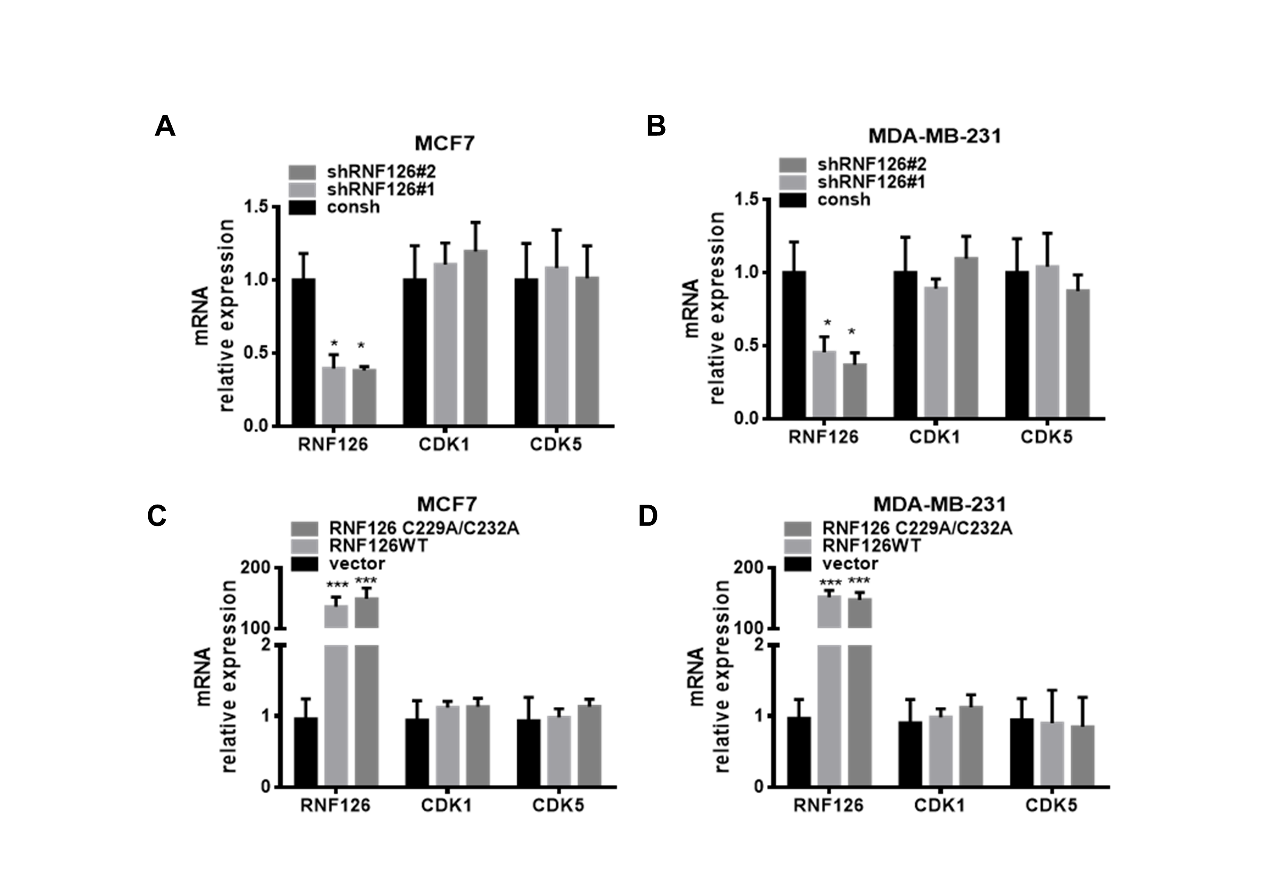


**Supplementary Figure 12.** (A, B) RNF126 and CDK1/CDK5 mRNA levels in MCF7 (A) or MDA-MB-231 (B) cells, with or without RNF126 knockdown by shRNAs (One-way ANOVA). (C, D) The level of CDK2 mRNA expression in MCF7 (C) or MDA-MB-231 (D) cells with RNF126WT/RNF126 C229A/C232A overexpression (One-way ANOVA). Data are presented as mean ± SD. *P < 0.05, **P < 0.01, and ***P < 0.001. All presented results are from three independent experiments.

**
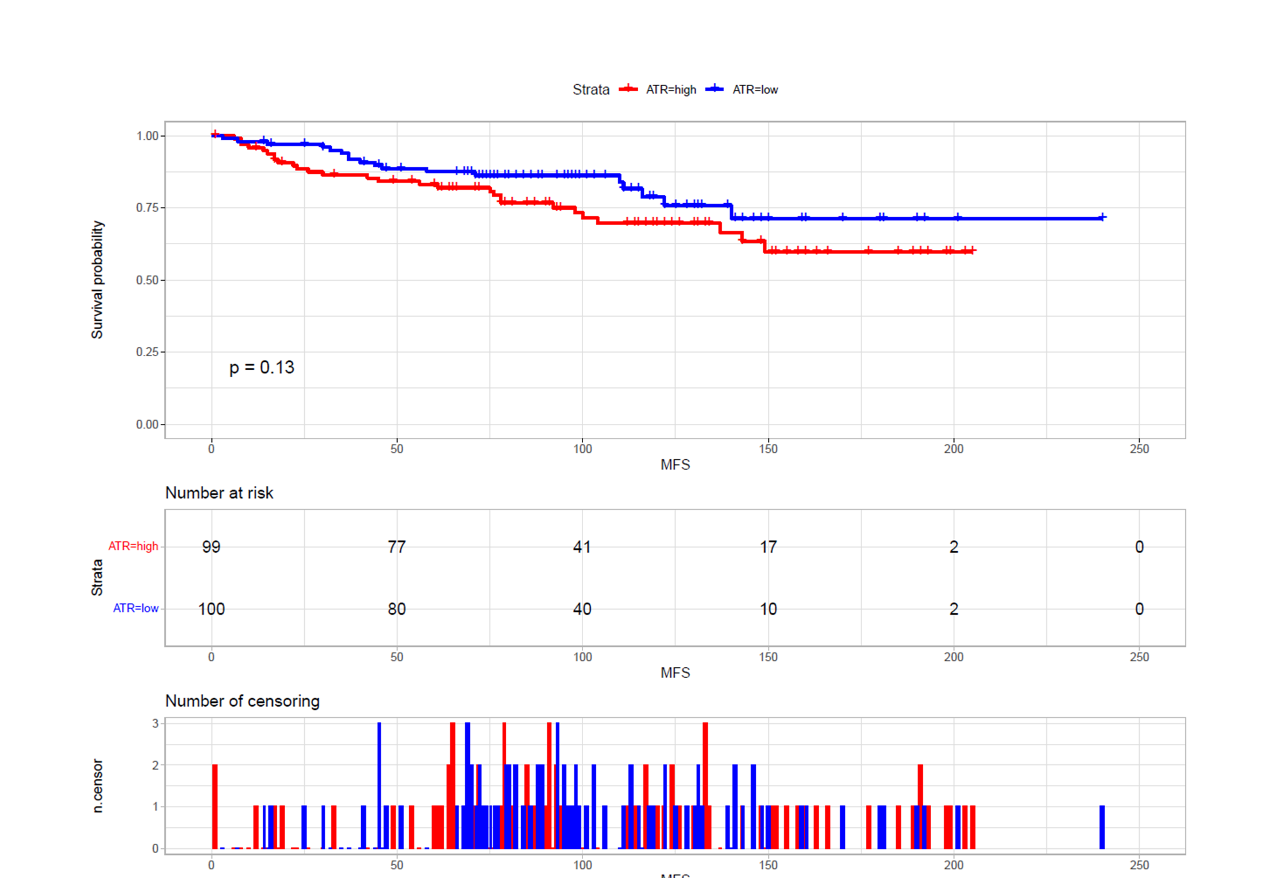
**

**Supplementary Figure 13.** Metastases free survival analysis in patients between high ATR and low ATR expression in the GSE11121 cohorts. No statistical differences in metastases-free survival prognosis between breast cancer patients with higher or lower ATR expression (n=199).
