# Supplementary material for: Ring finger protein 126 promotes breast cancer metastasis and serves as a potential target to improve the therapeutic sensitivity of ATR inhibitors
Source: Breast Cancer Res. 2022 Dec 20;24:92. doi: 10.1186/s13058-022-01586-0 (PMC9764525; doi:10.1186/s13058-022-01586-0)
Supplement: Supplementary file 2 — Additional file 2: Table S1. The univariable cox proportional hazards regression analysis results in GSE11121. Table S2. Gene Ontology Pathway Enrichment Analysis of all metastases-related genes. Table S3. Kyoto encyclopedia of genes and genomes pathway enrichment analysis of all metastases-related genes in GSE11121. Table S4. GO analysis of genes in the turquoise module. Table S5. KEGG analysis of genes in the turquoise module. Table S6. GO analysis of genes in the blue module. Table S7. GO analysis of genes in the brown module. Table S8. GO analysis of genes in the yellow module. Table S9. KEGG analysis of genes in the yellow module. [file 13058_2022_1586_MOESM2_ESM.pdf]

Supplementary table 1. The univariable cox proportional hazards regression analysis results in GSE11121										
gene	coef	se	z	p	HR	HRse	HRz	HRp	HRCILL	HRCIUL
ZZZ3	-0.52	0.304	-1.713	0.087	0.594	0.18	-2.247	0.025	0.328	1.078
ZZEF1	-0.458	0.3	-1.525	0.127	0.633	0.19	-1.934	0.053	0.351	1.14
ZYX	-0.828	0.315	-2.63	0.009	0.437	0.138	-4.092	0	0.236	0.81
ZXDC	0.881	0.315	2.799	0.005	2.415	0.76	1.86	0.063	1.303	4.476
ZXDB	0.165	0.296	0.558	0.577	1.18	0.349	0.514	0.607	0.66	2.107
ZWINT	0.637	0.306	2.081	0.037	1.89	0.578	1.54	0.124	1.038	3.442
ZWILCH	-0.004	0.297	-0.014	0.988	0.996	0.295	-0.014	0.988	0.557	1.781
ZW10	0.267	0.3	0.887	0.375	1.306	0.392	0.779	0.436	0.724	2.353
ZSWIM8	0.07	0.295	0.236	0.814	1.072	0.317	0.228	0.82	0.601	1.912
ZSWIM1	0.082	0.296	0.276	0.783	1.085	0.321	0.265	0.791	0.607	1.938
ZSCAN9	0.04	0.295	0.135	0.893	1.041	0.307	0.132	0.895	0.584	1.855
ZSCAN5A	-0.164	0.296	-0.552	0.581	0.849	0.252	-0.599	0.549	0.475	1.518
ZSCAN32	-0.078	0.296	-0.264	0.792	0.925	0.273	-0.275	0.784	0.518	1.651
ZSCAN31	0.088	0.296	0.297	0.766	1.092	0.323	0.284	0.776	0.612	1.949
ZSCAN26	0.007	0.295	0.023	0.982	1.007	0.297	0.023	0.982	0.565	1.795
ZSCAN2	0.491	0.3	1.637	0.102	1.633	0.49	1.294	0.196	0.908	2.939
ZSCAN18	-0.294	0.298	-0.989	0.323	0.745	0.222	-1.15	0.25	0.416	1.335
ZSCAN16	0.631	0.303	2.08	0.038	1.879	0.569	1.543	0.123	1.037	3.403
ZSCAN12	-0.115	0.295	-0.388	0.698	0.892	0.263	-0.411	0.681	0.5	1.59
ZRSR2	0.182	0.296	0.615	0.538	1.2	0.355	0.563	0.574	0.672	2.142
ZPR1	0.41	0.3	1.37	0.171	1.507	0.452	1.123	0.261	0.838	2.712
ZPBP	-0.399	0.3	-1.329	0.184	0.671	0.202	-1.633	0.102	0.372	1.209
ZP2	0.396	0.3	1.322	0.186	1.486	0.446	1.091	0.275	0.826	2.675
ZNRF4	0.44	0.3	1.466	0.143	1.553	0.466	1.186	0.236	0.862	2.796
ZNRD2	0.62	0.304	2.043	0.041	1.859	0.564	1.523	0.128	1.026	3.37
ZNHIT6	0.031	0.296	0.104	0.917	1.031	0.305	0.103	0.918	0.578	1.842
ZNHIT3	-0.095	0.296	-0.321	0.748	0.909	0.269	-0.337	0.736	0.509	1.625
ZNHIT2	0.13	0.296	0.438	0.661	1.138	0.337	0.411	0.681	0.637	2.033
ZNHIT1	0.005	0.296	0.017	0.986	1.005	0.297	0.017	0.986	0.563	1.795
ZNF93	-0.11	0.295	-0.372	0.71	0.896	0.265	-0.393	0.695	0.502	1.599
ZNF91	0.6	0.304	1.974	0.048	1.822	0.553	1.484	0.138	1.004	3.304
ZNF862	-1.053	0.328	-3.213	0.001	0.349	0.114	-5.696	0	0.183	0.663
ZNF85	0.127	0.296	0.429	0.668	1.135	0.336	0.403	0.687	0.635	2.029
ZNF84	-0.208	0.298	-0.7	0.484	0.812	0.242	-0.778	0.437	0.453	1.455
ZNF839	-0.163	0.296	-0.549	0.583	0.85	0.252	-0.596	0.551	0.476	1.519
ZNF835	-0.424	0.302	-1.403	0.161	0.654	0.198	-1.748	0.081	0.362	1.183

ZNF83	-0.297	0.297	-1.001	0.317	0.743	0.221	-1.166	0.244	0.415	1.33
ZNF821	0.149	0.295	0.505	0.614	1.161	0.343	0.469	0.639	0.651	2.071
ZNF814	0.397	0.3	1.325	0.185	1.488	0.446	1.094	0.274	0.827	2.676
ZNF813	-0.105	0.297	-0.352	0.725	0.901	0.267	-0.371	0.71	0.504	1.611
ZNF81	0.028	0.295	0.094	0.925	1.028	0.304	0.093	0.926	0.576	1.834
ZNF804A	0.296	0.298	0.996	0.319	1.345	0.4	0.862	0.389	0.751	2.41
ZNF80	-0.055	0.296	-0.187	0.852	0.946	0.28	-0.192	0.848	0.53	1.689
ZNF8	0.053	0.297	0.179	0.858	1.055	0.313	0.175	0.861	0.589	1.888
ZNF79	-0.461	0.303	-1.524	0.127	0.631	0.191	-1.937	0.053	0.349	1.141
ZNF787	-0.124	0.297	-0.418	0.676	0.883	0.262	-0.445	0.656	0.494	1.58
ZNF780B	0.333	0.298	1.119	0.263	1.395	0.415	0.952	0.341	0.779	2.5
ZNF771	0.23	0.296	0.775	0.438	1.258	0.373	0.692	0.489	0.704	2.248
ZNF770	0.052	0.296	0.175	0.861	1.053	0.312	0.171	0.865	0.589	1.883
ZNF767P	-0.065	0.296	-0.218	0.827	0.937	0.277	-0.225	0.822	0.525	1.674
ZNF764	0.6	0.303	1.984	0.047	1.822	0.551	1.492	0.136	1.007	3.297
ZNF76	0.553	0.301	1.838	0.066	1.738	0.523	1.412	0.158	0.964	3.134
ZNF75D	-0.203	0.296	-0.685	0.493	0.816	0.242	-0.76	0.447	0.457	1.459
ZNF750	0.413	0.3	1.376	0.169	1.511	0.453	1.127	0.26	0.839	2.721
ZNF749	-0.382	0.303	-1.263	0.207	0.682	0.207	-1.538	0.124	0.377	1.235
ZNF747	-0.308	0.298	-1.034	0.301	0.735	0.219	-1.211	0.226	0.41	1.317
ZNF74	0.232	0.296	0.782	0.434	1.261	0.374	0.698	0.485	0.705	2.254
ZNF721	0.497	0.312	1.593	0.111	1.644	0.513	1.255	0.21	0.892	3.031
ZNF717	0.151	0.296	0.511	0.61	1.163	0.345	0.474	0.636	0.651	2.079
ZNF711	0.705	0.31	2.274	0.023	2.023	0.627	1.632	0.103	1.102	3.713
ZNF710	0.113	0.295	0.383	0.701	1.12	0.331	0.362	0.717	0.628	1.998
ZNF706	0.278	0.298	0.933	0.351	1.32	0.393	0.815	0.415	0.737	2.366
ZNF702P	0.347	0.302	1.149	0.251	1.415	0.428	0.97	0.332	0.782	2.56
ZNF701	0.202	0.297	0.68	0.496	1.224	0.363	0.616	0.538	0.684	2.189
ZNF7	0.556	0.306	1.816	0.069	1.743	0.533	1.393	0.164	0.957	3.175
ZNF696	0.47	0.3	1.568	0.117	1.6	0.479	1.251	0.211	0.889	2.878
ZNF692	-0.389	0.298	-1.303	0.193	0.678	0.202	-1.592	0.111	0.378	1.217
ZNF688	-0.153	0.296	-0.517	0.605	0.858	0.254	-0.559	0.576	0.48	1.533
ZNF682	0.009	0.296	0.031	0.975	1.009	0.298	0.031	0.976	0.565	1.802
ZNF675	0.139	0.296	0.47	0.638	1.149	0.34	0.439	0.661	0.644	2.051
ZNF674	0.897	0.316	2.841	0.005	2.452	0.774	1.876	0.061	1.32	4.551
ZNF672	-0.197	0.297	-0.662	0.508	0.821	0.244	-0.732	0.464	0.459	1.47
ZNF671	-0.101	0.297	-0.339	0.734	0.904	0.268	-0.357	0.721	0.505	1.618
ZNF669	0.166	0.298	0.557	0.578	1.181	0.352	0.513	0.608	0.658	2.119

ZNF668	-0.221	0.299	-0.741	0.459	0.802	0.239	-0.829	0.407	0.446	1.439
ZNF667	0.058	0.295	0.195	0.846	1.059	0.313	0.189	0.85	0.594	1.89
ZNF665	-0.36	0.299	-1.202	0.229	0.698	0.209	-1.447	0.148	0.388	1.255
ZNF654	-0.372	0.298	-1.246	0.213	0.69	0.206	-1.509	0.131	0.384	1.237
ZNF652	-0.245	0.297	-0.826	0.409	0.783	0.232	-0.936	0.349	0.437	1.4
ZNF646	0.595	0.306	1.945	0.052	1.814	0.555	1.466	0.143	0.996	3.304
ZNF639	0.009	0.296	0.029	0.977	1.009	0.299	0.029	0.977	0.565	1.802
ZNF638	0.194	0.297	0.656	0.512	1.215	0.36	0.596	0.551	0.679	2.172
ZNF629	0.169	0.296	0.572	0.568	1.185	0.351	0.526	0.599	0.663	2.117
ZNF623	0.481	0.3	1.603	0.109	1.617	0.485	1.273	0.203	0.899	2.91
ZNF614	-0.273	0.297	-0.918	0.358	0.761	0.226	-1.056	0.291	0.425	1.363
ZNF613	-0.358	0.298	-1.202	0.229	0.699	0.208	-1.445	0.148	0.39	1.253
ZNF611	0.396	0.303	1.307	0.191	1.486	0.45	1.079	0.28	0.821	2.69
ZNF609	0.029	0.295	0.097	0.922	1.029	0.304	0.096	0.924	0.577	1.835
ZNF606	0.21	0.296	0.708	0.479	1.233	0.365	0.639	0.523	0.69	2.204
ZNF593	0.097	0.295	0.328	0.743	1.102	0.325	0.313	0.754	0.618	1.965
ZNF592	0.204	0.296	0.69	0.49	1.227	0.363	0.624	0.533	0.686	2.192
ZNF589	-0.364	0.298	-1.222	0.222	0.695	0.207	-1.474	0.14	0.387	1.246
ZNF587B	0.008	0.296	0.027	0.978	1.008	0.298	0.027	0.978	0.564	1.801
ZNF587	0.34	0.297	1.144	0.253	1.405	0.417	0.97	0.332	0.785	2.514
ZNF586	-0.126	0.296	-0.427	0.669	0.881	0.261	-0.455	0.649	0.493	1.574
ZNF580	-0.285	0.301	-0.948	0.343	0.752	0.226	-1.096	0.273	0.417	1.356
ZNF576	-0.011	0.296	-0.038	0.97	0.989	0.293	-0.038	0.969	0.554	1.766
ZNF574	0.067	0.297	0.227	0.821	1.07	0.318	0.219	0.826	0.598	1.915
ZNF573	-0.039	0.295	-0.132	0.895	0.962	0.284	-0.134	0.893	0.539	1.715
ZNF571	-0.08	0.297	-0.27	0.787	0.923	0.274	-0.281	0.779	0.516	1.652
ZNF562	0.658	0.306	2.151	0.031	1.93	0.59	1.576	0.115	1.06	3.514
ZNF557	-0.195	0.299	-0.651	0.515	0.823	0.246	-0.719	0.472	0.458	1.479
ZNF556	0.233	0.298	0.784	0.433	1.263	0.376	0.699	0.485	0.705	2.263
ZNF552	-0.1	0.297	-0.339	0.735	0.904	0.268	-0.356	0.722	0.506	1.618
ZNF551	0.183	0.296	0.619	0.536	1.201	0.356	0.566	0.572	0.672	2.146
ZNF550	-0.389	0.3	-1.297	0.195	0.678	0.203	-1.585	0.113	0.377	1.22
ZNF549	0.406	0.3	1.354	0.176	1.501	0.45	1.113	0.266	0.834	2.701
ZNF544	0.186	0.295	0.63	0.529	1.204	0.356	0.575	0.566	0.675	2.149
ZNF536	0.013	0.296	0.043	0.966	1.013	0.3	0.043	0.966	0.567	1.81
ZNF532	0.038	0.296	0.127	0.899	1.038	0.307	0.125	0.901	0.582	1.854
ZNF529	-0.292	0.298	-0.982	0.326	0.746	0.222	-1.14	0.254	0.416	1.338
ZNF528	0.097	0.295	0.328	0.743	1.102	0.325	0.312	0.755	0.617	1.966

ZNF518A	0.029	0.295	0.1	0.92	1.03	0.304	0.098	0.922	0.577	1.837
ZNF516	-0.819	0.315	-2.601	0.009	0.441	0.139	-4.029	0	0.238	0.817
ZNF512B	-0.293	0.298	-0.984	0.325	0.746	0.222	-1.144	0.253	0.416	1.337
ZNF510	-0.268	0.298	-0.898	0.369	0.765	0.228	-1.03	0.303	0.427	1.372
ZNF507	0.177	0.297	0.597	0.55	1.194	0.354	0.547	0.584	0.668	2.134
ZNF506	-0.008	0.295	-0.028	0.978	0.992	0.293	-0.028	0.978	0.556	1.769
ZNF500	0.387	0.3	1.291	0.197	1.472	0.441	1.07	0.284	0.818	2.647
ZNF493	0.335	0.298	1.125	0.261	1.398	0.416	0.956	0.339	0.78	2.506
ZNF492	0.133	0.296	0.448	0.654	1.142	0.338	0.419	0.675	0.639	2.041
ZNF484	0.079	0.295	0.267	0.79	1.082	0.32	0.257	0.798	0.606	1.93
ZNF480	-0.279	0.298	-0.936	0.349	0.757	0.225	-1.079	0.28	0.422	1.356
ZNF473	0.143	0.296	0.485	0.628	1.154	0.341	0.452	0.651	0.647	2.06
ZNF471	-0.197	0.295	-0.667	0.505	0.821	0.243	-0.737	0.461	0.46	1.465
ZNF468	0.658	0.307	2.145	0.032	1.93	0.592	1.572	0.116	1.058	3.521
ZNF467	0.007	0.296	0.023	0.982	1.007	0.298	0.023	0.982	0.564	1.798
ZNF460	-0.101	0.295	-0.343	0.732	0.904	0.267	-0.361	0.718	0.506	1.612
ZNF451	-0.017	0.295	-0.059	0.953	0.983	0.29	-0.06	0.952	0.551	1.753
ZNF45	0.017	0.295	0.056	0.955	1.017	0.3	0.056	0.956	0.57	1.814
ZNF446	-0.352	0.3	-1.175	0.24	0.703	0.211	-1.408	0.159	0.391	1.265
ZNF444	0.232	0.297	0.78	0.436	1.261	0.375	0.696	0.487	0.704	2.259
ZNF443	-0.132	0.296	-0.447	0.655	0.876	0.259	-0.478	0.633	0.49	1.566
ZNF442	-0.352	0.3	-1.173	0.241	0.704	0.211	-1.405	0.16	0.391	1.266
ZNF440	0.331	0.298	1.113	0.266	1.393	0.415	0.948	0.343	0.777	2.496
ZNF432	-0.034	0.295	-0.115	0.908	0.966	0.285	-0.117	0.906	0.542	1.724
ZNF430	-0.396	0.298	-1.329	0.184	0.673	0.2	-1.63	0.103	0.376	1.207
ZNF43	0.047	0.295	0.16	0.873	1.048	0.31	0.156	0.876	0.588	1.87
ZNF428	0.348	0.298	1.167	0.243	1.416	0.423	0.985	0.324	0.789	2.542
ZNF426	-0.211	0.296	-0.712	0.476	0.81	0.24	-0.793	0.428	0.453	1.447
ZNF423	-0.678	0.306	-2.216	0.027	0.508	0.155	-3.17	0.002	0.279	0.925
ZNF419	-0.262	0.298	-0.88	0.379	0.77	0.229	-1.006	0.314	0.429	1.379
ZNF415	-0.153	0.297	-0.515	0.607	0.858	0.255	-0.556	0.578	0.48	1.535
ZNF41	0.27	0.297	0.912	0.362	1.31	0.389	0.799	0.424	0.733	2.343
ZNF408	0.227	0.297	0.767	0.443	1.255	0.373	0.686	0.493	0.702	2.246
ZNF407	-0.446	0.302	-1.475	0.14	0.64	0.194	-1.859	0.063	0.354	1.158
ZNF395	-0.311	0.298	-1.043	0.297	0.733	0.218	-1.224	0.221	0.409	1.314
ZNF394	-0.343	0.304	-1.126	0.26	0.71	0.216	-1.343	0.179	0.391	1.289
ZNF391	-0.195	0.297	-0.658	0.51	0.823	0.244	-0.727	0.467	0.46	1.472
ZNF385D	0.569	0.306	1.864	0.062	1.767	0.54	1.421	0.155	0.971	3.216

ZNF384	-0.33	0.298	-1.108	0.268	0.719	0.214	-1.313	0.189	0.401	1.289
ZNF37BP	0.093	0.296	0.316	0.752	1.098	0.325	0.302	0.763	0.615	1.96
ZNF37A	0.298	0.298	1.002	0.317	1.348	0.401	0.866	0.387	0.752	2.416
ZNF365	-0.114	0.296	-0.386	0.7	0.892	0.264	-0.409	0.683	0.5	1.592
ZNF362	-0.085	0.295	-0.289	0.773	0.918	0.271	-0.301	0.763	0.515	1.638
ZNF358	0.017	0.297	0.057	0.954	1.017	0.302	0.057	0.955	0.568	1.822
ZNF354A	-0.058	0.296	-0.196	0.845	0.944	0.279	-0.201	0.84	0.529	1.685
ZNF350	0.515	0.303	1.7	0.089	1.674	0.507	1.329	0.184	0.924	3.031
ZNF35	0.654	0.305	2.146	0.032	1.924	0.587	1.575	0.115	1.058	3.498
ZNF345	-0.244	0.298	-0.821	0.412	0.783	0.233	-0.93	0.353	0.437	1.404
ZNF343	0.129	0.295	0.435	0.663	1.137	0.336	0.409	0.683	0.637	2.029
ZNF34	0.457	0.3	1.523	0.128	1.579	0.473	1.223	0.221	0.877	2.842
ZNF33B	0.079	0.295	0.267	0.789	1.082	0.319	0.257	0.797	0.607	1.93
ZNF337	0.092	0.299	0.308	0.758	1.096	0.328	0.294	0.769	0.611	1.969
ZNF335	-0.136	0.297	-0.456	0.648	0.873	0.259	-0.489	0.625	0.488	1.563
ZNF334	-0.048	0.296	-0.163	0.87	0.953	0.282	-0.167	0.867	0.534	1.701
ZNF331	-0.398	0.3	-1.328	0.184	0.671	0.201	-1.632	0.103	0.373	1.209
ZNF330	-0.09	0.297	-0.304	0.761	0.914	0.271	-0.318	0.75	0.511	1.634
ZNF329	0.157	0.297	0.528	0.597	1.17	0.347	0.489	0.625	0.654	2.092
ZNF324B	0.527	0.3	1.755	0.079	1.694	0.509	1.364	0.173	0.94	3.052
ZNF324	-0.147	0.297	-0.496	0.62	0.863	0.256	-0.534	0.593	0.483	1.544
ZNF322	-0.198	0.298	-0.666	0.505	0.82	0.244	-0.737	0.461	0.458	1.47
ZNF32	0.14	0.295	0.473	0.636	1.15	0.34	0.442	0.659	0.645	2.051
ZNF318	0.239	0.298	0.802	0.423	1.27	0.378	0.713	0.476	0.708	2.278
ZNF304	-0.351	0.3	-1.172	0.241	0.704	0.211	-1.405	0.16	0.391	1.266
ZNF302	-0.131	0.297	-0.441	0.66	0.877	0.26	-0.471	0.638	0.491	1.57
ZNF3	-0.005	0.296	-0.018	0.985	0.995	0.294	-0.018	0.985	0.557	1.776
ZNF292	0.287	0.296	0.97	0.332	1.333	0.395	0.843	0.399	0.746	2.382
ZNF287	0.413	0.3	1.375	0.169	1.511	0.454	1.126	0.26	0.839	2.721
ZNF286A	-0.531	0.303	-1.754	0.079	0.588	0.178	-2.314	0.021	0.325	1.064
ZNF282	-0.309	0.3	-1.03	0.303	0.734	0.22	-1.207	0.228	0.408	1.321
ZNF281	-0.028	0.296	-0.095	0.924	0.972	0.287	-0.097	0.923	0.545	1.735
ZNF280B	0.537	0.306	1.757	0.079	1.711	0.523	1.359	0.174	0.94	3.116
ZNF280A	-0.242	0.297	-0.813	0.416	0.785	0.233	-0.92	0.358	0.439	1.406
ZNF277	-0.656	0.307	-2.139	0.032	0.519	0.159	-3.023	0.003	0.285	0.947
ZNF276	-0.057	0.295	-0.192	0.848	0.945	0.279	-0.198	0.843	0.53	1.685
ZNF274	0.089	0.297	0.3	0.764	1.093	0.324	0.287	0.774	0.611	1.954
ZNF273	-0.504	0.302	-1.666	0.096	0.604	0.183	-2.166	0.03	0.334	1.093

ZNF271P	-0.144	0.296	-0.485	0.628	0.866	0.257	-0.521	0.602	0.485	1.548
ZNF268	0.522	0.302	1.728	0.084	1.685	0.509	1.346	0.178	0.932	3.046
ZNF267	-0.131	0.295	-0.443	0.658	0.877	0.259	-0.473	0.636	0.492	1.565
ZNF266	-0.565	0.302	-1.867	0.062	0.569	0.172	-2.509	0.012	0.314	1.029
ZNF264	0.046	0.295	0.155	0.877	1.047	0.309	0.151	0.88	0.587	1.867
ZNF26	-0.158	0.296	-0.534	0.593	0.854	0.252	-0.578	0.563	0.478	1.524
ZNF259P1	-0.236	0.299	-0.791	0.429	0.789	0.236	-0.892	0.372	0.44	1.418
ZNF257	0.326	0.297	1.097	0.273	1.385	0.411	0.936	0.349	0.774	2.479
ZNF254	-0.032	0.298	-0.108	0.914	0.968	0.288	-0.11	0.912	0.54	1.735
ZNF253	-0.572	0.306	-1.872	0.061	0.564	0.172	-2.527	0.012	0.31	1.027
ZNF250	1.039	0.321	3.233	0.001	2.827	0.908	2.011	0.044	1.506	5.307
ZNF248	0.001	0.295	0.003	0.998	1.001	0.295	0.003	0.998	0.561	1.785
ZNF24	-0.731	0.311	-2.354	0.019	0.481	0.15	-3.468	0.001	0.262	0.885
ZNF239	0.303	0.298	1.019	0.308	1.354	0.403	0.879	0.379	0.756	2.427
ZNF236	-0.048	0.296	-0.161	0.872	0.953	0.282	-0.165	0.869	0.534	1.702
ZNF235	-0.056	0.296	-0.188	0.851	0.946	0.28	-0.193	0.847	0.529	1.691
ZNF234	-0.119	0.296	-0.401	0.688	0.888	0.263	-0.426	0.67	0.497	1.587
ZNF232	0.474	0.3	1.583	0.113	1.607	0.482	1.261	0.207	0.893	2.892
ZNF230	-0.197	0.298	-0.659	0.51	0.821	0.245	-0.728	0.467	0.458	1.475
ZNF23	0.03	0.295	0.102	0.919	1.03	0.304	0.1	0.92	0.578	1.837
ZNF227	-0.559	0.31	-1.802	0.072	0.572	0.177	-2.415	0.016	0.311	1.05
ZNF226	-0.296	0.304	-0.975	0.329	0.744	0.226	-1.135	0.256	0.41	1.349
ZNF225	-0.241	0.298	-0.808	0.419	0.786	0.234	-0.914	0.361	0.438	1.41
ZNF224	-0.013	0.296	-0.043	0.966	0.987	0.292	-0.043	0.966	0.553	1.763
ZNF223	-0.199	0.298	-0.669	0.504	0.819	0.244	-0.74	0.459	0.457	1.469
ZNF222	-0.079	0.298	-0.267	0.79	0.924	0.275	-0.277	0.781	0.515	1.655
ZNF221	-0.222	0.301	-0.737	0.461	0.801	0.241	-0.825	0.409	0.444	1.445
ZNF22	0.476	0.3	1.586	0.113	1.609	0.482	1.262	0.207	0.894	2.896
ZNF219	0.287	0.298	0.965	0.335	1.332	0.396	0.839	0.402	0.744	2.387
ZNF217	0.109	0.295	0.369	0.712	1.115	0.329	0.35	0.727	0.625	1.989
ZNF215	-0.469	0.303	-1.549	0.121	0.626	0.189	-1.976	0.048	0.346	1.132
ZNF214	0.093	0.297	0.312	0.755	1.097	0.326	0.298	0.766	0.613	1.965
ZNF213-AS1	-0.324	0.304	-1.064	0.287	0.723	0.22	-1.257	0.209	0.398	1.314
ZNF212	0.739	0.306	2.417	0.016	2.094	0.64	1.708	0.088	1.15	3.813
ZNF211	-0.434	0.303	-1.434	0.152	0.648	0.196	-1.795	0.073	0.358	1.173
ZNF207	-0.012	0.295	-0.041	0.967	0.988	0.292	-0.042	0.967	0.554	1.762
ZNF205-AS1	0.158	0.295	0.535	0.593	1.171	0.346	0.495	0.621	0.657	2.089
ZNF205	-0.08	0.298	-0.269	0.788	0.923	0.275	-0.28	0.779	0.514	1.656

ZNF204P	0.411	0.298	1.379	0.168	1.509	0.45	1.131	0.258	0.841	2.707
ZNF202	-0.458	0.301	-1.523	0.128	0.632	0.19	-1.931	0.053	0.35	1.141
ZNF200	-0.049	0.296	-0.167	0.868	0.952	0.282	-0.171	0.864	0.533	1.7
ZNF197	0.849	0.315	2.697	0.007	2.338	0.736	1.817	0.069	1.261	4.334
ZNF195	-0.395	0.3	-1.319	0.187	0.674	0.202	-1.618	0.106	0.374	1.212
ZNF189	-0.403	0.3	-1.346	0.178	0.668	0.2	-1.657	0.097	0.371	1.202
ZNF185	0.319	0.3	1.065	0.287	1.376	0.412	0.912	0.362	0.765	2.474
ZNF184	0.01	0.295	0.034	0.973	1.01	0.298	0.034	0.973	0.567	1.801
ZNF180	-0.072	0.296	-0.242	0.809	0.931	0.275	-0.251	0.802	0.521	1.662
ZNF175	-0.309	0.298	-1.037	0.3	0.734	0.219	-1.215	0.224	0.41	1.316
ZNF174	-0.212	0.297	-0.714	0.475	0.809	0.24	-0.796	0.426	0.452	1.447
ZNF165	0.589	0.303	1.947	0.052	1.802	0.545	1.471	0.141	0.996	3.261
ZNF160	0.116	0.296	0.391	0.696	1.123	0.333	0.369	0.712	0.628	2.008
ZNF16	0.419	0.3	1.398	0.162	1.52	0.455	1.142	0.253	0.845	2.735
ZNF157	-0.148	0.297	-0.5	0.617	0.862	0.256	-0.538	0.59	0.482	1.542
ZNF155	-0.49	0.3	-1.633	0.103	0.613	0.184	-2.106	0.035	0.34	1.103
ZNF154	-0.123	0.296	-0.417	0.677	0.884	0.262	-0.444	0.657	0.495	1.579
ZNF148	1.002	0.322	3.111	0.002	2.724	0.877	1.965	0.049	1.449	5.12
ZNF146	-0.204	0.298	-0.685	0.493	0.815	0.243	-0.76	0.447	0.455	1.462
ZNF143	-0.339	0.298	-1.138	0.255	0.713	0.212	-1.354	0.176	0.397	1.277
ZNF142	0.317	0.3	1.058	0.29	1.373	0.411	0.907	0.365	0.763	2.47
ZNF141	0.165	0.295	0.56	0.575	1.18	0.348	0.516	0.606	0.661	2.105
ZNF140	-0.354	0.303	-1.168	0.243	0.702	0.213	-1.402	0.161	0.388	1.271
ZNF14	0.691	0.306	2.257	0.024	1.995	0.611	1.63	0.103	1.095	3.635
ZNF137P	0.335	0.298	1.126	0.26	1.399	0.417	0.956	0.339	0.78	2.508
ZNF136	-0.37	0.3	-1.234	0.217	0.691	0.207	-1.493	0.135	0.384	1.243
ZNF135	-0.034	0.295	-0.114	0.909	0.967	0.285	-0.116	0.908	0.542	1.724
ZNF134	-0.212	0.299	-0.709	0.478	0.809	0.242	-0.79	0.43	0.451	1.453
ZNF133	0.401	0.3	1.337	0.181	1.493	0.448	1.102	0.271	0.83	2.688
ZNF132	-0.661	0.31	-2.132	0.033	0.516	0.16	-3.022	0.003	0.281	0.948
ZNF124	0.277	0.296	0.937	0.349	1.32	0.391	0.818	0.413	0.739	2.359
ZNF12	-0.234	0.296	-0.79	0.429	0.791	0.234	-0.89	0.373	0.443	1.414
ZNF117	0.548	0.306	1.794	0.073	1.73	0.529	1.381	0.167	0.951	3.15
ZNF112	0.033	0.296	0.113	0.91	1.034	0.306	0.111	0.912	0.579	1.847
ZNF107	-0.043	0.295	-0.145	0.885	0.958	0.283	-0.148	0.882	0.537	1.71
ZNF106	-0.488	0.3	-1.626	0.104	0.614	0.184	-2.097	0.036	0.341	1.105
ZNF10	-0.51	0.301	-1.697	0.09	0.6	0.18	-2.214	0.027	0.333	1.082
ZMYND8	-0.028	0.295	-0.095	0.924	0.972	0.287	-0.097	0.923	0.545	1.733

ZMYND11	-0.032	0.296	-0.106	0.915	0.969	0.287	-0.108	0.914	0.542	1.732
ZMYND10	-0.481	0.306	-1.572	0.116	0.618	0.189	-2.019	0.043	0.339	1.126
ZMYM6	-0.399	0.3	-1.33	0.183	0.671	0.201	-1.634	0.102	0.373	1.208
ZMYM5	0.523	0.303	1.723	0.085	1.686	0.512	1.342	0.18	0.931	3.056
ZMYM4	-0.112	0.296	-0.377	0.706	0.894	0.265	-0.399	0.69	0.5	1.599
ZMYM3	0.604	0.303	1.996	0.046	1.83	0.554	1.498	0.134	1.011	3.314
ZMYM2	1.09	0.328	3.321	0.001	2.973	0.976	2.023	0.043	1.563	5.657
ZMYM1	0.02	0.295	0.069	0.945	1.021	0.301	0.069	0.945	0.572	1.821
ZMPSTE24	0.143	0.297	0.482	0.63	1.154	0.343	0.449	0.653	0.644	2.066
ZMIZ2	0.248	0.296	0.837	0.403	1.281	0.379	0.741	0.459	0.717	2.289
ZMIZ1	0.202	0.298	0.68	0.497	1.224	0.364	0.615	0.538	0.683	2.194
ZMAT5	0.144	0.295	0.488	0.625	1.155	0.341	0.455	0.649	0.647	2.061
ZMAT4	-0.2	0.298	-0.671	0.502	0.819	0.244	-0.743	0.458	0.457	1.468
ZMAT3	-0.94	0.316	-2.973	0.003	0.391	0.123	-4.934	0	0.21	0.726
ZKSCAN8	0.404	0.298	1.356	0.175	1.497	0.446	1.115	0.265	0.835	2.685
ZKSCAN7	-0.463	0.303	-1.531	0.126	0.629	0.19	-1.948	0.051	0.348	1.138
ZKSCAN5	-0.557	0.306	-1.823	0.068	0.573	0.175	-2.44	0.015	0.315	1.043
ZKSCAN4	0.071	0.295	0.242	0.809	1.074	0.317	0.233	0.815	0.602	1.916
ZKSCAN3	0.587	0.303	1.939	0.053	1.798	0.544	1.466	0.143	0.994	3.255
ZKSCAN1	0.058	0.297	0.195	0.845	1.06	0.314	0.19	0.85	0.592	1.895
ZIC4	0.149	0.297	0.501	0.616	1.16	0.345	0.466	0.641	0.648	2.077
ZIC3	-0.457	0.302	-1.511	0.131	0.633	0.192	-1.915	0.055	0.35	1.145
ZIC1	-0.399	0.3	-1.33	0.184	0.671	0.201	-1.634	0.102	0.373	1.208
ZHX3	-0.68	0.306	-2.221	0.026	0.506	0.155	-3.181	0.001	0.278	0.923
ZHX2	-0.352	0.3	-1.174	0.241	0.703	0.211	-1.407	0.159	0.39	1.266
ZGPAT	0.038	0.295	0.129	0.897	1.039	0.307	0.127	0.899	0.583	1.852
ZG16	0.158	0.296	0.532	0.595	1.171	0.347	0.492	0.623	0.655	2.092
ZFYVE9	0.479	0.303	1.582	0.114	1.615	0.489	1.257	0.209	0.892	2.923
ZFYVE26	0.352	0.299	1.179	0.239	1.422	0.425	0.993	0.321	0.792	2.554
ZFYVE21	-0.29	0.301	-0.963	0.336	0.749	0.225	-1.116	0.264	0.415	1.35
ZFYVE16	-0.602	0.31	-1.942	0.052	0.548	0.17	-2.665	0.008	0.298	1.005
ZFY	0.073	0.295	0.248	0.804	1.076	0.318	0.239	0.811	0.603	1.92
ZFX	-0.276	0.298	-0.927	0.354	0.759	0.226	-1.068	0.286	0.423	1.36
ZFR2	0.067	0.296	0.228	0.82	1.07	0.316	0.22	0.826	0.599	1.91
ZFR	0.149	0.296	0.504	0.615	1.161	0.344	0.468	0.64	0.65	2.074
ZFPM2	-0.515	0.302	-1.705	0.088	0.597	0.181	-2.23	0.026	0.33	1.08
ZFPL1	0.005	0.295	0.016	0.987	1.005	0.297	0.016	0.987	0.563	1.792
ZFP69B	0.616	0.306	2.009	0.044	1.851	0.567	1.5	0.134	1.015	3.375

ZFP64	0.229	0.296	0.774	0.439	1.258	0.372	0.692	0.489	0.704	2.247
ZFP37	0.349	0.3	1.165	0.244	1.418	0.425	0.983	0.325	0.788	2.552
ZFP36L2	-0.733	0.306	-2.395	0.017	0.481	0.147	-3.532	0	0.264	0.875
ZFP36L1	-0.113	0.295	-0.382	0.702	0.893	0.264	-0.405	0.686	0.501	1.593
ZFP36	-0.174	0.296	-0.587	0.558	0.841	0.249	-0.641	0.522	0.471	1.502
ZFP30	-0.1	0.296	-0.337	0.736	0.905	0.268	-0.355	0.723	0.507	1.616
ZFP2	-0.431	0.303	-1.423	0.155	0.65	0.197	-1.779	0.075	0.359	1.177
ZFHX4	-0.569	0.302	-1.88	0.06	0.566	0.171	-2.532	0.011	0.313	1.025
ZFHX3	-0.183	0.297	-0.617	0.537	0.833	0.247	-0.677	0.498	0.466	1.489
ZFHX2	0.536	0.301	1.783	0.075	1.709	0.513	1.38	0.168	0.948	3.079
ZFC3H1	-0.072	0.296	-0.242	0.809	0.931	0.276	-0.25	0.802	0.521	1.663
ZFAND6	-0.437	0.302	-1.446	0.148	0.646	0.195	-1.814	0.07	0.357	1.168
ZFAND5	-0.324	0.298	-1.087	0.277	0.723	0.216	-1.283	0.199	0.403	1.298
ZFAND3	-1.002	0.317	-3.164	0.002	0.367	0.116	-5.445	0	0.197	0.683
ZFAND1	0.128	0.296	0.433	0.665	1.136	0.336	0.406	0.685	0.637	2.028
ZER1	-0.794	0.31	-2.559	0.011	0.452	0.14	-3.907	0	0.246	0.83
ZEB2	-0.455	0.3	-1.515	0.13	0.635	0.191	-1.918	0.055	0.352	1.143
ZEB1	-0.661	0.306	-2.162	0.031	0.516	0.158	-3.065	0.002	0.283	0.94
ZDHHC8P1	-0.001	0.295	-0.005	0.996	0.999	0.295	-0.005	0.996	0.56	1.782
ZDHHC7	-0.023	0.295	-0.077	0.938	0.977	0.289	-0.078	0.938	0.548	1.743
ZDHHC6	0.293	0.3	0.976	0.329	1.34	0.402	0.846	0.397	0.745	2.411
ZDHHC4	-0.12	0.295	-0.405	0.686	0.887	0.262	-0.43	0.667	0.497	1.583
ZDHHC3	-0.33	0.3	-1.101	0.271	0.719	0.216	-1.304	0.192	0.399	1.294
ZDHHC18	0.191	0.296	0.645	0.519	1.211	0.359	0.587	0.557	0.677	2.164
ZDHHC17	-0.39	0.298	-1.311	0.19	0.677	0.202	-1.603	0.109	0.378	1.213
ZDHHC14	-0.17	0.3	-0.568	0.57	0.844	0.253	-0.619	0.536	0.469	1.518
ZDHHC13	1.288	0.332	3.882	0	3.625	1.203	2.183	0.029	1.892	6.946
ZCWPW1	-0.358	0.3	-1.193	0.233	0.699	0.21	-1.434	0.152	0.389	1.259
ZCCHC8	-0.598	0.306	-1.956	0.051	0.55	0.168	-2.676	0.007	0.302	1.001
ZCCHC4	0.021	0.296	0.072	0.942	1.022	0.302	0.072	0.943	0.572	1.824
ZCCHC24	-0.531	0.302	-1.757	0.079	0.588	0.178	-2.318	0.02	0.325	1.063
ZCCHC2	0.137	0.295	0.463	0.643	1.147	0.339	0.433	0.665	0.643	2.046
ZCCHC14	-0.297	0.298	-0.997	0.319	0.743	0.221	-1.16	0.246	0.415	1.332
ZCCHC10	-0.279	0.301	-0.925	0.355	0.757	0.228	-1.067	0.286	0.419	1.366
ZC4H2	0.323	0.298	1.085	0.278	1.381	0.411	0.927	0.354	0.771	2.474
ZC3HAV1	0.045	0.298	0.149	0.881	1.046	0.312	0.146	0.884	0.583	1.875
ZC3H7B	0.304	0.298	1.021	0.307	1.355	0.403	0.881	0.378	0.756	2.428
ZC3H7A	-0.335	0.298	-1.124	0.261	0.716	0.213	-1.335	0.182	0.399	1.282

ZC3H4	0.884	0.315	2.804	0.005	2.421	0.763	1.862	0.063	1.305	4.491
ZC3H3	0.145	0.296	0.491	0.623	1.156	0.342	0.457	0.648	0.648	2.064
ZC3H15	0.519	0.302	1.715	0.086	1.68	0.508	1.338	0.181	0.929	3.038
ZC3H14	-0.127	0.296	-0.427	0.669	0.881	0.261	-0.455	0.649	0.493	1.575
ZC3H13	-0.141	0.296	-0.477	0.633	0.868	0.257	-0.512	0.608	0.486	1.552
ZC2HC1C	0.309	0.297	1.042	0.297	1.363	0.404	0.897	0.37	0.762	2.438
ZC2HC1A	0.712	0.306	2.327	0.02	2.038	0.624	1.665	0.096	1.119	3.713
ZBTB7C	-0.326	0.3	-1.087	0.277	0.722	0.217	-1.285	0.199	0.401	1.299
ZBTB7B	-0.403	0.302	-1.334	0.182	0.668	0.202	-1.643	0.1	0.369	1.209
ZBTB7A	0.226	0.297	0.762	0.446	1.254	0.372	0.682	0.495	0.701	2.244
ZBTB6	-0.108	0.295	-0.365	0.715	0.898	0.265	-0.385	0.7	0.503	1.602
ZBTB5	-0.103	0.296	-0.348	0.728	0.902	0.267	-0.366	0.714	0.505	1.611
ZBTB48	-1.106	0.347	-3.186	0.001	0.331	0.115	-5.825	0	0.168	0.653
ZBTB44	-0.361	0.299	-1.209	0.227	0.697	0.208	-1.456	0.145	0.388	1.252
ZBTB43	0.491	0.302	1.624	0.104	1.634	0.494	1.284	0.199	0.904	2.955
ZBTB40	-0.189	0.296	-0.638	0.524	0.828	0.245	-0.702	0.483	0.463	1.479
ZBTB39	0.427	0.3	1.424	0.154	1.532	0.459	1.159	0.246	0.852	2.756
ZBTB38	0.295	0.298	0.991	0.321	1.343	0.4	0.859	0.391	0.75	2.406
ZBTB33	-0.142	0.295	-0.481	0.631	0.868	0.256	-0.516	0.606	0.486	1.548
ZBTB32	0.113	0.295	0.384	0.701	1.12	0.331	0.363	0.717	0.628	1.999
ZBTB3	0.07	0.295	0.237	0.812	1.073	0.317	0.229	0.819	0.601	1.914
ZBTB25	0.379	0.3	1.264	0.206	1.46	0.438	1.052	0.293	0.812	2.628
ZBTB24	0.459	0.307	1.495	0.135	1.582	0.486	1.199	0.231	0.867	2.888
ZBTB22	-0.016	0.296	-0.055	0.956	0.984	0.291	-0.055	0.956	0.551	1.757
ZBTB20	-0.23	0.296	-0.775	0.438	0.795	0.235	-0.872	0.383	0.445	1.42
ZBTB18	-0.062	0.295	-0.209	0.834	0.94	0.278	-0.216	0.829	0.527	1.677
ZBTB17	-0.164	0.296	-0.554	0.579	0.849	0.251	-0.602	0.547	0.475	1.516
ZBTB16	-0.33	0.3	-1.102	0.271	0.719	0.215	-1.305	0.192	0.399	1.293
ZBTB14	0.126	0.296	0.425	0.671	1.134	0.336	0.399	0.69	0.634	2.028
ZBTB11	-0.261	0.296	-0.881	0.378	0.77	0.228	-1.007	0.314	0.431	1.377
ZBTB10	0.354	0.3	1.182	0.237	1.425	0.427	0.995	0.32	0.792	2.565
ZBTB1	-0.49	0.302	-1.621	0.105	0.613	0.185	-2.091	0.037	0.339	1.108
ZBP1	0.183	0.296	0.617	0.537	1.201	0.356	0.564	0.573	0.672	2.146
ZBED8	-0.437	0.302	-1.445	0.148	0.646	0.195	-1.812	0.07	0.357	1.168
ZBED5	-0.208	0.296	-0.701	0.483	0.812	0.241	-0.779	0.436	0.455	1.452
ZBED4	0.002	0.296	0.005	0.996	1.002	0.297	0.005	0.996	0.56	1.79
ZBED2	-0.304	0.297	-1.023	0.306	0.738	0.219	-1.195	0.232	0.412	1.321
ZBED1	0.041	0.296	0.138	0.89	1.042	0.308	0.136	0.892	0.583	1.861

ZBBX	0.05	0.295	0.17	0.865	1.051	0.311	0.165	0.869	0.589	1.876
ZAP70	-0.751	0.31	-2.421	0.015	0.472	0.146	-3.608	0	0.257	0.867
YY1AP1	0.361	0.299	1.208	0.227	1.435	0.428	1.014	0.31	0.799	2.576
YY1	0.199	0.299	0.665	0.506	1.22	0.365	0.603	0.547	0.679	2.191
YWHAZ	0.455	0.303	1.502	0.133	1.576	0.477	1.207	0.227	0.871	2.852
YWHAQ	0.417	0.3	1.393	0.163	1.518	0.455	1.139	0.255	0.844	2.731
YWHAH	-0.429	0.302	-1.42	0.155	0.651	0.197	-1.774	0.076	0.36	1.177
YWHAE	-0.061	0.296	-0.207	0.836	0.941	0.278	-0.213	0.831	0.527	1.68
YWHAB	0.154	0.299	0.514	0.607	1.166	0.349	0.477	0.634	0.649	2.097
YTHDF3	0.396	0.302	1.309	0.191	1.486	0.449	1.081	0.28	0.821	2.687
YTHDF2	0.052	0.297	0.177	0.86	1.054	0.313	0.172	0.863	0.589	1.885
YTHDF1	0.816	0.315	2.593	0.01	2.261	0.712	1.772	0.076	1.22	4.191
YTHDC2	-0.012	0.296	-0.041	0.968	0.988	0.292	-0.041	0.967	0.554	1.763
YTHDC1	0.255	0.301	0.849	0.396	1.291	0.388	0.749	0.454	0.716	2.328
YRDC	0.683	0.306	2.235	0.025	1.98	0.605	1.619	0.105	1.088	3.604
YPEL5	-0.435	0.302	-1.44	0.15	0.647	0.196	-1.804	0.071	0.358	1.17
YPEL1	0.615	0.306	2.013	0.044	1.85	0.565	1.504	0.133	1.016	3.367
YOD1	-0.19	0.296	-0.64	0.522	0.827	0.245	-0.705	0.481	0.463	1.479
YME1L1	0.466	0.301	1.549	0.121	1.593	0.479	1.238	0.216	0.884	2.871
YLPM1	-0.025	0.295	-0.085	0.932	0.975	0.288	-0.086	0.931	0.547	1.739
YKT6	0.241	0.295	0.817	0.414	1.273	0.376	0.726	0.468	0.713	2.272
YJU2	-0.257	0.301	-0.855	0.393	0.773	0.233	-0.975	0.33	0.429	1.394
YIPF6	0.618	0.304	2.034	0.042	1.855	0.564	1.518	0.129	1.023	3.365
YIPF5	-0.331	0.3	-1.104	0.27	0.718	0.215	-1.309	0.191	0.399	1.292
YIPF4	-0.152	0.296	-0.513	0.608	0.859	0.254	-0.554	0.579	0.481	1.535
YIPF3	0.341	0.298	1.144	0.252	1.406	0.418	0.97	0.332	0.785	2.519
YIPF2	0.019	0.295	0.065	0.948	1.019	0.301	0.064	0.949	0.571	1.818
YIPF1	-0.357	0.3	-1.191	0.234	0.7	0.21	-1.43	0.153	0.389	1.259
YIF1A	0.386	0.298	1.298	0.194	1.472	0.438	1.077	0.282	0.821	2.637
YES1	0.091	0.298	0.306	0.76	1.095	0.326	0.292	0.77	0.611	1.963
YEATS4	0.122	0.296	0.411	0.681	1.13	0.335	0.387	0.699	0.632	2.019
YEATS2	0.89	0.321	2.778	0.005	2.436	0.781	1.839	0.066	1.3	4.566
YBX3	-0.034	0.296	-0.115	0.908	0.967	0.286	-0.117	0.907	0.541	1.726
YBX2	0.386	0.298	1.299	0.194	1.472	0.438	1.077	0.281	0.821	2.637
YBX1	-0.11	0.295	-0.374	0.709	0.895	0.265	-0.395	0.693	0.502	1.598
YARS2	0.377	0.3	1.257	0.209	1.458	0.437	1.047	0.295	0.81	2.624
YARS1	-0.151	0.296	-0.508	0.612	0.86	0.255	-0.548	0.584	0.481	1.538
YAP1	0.629	0.306	2.056	0.04	1.876	0.574	1.526	0.127	1.03	3.417

YAF2	-0.144	0.296	-0.486	0.627	0.866	0.256	-0.523	0.601	0.485	1.547
XYLT2	0.112	0.297	0.377	0.706	1.118	0.332	0.357	0.721	0.625	2.001
XYLT1	-0.189	0.296	-0.64	0.522	0.828	0.245	-0.705	0.481	0.464	1.477
XYLB	0.413	0.298	1.384	0.167	1.511	0.451	1.134	0.257	0.842	2.711
XRCC6	-0.124	0.296	-0.419	0.675	0.883	0.261	-0.446	0.655	0.495	1.578
XRCC5	-0.198	0.296	-0.668	0.504	0.82	0.243	-0.738	0.46	0.459	1.467
XRCC4	0.453	0.298	1.519	0.129	1.573	0.469	1.221	0.222	0.877	2.822
XRCC3	0.534	0.306	1.745	0.081	1.705	0.521	1.352	0.176	0.936	3.105
XRCC2	0.769	0.31	2.482	0.013	2.158	0.669	1.731	0.083	1.176	3.961
XRCC1	0.23	0.296	0.777	0.437	1.258	0.372	0.694	0.488	0.705	2.247
XPOT	0.373	0.3	1.242	0.214	1.452	0.436	1.037	0.3	0.806	2.617
XPO7	-0.053	0.295	-0.18	0.857	0.948	0.28	-0.185	0.853	0.532	1.691
XPO6	0.34	0.3	1.136	0.256	1.406	0.421	0.963	0.336	0.781	2.529
XPO4	0.283	0.296	0.956	0.339	1.327	0.393	0.832	0.405	0.743	2.372
XPO1	0.306	0.302	1.014	0.311	1.358	0.41	0.874	0.382	0.752	2.453
XPNPEP3	0.304	0.298	1.023	0.306	1.356	0.403	0.882	0.378	0.757	2.429
XPNPEP2	-0.09	0.295	-0.304	0.761	0.914	0.27	-0.319	0.75	0.512	1.631
XPNPEP1	0.073	0.296	0.246	0.806	1.075	0.318	0.237	0.813	0.602	1.921
XPC	-0.098	0.295	-0.33	0.741	0.907	0.268	-0.347	0.728	0.509	1.618
XPA	0.35	0.298	1.174	0.24	1.419	0.423	0.991	0.322	0.791	2.546
XKR8	-0.577	0.304	-1.899	0.058	0.562	0.171	-2.569	0.01	0.31	1.019
XK	0.081	0.295	0.273	0.785	1.084	0.32	0.262	0.793	0.608	1.934
XIST	-0.318	0.298	-1.068	0.286	0.728	0.217	-1.257	0.209	0.406	1.304
XIAP	-0.148	0.296	-0.502	0.616	0.862	0.255	-0.541	0.588	0.483	1.539
XDH	0.082	0.297	0.277	0.782	1.086	0.323	0.266	0.79	0.606	1.945
XCR1	0.202	0.296	0.683	0.495	1.224	0.363	0.618	0.536	0.685	2.188
XCL1	-0.107	0.296	-0.362	0.717	0.899	0.266	-0.382	0.703	0.504	1.604
XBP1	-0.159	0.296	-0.537	0.591	0.853	0.253	-0.583	0.56	0.477	1.524
XAF1	0.394	0.301	1.309	0.19	1.483	0.446	1.082	0.279	0.822	2.673
XAB2	-0.661	0.31	-2.133	0.033	0.516	0.16	-3.022	0.003	0.281	0.948
WWTR1	-0.45	0.298	-1.51	0.131	0.637	0.19	-1.907	0.056	0.355	1.144
WWP2	0.651	0.303	2.148	0.032	1.917	0.581	1.579	0.114	1.059	3.471
WWP1	-0.251	0.298	-0.842	0.4	0.778	0.232	-0.957	0.339	0.434	1.395
WWOX	0.279	0.298	0.936	0.349	1.322	0.394	0.817	0.414	0.737	2.37
WWC3	-0.172	0.295	-0.584	0.559	0.842	0.249	-0.637	0.524	0.472	1.501
WWC1	0.229	0.296	0.772	0.44	1.257	0.372	0.69	0.49	0.703	2.246
WTAP	-0.282	0.3	-0.941	0.347	0.754	0.226	-1.087	0.277	0.419	1.357
WT1-AS	0.238	0.298	0.798	0.425	1.268	0.378	0.711	0.477	0.708	2.273

WT1	-0.009	0.296	-0.03	0.976	0.991	0.293	-0.03	0.976	0.555	1.77
WSCD2	-0.54	0.302	-1.784	0.074	0.583	0.176	-2.366	0.018	0.322	1.055
WSB2	-0.173	0.298	-0.58	0.562	0.841	0.251	-0.633	0.527	0.469	1.509
WSB1	0.128	0.296	0.433	0.665	1.137	0.337	0.406	0.685	0.636	2.032
WRNIP1	-0.066	0.296	-0.223	0.824	0.936	0.277	-0.23	0.818	0.525	1.671
WRN	0.123	0.297	0.416	0.678	1.131	0.336	0.391	0.696	0.632	2.024
WRAP73	0.018	0.3	0.061	0.952	1.018	0.305	0.06	0.952	0.566	1.832
WRAP53	0.061	0.296	0.207	0.836	1.063	0.315	0.201	0.841	0.595	1.899
WNT8B	0.444	0.299	1.485	0.137	1.56	0.467	1.199	0.23	0.868	2.803
WNT7B	-0.09	0.295	-0.303	0.762	0.914	0.27	-0.317	0.751	0.513	1.631
WNT7A	-0.072	0.297	-0.241	0.809	0.931	0.276	-0.25	0.803	0.52	1.665
WNT6	0.001	0.299	0.005	0.996	1.001	0.299	0.005	0.996	0.557	1.799
WNT5B	0.247	0.3	0.823	0.41	1.28	0.384	0.729	0.466	0.711	2.306
WNT5A	-0.481	0.302	-1.59	0.112	0.618	0.187	-2.042	0.041	0.342	1.118
WNT4	-0.873	0.328	-2.662	0.008	0.418	0.137	-4.252	0	0.22	0.794
WNT2B	0.14	0.299	0.468	0.64	1.15	0.343	0.437	0.662	0.641	2.064
WNT2	-0.244	0.296	-0.824	0.41	0.783	0.232	-0.934	0.351	0.438	1.4
WNT16	0.3	0.298	1.006	0.314	1.35	0.403	0.869	0.385	0.752	2.423
WNT11	0.091	0.295	0.309	0.757	1.096	0.324	0.295	0.768	0.614	1.954
WNT10B	0.634	0.31	2.048	0.041	1.885	0.584	1.516	0.129	1.027	3.459
WNT1	-0.052	0.295	-0.176	0.86	0.949	0.28	-0.181	0.856	0.532	1.693
WNK1	-0.857	0.315	-2.718	0.007	0.425	0.134	-4.3	0	0.229	0.787
WLS	-0.762	0.315	-2.421	0.015	0.467	0.147	-3.632	0	0.252	0.865
WIZ	0.731	0.31	2.358	0.018	2.077	0.644	1.673	0.094	1.131	3.813
WIPI2	0.427	0.3	1.42	0.155	1.532	0.46	1.156	0.248	0.85	2.76
WIPI1	-0.367	0.3	-1.224	0.221	0.693	0.208	-1.479	0.139	0.385	1.247
WIPF2	0.362	0.298	1.216	0.224	1.436	0.428	1.02	0.308	0.801	2.574
WIPF1	-0.784	0.306	-2.56	0.01	0.457	0.14	-3.887	0	0.251	0.832
WIF1	-0.127	0.296	-0.429	0.668	0.881	0.26	-0.458	0.647	0.494	1.572
WHRN	0.11	0.296	0.372	0.71	1.117	0.331	0.352	0.725	0.625	1.996
WFS1	0.115	0.295	0.389	0.697	1.122	0.331	0.368	0.713	0.629	2.001
WFDC8	0.407	0.3	1.356	0.175	1.502	0.45	1.114	0.265	0.834	2.703
WFDC2	0.258	0.296	0.871	0.384	1.294	0.383	0.768	0.443	0.724	2.313
WFDC1	-0.303	0.298	-1.015	0.31	0.739	0.22	-1.186	0.236	0.412	1.325
WEE1	-0.143	0.298	-0.48	0.631	0.867	0.259	-0.516	0.606	0.483	1.555
WDTC1	-0.168	0.296	-0.566	0.571	0.846	0.251	-0.616	0.538	0.473	1.511
WDR91	-0.327	0.3	-1.091	0.275	0.721	0.216	-1.29	0.197	0.401	1.297
WDR83OS	-0.339	0.304	-1.114	0.265	0.712	0.217	-1.327	0.185	0.392	1.294

WDR82	0.033	0.296	0.112	0.911	1.034	0.306	0.11	0.913	0.578	1.848
WDR77	0.388	0.3	1.294	0.195	1.474	0.441	1.073	0.283	0.819	2.651
WDR76	1.349	0.337	3.998	0	3.854	1.301	2.194	0.028	1.989	7.468
WDR74	0.098	0.295	0.331	0.741	1.103	0.326	0.315	0.753	0.618	1.968
WDR73	0.21	0.296	0.708	0.479	1.233	0.366	0.638	0.523	0.69	2.205
WDR70	0.979	0.321	3.049	0.002	2.661	0.854	1.945	0.052	1.418	4.992
WDR7	0.033	0.296	0.113	0.91	1.034	0.306	0.111	0.911	0.579	1.847
WDR62	0.434	0.3	1.448	0.148	1.544	0.463	1.174	0.24	0.858	2.778
WDR61	-0.043	0.297	-0.144	0.886	0.958	0.285	-0.147	0.883	0.535	1.715
WDR6	-0.634	0.304	-2.087	0.037	0.53	0.161	-2.914	0.004	0.292	0.962
WDR5B	0.133	0.297	0.449	0.653	1.142	0.339	0.421	0.674	0.639	2.043
WDR59	0.04	0.295	0.136	0.892	1.041	0.308	0.133	0.894	0.583	1.857
WDR55	0.452	0.302	1.496	0.135	1.572	0.475	1.203	0.229	0.869	2.842
WDR48	-0.218	0.298	-0.732	0.464	0.804	0.239	-0.818	0.414	0.449	1.441
WDR47	0.246	0.296	0.829	0.407	1.278	0.379	0.735	0.462	0.715	2.284
WDR46	0.172	0.296	0.58	0.562	1.188	0.352	0.533	0.594	0.664	2.123
WDR45B	0.497	0.306	1.624	0.104	1.644	0.503	1.28	0.201	0.902	2.995
WDR45	-0.577	0.31	-1.857	0.063	0.562	0.174	-2.512	0.012	0.306	1.032
WDR44	-0.339	0.303	-1.118	0.264	0.713	0.216	-1.331	0.183	0.394	1.29
WDR43	0.881	0.321	2.747	0.006	2.412	0.773	1.827	0.068	1.287	4.521
WDR41	0.05	0.296	0.169	0.865	1.052	0.312	0.165	0.869	0.588	1.879
WDR4	0.24	0.295	0.811	0.418	1.271	0.375	0.721	0.471	0.712	2.267
WDR37	-0.637	0.306	-2.084	0.037	0.529	0.162	-2.915	0.004	0.291	0.963
WDR3	0.488	0.303	1.613	0.107	1.629	0.493	1.276	0.202	0.9	2.948
WDR26	0.204	0.298	0.686	0.493	1.227	0.366	0.62	0.535	0.684	2.201
WDR25	-0.239	0.296	-0.806	0.421	0.788	0.233	-0.91	0.363	0.441	1.408
WDR19	-0.405	0.3	-1.349	0.177	0.667	0.2	-1.663	0.096	0.371	1.201
WDR18	0.31	0.297	1.042	0.297	1.364	0.406	0.896	0.37	0.761	2.443
WDR13	-0.313	0.301	-1.042	0.297	0.731	0.22	-1.223	0.221	0.406	1.318
WDR12	0.293	0.298	0.984	0.325	1.34	0.399	0.853	0.394	0.748	2.402
WDR11	0.168	0.296	0.566	0.571	1.182	0.35	0.521	0.602	0.662	2.112
WDR1	0.41	0.3	1.369	0.171	1.507	0.452	1.123	0.261	0.838	2.711
WDHD1	-0.004	0.295	-0.015	0.988	0.996	0.294	-0.015	0.988	0.558	1.777
WDFY3	0.232	0.298	0.778	0.437	1.261	0.376	0.694	0.487	0.703	2.263
WDCP	0.096	0.296	0.324	0.746	1.1	0.325	0.309	0.758	0.616	1.965
WBP4	0.008	0.295	0.027	0.978	1.008	0.297	0.027	0.978	0.565	1.797
WBP2	-0.23	0.297	-0.773	0.439	0.795	0.236	-0.869	0.385	0.444	1.422
WBP1L	-0.249	0.298	-0.838	0.402	0.779	0.232	-0.951	0.342	0.435	1.397

WBP11	0.321	0.296	1.084	0.278	1.379	0.409	0.927	0.354	0.771	2.465
WASL	-0.04	0.298	-0.134	0.894	0.961	0.286	-0.137	0.891	0.536	1.724
WASHC5	0.349	0.299	1.164	0.244	1.417	0.424	0.983	0.326	0.788	2.549
WASHC4	-0.762	0.315	-2.421	0.015	0.467	0.147	-3.629	0	0.252	0.865
WASHC3	-0.338	0.308	-1.098	0.272	0.713	0.22	-1.307	0.191	0.39	1.304
WASHC2C	-0.223	0.296	-0.751	0.453	0.8	0.237	-0.841	0.4	0.448	1.431
WASF3	0.186	0.296	0.627	0.53	1.204	0.357	0.573	0.567	0.674	2.151
WASF2	-0.444	0.302	-1.47	0.142	0.641	0.194	-1.85	0.064	0.355	1.16
WASF1	-0.103	0.296	-0.347	0.729	0.902	0.267	-0.365	0.715	0.505	1.612
WAS	-0.449	0.3	-1.497	0.134	0.638	0.192	-1.89	0.059	0.354	1.149
WARS2	-0.439	0.302	-1.452	0.147	0.645	0.195	-1.822	0.068	0.357	1.166
WARS1	-0.337	0.297	-1.135	0.256	0.714	0.212	-1.35	0.177	0.399	1.278
WAPL	-0.278	0.298	-0.934	0.35	0.757	0.225	-1.076	0.282	0.423	1.357
WAC	-0.396	0.299	-1.322	0.186	0.673	0.201	-1.622	0.105	0.374	1.21
VWF	-0.123	0.295	-0.416	0.677	0.884	0.261	-0.443	0.658	0.496	1.578
VWA8	-0.24	0.296	-0.81	0.418	0.787	0.233	-0.915	0.36	0.44	1.406
VWA7	-0.557	0.306	-1.82	0.069	0.573	0.175	-2.435	0.015	0.314	1.044
VWA5A	-0.589	0.303	-1.944	0.052	0.555	0.168	-2.647	0.008	0.306	1.005
VWA1	-0.622	0.306	-2.035	0.042	0.537	0.164	-2.823	0.005	0.295	0.977
VTI1B	-0.602	0.306	-1.971	0.049	0.547	0.167	-2.704	0.007	0.301	0.997
VTCN1	-0.322	0.3	-1.075	0.283	0.725	0.217	-1.268	0.205	0.403	1.304
VSX1	-0.043	0.299	-0.145	0.885	0.958	0.286	-0.148	0.882	0.533	1.72
VSTM4	-0.218	0.296	-0.738	0.46	0.804	0.238	-0.825	0.409	0.45	1.435
VSNL1	-0.013	0.295	-0.045	0.964	0.987	0.291	-0.045	0.964	0.553	1.76
VSIG4	0.063	0.295	0.212	0.832	1.065	0.314	0.205	0.837	0.597	1.899
VRTN	0.089	0.296	0.301	0.763	1.093	0.324	0.288	0.773	0.612	1.953
VRK3	-0.414	0.3	-1.381	0.167	0.661	0.198	-1.711	0.087	0.367	1.189
VRK2	0.951	0.316	3.012	0.003	2.588	0.817	1.943	0.052	1.394	4.806
VRK1	0.408	0.298	1.369	0.171	1.503	0.448	1.125	0.261	0.839	2.694
VPS9D1	0.406	0.298	1.365	0.172	1.501	0.447	1.121	0.262	0.838	2.691
VPS8	-0.103	0.295	-0.35	0.726	0.902	0.266	-0.369	0.712	0.505	1.609
VPS72	0.713	0.307	2.326	0.02	2.04	0.625	1.663	0.096	1.119	3.72
VPS54	-0.212	0.296	-0.715	0.475	0.809	0.24	-0.796	0.426	0.453	1.446
VPS53	0.243	0.298	0.815	0.415	1.275	0.379	0.724	0.469	0.711	2.284
VPS52	0.101	0.297	0.341	0.733	1.106	0.328	0.324	0.746	0.619	1.979
VPS51	-0.012	0.296	-0.041	0.967	0.988	0.292	-0.042	0.967	0.553	1.764
VPS50	-0.457	0.3	-1.523	0.128	0.633	0.19	-1.93	0.054	0.352	1.14
VPS4B	-0.536	0.303	-1.768	0.077	0.585	0.177	-2.339	0.019	0.323	1.06

VPS4A	0.46	0.3	1.534	0.125	1.583	0.474	1.23	0.219	0.88	2.848
VPS45	-0.103	0.299	-0.343	0.732	0.903	0.27	-0.361	0.718	0.502	1.622
VPS41	0.402	0.3	1.341	0.18	1.495	0.448	1.105	0.269	0.831	2.688
VPS39	-1.099	0.336	-3.271	0.001	0.333	0.112	-5.955	0	0.173	0.644
VPS37C	-0.056	0.295	-0.189	0.85	0.946	0.279	-0.194	0.846	0.53	1.687
VPS37B	-0.173	0.296	-0.584	0.559	0.841	0.249	-0.637	0.524	0.471	1.502
VPS35L	-0.319	0.298	-1.072	0.284	0.727	0.216	-1.263	0.207	0.405	1.303
VPS35	0.938	0.321	2.925	0.003	2.554	0.819	1.898	0.058	1.363	4.787
VPS33B	0.507	0.303	1.675	0.094	1.66	0.503	1.314	0.189	0.917	3.006
VPS33A	0.058	0.296	0.196	0.844	1.06	0.313	0.191	0.849	0.594	1.891
VPS28	0.06	0.296	0.204	0.839	1.062	0.314	0.198	0.843	0.595	1.896
VPS26C	0.278	0.297	0.935	0.35	1.32	0.392	0.816	0.414	0.737	2.363
VPS26A	-0.119	0.296	-0.401	0.688	0.888	0.263	-0.426	0.67	0.497	1.587
VPS16	0.066	0.295	0.224	0.823	1.068	0.316	0.217	0.828	0.599	1.907
VPS13D	-0.069	0.295	-0.232	0.817	0.934	0.276	-0.24	0.81	0.523	1.666
VPS13C	-0.992	0.318	-3.119	0.002	0.371	0.118	-5.335	0	0.199	0.692
VPS13B	-0.162	0.296	-0.547	0.584	0.85	0.252	-0.594	0.553	0.476	1.52
VPS13A	-0.419	0.301	-1.394	0.163	0.657	0.198	-1.732	0.083	0.365	1.186
VPS11	0.064	0.295	0.215	0.83	1.066	0.315	0.209	0.835	0.597	1.902
VPREB3	0.073	0.297	0.246	0.806	1.076	0.319	0.237	0.813	0.601	1.924
VPREB1	0.081	0.295	0.275	0.784	1.084	0.32	0.264	0.792	0.608	1.935
VOPP1	0.159	0.296	0.537	0.591	1.172	0.347	0.497	0.619	0.657	2.092
VNN3	-0.403	0.3	-1.344	0.179	0.669	0.2	-1.654	0.098	0.372	1.203
VNN2	-0.524	0.302	-1.733	0.083	0.592	0.179	-2.278	0.023	0.327	1.071
VNN1	-0.05	0.296	-0.168	0.866	0.951	0.282	-0.173	0.863	0.532	1.7
VN1R1	-0.03	0.296	-0.102	0.919	0.97	0.287	-0.103	0.918	0.543	1.733
VLDLR	-0.089	0.297	-0.301	0.764	0.915	0.271	-0.314	0.753	0.512	1.636
VKORC1	-0.237	0.298	-0.795	0.427	0.789	0.235	-0.897	0.37	0.44	1.415
VIPR2	-0.324	0.3	-1.079	0.28	0.723	0.217	-1.274	0.203	0.402	1.302
VIPR1	-0.403	0.299	-1.346	0.178	0.669	0.2	-1.657	0.097	0.372	1.202
VIPAS39	0.142	0.296	0.478	0.632	1.152	0.341	0.446	0.656	0.645	2.058
VIP	0.338	0.298	1.134	0.257	1.402	0.418	0.962	0.336	0.782	2.514
VIM	-0.231	0.296	-0.78	0.435	0.794	0.235	-0.878	0.38	0.444	1.418
VILL	-0.763	0.315	-2.423	0.015	0.466	0.147	-3.635	0	0.251	0.864
VIL1	0.869	0.315	2.756	0.006	2.383	0.751	1.842	0.065	1.285	4.42
VHL	0.343	0.297	1.154	0.249	1.409	0.419	0.977	0.329	0.787	2.522
VGLL4	-0.315	0.297	-1.062	0.288	0.73	0.217	-1.249	0.212	0.408	1.305
VGLL3	-0.61	0.303	-2.011	0.044	0.543	0.165	-2.771	0.006	0.3	0.985

VGLL1	0.41	0.3	1.368	0.171	1.506	0.451	1.122	0.262	0.837	2.709
VGF	0.131	0.297	0.442	0.659	1.14	0.338	0.414	0.679	0.638	2.038
VEZT	-0.625	0.306	-2.043	0.041	0.535	0.164	-2.838	0.005	0.294	0.975
VEZF1	0.197	0.296	0.665	0.506	1.218	0.361	0.604	0.546	0.681	2.177
VENTXP1	0.159	0.295	0.538	0.591	1.172	0.346	0.497	0.619	0.657	2.091
VENTX	0.116	0.296	0.392	0.695	1.123	0.333	0.371	0.711	0.628	2.009
VEGFC	-0.378	0.3	-1.261	0.207	0.685	0.206	-1.533	0.125	0.38	1.233
VEGFB	0.144	0.296	0.486	0.627	1.155	0.342	0.453	0.651	0.646	2.065
VEGFA	0.318	0.298	1.068	0.286	1.374	0.409	0.915	0.36	0.767	2.463
VDR	0.308	0.296	1.04	0.298	1.361	0.403	0.895	0.371	0.761	2.433
VDAC3	0.993	0.321	3.094	0.002	2.699	0.866	1.962	0.05	1.439	5.062
VDAC2	0.127	0.295	0.429	0.668	1.135	0.335	0.403	0.687	0.636	2.024
VDAC1	-0.548	0.303	-1.809	0.07	0.578	0.175	-2.409	0.016	0.319	1.047
VCX2	0.158	0.296	0.532	0.595	1.171	0.347	0.492	0.623	0.655	2.093
VCPKMT	0.101	0.295	0.343	0.731	1.107	0.327	0.327	0.744	0.62	1.974
VCPIP1	-0.076	0.296	-0.256	0.798	0.927	0.274	-0.266	0.79	0.519	1.655
VCP	0.073	0.295	0.248	0.804	1.076	0.318	0.239	0.811	0.603	1.919
VCL	0.423	0.303	1.399	0.162	1.527	0.462	1.14	0.254	0.844	2.763
VCAN	-0.496	0.3	-1.656	0.098	0.609	0.182	-2.144	0.032	0.338	1.095
VCAM1	-0.395	0.298	-1.324	0.186	0.674	0.201	-1.623	0.105	0.376	1.209
VBP1	0.155	0.296	0.525	0.6	1.168	0.345	0.486	0.627	0.654	2.084
VAX2	0.185	0.296	0.624	0.532	1.203	0.357	0.57	0.569	0.673	2.151
VAV3	-0.303	0.3	-1.012	0.311	0.738	0.221	-1.183	0.237	0.41	1.329
VAV2	-0.014	0.296	-0.047	0.962	0.986	0.292	-0.048	0.962	0.552	1.76
VAV1	-0.418	0.3	-1.393	0.164	0.659	0.198	-1.729	0.084	0.366	1.186
VAT1	0.268	0.3	0.891	0.373	1.307	0.392	0.782	0.434	0.725	2.354
VASP	-0.142	0.297	-0.478	0.632	0.868	0.257	-0.514	0.607	0.485	1.552
VASH2	0.373	0.298	1.253	0.21	1.452	0.432	1.046	0.295	0.81	2.601
VASH1-AS1	-0.158	0.296	-0.533	0.594	0.854	0.253	-0.577	0.564	0.479	1.525
VASH1	0.057	0.295	0.194	0.846	1.059	0.312	0.189	0.85	0.594	1.888
VARS1	0.149	0.296	0.504	0.614	1.161	0.344	0.468	0.639	0.65	2.076
VAPB	0.665	0.31	2.144	0.032	1.944	0.602	1.567	0.117	1.059	3.568
VAPA	0.142	0.296	0.482	0.63	1.153	0.341	0.449	0.653	0.646	2.058
VANGL1	0.067	0.297	0.225	0.822	1.069	0.317	0.217	0.828	0.598	1.912
VAMP8	-0.117	0.295	-0.395	0.693	0.89	0.263	-0.419	0.675	0.499	1.587
VAMP7	0.583	0.302	1.928	0.054	1.791	0.541	1.461	0.144	0.99	3.239
VAMP5	-0.28	0.297	-0.945	0.345	0.755	0.224	-1.091	0.275	0.422	1.351
VAMP4	-0.231	0.3	-0.772	0.44	0.793	0.238	-0.868	0.385	0.441	1.428

VAMP3	-0.456	0.307	-1.487	0.137	0.634	0.194	-1.884	0.06	0.347	1.156
VAMP2	-0.246	0.298	-0.824	0.41	0.782	0.233	-0.934	0.35	0.436	1.402
VAMP1	-0.568	0.303	-1.877	0.061	0.567	0.171	-2.527	0.011	0.313	1.025
VAC14	0.201	0.296	0.679	0.497	1.223	0.363	0.615	0.539	0.684	2.186
UXT	-0.213	0.298	-0.717	0.474	0.808	0.241	-0.799	0.424	0.451	1.448
UXS1	0.025	0.296	0.085	0.933	1.025	0.303	0.084	0.933	0.574	1.831
UVRAG	-0.054	0.296	-0.183	0.855	0.947	0.281	-0.188	0.851	0.53	1.693
UTY	0.222	0.296	0.749	0.454	1.248	0.369	0.672	0.502	0.699	2.228
UTS2	-0.681	0.306	-2.226	0.026	0.506	0.155	-3.19	0.001	0.278	0.922
UTRN	-0.459	0.3	-1.532	0.126	0.632	0.189	-1.944	0.052	0.351	1.137
UTP6	0.043	0.296	0.145	0.885	1.044	0.309	0.142	0.887	0.584	1.865
UTP3	0.438	0.299	1.465	0.143	1.549	0.463	1.187	0.235	0.863	2.783
UTP25	0.615	0.306	2.009	0.045	1.85	0.566	1.501	0.133	1.015	3.371
UTP20	0.059	0.296	0.2	0.841	1.061	0.314	0.194	0.846	0.594	1.896
UTP18	0.341	0.298	1.145	0.252	1.406	0.418	0.97	0.332	0.785	2.519
UTP14A	0.447	0.3	1.49	0.136	1.563	0.469	1.202	0.229	0.869	2.813
UTP11	0.228	0.296	0.768	0.442	1.256	0.372	0.687	0.492	0.703	2.244
UTF1	-0.01	0.296	-0.032	0.974	0.991	0.293	-0.032	0.974	0.555	1.768
UST	-0.038	0.298	-0.128	0.898	0.962	0.287	-0.131	0.896	0.537	1.726
USPL1	0.497	0.303	1.638	0.101	1.644	0.499	1.291	0.197	0.907	2.98
USP9Y	-0.235	0.297	-0.793	0.428	0.79	0.234	-0.894	0.371	0.442	1.413
USP9X	-0.034	0.295	-0.116	0.908	0.966	0.285	-0.118	0.906	0.542	1.724
USP8	-0.309	0.3	-1.029	0.303	0.734	0.22	-1.206	0.228	0.408	1.322
USP7	-0.09	0.297	-0.303	0.762	0.914	0.272	-0.317	0.752	0.51	1.637
USP6NL	0.932	0.316	2.951	0.003	2.54	0.803	1.919	0.055	1.368	4.719
USP53	-0.277	0.298	-0.929	0.353	0.758	0.226	-1.071	0.284	0.423	1.359
USP5	0.369	0.3	1.232	0.218	1.447	0.434	1.03	0.303	0.804	2.604
USP49	-0.279	0.3	-0.93	0.352	0.756	0.227	-1.073	0.283	0.42	1.362
USP48	-0.231	0.299	-0.775	0.438	0.793	0.237	-0.873	0.383	0.442	1.424
USP47	0.179	0.295	0.605	0.545	1.196	0.353	0.554	0.58	0.67	2.133
USP46	0.017	0.295	0.056	0.955	1.017	0.3	0.056	0.955	0.57	1.814
USP4	-0.046	0.295	-0.156	0.876	0.955	0.282	-0.16	0.873	0.536	1.702
USP39	0.408	0.3	1.36	0.174	1.503	0.451	1.117	0.264	0.835	2.705
USP36	0.391	0.298	1.314	0.189	1.479	0.44	1.088	0.277	0.825	2.651
USP34	0.098	0.297	0.329	0.742	1.102	0.327	0.313	0.754	0.616	1.972
USP33	-0.294	0.3	-0.981	0.326	0.745	0.223	-1.141	0.254	0.414	1.341
USP32P2	0.069	0.295	0.232	0.816	1.071	0.316	0.224	0.823	0.6	1.911
USP32	0.174	0.296	0.59	0.555	1.191	0.352	0.542	0.588	0.667	2.125

USP3	0.071	0.295	0.241	0.81	1.074	0.317	0.232	0.816	0.602	1.914
USP29	0.28	0.301	0.932	0.351	1.324	0.398	0.813	0.416	0.734	2.387
USP27X	0.005	0.295	0.018	0.985	1.005	0.297	0.018	0.985	0.564	1.793
USP25	0.212	0.296	0.716	0.474	1.236	0.366	0.645	0.519	0.692	2.21
USP24	-0.029	0.295	-0.099	0.921	0.971	0.287	-0.1	0.92	0.544	1.733
USP22	-0.195	0.297	-0.655	0.513	0.823	0.245	-0.723	0.47	0.46	1.474
USP21	-0.207	0.298	-0.695	0.487	0.813	0.242	-0.772	0.44	0.453	1.459
USP20	-0.107	0.295	-0.362	0.717	0.899	0.265	-0.382	0.702	0.504	1.603
USP2	-0.234	0.297	-0.788	0.431	0.791	0.235	-0.888	0.375	0.442	1.416
USP19	-0.009	0.296	-0.03	0.976	0.991	0.293	-0.03	0.976	0.555	1.769
USP18	-0.04	0.295	-0.137	0.891	0.96	0.284	-0.139	0.889	0.538	1.713
USP16	-0.113	0.3	-0.376	0.707	0.893	0.268	-0.398	0.69	0.496	1.609
USP14	0.616	0.303	2.029	0.042	1.851	0.562	1.515	0.13	1.021	3.356
USP13	0.487	0.3	1.627	0.104	1.628	0.488	1.287	0.198	0.905	2.929
USP12	1.048	0.322	3.25	0.001	2.851	0.919	2.014	0.044	1.516	5.364
USP11	0.257	0.298	0.862	0.389	1.293	0.385	0.761	0.447	0.721	2.316
USP10	0.957	0.315	3.036	0.002	2.605	0.822	1.954	0.051	1.404	4.834
USP1	0.406	0.298	1.361	0.174	1.5	0.447	1.119	0.263	0.836	2.691
USO1	-0.422	0.3	-1.406	0.16	0.656	0.197	-1.749	0.08	0.364	1.181
USH2A	0.032	0.295	0.11	0.913	1.033	0.305	0.108	0.914	0.579	1.842
USH1C	0.835	0.315	2.652	0.008	2.304	0.726	1.798	0.072	1.243	4.271
USF2	-0.314	0.3	-1.046	0.295	0.731	0.219	-1.229	0.219	0.406	1.315
USE1	0.08	0.295	0.27	0.787	1.083	0.32	0.259	0.795	0.607	1.931
USB1	0.354	0.298	1.19	0.234	1.425	0.424	1.002	0.316	0.795	2.553
UROS	-0.589	0.31	-1.899	0.058	0.555	0.172	-2.586	0.01	0.302	1.019
UROD	0.033	0.297	0.111	0.912	1.033	0.307	0.109	0.913	0.578	1.848
URM1	0.355	0.3	1.183	0.237	1.427	0.429	0.996	0.319	0.792	2.57
URI1	0.382	0.3	1.275	0.202	1.465	0.439	1.06	0.289	0.814	2.637
URGCP	-0.013	0.297	-0.044	0.965	0.987	0.293	-0.045	0.964	0.551	1.767
URB2	0.607	0.303	2.004	0.045	1.835	0.556	1.502	0.133	1.013	3.322
URB1	-0.147	0.296	-0.495	0.62	0.864	0.255	-0.534	0.594	0.484	1.542
UQCRQ	-0.053	0.295	-0.18	0.857	0.948	0.28	-0.185	0.853	0.532	1.691
UQCRFS1	0.284	0.296	0.959	0.338	1.329	0.394	0.835	0.404	0.743	2.375
UQCRC2	0.329	0.298	1.103	0.27	1.39	0.415	0.94	0.347	0.774	2.495
UQCRC1	0.035	0.297	0.119	0.905	1.036	0.307	0.117	0.907	0.579	1.853
UQCRB	0.064	0.296	0.216	0.829	1.066	0.315	0.209	0.835	0.597	1.903
UQCR11	0.303	0.298	1.015	0.31	1.354	0.404	0.876	0.381	0.754	2.429
UQCR10	0.234	0.296	0.789	0.43	1.263	0.374	0.704	0.481	0.707	2.258

UQCC1	-0.018	0.295	-0.059	0.953	0.983	0.29	-0.06	0.952	0.551	1.753
UPP1	0.422	0.298	1.416	0.157	1.525	0.455	1.155	0.248	0.85	2.736
UPK3A	0.249	0.298	0.837	0.402	1.283	0.382	0.741	0.459	0.716	2.299
UPK2	0.184	0.296	0.622	0.534	1.202	0.356	0.568	0.57	0.673	2.149
UPK1B	0.523	0.302	1.73	0.084	1.687	0.51	1.347	0.178	0.933	3.052
UPK1A	-0.569	0.302	-1.881	0.06	0.566	0.171	-2.534	0.011	0.313	1.024
UPF3B	0.484	0.306	1.58	0.114	1.623	0.497	1.252	0.211	0.89	2.959
UPF3A	-0.277	0.298	-0.931	0.352	0.758	0.226	-1.073	0.283	0.423	1.358
UPF2	0.443	0.303	1.465	0.143	1.558	0.472	1.183	0.237	0.861	2.82
UPF1	0.732	0.306	2.39	0.017	2.08	0.637	1.695	0.09	1.141	3.791
UPB1	0.16	0.296	0.541	0.589	1.174	0.348	0.5	0.617	0.657	2.097
UNKL	0.465	0.3	1.549	0.121	1.592	0.478	1.239	0.216	0.884	2.868
UNG	0.117	0.295	0.396	0.692	1.124	0.332	0.373	0.709	0.63	2.005
UNC93B1	0.104	0.295	0.353	0.724	1.11	0.328	0.335	0.738	0.622	1.98
UNC93A	-0.063	0.295	-0.213	0.832	0.939	0.277	-0.219	0.826	0.527	1.675
UNC5C	-0.08	0.297	-0.268	0.789	0.924	0.274	-0.279	0.78	0.516	1.652
UNC5B	-0.436	0.3	-1.452	0.146	0.647	0.194	-1.82	0.069	0.359	1.165
UNC50	-0.003	0.295	-0.009	0.993	0.997	0.294	-0.009	0.993	0.56	1.778
UNC45A	1.16	0.328	3.533	0	3.19	1.047	2.091	0.037	1.676	6.07
UNC13B	0	0.295	0.001	0.999	1	0.295	0.001	0.999	0.561	1.784
UNC13A	-0.211	0.298	-0.709	0.478	0.81	0.241	-0.789	0.43	0.452	1.451
UNC119B	-0.534	0.303	-1.76	0.078	0.586	0.178	-2.326	0.02	0.323	1.063
UNC119	0.005	0.298	0.018	0.986	1.005	0.3	0.018	0.986	0.56	1.804
UMPS	0.54	0.306	1.764	0.078	1.716	0.525	1.363	0.173	0.942	3.125
UMOD	0.253	0.298	0.849	0.396	1.288	0.383	0.75	0.453	0.718	2.308
ULK4	-0.672	0.305	-2.205	0.027	0.511	0.156	-3.144	0.002	0.281	0.928
ULK2	-0.035	0.295	-0.118	0.906	0.966	0.285	-0.12	0.905	0.541	1.723
ULK1	-0.896	0.316	-2.837	0.005	0.408	0.129	-4.59	0	0.22	0.758
ULBP2	0.397	0.3	1.322	0.186	1.487	0.446	1.091	0.275	0.826	2.676
ULBP1	0.058	0.295	0.195	0.845	1.059	0.313	0.19	0.85	0.594	1.889
UIMC1	0.272	0.298	0.915	0.36	1.313	0.391	0.801	0.423	0.733	2.352
UHRF1BP1L	-0.382	0.307	-1.246	0.213	0.682	0.209	-1.517	0.129	0.374	1.245
UGT8	0.168	0.298	0.565	0.572	1.183	0.352	0.52	0.603	0.66	2.121
UGT2B4	0.308	0.297	1.035	0.301	1.36	0.404	0.891	0.373	0.76	2.435
UGT2B28	0.143	0.297	0.482	0.63	1.154	0.343	0.449	0.653	0.645	2.065
UGT2B17	0.541	0.306	1.77	0.077	1.718	0.525	1.367	0.172	0.944	3.129
UGT2B15	0.02	0.298	0.066	0.947	1.02	0.304	0.066	0.948	0.569	1.828
UGT2A3	0.042	0.295	0.142	0.887	1.043	0.308	0.14	0.889	0.585	1.859

UGP2	0.101	0.295	0.341	0.733	1.106	0.327	0.325	0.745	0.62	1.973
UGGT2	0.237	0.297	0.799	0.424	1.267	0.376	0.712	0.477	0.709	2.266
UGGT1	0.581	0.306	1.899	0.058	1.787	0.546	1.441	0.15	0.982	3.254
UGDH	-0.519	0.3	-1.729	0.084	0.595	0.179	-2.266	0.023	0.331	1.072
UGCG	-0.354	0.3	-1.183	0.237	0.702	0.21	-1.42	0.156	0.39	1.262
UFSP2	-0.001	0.296	-0.003	0.997	0.999	0.295	-0.003	0.997	0.56	1.784
UFM1	0.432	0.302	1.427	0.153	1.54	0.465	1.159	0.246	0.851	2.784
UFL1	-0.115	0.295	-0.39	0.696	0.891	0.263	-0.414	0.679	0.5	1.59
UFD1	-0.205	0.296	-0.69	0.49	0.815	0.242	-0.766	0.444	0.456	1.457
UFC1	-0.46	0.302	-1.522	0.128	0.631	0.191	-1.933	0.053	0.349	1.142
UEVLD	0.005	0.299	0.015	0.988	1.005	0.3	0.015	0.988	0.559	1.805
UCP3	0.356	0.3	1.186	0.236	1.428	0.429	0.997	0.319	0.792	2.572
UCP2	-0.057	0.295	-0.192	0.848	0.945	0.279	-0.197	0.844	0.53	1.686
UCP1	0.214	0.296	0.721	0.471	1.238	0.367	0.649	0.516	0.693	2.212
UCN	0.293	0.298	0.984	0.325	1.341	0.399	0.853	0.393	0.748	2.403
UCKL1	0.604	0.306	1.975	0.048	1.829	0.559	1.483	0.138	1.005	3.329
UCHL5	0.716	0.31	2.311	0.021	2.046	0.634	1.65	0.099	1.115	3.755
UCHL3	0.064	0.295	0.218	0.828	1.066	0.315	0.211	0.833	0.598	1.902
UCHL1	0.061	0.295	0.205	0.837	1.063	0.314	0.199	0.842	0.596	1.895
UBXN8	-0.02	0.296	-0.067	0.947	0.98	0.29	-0.067	0.946	0.549	1.752
UBXN7	-0.096	0.297	-0.322	0.748	0.909	0.27	-0.338	0.736	0.507	1.628
UBXN4	0.821	0.315	2.609	0.009	2.273	0.715	1.779	0.075	1.227	4.212
UBXN2B	0.414	0.3	1.38	0.168	1.512	0.453	1.13	0.258	0.84	2.721
UBXN1	-0.37	0.298	-1.239	0.215	0.691	0.206	-1.499	0.134	0.385	1.24
UBTF	-0.736	0.31	-2.372	0.018	0.479	0.149	-3.506	0	0.261	0.88
UBTD1	0.115	0.295	0.391	0.696	1.122	0.332	0.369	0.712	0.629	2.003
UBR7	0.017	0.297	0.057	0.954	1.017	0.302	0.057	0.955	0.568	1.821
UBR5	-0.569	0.303	-1.877	0.06	0.566	0.172	-2.529	0.011	0.313	1.025
UBR4	-0.145	0.296	-0.49	0.624	0.865	0.256	-0.528	0.598	0.485	1.544
UBR2	-0.061	0.296	-0.207	0.836	0.941	0.278	-0.213	0.831	0.527	1.679
UBQLN4	0.235	0.298	0.788	0.431	1.265	0.377	0.702	0.482	0.705	2.267
UBQLN3	0.398	0.299	1.332	0.183	1.488	0.444	1.099	0.272	0.829	2.672
UBQLN2	-0.519	0.306	-1.695	0.09	0.595	0.182	-2.222	0.026	0.327	1.084
UBP1	-0.039	0.295	-0.133	0.894	0.961	0.284	-0.136	0.892	0.539	1.714
UBOX5	0.003	0.296	0.009	0.993	1.003	0.297	0.009	0.993	0.562	1.79
UBN1	0.027	0.296	0.09	0.928	1.027	0.304	0.089	0.929	0.575	1.834
UBL5	0.894	0.312	2.868	0.004	2.445	0.762	1.896	0.058	1.327	4.505
UBL4A	0.422	0.298	1.415	0.157	1.525	0.455	1.154	0.248	0.85	2.737

UBL3	-0.537	0.306	-1.755	0.079	0.585	0.179	-2.323	0.02	0.321	1.065
UBIAD1	0.056	0.297	0.188	0.851	1.058	0.314	0.183	0.855	0.591	1.892
UBFD1	0.68	0.31	2.192	0.028	1.974	0.613	1.59	0.112	1.075	3.627
UBE4B	-0.044	0.296	-0.148	0.882	0.957	0.283	-0.152	0.879	0.536	1.709
UBE4A	-0.509	0.303	-1.683	0.092	0.601	0.182	-2.194	0.028	0.332	1.088
UBE3C	0.098	0.296	0.332	0.74	1.103	0.327	0.316	0.752	0.617	1.971
UBE3B	-0.629	0.304	-2.073	0.038	0.533	0.162	-2.886	0.004	0.294	0.966
UBE3A	-0.966	0.328	-2.95	0.003	0.381	0.125	-4.971	0	0.2	0.723
UBE2Z	0.077	0.297	0.259	0.796	1.08	0.32	0.249	0.803	0.604	1.932
UBE2W	0.406	0.298	1.361	0.173	1.501	0.447	1.119	0.263	0.836	2.692
UBE2V2	0.7	0.306	2.291	0.022	2.014	0.616	1.647	0.1	1.106	3.667
UBE2S	0.541	0.3	1.802	0.072	1.718	0.516	1.391	0.164	0.954	3.095
UBE2Q1	0.012	0.296	0.039	0.969	1.012	0.299	0.039	0.969	0.566	1.807
UBE2O	0.84	0.315	2.669	0.008	2.316	0.729	1.806	0.071	1.25	4.292
UBE2NL	0.072	0.295	0.244	0.808	1.075	0.317	0.235	0.814	0.603	1.916
UBE2N	0.191	0.296	0.646	0.518	1.211	0.358	0.588	0.556	0.678	2.162
UBE2M	0.072	0.296	0.245	0.807	1.075	0.318	0.236	0.813	0.602	1.92
UBE2L6	0.01	0.296	0.032	0.974	1.01	0.298	0.032	0.974	0.566	1.802
UBE2L3	0.363	0.297	1.221	0.222	1.437	0.427	1.024	0.306	0.803	2.573
UBE2K	0.923	0.315	2.927	0.003	2.516	0.793	1.911	0.056	1.356	4.669
UBE2J1	-0.561	0.301	-1.861	0.063	0.571	0.172	-2.495	0.013	0.316	1.03
UBE2I	0.157	0.295	0.532	0.594	1.17	0.346	0.493	0.622	0.656	2.088
UBE2H	-0.5	0.302	-1.654	0.098	0.607	0.183	-2.147	0.032	0.335	1.097
UBE2G2	-0.068	0.295	-0.231	0.817	0.934	0.276	-0.24	0.811	0.524	1.666
UBE2G1	-0.468	0.306	-1.53	0.126	0.626	0.192	-1.951	0.051	0.344	1.141
UBE2E3	-0.112	0.296	-0.378	0.706	0.894	0.265	-0.399	0.69	0.501	1.597
UBE2E1	0.319	0.297	1.074	0.283	1.376	0.409	0.919	0.358	0.769	2.462
UBE2D4	-0.269	0.298	-0.903	0.366	0.764	0.227	-1.037	0.3	0.426	1.37
UBE2D3	0.026	0.296	0.086	0.931	1.026	0.304	0.085	0.932	0.574	1.834
UBE2D2	0.132	0.298	0.442	0.658	1.141	0.339	0.415	0.678	0.637	2.044
UBE2D1	0.254	0.298	0.854	0.393	1.29	0.384	0.754	0.451	0.719	2.312
UBE2C	0.846	0.315	2.688	0.007	2.33	0.734	1.814	0.07	1.257	4.319
UBE2B	-0.108	0.295	-0.366	0.715	0.898	0.265	-0.386	0.699	0.503	1.602
UBE2A	0.557	0.301	1.847	0.065	1.745	0.526	1.417	0.157	0.967	3.15
UBC	0.003	0.295	0.01	0.992	1.003	0.296	0.01	0.992	0.563	1.788
UBB	0.674	0.306	2.206	0.027	1.962	0.6	1.605	0.109	1.078	3.571
UBASH3A	-0.554	0.306	-1.81	0.07	0.575	0.176	-2.418	0.016	0.315	1.047
UBAP2L	-0.076	0.296	-0.258	0.797	0.927	0.274	-0.268	0.789	0.519	1.654

UBAP2	0.575	0.303	1.9	0.057	1.777	0.537	1.445	0.149	0.982	3.214
UBAP1	-0.168	0.296	-0.569	0.569	0.845	0.25	-0.62	0.535	0.474	1.508
UBAC1	-0.34	0.3	-1.136	0.256	0.712	0.213	-1.353	0.176	0.396	1.28
UBA6	-0.126	0.297	-0.425	0.671	0.881	0.262	-0.453	0.65	0.492	1.578
UBA52	-0.407	0.3	-1.357	0.175	0.666	0.2	-1.675	0.094	0.37	1.198
UBA5	0.171	0.297	0.578	0.564	1.187	0.352	0.531	0.596	0.663	2.124
UBA3	-0.02	0.296	-0.069	0.945	0.98	0.29	-0.07	0.944	0.548	1.751
UBA2	0.469	0.303	1.546	0.122	1.598	0.484	1.234	0.217	0.882	2.895
UBA1	-0.146	0.297	-0.492	0.622	0.864	0.256	-0.53	0.596	0.483	1.546
UAP1L1	0.457	0.3	1.524	0.128	1.579	0.473	1.223	0.221	0.877	2.841
UAP1	-0.31	0.298	-1.043	0.297	0.733	0.218	-1.223	0.221	0.409	1.314
U2SURP	0.295	0.301	0.982	0.326	1.344	0.404	0.85	0.395	0.745	2.424
U2AF2	0.587	0.303	1.938	0.053	1.798	0.544	1.466	0.143	0.993	3.254
U2AF1	1.133	0.323	3.502	0	3.104	1.004	2.096	0.036	1.647	5.85
TYW1	0.083	0.295	0.28	0.78	1.086	0.321	0.269	0.788	0.609	1.938
TYRP1	0.085	0.296	0.286	0.775	1.088	0.322	0.275	0.784	0.61	1.943
TYROBP	0.423	0.298	1.418	0.156	1.526	0.455	1.156	0.248	0.851	2.739
TYRO3	0.222	0.296	0.748	0.455	1.248	0.37	0.671	0.502	0.698	2.231
TYR	0.085	0.295	0.287	0.774	1.088	0.321	0.275	0.783	0.61	1.941
TYMS	0.721	0.306	2.357	0.018	2.057	0.629	1.679	0.093	1.129	3.747
TYMP	0.189	0.296	0.639	0.523	1.208	0.358	0.582	0.561	0.676	2.158
TYK2	-0.316	0.298	-1.061	0.289	0.729	0.217	-1.248	0.212	0.407	1.307
TXNRD2	0.095	0.295	0.322	0.748	1.1	0.325	0.307	0.759	0.616	1.962
TXNRD1	0.557	0.306	1.819	0.069	1.745	0.534	1.395	0.163	0.958	3.179
TXNL4B	0.45	0.303	1.482	0.138	1.568	0.476	1.193	0.233	0.865	2.841
TXNL4A	-0.18	0.301	-0.598	0.55	0.836	0.251	-0.655	0.513	0.463	1.507
TXNL1	0.018	0.296	0.062	0.951	1.018	0.301	0.061	0.951	0.57	1.818
TXNIP	-0.774	0.31	-2.498	0.013	0.461	0.143	-3.771	0	0.251	0.846
TXNDC9	-0.089	0.297	-0.3	0.764	0.915	0.272	-0.313	0.754	0.511	1.637
TXNDC15	-0.036	0.295	-0.121	0.904	0.965	0.285	-0.123	0.902	0.541	1.721
TXN2	-0.128	0.295	-0.435	0.664	0.88	0.26	-0.464	0.643	0.493	1.569
TXN	1.083	0.328	3.305	0.001	2.955	0.969	2.018	0.044	1.554	5.617
TXLNGY	0.242	0.298	0.812	0.417	1.273	0.379	0.721	0.471	0.711	2.282
TXLNG	0.127	0.295	0.43	0.667	1.136	0.336	0.404	0.686	0.636	2.026
TXLNA	-0.611	0.306	-2	0.046	0.543	0.166	-2.757	0.006	0.298	0.988
TXK	-0.131	0.295	-0.443	0.658	0.877	0.259	-0.473	0.636	0.492	1.566
TWSG1	-0.155	0.302	-0.513	0.608	0.856	0.259	-0.555	0.579	0.473	1.549
TWNK	0.494	0.302	1.634	0.102	1.638	0.495	1.289	0.197	0.906	2.963

TWIST1	0.11	0.295	0.372	0.71	1.116	0.329	0.352	0.725	0.626	1.99
TWF2	0.577	0.303	1.901	0.057	1.78	0.54	1.445	0.149	0.982	3.226
TWF1	-0.515	0.307	-1.679	0.093	0.598	0.183	-2.196	0.028	0.328	1.09
TVP23B	0.03	0.295	0.103	0.918	1.031	0.304	0.102	0.919	0.578	1.838
TUT7	-0.298	0.297	-1.005	0.315	0.742	0.22	-1.171	0.242	0.415	1.328
TUT4	-0.434	0.3	-1.448	0.148	0.648	0.194	-1.813	0.07	0.36	1.166
TUT1	0.545	0.303	1.801	0.072	1.725	0.522	1.389	0.165	0.953	3.122
TUSC3	0.319	0.297	1.075	0.283	1.375	0.408	0.92	0.357	0.769	2.459
TUSC2	-0.146	0.296	-0.492	0.623	0.864	0.256	-0.529	0.597	0.483	1.545
TULP4	0.16	0.295	0.542	0.588	1.174	0.347	0.501	0.616	0.658	2.094
TULP3	0.244	0.296	0.823	0.41	1.276	0.378	0.73	0.465	0.714	2.281
TULP2	-0.583	0.301	-1.935	0.053	0.558	0.168	-2.626	0.009	0.309	1.008
TULP1	0.24	0.296	0.81	0.418	1.271	0.377	0.72	0.471	0.711	2.272
TUG1	0.274	0.301	0.912	0.362	1.316	0.396	0.798	0.425	0.73	2.372
TUFT1	0.039	0.296	0.133	0.894	1.04	0.307	0.13	0.896	0.583	1.856
TUFM	0.041	0.296	0.14	0.889	1.042	0.309	0.137	0.891	0.583	1.862
TUBGCP5	0.077	0.296	0.259	0.795	1.08	0.32	0.25	0.803	0.604	1.929
TUBGCP4	0.184	0.297	0.621	0.534	1.203	0.357	0.568	0.57	0.672	2.152
TUBGCP3	0.591	0.303	1.952	0.051	1.805	0.546	1.474	0.14	0.998	3.266
TUBGCP2	-0.23	0.3	-0.768	0.442	0.794	0.238	-0.864	0.388	0.441	1.43
TUBG2	-0.126	0.296	-0.424	0.671	0.882	0.261	-0.452	0.651	0.493	1.576
TUBG1	0.497	0.302	1.644	0.1	1.644	0.497	1.296	0.195	0.909	2.975
TUBD1	0.009	0.297	0.029	0.977	1.009	0.299	0.029	0.977	0.564	1.804
TUBBP5	0.458	0.302	1.516	0.13	1.581	0.478	1.216	0.224	0.874	2.858
TUBB7P	0.367	0.298	1.233	0.217	1.443	0.43	1.032	0.302	0.806	2.586
TUBB6	-0.234	0.296	-0.789	0.43	0.792	0.234	-0.889	0.374	0.443	1.414
TUBB4B	0.381	0.298	1.28	0.201	1.464	0.437	1.064	0.287	0.816	2.627
TUBB4A	0.223	0.296	0.752	0.452	1.25	0.37	0.674	0.5	0.699	2.234
TUBB3	0.06	0.296	0.202	0.84	1.061	0.314	0.196	0.845	0.594	1.896
TUBB2B	-0.066	0.295	-0.225	0.822	0.936	0.276	-0.232	0.816	0.524	1.67
TUBB2A	0.419	0.298	1.406	0.16	1.521	0.453	1.149	0.251	0.848	2.727
TUBB1	0.052	0.295	0.176	0.861	1.053	0.311	0.171	0.864	0.591	1.878
TUBB	0.142	0.295	0.482	0.63	1.153	0.341	0.449	0.653	0.646	2.057
TUBAL3	0.217	0.296	0.732	0.464	1.242	0.368	0.658	0.511	0.695	2.219
TUBA8	0.203	0.296	0.684	0.494	1.224	0.363	0.619	0.536	0.685	2.188
TUBA4B	0.611	0.303	2.019	0.044	1.843	0.558	1.51	0.131	1.018	3.337
TUBA4A	0.407	0.298	1.366	0.172	1.503	0.448	1.122	0.262	0.838	2.696
TUBA1C	0.504	0.3	1.678	0.093	1.655	0.496	1.319	0.187	0.919	2.979

TUBA1B	0.268	0.296	0.906	0.365	1.307	0.386	0.795	0.427	0.732	2.333
TUBA1A	0.136	0.295	0.459	0.646	1.145	0.338	0.43	0.668	0.642	2.044
TUB	-0.255	0.298	-0.857	0.391	0.775	0.231	-0.976	0.329	0.432	1.389
TTYH1	0.257	0.298	0.863	0.388	1.294	0.386	0.761	0.446	0.721	2.321
TTTY15	0.188	0.296	0.636	0.525	1.207	0.357	0.58	0.562	0.676	2.154
TTTY14	-0.476	0.302	-1.573	0.116	0.622	0.188	-2.015	0.044	0.344	1.124
TTR	-0.129	0.296	-0.436	0.663	0.879	0.261	-0.465	0.642	0.491	1.571
TTPAL	0.788	0.31	2.544	0.011	2.2	0.682	1.76	0.078	1.198	4.038
TTPA	0.08	0.295	0.272	0.786	1.083	0.32	0.261	0.794	0.608	1.932
TTN	0.051	0.296	0.171	0.864	1.052	0.311	0.167	0.868	0.589	1.879
TTLL7	0.33	0.298	1.111	0.267	1.392	0.414	0.946	0.344	0.777	2.493
TTLL5	-0.062	0.296	-0.21	0.834	0.94	0.278	-0.216	0.829	0.526	1.679
TTLL4	0.169	0.297	0.571	0.568	1.184	0.351	0.525	0.6	0.662	2.119
TTLL12	0.392	0.298	1.315	0.189	1.479	0.441	1.088	0.277	0.825	2.652
TTLL1	-0.339	0.3	-1.127	0.26	0.713	0.214	-1.342	0.18	0.396	1.284
TTK	0.493	0.302	1.63	0.103	1.637	0.495	1.287	0.198	0.905	2.962
TTI2	-0.18	0.297	-0.607	0.544	0.835	0.248	-0.665	0.506	0.467	1.494
TTI1	-0.233	0.297	-0.784	0.433	0.792	0.235	-0.882	0.378	0.443	1.418
TTF2	0.58	0.306	1.898	0.058	1.786	0.546	1.44	0.15	0.981	3.251
TTF1	0.45	0.3	1.501	0.133	1.569	0.471	1.208	0.227	0.871	2.826
TTC9	0.275	0.298	0.923	0.356	1.316	0.392	0.807	0.42	0.734	2.359
TTC4	0.744	0.308	2.415	0.016	2.104	0.648	1.704	0.088	1.15	3.847
TTC39A	-0.198	0.298	-0.664	0.507	0.82	0.245	-0.734	0.463	0.457	1.472
TTC38	-0.579	0.306	-1.894	0.058	0.561	0.171	-2.565	0.01	0.308	1.02
TTC37	-0.329	0.3	-1.096	0.273	0.72	0.216	-1.298	0.194	0.399	1.296
TTC33	0.44	0.3	1.464	0.143	1.552	0.466	1.185	0.236	0.862	2.795
TTC31	-0.552	0.306	-1.808	0.071	0.576	0.176	-2.413	0.016	0.316	1.048
TTC30A	0.151	0.296	0.509	0.611	1.162	0.344	0.472	0.637	0.651	2.076
TTC28	-0.659	0.306	-2.155	0.031	0.517	0.158	-3.05	0.002	0.284	0.942
TTC27	0.34	0.3	1.135	0.256	1.405	0.421	0.962	0.336	0.781	2.529
TTC26	-0.027	0.297	-0.089	0.929	0.974	0.289	-0.091	0.928	0.544	1.743
TTC23	-0.055	0.296	-0.187	0.852	0.946	0.28	-0.192	0.848	0.53	1.69
TTC22	0.081	0.295	0.274	0.784	1.084	0.32	0.263	0.792	0.608	1.934
TTC19	-0.331	0.298	-1.112	0.266	0.718	0.214	-1.318	0.188	0.4	1.288
TTC17	0.64	0.306	2.093	0.036	1.897	0.58	1.546	0.122	1.042	3.455
TTC13	0.734	0.316	2.326	0.02	2.084	0.658	1.648	0.099	1.123	3.869
TTC12	-0.026	0.297	-0.088	0.93	0.974	0.289	-0.089	0.929	0.545	1.743
TTC1	-0.259	0.3	-0.862	0.389	0.772	0.232	-0.984	0.325	0.429	1.39

TTBK2	0.312	0.298	1.046	0.295	1.366	0.407	0.899	0.369	0.762	2.45
TST	0.388	0.3	1.293	0.196	1.473	0.441	1.072	0.284	0.819	2.65
TSSK2	-0.473	0.3	-1.578	0.114	0.623	0.187	-2.018	0.044	0.346	1.121
TSSK1B	1.264	0.332	3.812	0	3.539	1.173	2.164	0.03	1.848	6.776
TSSC4	0.464	0.303	1.532	0.125	1.59	0.481	1.226	0.22	0.879	2.878
TSR3	0.004	0.295	0.014	0.989	1.004	0.297	0.014	0.989	0.563	1.791
TSR2	0.245	0.296	0.828	0.408	1.277	0.378	0.735	0.463	0.716	2.28
TSR1	0.077	0.295	0.26	0.795	1.08	0.319	0.25	0.802	0.606	1.925
TSPYL5	0.027	0.295	0.093	0.926	1.028	0.304	0.092	0.927	0.576	1.834
TSPYL4	-0.149	0.296	-0.503	0.615	0.862	0.255	-0.542	0.587	0.482	1.539
TSPYL2	-0.222	0.297	-0.748	0.455	0.801	0.238	-0.837	0.402	0.448	1.433
TSPYL1	-0.321	0.297	-1.082	0.279	0.725	0.215	-1.276	0.202	0.406	1.298
TSPY1	0.529	0.303	1.747	0.081	1.698	0.514	1.357	0.175	0.938	3.075
TSPOAP1	-0.147	0.296	-0.496	0.62	0.863	0.256	-0.534	0.593	0.483	1.542
TSPO2	0.111	0.296	0.373	0.709	1.117	0.331	0.353	0.724	0.625	1.996
TSPO	-0.267	0.298	-0.896	0.37	0.766	0.228	-1.027	0.304	0.427	1.373
TSPAN9	0.307	0.298	1.03	0.303	1.359	0.405	0.887	0.375	0.758	2.437
TSPAN8	-0.134	0.296	-0.452	0.651	0.875	0.259	-0.483	0.629	0.489	1.564
TSPAN7	-0.55	0.303	-1.817	0.069	0.577	0.175	-2.423	0.015	0.319	1.044
TSPAN6	-0.163	0.296	-0.55	0.582	0.85	0.251	-0.597	0.55	0.476	1.517
TSPAN5	0.178	0.296	0.601	0.548	1.195	0.354	0.55	0.582	0.669	2.135
TSPAN4	-0.325	0.298	-1.093	0.274	0.722	0.215	-1.291	0.197	0.403	1.295
TSPAN32	0.121	0.296	0.407	0.684	1.128	0.334	0.383	0.702	0.631	2.017
TSPAN31	0.302	0.297	1.017	0.309	1.353	0.402	0.878	0.38	0.756	2.421
TSPAN3	-0.579	0.304	-1.907	0.057	0.56	0.17	-2.582	0.01	0.309	1.016
TSPAN2	0.043	0.296	0.145	0.885	1.044	0.309	0.142	0.887	0.584	1.866
TSPAN15	-0.056	0.295	-0.19	0.849	0.945	0.279	-0.195	0.845	0.53	1.687
TSPAN14	0.351	0.298	1.177	0.239	1.42	0.423	0.993	0.321	0.792	2.546
TSPAN13	0.42	0.299	1.404	0.16	1.522	0.455	1.147	0.251	0.847	2.734
TSPAN12	0.26	0.298	0.875	0.381	1.298	0.386	0.771	0.441	0.724	2.325
TSPAN1	-0.199	0.296	-0.672	0.501	0.819	0.243	-0.744	0.457	0.459	1.464
TSNAXIP1	0.115	0.295	0.389	0.697	1.122	0.331	0.368	0.713	0.629	2.002
TSNAX	0.023	0.295	0.077	0.939	1.023	0.302	0.076	0.94	0.574	1.823
TSN	0.719	0.315	2.281	0.023	2.052	0.646	1.627	0.104	1.107	3.804
TSKU	-0.473	0.302	-1.567	0.117	0.623	0.188	-2.004	0.045	0.344	1.126
TSKS	0.078	0.296	0.262	0.793	1.081	0.32	0.252	0.801	0.605	1.93
TSHZ2	0.335	0.3	1.118	0.264	1.398	0.419	0.95	0.342	0.777	2.516
TSHR	-0.24	0.296	-0.81	0.418	0.787	0.233	-0.915	0.36	0.44	1.406

TSHB	-0.326	0.297	-1.095	0.273	0.722	0.215	-1.295	0.195	0.403	1.293
TSGA10	-0.158	0.298	-0.531	0.596	0.854	0.254	-0.575	0.565	0.476	1.531
TSG101	0.362	0.299	1.211	0.226	1.437	0.43	1.016	0.31	0.799	2.583
TSFM	0.187	0.295	0.632	0.527	1.205	0.356	0.577	0.564	0.676	2.15
TSEN34	0.937	0.315	2.976	0.003	2.553	0.804	1.931	0.053	1.377	4.734
TSEN2	-0.165	0.296	-0.559	0.576	0.848	0.251	-0.608	0.543	0.475	1.513
TSC22D4	-0.052	0.297	-0.174	0.862	0.95	0.282	-0.179	0.858	0.53	1.7
TSC22D3	-0.501	0.307	-1.633	0.103	0.606	0.186	-2.119	0.034	0.332	1.106
TSC22D2	0.515	0.303	1.701	0.089	1.673	0.506	1.329	0.184	0.925	3.028
TSC22D1	0.021	0.295	0.071	0.944	1.021	0.302	0.07	0.944	0.572	1.822
TSC2	-0.933	0.321	-2.91	0.004	0.393	0.126	-4.809	0	0.21	0.737
TSC1	-0.025	0.297	-0.083	0.934	0.976	0.29	-0.084	0.933	0.545	1.747
TSBP1	-0.062	0.297	-0.21	0.833	0.939	0.279	-0.217	0.828	0.525	1.681
TRRAP	-0.203	0.296	-0.685	0.493	0.816	0.242	-0.76	0.447	0.457	1.459
TRPV6	0.406	0.298	1.361	0.173	1.5	0.447	1.119	0.263	0.837	2.69
TRPV5	0.444	0.303	1.467	0.142	1.559	0.472	1.185	0.236	0.862	2.821
TRPV4	0.238	0.297	0.799	0.424	1.268	0.377	0.711	0.477	0.708	2.272
TRPV2	0.073	0.296	0.247	0.805	1.076	0.318	0.238	0.812	0.602	1.922
TRPS1	-0.202	0.298	-0.678	0.498	0.817	0.243	-0.751	0.453	0.456	1.465
TRPM8	0.073	0.295	0.247	0.805	1.076	0.318	0.238	0.812	0.603	1.919
TRPM6	0.312	0.3	1.037	0.3	1.366	0.41	0.891	0.373	0.758	2.461
TRPM4	0.064	0.295	0.218	0.828	1.066	0.315	0.211	0.833	0.598	1.903
TRPM3	-0.399	0.299	-1.336	0.182	0.671	0.2	-1.641	0.101	0.374	1.205
TRPM2	0.07	0.297	0.237	0.813	1.073	0.318	0.229	0.819	0.6	1.918
TRPM1	0.172	0.296	0.581	0.562	1.188	0.352	0.533	0.594	0.665	2.122
TRPC7	0.348	0.3	1.159	0.246	1.417	0.426	0.979	0.328	0.786	2.553
TRPC6	-0.263	0.298	-0.884	0.376	0.768	0.229	-1.012	0.312	0.429	1.377
TRPC5	0.041	0.295	0.14	0.888	1.042	0.308	0.137	0.891	0.585	1.858
TRPC4AP	0.322	0.298	1.08	0.28	1.38	0.411	0.924	0.356	0.769	2.474
TRPC4	0.156	0.295	0.528	0.597	1.169	0.345	0.489	0.625	0.655	2.085
TRPC3	-0.23	0.297	-0.775	0.438	0.794	0.236	-0.872	0.383	0.444	1.421
TRPC2	-0.323	0.298	-1.085	0.278	0.724	0.216	-1.28	0.2	0.404	1.298
TRPC1	0.019	0.296	0.064	0.949	1.019	0.302	0.064	0.949	0.57	1.821
TRPA1	0.063	0.295	0.212	0.832	1.065	0.315	0.205	0.837	0.597	1.9
TROAP	0.71	0.31	2.291	0.022	2.034	0.63	1.64	0.101	1.108	3.733
TRO	-0.411	0.3	-1.372	0.17	0.663	0.199	-1.697	0.09	0.369	1.193
TRNAU1AP	1.03	0.318	3.241	0.001	2.8	0.889	2.024	0.043	1.502	5.218
TRMU	0.211	0.295	0.714	0.475	1.235	0.365	0.644	0.52	0.692	2.203

TRMT9B	-0.181	0.296	-0.61	0.542	0.835	0.247	-0.669	0.503	0.467	1.491
TRMT61B	0.05	0.295	0.17	0.865	1.051	0.31	0.166	0.869	0.589	1.875
TRMT61A	0.487	0.3	1.622	0.105	1.627	0.489	1.284	0.199	0.903	2.931
TRMT5	-0.074	0.296	-0.25	0.803	0.929	0.275	-0.26	0.795	0.52	1.66
TRMT44	-0.344	0.3	-1.147	0.251	0.709	0.213	-1.368	0.171	0.394	1.276
TRMT2B	-0.291	0.299	-0.973	0.331	0.748	0.223	-1.129	0.259	0.416	1.343
TRMT2A	-0.493	0.303	-1.628	0.103	0.611	0.185	-2.104	0.035	0.338	1.106
TRMT1L	-0.187	0.3	-0.623	0.533	0.83	0.249	-0.685	0.493	0.461	1.493
TRMT13	-0.135	0.296	-0.458	0.647	0.873	0.258	-0.49	0.624	0.489	1.56
TRMT12	0.275	0.298	0.924	0.355	1.317	0.392	0.808	0.419	0.735	2.361
TRMT112	0.255	0.298	0.855	0.392	1.29	0.384	0.755	0.45	0.72	2.312
TRMT11	0.537	0.302	1.776	0.076	1.711	0.517	1.374	0.169	0.946	3.094
TRMT1	0.177	0.296	0.596	0.551	1.193	0.354	0.546	0.585	0.667	2.133
TRMO	0.177	0.296	0.598	0.55	1.194	0.353	0.548	0.584	0.668	2.133
TRIT1	1.01	0.321	3.147	0.002	2.746	0.881	1.981	0.048	1.464	5.151
TRIP6	-0.17	0.296	-0.573	0.566	0.844	0.25	-0.625	0.532	0.472	1.508
TRIP4	0.368	0.297	1.238	0.216	1.445	0.429	1.036	0.3	0.807	2.586
TRIP13	0.807	0.315	2.563	0.01	2.24	0.705	1.759	0.079	1.209	4.151
TRIP12	-0.4	0.298	-1.342	0.18	0.67	0.2	-1.65	0.099	0.374	1.202
TRIP11	0.164	0.296	0.555	0.579	1.178	0.348	0.512	0.609	0.66	2.103
TRIP10	-0.494	0.302	-1.636	0.102	0.61	0.184	-2.115	0.034	0.338	1.103
TRIOBP	0.132	0.296	0.447	0.655	1.142	0.338	0.419	0.675	0.639	2.04
TRIO	-0.571	0.303	-1.885	0.059	0.565	0.171	-2.541	0.011	0.312	1.023
TRIM9	0.177	0.296	0.598	0.55	1.193	0.353	0.548	0.584	0.669	2.13
TRIM8	-0.201	0.296	-0.678	0.498	0.818	0.242	-0.751	0.453	0.458	1.462
TRIM68	0.3	0.3	1.002	0.316	1.35	0.405	0.865	0.387	0.75	2.429
TRIM66	0.317	0.3	1.056	0.291	1.373	0.412	0.905	0.365	0.762	2.472
TRIM62	-0.032	0.295	-0.109	0.913	0.968	0.286	-0.11	0.912	0.543	1.727
TRIM58	-0.286	0.297	-0.963	0.336	0.751	0.223	-1.115	0.265	0.419	1.345
TRIM52	0.077	0.296	0.259	0.796	1.08	0.319	0.249	0.803	0.605	1.927
TRIM5	0.341	0.3	1.139	0.255	1.406	0.421	0.965	0.335	0.782	2.53
TRIM46	0.03	0.296	0.102	0.918	1.031	0.305	0.101	0.92	0.577	1.842
TRIM45	-0.128	0.296	-0.433	0.665	0.88	0.26	-0.462	0.644	0.493	1.57
TRIM44	0.025	0.296	0.084	0.933	1.025	0.303	0.083	0.934	0.574	1.831
TRIM38	0.392	0.3	1.308	0.191	1.48	0.444	1.082	0.279	0.822	2.665
TRIM37	0.18	0.296	0.606	0.544	1.197	0.355	0.555	0.579	0.67	2.139
TRIM36	0.019	0.295	0.063	0.949	1.019	0.301	0.063	0.95	0.571	1.817
TRIM33	0.15	0.295	0.508	0.612	1.162	0.343	0.472	0.637	0.651	2.073

TRIM32	0.688	0.306	2.248	0.025	1.99	0.609	1.625	0.104	1.092	3.627
TRIM31	-0.029	0.295	-0.098	0.922	0.972	0.287	-0.099	0.921	0.545	1.733
TRIM3	0.376	0.298	1.263	0.206	1.457	0.434	1.053	0.292	0.813	2.611
TRIM29	-0.586	0.302	-1.937	0.053	0.557	0.168	-2.634	0.008	0.308	1.007
TRIM28	-0.777	0.315	-2.468	0.014	0.46	0.145	-3.732	0	0.248	0.852
TRIM27	0.24	0.297	0.807	0.42	1.271	0.378	0.718	0.473	0.71	2.277
TRIM26	0.166	0.298	0.555	0.579	1.18	0.352	0.512	0.609	0.658	2.117
TRIM25	-0.267	0.298	-0.899	0.369	0.765	0.228	-1.03	0.303	0.427	1.371
TRIM24	-0.191	0.296	-0.645	0.519	0.826	0.245	-0.71	0.477	0.462	1.476
TRIM23	-0.524	0.306	-1.711	0.087	0.592	0.181	-2.249	0.025	0.325	1.079
TRIM22	-0.551	0.303	-1.819	0.069	0.577	0.175	-2.425	0.015	0.319	1.044
TRIM21	-0.003	0.295	-0.01	0.992	0.997	0.294	-0.01	0.992	0.559	1.778
TRIM2	-0.389	0.299	-1.3	0.193	0.678	0.203	-1.59	0.112	0.377	1.218
TRIM17	0.08	0.296	0.269	0.788	1.083	0.321	0.259	0.796	0.606	1.935
TRIM16	0.098	0.295	0.333	0.739	1.103	0.326	0.317	0.751	0.618	1.968
TRIM15	0.082	0.298	0.274	0.784	1.085	0.324	0.263	0.792	0.605	1.947
TRIM14	0.205	0.296	0.691	0.49	1.227	0.363	0.625	0.532	0.687	2.193
TRIM13	-0.156	0.296	-0.526	0.599	0.856	0.254	-0.569	0.569	0.479	1.53
TRIM10	0.041	0.295	0.14	0.888	1.042	0.308	0.138	0.891	0.584	1.859
TRIL	-0.03	0.295	-0.102	0.919	0.97	0.286	-0.103	0.918	0.544	1.73
TRIB3	0.081	0.295	0.273	0.785	1.084	0.32	0.262	0.793	0.608	1.934
TRIB2	-0.129	0.296	-0.438	0.662	0.879	0.26	-0.467	0.64	0.492	1.568
TRIB1	-0.047	0.295	-0.16	0.873	0.954	0.282	-0.164	0.869	0.534	1.702
TRIAP1	0.86	0.315	2.731	0.006	2.364	0.745	1.831	0.067	1.275	4.382
TRHR	-0.09	0.297	-0.302	0.762	0.914	0.271	-0.316	0.752	0.511	1.635
TRHDE	-0.616	0.31	-1.988	0.047	0.54	0.167	-2.748	0.006	0.294	0.991
TRH	-0.464	0.302	-1.536	0.125	0.629	0.19	-1.954	0.051	0.348	1.137
TREML2	-0.567	0.303	-1.868	0.062	0.567	0.172	-2.513	0.012	0.313	1.028
TREM2	-0.585	0.31	-1.887	0.059	0.557	0.173	-2.564	0.01	0.303	1.023
TREM1	0.598	0.302	1.976	0.048	1.818	0.55	1.488	0.137	1.005	3.288
TREH	0.606	0.306	1.98	0.048	1.832	0.56	1.486	0.137	1.006	3.336
TRDN	0.198	0.298	0.667	0.505	1.22	0.363	0.605	0.545	0.68	2.186
TRDMT1	-0.37	0.3	-1.232	0.218	0.691	0.207	-1.49	0.136	0.384	1.244
TRBC1	-0.518	0.303	-1.714	0.087	0.595	0.18	-2.246	0.025	0.329	1.077
TRAV8-3	-0.155	0.296	-0.523	0.601	0.857	0.253	-0.566	0.571	0.48	1.53
TRAV12-2	0.375	0.299	1.257	0.209	1.456	0.435	1.048	0.295	0.811	2.614
TRAT1	-0.298	0.298	-1	0.317	0.743	0.221	-1.165	0.244	0.414	1.331
TRAPPC9	0.625	0.31	2.015	0.044	1.869	0.58	1.498	0.134	1.017	3.433

TRAPPC8	-0.087	0.297	-0.293	0.769	0.917	0.272	-0.306	0.759	0.512	1.64
TRAPPC6A	0.004	0.296	0.014	0.989	1.004	0.297	0.014	0.989	0.562	1.795
TRAPPC3	0.208	0.296	0.701	0.483	1.231	0.365	0.633	0.527	0.689	2.199
TRAPPC2L	0.313	0.298	1.051	0.293	1.367	0.407	0.902	0.367	0.763	2.45
TRAPPC2	0.364	0.298	1.22	0.222	1.438	0.429	1.023	0.306	0.802	2.579
TRAPPC13	0.198	0.297	0.664	0.506	1.218	0.362	0.603	0.547	0.68	2.182
TRAPPC12	-0.399	0.3	-1.33	0.183	0.671	0.201	-1.634	0.102	0.373	1.208
TRAPPC11	-0.276	0.3	-0.918	0.359	0.759	0.228	-1.057	0.29	0.421	1.367
TRAPPC10	-0.08	0.295	-0.272	0.786	0.923	0.273	-0.283	0.777	0.517	1.646
TRAP1	0.286	0.298	0.961	0.337	1.331	0.396	0.835	0.403	0.743	2.386
TRANK1	-0.266	0.296	-0.897	0.37	0.767	0.227	-1.027	0.304	0.429	1.37
TRAM2	-0.265	0.296	-0.896	0.37	0.767	0.227	-1.026	0.305	0.429	1.371
TRAM1	-0.078	0.295	-0.265	0.791	0.925	0.273	-0.276	0.783	0.518	1.65
TRAK2	-0.128	0.295	-0.434	0.664	0.88	0.26	-0.463	0.643	0.493	1.569
TRAK1	-0.826	0.315	-2.624	0.009	0.438	0.138	-4.079	0	0.236	0.811
TRAIP	0.204	0.296	0.691	0.489	1.227	0.363	0.625	0.532	0.687	2.191
TRAFD1	-0.395	0.3	-1.32	0.187	0.673	0.202	-1.619	0.106	0.374	1.211
TRAF6	0.118	0.296	0.4	0.689	1.126	0.333	0.377	0.706	0.63	2.01
TRAF5	-0.036	0.295	-0.122	0.903	0.964	0.285	-0.125	0.901	0.541	1.721
TRAF4	0.436	0.298	1.465	0.143	1.547	0.461	1.187	0.235	0.863	2.773
TRAF3IP3	0.079	0.295	0.267	0.79	1.082	0.32	0.257	0.798	0.606	1.931
TRAF3IP2	-0.198	0.296	-0.668	0.504	0.82	0.243	-0.739	0.46	0.459	1.466
TRAF3IP1	0.011	0.295	0.038	0.97	1.011	0.298	0.037	0.97	0.567	1.803
TRAF3	-0.401	0.3	-1.337	0.181	0.67	0.201	-1.644	0.1	0.372	1.205
TRAF2	0.307	0.298	1.032	0.302	1.36	0.405	0.888	0.374	0.759	2.438
TRAF1	-0.628	0.304	-2.062	0.039	0.534	0.162	-2.87	0.004	0.294	0.969
TRADD	0.786	0.311	2.528	0.011	2.195	0.683	1.751	0.08	1.193	4.039
TRAC	-0.674	0.307	-2.198	0.028	0.51	0.156	-3.138	0.002	0.279	0.929
TRABD	-0.335	0.3	-1.119	0.263	0.715	0.214	-1.33	0.184	0.398	1.286
TRA2B	0.045	0.295	0.152	0.879	1.046	0.309	0.149	0.882	0.586	1.865
TRA2A	0.238	0.297	0.8	0.424	1.268	0.377	0.712	0.476	0.708	2.271
TPX2	0.694	0.31	2.239	0.025	2.001	0.62	1.615	0.106	1.09	3.674
TPTE	0.239	0.296	0.806	0.42	1.269	0.376	0.717	0.473	0.711	2.268
TPT1P8	-0.127	0.297	-0.429	0.668	0.88	0.261	-0.458	0.647	0.492	1.575
TPT1	-0.378	0.306	-1.237	0.216	0.685	0.209	-1.503	0.133	0.376	1.247
TPST2	-0.502	0.302	-1.66	0.097	0.605	0.183	-2.156	0.031	0.335	1.095
TPST1	0.495	0.301	1.644	0.1	1.64	0.493	1.297	0.195	0.909	2.957
TPSG1	-0.255	0.297	-0.859	0.39	0.775	0.23	-0.978	0.328	0.433	1.386

TPSD1	-0.441	0.306	-1.441	0.15	0.643	0.197	-1.811	0.07	0.353	1.172
TPSB2	-0.754	0.315	-2.394	0.017	0.47	0.148	-3.575	0	0.254	0.872
TPSAB1	-0.748	0.315	-2.375	0.018	0.473	0.149	-3.534	0	0.255	0.877
TPRKB	0.499	0.303	1.648	0.099	1.647	0.499	1.298	0.194	0.91	2.981
TPRA1	-0.255	0.296	-0.859	0.39	0.775	0.23	-0.979	0.328	0.434	1.386
TPR	-0.231	0.297	-0.779	0.436	0.794	0.235	-0.877	0.381	0.444	1.419
TPPP3	-0.523	0.306	-1.709	0.087	0.593	0.181	-2.245	0.025	0.326	1.08
TPPP	-0.039	0.298	-0.131	0.895	0.962	0.286	-0.134	0.893	0.537	1.723
TPP2	0.207	0.298	0.696	0.486	1.23	0.366	0.629	0.529	0.687	2.205
TPP1	-0.568	0.303	-1.877	0.061	0.567	0.172	-2.527	0.012	0.313	1.026
TPO	-0.192	0.298	-0.644	0.52	0.826	0.246	-0.71	0.478	0.461	1.48
TPMT	-0.125	0.296	-0.424	0.672	0.882	0.261	-0.451	0.652	0.494	1.576
TPM4	-0.377	0.3	-1.255	0.209	0.686	0.206	-1.525	0.127	0.381	1.236
TPM3	-0.248	0.298	-0.832	0.405	0.781	0.232	-0.944	0.345	0.436	1.399
TPM2	0.017	0.295	0.057	0.955	1.017	0.3	0.056	0.955	0.57	1.814
TPM1	-0.389	0.3	-1.296	0.195	0.678	0.203	-1.584	0.113	0.377	1.22
TPK1	-0.101	0.295	-0.342	0.732	0.904	0.267	-0.36	0.719	0.507	1.613
TPI1	0.561	0.302	1.854	0.064	1.752	0.53	1.42	0.156	0.969	3.168
TPH1	-0.125	0.296	-0.423	0.673	0.882	0.261	-0.45	0.653	0.494	1.577
TPGS2	-0.024	0.297	-0.081	0.935	0.976	0.29	-0.082	0.934	0.546	1.746
TPD52L2	-0.063	0.295	-0.213	0.831	0.939	0.277	-0.22	0.826	0.526	1.675
TPD52L1	0.828	0.315	2.629	0.009	2.288	0.72	1.788	0.074	1.234	4.239
TPD52	0.367	0.298	1.231	0.218	1.443	0.43	1.03	0.303	0.805	2.587
TPCN1	-0.125	0.296	-0.423	0.672	0.882	0.261	-0.45	0.652	0.494	1.576
TPBG	-0.455	0.303	-1.503	0.133	0.635	0.192	-1.903	0.057	0.351	1.148
TP73-AS1	-0.452	0.3	-1.509	0.131	0.636	0.191	-1.908	0.056	0.354	1.145
TP73	0.329	0.298	1.107	0.268	1.39	0.414	0.943	0.346	0.776	2.491
TP63	-0.693	0.306	-2.262	0.024	0.5	0.153	-3.263	0.001	0.274	0.912
TP53TG5	0.459	0.299	1.537	0.124	1.583	0.473	1.232	0.218	0.881	2.843
TP53TG1	-0.134	0.295	-0.452	0.651	0.875	0.259	-0.484	0.628	0.49	1.561
TP53I3	-0.373	0.3	-1.242	0.214	0.689	0.207	-1.506	0.132	0.383	1.24
TP53I11	-0.365	0.3	-1.218	0.223	0.694	0.208	-1.47	0.142	0.385	1.249
TP53BP2	-0.135	0.296	-0.456	0.648	0.874	0.259	-0.489	0.625	0.489	1.561
TP53BP1	0.056	0.296	0.189	0.85	1.058	0.313	0.184	0.854	0.593	1.887
TP53AIP1	-0.386	0.298	-1.293	0.196	0.68	0.203	-1.578	0.115	0.379	1.22
TP53	-0.163	0.295	-0.553	0.58	0.849	0.251	-0.601	0.548	0.476	1.515
TOX4	0.164	0.295	0.557	0.578	1.179	0.348	0.513	0.608	0.661	2.103
TOX3	-0.185	0.296	-0.623	0.533	0.831	0.246	-0.684	0.494	0.465	1.486

TOX	-0.055	0.295	-0.187	0.852	0.946	0.279	-0.192	0.848	0.53	1.688
TOR4A	0.296	0.298	0.993	0.321	1.344	0.4	0.86	0.39	0.75	2.41
TOR3A	0.593	0.302	1.962	0.05	1.81	0.548	1.48	0.139	1.001	3.275
TOR1B	0.969	0.321	3.017	0.003	2.636	0.847	1.932	0.053	1.404	4.949
TOR1AIP2	0.566	0.306	1.851	0.064	1.761	0.539	1.413	0.158	0.967	3.208
TOR1AIP1	-0.325	0.298	-1.091	0.275	0.723	0.215	-1.289	0.197	0.403	1.295
TOR1A	-0.083	0.296	-0.282	0.778	0.92	0.272	-0.294	0.769	0.516	1.642
TOPORS	-0.368	0.302	-1.218	0.223	0.692	0.209	-1.473	0.141	0.382	1.251
TOPBP1	0.529	0.302	1.749	0.08	1.697	0.513	1.358	0.174	0.938	3.069
TOP3B	-0.733	0.315	-2.33	0.02	0.48	0.151	-3.438	0.001	0.259	0.89
TOP3A	0.293	0.296	0.987	0.324	1.34	0.397	0.856	0.392	0.749	2.395
TOP2B	-0.391	0.3	-1.302	0.193	0.677	0.203	-1.593	0.111	0.376	1.218
TOP2A	0.834	0.315	2.649	0.008	2.302	0.725	1.797	0.072	1.242	4.267
TOP1	0.075	0.297	0.253	0.8	1.078	0.32	0.244	0.807	0.603	1.928
TONSL	0.058	0.295	0.198	0.843	1.06	0.313	0.192	0.848	0.594	1.892
TOMM70	0.549	0.302	1.815	0.069	1.731	0.523	1.397	0.162	0.957	3.13
TOMM40	0.821	0.311	2.642	0.008	2.274	0.707	1.802	0.072	1.236	4.182
TOMM34	0.215	0.298	0.722	0.47	1.24	0.37	0.649	0.516	0.691	2.226
TOMM22	0.245	0.298	0.82	0.412	1.277	0.381	0.728	0.467	0.712	2.291
TOMM20	0.041	0.295	0.139	0.89	1.042	0.308	0.136	0.892	0.584	1.858
TOM1L2	-0.443	0.3	-1.477	0.14	0.642	0.192	-1.858	0.063	0.357	1.156
TOM1L1	0.047	0.297	0.16	0.873	1.049	0.311	0.156	0.876	0.586	1.876
TOM1	-0.198	0.296	-0.668	0.504	0.82	0.243	-0.739	0.46	0.459	1.466
TOLLIP	-0.255	0.3	-0.851	0.395	0.775	0.232	-0.969	0.332	0.431	1.394
TOGARAM1	-0.754	0.315	-2.395	0.017	0.471	0.148	-3.575	0	0.254	0.872
TOE1	-0.124	0.297	-0.417	0.677	0.884	0.262	-0.444	0.657	0.494	1.581
TOB2	0.009	0.295	0.031	0.975	1.009	0.298	0.031	0.975	0.566	1.799
TOB1	0.179	0.298	0.601	0.548	1.196	0.357	0.55	0.582	0.667	2.145
TNXB	0.482	0.303	1.591	0.112	1.619	0.49	1.263	0.207	0.894	2.93
TNS4	0.444	0.302	1.471	0.141	1.56	0.471	1.187	0.235	0.863	2.82
TNS3	-0.235	0.296	-0.792	0.428	0.791	0.234	-0.893	0.372	0.443	1.413
TNS2	-0.536	0.306	-1.754	0.079	0.585	0.179	-2.321	0.02	0.321	1.065
TNS1	-0.046	0.296	-0.156	0.876	0.955	0.283	-0.16	0.873	0.535	1.705
TNRC6B	0.057	0.295	0.193	0.847	1.059	0.313	0.188	0.851	0.593	1.889
TNR	-0.325	0.297	-1.096	0.273	0.722	0.214	-1.295	0.195	0.404	1.293
TNPO3	-0.179	0.296	-0.604	0.546	0.836	0.248	-0.661	0.509	0.468	1.494
TNPO2	0.898	0.313	2.867	0.004	2.455	0.769	1.892	0.059	1.329	4.538
TNPO1	0.437	0.3	1.455	0.146	1.547	0.464	1.179	0.238	0.86	2.785

TNP2	0.158	0.296	0.534	0.593	1.171	0.347	0.494	0.621	0.656	2.093
TNP1	0.587	0.303	1.939	0.053	1.798	0.544	1.467	0.142	0.994	3.254
TNNT3	-0.411	0.3	-1.372	0.17	0.663	0.199	-1.696	0.09	0.369	1.193
TNNT2	0.416	0.3	1.387	0.165	1.515	0.454	1.135	0.256	0.842	2.726
TNNT1	0.259	0.297	0.871	0.384	1.295	0.384	0.768	0.443	0.724	2.317
TNNI3	0.418	0.3	1.397	0.163	1.519	0.455	1.141	0.254	0.845	2.733
TNNI2	-0.09	0.296	-0.304	0.761	0.914	0.271	-0.319	0.75	0.511	1.633
TNNI1	0.02	0.296	0.066	0.947	1.02	0.302	0.065	0.948	0.571	1.822
TNNC2	-0.058	0.295	-0.197	0.844	0.943	0.279	-0.203	0.839	0.529	1.683
TNNC1	-0.041	0.295	-0.138	0.89	0.96	0.283	-0.141	0.888	0.538	1.712
TNN	-0.536	0.3	-1.786	0.074	0.585	0.176	-2.363	0.018	0.325	1.054
TNMD	0.439	0.3	1.463	0.144	1.551	0.465	1.184	0.236	0.861	2.792
TNKS2	-0.664	0.306	-2.172	0.03	0.515	0.157	-3.084	0.002	0.283	0.937
TNKS	-0.643	0.31	-2.076	0.038	0.526	0.163	-2.912	0.004	0.287	0.965
TNK2	0.31	0.298	1.042	0.298	1.364	0.406	0.896	0.37	0.761	2.444
TNK1	-0.113	0.295	-0.383	0.702	0.893	0.264	-0.406	0.685	0.5	1.593
TNIP3	-0.112	0.296	-0.377	0.706	0.894	0.265	-0.399	0.69	0.501	1.597
TNIP2	-0.203	0.296	-0.685	0.493	0.817	0.241	-0.76	0.447	0.458	1.458
TNIP1	-0.921	0.321	-2.871	0.004	0.398	0.128	-4.712	0	0.212	0.747
TNIK	0.407	0.299	1.363	0.173	1.503	0.449	1.119	0.263	0.836	2.7
TNFSF9	-0.351	0.298	-1.179	0.238	0.704	0.21	-1.412	0.158	0.393	1.262
TNFSF8	-0.385	0.298	-1.289	0.197	0.681	0.203	-1.572	0.116	0.379	1.222
TNFSF4	-0.738	0.315	-2.346	0.019	0.478	0.15	-3.472	0.001	0.258	0.886
TNFSF18	-0.146	0.299	-0.49	0.624	0.864	0.258	-0.528	0.598	0.481	1.551
TNFSF15	0.462	0.302	1.528	0.126	1.587	0.48	1.224	0.221	0.878	2.87
TNFSF14	-0.019	0.295	-0.064	0.949	0.981	0.29	-0.065	0.948	0.55	1.75
TNFSF13	-0.082	0.296	-0.276	0.782	0.921	0.273	-0.288	0.773	0.516	1.647
TNFSF11	-0.082	0.296	-0.277	0.782	0.921	0.272	-0.289	0.773	0.516	1.644
TNFSF10	0.041	0.295	0.14	0.889	1.042	0.308	0.137	0.891	0.584	1.86
TNFRSF9	-0.188	0.296	-0.635	0.525	0.829	0.245	-0.698	0.485	0.464	1.479
TNFRSF8	-0.138	0.295	-0.467	0.641	0.871	0.257	-0.5	0.617	0.488	1.555
TNFRSF4	0.643	0.306	2.105	0.035	1.903	0.582	1.553	0.121	1.045	3.464
TNFRSF25	-0.625	0.304	-2.053	0.04	0.535	0.163	-2.853	0.004	0.295	0.972
TNFRSF21	0.45	0.3	1.502	0.133	1.569	0.47	1.21	0.226	0.872	2.822
TNFRSF1B	-0.312	0.297	-1.05	0.294	0.732	0.217	-1.232	0.218	0.409	1.31
TNFRSF1A	-0.62	0.304	-2.041	0.041	0.538	0.163	-2.829	0.005	0.296	0.976
TNFRSF17	-0.313	0.298	-1.049	0.294	0.731	0.218	-1.232	0.218	0.408	1.312
TNFRSF14	-0.047	0.295	-0.159	0.874	0.954	0.282	-0.162	0.871	0.535	1.701

TNFRSF13B	-0.32	0.298	-1.075	0.283	0.726	0.216	-1.267	0.205	0.405	1.302
TNFRSF12A	0.455	0.3	1.516	0.13	1.575	0.472	1.218	0.223	0.875	2.836
TNFRSF11B	-0.463	0.303	-1.531	0.126	0.629	0.19	-1.947	0.052	0.348	1.139
TNFRSF11A	0.461	0.3	1.54	0.124	1.586	0.475	1.234	0.217	0.882	2.854
TNFRSF10D	0.012	0.295	0.04	0.968	1.012	0.299	0.04	0.968	0.568	1.804
TNFRSF10C	0.418	0.298	1.402	0.161	1.519	0.453	1.146	0.252	0.847	2.725
TNFRSF10B	0.212	0.296	0.714	0.475	1.236	0.366	0.643	0.52	0.691	2.208
TNFAIP8	-0.424	0.299	-1.415	0.157	0.655	0.196	-1.762	0.078	0.364	1.177
TNFAIP6	-0.418	0.3	-1.395	0.163	0.658	0.197	-1.732	0.083	0.366	1.184
TNFAIP3	0.149	0.296	0.504	0.614	1.161	0.344	0.468	0.64	0.65	2.076
TNFAIP2	0.268	0.296	0.906	0.365	1.308	0.387	0.795	0.427	0.732	2.337
TNFAIP1	-0.63	0.306	-2.058	0.04	0.533	0.163	-2.866	0.004	0.292	0.97
TNF	0.303	0.298	1.017	0.309	1.354	0.404	0.877	0.38	0.755	2.43
TNC	0.268	0.298	0.9	0.368	1.307	0.389	0.79	0.43	0.729	2.343
TMX4	-0.344	0.297	-1.159	0.246	0.709	0.21	-1.384	0.166	0.396	1.268
TMX2	-0.104	0.296	-0.35	0.726	0.901	0.267	-0.369	0.712	0.504	1.611
TMX1	0.196	0.296	0.663	0.507	1.217	0.36	0.602	0.547	0.681	2.175
TMUB2	0.117	0.296	0.395	0.693	1.124	0.332	0.373	0.709	0.63	2.006
TMSB4Y	0.305	0.299	1.019	0.308	1.356	0.405	0.879	0.38	0.755	2.436
TMSB4X	0.149	0.296	0.503	0.615	1.16	0.344	0.467	0.641	0.649	2.074
TMSB10	0.773	0.315	2.458	0.014	2.167	0.682	1.712	0.087	1.17	4.016
TMPRSS6	-0.03	0.296	-0.102	0.919	0.97	0.287	-0.103	0.918	0.544	1.732
TMPRSS5	0.229	0.299	0.768	0.443	1.258	0.376	0.686	0.493	0.7	2.259
TMPRSS4	0.201	0.295	0.682	0.495	1.223	0.361	0.618	0.537	0.686	2.182
TMPRSS3	-0.615	0.306	-2.013	0.044	0.541	0.165	-2.781	0.005	0.297	0.984
TMPRSS2	0.654	0.31	2.113	0.035	1.924	0.596	1.551	0.121	1.048	3.53
TMPRSS15	0.568	0.306	1.857	0.063	1.764	0.539	1.417	0.156	0.969	3.212
TMPRSS11E	0.293	0.298	0.985	0.325	1.341	0.399	0.854	0.393	0.748	2.403
TMPRSS11D	0.219	0.296	0.742	0.458	1.245	0.368	0.666	0.505	0.697	2.223
TMPO	0.145	0.295	0.492	0.623	1.156	0.342	0.458	0.647	0.648	2.063
TMOD3	-0.256	0.301	-0.851	0.395	0.774	0.233	-0.969	0.332	0.43	1.396
TMOD2	0.852	0.315	2.705	0.007	2.344	0.738	1.821	0.069	1.264	4.344
TMOD1	-0.187	0.298	-0.629	0.529	0.829	0.247	-0.692	0.489	0.463	1.486
TMLHE	0.015	0.295	0.052	0.958	1.016	0.3	0.052	0.959	0.57	1.811
TMF1	-0.24	0.297	-0.81	0.418	0.786	0.233	-0.915	0.36	0.439	1.407
TMEM9B	-0.447	0.302	-1.479	0.139	0.64	0.193	-1.865	0.062	0.354	1.156
TMEM97	0.565	0.302	1.871	0.061	1.76	0.532	1.429	0.153	0.973	3.183
TMEM92-AS1	0.044	0.296	0.148	0.883	1.045	0.309	0.145	0.885	0.585	1.865

TMEM8B	-0.466	0.3	-1.554	0.12	0.628	0.188	-1.98	0.048	0.349	1.129
TMEM87A	-0.862	0.315	-2.738	0.006	0.422	0.133	-4.345	0	0.228	0.783
TMEM80	-0.08	0.297	-0.269	0.788	0.923	0.274	-0.28	0.78	0.516	1.652
TMEM74B	0.044	0.296	0.149	0.882	1.045	0.31	0.146	0.884	0.585	1.869
TMEM70	0.612	0.303	2.024	0.043	1.845	0.558	1.514	0.13	1.02	3.338
TMEM63A	-0.078	0.295	-0.264	0.792	0.925	0.273	-0.275	0.784	0.518	1.65
TMEM62	-0.246	0.298	-0.825	0.409	0.782	0.233	-0.936	0.349	0.436	1.402
TMEM59L	0.938	0.316	2.972	0.003	2.555	0.806	1.928	0.054	1.376	4.743
TMEM59	-0.249	0.301	-0.829	0.407	0.779	0.234	-0.942	0.346	0.432	1.405
TMEM53	-0.01	0.295	-0.033	0.974	0.99	0.292	-0.033	0.974	0.555	1.766
TMEM51	0.316	0.297	1.064	0.287	1.371	0.407	0.913	0.361	0.767	2.453
TMEM50B	-0.414	0.307	-1.349	0.177	0.661	0.203	-1.671	0.095	0.362	1.206
TMEM50A	-0.933	0.321	-2.911	0.004	0.393	0.126	-4.811	0	0.21	0.737
TMEM47	-0.325	0.298	-1.094	0.274	0.722	0.215	-1.292	0.196	0.403	1.294
TMEM45A	-0.321	0.298	-1.078	0.281	0.725	0.216	-1.272	0.204	0.404	1.3
TMEM43	-0.715	0.307	-2.331	0.02	0.489	0.15	-3.405	0.001	0.268	0.892
TMEM41B	-0.125	0.296	-0.421	0.674	0.883	0.261	-0.449	0.654	0.494	1.577
TMEM40	0.68	0.31	2.196	0.028	1.974	0.611	1.593	0.111	1.076	3.623
TMEM39B	-0.092	0.295	-0.312	0.755	0.912	0.269	-0.327	0.744	0.511	1.627
TMEM39A	-0.536	0.303	-1.773	0.076	0.585	0.177	-2.346	0.019	0.323	1.058
TMEM38B	-0.404	0.3	-1.346	0.178	0.668	0.2	-1.659	0.097	0.371	1.202
TMEM35A	-0.493	0.303	-1.625	0.104	0.611	0.185	-2.1	0.036	0.337	1.107
TMEM33	0.013	0.295	0.042	0.966	1.013	0.299	0.042	0.966	0.567	1.807
TMEM30B	-0.512	0.306	-1.671	0.095	0.6	0.184	-2.182	0.029	0.329	1.092
TMEM30A	-0.287	0.297	-0.965	0.334	0.751	0.223	-1.118	0.264	0.419	1.344
TMEM268	-0.247	0.298	-0.827	0.408	0.781	0.233	-0.938	0.348	0.436	1.402
TMEM267	0.527	0.3	1.756	0.079	1.694	0.509	1.365	0.172	0.941	3.052
TMEM260	-0.174	0.296	-0.589	0.556	0.84	0.248	-0.643	0.52	0.471	1.5
TMEM259	-0.443	0.306	-1.448	0.148	0.642	0.197	-1.822	0.068	0.352	1.17
TMEM258	0.158	0.296	0.535	0.593	1.172	0.347	0.494	0.621	0.655	2.095
TMEM255A	-0.169	0.297	-0.57	0.569	0.844	0.251	-0.621	0.534	0.471	1.511
TMEM254	-0.606	0.306	-1.983	0.047	0.545	0.167	-2.727	0.006	0.3	0.993
TMEM251	-0.207	0.298	-0.694	0.488	0.813	0.242	-0.771	0.441	0.453	1.458
TMEM248	0.036	0.295	0.121	0.903	1.036	0.306	0.119	0.905	0.581	1.848
TMEM243	-0.058	0.295	-0.197	0.844	0.944	0.278	-0.203	0.839	0.529	1.682
TMEM242	0.018	0.295	0.062	0.95	1.019	0.301	0.062	0.951	0.571	1.816
TMEM238L	0.17	0.295	0.576	0.564	1.186	0.35	0.53	0.596	0.665	2.115
TMEM231	0.012	0.295	0.04	0.968	1.012	0.299	0.039	0.969	0.567	1.805

TMEM230	-0.633	0.306	-2.069	0.039	0.531	0.162	-2.886	0.004	0.291	0.967
TMEM223	0.317	0.298	1.064	0.287	1.373	0.409	0.912	0.362	0.766	2.463
TMEM222	-0.228	0.298	-0.767	0.443	0.796	0.237	-0.861	0.389	0.444	1.426
TMEM214	-0.351	0.297	-1.183	0.237	0.704	0.209	-1.417	0.156	0.393	1.259
TMEM212	0.466	0.3	1.552	0.121	1.594	0.479	1.24	0.215	0.885	2.871
TMEM209	0.171	0.296	0.577	0.564	1.186	0.351	0.531	0.596	0.664	2.117
TMEM208	0.603	0.306	1.971	0.049	1.827	0.559	1.48	0.139	1.003	3.326
TMEM204	-0.34	0.298	-1.142	0.254	0.712	0.212	-1.36	0.174	0.397	1.276
TMEM19	-0.182	0.297	-0.614	0.539	0.833	0.247	-0.674	0.5	0.466	1.491
TMEM187	0.001	0.298	0.004	0.997	1.001	0.298	0.004	0.997	0.558	1.795
TMEM186	0.065	0.295	0.22	0.826	1.067	0.315	0.213	0.831	0.599	1.903
TMEM185B	0.017	0.296	0.057	0.955	1.017	0.3	0.056	0.955	0.57	1.815
TMEM184C	0.111	0.295	0.375	0.708	1.117	0.33	0.355	0.723	0.626	1.992
TMEM184B	-0.06	0.295	-0.203	0.839	0.942	0.278	-0.21	0.834	0.528	1.68
TMEM177	0.656	0.303	2.166	0.03	1.926	0.583	1.588	0.112	1.064	3.487
TMEM176B	-0.677	0.306	-2.21	0.027	0.508	0.156	-3.16	0.002	0.279	0.926
TMEM176A	-0.095	0.296	-0.322	0.747	0.909	0.269	-0.338	0.735	0.509	1.623
TMEM168	-0.757	0.31	-2.44	0.015	0.469	0.146	-3.648	0	0.255	0.862
TMEM165	0.634	0.306	2.075	0.038	1.886	0.577	1.536	0.124	1.036	3.434
TMEM161A	0.561	0.306	1.833	0.067	1.752	0.536	1.403	0.161	0.962	3.192
TMEM160	0.45	0.299	1.505	0.132	1.568	0.468	1.212	0.225	0.873	2.816
TMEM159	0.622	0.306	2.034	0.042	1.863	0.57	1.515	0.13	1.023	3.391
TMEM158	0.227	0.298	0.765	0.445	1.255	0.374	0.684	0.494	0.701	2.249
TMEM156	-0.529	0.306	-1.729	0.084	0.589	0.18	-2.279	0.023	0.323	1.073
TMEM151B	0.057	0.295	0.194	0.846	1.059	0.313	0.189	0.85	0.593	1.89
TMEM14B	0.516	0.302	1.705	0.088	1.675	0.506	1.332	0.183	0.926	3.029
TMEM14A	0.848	0.312	2.72	0.007	2.334	0.727	1.835	0.067	1.267	4.298
TMEM147-AS1	0.703	0.306	2.299	0.022	2.02	0.618	1.651	0.099	1.109	3.678
TMEM147	0.392	0.298	1.316	0.188	1.481	0.441	1.089	0.276	0.825	2.656
TMEM144	-0.428	0.303	-1.416	0.157	0.652	0.197	-1.768	0.077	0.36	1.179
TMEM143	0.467	0.302	1.544	0.123	1.595	0.482	1.234	0.217	0.882	2.885
TMEM140	-0.373	0.298	-1.252	0.21	0.689	0.205	-1.518	0.129	0.384	1.234
TMEM135	-0.285	0.298	-0.959	0.338	0.752	0.224	-1.11	0.267	0.42	1.347
TMEM134	0.26	0.296	0.877	0.381	1.297	0.384	0.772	0.44	0.725	2.318
TMEM132A	0.54	0.306	1.768	0.077	1.716	0.524	1.366	0.172	0.943	3.123
TMEM131L	0.155	0.297	0.522	0.602	1.167	0.346	0.484	0.629	0.653	2.088
TMEM131	0.008	0.295	0.027	0.978	1.008	0.298	0.027	0.978	0.565	1.798
TMEM127	-0.022	0.296	-0.074	0.941	0.978	0.289	-0.075	0.94	0.548	1.747

TMEM126B	-0.168	0.296	-0.568	0.57	0.845	0.25	-0.619	0.536	0.473	1.509
TMEM123	-0.043	0.296	-0.144	0.886	0.958	0.284	-0.147	0.883	0.536	1.712
TMEM121	-0.163	0.298	-0.547	0.584	0.849	0.253	-0.595	0.552	0.473	1.524
TMEM120B	0.12	0.295	0.406	0.685	1.127	0.333	0.383	0.702	0.632	2.011
TMEM115	-0.652	0.31	-2.103	0.035	0.521	0.162	-2.965	0.003	0.284	0.957
TMEM11	0.939	0.321	2.928	0.003	2.557	0.82	1.899	0.058	1.364	4.794
TMEM109	-0.209	0.296	-0.705	0.481	0.812	0.241	-0.784	0.433	0.454	1.451
TMEM106C	0.429	0.3	1.431	0.152	1.536	0.46	1.164	0.245	0.853	2.763
TMEM106B	0.091	0.295	0.31	0.757	1.096	0.324	0.296	0.767	0.614	1.955
TMEM104	0.813	0.315	2.584	0.01	2.255	0.709	1.769	0.077	1.217	4.177
TMEM100	-0.201	0.296	-0.678	0.498	0.818	0.242	-0.751	0.453	0.458	1.462
TMED9	0.04	0.298	0.136	0.892	1.041	0.31	0.133	0.894	0.581	1.865
TMED5	-0.451	0.303	-1.492	0.136	0.637	0.193	-1.886	0.059	0.352	1.152
TMED3	-0.459	0.3	-1.531	0.126	0.632	0.189	-1.943	0.052	0.351	1.137
TMED2	-0.277	0.299	-0.925	0.355	0.758	0.227	-1.066	0.286	0.422	1.363
TMED10	-0.375	0.3	-1.25	0.211	0.688	0.206	-1.516	0.13	0.382	1.237
TMED1	0.555	0.302	1.838	0.066	1.741	0.525	1.411	0.158	0.964	3.146
TMCO6	0.402	0.298	1.351	0.177	1.495	0.445	1.112	0.266	0.834	2.679
TMCO3	-0.556	0.306	-1.818	0.069	0.574	0.175	-2.431	0.015	0.315	1.044
TMCO1	-0.182	0.296	-0.613	0.54	0.834	0.247	-0.672	0.501	0.466	1.491
TMCC2	0.08	0.296	0.272	0.786	1.084	0.32	0.261	0.794	0.607	1.934
TMCC1	0.512	0.302	1.692	0.091	1.668	0.504	1.325	0.185	0.922	3.017
TMC7	-0.119	0.296	-0.401	0.688	0.888	0.263	-0.426	0.67	0.497	1.587
TMC6	0.137	0.296	0.462	0.644	1.146	0.339	0.432	0.666	0.642	2.046
TMC5	-0.955	0.321	-2.977	0.003	0.385	0.123	-4.982	0	0.205	0.722
TMBIM6	-0.283	0.3	-0.945	0.345	0.753	0.226	-1.092	0.275	0.419	1.356
TMBIM4	-0.599	0.303	-1.977	0.048	0.549	0.166	-2.708	0.007	0.303	0.995
TMA7	0.64	0.306	2.091	0.037	1.896	0.58	1.544	0.123	1.041	3.455
TMA16	0.762	0.31	2.459	0.014	2.143	0.664	1.721	0.085	1.167	3.934
TM9SF4	-0.291	0.297	-0.98	0.327	0.747	0.222	-1.138	0.255	0.418	1.338
TM9SF3	0.125	0.295	0.425	0.671	1.134	0.335	0.399	0.69	0.636	2.022
TM9SF2	-0.682	0.31	-2.202	0.028	0.506	0.157	-3.157	0.002	0.276	0.928
TM9SF1	-0.224	0.297	-0.752	0.452	0.799	0.238	-0.843	0.399	0.446	1.432
TM7SF3	0.017	0.296	0.058	0.953	1.017	0.301	0.058	0.954	0.57	1.817
TM7SF2	0.026	0.295	0.089	0.929	1.027	0.303	0.088	0.93	0.576	1.83
TM6SF2	0.066	0.295	0.224	0.823	1.068	0.315	0.217	0.828	0.599	1.906
TM6SF1	-0.976	0.321	-3.04	0.002	0.377	0.121	-5.151	0	0.201	0.707
TM4SF5	0.085	0.295	0.287	0.774	1.088	0.322	0.275	0.783	0.61	1.942

TM4SF4	0.203	0.296	0.684	0.494	1.225	0.363	0.619	0.536	0.685	2.189
TM4SF20	-0.02	0.296	-0.069	0.945	0.98	0.29	-0.07	0.945	0.549	1.75
TM4SF1	0.119	0.295	0.402	0.688	1.126	0.333	0.379	0.705	0.631	2.009
TM2D3	-0.21	0.296	-0.708	0.479	0.811	0.24	-0.788	0.431	0.454	1.449
TM2D1	-0.187	0.296	-0.633	0.527	0.829	0.246	-0.696	0.487	0.464	1.482
TLX3	-0.008	0.295	-0.026	0.979	0.992	0.293	-0.026	0.979	0.556	1.77
TLX2	0.245	0.297	0.827	0.408	1.278	0.379	0.733	0.463	0.715	2.285
TLX1	0.239	0.296	0.806	0.42	1.269	0.376	0.717	0.473	0.711	2.267
TLR8	-0.249	0.301	-0.827	0.409	0.78	0.235	-0.938	0.348	0.432	1.406
TLR7	-0.567	0.306	-1.856	0.063	0.567	0.173	-2.498	0.012	0.312	1.032
TLR6	0.01	0.295	0.035	0.972	1.01	0.298	0.034	0.972	0.567	1.802
TLR5	-0.12	0.297	-0.403	0.687	0.887	0.264	-0.428	0.669	0.496	1.588
TLR4	-0.612	0.306	-2	0.045	0.542	0.166	-2.759	0.006	0.298	0.988
TLR3	-0.481	0.3	-1.606	0.108	0.618	0.185	-2.063	0.039	0.344	1.112
TLR2	-0.386	0.302	-1.277	0.202	0.68	0.206	-1.559	0.119	0.376	1.229
TLR1	-0.126	0.296	-0.424	0.671	0.882	0.261	-0.452	0.651	0.493	1.576
TLN2	-0.062	0.296	-0.21	0.834	0.94	0.278	-0.217	0.828	0.527	1.677
TLN1	-0.099	0.295	-0.335	0.737	0.906	0.267	-0.352	0.725	0.508	1.616
TLL2	-0.198	0.296	-0.668	0.504	0.821	0.243	-0.738	0.46	0.459	1.466
TLL1	0.466	0.301	1.547	0.122	1.593	0.48	1.236	0.216	0.883	2.875
TLK2	0.118	0.295	0.4	0.689	1.125	0.332	0.377	0.706	0.631	2.008
TLK1	-0.463	0.3	-1.546	0.122	0.629	0.189	-1.967	0.049	0.35	1.132
TLE6	0.137	0.295	0.464	0.643	1.147	0.339	0.433	0.665	0.643	2.045
TLE5	-0.139	0.297	-0.467	0.641	0.87	0.259	-0.5	0.617	0.486	1.559
TLE4	-0.081	0.295	-0.276	0.783	0.922	0.272	-0.287	0.774	0.517	1.644
TLE3	-0.495	0.3	-1.652	0.099	0.609	0.183	-2.138	0.032	0.338	1.097
TLE2	0.178	0.297	0.602	0.547	1.195	0.355	0.551	0.582	0.668	2.138
TLE1	0.481	0.306	1.57	0.116	1.617	0.495	1.247	0.212	0.888	2.946
TLCD3A	0.16	0.295	0.54	0.589	1.173	0.346	0.5	0.617	0.658	2.093
TKTL1	-0.01	0.296	-0.032	0.974	0.99	0.293	-0.032	0.974	0.555	1.768
TKT	-0.206	0.297	-0.693	0.488	0.814	0.242	-0.77	0.442	0.454	1.458
TKFC	-0.34	0.298	-1.14	0.254	0.712	0.212	-1.357	0.175	0.397	1.277
TK2	0.129	0.296	0.435	0.664	1.137	0.336	0.408	0.683	0.637	2.031
TK1	0.874	0.315	2.778	0.005	2.397	0.754	1.852	0.064	1.294	4.442
TJP3	0.139	0.296	0.471	0.638	1.15	0.34	0.439	0.66	0.643	2.054
TJP2	0.539	0.303	1.779	0.075	1.714	0.519	1.376	0.169	0.947	3.101
TJP1	-0.428	0.3	-1.427	0.153	0.652	0.195	-1.781	0.075	0.362	1.173
TJAP1	0.791	0.315	2.51	0.012	2.205	0.695	1.735	0.083	1.189	4.088

TIPRL	0.209	0.296	0.707	0.48	1.233	0.365	0.638	0.524	0.69	2.203
TIPIN	0.526	0.3	1.752	0.08	1.692	0.508	1.362	0.173	0.939	3.047
TIPARP	-0.13	0.295	-0.441	0.659	0.878	0.259	-0.471	0.638	0.492	1.566
TINF2	-0.375	0.302	-1.242	0.214	0.687	0.208	-1.507	0.132	0.38	1.243
TINAGL1	0.486	0.302	1.607	0.108	1.625	0.491	1.273	0.203	0.899	2.939
TIMP4	-0.014	0.295	-0.048	0.962	0.986	0.291	-0.048	0.961	0.553	1.759
TIMP3	-0.84	0.311	-2.697	0.007	0.432	0.134	-4.226	0	0.234	0.795
TIMP2	0.5	0.3	1.665	0.096	1.649	0.495	1.31	0.19	0.915	2.97
TIMP1	-0.514	0.303	-1.697	0.09	0.598	0.181	-2.218	0.027	0.331	1.083
TIMM9	0.564	0.306	1.847	0.065	1.759	0.537	1.412	0.158	0.966	3.201
TIMM8B	0.166	0.296	0.561	0.574	1.181	0.35	0.517	0.605	0.661	2.111
TIMM8A	0.903	0.321	2.816	0.005	2.467	0.791	1.854	0.064	1.316	4.625
TIMM50	-0.108	0.296	-0.363	0.716	0.898	0.266	-0.384	0.701	0.502	1.605
TIMM44	0.369	0.298	1.239	0.215	1.446	0.431	1.036	0.3	0.807	2.593
TIMM22	0.146	0.295	0.495	0.621	1.157	0.342	0.46	0.645	0.649	2.064
TIMM17B	0.395	0.299	1.322	0.186	1.484	0.443	1.092	0.275	0.826	2.665
TIMM17A	0.561	0.302	1.857	0.063	1.753	0.53	1.421	0.155	0.969	3.17
TIMM13	0.734	0.31	2.368	0.018	2.083	0.646	1.678	0.093	1.135	3.824
TIMM10B	-0.043	0.295	-0.145	0.884	0.958	0.283	-0.148	0.882	0.537	1.709
TIMM10	0.617	0.306	2.018	0.044	1.853	0.566	1.506	0.132	1.018	3.373
TIMELESS	0.541	0.303	1.787	0.074	1.718	0.52	1.38	0.168	0.949	3.11
TIGD6	0.457	0.301	1.521	0.128	1.58	0.475	1.221	0.222	0.877	2.847
TIGAR	0.716	0.307	2.335	0.02	2.046	0.627	1.667	0.095	1.122	3.731
TIE1	-0.585	0.303	-1.932	0.053	0.557	0.169	-2.625	0.009	0.308	1.009
TICAM1	-0.156	0.296	-0.528	0.598	0.855	0.253	-0.571	0.568	0.479	1.529
TIAM2	0.407	0.3	1.357	0.175	1.502	0.45	1.115	0.265	0.835	2.703
TIAM1	-0.559	0.303	-1.846	0.065	0.572	0.173	-2.473	0.013	0.316	1.035
TIAL1	0.733	0.306	2.391	0.017	2.081	0.638	1.695	0.09	1.141	3.794
TIA1	-0.102	0.296	-0.343	0.732	0.903	0.268	-0.361	0.718	0.505	1.615
THYN1	-0.339	0.3	-1.131	0.258	0.712	0.214	-1.347	0.178	0.396	1.282
THY1	-0.33	0.298	-1.11	0.267	0.719	0.214	-1.315	0.188	0.401	1.288
THUMPD2	0.168	0.296	0.567	0.571	1.183	0.351	0.522	0.602	0.662	2.114
THUMPD1	-0.025	0.296	-0.083	0.934	0.976	0.289	-0.084	0.933	0.546	1.742
THTPA	0.108	0.296	0.365	0.715	1.114	0.329	0.346	0.73	0.624	1.988
THSD7A	-0.089	0.296	-0.301	0.763	0.915	0.27	-0.315	0.753	0.513	1.633
THSD4	-0.165	0.298	-0.553	0.58	0.848	0.252	-0.601	0.548	0.473	1.52
THRB	-0.055	0.295	-0.187	0.851	0.946	0.279	-0.193	0.847	0.53	1.688
THRAP3	-0.183	0.296	-0.618	0.537	0.833	0.247	-0.678	0.498	0.466	1.488

THRA	0.238	0.297	0.803	0.422	1.269	0.376	0.714	0.475	0.709	2.269
THPO	-0.188	0.296	-0.633	0.527	0.829	0.246	-0.697	0.486	0.464	1.481
THOP1	-0.051	0.295	-0.174	0.862	0.95	0.281	-0.178	0.858	0.532	1.695
THOC7	0.111	0.296	0.373	0.709	1.117	0.331	0.354	0.724	0.625	1.995
THOC6	0.162	0.296	0.547	0.584	1.176	0.348	0.505	0.614	0.658	2.102
THOC5	-0.378	0.3	-1.261	0.207	0.685	0.205	-1.532	0.125	0.381	1.233
THOC2	0.232	0.296	0.784	0.433	1.261	0.374	0.699	0.484	0.706	2.254
THOC1	0.239	0.296	0.807	0.42	1.27	0.376	0.717	0.473	0.71	2.27
THNSL2	-0.328	0.3	-1.093	0.274	0.721	0.216	-1.294	0.196	0.4	1.297
THNSL1	0.042	0.295	0.141	0.888	1.043	0.308	0.138	0.89	0.585	1.859
THG1L	-0.519	0.306	-1.696	0.09	0.595	0.182	-2.223	0.026	0.327	1.084
THEMIS2	-0.119	0.296	-0.404	0.686	0.887	0.262	-0.429	0.668	0.497	1.584
THEM6	0.571	0.306	1.866	0.062	1.769	0.541	1.422	0.155	0.972	3.222
THEG	-0.36	0.298	-1.21	0.226	0.698	0.208	-1.456	0.145	0.389	1.25
THBS4	-0.352	0.297	-1.186	0.236	0.703	0.209	-1.422	0.155	0.393	1.258
THBS3	-0.072	0.295	-0.244	0.807	0.931	0.274	-0.253	0.8	0.522	1.659
THBS2	-0.331	0.298	-1.112	0.266	0.718	0.214	-1.319	0.187	0.401	1.287
THBS1	-0.396	0.302	-1.311	0.19	0.673	0.203	-1.609	0.108	0.372	1.217
THBD	0.04	0.297	0.134	0.893	1.041	0.309	0.131	0.895	0.581	1.863
THAP9-AS1	1.069	0.324	3.304	0.001	2.913	0.942	2.03	0.042	1.545	5.492
THAP9	-0.46	0.302	-1.523	0.128	0.631	0.191	-1.934	0.053	0.349	1.141
THAP7	0.754	0.307	2.454	0.014	2.126	0.654	1.723	0.085	1.164	3.885
THAP4	-0.427	0.302	-1.411	0.158	0.653	0.197	-1.76	0.078	0.361	1.181
THAP3	-0.194	0.302	-0.644	0.52	0.823	0.249	-0.711	0.477	0.456	1.488
THAP12	0.029	0.296	0.098	0.922	1.029	0.305	0.097	0.923	0.577	1.838
THAP11	0.247	0.296	0.832	0.405	1.28	0.379	0.738	0.461	0.716	2.287
THAP10	0.163	0.296	0.55	0.582	1.177	0.348	0.507	0.612	0.659	2.101
THAP1	-0.684	0.31	-2.207	0.027	0.505	0.156	-3.167	0.002	0.275	0.926
THADA	0.172	0.299	0.577	0.564	1.188	0.355	0.53	0.596	0.662	2.134
TH	0.26	0.298	0.872	0.383	1.296	0.386	0.768	0.442	0.723	2.324
TGS1	0.62	0.306	2.028	0.043	1.859	0.568	1.512	0.131	1.021	3.384
TGOLN2	-0.37	0.298	-1.243	0.214	0.691	0.206	-1.504	0.132	0.385	1.238
TGM5	0.093	0.295	0.313	0.754	1.097	0.324	0.299	0.765	0.615	1.957
TGM4	0.114	0.295	0.386	0.7	1.121	0.331	0.365	0.715	0.628	2
TGM3	-0.032	0.295	-0.108	0.914	0.969	0.286	-0.11	0.912	0.543	1.728
TGM2	-0.301	0.298	-1.011	0.312	0.74	0.221	-1.179	0.238	0.413	1.327
TGM1	0.139	0.296	0.471	0.638	1.149	0.34	0.439	0.66	0.644	2.051
TGIF2	0.477	0.3	1.588	0.112	1.611	0.484	1.263	0.207	0.894	2.901

TGIF1	0.046	0.296	0.156	0.876	1.047	0.309	0.152	0.879	0.587	1.869
TGFBRAP1	-0.153	0.296	-0.516	0.606	0.858	0.254	-0.558	0.577	0.48	1.533
TGFBR3	-0.045	0.295	-0.154	0.878	0.956	0.282	-0.157	0.875	0.536	1.704
TGFBR2	-0.862	0.315	-2.736	0.006	0.422	0.133	-4.341	0	0.228	0.783
TGFBR1	-0.462	0.3	-1.538	0.124	0.63	0.189	-1.955	0.051	0.35	1.135
TGFBI	-0.142	0.296	-0.481	0.631	0.867	0.257	-0.517	0.605	0.486	1.549
TGFB3	-0.425	0.3	-1.417	0.156	0.654	0.196	-1.766	0.077	0.364	1.177
TGFB2	-0.272	0.297	-0.916	0.36	0.762	0.226	-1.053	0.293	0.425	1.364
TGFB1I1	-0.144	0.295	-0.488	0.626	0.866	0.256	-0.525	0.6	0.485	1.545
TGFB1	-1.064	0.328	-3.243	0.001	0.345	0.113	-5.786	0	0.181	0.656
TGFA	0.625	0.306	2.046	0.041	1.869	0.571	1.521	0.128	1.027	3.402
TGDS	-0.22	0.296	-0.741	0.459	0.803	0.238	-0.829	0.407	0.449	1.435
TG	-0.528	0.301	-1.752	0.08	0.59	0.178	-2.308	0.021	0.327	1.065
TFRC	0.456	0.3	1.521	0.128	1.577	0.472	1.222	0.222	0.877	2.837
TFR2	0.105	0.296	0.354	0.724	1.11	0.329	0.336	0.737	0.621	1.984
TFPT	0.043	0.297	0.145	0.885	1.044	0.311	0.142	0.887	0.583	1.87
TFPI2	0.099	0.295	0.334	0.738	1.104	0.326	0.318	0.75	0.619	1.969
TFPI	-0.716	0.306	-2.338	0.019	0.489	0.15	-3.416	0.001	0.268	0.891
TFIP11	0.445	0.3	1.484	0.138	1.561	0.468	1.198	0.231	0.867	2.809
TFG	0.378	0.3	1.263	0.207	1.46	0.437	1.051	0.293	0.812	2.626
TFF3	-0.238	0.298	-0.798	0.425	0.789	0.235	-0.901	0.368	0.44	1.413
TFF2	0.575	0.304	1.89	0.059	1.777	0.54	1.438	0.151	0.979	3.225
TFF1	-0.201	0.296	-0.679	0.497	0.818	0.242	-0.752	0.452	0.458	1.461
TFEC	0.228	0.296	0.77	0.441	1.256	0.372	0.688	0.491	0.703	2.246
TFEB	0.29	0.298	0.974	0.33	1.337	0.398	0.846	0.398	0.746	2.397
TFE3	-0.282	0.296	-0.953	0.341	0.754	0.224	-1.101	0.271	0.422	1.348
TFDP3	0.451	0.301	1.498	0.134	1.569	0.472	1.206	0.228	0.87	2.829
TFDP2	0.302	0.298	1.014	0.311	1.352	0.402	0.875	0.382	0.755	2.423
TFDP1	0.392	0.298	1.315	0.189	1.48	0.442	1.088	0.277	0.825	2.656
TFCP2L1	0.637	0.306	2.083	0.037	1.89	0.578	1.541	0.123	1.038	3.441
TFCP2	0.173	0.296	0.583	0.56	1.188	0.352	0.536	0.592	0.666	2.122
TFB2M	0.529	0.303	1.749	0.08	1.697	0.514	1.358	0.175	0.938	3.072
TFB1M	0.037	0.295	0.125	0.9	1.038	0.306	0.123	0.902	0.582	1.85
TFAP4	0.237	0.298	0.793	0.428	1.267	0.378	0.707	0.48	0.706	2.274
TFAP2C	0.275	0.301	0.915	0.36	1.317	0.396	0.8	0.424	0.73	2.374
TFAP2B	-0.067	0.295	-0.227	0.82	0.935	0.276	-0.235	0.814	0.524	1.667
TFAP2A	0.142	0.296	0.481	0.631	1.153	0.341	0.448	0.654	0.646	2.058
TFAM	1.039	0.328	3.171	0.002	2.827	0.926	1.972	0.049	1.487	5.373

TF	-0.147	0.295	-0.496	0.62	0.864	0.255	-0.535	0.593	0.484	1.541
TEX30	0.243	0.296	0.819	0.413	1.275	0.377	0.727	0.467	0.713	2.277
TEX28	-0.298	0.298	-1	0.317	0.742	0.221	-1.165	0.244	0.414	1.331
TEX264	0.198	0.297	0.666	0.506	1.218	0.362	0.604	0.546	0.681	2.18
TEX261	-0.17	0.297	-0.573	0.567	0.844	0.25	-0.624	0.533	0.472	1.509
TEX2	-0.459	0.299	-1.533	0.125	0.632	0.189	-1.946	0.052	0.351	1.136
TEX15	-0.136	0.296	-0.459	0.647	0.873	0.259	-0.491	0.623	0.489	1.56
TEX14	-0.453	0.306	-1.48	0.139	0.636	0.195	-1.872	0.061	0.349	1.158
TEX13B	-0.167	0.296	-0.565	0.572	0.846	0.251	-0.615	0.539	0.473	1.512
TEX13A	0.638	0.306	2.086	0.037	1.893	0.579	1.542	0.123	1.039	3.449
TEX12	-0.456	0.302	-1.508	0.132	0.634	0.192	-1.91	0.056	0.351	1.146
TEX11	0.059	0.295	0.2	0.841	1.061	0.313	0.194	0.846	0.595	1.892
TEX10	0.085	0.3	0.282	0.778	1.089	0.327	0.271	0.787	0.604	1.961
TET3	0.027	0.295	0.093	0.926	1.028	0.303	0.092	0.927	0.576	1.832
TESPA1	-0.372	0.3	-1.241	0.214	0.689	0.207	-1.504	0.133	0.383	1.24
TESMIN	-0.205	0.296	-0.691	0.489	0.815	0.241	-0.767	0.443	0.456	1.456
TESK2	0.028	0.295	0.096	0.924	1.029	0.304	0.094	0.925	0.577	1.835
TESK1	0.484	0.302	1.6	0.11	1.622	0.49	1.269	0.205	0.897	2.933
TESC	-0.165	0.296	-0.556	0.578	0.848	0.251	-0.605	0.545	0.475	1.515
TES	-0.662	0.304	-2.183	0.029	0.516	0.156	-3.096	0.002	0.284	0.935
TERT	0.418	0.299	1.399	0.162	1.519	0.454	1.143	0.253	0.846	2.729
TERF2IP	0.187	0.296	0.634	0.526	1.206	0.356	0.578	0.563	0.676	2.153
TERF2	0.233	0.296	0.787	0.431	1.262	0.374	0.702	0.483	0.707	2.256
TERF1	0.51	0.302	1.689	0.091	1.666	0.503	1.323	0.186	0.921	3.012
TEP1	0.163	0.296	0.549	0.583	1.177	0.348	0.507	0.612	0.659	2.102
TENT5C	-0.142	0.295	-0.479	0.632	0.868	0.256	-0.515	0.607	0.486	1.549
TENT5A	-0.227	0.296	-0.768	0.442	0.797	0.236	-0.862	0.389	0.446	1.422
TENT4A	0.931	0.321	2.901	0.004	2.536	0.813	1.888	0.059	1.352	4.755
TENM4	-0.472	0.302	-1.56	0.119	0.624	0.189	-1.993	0.046	0.345	1.128
TENM3	-0.381	0.3	-1.27	0.204	0.683	0.205	-1.545	0.122	0.38	1.23
TENM1	0.521	0.301	1.73	0.084	1.684	0.507	1.349	0.177	0.933	3.037
TELO2	0.447	0.302	1.48	0.139	1.564	0.473	1.194	0.233	0.865	2.828
TEKT2	0.528	0.301	1.756	0.079	1.696	0.51	1.364	0.172	0.941	3.058
TEK	-0.107	0.295	-0.361	0.718	0.899	0.265	-0.381	0.703	0.504	1.603
TEFM	0.146	0.296	0.494	0.622	1.157	0.343	0.459	0.646	0.648	2.068
TEF	0.222	0.296	0.749	0.454	1.248	0.37	0.671	0.502	0.698	2.232
TEDC2	0.253	0.298	0.849	0.396	1.287	0.383	0.75	0.453	0.719	2.306
TECTA	-0.414	0.3	-1.381	0.167	0.661	0.198	-1.71	0.087	0.367	1.19

TECR	-0.292	0.3	-0.974	0.33	0.747	0.224	-1.131	0.258	0.415	1.344
TECPR2	-0.079	0.296	-0.267	0.789	0.924	0.273	-0.278	0.781	0.518	1.649
TEC	0.412	0.298	1.385	0.166	1.51	0.449	1.135	0.256	0.843	2.706
TEAD4	0.498	0.302	1.649	0.099	1.646	0.497	1.298	0.194	0.91	2.976
TEAD3	0.617	0.31	1.992	0.046	1.854	0.574	1.487	0.137	1.01	3.403
TEAD1	0.585	0.301	1.942	0.052	1.795	0.541	1.47	0.142	0.994	3.239
TDRKH	-0.015	0.295	-0.05	0.96	0.985	0.291	-0.051	0.96	0.553	1.757
TDRD7	0.088	0.296	0.298	0.765	1.092	0.323	0.286	0.775	0.612	1.95
TDRD3	-0.307	0.298	-1.03	0.303	0.736	0.219	-1.205	0.228	0.411	1.319
TDRD12	0.429	0.299	1.431	0.152	1.535	0.46	1.164	0.245	0.853	2.761
TDRD1	0.436	0.3	1.456	0.145	1.547	0.464	1.18	0.238	0.86	2.783
TDP2	0.079	0.295	0.266	0.79	1.082	0.319	0.256	0.798	0.606	1.93
TDP1	0.311	0.297	1.049	0.294	1.365	0.405	0.902	0.367	0.763	2.442
TDO2	0.566	0.302	1.871	0.061	1.761	0.533	1.429	0.153	0.974	3.186
TDG	0.1	0.295	0.34	0.734	1.106	0.327	0.324	0.746	0.62	1.973
TCTN3	-0.636	0.31	-2.052	0.04	0.529	0.164	-2.868	0.004	0.288	0.972
TCTN2	0.106	0.295	0.358	0.72	1.111	0.328	0.34	0.734	0.623	1.982
TCTN1	-0.649	0.31	-2.096	0.036	0.522	0.162	-2.951	0.003	0.285	0.959
TCTA	-0.168	0.298	-0.565	0.572	0.845	0.252	-0.616	0.538	0.472	1.515
TCP11L1	-0.6	0.304	-1.977	0.048	0.549	0.167	-2.71	0.007	0.302	0.995
TCP11	0.083	0.297	0.279	0.78	1.087	0.323	0.268	0.789	0.607	1.945
TCP10L3	0.057	0.295	0.193	0.847	1.059	0.312	0.188	0.851	0.594	1.888
TCP10L	0.347	0.297	1.168	0.243	1.415	0.42	0.987	0.324	0.79	2.532
TCOF1	0.653	0.31	2.103	0.035	1.921	0.596	1.545	0.122	1.045	3.529
TCN2	0.576	0.306	1.885	0.059	1.779	0.544	1.433	0.152	0.977	3.239
TCN1	-0.013	0.295	-0.045	0.964	0.987	0.291	-0.045	0.964	0.553	1.76
TCL6	0.191	0.299	0.64	0.522	1.211	0.362	0.583	0.56	0.674	2.175
TCL1B	-0.528	0.301	-1.756	0.079	0.59	0.177	-2.314	0.021	0.327	1.063
TCL1A	-0.721	0.306	-2.356	0.018	0.486	0.149	-3.453	0.001	0.267	0.886
TCIRG1	-0.107	0.297	-0.362	0.718	0.898	0.266	-0.382	0.703	0.502	1.606
TCIM	-0.518	0.306	-1.69	0.091	0.596	0.183	-2.214	0.027	0.327	1.086
TCHH	-0.116	0.295	-0.394	0.694	0.89	0.263	-0.418	0.676	0.499	1.588
TCFL5	0.314	0.298	1.055	0.291	1.369	0.407	0.906	0.365	0.764	2.453
TCF7L2	0.28	0.3	0.934	0.35	1.323	0.396	0.815	0.415	0.735	2.38
TCF7L1	0.398	0.303	1.316	0.188	1.489	0.451	1.085	0.278	0.823	2.696
TCF7	-0.192	0.297	-0.645	0.519	0.825	0.245	-0.711	0.477	0.461	1.479
TCF4	-0.558	0.3	-1.859	0.063	0.572	0.172	-2.49	0.013	0.318	1.031
TCF3	0.231	0.297	0.777	0.437	1.26	0.375	0.694	0.488	0.703	2.258

TCF25	-0.123	0.295	-0.416	0.677	0.884	0.261	-0.443	0.658	0.496	1.578
TCF21	0.654	0.303	2.155	0.031	1.923	0.583	1.582	0.114	1.061	3.485
TCF20	0.413	0.307	1.345	0.179	1.511	0.464	1.102	0.271	0.828	2.759
TCF15	-0.019	0.296	-0.066	0.948	0.981	0.29	-0.066	0.947	0.549	1.751
TCF12	-0.549	0.304	-1.809	0.071	0.577	0.175	-2.41	0.016	0.318	1.047
TCERG1	0.598	0.306	1.958	0.05	1.819	0.556	1.473	0.141	0.999	3.311
TCEAL9	0.63	0.31	2.032	0.042	1.878	0.582	1.508	0.132	1.023	3.448
TCEAL4	0.143	0.296	0.482	0.63	1.153	0.341	0.449	0.653	0.646	2.059
TCEAL2	0.574	0.306	1.879	0.06	1.776	0.543	1.429	0.153	0.976	3.232
TCEAL1	0.131	0.296	0.442	0.659	1.14	0.337	0.414	0.679	0.638	2.036
TCEA2	-0.323	0.304	-1.063	0.288	0.724	0.22	-1.255	0.209	0.399	1.313
TCEA1	0.201	0.296	0.678	0.498	1.222	0.361	0.615	0.539	0.685	2.181
TCAP	0.736	0.31	2.376	0.017	2.088	0.647	1.682	0.093	1.138	3.834
TCAF1	0.009	0.295	0.031	0.975	1.009	0.298	0.031	0.975	0.566	1.8
TBXT	0.101	0.296	0.34	0.734	1.106	0.327	0.323	0.746	0.619	1.974
TBXAS1	-0.344	0.303	-1.136	0.256	0.709	0.215	-1.356	0.175	0.392	1.283
TBXA2R	-0.546	0.303	-1.802	0.072	0.579	0.176	-2.396	0.017	0.32	1.049
TBX6	-0.158	0.296	-0.535	0.593	0.854	0.252	-0.58	0.562	0.478	1.524
TBX5	0.152	0.295	0.514	0.608	1.164	0.344	0.477	0.634	0.652	2.076
TBX4	0.937	0.321	2.92	0.003	2.551	0.818	1.896	0.058	1.361	4.783
TBX3	-0.483	0.3	-1.608	0.108	0.617	0.185	-2.067	0.039	0.343	1.111
TBX21	0.165	0.296	0.558	0.577	1.18	0.349	0.514	0.607	0.66	2.108
TBX2	-0.097	0.297	-0.326	0.745	0.908	0.269	-0.342	0.732	0.508	1.623
TBX19	0.332	0.3	1.109	0.267	1.394	0.418	0.944	0.345	0.775	2.509
TBX10	0.276	0.296	0.931	0.352	1.317	0.39	0.814	0.416	0.737	2.354
TBX1	0.344	0.298	1.155	0.248	1.411	0.421	0.978	0.328	0.787	2.531
TBRG4	0.281	0.296	0.948	0.343	1.324	0.393	0.826	0.409	0.741	2.368
TBR1	0.056	0.295	0.189	0.85	1.057	0.312	0.184	0.854	0.593	1.885
TBPL1	0.441	0.302	1.459	0.145	1.554	0.47	1.18	0.238	0.86	2.81
TBP	0.047	0.296	0.158	0.874	1.048	0.31	0.155	0.877	0.587	1.87
TBL3	0.283	0.298	0.949	0.342	1.327	0.395	0.827	0.408	0.74	2.377
TBL2	0.23	0.297	0.775	0.439	1.259	0.374	0.692	0.489	0.703	2.255
TBL1Y	0.645	0.306	2.11	0.035	1.906	0.583	1.555	0.12	1.047	3.47
TBL1XR1	-0.687	0.31	-2.217	0.027	0.503	0.156	-3.187	0.001	0.274	0.923
TBL1X	-0.318	0.298	-1.068	0.285	0.728	0.217	-1.258	0.209	0.406	1.304
TBKBP1	-0.174	0.296	-0.589	0.556	0.84	0.248	-0.643	0.52	0.471	1.5
TBK1	-0.805	0.31	-2.594	0.009	0.447	0.139	-3.986	0	0.243	0.821
TBCEL	0.169	0.296	0.571	0.568	1.184	0.35	0.525	0.6	0.663	2.113

TBCE	0.56	0.302	1.85	0.064	1.75	0.529	1.417	0.157	0.967	3.166
TBCD	0.552	0.303	1.823	0.068	1.737	0.526	1.401	0.161	0.959	3.144
TBCCD1	0.573	0.302	1.896	0.058	1.774	0.536	1.443	0.149	0.981	3.208
TBCC	0.237	0.297	0.799	0.424	1.268	0.376	0.712	0.477	0.709	2.267
TBCB	-0.097	0.299	-0.323	0.747	0.908	0.272	-0.339	0.735	0.505	1.632
TBCA	0.609	0.302	2.018	0.044	1.839	0.555	1.511	0.131	1.018	3.323
TBC1D9B	-0.353	0.298	-1.186	0.236	0.702	0.209	-1.422	0.155	0.392	1.259
TBC1D9	-0.757	0.315	-2.405	0.016	0.469	0.148	-3.595	0	0.253	0.869
TBC1D8B	-0.439	0.3	-1.463	0.143	0.645	0.193	-1.837	0.066	0.358	1.161
TBC1D8	-0.719	0.31	-2.32	0.02	0.487	0.151	-3.396	0.001	0.265	0.894
TBC1D5	-0.302	0.298	-1.016	0.31	0.739	0.22	-1.187	0.235	0.412	1.324
TBC1D4	-0.557	0.306	-1.822	0.068	0.573	0.175	-2.439	0.015	0.315	1.043
TBC1D31	0.624	0.303	2.057	0.04	1.866	0.566	1.53	0.126	1.03	3.382
TBC1D30	-0.093	0.296	-0.314	0.753	0.911	0.27	-0.329	0.742	0.51	1.628
TBC1D2B	-0.512	0.3	-1.705	0.088	0.599	0.18	-2.227	0.026	0.333	1.08
TBC1D29P	-0.454	0.303	-1.502	0.133	0.635	0.192	-1.901	0.057	0.351	1.149
TBC1D22B	0.087	0.296	0.295	0.768	1.091	0.323	0.283	0.777	0.611	1.948
TBC1D22A	0.164	0.296	0.553	0.58	1.178	0.348	0.511	0.61	0.66	2.103
TBC1D2	-0.106	0.296	-0.359	0.72	0.899	0.267	-0.379	0.705	0.503	1.607
TBC1D19	0.008	0.296	0.027	0.978	1.008	0.298	0.027	0.978	0.565	1.8
TBC1D17	-0.74	0.315	-2.352	0.019	0.477	0.15	-3.484	0	0.257	0.884
TBC1D16	0.142	0.296	0.48	0.631	1.153	0.341	0.447	0.655	0.645	2.059
TBC1D15	-0.282	0.3	-0.939	0.348	0.755	0.226	-1.085	0.278	0.419	1.358
TBC1D13	0.385	0.3	1.284	0.199	1.469	0.44	1.066	0.287	0.817	2.643
TBC1D12	-0.58	0.303	-1.914	0.056	0.56	0.17	-2.593	0.01	0.309	1.014
TBC1D10B	-0.316	0.299	-1.06	0.289	0.729	0.218	-1.247	0.212	0.406	1.308
TBC1D1	-0.531	0.302	-1.757	0.079	0.588	0.178	-2.319	0.02	0.325	1.063
TAZ	0.11	0.296	0.373	0.709	1.117	0.331	0.353	0.724	0.625	1.996
TAX1BP1	-0.417	0.3	-1.392	0.164	0.659	0.198	-1.727	0.084	0.366	1.186
TATDN2	0.199	0.299	0.667	0.505	1.22	0.364	0.605	0.546	0.68	2.191
TAT	-0.318	0.298	-1.07	0.285	0.727	0.216	-1.26	0.208	0.406	1.303
TASP1	0.187	0.296	0.633	0.527	1.206	0.357	0.577	0.564	0.675	2.155
TASOR2	0.173	0.295	0.585	0.559	1.189	0.351	0.537	0.591	0.666	2.12
TASOR	-0.271	0.298	-0.909	0.363	0.763	0.227	-1.044	0.296	0.425	1.368
TASL	0.668	0.306	2.178	0.029	1.95	0.598	1.589	0.112	1.069	3.555
TAS2R9	0.017	0.295	0.059	0.953	1.018	0.3	0.058	0.953	0.571	1.814
TAS2R8	0.425	0.299	1.424	0.154	1.53	0.457	1.16	0.246	0.852	2.748
TAS2R7	0.028	0.295	0.095	0.924	1.028	0.304	0.094	0.925	0.577	1.834

TAS2R4	0.013	0.295	0.044	0.965	1.013	0.299	0.044	0.965	0.568	1.806
TAS2R3	0.17	0.296	0.575	0.565	1.186	0.351	0.529	0.597	0.664	2.118
TAS2R16	0.082	0.295	0.278	0.781	1.086	0.321	0.267	0.79	0.608	1.937
TAS2R14	0.587	0.302	1.942	0.052	1.799	0.544	1.469	0.142	0.995	3.253
TAS2R13	-0.218	0.297	-0.733	0.464	0.804	0.239	-0.819	0.413	0.449	1.44
TAS2R10	-0.486	0.302	-1.609	0.108	0.615	0.186	-2.072	0.038	0.34	1.112
TAS2R1	0.345	0.298	1.156	0.248	1.411	0.421	0.978	0.328	0.787	2.531
TARS1	0.246	0.296	0.832	0.405	1.279	0.378	0.738	0.461	0.716	2.284
TARP	0.148	0.295	0.502	0.616	1.16	0.342	0.466	0.641	0.65	2.068
TARDBP	-0.071	0.296	-0.24	0.811	0.932	0.275	-0.248	0.804	0.522	1.663
TARBP2	0.177	0.297	0.596	0.551	1.193	0.354	0.546	0.585	0.667	2.135
TARBP1	0.218	0.296	0.736	0.462	1.244	0.369	0.661	0.508	0.696	2.223
TAPT1	0.133	0.296	0.452	0.652	1.143	0.338	0.423	0.673	0.64	2.04
TAPBPL	0.536	0.304	1.765	0.078	1.709	0.519	1.366	0.172	0.942	3.098
TAPBP	-0.208	0.299	-0.694	0.487	0.813	0.243	-0.772	0.44	0.452	1.46
TAP2	0.199	0.296	0.673	0.501	1.22	0.362	0.61	0.542	0.683	2.181
TAP1	0.131	0.296	0.442	0.658	1.14	0.337	0.414	0.679	0.638	2.034
TAOK3	0.038	0.295	0.13	0.896	1.039	0.307	0.128	0.898	0.583	1.854
TAOK2	-0.065	0.295	-0.222	0.825	0.937	0.277	-0.229	0.819	0.525	1.671
TAOK1	0.321	0.298	1.08	0.28	1.379	0.411	0.923	0.356	0.769	2.472
TANK	-0.38	0.3	-1.269	0.204	0.684	0.205	-1.544	0.123	0.38	1.23
TANC2	0.518	0.303	1.712	0.087	1.679	0.508	1.336	0.181	0.928	3.038
TALDO1	0.67	0.303	2.213	0.027	1.955	0.592	1.613	0.107	1.08	3.54
TAL1	-0.27	0.299	-0.906	0.365	0.763	0.228	-1.04	0.298	0.425	1.37
TAGLN3	0.079	0.297	0.265	0.791	1.082	0.321	0.255	0.799	0.605	1.936
TAGLN2	-0.004	0.296	-0.014	0.988	0.996	0.294	-0.015	0.988	0.558	1.777
TAGLN	-0.651	0.306	-2.131	0.033	0.521	0.159	-3.003	0.003	0.286	0.949
TAF9B	-0.529	0.306	-1.731	0.083	0.589	0.18	-2.282	0.022	0.324	1.072
TAF7L	0.055	0.295	0.188	0.851	1.057	0.312	0.183	0.855	0.593	1.884
TAF7	-0.204	0.296	-0.689	0.491	0.815	0.242	-0.764	0.445	0.456	1.457
TAF6L	0.182	0.296	0.614	0.539	1.2	0.356	0.561	0.575	0.671	2.145
TAF6	0.017	0.295	0.058	0.954	1.017	0.3	0.058	0.954	0.57	1.814
TAF5L	0.016	0.295	0.054	0.957	1.016	0.3	0.054	0.957	0.57	1.813
TAF5	0.426	0.303	1.408	0.159	1.531	0.463	1.147	0.252	0.846	2.771
TAF4B	-0.253	0.297	-0.852	0.394	0.777	0.23	-0.969	0.333	0.434	1.389
TAF4	0.434	0.303	1.432	0.152	1.543	0.468	1.162	0.245	0.852	2.796
TAF2	0.933	0.321	2.911	0.004	2.543	0.815	1.893	0.058	1.356	4.766
TAF1D	0.093	0.301	0.308	0.758	1.097	0.33	0.294	0.769	0.608	1.979

TAF1C	0.455	0.3	1.516	0.129	1.576	0.473	1.218	0.223	0.875	2.839
TAF1B	0.057	0.295	0.193	0.847	1.059	0.313	0.187	0.851	0.594	1.888
TAF1A	0.003	0.295	0.009	0.993	1.003	0.296	0.009	0.993	0.562	1.788
TAF15	0.333	0.298	1.119	0.263	1.395	0.416	0.951	0.341	0.778	2.502
TAF13	-0.119	0.295	-0.404	0.686	0.887	0.262	-0.429	0.668	0.497	1.584
TAF12	-0.516	0.306	-1.69	0.091	0.597	0.182	-2.212	0.027	0.328	1.086
TAF11	1.159	0.336	3.449	0.001	3.185	1.07	2.042	0.041	1.649	6.154
TAF10	-0.294	0.298	-0.989	0.323	0.745	0.222	-1.149	0.25	0.416	1.335
TAF1	-0.396	0.3	-1.317	0.188	0.673	0.202	-1.616	0.106	0.374	1.213
TADA3	-0.569	0.307	-1.856	0.064	0.566	0.174	-2.5	0.012	0.31	1.033
TADA2A	0.572	0.306	1.869	0.062	1.771	0.542	1.424	0.154	0.973	3.225
TACSTD2	-0.197	0.299	-0.66	0.509	0.821	0.245	-0.729	0.466	0.457	1.475
TACR3	0.096	0.296	0.323	0.746	1.1	0.325	0.308	0.758	0.616	1.964
TACR2	0.144	0.295	0.488	0.626	1.155	0.341	0.454	0.65	0.647	2.061
TACR1	0.578	0.303	1.905	0.057	1.782	0.54	1.447	0.148	0.984	3.228
TACO1	0.689	0.306	2.249	0.025	1.992	0.61	1.625	0.104	1.093	3.63
TACC3	0.638	0.303	2.109	0.035	1.893	0.573	1.559	0.119	1.046	3.425
TACC2	0.216	0.296	0.729	0.466	1.241	0.367	0.656	0.512	0.694	2.217
TACC1	-0.869	0.315	-2.758	0.006	0.42	0.132	-4.393	0	0.226	0.778
TAC3	-0.202	0.296	-0.683	0.495	0.817	0.242	-0.757	0.449	0.457	1.46
TAC1	-0.462	0.302	-1.528	0.127	0.63	0.19	-1.942	0.052	0.348	1.14
TAB2	0.362	0.3	1.208	0.227	1.436	0.431	1.013	0.311	0.798	2.585
TAB1	0.939	0.321	2.93	0.003	2.559	0.82	1.9	0.057	1.365	4.796
TAAR5	0.286	0.297	0.966	0.334	1.332	0.395	0.84	0.401	0.745	2.381
TAAR3P	-0.11	0.296	-0.37	0.711	0.896	0.265	-0.391	0.695	0.502	1.6
TAAR2	-0.019	0.296	-0.066	0.947	0.981	0.29	-0.067	0.947	0.55	1.75
SZT2	-0.506	0.301	-1.68	0.093	0.603	0.182	-2.186	0.029	0.334	1.088
SZRD1	0.355	0.3	1.186	0.236	1.427	0.427	0.998	0.318	0.793	2.566
SYTL2	-0.014	0.295	-0.046	0.963	0.987	0.291	-0.046	0.963	0.553	1.76
SYT5	0.274	0.297	0.924	0.356	1.315	0.39	0.808	0.419	0.735	2.351
SYT2	0.104	0.295	0.351	0.725	1.109	0.328	0.334	0.739	0.622	1.979
SYT17	-0.282	0.298	-0.948	0.343	0.754	0.225	-1.095	0.273	0.421	1.352
SYT13	0.069	0.296	0.232	0.817	1.071	0.317	0.224	0.823	0.6	1.911
SYT12	0.258	0.296	0.87	0.384	1.294	0.383	0.767	0.443	0.724	2.312
SYT11	-0.719	0.31	-2.322	0.02	0.487	0.151	-3.398	0.001	0.266	0.894
SYT1	-0.23	0.298	-0.774	0.439	0.794	0.236	-0.87	0.384	0.443	1.423
SYPL1	-0.494	0.306	-1.616	0.106	0.61	0.187	-2.09	0.037	0.335	1.111
SYP	-0.254	0.298	-0.853	0.394	0.776	0.231	-0.971	0.332	0.432	1.391

SYNRG	-0.182	0.296	-0.614	0.539	0.834	0.247	-0.674	0.501	0.467	1.49
SYNPO2L	-0.125	0.296	-0.422	0.673	0.883	0.262	-0.449	0.653	0.494	1.578
SYNPO	-0.507	0.302	-1.676	0.094	0.602	0.182	-2.183	0.029	0.333	1.09
SYNM	0.069	0.295	0.235	0.814	1.072	0.317	0.227	0.821	0.601	1.912
SYNJ2	0.208	0.296	0.703	0.482	1.231	0.364	0.635	0.526	0.69	2.197
SYNJ1	0.169	0.295	0.572	0.567	1.184	0.35	0.526	0.599	0.664	2.112
SYNGR4	-0.084	0.295	-0.284	0.776	0.919	0.271	-0.297	0.767	0.515	1.64
SYNGR3	0.092	0.296	0.312	0.755	1.097	0.325	0.298	0.766	0.614	1.959
SYNGR2	-0.024	0.296	-0.082	0.934	0.976	0.289	-0.083	0.933	0.546	1.743
SYNGR1	-0.208	0.296	-0.703	0.482	0.812	0.24	-0.782	0.434	0.454	1.451
SYNE2	-0.116	0.296	-0.392	0.695	0.89	0.264	-0.416	0.677	0.498	1.591
SYNE1	-1.084	0.322	-3.364	0.001	0.338	0.109	-6.07	0	0.18	0.636
SYNDIG1	-0.73	0.31	-2.355	0.019	0.482	0.149	-3.468	0.001	0.263	0.885
SYNCRIP	0.118	0.296	0.398	0.691	1.125	0.333	0.375	0.707	0.629	2.011
SYNC	-0.329	0.3	-1.096	0.273	0.72	0.216	-1.298	0.194	0.4	1.296
SYN3	0.528	0.301	1.753	0.08	1.695	0.51	1.362	0.173	0.939	3.057
SYN2	0.08	0.297	0.271	0.787	1.084	0.322	0.26	0.795	0.606	1.939
SYN1	0.063	0.295	0.214	0.83	1.065	0.314	0.208	0.836	0.597	1.899
SYMPK	0.086	0.295	0.292	0.77	1.09	0.322	0.28	0.78	0.611	1.945
SYK	0.125	0.297	0.421	0.674	1.133	0.336	0.396	0.692	0.633	2.027
SYF2	0.31	0.297	1.042	0.297	1.363	0.405	0.896	0.37	0.761	2.442
SYDE1	-0.342	0.297	-1.153	0.249	0.71	0.211	-1.375	0.169	0.397	1.27
SYCP2	-0.018	0.295	-0.061	0.952	0.982	0.29	-0.061	0.951	0.551	1.751
SYCP1	-0.238	0.297	-0.804	0.422	0.788	0.234	-0.908	0.364	0.44	1.409
SYCE1L	-0.262	0.296	-0.884	0.377	0.77	0.228	-1.01	0.312	0.431	1.376
SYBU	-0.158	0.296	-0.534	0.593	0.854	0.253	-0.578	0.563	0.478	1.525
SWAP70	0.018	0.296	0.06	0.952	1.018	0.301	0.06	0.952	0.57	1.818
SVIL	-0.327	0.297	-1.103	0.27	0.721	0.214	-1.304	0.192	0.403	1.29
SVEP1	-0.737	0.31	-2.38	0.017	0.478	0.148	-3.519	0	0.261	0.878
SV2C	-0.192	0.296	-0.648	0.517	0.825	0.244	-0.714	0.475	0.462	1.475
SV2B	0.289	0.298	0.969	0.333	1.335	0.398	0.842	0.4	0.744	2.395
SV2A	-0.11	0.295	-0.373	0.709	0.896	0.265	-0.395	0.693	0.502	1.598
SUZ12P1	0.534	0.303	1.765	0.078	1.706	0.516	1.367	0.172	0.943	3.088
SUZ12	0.529	0.3	1.76	0.078	1.696	0.509	1.367	0.172	0.942	3.056
SUV39H2	0.599	0.304	1.974	0.048	1.821	0.553	1.484	0.138	1.004	3.303
SUV39H1	0.281	0.298	0.942	0.346	1.324	0.395	0.821	0.412	0.738	2.375
SUSD6	-0.783	0.31	-2.525	0.012	0.457	0.142	-3.831	0	0.249	0.839
SUSD5	-0.369	0.3	-1.232	0.218	0.691	0.207	-1.49	0.136	0.384	1.244

SUSD4	0.149	0.296	0.504	0.614	1.161	0.344	0.468	0.64	0.649	2.076
SURF2	0.621	0.303	2.048	0.041	1.86	0.564	1.526	0.127	1.027	3.368
SURF1	-0.307	0.301	-1.02	0.308	0.736	0.221	-1.194	0.232	0.408	1.326
SUPV3L1	0.034	0.295	0.116	0.907	1.035	0.306	0.114	0.909	0.58	1.847
SUPT7L	0.518	0.301	1.723	0.085	1.679	0.505	1.345	0.179	0.931	3.027
SUPT6H	-0.249	0.298	-0.836	0.403	0.78	0.232	-0.949	0.343	0.435	1.398
SUPT5H	0.091	0.295	0.309	0.757	1.096	0.323	0.295	0.768	0.614	1.954
SUPT4H1	0.217	0.296	0.732	0.464	1.242	0.368	0.658	0.511	0.695	2.22
SUPT3H	-0.066	0.297	-0.224	0.823	0.936	0.278	-0.231	0.817	0.523	1.674
SUPT20H	-0.177	0.296	-0.598	0.55	0.838	0.248	-0.654	0.513	0.469	1.497
SUPT16H	0.021	0.296	0.07	0.944	1.021	0.303	0.07	0.944	0.571	1.825
SUOX	-0.218	0.298	-0.734	0.463	0.804	0.239	-0.82	0.412	0.449	1.44
SUN2	0.322	0.3	1.074	0.283	1.38	0.413	0.918	0.358	0.767	2.482
SUN1	0.475	0.302	1.571	0.116	1.608	0.486	1.25	0.211	0.889	2.907
SUMO4	0.503	0.302	1.663	0.096	1.654	0.5	1.307	0.191	0.914	2.991
SUMO3	0.128	0.295	0.432	0.666	1.136	0.336	0.405	0.685	0.637	2.027
SUMO1	0.616	0.31	1.986	0.047	1.852	0.574	1.483	0.138	1.008	3.401
SULT4A1	0.375	0.298	1.259	0.208	1.455	0.433	1.05	0.294	0.812	2.608
SULT2B1	-0.026	0.296	-0.089	0.929	0.974	0.288	-0.09	0.928	0.546	1.739
SULT2A1	-0.064	0.295	-0.215	0.829	0.938	0.277	-0.222	0.824	0.526	1.674
SULT1E1	0.617	0.306	2.019	0.043	1.853	0.566	1.507	0.132	1.018	3.373
SULT1C2	0.603	0.306	1.972	0.049	1.828	0.559	1.48	0.139	1.004	3.329
SULT1B1	-0.137	0.296	-0.461	0.645	0.872	0.259	-0.494	0.621	0.488	1.559
SULT1A2	0.011	0.295	0.036	0.972	1.011	0.298	0.035	0.972	0.566	1.803
SULT1A1	0.099	0.295	0.334	0.738	1.104	0.326	0.318	0.75	0.619	1.969
SULF1	-0.503	0.302	-1.664	0.096	0.605	0.183	-2.163	0.031	0.334	1.093
SUGP2	0.193	0.296	0.65	0.516	1.212	0.359	0.591	0.554	0.678	2.166
SUGP1	0.029	0.296	0.098	0.922	1.029	0.304	0.097	0.923	0.577	1.837
SUGCT	-0.112	0.296	-0.379	0.705	0.894	0.265	-0.401	0.689	0.5	1.598
SUCO	0.137	0.295	0.464	0.643	1.147	0.339	0.434	0.665	0.643	2.046
SUCLG2	-0.367	0.3	-1.225	0.221	0.693	0.208	-1.481	0.139	0.385	1.247
SUCLG1	0.163	0.295	0.55	0.582	1.177	0.348	0.508	0.611	0.659	2.1
SUCLA2	-0.05	0.297	-0.169	0.866	0.951	0.282	-0.174	0.862	0.532	1.702
SUB1	0.598	0.306	1.958	0.05	1.819	0.556	1.474	0.141	1	3.311
STYXL1	-0.08	0.296	-0.272	0.786	0.923	0.273	-0.283	0.777	0.517	1.647
STYK1	-0.15	0.296	-0.509	0.611	0.86	0.254	-0.549	0.583	0.482	1.536
STXBP6	-0.015	0.297	-0.052	0.959	0.985	0.292	-0.052	0.958	0.55	1.762
STXBP5L	0.461	0.302	1.525	0.127	1.586	0.48	1.222	0.222	0.877	2.869

STXBP3	-0.138	0.297	-0.465	0.642	0.871	0.258	-0.498	0.618	0.487	1.558
STXBP2	-0.163	0.299	-0.544	0.586	0.85	0.254	-0.591	0.554	0.472	1.528
STXBP1	-0.454	0.3	-1.511	0.131	0.635	0.191	-1.912	0.056	0.353	1.144
STX8	0.225	0.296	0.761	0.447	1.253	0.371	0.681	0.496	0.701	2.238
STX7	-0.438	0.3	-1.464	0.143	0.645	0.193	-1.837	0.066	0.359	1.16
STX6	0.186	0.296	0.628	0.53	1.204	0.357	0.573	0.567	0.674	2.152
STX5	0.1	0.295	0.339	0.734	1.105	0.327	0.323	0.747	0.62	1.972
STX4	-0.307	0.296	-1.037	0.3	0.735	0.218	-1.214	0.225	0.411	1.315
STX3	0.799	0.31	2.578	0.01	2.223	0.689	1.776	0.076	1.211	4.079
STX2	0.459	0.3	1.53	0.126	1.582	0.474	1.227	0.22	0.879	2.846
STX1A	0.141	0.298	0.473	0.636	1.151	0.343	0.442	0.659	0.642	2.065
STX18	-0.159	0.296	-0.535	0.592	0.853	0.253	-0.58	0.562	0.477	1.526
STX17	0.527	0.306	1.723	0.085	1.694	0.518	1.339	0.18	0.93	3.084
STX16	0.179	0.298	0.6	0.549	1.196	0.357	0.549	0.583	0.667	2.145
STX12	-0.356	0.304	-1.174	0.24	0.7	0.213	-1.411	0.158	0.386	1.269
STX11	0.083	0.295	0.281	0.778	1.087	0.321	0.27	0.787	0.609	1.938
STX10	-0.065	0.296	-0.22	0.826	0.937	0.278	-0.228	0.82	0.524	1.674
STUM	-0.004	0.295	-0.012	0.99	0.996	0.294	-0.012	0.99	0.558	1.778
STUB1	0.16	0.296	0.541	0.588	1.174	0.348	0.5	0.617	0.657	2.098
STT3A	0.172	0.296	0.58	0.562	1.187	0.352	0.533	0.594	0.664	2.122
STS	-0.06	0.295	-0.205	0.838	0.941	0.278	-0.211	0.833	0.528	1.679
STRN4	0.14	0.295	0.472	0.637	1.15	0.34	0.441	0.659	0.644	2.051
STRN3	0.067	0.295	0.225	0.822	1.069	0.316	0.218	0.827	0.599	1.907
STRN	0.142	0.296	0.479	0.632	1.152	0.341	0.447	0.655	0.645	2.056
STRAP	0.474	0.302	1.57	0.117	1.607	0.486	1.25	0.211	0.889	2.906
STRADA	0.454	0.303	1.496	0.135	1.574	0.477	1.203	0.229	0.869	2.852
STRA6	0.225	0.298	0.757	0.449	1.253	0.373	0.678	0.498	0.699	2.246
STON1	-0.112	0.296	-0.38	0.704	0.894	0.265	-0.402	0.688	0.5	1.597
STOML2	0.314	0.298	1.056	0.291	1.369	0.407	0.906	0.365	0.764	2.453
STOML1	0.751	0.307	2.445	0.014	2.119	0.651	1.719	0.086	1.161	3.869
STOM	-0.12	0.295	-0.407	0.684	0.887	0.262	-0.432	0.666	0.497	1.582
STN1	-0.104	0.297	-0.35	0.727	0.902	0.267	-0.368	0.713	0.504	1.612
STMN4	-0.002	0.295	-0.008	0.994	0.998	0.295	-0.008	0.994	0.559	1.78
STMN3	-0.158	0.298	-0.531	0.595	0.854	0.254	-0.575	0.565	0.477	1.53
STMN2	0.116	0.295	0.392	0.695	1.123	0.332	0.37	0.711	0.629	2.003
STMN1	0.815	0.311	2.62	0.009	2.26	0.703	1.792	0.073	1.228	4.157
STK4	-0.062	0.295	-0.211	0.833	0.94	0.278	-0.218	0.828	0.527	1.677
STK39	-0.768	0.31	-2.478	0.013	0.464	0.144	-3.729	0	0.253	0.852

STK38L	-0.248	0.297	-0.836	0.403	0.78	0.231	-0.949	0.343	0.436	1.396
STK38	0.417	0.303	1.378	0.168	1.517	0.459	1.127	0.26	0.839	2.745
STK32B	-0.593	0.306	-1.941	0.052	0.553	0.169	-2.649	0.008	0.304	1.006
STK3	0.488	0.3	1.625	0.104	1.629	0.489	1.286	0.198	0.904	2.934
STK26	0.852	0.316	2.701	0.007	2.345	0.74	1.818	0.069	1.263	4.352
STK25	-0.045	0.295	-0.153	0.878	0.956	0.282	-0.157	0.875	0.536	1.705
STK24	0.168	0.296	0.565	0.572	1.182	0.35	0.521	0.603	0.662	2.113
STK19	-0.218	0.296	-0.735	0.462	0.804	0.238	-0.821	0.412	0.45	1.438
STK17B	0.008	0.295	0.028	0.978	1.008	0.298	0.028	0.978	0.565	1.798
STK17A	0.426	0.302	1.409	0.159	1.531	0.463	1.147	0.251	0.847	2.77
STK16	0.039	0.296	0.133	0.894	1.04	0.308	0.131	0.896	0.583	1.857
STK11	0.166	0.298	0.558	0.577	1.181	0.351	0.514	0.607	0.659	2.115
STK10	0.149	0.296	0.502	0.616	1.16	0.344	0.467	0.641	0.649	2.074
STIP1	0.078	0.296	0.263	0.792	1.081	0.32	0.253	0.8	0.606	1.93
STIMATE	0.511	0.303	1.686	0.092	1.666	0.505	1.32	0.187	0.92	3.017
STIM1	-0.261	0.3	-0.869	0.385	0.771	0.231	-0.993	0.321	0.428	1.387
STIL	0.6	0.306	1.961	0.05	1.822	0.557	1.475	0.14	1	3.317
STEAP4	-0.452	0.3	-1.51	0.131	0.636	0.191	-1.909	0.056	0.354	1.144
STEAP3	-0.101	0.297	-0.34	0.734	0.904	0.268	-0.358	0.72	0.505	1.617
STEAP1B	0.167	0.295	0.567	0.571	1.182	0.349	0.522	0.602	0.663	2.109
STEAP1	-0.727	0.315	-2.31	0.021	0.483	0.152	-3.395	0.001	0.261	0.896
STC2	-0.385	0.303	-1.27	0.204	0.68	0.206	-1.55	0.121	0.375	1.233
STC1	0.464	0.3	1.548	0.122	1.59	0.477	1.238	0.216	0.884	2.861
STAU2	0.109	0.295	0.369	0.712	1.115	0.329	0.35	0.727	0.625	1.989
STAU1	0.723	0.31	2.335	0.02	2.061	0.638	1.662	0.096	1.123	3.781
STATH	-0.322	0.3	-1.076	0.282	0.725	0.217	-1.269	0.204	0.403	1.303
STAT6	-0.551	0.302	-1.823	0.068	0.576	0.174	-2.433	0.015	0.319	1.042
STAT5B	-0.609	0.304	-2.004	0.045	0.544	0.165	-2.759	0.006	0.3	0.987
STAT5A	-0.726	0.307	-2.361	0.018	0.484	0.149	-3.468	0.001	0.265	0.884
STAT4	-0.378	0.298	-1.267	0.205	0.685	0.204	-1.54	0.123	0.382	1.229
STAT3	0.621	0.306	2.031	0.042	1.861	0.569	1.513	0.13	1.022	3.39
STAT2	0.225	0.297	0.758	0.448	1.252	0.372	0.679	0.497	0.7	2.24
STAT1	0.15	0.296	0.507	0.612	1.162	0.344	0.471	0.638	0.65	2.077
STARD8	-0.332	0.297	-1.118	0.263	0.718	0.213	-1.326	0.185	0.401	1.284
STARD7	0.494	0.3	1.649	0.099	1.639	0.491	1.301	0.193	0.911	2.948
STARD5	-0.081	0.296	-0.274	0.784	0.922	0.273	-0.285	0.776	0.517	1.646
STARD3	0.125	0.295	0.422	0.673	1.133	0.335	0.397	0.692	0.635	2.021
STARD13	-0.094	0.295	-0.32	0.749	0.91	0.269	-0.335	0.737	0.51	1.623

STAR	-0.091	0.295	-0.308	0.758	0.913	0.269	-0.322	0.747	0.512	1.628
STAP2	0.316	0.298	1.061	0.289	1.371	0.408	0.91	0.363	0.765	2.457
STAP1	-0.344	0.298	-1.154	0.249	0.709	0.211	-1.377	0.169	0.396	1.271
STAMBPL1	0.296	0.298	0.996	0.319	1.345	0.4	0.862	0.389	0.75	2.41
STAMBP	0.894	0.316	2.831	0.005	2.444	0.772	1.872	0.061	1.316	4.538
STAM2	0.18	0.296	0.606	0.544	1.197	0.354	0.555	0.579	0.67	2.138
STAM	0.421	0.299	1.41	0.159	1.524	0.456	1.15	0.25	0.848	2.739
STAG3L4	0.585	0.303	1.932	0.053	1.794	0.543	1.463	0.143	0.992	3.247
STAG3	0.247	0.298	0.829	0.407	1.28	0.381	0.735	0.462	0.714	2.295
STAG2	-0.289	0.297	-0.972	0.331	0.749	0.223	-1.126	0.26	0.418	1.342
STAG1	0.038	0.295	0.128	0.898	1.039	0.307	0.126	0.9	0.582	1.852
STAC	0.342	0.298	1.148	0.251	1.407	0.419	0.972	0.331	0.785	2.521
STAB2	-0.734	0.307	-2.395	0.017	0.48	0.147	-3.535	0	0.263	0.875
STAB1	0.003	0.296	0.011	0.991	1.003	0.297	0.011	0.991	0.561	1.794
ST8SIA5	-0.143	0.296	-0.484	0.629	0.867	0.257	-0.52	0.603	0.485	1.548
ST8SIA4	-0.861	0.316	-2.727	0.006	0.423	0.133	-4.326	0	0.228	0.785
ST8SIA3	0.417	0.303	1.376	0.169	1.517	0.459	1.126	0.26	0.838	2.746
ST8SIA2	-0.56	0.306	-1.832	0.067	0.571	0.175	-2.455	0.014	0.314	1.04
ST8SIA1	0.179	0.296	0.603	0.546	1.196	0.354	0.552	0.581	0.669	2.137
ST7L	-0.381	0.3	-1.273	0.203	0.683	0.205	-1.55	0.121	0.38	1.228
ST6GALNAC5	-0.168	0.298	-0.564	0.573	0.845	0.252	-0.614	0.539	0.471	1.516
ST6GALNAC4	-0.378	0.3	-1.262	0.207	0.685	0.205	-1.534	0.125	0.381	1.232
ST6GALNAC2	0.154	0.296	0.521	0.602	1.167	0.346	0.483	0.629	0.653	2.085
ST6GAL1	-0.09	0.295	-0.304	0.761	0.914	0.27	-0.318	0.75	0.513	1.631
ST3GAL6	-0.181	0.296	-0.612	0.54	0.834	0.247	-0.671	0.502	0.467	1.491
ST3GAL5	-0.039	0.295	-0.132	0.895	0.962	0.284	-0.135	0.893	0.539	1.715
ST3GAL4	0.321	0.3	1.073	0.283	1.379	0.413	0.917	0.359	0.767	2.481
ST3GAL2	0.357	0.3	1.193	0.233	1.429	0.428	1.003	0.316	0.795	2.571
ST3GAL1	0.041	0.295	0.139	0.89	1.042	0.307	0.136	0.892	0.584	1.858
ST20	0.474	0.302	1.568	0.117	1.606	0.485	1.249	0.212	0.888	2.905
ST18	0.029	0.296	0.098	0.922	1.029	0.305	0.097	0.923	0.576	1.839
ST14	0.411	0.298	1.379	0.168	1.509	0.45	1.13	0.258	0.841	2.708
ST13	-0.114	0.296	-0.386	0.699	0.892	0.264	-0.409	0.682	0.499	1.593
SSX3	0.766	0.315	2.435	0.015	2.152	0.677	1.701	0.089	1.161	3.987
SSX2IP	0.102	0.298	0.341	0.733	1.107	0.33	0.324	0.746	0.617	1.985
SSX1	0.225	0.296	0.761	0.447	1.253	0.371	0.681	0.496	0.701	2.24
SSUH2	0.025	0.296	0.084	0.933	1.025	0.303	0.083	0.934	0.574	1.83
SSTR5	0.603	0.306	1.973	0.049	1.828	0.559	1.481	0.139	1.004	3.328

SSTR4	0.175	0.296	0.589	0.556	1.191	0.353	0.54	0.589	0.666	2.128
SSTR3	0.688	0.307	2.244	0.025	1.99	0.61	1.622	0.105	1.091	3.631
SSTR2	0.175	0.296	0.591	0.555	1.191	0.353	0.542	0.588	0.667	2.129
SSTR1	0.134	0.297	0.452	0.652	1.144	0.34	0.423	0.673	0.639	2.049
SST	-0.033	0.295	-0.111	0.911	0.968	0.286	-0.113	0.91	0.543	1.725
SSRP1	-0.068	0.295	-0.231	0.817	0.934	0.276	-0.239	0.811	0.524	1.667
SSR4	-0.162	0.296	-0.547	0.584	0.85	0.252	-0.594	0.553	0.476	1.52
SSR3	-0.109	0.296	-0.367	0.713	0.897	0.265	-0.388	0.698	0.503	1.601
SSR2	-0.369	0.3	-1.231	0.218	0.691	0.207	-1.489	0.137	0.384	1.244
SSR1	-0.089	0.296	-0.301	0.764	0.915	0.271	-0.315	0.753	0.512	1.635
SSPN	-0.763	0.31	-2.463	0.014	0.466	0.144	-3.696	0	0.254	0.856
SSNA1	0.222	0.296	0.75	0.454	1.249	0.37	0.672	0.501	0.699	2.231
SSH3	-0.512	0.3	-1.709	0.088	0.599	0.18	-2.232	0.026	0.333	1.078
SSH1	-0.338	0.298	-1.135	0.256	0.713	0.212	-1.35	0.177	0.398	1.279
SSBP3	-0.103	0.295	-0.35	0.726	0.902	0.266	-0.369	0.712	0.506	1.609
SSBP2	0.158	0.296	0.535	0.593	1.172	0.347	0.494	0.621	0.655	2.094
SSBP1	0.256	0.297	0.864	0.387	1.292	0.383	0.762	0.446	0.723	2.311
SSB	0.763	0.31	2.462	0.014	2.145	0.665	1.723	0.085	1.168	3.936
SS18L2	-0.093	0.296	-0.315	0.753	0.911	0.269	-0.33	0.741	0.51	1.626
SS18L1	0.527	0.303	1.74	0.082	1.693	0.512	1.353	0.176	0.936	3.064
SS18	-0.907	0.321	-2.826	0.005	0.404	0.13	-4.602	0	0.215	0.757
SRY	-0.587	0.303	-1.94	0.052	0.556	0.168	-2.641	0.008	0.307	1.006
SRSF8	-0.25	0.296	-0.843	0.399	0.779	0.231	-0.958	0.338	0.436	1.392
SRSF7	0.32	0.3	1.067	0.286	1.378	0.414	0.913	0.361	0.765	2.483
SRSF6	-0.492	0.301	-1.634	0.102	0.612	0.184	-2.111	0.035	0.339	1.103
SRSF5	-0.627	0.306	-2.05	0.04	0.534	0.163	-2.851	0.004	0.293	0.973
SRSF4	0.26	0.296	0.879	0.38	1.297	0.384	0.773	0.439	0.726	2.318
SRSF3	0.842	0.311	2.708	0.007	2.322	0.722	1.83	0.067	1.262	4.271
SRSF11	0.315	0.298	1.059	0.29	1.37	0.408	0.908	0.364	0.765	2.456
SRSF10	0.172	0.296	0.583	0.56	1.188	0.351	0.535	0.593	0.665	2.121
SRSF1	1.127	0.336	3.354	0.001	3.088	1.038	2.012	0.044	1.598	5.966
SRRT	0.369	0.3	1.231	0.218	1.446	0.433	1.029	0.303	0.804	2.601
SRRM2	-0.393	0.299	-1.315	0.188	0.675	0.202	-1.611	0.107	0.376	1.212
SRRM1	0.235	0.296	0.793	0.428	1.265	0.375	0.706	0.48	0.708	2.26
SRRD	0.75	0.307	2.445	0.014	2.117	0.649	1.72	0.085	1.16	3.861
SRR	-0.01	0.295	-0.033	0.973	0.99	0.292	-0.034	0.973	0.555	1.766
SRPX2	-0.348	0.298	-1.168	0.243	0.706	0.21	-1.396	0.163	0.394	1.266
SRPX	-0.481	0.3	-1.606	0.108	0.618	0.185	-2.062	0.039	0.343	1.112

SRPRB	0.142	0.296	0.479	0.632	1.153	0.342	0.447	0.655	0.645	2.061
SRPRA	-0.816	0.315	-2.59	0.01	0.442	0.139	-4.003	0	0.239	0.82
SRPK3	-0.056	0.297	-0.188	0.851	0.946	0.281	-0.193	0.847	0.528	1.693
SRPK2	-0.051	0.295	-0.173	0.863	0.95	0.281	-0.178	0.859	0.532	1.695
SRPK1	0.413	0.304	1.362	0.173	1.512	0.459	1.115	0.265	0.834	2.741
SRP9	0.131	0.296	0.442	0.658	1.14	0.338	0.415	0.678	0.638	2.038
SRP72	0.168	0.296	0.57	0.569	1.183	0.35	0.524	0.6	0.663	2.112
SRP54	-0.027	0.297	-0.092	0.927	0.973	0.289	-0.093	0.926	0.543	1.743
SRP19	0.156	0.296	0.526	0.599	1.169	0.346	0.487	0.626	0.654	2.088
SRP14	0.074	0.296	0.25	0.803	1.077	0.319	0.241	0.81	0.603	1.923
SRM	-0.55	0.302	-1.819	0.069	0.577	0.174	-2.425	0.015	0.319	1.044
SRI	0.338	0.298	1.134	0.257	1.402	0.417	0.963	0.336	0.782	2.512
SRGN	0.228	0.298	0.763	0.445	1.256	0.375	0.682	0.495	0.7	2.253
SRGAP3	0	0.295	-0.001	0.999	1	0.295	-0.001	0.999	0.561	1.783
SRF	0.353	0.3	1.176	0.24	1.423	0.427	0.991	0.322	0.791	2.56
SREK1IP1	0.903	0.313	2.889	0.004	2.467	0.771	1.903	0.057	1.337	4.552
SREK1	-0.67	0.306	-2.188	0.029	0.512	0.157	-3.116	0.002	0.281	0.933
SREBF2	-0.179	0.296	-0.604	0.546	0.836	0.248	-0.662	0.508	0.468	1.494
SREBF1	-0.321	0.298	-1.078	0.281	0.725	0.216	-1.271	0.204	0.405	1.301
SRD5A3	0.025	0.295	0.083	0.934	1.025	0.303	0.082	0.934	0.575	1.828
SRD5A2	-0.486	0.3	-1.619	0.105	0.615	0.185	-2.085	0.037	0.341	1.108
SRD5A1	0.187	0.297	0.628	0.53	1.205	0.358	0.573	0.567	0.673	2.159
SRCAP	-0.13	0.296	-0.441	0.659	0.878	0.259	-0.471	0.637	0.492	1.567
SRC	0.278	0.297	0.935	0.35	1.32	0.392	0.817	0.414	0.738	2.364
SRBD1	-0.294	0.298	-0.984	0.325	0.746	0.222	-1.143	0.253	0.415	1.338
SQSTM1	-0.717	0.31	-2.312	0.021	0.488	0.151	-3.38	0.001	0.266	0.897
SQOR	-0.503	0.313	-1.605	0.109	0.605	0.189	-2.085	0.037	0.327	1.118
SQLE	0.449	0.307	1.46	0.144	1.566	0.481	1.177	0.239	0.858	2.86
SPX	-0.048	0.295	-0.161	0.872	0.954	0.282	-0.165	0.869	0.534	1.701
SPTSSA	0.101	0.295	0.342	0.732	1.106	0.327	0.325	0.745	0.62	1.973
SPTLC3	1.057	0.322	3.282	0.001	2.877	0.926	2.026	0.043	1.531	5.408
SPTLC2	0.691	0.31	2.229	0.026	1.996	0.619	1.61	0.107	1.087	3.665
SPTLC1	-0.16	0.296	-0.542	0.588	0.852	0.252	-0.588	0.557	0.477	1.522
SPTBN5	-0.141	0.295	-0.479	0.632	0.868	0.256	-0.514	0.607	0.487	1.549
SPTBN4	0.08	0.295	0.272	0.786	1.084	0.32	0.261	0.794	0.607	1.934
SPTBN2	0.121	0.296	0.409	0.682	1.129	0.334	0.385	0.7	0.632	2.017
SPTBN1	-0.149	0.296	-0.504	0.614	0.861	0.255	-0.544	0.586	0.483	1.538
SPTB	0.235	0.298	0.791	0.429	1.266	0.377	0.705	0.481	0.706	2.268

SPTAN1	-0.872	0.316	-2.763	0.006	0.418	0.132	-4.409	0	0.225	0.776
SPTA1	0.55	0.306	1.801	0.072	1.734	0.53	1.385	0.166	0.953	3.156
SPSB3	-0.027	0.296	-0.09	0.928	0.974	0.288	-0.092	0.927	0.545	1.738
SPSB1	-0.023	0.297	-0.079	0.937	0.977	0.29	-0.08	0.936	0.546	1.748
SPRYD7	0.551	0.306	1.8	0.072	1.735	0.531	1.384	0.166	0.952	3.16
SPRY4	-0.615	0.306	-2.008	0.045	0.54	0.166	-2.775	0.006	0.296	0.985
SPRY2	-0.086	0.295	-0.292	0.77	0.917	0.271	-0.305	0.76	0.515	1.636
SPRY1	-0.176	0.296	-0.595	0.552	0.838	0.248	-0.65	0.515	0.469	1.498
SPRR3	0.022	0.295	0.076	0.94	1.023	0.302	0.075	0.94	0.574	1.823
SPRR2C	0.331	0.297	1.114	0.265	1.392	0.413	0.949	0.343	0.778	2.492
SPRR1B	0.322	0.3	1.074	0.283	1.38	0.413	0.918	0.359	0.767	2.482
SPRR1A	-0.546	0.303	-1.803	0.071	0.579	0.175	-2.399	0.016	0.32	1.049
SPRING1	-0.211	0.297	-0.711	0.477	0.81	0.24	-0.792	0.428	0.453	1.449
SPRED2	-0.733	0.31	-2.364	0.018	0.481	0.149	-3.486	0	0.262	0.882
SPR	0.057	0.295	0.193	0.847	1.059	0.313	0.188	0.851	0.593	1.889
SPPL2B	-0.349	0.299	-1.169	0.242	0.705	0.211	-1.399	0.162	0.393	1.267
SPP2	-0.548	0.303	-1.805	0.071	0.578	0.175	-2.404	0.016	0.319	1.048
SPP1	0.266	0.296	0.898	0.369	1.305	0.386	0.788	0.431	0.73	2.331
SPOUT1	0.085	0.296	0.288	0.773	1.089	0.322	0.276	0.783	0.61	1.944
SPOP	-0.296	0.298	-0.996	0.319	0.744	0.221	-1.159	0.247	0.415	1.332
SPON1	-0.88	0.315	-2.793	0.005	0.415	0.131	-4.477	0	0.224	0.769
SPOCK3	0.377	0.302	1.248	0.212	1.458	0.441	1.04	0.298	0.806	2.637
SPOCK2	-0.211	0.297	-0.712	0.476	0.809	0.24	-0.793	0.428	0.452	1.448
SPOCK1	-0.056	0.295	-0.189	0.85	0.946	0.279	-0.195	0.846	0.53	1.687
SPO11	-0.632	0.303	-2.084	0.037	0.532	0.161	-2.907	0.004	0.293	0.963
SPN	0.392	0.298	1.315	0.188	1.48	0.441	1.088	0.277	0.825	2.655
SPINT3	-0.054	0.296	-0.183	0.855	0.947	0.281	-0.188	0.851	0.53	1.693
SPINT2	0.342	0.298	1.15	0.25	1.408	0.419	0.974	0.33	0.786	2.524
SPINT1	0.357	0.298	1.199	0.231	1.429	0.426	1.008	0.313	0.797	2.563
SPINK5	-0.218	0.296	-0.737	0.461	0.804	0.238	-0.823	0.41	0.45	1.437
SPINK4	0.146	0.295	0.495	0.62	1.158	0.342	0.461	0.645	0.649	2.065
SPINK2	-0.064	0.295	-0.217	0.828	0.938	0.277	-0.225	0.822	0.526	1.672
SPINK1	0.646	0.31	2.085	0.037	1.907	0.591	1.536	0.125	1.039	3.5
SPIN2A	-0.159	0.295	-0.537	0.591	0.853	0.252	-0.582	0.56	0.478	1.522
SPIN1	-0.35	0.298	-1.177	0.239	0.705	0.21	-1.409	0.159	0.393	1.262
SPIDR	-0.241	0.298	-0.808	0.419	0.786	0.234	-0.914	0.361	0.439	1.409
SPICE1	-0.061	0.295	-0.207	0.836	0.941	0.278	-0.213	0.831	0.527	1.678
SPIB	-0.063	0.296	-0.212	0.832	0.939	0.278	-0.219	0.827	0.525	1.679

SPI1	-0.249	0.298	-0.836	0.403	0.779	0.232	-0.949	0.343	0.434	1.399
SPHK2	0.326	0.298	1.094	0.274	1.385	0.412	0.934	0.35	0.773	2.482
SPHK1	-0.022	0.296	-0.076	0.94	0.978	0.289	-0.076	0.939	0.547	1.747
SPG21	-0.238	0.297	-0.801	0.423	0.788	0.234	-0.904	0.366	0.44	1.411
SPG11	-0.638	0.31	-2.059	0.04	0.528	0.164	-2.88	0.004	0.288	0.97
SPEN	-0.32	0.298	-1.076	0.282	0.726	0.216	-1.268	0.205	0.405	1.301
SPEF1	-0.292	0.298	-0.98	0.327	0.747	0.223	-1.138	0.255	0.416	1.339
SPECC1L	-0.02	0.295	-0.067	0.946	0.98	0.29	-0.068	0.946	0.549	1.749
SPDYE2	0.019	0.298	0.065	0.948	1.019	0.304	0.064	0.949	0.568	1.829
SPDL1	0.709	0.31	2.287	0.022	2.033	0.631	1.638	0.102	1.107	3.734
SPDEF	0.224	0.296	0.755	0.45	1.251	0.37	0.677	0.498	0.7	2.235
SPCS3	-0.196	0.296	-0.663	0.508	0.822	0.243	-0.732	0.464	0.46	1.468
SPCS2	-0.3	0.3	-1	0.317	0.741	0.222	-1.165	0.244	0.412	1.333
SPCS1	-0.054	0.296	-0.183	0.855	0.947	0.28	-0.188	0.851	0.531	1.691
SPC25	0.584	0.302	1.931	0.053	1.793	0.542	1.463	0.144	0.991	3.243
SPATS2L	-0.771	0.31	-2.488	0.013	0.462	0.143	-3.75	0	0.252	0.849
SPATS2	0.315	0.297	1.061	0.289	1.37	0.407	0.91	0.363	0.766	2.452
SPATA7	0.051	0.297	0.17	0.865	1.052	0.313	0.166	0.868	0.587	1.883
SPATA6L	0.152	0.296	0.513	0.608	1.164	0.345	0.476	0.634	0.651	2.08
SPATA6	-0.52	0.306	-1.701	0.089	0.595	0.182	-2.23	0.026	0.327	1.082
SPATA5L1	-0.045	0.297	-0.151	0.88	0.956	0.284	-0.155	0.877	0.534	1.713
SPATA31C2	0.573	0.303	1.894	0.058	1.774	0.537	1.441	0.15	0.98	3.212
SPATA2L	0.577	0.301	1.914	0.056	1.78	0.537	1.454	0.146	0.986	3.214
SPATA20	-0.099	0.295	-0.334	0.738	0.906	0.268	-0.351	0.726	0.508	1.616
SPATA2	-0.864	0.321	-2.696	0.007	0.421	0.135	-4.283	0	0.225	0.79
SPATA1	-0.084	0.295	-0.286	0.775	0.919	0.272	-0.298	0.765	0.515	1.64
SPAST	-0.772	0.308	-2.504	0.012	0.462	0.142	-3.775	0	0.253	0.846
SPART	-0.374	0.3	-1.249	0.212	0.688	0.206	-1.515	0.13	0.382	1.237
SPARCL1	-0.459	0.3	-1.533	0.125	0.632	0.189	-1.945	0.052	0.351	1.137
SPARC	-0.52	0.302	-1.72	0.085	0.595	0.18	-2.256	0.024	0.329	1.075
SPAM1	0.926	0.315	2.94	0.003	2.525	0.795	1.917	0.055	1.362	4.681
SPAG9	-0.139	0.297	-0.466	0.641	0.871	0.259	-0.5	0.617	0.486	1.559
SPAG8	0.152	0.296	0.515	0.606	1.165	0.344	0.478	0.633	0.652	2.079
SPAG7	-0.279	0.3	-0.93	0.352	0.757	0.227	-1.073	0.283	0.42	1.362
SPAG6	-0.049	0.295	-0.166	0.868	0.952	0.281	-0.17	0.865	0.534	1.699
SPAG5	0.984	0.321	3.069	0.002	2.675	0.857	1.953	0.051	1.427	5.013
SPAG4	-0.07	0.295	-0.239	0.811	0.932	0.275	-0.247	0.805	0.522	1.662
SPAG16	0.122	0.295	0.412	0.68	1.129	0.334	0.388	0.698	0.633	2.015

SPAG11A	-0.09	0.295	-0.304	0.761	0.914	0.27	-0.318	0.75	0.512	1.631
SPAG1	-0.013	0.295	-0.043	0.965	0.987	0.292	-0.044	0.965	0.553	1.761
SPACA9	0.092	0.296	0.31	0.757	1.096	0.325	0.296	0.767	0.613	1.959
SPACA1	0.083	0.296	0.28	0.779	1.087	0.322	0.269	0.788	0.608	1.943
SPA17	-0.262	0.298	-0.88	0.379	0.77	0.229	-1.006	0.314	0.429	1.379
SP4	-0.228	0.297	-0.768	0.443	0.796	0.236	-0.862	0.389	0.445	1.424
SP3P	0.067	0.295	0.226	0.821	1.069	0.316	0.218	0.827	0.599	1.907
SP3	-0.052	0.295	-0.176	0.86	0.949	0.28	-0.181	0.857	0.532	1.694
SP2	0.249	0.298	0.836	0.403	1.283	0.382	0.74	0.459	0.716	2.299
SP140L	-0.412	0.3	-1.372	0.17	0.663	0.199	-1.698	0.09	0.368	1.193
SP140	0.057	0.295	0.194	0.846	1.059	0.313	0.189	0.85	0.593	1.89
SP110	-0.042	0.296	-0.143	0.887	0.959	0.284	-0.146	0.884	0.537	1.712
SP100	-0.33	0.297	-1.112	0.266	0.719	0.213	-1.318	0.188	0.402	1.286
SP1	-0.265	0.298	-0.89	0.374	0.767	0.228	-1.019	0.308	0.428	1.375
SOX9	0.409	0.3	1.363	0.173	1.505	0.451	1.119	0.263	0.836	2.707
SOX5	-0.215	0.296	-0.727	0.467	0.806	0.239	-0.811	0.417	0.451	1.441
SOX4	0.87	0.321	2.714	0.007	2.387	0.765	1.812	0.07	1.273	4.474
SOX30	0.416	0.3	1.39	0.165	1.516	0.454	1.137	0.256	0.843	2.727
SOX3	0.258	0.298	0.868	0.385	1.295	0.386	0.765	0.444	0.722	2.321
SOX21	-0.351	0.3	-1.169	0.242	0.704	0.211	-1.401	0.161	0.391	1.268
SOX2	0.786	0.315	2.497	0.013	2.194	0.691	1.729	0.084	1.184	4.066
SOX18	-0.399	0.3	-1.333	0.183	0.671	0.201	-1.638	0.101	0.373	1.207
SOX17	-0.41	0.3	-1.367	0.172	0.664	0.199	-1.689	0.091	0.369	1.195
SOX15	-0.029	0.295	-0.099	0.921	0.971	0.287	-0.1	0.92	0.545	1.732
SOX14	-0.422	0.303	-1.396	0.163	0.655	0.198	-1.738	0.082	0.362	1.186
SOX13	-0.282	0.297	-0.949	0.342	0.754	0.224	-1.097	0.273	0.422	1.35
SOX12	0.368	0.3	1.23	0.219	1.445	0.433	1.029	0.304	0.803	2.601
SOX11	0.944	0.321	2.943	0.003	2.57	0.825	1.904	0.057	1.371	4.82
SOX10	-0.002	0.295	-0.007	0.994	0.998	0.295	-0.007	0.994	0.559	1.78
SOX1	-0.361	0.298	-1.211	0.226	0.697	0.208	-1.458	0.145	0.389	1.25
SOWAHC	-0.478	0.302	-1.582	0.114	0.62	0.187	-2.029	0.042	0.343	1.121
SOSTDC1	-0.152	0.296	-0.512	0.609	0.859	0.254	-0.553	0.58	0.481	1.535
SOS2	-0.146	0.296	-0.493	0.622	0.864	0.256	-0.531	0.595	0.483	1.544
SOS1	0.154	0.297	0.52	0.603	1.167	0.346	0.482	0.63	0.652	2.086
SORT1	0.172	0.295	0.583	0.56	1.188	0.351	0.535	0.592	0.666	2.119
SORL1	-0.291	0.298	-0.976	0.329	0.748	0.223	-1.132	0.258	0.417	1.341
SORD	-0.44	0.3	-1.468	0.142	0.644	0.193	-1.845	0.065	0.358	1.159
SORCS3	0.171	0.297	0.577	0.564	1.187	0.352	0.53	0.596	0.664	2.122

SORBS3	-0.353	0.3	-1.179	0.239	0.702	0.211	-1.413	0.158	0.39	1.264
SORBS2	-0.119	0.295	-0.401	0.688	0.888	0.262	-0.426	0.67	0.498	1.584
SORBS1	-0.207	0.299	-0.69	0.49	0.813	0.243	-0.767	0.443	0.453	1.462
SON	-0.226	0.296	-0.762	0.446	0.798	0.236	-0.855	0.393	0.447	1.426
SOGA1	0.103	0.296	0.348	0.728	1.108	0.328	0.331	0.741	0.621	1.979
SOD3	-0.28	0.296	-0.944	0.345	0.756	0.224	-1.089	0.276	0.423	1.351
SOD1	0.793	0.308	2.576	0.01	2.209	0.68	1.779	0.075	1.209	4.038
SOCS7	0.408	0.3	1.363	0.173	1.504	0.451	1.119	0.263	0.836	2.706
SOCS6	-0.496	0.306	-1.624	0.104	0.609	0.186	-2.103	0.035	0.334	1.108
SOCS5	-0.186	0.296	-0.627	0.53	0.83	0.246	-0.69	0.49	0.465	1.484
SOCS3	0.177	0.296	0.599	0.549	1.194	0.354	0.549	0.583	0.668	2.134
SOCS2	-0.528	0.302	-1.747	0.081	0.59	0.178	-2.302	0.021	0.326	1.066
SOCS1	-0.04	0.295	-0.134	0.893	0.961	0.284	-0.137	0.891	0.539	1.715
SOBP	0.051	0.297	0.173	0.863	1.053	0.313	0.168	0.866	0.588	1.884
SOAT2	0.137	0.297	0.463	0.643	1.147	0.34	0.433	0.665	0.642	2.051
SOAT1	0.165	0.295	0.56	0.575	1.18	0.348	0.516	0.606	0.661	2.105
SNX7	-0.18	0.298	-0.605	0.545	0.835	0.249	-0.663	0.507	0.466	1.498
SNX6	0.027	0.295	0.092	0.927	1.028	0.303	0.091	0.928	0.576	1.833
SNX5	-0.31	0.298	-1.04	0.298	0.734	0.218	-1.219	0.223	0.409	1.315
SNX4	0.038	0.295	0.128	0.899	1.038	0.307	0.125	0.9	0.582	1.852
SNX3	-0.284	0.296	-0.959	0.337	0.753	0.223	-1.109	0.267	0.421	1.345
SNX29P2	0.077	0.296	0.261	0.794	1.08	0.32	0.251	0.802	0.605	1.929
SNX27	-0.217	0.296	-0.732	0.464	0.805	0.238	-0.818	0.413	0.451	1.438
SNX24	-0.435	0.307	-1.419	0.156	0.647	0.199	-1.778	0.075	0.355	1.181
SNX2	-0.246	0.298	-0.827	0.408	0.782	0.233	-0.937	0.349	0.436	1.401
SNX19	-0.646	0.306	-2.109	0.035	0.524	0.161	-2.963	0.003	0.288	0.956
SNX17	-0.556	0.311	-1.785	0.074	0.574	0.179	-2.387	0.017	0.312	1.056
SNX16	-0.176	0.296	-0.596	0.551	0.838	0.248	-0.651	0.515	0.469	1.498
SNX15	-0.076	0.296	-0.256	0.798	0.927	0.275	-0.266	0.79	0.519	1.657
SNX13	0.225	0.296	0.759	0.448	1.252	0.371	0.68	0.496	0.701	2.238
SNX11	0.135	0.296	0.454	0.65	1.144	0.339	0.425	0.671	0.64	2.045
SNX10	0.493	0.302	1.629	0.103	1.637	0.495	1.286	0.198	0.905	2.961
SNX1	-0.492	0.302	-1.629	0.103	0.611	0.185	-2.105	0.035	0.338	1.105
SNW1	0.5	0.3	1.666	0.096	1.649	0.496	1.311	0.19	0.915	2.972
SNUPN	-0.209	0.298	-0.7	0.484	0.812	0.242	-0.778	0.436	0.453	1.455
SNU13	-0.386	0.302	-1.276	0.202	0.68	0.206	-1.557	0.119	0.376	1.23
SNTG2	0.13	0.295	0.441	0.66	1.139	0.336	0.413	0.68	0.638	2.032
SNTG1	-0.126	0.296	-0.427	0.67	0.881	0.261	-0.455	0.649	0.493	1.575

SNTB2	0.547	0.306	1.791	0.073	1.729	0.528	1.379	0.168	0.95	3.147
SNTB1	-0.706	0.31	-2.28	0.023	0.493	0.153	-3.314	0.001	0.269	0.906
SNTA1	0.406	0.3	1.356	0.175	1.501	0.45	1.114	0.265	0.834	2.702
SNRPG	0.902	0.315	2.862	0.004	2.465	0.777	1.885	0.059	1.329	4.572
SNRPF	0.26	0.3	0.869	0.385	1.298	0.389	0.765	0.444	0.721	2.334
SNRPE	0.534	0.303	1.765	0.078	1.706	0.516	1.367	0.171	0.943	3.087
SNRPD3	0.585	0.306	1.915	0.055	1.795	0.549	1.45	0.147	0.986	3.268
SNRPD2	0.189	0.295	0.641	0.521	1.208	0.357	0.584	0.559	0.677	2.156
SNRPD1	0.356	0.298	1.195	0.232	1.427	0.425	1.006	0.314	0.796	2.558
SNRPC	0.664	0.307	2.166	0.03	1.942	0.595	1.583	0.113	1.065	3.542
SNRPB2	0.677	0.31	2.184	0.029	1.967	0.61	1.587	0.113	1.072	3.611
SNRPB	0.371	0.3	1.238	0.216	1.449	0.434	1.034	0.301	0.805	2.607
SNRPA1	0.686	0.306	2.246	0.025	1.987	0.607	1.625	0.104	1.091	3.616
SNRPA	0.62	0.302	2.054	0.04	1.859	0.561	1.531	0.126	1.029	3.359
SNRNP70	-0.442	0.299	-1.477	0.14	0.643	0.192	-1.857	0.063	0.357	1.156
SNRNP40	0.08	0.295	0.271	0.787	1.083	0.32	0.26	0.795	0.607	1.932
SNRNP35	0.154	0.297	0.518	0.604	1.166	0.346	0.48	0.631	0.652	2.085
SNRNP27	0.011	0.296	0.039	0.969	1.012	0.299	0.038	0.969	0.566	1.806
SNRNP25	-0.164	0.298	-0.55	0.583	0.849	0.253	-0.597	0.55	0.473	1.523
SNRK	-0.856	0.315	-2.717	0.007	0.425	0.134	-4.297	0	0.229	0.788
SNPH	-0.01	0.296	-0.034	0.973	0.99	0.293	-0.034	0.973	0.555	1.767
SNORA21	-0.197	0.297	-0.664	0.506	0.821	0.244	-0.734	0.463	0.459	1.469
SNN	0.672	0.31	2.17	0.03	1.958	0.607	1.58	0.114	1.067	3.594
SNIP1	0.085	0.295	0.287	0.774	1.088	0.321	0.275	0.783	0.61	1.942
SNHG32	0.163	0.296	0.549	0.583	1.177	0.349	0.507	0.612	0.658	2.103
SNHG17	0.161	0.298	0.539	0.59	1.175	0.35	0.498	0.618	0.655	2.108
SNF8	0.489	0.306	1.597	0.11	1.63	0.499	1.263	0.206	0.895	2.969
SNED1	-0.262	0.296	-0.886	0.376	0.769	0.228	-1.013	0.311	0.43	1.375
SND1-IT1	0.01	0.295	0.033	0.974	1.01	0.298	0.033	0.974	0.566	1.8
SND1	0.011	0.297	0.036	0.971	1.011	0.3	0.036	0.971	0.565	1.808
SNCG	-0.31	0.298	-1.04	0.298	0.734	0.218	-1.22	0.223	0.409	1.315
SNCB	0.208	0.296	0.703	0.482	1.232	0.365	0.635	0.525	0.689	2.201
SNCAIP	0.044	0.295	0.149	0.881	1.045	0.309	0.146	0.884	0.586	1.864
SNCA	-0.173	0.296	-0.583	0.56	0.841	0.249	-0.636	0.525	0.471	1.504
SNAPC5	0.308	0.3	1.026	0.305	1.361	0.408	0.883	0.377	0.756	2.45
SNAPC4	0.444	0.302	1.47	0.141	1.56	0.471	1.187	0.235	0.862	2.82
SNAPC3	0.632	0.303	2.085	0.037	1.882	0.571	1.545	0.122	1.039	3.409
SNAPC2	0.068	0.296	0.231	0.817	1.071	0.317	0.223	0.823	0.6	1.912

SNAPC1	0.297	0.298	0.998	0.318	1.346	0.401	0.864	0.388	0.751	2.412
SNAP91	-0.208	0.296	-0.701	0.483	0.813	0.241	-0.779	0.436	0.455	1.452
SNAP29	0.703	0.306	2.295	0.022	2.019	0.618	1.648	0.099	1.108	3.68
SNAP25	0.453	0.303	1.496	0.135	1.573	0.477	1.203	0.229	0.869	2.849
SNAP23	-0.457	0.303	-1.509	0.131	0.633	0.192	-1.913	0.056	0.35	1.146
SNAI2	-0.794	0.31	-2.559	0.01	0.452	0.14	-3.908	0	0.246	0.83
SNAI1	-0.392	0.3	-1.307	0.191	0.676	0.203	-1.6	0.11	0.376	1.216
SMYD5	-0.02	0.295	-0.066	0.947	0.981	0.289	-0.067	0.946	0.55	1.748
SMYD3	-0.482	0.302	-1.594	0.111	0.618	0.187	-2.048	0.041	0.342	1.117
SMYD2	0.984	0.322	3.055	0.002	2.674	0.861	1.945	0.052	1.423	5.025
SMURF2	0.496	0.302	1.64	0.101	1.642	0.496	1.293	0.196	0.908	2.969
SMURF1	-0.499	0.306	-1.631	0.103	0.607	0.186	-2.115	0.034	0.334	1.106
SMUG1	0.149	0.296	0.505	0.613	1.161	0.343	0.469	0.639	0.65	2.073
SMU1	-0.002	0.295	-0.006	0.995	0.998	0.295	-0.006	0.995	0.56	1.781
SMTN	-0.775	0.308	-2.515	0.012	0.461	0.142	-3.799	0	0.252	0.843
SMS	0.297	0.298	0.998	0.318	1.346	0.4	0.864	0.388	0.751	2.411
SMR3B	-0.154	0.295	-0.522	0.602	0.857	0.253	-0.564	0.572	0.48	1.529
SMR3A	0.494	0.301	1.638	0.101	1.639	0.494	1.293	0.196	0.908	2.959
SMPX	-0.07	0.296	-0.235	0.814	0.933	0.276	-0.244	0.808	0.523	1.665
SMPDL3B	0.016	0.298	0.053	0.958	1.016	0.302	0.052	0.958	0.567	1.82
SMPDL3A	-0.655	0.31	-2.114	0.034	0.52	0.161	-2.986	0.003	0.283	0.953
SMPD3	-0.021	0.297	-0.072	0.943	0.979	0.291	-0.073	0.942	0.547	1.753
SMPD2	-0.644	0.31	-2.081	0.037	0.525	0.163	-2.921	0.003	0.286	0.963
SMPD1	-0.491	0.306	-1.605	0.109	0.612	0.187	-2.072	0.038	0.336	1.115
SMOX	0.206	0.297	0.696	0.486	1.229	0.365	0.629	0.529	0.687	2.198
SMO	0.298	0.297	1.002	0.316	1.347	0.4	0.867	0.386	0.752	2.412
SMNDC1	0.155	0.296	0.525	0.599	1.168	0.345	0.487	0.627	0.654	2.084
SMIM8	0.441	0.302	1.458	0.145	1.554	0.47	1.179	0.238	0.859	2.812
SMIM7	-0.474	0.302	-1.569	0.117	0.622	0.188	-2.007	0.045	0.344	1.125
SMIM27	0.001	0.295	0.002	0.998	1.001	0.295	0.002	0.998	0.561	1.784
SMIM2	0.143	0.295	0.484	0.628	1.154	0.341	0.451	0.652	0.647	2.058
SMIM14	0.351	0.3	1.171	0.242	1.42	0.425	0.988	0.323	0.789	2.554
SMIM10L1	0.506	0.3	1.684	0.092	1.658	0.498	1.322	0.186	0.921	2.988
SMG9	-0.657	0.31	-2.12	0.034	0.519	0.161	-2.997	0.003	0.283	0.952
SMG8	0.155	0.296	0.523	0.601	1.168	0.346	0.485	0.628	0.653	2.088
SMG7-AS1	0.377	0.298	1.266	0.206	1.458	0.434	1.055	0.292	0.813	2.613
SMG7	0.141	0.299	0.473	0.636	1.152	0.344	0.441	0.659	0.641	2.069
SMG6	-0.397	0.3	-1.325	0.185	0.672	0.202	-1.626	0.104	0.374	1.21

SMG5	0.147	0.296	0.497	0.619	1.159	0.343	0.462	0.644	0.648	2.071
SMCP	0.42	0.3	1.401	0.161	1.522	0.456	1.144	0.253	0.846	2.737
SMCO4	0.083	0.295	0.28	0.779	1.086	0.321	0.269	0.788	0.609	1.938
SMCHD1	-0.595	0.306	-1.947	0.052	0.551	0.169	-2.66	0.008	0.303	1.004
SMC6	0.311	0.298	1.045	0.296	1.365	0.406	0.898	0.369	0.762	2.447
SMC5	0.039	0.295	0.133	0.895	1.04	0.307	0.13	0.897	0.583	1.855
SMC4	0.157	0.295	0.531	0.596	1.17	0.345	0.491	0.623	0.656	2.087
SMC3	0.448	0.303	1.478	0.139	1.566	0.475	1.191	0.233	0.864	2.837
SMC2	0.159	0.297	0.535	0.593	1.172	0.348	0.494	0.621	0.655	2.096
SMC1A	0.28	0.301	0.931	0.352	1.323	0.398	0.812	0.417	0.734	2.385
SMARCE1	0.81	0.311	2.608	0.009	2.248	0.698	1.788	0.074	1.223	4.132
SMARCD3	-0.244	0.297	-0.819	0.413	0.784	0.233	-0.928	0.354	0.437	1.404
SMARCD2	0.121	0.295	0.411	0.681	1.129	0.333	0.387	0.699	0.633	2.014
SMARCD1	0.203	0.296	0.687	0.492	1.226	0.363	0.622	0.534	0.686	2.19
SMARCC2	-0.162	0.296	-0.547	0.584	0.85	0.252	-0.594	0.552	0.476	1.52
SMARCC1	0.379	0.3	1.265	0.206	1.461	0.438	1.052	0.293	0.812	2.63
SMARCB1	-0.206	0.297	-0.694	0.487	0.814	0.241	-0.771	0.441	0.455	1.455
SMARCAL1	0.173	0.296	0.582	0.56	1.188	0.352	0.535	0.593	0.665	2.124
SMARCA5	-0.123	0.296	-0.416	0.678	0.884	0.262	-0.442	0.658	0.495	1.581
SMARCA4	-0.257	0.299	-0.86	0.39	0.773	0.231	-0.98	0.327	0.43	1.39
SMARCA2	-0.654	0.31	-2.11	0.035	0.52	0.161	-2.978	0.003	0.283	0.955
SMARCA1	-0.064	0.296	-0.216	0.829	0.938	0.278	-0.223	0.823	0.525	1.675
SMAP1	0.573	0.302	1.894	0.058	1.773	0.536	1.442	0.149	0.98	3.208
SMAGP	-0.125	0.296	-0.422	0.673	0.883	0.261	-0.45	0.653	0.495	1.575
SMAD9	-0.389	0.3	-1.3	0.194	0.677	0.203	-1.59	0.112	0.377	1.219
SMAD7	-0.048	0.297	-0.161	0.872	0.953	0.283	-0.164	0.869	0.533	1.706
SMAD6	0.63	0.305	2.062	0.039	1.877	0.574	1.53	0.126	1.032	3.417
SMAD5-AS1	0.017	0.297	0.058	0.954	1.017	0.302	0.057	0.954	0.568	1.822
SMAD5	0.016	0.295	0.053	0.958	1.016	0.3	0.052	0.958	0.57	1.811
SMAD4	-0.53	0.305	-1.734	0.083	0.589	0.18	-2.286	0.022	0.324	1.072
SMAD3	-0.109	0.295	-0.371	0.711	0.896	0.265	-0.392	0.695	0.502	1.599
SMAD2	-0.341	0.3	-1.137	0.255	0.711	0.213	-1.355	0.175	0.395	1.279
SMAD1	-0.524	0.306	-1.714	0.087	0.592	0.181	-2.252	0.024	0.325	1.078
SLURP1	0.088	0.296	0.297	0.767	1.092	0.323	0.284	0.776	0.612	1.948
SLTM	0.042	0.295	0.144	0.886	1.043	0.308	0.141	0.888	0.585	1.861
SLPI	0.229	0.298	0.768	0.442	1.257	0.375	0.686	0.492	0.701	2.256
SLN	-0.3	0.297	-1.009	0.313	0.741	0.22	-1.177	0.239	0.414	1.327
SLK	-0.111	0.297	-0.373	0.71	0.895	0.266	-0.394	0.694	0.5	1.603

SLITRK5	0.565	0.303	1.865	0.062	1.759	0.533	1.425	0.154	0.972	3.185
SLITRK3	-0.765	0.31	-2.464	0.014	0.465	0.144	-3.702	0	0.253	0.855
SLIT3	-0.172	0.296	-0.582	0.561	0.842	0.249	-0.635	0.526	0.471	1.504
SLIT2	-0.488	0.3	-1.629	0.103	0.614	0.184	-2.101	0.036	0.341	1.104
SLIT1	0.159	0.3	0.529	0.597	1.172	0.351	0.489	0.625	0.651	2.109
SLIRP	0.638	0.304	2.096	0.036	1.893	0.576	1.55	0.121	1.042	3.438
SLFN12	-0.499	0.3	-1.664	0.096	0.607	0.182	-2.158	0.031	0.337	1.093
SLF2	-0.467	0.301	-1.553	0.12	0.627	0.189	-1.98	0.048	0.347	1.13
SLCO5A1	0.119	0.295	0.405	0.686	1.127	0.333	0.381	0.703	0.632	2.011
SLCO4C1	0.274	0.297	0.922	0.356	1.315	0.39	0.807	0.42	0.735	2.351
SLCO4A1	0.009	0.295	0.029	0.977	1.009	0.298	0.029	0.977	0.565	1.799
SLCO3A1	-0.021	0.295	-0.073	0.942	0.979	0.289	-0.074	0.941	0.549	1.745
SLCO2B1	-0.218	0.297	-0.736	0.462	0.804	0.238	-0.822	0.411	0.449	1.438
SLCO2A1	0.058	0.295	0.198	0.843	1.06	0.313	0.192	0.848	0.594	1.891
SLCO1C1	-0.263	0.298	-0.883	0.377	0.769	0.229	-1.01	0.312	0.429	1.378
SLCO1B3	0.247	0.297	0.833	0.405	1.28	0.38	0.738	0.46	0.716	2.289
SLCO1B1	-0.099	0.295	-0.336	0.737	0.905	0.267	-0.354	0.724	0.508	1.615
SLCO1A2	-0.159	0.296	-0.537	0.591	0.853	0.253	-0.582	0.56	0.477	1.524
SLC9A8	0.348	0.3	1.162	0.245	1.416	0.424	0.981	0.327	0.787	2.548
SLC9A7	-0.237	0.296	-0.8	0.424	0.789	0.234	-0.902	0.367	0.442	1.41
SLC9A6	-0.229	0.296	-0.773	0.44	0.795	0.236	-0.868	0.385	0.445	1.422
SLC9A5	0.121	0.297	0.409	0.683	1.129	0.335	0.385	0.7	0.631	2.02
SLC9A3R2	-0.43	0.3	-1.436	0.151	0.65	0.195	-1.794	0.073	0.361	1.17
SLC9A3R1	0.394	0.298	1.324	0.185	1.483	0.442	1.094	0.274	0.828	2.659
SLC9A3	-0.624	0.31	-2.013	0.044	0.536	0.166	-2.795	0.005	0.292	0.984
SLC9A2	0.085	0.296	0.289	0.773	1.089	0.322	0.277	0.782	0.61	1.945
SLC9A1	-0.259	0.298	-0.869	0.385	0.772	0.23	-0.992	0.321	0.431	1.384
SLC8B1	-1.12	0.336	-3.333	0.001	0.326	0.11	-6.145	0	0.169	0.63
SLC8A2	0.236	0.296	0.798	0.425	1.266	0.375	0.71	0.477	0.709	2.263
SLC8A1	-0.1	0.295	-0.338	0.736	0.905	0.267	-0.355	0.723	0.507	1.615
SLC7A9	0.072	0.297	0.243	0.808	1.075	0.32	0.235	0.814	0.6	1.926
SLC7A8	-0.095	0.295	-0.323	0.747	0.909	0.268	-0.339	0.735	0.51	1.622
SLC7A7	0.218	0.296	0.738	0.461	1.244	0.368	0.663	0.508	0.697	2.221
SLC7A6	0.454	0.303	1.499	0.134	1.574	0.477	1.205	0.228	0.87	2.85
SLC7A5	0.608	0.311	1.957	0.05	1.836	0.57	1.466	0.143	0.999	3.375
SLC7A4	0.225	0.298	0.755	0.45	1.252	0.373	0.676	0.499	0.699	2.244
SLC7A2	-0.743	0.315	-2.36	0.018	0.476	0.15	-3.501	0	0.257	0.882
SLC7A11	0.024	0.295	0.08	0.936	1.024	0.302	0.079	0.937	0.574	1.825

SLC7A10	0.055	0.295	0.185	0.853	1.056	0.312	0.18	0.857	0.592	1.884
SLC7A1	0.496	0.306	1.618	0.106	1.641	0.503	1.276	0.202	0.9	2.992
SLC6A9	-0.406	0.302	-1.344	0.179	0.666	0.201	-1.658	0.097	0.368	1.205
SLC6A8	0.337	0.298	1.131	0.258	1.4	0.417	0.961	0.337	0.782	2.509
SLC6A7	0.211	0.296	0.712	0.476	1.235	0.366	0.642	0.521	0.691	2.206
SLC6A6	0.14	0.296	0.473	0.636	1.151	0.341	0.442	0.659	0.644	2.056
SLC6A5	0.139	0.296	0.47	0.639	1.149	0.341	0.438	0.661	0.643	2.055
SLC6A4	-0.552	0.303	-1.823	0.068	0.576	0.174	-2.433	0.015	0.318	1.042
SLC6A3	-0.138	0.296	-0.467	0.641	0.871	0.258	-0.501	0.617	0.487	1.557
SLC6A20	0.018	0.295	0.062	0.951	1.018	0.301	0.061	0.951	0.571	1.817
SLC6A2	-0.082	0.295	-0.278	0.781	0.921	0.272	-0.29	0.772	0.516	1.644
SLC6A16	0.257	0.298	0.865	0.387	1.294	0.385	0.763	0.446	0.722	2.318
SLC6A15	-0.351	0.3	-1.172	0.241	0.704	0.211	-1.404	0.16	0.391	1.266
SLC6A14	-0.051	0.297	-0.172	0.863	0.95	0.282	-0.177	0.86	0.531	1.7
SLC6A13	0.243	0.298	0.814	0.415	1.274	0.38	0.723	0.47	0.711	2.285
SLC6A12	-0.01	0.295	-0.034	0.973	0.99	0.292	-0.034	0.973	0.555	1.767
SLC6A11	-0.178	0.296	-0.601	0.548	0.837	0.248	-0.658	0.51	0.469	1.495
SLC6A1	-0.247	0.298	-0.829	0.407	0.781	0.233	-0.94	0.347	0.436	1.4
SLC66A3	0.133	0.295	0.449	0.653	1.142	0.337	0.421	0.674	0.64	2.037
SLC66A2	-0.024	0.298	-0.08	0.936	0.976	0.291	-0.081	0.936	0.545	1.751
SLC66A1	-0.116	0.295	-0.392	0.695	0.891	0.263	-0.415	0.678	0.499	1.589
SLC5A7	0.343	0.298	1.152	0.249	1.409	0.42	0.975	0.329	0.786	2.527
SLC5A6	-0.037	0.296	-0.125	0.9	0.964	0.285	-0.128	0.898	0.539	1.721
SLC5A5	-0.538	0.303	-1.778	0.075	0.584	0.177	-2.355	0.019	0.323	1.057
SLC5A4	-0.113	0.295	-0.383	0.702	0.893	0.264	-0.405	0.685	0.501	1.593
SLC5A3	-0.291	0.297	-0.977	0.328	0.748	0.222	-1.134	0.257	0.417	1.339
SLC5A2	-0.438	0.301	-1.457	0.145	0.645	0.194	-1.829	0.067	0.358	1.163
SLC5A12	-0.105	0.296	-0.355	0.722	0.9	0.267	-0.375	0.708	0.504	1.608
SLC5A1	0.325	0.3	1.085	0.278	1.384	0.415	0.926	0.354	0.769	2.489
SLC52A2	0.891	0.321	2.78	0.005	2.438	0.782	1.84	0.066	1.301	4.571
SLC52A1	0.267	0.297	0.902	0.367	1.306	0.387	0.791	0.429	0.731	2.336
SLC50A1	-0.147	0.298	-0.492	0.622	0.864	0.257	-0.53	0.596	0.481	1.549
SLC4A8	-0.352	0.3	-1.175	0.24	0.703	0.211	-1.408	0.159	0.391	1.265
SLC4A7	-0.586	0.304	-1.93	0.054	0.557	0.169	-2.624	0.009	0.307	1.009
SLC4A5	0.332	0.298	1.117	0.264	1.394	0.415	0.95	0.342	0.778	2.498
SLC4A4	-0.03	0.295	-0.102	0.919	0.97	0.287	-0.104	0.917	0.544	1.731
SLC4A3	0.354	0.298	1.191	0.234	1.425	0.424	1.003	0.316	0.795	2.555
SLC4A2	-0.457	0.302	-1.511	0.131	0.633	0.191	-1.916	0.055	0.35	1.145

SLC4A1AP	0.507	0.302	1.678	0.093	1.661	0.502	1.316	0.188	0.918	3.005
SLC4A10	-0.179	0.295	-0.608	0.543	0.836	0.247	-0.666	0.506	0.468	1.491
SLC4A1	0.257	0.296	0.867	0.386	1.293	0.383	0.764	0.445	0.723	2.311
SLC49A3	-0.21	0.299	-0.704	0.482	0.811	0.242	-0.783	0.434	0.451	1.455
SLC48A1	-0.635	0.304	-2.092	0.036	0.53	0.161	-2.923	0.003	0.292	0.961
SLC47A1	-0.292	0.298	-0.982	0.326	0.747	0.222	-1.14	0.254	0.417	1.338
SLC46A3	0.559	0.303	1.846	0.065	1.749	0.53	1.414	0.157	0.966	3.166
SLC45A2	-0.472	0.302	-1.561	0.118	0.624	0.189	-1.995	0.046	0.345	1.128
SLC44A4	-0.472	0.302	-1.563	0.118	0.623	0.188	-1.998	0.046	0.345	1.127
SLC44A1	0.316	0.297	1.065	0.287	1.372	0.407	0.913	0.361	0.767	2.454
SLC43A3	0.02	0.297	0.067	0.947	1.02	0.303	0.066	0.947	0.57	1.825
SLC43A1	-0.137	0.296	-0.465	0.642	0.872	0.258	-0.498	0.618	0.488	1.556
SLC41A3	0.242	0.296	0.816	0.414	1.274	0.377	0.725	0.468	0.713	2.276
SLC3A2	0.437	0.298	1.465	0.143	1.548	0.461	1.187	0.235	0.863	2.776
SLC3A1	0.227	0.296	0.766	0.444	1.255	0.372	0.685	0.493	0.702	2.242
SLC39A9	-0.252	0.298	-0.847	0.397	0.777	0.231	-0.964	0.335	0.434	1.393
SLC39A8	0.393	0.3	1.312	0.189	1.482	0.444	1.085	0.278	0.824	2.666
SLC39A7	0.728	0.311	2.343	0.019	2.071	0.643	1.664	0.096	1.126	3.807
SLC39A6	-0.374	0.3	-1.248	0.212	0.688	0.206	-1.514	0.13	0.382	1.238
SLC39A4	0.296	0.296	1	0.317	1.345	0.399	0.865	0.387	0.752	2.405
SLC39A2	0.031	0.295	0.107	0.915	1.032	0.304	0.105	0.916	0.579	1.84
SLC39A14	0.015	0.296	0.049	0.961	1.015	0.3	0.049	0.961	0.568	1.811
SLC39A1	-0.437	0.302	-1.447	0.148	0.646	0.195	-1.815	0.069	0.357	1.168
SLC38A7	0.392	0.3	1.307	0.191	1.48	0.443	1.082	0.279	0.822	2.662
SLC38A6	-0.425	0.302	-1.407	0.159	0.653	0.198	-1.754	0.079	0.361	1.182
SLC38A4	-0.73	0.31	-2.354	0.019	0.482	0.149	-3.466	0.001	0.263	0.885
SLC38A3	0.119	0.299	0.398	0.691	1.126	0.337	0.375	0.707	0.627	2.024
SLC38A2	-0.47	0.301	-1.561	0.119	0.625	0.188	-1.992	0.046	0.347	1.128
SLC38A10	-0.446	0.3	-1.488	0.137	0.64	0.192	-1.876	0.061	0.356	1.152
SLC38A1	-0.453	0.301	-1.507	0.132	0.636	0.191	-1.907	0.057	0.353	1.146
SLC37A4	0.368	0.298	1.238	0.216	1.446	0.43	1.036	0.3	0.807	2.59
SLC37A1	0.004	0.296	0.015	0.988	1.004	0.298	0.015	0.988	0.562	1.795
SLC36A1	0.426	0.3	1.423	0.155	1.531	0.459	1.159	0.247	0.851	2.755
SLC35G2	0.12	0.295	0.407	0.684	1.128	0.333	0.384	0.701	0.632	2.011
SLC35F6	0.316	0.3	1.055	0.291	1.372	0.411	0.904	0.366	0.763	2.467
SLC35F5	-0.207	0.298	-0.695	0.487	0.813	0.242	-0.772	0.44	0.453	1.458
SLC35F2	0.063	0.295	0.215	0.83	1.065	0.314	0.208	0.835	0.598	1.9
SLC35E3	0.342	0.298	1.148	0.251	1.408	0.42	0.972	0.331	0.785	2.526

SLC35E1	0.234	0.301	0.776	0.437	1.263	0.381	0.692	0.489	0.7	2.28
SLC35D2	-0.622	0.306	-2.032	0.042	0.537	0.164	-2.818	0.005	0.295	0.978
SLC35D1	-0.453	0.302	-1.498	0.134	0.636	0.192	-1.895	0.058	0.352	1.15
SLC35C2	0.473	0.302	1.563	0.118	1.604	0.485	1.245	0.213	0.887	2.901
SLC35C1	0.211	0.298	0.708	0.479	1.235	0.369	0.639	0.523	0.688	2.217
SLC35B1	-0.259	0.298	-0.87	0.384	0.772	0.23	-0.994	0.32	0.43	1.383
SLC35A5	-0.125	0.296	-0.421	0.674	0.883	0.262	-0.448	0.654	0.494	1.578
SLC35A3	0.115	0.296	0.387	0.699	1.122	0.332	0.366	0.714	0.628	2.004
SLC35A2	0.515	0.303	1.701	0.089	1.673	0.506	1.329	0.184	0.925	3.027
SLC35A1	0.286	0.298	0.961	0.337	1.331	0.396	0.836	0.403	0.743	2.386
SLC34A2	-0.109	0.295	-0.371	0.711	0.896	0.265	-0.392	0.695	0.502	1.599
SLC34A1	-0.255	0.296	-0.86	0.39	0.775	0.23	-0.98	0.327	0.433	1.385
SLC33A1	0.341	0.3	1.135	0.256	1.406	0.422	0.962	0.336	0.781	2.533
SLC31A2	-0.2	0.296	-0.675	0.5	0.819	0.243	-0.747	0.455	0.458	1.464
SLC31A1	0.536	0.302	1.772	0.076	1.709	0.517	1.372	0.17	0.945	3.091
SLC30A9	-0.086	0.297	-0.288	0.773	0.918	0.272	-0.301	0.763	0.513	1.642
SLC30A6	-0.054	0.295	-0.184	0.854	0.947	0.28	-0.189	0.85	0.531	1.69
SLC30A5	-0.126	0.298	-0.424	0.672	0.881	0.263	-0.452	0.651	0.491	1.581
SLC30A4	-0.084	0.295	-0.283	0.777	0.92	0.272	-0.295	0.768	0.516	1.641
SLC30A3	0.242	0.296	0.817	0.414	1.274	0.377	0.725	0.468	0.713	2.276
SLC30A10	0.076	0.297	0.256	0.798	1.079	0.32	0.246	0.805	0.603	1.929
SLC30A1	-0.485	0.302	-1.605	0.108	0.615	0.186	-2.066	0.039	0.34	1.113
SLC2A9	0.24	0.296	0.811	0.417	1.272	0.377	0.721	0.471	0.712	2.273
SLC2A8	0.222	0.298	0.743	0.457	1.248	0.373	0.667	0.505	0.696	2.241
SLC2A6	0.604	0.303	1.993	0.046	1.83	0.555	1.496	0.135	1.01	3.315
SLC2A5	-0.143	0.297	-0.483	0.629	0.867	0.257	-0.519	0.604	0.484	1.55
SLC2A4RG	0.329	0.298	1.105	0.269	1.389	0.413	0.942	0.346	0.775	2.489
SLC2A4	0.006	0.295	0.02	0.984	1.006	0.297	0.02	0.984	0.564	1.794
SLC2A3	-0.129	0.296	-0.435	0.664	0.879	0.26	-0.464	0.643	0.493	1.569
SLC2A2	0.269	0.297	0.906	0.365	1.309	0.389	0.794	0.427	0.731	2.345
SLC2A11	-0.071	0.298	-0.238	0.812	0.932	0.278	-0.247	0.805	0.52	1.67
SLC2A10	0.048	0.295	0.162	0.871	1.049	0.31	0.158	0.874	0.588	1.871
SLC2A1	-0.009	0.296	-0.029	0.977	0.991	0.293	-0.03	0.976	0.555	1.771
SLC29A3	-0.504	0.306	-1.648	0.099	0.604	0.185	-2.142	0.032	0.332	1.1
SLC29A2	0.208	0.296	0.702	0.483	1.231	0.365	0.634	0.526	0.689	2.2
SLC29A1	-0.076	0.295	-0.259	0.796	0.926	0.274	-0.269	0.788	0.519	1.653
SLC28A3	0.081	0.297	0.273	0.785	1.084	0.322	0.262	0.793	0.606	1.941
SLC28A2	-0.207	0.296	-0.7	0.484	0.813	0.241	-0.778	0.437	0.455	1.452

SLC28A1	0.24	0.296	0.812	0.417	1.272	0.377	0.722	0.47	0.712	2.273
SLC27A6	0.083	0.295	0.283	0.777	1.087	0.321	0.271	0.786	0.61	1.938
SLC27A5	0.071	0.295	0.24	0.81	1.074	0.317	0.232	0.817	0.602	1.915
SLC27A3	0.146	0.297	0.491	0.623	1.157	0.343	0.457	0.647	0.647	2.069
SLC27A2	-0.295	0.3	-0.983	0.326	0.745	0.224	-1.143	0.253	0.413	1.341
SLC26A4	0.214	0.296	0.723	0.47	1.239	0.367	0.651	0.515	0.693	2.214
SLC26A3	-0.676	0.31	-2.184	0.029	0.508	0.158	-3.121	0.002	0.277	0.933
SLC26A2	0.674	0.306	2.202	0.028	1.963	0.601	1.602	0.109	1.077	3.577
SLC26A10	0.016	0.295	0.053	0.958	1.016	0.3	0.052	0.958	0.57	1.811
SLC26A1	0.438	0.3	1.462	0.144	1.55	0.465	1.183	0.237	0.861	2.789
SLC25A6	-0.381	0.3	-1.27	0.204	0.683	0.205	-1.546	0.122	0.38	1.23
SLC25A5	0.693	0.306	2.266	0.023	2	0.612	1.635	0.102	1.098	3.642
SLC25A46	0.72	0.306	2.353	0.019	2.054	0.628	1.677	0.093	1.128	3.741
SLC25A44	0.182	0.296	0.616	0.538	1.2	0.355	0.563	0.574	0.672	2.143
SLC25A42	0.567	0.303	1.874	0.061	1.763	0.533	1.43	0.153	0.974	3.19
SLC25A40	-0.067	0.297	-0.226	0.821	0.935	0.278	-0.234	0.815	0.522	1.673
SLC25A4	0.509	0.301	1.693	0.091	1.663	0.5	1.327	0.185	0.923	2.998
SLC25A38	-0.022	0.296	-0.074	0.941	0.978	0.289	-0.075	0.94	0.548	1.747
SLC25A37	0.026	0.296	0.088	0.93	1.027	0.304	0.087	0.93	0.574	1.835
SLC25A36	-0.393	0.298	-1.318	0.187	0.675	0.201	-1.615	0.106	0.376	1.211
SLC25A32	0.336	0.298	1.13	0.259	1.4	0.416	0.96	0.337	0.781	2.508
SLC25A31	-0.363	0.3	-1.212	0.226	0.696	0.208	-1.461	0.144	0.387	1.251
SLC25A3	-0.368	0.3	-1.228	0.22	0.692	0.208	-1.484	0.138	0.384	1.246
SLC25A28	-0.192	0.298	-0.644	0.519	0.825	0.246	-0.71	0.477	0.46	1.48
SLC25A24	0.14	0.295	0.473	0.636	1.15	0.34	0.441	0.659	0.644	2.052
SLC25A23	-0.213	0.298	-0.714	0.475	0.808	0.241	-0.796	0.426	0.451	1.45
SLC25A22	0.267	0.298	0.897	0.37	1.306	0.389	0.787	0.431	0.729	2.342
SLC25A21	-0.073	0.295	-0.246	0.805	0.93	0.275	-0.256	0.798	0.521	1.659
SLC25A20	0.268	0.298	0.9	0.368	1.307	0.389	0.79	0.43	0.729	2.343
SLC25A17	0.256	0.296	0.864	0.388	1.291	0.382	0.762	0.446	0.723	2.307
SLC25A16	0.745	0.31	2.405	0.016	2.107	0.653	1.696	0.09	1.148	3.867
SLC25A15	0.599	0.306	1.959	0.05	1.82	0.556	1.474	0.14	1	3.314
SLC25A14	0.55	0.3	1.831	0.067	1.733	0.52	1.409	0.159	0.962	3.121
SLC25A13	0.205	0.296	0.693	0.488	1.228	0.364	0.627	0.531	0.687	2.196
SLC25A12	-0.256	0.301	-0.851	0.395	0.774	0.233	-0.969	0.332	0.43	1.396
SLC25A11	-0.272	0.3	-0.908	0.364	0.762	0.228	-1.043	0.297	0.423	1.371
SLC25A10	0.197	0.296	0.666	0.506	1.218	0.361	0.604	0.546	0.682	2.176
SLC25A1	0.248	0.296	0.839	0.402	1.282	0.38	0.743	0.458	0.717	2.291

SLC24A3	-0.008	0.295	-0.027	0.979	0.992	0.293	-0.027	0.978	0.556	1.769
SLC24A2	-0.116	0.296	-0.392	0.695	0.891	0.263	-0.416	0.678	0.499	1.59
SLC24A1	0.176	0.296	0.594	0.552	1.192	0.353	0.545	0.586	0.667	2.131
SLC23A2	-0.599	0.303	-1.978	0.048	0.549	0.166	-2.709	0.007	0.303	0.995
SLC22A8	0.112	0.296	0.38	0.704	1.119	0.331	0.36	0.719	0.627	1.997
SLC22A7	-0.275	0.298	-0.921	0.357	0.76	0.227	-1.059	0.289	0.424	1.363
SLC22A6	0.36	0.3	1.201	0.23	1.433	0.43	1.009	0.313	0.797	2.579
SLC22A5	0.035	0.295	0.117	0.907	1.035	0.305	0.115	0.908	0.581	1.846
SLC22A4	-0.229	0.301	-0.763	0.445	0.795	0.239	-0.858	0.391	0.441	1.433
SLC22A3	0.223	0.296	0.751	0.453	1.249	0.37	0.673	0.501	0.699	2.232
SLC22A2	-0.005	0.296	-0.016	0.987	0.995	0.294	-0.016	0.987	0.557	1.777
SLC22A18AS	-0.058	0.295	-0.196	0.845	0.944	0.279	-0.202	0.84	0.529	1.684
SLC22A18	-0.211	0.296	-0.712	0.476	0.81	0.24	-0.793	0.428	0.453	1.447
SLC22A17	0.162	0.296	0.546	0.585	1.176	0.348	0.504	0.614	0.658	2.101
SLC22A14	0.189	0.296	0.64	0.522	1.208	0.357	0.583	0.56	0.677	2.158
SLC22A13	0.334	0.297	1.127	0.26	1.397	0.414	0.958	0.338	0.781	2.499
SLC22A11	-0.137	0.296	-0.461	0.645	0.872	0.258	-0.494	0.621	0.488	1.559
SLC22A1	-0.11	0.295	-0.372	0.71	0.896	0.265	-0.393	0.694	0.502	1.598
SLC20A2	-0.769	0.315	-2.444	0.015	0.463	0.146	-3.679	0	0.25	0.859
SLC20A1	0.38	0.3	1.267	0.205	1.462	0.438	1.054	0.292	0.812	2.63
SLC1A7	-0.213	0.296	-0.72	0.471	0.808	0.239	-0.803	0.422	0.452	1.444
SLC1A6	0.027	0.297	0.089	0.929	1.027	0.305	0.088	0.93	0.574	1.837
SLC1A5	-0.417	0.3	-1.387	0.165	0.659	0.198	-1.72	0.085	0.366	1.188
SLC1A4	-0.225	0.298	-0.755	0.45	0.799	0.238	-0.847	0.397	0.445	1.432
SLC1A3	0.214	0.297	0.722	0.471	1.239	0.367	0.65	0.516	0.693	2.215
SLC1A2	-0.149	0.296	-0.502	0.616	0.862	0.255	-0.541	0.588	0.482	1.54
SLC1A1	-0.451	0.3	-1.504	0.133	0.637	0.191	-1.9	0.057	0.354	1.147
SLC19A3	0.099	0.295	0.335	0.738	1.104	0.326	0.319	0.75	0.619	1.968
SLC19A2	-0.298	0.3	-0.993	0.321	0.742	0.223	-1.156	0.248	0.412	1.337
SLC19A1	0.836	0.311	2.691	0.007	2.307	0.717	1.823	0.068	1.255	4.243
SLC18A3	0.454	0.3	1.517	0.129	1.575	0.472	1.219	0.223	0.876	2.834
SLC18A2	-0.228	0.298	-0.767	0.443	0.796	0.237	-0.861	0.389	0.444	1.426
SLC18A1	-0.201	0.297	-0.677	0.499	0.818	0.243	-0.75	0.454	0.457	1.463
SLC17A9	0.067	0.296	0.227	0.821	1.069	0.316	0.219	0.827	0.599	1.91
SLC17A7	-0.061	0.296	-0.206	0.837	0.941	0.279	-0.212	0.832	0.527	1.681
SLC17A6	0.535	0.3	1.781	0.075	1.708	0.513	1.38	0.168	0.948	3.077
SLC17A5	0.377	0.3	1.258	0.208	1.458	0.437	1.048	0.295	0.81	2.622
SLC17A4	-0.318	0.298	-1.068	0.286	0.727	0.217	-1.257	0.209	0.405	1.305

SLC17A3	0.358	0.3	1.194	0.233	1.43	0.428	1.004	0.316	0.795	2.573
SLC17A2	0.687	0.306	2.242	0.025	1.987	0.609	1.622	0.105	1.09	3.622
SLC17A1	-0.033	0.296	-0.113	0.91	0.967	0.286	-0.115	0.909	0.542	1.727
SLC16A8	0.481	0.3	1.606	0.108	1.618	0.485	1.275	0.202	0.899	2.913
SLC16A7	-0.08	0.295	-0.271	0.787	0.923	0.273	-0.282	0.778	0.518	1.647
SLC16A6	-0.278	0.304	-0.913	0.361	0.758	0.23	-1.052	0.293	0.418	1.375
SLC16A5	-0.538	0.302	-1.781	0.075	0.584	0.176	-2.36	0.018	0.323	1.056
SLC16A4	-0.795	0.31	-2.559	0.01	0.452	0.14	-3.908	0	0.246	0.83
SLC16A2	-0.132	0.297	-0.444	0.657	0.877	0.26	-0.474	0.635	0.49	1.568
SLC16A10	1.101	0.328	3.356	0.001	3.006	0.986	2.035	0.042	1.581	5.717
SLC16A1	-0.089	0.296	-0.3	0.765	0.915	0.271	-0.313	0.754	0.513	1.634
SLC15A3	-0.012	0.295	-0.04	0.968	0.988	0.292	-0.041	0.968	0.554	1.762
SLC15A2	0.053	0.298	0.179	0.858	1.055	0.314	0.174	0.862	0.588	1.891
SLC15A1	0.639	0.31	2.062	0.039	1.894	0.586	1.524	0.127	1.032	3.475
SLC14A2	-0.146	0.298	-0.489	0.625	0.864	0.257	-0.527	0.598	0.482	1.549
SLC14A1	-0.326	0.3	-1.088	0.277	0.722	0.216	-1.286	0.199	0.401	1.299
SLC13A4	0.034	0.297	0.115	0.908	1.035	0.307	0.113	0.91	0.579	1.851
SLC13A3	-0.091	0.295	-0.309	0.758	0.913	0.269	-0.323	0.747	0.512	1.628
SLC13A2	-0.181	0.296	-0.61	0.542	0.835	0.247	-0.669	0.503	0.467	1.491
SLC13A1	0.455	0.302	1.506	0.132	1.577	0.477	1.21	0.226	0.872	2.851
SLC12A9	-0.042	0.296	-0.142	0.887	0.959	0.284	-0.145	0.885	0.537	1.713
SLC12A8	0.497	0.306	1.627	0.104	1.644	0.503	1.282	0.2	0.903	2.994
SLC12A7	-0.304	0.299	-1.018	0.309	0.738	0.22	-1.19	0.234	0.411	1.325
SLC12A6	0.015	0.295	0.051	0.959	1.015	0.3	0.05	0.96	0.569	1.81
SLC12A5	-0.038	0.295	-0.13	0.897	0.962	0.284	-0.132	0.895	0.539	1.717
SLC12A4	0.024	0.295	0.082	0.935	1.025	0.302	0.081	0.935	0.575	1.827
SLC12A3	-0.062	0.296	-0.21	0.834	0.94	0.278	-0.216	0.829	0.526	1.679
SLC12A2	-0.654	0.306	-2.139	0.032	0.52	0.159	-3.02	0.003	0.285	0.947
SLC12A1	0.229	0.297	0.772	0.44	1.257	0.373	0.69	0.49	0.703	2.248
SLC11A2	-0.258	0.296	-0.871	0.384	0.772	0.229	-0.994	0.32	0.432	1.381
SLC11A1	0	0.296	0.001	0.999	1	0.296	0.001	0.999	0.56	1.786
SLC10A3	0.119	0.295	0.403	0.687	1.126	0.333	0.38	0.704	0.631	2.01
SLC10A2	0.193	0.296	0.653	0.514	1.213	0.359	0.594	0.553	0.679	2.168
SLC10A1	0.018	0.295	0.06	0.952	1.018	0.3	0.059	0.953	0.571	1.815
SLBP	0.561	0.302	1.855	0.064	1.752	0.53	1.42	0.156	0.969	3.169
SLAMF8	0.303	0.298	1.017	0.309	1.354	0.404	0.878	0.38	0.755	2.429
SLAMF7	-0.027	0.295	-0.093	0.926	0.973	0.287	-0.094	0.925	0.546	1.735
SLAMF1	-0.246	0.297	-0.83	0.406	0.782	0.232	-0.941	0.347	0.437	1.398

SLA	0.038	0.295	0.129	0.898	1.039	0.307	0.126	0.9	0.582	1.853
SKP2	0.567	0.306	1.856	0.064	1.764	0.539	1.416	0.157	0.969	3.211
SKP1	0.33	0.298	1.108	0.268	1.391	0.414	0.944	0.345	0.776	2.494
SKIV2L	0.102	0.296	0.346	0.73	1.108	0.328	0.329	0.742	0.62	1.977
SKIL	0.261	0.298	0.876	0.381	1.298	0.386	0.771	0.441	0.724	2.325
SKI	0.254	0.298	0.853	0.394	1.289	0.384	0.753	0.451	0.719	2.31
SKAP2	-0.183	0.296	-0.62	0.536	0.832	0.246	-0.68	0.496	0.466	1.487
SKAP1	0.132	0.295	0.448	0.654	1.141	0.337	0.419	0.675	0.64	2.036
SKA1	0.78	0.306	2.546	0.011	2.181	0.668	1.768	0.077	1.197	3.976
SIX6	0.111	0.295	0.375	0.707	1.117	0.33	0.355	0.722	0.626	1.993
SIX5	-0.091	0.295	-0.308	0.758	0.913	0.27	-0.323	0.747	0.512	1.629
SIX3	0.125	0.296	0.42	0.674	1.133	0.336	0.395	0.693	0.634	2.025
SIX2	0.045	0.295	0.153	0.879	1.046	0.309	0.149	0.881	0.587	1.866
SIX1	0.282	0.298	0.949	0.343	1.326	0.395	0.827	0.408	0.74	2.377
SIVA1	0.484	0.302	1.604	0.109	1.622	0.489	1.272	0.203	0.898	2.93
SIT1	-0.019	0.295	-0.064	0.949	0.981	0.29	-0.065	0.948	0.55	1.751
SIRT7	0.144	0.296	0.486	0.627	1.155	0.342	0.452	0.651	0.646	2.064
SIRT6	-0.45	0.301	-1.494	0.135	0.638	0.192	-1.887	0.059	0.354	1.151
SIRT5	0.968	0.317	3.058	0.002	2.634	0.834	1.959	0.05	1.416	4.899
SIRT4	-0.492	0.302	-1.627	0.104	0.611	0.185	-2.102	0.036	0.338	1.106
SIRT3	-0.146	0.296	-0.495	0.621	0.864	0.255	-0.533	0.594	0.484	1.542
SIRT2	0.281	0.3	0.937	0.349	1.325	0.397	0.817	0.414	0.736	2.384
SIRT1	0.16	0.295	0.543	0.587	1.174	0.347	0.502	0.616	0.658	2.095
SIRPG	0.669	0.31	2.159	0.031	1.951	0.604	1.574	0.115	1.063	3.581
SIRPB1	-0.496	0.302	-1.641	0.101	0.609	0.184	-2.124	0.034	0.337	1.101
SIRPA	-0.351	0.299	-1.173	0.241	0.704	0.211	-1.405	0.16	0.392	1.265
SIPA1L3	-0.005	0.295	-0.017	0.986	0.995	0.294	-0.017	0.986	0.558	1.775
SIPA1L1	0.223	0.296	0.752	0.452	1.249	0.37	0.674	0.5	0.699	2.232
SIPA1	0.367	0.298	1.229	0.219	1.443	0.431	1.029	0.304	0.804	2.59
SINHCAF	-0.259	0.297	-0.873	0.383	0.772	0.229	-0.996	0.319	0.432	1.38
SIN3B	-0.397	0.3	-1.324	0.185	0.672	0.202	-1.626	0.104	0.374	1.21
SIM2	0.091	0.295	0.308	0.758	1.095	0.324	0.294	0.768	0.614	1.955
SIM1	-0.017	0.295	-0.059	0.953	0.983	0.29	-0.06	0.952	0.551	1.752
SIL1	0.366	0.299	1.224	0.221	1.441	0.43	1.026	0.305	0.803	2.588
SIKE1	0.403	0.3	1.345	0.179	1.497	0.449	1.107	0.268	0.832	2.693
SIK3	-0.455	0.302	-1.506	0.132	0.634	0.192	-1.908	0.056	0.351	1.147
SIK2	-0.268	0.296	-0.905	0.365	0.765	0.227	-1.038	0.299	0.428	1.367
SIGMAR1	-0.131	0.296	-0.442	0.658	0.877	0.26	-0.472	0.637	0.491	1.568

SIGLEC9	0.014	0.295	0.047	0.963	1.014	0.3	0.047	0.963	0.568	1.809
SIGLEC8	-0.5	0.302	-1.654	0.098	0.607	0.183	-2.146	0.032	0.335	1.097
SIGLEC7	0.161	0.296	0.544	0.586	1.175	0.348	0.503	0.615	0.657	2.101
SIGLEC6	-0.106	0.296	-0.36	0.719	0.899	0.266	-0.38	0.704	0.503	1.605
SIGLEC5	-0.467	0.302	-1.545	0.122	0.627	0.19	-1.969	0.049	0.346	1.134
SIGLEC15	-0.429	0.302	-1.418	0.156	0.651	0.197	-1.771	0.077	0.36	1.178
SIGLEC1	0.502	0.303	1.658	0.097	1.652	0.5	1.303	0.192	0.913	2.99
SIGIRR	-0.342	0.303	-1.13	0.259	0.71	0.215	-1.347	0.178	0.392	1.286
SIDT2	-0.113	0.296	-0.383	0.702	0.893	0.264	-0.405	0.685	0.5	1.594
SIDT1	-0.467	0.3	-1.559	0.119	0.627	0.188	-1.988	0.047	0.348	1.128
SIAH2	-0.354	0.3	-1.179	0.238	0.702	0.211	-1.414	0.157	0.39	1.264
SIAH1	-0.034	0.297	-0.115	0.909	0.967	0.287	-0.117	0.907	0.54	1.729
SI	0.165	0.296	0.557	0.577	1.179	0.349	0.514	0.608	0.66	2.108
SHTN1	-0.248	0.298	-0.833	0.405	0.78	0.232	-0.946	0.344	0.435	1.398
SHROOM2	0.34	0.298	1.143	0.253	1.405	0.418	0.969	0.333	0.784	2.519
SHQ1	-0.158	0.299	-0.53	0.596	0.854	0.255	-0.574	0.566	0.475	1.533
SHPK	0.176	0.296	0.594	0.553	1.192	0.353	0.544	0.586	0.667	2.131
SHOX2	-0.294	0.297	-0.988	0.323	0.746	0.222	-1.148	0.251	0.416	1.335
SHOX	0.028	0.295	0.094	0.925	1.028	0.304	0.093	0.926	0.576	1.834
SHOC2	0.253	0.296	0.855	0.393	1.288	0.382	0.755	0.45	0.721	2.302
SHMT2	0.711	0.31	2.295	0.022	2.036	0.631	1.642	0.101	1.109	3.736
SHMT1	0.098	0.296	0.333	0.739	1.103	0.326	0.317	0.751	0.618	1.969
SHLD2	0.188	0.296	0.634	0.526	1.207	0.358	0.578	0.563	0.675	2.157
SHISAL1	-0.676	0.306	-2.209	0.027	0.509	0.156	-3.157	0.002	0.279	0.927
SHISA6	0.208	0.296	0.703	0.482	1.231	0.365	0.634	0.526	0.689	2.2
SHH	-0.605	0.305	-1.985	0.047	0.546	0.166	-2.728	0.006	0.301	0.992
SHFL	-0.293	0.296	-0.989	0.323	0.746	0.221	-1.149	0.251	0.417	1.334
SHCBP1L	-0.192	0.298	-0.645	0.519	0.825	0.246	-0.712	0.477	0.461	1.479
SHCBP1	0.862	0.315	2.738	0.006	2.368	0.746	1.835	0.067	1.278	4.39
SHC3	0.324	0.298	1.087	0.277	1.382	0.411	0.929	0.353	0.771	2.477
SHC2	0.004	0.296	0.015	0.988	1.004	0.297	0.015	0.988	0.563	1.792
SHC1	-0.324	0.298	-1.089	0.276	0.723	0.215	-1.286	0.198	0.403	1.296
SHBG	0.057	0.295	0.192	0.848	1.058	0.313	0.187	0.852	0.593	1.888
SHB	0.661	0.306	2.16	0.031	1.937	0.593	1.58	0.114	1.063	3.528
SHARPIN	-0.133	0.297	-0.447	0.655	0.876	0.26	-0.478	0.633	0.489	1.567
SHANK2	0.228	0.298	0.767	0.443	1.257	0.374	0.686	0.493	0.701	2.252
SHANK1	-0.067	0.295	-0.228	0.82	0.935	0.276	-0.236	0.814	0.524	1.668
SH3YL1	-0.233	0.296	-0.787	0.431	0.792	0.234	-0.887	0.375	0.443	1.415

SH3TC2	-0.118	0.296	-0.399	0.69	0.889	0.263	-0.424	0.672	0.498	1.586
SH3TC1	-0.592	0.303	-1.952	0.051	0.553	0.168	-2.663	0.008	0.305	1.002
SH3PXD2A	-0.131	0.296	-0.441	0.659	0.878	0.26	-0.471	0.637	0.491	1.567
SH3GLB2	-0.389	0.303	-1.284	0.199	0.678	0.205	-1.57	0.116	0.374	1.227
SH3GLB1	-0.363	0.307	-1.185	0.236	0.695	0.213	-1.429	0.153	0.381	1.268
SH3GL3	-0.388	0.298	-1.3	0.193	0.678	0.202	-1.589	0.112	0.378	1.217
SH3GL2	-0.137	0.296	-0.461	0.645	0.872	0.258	-0.494	0.621	0.488	1.559
SH3GL1	0.038	0.296	0.128	0.898	1.038	0.307	0.125	0.9	0.582	1.854
SH3D21	-0.243	0.301	-0.807	0.42	0.785	0.236	-0.913	0.361	0.435	1.414
SH3BP5	0.009	0.297	0.03	0.976	1.009	0.3	0.03	0.976	0.564	1.806
SH3BP4	-0.236	0.298	-0.791	0.429	0.79	0.235	-0.892	0.372	0.441	1.416
SH3BP2	0.34	0.298	1.143	0.253	1.405	0.418	0.969	0.333	0.784	2.518
SH3BP1	-0.505	0.306	-1.652	0.099	0.604	0.184	-2.149	0.032	0.332	1.099
SH3BGRL3	-0.816	0.315	-2.588	0.01	0.442	0.139	-4.001	0	0.238	0.82
SH3BGRL	0.085	0.296	0.286	0.775	1.088	0.322	0.274	0.784	0.61	1.943
SH3BGR	0.704	0.307	2.291	0.022	2.022	0.621	1.645	0.1	1.107	3.692
SH2D4A	-0.005	0.295	-0.017	0.986	0.995	0.293	-0.017	0.986	0.558	1.774
SH2D3C	-0.025	0.296	-0.086	0.932	0.975	0.289	-0.087	0.931	0.545	1.743
SH2D3A	0.295	0.297	0.993	0.32	1.343	0.399	0.86	0.39	0.751	2.403
SH2D2A	0.068	0.297	0.228	0.82	1.07	0.318	0.22	0.826	0.598	1.915
SH2D1A	-0.231	0.297	-0.777	0.437	0.794	0.236	-0.874	0.382	0.444	1.421
SH2B3	-0.347	0.298	-1.165	0.244	0.707	0.211	-1.393	0.164	0.394	1.267
SH2B2	0.026	0.296	0.088	0.93	1.026	0.303	0.087	0.931	0.575	1.832
SH2B1	-0.414	0.299	-1.386	0.166	0.661	0.197	-1.718	0.086	0.368	1.187
SGTA	0.266	0.296	0.899	0.369	1.305	0.387	0.789	0.43	0.73	2.333
SGSM3	0.32	0.3	1.068	0.286	1.377	0.413	0.914	0.361	0.765	2.479
SGSM2	-0.043	0.295	-0.145	0.885	0.958	0.283	-0.148	0.882	0.537	1.709
SGSH	-0.836	0.315	-2.654	0.008	0.434	0.137	-4.148	0	0.234	0.804
SGPP1	-0.29	0.297	-0.975	0.33	0.748	0.222	-1.131	0.258	0.418	1.34
SGPL1	-0.13	0.296	-0.441	0.659	0.878	0.26	-0.471	0.638	0.492	1.567
SGMS1	-0.067	0.295	-0.227	0.821	0.935	0.276	-0.234	0.815	0.524	1.669
SGK2	0.417	0.298	1.398	0.162	1.518	0.453	1.143	0.253	0.846	2.723
SGK1	-0.517	0.303	-1.707	0.088	0.597	0.181	-2.235	0.025	0.33	1.08
SGF29	0.253	0.297	0.854	0.393	1.288	0.382	0.754	0.451	0.72	2.304
SGCG	-0.04	0.295	-0.137	0.891	0.96	0.284	-0.14	0.889	0.538	1.713
SGCE	-0.077	0.295	-0.259	0.796	0.926	0.274	-0.269	0.788	0.519	1.653
SGCD	-0.454	0.3	-1.514	0.13	0.635	0.19	-1.916	0.055	0.353	1.143
SGCB	0.267	0.297	0.901	0.368	1.307	0.388	0.79	0.429	0.73	2.338

SGCA	0.029	0.296	0.099	0.921	1.03	0.304	0.097	0.922	0.577	1.838
SFXN3	-0.336	0.3	-1.12	0.263	0.715	0.214	-1.331	0.183	0.397	1.286
SFXN1	0.579	0.303	1.908	0.056	1.783	0.541	1.449	0.147	0.984	3.232
SFTPD	0.181	0.296	0.611	0.541	1.198	0.355	0.559	0.576	0.671	2.141
SFTPC	0.336	0.298	1.127	0.26	1.399	0.417	0.958	0.338	0.78	2.507
SFTPB	-0.295	0.298	-0.99	0.322	0.745	0.222	-1.151	0.25	0.416	1.335
SFT2D2	0.872	0.321	2.719	0.007	2.393	0.768	1.814	0.07	1.276	4.487
SFSWAP	0.509	0.302	1.684	0.092	1.663	0.503	1.319	0.187	0.92	3.008
SFRP5	0.43	0.3	1.432	0.152	1.537	0.461	1.164	0.244	0.853	2.767
SFRP4	-1.007	0.316	-3.186	0.001	0.365	0.115	-5.497	0	0.197	0.679
SFRP1	-0.077	0.295	-0.261	0.794	0.926	0.273	-0.271	0.786	0.519	1.651
SFPQ	0.334	0.298	1.123	0.261	1.397	0.416	0.955	0.34	0.779	2.503
SFN	0.553	0.3	1.844	0.065	1.738	0.521	1.417	0.157	0.966	3.129
SFMBT1	0.314	0.297	1.06	0.289	1.369	0.406	0.91	0.363	0.766	2.449
SFI1	-0.258	0.298	-0.867	0.386	0.773	0.23	-0.99	0.322	0.431	1.384
SF3B5	0.447	0.3	1.493	0.136	1.564	0.469	1.204	0.229	0.869	2.814
SF3B4	0.077	0.296	0.26	0.795	1.08	0.319	0.25	0.802	0.605	1.927
SF3B3	0.6	0.306	1.961	0.05	1.822	0.557	1.475	0.14	1	3.318
SF3B2	0.285	0.297	0.962	0.336	1.33	0.395	0.837	0.403	0.744	2.379
SF3B1	-0.132	0.297	-0.444	0.657	0.876	0.26	-0.475	0.635	0.49	1.569
SF3A3	-0.112	0.295	-0.378	0.705	0.894	0.264	-0.4	0.689	0.501	1.595
SF3A2	-0.343	0.298	-1.153	0.249	0.71	0.211	-1.376	0.169	0.396	1.271
SF3A1	-0.004	0.296	-0.012	0.99	0.996	0.295	-0.012	0.99	0.558	1.779
SF1	0.137	0.296	0.463	0.643	1.147	0.34	0.433	0.665	0.642	2.05
SEZ6L2	-0.043	0.296	-0.145	0.885	0.958	0.284	-0.148	0.882	0.536	1.712
SEZ6L	-0.17	0.296	-0.573	0.566	0.844	0.25	-0.625	0.532	0.472	1.508
SETX	-0.305	0.297	-1.028	0.304	0.737	0.219	-1.202	0.229	0.412	1.318
SETMAR	-0.593	0.311	-1.911	0.056	0.552	0.172	-2.608	0.009	0.301	1.015
SETDB1	-0.131	0.295	-0.444	0.657	0.877	0.259	-0.475	0.635	0.492	1.564
SETD6	0.726	0.31	2.344	0.019	2.067	0.64	1.667	0.096	1.126	3.793
SETD5	-0.267	0.296	-0.902	0.367	0.765	0.227	-1.034	0.301	0.428	1.368
SETD4	0.204	0.296	0.69	0.49	1.227	0.363	0.624	0.533	0.687	2.192
SETD3	-0.268	0.298	-0.901	0.368	0.765	0.228	-1.033	0.301	0.427	1.37
SETD2	-0.324	0.298	-1.085	0.278	0.723	0.216	-1.282	0.2	0.403	1.298
SETD1B	0.011	0.295	0.037	0.97	1.011	0.298	0.037	0.97	0.567	1.803
SETD1A	-0.065	0.296	-0.221	0.825	0.937	0.277	-0.228	0.82	0.525	1.672
SETBP1	-0.607	0.31	-1.958	0.05	0.545	0.169	-2.694	0.007	0.297	1
SET	0.38	0.3	1.268	0.205	1.462	0.438	1.055	0.291	0.813	2.63

SESN1	-0.641	0.303	-2.113	0.035	0.527	0.16	-2.961	0.003	0.291	0.955
SERTAD3	0.002	0.3	0.007	0.995	1.002	0.3	0.007	0.995	0.557	1.803
SERTAD2	0.095	0.295	0.321	0.748	1.099	0.325	0.306	0.759	0.616	1.961
SERPINI2	-0.016	0.295	-0.055	0.956	0.984	0.291	-0.056	0.956	0.552	1.755
SERPINI1	-0.415	0.3	-1.383	0.167	0.66	0.198	-1.715	0.086	0.367	1.189
SERPINH1	-0.555	0.303	-1.835	0.066	0.574	0.174	-2.454	0.014	0.317	1.038
SERPING1	-0.685	0.306	-2.241	0.025	0.504	0.154	-3.219	0.001	0.277	0.918
SERPINF2	-0.204	0.297	-0.686	0.493	0.816	0.242	-0.761	0.447	0.456	1.46
SERPINF1	-1.309	0.337	-3.889	0	0.27	0.091	-8.031	0	0.14	0.522
SERPINE2	-0.696	0.304	-2.291	0.022	0.498	0.151	-3.312	0.001	0.275	0.904
SERPINE1	0.002	0.296	0.008	0.993	1.002	0.297	0.008	0.993	0.561	1.792
SERPIND1	-0.47	0.303	-1.549	0.121	0.625	0.19	-1.977	0.048	0.345	1.133
SERPINC1	-0.176	0.296	-0.596	0.551	0.838	0.248	-0.652	0.514	0.47	1.496
SERPINB9	-0.165	0.296	-0.557	0.577	0.848	0.251	-0.606	0.544	0.475	1.514
SERPINB8	0.078	0.295	0.264	0.792	1.081	0.319	0.254	0.8	0.606	1.928
SERPINB7	-0.562	0.306	-1.839	0.066	0.57	0.174	-2.467	0.014	0.313	1.038
SERPINB6	-0.209	0.298	-0.701	0.483	0.812	0.242	-0.779	0.436	0.453	1.455
SERPINB5	-0.128	0.295	-0.433	0.665	0.88	0.26	-0.461	0.645	0.493	1.57
SERPINB4	-0.124	0.296	-0.42	0.674	0.883	0.261	-0.448	0.654	0.495	1.577
SERPINB3	0.373	0.3	1.242	0.214	1.451	0.435	1.037	0.3	0.806	2.613
SERPINB2	-0.834	0.315	-2.644	0.008	0.434	0.137	-4.129	0	0.234	0.806
SERPINB13	-0.007	0.296	-0.023	0.981	0.993	0.294	-0.024	0.981	0.556	1.773
SERPINB10	0.596	0.31	1.924	0.054	1.815	0.562	1.449	0.147	0.989	3.332
SERPINB1	-0.376	0.3	-1.255	0.21	0.687	0.206	-1.523	0.128	0.382	1.235
SERPINA7	-0.143	0.296	-0.483	0.629	0.867	0.256	-0.519	0.604	0.485	1.548
SERPINA6	-0.189	0.298	-0.636	0.525	0.828	0.246	-0.7	0.484	0.462	1.483
SERPINA5	0.073	0.296	0.247	0.805	1.076	0.319	0.238	0.812	0.602	1.922
SERPINA4	0.479	0.303	1.583	0.113	1.615	0.489	1.258	0.208	0.892	2.922
SERPINA3	-0.538	0.306	-1.759	0.078	0.584	0.179	-2.331	0.02	0.32	1.063
SERPINA2	0.016	0.295	0.053	0.958	1.016	0.3	0.052	0.958	0.57	1.811
SERPINA10	0.49	0.3	1.635	0.102	1.633	0.489	1.293	0.196	0.907	2.938
SERPINA1	-0.683	0.315	-2.169	0.03	0.505	0.159	-3.111	0.002	0.273	0.936
SERP1	0.116	0.296	0.393	0.695	1.123	0.332	0.371	0.711	0.629	2.004
SERINC5	-0.392	0.3	-1.31	0.19	0.676	0.202	-1.604	0.109	0.376	1.215
SERINC3	-0.301	0.298	-1.009	0.313	0.74	0.221	-1.178	0.239	0.413	1.327
SERINC2	0.209	0.296	0.704	0.481	1.232	0.365	0.636	0.525	0.689	2.203
SERINC1	-0.695	0.307	-2.264	0.024	0.499	0.153	-3.27	0.001	0.274	0.911
SERHL2	-0.735	0.31	-2.368	0.018	0.48	0.149	-3.497	0	0.261	0.881

SERGEF	-0.113	0.297	-0.381	0.703	0.893	0.265	-0.404	0.686	0.499	1.598
SERBP1	-0.429	0.302	-1.42	0.156	0.651	0.197	-1.774	0.076	0.36	1.177
SEPTIN9	0.685	0.31	2.213	0.027	1.984	0.615	1.602	0.109	1.082	3.641
SEPTIN8	0.221	0.297	0.744	0.457	1.248	0.371	0.668	0.504	0.697	2.236
SEPTIN7P11	0.216	0.296	0.73	0.465	1.242	0.368	0.657	0.512	0.695	2.219
SEPTIN7	-0.13	0.295	-0.441	0.659	0.878	0.259	-0.471	0.637	0.492	1.566
SEPTIN6	0.081	0.295	0.273	0.785	1.084	0.32	0.262	0.793	0.608	1.933
SEPTIN4	-0.474	0.3	-1.58	0.114	0.623	0.187	-2.021	0.043	0.346	1.121
SEPTIN2	-0.417	0.298	-1.398	0.162	0.659	0.196	-1.735	0.083	0.368	1.182
SEPTIN11	-0.528	0.303	-1.744	0.081	0.59	0.179	-2.298	0.022	0.326	1.067
SEPTIN10	-0.785	0.315	-2.495	0.013	0.456	0.144	-3.791	0	0.246	0.845
SEPT5-GP1BB	0.19	0.296	0.643	0.52	1.21	0.358	0.585	0.558	0.677	2.16
SEPHS2	-0.233	0.297	-0.784	0.433	0.792	0.235	-0.883	0.377	0.443	1.418
SEPHS1	0.044	0.295	0.148	0.882	1.045	0.308	0.145	0.885	0.586	1.863
SENP7	-0.277	0.298	-0.93	0.352	0.758	0.226	-1.072	0.284	0.423	1.359
SENP6	0.032	0.296	0.107	0.915	1.032	0.305	0.105	0.916	0.578	1.842
SENP5	0.315	0.3	1.048	0.294	1.37	0.411	0.9	0.368	0.761	2.467
SENP3	-0.548	0.306	-1.793	0.073	0.578	0.177	-2.387	0.017	0.318	1.052
SENP2	0.25	0.296	0.843	0.399	1.284	0.38	0.746	0.456	0.718	2.294
SEMG2	-0.009	0.295	-0.03	0.976	0.991	0.293	-0.03	0.976	0.556	1.769
SEMG1	0.331	0.298	1.111	0.267	1.392	0.415	0.946	0.344	0.777	2.495
SEMA7A	-0.738	0.306	-2.409	0.016	0.478	0.146	-3.563	0	0.262	0.872
SEMA6D	0.184	0.297	0.622	0.534	1.202	0.357	0.568	0.57	0.672	2.15
SEMA6C	0.19	0.298	0.637	0.524	1.209	0.36	0.581	0.562	0.674	2.168
SEMA6B	0.007	0.295	0.023	0.981	1.007	0.297	0.023	0.981	0.564	1.796
SEMA6A	0.032	0.295	0.109	0.914	1.033	0.305	0.107	0.915	0.579	1.842
SEMA5A	-0.762	0.306	-2.486	0.013	0.467	0.143	-3.726	0	0.256	0.851
SEMA4G	0.226	0.296	0.762	0.446	1.253	0.371	0.682	0.495	0.701	2.24
SEMA4F	0.612	0.306	2.001	0.045	1.844	0.564	1.497	0.135	1.013	3.358
SEMA4D	-0.321	0.298	-1.078	0.281	0.725	0.216	-1.271	0.204	0.405	1.301
SEMA4C	0.509	0.3	1.696	0.09	1.663	0.499	1.329	0.184	0.924	2.995
SEMA4A	-0.611	0.304	-2.01	0.044	0.543	0.165	-2.77	0.006	0.299	0.985
SEMA3G	0.08	0.295	0.272	0.786	1.084	0.32	0.261	0.794	0.607	1.934
SEMA3F	0.647	0.306	2.118	0.034	1.91	0.584	1.559	0.119	1.049	3.477
SEMA3E	-0.437	0.3	-1.455	0.146	0.646	0.194	-1.825	0.068	0.359	1.164
SEMA3D	0.343	0.298	1.15	0.25	1.409	0.42	0.974	0.33	0.786	2.526
SEMA3C	-0.806	0.315	-2.559	0.011	0.447	0.141	-3.933	0	0.241	0.828
SEMA3B	-0.109	0.296	-0.369	0.712	0.897	0.265	-0.39	0.697	0.502	1.602

SEMA3A	-0.065	0.295	-0.22	0.826	0.937	0.277	-0.228	0.82	0.525	1.672
SEM1	0.712	0.31	2.296	0.022	2.037	0.631	1.643	0.1	1.11	3.739
SELPLG	-0.469	0.303	-1.55	0.121	0.626	0.189	-1.977	0.048	0.346	1.132
SELP	-0.539	0.301	-1.793	0.073	0.583	0.175	-2.376	0.018	0.323	1.052
SELL	-0.393	0.298	-1.318	0.187	0.675	0.201	-1.615	0.106	0.376	1.211
SELENOW	-0.328	0.298	-1.104	0.27	0.72	0.214	-1.307	0.191	0.402	1.29
SELENOT	0.078	0.297	0.264	0.792	1.081	0.321	0.254	0.8	0.605	1.934
SELENOP	-0.167	0.296	-0.563	0.573	0.846	0.251	-0.613	0.54	0.473	1.513
SELENOF	-0.408	0.303	-1.344	0.179	0.665	0.202	-1.659	0.097	0.367	1.206
SELENBP1	-0.281	0.298	-0.943	0.346	0.755	0.225	-1.089	0.276	0.421	1.354
SELE	-0.554	0.303	-1.828	0.067	0.575	0.174	-2.442	0.015	0.317	1.041
SEL1L3	-0.477	0.299	-1.596	0.111	0.621	0.185	-2.045	0.041	0.346	1.115
SEL1L	-0.413	0.3	-1.378	0.168	0.662	0.198	-1.706	0.088	0.368	1.19
SEH1L	0.069	0.295	0.235	0.814	1.072	0.316	0.227	0.82	0.601	1.912
SECTM1	0.438	0.298	1.468	0.142	1.55	0.463	1.189	0.234	0.864	2.782
SECISBP2L	0.066	0.295	0.225	0.822	1.069	0.315	0.217	0.828	0.599	1.905
SECISBP2	-0.551	0.306	-1.801	0.072	0.576	0.176	-2.402	0.016	0.317	1.05
SEC63	0.143	0.296	0.483	0.629	1.154	0.342	0.45	0.653	0.645	2.063
SEC62	-0.011	0.295	-0.036	0.971	0.989	0.292	-0.036	0.971	0.555	1.764
SEC61G	0.323	0.298	1.087	0.277	1.382	0.411	0.929	0.353	0.771	2.476
SEC61B	0.156	0.296	0.527	0.598	1.169	0.346	0.488	0.625	0.654	2.088
SEC61A2	-0.235	0.298	-0.79	0.429	0.79	0.235	-0.891	0.373	0.441	1.416
SEC61A1	-0.169	0.296	-0.571	0.568	0.845	0.25	-0.622	0.534	0.473	1.509
SEC31B	-0.217	0.296	-0.731	0.465	0.805	0.239	-0.817	0.414	0.45	1.439
SEC31A	-0.009	0.296	-0.032	0.975	0.991	0.293	-0.032	0.975	0.554	1.77
SEC24D	0.065	0.295	0.218	0.827	1.067	0.315	0.212	0.832	0.598	1.903
SEC24C	-0.426	0.3	-1.421	0.155	0.653	0.196	-1.771	0.077	0.363	1.176
SEC24B	-0.312	0.3	-1.043	0.297	0.732	0.219	-1.224	0.221	0.407	1.316
SEC24A	-0.18	0.296	-0.609	0.542	0.835	0.247	-0.667	0.505	0.468	1.491
SEC23IP	-0.425	0.302	-1.406	0.16	0.654	0.198	-1.753	0.08	0.361	1.182
SEC23B	-0.298	0.3	-0.996	0.319	0.742	0.222	-1.161	0.246	0.412	1.335
SEC23A	-0.365	0.297	-1.227	0.22	0.694	0.206	-1.481	0.139	0.388	1.244
SEC22B	0.121	0.296	0.41	0.682	1.129	0.335	0.386	0.7	0.632	2.018
SEC22A	0.606	0.303	2.001	0.045	1.834	0.556	1.5	0.134	1.012	3.322
SEC16A	-0.245	0.296	-0.826	0.409	0.783	0.232	-0.936	0.35	0.438	1.399
SEC14L5	0.158	0.296	0.532	0.595	1.171	0.347	0.492	0.622	0.655	2.092
SEC14L4	-0.206	0.296	-0.696	0.486	0.814	0.241	-0.773	0.44	0.455	1.455
SEC14L3	0.396	0.298	1.33	0.184	1.486	0.442	1.098	0.272	0.829	2.663

SEC14L2	0.063	0.297	0.212	0.832	1.065	0.316	0.205	0.838	0.595	1.905
SEC14L1P1	-0.289	0.298	-0.97	0.332	0.749	0.223	-1.124	0.261	0.418	1.343
SEC14L1	-0.216	0.296	-0.731	0.465	0.805	0.238	-0.816	0.415	0.451	1.439
SEC13	0.188	0.296	0.636	0.525	1.207	0.358	0.58	0.562	0.675	2.158
SEC11A	0.274	0.297	0.924	0.356	1.315	0.39	0.808	0.419	0.735	2.352
SDS	0.521	0.302	1.722	0.085	1.683	0.509	1.342	0.179	0.931	3.044
SDR39U1	0.582	0.303	1.925	0.054	1.79	0.542	1.459	0.144	0.99	3.239
SDK2	0.185	0.296	0.627	0.531	1.204	0.356	0.572	0.567	0.674	2.148
SDHD	-0.371	0.298	-1.246	0.213	0.69	0.205	-1.508	0.132	0.385	1.237
SDHC	0.153	0.296	0.516	0.606	1.165	0.345	0.478	0.632	0.652	2.081
SDHB	-0.223	0.306	-0.728	0.467	0.8	0.245	-0.815	0.415	0.439	1.458
SDHAF3	0.016	0.295	0.053	0.958	1.016	0.3	0.052	0.958	0.57	1.811
SDHAF1	0.571	0.301	1.895	0.058	1.77	0.534	1.444	0.149	0.981	3.196
SDHA	0.646	0.307	2.105	0.035	1.909	0.586	1.55	0.121	1.046	3.484
SDF4	-0.743	0.316	-2.353	0.019	0.476	0.15	-3.491	0	0.256	0.883
SDF2L1	0.41	0.298	1.374	0.169	1.507	0.449	1.127	0.26	0.84	2.703
SDF2	0.291	0.297	0.982	0.326	1.338	0.397	0.852	0.394	0.748	2.394
SDCBP	-0.598	0.306	-1.955	0.051	0.55	0.168	-2.675	0.007	0.302	1.002
SDC4	-0.17	0.296	-0.574	0.566	0.844	0.249	-0.626	0.531	0.473	1.506
SDC3	-0.646	0.306	-2.112	0.035	0.524	0.16	-2.967	0.003	0.288	0.955
SDC2	0.071	0.296	0.24	0.811	1.074	0.318	0.231	0.817	0.601	1.917
SDC1	-0.439	0.3	-1.46	0.144	0.645	0.194	-1.833	0.067	0.358	1.162
SDAD1	-0.036	0.297	-0.123	0.902	0.964	0.286	-0.125	0.901	0.539	1.724
SCYL3	-0.267	0.298	-0.899	0.369	0.765	0.228	-1.03	0.303	0.427	1.371
SCYL2	0.018	0.296	0.06	0.952	1.018	0.301	0.059	0.953	0.57	1.817
SCUBE3	0.06	0.295	0.204	0.839	1.062	0.314	0.198	0.843	0.595	1.895
SCUBE2	-0.145	0.297	-0.487	0.626	0.865	0.257	-0.524	0.6	0.484	1.548
SCTR	0.188	0.299	0.63	0.528	1.207	0.36	0.575	0.566	0.672	2.167
SCT	-0.309	0.304	-1.015	0.31	0.735	0.223	-1.189	0.235	0.405	1.333
SCRT1	-0.386	0.3	-1.287	0.198	0.68	0.204	-1.57	0.116	0.378	1.223
SCRN3	-0.318	0.3	-1.06	0.289	0.728	0.218	-1.248	0.212	0.404	1.31
SCRN1	0.311	0.298	1.044	0.296	1.365	0.406	0.898	0.369	0.761	2.446
SCRIB	1.13	0.336	3.363	0.001	3.096	1.04	2.015	0.044	1.602	5.981
SCRG1	-0.156	0.295	-0.529	0.597	0.855	0.253	-0.573	0.567	0.479	1.526
SCPEP1	-0.177	0.296	-0.598	0.55	0.838	0.248	-0.654	0.513	0.47	1.495
SCP2	-0.507	0.306	-1.658	0.097	0.602	0.184	-2.159	0.031	0.331	1.097
SCO2	-0.072	0.296	-0.242	0.809	0.931	0.276	-0.25	0.802	0.521	1.663
SCNN1G	-0.247	0.296	-0.835	0.404	0.781	0.231	-0.947	0.344	0.437	1.396

SCNN1D	0.081	0.296	0.274	0.784	1.084	0.321	0.263	0.792	0.608	1.936
SCNN1B	-0.121	0.296	-0.408	0.683	0.886	0.262	-0.434	0.664	0.496	1.584
SCNN1A	0.009	0.296	0.03	0.976	1.009	0.299	0.03	0.976	0.565	1.802
SCN9A	-0.132	0.295	-0.446	0.656	0.877	0.259	-0.476	0.634	0.491	1.564
SCN8A	0.132	0.295	0.447	0.655	1.141	0.337	0.419	0.676	0.64	2.035
SCN7A	-0.177	0.296	-0.598	0.55	0.838	0.248	-0.654	0.513	0.469	1.497
SCN5A	0.615	0.303	2.03	0.042	1.849	0.56	1.516	0.129	1.021	3.349
SCN4A	0.318	0.298	1.067	0.286	1.374	0.409	0.914	0.361	0.766	2.464
SCN3B	-0.192	0.296	-0.649	0.517	0.825	0.244	-0.715	0.475	0.462	1.474
SCN3A	0.336	0.298	1.128	0.259	1.399	0.417	0.958	0.338	0.78	2.509
SCN2B	-0.011	0.296	-0.038	0.97	0.989	0.292	-0.038	0.969	0.554	1.765
SCN2A	0.662	0.31	2.136	0.033	1.938	0.6	1.562	0.118	1.056	3.557
SCN1B	-0.024	0.295	-0.082	0.935	0.976	0.288	-0.083	0.934	0.547	1.742
SCN1A	0	0.295	0.001	0.999	1	0.295	0.001	0.999	0.561	1.783
SCN11A	0.407	0.3	1.36	0.174	1.503	0.45	1.117	0.264	0.836	2.703
SCN10A	0.532	0.302	1.759	0.079	1.702	0.515	1.364	0.173	0.941	3.08
SCML2	0.375	0.3	1.253	0.21	1.455	0.436	1.045	0.296	0.809	2.618
SCML1	-0.106	0.295	-0.36	0.719	0.899	0.266	-0.38	0.704	0.504	1.604
SCMH1	-0.12	0.297	-0.405	0.685	0.887	0.263	-0.43	0.667	0.496	1.586
SCLY	-0.334	0.298	-1.122	0.262	0.716	0.213	-1.332	0.183	0.399	1.284
SCIN	0.152	0.295	0.515	0.606	1.164	0.344	0.478	0.633	0.653	2.077
SCGN	-0.404	0.3	-1.349	0.177	0.668	0.2	-1.663	0.096	0.371	1.201
SCGB2A2	0.307	0.298	1.032	0.302	1.36	0.405	0.889	0.374	0.759	2.437
SCGB2A1	0.151	0.295	0.511	0.609	1.163	0.343	0.475	0.635	0.652	2.075
SCGB1D2	0.365	0.3	1.218	0.223	1.44	0.432	1.02	0.308	0.801	2.591
SCGB1D1	0.533	0.302	1.765	0.078	1.705	0.515	1.368	0.171	0.943	3.083
SCGB1A1	-0.514	0.302	-1.702	0.089	0.598	0.181	-2.225	0.026	0.331	1.081
SCG5	-0.248	0.296	-0.839	0.402	0.78	0.231	-0.952	0.341	0.437	1.394
SCG3	-0.332	0.3	-1.107	0.268	0.718	0.215	-1.313	0.189	0.399	1.291
SCG2	-0.209	0.297	-0.706	0.48	0.811	0.241	-0.785	0.433	0.454	1.451
SCFD1	-0.503	0.302	-1.665	0.096	0.605	0.183	-2.164	0.03	0.334	1.093
SCEL	-0.128	0.295	-0.434	0.664	0.88	0.26	-0.463	0.643	0.493	1.57
SCD5	0.291	0.298	0.978	0.328	1.338	0.398	0.848	0.396	0.747	2.397
SCD	-0.051	0.296	-0.174	0.862	0.95	0.281	-0.178	0.858	0.532	1.695
SCCPDH	-0.146	0.296	-0.493	0.622	0.864	0.256	-0.531	0.596	0.484	1.544
SCARF1	-0.006	0.295	-0.02	0.984	0.994	0.294	-0.02	0.984	0.557	1.774
SCARB2	0.104	0.295	0.353	0.724	1.11	0.328	0.335	0.737	0.622	1.98
SCARB1	0.094	0.297	0.316	0.752	1.098	0.326	0.301	0.763	0.614	1.966

SCARA3	-0.527	0.3	-1.755	0.079	0.591	0.177	-2.31	0.021	0.328	1.063
SCAPER	0.179	0.295	0.607	0.544	1.196	0.354	0.556	0.578	0.67	2.135
SCAP	0.194	0.298	0.65	0.515	1.214	0.362	0.591	0.554	0.677	2.176
SCAND2P	-0.137	0.296	-0.464	0.643	0.872	0.258	-0.497	0.619	0.488	1.558
SCAND1	0.242	0.296	0.817	0.414	1.274	0.378	0.726	0.468	0.713	2.277
SCAMP5	0.023	0.295	0.079	0.937	1.024	0.302	0.078	0.938	0.574	1.826
SCAMP4	-0.102	0.296	-0.344	0.731	0.903	0.267	-0.362	0.717	0.506	1.612
SCAMP3	0.195	0.297	0.658	0.51	1.216	0.361	0.598	0.55	0.68	2.175
SCAMP2	-0.566	0.306	-1.849	0.064	0.568	0.174	-2.487	0.013	0.312	1.034
SCAMP1	-0.249	0.3	-0.83	0.407	0.779	0.234	-0.943	0.346	0.433	1.404
SCAI	0.603	0.303	1.994	0.046	1.828	0.553	1.497	0.134	1.01	3.308
SCAF8	-0.452	0.3	-1.505	0.132	0.637	0.191	-1.903	0.057	0.354	1.146
SCAF4	0.141	0.295	0.477	0.633	1.151	0.34	0.445	0.656	0.645	2.054
SCAF11	0.587	0.306	1.921	0.055	1.799	0.55	1.453	0.146	0.988	3.276
SC5D	-0.64	0.306	-2.095	0.036	0.527	0.161	-2.935	0.003	0.29	0.96
SBSPON	-0.526	0.306	-1.722	0.085	0.591	0.181	-2.267	0.023	0.324	1.075
SBNO2	-0.224	0.298	-0.751	0.453	0.8	0.238	-0.841	0.4	0.446	1.433
SBNO1	0.444	0.298	1.49	0.136	1.559	0.465	1.203	0.229	0.869	2.797
SBF1	-0.474	0.301	-1.577	0.115	0.622	0.187	-2.017	0.044	0.345	1.122
SAYSD1	0.127	0.295	0.429	0.668	1.135	0.335	0.403	0.687	0.636	2.025
SAV1	-0.5	0.3	-1.665	0.096	0.607	0.182	-2.16	0.031	0.337	1.093
SATB2	-0.355	0.298	-1.192	0.233	0.701	0.209	-1.431	0.152	0.391	1.257
SATB1	0.116	0.295	0.392	0.695	1.123	0.332	0.37	0.711	0.629	2.003
SAT1	-0.272	0.298	-0.915	0.36	0.762	0.227	-1.052	0.293	0.425	1.365
SASH3	0.082	0.296	0.279	0.78	1.086	0.321	0.268	0.789	0.608	1.938
SASH1	0.25	0.298	0.839	0.401	1.284	0.383	0.743	0.458	0.716	2.303
SART3	0.005	0.296	0.016	0.988	1.005	0.297	0.016	0.988	0.563	1.793
SART1	0.694	0.31	2.242	0.025	2.002	0.62	1.617	0.106	1.091	3.674
SARS2	0.017	0.295	0.058	0.953	1.017	0.3	0.058	0.954	0.571	1.814
SARS1	-0.31	0.3	-1.036	0.3	0.733	0.22	-1.215	0.224	0.408	1.319
SARM1	-0.163	0.296	-0.549	0.583	0.85	0.252	-0.597	0.551	0.475	1.519
SARDH	-0.328	0.3	-1.093	0.275	0.721	0.216	-1.293	0.196	0.4	1.297
SARAF	-0.533	0.306	-1.743	0.081	0.587	0.179	-2.302	0.021	0.322	1.069
SAR1B	0.436	0.299	1.459	0.145	1.547	0.463	1.182	0.237	0.861	2.78
SAR1A	0.275	0.3	0.916	0.359	1.317	0.395	0.801	0.423	0.731	2.372
SAP30L	0.199	0.298	0.669	0.503	1.22	0.363	0.607	0.544	0.681	2.186
SAP30BP	0.542	0.302	1.795	0.073	1.72	0.52	1.385	0.166	0.951	3.111
SAP30	0.163	0.295	0.553	0.58	1.177	0.348	0.51	0.61	0.66	2.1

SAP18	0.365	0.298	1.223	0.221	1.44	0.43	1.025	0.306	0.803	2.584
SAP130	0.83	0.315	2.635	0.008	2.293	0.723	1.79	0.073	1.237	4.253
SAMSN1	-0.221	0.296	-0.745	0.456	0.802	0.238	-0.833	0.405	0.449	1.433
SAMM50	0.16	0.297	0.538	0.591	1.173	0.348	0.497	0.619	0.656	2.098
SAMHD1	-0.025	0.296	-0.086	0.931	0.975	0.288	-0.087	0.93	0.546	1.74
SAMD9	-0.478	0.302	-1.581	0.114	0.62	0.187	-2.026	0.043	0.343	1.122
SAMD4B	-0.19	0.296	-0.643	0.52	0.827	0.244	-0.708	0.479	0.463	1.476
SAMD4A	-0.578	0.303	-1.909	0.056	0.561	0.17	-2.585	0.01	0.31	1.015
SAMD14	0.867	0.315	2.753	0.006	2.38	0.75	1.841	0.066	1.284	4.413
SALL2	-0.053	0.295	-0.178	0.858	0.949	0.28	-0.183	0.855	0.532	1.692
SALL1	-0.499	0.302	-1.652	0.099	0.607	0.184	-2.142	0.032	0.336	1.098
SAGE1	-0.104	0.295	-0.353	0.724	0.901	0.266	-0.372	0.71	0.505	1.607
SAG	0.729	0.31	2.352	0.019	2.072	0.642	1.67	0.095	1.129	3.802
SAFB2	0.329	0.298	1.106	0.269	1.39	0.414	0.943	0.346	0.776	2.492
SAFB	0.153	0.295	0.519	0.603	1.166	0.344	0.481	0.63	0.653	2.08
SAE1	0.346	0.298	1.164	0.245	1.414	0.421	0.984	0.325	0.789	2.534
SACS	0.055	0.297	0.185	0.853	1.056	0.314	0.18	0.857	0.59	1.891
SACM1L	-0.312	0.298	-1.047	0.295	0.732	0.218	-1.228	0.219	0.409	1.312
SAC3D1	0.141	0.296	0.477	0.633	1.151	0.34	0.445	0.656	0.645	2.055
SAA3P	-0.065	0.296	-0.22	0.826	0.937	0.277	-0.228	0.82	0.525	1.673
S1PR5	-0.101	0.295	-0.342	0.733	0.904	0.267	-0.36	0.719	0.507	1.613
S1PR4	0.594	0.303	1.958	0.05	1.811	0.549	1.476	0.14	0.999	3.281
S1PR2	0.038	0.295	0.129	0.898	1.039	0.307	0.126	0.9	0.582	1.852
S1PR1	-0.512	0.301	-1.702	0.089	0.599	0.18	-2.222	0.026	0.332	1.081
S100PBP	0.001	0.295	0.004	0.997	1.001	0.296	0.004	0.997	0.561	1.786
S100P	0.043	0.297	0.145	0.885	1.044	0.31	0.142	0.887	0.584	1.867
S100G	0.354	0.3	1.182	0.237	1.425	0.427	0.995	0.32	0.792	2.563
S100B	0.055	0.295	0.187	0.852	1.057	0.312	0.182	0.856	0.593	1.885
S100A9	0.294	0.298	0.989	0.323	1.342	0.4	0.857	0.392	0.749	2.406
S100A8	0.442	0.303	1.459	0.145	1.556	0.471	1.179	0.238	0.859	2.817
S100A7	-0.572	0.304	-1.885	0.059	0.564	0.171	-2.543	0.011	0.311	1.023
S100A6	0.206	0.296	0.695	0.487	1.229	0.364	0.628	0.53	0.688	2.195
S100A5	0.01	0.295	0.035	0.972	1.01	0.298	0.035	0.972	0.567	1.802
S100A4	-0.22	0.298	-0.739	0.46	0.802	0.239	-0.827	0.408	0.448	1.438
S100A3	-0.373	0.3	-1.246	0.213	0.688	0.206	-1.511	0.131	0.383	1.239
S100A2	-0.145	0.296	-0.49	0.624	0.865	0.256	-0.527	0.598	0.484	1.546
S100A14	0.095	0.295	0.321	0.748	1.099	0.325	0.306	0.76	0.616	1.961
S100A13	-0.203	0.296	-0.686	0.493	0.816	0.242	-0.76	0.447	0.457	1.459

S100A12	0.06	0.297	0.201	0.841	1.062	0.316	0.195	0.846	0.593	1.902
S100A11	-0.265	0.299	-0.887	0.375	0.767	0.229	-1.016	0.31	0.427	1.378
S100A10	0.365	0.298	1.225	0.221	1.441	0.43	1.026	0.305	0.803	2.585
S100A1	0.474	0.306	1.55	0.121	1.606	0.491	1.234	0.217	0.882	2.924
RYR3	0.08	0.295	0.27	0.787	1.083	0.32	0.259	0.795	0.607	1.931
RYR2	-0.191	0.297	-0.645	0.519	0.826	0.245	-0.71	0.477	0.461	1.478
RYR1	-1.158	0.329	-3.523	0	0.314	0.103	-6.645	0	0.165	0.598
RYK	-0.002	0.296	-0.006	0.995	0.998	0.295	-0.006	0.995	0.559	1.782
RYBP	-0.655	0.31	-2.115	0.034	0.519	0.161	-2.987	0.003	0.283	0.953
RXYLT1	0.048	0.295	0.162	0.871	1.049	0.31	0.158	0.874	0.588	1.872
RXRG	0.245	0.298	0.824	0.41	1.278	0.38	0.73	0.465	0.713	2.29
RXRB	0.15	0.298	0.502	0.616	1.161	0.346	0.466	0.641	0.647	2.083
RXRA	-0.022	0.295	-0.075	0.94	0.978	0.289	-0.076	0.94	0.549	1.744
RXFP3	-0.275	0.297	-0.924	0.355	0.76	0.226	-1.064	0.287	0.424	1.361
RWDD3	0.136	0.296	0.459	0.646	1.145	0.339	0.429	0.668	0.641	2.045
RWDD2B	0.137	0.296	0.463	0.643	1.147	0.339	0.433	0.665	0.642	2.047
RWDD2A	-0.311	0.297	-1.047	0.295	0.733	0.217	-1.229	0.219	0.41	1.311
RWDD1	0.025	0.296	0.084	0.933	1.025	0.303	0.083	0.934	0.574	1.83
RUVBL2	0.067	0.295	0.227	0.821	1.069	0.316	0.219	0.827	0.599	1.908
RUVBL1	0.174	0.295	0.59	0.555	1.19	0.352	0.541	0.588	0.667	2.124
RUSF1	-0.993	0.317	-3.13	0.002	0.371	0.118	-5.355	0	0.199	0.69
RUSC2	0.434	0.3	1.447	0.148	1.543	0.463	1.174	0.24	0.857	2.777
RUSC1	0.367	0.303	1.214	0.225	1.444	0.437	1.016	0.31	0.798	2.613
RUNX3	-0.248	0.297	-0.837	0.402	0.78	0.231	-0.951	0.342	0.436	1.395
RUNX2	-0.963	0.321	-2.997	0.003	0.382	0.123	-5.039	0	0.203	0.717
RUNX1T1	0.144	0.295	0.489	0.625	1.155	0.341	0.455	0.649	0.648	2.061
RUNX1-IT1	0.243	0.298	0.816	0.415	1.275	0.379	0.724	0.469	0.711	2.285
RUNDC3B	-0.253	0.297	-0.852	0.394	0.777	0.23	-0.97	0.332	0.434	1.389
RUNDC3A	0.097	0.295	0.327	0.744	1.101	0.325	0.312	0.755	0.617	1.965
RUFY3	-0.334	0.3	-1.115	0.265	0.716	0.215	-1.324	0.185	0.398	1.288
RUFY2	-0.208	0.297	-0.701	0.483	0.812	0.241	-0.779	0.436	0.454	1.453
RUFY1	-0.141	0.295	-0.478	0.632	0.868	0.256	-0.514	0.607	0.487	1.549
RUBCNL	-0.243	0.296	-0.819	0.413	0.784	0.233	-0.927	0.354	0.439	1.402
RUBCN	0.681	0.31	2.196	0.028	1.977	0.613	1.593	0.111	1.076	3.631
RTRAF	-0.046	0.296	-0.155	0.877	0.955	0.283	-0.158	0.874	0.535	1.706
RTP4	0.135	0.296	0.457	0.648	1.145	0.339	0.427	0.669	0.64	2.047
RTN4	0.202	0.296	0.684	0.494	1.224	0.362	0.619	0.536	0.685	2.187
RTN3	0.134	0.296	0.453	0.651	1.143	0.338	0.424	0.672	0.641	2.04

RTN2	0.118	0.295	0.399	0.69	1.125	0.332	0.376	0.707	0.631	2.007
RTN1	-0.562	0.306	-1.837	0.066	0.57	0.174	-2.465	0.014	0.313	1.038
RTL8C	-0.084	0.295	-0.286	0.775	0.919	0.271	-0.298	0.766	0.515	1.64
RTL8A	0.459	0.3	1.532	0.126	1.583	0.474	1.228	0.219	0.88	2.848
RTF2	0.189	0.296	0.639	0.523	1.208	0.358	0.582	0.561	0.676	2.161
RTF1	-0.206	0.298	-0.69	0.49	0.814	0.243	-0.766	0.444	0.454	1.46
RTEL1	0.458	0.3	1.528	0.126	1.581	0.474	1.226	0.22	0.879	2.845
RTCB	-0.379	0.298	-1.272	0.203	0.684	0.204	-1.547	0.122	0.381	1.228
RTCA	0.015	0.296	0.05	0.96	1.015	0.3	0.049	0.961	0.569	1.811
RSU1	0.465	0.3	1.55	0.121	1.593	0.478	1.239	0.215	0.884	2.87
RSRC2	0.393	0.298	1.322	0.186	1.482	0.441	1.093	0.275	0.827	2.656
RSRC1	0.888	0.311	2.853	0.004	2.431	0.757	1.89	0.059	1.321	4.476
RSPH6A	0.183	0.297	0.615	0.539	1.2	0.357	0.562	0.574	0.67	2.149
RSPH14	0.49	0.302	1.622	0.105	1.633	0.493	1.282	0.2	0.903	2.952
RSL24D1	-0.555	0.303	-1.832	0.067	0.574	0.174	-2.449	0.014	0.317	1.04
RSL1D1	-0.944	0.315	-2.994	0.003	0.389	0.123	-4.981	0	0.21	0.722
RSF1	0.349	0.3	1.163	0.245	1.417	0.425	0.982	0.326	0.787	2.552
RSBN1	-0.008	0.296	-0.027	0.978	0.992	0.293	-0.028	0.978	0.556	1.77
RSAD2	0.477	0.302	1.577	0.115	1.611	0.487	1.254	0.21	0.891	2.914
RSAD1	-0.571	0.302	-1.891	0.059	0.565	0.171	-2.551	0.011	0.312	1.021
RS1	0.29	0.298	0.973	0.331	1.337	0.399	0.844	0.399	0.745	2.398
RRS1	0.487	0.302	1.609	0.108	1.627	0.492	1.274	0.203	0.899	2.943
RRP9	0.195	0.296	0.66	0.509	1.215	0.359	0.599	0.549	0.681	2.17
RRP8	0.621	0.306	2.031	0.042	1.861	0.569	1.513	0.13	1.022	3.388
RRP7A	0.225	0.296	0.761	0.447	1.253	0.371	0.681	0.496	0.701	2.238
RRP1B	0.529	0.306	1.728	0.084	1.697	0.519	1.342	0.18	0.931	3.091
RRP15	0.92	0.315	2.92	0.004	2.51	0.791	1.909	0.056	1.353	4.654
RRP12	0.265	0.299	0.885	0.376	1.303	0.39	0.778	0.437	0.725	2.342
RRP1	0.639	0.304	2.101	0.036	1.895	0.576	1.553	0.12	1.044	3.439
RRNAD1	0.158	0.296	0.533	0.594	1.171	0.347	0.493	0.622	0.655	2.093
RRN3P1	-0.574	0.303	-1.895	0.058	0.563	0.171	-2.561	0.01	0.311	1.02
RRN3	0.552	0.302	1.824	0.068	1.736	0.525	1.402	0.161	0.96	3.141
RRM2	0.9	0.321	2.807	0.005	2.46	0.789	1.851	0.064	1.312	4.612
RRM1	0.155	0.296	0.526	0.599	1.168	0.345	0.487	0.626	0.655	2.085
RRH	0.324	0.298	1.088	0.277	1.383	0.412	0.929	0.353	0.771	2.478
RREB1	-0.746	0.31	-2.402	0.016	0.474	0.147	-3.57	0	0.258	0.872
RRBP1	0.141	0.299	0.473	0.636	1.152	0.344	0.441	0.659	0.641	2.069
RRAS2	0.365	0.298	1.226	0.22	1.441	0.429	1.027	0.304	0.804	2.583

RRAS	-0.18	0.299	-0.601	0.548	0.835	0.25	-0.658	0.511	0.465	1.502
RRAGD	0.309	0.297	1.043	0.297	1.363	0.404	0.897	0.37	0.762	2.438
RRAGC	0.126	0.296	0.425	0.671	1.134	0.336	0.399	0.69	0.634	2.028
RRAGB	-0.301	0.298	-1.01	0.312	0.74	0.22	-1.179	0.238	0.413	1.327
RRAGA	0.343	0.299	1.146	0.252	1.41	0.422	0.97	0.332	0.784	2.535
RRAD	-0.211	0.298	-0.708	0.479	0.81	0.241	-0.788	0.431	0.452	1.452
RPUSD2	0.023	0.296	0.079	0.937	1.024	0.303	0.078	0.938	0.573	1.827
RPS9	0.069	0.295	0.232	0.816	1.071	0.316	0.224	0.822	0.6	1.91
RPS8	-0.047	0.295	-0.16	0.873	0.954	0.282	-0.163	0.87	0.535	1.702
RPS7	-0.121	0.297	-0.409	0.682	0.886	0.263	-0.435	0.663	0.495	1.584
RPS6KC1	0.215	0.297	0.725	0.468	1.24	0.368	0.653	0.514	0.693	2.217
RPS6KB2	0.811	0.321	2.527	0.012	2.25	0.722	1.731	0.083	1.2	4.22
RPS6KB1	0.256	0.298	0.86	0.39	1.292	0.384	0.759	0.448	0.721	2.315
RPS6KA6	-0.065	0.296	-0.221	0.825	0.937	0.277	-0.228	0.819	0.524	1.674
RPS6KA5	-0.256	0.298	-0.86	0.39	0.774	0.231	-0.98	0.327	0.432	1.388
RPS6KA4	-0.149	0.297	-0.5	0.617	0.862	0.256	-0.539	0.59	0.481	1.544
RPS6KA3	-0.274	0.297	-0.924	0.355	0.76	0.225	-1.063	0.288	0.425	1.36
RPS6KA2	-0.805	0.315	-2.557	0.011	0.447	0.141	-3.929	0	0.241	0.829
RPS6KA1	-0.407	0.303	-1.344	0.179	0.665	0.202	-1.66	0.097	0.367	1.205
RPS6	-0.461	0.302	-1.524	0.127	0.631	0.191	-1.936	0.053	0.349	1.141
RPS5	0.116	0.295	0.392	0.695	1.123	0.332	0.37	0.711	0.629	2.004
RPS4Y1	-0.138	0.296	-0.468	0.639	0.871	0.257	-0.502	0.615	0.488	1.554
RPS4X	-0.453	0.306	-1.479	0.139	0.636	0.195	-1.871	0.061	0.349	1.159
RPS3	-0.122	0.296	-0.411	0.681	0.885	0.262	-0.438	0.662	0.495	1.583
RPS2P45	0.019	0.297	0.063	0.95	1.019	0.303	0.062	0.95	0.569	1.823
RPS29	-0.179	0.296	-0.607	0.544	0.836	0.247	-0.664	0.506	0.468	1.492
RPS28	0.286	0.296	0.966	0.334	1.331	0.394	0.84	0.401	0.745	2.379
RPS27L	-0.146	0.299	-0.489	0.625	0.864	0.258	-0.527	0.598	0.481	1.551
RPS27A	0.024	0.298	0.08	0.936	1.024	0.306	0.079	0.937	0.571	1.838
RPS27	0.052	0.295	0.176	0.861	1.053	0.311	0.171	0.864	0.59	1.878
RPS25	-0.203	0.295	-0.687	0.492	0.816	0.241	-0.762	0.446	0.457	1.457
RPS24	-0.842	0.316	-2.661	0.008	0.431	0.136	-4.175	0	0.232	0.801
RPS23	0.412	0.298	1.383	0.167	1.51	0.45	1.133	0.257	0.842	2.708
RPS21	0.716	0.315	2.272	0.023	2.046	0.644	1.623	0.105	1.103	3.792
RPS2	-0.498	0.306	-1.628	0.104	0.608	0.186	-2.109	0.035	0.334	1.107
RPS19	-0.026	0.296	-0.088	0.93	0.974	0.288	-0.089	0.929	0.545	1.74
RPS18	0.247	0.298	0.828	0.408	1.28	0.381	0.734	0.463	0.714	2.294
RPS17	-0.467	0.302	-1.546	0.122	0.627	0.189	-1.971	0.049	0.347	1.133

RPS16	0.076	0.295	0.257	0.797	1.079	0.319	0.248	0.804	0.605	1.925
RPS15A	-0.224	0.298	-0.753	0.452	0.799	0.238	-0.844	0.399	0.446	1.432
RPS15	-0.053	0.297	-0.18	0.857	0.948	0.282	-0.184	0.854	0.529	1.698
RPS14	0.029	0.295	0.098	0.922	1.029	0.304	0.097	0.923	0.577	1.836
RPS12	0.228	0.297	0.767	0.443	1.256	0.372	0.686	0.493	0.702	2.245
RPS11	0.85	0.311	2.731	0.006	2.339	0.728	1.839	0.066	1.271	4.306
RPS10	0.181	0.295	0.612	0.54	1.198	0.354	0.56	0.575	0.672	2.138
RPRM	0.414	0.3	1.379	0.168	1.512	0.454	1.129	0.259	0.84	2.722
RPRD2	-0.285	0.298	-0.957	0.339	0.752	0.224	-1.107	0.268	0.42	1.348
RPRD1A	-0.024	0.297	-0.082	0.935	0.976	0.289	-0.083	0.934	0.546	1.745
RPP40	0.994	0.328	3.033	0.002	2.701	0.885	1.922	0.055	1.421	5.133
RPP38	0.239	0.296	0.808	0.419	1.27	0.376	0.719	0.472	0.711	2.27
RPP30	-0.196	0.298	-0.657	0.511	0.822	0.245	-0.726	0.468	0.459	1.474
RPP25	0.216	0.296	0.728	0.467	1.241	0.368	0.655	0.513	0.694	2.217
RPP14	-0.192	0.296	-0.647	0.518	0.826	0.244	-0.713	0.476	0.462	1.475
RPN2	0.265	0.296	0.895	0.371	1.304	0.386	0.786	0.432	0.73	2.329
RPN1	0.31	0.298	1.042	0.297	1.363	0.406	0.896	0.37	0.761	2.443
RPLP2	0.749	0.31	2.415	0.016	2.114	0.655	1.7	0.089	1.152	3.882
RPLP1	-0.005	0.296	-0.017	0.986	0.995	0.295	-0.017	0.986	0.557	1.777
RPLP0	-0.275	0.3	-0.915	0.36	0.76	0.228	-1.053	0.292	0.422	1.368
RPL9	-0.217	0.301	-0.72	0.471	0.805	0.242	-0.804	0.421	0.447	1.452
RPL8	0.347	0.297	1.172	0.241	1.416	0.42	0.99	0.322	0.792	2.531
RPL7	-0.316	0.3	-1.052	0.293	0.729	0.219	-1.237	0.216	0.405	1.313
RPL6	-0.435	0.3	-1.451	0.147	0.647	0.194	-1.817	0.069	0.36	1.165
RPL41	0.318	0.298	1.069	0.285	1.375	0.409	0.916	0.36	0.767	2.463
RPL3L	0.115	0.296	0.39	0.697	1.122	0.333	0.368	0.713	0.628	2.006
RPL39L	0.723	0.307	2.36	0.018	2.061	0.632	1.68	0.093	1.13	3.759
RPL39	0.763	0.315	2.424	0.015	2.144	0.674	1.696	0.09	1.157	3.972
RPL38	0.065	0.296	0.22	0.826	1.067	0.316	0.213	0.831	0.598	1.906
RPL37A	0.727	0.3	2.427	0.015	2.069	0.62	1.724	0.085	1.15	3.724
RPL37	0.248	0.3	0.828	0.408	1.282	0.384	0.733	0.463	0.712	2.306
RPL36AL	-0.541	0.301	-1.795	0.073	0.582	0.175	-2.381	0.017	0.323	1.051
RPL36	0.211	0.296	0.711	0.477	1.234	0.366	0.641	0.522	0.69	2.207
RPL35A	-0.16	0.296	-0.542	0.588	0.852	0.252	-0.588	0.556	0.477	1.52
RPL35	0.672	0.31	2.169	0.03	1.959	0.607	1.579	0.114	1.067	3.596
RPL34	0.05	0.296	0.168	0.866	1.051	0.311	0.164	0.87	0.589	1.877
RPL32	-0.152	0.296	-0.513	0.608	0.859	0.254	-0.555	0.579	0.481	1.535
RPL31	-0.264	0.298	-0.886	0.376	0.768	0.229	-1.014	0.311	0.429	1.377

RPL30	-0.285	0.3	-0.95	0.342	0.752	0.225	-1.1	0.271	0.418	1.353
RPL3	-0.59	0.306	-1.929	0.054	0.555	0.17	-2.628	0.009	0.305	1.01
RPL29	-0.912	0.321	-2.844	0.004	0.402	0.129	-4.644	0	0.214	0.753
RPL27A	1.041	0.328	3.178	0.001	2.833	0.928	1.975	0.048	1.491	5.385
RPL27	0.171	0.298	0.573	0.566	1.187	0.354	0.527	0.598	0.661	2.129
RPL26L1	0.677	0.306	2.213	0.027	1.968	0.602	1.608	0.108	1.08	3.584
RPL24	0.08	0.295	0.27	0.787	1.083	0.32	0.26	0.795	0.607	1.932
RPL23AP53	-0.28	0.3	-0.936	0.349	0.755	0.226	-1.08	0.28	0.42	1.359
RPL23AP32	0.354	0.301	1.179	0.238	1.425	0.428	0.993	0.321	0.791	2.569
RPL23A	-0.218	0.303	-0.719	0.472	0.804	0.244	-0.803	0.422	0.444	1.456
RPL22	-0.579	0.311	-1.864	0.062	0.561	0.174	-2.525	0.012	0.305	1.03
RPL19	-0.369	0.3	-1.231	0.218	0.691	0.207	-1.489	0.136	0.384	1.244
RPL18	0.157	0.295	0.531	0.595	1.17	0.345	0.492	0.623	0.656	2.087
RPL15	0.034	0.296	0.114	0.909	1.034	0.306	0.112	0.911	0.579	1.846
RPL14	0.158	0.295	0.534	0.593	1.171	0.346	0.494	0.621	0.656	2.089
RPL12	-0.347	0.3	-1.157	0.247	0.707	0.212	-1.383	0.167	0.393	1.272
RPL11	-0.268	0.297	-0.902	0.367	0.765	0.227	-1.034	0.301	0.427	1.37
RPL10L	0.063	0.295	0.213	0.832	1.065	0.314	0.206	0.837	0.597	1.898
RPL10A	-0.095	0.297	-0.321	0.748	0.909	0.27	-0.337	0.736	0.508	1.628
RPIA	0.044	0.295	0.15	0.881	1.045	0.309	0.147	0.883	0.586	1.866
RPH3AL	-0.307	0.3	-1.023	0.306	0.736	0.22	-1.197	0.231	0.409	1.324
RPH3A	0.349	0.297	1.174	0.24	1.418	0.422	0.991	0.322	0.792	2.539
RPGRIP1L	0.447	0.298	1.497	0.134	1.563	0.466	1.207	0.227	0.871	2.806
RPGRIP1	-0.092	0.296	-0.31	0.757	0.912	0.27	-0.325	0.745	0.511	1.63
RPGR	0.162	0.296	0.546	0.585	1.176	0.349	0.504	0.614	0.658	2.102
RPF1	-0.161	0.303	-0.533	0.594	0.851	0.258	-0.578	0.563	0.47	1.54
RPE65	-0.312	0.298	-1.046	0.296	0.732	0.218	-1.227	0.22	0.408	1.313
RPE	-0.075	0.295	-0.253	0.801	0.928	0.274	-0.262	0.793	0.52	1.656
RPARP-AS1	0.018	0.295	0.06	0.952	1.018	0.3	0.06	0.952	0.571	1.815
RPAP3	-0.102	0.296	-0.346	0.73	0.903	0.267	-0.364	0.716	0.505	1.613
RPAP2	0.558	0.303	1.845	0.065	1.747	0.529	1.414	0.157	0.966	3.162
RPAP1	0.222	0.298	0.744	0.457	1.249	0.372	0.668	0.504	0.696	2.24
RPAIN	-0.101	0.297	-0.341	0.733	0.904	0.268	-0.359	0.72	0.505	1.617
RPA4	-0.182	0.296	-0.614	0.539	0.834	0.247	-0.674	0.5	0.466	1.49
RPA3	0.394	0.298	1.322	0.186	1.482	0.441	1.093	0.274	0.827	2.657
RPA2	0.159	0.296	0.537	0.591	1.172	0.346	0.497	0.619	0.657	2.092
RPA1	-0.07	0.296	-0.237	0.813	0.932	0.276	-0.246	0.806	0.521	1.666
RP2	0.27	0.297	0.911	0.362	1.31	0.389	0.798	0.425	0.732	2.345

ROS1	-0.414	0.3	-1.38	0.168	0.661	0.198	-1.709	0.087	0.368	1.19
RORC	-0.939	0.315	-2.977	0.003	0.391	0.123	-4.936	0	0.211	0.726
RORB	0.021	0.295	0.071	0.943	1.021	0.301	0.07	0.944	0.573	1.821
RORA	-0.214	0.299	-0.716	0.474	0.807	0.242	-0.799	0.424	0.449	1.451
ROR2	-0.481	0.302	-1.59	0.112	0.618	0.187	-2.041	0.041	0.342	1.118
ROR1	0.02	0.295	0.069	0.945	1.021	0.301	0.068	0.945	0.572	1.821
ROM1	0.27	0.297	0.907	0.364	1.309	0.389	0.795	0.427	0.731	2.344
ROGDI	0.023	0.296	0.079	0.937	1.024	0.303	0.078	0.938	0.573	1.828
ROCK2	-0.032	0.295	-0.107	0.915	0.969	0.286	-0.109	0.914	0.543	1.729
ROCK1	0.085	0.295	0.286	0.775	1.088	0.321	0.275	0.784	0.61	1.941
ROBO4	-0.369	0.298	-1.239	0.215	0.691	0.206	-1.499	0.134	0.385	1.24
ROBO3	-0.496	0.302	-1.64	0.101	0.609	0.184	-2.123	0.034	0.337	1.102
ROBO1	-0.387	0.298	-1.299	0.194	0.679	0.202	-1.587	0.113	0.378	1.218
RO60	0.745	0.31	2.404	0.016	2.107	0.653	1.695	0.09	1.147	3.868
RNPS1	-0.074	0.295	-0.251	0.802	0.929	0.274	-0.261	0.794	0.52	1.657
RNPEPL1	0.265	0.298	0.891	0.373	1.304	0.388	0.783	0.434	0.728	2.336
RNPEP	-0.024	0.295	-0.081	0.935	0.976	0.288	-0.082	0.935	0.547	1.741
RNMT	-0.058	0.295	-0.198	0.843	0.943	0.279	-0.204	0.838	0.529	1.683
RNLS	-0.061	0.296	-0.205	0.838	0.941	0.279	-0.211	0.833	0.526	1.683
RNH1	-0.303	0.3	-1.009	0.313	0.739	0.222	-1.179	0.239	0.41	1.33
RNGTT	-0.171	0.297	-0.574	0.566	0.843	0.251	-0.626	0.531	0.471	1.509
RNFT2	0.204	0.296	0.687	0.492	1.226	0.363	0.622	0.534	0.686	2.191
RNFT1	-0.496	0.303	-1.638	0.101	0.609	0.184	-2.122	0.034	0.336	1.102
RNF8	0.395	0.3	1.32	0.187	1.485	0.445	1.09	0.276	0.826	2.671
RNF7	-0.018	0.295	-0.062	0.95	0.982	0.29	-0.063	0.95	0.551	1.751
RNF6	0.197	0.298	0.661	0.509	1.218	0.363	0.6	0.549	0.679	2.184
RNF5	-0.545	0.302	-1.808	0.071	0.58	0.175	-2.404	0.016	0.321	1.047
RNF44	-0.002	0.295	-0.008	0.994	0.998	0.295	-0.008	0.994	0.559	1.78
RNF43	-0.068	0.296	-0.229	0.819	0.935	0.276	-0.236	0.813	0.524	1.668
RNF41	-0.236	0.298	-0.793	0.428	0.79	0.235	-0.895	0.371	0.441	1.415
RNF40	-0.601	0.303	-1.982	0.047	0.548	0.166	-2.717	0.007	0.303	0.993
RNF4	-0.501	0.302	-1.659	0.097	0.606	0.183	-2.154	0.031	0.335	1.095
RNF39	-0.399	0.298	-1.337	0.181	0.671	0.2	-1.643	0.1	0.374	1.204
RNF38	-0.302	0.298	-1.012	0.312	0.74	0.22	-1.181	0.238	0.412	1.327
RNF34	0.078	0.296	0.265	0.791	1.082	0.32	0.254	0.799	0.605	1.933
RNF32	0.326	0.298	1.095	0.274	1.385	0.412	0.934	0.35	0.773	2.482
RNF25	-0.175	0.299	-0.585	0.559	0.84	0.251	-0.639	0.523	0.467	1.508
RNF24	-0.112	0.296	-0.377	0.707	0.894	0.265	-0.398	0.69	0.5	1.599

RNF220	-0.337	0.298	-1.133	0.257	0.714	0.212	-1.347	0.178	0.398	1.279
RNF216	-0.588	0.303	-1.94	0.052	0.555	0.168	-2.641	0.008	0.307	1.006
RNF208	-0.203	0.296	-0.686	0.492	0.816	0.242	-0.761	0.446	0.456	1.459
RNF2	0.444	0.301	1.476	0.14	1.559	0.469	1.192	0.233	0.865	2.81
RNF19B	-0.631	0.304	-2.078	0.038	0.532	0.162	-2.897	0.004	0.293	0.965
RNF19A	-0.155	0.298	-0.52	0.603	0.857	0.255	-0.563	0.574	0.478	1.535
RNF187	0.072	0.295	0.245	0.806	1.075	0.317	0.236	0.813	0.603	1.917
RNF186	0.203	0.296	0.687	0.492	1.226	0.363	0.622	0.534	0.686	2.19
RNF185-AS1	-0.342	0.299	-1.144	0.253	0.71	0.213	-1.364	0.173	0.395	1.277
RNF170	-0.333	0.3	-1.109	0.268	0.717	0.215	-1.316	0.188	0.398	1.291
RNF17	-0.099	0.296	-0.336	0.737	0.905	0.268	-0.353	0.724	0.507	1.616
RNF167	0.239	0.296	0.806	0.42	1.27	0.376	0.717	0.473	0.711	2.269
RNF146	0.054	0.296	0.183	0.854	1.056	0.313	0.179	0.858	0.591	1.887
RNF144A	-0.18	0.296	-0.61	0.542	0.835	0.247	-0.668	0.504	0.468	1.49
RNF141	0.318	0.297	1.071	0.284	1.374	0.408	0.918	0.359	0.768	2.459
RNF14	-0.425	0.307	-1.386	0.166	0.654	0.2	-1.727	0.084	0.359	1.192
RNF139	0.64	0.31	2.066	0.039	1.897	0.588	1.525	0.127	1.033	3.482
RNF138	-0.077	0.296	-0.259	0.795	0.926	0.274	-0.27	0.787	0.518	1.655
RNF130	-0.342	0.298	-1.149	0.251	0.71	0.211	-1.37	0.171	0.396	1.273
RNF13	-0.101	0.296	-0.341	0.733	0.904	0.268	-0.359	0.719	0.505	1.616
RNF128	0.336	0.298	1.129	0.259	1.4	0.417	0.959	0.337	0.781	2.508
RNF126P1	0.111	0.295	0.374	0.708	1.117	0.33	0.354	0.723	0.626	1.993
RNF126	0.69	0.307	2.246	0.025	1.993	0.612	1.622	0.105	1.092	3.64
RNF125	0.062	0.295	0.21	0.834	1.064	0.314	0.203	0.839	0.596	1.898
RNF123	-0.503	0.302	-1.663	0.096	0.605	0.183	-2.161	0.031	0.334	1.094
RNF122	-0.01	0.296	-0.034	0.973	0.99	0.293	-0.034	0.973	0.554	1.769
RNF121	0.155	0.296	0.524	0.6	1.168	0.346	0.486	0.627	0.654	2.088
RNF115	0.547	0.303	1.806	0.071	1.728	0.523	1.392	0.164	0.955	3.127
RNF114	0.41	0.303	1.356	0.175	1.507	0.456	1.112	0.266	0.833	2.728
RNF113A	0.155	0.299	0.517	0.605	1.167	0.349	0.479	0.632	0.65	2.096
RNF111	-0.69	0.31	-2.226	0.026	0.502	0.155	-3.206	0.001	0.273	0.921
RNF11	-0.341	0.298	-1.146	0.252	0.711	0.212	-1.366	0.172	0.397	1.274
RNF103	-0.481	0.302	-1.59	0.112	0.618	0.187	-2.041	0.041	0.342	1.118
RNF10	-0.135	0.3	-0.449	0.653	0.874	0.262	-0.481	0.63	0.486	1.573
RND3	0.078	0.296	0.263	0.793	1.081	0.32	0.253	0.8	0.605	1.933
RND2	1.037	0.319	3.253	0.001	2.822	0.9	2.025	0.043	1.51	5.272
RND1	0.192	0.296	0.65	0.516	1.212	0.359	0.591	0.555	0.678	2.166
RNASET2	-0.402	0.3	-1.342	0.18	0.669	0.2	-1.651	0.099	0.372	1.204

RNASEL	-0.826	0.311	-2.652	0.008	0.438	0.136	-4.124	0	0.238	0.806
RNASEH2B	-0.388	0.3	-1.295	0.195	0.679	0.203	-1.581	0.114	0.377	1.221
RNASEH2A	0.506	0.3	1.686	0.092	1.658	0.497	1.323	0.186	0.921	2.984
RNASEH1	0.252	0.296	0.852	0.394	1.287	0.381	0.753	0.451	0.72	2.3
RNASE6	-0.543	0.306	-1.777	0.076	0.581	0.178	-2.36	0.018	0.319	1.058
RNASE4	-0.615	0.31	-1.984	0.047	0.541	0.168	-2.741	0.006	0.295	0.993
RNASE3	-0.789	0.311	-2.539	0.011	0.455	0.141	-3.865	0	0.247	0.835
RNASE2	0.095	0.296	0.322	0.748	1.1	0.326	0.307	0.759	0.615	1.966
RNASE1	0.338	0.297	1.141	0.254	1.403	0.416	0.968	0.333	0.784	2.509
RMND5B	-0.119	0.296	-0.403	0.687	0.887	0.263	-0.428	0.668	0.497	1.586
RMND5A	-0.278	0.297	-0.934	0.35	0.758	0.225	-1.076	0.282	0.423	1.357
RMND1	-0.108	0.296	-0.363	0.717	0.898	0.266	-0.383	0.701	0.503	1.605
RMI1	0.195	0.296	0.658	0.51	1.215	0.36	0.598	0.55	0.68	2.173
RMDN3	0.063	0.295	0.214	0.83	1.065	0.315	0.207	0.836	0.597	1.901
RMDN1	0.073	0.296	0.245	0.806	1.075	0.318	0.237	0.813	0.602	1.919
RMC1	0.597	0.302	1.973	0.049	1.816	0.549	1.486	0.137	1.004	3.285
RLN2	0.233	0.296	0.785	0.433	1.262	0.374	0.7	0.484	0.706	2.256
RLN1	0.556	0.303	1.837	0.066	1.744	0.528	1.409	0.159	0.963	3.158
RLF	0.008	0.295	0.027	0.978	1.008	0.298	0.027	0.978	0.565	1.798
RLBP1	0.142	0.297	0.477	0.633	1.152	0.342	0.445	0.656	0.644	2.06
RITA1	0.279	0.298	0.937	0.349	1.322	0.394	0.818	0.414	0.737	2.37
RIT2	0.294	0.296	0.993	0.321	1.342	0.397	0.86	0.39	0.751	2.396
RIT1	0.637	0.304	2.099	0.036	1.892	0.574	1.552	0.121	1.043	3.43
RIPPLY3	0.196	0.297	0.659	0.51	1.216	0.361	0.599	0.549	0.68	2.175
RIPOR2	0.057	0.295	0.194	0.846	1.059	0.313	0.189	0.85	0.594	1.889
RIPOR1	-0.317	0.297	-1.067	0.286	0.728	0.217	-1.255	0.209	0.407	1.304
RIPK4	0.44	0.298	1.477	0.14	1.553	0.463	1.195	0.232	0.866	2.786
RIPK2	0.806	0.31	2.6	0.009	2.24	0.695	1.785	0.074	1.22	4.113
RIPK1	-0.497	0.301	-1.654	0.098	0.608	0.183	-2.142	0.032	0.337	1.096
RIOX2	0.065	0.298	0.217	0.828	1.067	0.318	0.21	0.834	0.595	1.914
RIOX1	0.058	0.295	0.196	0.845	1.06	0.313	0.191	0.849	0.594	1.89
RIOK3	-0.325	0.298	-1.091	0.275	0.722	0.215	-1.29	0.197	0.403	1.295
RIOK2	-0.246	0.301	-0.817	0.414	0.782	0.235	-0.926	0.354	0.434	1.41
RINT1	-0.192	0.296	-0.65	0.516	0.825	0.244	-0.716	0.474	0.462	1.474
RING1	-0.263	0.297	-0.885	0.376	0.769	0.229	-1.013	0.311	0.429	1.377
RIN3	0.418	0.3	1.393	0.163	1.518	0.455	1.139	0.255	0.844	2.732
RIN2	-0.536	0.302	-1.772	0.076	0.585	0.177	-2.345	0.019	0.324	1.058
RIN1	0.66	0.306	2.157	0.031	1.935	0.592	1.579	0.114	1.062	3.524

RIMS3	-0.133	0.296	-0.447	0.655	0.876	0.26	-0.478	0.633	0.49	1.566
RIMS2	0.012	0.295	0.041	0.967	1.012	0.299	0.041	0.968	0.568	1.805
RIMS1	-0.05	0.296	-0.169	0.866	0.951	0.281	-0.173	0.863	0.533	1.698
RIMBP2	0.421	0.3	1.406	0.16	1.524	0.456	1.147	0.251	0.847	2.741
RIF1	0.663	0.306	2.17	0.03	1.941	0.593	1.586	0.113	1.066	3.532
RIDA	0.441	0.299	1.478	0.14	1.555	0.464	1.195	0.232	0.866	2.792
RIC8B	-0.153	0.298	-0.513	0.608	0.858	0.255	-0.555	0.579	0.479	1.538
RIC3	-0.663	0.31	-2.138	0.033	0.516	0.16	-3.032	0.002	0.281	0.946
RIBC2	1.026	0.318	3.226	0.001	2.79	0.887	2.017	0.044	1.496	5.204
RHPN1-AS1	0.733	0.315	2.326	0.02	2.081	0.655	1.649	0.099	1.122	3.858
RHOT2	-0.386	0.299	-1.29	0.197	0.68	0.203	-1.575	0.115	0.379	1.221
RHOT1	0.337	0.298	1.131	0.258	1.401	0.417	0.96	0.337	0.781	2.512
RHOQ	-0.625	0.304	-2.059	0.04	0.535	0.162	-2.86	0.004	0.295	0.97
RHOH	-0.854	0.315	-2.712	0.007	0.426	0.134	-4.283	0	0.23	0.789
RHOG	-0.398	0.298	-1.335	0.182	0.672	0.2	-1.64	0.101	0.374	1.205
RHOF	-0.438	0.299	-1.465	0.143	0.645	0.193	-1.838	0.066	0.359	1.159
RHOD	0.071	0.295	0.239	0.811	1.073	0.317	0.231	0.817	0.602	1.915
RHOBTB3	-0.032	0.296	-0.109	0.913	0.968	0.286	-0.111	0.912	0.542	1.729
RHOBTB2	-0.168	0.296	-0.567	0.571	0.845	0.25	-0.618	0.537	0.473	1.51
RHOBTB1	-0.059	0.295	-0.2	0.842	0.943	0.278	-0.206	0.837	0.529	1.681
RHOB	-0.249	0.298	-0.835	0.404	0.78	0.232	-0.948	0.343	0.435	1.397
RHOA	-0.802	0.315	-2.549	0.011	0.448	0.141	-3.911	0	0.242	0.831
RHO	0.625	0.304	2.058	0.04	1.868	0.567	1.53	0.126	1.03	3.386
RHEB	0.443	0.3	1.477	0.14	1.557	0.467	1.193	0.233	0.865	2.801
RHD	-0.447	0.302	-1.479	0.139	0.639	0.193	-1.865	0.062	0.354	1.156
RHCG	0.354	0.3	1.18	0.238	1.425	0.427	0.994	0.32	0.791	2.565
RHBG	0.051	0.295	0.173	0.863	1.052	0.31	0.169	0.866	0.59	1.876
RHBDL1	0.106	0.295	0.36	0.719	1.112	0.328	0.341	0.733	0.623	1.984
RHBDF2	0.088	0.295	0.298	0.765	1.092	0.323	0.286	0.775	0.612	1.949
RHBDF1	0.135	0.298	0.452	0.652	1.144	0.341	0.422	0.673	0.638	2.052
RHBDD3	0.063	0.296	0.212	0.832	1.065	0.315	0.205	0.837	0.596	1.901
RHAG	0.096	0.297	0.322	0.748	1.1	0.327	0.307	0.759	0.615	1.969
RGSL1	-0.976	0.322	-3.031	0.002	0.377	0.121	-5.135	0	0.201	0.708
RGS9	0.37	0.298	1.243	0.214	1.448	0.432	1.039	0.299	0.808	2.597
RGS7	0.228	0.296	0.772	0.44	1.256	0.372	0.69	0.49	0.704	2.243
RGS6	-0.025	0.295	-0.084	0.933	0.975	0.288	-0.085	0.932	0.547	1.74
RGS5	-0.722	0.31	-2.329	0.02	0.486	0.151	-3.414	0.001	0.265	0.892
RGS4	-0.351	0.298	-1.176	0.239	0.704	0.21	-1.409	0.159	0.392	1.263

RGS3	0.293	0.298	0.986	0.324	1.341	0.399	0.854	0.393	0.748	2.403
RGS20	0.151	0.295	0.51	0.61	1.163	0.343	0.474	0.636	0.652	2.075
RGS2	-0.055	0.295	-0.188	0.851	0.946	0.279	-0.193	0.847	0.53	1.687
RGS19	0.384	0.3	1.283	0.199	1.469	0.44	1.065	0.287	0.817	2.642
RGS17	-0.292	0.296	-0.985	0.325	0.747	0.221	-1.144	0.253	0.418	1.335
RGS16	-0.365	0.3	-1.218	0.223	0.694	0.208	-1.469	0.142	0.386	1.249
RGS14	0.027	0.296	0.092	0.927	1.027	0.304	0.09	0.928	0.575	1.835
RGS13	-0.35	0.298	-1.177	0.239	0.704	0.21	-1.41	0.159	0.393	1.262
RGS12	0.378	0.298	1.268	0.205	1.459	0.435	1.056	0.291	0.814	2.618
RGS11	0.261	0.298	0.878	0.38	1.298	0.386	0.773	0.44	0.725	2.327
RGS10	-0.432	0.303	-1.427	0.154	0.649	0.196	-1.785	0.074	0.359	1.175
RGS1	-0.346	0.3	-1.153	0.249	0.708	0.212	-1.378	0.168	0.393	1.274
RGR	0.339	0.3	1.131	0.258	1.403	0.42	0.96	0.337	0.78	2.525
RGP1	0.248	0.296	0.838	0.402	1.282	0.38	0.742	0.458	0.717	2.291
RGN	-0.209	0.296	-0.707	0.48	0.811	0.24	-0.786	0.432	0.454	1.449
RGL2	-0.39	0.298	-1.309	0.191	0.677	0.202	-1.601	0.109	0.377	1.214
RGL1	-0.42	0.298	-1.41	0.159	0.657	0.196	-1.752	0.08	0.366	1.178
RGCC	-0.664	0.31	-2.142	0.032	0.515	0.16	-3.042	0.002	0.28	0.945
RFXAP	0.656	0.307	2.137	0.033	1.926	0.591	1.568	0.117	1.056	3.515
RFXANK	0.6	0.302	1.986	0.047	1.822	0.55	1.494	0.135	1.008	3.292
RFX7	0	0.296	-0.001	1	1	0.296	-0.001	1	0.56	1.786
RFX5	0.375	0.298	1.257	0.209	1.455	0.434	1.048	0.294	0.811	2.61
RFX4	0.742	0.315	2.355	0.019	2.1	0.661	1.663	0.096	1.132	3.893
RFX3	0.313	0.3	1.044	0.296	1.368	0.41	0.897	0.37	0.76	2.462
RFX2	0.01	0.295	0.035	0.972	1.01	0.298	0.035	0.972	0.566	1.802
RFX1	0.264	0.296	0.891	0.373	1.302	0.385	0.783	0.433	0.729	2.326
RFWD3	0.538	0.302	1.778	0.075	1.712	0.518	1.375	0.169	0.947	3.098
RFTN1	-0.701	0.306	-2.292	0.022	0.496	0.152	-3.322	0.001	0.272	0.903
RFPL3S	0.566	0.303	1.869	0.062	1.76	0.533	1.428	0.153	0.973	3.186
RFPL3	-0.122	0.3	-0.407	0.684	0.885	0.266	-0.433	0.665	0.492	1.593
RFPL2	-0.134	0.296	-0.453	0.651	0.875	0.259	-0.484	0.628	0.49	1.561
RFPL1S	-0.414	0.298	-1.387	0.165	0.661	0.197	-1.718	0.086	0.369	1.186
RFPL1	0.487	0.3	1.625	0.104	1.628	0.488	1.286	0.198	0.904	2.93
RFNG	0.116	0.295	0.393	0.694	1.123	0.332	0.371	0.711	0.63	2.003
RFK	-0.389	0.302	-1.288	0.198	0.677	0.205	-1.574	0.115	0.374	1.225
RFC5	0.579	0.3	1.929	0.054	1.784	0.536	1.464	0.143	0.991	3.214
RFC4	0.789	0.31	2.546	0.011	2.201	0.682	1.761	0.078	1.199	4.039
RFC3	0.68	0.306	2.223	0.026	1.974	0.604	1.613	0.107	1.084	3.595

RFC2	0.481	0.3	1.606	0.108	1.618	0.485	1.275	0.202	0.899	2.91
RFC1	0.121	0.295	0.41	0.682	1.129	0.333	0.386	0.699	0.633	2.014
REXO5	0.101	0.296	0.341	0.733	1.106	0.328	0.325	0.745	0.619	1.977
REXO4	-0.041	0.296	-0.139	0.89	0.96	0.285	-0.142	0.887	0.537	1.716
REXO2	-0.265	0.298	-0.891	0.373	0.767	0.228	-1.02	0.307	0.428	1.375
REX1BD	0.132	0.297	0.444	0.657	1.141	0.339	0.416	0.678	0.638	2.041
REV3L	-0.123	0.296	-0.416	0.678	0.884	0.262	-0.442	0.658	0.495	1.58
REV1	0.457	0.303	1.511	0.131	1.579	0.478	1.213	0.225	0.873	2.858
RETSAT	-0.432	0.3	-1.44	0.15	0.649	0.195	-1.801	0.072	0.361	1.169
RETREG3	0.247	0.298	0.831	0.406	1.281	0.381	0.736	0.462	0.715	2.295
RETREG2	0.055	0.296	0.186	0.852	1.057	0.313	0.181	0.856	0.591	1.887
RETREG1	0.123	0.296	0.414	0.679	1.13	0.335	0.39	0.697	0.633	2.02
RETN	0.371	0.3	1.238	0.216	1.449	0.434	1.034	0.301	0.806	2.606
RET	-0.25	0.298	-0.84	0.401	0.779	0.232	-0.954	0.34	0.435	1.396
REST	0.236	0.298	0.794	0.427	1.267	0.377	0.707	0.48	0.706	2.271
RESF1	-0.132	0.296	-0.445	0.656	0.877	0.259	-0.476	0.634	0.491	1.565
RERGL	-0.19	0.296	-0.641	0.521	0.827	0.245	-0.706	0.48	0.463	1.478
RERE	-0.462	0.302	-1.528	0.126	0.63	0.19	-1.942	0.052	0.349	1.139
RER1	-1.106	0.328	-3.37	0.001	0.331	0.109	-6.162	0	0.174	0.63
REPS2	0.311	0.297	1.044	0.296	1.364	0.406	0.898	0.369	0.762	2.444
REPS1	-0.224	0.297	-0.757	0.449	0.799	0.237	-0.848	0.396	0.447	1.429
REPIN1	-0.395	0.3	-1.318	0.187	0.674	0.202	-1.616	0.106	0.374	1.212
RENBP	0.049	0.295	0.165	0.869	1.05	0.31	0.161	0.872	0.589	1.872
REN	-0.398	0.3	-1.328	0.184	0.672	0.201	-1.632	0.103	0.373	1.208
REM1	-0.057	0.295	-0.192	0.848	0.945	0.279	-0.197	0.844	0.53	1.686
RELN	-0.128	0.295	-0.435	0.664	0.88	0.26	-0.464	0.643	0.493	1.569
RELB	-0.269	0.3	-0.897	0.37	0.764	0.229	-1.029	0.303	0.424	1.376
RELA	-0.232	0.296	-0.782	0.434	0.793	0.235	-0.88	0.379	0.444	1.418
REL	-0.2	0.299	-0.671	0.503	0.819	0.244	-0.742	0.458	0.456	1.47
REG3A	0.209	0.299	0.699	0.485	1.232	0.368	0.63	0.528	0.686	2.212
REG1CP	-0.42	0.302	-1.391	0.164	0.657	0.198	-1.729	0.084	0.363	1.188
REG1B	-0.204	0.297	-0.687	0.492	0.816	0.242	-0.762	0.446	0.456	1.459
REG1A	-0.167	0.296	-0.564	0.573	0.846	0.251	-0.614	0.539	0.474	1.512
REEP5	-0.126	0.296	-0.426	0.67	0.881	0.261	-0.454	0.65	0.493	1.575
REEP4	0.042	0.296	0.141	0.888	1.043	0.308	0.138	0.89	0.584	1.862
REEP2	0.195	0.296	0.658	0.511	1.215	0.36	0.598	0.55	0.68	2.171
REEP1	-0.521	0.311	-1.678	0.093	0.594	0.184	-2.203	0.028	0.323	1.092
RECQL5	0.042	0.296	0.142	0.887	1.043	0.308	0.139	0.889	0.584	1.861

RECQL4	0.737	0.31	2.38	0.017	2.09	0.647	1.684	0.092	1.139	3.836
RECQL	-0.236	0.296	-0.797	0.425	0.79	0.234	-0.899	0.369	0.442	1.41
RECK	0.342	0.298	1.147	0.251	1.407	0.419	0.971	0.331	0.785	2.524
REC8	0.03	0.296	0.1	0.92	1.03	0.305	0.098	0.922	0.577	1.84
RDX	-0.456	0.3	-1.517	0.129	0.634	0.19	-1.922	0.055	0.352	1.142
RDH8	0.293	0.297	0.989	0.323	1.341	0.398	0.857	0.391	0.75	2.399
RDH16	0.361	0.298	1.211	0.226	1.434	0.427	1.017	0.309	0.8	2.572
RDH11	-0.291	0.298	-0.978	0.328	0.747	0.222	-1.135	0.256	0.417	1.339
RCVRN	0.475	0.306	1.554	0.12	1.608	0.492	1.237	0.216	0.883	2.928
RCOR3	0.455	0.299	1.52	0.129	1.576	0.472	1.221	0.222	0.877	2.833
RCOR1	0.009	0.297	0.03	0.976	1.009	0.299	0.03	0.976	0.564	1.805
RCN3	-0.856	0.315	-2.717	0.007	0.425	0.134	-4.295	0	0.229	0.788
RCN2	0.001	0.296	0.002	0.998	1.001	0.296	0.002	0.998	0.561	1.786
RCN1	0.068	0.297	0.23	0.818	1.07	0.318	0.222	0.824	0.599	1.914
RCL1	-0.252	0.299	-0.844	0.399	0.777	0.232	-0.96	0.337	0.433	1.396
RCHY1	-0.064	0.298	-0.214	0.831	0.938	0.279	-0.221	0.825	0.524	1.682
RCE1	-0.247	0.298	-0.829	0.407	0.781	0.233	-0.941	0.347	0.436	1.4
RCC1L	0.031	0.296	0.104	0.917	1.031	0.305	0.103	0.918	0.578	1.842
RCC1	0.594	0.306	1.943	0.052	1.811	0.553	1.465	0.143	0.995	3.297
RCBTB2	-0.356	0.298	-1.194	0.232	0.701	0.209	-1.434	0.152	0.391	1.256
RCBTB1	0.078	0.296	0.263	0.793	1.081	0.319	0.253	0.801	0.606	1.929
RCAN3	0.476	0.3	1.586	0.113	1.609	0.483	1.262	0.207	0.894	2.897
RCAN2	-0.637	0.306	-2.082	0.037	0.529	0.162	-2.911	0.004	0.291	0.963
RCAN1	0.015	0.296	0.052	0.958	1.016	0.3	0.052	0.959	0.569	1.813
RC3H2	0.278	0.296	0.937	0.349	1.32	0.391	0.818	0.413	0.739	2.359
RC3H1	0.347	0.3	1.156	0.248	1.414	0.424	0.977	0.329	0.786	2.546
RBX1	0.513	0.302	1.698	0.09	1.671	0.505	1.328	0.184	0.924	3.021
RBPMS	-0.035	0.297	-0.117	0.907	0.966	0.287	-0.119	0.906	0.54	1.728
RBPJL	-0.438	0.3	-1.46	0.144	0.645	0.193	-1.832	0.067	0.359	1.161
RBPJ	-0.549	0.301	-1.826	0.068	0.577	0.174	-2.433	0.015	0.32	1.041
RBP4	0.082	0.295	0.279	0.78	1.086	0.321	0.268	0.789	0.609	1.937
RBP3	0.46	0.3	1.532	0.126	1.584	0.475	1.228	0.219	0.879	2.851
RBP1	0.496	0.302	1.643	0.1	1.641	0.495	1.296	0.195	0.909	2.965
RBMXL2	0.094	0.3	0.313	0.754	1.098	0.329	0.299	0.765	0.611	1.976
RBMX2	0.66	0.306	2.161	0.031	1.936	0.592	1.581	0.114	1.063	3.524
RBMS3	-0.468	0.299	-1.567	0.117	0.626	0.187	-1.998	0.046	0.349	1.125
RBMS2	-0.383	0.3	-1.28	0.201	0.682	0.204	-1.559	0.119	0.379	1.226
RBMS1	-0.01	0.298	-0.034	0.973	0.99	0.295	-0.034	0.973	0.552	1.775

RBM8A	0.126	0.295	0.427	0.67	1.134	0.335	0.401	0.688	0.636	2.024
RBM7	-0.623	0.306	-2.038	0.042	0.536	0.164	-2.828	0.005	0.295	0.976
RBM6	-0.258	0.297	-0.869	0.385	0.772	0.23	-0.991	0.322	0.431	1.383
RBM5	-0.638	0.306	-2.086	0.037	0.528	0.162	-2.918	0.004	0.29	0.962
RBM4B	-0.202	0.296	-0.682	0.495	0.817	0.242	-0.755	0.45	0.457	1.46
RBM48	0.085	0.295	0.287	0.774	1.088	0.321	0.275	0.783	0.61	1.942
RBM47	-0.027	0.296	-0.09	0.928	0.974	0.288	-0.091	0.927	0.545	1.74
RBM42	0.004	0.296	0.012	0.99	1.004	0.297	0.012	0.99	0.562	1.794
RBM41	0.009	0.295	0.032	0.974	1.01	0.298	0.032	0.975	0.566	1.801
RBM39	-0.158	0.297	-0.531	0.596	0.854	0.254	-0.575	0.565	0.477	1.529
RBM38	0.564	0.306	1.846	0.065	1.758	0.538	1.41	0.158	0.966	3.201
RBM34	0.176	0.297	0.593	0.553	1.193	0.354	0.544	0.586	0.666	2.134
RBM3	-0.34	0.3	-1.135	0.256	0.712	0.213	-1.352	0.176	0.396	1.28
RBM28	0.631	0.306	2.065	0.039	1.879	0.574	1.531	0.126	1.033	3.421
RBM26	-0.087	0.296	-0.294	0.769	0.917	0.271	-0.307	0.758	0.513	1.637
RBM25	-0.138	0.296	-0.468	0.64	0.871	0.257	-0.502	0.616	0.488	1.554
RBM23	0.217	0.296	0.732	0.464	1.242	0.368	0.658	0.51	0.695	2.219
RBM22	0.049	0.295	0.166	0.868	1.05	0.31	0.162	0.872	0.589	1.873
RBM19	-0.063	0.295	-0.215	0.83	0.939	0.277	-0.222	0.825	0.527	1.673
RBM17	0.317	0.298	1.066	0.286	1.373	0.409	0.913	0.361	0.766	2.461
RBM15B	0.408	0.303	1.348	0.178	1.504	0.456	1.106	0.269	0.831	2.725
RBM15	0.469	0.302	1.552	0.121	1.598	0.483	1.239	0.215	0.884	2.89
RBM14	0.203	0.296	0.685	0.493	1.225	0.363	0.62	0.535	0.686	2.189
RBM12B-AS1	0.122	0.295	0.414	0.679	1.13	0.334	0.39	0.697	0.634	2.016
RBM12B	-0.266	0.297	-0.896	0.37	0.767	0.228	-1.026	0.305	0.429	1.371
RBM12	0.659	0.306	2.152	0.031	1.933	0.592	1.576	0.115	1.06	3.522
RBM10	0.074	0.296	0.249	0.803	1.076	0.318	0.24	0.81	0.603	1.922
RBL2	-0.449	0.3	-1.494	0.135	0.639	0.192	-1.885	0.059	0.354	1.15
RBL1	0.676	0.31	2.184	0.029	1.966	0.609	1.587	0.112	1.072	3.607
RBKS	-0.43	0.307	-1.401	0.161	0.651	0.2	-1.75	0.08	0.357	1.187
RBFOX2	-0.588	0.301	-1.95	0.051	0.556	0.167	-2.654	0.008	0.308	1.003
RBFOX1	0.35	0.297	1.176	0.24	1.419	0.422	0.992	0.321	0.792	2.541
RBFADN	-0.082	0.295	-0.277	0.782	0.922	0.272	-0.288	0.773	0.517	1.644
RBFA	0.405	0.298	1.362	0.173	1.5	0.446	1.12	0.263	0.837	2.688
RBCK1	0.018	0.295	0.059	0.953	1.018	0.301	0.059	0.953	0.57	1.816
RBBP9	-0.112	0.296	-0.379	0.705	0.894	0.265	-0.401	0.688	0.5	1.597
RBBP8	-0.181	0.298	-0.609	0.543	0.834	0.248	-0.667	0.504	0.465	1.495
RBBP7	0.612	0.306	2.004	0.045	1.845	0.564	1.498	0.134	1.013	3.358

RBBP6	0.143	0.298	0.481	0.631	1.154	0.344	0.448	0.654	0.643	2.071
RBBP5	0.351	0.3	1.171	0.242	1.421	0.426	0.988	0.323	0.789	2.558
RBBP4	-0.431	0.3	-1.433	0.152	0.65	0.195	-1.791	0.073	0.361	1.171
RB1CC1	-0.107	0.296	-0.36	0.719	0.899	0.266	-0.38	0.704	0.503	1.606
RB1	0.102	0.295	0.345	0.73	1.107	0.327	0.328	0.743	0.62	1.976
RAX	0.146	0.295	0.493	0.622	1.157	0.342	0.459	0.646	0.648	2.064
RAVER2	-0.07	0.296	-0.237	0.812	0.932	0.276	-0.246	0.806	0.522	1.664
RASSF9	-0.058	0.296	-0.197	0.844	0.943	0.279	-0.203	0.839	0.528	1.686
RASSF8	0.271	0.297	0.915	0.36	1.312	0.389	0.802	0.423	0.734	2.346
RASSF7	-0.026	0.295	-0.087	0.93	0.975	0.288	-0.088	0.93	0.546	1.738
RASSF4	0.01	0.296	0.032	0.974	1.01	0.299	0.032	0.974	0.566	1.803
RASSF2	-0.748	0.307	-2.439	0.015	0.473	0.145	-3.629	0	0.259	0.863
RASSF1	-0.647	0.311	-2.082	0.037	0.524	0.163	-2.928	0.003	0.285	0.963
RASL12	-0.119	0.297	-0.401	0.688	0.888	0.264	-0.426	0.67	0.496	1.589
RASL11B	-0.073	0.295	-0.247	0.805	0.93	0.274	-0.256	0.798	0.521	1.658
RASL10A	0.012	0.295	0.04	0.968	1.012	0.298	0.039	0.969	0.568	1.804
RASIP1	-0.171	0.296	-0.577	0.564	0.843	0.25	-0.629	0.529	0.472	1.506
RASGRP3	-0.36	0.298	-1.208	0.227	0.698	0.208	-1.454	0.146	0.389	1.251
RASGRP2	0.286	0.298	0.962	0.336	1.332	0.397	0.836	0.403	0.743	2.387
RASGRP1	0.296	0.298	0.995	0.32	1.345	0.4	0.861	0.389	0.75	2.409
RASGRF1	0.252	0.297	0.85	0.395	1.287	0.382	0.751	0.453	0.719	2.304
RASAL2	0.094	0.296	0.317	0.751	1.099	0.325	0.303	0.762	0.615	1.963
RASAL1	0.352	0.303	1.161	0.246	1.421	0.431	0.979	0.328	0.785	2.574
RASA3	-0.294	0.298	-0.988	0.323	0.745	0.222	-1.148	0.251	0.416	1.336
RASA2	0.265	0.298	0.889	0.374	1.303	0.388	0.781	0.435	0.727	2.335
RASA1	0.266	0.298	0.893	0.372	1.304	0.388	0.784	0.433	0.728	2.337
RARS2	0.116	0.297	0.39	0.697	1.123	0.333	0.368	0.713	0.628	2.008
RARS1	-0.404	0.302	-1.336	0.182	0.668	0.202	-1.646	0.1	0.369	1.208
RARRES2	-0.497	0.302	-1.645	0.1	0.608	0.184	-2.132	0.033	0.336	1.1
RARRES1	0.114	0.3	0.381	0.703	1.121	0.336	0.36	0.719	0.623	2.017
RARG	-0.047	0.296	-0.158	0.874	0.954	0.282	-0.162	0.871	0.534	1.704
RARB	-0.368	0.298	-1.234	0.217	0.692	0.206	-1.492	0.136	0.386	1.242
RARA	0.664	0.306	2.17	0.03	1.942	0.594	1.586	0.113	1.066	3.538
RAPSN	0.372	0.298	1.247	0.212	1.45	0.432	1.042	0.297	0.809	2.599
RAPGEFL1	0.29	0.298	0.975	0.329	1.337	0.398	0.847	0.397	0.746	2.395
RAPGEF6	-0.376	0.3	-1.256	0.209	0.686	0.206	-1.525	0.127	0.382	1.235
RAPGEF5	0.372	0.298	1.251	0.211	1.451	0.432	1.044	0.296	0.81	2.6
RAPGEF4	-0.531	0.306	-1.737	0.082	0.588	0.18	-2.293	0.022	0.323	1.07

RAPGEF3	0.024	0.295	0.081	0.936	1.024	0.302	0.08	0.936	0.574	1.826
RAPGEF2	-0.285	0.297	-0.962	0.336	0.752	0.223	-1.113	0.266	0.42	1.345
RAPGEF1	-0.158	0.296	-0.534	0.593	0.854	0.253	-0.579	0.563	0.478	1.525
RAP2C	0.453	0.301	1.505	0.132	1.573	0.473	1.21	0.226	0.872	2.837
RAP2B	0.457	0.302	1.511	0.131	1.579	0.478	1.213	0.225	0.873	2.857
RAP2A	0.599	0.303	1.978	0.048	1.82	0.551	1.488	0.137	1.005	3.294
RAP1GDS1	0.446	0.298	1.496	0.135	1.561	0.465	1.207	0.227	0.871	2.8
RAP1GAP2	-0.098	0.296	-0.331	0.741	0.907	0.268	-0.348	0.728	0.508	1.619
RAP1GAP	-0.192	0.296	-0.648	0.517	0.825	0.244	-0.715	0.475	0.462	1.475
RAP1B	-0.003	0.297	-0.011	0.991	0.997	0.296	-0.011	0.991	0.556	1.785
RAP1A	-0.197	0.298	-0.66	0.509	0.822	0.245	-0.729	0.466	0.458	1.473
RANGRF	-0.141	0.296	-0.474	0.635	0.869	0.257	-0.509	0.611	0.486	1.553
RANGAP1	0.35	0.298	1.177	0.239	1.42	0.423	0.993	0.321	0.792	2.544
RANBP9	0.369	0.3	1.232	0.218	1.446	0.433	1.03	0.303	0.804	2.601
RANBP6	-0.115	0.297	-0.387	0.699	0.891	0.264	-0.41	0.681	0.498	1.594
RANBP3	-0.047	0.296	-0.16	0.873	0.954	0.282	-0.163	0.87	0.534	1.703
RANBP2	0.145	0.299	0.485	0.628	1.156	0.346	0.452	0.652	0.643	2.079
RANBP17	0.223	0.296	0.752	0.452	1.249	0.37	0.674	0.5	0.699	2.232
RANBP10	0.333	0.298	1.119	0.263	1.395	0.415	0.952	0.341	0.779	2.499
RANBP1	1.031	0.321	3.212	0.001	2.805	0.901	2.004	0.045	1.495	5.264
RAN	0.454	0.303	1.497	0.134	1.574	0.477	1.204	0.229	0.869	2.85
RAMP3	-0.056	0.295	-0.19	0.85	0.946	0.279	-0.195	0.845	0.53	1.686
RAMP2	-0.497	0.307	-1.622	0.105	0.608	0.186	-2.101	0.036	0.334	1.109
RAMP1	0.084	0.295	0.285	0.776	1.088	0.321	0.273	0.785	0.61	1.94
RALYL	0.255	0.297	0.858	0.391	1.291	0.384	0.757	0.449	0.72	2.312
RALY	0.399	0.3	1.331	0.183	1.49	0.447	1.097	0.273	0.828	2.682
RALGPS2	-0.366	0.298	-1.226	0.22	0.694	0.207	-1.481	0.139	0.387	1.244
RALGPS1	-0.307	0.3	-1.024	0.306	0.736	0.221	-1.199	0.231	0.409	1.324
RALGDS	-0.175	0.296	-0.592	0.554	0.839	0.249	-0.647	0.518	0.47	1.5
RALGAPB	-0.225	0.296	-0.76	0.447	0.798	0.236	-0.853	0.394	0.447	1.427
RALGAPA1	-0.571	0.304	-1.88	0.06	0.565	0.172	-2.536	0.011	0.312	1.025
RALBP1	-0.231	0.298	-0.776	0.437	0.794	0.236	-0.873	0.382	0.443	1.422
RALB	-0.034	0.295	-0.115	0.908	0.966	0.285	-0.117	0.906	0.542	1.723
RALA	1.033	0.321	3.218	0.001	2.811	0.903	2.006	0.045	1.498	5.274
RAI2	-0.432	0.307	-1.408	0.159	0.649	0.199	-1.761	0.078	0.356	1.185
RAI14	-0.581	0.306	-1.9	0.057	0.56	0.171	-2.576	0.01	0.307	1.018
RAI1	0.001	0.295	0.005	0.996	1.001	0.296	0.005	0.996	0.561	1.786
RAG2	-0.556	0.306	-1.818	0.069	0.574	0.175	-2.431	0.015	0.315	1.044

RAG1	-0.04	0.295	-0.135	0.893	0.961	0.284	-0.138	0.891	0.538	1.715
RAF1	-0.097	0.295	-0.328	0.743	0.908	0.268	-0.345	0.73	0.509	1.619
RAE1	1.056	0.328	3.223	0.001	2.875	0.942	1.99	0.047	1.513	5.463
RADX	-0.294	0.298	-0.988	0.323	0.745	0.222	-1.149	0.251	0.416	1.336
RAD9A	0.4	0.3	1.334	0.182	1.491	0.447	1.099	0.272	0.829	2.683
RAD54L2	0.694	0.308	2.256	0.024	2.002	0.616	1.627	0.104	1.095	3.66
RAD54L	1.053	0.321	3.276	0.001	2.865	0.921	2.026	0.043	1.526	5.38
RAD54B	0.399	0.3	1.332	0.183	1.49	0.446	1.098	0.272	0.829	2.681
RAD52	-0.076	0.296	-0.257	0.797	0.927	0.274	-0.268	0.789	0.519	1.654
RAD51D	-0.318	0.299	-1.062	0.288	0.728	0.218	-1.25	0.211	0.405	1.309
RAD51C	0.156	0.295	0.527	0.598	1.169	0.345	0.488	0.625	0.655	2.085
RAD51B	-0.231	0.301	-0.767	0.443	0.794	0.239	-0.862	0.388	0.44	1.432
RAD51AP1	0.978	0.321	3.051	0.002	2.66	0.853	1.946	0.052	1.419	4.986
RAD51	0.478	0.3	1.597	0.11	1.613	0.483	1.269	0.204	0.897	2.902
RAD50	-0.076	0.295	-0.259	0.796	0.926	0.273	-0.269	0.788	0.52	1.652
RAD23B	-0.157	0.296	-0.53	0.596	0.855	0.253	-0.574	0.566	0.479	1.526
RAD23A	0.097	0.297	0.328	0.743	1.102	0.327	0.313	0.755	0.616	1.971
RAD21L1	0.096	0.295	0.325	0.745	1.101	0.325	0.31	0.757	0.617	1.964
RAD21	0.765	0.31	2.47	0.014	2.15	0.666	1.726	0.084	1.171	3.945
RAD17	-0.146	0.296	-0.493	0.622	0.864	0.256	-0.531	0.595	0.483	1.545
RAD1	0.823	0.315	2.614	0.009	2.278	0.718	1.781	0.075	1.229	4.225
RACGAP1	0.844	0.315	2.683	0.007	2.327	0.732	1.812	0.07	1.256	4.312
RAC3	-0.009	0.295	-0.032	0.975	0.991	0.293	-0.032	0.974	0.555	1.768
RAC2	-0.572	0.303	-1.889	0.059	0.565	0.171	-2.549	0.011	0.312	1.022
RAC1	0.045	0.296	0.153	0.878	1.046	0.31	0.15	0.881	0.586	1.87
RABL6	-0.067	0.295	-0.227	0.821	0.935	0.276	-0.234	0.815	0.525	1.668
RABL3	-0.065	0.296	-0.22	0.826	0.937	0.277	-0.227	0.82	0.525	1.673
RABIF	0.322	0.296	1.085	0.278	1.379	0.409	0.928	0.353	0.772	2.466
RABGGTB	0.016	0.296	0.053	0.957	1.016	0.3	0.053	0.958	0.569	1.814
RABGGTA	0.015	0.295	0.051	0.959	1.015	0.3	0.051	0.96	0.569	1.811
RABGAP1L	-0.086	0.295	-0.292	0.77	0.917	0.271	-0.305	0.76	0.514	1.637
RABGAP1	-0.47	0.301	-1.561	0.119	0.625	0.188	-1.993	0.046	0.346	1.128
RABEPK	0.326	0.297	1.1	0.271	1.386	0.411	0.938	0.348	0.775	2.478
RABEP2	-0.088	0.295	-0.299	0.765	0.915	0.27	-0.313	0.754	0.513	1.633
RABEP1	-0.069	0.297	-0.231	0.817	0.934	0.277	-0.24	0.811	0.522	1.67
RABAC1	0.315	0.297	1.058	0.29	1.37	0.407	0.908	0.364	0.765	2.452
RAB9BP1	0.407	0.3	1.357	0.175	1.502	0.45	1.115	0.265	0.835	2.704
RAB9A	0.05	0.295	0.171	0.864	1.052	0.311	0.167	0.868	0.589	1.877

RAB8B	-0.07	0.297	-0.237	0.813	0.932	0.277	-0.245	0.806	0.521	1.669
RAB8A	0.297	0.298	0.995	0.32	1.346	0.402	0.861	0.389	0.75	2.415
RAB7A	-0.449	0.302	-1.485	0.138	0.638	0.193	-1.874	0.061	0.353	1.155
RAB6B	0.168	0.299	0.562	0.574	1.183	0.353	0.517	0.605	0.658	2.125
RAB5C	0.579	0.306	1.892	0.058	1.784	0.546	1.436	0.151	0.979	3.251
RAB5B	-0.656	0.307	-2.14	0.032	0.519	0.159	-3.025	0.002	0.284	0.946
RAB5A	0.387	0.298	1.299	0.194	1.472	0.438	1.077	0.281	0.821	2.639
RAB40C	-0.849	0.315	-2.697	0.007	0.428	0.135	-4.249	0	0.231	0.793
RAB40B	-0.344	0.298	-1.153	0.249	0.709	0.211	-1.376	0.169	0.395	1.272
RAB40AL	-0.379	0.3	-1.264	0.206	0.685	0.205	-1.537	0.124	0.381	1.232
RAB40A	0.271	0.297	0.913	0.361	1.311	0.389	0.8	0.424	0.733	2.346
RAB3IL1	-0.102	0.296	-0.346	0.73	0.903	0.267	-0.364	0.716	0.505	1.613
RAB3GAP2	0.416	0.303	1.375	0.169	1.516	0.459	1.125	0.261	0.838	2.743
RAB3GAP1	0.065	0.297	0.22	0.826	1.068	0.317	0.213	0.831	0.596	1.912
RAB3D	-0.113	0.296	-0.381	0.703	0.893	0.265	-0.404	0.687	0.5	1.597
RAB3B	0.167	0.296	0.565	0.572	1.182	0.35	0.52	0.603	0.661	2.114
RAB3A	-0.04	0.298	-0.136	0.892	0.96	0.286	-0.139	0.89	0.536	1.721
RAB38	0.386	0.297	1.298	0.194	1.471	0.437	1.077	0.282	0.821	2.634
RAB36	-0.356	0.3	-1.189	0.234	0.7	0.21	-1.429	0.153	0.389	1.26
RAB35	-0.013	0.297	-0.042	0.966	0.987	0.293	-0.043	0.966	0.552	1.767
RAB33B	-0.743	0.308	-2.41	0.016	0.476	0.147	-3.574	0	0.26	0.871
RAB33A	-0.327	0.3	-1.092	0.275	0.721	0.216	-1.293	0.196	0.401	1.297
RAB32	0.069	0.295	0.233	0.816	1.071	0.316	0.225	0.822	0.601	1.911
RAB31	-0.619	0.31	-1.999	0.046	0.538	0.167	-2.768	0.006	0.293	0.988
RAB30	-0.265	0.298	-0.892	0.372	0.767	0.228	-1.022	0.307	0.428	1.374
RAB2A	-0.078	0.296	-0.264	0.792	0.925	0.273	-0.275	0.783	0.518	1.651
RAB29	0.306	0.3	1.02	0.308	1.358	0.407	0.879	0.38	0.754	2.444
RAB28	0.139	0.296	0.471	0.638	1.15	0.34	0.439	0.66	0.643	2.054
RAB27B	-0.34	0.3	-1.132	0.258	0.712	0.214	-1.348	0.178	0.395	1.282
RAB27A	-0.381	0.3	-1.273	0.203	0.683	0.205	-1.549	0.121	0.379	1.229
RAB26	0.186	0.296	0.629	0.529	1.205	0.357	0.574	0.566	0.674	2.152
RAB25	-0.34	0.303	-1.121	0.262	0.712	0.216	-1.335	0.182	0.393	1.29
RAB23	-0.483	0.302	-1.598	0.11	0.617	0.187	-2.054	0.04	0.341	1.116
RAB22A	0.342	0.298	1.149	0.25	1.408	0.419	0.973	0.33	0.786	2.524
RAB21	-0.278	0.304	-0.915	0.36	0.757	0.23	-1.055	0.292	0.418	1.374
RAB20	0.044	0.295	0.148	0.882	1.045	0.309	0.145	0.885	0.586	1.864
RAB1B	0.059	0.295	0.201	0.84	1.061	0.313	0.195	0.845	0.595	1.893
RAB1A	-0.014	0.297	-0.049	0.961	0.986	0.293	-0.049	0.961	0.551	1.764

RAB17	-0.338	0.299	-1.131	0.258	0.713	0.213	-1.345	0.179	0.397	1.281
RAB15	0.298	0.298	1.003	0.316	1.348	0.401	0.867	0.386	0.752	2.414
RAB14	-0.769	0.315	-2.446	0.014	0.463	0.146	-3.682	0	0.25	0.858
RAB13	-0.192	0.298	-0.643	0.52	0.826	0.246	-0.709	0.478	0.461	1.48
RAB11FIP5	0.148	0.297	0.498	0.619	1.159	0.344	0.463	0.643	0.648	2.076
RAB11FIP4	0.203	0.296	0.685	0.494	1.225	0.363	0.62	0.535	0.685	2.189
RAB11FIP3	-0.336	0.298	-1.127	0.26	0.715	0.213	-1.339	0.18	0.399	1.282
RAB11FIP2	-0.491	0.302	-1.625	0.104	0.612	0.185	-2.099	0.036	0.338	1.106
RAB11FIP1	-0.968	0.329	-2.945	0.003	0.38	0.125	-4.967	0	0.199	0.723
RAB11B	0.226	0.296	0.763	0.445	1.254	0.372	0.683	0.494	0.701	2.242
RAB11A	-0.039	0.299	-0.129	0.897	0.962	0.288	-0.132	0.895	0.535	1.729
R3HDM4	-0.326	0.298	-1.093	0.275	0.722	0.215	-1.291	0.197	0.403	1.295
R3HDM2	-0.5	0.303	-1.652	0.099	0.607	0.184	-2.143	0.032	0.335	1.098
R3HDM1	0.296	0.298	0.993	0.321	1.344	0.4	0.859	0.39	0.75	2.409
R3HCC1L	-0.038	0.295	-0.13	0.897	0.962	0.284	-0.132	0.895	0.54	1.716
R3HCC1	0.223	0.296	0.753	0.451	1.25	0.37	0.675	0.5	0.7	2.232
QTRT2	0.52	0.3	1.734	0.083	1.682	0.504	1.352	0.176	0.934	3.028
QTRT1	-0.116	0.298	-0.389	0.697	0.89	0.266	-0.412	0.68	0.496	1.598
QSOX1	-0.321	0.3	-1.069	0.285	0.726	0.218	-1.261	0.207	0.403	1.306
QSER1	0.138	0.296	0.465	0.642	1.147	0.339	0.435	0.664	0.643	2.048
QRSL1	-0.356	0.299	-1.193	0.233	0.7	0.209	-1.434	0.152	0.39	1.257
QRICH1	0.254	0.298	0.854	0.393	1.289	0.384	0.754	0.451	0.72	2.31
QPRT	0.815	0.315	2.588	0.01	2.258	0.711	1.77	0.077	1.219	4.185
QPCTL	-0.06	0.295	-0.204	0.839	0.942	0.278	-0.21	0.834	0.528	1.68
QPCT	-0.06	0.296	-0.204	0.838	0.941	0.278	-0.21	0.833	0.527	1.681
QKI	-0.029	0.297	-0.099	0.921	0.971	0.288	-0.101	0.92	0.543	1.738
QDPR	-0.788	0.321	-2.452	0.014	0.455	0.146	-3.729	0	0.242	0.854
PZP	0.527	0.306	1.724	0.085	1.694	0.518	1.34	0.18	0.93	3.084
PYY2	0.621	0.304	2.045	0.041	1.862	0.566	1.523	0.128	1.026	3.377
PYY	0.181	0.296	0.611	0.541	1.198	0.355	0.559	0.576	0.671	2.142
PYROXD1	-0.774	0.315	-2.457	0.014	0.461	0.145	-3.71	0	0.249	0.855
PYHIN1	0.063	0.297	0.212	0.832	1.065	0.317	0.206	0.837	0.595	1.908
PYGO1	-0.457	0.301	-1.52	0.128	0.633	0.19	-1.927	0.054	0.351	1.141
PYGM	-0.563	0.303	-1.859	0.063	0.57	0.172	-2.496	0.013	0.315	1.031
PYGL	-0.775	0.315	-2.464	0.014	0.461	0.145	-3.723	0	0.249	0.853
PYGB	0.275	0.297	0.925	0.355	1.316	0.391	0.809	0.419	0.735	2.355
PYCR3	0.012	0.296	0.04	0.968	1.012	0.299	0.04	0.968	0.567	1.807
PYCR1	0.282	0.298	0.946	0.344	1.325	0.394	0.825	0.41	0.74	2.374

PYCARD	0.266	0.297	0.898	0.369	1.305	0.387	0.788	0.431	0.73	2.335
PXN	-0.173	0.297	-0.584	0.559	0.841	0.25	-0.638	0.523	0.47	1.504
PXMP4	-0.346	0.297	-1.165	0.244	0.708	0.21	-1.392	0.164	0.395	1.266
PXMP2	1.094	0.321	3.403	0.001	2.986	0.96	2.069	0.039	1.59	5.608
PXDN	0.167	0.298	0.561	0.575	1.182	0.352	0.517	0.605	0.659	2.119
PXDC1	-0.026	0.296	-0.089	0.929	0.974	0.288	-0.09	0.928	0.546	1.738
PWWP3A	-0.28	0.298	-0.939	0.348	0.756	0.225	-1.084	0.279	0.422	1.355
PWWP2A	-0.338	0.3	-1.125	0.26	0.713	0.214	-1.339	0.181	0.396	1.285
PWP2	0.432	0.3	1.443	0.149	1.541	0.462	1.171	0.241	0.856	2.772
PWP1	0.329	0.298	1.104	0.27	1.389	0.414	0.94	0.347	0.775	2.492
PWAR5	0.569	0.306	1.862	0.063	1.766	0.54	1.42	0.156	0.97	3.215
PVT1	0.257	0.297	0.868	0.386	1.293	0.384	0.765	0.444	0.723	2.313
PVRIG	-0.976	0.321	-3.037	0.002	0.377	0.121	-5.146	0	0.201	0.707
PVR	0.026	0.296	0.087	0.93	1.026	0.304	0.086	0.931	0.575	1.832
PVALB	-0.052	0.295	-0.176	0.86	0.949	0.28	-0.181	0.857	0.532	1.694
PUS7L	-0.09	0.295	-0.303	0.762	0.914	0.27	-0.317	0.751	0.513	1.631
PUS7	0.028	0.295	0.094	0.925	1.028	0.304	0.093	0.926	0.576	1.835
PUS3	-0.259	0.298	-0.868	0.385	0.772	0.23	-0.991	0.322	0.431	1.384
PUS1	-0.205	0.297	-0.691	0.489	0.815	0.242	-0.767	0.443	0.455	1.457
PURG	-0.093	0.296	-0.313	0.754	0.911	0.27	-0.328	0.743	0.51	1.627
PURA	-0.639	0.306	-2.09	0.037	0.528	0.161	-2.927	0.003	0.29	0.961
PUM3	0.534	0.302	1.765	0.078	1.705	0.516	1.368	0.171	0.943	3.085
PUM2	-0.065	0.296	-0.221	0.825	0.937	0.277	-0.228	0.82	0.525	1.672
PUM1	0.266	0.298	0.894	0.371	1.305	0.388	0.785	0.432	0.728	2.338
PUF60	0.751	0.315	2.383	0.017	2.118	0.667	1.676	0.094	1.143	3.928
PUDP	-0.216	0.298	-0.726	0.468	0.806	0.24	-0.81	0.418	0.45	1.444
PTX3	0.324	0.3	1.082	0.279	1.383	0.415	0.924	0.355	0.769	2.49
PTTG3P	0.676	0.306	2.209	0.027	1.965	0.601	1.606	0.108	1.079	3.579
PTTG2	-0.094	0.295	-0.318	0.751	0.91	0.269	-0.333	0.739	0.51	1.624
PTTG1IP	0.347	0.298	1.165	0.244	1.414	0.421	0.984	0.325	0.789	2.535
PTTG1	0.609	0.306	1.994	0.046	1.839	0.562	1.493	0.135	1.01	3.348
PTS	0.095	0.295	0.32	0.749	1.099	0.325	0.306	0.76	0.616	1.961
PTRH2	0.157	0.297	0.527	0.598	1.169	0.347	0.488	0.626	0.653	2.094
PTPRZ1	-0.063	0.295	-0.213	0.832	0.939	0.277	-0.22	0.826	0.527	1.675
PTPRU	0.321	0.304	1.057	0.291	1.378	0.419	0.904	0.366	0.76	2.5
PTPRT	-0.226	0.298	-0.757	0.449	0.798	0.238	-0.85	0.396	0.445	1.431
PTPRS	0.396	0.3	1.32	0.187	1.485	0.445	1.09	0.276	0.825	2.674
PTPRR	-0.371	0.302	-1.227	0.22	0.69	0.209	-1.486	0.137	0.382	1.248

PTPRO	0.009	0.297	0.03	0.976	1.009	0.3	0.03	0.976	0.564	1.806
PTPRN2	-0.166	0.299	-0.554	0.579	0.847	0.253	-0.603	0.547	0.472	1.522
PTPRN	0.319	0.3	1.063	0.288	1.376	0.412	0.911	0.363	0.764	2.476
PTPRM	-0.714	0.306	-2.332	0.02	0.49	0.15	-3.403	0.001	0.269	0.892
PTPRK	-0.12	0.296	-0.406	0.685	0.887	0.263	-0.431	0.666	0.496	1.585
PTPRJ	0.15	0.295	0.509	0.61	1.162	0.343	0.473	0.636	0.652	2.073
PTPRH	0.112	0.295	0.381	0.703	1.119	0.33	0.36	0.719	0.627	1.996
PTPRG	-0.411	0.302	-1.361	0.174	0.663	0.2	-1.683	0.092	0.366	1.199
PTPRF	0.089	0.295	0.302	0.762	1.093	0.323	0.289	0.772	0.613	1.95
PTPRE	-0.776	0.311	-2.5	0.012	0.46	0.143	-3.779	0	0.25	0.846
PTPRD	-0.487	0.302	-1.61	0.107	0.615	0.186	-2.073	0.038	0.34	1.112
PTPRCAP	-0.327	0.297	-1.101	0.271	0.721	0.214	-1.303	0.193	0.403	1.291
PTPRC	-0.281	0.296	-0.949	0.342	0.755	0.224	-1.096	0.273	0.422	1.349
PTPRB	-0.8	0.315	-2.541	0.011	0.45	0.141	-3.892	0	0.243	0.833
PTPRA	-0.439	0.3	-1.465	0.143	0.645	0.193	-1.839	0.066	0.358	1.16
PTPN9	-0.001	0.296	-0.002	0.998	0.999	0.295	-0.002	0.998	0.56	1.783
PTPN7	0.482	0.302	1.593	0.111	1.619	0.49	1.264	0.206	0.895	2.929
PTPN6	-0.475	0.302	-1.572	0.116	0.622	0.188	-2.013	0.044	0.344	1.124
PTPN4	0.442	0.3	1.476	0.14	1.556	0.466	1.193	0.233	0.865	2.798
PTPN3	-0.597	0.306	-1.951	0.051	0.551	0.168	-2.669	0.008	0.302	1.003
PTPN22	-0.465	0.3	-1.55	0.121	0.628	0.188	-1.973	0.048	0.349	1.131
PTPN21	0.145	0.296	0.49	0.624	1.156	0.342	0.456	0.649	0.647	2.065
PTPN20	0.084	0.295	0.283	0.777	1.087	0.321	0.272	0.786	0.609	1.94
PTPN2	0.385	0.301	1.278	0.201	1.469	0.442	1.061	0.289	0.814	2.65
PTPN18	0.013	0.296	0.043	0.966	1.013	0.3	0.042	0.966	0.567	1.81
PTPN14	0.363	0.302	1.202	0.23	1.438	0.435	1.008	0.314	0.795	2.601
PTPN13	-0.304	0.298	-1.021	0.307	0.738	0.22	-1.193	0.233	0.412	1.323
PTPN12	-0.044	0.295	-0.149	0.881	0.957	0.283	-0.153	0.879	0.536	1.707
PTPN11	0.041	0.296	0.138	0.89	1.042	0.308	0.135	0.892	0.583	1.86
PTPN1	0.155	0.295	0.524	0.6	1.168	0.345	0.486	0.627	0.654	2.083
PTPA	-0.061	0.296	-0.205	0.837	0.941	0.278	-0.212	0.832	0.527	1.68
PTP4A3	-0.053	0.295	-0.18	0.857	0.948	0.28	-0.185	0.853	0.531	1.691
PTP4A2	-0.322	0.298	-1.078	0.281	0.725	0.216	-1.271	0.204	0.404	1.301
PTP4A1	0.093	0.295	0.314	0.753	1.097	0.324	0.3	0.764	0.615	1.957
PTOV1-AS2	0.038	0.295	0.128	0.898	1.038	0.307	0.125	0.9	0.582	1.852
PTOV1	-0.086	0.297	-0.29	0.772	0.918	0.272	-0.303	0.762	0.513	1.642
PTN	0.098	0.295	0.332	0.74	1.103	0.326	0.316	0.752	0.618	1.968
PTMS	0.158	0.296	0.534	0.593	1.171	0.347	0.494	0.621	0.656	2.093

PTMA	-0.109	0.296	-0.369	0.712	0.896	0.266	-0.39	0.696	0.502	1.602
PTK7	-0.118	0.296	-0.399	0.69	0.889	0.263	-0.424	0.672	0.498	1.586
PTK6	0.07	0.295	0.239	0.811	1.073	0.317	0.23	0.818	0.602	1.913
PTK2B	-0.201	0.299	-0.673	0.501	0.818	0.244	-0.745	0.456	0.456	1.469
PTK2	0.706	0.31	2.279	0.023	2.025	0.627	1.635	0.102	1.104	3.715
PTHLH	-0.922	0.321	-2.87	0.004	0.398	0.128	-4.713	0	0.212	0.747
PTH2R	-0.243	0.296	-0.82	0.412	0.784	0.232	-0.928	0.354	0.439	1.402
PTH1R	-0.589	0.306	-1.927	0.054	0.555	0.17	-2.624	0.009	0.305	1.01
PTH	0.091	0.296	0.309	0.758	1.096	0.324	0.295	0.768	0.614	1.955
PTGS2	-0.173	0.296	-0.583	0.56	0.841	0.249	-0.637	0.524	0.471	1.503
PTGS1	-0.498	0.302	-1.646	0.1	0.608	0.184	-2.133	0.033	0.336	1.1
PTGIS	-0.691	0.306	-2.257	0.024	0.501	0.153	-3.252	0.001	0.275	0.913
PTGIR	-0.164	0.296	-0.552	0.581	0.849	0.252	-0.6	0.548	0.475	1.518
PTGFR	-0.256	0.296	-0.863	0.388	0.774	0.229	-0.983	0.326	0.433	1.384
PTGES3	-0.228	0.296	-0.767	0.443	0.797	0.236	-0.862	0.389	0.446	1.424
PTGES2	-0.14	0.296	-0.473	0.636	0.869	0.257	-0.508	0.611	0.486	1.553
PTGES	0.146	0.296	0.495	0.62	1.158	0.342	0.461	0.645	0.649	2.066
PTGER4P2-CDK2	-0.195	0.297	-0.655	0.512	0.823	0.245	-0.723	0.47	0.46	1.474
PTGER4	-0.32	0.297	-1.075	0.282	0.727	0.216	-1.267	0.205	0.406	1.301
PTGER3	-0.214	0.298	-0.719	0.472	0.807	0.241	-0.801	0.423	0.45	1.448
PTGER2	-0.034	0.296	-0.115	0.909	0.967	0.286	-0.117	0.907	0.541	1.726
PTGER1	0.023	0.296	0.078	0.938	1.023	0.302	0.077	0.938	0.573	1.827
PTGDS	-0.607	0.304	-1.999	0.046	0.545	0.165	-2.751	0.006	0.3	0.988
PTGDR2	-0.469	0.299	-1.568	0.117	0.626	0.187	-2.001	0.045	0.348	1.124
PTGDR	0.237	0.296	0.801	0.423	1.268	0.376	0.713	0.476	0.709	2.267
PTER	-0.359	0.307	-1.17	0.242	0.698	0.214	-1.408	0.159	0.383	1.274
PTENP1	0.069	0.297	0.232	0.817	1.071	0.318	0.224	0.823	0.599	1.916
PTEN	0.259	0.297	0.871	0.384	1.296	0.385	0.767	0.443	0.723	2.321
PTDSS2	-0.126	0.297	-0.425	0.671	0.882	0.261	-0.452	0.651	0.493	1.577
PTDSS1	0.338	0.3	1.127	0.26	1.402	0.421	0.957	0.339	0.779	2.524
PTCRA	0.092	0.299	0.306	0.759	1.096	0.328	0.293	0.77	0.61	1.971
PTCH2	0.252	0.298	0.844	0.398	1.286	0.384	0.747	0.455	0.717	2.307
PTCH1	-0.007	0.296	-0.025	0.98	0.993	0.294	-0.025	0.98	0.556	1.774
PTCD3	0.1	0.297	0.337	0.736	1.105	0.329	0.32	0.749	0.617	1.98
PTCD2	0.155	0.296	0.523	0.601	1.167	0.345	0.484	0.628	0.654	2.084
PTBP3	0.415	0.298	1.392	0.164	1.514	0.451	1.14	0.254	0.845	2.713
PTBP2	0.355	0.3	1.185	0.236	1.426	0.427	0.998	0.318	0.793	2.566
PTBP1	0.016	0.295	0.054	0.957	1.016	0.3	0.054	0.957	0.57	1.811

PTAFR	-0.558	0.302	-1.849	0.064	0.572	0.173	-2.477	0.013	0.317	1.034
PSTPIP2	0.27	0.296	0.913	0.361	1.31	0.388	0.8	0.424	0.733	2.341
PSTPIP1	-0.25	0.298	-0.837	0.403	0.779	0.232	-0.95	0.342	0.434	1.398
PSRC1	0.307	0.298	1.03	0.303	1.359	0.405	0.887	0.375	0.758	2.437
PSPN	0.173	0.298	0.58	0.562	1.189	0.354	0.532	0.594	0.663	2.133
PSPH	0.382	0.3	1.275	0.202	1.465	0.439	1.06	0.289	0.814	2.635
PSPC1	0.766	0.31	2.47	0.014	2.152	0.667	1.726	0.084	1.171	3.951
PSORS1C2	0.438	0.302	1.45	0.147	1.55	0.469	1.174	0.24	0.857	2.803
PSORS1C1	-0.121	0.298	-0.405	0.686	0.886	0.264	-0.43	0.667	0.494	1.59
PSMG2	0.146	0.298	0.491	0.623	1.158	0.345	0.457	0.648	0.645	2.076
PSMG1	0.746	0.31	2.406	0.016	2.108	0.653	1.696	0.09	1.148	3.869
PSMF1	0.16	0.296	0.539	0.59	1.173	0.347	0.499	0.618	0.657	2.095
PSME4	0.4	0.3	1.333	0.182	1.492	0.448	1.099	0.272	0.829	2.686
PSME3IP1	0.428	0.3	1.427	0.154	1.535	0.461	1.161	0.246	0.852	2.764
PSME3	0.133	0.296	0.449	0.654	1.142	0.339	0.42	0.674	0.639	2.042
PSME1	0.28	0.298	0.94	0.347	1.323	0.394	0.82	0.412	0.738	2.371
PSMD9	0.153	0.295	0.519	0.604	1.166	0.344	0.481	0.631	0.653	2.08
PSMD8	0.205	0.297	0.692	0.489	1.228	0.365	0.625	0.532	0.686	2.199
PSMD7	0.71	0.306	2.32	0.02	2.034	0.623	1.661	0.097	1.116	3.707
PSMD6	-0.108	0.296	-0.365	0.715	0.897	0.266	-0.386	0.7	0.502	1.604
PSMD5	-0.105	0.297	-0.353	0.724	0.901	0.267	-0.372	0.71	0.503	1.611
PSMD4	0.343	0.296	1.157	0.247	1.409	0.418	0.979	0.327	0.788	2.519
PSMD3	0.28	0.296	0.946	0.344	1.323	0.392	0.825	0.409	0.741	2.365
PSMD2	1.019	0.321	3.176	0.001	2.771	0.889	1.992	0.046	1.477	5.197
PSMD14	0.585	0.306	1.914	0.056	1.795	0.549	1.449	0.147	0.986	3.269
PSMD13	0.709	0.306	2.315	0.021	2.031	0.622	1.659	0.097	1.115	3.701
PSMD12	0.93	0.321	2.901	0.004	2.535	0.813	1.889	0.059	1.352	4.753
PSMD11	0.152	0.296	0.511	0.609	1.164	0.345	0.475	0.635	0.651	2.08
PSMD10	0.091	0.295	0.307	0.759	1.095	0.323	0.294	0.769	0.614	1.953
PSMD1	0.461	0.3	1.537	0.124	1.586	0.476	1.232	0.218	0.881	2.855
PSMC6	0.85	0.309	2.752	0.006	2.339	0.722	1.854	0.064	1.277	4.284
PSMC5	0.28	0.296	0.945	0.345	1.323	0.392	0.824	0.41	0.74	2.366
PSMC4	0.198	0.296	0.669	0.504	1.219	0.361	0.607	0.544	0.682	2.177
PSMC3IP	0.431	0.303	1.42	0.156	1.539	0.467	1.154	0.249	0.849	2.789
PSMC3	0.346	0.298	1.161	0.246	1.414	0.421	0.982	0.326	0.788	2.536
PSMC2	0.541	0.302	1.788	0.074	1.717	0.519	1.381	0.167	0.949	3.106
PSMC1	0.79	0.311	2.545	0.011	2.204	0.684	1.759	0.079	1.199	4.051
PSMB9	0.108	0.296	0.365	0.715	1.114	0.329	0.346	0.729	0.624	1.989

PSMB8	-0.097	0.296	-0.327	0.744	0.908	0.268	-0.343	0.731	0.509	1.62
PSMB7	0.808	0.31	2.602	0.009	2.243	0.696	1.785	0.074	1.221	4.12
PSMB6	0.46	0.298	1.545	0.122	1.585	0.473	1.238	0.216	0.884	2.843
PSMB5	0.442	0.298	1.483	0.138	1.556	0.464	1.199	0.231	0.868	2.79
PSMB4	0.225	0.296	0.759	0.448	1.252	0.371	0.68	0.497	0.701	2.237
PSMB3	0.83	0.31	2.676	0.007	2.293	0.711	1.819	0.069	1.249	4.21
PSMB2	0.942	0.316	2.983	0.003	2.566	0.81	1.932	0.053	1.381	4.765
PSMB10	-0.225	0.298	-0.754	0.451	0.799	0.238	-0.845	0.398	0.445	1.433
PSMB1	0.16	0.296	0.539	0.59	1.173	0.348	0.498	0.619	0.656	2.097
PSMA7	1.399	0.346	4.044	0	4.053	1.402	2.177	0.03	2.057	7.985
PSMA5	0.464	0.3	1.547	0.122	1.59	0.476	1.238	0.216	0.884	2.861
PSMA4	0.703	0.31	2.269	0.023	2.02	0.626	1.63	0.103	1.1	3.706
PSMA3	0.268	0.296	0.907	0.364	1.307	0.386	0.796	0.426	0.733	2.333
PSMA2	0.731	0.306	2.387	0.017	2.076	0.635	1.694	0.09	1.14	3.782
PSMA1	1.237	0.329	3.761	0	3.445	1.133	2.158	0.031	1.808	6.563
PSKH1	0.53	0.302	1.758	0.079	1.7	0.513	1.364	0.173	0.941	3.071
PSIP1	0.269	0.298	0.903	0.366	1.308	0.389	0.792	0.428	0.73	2.344
PSG9	-0.099	0.297	-0.334	0.738	0.906	0.269	-0.351	0.726	0.506	1.621
PSG7	-0.128	0.295	-0.433	0.665	0.88	0.26	-0.461	0.645	0.493	1.57
PSG6	0.027	0.296	0.09	0.928	1.027	0.304	0.089	0.929	0.575	1.836
PSG5	-0.304	0.297	-1.023	0.306	0.738	0.219	-1.195	0.232	0.413	1.321
PSG4	-0.357	0.298	-1.198	0.231	0.7	0.208	-1.44	0.15	0.391	1.254
PSG3	0.712	0.31	2.296	0.022	2.037	0.632	1.643	0.1	1.11	3.74
PSG2	0.245	0.297	0.822	0.411	1.277	0.38	0.73	0.466	0.713	2.287
PSG11	0.353	0.298	1.186	0.236	1.423	0.424	0.999	0.318	0.794	2.551
PSG1	0.261	0.298	0.877	0.381	1.298	0.386	0.772	0.44	0.725	2.326
PSENEN	0.531	0.301	1.767	0.077	1.701	0.511	1.371	0.17	0.944	3.066
PSEN2	0.228	0.296	0.769	0.442	1.256	0.372	0.688	0.491	0.703	2.243
PSEN1	-0.105	0.3	-0.351	0.726	0.9	0.27	-0.37	0.711	0.5	1.62
PSD4	-0.219	0.297	-0.739	0.46	0.803	0.238	-0.826	0.409	0.449	1.437
PSD3	-0.624	0.306	-2.043	0.041	0.536	0.164	-2.837	0.005	0.294	0.975
PSD	0.039	0.295	0.132	0.895	1.04	0.307	0.13	0.897	0.583	1.854
PSCA	0.283	0.301	0.941	0.347	1.327	0.4	0.819	0.413	0.736	2.395
PSAT1	0.065	0.297	0.218	0.827	1.067	0.317	0.211	0.833	0.596	1.911
PSAP	0.018	0.295	0.063	0.95	1.019	0.301	0.062	0.951	0.571	1.817
PRX	0.25	0.297	0.842	0.4	1.284	0.382	0.745	0.456	0.717	2.299
PRUNE2	-0.111	0.295	-0.376	0.707	0.895	0.264	-0.398	0.691	0.502	1.597
PRUNE1	0.006	0.295	0.019	0.985	1.006	0.297	0.019	0.985	0.564	1.794

PRTN3	-0.117	0.295	-0.397	0.691	0.889	0.263	-0.421	0.674	0.499	1.587
PRSS8	-0.463	0.3	-1.541	0.123	0.629	0.189	-1.96	0.05	0.349	1.134
PRSS53	0.004	0.296	0.012	0.99	1.004	0.297	0.012	0.99	0.562	1.793
PRSS50	0.239	0.297	0.807	0.42	1.27	0.377	0.718	0.473	0.71	2.271
PRSS3P2	0.179	0.296	0.605	0.545	1.196	0.354	0.554	0.579	0.67	2.137
PRSS3	0.046	0.296	0.157	0.875	1.047	0.31	0.153	0.878	0.587	1.87
PRSS23	-0.259	0.302	-0.857	0.391	0.772	0.233	-0.978	0.328	0.427	1.395
PRSS22	0.204	0.296	0.69	0.49	1.227	0.363	0.624	0.533	0.687	2.192
PRSS21	1.088	0.322	3.382	0.001	2.967	0.954	2.061	0.039	1.58	5.573
PRSS2	0.55	0.3	1.834	0.067	1.733	0.519	1.411	0.158	0.963	3.118
PRSS16	-0.201	0.298	-0.673	0.501	0.818	0.244	-0.745	0.456	0.456	1.468
PRSS12	-0.093	0.296	-0.313	0.754	0.912	0.269	-0.328	0.743	0.511	1.627
PRRX2	0.503	0.3	1.676	0.094	1.654	0.497	1.317	0.188	0.918	2.98
PRRX1	-0.93	0.316	-2.941	0.003	0.395	0.125	-4.852	0	0.212	0.733
PRRG4	-0.135	0.295	-0.458	0.647	0.873	0.258	-0.491	0.624	0.49	1.558
PRRG3	0.153	0.296	0.518	0.604	1.166	0.345	0.481	0.631	0.653	2.081
PRRG2	0.322	0.297	1.083	0.279	1.38	0.41	0.926	0.355	0.77	2.472
PRRG1	-0.011	0.297	-0.037	0.971	0.989	0.293	-0.037	0.971	0.553	1.769
PRRC2C	0.141	0.3	0.469	0.639	1.151	0.345	0.438	0.662	0.64	2.071
PRRC2B	-0.54	0.301	-1.795	0.073	0.583	0.175	-2.38	0.017	0.323	1.051
PRRC2A	0.101	0.296	0.341	0.733	1.106	0.327	0.324	0.746	0.62	1.974
PRRC1	0.347	0.3	1.157	0.247	1.414	0.424	0.978	0.328	0.786	2.544
PRR7	0.136	0.299	0.457	0.648	1.146	0.342	0.427	0.669	0.638	2.057
PRR5L	-0.962	0.321	-2.995	0.003	0.382	0.123	-5.033	0	0.204	0.717
PRR5	-0.712	0.307	-2.321	0.02	0.49	0.151	-3.385	0.001	0.269	0.895
PRR36	0.286	0.299	0.959	0.337	1.332	0.398	0.834	0.404	0.742	2.391
PRR34	0.373	0.301	1.238	0.216	1.452	0.437	1.033	0.301	0.805	2.62
PRR3	0.217	0.296	0.733	0.463	1.243	0.368	0.659	0.51	0.695	2.222
PRR16	-0.499	0.302	-1.651	0.099	0.607	0.184	-2.141	0.032	0.336	1.098
PRR15L	0.434	0.3	1.449	0.147	1.544	0.462	1.176	0.24	0.858	2.777
PRR14L	0.129	0.297	0.433	0.665	1.138	0.338	0.407	0.684	0.635	2.038
PRR14	0.201	0.296	0.68	0.497	1.223	0.362	0.616	0.538	0.684	2.186
PRR13	-0.112	0.295	-0.381	0.703	0.894	0.264	-0.403	0.687	0.501	1.594
PRR11	0.36	0.3	1.203	0.229	1.434	0.43	1.01	0.313	0.797	2.58
PRPSAP2	0.52	0.302	1.722	0.085	1.683	0.509	1.342	0.18	0.931	3.043
PRPSAP1	0.266	0.298	0.895	0.371	1.305	0.389	0.786	0.432	0.728	2.34
PRPS2	0.328	0.298	1.101	0.271	1.387	0.413	0.939	0.348	0.774	2.486
PRPS1L1	0.679	0.306	2.22	0.026	1.971	0.603	1.612	0.107	1.083	3.589

PRPS1	0.993	0.321	3.093	0.002	2.7	0.867	1.961	0.05	1.439	5.066
PRPH2	-0.229	0.296	-0.774	0.439	0.795	0.235	-0.87	0.384	0.445	1.421
PRPH	0.167	0.296	0.565	0.572	1.182	0.35	0.52	0.603	0.661	2.113
PRPF8	-0.223	0.297	-0.751	0.452	0.8	0.238	-0.842	0.4	0.447	1.432
PRPF6	0.13	0.295	0.441	0.66	1.139	0.336	0.413	0.68	0.638	2.032
PRPF4B	0.328	0.3	1.096	0.273	1.389	0.416	0.934	0.35	0.772	2.498
PRPF40A	0.458	0.3	1.529	0.126	1.582	0.474	1.226	0.22	0.879	2.847
PRPF4	0.158	0.295	0.533	0.594	1.171	0.346	0.494	0.622	0.656	2.088
PRPF39	0.145	0.296	0.491	0.624	1.156	0.342	0.457	0.648	0.647	2.066
PRPF38B	0.356	0.298	1.196	0.232	1.428	0.426	1.006	0.315	0.796	2.562
PRPF31	0.513	0.3	1.71	0.087	1.67	0.501	1.338	0.181	0.928	3.006
PRPF3	0.378	0.298	1.268	0.205	1.459	0.434	1.056	0.291	0.814	2.615
PRPF19	0.29	0.298	0.976	0.329	1.337	0.398	0.847	0.397	0.746	2.397
PRPF18	0.347	0.298	1.167	0.243	1.415	0.421	0.986	0.324	0.79	2.536
PROZ	0.341	0.3	1.139	0.255	1.407	0.422	0.965	0.334	0.782	2.531
PROX1	-0.128	0.295	-0.433	0.665	0.88	0.26	-0.462	0.644	0.493	1.57
PROSER1	-0.096	0.296	-0.323	0.747	0.909	0.269	-0.338	0.735	0.509	1.624
PROS1	-0.45	0.3	-1.503	0.133	0.637	0.191	-1.898	0.058	0.354	1.147
PRORY	0.263	0.296	0.887	0.375	1.3	0.385	0.78	0.435	0.728	2.324
PRORP	0.543	0.306	1.775	0.076	1.721	0.526	1.37	0.171	0.945	3.132
PROP1	0.059	0.295	0.199	0.843	1.06	0.313	0.193	0.847	0.594	1.892
PROM1	0.418	0.302	1.384	0.166	1.519	0.459	1.131	0.258	0.84	2.747
PRODH2	0.179	0.295	0.605	0.545	1.196	0.353	0.554	0.58	0.67	2.133
PROCR	0.03	0.296	0.101	0.919	1.03	0.305	0.1	0.921	0.577	1.842
PROC	-0.184	0.298	-0.618	0.537	0.832	0.248	-0.679	0.497	0.464	1.492
PRNP	-0.401	0.299	-1.343	0.179	0.669	0.2	-1.653	0.098	0.373	1.202
PRND	0.045	0.295	0.154	0.878	1.046	0.309	0.15	0.881	0.587	1.867
PRMT8	0.538	0.302	1.778	0.075	1.712	0.518	1.376	0.169	0.947	3.097
PRMT7	0.363	0.299	1.212	0.225	1.438	0.43	1.017	0.309	0.799	2.585
PRMT5	-0.205	0.298	-0.689	0.491	0.815	0.243	-0.765	0.445	0.454	1.46
PRMT3	0.298	0.296	1.005	0.315	1.347	0.399	0.869	0.385	0.754	2.406
PRMT2	-0.187	0.296	-0.633	0.527	0.829	0.246	-0.696	0.486	0.464	1.481
PRMT1	-0.036	0.295	-0.121	0.904	0.965	0.285	-0.123	0.902	0.541	1.721
PRM2	0.166	0.296	0.562	0.574	1.181	0.35	0.517	0.605	0.661	2.11
PRM1	0.143	0.298	0.479	0.632	1.153	0.343	0.446	0.655	0.644	2.066
PRLR	0.271	0.296	0.913	0.361	1.311	0.389	0.8	0.424	0.733	2.343
PRLH	0.406	0.302	1.343	0.179	1.5	0.453	1.104	0.27	0.83	2.713
PRL	0.03	0.296	0.101	0.92	1.03	0.305	0.099	0.921	0.577	1.839

PRKY	-0.055	0.296	-0.185	0.853	0.947	0.28	-0.19	0.849	0.53	1.692
PRKX	0.385	0.303	1.27	0.204	1.47	0.446	1.054	0.292	0.811	2.664
PRKRA	-0.393	0.303	-1.298	0.194	0.675	0.204	-1.59	0.112	0.373	1.222
PRKN	-0.505	0.302	-1.669	0.095	0.604	0.183	-2.17	0.03	0.334	1.092
PRKG2	0.413	0.302	1.364	0.173	1.511	0.457	1.118	0.264	0.835	2.733
PRKG1	0.051	0.295	0.172	0.864	1.052	0.311	0.167	0.867	0.59	1.876
PRKDC	0.892	0.321	2.776	0.005	2.44	0.784	1.837	0.066	1.3	4.58
PRKD3	0.045	0.295	0.151	0.88	1.046	0.309	0.148	0.883	0.586	1.866
PRKD2	-0.425	0.298	-1.423	0.155	0.654	0.195	-1.773	0.076	0.364	1.174
PRKD1	0.012	0.295	0.04	0.968	1.012	0.299	0.04	0.968	0.567	1.804
PRKCZ-AS1	-0.564	0.303	-1.861	0.063	0.569	0.172	-2.499	0.012	0.314	1.031
PRKCZ	-0.301	0.298	-1.013	0.311	0.74	0.22	-1.183	0.237	0.413	1.325
PRKCSH	-0.07	0.295	-0.238	0.812	0.932	0.275	-0.247	0.805	0.522	1.663
PRKCQ	-0.058	0.295	-0.195	0.845	0.944	0.279	-0.201	0.841	0.529	1.684
PRKCI	0.805	0.316	2.547	0.011	2.237	0.707	1.749	0.08	1.204	4.156
PRKCH	-0.675	0.303	-2.226	0.026	0.509	0.154	-3.179	0.001	0.281	0.923
PRKCG	0.177	0.295	0.601	0.548	1.194	0.353	0.55	0.582	0.669	2.131
PRKCE	-0.176	0.296	-0.596	0.551	0.838	0.248	-0.651	0.515	0.47	1.497
PRKCD	-0.356	0.298	-1.195	0.232	0.701	0.209	-1.436	0.151	0.391	1.256
PRKCB	-0.554	0.303	-1.827	0.068	0.575	0.174	-2.441	0.015	0.317	1.041
PRKCA	-0.444	0.3	-1.481	0.139	0.642	0.192	-1.863	0.062	0.357	1.154
PRKAR2B	-0.741	0.315	-2.352	0.019	0.477	0.15	-3.484	0	0.257	0.884
PRKAR2A	-0.291	0.296	-0.983	0.326	0.747	0.221	-1.141	0.254	0.418	1.336
PRKAR1B	0.238	0.298	0.799	0.424	1.269	0.378	0.711	0.477	0.707	2.276
PRKAR1A	-0.181	0.296	-0.611	0.541	0.834	0.247	-0.67	0.503	0.467	1.491
PRKAG2	0.033	0.295	0.111	0.912	1.033	0.305	0.109	0.913	0.579	1.843
PRKAG1	-0.567	0.31	-1.827	0.068	0.567	0.176	-2.457	0.014	0.309	1.042
PRKACG	0.344	0.298	1.156	0.248	1.411	0.42	0.978	0.328	0.787	2.531
PRKACB	-0.232	0.296	-0.783	0.434	0.793	0.235	-0.881	0.378	0.444	1.417
PRKACA	-0.207	0.296	-0.699	0.485	0.813	0.241	-0.776	0.438	0.455	1.453
PRKAB2	0.575	0.303	1.9	0.057	1.778	0.538	1.445	0.149	0.982	3.219
PRKAB1	-0.703	0.315	-2.233	0.026	0.495	0.156	-3.238	0.001	0.267	0.918
PRKAA2	-0.203	0.296	-0.685	0.493	0.816	0.242	-0.76	0.447	0.457	1.458
PRKAA1	-0.345	0.3	-1.15	0.25	0.708	0.212	-1.373	0.17	0.394	1.275
PRINS	0.151	0.298	0.506	0.613	1.163	0.346	0.469	0.639	0.648	2.084
PRIM2	0.143	0.297	0.481	0.63	1.154	0.342	0.448	0.654	0.645	2.064
PRIM1	0.248	0.296	0.836	0.403	1.281	0.379	0.74	0.459	0.717	2.289
PRICKLE3	-0.16	0.297	-0.538	0.59	0.852	0.253	-0.584	0.559	0.476	1.526

PRG4	0.217	0.296	0.731	0.465	1.242	0.368	0.657	0.511	0.695	2.22
PRG3	0.09	0.296	0.304	0.761	1.094	0.323	0.291	0.771	0.613	1.953
PRG2	-0.524	0.303	-1.729	0.084	0.592	0.179	-2.272	0.023	0.327	1.072
PRF1	-0.13	0.296	-0.441	0.659	0.878	0.26	-0.471	0.637	0.492	1.567
PREX2	-0.136	0.296	-0.459	0.646	0.873	0.258	-0.492	0.623	0.489	1.56
PREPL	-0.515	0.302	-1.702	0.089	0.598	0.181	-2.225	0.026	0.33	1.081
PREP	0.065	0.296	0.221	0.825	1.067	0.316	0.214	0.831	0.598	1.906
PRELP	-0.335	0.297	-1.128	0.259	0.716	0.212	-1.34	0.18	0.4	1.28
PRELID3B	0.245	0.298	0.822	0.411	1.278	0.381	0.729	0.466	0.713	2.291
PRELID3A	0.278	0.298	0.935	0.35	1.321	0.393	0.817	0.414	0.737	2.368
PREB	-0.219	0.296	-0.74	0.459	0.803	0.238	-0.827	0.408	0.449	1.436
PRDX6	0.826	0.315	2.623	0.009	2.283	0.719	1.786	0.074	1.232	4.232
PRDX4	0.023	0.296	0.078	0.938	1.023	0.303	0.077	0.938	0.573	1.828
PRDX3	-0.268	0.3	-0.894	0.372	0.765	0.229	-1.025	0.306	0.425	1.377
PRDX2	0.64	0.305	2.098	0.036	1.897	0.579	1.549	0.121	1.043	3.45
PRDX1	0.074	0.295	0.252	0.801	1.077	0.318	0.243	0.808	0.604	1.92
PRDM9	0.395	0.298	1.326	0.185	1.484	0.442	1.096	0.273	0.828	2.661
PRDM8	0.068	0.298	0.227	0.82	1.07	0.319	0.22	0.826	0.597	1.919
PRDM5	0.676	0.31	2.178	0.029	1.965	0.61	1.583	0.113	1.07	3.61
PRDM4	0.231	0.296	0.779	0.436	1.26	0.373	0.696	0.487	0.705	2.252
PRDM2	-0.739	0.315	-2.349	0.019	0.478	0.15	-3.477	0.001	0.258	0.885
PRDM16	-0.022	0.295	-0.075	0.941	0.978	0.289	-0.075	0.94	0.549	1.744
PRDM14	-0.214	0.298	-0.717	0.473	0.807	0.241	-0.799	0.424	0.45	1.449
PRDM13	0.333	0.298	1.117	0.264	1.395	0.416	0.95	0.342	0.778	2.502
PRDM12	0.987	0.328	3.012	0.003	2.683	0.879	1.914	0.056	1.412	5.099
PRDM11	-0.278	0.3	-0.926	0.354	0.757	0.227	-1.067	0.286	0.421	1.364
PRDM10	-0.221	0.296	-0.747	0.455	0.801	0.238	-0.836	0.403	0.448	1.433
PRDM1	0.551	0.3	1.835	0.067	1.735	0.521	1.411	0.158	0.963	3.125
PRCP	-0.444	0.3	-1.482	0.138	0.641	0.192	-1.866	0.062	0.356	1.154
PRCC	0.28	0.297	0.945	0.345	1.323	0.392	0.824	0.41	0.74	2.367
PRC1	0.911	0.315	2.894	0.004	2.486	0.782	1.899	0.058	1.342	4.606
PRB4	0.053	0.296	0.18	0.857	1.055	0.312	0.175	0.861	0.591	1.884
PRB3	0.662	0.303	2.185	0.029	1.939	0.587	1.598	0.11	1.071	3.511
PRB1	0.319	0.298	1.072	0.284	1.376	0.41	0.918	0.359	0.768	2.466
PRAMEF12	-0.001	0.296	-0.003	0.997	0.999	0.296	-0.003	0.997	0.559	1.786
PRAMEF11	0.041	0.296	0.138	0.89	1.042	0.309	0.135	0.892	0.583	1.861
PRAMEF10	0.251	0.296	0.848	0.397	1.285	0.381	0.75	0.453	0.719	2.297
PRAME	0.259	0.301	0.861	0.389	1.295	0.389	0.758	0.448	0.719	2.335

PRAF2	-0.367	0.303	-1.212	0.225	0.693	0.21	-1.465	0.143	0.383	1.254
PQBP1	-0.499	0.306	-1.633	0.102	0.607	0.186	-2.118	0.034	0.333	1.105
PPY2P	-0.255	0.298	-0.855	0.393	0.775	0.231	-0.974	0.33	0.432	1.39
PPY	0.21	0.298	0.705	0.481	1.234	0.367	0.636	0.525	0.688	2.211
PPWD1	0.015	0.296	0.051	0.959	1.015	0.301	0.051	0.959	0.568	1.814
PPT1	0.183	0.298	0.616	0.538	1.201	0.358	0.563	0.574	0.67	2.153
PPRC1	0.4	0.298	1.342	0.18	1.492	0.445	1.106	0.269	0.832	2.677
PPP6R3	0.144	0.297	0.484	0.629	1.155	0.343	0.451	0.652	0.645	2.067
PPP6R2	-0.008	0.298	-0.025	0.98	0.992	0.296	-0.025	0.98	0.553	1.781
PPP6R1	0.458	0.3	1.53	0.126	1.581	0.474	1.227	0.22	0.879	2.845
PPP6C	0.366	0.298	1.229	0.219	1.442	0.43	1.029	0.303	0.804	2.587
PPP5C	-0.308	0.3	-1.027	0.305	0.735	0.22	-1.202	0.229	0.409	1.323
PPP4R4	0.413	0.298	1.388	0.165	1.512	0.45	1.137	0.255	0.844	2.709
PPP4R3B	0.429	0.3	1.433	0.152	1.536	0.46	1.165	0.244	0.854	2.765
PPP4R3A	-0.2	0.299	-0.669	0.503	0.819	0.245	-0.741	0.459	0.455	1.471
PPP4R2	0.3	0.298	1.008	0.313	1.35	0.402	0.871	0.384	0.753	2.42
PPP4R1	-0.239	0.3	-0.795	0.426	0.788	0.236	-0.898	0.369	0.438	1.418
PPP4C	0.437	0.299	1.463	0.144	1.548	0.462	1.185	0.236	0.862	2.78
PPP3R1	0.289	0.298	0.969	0.332	1.335	0.398	0.842	0.4	0.744	2.395
PPP3CC	-0.511	0.311	-1.641	0.101	0.6	0.187	-2.142	0.032	0.326	1.104
PPP3CB	-0.282	0.298	-0.947	0.344	0.754	0.225	-1.094	0.274	0.421	1.352
PPP3CA	0.025	0.295	0.086	0.931	1.026	0.303	0.085	0.932	0.575	1.829
PPP2R5E	0.474	0.3	1.58	0.114	1.607	0.482	1.259	0.208	0.892	2.893
PPP2R5D	0.49	0.302	1.622	0.105	1.633	0.494	1.282	0.2	0.903	2.953
PPP2R5C	-0.337	0.3	-1.123	0.261	0.714	0.214	-1.335	0.182	0.397	1.285
PPP2R5B	0.165	0.295	0.56	0.575	1.18	0.348	0.516	0.606	0.661	2.105
PPP2R5A	-0.424	0.303	-1.401	0.161	0.655	0.198	-1.744	0.081	0.362	1.184
PPP2R3C	-0.31	0.298	-1.041	0.298	0.734	0.218	-1.221	0.222	0.409	1.314
PPP2R3A	0.444	0.302	1.47	0.142	1.559	0.471	1.187	0.235	0.862	2.819
PPP2R2D	-0.204	0.296	-0.689	0.491	0.815	0.242	-0.765	0.445	0.456	1.457
PPP2R2B	0.04	0.295	0.136	0.892	1.041	0.307	0.134	0.894	0.584	1.857
PPP2R2A	-0.012	0.295	-0.04	0.968	0.988	0.292	-0.04	0.968	0.554	1.763
PPP2R1B	-0.237	0.298	-0.794	0.427	0.789	0.235	-0.896	0.37	0.44	1.416
PPP2R1A	-0.217	0.296	-0.733	0.464	0.805	0.238	-0.818	0.413	0.45	1.438
PPP2CB	-0.437	0.302	-1.445	0.148	0.646	0.195	-1.812	0.07	0.357	1.168
PPP2CA	-0.06	0.297	-0.203	0.839	0.942	0.279	-0.209	0.835	0.527	1.684
PPP1R9A	0.121	0.296	0.409	0.683	1.129	0.334	0.385	0.7	0.632	2.014
PPP1R8	0.338	0.298	1.137	0.255	1.403	0.418	0.965	0.335	0.783	2.514

PPP1R7	-0.588	0.31	-1.897	0.058	0.556	0.172	-2.582	0.01	0.303	1.02
PPP1R3D	0.033	0.295	0.113	0.91	1.034	0.305	0.111	0.911	0.58	1.844
PPP1R3C	-0.54	0.302	-1.788	0.074	0.583	0.176	-2.371	0.018	0.322	1.053
PPP1R3A	0.198	0.298	0.665	0.506	1.219	0.363	0.603	0.547	0.68	2.184
PPP1R37	0.304	0.3	1.014	0.311	1.355	0.406	0.874	0.382	0.753	2.437
PPP1R2C	0.193	0.295	0.654	0.513	1.213	0.358	0.595	0.552	0.68	2.165
PPP1R26	-0.314	0.3	-1.046	0.296	0.731	0.219	-1.229	0.219	0.406	1.315
PPP1R2	0.387	0.298	1.298	0.194	1.473	0.439	1.076	0.282	0.821	2.643
PPP1R1A	0.13	0.295	0.439	0.661	1.138	0.336	0.412	0.681	0.638	2.031
PPP1R17	-0.167	0.296	-0.564	0.573	0.846	0.251	-0.614	0.539	0.473	1.512
PPP1R16B	-0.432	0.298	-1.447	0.148	0.649	0.194	-1.81	0.07	0.362	1.165
PPP1R15A	0.013	0.296	0.043	0.966	1.013	0.3	0.042	0.966	0.567	1.809
PPP1R14D	0.527	0.306	1.722	0.085	1.694	0.518	1.339	0.181	0.93	3.085
PPP1R14B	0.614	0.306	2.01	0.044	1.849	0.565	1.502	0.133	1.015	3.366
PPP1R13L	0.06	0.295	0.202	0.84	1.062	0.314	0.196	0.844	0.595	1.894
PPP1R13B	0.262	0.298	0.879	0.379	1.299	0.387	0.773	0.439	0.725	2.328
PPP1R12B	-0.701	0.304	-2.302	0.021	0.496	0.151	-3.335	0.001	0.273	0.901
PPP1R12A	0.072	0.296	0.243	0.808	1.075	0.318	0.235	0.815	0.602	1.918
PPP1R11	-0.206	0.296	-0.696	0.486	0.814	0.241	-0.773	0.439	0.455	1.454
PPP1R10	-0.317	0.3	-1.055	0.292	0.729	0.219	-1.241	0.215	0.404	1.312
PPP1CC	-0.417	0.3	-1.393	0.164	0.659	0.197	-1.728	0.084	0.366	1.185
PPP1CB	0.053	0.295	0.179	0.858	1.054	0.311	0.174	0.862	0.591	1.881
PPP1CA	0.185	0.295	0.625	0.532	1.203	0.355	0.571	0.568	0.674	2.145
PPOX	-0.173	0.298	-0.581	0.561	0.841	0.251	-0.634	0.526	0.468	1.509
PPME1	-0.036	0.296	-0.122	0.903	0.964	0.285	-0.125	0.901	0.54	1.722
PPM1H	0.251	0.298	0.844	0.399	1.286	0.383	0.746	0.455	0.717	2.304
PPM1G	0.253	0.296	0.855	0.393	1.288	0.382	0.755	0.45	0.721	2.302
PPM1F	0.184	0.299	0.615	0.539	1.202	0.36	0.562	0.574	0.669	2.161
PPM1E	-0.012	0.295	-0.042	0.967	0.988	0.292	-0.042	0.966	0.554	1.762
PPM1D	1.061	0.322	3.298	0.001	2.889	0.929	2.033	0.042	1.538	5.428
PPM1B	0.104	0.295	0.353	0.724	1.11	0.328	0.335	0.738	0.622	1.979
PPM1A	-0.021	0.301	-0.071	0.943	0.979	0.294	-0.072	0.943	0.543	1.765
PPL	0.285	0.3	0.952	0.341	1.33	0.399	0.828	0.408	0.739	2.394
PPIP5K2	0.041	0.296	0.138	0.891	1.042	0.309	0.135	0.893	0.583	1.862
PPIP5K1	-0.103	0.297	-0.346	0.729	0.902	0.268	-0.365	0.715	0.505	1.614
PPIL2	0.078	0.296	0.264	0.792	1.081	0.32	0.254	0.8	0.606	1.93
PPIH	0.661	0.305	2.166	0.03	1.937	0.591	1.585	0.113	1.065	3.522
PPIG	0.887	0.313	2.831	0.005	2.427	0.76	1.878	0.06	1.314	4.483

PPIF	0.337	0.298	1.133	0.257	1.401	0.417	0.962	0.336	0.782	2.511
PPIEL	-0.489	0.302	-1.617	0.106	0.613	0.185	-2.086	0.037	0.339	1.109
PPIE	0.309	0.296	1.043	0.297	1.362	0.404	0.897	0.37	0.762	2.435
PPID	0.437	0.302	1.444	0.149	1.547	0.468	1.17	0.242	0.856	2.799
PPIC	-0.14	0.295	-0.475	0.635	0.869	0.257	-0.51	0.61	0.487	1.551
PPIB	0.175	0.296	0.591	0.555	1.191	0.352	0.542	0.588	0.667	2.125
PPIA	0.195	0.296	0.658	0.511	1.215	0.36	0.598	0.55	0.68	2.17
PPFIBP2	-0.125	0.297	-0.421	0.674	0.883	0.262	-0.449	0.654	0.494	1.578
PPFIBP1	0.065	0.295	0.219	0.826	1.067	0.315	0.212	0.832	0.598	1.904
PPFIA4	0.659	0.306	2.157	0.031	1.933	0.591	1.58	0.114	1.062	3.519
PPFIA3	-0.437	0.302	-1.446	0.148	0.646	0.195	-1.813	0.07	0.357	1.168
PPFIA2	0.258	0.297	0.868	0.386	1.294	0.385	0.765	0.444	0.723	2.319
PPFIA1	0.657	0.303	2.166	0.03	1.928	0.585	1.588	0.112	1.065	3.494
PPEF2	-0.178	0.297	-0.6	0.548	0.837	0.248	-0.657	0.511	0.468	1.497
PPEF1	-0.478	0.302	-1.58	0.114	0.62	0.187	-2.025	0.043	0.343	1.122
PPDPF	-0.353	0.3	-1.177	0.239	0.703	0.211	-1.411	0.158	0.39	1.265
PPCS	-0.289	0.301	-0.961	0.336	0.749	0.225	-1.115	0.265	0.415	1.351
PPCDC	0.636	0.304	2.095	0.036	1.888	0.573	1.55	0.121	1.042	3.424
PPBPP2	0.181	0.296	0.611	0.541	1.199	0.355	0.559	0.576	0.67	2.143
PPBP	-0.396	0.3	-1.321	0.186	0.673	0.202	-1.621	0.105	0.374	1.211
PPAT	0.56	0.306	1.832	0.067	1.751	0.536	1.403	0.161	0.962	3.189
PPARGC1A	-0.153	0.295	-0.517	0.605	0.858	0.253	-0.559	0.576	0.481	1.531
PPARG	-0.562	0.306	-1.838	0.066	0.57	0.174	-2.465	0.014	0.313	1.038
PPARD	0.878	0.321	2.739	0.006	2.406	0.771	1.823	0.068	1.284	4.51
PPARA	-0.018	0.295	-0.062	0.95	0.982	0.29	-0.063	0.95	0.55	1.752
PPA2	0.572	0.303	1.885	0.059	1.771	0.537	1.436	0.151	0.977	3.21
PPA1	-0.447	0.3	-1.49	0.136	0.64	0.192	-1.878	0.06	0.355	1.152
POU6F2	0.107	0.296	0.36	0.719	1.112	0.329	0.342	0.733	0.623	1.986
POU6F1	-0.155	0.295	-0.524	0.601	0.857	0.253	-0.566	0.571	0.48	1.529
POU5F1P4	-0.328	0.3	-1.094	0.274	0.721	0.216	-1.294	0.196	0.401	1.296
POU5F1P3	-0.116	0.295	-0.394	0.693	0.89	0.263	-0.418	0.676	0.499	1.588
POU5F1B	-0.222	0.296	-0.75	0.453	0.801	0.237	-0.84	0.401	0.448	1.431
POU4F3	0.624	0.303	2.058	0.04	1.867	0.566	1.531	0.126	1.03	3.382
POU4F2	-0.112	0.296	-0.379	0.705	0.894	0.265	-0.401	0.689	0.5	1.597
POU4F1	0.186	0.297	0.628	0.53	1.205	0.357	0.573	0.567	0.674	2.155
POU3F4	0.221	0.296	0.746	0.456	1.247	0.369	0.669	0.503	0.698	2.228
POU3F3	-0.473	0.3	-1.576	0.115	0.623	0.187	-2.015	0.044	0.346	1.122
POU3F2	0.23	0.297	0.773	0.439	1.259	0.374	0.691	0.49	0.703	2.255

POU3F1	-0.322	0.301	-1.07	0.285	0.725	0.218	-1.262	0.207	0.402	1.307
POU2F3	0.147	0.296	0.497	0.619	1.158	0.343	0.462	0.644	0.649	2.068
POU2F2	0.43	0.3	1.433	0.152	1.537	0.461	1.165	0.244	0.854	2.765
POU2F1	0.209	0.296	0.704	0.481	1.232	0.365	0.636	0.525	0.689	2.202
POU2AF1	-0.43	0.3	-1.436	0.151	0.65	0.195	-1.795	0.073	0.361	1.17
POU1F1	-0.46	0.303	-1.52	0.129	0.631	0.191	-1.929	0.054	0.349	1.143
POTEKP	0.162	0.296	0.548	0.584	1.176	0.348	0.506	0.613	0.659	2.099
POT1	-0.02	0.295	-0.067	0.947	0.98	0.29	-0.067	0.946	0.549	1.749
POSTN	-0.249	0.296	-0.841	0.401	0.78	0.231	-0.954	0.34	0.436	1.393
PORCN	-0.526	0.302	-1.74	0.082	0.591	0.179	-2.29	0.022	0.327	1.069
POR	-0.257	0.298	-0.862	0.389	0.774	0.23	-0.983	0.326	0.432	1.386
POPDC3	0.234	0.298	0.786	0.432	1.264	0.377	0.7	0.484	0.705	2.268
POPDC2	-0.112	0.295	-0.378	0.706	0.894	0.264	-0.4	0.69	0.501	1.596
POP7	0.452	0.299	1.512	0.13	1.571	0.47	1.217	0.224	0.875	2.823
POP5	-0.234	0.3	-0.779	0.436	0.791	0.238	-0.878	0.38	0.439	1.426
POP4	0.645	0.302	2.135	0.033	1.907	0.577	1.573	0.116	1.054	3.449
POP1	0.256	0.298	0.859	0.39	1.292	0.385	0.758	0.449	0.72	2.316
PON3	0.11	0.296	0.373	0.709	1.117	0.33	0.354	0.724	0.626	1.993
PON2	-0.519	0.302	-1.717	0.086	0.595	0.18	-2.251	0.024	0.329	1.076
PON1	0.437	0.3	1.455	0.146	1.548	0.464	1.179	0.238	0.859	2.787
POMZP3	0.335	0.298	1.126	0.26	1.398	0.416	0.957	0.339	0.78	2.506
POMT2	0.772	0.31	2.486	0.013	2.163	0.672	1.733	0.083	1.177	3.975
POMT1	0.114	0.295	0.386	0.699	1.121	0.331	0.365	0.715	0.628	1.999
POMP	1.698	0.373	4.547	0	5.464	2.041	2.188	0.029	2.628	11.36
POMGNT1	-0.319	0.304	-1.051	0.293	0.727	0.221	-1.238	0.216	0.401	1.318
POMC	0.246	0.297	0.83	0.407	1.28	0.38	0.735	0.462	0.715	2.291
POM121L9P	0.049	0.296	0.167	0.868	1.051	0.311	0.163	0.871	0.588	1.875
POM121L2	-0.498	0.302	-1.645	0.1	0.608	0.184	-2.132	0.033	0.336	1.1
POM121	0.361	0.3	1.205	0.228	1.435	0.43	1.012	0.312	0.798	2.581
POLRMT	0.38	0.297	1.277	0.201	1.462	0.435	1.063	0.288	0.816	2.619
POLR3K	0.409	0.299	1.369	0.171	1.505	0.449	1.124	0.261	0.838	2.702
POLR3G	0.441	0.299	1.478	0.139	1.555	0.464	1.195	0.232	0.866	2.793
POLR3F	0.154	0.297	0.52	0.603	1.167	0.346	0.482	0.63	0.652	2.087
POLR3D	-0.342	0.3	-1.143	0.253	0.71	0.213	-1.363	0.173	0.395	1.277
POLR3C	0.27	0.296	0.91	0.363	1.31	0.388	0.797	0.425	0.733	2.341
POLR3B	0.421	0.303	1.387	0.165	1.523	0.462	1.132	0.257	0.841	2.76
POLR2L	-0.37	0.304	-1.218	0.223	0.691	0.21	-1.474	0.141	0.381	1.253
POLR2K	0.446	0.299	1.489	0.136	1.562	0.468	1.201	0.23	0.868	2.809

POLR2J4	-0.118	0.295	-0.399	0.69	0.889	0.263	-0.424	0.672	0.498	1.586
POLR2J	0.335	0.299	1.12	0.263	1.398	0.419	0.952	0.341	0.778	2.514
POLR2I	0.355	0.297	1.196	0.232	1.426	0.423	1.007	0.314	0.797	2.552
POLR2H	0.793	0.31	2.56	0.01	2.209	0.684	1.767	0.077	1.204	4.054
POLR2G	0.177	0.296	0.598	0.55	1.193	0.353	0.548	0.584	0.669	2.13
POLR2F	0.662	0.306	2.167	0.03	1.939	0.593	1.584	0.113	1.065	3.531
POLR2E	0.008	0.301	0.027	0.978	1.008	0.304	0.027	0.978	0.559	1.819
POLR2D	0.121	0.297	0.407	0.684	1.129	0.335	0.383	0.701	0.63	2.02
POLR2C	-0.201	0.297	-0.677	0.499	0.818	0.243	-0.75	0.454	0.457	1.464
POLR2B	-0.075	0.297	-0.253	0.801	0.928	0.276	-0.262	0.793	0.518	1.661
POLR2A	-0.205	0.296	-0.694	0.488	0.814	0.241	-0.77	0.441	0.456	1.454
POLR1G	0.175	0.296	0.592	0.554	1.192	0.353	0.543	0.587	0.667	2.13
POLR1F	-0.251	0.298	-0.844	0.398	0.778	0.231	-0.96	0.337	0.434	1.394
POLR1E	0.036	0.296	0.121	0.904	1.037	0.307	0.119	0.905	0.58	1.851
POLR1D	0.016	0.296	0.055	0.956	1.016	0.301	0.055	0.956	0.569	1.815
POLR1C	0.362	0.298	1.216	0.224	1.436	0.427	1.02	0.308	0.801	2.573
POLR1B	0.24	0.302	0.797	0.426	1.272	0.384	0.708	0.479	0.704	2.297
POLQ	0.389	0.301	1.293	0.196	1.475	0.444	1.071	0.284	0.818	2.66
POLM	-0.101	0.295	-0.34	0.734	0.904	0.267	-0.358	0.72	0.507	1.614
POLL	0.682	0.306	2.228	0.026	1.979	0.606	1.615	0.106	1.086	3.607
POLI	-0.48	0.306	-1.57	0.116	0.619	0.189	-2.015	0.044	0.34	1.127
POLH	0.111	0.296	0.374	0.708	1.117	0.33	0.354	0.723	0.625	1.995
POLG2	0.063	0.295	0.213	0.831	1.065	0.315	0.207	0.836	0.597	1.901
POLG	0.695	0.306	2.272	0.023	2.004	0.613	1.638	0.101	1.1	3.649
POLE3	0.217	0.296	0.73	0.465	1.242	0.368	0.657	0.511	0.695	2.22
POLE2	0.525	0.3	1.751	0.08	1.69	0.507	1.362	0.173	0.939	3.042
POLE	-0.142	0.296	-0.478	0.633	0.868	0.257	-0.513	0.608	0.486	1.551
POLDIP3	-0.366	0.303	-1.207	0.228	0.694	0.21	-1.457	0.145	0.383	1.256
POLDIP2	0.06	0.296	0.202	0.84	1.062	0.315	0.196	0.845	0.594	1.897
POLD4	0.404	0.298	1.354	0.176	1.498	0.447	1.114	0.265	0.835	2.688
POLD3	-0.083	0.296	-0.28	0.78	0.921	0.273	-0.291	0.771	0.515	1.645
POLD2	0.179	0.295	0.607	0.544	1.196	0.353	0.556	0.579	0.671	2.134
POLD1	0.421	0.298	1.414	0.157	1.524	0.454	1.154	0.248	0.85	2.731
POLB	-0.268	0.299	-0.897	0.37	0.765	0.228	-1.029	0.303	0.426	1.374
POLA2	0.323	0.298	1.084	0.278	1.381	0.411	0.927	0.354	0.771	2.475
POLA1	0.201	0.299	0.671	0.502	1.222	0.366	0.608	0.543	0.68	2.197
POGZ	-0.169	0.295	-0.571	0.568	0.845	0.25	-0.621	0.534	0.474	1.507
POGLUT2	0.088	0.296	0.296	0.767	1.092	0.323	0.283	0.777	0.611	1.951

POGLUT1	0.455	0.3	1.514	0.13	1.576	0.473	1.217	0.224	0.875	2.84
POGK	-0.115	0.296	-0.388	0.698	0.892	0.264	-0.411	0.681	0.5	1.591
POFUT2	0.326	0.298	1.096	0.273	1.386	0.413	0.935	0.35	0.773	2.484
POFUT1	-0.575	0.31	-1.853	0.064	0.563	0.175	-2.504	0.012	0.306	1.034
POF1B	0.335	0.3	1.117	0.264	1.398	0.42	0.949	0.343	0.776	2.518
PODXL2	0.263	0.297	0.884	0.377	1.3	0.386	0.777	0.437	0.726	2.327
PODXL	-0.556	0.306	-1.82	0.069	0.573	0.175	-2.434	0.015	0.315	1.044
PODNL1	-0.035	0.295	-0.12	0.905	0.965	0.285	-0.122	0.903	0.541	1.722
PNRC2	-0.575	0.306	-1.88	0.06	0.563	0.172	-2.54	0.011	0.309	1.025
PNRC1	-0.381	0.3	-1.271	0.204	0.683	0.205	-1.548	0.122	0.38	1.229
PNPO	0.285	0.298	0.956	0.339	1.33	0.396	0.832	0.405	0.741	2.385
PNPLA6	-0.392	0.302	-1.299	0.194	0.675	0.204	-1.59	0.112	0.373	1.221
PNPLA4	-0.547	0.306	-1.788	0.074	0.579	0.177	-2.379	0.017	0.318	1.054
PNPLA3	0.752	0.31	2.423	0.015	2.122	0.659	1.703	0.089	1.155	3.9
PNPLA2	-0.391	0.298	-1.31	0.19	0.677	0.202	-1.603	0.109	0.377	1.214
PNP	0.376	0.3	1.255	0.209	1.457	0.437	1.046	0.296	0.81	2.621
PNOC	-0.235	0.297	-0.791	0.429	0.791	0.235	-0.891	0.373	0.442	1.415
PNO1	0.253	0.296	0.854	0.393	1.288	0.382	0.754	0.451	0.721	2.302
PNN	0.384	0.3	1.28	0.201	1.468	0.44	1.063	0.288	0.815	2.642
PNMT	0.5	0.303	1.651	0.099	1.648	0.499	1.299	0.194	0.911	2.984
PNMA8A	-0.582	0.306	-1.903	0.057	0.559	0.171	-2.582	0.01	0.307	1.018
PNMA3	-0.289	0.297	-0.974	0.33	0.749	0.223	-1.129	0.259	0.418	1.341
PNMA2	-0.378	0.298	-1.269	0.204	0.685	0.204	-1.542	0.123	0.382	1.228
PNMA1	-0.249	0.298	-0.835	0.404	0.78	0.232	-0.948	0.343	0.435	1.398
PNLIPRP2	0.253	0.296	0.854	0.393	1.287	0.381	0.755	0.451	0.721	2.299
PNLIPRP1	0.251	0.296	0.846	0.398	1.285	0.38	0.748	0.454	0.719	2.296
PNLIP	0.178	0.296	0.602	0.547	1.195	0.354	0.552	0.581	0.669	2.135
PNKP	-0.232	0.301	-0.772	0.44	0.793	0.238	-0.869	0.385	0.44	1.429
PNISR	-0.483	0.3	-1.61	0.108	0.617	0.185	-2.069	0.039	0.343	1.111
PMVK	0.247	0.296	0.836	0.403	1.281	0.379	0.74	0.459	0.717	2.288
PMS2P4	0.357	0.298	1.199	0.23	1.43	0.426	1.008	0.313	0.797	2.564
PMS2P3	0.085	0.296	0.288	0.773	1.089	0.322	0.276	0.782	0.61	1.944
PMS2P2	-0.545	0.303	-1.803	0.071	0.58	0.175	-2.397	0.017	0.32	1.049
PMS2P1	0.516	0.306	1.686	0.092	1.676	0.513	1.317	0.188	0.92	3.054
PMS1	-0.242	0.298	-0.815	0.415	0.785	0.234	-0.922	0.357	0.438	1.406
PMPCB	-0.545	0.31	-1.758	0.079	0.58	0.18	-2.338	0.019	0.316	1.065
PMPCA	-0.002	0.295	-0.007	0.995	0.998	0.295	-0.007	0.995	0.56	1.78
PMP22	-0.092	0.295	-0.311	0.756	0.912	0.269	-0.326	0.744	0.511	1.627

PMP2	0.094	0.295	0.318	0.751	1.098	0.324	0.303	0.762	0.616	1.958
PMM2	0.345	0.298	1.158	0.247	1.413	0.421	0.979	0.327	0.787	2.535
PMM1	0.264	0.297	0.89	0.373	1.302	0.387	0.782	0.434	0.728	2.331
PML	0.117	0.297	0.393	0.694	1.124	0.333	0.371	0.711	0.628	2.01
PMFBP1	0.097	0.295	0.33	0.741	1.102	0.326	0.314	0.753	0.618	1.966
PMF1	0.125	0.295	0.423	0.672	1.133	0.335	0.398	0.691	0.635	2.022
PMEPA1	-0.277	0.296	-0.935	0.35	0.758	0.225	-1.077	0.281	0.424	1.355
PMEL	0.627	0.306	2.052	0.04	1.872	0.572	1.524	0.127	1.028	3.408
PMCHL1	0.592	0.306	1.936	0.053	1.808	0.553	1.461	0.144	0.993	3.293
PMCH	0.008	0.296	0.029	0.977	1.008	0.298	0.028	0.977	0.565	1.8
PMAIP1	0.17	0.295	0.574	0.566	1.185	0.35	0.528	0.597	0.664	2.114
PLXND1	0.025	0.296	0.086	0.932	1.026	0.304	0.084	0.933	0.574	1.832
PLXNC1	-0.764	0.31	-2.466	0.014	0.466	0.144	-3.702	0	0.254	0.855
PLXNB3	0.311	0.298	1.044	0.297	1.364	0.406	0.897	0.37	0.761	2.445
PLXNB2	-0.089	0.297	-0.3	0.764	0.915	0.271	-0.314	0.753	0.512	1.636
PLXNB1	-0.895	0.321	-2.792	0.005	0.409	0.131	-4.514	0	0.218	0.766
PLXNA3	0.075	0.296	0.253	0.801	1.077	0.318	0.243	0.808	0.604	1.923
PLXNA2	-0.04	0.295	-0.136	0.892	0.961	0.283	-0.139	0.889	0.539	1.712
PLXNA1	0.321	0.303	1.058	0.29	1.378	0.418	0.905	0.365	0.761	2.497
PLXDC2	0.146	0.296	0.493	0.622	1.157	0.343	0.458	0.647	0.647	2.068
PLXDC1	-0.468	0.299	-1.566	0.117	0.627	0.187	-1.996	0.046	0.349	1.125
PLVAP	-0.442	0.298	-1.482	0.138	0.643	0.192	-1.864	0.062	0.358	1.153
PLTP	-0.448	0.3	-1.492	0.136	0.639	0.192	-1.882	0.06	0.354	1.151
PLSCR4	-0.418	0.3	-1.397	0.162	0.658	0.197	-1.735	0.083	0.366	1.184
PLSCR2	0.189	0.296	0.637	0.524	1.208	0.358	0.581	0.561	0.676	2.158
PLSCR1	0.524	0.302	1.734	0.083	1.689	0.511	1.35	0.177	0.934	3.055
PLS3	-0.141	0.295	-0.477	0.633	0.869	0.256	-0.512	0.608	0.487	1.549
PLS1	0.786	0.315	2.497	0.013	2.196	0.692	1.729	0.084	1.184	4.071
PLPPR4	0.006	0.295	0.02	0.984	1.006	0.297	0.02	0.984	0.564	1.794
PLPPR3	0.265	0.298	0.889	0.374	1.304	0.389	0.781	0.435	0.727	2.339
PLPPR2	-0.364	0.3	-1.216	0.224	0.695	0.208	-1.467	0.142	0.386	1.25
PLPPR1	-0.268	0.297	-0.903	0.366	0.765	0.227	-1.036	0.3	0.427	1.368
PLPP3	-0.181	0.296	-0.613	0.54	0.834	0.247	-0.673	0.501	0.467	1.489
PLPP2	-0.121	0.295	-0.41	0.681	0.886	0.262	-0.436	0.663	0.497	1.58
PLPP1	-0.795	0.315	-2.527	0.012	0.451	0.142	-3.861	0	0.244	0.837
PLPBP	-0.182	0.298	-0.612	0.541	0.833	0.248	-0.671	0.502	0.465	1.494
PLP2	0.315	0.296	1.063	0.288	1.37	0.406	0.912	0.362	0.767	2.449
PLP1	-0.41	0.298	-1.374	0.17	0.664	0.198	-1.698	0.09	0.37	1.191

PLOD3	-0.12	0.295	-0.405	0.686	0.887	0.262	-0.43	0.667	0.497	1.583
PLOD2	0.33	0.3	1.099	0.272	1.391	0.418	0.936	0.349	0.772	2.505
PLOD1	-0.422	0.299	-1.414	0.157	0.655	0.196	-1.759	0.079	0.365	1.177
PLN	-0.921	0.321	-2.871	0.004	0.398	0.128	-4.712	0	0.212	0.747
PLLP	0.002	0.296	0.008	0.994	1.002	0.297	0.008	0.994	0.561	1.79
PLK4	0.666	0.306	2.179	0.029	1.946	0.595	1.591	0.112	1.069	3.543
PLK3	-0.174	0.296	-0.588	0.556	0.84	0.249	-0.642	0.521	0.47	1.501
PLK2	-0.488	0.302	-1.614	0.107	0.614	0.186	-2.08	0.038	0.339	1.11
PLK1	0.671	0.306	2.19	0.029	1.956	0.599	1.596	0.111	1.073	3.564
PLIN3	-0.428	0.3	-1.428	0.153	0.652	0.195	-1.782	0.075	0.362	1.173
PLIN2	0.599	0.306	1.955	0.051	1.82	0.557	1.471	0.141	0.999	3.317
PLIN1	0.123	0.296	0.417	0.677	1.131	0.335	0.392	0.695	0.633	2.022
PLGRKT	-0.186	0.303	-0.616	0.538	0.83	0.251	-0.677	0.498	0.458	1.502
PLG	0.405	0.298	1.359	0.174	1.5	0.448	1.117	0.264	0.836	2.693
PLEKHS1	0.199	0.296	0.673	0.501	1.221	0.361	0.61	0.542	0.683	2.181
PLEKHO2	0.251	0.298	0.842	0.4	1.286	0.384	0.744	0.457	0.716	2.307
PLEKHO1	-0.583	0.306	-1.907	0.057	0.558	0.171	-2.588	0.01	0.307	1.016
PLEKHM2	-0.348	0.3	-1.161	0.246	0.706	0.212	-1.388	0.165	0.393	1.27
PLEKHM1	0.335	0.298	1.125	0.261	1.398	0.417	0.956	0.339	0.78	2.508
PLEKHH3	0.23	0.296	0.778	0.437	1.259	0.373	0.695	0.487	0.705	2.25
PLEKHG6	0.465	0.3	1.551	0.121	1.592	0.477	1.24	0.215	0.884	2.865
PLEKHG3	0.292	0.297	0.982	0.326	1.339	0.398	0.852	0.394	0.748	2.398
PLEKHF2	-0.225	0.296	-0.759	0.448	0.799	0.237	-0.85	0.395	0.447	1.427
PLEKHF1	0.196	0.301	0.652	0.514	1.217	0.366	0.592	0.554	0.675	2.193
PLEKHB2	-0.35	0.3	-1.167	0.243	0.705	0.211	-1.397	0.162	0.392	1.268
PLEKHB1	0.166	0.295	0.562	0.574	1.181	0.349	0.518	0.605	0.662	2.106
PLEKHA8P1	-0.012	0.295	-0.039	0.969	0.989	0.292	-0.039	0.969	0.554	1.763
PLEKHA6	0.139	0.295	0.47	0.639	1.149	0.339	0.439	0.661	0.644	2.049
PLEKHA5	0.2	0.295	0.679	0.497	1.222	0.361	0.615	0.539	0.685	2.18
PLEKHA4	0.128	0.295	0.435	0.664	1.137	0.336	0.408	0.683	0.637	2.029
PLEKHA2	0.158	0.299	0.53	0.596	1.172	0.35	0.49	0.624	0.653	2.103
PLEKHA1	0.034	0.295	0.117	0.907	1.035	0.305	0.115	0.909	0.581	1.845
PLEK2	0.145	0.296	0.492	0.623	1.157	0.342	0.457	0.647	0.648	2.065
PLEK	-0.199	0.297	-0.672	0.501	0.819	0.243	-0.744	0.457	0.458	1.465
PLEC	0.085	0.296	0.288	0.773	1.089	0.323	0.276	0.782	0.609	1.947
PLD3	-0.561	0.306	-1.834	0.067	0.571	0.175	-2.459	0.014	0.313	1.039
PLD2	-0.319	0.298	-1.073	0.283	0.727	0.216	-1.264	0.206	0.406	1.302
PLD1	-0.039	0.297	-0.132	0.895	0.962	0.285	-0.135	0.893	0.538	1.72

PLCXD1	-0.075	0.296	-0.255	0.799	0.927	0.274	-0.265	0.791	0.519	1.656
PLCL2	-0.507	0.3	-1.692	0.091	0.602	0.181	-2.204	0.028	0.335	1.084
PLCL1	-0.097	0.297	-0.327	0.744	0.908	0.269	-0.343	0.732	0.507	1.624
PLCH2	0.074	0.295	0.249	0.803	1.076	0.318	0.24	0.81	0.603	1.92
PLCH1	0.618	0.31	1.994	0.046	1.856	0.575	1.487	0.137	1.011	3.408
PLCG2	-0.113	0.296	-0.381	0.703	0.893	0.265	-0.403	0.687	0.5	1.597
PLCG1	0.632	0.31	2.036	0.042	1.881	0.584	1.509	0.131	1.024	3.457
PLCE1	0.023	0.295	0.078	0.938	1.023	0.302	0.077	0.938	0.574	1.825
PLCD1	-0.15	0.295	-0.509	0.611	0.86	0.254	-0.549	0.583	0.482	1.535
PLCB4	-0.259	0.298	-0.869	0.385	0.772	0.23	-0.992	0.321	0.43	1.385
PLCB3	0.604	0.306	1.978	0.048	1.83	0.559	1.484	0.138	1.005	3.331
PLCB2	-0.664	0.307	-2.162	0.031	0.515	0.158	-3.068	0.002	0.282	0.94
PLCB1	-0.022	0.296	-0.076	0.94	0.978	0.289	-0.077	0.939	0.548	1.746
PLBD1	0.098	0.297	0.33	0.742	1.103	0.327	0.314	0.754	0.617	1.972
PLAUR	0.019	0.295	0.064	0.949	1.019	0.301	0.063	0.949	0.571	1.817
PLAU	0.218	0.296	0.736	0.461	1.244	0.369	0.662	0.508	0.696	2.224
PLAT	-0.964	0.328	-2.938	0.003	0.381	0.125	-4.943	0	0.201	0.726
PLAGL2	0.842	0.315	2.674	0.008	2.32	0.73	1.808	0.071	1.252	4.3
PLAGL1	-0.402	0.3	-1.339	0.18	0.669	0.201	-1.648	0.099	0.372	1.204
PLAG1	-0.018	0.295	-0.062	0.951	0.982	0.29	-0.063	0.95	0.551	1.751
PLAC8	-0.578	0.306	-1.89	0.059	0.561	0.172	-2.558	0.011	0.308	1.022
PLAC4	0.263	0.298	0.883	0.377	1.301	0.387	0.776	0.438	0.726	2.331
PLAC1	0.059	0.295	0.2	0.842	1.061	0.313	0.194	0.846	0.595	1.891
PLAAT4	-0.032	0.297	-0.107	0.915	0.969	0.288	-0.109	0.914	0.541	1.735
PLAAT3	0.546	0.301	1.815	0.07	1.726	0.519	1.399	0.162	0.957	3.112
PLAAT2	0.084	0.295	0.284	0.777	1.087	0.321	0.272	0.785	0.61	1.94
PLAAT1	0.722	0.306	2.357	0.018	2.058	0.631	1.678	0.093	1.129	3.752
PLAA	0.851	0.312	2.731	0.006	2.341	0.729	1.839	0.066	1.272	4.312
PLA2R1	-0.095	0.296	-0.322	0.748	0.909	0.269	-0.338	0.736	0.509	1.625
PLA2G7	0.692	0.307	2.257	0.024	1.998	0.612	1.629	0.103	1.095	3.643
PLA2G6	0.187	0.302	0.619	0.536	1.206	0.364	0.564	0.572	0.667	2.18
PLA2G5	-0.348	0.298	-1.168	0.243	0.706	0.21	-1.397	0.162	0.394	1.266
PLA2G4C	-0.283	0.3	-0.946	0.344	0.753	0.226	-1.093	0.274	0.419	1.355
PLA2G4A	0.057	0.295	0.193	0.847	1.059	0.313	0.187	0.851	0.593	1.888
PLA2G3	-0.275	0.3	-0.918	0.358	0.759	0.228	-1.057	0.29	0.422	1.366
PLA2G2F	0.427	0.302	1.412	0.158	1.532	0.463	1.15	0.25	0.847	2.771
PLA2G2E	0.002	0.295	0.006	0.996	1.002	0.296	0.006	0.996	0.561	1.787
PLA2G2D	-0.447	0.299	-1.493	0.135	0.64	0.191	-1.883	0.06	0.356	1.15

PLA2G2A	0.328	0.298	1.102	0.27	1.388	0.413	0.94	0.347	0.775	2.487
PLA2G1B	-0.165	0.296	-0.557	0.578	0.848	0.251	-0.605	0.545	0.475	1.514
PLA2G15	-0.092	0.296	-0.311	0.756	0.912	0.27	-0.325	0.745	0.51	1.63
PLA2G12A	0.78	0.31	2.514	0.012	2.182	0.677	1.745	0.081	1.187	4.008
PLA1A	0.104	0.295	0.354	0.723	1.11	0.328	0.336	0.737	0.622	1.98
PKP4	0.305	0.298	1.026	0.305	1.357	0.404	0.884	0.377	0.757	2.432
PKP3	-0.126	0.296	-0.424	0.671	0.882	0.261	-0.452	0.651	0.494	1.576
PKP2	-0.243	0.297	-0.819	0.413	0.784	0.233	-0.927	0.354	0.439	1.402
PKP1	-0.3	0.298	-1.006	0.315	0.741	0.221	-1.173	0.241	0.413	1.329
PKNOX2	0.134	0.296	0.455	0.649	1.144	0.338	0.425	0.671	0.641	2.041
PKNOX1	-0.073	0.297	-0.245	0.807	0.93	0.276	-0.254	0.8	0.52	1.664
PKN2	0.167	0.296	0.567	0.571	1.182	0.35	0.522	0.602	0.662	2.11
PKN1	-0.653	0.306	-2.137	0.033	0.52	0.159	-3.016	0.003	0.286	0.947
PKMYT1	0.48	0.303	1.586	0.113	1.616	0.489	1.26	0.208	0.893	2.925
PKM	-0.091	0.296	-0.308	0.758	0.913	0.27	-0.323	0.747	0.511	1.632
PKLR	0.227	0.297	0.765	0.445	1.254	0.372	0.684	0.494	0.702	2.243
PKIG	-0.06	0.297	-0.203	0.839	0.941	0.28	-0.209	0.834	0.526	1.685
PKIA	-0.263	0.297	-0.886	0.375	0.769	0.228	-1.014	0.311	0.43	1.375
PKDREJ	-0.1	0.295	-0.339	0.734	0.905	0.267	-0.357	0.721	0.507	1.614
PKD2L2	-0.298	0.298	-1	0.317	0.742	0.221	-1.165	0.244	0.414	1.331
PKD2L1	0.481	0.3	1.604	0.109	1.617	0.485	1.273	0.203	0.899	2.91
PKD2	-0.834	0.311	-2.683	0.007	0.434	0.135	-4.19	0	0.236	0.799
PJA2	-0.74	0.315	-2.352	0.019	0.477	0.15	-3.484	0	0.257	0.884
PJA1	0.833	0.313	2.665	0.008	2.3	0.719	1.808	0.071	1.247	4.245
PIWIL2	-0.132	0.296	-0.447	0.655	0.876	0.259	-0.478	0.633	0.49	1.566
PIWIL1	0.113	0.295	0.381	0.703	1.119	0.33	0.36	0.719	0.627	1.996
PITX3	-0.229	0.296	-0.773	0.44	0.795	0.236	-0.868	0.385	0.445	1.422
PITX2	-0.405	0.3	-1.353	0.176	0.667	0.2	-1.669	0.095	0.371	1.199
PITX1	0.45	0.3	1.5	0.134	1.569	0.471	1.208	0.227	0.871	2.825
PITRM1	0.189	0.296	0.638	0.523	1.208	0.358	0.581	0.561	0.676	2.16
PITPNM3	0.257	0.298	0.862	0.389	1.293	0.385	0.76	0.447	0.721	2.318
PITPNM1	0.628	0.31	2.024	0.043	1.873	0.581	1.503	0.133	1.02	3.44
PITPNC1	0.404	0.299	1.349	0.177	1.498	0.448	1.11	0.267	0.833	2.693
PITPNB	-0.315	0.297	-1.061	0.289	0.729	0.217	-1.247	0.212	0.407	1.307
PITPNA	-0.129	0.296	-0.435	0.663	0.879	0.26	-0.465	0.642	0.493	1.569
PIR	0.575	0.302	1.901	0.057	1.777	0.537	1.446	0.148	0.982	3.213
PIPOX	0.354	0.298	1.187	0.235	1.424	0.425	1	0.318	0.794	2.555
PIP5K1C	-0.573	0.306	-1.875	0.061	0.564	0.172	-2.531	0.011	0.309	1.026

PIP5K1B	0.152	0.298	0.51	0.61	1.164	0.347	0.474	0.636	0.649	2.089
PIP5K1A	0.255	0.298	0.856	0.392	1.291	0.385	0.755	0.45	0.719	2.315
PIP4K2C	0.5	0.3	1.667	0.095	1.648	0.494	1.312	0.189	0.916	2.965
PIP4K2B	-0.07	0.296	-0.237	0.813	0.932	0.276	-0.245	0.806	0.522	1.666
PIP4K2A	-0.793	0.315	-2.516	0.012	0.452	0.143	-3.84	0	0.244	0.839
PIP	-0.45	0.302	-1.487	0.137	0.638	0.193	-1.878	0.06	0.353	1.154
PINLYP	-0.166	0.295	-0.562	0.574	0.847	0.25	-0.611	0.541	0.475	1.511
PINK1	-0.536	0.306	-1.751	0.08	0.585	0.179	-2.317	0.021	0.321	1.066
PIN4	0.72	0.31	2.322	0.02	2.053	0.636	1.655	0.098	1.119	3.769
PIN1P1	0.572	0.304	1.882	0.06	1.772	0.539	1.433	0.152	0.977	3.216
PIN1	0.315	0.297	1.061	0.289	1.37	0.407	0.91	0.363	0.766	2.451
PIMREG	0.774	0.31	2.499	0.012	2.168	0.672	1.739	0.082	1.182	3.98
PIM2	-0.157	0.296	-0.529	0.597	0.855	0.253	-0.573	0.567	0.478	1.528
PIM1	0.004	0.296	0.015	0.988	1.004	0.297	0.015	0.988	0.563	1.793
PILRA	0.062	0.295	0.21	0.834	1.064	0.314	0.203	0.839	0.597	1.897
PIKFYVE	-0.528	0.306	-1.726	0.084	0.59	0.18	-2.273	0.023	0.324	1.074
PIK3R5	-0.304	0.297	-1.024	0.306	0.738	0.219	-1.197	0.231	0.412	1.32
PIK3R4	0.383	0.3	1.277	0.202	1.466	0.439	1.061	0.289	0.815	2.638
PIK3R3	-0.022	0.295	-0.075	0.94	0.978	0.289	-0.076	0.939	0.549	1.744
PIK3R2	-0.607	0.306	-1.985	0.047	0.545	0.167	-2.73	0.006	0.3	0.992
PIK3R1	-0.62	0.31	-2.002	0.045	0.538	0.167	-2.774	0.006	0.293	0.987
PIK3IP1	0.201	0.296	0.68	0.497	1.223	0.361	0.616	0.538	0.685	2.182
PIK3CG	-0.253	0.296	-0.854	0.393	0.776	0.23	-0.972	0.331	0.434	1.388
PIK3CD	-0.291	0.298	-0.978	0.328	0.747	0.223	-1.136	0.256	0.417	1.34
PIK3CB	0.253	0.298	0.851	0.395	1.288	0.384	0.751	0.452	0.719	2.309
PIK3CA	0.15	0.296	0.508	0.611	1.162	0.344	0.472	0.637	0.651	2.075
PIK3C3	-0.776	0.321	-2.42	0.016	0.46	0.148	-3.659	0	0.245	0.863
PIK3C2G	-0.579	0.304	-1.904	0.057	0.561	0.17	-2.579	0.01	0.309	1.017
PIK3C2B	0.312	0.3	1.041	0.298	1.366	0.41	0.894	0.371	0.759	2.458
PIK3C2A	-0.373	0.3	-1.246	0.213	0.688	0.206	-1.511	0.131	0.383	1.238
PIH1D1	0.159	0.296	0.535	0.592	1.172	0.347	0.495	0.621	0.656	2.094
PIGZ	0.446	0.3	1.488	0.137	1.562	0.468	1.2	0.23	0.868	2.81
PIGV	-0.234	0.298	-0.785	0.433	0.791	0.236	-0.884	0.377	0.441	1.42
PIGT	-0.077	0.296	-0.262	0.793	0.925	0.274	-0.273	0.785	0.518	1.652
PIGR	-0.142	0.295	-0.48	0.631	0.868	0.256	-0.515	0.606	0.486	1.549
PIGQ	-0.006	0.296	-0.021	0.984	0.994	0.294	-0.021	0.983	0.556	1.776
PIGP	-0.076	0.298	-0.254	0.8	0.927	0.276	-0.264	0.792	0.517	1.662
PIGO	-0.216	0.297	-0.729	0.466	0.806	0.239	-0.814	0.416	0.45	1.44

PIGN	-0.251	0.3	-0.839	0.402	0.778	0.233	-0.953	0.34	0.432	1.4
PIGL	0.286	0.299	0.957	0.338	1.331	0.397	0.833	0.405	0.741	2.389
PIGK	-0.147	0.301	-0.489	0.625	0.863	0.26	-0.527	0.598	0.478	1.558
PIGH	-0.154	0.298	-0.516	0.606	0.857	0.256	-0.558	0.577	0.478	1.538
PIGG	-0.29	0.298	-0.975	0.329	0.748	0.223	-1.132	0.258	0.417	1.341
PIGF	-0.487	0.303	-1.607	0.108	0.615	0.186	-2.071	0.038	0.34	1.113
PIGC	0.505	0.3	1.683	0.092	1.657	0.497	1.321	0.186	0.92	2.982
PIGB	-0.131	0.3	-0.436	0.663	0.878	0.263	-0.466	0.642	0.488	1.579
PIGA	0.209	0.296	0.705	0.481	1.232	0.365	0.637	0.524	0.69	2.202
PIEZO2	-0.811	0.315	-2.577	0.01	0.444	0.14	-3.972	0	0.24	0.824
PIEZO1	0.386	0.3	1.286	0.198	1.471	0.441	1.067	0.286	0.817	2.649
PIDD1	0.375	0.299	1.257	0.209	1.456	0.434	1.048	0.294	0.811	2.613
PID1	-0.621	0.303	-2.045	0.041	0.538	0.163	-2.834	0.005	0.297	0.974
PICK1	0.291	0.298	0.98	0.327	1.338	0.398	0.85	0.395	0.747	2.398
PICALM	-0.507	0.304	-1.668	0.095	0.602	0.183	-2.173	0.03	0.332	1.093
PIBF1	0.117	0.296	0.397	0.691	1.125	0.333	0.375	0.708	0.63	2.008
PIAS4	0.027	0.296	0.092	0.927	1.028	0.304	0.091	0.928	0.575	1.836
PIAS3	-0.102	0.295	-0.345	0.73	0.903	0.267	-0.363	0.716	0.506	1.611
PIAS2	0.004	0.296	0.013	0.99	1.004	0.298	0.013	0.99	0.562	1.795
PIAS1	0.416	0.299	1.393	0.164	1.516	0.453	1.139	0.255	0.844	2.723
PI4KB	-0.538	0.301	-1.785	0.074	0.584	0.176	-2.365	0.018	0.324	1.054
PI4KA	-0.658	0.308	-2.138	0.033	0.518	0.159	-3.025	0.002	0.283	0.947
PI4K2A	-0.065	0.295	-0.22	0.826	0.937	0.277	-0.227	0.82	0.525	1.672
PI3	0.029	0.295	0.097	0.923	1.029	0.304	0.096	0.924	0.577	1.835
PI15	-0.414	0.302	-1.37	0.171	0.661	0.2	-1.697	0.09	0.366	1.195
PHYHIP	0.181	0.296	0.61	0.542	1.198	0.355	0.558	0.577	0.67	2.141
PHYH	0.291	0.297	0.981	0.327	1.338	0.397	0.851	0.395	0.748	2.392
PHTF2	-0.113	0.296	-0.381	0.703	0.893	0.265	-0.403	0.687	0.5	1.596
PHTF1	-0.602	0.31	-1.944	0.052	0.548	0.17	-2.667	0.008	0.298	1.005
PHOX2B	-0.486	0.302	-1.609	0.108	0.615	0.186	-2.071	0.038	0.34	1.112
PHOX2A	0.036	0.295	0.121	0.904	1.036	0.306	0.119	0.906	0.581	1.848
PHLPP2	0.493	0.3	1.646	0.1	1.637	0.491	1.299	0.194	0.91	2.945
PHLPP1	-0.277	0.298	-0.931	0.352	0.758	0.226	-1.072	0.284	0.423	1.359
PHLDB1	-0.457	0.303	-1.509	0.131	0.633	0.192	-1.913	0.056	0.35	1.146
PHLDA3	0.038	0.298	0.128	0.898	1.039	0.31	0.126	0.9	0.579	1.864
PHLDA2	0.24	0.296	0.81	0.418	1.271	0.376	0.72	0.472	0.711	2.27
PHLDA1	0.286	0.298	0.962	0.336	1.332	0.397	0.836	0.403	0.743	2.388
PHKG2	-0.131	0.296	-0.442	0.658	0.877	0.26	-0.473	0.636	0.491	1.568

PHKG1	-0.146	0.295	-0.496	0.62	0.864	0.255	-0.534	0.593	0.484	1.541
PHKB	-0.157	0.296	-0.529	0.597	0.855	0.253	-0.572	0.567	0.479	1.528
PHKA2	-0.16	0.296	-0.541	0.589	0.852	0.253	-0.587	0.557	0.476	1.523
PHKA1	0.079	0.295	0.268	0.789	1.082	0.319	0.257	0.797	0.607	1.929
PHIP	-0.105	0.295	-0.355	0.723	0.901	0.266	-0.374	0.709	0.505	1.606
PHGDH	0.452	0.302	1.495	0.135	1.572	0.475	1.203	0.229	0.869	2.844
PHF8	0.004	0.295	0.013	0.99	1.004	0.296	0.013	0.99	0.563	1.79
PHF7	-0.183	0.297	-0.617	0.537	0.833	0.247	-0.678	0.498	0.465	1.49
PHF3	0.289	0.3	0.964	0.335	1.336	0.401	0.837	0.402	0.742	2.406
PHF24	-0.104	0.296	-0.351	0.725	0.901	0.266	-0.37	0.711	0.505	1.609
PHF21A	-0.011	0.295	-0.038	0.97	0.989	0.292	-0.038	0.97	0.554	1.764
PHF20L1	0.763	0.315	2.422	0.015	2.144	0.675	1.694	0.09	1.156	3.974
PHF20	0.022	0.296	0.074	0.941	1.022	0.302	0.073	0.941	0.572	1.826
PHF2	-0.497	0.301	-1.653	0.098	0.608	0.183	-2.142	0.032	0.338	1.096
PHF14	0.178	0.296	0.6	0.549	1.194	0.354	0.55	0.583	0.669	2.134
PHF11	-0.118	0.297	-0.399	0.69	0.888	0.264	-0.423	0.672	0.497	1.589
PHF10	0.216	0.298	0.727	0.467	1.241	0.369	0.654	0.513	0.693	2.225
PHF1	-0.427	0.299	-1.428	0.153	0.652	0.195	-1.781	0.075	0.363	1.173
PHEX	-0.09	0.296	-0.302	0.762	0.914	0.271	-0.316	0.752	0.511	1.635
PHC3	0.432	0.3	1.439	0.15	1.54	0.462	1.169	0.243	0.855	2.771
PHC2	-0.162	0.295	-0.549	0.583	0.85	0.251	-0.595	0.552	0.477	1.517
PHC1	-1.045	0.328	-3.184	0.001	0.352	0.115	-5.616	0	0.185	0.669
PHB2	0.015	0.295	0.05	0.96	1.015	0.3	0.05	0.96	0.569	1.81
PHB	0.363	0.3	1.212	0.226	1.438	0.431	1.016	0.31	0.799	2.586
PHACTR4	-0.171	0.296	-0.579	0.562	0.842	0.249	-0.632	0.527	0.472	1.504
PHACTR2	-0.481	0.3	-1.603	0.109	0.618	0.185	-2.058	0.04	0.344	1.113
PHACTR1	0.046	0.296	0.156	0.876	1.047	0.309	0.153	0.878	0.587	1.869
PGS1	0.499	0.302	1.651	0.099	1.647	0.498	1.3	0.194	0.911	2.978
PGRMC2	-0.418	0.302	-1.381	0.167	0.659	0.199	-1.715	0.086	0.364	1.191
PGRMC1	0.372	0.3	1.242	0.214	1.451	0.435	1.037	0.3	0.806	2.61
PGR	-0.591	0.31	-1.905	0.057	0.554	0.172	-2.598	0.009	0.302	1.017
PGPEP1	0.211	0.298	0.71	0.478	1.235	0.368	0.64	0.522	0.689	2.214
PGP	-0.113	0.295	-0.382	0.702	0.893	0.264	-0.404	0.686	0.501	1.594
PGM5	0.26	0.297	0.876	0.381	1.297	0.385	0.772	0.44	0.725	2.32
PGM3	-0.358	0.3	-1.195	0.232	0.699	0.21	-1.437	0.151	0.388	1.258
PGM1	0.146	0.296	0.492	0.623	1.157	0.342	0.458	0.647	0.648	2.064
PGLYRP4	-0.062	0.297	-0.21	0.834	0.94	0.279	-0.216	0.829	0.525	1.681
PGLYRP1	0.095	0.296	0.32	0.749	1.099	0.325	0.305	0.76	0.616	1.962

PGLS	0.442	0.3	1.472	0.141	1.555	0.467	1.19	0.234	0.864	2.8
PGK2	0.36	0.298	1.207	0.227	1.433	0.427	1.014	0.311	0.799	2.571
PGK1	1.096	0.328	3.345	0.001	2.994	0.981	2.032	0.042	1.575	5.691
PGGT1B	0.331	0.298	1.111	0.266	1.393	0.415	0.946	0.344	0.776	2.499
PGGHG	0.177	0.296	0.597	0.551	1.193	0.353	0.547	0.584	0.668	2.132
PGF	0.274	0.304	0.9	0.368	1.315	0.4	0.788	0.431	0.724	2.387
PGD	-0.824	0.321	-2.568	0.01	0.439	0.141	-3.987	0	0.234	0.823
PGC	-0.054	0.297	-0.181	0.857	0.948	0.281	-0.186	0.853	0.53	1.695
PGBD5	-0.001	0.295	-0.005	0.996	0.999	0.295	-0.005	0.996	0.56	1.78
PGAP6	0.12	0.296	0.404	0.686	1.127	0.333	0.381	0.703	0.631	2.013
PGAP4	-0.009	0.295	-0.031	0.975	0.991	0.292	-0.031	0.975	0.556	1.767
PGAP3	0.492	0.303	1.627	0.104	1.636	0.495	1.285	0.199	0.904	2.961
PGAP2	0.139	0.296	0.47	0.638	1.15	0.341	0.439	0.661	0.643	2.055
PGAP1	0.18	0.296	0.606	0.544	1.197	0.354	0.555	0.579	0.67	2.139
PGAM2	0.343	0.298	1.152	0.249	1.409	0.42	0.975	0.329	0.786	2.527
PGAM1	0.471	0.3	1.571	0.116	1.601	0.48	1.253	0.21	0.89	2.881
PFN2	0.615	0.306	2.013	0.044	1.85	0.565	1.503	0.133	1.016	3.367
PFN1	-0.014	0.295	-0.046	0.963	0.986	0.291	-0.047	0.963	0.553	1.759
PFKP	0.257	0.296	0.867	0.386	1.293	0.383	0.765	0.445	0.723	2.31
PFKM	0.534	0.306	1.747	0.081	1.706	0.521	1.354	0.176	0.937	3.105
PFKL	0.559	0.306	1.828	0.068	1.749	0.535	1.4	0.161	0.96	3.185
PFKFB4	-0.231	0.3	-0.771	0.441	0.794	0.238	-0.868	0.386	0.441	1.428
PFKFB3	0.751	0.307	2.446	0.014	2.12	0.651	1.72	0.086	1.161	3.87
PFKFB2	0.014	0.295	0.047	0.962	1.014	0.299	0.047	0.963	0.569	1.808
PFKFB1	0.443	0.3	1.479	0.139	1.558	0.467	1.195	0.232	0.866	2.802
PFDN5	-0.392	0.3	-1.307	0.191	0.676	0.203	-1.601	0.109	0.376	1.216
PFDN4	0.403	0.3	1.343	0.179	1.496	0.448	1.105	0.269	0.831	2.692
PFDN2	0.589	0.306	1.924	0.054	1.802	0.552	1.454	0.146	0.989	3.283
PFDN1	0.27	0.298	0.907	0.365	1.31	0.39	0.794	0.427	0.731	2.349
PFAS	0.565	0.303	1.867	0.062	1.759	0.532	1.426	0.154	0.972	3.183
PF4V1	0.454	0.299	1.518	0.129	1.575	0.471	1.22	0.222	0.876	2.831
PF4	-0.614	0.306	-2.007	0.045	0.541	0.166	-2.771	0.006	0.297	0.986
PEX7	-0.501	0.306	-1.636	0.102	0.606	0.185	-2.124	0.034	0.333	1.104
PEX6	0.102	0.295	0.346	0.73	1.107	0.327	0.328	0.743	0.621	1.976
PEX5L	0.001	0.295	0.003	0.998	1.001	0.295	0.003	0.998	0.561	1.785
PEX5	0.028	0.295	0.096	0.924	1.029	0.304	0.094	0.925	0.577	1.834
PEX3	-0.391	0.3	-1.301	0.193	0.677	0.203	-1.592	0.111	0.376	1.218
PEX26	0.275	0.298	0.925	0.355	1.317	0.392	0.809	0.419	0.735	2.36

PEX2	-0.768	0.315	-2.439	0.015	0.464	0.146	-3.669	0	0.25	0.86
PEX19	-0.269	0.3	-0.897	0.369	0.764	0.229	-1.03	0.303	0.424	1.376
PEX16	0.212	0.298	0.712	0.476	1.236	0.368	0.642	0.521	0.69	2.215
PEX14	-0.382	0.299	-1.281	0.2	0.682	0.204	-1.56	0.119	0.38	1.225
PEX13	0.358	0.298	1.201	0.23	1.43	0.425	1.01	0.312	0.798	2.562
PEX12	-0.375	0.3	-1.25	0.211	0.688	0.206	-1.516	0.129	0.382	1.237
PEX11B	-0.57	0.306	-1.866	0.062	0.565	0.173	-2.515	0.012	0.311	1.029
PEX11A	0.005	0.297	0.017	0.986	1.005	0.298	0.017	0.986	0.562	1.798
PEX10	-0.548	0.31	-1.768	0.077	0.578	0.179	-2.355	0.019	0.315	1.061
PEX1	-0.762	0.315	-2.423	0.015	0.467	0.147	-3.633	0	0.252	0.864
PES1	0.447	0.3	1.49	0.136	1.563	0.468	1.202	0.229	0.869	2.812
PERP	-0.321	0.298	-1.077	0.281	0.726	0.216	-1.27	0.204	0.405	1.301
PER3	-0.349	0.3	-1.162	0.245	0.706	0.212	-1.39	0.165	0.392	1.271
PER2	-0.222	0.296	-0.75	0.453	0.801	0.237	-0.839	0.401	0.448	1.431
PEPD	0.513	0.3	1.709	0.088	1.67	0.502	1.337	0.181	0.927	3.009
PENK	0.199	0.299	0.664	0.507	1.22	0.365	0.602	0.547	0.679	2.192
PEMT	0.469	0.299	1.566	0.117	1.598	0.479	1.25	0.211	0.889	2.874
PELP1	0.733	0.306	2.395	0.017	2.082	0.637	1.697	0.09	1.142	3.793
PELO	0.394	0.298	1.322	0.186	1.484	0.443	1.092	0.275	0.827	2.663
PELI2	-0.266	0.296	-0.897	0.37	0.767	0.227	-1.027	0.304	0.429	1.37
PELI1	-0.422	0.3	-1.407	0.16	0.656	0.197	-1.75	0.08	0.364	1.181
PEG3	-0.321	0.298	-1.077	0.281	0.725	0.216	-1.271	0.204	0.404	1.301
PEG10	-0.186	0.295	-0.629	0.529	0.83	0.245	-0.692	0.489	0.465	1.482
PEF1	-0.941	0.321	-2.936	0.003	0.39	0.125	-4.877	0	0.208	0.731
PECR	-0.176	0.298	-0.59	0.555	0.839	0.25	-0.646	0.519	0.467	1.505
PECAM1	-0.261	0.296	-0.881	0.378	0.77	0.228	-1.007	0.314	0.431	1.376
PEBP1	-0.236	0.298	-0.793	0.428	0.79	0.235	-0.894	0.371	0.441	1.415
PEAK1	0.423	0.298	1.418	0.156	1.526	0.455	1.156	0.248	0.851	2.739
PEA15	-1.074	0.328	-3.278	0.001	0.342	0.112	-5.883	0	0.18	0.649
PDZRN4	-0.793	0.315	-2.519	0.012	0.453	0.142	-3.842	0	0.244	0.839
PDZRN3	-0.887	0.315	-2.817	0.005	0.412	0.13	-4.535	0	0.222	0.763
PDZK1IP1	0.24	0.296	0.809	0.419	1.271	0.377	0.719	0.472	0.711	2.271
PDZK1	-0.082	0.298	-0.276	0.783	0.921	0.274	-0.287	0.774	0.514	1.651
PDZD8	0.367	0.298	1.229	0.219	1.443	0.43	1.029	0.304	0.804	2.589
PDZD7	0.464	0.3	1.544	0.123	1.59	0.478	1.235	0.217	0.883	2.866
PDZD3	0.07	0.295	0.236	0.813	1.072	0.316	0.228	0.82	0.601	1.912
PDZD2	-0.334	0.298	-1.123	0.261	0.716	0.213	-1.334	0.182	0.399	1.283
PDYN	-0.047	0.295	-0.159	0.874	0.954	0.282	-0.163	0.871	0.535	1.702

PDXP-DT	0.126	0.295	0.426	0.67	1.134	0.335	0.4	0.689	0.636	2.024
PDXK	-0.334	0.298	-1.124	0.261	0.716	0.213	-1.334	0.182	0.4	1.283
PDX1	0.101	0.295	0.342	0.733	1.106	0.327	0.325	0.745	0.62	1.973
PDSS2	0.697	0.31	2.25	0.024	2.007	0.622	1.62	0.105	1.094	3.683
PDSS1	0.436	0.303	1.44	0.15	1.546	0.468	1.167	0.243	0.854	2.798
PDS5B	0.32	0.298	1.074	0.283	1.377	0.41	0.919	0.358	0.768	2.469
PDS5A	-0.438	0.303	-1.447	0.148	0.645	0.195	-1.815	0.07	0.357	1.168
PDPR	-0.443	0.301	-1.476	0.14	0.642	0.193	-1.857	0.063	0.356	1.157
PDPN	-0.537	0.302	-1.776	0.076	0.585	0.177	-2.351	0.019	0.323	1.057
PDPK1	0.601	0.304	1.975	0.048	1.824	0.555	1.485	0.138	1.005	3.31
PDP1	-0.075	0.295	-0.254	0.8	0.928	0.274	-0.263	0.792	0.52	1.654
PDLIM7	-0.78	0.31	-2.515	0.012	0.458	0.142	-3.81	0	0.25	0.842
PDLIM5	-0.892	0.321	-2.779	0.005	0.41	0.132	-4.488	0	0.218	0.769
PDLIM4	-0.254	0.298	-0.853	0.394	0.776	0.231	-0.971	0.332	0.433	1.39
PDLIM3	0.033	0.295	0.111	0.911	1.033	0.305	0.109	0.913	0.579	1.843
PDLIM2	-0.172	0.296	-0.582	0.56	0.842	0.249	-0.635	0.525	0.471	1.504
PDLIM1	-0.688	0.31	-2.222	0.026	0.502	0.156	-3.197	0.001	0.274	0.922
PDK4	-0.39	0.303	-1.285	0.199	0.677	0.205	-1.572	0.116	0.374	1.227
PDK3	0.346	0.298	1.16	0.246	1.413	0.421	0.981	0.326	0.788	2.532
PDK2	-0.069	0.295	-0.235	0.814	0.933	0.275	-0.243	0.808	0.523	1.664
PDK1	-0.073	0.299	-0.244	0.807	0.93	0.278	-0.253	0.8	0.518	1.67
PDIA6	0.358	0.305	1.175	0.24	1.431	0.436	0.987	0.323	0.787	2.601
PDIA4	0.086	0.295	0.29	0.772	1.089	0.322	0.278	0.781	0.611	1.944
PDIA3	-0.312	0.298	-1.05	0.294	0.732	0.218	-1.232	0.218	0.408	1.311
PDIA2	0.242	0.298	0.813	0.416	1.274	0.379	0.722	0.47	0.711	2.283
PDHX	0.242	0.296	0.817	0.414	1.274	0.378	0.726	0.468	0.713	2.277
PDHB	0.331	0.298	1.111	0.267	1.392	0.415	0.946	0.344	0.777	2.496
PDHA2	0.021	0.296	0.071	0.943	1.021	0.302	0.07	0.944	0.572	1.823
PDHA1	0.181	0.295	0.611	0.541	1.198	0.354	0.559	0.576	0.671	2.138
PDGFRL	-0.843	0.315	-2.679	0.007	0.43	0.135	-4.205	0	0.232	0.798
PDGFRB	-0.765	0.31	-2.466	0.014	0.465	0.144	-3.705	0	0.253	0.855
PDGFRA	-0.632	0.304	-2.081	0.037	0.532	0.161	-2.901	0.004	0.293	0.964
PDGFD	-0.653	0.306	-2.135	0.033	0.521	0.159	-3.012	0.003	0.286	0.948
PDGFC	-0.967	0.321	-3.016	0.003	0.38	0.122	-5.086	0	0.203	0.713
PDGFB	0.302	0.297	1.017	0.309	1.352	0.401	0.878	0.38	0.756	2.419
PDGFA	-0.313	0.298	-1.052	0.293	0.731	0.218	-1.235	0.217	0.407	1.311
PDE9A	-0.671	0.306	-2.194	0.028	0.511	0.156	-3.127	0.002	0.281	0.931
PDE8B	0.445	0.302	1.472	0.141	1.56	0.472	1.188	0.235	0.863	2.822

PDE8A	-0.308	0.298	-1.036	0.3	0.735	0.219	-1.214	0.225	0.41	1.317
PDE7B	0.08	0.295	0.272	0.785	1.084	0.32	0.262	0.793	0.608	1.933
PDE6H	-0.418	0.302	-1.384	0.166	0.658	0.199	-1.719	0.086	0.364	1.19
PDE6G	0.029	0.296	0.097	0.922	1.029	0.305	0.096	0.924	0.576	1.838
PDE6D	0.043	0.3	0.143	0.886	1.044	0.313	0.14	0.889	0.58	1.878
PDE6C	-0.023	0.295	-0.08	0.937	0.977	0.288	-0.08	0.936	0.548	1.742
PDE6B	-0.074	0.3	-0.246	0.805	0.929	0.278	-0.256	0.798	0.516	1.671
PDE6A	0.333	0.298	1.12	0.263	1.396	0.415	0.953	0.341	0.779	2.5
PDE5A	-0.132	0.296	-0.446	0.656	0.876	0.26	-0.477	0.634	0.49	1.566
PDE4DIP	-0.287	0.3	-0.958	0.338	0.75	0.225	-1.11	0.267	0.417	1.35
PDE4D	-0.254	0.296	-0.857	0.391	0.776	0.23	-0.976	0.329	0.434	1.387
PDE4B	-0.203	0.296	-0.685	0.493	0.816	0.242	-0.759	0.448	0.457	1.459
PDE4A	-0.253	0.298	-0.851	0.395	0.776	0.231	-0.968	0.333	0.433	1.392
PDE3B	0.093	0.297	0.313	0.754	1.098	0.326	0.299	0.765	0.613	1.966
PDE3A	0.186	0.296	0.628	0.53	1.204	0.356	0.573	0.567	0.675	2.149
PDE2A	-0.451	0.302	-1.491	0.136	0.637	0.193	-1.883	0.06	0.352	1.153
PDE1C	-0.616	0.306	-2.012	0.044	0.54	0.165	-2.781	0.005	0.296	0.984
PDE1B	0.022	0.295	0.076	0.939	1.023	0.302	0.075	0.94	0.573	1.825
PDE1A	-1.131	0.328	-3.448	0.001	0.323	0.106	-6.399	0	0.17	0.614
PDE12	0.159	0.295	0.54	0.589	1.173	0.346	0.499	0.618	0.657	2.093
PDE11A	-0.175	0.296	-0.59	0.555	0.84	0.249	-0.645	0.519	0.47	1.5
PDE10A	-0.725	0.306	-2.367	0.018	0.485	0.148	-3.475	0.001	0.266	0.883
PDCL	0.052	0.295	0.175	0.861	1.053	0.311	0.171	0.864	0.59	1.879
PDCD6IP	-0.082	0.296	-0.277	0.782	0.921	0.272	-0.289	0.773	0.516	1.644
PDCD6	0.692	0.304	2.274	0.023	1.998	0.608	1.641	0.101	1.1	3.626
PDCD5	0.636	0.303	2.097	0.036	1.888	0.572	1.552	0.121	1.042	3.421
PDCD4-AS1	-0.2	0.296	-0.674	0.5	0.819	0.243	-0.746	0.456	0.458	1.463
PDCD2	0.057	0.296	0.192	0.848	1.058	0.313	0.186	0.852	0.593	1.89
PDCD1LG2	0.05	0.295	0.169	0.866	1.051	0.31	0.165	0.869	0.589	1.875
PDCD11	0.329	0.3	1.095	0.273	1.389	0.417	0.933	0.351	0.771	2.502
PDCD10	0.864	0.315	2.739	0.006	2.373	0.748	1.834	0.067	1.279	4.403
PDCD1	0.322	0.297	1.083	0.279	1.38	0.41	0.926	0.354	0.771	2.472
PDC	0.117	0.295	0.396	0.692	1.124	0.332	0.374	0.709	0.63	2.005
PDAP1	0.264	0.296	0.891	0.373	1.302	0.385	0.784	0.433	0.729	2.326
PCYT2	0.246	0.296	0.831	0.406	1.279	0.379	0.737	0.461	0.716	2.287
PCYT1B	-0.558	0.303	-1.84	0.066	0.573	0.173	-2.463	0.014	0.316	1.037
PCYT1A	0.294	0.298	0.989	0.323	1.342	0.4	0.857	0.392	0.749	2.406
PCYOX1L	0.289	0.298	0.972	0.331	1.336	0.398	0.844	0.399	0.745	2.394

PCYOX1	0.082	0.296	0.279	0.78	1.086	0.321	0.268	0.789	0.608	1.939
PCTP	0.397	0.3	1.324	0.185	1.487	0.446	1.093	0.274	0.827	2.677
PCSK7	-0.757	0.31	-2.442	0.015	0.469	0.145	-3.652	0	0.255	0.861
PCSK6	-0.366	0.298	-1.23	0.219	0.693	0.206	-1.486	0.137	0.387	1.243
PCSK5	-0.44	0.3	-1.467	0.142	0.644	0.193	-1.842	0.065	0.358	1.159
PCSK2	0.621	0.303	2.048	0.041	1.86	0.564	1.526	0.127	1.027	3.369
PCSK1N	-0.153	0.296	-0.515	0.607	0.859	0.254	-0.556	0.578	0.48	1.534
PCSK1	-0.058	0.295	-0.195	0.845	0.944	0.279	-0.201	0.841	0.529	1.684
PCP4	0.391	0.3	1.305	0.192	1.478	0.443	1.08	0.28	0.822	2.66
PCOTH	-0.257	0.297	-0.863	0.388	0.774	0.23	-0.984	0.325	0.432	1.386
PCOLCE2	0.394	0.3	1.316	0.188	1.483	0.444	1.088	0.277	0.825	2.668
PCOLCE	-0.878	0.315	-2.789	0.005	0.416	0.131	-4.466	0	0.224	0.77
PCNX4	-0.07	0.296	-0.237	0.812	0.932	0.276	-0.246	0.806	0.522	1.664
PCNX2	-0.18	0.296	-0.61	0.542	0.835	0.247	-0.668	0.504	0.468	1.49
PCNX1	-0.057	0.295	-0.194	0.846	0.944	0.279	-0.199	0.842	0.53	1.684
PCNT	0.075	0.298	0.251	0.802	1.078	0.321	0.242	0.809	0.601	1.931
PCNP	-0.215	0.296	-0.726	0.468	0.807	0.239	-0.81	0.418	0.451	1.441
PCNA	0.562	0.302	1.859	0.063	1.755	0.531	1.422	0.155	0.97	3.173
PCMTD2	0.074	0.296	0.25	0.803	1.077	0.318	0.241	0.81	0.603	1.922
PCMT1	0.225	0.296	0.761	0.447	1.253	0.371	0.681	0.496	0.701	2.239
PCM1	-0.435	0.302	-1.438	0.15	0.647	0.196	-1.801	0.072	0.358	1.171
PCLO	0.105	0.296	0.355	0.723	1.111	0.329	0.337	0.736	0.622	1.984
PCLAF	0.692	0.306	2.265	0.024	1.998	0.611	1.634	0.102	1.098	3.637
PCK2	0.765	0.31	2.467	0.014	2.149	0.667	1.724	0.085	1.17	3.947
PCK1	0.236	0.297	0.797	0.426	1.267	0.376	0.709	0.478	0.708	2.265
PCIF1	-0.329	0.298	-1.106	0.269	0.719	0.214	-1.31	0.19	0.401	1.29
PCID2	0.308	0.297	1.036	0.3	1.361	0.404	0.892	0.372	0.76	2.436
PCGF3	0.215	0.295	0.727	0.467	1.239	0.366	0.654	0.513	0.695	2.212
PCGF2	-0.592	0.304	-1.948	0.051	0.553	0.168	-2.657	0.008	0.305	1.004
PCGF1	0.045	0.296	0.151	0.88	1.046	0.309	0.148	0.883	0.586	1.867
PCF11	-0.108	0.296	-0.363	0.716	0.898	0.266	-0.384	0.701	0.503	1.604
PCDHGC3	-0.178	0.296	-0.602	0.547	0.837	0.248	-0.659	0.51	0.468	1.495
PCDHGB6	0.145	0.296	0.492	0.623	1.157	0.342	0.458	0.647	0.648	2.065
PCDHGB5	-0.029	0.295	-0.1	0.921	0.971	0.287	-0.101	0.919	0.544	1.732
PCDHGA9	-0.027	0.297	-0.092	0.927	0.973	0.289	-0.093	0.926	0.544	1.741
PCDHGA8	0.639	0.306	2.087	0.037	1.894	0.58	1.543	0.123	1.04	3.45
PCDHGA3	-0.511	0.301	-1.698	0.089	0.6	0.18	-2.217	0.027	0.333	1.082
PCDHGA10	-0.111	0.296	-0.375	0.707	0.895	0.265	-0.397	0.691	0.5	1.6

PCDHGA1	-0.079	0.296	-0.266	0.79	0.924	0.274	-0.277	0.782	0.517	1.652
PCDHB8	0.457	0.3	1.527	0.127	1.58	0.473	1.225	0.22	0.878	2.841
PCDHB6	0.185	0.296	0.625	0.532	1.203	0.356	0.57	0.569	0.673	2.15
PCDHB3	0.227	0.297	0.765	0.445	1.255	0.372	0.684	0.494	0.702	2.243
PCDHB17P	-0.123	0.296	-0.417	0.676	0.884	0.261	-0.444	0.657	0.495	1.578
PCDHB13	0.064	0.295	0.216	0.829	1.066	0.315	0.209	0.834	0.598	1.901
PCDHB12	-0.428	0.3	-1.426	0.154	0.652	0.195	-1.78	0.075	0.362	1.174
PCDHB11	0.029	0.296	0.098	0.922	1.029	0.304	0.096	0.923	0.576	1.838
PCDHB1	0.038	0.297	0.128	0.898	1.039	0.308	0.126	0.9	0.581	1.859
PCDHA9	0.338	0.308	1.095	0.274	1.402	0.432	0.929	0.353	0.766	2.565
PCDHA5	0.346	0.298	1.163	0.245	1.413	0.421	0.983	0.326	0.789	2.533
PCDHA2	-0.034	0.295	-0.117	0.907	0.966	0.285	-0.119	0.906	0.542	1.723
PCDHA10	-0.33	0.3	-1.1	0.272	0.719	0.216	-1.302	0.193	0.4	1.294
PCDH9	-0.519	0.302	-1.717	0.086	0.595	0.18	-2.252	0.024	0.329	1.076
PCDH8	0.359	0.298	1.208	0.227	1.432	0.426	1.015	0.31	0.799	2.567
PCDH7	-0.951	0.321	-2.966	0.003	0.386	0.124	-4.953	0	0.206	0.724
PCDH17	0.15	0.296	0.508	0.611	1.162	0.344	0.472	0.637	0.651	2.075
PCDH12	-0.308	0.3	-1.028	0.304	0.735	0.22	-1.204	0.228	0.408	1.322
PCDH11X	0.09	0.296	0.305	0.76	1.095	0.324	0.292	0.77	0.613	1.954
PCDH1	-0.171	0.297	-0.575	0.565	0.843	0.251	-0.627	0.531	0.471	1.509
PCCB	0.102	0.295	0.344	0.731	1.107	0.327	0.327	0.743	0.621	1.974
PCCA	0.12	0.296	0.405	0.686	1.127	0.333	0.381	0.703	0.631	2.013
PCBP4	0.448	0.3	1.494	0.135	1.565	0.469	1.204	0.229	0.869	2.818
PCBP3	0.014	0.297	0.046	0.963	1.014	0.301	0.046	0.963	0.567	1.814
PCBP2	-0.775	0.311	-2.492	0.013	0.461	0.143	-3.765	0	0.25	0.847
PCBP1	-0.325	0.301	-1.083	0.279	0.722	0.217	-1.28	0.201	0.401	1.302
PCBD1	-0.574	0.306	-1.878	0.06	0.563	0.172	-2.537	0.011	0.31	1.025
PC	0.101	0.295	0.343	0.731	1.107	0.327	0.327	0.744	0.62	1.975
PBXIP1	0.089	0.296	0.302	0.763	1.093	0.323	0.289	0.773	0.613	1.951
PBX3	-0.588	0.306	-1.925	0.054	0.555	0.17	-2.621	0.009	0.305	1.011
PBX2	0.451	0.302	1.491	0.136	1.57	0.475	1.2	0.23	0.868	2.84
PBX1	-0.1	0.295	-0.34	0.734	0.904	0.267	-0.358	0.721	0.507	1.613
PBRM1	0.145	0.296	0.489	0.625	1.156	0.343	0.455	0.649	0.647	2.067
PBOV1	0.633	0.306	2.065	0.039	1.883	0.577	1.53	0.126	1.033	3.432
PBLD	0.588	0.303	1.94	0.052	1.8	0.546	1.467	0.142	0.994	3.261
PBK	0.946	0.315	3.001	0.003	2.576	0.812	1.941	0.052	1.389	4.78
PAXIP1	0.101	0.295	0.343	0.731	1.107	0.327	0.326	0.744	0.621	1.973
PAXBP1	0.178	0.296	0.6	0.548	1.195	0.354	0.55	0.582	0.668	2.135

PAX9	-0.101	0.295	-0.341	0.733	0.904	0.267	-0.359	0.72	0.507	1.613
PAX8	-0.367	0.299	-1.228	0.219	0.693	0.207	-1.484	0.138	0.386	1.244
PAX7	0.61	0.306	1.996	0.046	1.841	0.563	1.494	0.135	1.011	3.353
PAX6	0.201	0.296	0.678	0.498	1.222	0.362	0.615	0.539	0.684	2.184
PAX5	-0.686	0.306	-2.238	0.025	0.504	0.154	-3.216	0.001	0.276	0.918
PAX4	0.064	0.295	0.217	0.828	1.066	0.315	0.211	0.833	0.598	1.901
PAX3	0.214	0.298	0.719	0.472	1.239	0.369	0.647	0.517	0.691	2.222
PAX2	0.048	0.296	0.161	0.872	1.049	0.31	0.158	0.875	0.588	1.872
PAX1	-0.265	0.296	-0.894	0.371	0.767	0.227	-1.023	0.306	0.429	1.372
PAWR	-0.347	0.298	-1.166	0.244	0.707	0.21	-1.394	0.163	0.394	1.267
PATZ1	0.194	0.296	0.655	0.512	1.214	0.36	0.596	0.551	0.679	2.17
PATJ	-0.339	0.3	-1.13	0.258	0.713	0.214	-1.345	0.179	0.396	1.282
PASK	-0.297	0.298	-0.997	0.319	0.743	0.222	-1.16	0.246	0.414	1.333
PARVB	-0.263	0.297	-0.883	0.377	0.769	0.229	-1.01	0.313	0.429	1.377
PARVA	-1.049	0.328	-3.201	0.001	0.35	0.115	-5.659	0	0.184	0.666
PART1	-0.053	0.295	-0.179	0.858	0.948	0.28	-0.184	0.854	0.531	1.692
PARPBP	0.487	0.3	1.625	0.104	1.627	0.488	1.286	0.198	0.904	2.927
PARP8	-0.258	0.296	-0.872	0.383	0.772	0.229	-0.995	0.32	0.432	1.38
PARP6	0.195	0.299	0.655	0.513	1.216	0.363	0.595	0.552	0.677	2.183
PARP4	0.247	0.3	0.822	0.411	1.28	0.385	0.728	0.466	0.71	2.307
PARP3	-0.357	0.3	-1.188	0.235	0.7	0.21	-1.428	0.153	0.389	1.261
PARP2	0.554	0.306	1.809	0.07	1.74	0.533	1.389	0.165	0.955	3.17
PARP16	-0.255	0.298	-0.858	0.391	0.775	0.231	-0.977	0.329	0.432	1.388
PARP12	0.487	0.3	1.622	0.105	1.627	0.488	1.285	0.199	0.904	2.929
PARP11	0.66	0.303	2.177	0.029	1.935	0.587	1.594	0.111	1.068	3.506
PARP1	0.235	0.296	0.792	0.428	1.264	0.374	0.706	0.48	0.708	2.259
PARN	-0.239	0.296	-0.808	0.419	0.787	0.233	-0.913	0.361	0.441	1.407
PARM1-AS1	-0.011	0.296	-0.037	0.971	0.989	0.292	-0.037	0.97	0.554	1.765
PARM1	0.408	0.3	1.362	0.173	1.504	0.45	1.118	0.263	0.836	2.705
PARL	0.84	0.315	2.671	0.008	2.317	0.729	1.807	0.071	1.251	4.294
PARK7	-0.249	0.3	-0.828	0.408	0.78	0.234	-0.94	0.347	0.433	1.405
PARG	0.613	0.303	2.024	0.043	1.846	0.559	1.513	0.13	1.02	3.341
PARD6B	-1.008	0.328	-3.074	0.002	0.365	0.12	-5.307	0	0.192	0.694
PARD6A	0.607	0.302	2.013	0.044	1.835	0.554	1.509	0.131	1.016	3.315
PARD3	0.456	0.302	1.51	0.131	1.578	0.477	1.212	0.225	0.873	2.855
PAQR6	0.223	0.296	0.755	0.451	1.25	0.37	0.676	0.499	0.7	2.234
PAQR5	0.359	0.298	1.205	0.228	1.431	0.426	1.013	0.311	0.799	2.565
PAQR4	0.493	0.302	1.632	0.103	1.637	0.495	1.288	0.198	0.906	2.961

PAQR3	0.199	0.299	0.666	0.505	1.22	0.364	0.604	0.546	0.68	2.191
PAPSS2	0.448	0.3	1.495	0.135	1.565	0.469	1.205	0.228	0.87	2.815
PAPSS1	-0.6	0.306	-1.961	0.05	0.549	0.168	-2.687	0.007	0.301	1
PAPPA2	0.015	0.296	0.049	0.961	1.015	0.3	0.049	0.961	0.568	1.812
PAPPA	0.173	0.297	0.581	0.561	1.189	0.353	0.534	0.593	0.664	2.128
PAPOLG	-0.533	0.303	-1.761	0.078	0.587	0.178	-2.326	0.02	0.324	1.062
PAPOLB	-0.154	0.296	-0.522	0.602	0.857	0.254	-0.564	0.573	0.48	1.531
PAPOLA	-0.303	0.298	-1.017	0.309	0.739	0.22	-1.187	0.235	0.412	1.324
PAOX	-0.23	0.298	-0.772	0.44	0.795	0.236	-0.868	0.385	0.443	1.424
PANX1	0.02	0.295	0.069	0.945	1.021	0.301	0.068	0.945	0.572	1.82
PANK4	-0.01	0.295	-0.033	0.974	0.99	0.293	-0.033	0.974	0.555	1.767
PANK3	-0.665	0.31	-2.146	0.032	0.514	0.159	-3.048	0.002	0.28	0.944
PANK2	0.729	0.31	2.352	0.019	2.073	0.642	1.67	0.095	1.129	3.805
PAN2	-0.44	0.3	-1.465	0.143	0.644	0.193	-1.839	0.066	0.358	1.16
PAMR1	-0.256	0.298	-0.861	0.389	0.774	0.23	-0.982	0.326	0.432	1.387
PAM16	0.171	0.295	0.579	0.562	1.187	0.351	0.533	0.594	0.665	2.118
PAM	-0.254	0.298	-0.854	0.393	0.776	0.231	-0.972	0.331	0.433	1.39
PALMD	-0.333	0.298	-1.118	0.263	0.717	0.213	-1.327	0.184	0.4	1.285
PALM	-0.13	0.295	-0.44	0.66	0.878	0.259	-0.47	0.638	0.492	1.566
PALLD	-0.426	0.3	-1.42	0.156	0.653	0.196	-1.77	0.077	0.363	1.176
PALB2	0.46	0.302	1.523	0.128	1.585	0.479	1.22	0.222	0.876	2.866
PAK6	0.069	0.295	0.235	0.814	1.072	0.316	0.227	0.821	0.601	1.912
PAK5	0.039	0.296	0.132	0.895	1.04	0.308	0.129	0.897	0.582	1.858
PAK4	-0.553	0.302	-1.831	0.067	0.575	0.174	-2.445	0.014	0.318	1.04
PAK3	0.068	0.298	0.23	0.818	1.071	0.319	0.222	0.824	0.597	1.921
PAK2	0.057	0.295	0.193	0.847	1.059	0.313	0.187	0.851	0.593	1.889
PAK1IP1	0.135	0.295	0.456	0.649	1.144	0.338	0.426	0.67	0.641	2.041
PAK1	-0.077	0.296	-0.26	0.795	0.926	0.274	-0.27	0.787	0.518	1.655
PAIP2B	-0.502	0.302	-1.663	0.096	0.605	0.183	-2.16	0.031	0.335	1.094
PAIP1	0.257	0.296	0.868	0.385	1.293	0.383	0.766	0.444	0.724	2.312
PAICS	0.546	0.306	1.786	0.074	1.726	0.528	1.376	0.169	0.948	3.142
PAGR1	-0.377	0.302	-1.247	0.212	0.686	0.207	-1.515	0.13	0.379	1.241
PAGE4	0.15	0.296	0.505	0.613	1.161	0.344	0.469	0.639	0.65	2.075
PAGE1	-0.047	0.295	-0.158	0.875	0.954	0.282	-0.162	0.872	0.535	1.703
PAFAH2	-0.377	0.302	-1.248	0.212	0.686	0.207	-1.516	0.13	0.379	1.24
PAFAH1B3	0.346	0.299	1.155	0.248	1.413	0.423	0.977	0.329	0.786	2.539
PAFAH1B2	0.299	0.298	1.004	0.315	1.349	0.402	0.868	0.385	0.752	2.42
PAFAH1B1	-0.613	0.306	-2.007	0.045	0.542	0.166	-2.77	0.006	0.297	0.986

PAF1	0.347	0.299	1.162	0.245	1.415	0.423	0.981	0.326	0.788	2.544
PAEP	0.601	0.306	1.967	0.049	1.824	0.557	1.478	0.139	1.002	3.32
PADI4	0.023	0.298	0.078	0.938	1.024	0.304	0.077	0.938	0.571	1.834
PADI3	-0.413	0.301	-1.372	0.17	0.662	0.199	-1.698	0.09	0.367	1.193
PADI2	-0.019	0.296	-0.065	0.948	0.981	0.29	-0.066	0.947	0.549	1.752
PADI1	-0.045	0.295	-0.152	0.879	0.956	0.282	-0.156	0.876	0.536	1.705
PACSIN3	0.237	0.297	0.798	0.425	1.268	0.377	0.711	0.477	0.708	2.27
PACSIN2	-0.49	0.3	-1.631	0.103	0.613	0.184	-2.104	0.035	0.34	1.104
PACS2	-0.256	0.296	-0.864	0.387	0.774	0.229	-0.985	0.325	0.433	1.383
PACS1	0.72	0.305	2.36	0.018	2.055	0.627	1.682	0.092	1.13	3.738
PACRG	-0.362	0.299	-1.213	0.225	0.696	0.208	-1.461	0.144	0.388	1.25
PACC1	0.123	0.295	0.415	0.678	1.131	0.334	0.391	0.696	0.634	2.017
PABPN1	-0.028	0.301	-0.092	0.927	0.973	0.292	-0.093	0.926	0.54	1.753
PABPC4	-0.278	0.296	-0.938	0.348	0.757	0.225	-1.081	0.28	0.423	1.354
PABPC3	0.018	0.295	0.061	0.951	1.018	0.301	0.061	0.952	0.571	1.817
PABPC1	-0.036	0.297	-0.122	0.903	0.964	0.286	-0.124	0.901	0.539	1.725
PAAF1	0.186	0.296	0.628	0.53	1.205	0.357	0.573	0.566	0.674	2.152
PA2G4	0.394	0.3	1.314	0.189	1.482	0.444	1.086	0.277	0.824	2.667
P4HTM	-0.533	0.306	-1.745	0.081	0.587	0.179	-2.306	0.021	0.322	1.068
P4HB	-0.367	0.298	-1.231	0.218	0.693	0.206	-1.488	0.137	0.386	1.242
P4HA2	0.375	0.298	1.259	0.208	1.455	0.433	1.05	0.294	0.812	2.608
P4HA1	-0.14	0.296	-0.474	0.636	0.869	0.258	-0.509	0.611	0.486	1.554
P3H4	-0.108	0.296	-0.366	0.715	0.897	0.266	-0.386	0.699	0.502	1.604
P3H3	-0.958	0.321	-2.986	0.003	0.383	0.123	-5.009	0	0.204	0.719
P3H2	-0.236	0.296	-0.797	0.426	0.79	0.234	-0.899	0.369	0.442	1.411
P3H1	0.029	0.295	0.099	0.921	1.03	0.304	0.098	0.922	0.577	1.837
P2RY6	0.465	0.303	1.535	0.125	1.591	0.482	1.228	0.219	0.879	2.88
P2RY4	-0.109	0.296	-0.367	0.713	0.897	0.265	-0.388	0.698	0.502	1.602
P2RY2	0.002	0.295	0.006	0.995	1.002	0.296	0.006	0.995	0.562	1.786
P2RY14	-0.385	0.298	-1.294	0.196	0.68	0.203	-1.578	0.114	0.38	1.219
P2RY13	-0.945	0.321	-2.946	0.003	0.389	0.125	-4.904	0	0.207	0.729
P2RY10	-0.034	0.295	-0.116	0.908	0.966	0.285	-0.118	0.906	0.542	1.724
P2RY1	0.036	0.295	0.121	0.904	1.036	0.306	0.119	0.905	0.581	1.849
P2RX7	0.047	0.295	0.16	0.873	1.049	0.31	0.157	0.876	0.588	1.871
P2RX6	0.363	0.3	1.21	0.226	1.437	0.431	1.015	0.31	0.799	2.585
P2RX5	-0.434	0.299	-1.453	0.146	0.648	0.193	-1.82	0.069	0.361	1.163
P2RX4	-0.487	0.306	-1.592	0.111	0.614	0.188	-2.052	0.04	0.337	1.119
P2RX3	-0.086	0.299	-0.289	0.772	0.917	0.274	-0.302	0.763	0.511	1.648

P2RX2	0.505	0.3	1.682	0.093	1.656	0.497	1.321	0.187	0.92	2.981
P2RX1	-0.637	0.306	-2.08	0.037	0.529	0.162	-2.909	0.004	0.29	0.964
OXTR	-0.088	0.296	-0.297	0.767	0.916	0.271	-0.31	0.756	0.512	1.637
OXT	-0.133	0.296	-0.45	0.653	0.875	0.259	-0.481	0.63	0.49	1.564
OXSR1	0.277	0.298	0.929	0.353	1.319	0.392	0.812	0.417	0.736	2.363
OXSM	0.284	0.298	0.953	0.34	1.328	0.395	0.83	0.407	0.741	2.379
OXR1	-0.007	0.295	-0.023	0.982	0.993	0.293	-0.023	0.982	0.557	1.772
OXLD1	0.457	0.3	1.524	0.128	1.579	0.473	1.223	0.221	0.877	2.84
OXCT2	0.475	0.302	1.572	0.116	1.608	0.486	1.251	0.211	0.889	2.909
OXCT1	-0.371	0.298	-1.246	0.213	0.69	0.206	-1.509	0.131	0.385	1.237
OXA1L	-0.445	0.302	-1.472	0.141	0.641	0.194	-1.853	0.064	0.354	1.159
OVOL3	-0.154	0.296	-0.521	0.602	0.857	0.254	-0.564	0.573	0.479	1.532
OVOL2	-0.122	0.298	-0.408	0.683	0.886	0.264	-0.434	0.664	0.494	1.588
OVOL1	-0.272	0.298	-0.915	0.36	0.762	0.227	-1.052	0.293	0.425	1.365
OVGP1	-0.052	0.296	-0.175	0.861	0.95	0.281	-0.18	0.857	0.532	1.695
OTULINL	-0.24	0.298	-0.803	0.422	0.787	0.235	-0.908	0.364	0.439	1.412
OTUD7B	-0.044	0.295	-0.151	0.88	0.957	0.282	-0.154	0.878	0.536	1.706
OTUD4	0.065	0.3	0.215	0.83	1.067	0.32	0.208	0.835	0.592	1.921
OTUD3	-0.157	0.296	-0.53	0.596	0.855	0.253	-0.574	0.566	0.478	1.527
OTUB2	0.075	0.297	0.252	0.801	1.078	0.32	0.243	0.808	0.602	1.928
OTOR	0.471	0.303	1.556	0.12	1.601	0.485	1.241	0.215	0.885	2.898
OTOF	-0.145	0.297	-0.488	0.626	0.865	0.257	-0.525	0.6	0.484	1.547
OTC	-0.337	0.298	-1.13	0.259	0.714	0.213	-1.343	0.179	0.398	1.281
OSTM1	-0.643	0.31	-2.076	0.038	0.526	0.163	-2.912	0.004	0.286	0.965
OSTF1	1.004	0.322	3.115	0.002	2.729	0.879	1.966	0.049	1.451	5.132
OSR2	0.141	0.295	0.478	0.633	1.152	0.34	0.446	0.656	0.646	2.054
OSMR	-0.57	0.303	-1.882	0.06	0.565	0.171	-2.538	0.011	0.312	1.024
OSM	0.378	0.298	1.268	0.205	1.459	0.435	1.056	0.291	0.813	2.618
OSGIN2	0.368	0.298	1.235	0.217	1.444	0.43	1.034	0.301	0.806	2.589
OSGIN1	-0.171	0.296	-0.578	0.563	0.843	0.25	-0.631	0.528	0.471	1.506
OSGEPL1	-0.301	0.297	-1.011	0.312	0.74	0.22	-1.18	0.238	0.413	1.326
OSGEP	0.029	0.295	0.098	0.922	1.029	0.304	0.096	0.923	0.577	1.837
OSER1	0.131	0.296	0.441	0.659	1.14	0.338	0.413	0.679	0.637	2.038
OSBPL9	-0.089	0.295	-0.301	0.764	0.915	0.27	-0.314	0.753	0.513	1.632
OSBPL8	-0.674	0.31	-2.174	0.03	0.51	0.158	-3.104	0.002	0.278	0.936
OSBPL7	0.229	0.296	0.773	0.44	1.257	0.372	0.691	0.49	0.704	2.246
OSBPL3	-0.192	0.297	-0.646	0.518	0.825	0.245	-0.712	0.476	0.461	1.478
OSBPL2	0.36	0.298	1.21	0.226	1.434	0.427	1.016	0.31	0.8	2.569

OSBPL1A	-0.821	0.321	-2.556	0.011	0.44	0.141	-3.962	0	0.235	0.826
OSBPL11	0.492	0.3	1.64	0.101	1.635	0.49	1.295	0.195	0.908	2.943
OSBPL10	0.23	0.296	0.776	0.438	1.258	0.373	0.693	0.488	0.704	2.249
OSBP2	-0.463	0.3	-1.544	0.123	0.63	0.189	-1.963	0.05	0.35	1.133
OSBP	0.035	0.295	0.118	0.906	1.035	0.305	0.116	0.908	0.581	1.846
OS9	-0.954	0.314	-3.039	0.002	0.385	0.121	-5.084	0	0.208	0.713
ORMDL2	0.147	0.295	0.497	0.619	1.158	0.342	0.462	0.644	0.649	2.066
ORM1	0.053	0.295	0.181	0.856	1.055	0.312	0.176	0.86	0.591	1.882
ORC6	0.866	0.321	2.698	0.007	2.378	0.763	1.805	0.071	1.267	4.461
ORC5	-0.287	0.298	-0.964	0.335	0.751	0.223	-1.117	0.264	0.419	1.345
ORC4	0.469	0.302	1.552	0.121	1.599	0.484	1.239	0.215	0.884	2.893
ORC3	0.164	0.296	0.554	0.58	1.178	0.348	0.511	0.609	0.66	2.103
ORC2	0.14	0.295	0.473	0.636	1.15	0.34	0.441	0.659	0.644	2.052
ORC1	0.614	0.306	2.009	0.045	1.848	0.565	1.501	0.133	1.015	3.365
ORAI3	-0.125	0.295	-0.423	0.673	0.883	0.261	-0.45	0.653	0.495	1.574
ORAI2	-0.326	0.3	-1.085	0.278	0.722	0.217	-1.283	0.199	0.401	1.3
OR7E47P	0.295	0.297	0.993	0.321	1.342	0.398	0.86	0.39	0.751	2.401
OR7E2P	-0.575	0.306	-1.882	0.06	0.563	0.172	-2.543	0.011	0.309	1.024
OR7E24	0.114	0.296	0.387	0.699	1.121	0.331	0.365	0.715	0.628	2.001
OR7E19P	-0.441	0.3	-1.47	0.142	0.643	0.193	-1.848	0.065	0.357	1.158
OR7E156P	0.237	0.298	0.798	0.425	1.268	0.378	0.71	0.478	0.707	2.273
OR7E14P	0.273	0.297	0.919	0.358	1.314	0.39	0.804	0.421	0.734	2.352
OR7E12P	0.292	0.298	0.98	0.327	1.339	0.398	0.85	0.395	0.747	2.399
OR7C2	-0.632	0.306	-2.067	0.039	0.531	0.163	-2.883	0.004	0.292	0.968
OR7C1	0.372	0.298	1.249	0.212	1.451	0.432	1.043	0.297	0.809	2.601
OR7A5	0.527	0.307	1.719	0.086	1.694	0.519	1.336	0.181	0.929	3.09
OR7A17	-0.224	0.297	-0.755	0.45	0.799	0.237	-0.846	0.397	0.447	1.43
OR7A10	0.146	0.297	0.492	0.623	1.157	0.343	0.458	0.647	0.647	2.07
OR6A2	-0.027	0.297	-0.089	0.929	0.974	0.289	-0.091	0.928	0.544	1.743
OR5I1	0.062	0.295	0.209	0.834	1.064	0.314	0.203	0.839	0.596	1.898
OR52A1	0.136	0.296	0.46	0.645	1.146	0.34	0.43	0.667	0.641	2.048
OR51E2	0.254	0.298	0.853	0.393	1.289	0.384	0.754	0.451	0.719	2.31
OR3A3	0.459	0.303	1.516	0.129	1.582	0.479	1.216	0.224	0.874	2.863
OR3A2	0.406	0.303	1.343	0.179	1.501	0.454	1.104	0.27	0.83	2.716
OR3A1	-0.117	0.295	-0.395	0.693	0.89	0.263	-0.419	0.675	0.499	1.588
OR2W1	0.026	0.295	0.088	0.93	1.026	0.303	0.087	0.931	0.576	1.83
OR2S2	0.03	0.295	0.102	0.919	1.031	0.304	0.1	0.92	0.578	1.838
OR2J3	-0.159	0.295	-0.537	0.591	0.853	0.252	-0.582	0.56	0.478	1.522

OR2J2	0.202	0.296	0.682	0.495	1.224	0.362	0.617	0.537	0.685	2.186
OR2H2	-0.058	0.296	-0.197	0.844	0.943	0.279	-0.203	0.839	0.529	1.684
OR2H1	0.042	0.296	0.142	0.887	1.043	0.308	0.139	0.889	0.584	1.862
OR2F2	0.091	0.295	0.307	0.759	1.095	0.323	0.294	0.769	0.614	1.952
OR2F1	0.069	0.296	0.232	0.816	1.071	0.317	0.225	0.822	0.6	1.912
OR2C1	0.088	0.296	0.297	0.767	1.092	0.324	0.284	0.776	0.611	1.952
OR2B6	0.155	0.295	0.524	0.6	1.167	0.345	0.486	0.627	0.654	2.083
OR2B2	-0.083	0.296	-0.28	0.78	0.921	0.272	-0.292	0.771	0.516	1.644
OR1G1	-0.33	0.298	-1.107	0.268	0.719	0.214	-1.311	0.19	0.401	1.289
OR1F2P	0.182	0.296	0.613	0.54	1.199	0.355	0.561	0.575	0.671	2.144
OR1F1	0.533	0.302	1.764	0.078	1.704	0.515	1.367	0.172	0.942	3.082
OR1E1	-0.131	0.295	-0.445	0.657	0.877	0.259	-0.475	0.635	0.492	1.564
OR1D2	0.02	0.295	0.069	0.945	1.021	0.301	0.068	0.946	0.572	1.82
OR1A2	0.203	0.296	0.685	0.493	1.225	0.363	0.62	0.535	0.685	2.19
OR1A1	-0.42	0.3	-1.399	0.162	0.657	0.197	-1.738	0.082	0.365	1.184
OR12D3	-0.101	0.296	-0.342	0.733	0.904	0.267	-0.359	0.719	0.507	1.613
OR12D2	0.124	0.295	0.419	0.675	1.132	0.334	0.394	0.693	0.634	2.019
OR11A1	0.014	0.295	0.049	0.961	1.014	0.299	0.048	0.961	0.569	1.809
OR10J1	-0.371	0.3	-1.236	0.217	0.69	0.207	-1.496	0.135	0.384	1.243
OR10H3	-0.412	0.3	-1.373	0.17	0.662	0.199	-1.7	0.089	0.368	1.192
OR10H2	0.788	0.31	2.542	0.011	2.199	0.682	1.759	0.079	1.198	4.036
OR10H1	0.036	0.295	0.122	0.903	1.037	0.306	0.12	0.905	0.581	1.848
OR10C1	0.136	0.296	0.459	0.646	1.146	0.339	0.429	0.668	0.641	2.048
OPTN	0.657	0.306	2.147	0.032	1.929	0.59	1.573	0.116	1.059	3.515
OPRPN	-0.308	0.298	-1.035	0.3	0.735	0.219	-1.213	0.225	0.41	1.317
OPRM1	0.4	0.3	1.333	0.182	1.491	0.447	1.099	0.272	0.829	2.683
OPRL1	0.059	0.296	0.199	0.842	1.061	0.314	0.194	0.846	0.594	1.894
OPRK1	0.396	0.3	1.317	0.188	1.485	0.446	1.088	0.277	0.824	2.677
OPRD1	-0.588	0.303	-1.942	0.052	0.556	0.168	-2.643	0.008	0.307	1.005
OPN3	0.291	0.298	0.978	0.328	1.338	0.398	0.848	0.396	0.746	2.397
OPN1SW	0.233	0.296	0.787	0.431	1.263	0.374	0.702	0.483	0.707	2.256
OPLAH	0.197	0.296	0.666	0.506	1.218	0.361	0.604	0.546	0.682	2.176
OPHN1	0.062	0.295	0.21	0.834	1.064	0.314	0.204	0.839	0.597	1.897
OPCML	-0.934	0.321	-2.911	0.004	0.393	0.126	-4.815	0	0.209	0.737
OPA3	0.627	0.306	2.051	0.04	1.872	0.573	1.523	0.128	1.028	3.409
OPA1	0.462	0.303	1.526	0.127	1.587	0.48	1.223	0.221	0.877	2.871
ONECUT2	0.188	0.296	0.634	0.526	1.206	0.357	0.578	0.563	0.675	2.156
ONECUT1	-0.018	0.296	-0.061	0.952	0.982	0.29	-0.061	0.951	0.55	1.753

OMP	0.299	0.297	1.006	0.315	1.349	0.401	0.869	0.385	0.753	2.416
OMD	-1.257	0.337	-3.735	0	0.284	0.096	-7.475	0	0.147	0.55
OLR1	0.243	0.296	0.821	0.412	1.275	0.378	0.728	0.466	0.713	2.28
OLIG2	-0.287	0.296	-0.966	0.334	0.751	0.223	-1.119	0.263	0.42	1.343
OLFML3	-0.862	0.315	-2.739	0.006	0.422	0.133	-4.347	0	0.228	0.782
OLFML2B	-0.576	0.302	-1.906	0.057	0.562	0.17	-2.577	0.01	0.311	1.017
OLFML2A	-0.561	0.306	-1.834	0.067	0.571	0.174	-2.459	0.014	0.314	1.039
OLFML1	-0.541	0.302	-1.789	0.074	0.582	0.176	-2.373	0.018	0.322	1.053
OLFM4	-0.04	0.295	-0.135	0.892	0.961	0.283	-0.138	0.89	0.539	1.713
OLFM1	-0.105	0.296	-0.353	0.724	0.901	0.267	-0.372	0.71	0.504	1.61
OLAH	0.187	0.296	0.633	0.527	1.206	0.357	0.577	0.564	0.675	2.155
OLA1	0.147	0.295	0.496	0.62	1.158	0.342	0.461	0.645	0.649	2.066
OIP5	0.398	0.3	1.328	0.184	1.489	0.446	1.095	0.273	0.827	2.679
OGT	0.242	0.298	0.813	0.416	1.274	0.379	0.722	0.47	0.711	2.283
OGN	-1.027	0.321	-3.198	0.001	0.358	0.115	-5.581	0	0.191	0.672
OGG1	-0.225	0.296	-0.76	0.447	0.798	0.237	-0.852	0.394	0.447	1.427
OGFRL1	0.752	0.315	2.39	0.017	2.121	0.667	1.68	0.093	1.145	3.93
OGFR	-0.344	0.298	-1.154	0.248	0.709	0.211	-1.378	0.168	0.395	1.271
OGFOD3	0.38	0.298	1.275	0.202	1.462	0.436	1.06	0.289	0.815	2.623
OGFOD2	-0.051	0.297	-0.171	0.864	0.95	0.282	-0.176	0.86	0.531	1.701
OGFOD1	-0.249	0.297	-0.838	0.402	0.78	0.232	-0.951	0.341	0.435	1.396
OGDHL	-0.146	0.296	-0.494	0.621	0.864	0.256	-0.532	0.594	0.483	1.543
OGDH	-0.358	0.3	-1.195	0.232	0.699	0.209	-1.438	0.151	0.388	1.258
OGA	-0.324	0.299	-1.085	0.278	0.723	0.216	-1.282	0.2	0.402	1.299
OFD1	0.375	0.3	1.25	0.211	1.454	0.436	1.043	0.297	0.809	2.616
ODR4	0.074	0.295	0.251	0.802	1.077	0.318	0.242	0.809	0.604	1.921
ODF2	-0.731	0.321	-2.276	0.023	0.481	0.155	-3.354	0.001	0.256	0.903
ODF1	0.031	0.296	0.103	0.918	1.031	0.305	0.102	0.919	0.577	1.842
ODC1	-0.252	0.298	-0.846	0.398	0.778	0.231	-0.961	0.336	0.434	1.393
ODAM	0.308	0.3	1.027	0.304	1.361	0.408	0.884	0.377	0.756	2.449
OCRL	0.431	0.3	1.437	0.151	1.538	0.461	1.167	0.243	0.855	2.769
OCM2	0.167	0.295	0.564	0.573	1.181	0.349	0.52	0.603	0.662	2.107
OCLN	-0.054	0.297	-0.183	0.855	0.947	0.281	-0.188	0.851	0.529	1.696
OCEL1	0.416	0.301	1.384	0.166	1.516	0.456	1.132	0.258	0.841	2.735
OCA2	-0.481	0.306	-1.572	0.116	0.618	0.189	-2.02	0.043	0.339	1.126
OBSL1	0.154	0.296	0.52	0.603	1.166	0.345	0.482	0.63	0.653	2.084
OBSCN	-0.015	0.295	-0.052	0.958	0.985	0.291	-0.053	0.958	0.552	1.756
OBP2A	-0.243	0.296	-0.819	0.413	0.784	0.232	-0.927	0.354	0.439	1.402

OBI1	0.215	0.296	0.725	0.468	1.24	0.367	0.653	0.514	0.694	2.215
OAZ3	-0.312	0.298	-1.049	0.294	0.732	0.218	-1.231	0.218	0.409	1.311
OAZ2	-0.212	0.297	-0.716	0.474	0.809	0.24	-0.798	0.425	0.452	1.446
OAZ1	0.535	0.306	1.749	0.08	1.707	0.522	1.354	0.176	0.937	3.107
OAT	-0.048	0.295	-0.164	0.87	0.953	0.281	-0.168	0.867	0.534	1.7
OASL	0.164	0.297	0.551	0.581	1.178	0.349	0.509	0.611	0.659	2.106
OAS3	0.038	0.296	0.128	0.898	1.039	0.307	0.126	0.9	0.582	1.854
OAS2	-0.131	0.296	-0.445	0.657	0.877	0.259	-0.475	0.635	0.491	1.565
OAS1	0.006	0.296	0.019	0.985	1.006	0.297	0.019	0.985	0.563	1.796
OARD1	0.035	0.295	0.12	0.905	1.036	0.306	0.118	0.906	0.581	1.848
NYX	0.023	0.296	0.077	0.939	1.023	0.303	0.076	0.94	0.573	1.827
NYNRIN	0.636	0.306	2.081	0.037	1.889	0.578	1.54	0.124	1.038	3.44
NXT2	-0.252	0.298	-0.845	0.398	0.778	0.231	-0.961	0.336	0.434	1.393
NXT1	0.398	0.298	1.337	0.181	1.489	0.443	1.103	0.27	0.831	2.668
NXPH4	-0.063	0.295	-0.213	0.831	0.939	0.277	-0.22	0.826	0.526	1.675
NXPH3	-0.584	0.302	-1.933	0.053	0.558	0.168	-2.625	0.009	0.308	1.008
NXPE4	-0.554	0.306	-1.814	0.07	0.575	0.176	-2.423	0.015	0.316	1.046
NXPE3	-0.016	0.296	-0.053	0.958	0.984	0.291	-0.054	0.957	0.551	1.757
NXF3	-0.271	0.296	-0.917	0.359	0.762	0.226	-1.053	0.292	0.427	1.362
NXF1	-0.651	0.307	-2.121	0.034	0.522	0.16	-2.989	0.003	0.286	0.952
NVL	0.226	0.296	0.764	0.445	1.254	0.371	0.684	0.494	0.702	2.24
NUTF2	0.743	0.31	2.398	0.016	2.102	0.651	1.692	0.091	1.145	3.859
NUSAP1	0.717	0.306	2.345	0.019	2.048	0.626	1.674	0.094	1.125	3.728
NUS1P3	-0.054	0.295	-0.184	0.854	0.947	0.279	-0.189	0.85	0.531	1.689
NUPR1	-0.135	0.296	-0.455	0.649	0.874	0.259	-0.487	0.626	0.489	1.562
NUP98	-0.311	0.298	-1.042	0.297	0.733	0.218	-1.222	0.222	0.409	1.315
NUP93	0.151	0.295	0.51	0.61	1.162	0.343	0.473	0.636	0.652	2.074
NUP88	0.262	0.296	0.883	0.377	1.299	0.385	0.777	0.437	0.727	2.322
NUP85	0.351	0.3	1.173	0.241	1.421	0.426	0.989	0.323	0.79	2.557
NUP62CL	-0.594	0.31	-1.916	0.055	0.552	0.171	-2.617	0.009	0.301	1.014
NUP62	0.456	0.3	1.521	0.128	1.577	0.472	1.221	0.222	0.877	2.837
NUP58	0.123	0.295	0.418	0.676	1.131	0.334	0.393	0.694	0.634	2.019
NUP54	0.448	0.3	1.496	0.135	1.566	0.469	1.206	0.228	0.87	2.816
NUP50	-0.291	0.298	-0.98	0.327	0.747	0.222	-1.137	0.255	0.417	1.339
NUP43	0.363	0.3	1.211	0.226	1.438	0.431	1.015	0.31	0.799	2.587
NUP42	0.021	0.295	0.07	0.944	1.021	0.301	0.07	0.944	0.573	1.821
NUP37	0.63	0.303	2.077	0.038	1.878	0.57	1.541	0.123	1.036	3.403
NUP214	0.127	0.3	0.424	0.671	1.136	0.34	0.398	0.69	0.631	2.043

NUP210	-0.065	0.295	-0.218	0.827	0.938	0.277	-0.226	0.821	0.525	1.673
NUP205	0.111	0.296	0.374	0.708	1.117	0.331	0.354	0.723	0.625	1.997
NUP188	-0.053	0.295	-0.178	0.858	0.949	0.28	-0.183	0.855	0.532	1.693
NUP160	0.137	0.296	0.461	0.645	1.146	0.34	0.431	0.666	0.641	2.049
NUP155	0.871	0.315	2.764	0.006	2.39	0.753	1.845	0.065	1.289	4.433
NUP153	0.263	0.298	0.882	0.378	1.301	0.388	0.776	0.438	0.725	2.336
NUP133	0.168	0.296	0.566	0.571	1.182	0.35	0.521	0.602	0.662	2.113
NUP107	0.446	0.303	1.47	0.141	1.561	0.473	1.186	0.235	0.862	2.827
NUMA1	-0.461	0.302	-1.526	0.127	0.631	0.191	-1.939	0.053	0.349	1.14
NUFIP1	0.534	0.306	1.746	0.081	1.705	0.521	1.353	0.176	0.937	3.103
NUDT9	0.502	0.3	1.674	0.094	1.651	0.495	1.316	0.188	0.918	2.971
NUDT7	-0.514	0.3	-1.712	0.087	0.598	0.18	-2.239	0.025	0.332	1.077
NUDT6	-0.569	0.311	-1.833	0.067	0.566	0.176	-2.47	0.014	0.308	1.04
NUDT4	-0.716	0.31	-2.307	0.021	0.489	0.152	-3.371	0.001	0.266	0.898
NUDT3	-0.149	0.296	-0.502	0.616	0.862	0.255	-0.541	0.588	0.483	1.54
NUDT21	0.451	0.298	1.512	0.13	1.569	0.468	1.217	0.223	0.875	2.814
NUDT2	0.286	0.298	0.961	0.336	1.332	0.397	0.836	0.403	0.743	2.388
NUDT18	-0.181	0.298	-0.606	0.545	0.835	0.249	-0.664	0.507	0.465	1.498
NUDT15	0.3	0.298	1.007	0.314	1.349	0.402	0.87	0.384	0.753	2.419
NUDT13	-0.273	0.298	-0.918	0.359	0.761	0.226	-1.055	0.291	0.425	1.364
NUDT11	0.052	0.295	0.177	0.86	1.054	0.311	0.172	0.863	0.59	1.88
NUDT1	0.973	0.314	3.101	0.002	2.646	0.83	1.983	0.047	1.431	4.893
NUDCD3	0.308	0.298	1.035	0.301	1.361	0.405	0.891	0.373	0.759	2.44
NUDC	-0.415	0.303	-1.37	0.171	0.661	0.2	-1.698	0.09	0.365	1.195
NUCKS1	0.445	0.302	1.471	0.141	1.56	0.471	1.187	0.235	0.863	2.821
NUCB2	-0.028	0.296	-0.096	0.924	0.972	0.287	-0.097	0.923	0.545	1.735
NUCB1	-0.921	0.321	-2.872	0.004	0.398	0.128	-4.715	0	0.212	0.746
NUBPL	-0.028	0.296	-0.093	0.926	0.973	0.288	-0.094	0.925	0.545	1.736
NUBP2	0.89	0.315	2.827	0.005	2.435	0.767	1.872	0.061	1.314	4.514
NUBP1	-0.006	0.297	-0.022	0.983	0.994	0.295	-0.022	0.983	0.555	1.778
NUAK1	-0.273	0.298	-0.916	0.36	0.761	0.227	-1.053	0.292	0.425	1.364
NTSR2	0.273	0.298	0.916	0.36	1.314	0.391	0.802	0.423	0.733	2.355
NTSR1	0.264	0.297	0.891	0.373	1.303	0.387	0.783	0.434	0.728	2.331
NTS	0.016	0.295	0.056	0.956	1.017	0.3	0.055	0.956	0.57	1.814
NTRK3	0.046	0.296	0.155	0.877	1.047	0.31	0.152	0.88	0.586	1.87
NTRK2	-0.242	0.298	-0.812	0.417	0.785	0.234	-0.919	0.358	0.438	1.407
NTRK1	-0.45	0.3	-1.503	0.133	0.637	0.191	-1.898	0.058	0.354	1.147
NTNG1	0.089	0.295	0.301	0.763	1.093	0.323	0.288	0.773	0.613	1.95

NTN3	0.441	0.3	1.472	0.141	1.555	0.466	1.19	0.234	0.864	2.798
NTN1	-0.177	0.296	-0.596	0.551	0.838	0.248	-0.651	0.515	0.469	1.498
NTM	0	0.296	-0.001	0.999	1	0.296	-0.001	0.999	0.56	1.785
NTHL1	0.605	0.304	1.993	0.046	1.831	0.556	1.496	0.135	1.01	3.32
NTF3	-0.965	0.328	-2.945	0.003	0.381	0.125	-4.959	0	0.2	0.724
NTAQ1	0.035	0.295	0.118	0.906	1.035	0.305	0.116	0.908	0.581	1.846
NTAN1	-0.008	0.297	-0.026	0.979	0.992	0.294	-0.026	0.979	0.555	1.775
NT5M	0.035	0.296	0.119	0.906	1.036	0.306	0.117	0.907	0.58	1.85
NT5E	-0.156	0.295	-0.53	0.596	0.855	0.253	-0.573	0.566	0.479	1.526
NT5DC3	0.375	0.3	1.25	0.211	1.455	0.436	1.043	0.297	0.808	2.618
NT5DC2	0.537	0.303	1.775	0.076	1.711	0.518	1.373	0.17	0.946	3.097
NT5C2	0.5	0.302	1.655	0.098	1.649	0.499	1.302	0.193	0.912	2.982
NSUN7	-0.148	0.296	-0.499	0.618	0.862	0.256	-0.538	0.591	0.482	1.542
NSUN6	0.27	0.3	0.9	0.368	1.31	0.392	0.789	0.43	0.728	2.356
NSUN5P1	0.263	0.298	0.881	0.378	1.3	0.388	0.775	0.438	0.725	2.333
NSUN5	0.305	0.297	1.027	0.304	1.356	0.403	0.886	0.376	0.758	2.427
NSUN3	0.329	0.297	1.11	0.267	1.39	0.412	0.945	0.344	0.777	2.486
NSMCE4A	0.003	0.299	0.012	0.991	1.003	0.3	0.012	0.991	0.558	1.804
NSMAF	0.44	0.302	1.456	0.145	1.553	0.47	1.178	0.239	0.859	2.809
NSL1	0.204	0.298	0.686	0.492	1.227	0.365	0.621	0.535	0.684	2.2
NSG2	0.154	0.295	0.522	0.601	1.167	0.345	0.484	0.628	0.654	2.082
NSG1	-0.538	0.3	-1.793	0.073	0.584	0.175	-2.375	0.018	0.324	1.051
NSFL1C	0.19	0.296	0.643	0.52	1.21	0.358	0.586	0.558	0.677	2.16
NSF	-0.328	0.298	-1.101	0.271	0.72	0.215	-1.303	0.193	0.401	1.292
NSDHL	0.369	0.297	1.243	0.214	1.447	0.43	1.04	0.299	0.808	2.589
NSD3	-0.15	0.297	-0.505	0.614	0.861	0.255	-0.545	0.586	0.481	1.54
NSD2	0.445	0.298	1.491	0.136	1.56	0.465	1.203	0.229	0.869	2.799
NSD1	0.242	0.296	0.817	0.414	1.274	0.378	0.726	0.468	0.713	2.277
NSA2	-0.37	0.303	-1.222	0.222	0.691	0.209	-1.479	0.139	0.381	1.25
NRXN3	-0.293	0.3	-0.979	0.328	0.746	0.224	-1.137	0.255	0.414	1.342
NRXN2	0.439	0.302	1.45	0.147	1.55	0.469	1.174	0.24	0.857	2.804
NRXN1	-0.228	0.296	-0.771	0.441	0.796	0.236	-0.866	0.386	0.445	1.422
NRTN	0.176	0.297	0.594	0.553	1.193	0.354	0.544	0.586	0.667	2.133
NRSN2	-0.617	0.303	-2.037	0.042	0.54	0.163	-2.817	0.005	0.298	0.977
NRP2	-0.643	0.306	-2.103	0.035	0.525	0.161	-2.951	0.003	0.288	0.957
NRP1	-0.518	0.302	-1.714	0.087	0.595	0.18	-2.246	0.025	0.329	1.077
NRN1	-0.243	0.296	-0.82	0.412	0.785	0.232	-0.928	0.353	0.44	1.401
NRL	0.934	0.313	2.981	0.003	2.546	0.798	1.937	0.053	1.377	4.706

NRIP3	0.394	0.298	1.321	0.186	1.483	0.443	1.092	0.275	0.826	2.663
NRIP2	-0.325	0.296	-1.098	0.272	0.722	0.214	-1.298	0.194	0.404	1.291
NRIP1	-0.383	0.304	-1.262	0.207	0.682	0.207	-1.538	0.124	0.376	1.236
NRGN	0.2	0.298	0.672	0.502	1.222	0.364	0.609	0.543	0.681	2.19
NRG2	0.279	0.298	0.937	0.349	1.322	0.393	0.818	0.413	0.738	2.368
NRG1	-0.22	0.297	-0.739	0.46	0.803	0.239	-0.826	0.409	0.448	1.438
NRF1	0.167	0.296	0.563	0.573	1.181	0.35	0.519	0.604	0.661	2.111
NREP	-0.251	0.296	-0.848	0.396	0.778	0.23	-0.964	0.335	0.435	1.39
NRDE2	-0.301	0.298	-1.01	0.313	0.74	0.221	-1.178	0.239	0.413	1.327
NRDC	-0.353	0.299	-1.18	0.238	0.703	0.21	-1.415	0.157	0.391	1.263
NRCAM	0.191	0.295	0.647	0.517	1.211	0.358	0.589	0.556	0.679	2.161
NRBP1	0.125	0.296	0.423	0.672	1.133	0.335	0.398	0.691	0.635	2.023
NRBF2	0.55	0.302	1.819	0.069	1.733	0.524	1.399	0.162	0.958	3.135
NRAS	0.462	0.3	1.538	0.124	1.587	0.476	1.232	0.218	0.881	2.858
NRAP	-0.278	0.296	-0.94	0.347	0.757	0.224	-1.084	0.278	0.424	1.353
NR6A1	-0.409	0.3	-1.365	0.172	0.664	0.199	-1.686	0.092	0.369	1.196
NR5A2	0.047	0.295	0.159	0.874	1.048	0.309	0.155	0.877	0.588	1.87
NR5A1	0.085	0.295	0.288	0.773	1.089	0.322	0.276	0.783	0.61	1.943
NR4A3	0.231	0.296	0.781	0.435	1.26	0.373	0.697	0.486	0.705	2.251
NR4A2	-0.035	0.297	-0.117	0.906	0.966	0.287	-0.12	0.905	0.539	1.729
NR4A1	0.581	0.306	1.901	0.057	1.788	0.546	1.442	0.149	0.982	3.254
NR3C2	-0.512	0.3	-1.705	0.088	0.599	0.18	-2.226	0.026	0.333	1.08
NR3C1	-0.238	0.296	-0.804	0.421	0.788	0.233	-0.908	0.364	0.441	1.408
NR2F6	0.899	0.314	2.866	0.004	2.456	0.77	1.891	0.059	1.329	4.541
NR2F2	0.35	0.3	1.168	0.243	1.419	0.425	0.985	0.325	0.789	2.552
NR2E3	-0.032	0.295	-0.109	0.913	0.968	0.286	-0.111	0.912	0.543	1.728
NR2E1	0.244	0.297	0.821	0.412	1.276	0.378	0.729	0.466	0.713	2.282
NR2C2	0.378	0.298	1.268	0.205	1.46	0.436	1.056	0.291	0.814	2.62
NR2C1	-0.459	0.302	-1.518	0.129	0.632	0.191	-1.926	0.054	0.349	1.143
NR1I3	-0.12	0.296	-0.406	0.685	0.887	0.262	-0.431	0.666	0.497	1.584
NR1I2	-0.391	0.302	-1.295	0.195	0.676	0.204	-1.585	0.113	0.374	1.223
NR1H4	0.305	0.298	1.024	0.306	1.356	0.403	0.883	0.377	0.757	2.43
NR1H3	-0.38	0.298	-1.272	0.203	0.684	0.204	-1.547	0.122	0.381	1.228
NR1H2	-0.34	0.3	-1.135	0.257	0.712	0.213	-1.351	0.177	0.395	1.281
NR1D2	-0.23	0.298	-0.773	0.439	0.794	0.237	-0.869	0.385	0.443	1.424
NR0B2	0.331	0.298	1.11	0.267	1.392	0.415	0.945	0.344	0.776	2.496
NR0B1	0.219	0.296	0.74	0.459	1.245	0.369	0.665	0.506	0.697	2.225
NQO2	0.234	0.298	0.786	0.432	1.264	0.376	0.701	0.483	0.705	2.264

NQO1	0.684	0.31	2.208	0.027	1.982	0.614	1.6	0.11	1.08	3.636
NPY6R	0.027	0.296	0.09	0.928	1.027	0.304	0.089	0.929	0.575	1.836
NPY5R	0.214	0.298	0.72	0.472	1.239	0.369	0.648	0.517	0.691	2.22
NPY2R	0.439	0.303	1.45	0.147	1.551	0.47	1.173	0.241	0.857	2.809
NPY1R	0.091	0.296	0.308	0.758	1.095	0.324	0.294	0.769	0.613	1.956
NPY	0.044	0.296	0.15	0.881	1.045	0.309	0.147	0.883	0.586	1.866
NPVF	0.466	0.3	1.556	0.12	1.594	0.478	1.244	0.214	0.886	2.868
NPTXR	0.002	0.295	0.005	0.996	1.002	0.296	0.005	0.996	0.562	1.786
NPTX2	-0.945	0.328	-2.884	0.004	0.389	0.127	-4.798	0	0.205	0.739
NPTX1	-0.354	0.3	-1.181	0.238	0.702	0.21	-1.417	0.157	0.39	1.263
NPTN	0.328	0.298	1.102	0.27	1.388	0.413	0.94	0.347	0.775	2.487
NPRL3	0.045	0.298	0.151	0.88	1.046	0.312	0.147	0.883	0.583	1.876
NPRL2	-0.6	0.31	-1.937	0.053	0.549	0.17	-2.654	0.008	0.299	1.007
NPR3	0.313	0.298	1.049	0.294	1.367	0.408	0.9	0.368	0.762	2.453
NPR2	0.142	0.296	0.481	0.63	1.153	0.341	0.449	0.654	0.646	2.058
NPR1	-0.635	0.306	-2.076	0.038	0.53	0.162	-2.9	0.004	0.291	0.965
NPPC	-0.173	0.296	-0.585	0.559	0.841	0.249	-0.639	0.523	0.471	1.503
NPPB	0.119	0.295	0.404	0.686	1.127	0.333	0.381	0.703	0.632	2.01
NPPA	0.33	0.298	1.107	0.268	1.391	0.415	0.943	0.346	0.775	2.495
NPM3	0.37	0.298	1.24	0.215	1.447	0.431	1.037	0.3	0.807	2.596
NPM1	0.157	0.297	0.528	0.598	1.17	0.347	0.488	0.625	0.654	2.092
NPLOC4	0.268	0.296	0.904	0.366	1.307	0.387	0.793	0.428	0.732	2.336
NPL	0.035	0.295	0.117	0.907	1.035	0.305	0.115	0.908	0.581	1.846
NPIPB15	0.403	0.299	1.351	0.177	1.497	0.447	1.112	0.266	0.834	2.687
NPIPA1	-0.121	0.296	-0.409	0.683	0.886	0.262	-0.434	0.664	0.496	1.582
NPHS2	0.051	0.295	0.173	0.862	1.052	0.311	0.169	0.866	0.59	1.877
NPHS1	0.516	0.302	1.705	0.088	1.675	0.507	1.332	0.183	0.926	3.03
NPHP4	0.512	0.302	1.694	0.09	1.668	0.504	1.325	0.185	0.923	3.017
NPHP1	-0.006	0.295	-0.019	0.985	0.995	0.294	-0.019	0.985	0.558	1.774
NPFFR1	0.284	0.299	0.952	0.341	1.329	0.397	0.828	0.407	0.74	2.385
NPFF	-0.288	0.297	-0.967	0.333	0.75	0.223	-1.121	0.262	0.419	1.343
NPEPPSP1	-0.331	0.299	-1.108	0.268	0.718	0.215	-1.313	0.189	0.4	1.29
NPEPPS	0.558	0.303	1.843	0.065	1.747	0.529	1.413	0.158	0.965	3.161
NPEPL1	0.275	0.298	0.925	0.355	1.317	0.392	0.808	0.419	0.735	2.361
NPDC1	-0.254	0.298	-0.852	0.394	0.776	0.231	-0.97	0.332	0.433	1.391
NPC2	-0.104	0.295	-0.351	0.725	0.901	0.266	-0.37	0.711	0.505	1.608
NPC1L1	-0.301	0.298	-1.011	0.312	0.74	0.22	-1.18	0.238	0.413	1.326
NPC1	0.017	0.295	0.057	0.955	1.017	0.3	0.056	0.955	0.57	1.813

NPBWR2	0.474	0.302	1.568	0.117	1.607	0.486	1.249	0.212	0.888	2.908
NPAT	-0.48	0.3	-1.601	0.109	0.619	0.186	-2.054	0.04	0.344	1.114
NPAS3	-0.235	0.296	-0.794	0.427	0.791	0.234	-0.895	0.371	0.442	1.413
NPAS2	-0.357	0.298	-1.199	0.23	0.7	0.208	-1.441	0.15	0.391	1.254
NPAS1	-0.036	0.296	-0.121	0.903	0.965	0.285	-0.123	0.902	0.54	1.723
NPAP1	0.132	0.3	0.442	0.659	1.142	0.342	0.414	0.679	0.634	2.054
NOX5	-0.225	0.298	-0.755	0.45	0.799	0.238	-0.846	0.398	0.446	1.431
NOX4	-0.22	0.296	-0.744	0.457	0.802	0.238	-0.833	0.405	0.449	1.433
NOX3	-0.075	0.297	-0.254	0.8	0.927	0.276	-0.263	0.792	0.518	1.661
NOX1	0.213	0.298	0.716	0.474	1.238	0.369	0.645	0.519	0.69	2.219
NOVA2	0.253	0.296	0.855	0.393	1.288	0.382	0.755	0.45	0.721	2.302
NOVA1	-0.491	0.302	-1.623	0.105	0.612	0.185	-2.095	0.036	0.339	1.107
NOTCH4	0.341	0.298	1.145	0.252	1.406	0.419	0.97	0.332	0.784	2.521
NOTCH3	-0.057	0.296	-0.193	0.847	0.944	0.28	-0.199	0.843	0.528	1.688
NOTCH2NLA	-0.318	0.298	-1.069	0.285	0.727	0.217	-1.258	0.208	0.406	1.304
NOTCH2	-0.197	0.297	-0.663	0.508	0.821	0.244	-0.732	0.464	0.459	1.47
NOTCH1	0.347	0.303	1.143	0.253	1.415	0.429	0.966	0.334	0.781	2.563
NOSIP	0.345	0.298	1.158	0.247	1.412	0.421	0.979	0.328	0.787	2.532
NOS3	-0.621	0.303	-2.049	0.04	0.537	0.163	-2.841	0.004	0.296	0.973
NOS2	0.037	0.296	0.126	0.9	1.038	0.307	0.124	0.902	0.581	1.854
NOS1AP	-0.627	0.31	-2.025	0.043	0.534	0.165	-2.817	0.005	0.291	0.98
NOS1	-0.264	0.298	-0.888	0.374	0.768	0.229	-1.017	0.309	0.428	1.376
NOP2	0.59	0.306	1.929	0.054	1.803	0.551	1.458	0.145	0.991	3.283
NOP16	0.189	0.296	0.639	0.523	1.208	0.358	0.582	0.561	0.676	2.159
NOP14-AS1	0.023	0.296	0.077	0.939	1.023	0.303	0.076	0.94	0.573	1.827
NOP14	0.274	0.298	0.92	0.358	1.315	0.392	0.805	0.421	0.734	2.357
NOP10	0.155	0.296	0.525	0.6	1.168	0.346	0.486	0.627	0.654	2.086
NONO	0.333	0.298	1.12	0.263	1.396	0.415	0.952	0.341	0.779	2.501
NOMO2	0.411	0.298	1.379	0.168	1.509	0.45	1.13	0.258	0.841	2.707
NOLC1	-0.051	0.295	-0.174	0.862	0.95	0.281	-0.178	0.859	0.533	1.695
NOL9	-0.306	0.298	-1.028	0.304	0.736	0.219	-1.203	0.229	0.411	1.32
NOL8	-0.289	0.298	-0.97	0.332	0.749	0.223	-1.125	0.261	0.418	1.343
NOL7	0.877	0.311	2.822	0.005	2.403	0.747	1.879	0.06	1.307	4.418
NOL6	0.288	0.298	0.969	0.333	1.334	0.397	0.842	0.4	0.745	2.391
NOL4	0.649	0.306	2.123	0.034	1.914	0.585	1.562	0.118	1.051	3.484
NOL3	0.184	0.297	0.619	0.536	1.202	0.356	0.566	0.572	0.672	2.149
NOL12	0.155	0.296	0.524	0.601	1.168	0.346	0.485	0.628	0.654	2.086
NOL11	0.471	0.302	1.557	0.119	1.601	0.484	1.242	0.214	0.885	2.895

NOL10	-0.331	0.298	-1.11	0.267	0.718	0.214	-1.315	0.188	0.401	1.288
NODAL	0.198	0.299	0.66	0.509	1.219	0.365	0.599	0.549	0.678	2.192
NOD2	0.219	0.296	0.74	0.46	1.245	0.368	0.664	0.507	0.697	2.223
NOD1	-0.037	0.295	-0.125	0.9	0.964	0.284	-0.128	0.898	0.54	1.719
NOCT	0.308	0.298	1.034	0.301	1.36	0.405	0.89	0.374	0.759	2.437
NOC4L	0.337	0.298	1.132	0.258	1.401	0.417	0.961	0.337	0.782	2.511
NOC3L	0.14	0.297	0.471	0.638	1.15	0.342	0.439	0.661	0.642	2.06
NOC2L	0.377	0.3	1.254	0.21	1.457	0.438	1.045	0.296	0.809	2.625
NNT	0.465	0.299	1.553	0.12	1.592	0.476	1.242	0.214	0.885	2.861
NNMT	-0.461	0.3	-1.536	0.125	0.631	0.189	-1.951	0.051	0.35	1.136
NNAT	0.388	0.299	1.296	0.195	1.473	0.441	1.074	0.283	0.82	2.648
NMUR1	0.218	0.297	0.735	0.462	1.244	0.369	0.66	0.509	0.695	2.226
NMU	0.608	0.303	2.008	0.045	1.837	0.556	1.505	0.132	1.015	3.324
NMT2	0.373	0.298	1.253	0.21	1.452	0.432	1.046	0.296	0.81	2.602
NMT1	0.147	0.295	0.498	0.618	1.158	0.342	0.463	0.643	0.649	2.066
NMRK2	-0.116	0.295	-0.393	0.694	0.89	0.263	-0.417	0.677	0.499	1.588
NMRK1	-0.218	0.296	-0.735	0.462	0.804	0.238	-0.822	0.411	0.45	1.438
NMNAT2	-0.067	0.295	-0.226	0.821	0.935	0.276	-0.234	0.815	0.524	1.669
NMI	0.169	0.296	0.57	0.569	1.184	0.35	0.525	0.6	0.663	2.114
NME8	-0.526	0.302	-1.74	0.082	0.591	0.179	-2.289	0.022	0.327	1.069
NME7	0.086	0.298	0.289	0.772	1.09	0.324	0.277	0.782	0.608	1.953
NME6	0.845	0.315	2.684	0.007	2.327	0.732	1.812	0.07	1.256	4.313
NME5	-0.245	0.298	-0.822	0.411	0.783	0.233	-0.931	0.352	0.437	1.404
NME4	-0.214	0.298	-0.717	0.473	0.808	0.241	-0.8	0.424	0.451	1.448
NME3	-0.218	0.298	-0.732	0.464	0.804	0.24	-0.817	0.414	0.448	1.442
NME1	0.632	0.31	2.039	0.041	1.881	0.583	1.511	0.131	1.025	3.454
NMD3	0.101	0.295	0.341	0.733	1.106	0.327	0.325	0.746	0.62	1.973
NMBR	-0.333	0.3	-1.112	0.266	0.717	0.215	-1.32	0.187	0.398	1.289
NMB	0.62	0.303	2.045	0.041	1.859	0.564	1.524	0.128	1.026	3.369
NLRX1	-0.269	0.296	-0.907	0.365	0.764	0.227	-1.04	0.298	0.428	1.366
NLRP3	0.237	0.296	0.799	0.424	1.267	0.375	0.712	0.477	0.709	2.264
NLRP2	0.267	0.298	0.898	0.369	1.306	0.389	0.788	0.43	0.729	2.341
NLRP1	-0.547	0.301	-1.817	0.069	0.579	0.174	-2.419	0.016	0.321	1.044
NLK	0.07	0.295	0.238	0.812	1.073	0.317	0.23	0.818	0.602	1.914
NLGN4Y	0.012	0.296	0.042	0.967	1.012	0.299	0.041	0.967	0.567	1.808
NLGN4X	-0.12	0.295	-0.406	0.685	0.887	0.262	-0.431	0.666	0.497	1.582
NLGN3	0.223	0.296	0.752	0.452	1.249	0.37	0.674	0.5	0.699	2.233
NLGN1	0.196	0.296	0.66	0.509	1.216	0.36	0.6	0.549	0.68	2.173

NLE1	-0.128	0.297	-0.43	0.667	0.88	0.261	-0.459	0.646	0.492	1.574
NKX6-1	0.201	0.297	0.678	0.498	1.223	0.363	0.614	0.539	0.683	2.188
NKX3-2	-0.027	0.295	-0.09	0.928	0.974	0.288	-0.092	0.927	0.546	1.737
NKX3-1	-0.164	0.298	-0.551	0.582	0.849	0.253	-0.599	0.549	0.473	1.522
NKX2-8	0.192	0.297	0.647	0.518	1.211	0.359	0.589	0.556	0.677	2.166
NKX2-5	0.202	0.296	0.681	0.496	1.224	0.362	0.617	0.537	0.685	2.186
NKX2-2	0.44	0.302	1.455	0.146	1.553	0.47	1.177	0.239	0.858	2.809
NKX2-1	-0.028	0.295	-0.095	0.924	0.972	0.287	-0.097	0.923	0.545	1.734
NKTR	0.329	0.3	1.1	0.272	1.39	0.417	0.937	0.349	0.773	2.501
NKRF	0.203	0.298	0.682	0.495	1.225	0.365	0.617	0.537	0.683	2.197
NKIRAS2	0.592	0.306	1.935	0.053	1.807	0.552	1.461	0.144	0.992	3.289
NKG7	-0.644	0.307	-2.101	0.036	0.525	0.161	-2.949	0.003	0.288	0.958
NKAPD1	0.019	0.298	0.063	0.95	1.019	0.303	0.062	0.95	0.568	1.826
NKAIN1	0.178	0.297	0.599	0.549	1.195	0.355	0.549	0.583	0.667	2.14
NIT2	0.135	0.295	0.458	0.647	1.145	0.338	0.428	0.669	0.642	2.042
NIT1	0.22	0.296	0.743	0.457	1.246	0.369	0.667	0.505	0.698	2.225
NISCH	-0.172	0.296	-0.579	0.562	0.842	0.25	-0.632	0.527	0.471	1.505
NIPSNAP3B	0.104	0.295	0.353	0.724	1.11	0.328	0.335	0.737	0.622	1.98
NIPSNAP2	0.386	0.298	1.296	0.195	1.471	0.438	1.075	0.282	0.821	2.636
NIPSNAP1	0.224	0.298	0.752	0.452	1.251	0.372	0.674	0.5	0.698	2.241
NIPBL	0.292	0.3	0.973	0.331	1.339	0.402	0.844	0.399	0.743	2.413
NIPAL3	-0.121	0.297	-0.407	0.684	0.886	0.263	-0.433	0.665	0.495	1.586
NIPAL2	-0.22	0.296	-0.746	0.456	0.802	0.237	-0.835	0.404	0.449	1.432
NIPA2	-0.115	0.295	-0.389	0.698	0.892	0.263	-0.412	0.681	0.5	1.591
NIP7	0.111	0.295	0.377	0.706	1.118	0.33	0.356	0.721	0.627	1.994
NINL	0.699	0.306	2.286	0.022	2.013	0.616	1.645	0.1	1.105	3.666
NINJ2	-0.63	0.306	-2.061	0.039	0.532	0.163	-2.872	0.004	0.292	0.97
NINJ1	-0.141	0.296	-0.475	0.635	0.869	0.257	-0.51	0.61	0.486	1.553
NIN	0.279	0.3	0.931	0.352	1.322	0.396	0.813	0.416	0.735	2.378
NIF3L1	0.11	0.296	0.371	0.71	1.116	0.331	0.352	0.725	0.625	1.995
NID2	-0.544	0.3	-1.811	0.07	0.581	0.174	-2.406	0.016	0.322	1.046
NID1	-0.341	0.297	-1.151	0.25	0.711	0.211	-1.372	0.17	0.397	1.271
NIBAN1	-0.187	0.296	-0.631	0.528	0.829	0.246	-0.694	0.488	0.464	1.482
NHP2	0.215	0.296	0.725	0.468	1.24	0.367	0.653	0.514	0.694	2.216
NHLRC2	-0.557	0.306	-1.822	0.069	0.573	0.175	-2.437	0.015	0.315	1.043
NHLH2	0.029	0.295	0.098	0.922	1.029	0.304	0.096	0.923	0.577	1.835
NHLH1	0.057	0.295	0.194	0.846	1.059	0.313	0.189	0.85	0.594	1.888
NGRN	0.343	0.3	1.143	0.253	1.409	0.422	0.968	0.333	0.783	2.535

NGLY1	-0.593	0.311	-1.909	0.056	0.553	0.172	-2.605	0.009	0.301	1.016
NGFR	0.233	0.297	0.786	0.432	1.262	0.374	0.701	0.483	0.706	2.258
NGF	-0.524	0.303	-1.733	0.083	0.592	0.179	-2.278	0.023	0.327	1.071
NGDN	0.646	0.31	2.087	0.037	1.908	0.591	1.537	0.124	1.04	3.502
NGB	-0.249	0.296	-0.842	0.4	0.779	0.231	-0.956	0.339	0.436	1.393
NFYC	-0.402	0.303	-1.329	0.184	0.669	0.202	-1.636	0.102	0.37	1.211
NFYB	-0.111	0.295	-0.376	0.707	0.895	0.264	-0.398	0.691	0.502	1.596
NFYA	0.363	0.303	1.198	0.231	1.437	0.435	1.005	0.315	0.794	2.602
NFX1	-0.54	0.303	-1.781	0.075	0.583	0.177	-2.361	0.018	0.322	1.056
NFU1	-0.46	0.307	-1.498	0.134	0.631	0.194	-1.903	0.057	0.346	1.152
NFS1	0.208	0.299	0.697	0.486	1.232	0.368	0.629	0.529	0.685	2.214
NFRKB	0.422	0.3	1.407	0.159	1.524	0.457	1.148	0.251	0.847	2.743
NFKBIL1	0.059	0.295	0.201	0.841	1.061	0.313	0.195	0.846	0.595	1.892
NFKBIE	0.566	0.303	1.87	0.061	1.761	0.533	1.428	0.153	0.973	3.186
NFKBIB	0.018	0.296	0.063	0.95	1.019	0.301	0.062	0.951	0.571	1.818
NFKBIA	0.203	0.299	0.678	0.498	1.225	0.366	0.614	0.539	0.682	2.199
NFKB2	-0.469	0.303	-1.548	0.122	0.625	0.19	-1.976	0.048	0.345	1.133
NFKB1	-0.559	0.303	-1.846	0.065	0.572	0.173	-2.473	0.013	0.316	1.035
NFIX	0.858	0.311	2.76	0.006	2.36	0.734	1.852	0.064	1.282	4.342
NFIL3	0.126	0.296	0.425	0.671	1.134	0.336	0.399	0.69	0.634	2.027
NFIC	-0.179	0.299	-0.6	0.549	0.836	0.25	-0.657	0.511	0.466	1.501
NFIB	0.168	0.299	0.562	0.574	1.183	0.353	0.518	0.605	0.659	2.125
NFE2L3	0.042	0.295	0.143	0.886	1.043	0.308	0.14	0.889	0.585	1.861
NFE2L2	-0.165	0.295	-0.558	0.577	0.848	0.251	-0.606	0.544	0.475	1.513
NFE2L1	-0.095	0.298	-0.319	0.749	0.909	0.271	-0.335	0.738	0.507	1.632
NFE2	-0.187	0.296	-0.631	0.528	0.829	0.246	-0.694	0.488	0.464	1.482
NFATC4	-0.144	0.296	-0.486	0.627	0.866	0.256	-0.522	0.601	0.485	1.546
NFATC3	0.033	0.295	0.112	0.911	1.034	0.305	0.11	0.912	0.58	1.843
NFATC2IP	0.336	0.3	1.122	0.262	1.399	0.419	0.953	0.341	0.778	2.518
NFATC1	-0.182	0.296	-0.615	0.538	0.833	0.247	-0.675	0.5	0.467	1.489
NFAT5	0.066	0.296	0.223	0.823	1.068	0.317	0.216	0.829	0.598	1.91
NFASC	0.279	0.298	0.937	0.349	1.321	0.393	0.817	0.414	0.737	2.368
NF2	-0.318	0.298	-1.068	0.285	0.728	0.217	-1.258	0.209	0.406	1.304
NF1	-0.251	0.297	-0.845	0.398	0.778	0.231	-0.96	0.337	0.434	1.393
NEUROG3	0.651	0.306	2.124	0.034	1.917	0.587	1.561	0.119	1.051	3.495
NEUROG2	0.46	0.3	1.534	0.125	1.584	0.475	1.23	0.219	0.88	2.85
NEUROG1	-0.199	0.296	-0.673	0.501	0.819	0.243	-0.745	0.456	0.459	1.464
NEUROD6	0.756	0.306	2.471	0.013	2.129	0.651	1.734	0.083	1.169	3.878

NEUROD4	0.092	0.295	0.311	0.756	1.096	0.324	0.297	0.766	0.614	1.955
NEUROD2	-0.297	0.298	-0.996	0.319	0.743	0.221	-1.159	0.246	0.415	1.333
NEUROD1	0.255	0.297	0.861	0.389	1.291	0.383	0.76	0.447	0.722	2.309
NEURL1	-0.042	0.296	-0.142	0.887	0.959	0.283	-0.145	0.885	0.537	1.711
NEU3	-0.184	0.296	-0.622	0.534	0.832	0.246	-0.683	0.495	0.466	1.486
NEU2	0.266	0.296	0.898	0.369	1.305	0.386	0.788	0.431	0.73	2.331
NEU1	-0.412	0.302	-1.362	0.173	0.662	0.2	-1.686	0.092	0.366	1.198
NETO2	0.267	0.298	0.894	0.371	1.305	0.389	0.785	0.433	0.728	2.341
NET1	0.335	0.298	1.126	0.26	1.398	0.416	0.957	0.339	0.78	2.506
NES	0.23	0.298	0.771	0.441	1.258	0.374	0.689	0.491	0.702	2.254
NEO1	-0.363	0.303	-1.198	0.231	0.696	0.211	-1.444	0.149	0.385	1.259
NENF	0.403	0.3	1.346	0.178	1.497	0.448	1.108	0.268	0.832	2.692
NEMP1	0.439	0.3	1.465	0.143	1.551	0.465	1.186	0.236	0.862	2.79
NEMF	-0.017	0.296	-0.057	0.954	0.983	0.291	-0.058	0.954	0.551	1.755
NELL2	0.224	0.296	0.755	0.45	1.251	0.37	0.677	0.499	0.7	2.235
NELL1	0.352	0.298	1.183	0.237	1.422	0.423	0.997	0.319	0.794	2.549
NELFCD	0.187	0.299	0.624	0.532	1.206	0.361	0.569	0.569	0.67	2.168
NELFB	0.062	0.295	0.21	0.833	1.064	0.314	0.204	0.838	0.597	1.897
NEK9	0.187	0.296	0.632	0.528	1.206	0.357	0.576	0.565	0.675	2.154
NEK7	-0.567	0.307	-1.85	0.064	0.567	0.174	-2.49	0.013	0.311	1.034
NEK4	-0.095	0.296	-0.323	0.747	0.909	0.269	-0.339	0.735	0.509	1.623
NEK3	0.018	0.295	0.062	0.951	1.018	0.301	0.061	0.951	0.571	1.816
NEK2	0.777	0.315	2.467	0.014	2.175	0.685	1.715	0.086	1.173	4.032
NEK11	-0.087	0.295	-0.294	0.769	0.917	0.271	-0.307	0.759	0.514	1.635
NEK1	0.291	0.298	0.978	0.328	1.338	0.399	0.849	0.396	0.746	2.399
NEIL3	0.282	0.298	0.945	0.345	1.326	0.395	0.823	0.41	0.739	2.379
NEFM	0.046	0.295	0.154	0.877	1.047	0.309	0.151	0.88	0.587	1.867
NEFL	-0.703	0.306	-2.298	0.022	0.495	0.152	-3.334	0.001	0.272	0.902
NEFH	-0.252	0.296	-0.851	0.395	0.777	0.23	-0.967	0.333	0.435	1.389
NEDD9	0.144	0.296	0.486	0.627	1.155	0.342	0.453	0.651	0.646	2.064
NEDD8	0.413	0.299	1.384	0.167	1.512	0.452	1.133	0.257	0.842	2.715
NEDD4L	0.142	0.295	0.48	0.631	1.152	0.34	0.448	0.654	0.646	2.055
NEDD4	-0.537	0.303	-1.776	0.076	0.584	0.177	-2.352	0.019	0.323	1.057
NECTIN3	-0.216	0.296	-0.73	0.465	0.806	0.239	-0.815	0.415	0.451	1.439
NECTIN2	0.105	0.296	0.354	0.723	1.11	0.328	0.336	0.737	0.622	1.982
NECTIN1	-0.068	0.295	-0.23	0.818	0.934	0.276	-0.238	0.812	0.524	1.667
NECAP2	-0.617	0.306	-2.018	0.044	0.54	0.165	-2.792	0.005	0.296	0.982
NECAP1	-0.099	0.296	-0.335	0.738	0.906	0.268	-0.352	0.725	0.507	1.618

NECAB3	-0.005	0.295	-0.016	0.988	0.995	0.294	-0.016	0.988	0.558	1.775
NECAB2	0.094	0.295	0.319	0.75	1.099	0.325	0.304	0.761	0.616	1.96
NEBL	0.415	0.298	1.393	0.164	1.515	0.451	1.14	0.254	0.845	2.716
NEB	0.071	0.295	0.24	0.81	1.074	0.317	0.232	0.817	0.602	1.915
NDUFV2	0.017	0.298	0.057	0.954	1.017	0.303	0.057	0.955	0.567	1.825
NDUFV1	0.317	0.298	1.066	0.287	1.374	0.409	0.913	0.361	0.766	2.463
NDUFS7	0.09	0.295	0.306	0.759	1.095	0.323	0.293	0.77	0.614	1.952
NDUFS6	1.018	0.321	3.174	0.002	2.768	0.888	1.991	0.046	1.476	5.192
NDUFS4	0.165	0.299	0.553	0.581	1.18	0.353	0.509	0.611	0.656	2.12
NDUFS3	0.355	0.297	1.198	0.231	1.427	0.423	1.008	0.313	0.798	2.553
NDUFS2	0.052	0.297	0.177	0.86	1.054	0.313	0.172	0.863	0.589	1.885
NDUFS1	0.503	0.3	1.678	0.093	1.654	0.497	1.318	0.187	0.919	2.979
NDUFC1	0.523	0.301	1.738	0.082	1.688	0.508	1.353	0.176	0.935	3.044
NDUFB8	-0.093	0.299	-0.311	0.756	0.911	0.272	-0.325	0.745	0.508	1.637
NDUFB7	0.757	0.308	2.458	0.014	2.132	0.656	1.724	0.085	1.166	3.898
NDUFB6	0.651	0.303	2.149	0.032	1.918	0.581	1.579	0.114	1.059	3.473
NDUFB5	0.886	0.315	2.809	0.005	2.425	0.765	1.864	0.062	1.307	4.499
NDUFB4	0.488	0.3	1.629	0.103	1.629	0.488	1.289	0.197	0.906	2.932
NDUFB3	0.003	0.296	0.011	0.991	1.003	0.297	0.011	0.991	0.562	1.791
NDUFB2-AS1	0.499	0.302	1.651	0.099	1.647	0.498	1.3	0.194	0.911	2.98
NDUFB2	0.491	0.299	1.643	0.1	1.634	0.488	1.299	0.194	0.91	2.935
NDUFB11	0.004	0.295	0.013	0.99	1.004	0.296	0.013	0.99	0.563	1.79
NDUFB1	0.26	0.296	0.879	0.379	1.298	0.384	0.774	0.439	0.726	2.319
NDUFAF7	0.095	0.295	0.32	0.749	1.099	0.325	0.306	0.76	0.616	1.961
NDUFAF5	0.185	0.296	0.626	0.531	1.204	0.356	0.571	0.568	0.674	2.15
NDUFAF4	0.312	0.297	1.047	0.295	1.366	0.406	0.9	0.368	0.762	2.446
NDUFAF3	-0.009	0.297	-0.031	0.975	0.991	0.294	-0.031	0.975	0.553	1.774
NDUFAF1	-0.201	0.305	-0.661	0.509	0.818	0.249	-0.732	0.464	0.45	1.485
NDUFAB1	0.595	0.306	1.946	0.052	1.812	0.554	1.467	0.142	0.996	3.299
NDUFA9	0.428	0.298	1.434	0.151	1.534	0.457	1.167	0.243	0.855	2.751
NDUFA8	0.775	0.31	2.498	0.012	2.17	0.673	1.739	0.082	1.182	3.984
NDUFA7	-0.057	0.297	-0.192	0.848	0.945	0.281	-0.197	0.844	0.528	1.691
NDUFA6	0.36	0.298	1.209	0.227	1.433	0.427	1.015	0.31	0.8	2.57
NDUFA5	0.007	0.296	0.024	0.981	1.007	0.298	0.024	0.981	0.564	1.8
NDUFA4L2	-0.073	0.296	-0.248	0.804	0.929	0.275	-0.257	0.797	0.521	1.659
NDUFA4	1.227	0.336	3.646	0	3.41	1.147	2.101	0.036	1.764	6.593
NDUFA3	0.149	0.296	0.503	0.615	1.16	0.343	0.468	0.64	0.65	2.072
NDUFA2	0.266	0.296	0.896	0.37	1.304	0.386	0.787	0.431	0.73	2.331

NDUFA13	0.717	0.307	2.332	0.02	2.048	0.63	1.665	0.096	1.121	3.743
NDUFA10	-0.45	0.3	-1.5	0.134	0.638	0.191	-1.894	0.058	0.355	1.148
NDUFA1	0.179	0.297	0.602	0.547	1.195	0.354	0.551	0.581	0.669	2.138
NDST4	0.152	0.296	0.515	0.607	1.165	0.345	0.477	0.633	0.652	2.081
NDST3	-0.095	0.295	-0.322	0.748	0.909	0.269	-0.337	0.736	0.51	1.622
NDST2	0.255	0.297	0.859	0.39	1.29	0.383	0.758	0.448	0.721	2.308
NDST1	0.143	0.298	0.481	0.63	1.154	0.344	0.449	0.654	0.644	2.069
NDRG4	-0.137	0.296	-0.461	0.645	0.872	0.258	-0.494	0.621	0.488	1.559
NDRG3	0.054	0.295	0.183	0.854	1.056	0.312	0.179	0.858	0.592	1.883
NDRG2	0.105	0.296	0.353	0.724	1.11	0.329	0.336	0.737	0.621	1.986
NDRG1	0.047	0.296	0.158	0.875	1.048	0.31	0.154	0.878	0.587	1.871
NDP	-0.792	0.315	-2.514	0.012	0.453	0.143	-3.833	0	0.244	0.84
NDOR1	-0.477	0.302	-1.579	0.114	0.621	0.188	-2.024	0.043	0.343	1.122
NDNF	-0.612	0.308	-1.991	0.046	0.542	0.167	-2.747	0.006	0.297	0.99
NDN	-0.529	0.302	-1.75	0.08	0.589	0.178	-2.306	0.021	0.326	1.066
NDFIP1	-0.505	0.302	-1.671	0.095	0.603	0.182	-2.174	0.03	0.334	1.091
NDEL1	0.641	0.303	2.114	0.035	1.899	0.576	1.56	0.119	1.048	3.442
NDE1	0.321	0.298	1.077	0.282	1.378	0.411	0.921	0.357	0.768	2.472
NDC80	0.878	0.315	2.791	0.005	2.407	0.757	1.858	0.063	1.299	4.46
NDC1	0.366	0.298	1.229	0.219	1.442	0.429	1.03	0.303	0.805	2.584
NCSTN	-0.662	0.317	-2.091	0.036	0.516	0.163	-2.965	0.003	0.277	0.959
NCS1	0.248	0.298	0.833	0.405	1.281	0.381	0.738	0.461	0.715	2.296
NCR3	-0.383	0.3	-1.279	0.201	0.682	0.204	-1.559	0.119	0.379	1.226
NCR2	0.176	0.296	0.594	0.552	1.192	0.353	0.545	0.586	0.667	2.13
NCR1	-0.047	0.295	-0.158	0.874	0.954	0.282	-0.162	0.871	0.535	1.703
NCOR2	-0.465	0.3	-1.55	0.121	0.628	0.188	-1.973	0.049	0.349	1.131
NCOR1	-0.058	0.296	-0.197	0.844	0.944	0.279	-0.202	0.84	0.529	1.684
NCOA6	-0.136	0.296	-0.459	0.646	0.873	0.258	-0.492	0.623	0.489	1.558
NCOA4	-0.425	0.3	-1.418	0.156	0.654	0.196	-1.768	0.077	0.363	1.176
NCOA3	-0.004	0.296	-0.015	0.988	0.996	0.295	-0.015	0.988	0.557	1.779
NCOA2	0.071	0.295	0.239	0.811	1.073	0.317	0.23	0.818	0.601	1.914
NCOA1	-0.49	0.301	-1.629	0.103	0.613	0.184	-2.102	0.036	0.34	1.105
NCLN	0.13	0.297	0.44	0.66	1.139	0.338	0.412	0.68	0.637	2.038
NCL	0.156	0.3	0.518	0.604	1.168	0.351	0.48	0.631	0.649	2.104
NCKIPSD	-0.135	0.295	-0.456	0.648	0.874	0.258	-0.488	0.626	0.49	1.559
NCKAP1L	-0.179	0.296	-0.606	0.545	0.836	0.248	-0.663	0.507	0.468	1.494
NCKAP1	0.286	0.297	0.965	0.335	1.332	0.395	0.839	0.401	0.744	2.383
NCK2	0.435	0.307	1.415	0.157	1.545	0.475	1.148	0.251	0.846	2.822

NCK1	-0.003	0.295	-0.011	0.992	0.997	0.294	-0.011	0.992	0.559	1.778
NCF4	0.049	0.295	0.167	0.867	1.051	0.31	0.163	0.87	0.589	1.873
NCF2	0.361	0.299	1.21	0.226	1.435	0.429	1.015	0.31	0.799	2.578
NCDN	-0.133	0.298	-0.445	0.656	0.876	0.261	-0.476	0.634	0.488	1.571
NCBP3	0.409	0.304	1.348	0.178	1.506	0.457	1.106	0.269	0.831	2.73
NCBP2	0.625	0.31	2.018	0.044	1.869	0.579	1.5	0.134	1.018	3.43
NCBP1	0.479	0.3	1.595	0.111	1.614	0.485	1.267	0.205	0.896	2.907
NCAPH2	0.472	0.302	1.561	0.118	1.603	0.485	1.244	0.213	0.886	2.9
NCAPH	0.753	0.31	2.432	0.015	2.124	0.658	1.708	0.088	1.157	3.897
NCAPG2	0.432	0.298	1.45	0.147	1.54	0.459	1.177	0.239	0.859	2.76
NCAPG	0.697	0.306	2.28	0.023	2.008	0.614	1.642	0.101	1.103	3.655
NCAPD3	0.443	0.3	1.479	0.139	1.558	0.467	1.195	0.232	0.866	2.802
NCAPD2	0.397	0.298	1.335	0.182	1.488	0.443	1.102	0.27	0.83	2.667
NCAN	0.166	0.301	0.553	0.58	1.181	0.355	0.509	0.61	0.655	2.129
NCAM2	-0.002	0.296	-0.007	0.995	0.998	0.295	-0.007	0.995	0.559	1.781
NCAM1	0.077	0.298	0.259	0.796	1.08	0.322	0.249	0.803	0.603	1.937
NCALD	0.058	0.296	0.195	0.845	1.059	0.313	0.19	0.849	0.594	1.891
NBR2	0.522	0.302	1.73	0.084	1.685	0.508	1.348	0.178	0.933	3.044
NBR1	-0.378	0.3	-1.262	0.207	0.685	0.205	-1.534	0.125	0.381	1.233
NBPF1	-0.869	0.315	-2.759	0.006	0.419	0.132	-4.397	0	0.226	0.777
NBN	-0.525	0.303	-1.735	0.083	0.591	0.179	-2.282	0.022	0.327	1.071
NBL1	-0.459	0.303	-1.515	0.13	0.632	0.191	-1.923	0.055	0.349	1.144
NBEAL2	-0.282	0.296	-0.951	0.341	0.754	0.223	-1.099	0.272	0.422	1.348
NBEA	-0.354	0.3	-1.181	0.238	0.702	0.21	-1.417	0.157	0.39	1.263
NBAS	-0.037	0.295	-0.125	0.901	0.964	0.284	-0.127	0.899	0.541	1.719
NAXD	0.243	0.296	0.82	0.412	1.275	0.378	0.728	0.467	0.713	2.28
NAV3	-0.846	0.321	-2.637	0.008	0.429	0.138	-4.145	0	0.229	0.805
NAV2	0.069	0.295	0.234	0.815	1.071	0.316	0.226	0.821	0.601	1.911
NAT9	0.286	0.298	0.96	0.337	1.332	0.397	0.835	0.404	0.742	2.389
NAT8B	-0.326	0.3	-1.087	0.277	0.722	0.216	-1.285	0.199	0.401	1.299
NAT2	-0.649	0.31	-2.095	0.036	0.523	0.162	-2.949	0.003	0.285	0.959
NAT10	0.171	0.296	0.576	0.564	1.186	0.351	0.53	0.596	0.664	2.12
NAT1	-0.478	0.302	-1.58	0.114	0.62	0.188	-2.025	0.043	0.343	1.122
NASP	0.315	0.297	1.061	0.289	1.37	0.406	0.91	0.363	0.766	2.45
NARS2	0.058	0.296	0.196	0.845	1.06	0.314	0.19	0.849	0.593	1.892
NARS1	0.333	0.298	1.116	0.264	1.395	0.416	0.95	0.342	0.778	2.501
NARF	0.6	0.302	1.984	0.047	1.822	0.551	1.492	0.136	1.007	3.294
NAPG	0.248	0.296	0.838	0.402	1.282	0.38	0.742	0.458	0.717	2.29

NAPA	-0.634	0.31	-2.045	0.041	0.531	0.164	-2.855	0.004	0.289	0.974
NAP1L4	0.053	0.295	0.18	0.857	1.055	0.311	0.176	0.861	0.592	1.88
NAP1L3	-0.413	0.3	-1.379	0.168	0.661	0.198	-1.708	0.088	0.368	1.19
NAP1L2	0.043	0.295	0.144	0.885	1.043	0.308	0.141	0.888	0.585	1.861
NAP1L1	-0.666	0.307	-2.165	0.03	0.514	0.158	-3.075	0.002	0.281	0.939
NANS	0.407	0.3	1.358	0.175	1.503	0.451	1.115	0.265	0.835	2.705
NANOG	-0.06	0.296	-0.203	0.839	0.942	0.278	-0.21	0.834	0.528	1.681
NAMPT	-0.181	0.296	-0.612	0.541	0.834	0.247	-0.671	0.502	0.467	1.491
NAIP	-0.424	0.302	-1.401	0.161	0.655	0.198	-1.744	0.081	0.362	1.184
NAGPA	-0.095	0.295	-0.321	0.748	0.909	0.268	-0.337	0.736	0.51	1.622
NAGLU	0.013	0.295	0.042	0.966	1.013	0.299	0.042	0.966	0.568	1.805
NAGK	0.147	0.295	0.498	0.619	1.158	0.342	0.463	0.644	0.649	2.067
NAGA	-0.454	0.303	-1.502	0.133	0.635	0.192	-1.901	0.057	0.351	1.149
NAE1	0.263	0.296	0.887	0.375	1.301	0.385	0.78	0.435	0.728	2.324
NADSYN1	0.016	0.295	0.055	0.956	1.016	0.3	0.055	0.956	0.57	1.814
NADK	-0.686	0.31	-2.212	0.027	0.504	0.156	-3.178	0.001	0.274	0.925
NACC2	-0.076	0.295	-0.258	0.796	0.927	0.273	-0.268	0.788	0.52	1.652
NACAD	0.095	0.295	0.323	0.747	1.1	0.325	0.308	0.758	0.617	1.962
NACA4P	0.321	0.305	1.051	0.293	1.378	0.42	0.9	0.368	0.758	2.505
NACA2	-0.12	0.296	-0.407	0.684	0.887	0.262	-0.432	0.666	0.497	1.582
NACA	-0.466	0.306	-1.522	0.128	0.627	0.192	-1.939	0.052	0.344	1.144
NABP2	0.154	0.296	0.518	0.604	1.166	0.346	0.48	0.631	0.652	2.084
NABP1	-0.571	0.303	-1.888	0.059	0.565	0.171	-2.547	0.011	0.312	1.022
NAB2	0.126	0.295	0.425	0.671	1.134	0.335	0.4	0.69	0.636	2.023
NAB1	0.132	0.296	0.447	0.655	1.142	0.338	0.419	0.675	0.639	2.04
NAALADL1	-0.692	0.306	-2.259	0.024	0.5	0.153	-3.257	0.001	0.275	0.913
NAALAD2	-0.183	0.295	-0.619	0.536	0.833	0.246	-0.68	0.497	0.467	1.486
NAAA	-0.064	0.296	-0.217	0.828	0.938	0.277	-0.225	0.822	0.525	1.674
NAA80	-0.204	0.296	-0.689	0.491	0.815	0.241	-0.765	0.445	0.456	1.457
NAA60	-0.063	0.295	-0.212	0.832	0.939	0.277	-0.219	0.827	0.527	1.675
NAA50	0.245	0.3	0.818	0.413	1.278	0.383	0.725	0.468	0.71	2.298
NAA40	0.366	0.303	1.209	0.227	1.442	0.437	1.012	0.311	0.797	2.611
NAA35	0.086	0.297	0.29	0.772	1.09	0.324	0.278	0.781	0.609	1.952
NAA16	-0.271	0.296	-0.914	0.36	0.763	0.226	-1.05	0.294	0.427	1.363
NAA15	0.982	0.321	3.064	0.002	2.671	0.856	1.951	0.051	1.425	5.007
NAA11	0.443	0.303	1.463	0.143	1.557	0.471	1.182	0.237	0.86	2.818
NAA10	0.326	0.296	1.101	0.271	1.386	0.411	0.94	0.347	0.775	2.478
N6AMT1	-0.369	0.303	-1.217	0.224	0.692	0.21	-1.471	0.141	0.382	1.253

N4BP3	0.58	0.302	1.919	0.055	1.787	0.54	1.456	0.145	0.988	3.232
N4BP2L2-IT2	0.091	0.295	0.307	0.759	1.095	0.323	0.294	0.769	0.614	1.953
N4BP2L2	-0.089	0.295	-0.3	0.764	0.915	0.27	-0.314	0.753	0.513	1.632
N4BP2L1	0.222	0.298	0.746	0.456	1.248	0.371	0.669	0.504	0.697	2.237
N4BP1	-0.233	0.296	-0.788	0.431	0.792	0.235	-0.888	0.375	0.443	1.415
MZT2B	0.157	0.297	0.528	0.597	1.17	0.348	0.489	0.625	0.654	2.094
MZF1	-0.117	0.297	-0.395	0.693	0.889	0.264	-0.419	0.675	0.497	1.59
MZB1	-0.336	0.298	-1.127	0.26	0.715	0.213	-1.339	0.181	0.398	1.282
MYT1L	0.278	0.298	0.936	0.349	1.321	0.393	0.817	0.414	0.737	2.367
MYT1	0.305	0.298	1.026	0.305	1.357	0.404	0.884	0.377	0.757	2.432
MYRIP	-0.225	0.298	-0.755	0.45	0.799	0.238	-0.847	0.397	0.446	1.431
MYRF	0.518	0.302	1.713	0.087	1.678	0.507	1.337	0.181	0.928	3.035
MYOZ3	-0.478	0.3	-1.591	0.112	0.62	0.186	-2.04	0.041	0.344	1.117
MYOZ2	0.204	0.296	0.688	0.492	1.226	0.363	0.622	0.534	0.686	2.191
MYOZ1	0.287	0.298	0.963	0.336	1.332	0.397	0.837	0.403	0.743	2.388
MYOT	0.45	0.298	1.51	0.131	1.569	0.468	1.216	0.224	0.874	2.813
MYOM2	-0.245	0.298	-0.823	0.411	0.783	0.233	-0.933	0.351	0.437	1.403
MYOM1	-0.52	0.303	-1.719	0.086	0.594	0.18	-2.255	0.024	0.328	1.076
MYOG	-0.014	0.295	-0.049	0.961	0.986	0.291	-0.049	0.961	0.553	1.758
MYOF	-0.754	0.31	-2.431	0.015	0.471	0.146	-3.628	0	0.256	0.864
MYOD1	0.321	0.298	1.076	0.282	1.378	0.411	0.92	0.357	0.768	2.471
MYOC	-0.056	0.295	-0.189	0.85	0.946	0.279	-0.194	0.846	0.53	1.687
MYO9B	-0.163	0.296	-0.553	0.58	0.849	0.251	-0.601	0.548	0.476	1.515
MYO9A	0.318	0.298	1.066	0.286	1.374	0.41	0.913	0.361	0.766	2.465
MYO7B	-0.047	0.296	-0.16	0.873	0.954	0.282	-0.163	0.87	0.534	1.704
MYO7A	0.429	0.3	1.429	0.153	1.536	0.461	1.162	0.245	0.853	2.767
MYO6	-0.348	0.3	-1.159	0.246	0.706	0.212	-1.386	0.166	0.392	1.272
MYO5C	-0.47	0.302	-1.555	0.12	0.625	0.189	-1.985	0.047	0.346	1.13
MYO5A	-0.179	0.296	-0.605	0.545	0.836	0.248	-0.663	0.508	0.468	1.494
MYO3A	0.099	0.295	0.336	0.737	1.104	0.326	0.32	0.749	0.619	1.97
MYO1F	-0.371	0.298	-1.246	0.213	0.69	0.206	-1.509	0.131	0.385	1.237
MYO1E	-0.141	0.295	-0.478	0.633	0.868	0.256	-0.513	0.608	0.487	1.549
MYO1D	-0.299	0.298	-1.003	0.316	0.742	0.221	-1.169	0.242	0.414	1.33
MYO1C	-0.455	0.3	-1.519	0.129	0.634	0.19	-1.924	0.054	0.353	1.141
MYO1B	-0.241	0.297	-0.811	0.418	0.786	0.233	-0.916	0.359	0.439	1.407
MYO1A	-0.001	0.295	-0.004	0.997	0.999	0.295	-0.004	0.997	0.56	1.781
MYO19	0.928	0.321	2.893	0.004	2.531	0.812	1.885	0.059	1.349	4.747
MYO16	0.486	0.303	1.607	0.108	1.626	0.492	1.273	0.203	0.899	2.942

MYO15B	-0.268	0.297	-0.901	0.368	0.765	0.227	-1.032	0.302	0.427	1.37
MYO15A	0.087	0.296	0.295	0.768	1.091	0.323	0.282	0.778	0.611	1.948
MYO10	0.122	0.295	0.414	0.679	1.13	0.334	0.389	0.697	0.633	2.016
MYNN	0.417	0.298	1.399	0.162	1.518	0.453	1.144	0.253	0.846	2.724
MYMX	-0.316	0.3	-1.055	0.291	0.729	0.218	-1.241	0.215	0.405	1.312
MYLPF	0.041	0.297	0.137	0.891	1.041	0.309	0.134	0.893	0.582	1.863
MYLK3	0.057	0.296	0.194	0.847	1.059	0.313	0.188	0.851	0.593	1.89
MYLK	-0.207	0.296	-0.7	0.484	0.813	0.241	-0.778	0.437	0.455	1.452
MYLIP	-0.674	0.305	-2.214	0.027	0.509	0.155	-3.161	0.002	0.28	0.926
MYL9	-0.471	0.3	-1.572	0.116	0.624	0.187	-2.008	0.045	0.347	1.123
MYL7	0.087	0.296	0.295	0.768	1.091	0.323	0.282	0.778	0.611	1.947
MYL6B	0.717	0.31	2.314	0.021	2.049	0.635	1.652	0.099	1.116	3.76
MYL6	0.175	0.296	0.591	0.554	1.191	0.352	0.543	0.587	0.667	2.127
MYL5	0.03	0.295	0.1	0.92	1.03	0.304	0.099	0.921	0.578	1.837
MYL4	-0.018	0.296	-0.06	0.952	0.982	0.291	-0.061	0.951	0.55	1.754
MYL3	0.184	0.295	0.621	0.534	1.201	0.355	0.568	0.57	0.673	2.143
MYL2	-0.204	0.296	-0.69	0.49	0.815	0.241	-0.766	0.444	0.456	1.457
MYL12B	-0.067	0.296	-0.228	0.82	0.935	0.276	-0.236	0.814	0.524	1.669
MYL12A	0.042	0.299	0.14	0.889	1.043	0.312	0.137	0.891	0.58	1.873
MYL10	-0.126	0.296	-0.425	0.671	0.882	0.261	-0.453	0.651	0.493	1.576
MYL1	0.232	0.296	0.785	0.433	1.262	0.374	0.7	0.484	0.706	2.255
MYH9	-0.383	0.298	-1.282	0.2	0.682	0.203	-1.562	0.118	0.38	1.224
MYH8	0.144	0.296	0.485	0.628	1.154	0.342	0.451	0.652	0.646	2.064
MYH7B	0.299	0.3	0.997	0.319	1.348	0.404	0.862	0.389	0.749	2.426
MYH7	0.425	0.302	1.406	0.16	1.53	0.463	1.145	0.252	0.846	2.768
MYH6	0.398	0.303	1.314	0.189	1.488	0.45	1.084	0.278	0.822	2.692
MYH4	-0.395	0.3	-1.317	0.188	0.674	0.202	-1.614	0.106	0.374	1.213
MYH3	0.555	0.302	1.837	0.066	1.743	0.527	1.41	0.159	0.964	3.151
MYH2	0.276	0.298	0.927	0.354	1.318	0.392	0.81	0.418	0.735	2.361
MYH15	0.594	0.306	1.942	0.052	1.811	0.553	1.465	0.143	0.995	3.296
MYH14	-0.355	0.298	-1.19	0.234	0.701	0.209	-1.428	0.153	0.391	1.258
MYH13	0.328	0.298	1.101	0.271	1.388	0.413	0.938	0.348	0.774	2.488
MYH11	-0.301	0.296	-1.016	0.31	0.74	0.219	-1.186	0.236	0.414	1.323
MYH10	-0.401	0.3	-1.337	0.181	0.67	0.201	-1.645	0.1	0.372	1.205
MYH1	0.144	0.296	0.485	0.627	1.154	0.342	0.452	0.651	0.646	2.062
MYG1	-0.545	0.306	-1.784	0.074	0.58	0.177	-2.371	0.018	0.319	1.055
MYF6	-0.119	0.296	-0.402	0.687	0.888	0.263	-0.427	0.669	0.496	1.587
MYF5	1.093	0.325	3.363	0.001	2.984	0.97	2.045	0.041	1.578	5.644

MYEF2	0.941	0.317	2.967	0.003	2.564	0.814	1.922	0.055	1.376	4.776
MYDGF	0.278	0.298	0.933	0.351	1.32	0.394	0.814	0.416	0.736	2.368
MYD88	0.126	0.296	0.424	0.672	1.134	0.336	0.398	0.691	0.634	2.026
MYCT1	-0.379	0.3	-1.265	0.206	0.685	0.205	-1.538	0.124	0.381	1.232
MYCNOS	0.521	0.306	1.706	0.088	1.684	0.515	1.329	0.184	0.925	3.065
MYCN	-0.14	0.295	-0.476	0.634	0.869	0.257	-0.511	0.61	0.487	1.551
MYCL	-0.051	0.295	-0.172	0.863	0.95	0.281	-0.176	0.86	0.533	1.695
MYCBP2	-0.403	0.298	-1.351	0.177	0.669	0.199	-1.664	0.096	0.373	1.199
MYC	0.641	0.306	2.096	0.036	1.898	0.58	1.548	0.122	1.043	3.454
MYBPH	-0.855	0.312	-2.738	0.006	0.425	0.133	-4.327	0	0.231	0.784
MYBPC3	0.02	0.297	0.067	0.946	1.02	0.303	0.067	0.947	0.57	1.826
MYBPC2	0.603	0.303	1.99	0.047	1.828	0.554	1.494	0.135	1.009	3.312
MYBPC1	-0.197	0.296	-0.663	0.507	0.822	0.243	-0.733	0.464	0.46	1.469
MYBL2	0.387	0.298	1.3	0.194	1.472	0.438	1.078	0.281	0.822	2.639
MYBL1	0.511	0.302	1.691	0.091	1.667	0.504	1.324	0.186	0.922	3.016
MYBBP1A	0.225	0.298	0.756	0.45	1.253	0.374	0.677	0.498	0.698	2.248
MYB	-0.434	0.302	-1.437	0.151	0.648	0.196	-1.8	0.072	0.358	1.171
MXRA8	-0.274	0.296	-0.924	0.356	0.761	0.225	-1.063	0.288	0.425	1.359
MXRA7	-0.493	0.302	-1.631	0.103	0.611	0.185	-2.108	0.035	0.338	1.105
MXRA5	-0.45	0.3	-1.504	0.133	0.637	0.191	-1.899	0.058	0.354	1.147
MXI1	-0.129	0.296	-0.435	0.664	0.879	0.26	-0.464	0.643	0.492	1.57
MXD3	0.511	0.3	1.702	0.089	1.666	0.5	1.333	0.183	0.926	3.001
MXD1	-0.233	0.297	-0.786	0.432	0.792	0.235	-0.886	0.376	0.443	1.416
MX2	0.279	0.301	0.928	0.353	1.322	0.398	0.81	0.418	0.733	2.384
MX1	0.384	0.3	1.281	0.2	1.468	0.44	1.063	0.288	0.816	2.643
MVP	-0.447	0.303	-1.476	0.14	0.639	0.194	-1.861	0.063	0.353	1.158
MVK	-0.203	0.301	-0.674	0.5	0.817	0.245	-0.748	0.455	0.453	1.472
MVD	-0.023	0.295	-0.076	0.939	0.978	0.289	-0.077	0.938	0.548	1.744
MVB12B	0.732	0.307	2.385	0.017	2.079	0.638	1.692	0.091	1.139	3.793
MUTYH	0.393	0.302	1.301	0.193	1.482	0.448	1.075	0.282	0.819	2.681
MUSK	0.351	0.3	1.172	0.241	1.421	0.426	0.988	0.323	0.79	2.557
MUS81	0.27	0.297	0.909	0.363	1.31	0.389	0.797	0.425	0.732	2.343
MUL1	-0.035	0.296	-0.118	0.906	0.966	0.286	-0.12	0.905	0.541	1.725
MUC8	0.189	0.298	0.634	0.526	1.208	0.36	0.578	0.563	0.674	2.167
MUC7	0.045	0.295	0.154	0.878	1.046	0.309	0.15	0.881	0.587	1.866
MUC6	0.112	0.295	0.38	0.704	1.119	0.33	0.359	0.72	0.627	1.995
MUC5B	0.154	0.298	0.518	0.605	1.167	0.348	0.48	0.631	0.651	2.092
MUC5AC	0.191	0.298	0.641	0.522	1.21	0.36	0.584	0.559	0.675	2.168

MUC4	0.184	0.296	0.623	0.533	1.202	0.356	0.569	0.57	0.673	2.148
MUC3B	-0.069	0.295	-0.233	0.816	0.933	0.276	-0.241	0.809	0.523	1.666
MUC3A	0.065	0.298	0.216	0.829	1.067	0.318	0.209	0.834	0.594	1.914
MUC2	0.37	0.3	1.236	0.216	1.448	0.434	1.033	0.301	0.805	2.606
MUC16	-0.219	0.296	-0.741	0.459	0.803	0.238	-0.828	0.408	0.45	1.434
MUC13	0.137	0.296	0.461	0.645	1.147	0.34	0.431	0.666	0.641	2.05
MUC1	-0.374	0.3	-1.249	0.212	0.688	0.206	-1.514	0.13	0.382	1.237
MTX2	-0.027	0.295	-0.093	0.926	0.973	0.287	-0.094	0.925	0.546	1.736
MTX1	1.07	0.321	3.33	0.001	2.916	0.937	2.044	0.041	1.553	5.475
MTUS2	0.798	0.31	2.575	0.01	2.221	0.688	1.774	0.076	1.21	4.076
MTUS1	-0.967	0.321	-3.016	0.003	0.38	0.122	-5.084	0	0.203	0.713
MTTP	-0.045	0.295	-0.153	0.878	0.956	0.282	-0.156	0.876	0.536	1.705
MTSS2	0.366	0.298	1.229	0.219	1.442	0.43	1.029	0.304	0.804	2.588
MTSS1	0.361	0.298	1.214	0.225	1.435	0.427	1.019	0.308	0.801	2.572
MTRR	0.065	0.295	0.222	0.825	1.068	0.315	0.215	0.83	0.598	1.905
MTRF1L	0.095	0.297	0.319	0.75	1.099	0.326	0.304	0.761	0.614	1.967
MTRF1	0.204	0.297	0.687	0.492	1.226	0.364	0.621	0.534	0.686	2.192
MTREX	-0.124	0.296	-0.419	0.675	0.883	0.262	-0.446	0.656	0.494	1.579
MTR	0.018	0.295	0.062	0.951	1.018	0.301	0.061	0.951	0.571	1.817
MTPAP	-0.059	0.295	-0.198	0.843	0.943	0.279	-0.204	0.838	0.529	1.683
MTOR	0.072	0.295	0.242	0.809	1.074	0.317	0.234	0.815	0.602	1.917
MTO1	0.365	0.299	1.22	0.222	1.44	0.43	1.023	0.306	0.802	2.586
MTNR1B	-0.128	0.296	-0.434	0.665	0.88	0.26	-0.463	0.644	0.493	1.57
MTNR1A	-0.538	0.303	-1.774	0.076	0.584	0.177	-2.35	0.019	0.322	1.058
MTMR9	-0.53	0.304	-1.741	0.082	0.589	0.179	-2.295	0.022	0.324	1.069
MTMR7	1.164	0.328	3.544	0	3.201	1.051	2.094	0.036	1.682	6.093
MTMR6	0.086	0.295	0.292	0.771	1.09	0.322	0.279	0.78	0.611	1.944
MTMR4	0.268	0.298	0.9	0.368	1.307	0.389	0.79	0.43	0.729	2.343
MTMR3	0.022	0.296	0.075	0.94	1.023	0.303	0.074	0.941	0.573	1.826
MTMR2	0.227	0.297	0.765	0.445	1.255	0.373	0.684	0.494	0.701	2.246
MTMR14	0.671	0.31	2.168	0.03	1.957	0.606	1.579	0.114	1.067	3.591
MTMR12	0.842	0.315	2.674	0.007	2.321	0.731	1.808	0.071	1.252	4.301
MTMR11	-0.182	0.296	-0.615	0.539	0.833	0.247	-0.674	0.5	0.466	1.49
MTMR10	-0.087	0.296	-0.295	0.768	0.916	0.271	-0.308	0.758	0.513	1.637
MTMR1	0.328	0.298	1.101	0.271	1.389	0.414	0.938	0.348	0.774	2.492
MTM1	0.101	0.298	0.34	0.733	1.107	0.33	0.324	0.746	0.617	1.984
MTIF2	0.3	0.298	1.007	0.314	1.349	0.402	0.87	0.384	0.753	2.418
MTHFSD	0.208	0.296	0.704	0.482	1.232	0.365	0.635	0.525	0.689	2.201

MTHFR	0.132	0.297	0.445	0.657	1.141	0.339	0.416	0.677	0.638	2.042
MTHFD2L	0.636	0.306	2.079	0.038	1.888	0.578	1.538	0.124	1.037	3.439
MTHFD2	0.341	0.3	1.136	0.256	1.407	0.423	0.962	0.336	0.781	2.535
MTHFD1	1.025	0.321	3.196	0.001	2.788	0.894	1.999	0.046	1.486	5.228
MTG1	0.237	0.296	0.799	0.425	1.267	0.375	0.711	0.477	0.709	2.264
MTFR1	0.147	0.296	0.499	0.618	1.159	0.343	0.464	0.643	0.649	2.069
MTF2	-0.305	0.297	-1.029	0.304	0.737	0.219	-1.203	0.229	0.412	1.319
MTF1	-0.135	0.297	-0.454	0.65	0.874	0.259	-0.486	0.627	0.488	1.564
MTERF4	-0.135	0.296	-0.456	0.649	0.874	0.259	-0.488	0.626	0.489	1.561
MTERF3	0.581	0.306	1.901	0.057	1.788	0.547	1.442	0.149	0.982	3.256
MTERF1	0.134	0.295	0.453	0.651	1.143	0.338	0.424	0.672	0.641	2.039
MTDH	1.076	0.328	3.284	0.001	2.934	0.962	2.011	0.044	1.543	5.577
MTCL1	-0.04	0.297	-0.133	0.894	0.961	0.286	-0.136	0.892	0.537	1.722
MTCH2	0.543	0.299	1.815	0.069	1.722	0.515	1.401	0.161	0.958	3.095
MTCH1	0.568	0.306	1.856	0.063	1.765	0.54	1.416	0.157	0.969	3.217
MTARC2	0.381	0.298	1.278	0.201	1.463	0.436	1.062	0.288	0.816	2.624
MTARC1	-0.275	0.298	-0.926	0.355	0.759	0.226	-1.066	0.287	0.424	1.36
MTAP	-0.018	0.295	-0.061	0.952	0.982	0.29	-0.061	0.951	0.551	1.752
MTA2	0.322	0.298	1.078	0.281	1.38	0.412	0.922	0.356	0.769	2.476
MTA1	-0.479	0.3	-1.597	0.11	0.62	0.186	-2.048	0.041	0.344	1.115
MT4	-0.054	0.295	-0.181	0.856	0.948	0.28	-0.186	0.852	0.531	1.691
MT3	-0.055	0.296	-0.185	0.854	0.947	0.281	-0.19	0.85	0.53	1.692
MT2A	0.335	0.296	1.13	0.259	1.398	0.414	0.96	0.337	0.782	2.498
MT1X	0.402	0.298	1.35	0.177	1.494	0.445	1.112	0.266	0.834	2.678
MT1M	-0.102	0.299	-0.342	0.732	0.903	0.27	-0.36	0.719	0.502	1.622
MT1HL1	0.209	0.295	0.707	0.479	1.232	0.364	0.638	0.523	0.691	2.198
MT1H	0.583	0.302	1.927	0.054	1.791	0.541	1.461	0.144	0.99	3.239
MT1G	0.413	0.298	1.388	0.165	1.512	0.45	1.137	0.255	0.843	2.709
MT1F	0.239	0.295	0.809	0.419	1.27	0.375	0.719	0.472	0.712	2.265
MT1E	0.328	0.296	1.109	0.268	1.389	0.411	0.945	0.345	0.777	2.482
MSX2	0.009	0.295	0.031	0.975	1.009	0.298	0.031	0.975	0.566	1.8
MSX1	-0.007	0.296	-0.022	0.982	0.993	0.294	-0.023	0.982	0.556	1.774
MSTN	-0.367	0.3	-1.222	0.222	0.693	0.208	-1.476	0.14	0.385	1.248
MST1R	0.073	0.295	0.247	0.805	1.076	0.318	0.238	0.812	0.603	1.918
MST1L	-0.125	0.296	-0.421	0.674	0.883	0.261	-0.448	0.654	0.494	1.577
MST1	-0.267	0.296	-0.901	0.368	0.766	0.227	-1.033	0.302	0.428	1.369
MSRB2	0.556	0.303	1.834	0.067	1.744	0.529	1.406	0.16	0.962	3.16
MSRB1	-0.402	0.3	-1.339	0.18	0.669	0.201	-1.648	0.099	0.372	1.204

MSRA	0.432	0.3	1.439	0.15	1.54	0.462	1.169	0.242	0.855	2.771
MSR1	-0.204	0.296	-0.688	0.491	0.816	0.242	-0.763	0.445	0.456	1.457
MSN	-0.364	0.298	-1.22	0.223	0.695	0.207	-1.471	0.141	0.387	1.247
MSMO1	0.266	0.296	0.897	0.37	1.304	0.386	0.788	0.431	0.73	2.331
MSMB	-0.287	0.298	-0.965	0.335	0.75	0.223	-1.118	0.264	0.419	1.345
MSLN	0.335	0.298	1.124	0.261	1.398	0.417	0.955	0.34	0.779	2.507
MSL3	-0.116	0.296	-0.394	0.694	0.89	0.263	-0.418	0.676	0.499	1.588
MSL2	0.022	0.295	0.076	0.94	1.023	0.302	0.075	0.94	0.573	1.823
MSL1	0.112	0.295	0.38	0.704	1.119	0.33	0.359	0.719	0.627	1.996
MSI1	-0.084	0.295	-0.284	0.777	0.92	0.272	-0.296	0.767	0.515	1.641
MSH6	0.126	0.299	0.422	0.673	1.134	0.339	0.397	0.691	0.632	2.037
MSH4	-0.175	0.296	-0.592	0.554	0.839	0.249	-0.647	0.518	0.469	1.5
MSH3	0.04	0.296	0.136	0.892	1.041	0.308	0.133	0.894	0.583	1.859
MSH2	0.252	0.296	0.851	0.395	1.287	0.381	0.752	0.452	0.72	2.299
MSC	-0.347	0.3	-1.156	0.248	0.707	0.212	-1.382	0.167	0.393	1.273
MSANTD2	0.27	0.3	0.901	0.368	1.31	0.392	0.79	0.43	0.728	2.356
MS4A6A	-0.532	0.302	-1.76	0.078	0.587	0.178	-2.324	0.02	0.325	1.062
MS4A5	-0.177	0.296	-0.599	0.549	0.838	0.248	-0.655	0.512	0.469	1.496
MS4A4A	0.181	0.296	0.611	0.541	1.199	0.355	0.559	0.576	0.67	2.143
MS4A3	-0.125	0.296	-0.421	0.674	0.883	0.261	-0.449	0.654	0.494	1.577
MS4A2	-0.94	0.321	-2.923	0.003	0.391	0.126	-4.851	0	0.208	0.734
MS4A12	-0.303	0.297	-1.018	0.309	0.739	0.22	-1.189	0.234	0.412	1.323
MS4A1	-1.043	0.322	-3.237	0.001	0.352	0.114	-5.703	0	0.187	0.663
MRTO4	0.331	0.298	1.114	0.265	1.393	0.415	0.948	0.343	0.777	2.496
MRTFB	-0.401	0.3	-1.336	0.182	0.669	0.201	-1.644	0.1	0.372	1.206
MRTFA	0.223	0.296	0.755	0.45	1.25	0.37	0.676	0.499	0.7	2.234
MRS2	-0.014	0.297	-0.049	0.961	0.986	0.292	-0.049	0.961	0.551	1.763
MRPS7	0.144	0.295	0.489	0.625	1.155	0.341	0.455	0.649	0.648	2.06
MRPS35	-0.439	0.3	-1.464	0.143	0.645	0.193	-1.838	0.066	0.358	1.161
MRPS34	-0.174	0.295	-0.588	0.557	0.841	0.248	-0.642	0.521	0.471	1.5
MRPS33	0.03	0.295	0.101	0.92	1.03	0.304	0.099	0.921	0.578	1.837
MRPS31	-0.031	0.295	-0.103	0.918	0.97	0.286	-0.105	0.916	0.544	1.73
MRPS30	-0.09	0.296	-0.303	0.762	0.914	0.271	-0.317	0.751	0.512	1.634
MRPS28	0.514	0.3	1.714	0.087	1.672	0.501	1.34	0.18	0.929	3.009
MRPS27	-0.542	0.311	-1.745	0.081	0.582	0.181	-2.316	0.021	0.316	1.069
MRPS22	0.527	0.3	1.754	0.079	1.694	0.509	1.364	0.173	0.94	3.051
MRPS2	-0.432	0.303	-1.426	0.154	0.649	0.197	-1.784	0.075	0.359	1.175
MRPS18C	0.273	0.296	0.921	0.357	1.314	0.389	0.806	0.42	0.735	2.347

MRPS18B	0.807	0.312	2.589	0.01	2.242	0.699	1.776	0.076	1.217	4.13
MRPS18A	1.34	0.339	3.951	0	3.819	1.296	2.176	0.03	1.965	7.426
MRPS17	0.68	0.306	2.225	0.026	1.975	0.604	1.614	0.107	1.084	3.596
MRPS16	0.674	0.304	2.219	0.026	1.961	0.595	1.615	0.106	1.082	3.556
MRPS15	0.006	0.295	0.019	0.985	1.006	0.297	0.019	0.985	0.564	1.793
MRPS14	0.037	0.295	0.126	0.9	1.038	0.306	0.123	0.902	0.582	1.85
MRPS12	0.541	0.302	1.789	0.074	1.718	0.519	1.382	0.167	0.95	3.106
MRPS11	0.3	0.298	1.006	0.315	1.349	0.402	0.869	0.385	0.753	2.419
MRPS10	0.933	0.321	2.909	0.004	2.543	0.816	1.891	0.059	1.356	4.77
MRPL9	0.435	0.299	1.456	0.146	1.546	0.462	1.18	0.238	0.86	2.778
MRPL58	0.167	0.296	0.564	0.573	1.182	0.35	0.519	0.604	0.661	2.111
MRPL57	0.896	0.315	2.842	0.004	2.449	0.772	1.877	0.06	1.321	4.543
MRPL52	0.52	0.302	1.722	0.085	1.683	0.508	1.342	0.18	0.93	3.042
MRPL49	-0.257	0.296	-0.866	0.386	0.774	0.229	-0.988	0.323	0.433	1.383
MRPL48	0.439	0.3	1.465	0.143	1.551	0.465	1.186	0.236	0.862	2.791
MRPL46	0.458	0.3	1.528	0.126	1.581	0.474	1.226	0.22	0.879	2.846
MRPL44	-0.115	0.296	-0.389	0.697	0.891	0.263	-0.412	0.68	0.5	1.591
MRPL42	-0.113	0.296	-0.38	0.704	0.894	0.265	-0.402	0.688	0.5	1.597
MRPL41	0.057	0.296	0.192	0.848	1.059	0.313	0.187	0.852	0.592	1.891
MRPL40	-0.056	0.297	-0.19	0.85	0.945	0.281	-0.195	0.845	0.528	1.692
MRPL4	0.261	0.297	0.879	0.38	1.298	0.386	0.773	0.439	0.725	2.325
MRPL39	0.157	0.295	0.531	0.595	1.17	0.345	0.491	0.623	0.656	2.086
MRPL35	-0.067	0.297	-0.224	0.823	0.936	0.278	-0.231	0.817	0.522	1.676
MRPL34	0.176	0.298	0.59	0.555	1.192	0.356	0.541	0.588	0.665	2.14
MRPL33	0.355	0.298	1.19	0.234	1.426	0.425	1.002	0.316	0.795	2.559
MRPL3	0.314	0.298	1.055	0.291	1.369	0.408	0.905	0.365	0.764	2.454
MRPL28	0.472	0.299	1.574	0.115	1.602	0.48	1.255	0.209	0.891	2.882
MRPL24	-0.05	0.295	-0.168	0.866	0.951	0.281	-0.173	0.863	0.533	1.698
MRPL23	0.808	0.311	2.599	0.009	2.243	0.697	1.783	0.075	1.22	4.125
MRPL22	0.329	0.297	1.11	0.267	1.39	0.412	0.946	0.344	0.777	2.486
MRPL20	0.245	0.298	0.824	0.41	1.278	0.381	0.731	0.465	0.713	2.291
MRPL2	0.284	0.298	0.955	0.339	1.329	0.396	0.831	0.406	0.741	2.382
MRPL19	0.177	0.296	0.597	0.55	1.193	0.353	0.547	0.584	0.668	2.132
MRPL18	0.373	0.298	1.253	0.21	1.452	0.432	1.046	0.296	0.81	2.603
MRPL17	0.575	0.302	1.905	0.057	1.777	0.536	1.449	0.147	0.984	3.21
MRPL16	0.718	0.31	2.32	0.02	2.051	0.635	1.655	0.098	1.118	3.764
MRPL15	0.477	0.3	1.592	0.111	1.611	0.483	1.266	0.206	0.896	2.9
MRPL13	0.928	0.315	2.945	0.003	2.53	0.797	1.919	0.055	1.364	4.692

MRPL12	0.522	0.302	1.727	0.084	1.685	0.509	1.346	0.178	0.932	3.048
MRPL11	0.483	0.303	1.597	0.11	1.621	0.491	1.266	0.205	0.896	2.934
MROH9	-0.118	0.296	-0.397	0.691	0.889	0.263	-0.421	0.673	0.498	1.588
MROH7	-0.644	0.304	-2.116	0.034	0.525	0.16	-2.969	0.003	0.289	0.954
MRNIP	-0.604	0.306	-1.975	0.048	0.546	0.167	-2.713	0.007	0.3	0.995
MRM3	0.222	0.295	0.751	0.453	1.248	0.369	0.673	0.501	0.7	2.227
MRM2	1.058	0.321	3.291	0.001	2.88	0.926	2.031	0.042	1.534	5.407
MRM1	-0.165	0.296	-0.558	0.577	0.848	0.251	-0.607	0.544	0.474	1.515
MRGBP	0.527	0.302	1.741	0.082	1.693	0.512	1.353	0.176	0.936	3.063
MRFAP1L1	-0.288	0.297	-0.967	0.334	0.75	0.223	-1.12	0.263	0.419	1.344
MREG	-0.459	0.303	-1.518	0.129	0.632	0.191	-1.927	0.054	0.349	1.143
MRE11	0.451	0.3	1.506	0.132	1.57	0.471	1.212	0.225	0.873	2.826
MRC2	-0.43	0.298	-1.441	0.15	0.651	0.194	-1.8	0.072	0.363	1.167
MRC1	-0.343	0.299	-1.148	0.251	0.71	0.212	-1.369	0.171	0.395	1.275
MRAS	0.001	0.295	0.004	0.997	1.001	0.295	0.004	0.997	0.561	1.785
MR1	0.095	0.295	0.322	0.748	1.1	0.325	0.307	0.759	0.617	1.961
MPZL2	0.405	0.3	1.351	0.177	1.499	0.449	1.111	0.267	0.833	2.697
MPZL1	-0.681	0.307	-2.223	0.026	0.506	0.155	-3.187	0.001	0.277	0.922
MPZ	-0.221	0.297	-0.745	0.456	0.802	0.238	-0.834	0.405	0.448	1.434
MPV17	0.037	0.295	0.126	0.9	1.038	0.306	0.124	0.902	0.582	1.851
MPST	0.335	0.298	1.124	0.261	1.398	0.417	0.955	0.34	0.779	2.507
MPRIP	-0.414	0.299	-1.382	0.167	0.661	0.198	-1.712	0.087	0.368	1.189
MPPED2	-0.473	0.302	-1.565	0.118	0.623	0.188	-2.001	0.045	0.344	1.127
MPPED1	0.512	0.306	1.673	0.094	1.668	0.51	1.309	0.19	0.916	3.039
MPPE1	-0.039	0.295	-0.132	0.895	0.962	0.284	-0.134	0.893	0.539	1.715
MPP6	-0.352	0.298	-1.178	0.239	0.704	0.21	-1.412	0.158	0.392	1.263
MPP5	0.056	0.295	0.19	0.85	1.058	0.312	0.184	0.854	0.593	1.887
MPP3	0.204	0.299	0.684	0.494	1.227	0.366	0.619	0.536	0.683	2.202
MPP2	-0.249	0.298	-0.837	0.402	0.779	0.232	-0.951	0.342	0.435	1.397
MPP1	-0.241	0.298	-0.808	0.419	0.786	0.234	-0.913	0.361	0.439	1.409
MPO	0.222	0.296	0.75	0.453	1.249	0.37	0.673	0.501	0.699	2.232
MPL	0.333	0.297	1.121	0.262	1.395	0.415	0.953	0.34	0.779	2.498
MPIG6B	0.096	0.295	0.327	0.744	1.101	0.325	0.311	0.756	0.617	1.964
MPI	-0.228	0.298	-0.765	0.444	0.796	0.237	-0.859	0.39	0.445	1.427
MPHOSPH9	-0.219	0.298	-0.734	0.463	0.804	0.239	-0.821	0.412	0.448	1.44
MPHOSPH8	-0.198	0.296	-0.668	0.504	0.821	0.243	-0.738	0.46	0.459	1.466
MPHOSPH6	0.419	0.3	1.399	0.162	1.521	0.456	1.143	0.253	0.845	2.736
MPHOSPH10	0.573	0.306	1.875	0.061	1.773	0.542	1.427	0.153	0.974	3.227

MPG	-0.845	0.315	-2.683	0.007	0.43	0.135	-4.216	0	0.232	0.796
MPDZ	0.31	0.298	1.042	0.297	1.364	0.406	0.896	0.37	0.761	2.445
MPDU1	-0.381	0.3	-1.271	0.204	0.683	0.205	-1.548	0.122	0.38	1.229
MPC2	0.008	0.295	0.029	0.977	1.008	0.298	0.029	0.977	0.565	1.799
MPC1	0.263	0.297	0.885	0.376	1.301	0.386	0.778	0.437	0.727	2.328
MOXD1	-0.791	0.311	-2.541	0.011	0.453	0.141	-3.874	0	0.246	0.834
MOSPD3	-0.364	0.3	-1.215	0.224	0.695	0.208	-1.466	0.143	0.386	1.25
MOSPD2	-0.086	0.295	-0.291	0.771	0.918	0.271	-0.303	0.762	0.514	1.637
MOSPD1	0.327	0.3	1.087	0.277	1.386	0.416	0.928	0.354	0.769	2.497
MOS	-0.017	0.295	-0.056	0.955	0.983	0.29	-0.057	0.955	0.551	1.755
MORN1	0.099	0.295	0.335	0.738	1.104	0.326	0.319	0.75	0.619	1.968
MORF4L2	0.347	0.298	1.165	0.244	1.414	0.421	0.984	0.325	0.789	2.534
MORF4L1	0.34	0.3	1.135	0.257	1.405	0.421	0.962	0.336	0.781	2.527
MORC4	-0.151	0.296	-0.51	0.61	0.86	0.255	-0.551	0.582	0.481	1.537
MORC3	-0.091	0.297	-0.305	0.76	0.913	0.271	-0.319	0.749	0.511	1.634
MORC2	1.169	0.336	3.48	0.001	3.22	1.082	2.052	0.04	1.667	6.221
MORC1	0.09	0.295	0.303	0.762	1.094	0.323	0.29	0.772	0.613	1.951
MON2	-0.297	0.296	-1.004	0.315	0.743	0.22	-1.169	0.242	0.415	1.328
MON1B	-0.355	0.298	-1.19	0.234	0.702	0.209	-1.428	0.153	0.391	1.258
MOK	-0.76	0.31	-2.453	0.014	0.468	0.145	-3.675	0	0.255	0.858
MOGS	0.217	0.296	0.732	0.464	1.242	0.368	0.658	0.51	0.695	2.218
MOGAT2	0.347	0.298	1.166	0.244	1.415	0.422	0.985	0.325	0.789	2.537
MOG	-0.187	0.298	-0.627	0.53	0.83	0.247	-0.69	0.49	0.463	1.487
MOCS3	-0.175	0.296	-0.591	0.555	0.839	0.249	-0.646	0.518	0.47	1.5
MOCS2	-0.235	0.298	-0.788	0.431	0.791	0.236	-0.888	0.375	0.441	1.418
MOCS1	0.415	0.3	1.384	0.166	1.514	0.454	1.133	0.257	0.841	2.723
MOCOS	0.105	0.295	0.356	0.722	1.111	0.328	0.338	0.736	0.623	1.981
MOBP	0.034	0.296	0.116	0.908	1.035	0.306	0.114	0.909	0.58	1.847
MOB4	0.319	0.298	1.071	0.284	1.375	0.409	0.917	0.359	0.768	2.464
MOB3B	0.032	0.296	0.107	0.915	1.032	0.305	0.105	0.916	0.578	1.843
MOB1A	-0.131	0.295	-0.443	0.657	0.877	0.259	-0.474	0.636	0.492	1.565
MOAP1	-0.174	0.296	-0.585	0.558	0.841	0.249	-0.639	0.523	0.47	1.503
MNX1	0.253	0.298	0.849	0.396	1.287	0.383	0.75	0.453	0.718	2.307
MNT	-0.178	0.296	-0.602	0.547	0.837	0.248	-0.659	0.51	0.468	1.496
MNS1	0.194	0.296	0.657	0.511	1.215	0.359	0.598	0.55	0.68	2.168
MNDA	-0.092	0.295	-0.312	0.755	0.912	0.269	-0.326	0.744	0.511	1.627
MNAT1	0.585	0.303	1.93	0.054	1.795	0.544	1.461	0.144	0.991	3.251
MN1	-0.739	0.31	-2.385	0.017	0.478	0.148	-3.531	0	0.26	0.877

MMUT	0.328	0.297	1.105	0.269	1.388	0.412	0.942	0.346	0.776	2.484
MMS19	0.137	0.296	0.462	0.644	1.146	0.339	0.432	0.666	0.642	2.046
MMRN2	-0.352	0.298	-1.182	0.237	0.703	0.209	-1.416	0.157	0.392	1.261
MMRN1	-0.054	0.295	-0.184	0.854	0.947	0.279	-0.189	0.85	0.531	1.689
MMP9	0.309	0.3	1.03	0.303	1.362	0.408	0.886	0.376	0.757	2.451
MMP8	-0.062	0.296	-0.209	0.835	0.94	0.279	-0.215	0.83	0.526	1.68
MMP7	0.025	0.298	0.083	0.934	1.025	0.305	0.082	0.935	0.572	1.837
MMP3	-0.096	0.296	-0.324	0.746	0.909	0.269	-0.34	0.734	0.509	1.622
MMP28	-0.715	0.304	-2.354	0.019	0.489	0.149	-3.437	0.001	0.27	0.887
MMP27	-0.179	0.296	-0.605	0.545	0.836	0.248	-0.662	0.508	0.468	1.494
MMP26	0.18	0.295	0.608	0.543	1.197	0.354	0.557	0.578	0.671	2.135
MMP25	-0.07	0.295	-0.238	0.812	0.932	0.275	-0.246	0.806	0.522	1.663
MMP24OS	-0.079	0.295	-0.267	0.789	0.924	0.273	-0.278	0.781	0.518	1.649
MMP24	-0.054	0.295	-0.184	0.854	0.947	0.28	-0.189	0.85	0.531	1.689
MMP20	-0.311	0.298	-1.045	0.296	0.733	0.218	-1.226	0.22	0.409	1.313
MMP2	-0.512	0.3	-1.709	0.088	0.599	0.18	-2.232	0.026	0.333	1.078
MMP19	-0.508	0.302	-1.679	0.093	0.602	0.182	-2.188	0.029	0.333	1.089
MMP17	-0.452	0.302	-1.494	0.135	0.637	0.192	-1.889	0.059	0.352	1.151
MMP16	0.034	0.296	0.116	0.907	1.035	0.306	0.114	0.909	0.579	1.849
MMP15	0.073	0.297	0.246	0.806	1.076	0.319	0.237	0.813	0.601	1.924
MMP14	-0.205	0.297	-0.69	0.49	0.815	0.242	-0.766	0.444	0.456	1.457
MMP13	-0.113	0.295	-0.382	0.703	0.893	0.264	-0.404	0.686	0.501	1.594
MMP12	0.489	0.303	1.615	0.106	1.63	0.493	1.278	0.201	0.901	2.95
MMP11	0.016	0.295	0.054	0.957	1.016	0.3	0.054	0.957	0.57	1.812
MMP10	-0.363	0.3	-1.209	0.226	0.696	0.209	-1.458	0.145	0.386	1.253
MMP1	0.415	0.3	1.386	0.166	1.515	0.454	1.134	0.257	0.842	2.726
MME	-0.171	0.296	-0.579	0.563	0.843	0.249	-0.631	0.528	0.472	1.505
MMD	-0.023	0.295	-0.077	0.939	0.978	0.289	-0.078	0.938	0.548	1.744
MMADHC	-0.118	0.296	-0.396	0.692	0.889	0.264	-0.421	0.674	0.497	1.59
MMACHC	0.486	0.3	1.621	0.105	1.626	0.488	1.283	0.199	0.903	2.926
MLYCD	0.208	0.297	0.699	0.484	1.231	0.366	0.631	0.528	0.688	2.203
MLXIPL	-0.021	0.295	-0.071	0.944	0.979	0.289	-0.071	0.943	0.549	1.748
MLXIP	-0.264	0.298	-0.886	0.376	0.768	0.229	-1.014	0.31	0.428	1.378
MLX	0.317	0.298	1.065	0.287	1.373	0.409	0.913	0.361	0.766	2.46
MLST8	-0.236	0.298	-0.791	0.429	0.79	0.235	-0.892	0.372	0.44	1.417
MLPH	-0.156	0.296	-0.527	0.598	0.856	0.253	-0.57	0.568	0.479	1.529
MLNR	0.198	0.296	0.67	0.503	1.219	0.361	0.608	0.543	0.682	2.179
MLN	-0.26	0.297	-0.876	0.381	0.771	0.229	-1	0.317	0.431	1.379

MLLT3	-0.337	0.3	-1.123	0.261	0.714	0.214	-1.335	0.182	0.397	1.285
MLLT11	0.167	0.295	0.564	0.573	1.181	0.349	0.52	0.603	0.662	2.107
MLLT10	-0.113	0.299	-0.379	0.705	0.893	0.267	-0.401	0.688	0.497	1.603
MLLT1	-0.388	0.3	-1.295	0.195	0.678	0.203	-1.582	0.114	0.377	1.221
MLH3	-0.079	0.296	-0.268	0.789	0.924	0.274	-0.279	0.78	0.517	1.651
MLH1	-0.129	0.296	-0.435	0.663	0.879	0.26	-0.465	0.642	0.492	1.571
MLF2	0.112	0.295	0.379	0.705	1.118	0.33	0.358	0.72	0.627	1.994
MLF1	0.359	0.298	1.207	0.228	1.432	0.427	1.014	0.311	0.799	2.568
MLEC	-0.591	0.303	-1.948	0.051	0.554	0.168	-2.655	0.008	0.306	1.004
MLC1	0.076	0.295	0.256	0.798	1.079	0.319	0.247	0.805	0.605	1.925
MLANA	-0.624	0.306	-2.037	0.042	0.536	0.164	-2.827	0.005	0.294	0.977
MKS1	-0.033	0.296	-0.111	0.911	0.968	0.286	-0.113	0.91	0.542	1.729
MKRN7P	0.02	0.295	0.067	0.946	1.02	0.301	0.067	0.947	0.572	1.818
MKRN3	-0.377	0.299	-1.262	0.207	0.686	0.205	-1.533	0.125	0.381	1.232
MKRN2	-0.18	0.296	-0.607	0.544	0.836	0.248	-0.664	0.506	0.468	1.493
MKRN1	-0.499	0.301	-1.657	0.098	0.607	0.183	-2.149	0.032	0.337	1.096
MKNK2	-0.478	0.303	-1.578	0.115	0.62	0.188	-2.022	0.043	0.343	1.123
MKNK1	-0.548	0.302	-1.816	0.069	0.578	0.174	-2.418	0.016	0.32	1.044
MKLN1	-0.271	0.3	-0.904	0.366	0.763	0.229	-1.038	0.299	0.424	1.373
MKKS	0.021	0.295	0.073	0.942	1.022	0.301	0.072	0.943	0.573	1.822
MKI67	0.742	0.31	2.393	0.017	2.1	0.651	1.69	0.091	1.144	3.855
MITF	-0.232	0.296	-0.784	0.433	0.793	0.235	-0.882	0.378	0.444	1.417
MISP	-0.157	0.295	-0.53	0.596	0.855	0.253	-0.574	0.566	0.479	1.526
MIS18BP1	0.999	0.321	3.113	0.002	2.716	0.872	1.969	0.049	1.448	5.096
MIS18A	1.099	0.328	3.349	0.001	3.002	0.985	2.032	0.042	1.578	5.712
MIS12	0.358	0.298	1.201	0.23	1.431	0.426	1.01	0.313	0.798	2.565
MIR9-1HG	0.152	0.298	0.509	0.611	1.164	0.347	0.472	0.637	0.649	2.089
MIR622	0.239	0.296	0.807	0.419	1.27	0.377	0.718	0.473	0.711	2.271
MIR3916	-0.221	0.298	-0.741	0.458	0.802	0.239	-0.83	0.407	0.447	1.438
MIR1244-3	-0.008	0.296	-0.028	0.978	0.992	0.294	-0.028	0.977	0.555	1.772
MIPEP	0.032	0.296	0.109	0.913	1.033	0.305	0.108	0.914	0.579	1.844
MIP	-0.071	0.295	-0.242	0.809	0.931	0.275	-0.25	0.802	0.522	1.661
MIOS	0.427	0.3	1.423	0.155	1.532	0.459	1.158	0.247	0.851	2.758
MINPP1	0.453	0.299	1.515	0.13	1.573	0.47	1.218	0.223	0.875	2.826
MINK1	0.15	0.296	0.506	0.613	1.161	0.344	0.47	0.639	0.65	2.074
MINDY3	0.05	0.296	0.169	0.866	1.051	0.311	0.165	0.869	0.589	1.877
MINDY2	-0.968	0.328	-2.95	0.003	0.38	0.125	-4.975	0	0.2	0.723
MINDY1	0.095	0.295	0.323	0.747	1.1	0.325	0.308	0.758	0.616	1.963

MINAR1	-0.215	0.296	-0.726	0.468	0.807	0.239	-0.81	0.418	0.452	1.441
MILR1	0.387	0.298	1.301	0.193	1.473	0.439	1.078	0.281	0.822	2.642
MIIP	-0.827	0.311	-2.658	0.008	0.437	0.136	-4.135	0	0.237	0.805
MIF	0.166	0.295	0.562	0.574	1.181	0.349	0.518	0.604	0.662	2.106
MIER2	0.449	0.302	1.485	0.137	1.567	0.473	1.197	0.231	0.866	2.832
MIEF1	0.394	0.3	1.315	0.188	1.483	0.445	1.087	0.277	0.824	2.669
MID2	0.111	0.296	0.374	0.709	1.117	0.331	0.354	0.724	0.625	1.995
MID1IP1	-0.493	0.302	-1.633	0.103	0.61	0.185	-2.111	0.035	0.338	1.104
MID1	0.01	0.299	0.035	0.972	1.01	0.302	0.035	0.972	0.562	1.817
MICU2	0.588	0.304	1.933	0.053	1.8	0.547	1.462	0.144	0.992	3.267
MICU1	-0.171	0.296	-0.578	0.563	0.843	0.25	-0.63	0.529	0.471	1.506
MICOS10P1	0.306	0.3	1.021	0.307	1.359	0.408	0.88	0.379	0.755	2.446
MICB	0.664	0.306	2.166	0.03	1.942	0.595	1.583	0.113	1.065	3.54
MICALL2	-0.018	0.295	-0.062	0.95	0.982	0.29	-0.063	0.95	0.551	1.75
MICALL1	0.869	0.321	2.709	0.007	2.384	0.765	1.81	0.07	1.272	4.471
MICAL3	-0.083	0.296	-0.282	0.778	0.92	0.272	-0.294	0.769	0.515	1.642
MICAL2	0.051	0.296	0.172	0.864	1.052	0.311	0.168	0.867	0.589	1.878
MICAL1	0.161	0.296	0.543	0.587	1.175	0.348	0.502	0.616	0.657	2.099
MICA	0.409	0.298	1.37	0.171	1.505	0.449	1.124	0.261	0.839	2.701
MIA3	-0.322	0.298	-1.08	0.28	0.725	0.216	-1.273	0.203	0.404	1.3
MIA2	0.418	0.299	1.399	0.162	1.52	0.455	1.143	0.253	0.845	2.731
MIA	-0.154	0.296	-0.522	0.602	0.857	0.254	-0.564	0.573	0.48	1.531
MGST3	0.599	0.302	1.986	0.047	1.82	0.549	1.494	0.135	1.008	3.289
MGST2	-0.103	0.296	-0.347	0.728	0.902	0.267	-0.366	0.714	0.505	1.613
MGRN1	-0.309	0.296	-1.044	0.296	0.734	0.217	-1.224	0.221	0.411	1.312
MGP	0.146	0.296	0.495	0.621	1.158	0.342	0.461	0.645	0.649	2.066
MGMT	0.231	0.296	0.78	0.435	1.26	0.373	0.696	0.486	0.705	2.252
MGLL	0.116	0.295	0.392	0.695	1.123	0.332	0.37	0.711	0.629	2.003
MGC4859	0.293	0.297	0.986	0.324	1.34	0.398	0.855	0.393	0.749	2.398
MGC2889	0.256	0.296	0.866	0.387	1.292	0.383	0.763	0.445	0.723	2.31
MGAT5	0.248	0.298	0.834	0.404	1.282	0.382	0.738	0.46	0.715	2.297
MGAT4C	0.473	0.3	1.579	0.114	1.605	0.481	1.258	0.208	0.892	2.887
MGAT4B	-0.222	0.298	-0.746	0.456	0.801	0.239	-0.836	0.403	0.446	1.436
MGAT4A	0.293	0.296	0.987	0.324	1.34	0.397	0.856	0.392	0.749	2.395
MGAT3	-0.457	0.302	-1.513	0.13	0.633	0.191	-1.919	0.055	0.35	1.145
MGAT2	-0.152	0.295	-0.513	0.608	0.859	0.254	-0.554	0.579	0.482	1.533
MGAT1	-0.283	0.298	-0.951	0.341	0.753	0.224	-1.1	0.271	0.42	1.35
MGAM2	0.104	0.296	0.351	0.725	1.109	0.328	0.334	0.739	0.622	1.98

MGAM	-0.371	0.3	-1.239	0.215	0.69	0.207	-1.5	0.134	0.383	1.241
MGA	-0.154	0.296	-0.52	0.603	0.857	0.254	-0.562	0.574	0.48	1.532
MFSD9	-0.001	0.295	-0.005	0.996	0.999	0.295	-0.005	0.996	0.56	1.78
MFSD6	-0.031	0.297	-0.105	0.916	0.969	0.288	-0.107	0.915	0.542	1.734
MFSD5	-0.567	0.306	-1.856	0.064	0.567	0.173	-2.497	0.013	0.312	1.032
MFSD13A	0.622	0.303	2.053	0.04	1.862	0.564	1.529	0.126	1.029	3.37
MFSD12	0.11	0.296	0.371	0.71	1.116	0.33	0.352	0.725	0.625	1.994
MFSD11	-0.11	0.295	-0.371	0.711	0.896	0.265	-0.392	0.695	0.502	1.599
MFSD10	0.05	0.296	0.17	0.865	1.051	0.311	0.166	0.869	0.589	1.877
MFSD1	0.062	0.295	0.21	0.833	1.064	0.314	0.204	0.838	0.596	1.899
MFNG	-0.656	0.306	-2.144	0.032	0.519	0.159	-3.03	0.002	0.285	0.945
MFN2	-0.3	0.301	-0.999	0.318	0.741	0.223	-1.166	0.244	0.411	1.335
MFN1	0.41	0.3	1.369	0.171	1.507	0.452	1.123	0.261	0.838	2.711
MFHAS1	0.42	0.299	1.403	0.161	1.521	0.455	1.146	0.252	0.847	2.733
MFGE8	-0.175	0.296	-0.59	0.555	0.84	0.249	-0.644	0.519	0.47	1.501
MFF	-0.54	0.301	-1.795	0.073	0.583	0.175	-2.38	0.017	0.323	1.051
MFAP5	-0.606	0.306	-1.983	0.047	0.546	0.167	-2.725	0.006	0.3	0.993
MFAP4	-0.817	0.31	-2.636	0.008	0.442	0.137	-4.078	0	0.241	0.811
MFAP3L	0.342	0.3	1.137	0.255	1.407	0.423	0.963	0.335	0.781	2.536
MFAP3	-0.423	0.3	-1.412	0.158	0.655	0.196	-1.757	0.079	0.364	1.179
MFAP2	-0.251	0.296	-0.848	0.396	0.778	0.23	-0.964	0.335	0.435	1.39
MFAP1	0.143	0.295	0.483	0.629	1.153	0.341	0.45	0.653	0.646	2.058
MEX3D	-0.689	0.31	-2.227	0.026	0.502	0.155	-3.206	0.001	0.274	0.921
MEX3C	0.053	0.295	0.179	0.858	1.054	0.311	0.174	0.862	0.591	1.88
METTL9	0.224	0.298	0.752	0.452	1.251	0.372	0.674	0.5	0.698	2.242
METTL8	-0.12	0.297	-0.403	0.687	0.887	0.263	-0.428	0.668	0.496	1.587
METTL7A	-0.159	0.296	-0.536	0.592	0.853	0.253	-0.581	0.561	0.477	1.525
METTL5	0.426	0.299	1.424	0.154	1.531	0.457	1.16	0.246	0.852	2.75
METTL4	0.219	0.296	0.739	0.46	1.245	0.369	0.664	0.507	0.696	2.225
METTL3	-0.188	0.296	-0.636	0.525	0.828	0.245	-0.699	0.484	0.463	1.48
METTL2B	0.185	0.296	0.627	0.531	1.204	0.356	0.572	0.567	0.674	2.148
METTL22	0.509	0.306	1.664	0.096	1.663	0.508	1.304	0.192	0.914	3.028
METTL18	-0.189	0.298	-0.635	0.526	0.828	0.246	-0.699	0.485	0.462	1.484
METTL17	0.168	0.295	0.567	0.571	1.182	0.349	0.522	0.602	0.663	2.109
METTL1	0.197	0.299	0.658	0.51	1.217	0.363	0.598	0.55	0.678	2.185
METRN	0.286	0.298	0.961	0.337	1.331	0.397	0.835	0.403	0.743	2.387
METAP2	-0.073	0.297	-0.245	0.807	0.93	0.276	-0.254	0.8	0.52	1.663
METAP1	0.151	0.297	0.509	0.611	1.163	0.346	0.472	0.637	0.65	2.082

MET	-0.682	0.305	-2.235	0.025	0.505	0.154	-3.206	0.001	0.278	0.919
MEST	-0.327	0.3	-1.09	0.276	0.721	0.216	-1.289	0.197	0.401	1.298
MERTK	-0.131	0.296	-0.444	0.657	0.877	0.259	-0.475	0.635	0.491	1.566
MEPE	0.149	0.296	0.502	0.616	1.16	0.344	0.466	0.641	0.649	2.074
MEPCE	-0.248	0.298	-0.834	0.404	0.78	0.232	-0.946	0.344	0.435	1.399
MEP1B	-0.264	0.296	-0.891	0.373	0.768	0.228	-1.019	0.308	0.429	1.373
MEP1A	0.426	0.3	1.418	0.156	1.53	0.459	1.155	0.248	0.85	2.756
MEOX2	-0.26	0.296	-0.879	0.379	0.771	0.228	-1.004	0.315	0.431	1.377
MEOX1	-0.184	0.296	-0.624	0.532	0.832	0.246	-0.685	0.493	0.466	1.484
MEN1	0.344	0.3	1.149	0.251	1.411	0.423	0.972	0.331	0.784	2.538
MELTF	-0.28	0.297	-0.942	0.346	0.756	0.224	-1.087	0.277	0.422	1.353
MELK	0.511	0.3	1.703	0.089	1.666	0.5	1.333	0.182	0.926	2.999
MEIS3P1	-0.267	0.3	-0.89	0.374	0.765	0.23	-1.02	0.308	0.425	1.379
MEIS2	-0.263	0.296	-0.887	0.375	0.769	0.228	-1.014	0.311	0.43	1.375
MEIS1	-0.631	0.306	-2.063	0.039	0.532	0.163	-2.875	0.004	0.292	0.969
MEGF9	-0.437	0.3	-1.458	0.145	0.646	0.194	-1.829	0.067	0.359	1.162
MEGF8	0.433	0.299	1.447	0.148	1.541	0.461	1.175	0.24	0.858	2.769
MEGF6	-0.173	0.296	-0.583	0.56	0.841	0.249	-0.636	0.525	0.471	1.504
MEG3	-0.265	0.297	-0.89	0.374	0.768	0.228	-1.019	0.308	0.429	1.375
MEFV	-0.164	0.296	-0.553	0.58	0.849	0.251	-0.601	0.548	0.475	1.517
MEF2D	-0.073	0.295	-0.246	0.806	0.93	0.275	-0.255	0.799	0.521	1.659
MEF2C	-0.959	0.316	-3.031	0.002	0.383	0.121	-5.086	0	0.206	0.713
MEF2A	0.117	0.295	0.398	0.691	1.125	0.332	0.375	0.708	0.63	2.006
MED9	0.498	0.3	1.66	0.097	1.645	0.493	1.308	0.191	0.914	2.959
MED8	-0.041	0.297	-0.139	0.89	0.96	0.285	-0.141	0.888	0.536	1.718
MED7	0.237	0.296	0.801	0.423	1.268	0.375	0.713	0.476	0.709	2.265
MED6	-0.09	0.297	-0.302	0.763	0.914	0.271	-0.316	0.752	0.511	1.636
MED4	0.293	0.297	0.988	0.323	1.341	0.398	0.856	0.392	0.749	2.398
MED31	0.22	0.296	0.744	0.457	1.246	0.368	0.668	0.504	0.698	2.225
MED28	-0.377	0.298	-1.264	0.206	0.686	0.204	-1.535	0.125	0.383	1.231
MED27	0.89	0.311	2.859	0.004	2.436	0.759	1.893	0.058	1.323	4.485
MED25	-0.439	0.302	-1.453	0.146	0.644	0.195	-1.824	0.068	0.356	1.166
MED24	0.321	0.297	1.082	0.279	1.379	0.41	0.926	0.355	0.771	2.468
MED23	-0.414	0.3	-1.382	0.167	0.661	0.198	-1.712	0.087	0.367	1.189
MED22	0.339	0.298	1.136	0.256	1.403	0.418	0.964	0.335	0.782	2.516
MED20	0.172	0.295	0.582	0.561	1.188	0.351	0.535	0.593	0.666	2.119
MED18	0.189	0.296	0.638	0.524	1.208	0.357	0.581	0.561	0.676	2.156
MED17	-0.187	0.298	-0.629	0.529	0.829	0.247	-0.692	0.489	0.463	1.486

MED16	0.251	0.296	0.846	0.397	1.285	0.381	0.748	0.454	0.719	2.298
MED15	0.197	0.296	0.664	0.507	1.217	0.361	0.603	0.547	0.681	2.175
MED14	0.528	0.302	1.745	0.081	1.695	0.512	1.356	0.175	0.937	3.065
MED13L	-0.354	0.3	-1.18	0.238	0.702	0.211	-1.416	0.157	0.39	1.264
MED13	-0.254	0.296	-0.857	0.391	0.776	0.23	-0.976	0.329	0.434	1.386
MED12	-0.196	0.296	-0.661	0.509	0.822	0.243	-0.73	0.465	0.461	1.469
MED1	0.719	0.31	2.319	0.02	2.052	0.636	1.654	0.098	1.118	3.767
MECR	0.501	0.3	1.672	0.095	1.651	0.495	1.315	0.189	0.917	2.972
MECP2	0.912	0.317	2.878	0.004	2.489	0.789	1.888	0.059	1.338	4.632
MECOM	-0.072	0.295	-0.244	0.807	0.93	0.275	-0.253	0.8	0.522	1.66
MEAK7	0.176	0.298	0.59	0.555	1.192	0.355	0.541	0.588	0.665	2.139
MEAF6	0.039	0.296	0.132	0.895	1.04	0.308	0.129	0.897	0.582	1.858
MEA1	0.318	0.298	1.065	0.287	1.374	0.41	0.913	0.361	0.766	2.466
ME3	-0.187	0.297	-0.63	0.528	0.829	0.246	-0.693	0.488	0.463	1.484
ME2	0.506	0.298	1.697	0.09	1.659	0.495	1.332	0.183	0.925	2.977
ME1	0.423	0.3	1.41	0.159	1.526	0.458	1.15	0.25	0.848	2.748
MDN1	-0.037	0.297	-0.125	0.901	0.964	0.286	-0.127	0.899	0.539	1.724
MDM4	0.611	0.306	1.998	0.046	1.843	0.564	1.495	0.135	1.012	3.357
MDM2	0.242	0.297	0.813	0.416	1.274	0.379	0.722	0.47	0.711	2.282
MDM1	-0.286	0.298	-0.961	0.337	0.751	0.224	-1.112	0.266	0.419	1.347
MDK	0.147	0.295	0.496	0.62	1.158	0.342	0.462	0.644	0.649	2.066
MDH2	0.348	0.3	1.163	0.245	1.417	0.425	0.982	0.326	0.787	2.549
MDH1	0.698	0.305	2.287	0.022	2.009	0.613	1.647	0.1	1.105	3.652
MDFIC	-0.634	0.306	-2.073	0.038	0.531	0.162	-2.894	0.004	0.291	0.966
MDFI	0.492	0.301	1.633	0.102	1.635	0.492	1.29	0.197	0.906	2.95
MDC1	0.701	0.306	2.294	0.022	2.016	0.616	1.649	0.099	1.108	3.67
MCUR1	0.366	0.298	1.228	0.219	1.442	0.429	1.029	0.304	0.804	2.584
MCUB	0.13	0.295	0.441	0.66	1.139	0.336	0.413	0.68	0.639	2.031
MCTS1	0.724	0.307	2.361	0.018	2.063	0.633	1.68	0.093	1.131	3.763
MCTP2	0.377	0.303	1.243	0.214	1.458	0.442	1.036	0.3	0.805	2.642
MCTP1	-0.34	0.298	-1.141	0.254	0.712	0.212	-1.358	0.174	0.397	1.276
MCRS1	-0.482	0.303	-1.594	0.111	0.617	0.187	-2.048	0.041	0.341	1.117
MCPH1	-0.243	0.3	-0.812	0.417	0.784	0.235	-0.919	0.358	0.436	1.411
MCOLN3	-0.112	0.296	-0.38	0.704	0.894	0.265	-0.402	0.688	0.5	1.597
MCOLN1	0.09	0.296	0.303	0.762	1.094	0.324	0.29	0.772	0.612	1.955
MCMBP	-0.321	0.299	-1.074	0.283	0.725	0.217	-1.267	0.205	0.404	1.303
MCM9	0.057	0.296	0.193	0.847	1.059	0.313	0.188	0.851	0.593	1.89
MCM7	0.453	0.3	1.512	0.131	1.573	0.471	1.216	0.224	0.874	2.83

MCM6	0.784	0.315	2.491	0.013	2.19	0.689	1.726	0.084	1.182	4.058
MCM5	0.717	0.31	2.314	0.021	2.047	0.634	1.652	0.099	1.116	3.757
MCM4	0.194	0.297	0.654	0.513	1.214	0.361	0.594	0.552	0.678	2.173
MCM3AP-AS1	0.468	0.302	1.55	0.121	1.597	0.483	1.238	0.216	0.883	2.889
MCM3AP	-0.25	0.298	-0.841	0.401	0.779	0.232	-0.955	0.339	0.435	1.395
MCM3	0.261	0.296	0.881	0.378	1.298	0.384	0.775	0.438	0.726	2.32
MCM2	0.641	0.306	2.097	0.036	1.899	0.581	1.548	0.122	1.043	3.457
MCM10	0.673	0.306	2.201	0.028	1.959	0.599	1.602	0.109	1.076	3.567
MCL1	-0.6	0.303	-1.978	0.048	0.549	0.166	-2.711	0.007	0.303	0.994
MCHR1	-0.441	0.302	-1.461	0.144	0.644	0.194	-1.836	0.066	0.356	1.163
MCFD2	-0.11	0.297	-0.37	0.711	0.896	0.266	-0.391	0.696	0.5	1.604
MCF2L2	-0.055	0.296	-0.186	0.853	0.947	0.28	-0.191	0.848	0.53	1.689
MCF2L-AS1	0.584	0.303	1.929	0.054	1.794	0.543	1.461	0.144	0.991	3.247
MCF2L	0.297	0.298	0.996	0.319	1.346	0.402	0.862	0.389	0.75	2.416
MCF2	0.506	0.303	1.668	0.095	1.658	0.502	1.309	0.19	0.915	3.003
MCCC2	-0.546	0.306	-1.788	0.074	0.579	0.177	-2.38	0.017	0.318	1.054
MCCC1	0.477	0.302	1.578	0.114	1.611	0.487	1.255	0.209	0.891	2.914
MCC	-0.245	0.298	-0.823	0.41	0.783	0.233	-0.933	0.351	0.437	1.402
MCAT	-0.026	0.295	-0.089	0.929	0.974	0.288	-0.09	0.928	0.546	1.738
MCAM	0.155	0.296	0.522	0.602	1.167	0.346	0.484	0.629	0.653	2.085
MC5R	-0.158	0.297	-0.532	0.595	0.854	0.254	-0.576	0.565	0.477	1.529
MC4R	0.405	0.298	1.36	0.174	1.499	0.446	1.118	0.264	0.836	2.687
MC3R	-0.021	0.296	-0.072	0.943	0.979	0.289	-0.072	0.942	0.549	1.747
MC2R	0.266	0.298	0.895	0.371	1.305	0.388	0.785	0.432	0.728	2.338
MC1R	0.514	0.3	1.71	0.087	1.672	0.502	1.337	0.181	0.928	3.013
MBTPS2	-0.719	0.315	-2.284	0.022	0.487	0.153	-3.343	0.001	0.263	0.903
MBTPS1	-0.295	0.297	-0.991	0.322	0.745	0.222	-1.153	0.249	0.416	1.334
MBTD1	-0.277	0.297	-0.934	0.35	0.758	0.225	-1.076	0.282	0.424	1.356
MBP	0.761	0.31	2.454	0.014	2.14	0.664	1.718	0.086	1.166	3.93
MBOAT7	-0.138	0.299	-0.463	0.643	0.871	0.26	-0.497	0.619	0.485	1.565
MBOAT2	0.226	0.297	0.759	0.448	1.253	0.373	0.68	0.497	0.7	2.246
MBNL3	0.002	0.295	0.007	0.995	1.002	0.296	0.007	0.995	0.562	1.787
MBNL2	-0.247	0.296	-0.833	0.405	0.781	0.232	-0.944	0.345	0.437	1.397
MBNL1	-0.525	0.301	-1.746	0.081	0.592	0.178	-2.296	0.022	0.328	1.066
MBL2	0.382	0.3	1.276	0.202	1.466	0.439	1.061	0.289	0.815	2.636
MBIP	0.199	0.296	0.671	0.502	1.22	0.361	0.609	0.543	0.683	2.18
MBD5	0.196	0.299	0.656	0.512	1.217	0.363	0.596	0.551	0.677	2.185
MBD4	0.67	0.31	2.16	0.031	1.953	0.606	1.574	0.115	1.064	3.587

MBD3	0.212	0.296	0.714	0.475	1.236	0.366	0.644	0.52	0.691	2.208
MBD2	0.425	0.3	1.417	0.156	1.53	0.459	1.155	0.248	0.85	2.755
MBD1	-0.078	0.295	-0.263	0.793	0.925	0.273	-0.273	0.785	0.519	1.651
MB	0.086	0.295	0.291	0.771	1.09	0.321	0.279	0.781	0.611	1.943
MAZ	-0.371	0.298	-1.247	0.213	0.69	0.205	-1.509	0.131	0.385	1.237
MAX	0.074	0.296	0.249	0.804	1.076	0.319	0.24	0.811	0.602	1.923
MAVS	-0.175	0.298	-0.588	0.556	0.839	0.25	-0.643	0.52	0.468	1.505
MAU2	0.561	0.31	1.81	0.07	1.753	0.544	1.385	0.166	0.954	3.221
MATN4	0.334	0.298	1.121	0.262	1.396	0.416	0.953	0.34	0.779	2.503
MATN3	-0.683	0.31	-2.203	0.028	0.505	0.157	-3.16	0.002	0.275	0.927
MATN2	0.329	0.3	1.1	0.272	1.39	0.416	0.937	0.349	0.773	2.5
MATN1	-0.192	0.296	-0.649	0.516	0.825	0.244	-0.716	0.474	0.462	1.474
MATK	-0.22	0.296	-0.744	0.457	0.802	0.238	-0.832	0.406	0.449	1.434
MAT2B	-0.242	0.296	-0.818	0.414	0.785	0.233	-0.925	0.355	0.439	1.403
MAT2A	0.127	0.296	0.431	0.667	1.136	0.336	0.405	0.686	0.636	2.028
MAT1A	0.061	0.295	0.207	0.836	1.063	0.314	0.201	0.841	0.596	1.897
MAST4	-0.16	0.299	-0.536	0.592	0.852	0.255	-0.581	0.561	0.474	1.531
MAST3	-0.513	0.307	-1.674	0.094	0.599	0.184	-2.187	0.029	0.328	1.092
MAST2	0.47	0.3	1.569	0.117	1.6	0.479	1.252	0.211	0.89	2.879
MAST1	0.086	0.296	0.292	0.77	1.09	0.322	0.28	0.779	0.611	1.946
MASP2	-0.368	0.3	-1.224	0.221	0.692	0.208	-1.479	0.139	0.384	1.248
MASP1	-0.178	0.296	-0.6	0.548	0.837	0.248	-0.657	0.511	0.468	1.496
MAS1	0.338	0.298	1.137	0.256	1.403	0.417	0.964	0.335	0.783	2.513
MARK4	0.089	0.295	0.302	0.763	1.093	0.323	0.289	0.773	0.613	1.951
MARK3	-0.357	0.3	-1.193	0.233	0.699	0.21	-1.434	0.152	0.389	1.258
MARK2	0.093	0.296	0.315	0.753	1.098	0.325	0.301	0.764	0.615	1.96
MARK1	0.147	0.296	0.495	0.62	1.158	0.343	0.461	0.645	0.648	2.071
MARF1	-0.579	0.303	-1.908	0.056	0.561	0.17	-2.585	0.01	0.309	1.016
MARCO	0.14	0.296	0.474	0.635	1.15	0.34	0.442	0.658	0.645	2.053
MARCKSL1	-0.023	0.296	-0.079	0.937	0.977	0.289	-0.08	0.936	0.547	1.745
MARCKS	-0.047	0.295	-0.16	0.873	0.954	0.281	-0.164	0.87	0.535	1.701
MARCHF8	-0.772	0.315	-2.45	0.014	0.462	0.146	-3.693	0	0.249	0.857
MARCHF7	-0.116	0.295	-0.393	0.695	0.891	0.263	-0.416	0.677	0.499	1.589
MARCHF6	-0.272	0.298	-0.913	0.361	0.762	0.227	-1.049	0.294	0.425	1.366
MARCHF5	0.273	0.296	0.922	0.357	1.314	0.389	0.807	0.42	0.735	2.348
MARCHF3	0.251	0.297	0.845	0.398	1.285	0.381	0.747	0.455	0.718	2.299
MARCHF2	-0.465	0.307	-1.514	0.13	0.628	0.193	-1.928	0.054	0.344	1.147
MARCHF1	-0.019	0.296	-0.065	0.949	0.981	0.29	-0.065	0.948	0.55	1.751

MAPT	-0.537	0.306	-1.757	0.079	0.584	0.179	-2.327	0.02	0.321	1.064
MAPRE3	-0.149	0.298	-0.501	0.616	0.861	0.257	-0.54	0.589	0.48	1.544
MAPRE2	-0.643	0.306	-2.102	0.036	0.526	0.161	-2.948	0.003	0.289	0.958
MAPRE1	0.678	0.31	2.187	0.029	1.97	0.611	1.588	0.112	1.073	3.618
MAPKBP1	-0.418	0.3	-1.395	0.163	0.658	0.197	-1.731	0.083	0.366	1.185
MAPKAPK5-AS1	0.32	0.299	1.072	0.284	1.378	0.412	0.917	0.359	0.767	2.475
MAPKAPK5	0.098	0.295	0.332	0.74	1.103	0.325	0.316	0.752	0.619	1.966
MAPKAPK3	-0.635	0.306	-2.074	0.038	0.53	0.162	-2.896	0.004	0.291	0.966
MAPKAPK2	0.565	0.306	1.847	0.065	1.76	0.539	1.411	0.158	0.966	3.207
MAPKAP1	-0.024	0.295	-0.08	0.936	0.977	0.288	-0.081	0.935	0.547	1.742
MAPK9	0.173	0.296	0.584	0.559	1.189	0.352	0.536	0.592	0.665	2.123
MAPK8IP3	0.149	0.298	0.501	0.616	1.161	0.346	0.465	0.642	0.647	2.083
MAPK8IP2	-0.348	0.303	-1.151	0.25	0.706	0.214	-1.377	0.169	0.39	1.278
MAPK8IP1	-0.072	0.295	-0.243	0.808	0.931	0.275	-0.252	0.801	0.522	1.66
MAPK8	0.389	0.299	1.3	0.194	1.475	0.441	1.077	0.281	0.821	2.652
MAPK7	-0.03	0.295	-0.102	0.919	0.97	0.287	-0.104	0.917	0.544	1.731
MAPK6	-0.314	0.298	-1.053	0.292	0.731	0.218	-1.237	0.216	0.407	1.311
MAPK4	-0.499	0.3	-1.66	0.097	0.607	0.182	-2.153	0.031	0.337	1.094
MAPK3	-0.407	0.303	-1.343	0.179	0.666	0.202	-1.657	0.098	0.368	1.206
MAPK1IP1L	0.506	0.302	1.672	0.094	1.658	0.501	1.313	0.189	0.917	2.999
MAPK14	0.852	0.308	2.764	0.006	2.343	0.722	1.861	0.063	1.281	4.286
MAPK13	0.009	0.295	0.031	0.975	1.009	0.298	0.031	0.975	0.566	1.8
MAPK12	0.127	0.295	0.43	0.667	1.135	0.335	0.404	0.686	0.636	2.025
MAPK11	-0.162	0.295	-0.549	0.583	0.85	0.251	-0.596	0.551	0.477	1.517
MAPK10	0.09	0.296	0.304	0.761	1.094	0.323	0.291	0.771	0.613	1.953
MAPK1	-0.073	0.296	-0.245	0.807	0.93	0.276	-0.254	0.8	0.52	1.662
MAP9	0.171	0.296	0.578	0.563	1.186	0.351	0.531	0.595	0.664	2.119
MAP7D3	-0.21	0.297	-0.707	0.48	0.811	0.241	-0.786	0.432	0.453	1.451
MAP7D1	0.034	0.295	0.115	0.909	1.034	0.305	0.113	0.91	0.58	1.845
MAP7	-0.015	0.295	-0.053	0.958	0.985	0.29	-0.053	0.958	0.552	1.755
MAP6D1	0.551	0.303	1.818	0.069	1.735	0.526	1.398	0.162	0.958	3.144
MAP4K5	-0.178	0.296	-0.599	0.549	0.837	0.248	-0.656	0.512	0.468	1.497
MAP4K4	-0.596	0.302	-1.975	0.048	0.551	0.166	-2.701	0.007	0.305	0.995
MAP4K3	-0.059	0.295	-0.2	0.841	0.943	0.278	-0.206	0.837	0.528	1.682
MAP4K2	0.556	0.304	1.828	0.068	1.743	0.53	1.403	0.161	0.961	3.162
MAP4K1	0.026	0.296	0.088	0.93	1.026	0.303	0.087	0.931	0.575	1.832
MAP4	-0.101	0.295	-0.34	0.734	0.904	0.267	-0.358	0.72	0.507	1.613
MAP3K9	0.16	0.296	0.54	0.59	1.173	0.348	0.499	0.618	0.657	2.097

MAP3K8	-0.037	0.295	-0.127	0.899	0.963	0.284	-0.129	0.897	0.54	1.717
MAP3K7CL	-0.532	0.31	-1.718	0.086	0.587	0.182	-2.267	0.023	0.32	1.078
MAP3K7	-0.076	0.296	-0.258	0.796	0.926	0.274	-0.268	0.789	0.518	1.655
MAP3K6	-0.541	0.306	-1.769	0.077	0.582	0.178	-2.346	0.019	0.32	1.06
MAP3K5	-0.471	0.302	-1.559	0.119	0.624	0.189	-1.992	0.046	0.345	1.129
MAP3K4	-0.09	0.296	-0.304	0.761	0.914	0.271	-0.319	0.75	0.511	1.634
MAP3K3	-0.126	0.295	-0.426	0.67	0.882	0.26	-0.454	0.65	0.494	1.573
MAP3K20	0.339	0.298	1.138	0.255	1.403	0.418	0.966	0.334	0.783	2.515
MAP3K2	-0.571	0.306	-1.867	0.062	0.565	0.173	-2.517	0.012	0.311	1.029
MAP3K19	0.208	0.296	0.701	0.483	1.231	0.365	0.633	0.527	0.689	2.2
MAP3K14	-0.15	0.297	-0.504	0.614	0.861	0.256	-0.544	0.587	0.481	1.54
MAP3K13	-0.141	0.296	-0.476	0.634	0.869	0.257	-0.511	0.609	0.487	1.55
MAP3K12	-0.387	0.302	-1.278	0.201	0.679	0.205	-1.56	0.119	0.376	1.229
MAP3K11	0.816	0.316	2.587	0.01	2.262	0.714	1.768	0.077	1.219	4.199
MAP3K10	0.552	0.302	1.825	0.068	1.737	0.525	1.403	0.161	0.96	3.141
MAP3K1	0.163	0.296	0.552	0.581	1.177	0.348	0.51	0.61	0.66	2.101
MAP2K7	0.169	0.296	0.572	0.567	1.184	0.35	0.526	0.599	0.663	2.115
MAP2K6	0.314	0.3	1.047	0.295	1.369	0.41	0.899	0.369	0.761	2.464
MAP2K5	-0.114	0.296	-0.384	0.701	0.892	0.264	-0.407	0.684	0.499	1.595
MAP2K4	0.021	0.295	0.07	0.945	1.021	0.301	0.069	0.945	0.572	1.82
MAP2K2	-0.529	0.302	-1.749	0.08	0.589	0.178	-2.304	0.021	0.326	1.066
MAP2K1	-0.007	0.295	-0.023	0.982	0.993	0.293	-0.023	0.982	0.557	1.772
MAP2	0.316	0.3	1.053	0.292	1.372	0.411	0.903	0.366	0.762	2.469
MAP1S	0.064	0.295	0.217	0.828	1.066	0.315	0.21	0.834	0.598	1.902
MAP1LC3C	-0.157	0.296	-0.531	0.595	0.854	0.253	-0.575	0.565	0.478	1.527
MAP1LC3B	-0.606	0.306	-1.981	0.048	0.546	0.167	-2.722	0.006	0.3	0.994
MAP1B	0.292	0.3	0.972	0.331	1.339	0.402	0.843	0.399	0.743	2.412
MAP1A	-0.48	0.299	-1.603	0.109	0.619	0.185	-2.057	0.04	0.344	1.113
MAP11	0.672	0.306	2.194	0.028	1.958	0.599	1.598	0.11	1.074	3.568
MAOB	-0.161	0.296	-0.542	0.588	0.852	0.252	-0.588	0.557	0.476	1.522
MAOA	-0.56	0.306	-1.832	0.067	0.571	0.175	-2.455	0.014	0.314	1.04
MANSC1	0.354	0.298	1.189	0.235	1.425	0.424	1.001	0.317	0.795	2.553
MANF	0.486	0.3	1.619	0.105	1.627	0.489	1.282	0.2	0.903	2.931
MANEA	-0.091	0.295	-0.308	0.758	0.913	0.27	-0.322	0.747	0.512	1.629
MANBA	-0.255	0.297	-0.858	0.391	0.775	0.23	-0.977	0.329	0.433	1.388
MAN2C1	-0.322	0.297	-1.086	0.278	0.725	0.215	-1.281	0.2	0.405	1.296
MAN2B2	-0.265	0.302	-0.878	0.38	0.767	0.231	-1.005	0.315	0.425	1.386
MAN2B1	-0.101	0.297	-0.34	0.734	0.904	0.268	-0.357	0.721	0.506	1.617

MAN2A2	-0.011	0.295	-0.038	0.97	0.989	0.292	-0.038	0.97	0.555	1.764
MAN2A1	-0.412	0.302	-1.364	0.173	0.662	0.2	-1.688	0.091	0.366	1.197
MAN1C1	-0.03	0.297	-0.099	0.921	0.971	0.288	-0.101	0.92	0.543	1.737
MAN1B1	-0.611	0.31	-1.971	0.049	0.543	0.168	-2.716	0.007	0.296	0.997
MAN1A2	-0.559	0.306	-1.829	0.067	0.572	0.175	-2.45	0.014	0.314	1.041
MAN1A1	-0.839	0.315	-2.665	0.008	0.432	0.136	-4.173	0	0.233	0.801
MAMLD1	-0.026	0.295	-0.09	0.929	0.974	0.288	-0.091	0.928	0.546	1.738
MAML3	-0.842	0.316	-2.669	0.008	0.431	0.136	-4.189	0	0.232	0.799
MAML1	-0.59	0.303	-1.944	0.052	0.554	0.168	-2.648	0.008	0.306	1.005
MALT1	0.096	0.295	0.324	0.746	1.1	0.325	0.309	0.757	0.617	1.962
MALL	-0.134	0.295	-0.454	0.65	0.875	0.258	-0.486	0.627	0.49	1.56
MAL	0.114	0.296	0.386	0.699	1.121	0.331	0.365	0.715	0.628	2.001
MAK16	-0.211	0.296	-0.711	0.477	0.81	0.24	-0.791	0.429	0.453	1.448
MAK	-0.613	0.306	-2.004	0.045	0.542	0.166	-2.766	0.006	0.297	0.987
MAIP1	0.995	0.328	3.039	0.002	2.706	0.886	1.924	0.054	1.424	5.142
MAGT1	0.596	0.303	1.967	0.049	1.814	0.549	1.482	0.138	1.002	3.284
MAGOHB	0.344	0.3	1.148	0.251	1.411	0.423	0.972	0.331	0.784	2.537
MAGOH2P	0.184	0.296	0.621	0.534	1.202	0.355	0.567	0.57	0.673	2.146
MAGIX	-0.051	0.295	-0.174	0.862	0.95	0.28	-0.179	0.858	0.533	1.694
MAGI2	-0.336	0.3	-1.12	0.263	0.715	0.214	-1.331	0.183	0.397	1.287
MAGI1	-0.166	0.296	-0.56	0.576	0.847	0.251	-0.609	0.543	0.474	1.514
MAGEL2	-0.216	0.295	-0.732	0.464	0.805	0.238	-0.818	0.414	0.451	1.437
MAGEH1	-0.04	0.295	-0.137	0.891	0.96	0.283	-0.14	0.889	0.539	1.712
MAGEF1	1.056	0.321	3.288	0.001	2.875	0.923	2.03	0.042	1.532	5.396
MAGED2	0.061	0.295	0.205	0.838	1.062	0.314	0.199	0.842	0.595	1.896
MAGED1	0.068	0.295	0.23	0.818	1.07	0.316	0.223	0.824	0.6	1.909
MAGEC3	0.223	0.298	0.749	0.454	1.25	0.373	0.671	0.502	0.697	2.243
MAGEC2	0.179	0.295	0.606	0.544	1.196	0.353	0.555	0.579	0.67	2.135
MAGEC1	-0.067	0.296	-0.226	0.821	0.935	0.276	-0.234	0.815	0.524	1.669
MAGEB4	0.187	0.296	0.63	0.529	1.205	0.357	0.575	0.565	0.674	2.155
MAGEB3	-0.169	0.296	-0.571	0.568	0.844	0.25	-0.622	0.534	0.473	1.509
MAGEB2	0.013	0.297	0.044	0.965	1.013	0.301	0.044	0.965	0.566	1.814
MAGEB1	0.741	0.307	2.412	0.016	2.099	0.645	1.703	0.088	1.149	3.833
MAGEA8	1.088	0.336	3.235	0.001	2.967	0.997	1.972	0.049	1.535	5.733
MAGEA6	-0.345	0.3	-1.151	0.25	0.708	0.212	-1.374	0.169	0.394	1.275
MAGEA4	-0.035	0.296	-0.119	0.905	0.965	0.286	-0.121	0.904	0.54	1.725
MAGEA12	0.295	0.297	0.995	0.32	1.343	0.399	0.861	0.389	0.751	2.403
MAGEA11	0.62	0.303	2.043	0.041	1.859	0.564	1.523	0.128	1.026	3.368

MAGEA10	0.525	0.3	1.752	0.08	1.691	0.507	1.363	0.173	0.939	3.045
MAGEA1	0.256	0.296	0.864	0.388	1.292	0.383	0.762	0.446	0.723	2.309
MAG	-0.277	0.297	-0.933	0.351	0.758	0.225	-1.075	0.283	0.423	1.357
MAFK	0.078	0.297	0.262	0.793	1.081	0.321	0.252	0.801	0.604	1.934
MAFG	0.772	0.315	2.453	0.014	2.163	0.681	1.709	0.087	1.168	4.008
MAFF	0.128	0.295	0.433	0.665	1.136	0.336	0.407	0.684	0.637	2.027
MAFB	-0.144	0.295	-0.488	0.626	0.866	0.256	-0.525	0.6	0.485	1.545
MAF	-0.807	0.308	-2.618	0.009	0.446	0.138	-4.026	0	0.244	0.816
MAEA	0.17	0.296	0.574	0.566	1.185	0.35	0.528	0.598	0.664	2.115
MADD	0.044	0.296	0.148	0.883	1.045	0.309	0.144	0.885	0.585	1.866
MADCAM1	0.687	0.31	2.218	0.027	1.987	0.615	1.604	0.109	1.083	3.646
MAD2L1BP	0.073	0.295	0.249	0.803	1.076	0.318	0.24	0.81	0.604	1.919
MAD2L1	0.661	0.31	2.132	0.033	1.937	0.6	1.56	0.119	1.055	3.556
MAD1L1	-0.719	0.315	-2.282	0.022	0.487	0.153	-3.34	0.001	0.263	0.903
MACROH2A2	1.073	0.329	3.266	0.001	2.925	0.961	2.002	0.045	1.536	5.571
MACROH2A1	0.781	0.315	2.481	0.013	2.183	0.687	1.722	0.085	1.178	4.046
MACROD1	0.42	0.299	1.407	0.159	1.523	0.455	1.148	0.251	0.848	2.735
MACO1	0.115	0.295	0.39	0.697	1.122	0.331	0.368	0.713	0.629	2.002
MACIR	-0.067	0.297	-0.225	0.822	0.935	0.278	-0.233	0.816	0.523	1.674
MACF1	-0.559	0.306	-1.828	0.068	0.572	0.175	-2.448	0.014	0.314	1.041
MAB21L4	0.478	0.303	1.58	0.114	1.613	0.488	1.256	0.209	0.891	2.92
MAB21L2	-0.292	0.298	-0.981	0.327	0.746	0.222	-1.14	0.254	0.416	1.339
M6PR	-0.725	0.315	-2.303	0.021	0.485	0.152	-3.381	0.001	0.262	0.898
LZTS3	0.134	0.296	0.451	0.652	1.143	0.339	0.422	0.673	0.639	2.043
LZTS1	0.052	0.296	0.176	0.86	1.054	0.312	0.172	0.864	0.59	1.881
LZTR1	0.066	0.297	0.224	0.823	1.069	0.317	0.217	0.829	0.597	1.912
LZTFL1	-0.591	0.306	-1.935	0.053	0.554	0.169	-2.639	0.008	0.304	1.008
LYZL6	-0.143	0.296	-0.482	0.63	0.867	0.257	-0.518	0.604	0.485	1.549
LYZ	0.168	0.298	0.562	0.574	1.183	0.353	0.518	0.605	0.659	2.122
LYVE1	-0.268	0.297	-0.903	0.366	0.765	0.227	-1.036	0.3	0.428	1.368
LYST	-0.279	0.296	-0.94	0.347	0.757	0.224	-1.084	0.278	0.423	1.353
LYRM9	0.015	0.297	0.052	0.959	1.015	0.302	0.051	0.959	0.567	1.817
LYRM4	-0.225	0.296	-0.759	0.448	0.798	0.237	-0.852	0.394	0.447	1.427
LYRM2	0.566	0.303	1.869	0.062	1.761	0.533	1.427	0.154	0.973	3.188
LYRM1	-0.32	0.298	-1.077	0.282	0.726	0.216	-1.269	0.204	0.405	1.301
LYPLA2	-0.205	0.296	-0.692	0.489	0.815	0.241	-0.768	0.443	0.456	1.456
LYPLA1	0.014	0.296	0.046	0.963	1.014	0.3	0.046	0.963	0.567	1.811
LYPD3	-0.267	0.301	-0.887	0.375	0.766	0.23	-1.016	0.309	0.425	1.381

LYPD1	0.203	0.295	0.686	0.493	1.225	0.362	0.621	0.535	0.686	2.185
LYN	-0.051	0.296	-0.173	0.863	0.95	0.281	-0.177	0.859	0.532	1.698
LYL1	-0.365	0.298	-1.224	0.221	0.694	0.207	-1.478	0.14	0.387	1.245
LY96	-0.675	0.306	-2.207	0.027	0.509	0.156	-3.153	0.002	0.279	0.927
LY9	-0.581	0.303	-1.918	0.055	0.559	0.17	-2.601	0.009	0.309	1.013
LY86	-0.505	0.306	-1.65	0.099	0.603	0.185	-2.147	0.032	0.331	1.1
LY75	-0.344	0.298	-1.156	0.248	0.709	0.211	-1.38	0.168	0.396	1.27
LY6H	-0.048	0.295	-0.161	0.872	0.954	0.282	-0.165	0.869	0.535	1.701
LY6G6E	-0.193	0.296	-0.653	0.514	0.824	0.244	-0.72	0.471	0.461	1.473
LY6G6C	0.213	0.298	0.714	0.475	1.237	0.369	0.643	0.52	0.69	2.219
LY6G5C	0.294	0.298	0.986	0.324	1.341	0.399	0.855	0.393	0.748	2.405
LY6E	0.672	0.31	2.17	0.03	1.959	0.607	1.58	0.114	1.067	3.595
LY6D	0.488	0.302	1.614	0.107	1.629	0.493	1.277	0.202	0.901	2.948
LXN	-0.379	0.3	-1.263	0.207	0.685	0.205	-1.535	0.125	0.381	1.232
LUZP4	-0.265	0.299	-0.887	0.375	0.767	0.229	-1.016	0.31	0.427	1.378
LUZP2	-0.08	0.296	-0.272	0.786	0.923	0.273	-0.283	0.777	0.517	1.647
LUZP1	0.296	0.297	0.995	0.32	1.344	0.4	0.861	0.389	0.75	2.408
LUNAR1	-0.141	0.295	-0.476	0.634	0.869	0.257	-0.511	0.609	0.487	1.55
LUM	-0.685	0.303	-2.257	0.024	0.504	0.153	-3.242	0.001	0.278	0.914
LUC7L3	0.48	0.3	1.6	0.11	1.615	0.484	1.271	0.204	0.898	2.907
LUC7L	0.126	0.296	0.427	0.67	1.134	0.336	0.401	0.689	0.635	2.026
LTN1	-0.778	0.31	-2.509	0.012	0.459	0.142	-3.797	0	0.25	0.843
LTK	-0.349	0.297	-1.175	0.24	0.705	0.21	-1.406	0.16	0.394	1.263
LTF	-0.479	0.3	-1.599	0.11	0.619	0.186	-2.051	0.04	0.344	1.114
LTC4S	-0.291	0.303	-0.96	0.337	0.747	0.227	-1.114	0.265	0.412	1.354
LTBR	-0.355	0.3	-1.184	0.237	0.702	0.21	-1.421	0.155	0.39	1.262
LTBP4	0.304	0.298	1.02	0.308	1.355	0.403	0.88	0.379	0.756	2.428
LTBP3	-0.732	0.315	-2.324	0.02	0.481	0.152	-3.427	0.001	0.259	0.892
LTBP2	-0.45	0.298	-1.509	0.131	0.638	0.19	-1.906	0.057	0.355	1.144
LTBP1	-0.5	0.301	-1.664	0.096	0.606	0.182	-2.159	0.031	0.336	1.093
LTB4R2	0.382	0.3	1.273	0.203	1.465	0.439	1.058	0.29	0.814	2.637
LTB4R	-0.083	0.296	-0.281	0.779	0.92	0.272	-0.293	0.769	0.515	1.643
LTB	-0.62	0.306	-2.026	0.043	0.538	0.165	-2.807	0.005	0.295	0.98
LTA4H	-0.136	0.296	-0.459	0.646	0.873	0.259	-0.491	0.623	0.488	1.56
LTA	-0.26	0.298	-0.873	0.383	0.771	0.23	-0.997	0.319	0.43	1.383
LST1	-0.212	0.296	-0.715	0.474	0.809	0.24	-0.797	0.426	0.453	1.446
LSS	0.266	0.298	0.894	0.372	1.305	0.389	0.785	0.433	0.728	2.339
LSR	0.361	0.298	1.211	0.226	1.434	0.427	1.017	0.309	0.8	2.57

LSP1	-0.557	0.303	-1.839	0.066	0.573	0.174	-2.46	0.014	0.317	1.037
LSM8	0.476	0.3	1.588	0.112	1.61	0.483	1.263	0.207	0.894	2.898
LSM7	0.091	0.296	0.309	0.758	1.096	0.324	0.295	0.768	0.614	1.956
LSM6	0.396	0.3	1.32	0.187	1.485	0.445	1.091	0.275	0.826	2.671
LSM5	0.483	0.302	1.6	0.11	1.622	0.49	1.268	0.205	0.897	2.933
LSM4	0.008	0.295	0.027	0.978	1.008	0.298	0.027	0.978	0.565	1.799
LSM3	-0.1	0.297	-0.338	0.735	0.905	0.268	-0.355	0.722	0.506	1.618
LSM2	-0.084	0.295	-0.284	0.776	0.92	0.272	-0.296	0.767	0.515	1.64
LSM14B	1.288	0.337	3.818	0	3.625	1.223	2.147	0.032	1.872	7.022
LSM14A	-0.278	0.3	-0.924	0.355	0.758	0.228	-1.065	0.287	0.421	1.365
LSM12	0.24	0.296	0.809	0.418	1.271	0.377	0.72	0.472	0.711	2.272
LSM1	0.475	0.3	1.581	0.114	1.608	0.483	1.258	0.208	0.892	2.896
LSG1	0.496	0.301	1.65	0.099	1.642	0.494	1.301	0.193	0.911	2.959
LSAMP	-0.345	0.298	-1.159	0.247	0.708	0.211	-1.383	0.167	0.395	1.269
LRTM1	0.166	0.296	0.562	0.574	1.181	0.35	0.518	0.605	0.661	2.11
LRRTM4	-0.138	0.296	-0.465	0.642	0.871	0.258	-0.499	0.618	0.487	1.557
LRRTM2	-0.068	0.295	-0.229	0.819	0.935	0.276	-0.237	0.813	0.524	1.667
LRRN3	-0.849	0.315	-2.699	0.007	0.428	0.135	-4.252	0	0.231	0.792
LRRN2	0.47	0.303	1.555	0.12	1.601	0.484	1.24	0.215	0.885	2.896
LRRK1	-0.671	0.31	-2.164	0.031	0.511	0.159	-3.083	0.002	0.278	0.939
LRRFIP2	0.327	0.297	1.101	0.271	1.387	0.412	0.939	0.348	0.775	2.482
LRRFIP1	-0.152	0.297	-0.513	0.608	0.859	0.255	-0.554	0.58	0.48	1.536
LRRC8E	-0.023	0.296	-0.078	0.938	0.977	0.289	-0.079	0.937	0.547	1.746
LRRC8D	0.621	0.306	2.031	0.042	1.861	0.569	1.513	0.13	1.022	3.387
LRRC8B	0.218	0.295	0.738	0.46	1.244	0.367	0.663	0.507	0.697	2.219
LRRC75B	0.442	0.302	1.462	0.144	1.556	0.47	1.181	0.237	0.86	2.813
LRRC61	0.076	0.296	0.256	0.798	1.079	0.319	0.247	0.805	0.604	1.927
LRRC6	0.005	0.295	0.016	0.987	1.005	0.296	0.016	0.987	0.564	1.792
LRRC59	0.206	0.296	0.695	0.487	1.229	0.364	0.628	0.53	0.688	2.196
LRRC49	-0.229	0.3	-0.764	0.445	0.795	0.239	-0.859	0.39	0.442	1.432
LRRC47	0.055	0.296	0.186	0.853	1.057	0.313	0.181	0.857	0.591	1.888
LRRC42	0.036	0.295	0.123	0.902	1.037	0.306	0.121	0.904	0.581	1.85
LRRC41	-0.118	0.296	-0.398	0.691	0.889	0.263	-0.422	0.673	0.497	1.589
LRRC40	-0.032	0.295	-0.109	0.913	0.968	0.286	-0.111	0.912	0.543	1.727
LRRC37BP1	0.196	0.296	0.663	0.507	1.217	0.36	0.602	0.547	0.681	2.174
LRRC37A2	-0.381	0.3	-1.27	0.204	0.683	0.205	-1.546	0.122	0.379	1.23
LRRC36	-0.098	0.296	-0.333	0.739	0.906	0.268	-0.35	0.727	0.508	1.618
LRRC32	-0.646	0.306	-2.115	0.034	0.524	0.16	-2.973	0.003	0.288	0.954

LRRC31	0.126	0.296	0.424	0.671	1.134	0.336	0.399	0.69	0.634	2.027
LRRC3	-0.371	0.3	-1.236	0.216	0.69	0.207	-1.497	0.135	0.383	1.243
LRRC23	0.09	0.295	0.306	0.76	1.095	0.323	0.292	0.77	0.614	1.952
LRRC20	0.55	0.302	1.82	0.069	1.734	0.524	1.4	0.162	0.959	3.136
LRRC2	0.162	0.298	0.544	0.587	1.176	0.35	0.502	0.616	0.656	2.108
LRRC19	0.176	0.295	0.597	0.551	1.193	0.352	0.547	0.584	0.668	2.129
LRRC17	-0.78	0.315	-2.478	0.013	0.459	0.144	-3.753	0	0.247	0.85
LRRC15	-0.758	0.31	-2.447	0.014	0.469	0.145	-3.66	0	0.255	0.86
LRRC14	0.428	0.3	1.429	0.153	1.534	0.46	1.163	0.245	0.853	2.76
LRRC1	0.212	0.297	0.716	0.474	1.237	0.367	0.645	0.519	0.692	2.212
LRPPRC	-0.092	0.296	-0.309	0.757	0.912	0.27	-0.324	0.746	0.511	1.631
LRPAP1	0.66	0.304	2.168	0.03	1.934	0.588	1.587	0.112	1.065	3.511
LRP8	0.588	0.303	1.94	0.052	1.8	0.545	1.467	0.142	0.994	3.258
LRP6	0.563	0.302	1.863	0.063	1.756	0.531	1.424	0.154	0.971	3.176
LRP5L	0.051	0.295	0.172	0.864	1.052	0.31	0.167	0.867	0.59	1.876
LRP5	0.432	0.301	1.435	0.151	1.54	0.464	1.165	0.244	0.854	2.778
LRP4	-0.587	0.302	-1.947	0.052	0.556	0.168	-2.649	0.008	0.308	1.004
LRP3	1.169	0.336	3.478	0.001	3.22	1.083	2.051	0.04	1.666	6.224
LRP2BP	-0.055	0.296	-0.185	0.853	0.947	0.28	-0.19	0.849	0.53	1.69
LRP2	-0.308	0.3	-1.026	0.305	0.735	0.221	-1.201	0.23	0.408	1.324
LRP1B	0.525	0.303	1.736	0.083	1.691	0.512	1.35	0.177	0.934	3.06
LRP12	0.024	0.295	0.082	0.935	1.024	0.302	0.081	0.936	0.574	1.827
LRP10	-0.944	0.321	-2.946	0.003	0.389	0.125	-4.901	0	0.207	0.729
LRP1	-0.647	0.304	-2.127	0.033	0.524	0.159	-2.99	0.003	0.289	0.951
LRIT1	-0.14	0.299	-0.47	0.638	0.869	0.26	-0.505	0.614	0.484	1.561
LRIG2	0.558	0.302	1.844	0.065	1.746	0.528	1.413	0.158	0.965	3.159
LRIG1	-0.521	0.302	-1.725	0.085	0.594	0.179	-2.263	0.024	0.328	1.074
LRIF1	0.088	0.296	0.299	0.765	1.092	0.323	0.286	0.775	0.612	1.95
LRFN4	-0.024	0.297	-0.079	0.937	0.977	0.29	-0.08	0.936	0.546	1.747
LRFN3	0.224	0.297	0.755	0.45	1.252	0.372	0.676	0.499	0.699	2.241
LRCH4	-0.176	0.295	-0.596	0.551	0.839	0.248	-0.651	0.515	0.47	1.496
LRCH3	-0.242	0.297	-0.816	0.415	0.785	0.233	-0.923	0.356	0.439	1.404
LRCH1	0.257	0.298	0.862	0.389	1.293	0.385	0.76	0.447	0.721	2.319
LRBA	-0.428	0.302	-1.416	0.157	0.652	0.197	-1.767	0.077	0.36	1.179
LRAT	-0.307	0.298	-1.032	0.302	0.736	0.219	-1.208	0.227	0.411	1.318
LPXN	-0.266	0.298	-0.894	0.371	0.766	0.228	-1.025	0.306	0.427	1.374
LPP	0.187	0.296	0.633	0.527	1.206	0.357	0.577	0.564	0.675	2.155
LPO	-0.037	0.295	-0.124	0.901	0.964	0.285	-0.126	0.899	0.54	1.72

LPL	0.661	0.31	2.135	0.033	1.938	0.6	1.562	0.118	1.056	3.556
LPIN2	-0.223	0.299	-0.745	0.456	0.8	0.239	-0.835	0.404	0.445	1.438
LPIN1	0.013	0.295	0.045	0.964	1.013	0.299	0.045	0.964	0.568	1.807
LPGAT1	-0.037	0.295	-0.124	0.901	0.964	0.284	-0.127	0.899	0.541	1.718
LPCAT4	-0.095	0.296	-0.32	0.749	0.91	0.269	-0.336	0.737	0.509	1.624
LPCAT3	-1.048	0.328	-3.195	0.001	0.351	0.115	-5.647	0	0.184	0.667
LPCAT1	0.687	0.306	2.247	0.025	1.989	0.608	1.625	0.104	1.092	3.622
LPAR6	-0.47	0.3	-1.566	0.117	0.625	0.188	-2	0.046	0.347	1.125
LPAR4	-0.166	0.296	-0.559	0.576	0.847	0.251	-0.608	0.543	0.474	1.515
LPAR3	-0.376	0.3	-1.256	0.209	0.686	0.206	-1.525	0.127	0.381	1.235
LPAR2	0.37	0.3	1.234	0.217	1.448	0.435	1.031	0.302	0.804	2.608
LPAR1	-0.567	0.306	-1.854	0.064	0.567	0.173	-2.496	0.013	0.311	1.033
LPAL2	-0.246	0.296	-0.83	0.406	0.782	0.232	-0.941	0.347	0.438	1.397
LPA	-0.343	0.298	-1.151	0.25	0.71	0.211	-1.373	0.17	0.396	1.272
LOXL2	-0.2	0.296	-0.675	0.499	0.819	0.242	-0.748	0.455	0.459	1.462
LOXL1	-0.739	0.31	-2.384	0.017	0.478	0.148	-3.529	0	0.26	0.877
LOX	-0.364	0.298	-1.222	0.222	0.695	0.207	-1.474	0.141	0.388	1.246
LORICRIN	-0.139	0.296	-0.47	0.638	0.87	0.258	-0.505	0.614	0.487	1.555
LONRF3	-0.089	0.295	-0.302	0.763	0.915	0.27	-0.316	0.752	0.513	1.632
LONRF1	0.183	0.296	0.618	0.537	1.201	0.356	0.564	0.573	0.672	2.146
LONP1	0.147	0.297	0.493	0.622	1.158	0.344	0.458	0.647	0.646	2.074
LOC79999	-0.05	0.296	-0.169	0.866	0.951	0.282	-0.173	0.862	0.532	1.7
LOC730101	0.222	0.296	0.75	0.454	1.249	0.37	0.672	0.501	0.699	2.231
LOC728392	-0.217	0.296	-0.733	0.464	0.805	0.238	-0.818	0.413	0.45	1.439
LOC647070	0.504	0.302	1.667	0.095	1.656	0.501	1.31	0.19	0.915	2.995
LOC51145	0.664	0.31	2.143	0.032	1.942	0.601	1.566	0.117	1.058	3.563
LOC441666	0.172	0.296	0.582	0.56	1.188	0.351	0.535	0.593	0.666	2.12
LOC441204	-0.235	0.298	-0.789	0.43	0.791	0.235	-0.889	0.374	0.441	1.417
LOC440792	-0.202	0.297	-0.681	0.496	0.817	0.242	-0.754	0.451	0.457	1.461
LOC389906	0.352	0.3	1.174	0.24	1.422	0.426	0.99	0.322	0.79	2.559
LOC220077	-0.543	0.302	-1.796	0.073	0.581	0.176	-2.385	0.017	0.321	1.051
LOC202181	-0.356	0.298	-1.196	0.232	0.7	0.209	-1.437	0.151	0.391	1.255
LOC155060	-0.465	0.301	-1.542	0.123	0.628	0.189	-1.962	0.05	0.348	1.134
LOC103344931	-0.138	0.295	-0.468	0.64	0.871	0.257	-0.502	0.616	0.488	1.554
LOC101929550	0.16	0.3	0.534	0.593	1.174	0.352	0.493	0.622	0.652	2.114
LOC101929148	-0.644	0.306	-2.104	0.035	0.525	0.161	-2.954	0.003	0.288	0.957
LOC101929073	0.106	0.296	0.357	0.721	1.112	0.329	0.339	0.735	0.622	1.987
LOC101928278	0.616	0.31	1.989	0.047	1.851	0.573	1.485	0.138	1.009	3.397

LOC101927770	0.442	0.3	1.476	0.14	1.556	0.466	1.193	0.233	0.865	2.8
LOC101927610	-0.111	0.295	-0.375	0.708	0.895	0.264	-0.396	0.692	0.502	1.597
LOC101927550	-0.18	0.296	-0.609	0.543	0.835	0.247	-0.667	0.505	0.468	1.491
LOC101926913	0.311	0.298	1.047	0.295	1.365	0.406	0.899	0.368	0.762	2.446
LOC100996756	0.032	0.296	0.108	0.914	1.033	0.305	0.107	0.915	0.578	1.843
LOC100996325	0.198	0.297	0.667	0.505	1.219	0.362	0.605	0.545	0.681	2.182
LOC100506282	-0.034	0.295	-0.114	0.909	0.967	0.286	-0.116	0.908	0.542	1.725
LOC100505915	0.327	0.3	1.093	0.274	1.387	0.416	0.932	0.351	0.771	2.496
LOC100505498	0.091	0.296	0.307	0.759	1.095	0.324	0.293	0.769	0.613	1.955
LOC100294145	0.135	0.296	0.457	0.648	1.145	0.339	0.427	0.669	0.641	2.044
LOC100289518	0.587	0.303	1.94	0.052	1.799	0.545	1.467	0.142	0.994	3.257
LOC100289473	-0.003	0.296	-0.011	0.991	0.997	0.295	-0.011	0.991	0.558	1.779
LOC100288570	-0.119	0.295	-0.401	0.688	0.888	0.262	-0.426	0.67	0.498	1.585
LOC100287387	-0.618	0.303	-2.036	0.042	0.539	0.164	-2.818	0.005	0.297	0.977
LOC100131532	-0.203	0.296	-0.684	0.494	0.817	0.242	-0.758	0.448	0.457	1.459
LOC100130449	-0.031	0.296	-0.106	0.915	0.969	0.286	-0.108	0.914	0.543	1.73
LOC100130331	0.256	0.298	0.859	0.39	1.291	0.384	0.758	0.448	0.721	2.315
LOC100129503	-0.005	0.295	-0.015	0.988	0.995	0.294	-0.016	0.988	0.558	1.775
LNPEP	0.186	0.295	0.628	0.53	1.204	0.355	0.574	0.566	0.675	2.147
LMX1B	-0.131	0.298	-0.438	0.662	0.878	0.262	-0.467	0.64	0.489	1.575
LMTK2	0.859	0.321	2.677	0.007	2.361	0.757	1.796	0.072	1.259	4.427
LMOD1	-0.452	0.3	-1.508	0.132	0.636	0.191	-1.907	0.057	0.354	1.145
LMO7	-0.227	0.296	-0.767	0.443	0.797	0.236	-0.861	0.389	0.446	1.424
LMO4	0.463	0.302	1.529	0.126	1.588	0.48	1.224	0.221	0.878	2.874
LMO3	-0.017	0.295	-0.059	0.953	0.983	0.29	-0.059	0.953	0.551	1.753
LMO2	-0.735	0.31	-2.372	0.018	0.479	0.149	-3.503	0	0.261	0.88
LMO1	0.246	0.296	0.831	0.406	1.279	0.379	0.737	0.461	0.716	2.288
LMNB2	0.791	0.315	2.514	0.012	2.206	0.694	1.737	0.082	1.191	4.087
LMNB1	0.301	0.298	1.013	0.311	1.352	0.402	0.874	0.382	0.754	2.422
LMNA	-0.182	0.296	-0.615	0.539	0.834	0.246	-0.675	0.5	0.467	1.488
LMF2	0.14	0.297	0.47	0.638	1.15	0.341	0.439	0.661	0.643	2.056
LMF1	0.326	0.298	1.095	0.273	1.386	0.413	0.935	0.35	0.773	2.483
LMCD1	-0.566	0.306	-1.853	0.064	0.568	0.173	-2.492	0.013	0.312	1.033
LMBRD1	-0.477	0.302	-1.58	0.114	0.62	0.188	-2.024	0.043	0.343	1.122
LMBR1L	-0.379	0.297	-1.274	0.203	0.685	0.204	-1.549	0.121	0.382	1.226
LMAN2L	-0.192	0.296	-0.648	0.517	0.825	0.244	-0.714	0.475	0.462	1.475
LMAN2	0.188	0.296	0.635	0.526	1.207	0.358	0.578	0.563	0.675	2.158
LMAN1L	0.215	0.296	0.725	0.468	1.239	0.367	0.652	0.514	0.694	2.214

LMAN1	-0.633	0.31	-2.044	0.041	0.531	0.164	-2.851	0.004	0.289	0.974
LLPH	-0.041	0.295	-0.137	0.891	0.96	0.284	-0.14	0.888	0.538	1.713
LLGL2	0.335	0.298	1.127	0.26	1.399	0.416	0.958	0.338	0.781	2.506
LLGL1	0.141	0.297	0.476	0.634	1.152	0.342	0.444	0.657	0.644	2.061
LITAF	-0.325	0.3	-1.085	0.278	0.722	0.216	-1.282	0.2	0.402	1.3
LIPT1	-0.651	0.31	-2.098	0.036	0.522	0.162	-2.956	0.003	0.284	0.958
LIPG	0.073	0.297	0.246	0.805	1.076	0.32	0.238	0.812	0.601	1.927
LIPF	0.065	0.295	0.221	0.825	1.067	0.315	0.214	0.831	0.598	1.903
LIPE	-0.292	0.298	-0.979	0.328	0.747	0.222	-1.137	0.256	0.417	1.339
LIPC	-0.303	0.298	-1.018	0.309	0.739	0.22	-1.188	0.235	0.412	1.324
LIPA	-0.243	0.298	-0.817	0.414	0.784	0.233	-0.925	0.355	0.438	1.405
LINS1	0.007	0.296	0.023	0.982	1.007	0.298	0.023	0.982	0.564	1.797
LINC02809	-0.1	0.296	-0.338	0.736	0.905	0.268	-0.355	0.723	0.507	1.616
LINC02249	-0.356	0.299	-1.192	0.233	0.7	0.209	-1.432	0.152	0.39	1.258
LINC02172	-0.451	0.3	-1.504	0.133	0.637	0.191	-1.9	0.057	0.354	1.146
LINC01963	-0.552	0.306	-1.805	0.071	0.576	0.176	-2.409	0.016	0.316	1.048
LINC01949	0.262	0.301	0.871	0.384	1.299	0.391	0.766	0.443	0.721	2.343
LINC01711	-0.146	0.295	-0.495	0.62	0.864	0.255	-0.533	0.594	0.484	1.541
LINC01587	-0.224	0.297	-0.754	0.451	0.8	0.237	-0.845	0.398	0.447	1.43
LINC01565	-0.049	0.295	-0.166	0.868	0.952	0.281	-0.17	0.865	0.534	1.698
LINC01558	0.365	0.3	1.217	0.224	1.44	0.432	1.019	0.308	0.8	2.593
LINC01482	-0.284	0.3	-0.949	0.343	0.752	0.226	-1.098	0.272	0.418	1.354
LINC01361	0.258	0.298	0.865	0.387	1.294	0.385	0.763	0.445	0.722	2.318
LINC01278	-0.008	0.296	-0.027	0.978	0.992	0.293	-0.027	0.978	0.556	1.771
LINC01260	-0.147	0.295	-0.499	0.618	0.863	0.255	-0.538	0.591	0.484	1.54
LINC01140	-0.036	0.295	-0.124	0.902	0.964	0.285	-0.126	0.9	0.541	1.719
LINC01136	0.032	0.296	0.109	0.913	1.033	0.305	0.107	0.915	0.578	1.844
LINC00965	0.106	0.295	0.358	0.72	1.111	0.328	0.34	0.734	0.623	1.983
LINC00963	-0.42	0.302	-1.389	0.165	0.657	0.199	-1.727	0.084	0.363	1.188
LINC00939	-0.224	0.296	-0.757	0.449	0.799	0.237	-0.848	0.396	0.447	1.429
LINC00894	0.558	0.306	1.824	0.068	1.747	0.534	1.398	0.162	0.959	3.182
LINC00837	-0.384	0.3	-1.283	0.199	0.681	0.204	-1.565	0.118	0.379	1.225
LINC00667	-0.202	0.296	-0.681	0.496	0.817	0.242	-0.755	0.45	0.457	1.46
LINC00663	0.033	0.296	0.113	0.91	1.034	0.306	0.111	0.911	0.579	1.846
LINC00652	-0.079	0.295	-0.267	0.79	0.924	0.273	-0.277	0.781	0.518	1.649
LINC00597	0.385	0.3	1.285	0.199	1.47	0.44	1.066	0.286	0.817	2.644
LINC00588	-0.334	0.3	-1.114	0.265	0.716	0.215	-1.323	0.186	0.398	1.288
LINC00574	0.436	0.299	1.459	0.144	1.546	0.462	1.183	0.237	0.861	2.776

LINC00563	-0.192	0.296	-0.647	0.518	0.826	0.245	-0.713	0.476	0.462	1.475
LINC00474	0.438	0.302	1.448	0.148	1.549	0.468	1.173	0.241	0.857	2.802
LINC00472	-0.601	0.306	-1.966	0.049	0.548	0.168	-2.695	0.007	0.301	0.998
LINC00342	-0.156	0.296	-0.528	0.597	0.855	0.253	-0.572	0.568	0.479	1.528
LINC00339	0.104	0.295	0.352	0.725	1.109	0.328	0.334	0.738	0.622	1.98
LINC00328	0.493	0.3	1.64	0.101	1.637	0.492	1.295	0.195	0.908	2.949
LINC00312	-0.026	0.297	-0.088	0.93	0.974	0.29	-0.089	0.929	0.544	1.745
LINC00302	0.302	0.297	1.015	0.31	1.352	0.402	0.876	0.381	0.755	2.423
LINC00260	-0.268	0.298	-0.899	0.369	0.765	0.228	-1.031	0.303	0.427	1.371
LINC00216	0.306	0.298	1.028	0.304	1.358	0.404	0.886	0.376	0.758	2.433
LINC00115	0.06	0.295	0.203	0.839	1.062	0.313	0.197	0.844	0.596	1.893
LIN7C	0.356	0.297	1.2	0.23	1.428	0.424	1.01	0.313	0.798	2.554
LIN7B	-0.198	0.298	-0.664	0.507	0.821	0.244	-0.734	0.463	0.458	1.471
LIN7A	0.062	0.296	0.21	0.833	1.064	0.315	0.204	0.838	0.596	1.901
LIN37	0.221	0.297	0.742	0.458	1.247	0.371	0.666	0.506	0.696	2.234
LIN28A	0.095	0.295	0.32	0.749	1.099	0.325	0.306	0.76	0.616	1.961
LIMS2	-0.327	0.298	-1.099	0.272	0.721	0.215	-1.3	0.194	0.402	1.292
LIMK2	-0.038	0.295	-0.13	0.896	0.962	0.284	-0.133	0.894	0.54	1.716
LIMK1	-0.205	0.298	-0.689	0.491	0.815	0.242	-0.764	0.445	0.455	1.46
LIME1	0.134	0.296	0.454	0.65	1.144	0.338	0.425	0.671	0.64	2.042
LIMD2	-0.278	0.298	-0.933	0.351	0.757	0.226	-1.076	0.282	0.422	1.358
LIMD1	0.351	0.3	1.17	0.242	1.42	0.426	0.987	0.324	0.789	2.555
LIMCH1	-0.58	0.306	-1.896	0.058	0.56	0.171	-2.569	0.01	0.308	1.02
LIMA1	-0.854	0.312	-2.739	0.006	0.426	0.133	-4.326	0	0.231	0.784
LIM2	-0.031	0.296	-0.103	0.918	0.97	0.288	-0.105	0.916	0.542	1.734
LILRP2	-0.186	0.297	-0.625	0.532	0.831	0.247	-0.687	0.492	0.464	1.487
LILRB5	0.267	0.298	0.895	0.371	1.306	0.389	0.785	0.432	0.728	2.343
LILRB4	-0.261	0.298	-0.875	0.382	0.771	0.229	-1	0.318	0.43	1.381
LILRB3	0.762	0.307	2.478	0.013	2.142	0.659	1.734	0.083	1.173	3.914
LILRB2	-0.051	0.295	-0.173	0.862	0.95	0.281	-0.178	0.859	0.532	1.695
LILRB1	-0.318	0.298	-1.068	0.285	0.728	0.217	-1.257	0.209	0.406	1.304
LILRA6	-0.424	0.3	-1.413	0.158	0.654	0.196	-1.761	0.078	0.363	1.178
LILRA5	0.683	0.31	2.206	0.027	1.98	0.613	1.598	0.11	1.079	3.632
LILRA4	0.077	0.296	0.26	0.795	1.08	0.32	0.25	0.803	0.605	1.929
LILRA3	0.135	0.296	0.455	0.649	1.144	0.338	0.426	0.67	0.641	2.043
LILRA2	-0.009	0.295	-0.03	0.976	0.991	0.293	-0.03	0.976	0.556	1.768
LILRA1	0.05	0.296	0.168	0.867	1.051	0.311	0.164	0.87	0.588	1.878
LIG4	0.478	0.301	1.591	0.112	1.613	0.485	1.265	0.206	0.895	2.907

LIG3	-0.019	0.295	-0.066	0.948	0.981	0.29	-0.066	0.947	0.55	1.75
LIG1	0.535	0.302	1.771	0.077	1.708	0.516	1.371	0.17	0.944	3.089
LIFR	0.337	0.298	1.13	0.259	1.401	0.418	0.959	0.337	0.781	2.513
LIF	0.241	0.296	0.814	0.416	1.273	0.377	0.723	0.47	0.712	2.274
LIAS	-0.355	0.3	-1.185	0.236	0.701	0.21	-1.423	0.155	0.39	1.261
LHX6	-0.248	0.296	-0.838	0.402	0.78	0.231	-0.951	0.342	0.436	1.395
LHX5	0.514	0.3	1.711	0.087	1.671	0.502	1.338	0.181	0.928	3.01
LHX3	0.083	0.298	0.278	0.781	1.086	0.324	0.267	0.789	0.606	1.948
LHX2	0.139	0.295	0.469	0.639	1.149	0.339	0.438	0.661	0.644	2.049
LHX1	0.691	0.306	2.261	0.024	1.996	0.61	1.632	0.103	1.096	3.634
LHPP	-0.272	0.298	-0.915	0.36	0.762	0.227	-1.052	0.293	0.425	1.365
LHFPL6	-0.733	0.31	-2.365	0.018	0.481	0.149	-3.488	0	0.262	0.882
LHFPL2	0.04	0.296	0.134	0.894	1.04	0.308	0.131	0.896	0.582	1.859
LHCGR	0.384	0.298	1.289	0.197	1.468	0.437	1.071	0.284	0.819	2.629
LGSN	0.571	0.304	1.881	0.06	1.77	0.537	1.433	0.152	0.976	3.209
LGR5	-0.106	0.295	-0.358	0.72	0.9	0.266	-0.378	0.705	0.504	1.605
LGR4	0.412	0.303	1.36	0.174	1.509	0.457	1.115	0.265	0.834	2.731
LGMN	0.44	0.302	1.457	0.145	1.553	0.47	1.178	0.239	0.859	2.809
LGI2	0.261	0.296	0.88	0.379	1.298	0.385	0.775	0.439	0.726	2.32
LGI1	0.15	0.297	0.506	0.613	1.162	0.345	0.47	0.638	0.65	2.079
LGALSL	0.514	0.3	1.712	0.087	1.671	0.502	1.339	0.181	0.928	3.01
LGALS9	0.047	0.295	0.16	0.873	1.048	0.31	0.156	0.876	0.588	1.87
LGALS8	-0.284	0.298	-0.955	0.34	0.753	0.224	-1.105	0.269	0.42	1.349
LGALS4	-0.198	0.297	-0.667	0.505	0.82	0.244	-0.738	0.46	0.458	1.468
LGALS3BP	0.399	0.3	1.328	0.184	1.49	0.448	1.095	0.274	0.827	2.685
LGALS3	0.047	0.295	0.16	0.872	1.049	0.309	0.157	0.875	0.588	1.87
LGALS2	-0.16	0.296	-0.541	0.589	0.852	0.252	-0.586	0.558	0.477	1.523
LGALS14	0.169	0.296	0.572	0.568	1.184	0.351	0.526	0.599	0.663	2.117
LGALS13	-0.304	0.298	-1.02	0.308	0.738	0.22	-1.192	0.233	0.412	1.323
LGALS1	0.066	0.295	0.224	0.823	1.068	0.315	0.217	0.828	0.599	1.904
LFNG	0.151	0.296	0.51	0.61	1.163	0.345	0.474	0.636	0.651	2.079
LETMD1	-0.453	0.3	-1.51	0.131	0.636	0.191	-1.911	0.056	0.353	1.144
LETM1	0.369	0.298	1.237	0.216	1.446	0.431	1.034	0.301	0.806	2.593
LEPROTL1	-0.575	0.306	-1.883	0.06	0.563	0.172	-2.545	0.011	0.309	1.024
LEPROT	0.142	0.297	0.479	0.632	1.153	0.343	0.447	0.655	0.644	2.065
LEP	0.768	0.315	2.441	0.015	2.156	0.678	1.704	0.088	1.163	3.994
LENEP	-0.132	0.296	-0.447	0.655	0.876	0.259	-0.478	0.633	0.49	1.565
LEMD3	0.348	0.298	1.169	0.242	1.416	0.421	0.987	0.323	0.79	2.537

LEFTY1	0.421	0.3	1.406	0.16	1.524	0.457	1.147	0.251	0.847	2.741
LEF1	-0.872	0.315	-2.77	0.006	0.418	0.132	-4.421	0	0.226	0.775
LECT2	0.178	0.297	0.602	0.547	1.195	0.354	0.551	0.582	0.668	2.137
LDOC1	0.654	0.31	2.109	0.035	1.923	0.596	1.548	0.122	1.047	3.529
LDLRAP1	-0.422	0.303	-1.393	0.164	0.656	0.199	-1.733	0.083	0.362	1.187
LDLRAD4	-0.086	0.297	-0.288	0.773	0.918	0.273	-0.301	0.763	0.513	1.644
LDLR	-0.17	0.296	-0.575	0.565	0.843	0.25	-0.627	0.531	0.472	1.507
LDHC	0.046	0.295	0.156	0.876	1.047	0.309	0.152	0.879	0.587	1.867
LDHB	0.044	0.295	0.149	0.882	1.045	0.308	0.145	0.884	0.586	1.863
LDHAL6B	-0.082	0.296	-0.277	0.782	0.921	0.272	-0.289	0.773	0.516	1.645
LDHA	0.167	0.295	0.565	0.572	1.182	0.349	0.52	0.603	0.662	2.108
LDB3	0.168	0.297	0.567	0.571	1.183	0.351	0.522	0.602	0.661	2.117
LDB2	-0.828	0.315	-2.628	0.009	0.437	0.138	-4.088	0	0.236	0.81
LDB1	0.361	0.3	1.204	0.228	1.435	0.43	1.011	0.312	0.797	2.582
LDAH	-0.272	0.298	-0.913	0.361	0.762	0.227	-1.049	0.294	0.425	1.366
LCT	0.654	0.31	2.111	0.035	1.923	0.596	1.55	0.121	1.048	3.529
LCP2	-0.577	0.306	-1.886	0.059	0.562	0.172	-2.552	0.011	0.308	1.023
LCP1	-0.227	0.297	-0.766	0.444	0.797	0.236	-0.86	0.39	0.446	1.425
LCN2	-0.063	0.296	-0.212	0.832	0.939	0.278	-0.219	0.827	0.525	1.679
LCN1	-0.016	0.295	-0.053	0.957	0.984	0.291	-0.054	0.957	0.552	1.756
LCMT2	-0.198	0.296	-0.669	0.504	0.82	0.243	-0.74	0.46	0.459	1.466
LCMT1	0.1	0.296	0.338	0.735	1.105	0.327	0.322	0.748	0.619	1.974
LCK	-0.021	0.296	-0.07	0.944	0.98	0.29	-0.071	0.944	0.549	1.749
LCE2B	-0.239	0.297	-0.806	0.42	0.787	0.234	-0.91	0.363	0.44	1.409
LCAT	-0.684	0.311	-2.202	0.028	0.505	0.157	-3.16	0.002	0.275	0.928
LBX1	0.333	0.3	1.11	0.267	1.395	0.418	0.944	0.345	0.775	2.511
LBR	-0.218	0.297	-0.734	0.463	0.804	0.239	-0.82	0.412	0.45	1.438
LBP	0.278	0.301	0.923	0.356	1.32	0.397	0.806	0.42	0.732	2.381
LBH	-0.88	0.312	-2.818	0.005	0.415	0.13	-4.516	0	0.225	0.765
LAX1	-0.43	0.299	-1.439	0.15	0.65	0.194	-1.797	0.072	0.362	1.169
LATS1	0.408	0.3	1.362	0.173	1.504	0.451	1.118	0.263	0.836	2.705
LAT2	-0.781	0.315	-2.482	0.013	0.458	0.144	-3.762	0	0.247	0.848
LAT	-0.253	0.296	-0.855	0.393	0.776	0.23	-0.973	0.33	0.434	1.387
LASP1	0.108	0.295	0.364	0.716	1.114	0.329	0.345	0.73	0.624	1.987
LAS1L	0.684	0.306	2.236	0.025	1.981	0.606	1.62	0.105	1.088	3.608
LARS2	0.765	0.307	2.494	0.013	2.149	0.66	1.743	0.081	1.178	3.922
LARS1	0.128	0.298	0.429	0.668	1.136	0.339	0.403	0.687	0.634	2.038
LARP7	0.215	0.297	0.725	0.468	1.24	0.368	0.653	0.514	0.693	2.219

LARP6	-0.118	0.295	-0.401	0.689	0.888	0.262	-0.425	0.671	0.498	1.585
LARP4B	1.115	0.336	3.317	0.001	3.049	1.025	2	0.046	1.578	5.892
LARP4	-0.423	0.302	-1.399	0.162	0.655	0.198	-1.741	0.082	0.362	1.185
LARP1	0.353	0.3	1.174	0.24	1.423	0.427	0.989	0.323	0.79	2.563
LARGE1	0.202	0.296	0.682	0.495	1.224	0.363	0.618	0.537	0.685	2.187
LAPTM5	0.011	0.295	0.039	0.969	1.011	0.299	0.039	0.969	0.567	1.804
LAPTM4B	1.027	0.328	3.134	0.002	2.792	0.915	1.959	0.05	1.469	5.307
LAPTM4A	0.098	0.296	0.331	0.741	1.103	0.326	0.315	0.753	0.618	1.968
LAP3	0.498	0.303	1.646	0.1	1.646	0.498	1.297	0.195	0.909	2.978
LANCL2	0.515	0.3	1.717	0.086	1.674	0.502	1.342	0.18	0.93	3.014
LANCL1	-0.2	0.298	-0.673	0.501	0.818	0.244	-0.745	0.456	0.457	1.467
LAMTOR5	0.585	0.303	1.931	0.053	1.795	0.544	1.462	0.144	0.991	3.25
LAMTOR3	0.037	0.297	0.125	0.901	1.038	0.308	0.122	0.903	0.58	1.857
LAMTOR2	0.374	0.299	1.25	0.211	1.453	0.434	1.043	0.297	0.809	2.609
LAMP5	-1.048	0.321	-3.263	0.001	0.351	0.113	-5.765	0	0.187	0.658
LAMP3	0.362	0.3	1.207	0.227	1.436	0.43	1.013	0.311	0.798	2.584
LAMP2	0.493	0.3	1.645	0.1	1.638	0.491	1.298	0.194	0.91	2.948
LAMP1	0.782	0.31	2.523	0.012	2.187	0.678	1.75	0.08	1.191	4.015
LAMC3	0.404	0.3	1.348	0.178	1.498	0.449	1.109	0.267	0.832	2.696
LAMC2	-0.006	0.295	-0.022	0.983	0.994	0.293	-0.022	0.983	0.557	1.772
LAMC1	-0.727	0.307	-2.37	0.018	0.483	0.148	-3.484	0	0.265	0.882
LAMB4	0.596	0.306	1.947	0.051	1.814	0.555	1.467	0.142	0.996	3.304
LAMB3	-0.389	0.3	-1.299	0.194	0.678	0.203	-1.588	0.112	0.377	1.219
LAMB2	-0.487	0.302	-1.613	0.107	0.614	0.186	-2.079	0.038	0.34	1.111
LAMB1	-0.414	0.298	-1.388	0.165	0.661	0.197	-1.719	0.086	0.369	1.186
LAMA5	0.071	0.297	0.239	0.811	1.074	0.319	0.23	0.818	0.599	1.923
LAMA4	-0.226	0.296	-0.763	0.446	0.798	0.236	-0.856	0.392	0.447	1.425
LAMA3	-0.452	0.302	-1.494	0.135	0.637	0.193	-1.888	0.059	0.352	1.152
LAMA2	-0.806	0.31	-2.598	0.009	0.447	0.139	-3.994	0	0.243	0.82
LAMA1	0.265	0.296	0.895	0.371	1.304	0.386	0.786	0.432	0.729	2.331
LALBA	-0.686	0.31	-2.216	0.027	0.503	0.156	-3.184	0.001	0.274	0.924
LAIR2	0	0.296	-0.001	0.999	1	0.295	-0.001	0.999	0.56	1.784
LAIR1	-0.053	0.296	-0.181	0.857	0.948	0.281	-0.186	0.853	0.531	1.694
LAGE3	0.62	0.306	2.03	0.042	1.86	0.568	1.513	0.13	1.022	3.385
LAG3	0.091	0.297	0.306	0.76	1.095	0.326	0.292	0.77	0.611	1.962
LAD1	0.441	0.306	1.438	0.15	1.554	0.476	1.163	0.245	0.852	2.833
LACTB2	-0.073	0.296	-0.246	0.806	0.93	0.275	-0.255	0.799	0.521	1.659
L3MBTL1	0.524	0.302	1.731	0.083	1.688	0.511	1.348	0.178	0.933	3.054

L2HGDH	0.173	0.297	0.584	0.56	1.189	0.353	0.536	0.592	0.665	2.127
L1TD1	0.238	0.296	0.805	0.421	1.269	0.376	0.716	0.474	0.71	2.268
L1CAM	0.142	0.296	0.479	0.632	1.153	0.342	0.447	0.655	0.645	2.06
KYNU	-0.08	0.296	-0.27	0.788	0.923	0.273	-0.281	0.779	0.517	1.65
KYAT3	-0.682	0.316	-2.161	0.031	0.506	0.16	-3.099	0.002	0.272	0.938
KYAT1	-0.312	0.298	-1.047	0.295	0.732	0.218	-1.229	0.219	0.408	1.313
KXD1	-0.139	0.296	-0.468	0.64	0.87	0.258	-0.502	0.616	0.487	1.556
KTN1	-0.286	0.3	-0.954	0.34	0.751	0.225	-1.105	0.269	0.417	1.352
KSR1	-0.224	0.296	-0.755	0.45	0.8	0.237	-0.847	0.397	0.447	1.429
KRTAP9-9	0.269	0.297	0.908	0.364	1.309	0.388	0.796	0.426	0.732	2.341
KRTAP5-9	0.098	0.296	0.331	0.741	1.103	0.327	0.315	0.753	0.617	1.972
KRTAP5-8	-0.742	0.31	-2.391	0.017	0.476	0.148	-3.545	0	0.259	0.875
KRTAP4-7	-0.098	0.295	-0.33	0.741	0.907	0.268	-0.347	0.729	0.508	1.618
KRTAP1-3	0.217	0.296	0.731	0.465	1.242	0.368	0.657	0.511	0.695	2.22
KRTAP1-1	0.001	0.295	0.005	0.996	1.001	0.295	0.005	0.996	0.562	1.785
KRT9	0.447	0.303	1.479	0.139	1.564	0.473	1.192	0.233	0.864	2.83
KRT8P12	0.666	0.306	2.177	0.029	1.946	0.595	1.59	0.112	1.069	3.543
KRT86	0.058	0.295	0.198	0.843	1.06	0.313	0.192	0.848	0.594	1.891
KRT85	-0.311	0.298	-1.045	0.296	0.733	0.218	-1.226	0.22	0.409	1.313
KRT84	-0.175	0.296	-0.591	0.554	0.84	0.248	-0.646	0.518	0.47	1.499
KRT83	0.176	0.295	0.595	0.552	1.192	0.352	0.546	0.585	0.668	2.127
KRT81	0.285	0.297	0.961	0.336	1.33	0.395	0.836	0.403	0.743	2.381
KRT8	0.417	0.3	1.392	0.164	1.518	0.455	1.138	0.255	0.843	2.731
KRT76	0.014	0.295	0.048	0.962	1.014	0.299	0.048	0.962	0.569	1.808
KRT75	0.222	0.296	0.749	0.454	1.248	0.37	0.672	0.502	0.699	2.231
KRT7	0.05	0.297	0.168	0.867	1.051	0.312	0.164	0.87	0.588	1.88
KRT6B	-0.112	0.295	-0.381	0.703	0.894	0.264	-0.403	0.687	0.501	1.594
KRT6A	-0.237	0.297	-0.799	0.425	0.789	0.234	-0.901	0.367	0.441	1.412
KRT5	0.012	0.296	0.04	0.968	1.012	0.299	0.04	0.968	0.567	1.806
KRT4	0.329	0.3	1.097	0.272	1.389	0.416	0.935	0.35	0.772	2.5
KRT38	-0.356	0.298	-1.195	0.232	0.7	0.209	-1.435	0.151	0.39	1.256
KRT37	0.025	0.296	0.085	0.932	1.026	0.303	0.084	0.933	0.574	1.831
KRT36	-0.327	0.299	-1.097	0.273	0.721	0.215	-1.298	0.194	0.401	1.294
KRT35	0.384	0.3	1.282	0.2	1.468	0.44	1.065	0.287	0.816	2.642
KRT33B	0.117	0.295	0.396	0.692	1.124	0.332	0.373	0.709	0.63	2.005
KRT33A	-0.152	0.296	-0.514	0.607	0.859	0.254	-0.555	0.579	0.481	1.534
KRT32	-0.076	0.296	-0.257	0.798	0.927	0.274	-0.267	0.79	0.519	1.655
KRT31	0.087	0.295	0.296	0.767	1.091	0.322	0.284	0.777	0.612	1.946

KRT3	0.524	0.302	1.734	0.083	1.689	0.511	1.35	0.177	0.934	3.055
KRT24	0.204	0.296	0.687	0.492	1.226	0.363	0.622	0.534	0.686	2.191
KRT23	0.42	0.3	1.402	0.161	1.522	0.456	1.145	0.252	0.846	2.738
KRT20	0.402	0.3	1.341	0.18	1.495	0.448	1.105	0.269	0.831	2.69
KRT2	-0.279	0.299	-0.934	0.35	0.756	0.226	-1.078	0.281	0.421	1.359
KRT19P2	0.334	0.298	1.12	0.263	1.397	0.417	0.952	0.341	0.778	2.507
KRT19	0.275	0.296	0.926	0.354	1.316	0.39	0.81	0.418	0.736	2.352
KRT18	0.289	0.296	0.975	0.33	1.335	0.396	0.847	0.397	0.747	2.386
KRT16	0.074	0.295	0.251	0.802	1.077	0.318	0.242	0.809	0.604	1.921
KRT15	-0.042	0.295	-0.144	0.886	0.958	0.283	-0.147	0.883	0.537	1.71
KRT14	0.089	0.295	0.301	0.763	1.093	0.323	0.288	0.773	0.613	1.95
KRT13	0.116	0.295	0.393	0.694	1.123	0.332	0.371	0.711	0.63	2.003
KRT12	0.154	0.296	0.521	0.603	1.166	0.345	0.483	0.629	0.654	2.082
KRT10	0.485	0.3	1.615	0.106	1.623	0.487	1.28	0.201	0.902	2.924
KRT1	0.219	0.296	0.738	0.46	1.244	0.369	0.663	0.507	0.696	2.224
KRR1	0.322	0.298	1.08	0.28	1.38	0.412	0.924	0.356	0.769	2.477
KRIT1	0.033	0.295	0.112	0.911	1.034	0.305	0.11	0.912	0.58	1.843
KRI1	-0.212	0.296	-0.715	0.475	0.809	0.24	-0.796	0.426	0.453	1.446
KREMEN2	0.089	0.295	0.301	0.763	1.093	0.323	0.288	0.773	0.613	1.949
KRCC1	-0.01	0.296	-0.034	0.973	0.99	0.293	-0.035	0.972	0.554	1.768
KRBOX4	0.188	0.297	0.634	0.526	1.207	0.358	0.578	0.564	0.675	2.159
KRAS	0.306	0.296	1.034	0.301	1.358	0.403	0.89	0.373	0.76	2.428
KPTN	0.65	0.311	2.088	0.037	1.916	0.596	1.535	0.125	1.041	3.526
KPNB1	0.54	0.302	1.787	0.074	1.716	0.519	1.381	0.167	0.949	3.104
KPNA6	-0.146	0.296	-0.494	0.621	0.864	0.256	-0.533	0.594	0.483	1.544
KPNA5	-0.259	0.298	-0.87	0.384	0.772	0.23	-0.993	0.321	0.43	1.384
KPNA4	0.38	0.3	1.267	0.205	1.462	0.438	1.054	0.292	0.812	2.631
KPNA3	0.142	0.296	0.48	0.631	1.152	0.341	0.447	0.655	0.645	2.058
KPNA2	0.349	0.298	1.171	0.242	1.417	0.422	0.988	0.323	0.79	2.541
KPNA1	0.581	0.302	1.922	0.055	1.789	0.541	1.458	0.145	0.989	3.236
KNTC1	0.25	0.298	0.839	0.402	1.284	0.383	0.742	0.458	0.716	2.304
KNOP1	-0.017	0.295	-0.058	0.954	0.983	0.29	-0.059	0.953	0.551	1.753
KNL1	-0.276	0.296	-0.931	0.352	0.759	0.225	-1.073	0.283	0.424	1.356
KNG1	-0.243	0.297	-0.818	0.413	0.784	0.233	-0.926	0.354	0.438	1.404
KMT5B	-0.015	0.295	-0.052	0.959	0.985	0.291	-0.052	0.958	0.552	1.756
KMT5A	-0.399	0.303	-1.317	0.188	0.671	0.203	-1.618	0.106	0.371	1.215
KMT2D	-0.29	0.297	-0.977	0.329	0.748	0.222	-1.133	0.257	0.418	1.339
KMT2B	0.161	0.296	0.545	0.586	1.175	0.348	0.503	0.615	0.658	2.1

KMT2A	-0.381	0.298	-1.278	0.201	0.683	0.204	-1.556	0.12	0.381	1.225
KMO	-0.417	0.3	-1.391	0.164	0.659	0.198	-1.726	0.084	0.366	1.186
KLRG1	0.256	0.301	0.85	0.395	1.292	0.389	0.75	0.453	0.716	2.33
KLRF1	-0.553	0.303	-1.826	0.068	0.575	0.174	-2.438	0.015	0.318	1.041
KLRD1	-0.759	0.307	-2.474	0.013	0.468	0.144	-3.705	0	0.256	0.854
KLRC4	-0.266	0.298	-0.892	0.373	0.767	0.228	-1.021	0.307	0.428	1.375
KLRC3	-0.851	0.315	-2.702	0.007	0.427	0.134	-4.261	0	0.23	0.792
KLRB1	-1.66	0.372	-4.456	0	0.19	0.071	-11.431	0	0.092	0.395
KLRA1P	-0.777	0.315	-2.469	0.014	0.46	0.145	-3.735	0	0.248	0.852
KLKB1	0.042	0.295	0.143	0.886	1.043	0.308	0.14	0.889	0.585	1.861
KLK8	-0.244	0.296	-0.824	0.41	0.783	0.232	-0.933	0.351	0.438	1.4
KLK7	0.252	0.298	0.846	0.397	1.286	0.383	0.748	0.454	0.718	2.305
KLK6	0.18	0.296	0.608	0.543	1.197	0.354	0.556	0.578	0.67	2.139
KLK5	0.33	0.3	1.1	0.271	1.39	0.417	0.937	0.349	0.773	2.501
KLK3	-0.512	0.302	-1.693	0.09	0.599	0.181	-2.211	0.027	0.331	1.084
KLK2	0.132	0.295	0.448	0.654	1.141	0.337	0.42	0.675	0.64	2.037
KLK15	0.547	0.302	1.81	0.07	1.729	0.523	1.394	0.163	0.956	3.127
KLK14	-0.395	0.3	-1.314	0.189	0.674	0.202	-1.611	0.107	0.374	1.214
KLK13	-0.184	0.296	-0.622	0.534	0.832	0.247	-0.683	0.495	0.465	1.487
KLK12	0.015	0.296	0.05	0.96	1.015	0.3	0.049	0.961	0.568	1.811
KLK11	0.013	0.297	0.044	0.965	1.013	0.301	0.044	0.965	0.566	1.813
KLK10	-0.124	0.296	-0.42	0.675	0.883	0.262	-0.447	0.655	0.494	1.578
KLK1	0.089	0.297	0.299	0.765	1.093	0.324	0.286	0.775	0.611	1.955
KLHL9	-0.128	0.295	-0.434	0.664	0.88	0.26	-0.463	0.643	0.493	1.569
KLHL7	0.679	0.306	2.221	0.026	1.972	0.603	1.612	0.107	1.083	3.589
KLHL5	0.335	0.298	1.123	0.261	1.397	0.416	0.955	0.34	0.779	2.505
KLHL41	-0.147	0.296	-0.496	0.62	0.863	0.256	-0.535	0.593	0.483	1.543
KLHL4	0.169	0.295	0.571	0.568	1.184	0.35	0.526	0.599	0.664	2.112
KLHL36	0.566	0.306	1.853	0.064	1.762	0.538	1.415	0.157	0.968	3.206
KLHL35	0.041	0.298	0.136	0.892	1.042	0.311	0.134	0.894	0.58	1.869
KLHL3	-0.557	0.302	-1.842	0.065	0.573	0.173	-2.465	0.014	0.317	1.036
KLHL29	0.367	0.298	1.234	0.217	1.444	0.43	1.032	0.302	0.806	2.587
KLHL28	0.209	0.296	0.706	0.48	1.232	0.365	0.637	0.524	0.69	2.203
KLHL26	-0.155	0.299	-0.518	0.605	0.857	0.256	-0.56	0.576	0.477	1.539
KLHL25	0.392	0.298	1.317	0.188	1.48	0.441	1.089	0.276	0.826	2.654
KLHL24	1.028	0.321	3.198	0.001	2.796	0.899	1.998	0.046	1.489	5.25
KLHL22	0.348	0.298	1.169	0.242	1.416	0.422	0.987	0.323	0.79	2.538
KLHL21	-0.21	0.301	-0.698	0.485	0.811	0.244	-0.776	0.438	0.45	1.462

KLHL20	0.548	0.311	1.762	0.078	1.73	0.539	1.356	0.175	0.94	3.185
KLHL2	-0.141	0.295	-0.476	0.634	0.869	0.257	-0.511	0.609	0.487	1.55
KLHL18	-0.124	0.295	-0.421	0.674	0.883	0.261	-0.449	0.654	0.495	1.576
KLHL12	-0.391	0.3	-1.306	0.191	0.676	0.203	-1.599	0.11	0.376	1.216
KLHL11	0.1	0.296	0.338	0.735	1.105	0.327	0.322	0.748	0.619	1.974
KLHDC8A	0.573	0.306	1.875	0.061	1.774	0.542	1.427	0.153	0.974	3.229
KLHDC4	0.306	0.297	1.029	0.303	1.357	0.403	0.887	0.375	0.759	2.428
KLHDC3	0.056	0.296	0.188	0.851	1.057	0.313	0.183	0.855	0.592	1.888
KLHDC2	-0.504	0.302	-1.666	0.096	0.604	0.183	-2.166	0.03	0.334	1.093
KLHDC10	0.047	0.295	0.159	0.873	1.048	0.309	0.156	0.876	0.588	1.869
KLF9	-0.014	0.295	-0.048	0.962	0.986	0.291	-0.048	0.962	0.553	1.758
KLF8	0.189	0.296	0.639	0.523	1.208	0.358	0.582	0.56	0.676	2.159
KLF7	-0.008	0.296	-0.025	0.98	0.993	0.294	-0.025	0.98	0.555	1.774
KLF6	0.316	0.297	1.063	0.288	1.371	0.408	0.911	0.362	0.766	2.456
KLF5	0.361	0.3	1.206	0.228	1.435	0.43	1.012	0.311	0.798	2.582
KLF4	-0.509	0.302	-1.683	0.092	0.601	0.182	-2.193	0.028	0.333	1.087
KLF3-AS1	-0.258	0.296	-0.874	0.382	0.772	0.228	-0.997	0.319	0.433	1.379
KLF3	-0.646	0.306	-2.11	0.035	0.524	0.16	-2.966	0.003	0.288	0.955
KLF2	-0.136	0.297	-0.457	0.647	0.873	0.259	-0.49	0.624	0.488	1.562
KLF15	0.448	0.302	1.483	0.138	1.566	0.473	1.195	0.232	0.866	2.832
KLF13	-0.104	0.3	-0.348	0.728	0.901	0.27	-0.367	0.714	0.501	1.621
KLF12	0.124	0.297	0.417	0.676	1.132	0.336	0.393	0.695	0.632	2.026
KLF11	0.035	0.295	0.118	0.906	1.035	0.306	0.116	0.908	0.581	1.846
KLF10	-0.337	0.298	-1.132	0.258	0.714	0.213	-1.346	0.178	0.398	1.28
KLF1	0.75	0.31	2.421	0.015	2.118	0.657	1.703	0.089	1.154	3.888
KLC2	-0.043	0.296	-0.144	0.885	0.958	0.284	-0.148	0.883	0.537	1.711
KLC1	0.146	0.296	0.492	0.622	1.157	0.343	0.458	0.647	0.647	2.068
KL	0.05	0.297	0.168	0.867	1.051	0.312	0.163	0.87	0.588	1.879
KIZ	-0.549	0.306	-1.795	0.073	0.578	0.177	-2.391	0.017	0.317	1.052
KITLG	-0.359	0.3	-1.197	0.231	0.699	0.209	-1.44	0.15	0.388	1.257
KIT	-0.192	0.296	-0.647	0.517	0.825	0.245	-0.714	0.475	0.462	1.475
KISS1	0.632	0.307	2.059	0.039	1.882	0.578	1.526	0.127	1.031	3.436
KIRREL1	0.309	0.297	1.039	0.299	1.362	0.405	0.894	0.371	0.761	2.438
KIR3DX1	0.114	0.296	0.384	0.701	1.12	0.332	0.363	0.717	0.627	2.003
KIR3DS1	0.099	0.295	0.334	0.739	1.104	0.326	0.318	0.751	0.619	1.969
KIR3DL3	0.224	0.296	0.756	0.449	1.251	0.371	0.678	0.498	0.7	2.237
KIR3DL1	-0.567	0.306	-1.856	0.063	0.567	0.173	-2.498	0.013	0.311	1.032
KIR2DS5	-0.349	0.3	-1.165	0.244	0.705	0.211	-1.394	0.163	0.392	1.269

KIR2DS4	-0.467	0.3	-1.557	0.119	0.627	0.188	-1.985	0.047	0.348	1.128
KIR2DS3	-0.196	0.296	-0.661	0.509	0.822	0.243	-0.73	0.465	0.46	1.469
KIR2DS2	0.384	0.3	1.279	0.201	1.467	0.44	1.062	0.288	0.815	2.642
KIR2DS1	0.473	0.302	1.564	0.118	1.605	0.485	1.246	0.213	0.887	2.903
KIR2DL5A	0.096	0.297	0.324	0.746	1.101	0.327	0.309	0.758	0.615	1.969
KIR2DL4	-0.164	0.296	-0.554	0.579	0.849	0.251	-0.603	0.547	0.475	1.517
KIR2DL3	0.27	0.298	0.907	0.364	1.31	0.39	0.795	0.427	0.731	2.348
KIR2DL2	-0.233	0.297	-0.787	0.432	0.792	0.235	-0.886	0.376	0.442	1.417
KIR2DL1	0.254	0.298	0.852	0.394	1.289	0.384	0.753	0.452	0.719	2.309
KIN	1.2	0.336	3.568	0	3.319	1.116	2.078	0.038	1.717	6.416
KIFC3	-0.5	0.301	-1.663	0.096	0.607	0.182	-2.157	0.031	0.337	1.094
KIFC1	0.875	0.315	2.778	0.005	2.398	0.755	1.852	0.064	1.294	4.444
KIFBP	-0.139	0.299	-0.465	0.642	0.87	0.26	-0.498	0.618	0.484	1.564
KIFAP3	-0.388	0.303	-1.28	0.2	0.679	0.206	-1.564	0.118	0.375	1.229
KIF5C	-0.446	0.3	-1.49	0.136	0.64	0.192	-1.877	0.06	0.356	1.151
KIF5B	-0.02	0.295	-0.066	0.947	0.981	0.289	-0.067	0.947	0.55	1.748
KIF5A	0.102	0.298	0.341	0.733	1.107	0.33	0.324	0.746	0.618	1.984
KIF4A	0.342	0.298	1.149	0.251	1.408	0.42	0.973	0.331	0.785	2.526
KIF3C	0.679	0.306	2.22	0.026	1.971	0.602	1.612	0.107	1.083	3.588
KIF3B	-0.349	0.298	-1.172	0.241	0.705	0.21	-1.402	0.161	0.393	1.265
KIF3A	0.377	0.299	1.264	0.206	1.459	0.435	1.053	0.292	0.813	2.618
KIF2C	0.516	0.303	1.705	0.088	1.675	0.507	1.332	0.183	0.926	3.03
KIF2A	-0.136	0.296	-0.458	0.647	0.873	0.259	-0.491	0.624	0.489	1.56
KIF26B	-0.512	0.306	-1.672	0.094	0.599	0.183	-2.184	0.029	0.329	1.092
KIF25-AS1	0.686	0.304	2.258	0.024	1.985	0.603	1.634	0.102	1.095	3.6
KIF25	-0.576	0.31	-1.859	0.063	0.562	0.174	-2.513	0.012	0.306	1.032
KIF24	-0.557	0.301	-1.849	0.064	0.573	0.172	-2.474	0.013	0.318	1.034
KIF23	0.456	0.298	1.53	0.126	1.578	0.47	1.228	0.219	0.88	2.83
KIF22	0.587	0.304	1.933	0.053	1.798	0.546	1.462	0.144	0.992	3.261
KIF21B	-0.288	0.298	-0.966	0.334	0.75	0.223	-1.12	0.263	0.418	1.345
KIF20B	0.288	0.298	0.967	0.334	1.333	0.397	0.84	0.401	0.744	2.389
KIF20A	0.883	0.315	2.805	0.005	2.418	0.761	1.863	0.062	1.305	4.481
KIF1C	0.43	0.3	1.432	0.152	1.537	0.462	1.164	0.245	0.853	2.769
KIF1B	-0.007	0.295	-0.024	0.981	0.993	0.293	-0.024	0.981	0.556	1.771
KIF1A	0.355	0.298	1.192	0.233	1.426	0.425	1.003	0.316	0.796	2.557
KIF18B	0.969	0.321	3.021	0.003	2.635	0.845	1.935	0.053	1.406	4.942
KIF18A	0.885	0.321	2.757	0.006	2.422	0.777	1.83	0.067	1.292	4.543
KIF17	0.074	0.296	0.252	0.801	1.077	0.318	0.243	0.808	0.604	1.923

KIF16B	-0.081	0.297	-0.272	0.785	0.922	0.274	-0.284	0.777	0.515	1.651
KIF15	0.595	0.306	1.945	0.052	1.813	0.554	1.466	0.143	0.995	3.301
KIF14	0.578	0.306	1.888	0.059	1.782	0.545	1.434	0.152	0.978	3.247
KIF13B	-0.609	0.31	-1.963	0.05	0.544	0.169	-2.703	0.007	0.296	0.999
KIF13A	-0.452	0.303	-1.494	0.135	0.636	0.193	-1.889	0.059	0.351	1.152
KIF11	0.453	0.3	1.513	0.13	1.573	0.471	1.216	0.224	0.875	2.831
KIDINS220	-0.157	0.296	-0.53	0.596	0.855	0.253	-0.574	0.566	0.478	1.527
KIAA1614	0.058	0.296	0.198	0.843	1.06	0.314	0.192	0.848	0.594	1.893
KIAA1549L	0.367	0.3	1.223	0.221	1.443	0.433	1.024	0.306	0.802	2.598
KIAA1109	-0.087	0.295	-0.293	0.77	0.917	0.271	-0.306	0.76	0.514	1.636
KIAA0930	-0.473	0.302	-1.562	0.118	0.623	0.189	-1.997	0.046	0.345	1.128
KIAA0895	0.615	0.304	2.021	0.043	1.85	0.563	1.51	0.131	1.019	3.357
KIAA0754	-0.16	0.295	-0.54	0.589	0.852	0.252	-0.586	0.558	0.478	1.521
KIAA0753	-0.184	0.296	-0.619	0.536	0.832	0.247	-0.68	0.497	0.466	1.488
KIAA0586	0.116	0.295	0.394	0.694	1.123	0.332	0.372	0.71	0.63	2.004
KIAA0513	0.016	0.296	0.054	0.957	1.016	0.301	0.054	0.957	0.568	1.817
KIAA0408	0.711	0.31	2.293	0.022	2.035	0.631	1.641	0.101	1.109	3.736
KIAA0319L	-0.487	0.306	-1.59	0.112	0.614	0.188	-2.049	0.04	0.337	1.12
KIAA0319	-0.112	0.296	-0.376	0.707	0.894	0.265	-0.398	0.69	0.501	1.598
KIAA0232	0.178	0.297	0.6	0.548	1.195	0.355	0.55	0.583	0.668	2.14
KIAA0100	-0.094	0.299	-0.315	0.753	0.91	0.272	-0.33	0.741	0.507	1.635
KIAA0087	-0.444	0.302	-1.468	0.142	0.642	0.194	-1.848	0.065	0.355	1.16
KIAA0040	-0.918	0.321	-2.863	0.004	0.399	0.128	-4.691	0	0.213	0.749
KHSRP	-0.411	0.299	-1.371	0.17	0.663	0.199	-1.696	0.09	0.369	1.193
KHNYN	0.267	0.298	0.897	0.37	1.306	0.389	0.787	0.431	0.729	2.339
KHK	0.215	0.296	0.727	0.467	1.24	0.367	0.654	0.513	0.694	2.216
KHDRBS3	0.214	0.298	0.719	0.472	1.239	0.369	0.647	0.517	0.691	2.222
KHDRBS2	0.501	0.302	1.657	0.098	1.65	0.499	1.303	0.193	0.912	2.984
KHDRBS1	0.248	0.296	0.836	0.403	1.281	0.379	0.741	0.459	0.717	2.289
KHDC4	-0.009	0.296	-0.029	0.977	0.991	0.294	-0.03	0.976	0.555	1.771
KERA	-0.336	0.298	-1.127	0.26	0.715	0.213	-1.339	0.18	0.399	1.281
KEL	0.301	0.296	1.014	0.31	1.351	0.401	0.876	0.381	0.756	2.415
KEAP1	-0.258	0.3	-0.859	0.39	0.773	0.232	-0.98	0.327	0.429	1.391
KDSR	0.062	0.295	0.209	0.834	1.064	0.314	0.203	0.839	0.597	1.896
KDR	-0.158	0.296	-0.532	0.595	0.854	0.253	-0.576	0.565	0.478	1.527
KDM8	-0.42	0.302	-1.388	0.165	0.657	0.199	-1.725	0.084	0.363	1.189
KDM7A	0.261	0.3	0.868	0.385	1.298	0.389	0.764	0.445	0.721	2.336
KDM6B	-0.136	0.296	-0.46	0.646	0.873	0.258	-0.492	0.622	0.489	1.558

KDM6A	-0.035	0.295	-0.12	0.904	0.965	0.285	-0.122	0.903	0.541	1.721
KDM5D	-0.123	0.296	-0.414	0.679	0.885	0.262	-0.441	0.659	0.495	1.58
KDM5C	0.349	0.3	1.163	0.245	1.417	0.425	0.982	0.326	0.788	2.55
KDM5B	-0.368	0.3	-1.227	0.22	0.692	0.207	-1.484	0.138	0.385	1.245
KDM5A	0.506	0.3	1.685	0.092	1.658	0.498	1.322	0.186	0.921	2.988
KDM4D	-0.092	0.295	-0.31	0.756	0.912	0.269	-0.325	0.745	0.512	1.627
KDM4C	-0.041	0.296	-0.138	0.89	0.96	0.284	-0.141	0.888	0.537	1.715
KDM4B	-0.216	0.298	-0.726	0.468	0.805	0.24	-0.81	0.418	0.449	1.445
KDM4A	-0.454	0.302	-1.503	0.133	0.635	0.192	-1.903	0.057	0.351	1.148
KDM3B	-0.033	0.295	-0.111	0.912	0.968	0.286	-0.112	0.911	0.543	1.726
KDM3A	-0.647	0.305	-2.12	0.034	0.524	0.16	-2.981	0.003	0.288	0.952
KDM2A	0.699	0.315	2.219	0.026	2.011	0.633	1.597	0.11	1.085	3.729
KDM1A	0.328	0.297	1.103	0.27	1.388	0.412	0.941	0.347	0.775	2.483
KDELR3	-0.417	0.3	-1.391	0.164	0.659	0.197	-1.726	0.084	0.366	1.186
KDELR2	0.485	0.302	1.603	0.109	1.624	0.491	1.271	0.204	0.898	2.937
KDELR1	-0.101	0.296	-0.341	0.733	0.904	0.267	-0.359	0.72	0.507	1.614
KCTD9	0.04	0.296	0.135	0.892	1.041	0.308	0.133	0.894	0.582	1.86
KCTD5	0.59	0.306	1.93	0.054	1.804	0.551	1.458	0.145	0.991	3.283
KCTD3	0.112	0.296	0.38	0.704	1.119	0.331	0.359	0.72	0.626	2
KCTD20	-0.104	0.296	-0.35	0.726	0.902	0.267	-0.369	0.712	0.505	1.611
KCTD2	-0.197	0.297	-0.663	0.507	0.821	0.244	-0.733	0.463	0.458	1.47
KCTD17	-0.065	0.296	-0.219	0.827	0.937	0.278	-0.226	0.821	0.524	1.676
KCTD15	0.549	0.311	1.765	0.078	1.731	0.538	1.358	0.174	0.941	3.185
KCTD13	-0.35	0.3	-1.167	0.243	0.705	0.211	-1.397	0.162	0.392	1.268
KCTD12	-0.55	0.301	-1.829	0.067	0.577	0.173	-2.438	0.015	0.32	1.04
KCNV2	0.395	0.3	1.319	0.187	1.485	0.445	1.089	0.276	0.825	2.672
KCNV1	0.056	0.295	0.191	0.849	1.058	0.312	0.185	0.853	0.593	1.887
KCNS3	0.015	0.295	0.052	0.958	1.016	0.3	0.052	0.959	0.569	1.811
KCNS1	-0.213	0.296	-0.718	0.473	0.808	0.24	-0.8	0.424	0.452	1.445
KCNQ4	0.368	0.298	1.236	0.216	1.445	0.43	1.034	0.301	0.806	2.588
KCNQ3	0.194	0.295	0.657	0.511	1.214	0.359	0.597	0.55	0.681	2.166
KCNQ2	0.233	0.296	0.788	0.431	1.263	0.374	0.703	0.482	0.707	2.257
KCNQ1DN	0.507	0.302	1.676	0.094	1.66	0.502	1.314	0.189	0.918	3.003
KCNQ1	0.519	0.302	1.718	0.086	1.68	0.508	1.34	0.18	0.93	3.038
KCNN4	-0.249	0.297	-0.838	0.402	0.78	0.231	-0.952	0.341	0.436	1.395
KCNN3	-0.834	0.315	-2.647	0.008	0.435	0.137	-4.133	0	0.234	0.805
KCNN2	0.145	0.295	0.491	0.623	1.156	0.342	0.457	0.647	0.648	2.063
KCNN1	0.13	0.296	0.44	0.66	1.139	0.338	0.412	0.68	0.637	2.036

KCNMB4	0.059	0.296	0.199	0.842	1.061	0.314	0.194	0.846	0.594	1.894
KCNMB3	0.413	0.3	1.376	0.169	1.512	0.454	1.127	0.26	0.839	2.724
KCNMB2	0.124	0.295	0.421	0.674	1.132	0.334	0.396	0.692	0.635	2.02
KCNMB1	-0.424	0.298	-1.425	0.154	0.654	0.195	-1.775	0.076	0.365	1.173
KCNMA1	-0.476	0.3	-1.586	0.113	0.621	0.186	-2.032	0.042	0.345	1.119
KCNK7	-0.013	0.295	-0.043	0.966	0.987	0.292	-0.043	0.965	0.553	1.762
KCNK5	0.901	0.321	2.808	0.005	2.462	0.79	1.851	0.064	1.312	4.616
KCNK3	0.313	0.298	1.052	0.293	1.367	0.407	0.903	0.367	0.763	2.45
KCNK2	-0.071	0.295	-0.241	0.81	0.931	0.275	-0.25	0.803	0.522	1.661
KCNK15	0.259	0.297	0.874	0.382	1.296	0.384	0.77	0.442	0.724	2.318
KCNK13	-0.061	0.296	-0.205	0.838	0.941	0.279	-0.211	0.833	0.526	1.683
KCNK12	-0.553	0.303	-1.826	0.068	0.575	0.174	-2.44	0.015	0.317	1.041
KCNK10	0.596	0.306	1.949	0.051	1.815	0.555	1.468	0.142	0.997	3.307
KCNK1	0.312	0.3	1.039	0.299	1.366	0.41	0.893	0.372	0.759	2.46
KCNJ9	0.154	0.296	0.521	0.602	1.167	0.346	0.483	0.629	0.653	2.086
KCNJ8	0.286	0.298	0.961	0.337	1.331	0.396	0.835	0.403	0.743	2.385
KCNJ6	-0.465	0.302	-1.539	0.124	0.628	0.19	-1.96	0.05	0.347	1.136
KCNJ5	0.24	0.296	0.809	0.419	1.271	0.377	0.719	0.472	0.711	2.272
KCNJ4	-0.263	0.298	-0.885	0.376	0.769	0.229	-1.012	0.312	0.429	1.377
KCNJ3	-0.063	0.296	-0.213	0.831	0.939	0.278	-0.22	0.826	0.526	1.676
KCNJ2	-0.252	0.298	-0.848	0.396	0.777	0.231	-0.965	0.335	0.434	1.392
KCNJ16	-0.189	0.297	-0.638	0.524	0.828	0.246	-0.702	0.483	0.463	1.48
KCNJ15	-0.231	0.298	-0.776	0.438	0.793	0.237	-0.873	0.383	0.442	1.424
KCNJ14	0.192	0.299	0.644	0.519	1.212	0.362	0.586	0.558	0.675	2.176
KCNJ13	0.489	0.302	1.617	0.106	1.631	0.493	1.279	0.201	0.901	2.95
KCNJ10	0.373	0.3	1.242	0.214	1.451	0.435	1.037	0.3	0.806	2.613
KCNJ1	-0.537	0.301	-1.781	0.075	0.585	0.176	-2.358	0.018	0.324	1.055
KCNIP2	0.247	0.298	0.83	0.406	1.28	0.381	0.735	0.462	0.714	2.295
KCNIP1	0.166	0.296	0.559	0.576	1.18	0.35	0.515	0.607	0.66	2.109
KCNH6	0.336	0.298	1.128	0.259	1.399	0.417	0.958	0.338	0.78	2.509
KCNH4	0.316	0.3	1.056	0.291	1.372	0.411	0.905	0.366	0.763	2.468
KCNH2	0.518	0.306	1.69	0.091	1.679	0.514	1.319	0.187	0.921	3.061
KCNH1	-0.007	0.296	-0.024	0.98	0.993	0.294	-0.025	0.98	0.556	1.773
KCNG2	-0.405	0.303	-1.339	0.181	0.667	0.202	-1.651	0.099	0.369	1.207
KCNG1	0.578	0.303	1.908	0.056	1.783	0.54	1.449	0.147	0.985	3.228
KCNF1	0.459	0.3	1.531	0.126	1.583	0.475	1.227	0.22	0.879	2.849
KCNE5	0.569	0.302	1.883	0.06	1.767	0.534	1.436	0.151	0.977	3.196
KCNE4	-0.659	0.311	-2.119	0.034	0.518	0.161	-2.999	0.003	0.281	0.952

KCNE2	-0.256	0.298	-0.858	0.391	0.774	0.231	-0.978	0.328	0.432	1.388
KCNE1	0.008	0.295	0.028	0.977	1.008	0.298	0.028	0.977	0.565	1.798
KCND3	-0.375	0.308	-1.217	0.223	0.687	0.212	-1.477	0.14	0.376	1.257
KCND2	-0.319	0.298	-1.074	0.283	0.727	0.216	-1.265	0.206	0.406	1.302
KCND1	-0.024	0.298	-0.08	0.936	0.976	0.291	-0.081	0.935	0.544	1.751
KCNC4	0.411	0.298	1.377	0.168	1.508	0.45	1.129	0.259	0.84	2.708
KCNC3	-0.407	0.301	-1.351	0.177	0.666	0.2	-1.666	0.096	0.369	1.201
KCNC2	-0.112	0.296	-0.378	0.705	0.894	0.265	-0.4	0.689	0.5	1.598
KCNC1	0.29	0.298	0.974	0.33	1.336	0.398	0.846	0.398	0.746	2.394
KCNB2	0.277	0.296	0.935	0.35	1.319	0.391	0.817	0.414	0.738	2.357
KCNB1	0.766	0.31	2.467	0.014	2.151	0.668	1.723	0.085	1.17	3.952
KCNAB3	0.071	0.296	0.239	0.811	1.073	0.317	0.23	0.818	0.601	1.916
KCNAB2	0.056	0.296	0.19	0.85	1.058	0.313	0.184	0.854	0.593	1.888
KCNAB1	-0.017	0.295	-0.059	0.953	0.983	0.29	-0.06	0.952	0.551	1.752
KCNA5	-0.494	0.302	-1.636	0.102	0.61	0.184	-2.117	0.034	0.337	1.103
KCNA4	0.202	0.296	0.683	0.495	1.224	0.362	0.619	0.536	0.686	2.184
KCNA3	0.585	0.306	1.91	0.056	1.795	0.55	1.446	0.148	0.985	3.271
KCNA2	0.353	0.298	1.185	0.236	1.423	0.424	0.999	0.318	0.794	2.551
KCNA10	0.253	0.298	0.849	0.396	1.288	0.384	0.75	0.453	0.718	2.309
KCNA1	0.131	0.296	0.445	0.657	1.14	0.337	0.417	0.677	0.639	2.036
KCMF1	0.788	0.31	2.545	0.011	2.2	0.682	1.761	0.078	1.199	4.038
KBTBD4	-0.264	0.301	-0.878	0.38	0.768	0.231	-1.005	0.315	0.426	1.385
KBTBD2	0.562	0.302	1.858	0.063	1.754	0.53	1.422	0.155	0.97	3.172
KBTBD11	-0.24	0.298	-0.806	0.42	0.787	0.234	-0.911	0.362	0.439	1.41
KAZN	-0.74	0.315	-2.348	0.019	0.477	0.15	-3.478	0.001	0.257	0.885
KAZALD1	0.474	0.3	1.579	0.114	1.606	0.482	1.257	0.209	0.892	2.893
KATNIP	0.148	0.296	0.5	0.617	1.16	0.343	0.465	0.642	0.649	2.072
KATNBL1	-0.41	0.3	-1.367	0.172	0.664	0.199	-1.689	0.091	0.369	1.195
KATNB1	0.441	0.3	1.471	0.141	1.554	0.466	1.19	0.234	0.864	2.795
KATNA1	0.412	0.3	1.374	0.169	1.509	0.452	1.126	0.26	0.839	2.715
KAT8	0.289	0.297	0.971	0.331	1.335	0.397	0.844	0.399	0.745	2.391
KAT7	0.199	0.299	0.667	0.505	1.221	0.365	0.605	0.546	0.679	2.193
KAT6B	-0.017	0.295	-0.058	0.954	0.983	0.29	-0.059	0.953	0.551	1.752
KAT6A	-0.106	0.296	-0.36	0.719	0.899	0.266	-0.38	0.704	0.503	1.605
KAT5	-0.228	0.298	-0.766	0.444	0.796	0.237	-0.86	0.39	0.444	1.427
KAT2B	0.014	0.295	0.047	0.962	1.014	0.299	0.047	0.963	0.569	1.808
KAT2A	-0.03	0.297	-0.103	0.918	0.97	0.288	-0.104	0.917	0.542	1.736
KARS1	0.98	0.321	3.055	0.002	2.665	0.855	1.947	0.052	1.421	4.997

KANSL3	-0.08	0.297	-0.27	0.787	0.923	0.275	-0.281	0.779	0.515	1.653
KANSL2	0.71	0.31	2.294	0.022	2.034	0.63	1.642	0.101	1.109	3.733
KANSL1L	-0.11	0.295	-0.373	0.709	0.896	0.265	-0.394	0.693	0.502	1.598
KANK3	0.273	0.297	0.918	0.358	1.314	0.391	0.804	0.422	0.734	2.354
KANK2	-0.242	0.295	-0.818	0.413	0.785	0.232	-0.926	0.355	0.44	1.401
KANK1	-0.412	0.299	-1.375	0.169	0.662	0.198	-1.701	0.089	0.368	1.191
KALRN	-0.067	0.295	-0.226	0.821	0.936	0.276	-0.233	0.815	0.524	1.669
JUP	0.405	0.3	1.352	0.176	1.499	0.449	1.112	0.266	0.833	2.697
JUND	-0.028	0.295	-0.096	0.924	0.972	0.287	-0.097	0.922	0.545	1.734
JUNB	-0.114	0.295	-0.387	0.699	0.892	0.263	-0.41	0.682	0.5	1.591
JUN	0.302	0.297	1.015	0.31	1.352	0.402	0.876	0.381	0.755	2.421
JTB	0.202	0.296	0.682	0.495	1.224	0.362	0.618	0.537	0.685	2.187
JRKL	0.093	0.296	0.314	0.754	1.097	0.324	0.3	0.764	0.615	1.958
JRK	0.654	0.31	2.112	0.035	1.924	0.596	1.55	0.121	1.048	3.53
JPT2	-0.013	0.295	-0.045	0.964	0.987	0.291	-0.045	0.964	0.554	1.76
JPT1	0.675	0.306	2.207	0.027	1.964	0.6	1.605	0.108	1.079	3.576
JPH3	-0.031	0.296	-0.104	0.917	0.97	0.287	-0.106	0.916	0.543	1.73
JPH2	0.017	0.295	0.056	0.955	1.017	0.3	0.056	0.955	0.57	1.813
JOSD1	-0.142	0.296	-0.481	0.631	0.867	0.257	-0.516	0.606	0.485	1.55
JMJD6	0.525	0.303	1.735	0.083	1.69	0.512	1.35	0.177	0.934	3.059
JMJD4	0.916	0.315	2.907	0.004	2.498	0.787	1.904	0.057	1.347	4.632
JMJD1C	-0.191	0.296	-0.646	0.519	0.826	0.245	-0.711	0.477	0.462	1.476
JHY	-0.326	0.3	-1.087	0.277	0.722	0.216	-1.285	0.199	0.401	1.299
JCHAIN	-0.526	0.302	-1.739	0.082	0.591	0.179	-2.289	0.022	0.327	1.069
JCAD	-0.869	0.315	-2.761	0.006	0.419	0.132	-4.4	0	0.226	0.777
JARID2	-0.071	0.295	-0.241	0.81	0.931	0.275	-0.25	0.803	0.522	1.661
JAM3	-0.518	0.301	-1.721	0.085	0.596	0.179	-2.254	0.024	0.331	1.075
JAM2	-0.587	0.302	-1.943	0.052	0.556	0.168	-2.644	0.008	0.307	1.005
JAKMIP2	-0.25	0.297	-0.842	0.4	0.779	0.231	-0.956	0.339	0.435	1.394
JAK3	-0.148	0.296	-0.499	0.618	0.863	0.255	-0.538	0.591	0.483	1.541
JAK2	-0.867	0.315	-2.751	0.006	0.42	0.132	-4.379	0	0.226	0.779
JAK1	-0.902	0.315	-2.866	0.004	0.406	0.128	-4.654	0	0.219	0.752
JAG2	-0.119	0.297	-0.402	0.688	0.887	0.264	-0.427	0.67	0.495	1.59
JAG1	-0.602	0.306	-1.971	0.049	0.548	0.167	-2.704	0.007	0.301	0.997
JADE3	0.171	0.297	0.575	0.565	1.186	0.352	0.529	0.597	0.663	2.123
JADE2	-0.052	0.295	-0.177	0.86	0.949	0.28	-0.182	0.856	0.532	1.693
JADE1	0.179	0.296	0.603	0.547	1.196	0.354	0.552	0.581	0.669	2.137
IZUMO4	0.07	0.297	0.237	0.813	1.073	0.319	0.229	0.819	0.599	1.92

IVNS1ABP	0.093	0.295	0.316	0.752	1.098	0.324	0.302	0.763	0.616	1.957
IVL	-0.185	0.296	-0.627	0.531	0.831	0.246	-0.688	0.491	0.465	1.483
IVD	-0.288	0.298	-0.968	0.333	0.75	0.223	-1.122	0.262	0.418	1.343
ITSN2	0.034	0.295	0.116	0.908	1.035	0.305	0.114	0.909	0.58	1.845
ITSN1	-0.312	0.3	-1.042	0.297	0.732	0.219	-1.223	0.221	0.406	1.317
ITPRID2	-0.276	0.298	-0.927	0.354	0.759	0.226	-1.068	0.286	0.423	1.36
ITPR3	0.32	0.3	1.067	0.286	1.377	0.413	0.913	0.361	0.765	2.48
ITPR2	0.092	0.296	0.311	0.756	1.096	0.324	0.297	0.767	0.614	1.956
ITPR1	-0.431	0.303	-1.423	0.155	0.65	0.197	-1.778	0.075	0.359	1.177
ITPKC	0.476	0.3	1.591	0.112	1.61	0.482	1.265	0.206	0.895	2.897
ITPKB	-0.038	0.295	-0.128	0.898	0.963	0.284	-0.131	0.896	0.54	1.718
ITPKA	0.168	0.296	0.567	0.571	1.183	0.351	0.522	0.602	0.662	2.115
ITPK1	0.569	0.303	1.876	0.061	1.766	0.535	1.431	0.152	0.975	3.199
ITPA	-0.034	0.295	-0.116	0.907	0.966	0.285	-0.118	0.906	0.542	1.724
ITM2C	-0.31	0.298	-1.043	0.297	0.733	0.218	-1.223	0.221	0.409	1.314
ITM2B	-0.423	0.298	-1.418	0.156	0.655	0.195	-1.765	0.077	0.365	1.175
ITM2A	-1.019	0.317	-3.211	0.001	0.361	0.115	-5.58	0	0.194	0.672
ITK	-0.726	0.307	-2.363	0.018	0.484	0.149	-3.473	0.001	0.265	0.883
ITIH5	0.472	0.302	1.563	0.118	1.604	0.485	1.246	0.213	0.887	2.9
ITIH4	0.236	0.298	0.794	0.427	1.267	0.377	0.707	0.48	0.707	2.27
ITIH3	-0.699	0.307	-2.277	0.023	0.497	0.153	-3.295	0.001	0.273	0.907
ITIH2	0.207	0.298	0.695	0.487	1.23	0.367	0.628	0.53	0.686	2.206
ITIH1	-0.013	0.295	-0.045	0.964	0.987	0.291	-0.045	0.964	0.554	1.759
ITGBL1	-1.224	0.336	-3.643	0	0.294	0.099	-7.147	0	0.152	0.568
ITGB8	0.233	0.296	0.786	0.432	1.262	0.374	0.701	0.483	0.706	2.255
ITGB7	-0.057	0.296	-0.194	0.846	0.944	0.279	-0.2	0.842	0.529	1.686
ITGB5	-0.247	0.296	-0.833	0.405	0.781	0.231	-0.944	0.345	0.437	1.396
ITGB4	-0.339	0.298	-1.138	0.255	0.713	0.212	-1.355	0.175	0.398	1.277
ITGB3BP	0.024	0.298	0.08	0.936	1.024	0.305	0.079	0.937	0.571	1.837
ITGB3	0.507	0.3	1.691	0.091	1.661	0.498	1.326	0.185	0.923	2.989
ITGB2	-0.223	0.296	-0.752	0.452	0.8	0.237	-0.842	0.4	0.448	1.431
ITGB1BP2	-0.101	0.295	-0.343	0.732	0.904	0.267	-0.361	0.718	0.507	1.612
ITGB1BP1	0.092	0.295	0.312	0.755	1.097	0.324	0.298	0.766	0.615	1.957
ITGB1	-0.096	0.295	-0.324	0.746	0.909	0.268	-0.34	0.734	0.509	1.621
ITGAX	-0.011	0.295	-0.037	0.971	0.989	0.292	-0.037	0.97	0.555	1.764
ITGAV	-0.605	0.304	-1.991	0.046	0.546	0.166	-2.736	0.006	0.301	0.991
ITGAM	0.04	0.296	0.136	0.892	1.041	0.308	0.134	0.894	0.583	1.858
ITGAL	0.122	0.295	0.412	0.68	1.129	0.334	0.388	0.698	0.633	2.015

ITGAE	0.051	0.296	0.174	0.862	1.053	0.312	0.169	0.866	0.589	1.882
ITGA9	-0.299	0.297	-1.006	0.314	0.742	0.22	-1.173	0.241	0.414	1.327
ITGA8	-0.332	0.297	-1.118	0.264	0.718	0.213	-1.326	0.185	0.401	1.284
ITGA7	-0.196	0.296	-0.661	0.509	0.822	0.244	-0.73	0.465	0.46	1.47
ITGA6	-0.129	0.295	-0.437	0.662	0.879	0.26	-0.466	0.641	0.493	1.568
ITGA5	-0.687	0.306	-2.242	0.025	0.503	0.154	-3.224	0.001	0.276	0.917
ITGA4	-0.136	0.296	-0.459	0.646	0.873	0.258	-0.492	0.623	0.489	1.56
ITGA3	0.098	0.296	0.333	0.739	1.103	0.326	0.317	0.751	0.618	1.97
ITGA2B	0.404	0.3	1.348	0.178	1.498	0.449	1.109	0.267	0.832	2.695
ITGA2	-0.28	0.298	-0.941	0.347	0.756	0.225	-1.085	0.278	0.422	1.354
ITGA10	-0.04	0.296	-0.135	0.893	0.961	0.284	-0.138	0.89	0.538	1.716
ITGA1	-0.089	0.296	-0.302	0.763	0.915	0.27	-0.316	0.752	0.512	1.633
ITFG2	0.166	0.296	0.561	0.575	1.181	0.349	0.517	0.605	0.661	2.107
ITFG1	0.335	0.298	1.125	0.261	1.398	0.416	0.956	0.339	0.78	2.505
ITCH	0.871	0.321	2.711	0.007	2.39	0.768	1.81	0.07	1.273	4.487
ISYNA1	0.056	0.295	0.191	0.848	1.058	0.312	0.186	0.852	0.593	1.887
IST1	0.36	0.297	1.214	0.225	1.433	0.425	1.019	0.308	0.801	2.564
ISOC2	0.353	0.3	1.178	0.239	1.423	0.426	0.993	0.321	0.791	2.56
ISOC1	0.36	0.298	1.207	0.228	1.433	0.427	1.013	0.311	0.799	2.571
ISLR	-0.967	0.316	-3.059	0.002	0.38	0.12	-5.158	0	0.204	0.706
ISL1	0.209	0.299	0.7	0.484	1.233	0.369	0.631	0.528	0.686	2.215
ISG20L2	0.282	0.296	0.951	0.341	1.326	0.393	0.829	0.407	0.742	2.37
ISG20	0.513	0.302	1.698	0.09	1.671	0.505	1.328	0.184	0.924	3.023
ISG15	0.431	0.3	1.438	0.151	1.539	0.461	1.168	0.243	0.855	2.769
ISCU	-0.322	0.298	-1.084	0.278	0.724	0.216	-1.279	0.201	0.404	1.298
ISCA1	-0.063	0.297	-0.214	0.831	0.939	0.278	-0.221	0.825	0.525	1.678
IRX5	0.32	0.297	1.076	0.282	1.377	0.409	0.921	0.357	0.769	2.464
IRX4	0.495	0.302	1.636	0.102	1.64	0.496	1.291	0.197	0.907	2.967
IRS4	0.177	0.296	0.6	0.548	1.194	0.353	0.55	0.582	0.669	2.131
IRS2	-0.617	0.306	-2.018	0.044	0.539	0.165	-2.791	0.005	0.296	0.982
IRS1	-0.311	0.3	-1.038	0.299	0.732	0.22	-1.218	0.223	0.407	1.319
IRGQ	-0.656	0.308	-2.129	0.033	0.519	0.16	-3.009	0.003	0.284	0.949
IRGC	0.073	0.295	0.249	0.803	1.076	0.318	0.24	0.81	0.603	1.92
IRF9	0.074	0.295	0.249	0.803	1.076	0.318	0.24	0.81	0.603	1.921
IRF8	0.132	0.296	0.446	0.655	1.141	0.338	0.418	0.676	0.638	2.04
IRF7	0.151	0.296	0.509	0.611	1.163	0.344	0.472	0.637	0.651	2.077
IRF6	-0.396	0.3	-1.323	0.186	0.673	0.202	-1.623	0.105	0.374	1.21
IRF5	-0.583	0.303	-1.926	0.054	0.558	0.169	-2.615	0.009	0.308	1.01

IRF4	-0.173	0.296	-0.583	0.56	0.841	0.249	-0.637	0.524	0.471	1.503
IRF3	-0.36	0.298	-1.209	0.227	0.697	0.208	-1.456	0.146	0.389	1.251
IRF2BP1	-0.027	0.296	-0.092	0.927	0.973	0.288	-0.093	0.926	0.545	1.738
IRF2	0.004	0.297	0.014	0.989	1.004	0.299	0.014	0.989	0.561	1.799
IRF1	-0.416	0.3	-1.387	0.165	0.66	0.198	-1.72	0.085	0.366	1.188
IREB2	-0.262	0.3	-0.872	0.383	0.77	0.231	-0.996	0.319	0.427	1.387
IRAK4	-0.353	0.298	-1.185	0.236	0.703	0.209	-1.42	0.155	0.392	1.26
IRAK3	-0.776	0.315	-2.466	0.014	0.46	0.145	-3.728	0	0.248	0.853
IRAK1	0.258	0.296	0.87	0.384	1.294	0.383	0.767	0.443	0.724	2.313
IRAG2	-1.06	0.322	-3.286	0.001	0.347	0.112	-5.847	0	0.184	0.652
IQSEC3	-0.294	0.297	-0.992	0.321	0.745	0.221	-1.154	0.249	0.416	1.333
IQSEC2	0.238	0.296	0.803	0.422	1.268	0.376	0.715	0.475	0.71	2.266
IQSEC1	-0.714	0.306	-2.332	0.02	0.49	0.15	-3.405	0.001	0.269	0.892
IQGAP2	-0.468	0.3	-1.563	0.118	0.626	0.188	-1.993	0.046	0.348	1.126
IQGAP1	-0.505	0.302	-1.672	0.095	0.603	0.182	-2.176	0.03	0.334	1.091
IQCK	-0.005	0.296	-0.018	0.986	0.995	0.295	-0.018	0.986	0.557	1.778
IQCH	-0.089	0.297	-0.298	0.766	0.915	0.272	-0.312	0.755	0.511	1.638
IQCG	-0.111	0.296	-0.376	0.707	0.895	0.264	-0.397	0.691	0.502	1.597
IQCE	-0.529	0.302	-1.751	0.08	0.589	0.178	-2.308	0.021	0.326	1.065
IQCC	-0.016	0.298	-0.053	0.957	0.984	0.293	-0.054	0.957	0.549	1.765
IQCB1	0.358	0.298	1.202	0.229	1.43	0.426	1.011	0.312	0.798	2.563
IQCA1	-0.177	0.297	-0.598	0.55	0.838	0.248	-0.654	0.513	0.468	1.498
IPPK	0.291	0.298	0.977	0.328	1.337	0.398	0.848	0.396	0.746	2.396
IPP	-0.015	0.295	-0.052	0.959	0.985	0.291	-0.052	0.958	0.552	1.757
IPO9	0.604	0.306	1.978	0.048	1.83	0.559	1.484	0.138	1.006	3.331
IPO8	0.069	0.295	0.235	0.814	1.072	0.317	0.227	0.821	0.601	1.912
IPO7	0.504	0.303	1.664	0.096	1.655	0.501	1.307	0.191	0.914	2.995
IPO5	-0.008	0.295	-0.027	0.979	0.992	0.293	-0.027	0.979	0.557	1.769
IPO4	0.485	0.302	1.605	0.108	1.624	0.491	1.272	0.203	0.898	2.937
IPO13	0.001	0.296	0.003	0.998	1.001	0.297	0.003	0.998	0.56	1.789
IPCEF1	-0.47	0.299	-1.573	0.116	0.625	0.187	-2.008	0.045	0.348	1.123
IP6K2	-0.404	0.302	-1.337	0.181	0.667	0.202	-1.648	0.099	0.369	1.207
IP6K1	0.35	0.298	1.173	0.241	1.419	0.423	0.99	0.322	0.791	2.544
INVS	0.533	0.306	1.743	0.081	1.704	0.521	1.351	0.177	0.936	3.102
INTS9	-0.648	0.306	-2.12	0.034	0.523	0.16	-2.983	0.003	0.287	0.952
INTS8	0.104	0.297	0.35	0.726	1.11	0.33	0.332	0.74	0.62	1.987
INTS7	0.128	0.296	0.431	0.666	1.136	0.336	0.405	0.686	0.636	2.03
INTS6	0.393	0.3	1.31	0.19	1.482	0.445	1.083	0.279	0.823	2.669

INTS5	0.461	0.302	1.526	0.127	1.586	0.479	1.222	0.222	0.877	2.868
INTS3	0.243	0.296	0.819	0.413	1.275	0.378	0.727	0.467	0.713	2.278
INTS14	0.095	0.295	0.321	0.748	1.099	0.324	0.306	0.76	0.617	1.96
INTS13	0.398	0.3	1.329	0.184	1.489	0.446	1.096	0.273	0.828	2.678
INTS12	-0.259	0.311	-0.833	0.405	0.772	0.24	-0.951	0.341	0.42	1.419
INTS1	-0.044	0.295	-0.148	0.883	0.957	0.283	-0.151	0.88	0.537	1.707
INSRR	-0.223	0.296	-0.752	0.452	0.8	0.237	-0.842	0.4	0.448	1.431
INSR	-0.447	0.306	-1.459	0.145	0.64	0.196	-1.84	0.066	0.351	1.166
INSM1	0.08	0.295	0.27	0.787	1.083	0.32	0.259	0.795	0.607	1.931
INSL6	0.371	0.3	1.235	0.217	1.449	0.435	1.032	0.302	0.804	2.61
INSL5	0.121	0.295	0.411	0.681	1.129	0.333	0.387	0.699	0.633	2.014
INSL4	-0.139	0.298	-0.465	0.642	0.87	0.26	-0.499	0.618	0.485	1.562
INSL3	-0.791	0.311	-2.547	0.011	0.453	0.141	-3.883	0	0.247	0.833
INSIG2	0.055	0.295	0.185	0.853	1.056	0.312	0.18	0.857	0.592	1.884
INSIG1	0.124	0.296	0.418	0.676	1.132	0.335	0.393	0.694	0.634	2.02
INS	0.896	0.316	2.838	0.005	2.449	0.773	1.875	0.061	1.319	4.547
INPPL1	0.095	0.295	0.323	0.747	1.1	0.325	0.308	0.758	0.617	1.963
INPP5K	0.085	0.297	0.286	0.775	1.088	0.323	0.274	0.784	0.608	1.947
INPP5J	-0.143	0.296	-0.481	0.63	0.867	0.257	-0.517	0.605	0.485	1.55
INPP5F	0.031	0.296	0.104	0.917	1.031	0.305	0.103	0.918	0.578	1.841
INPP5E	-0.016	0.296	-0.056	0.956	0.984	0.291	-0.056	0.955	0.551	1.756
INPP5D	-0.817	0.311	-2.627	0.009	0.442	0.137	-4.062	0	0.24	0.813
INPP5B	0.014	0.296	0.048	0.962	1.014	0.3	0.048	0.962	0.568	1.811
INPP5A	0.153	0.295	0.517	0.605	1.165	0.344	0.48	0.631	0.653	2.079
INPP4B	0.254	0.298	0.853	0.394	1.29	0.385	0.753	0.451	0.719	2.314
INPP4A	-0.38	0.298	-1.273	0.203	0.684	0.204	-1.548	0.122	0.381	1.228
INPP1	-0.673	0.31	-2.175	0.03	0.51	0.158	-3.103	0.002	0.278	0.936
INO80D	0.391	0.303	1.292	0.196	1.479	0.448	1.069	0.285	0.817	2.677
INO80B	0.379	0.298	1.274	0.203	1.462	0.435	1.06	0.289	0.815	2.62
INHBE	-0.016	0.295	-0.054	0.957	0.984	0.291	-0.054	0.957	0.552	1.756
INHBC	0.574	0.303	1.896	0.058	1.775	0.537	1.443	0.149	0.981	3.211
INHBB	-0.342	0.298	-1.15	0.25	0.71	0.211	-1.371	0.17	0.396	1.273
INHBA	-0.551	0.306	-1.804	0.071	0.576	0.176	-2.406	0.016	0.317	1.049
INHA	-0.112	0.295	-0.378	0.705	0.894	0.264	-0.4	0.689	0.501	1.595
ING4	0.016	0.295	0.054	0.957	1.016	0.3	0.054	0.957	0.57	1.811
ING3	0.139	0.296	0.47	0.639	1.149	0.34	0.439	0.661	0.644	2.051
ING2	0.378	0.299	1.263	0.207	1.459	0.437	1.052	0.293	0.812	2.624
ING1	-0.061	0.295	-0.205	0.837	0.941	0.278	-0.212	0.832	0.528	1.678

INF2	0.5	0.302	1.654	0.098	1.649	0.498	1.302	0.193	0.912	2.982
INE1	0.066	0.297	0.221	0.825	1.068	0.317	0.214	0.831	0.597	1.911
INCENP	-0.119	0.297	-0.4	0.689	0.888	0.263	-0.425	0.671	0.497	1.588
INAVA	0.96	0.328	2.925	0.003	2.611	0.857	1.88	0.06	1.372	4.967
INA	-0.076	0.295	-0.258	0.797	0.927	0.274	-0.268	0.789	0.519	1.653
IMPG2	0.353	0.3	1.179	0.238	1.424	0.427	0.993	0.321	0.791	2.562
IMPG1	-0.226	0.296	-0.765	0.444	0.797	0.236	-0.858	0.391	0.446	1.425
IMPDH2	-0.437	0.303	-1.443	0.149	0.646	0.195	-1.81	0.07	0.357	1.169
IMPDH1	0.586	0.306	1.917	0.055	1.797	0.549	1.451	0.147	0.987	3.271
IMPACT	-0.602	0.306	-1.969	0.049	0.548	0.167	-2.701	0.007	0.301	0.997
IMPA2	0.174	0.295	0.588	0.556	1.19	0.351	0.54	0.589	0.667	2.123
IMPA1	0.078	0.296	0.263	0.793	1.081	0.32	0.253	0.801	0.605	1.93
IMP4	-0.135	0.297	-0.454	0.65	0.874	0.259	-0.486	0.627	0.489	1.563
IMP3	-0.176	0.296	-0.595	0.552	0.838	0.248	-0.65	0.515	0.469	1.498
IMMT	0.343	0.296	1.158	0.247	1.409	0.418	0.98	0.327	0.788	2.519
ILVBL	0.441	0.3	1.472	0.141	1.554	0.466	1.191	0.234	0.864	2.796
ILRUN	0.181	0.297	0.609	0.543	1.198	0.355	0.557	0.578	0.67	2.143
ILKAP	0.141	0.295	0.479	0.632	1.152	0.34	0.447	0.655	0.646	2.055
ILK	-0.858	0.321	-2.676	0.007	0.424	0.136	-4.236	0	0.226	0.795
ILF3	-0.137	0.296	-0.462	0.644	0.872	0.258	-0.495	0.621	0.488	1.559
ILF2	0.143	0.296	0.483	0.629	1.154	0.341	0.45	0.652	0.646	2.059
IL9R	0.469	0.3	1.564	0.118	1.598	0.479	1.249	0.212	0.888	2.877
IL9	0.045	0.296	0.151	0.88	1.046	0.309	0.147	0.883	0.586	1.867
IL7R	0.143	0.296	0.485	0.628	1.154	0.341	0.452	0.651	0.646	2.061
IL7	-0.166	0.299	-0.555	0.579	0.847	0.253	-0.604	0.546	0.472	1.521
IL6ST	-0.783	0.315	-2.488	0.013	0.457	0.144	-3.776	0	0.247	0.847
IL6R	-0.212	0.296	-0.716	0.474	0.809	0.24	-0.797	0.425	0.453	1.445
IL6	-0.176	0.297	-0.594	0.553	0.839	0.249	-0.649	0.516	0.469	1.499
IL5RA	0.075	0.296	0.254	0.799	1.078	0.319	0.245	0.807	0.604	1.924
IL5	0.396	0.298	1.329	0.184	1.486	0.443	1.097	0.272	0.829	2.665
IL4R	-0.614	0.3	-2.044	0.041	0.541	0.163	-2.823	0.005	0.3	0.975
IL4	0.966	0.321	3.012	0.003	2.628	0.843	1.931	0.053	1.401	4.928
IL3RA	0.086	0.295	0.291	0.771	1.09	0.322	0.279	0.78	0.611	1.944
IL37	-0.074	0.296	-0.25	0.802	0.929	0.275	-0.26	0.795	0.52	1.658
IL36RN	0.346	0.299	1.159	0.246	1.414	0.422	0.98	0.327	0.787	2.539
IL36G	-0.084	0.295	-0.286	0.775	0.919	0.272	-0.298	0.766	0.515	1.64
IL36A	0.679	0.31	2.19	0.029	1.972	0.611	1.59	0.112	1.074	3.62
IL33	-0.382	0.298	-1.284	0.199	0.682	0.203	-1.565	0.118	0.381	1.223

IL32	-0.545	0.306	-1.783	0.075	0.58	0.177	-2.37	0.018	0.319	1.056
IL3	0.416	0.3	1.389	0.165	1.516	0.454	1.136	0.256	0.843	2.727
IL2RG	-0.406	0.298	-1.363	0.173	0.666	0.199	-1.681	0.093	0.371	1.195
IL2RB	-0.307	0.297	-1.034	0.301	0.735	0.219	-1.21	0.226	0.41	1.317
IL2RA	-0.281	0.298	-0.946	0.344	0.755	0.225	-1.092	0.275	0.421	1.352
IL27RA	-0.157	0.295	-0.531	0.596	0.855	0.253	-0.575	0.566	0.479	1.525
IL26	-0.129	0.295	-0.437	0.662	0.879	0.26	-0.467	0.641	0.493	1.568
IL25	0.199	0.299	0.667	0.505	1.22	0.364	0.605	0.545	0.68	2.191
IL24	0.296	0.298	0.994	0.32	1.345	0.401	0.86	0.39	0.75	2.411
IL23A	0.111	0.295	0.376	0.707	1.118	0.33	0.356	0.722	0.627	1.993
IL22RA1	0.272	0.298	0.914	0.361	1.313	0.391	0.8	0.424	0.733	2.352
IL22	0.241	0.296	0.816	0.414	1.273	0.377	0.725	0.468	0.713	2.273
IL21R	-0.846	0.311	-2.722	0.006	0.429	0.133	-4.281	0	0.233	0.789
IL21	-0.714	0.31	-2.305	0.021	0.489	0.152	-3.365	0.001	0.267	0.899
IL20RA	-1.089	0.336	-3.239	0.001	0.337	0.113	-5.862	0	0.174	0.651
IL2	-0.125	0.296	-0.422	0.673	0.883	0.261	-0.449	0.653	0.494	1.577
IL1RN	0.621	0.306	2.031	0.042	1.86	0.569	1.513	0.13	1.022	3.387
IL1RL2	0.198	0.296	0.669	0.504	1.219	0.361	0.607	0.544	0.682	2.178
IL1RL1	-0.134	0.296	-0.453	0.651	0.874	0.259	-0.485	0.628	0.489	1.563
IL1RAPL2	0.308	0.306	1.006	0.314	1.36	0.416	0.866	0.387	0.747	2.478
IL1RAPL1	-0.545	0.306	-1.781	0.075	0.58	0.177	-2.368	0.018	0.318	1.056
IL1RAP	-0.564	0.306	-1.844	0.065	0.569	0.174	-2.476	0.013	0.313	1.036
IL1R2	-0.089	0.296	-0.301	0.763	0.915	0.27	-0.315	0.753	0.513	1.633
IL1R1	-0.562	0.301	-1.866	0.062	0.57	0.172	-2.505	0.012	0.316	1.029
IL1B	-0.301	0.3	-1.003	0.316	0.74	0.222	-1.17	0.242	0.411	1.332
IL1A	0.669	0.31	2.16	0.031	1.953	0.605	1.575	0.115	1.064	3.585
IL19	0.513	0.3	1.711	0.087	1.67	0.501	1.339	0.181	0.928	3.006
IL18RAP	-0.501	0.3	-1.668	0.095	0.606	0.182	-2.165	0.03	0.336	1.092
IL18R1	-0.456	0.302	-1.507	0.132	0.634	0.192	-1.909	0.056	0.35	1.147
IL18BP	0.085	0.295	0.289	0.772	1.089	0.321	0.277	0.782	0.611	1.942
IL18	-0.257	0.298	-0.864	0.388	0.773	0.23	-0.985	0.325	0.432	1.386
IL17RC	0.123	0.295	0.416	0.678	1.131	0.334	0.391	0.696	0.634	2.017
IL17RB	0.119	0.295	0.402	0.688	1.126	0.333	0.379	0.705	0.631	2.009
IL17RA	0.238	0.298	0.799	0.425	1.268	0.378	0.711	0.477	0.708	2.273
IL17B	0.201	0.296	0.679	0.497	1.223	0.362	0.615	0.539	0.684	2.184
IL17A	0.023	0.295	0.078	0.938	1.023	0.302	0.077	0.939	0.574	1.825
IL16	-0.402	0.298	-1.35	0.177	0.669	0.199	-1.662	0.097	0.373	1.2
IL15RA	0.092	0.296	0.312	0.755	1.097	0.324	0.298	0.766	0.614	1.958

IL15	-0.246	0.298	-0.824	0.41	0.782	0.233	-0.934	0.35	0.436	1.403
IL13RA2	-0.131	0.298	-0.441	0.659	0.877	0.261	-0.471	0.638	0.489	1.573
IL13RA1	-0.537	0.303	-1.773	0.076	0.584	0.177	-2.347	0.019	0.323	1.058
IL13	0.14	0.296	0.472	0.637	1.15	0.341	0.441	0.659	0.644	2.056
IL12RB2	-0.109	0.296	-0.368	0.713	0.897	0.266	-0.389	0.697	0.502	1.603
IL12RB1	-0.096	0.297	-0.323	0.747	0.909	0.269	-0.339	0.734	0.508	1.625
IL12B	-0.232	0.298	-0.78	0.435	0.793	0.236	-0.878	0.38	0.442	1.421
IL12A	0.284	0.298	0.953	0.341	1.328	0.395	0.83	0.407	0.741	2.38
IL11RA	-0.048	0.295	-0.162	0.871	0.953	0.281	-0.166	0.868	0.535	1.7
IL11	-0.548	0.303	-1.811	0.07	0.578	0.175	-2.412	0.016	0.319	1.046
IL10RB	-0.078	0.295	-0.265	0.791	0.925	0.273	-0.276	0.783	0.519	1.649
IL10RA	-0.11	0.295	-0.374	0.709	0.895	0.265	-0.395	0.693	0.502	1.598
IL10	0.161	0.297	0.544	0.586	1.175	0.349	0.502	0.615	0.657	2.102
IKZF5	-0.089	0.296	-0.301	0.763	0.915	0.271	-0.315	0.753	0.512	1.635
IKZF4	-0.428	0.3	-1.427	0.154	0.652	0.196	-1.781	0.075	0.362	1.173
IKZF3	-0.02	0.295	-0.069	0.945	0.98	0.289	-0.07	0.944	0.549	1.748
IKZF2	-0.116	0.296	-0.393	0.695	0.89	0.263	-0.416	0.677	0.499	1.589
IKZF1	-0.049	0.296	-0.165	0.869	0.952	0.282	-0.169	0.865	0.533	1.7
IKBKG	0.297	0.301	0.985	0.324	1.345	0.405	0.853	0.394	0.746	2.426
IKBKE	-0.157	0.296	-0.532	0.595	0.854	0.253	-0.576	0.565	0.478	1.526
IKBKB	-0.45	0.303	-1.488	0.137	0.637	0.193	-1.88	0.06	0.352	1.153
IK	0.438	0.299	1.464	0.143	1.55	0.464	1.186	0.236	0.862	2.786
IHH	0.391	0.298	1.313	0.189	1.479	0.441	1.086	0.277	0.825	2.652
IGSF9B	0.314	0.298	1.054	0.292	1.369	0.408	0.904	0.366	0.763	2.456
IGSF6	0.215	0.296	0.724	0.469	1.239	0.367	0.652	0.514	0.693	2.216
IGSF3	0.047	0.296	0.16	0.873	1.049	0.31	0.157	0.875	0.587	1.872
IGSF1	-0.075	0.297	-0.252	0.801	0.928	0.275	-0.262	0.793	0.519	1.659
IGLV6-57	-0.35	0.298	-1.173	0.241	0.705	0.21	-1.404	0.16	0.393	1.265
IGLV1-44	-0.423	0.298	-1.418	0.156	0.655	0.195	-1.765	0.078	0.365	1.176
IGLV@	0.342	0.298	1.148	0.251	1.407	0.419	0.972	0.331	0.785	2.521
IGLL3P	-0.323	0.298	-1.083	0.279	0.724	0.216	-1.278	0.201	0.403	1.299
IGLL1	0.066	0.296	0.224	0.823	1.069	0.317	0.216	0.829	0.598	1.91
IGLJ3	-0.48	0.3	-1.597	0.11	0.619	0.186	-2.05	0.04	0.344	1.115
IGLC1	-0.378	0.298	-1.268	0.205	0.685	0.204	-1.541	0.123	0.382	1.229
IGKC	-0.399	0.299	-1.336	0.181	0.671	0.2	-1.642	0.101	0.374	1.205
IGK	-0.56	0.301	-1.863	0.063	0.571	0.172	-2.497	0.013	0.317	1.03
IGHV5-78	-0.295	0.298	-0.99	0.322	0.745	0.222	-1.151	0.25	0.416	1.335
IGHMBP2	0.112	0.295	0.378	0.705	1.118	0.33	0.358	0.72	0.627	1.996

IGHM	-0.39	0.299	-1.305	0.192	0.677	0.202	-1.596	0.111	0.377	1.216
IGHG1	-1	0.317	-3.156	0.002	0.368	0.117	-5.425	0	0.198	0.684
IGHD	-1.009	0.321	-3.143	0.002	0.365	0.117	-5.428	0	0.194	0.684
IGH	-0.738	0.31	-2.381	0.017	0.478	0.148	-3.522	0	0.261	0.878
IGFLR1	-0.05	0.295	-0.17	0.865	0.951	0.281	-0.175	0.861	0.533	1.697
IGFBP7	-0.594	0.303	-1.963	0.05	0.552	0.167	-2.681	0.007	0.305	0.999
IGFBP6	-0.713	0.31	-2.302	0.021	0.49	0.152	-3.359	0.001	0.267	0.899
IGFBP5	0.062	0.295	0.208	0.835	1.063	0.314	0.202	0.84	0.596	1.897
IGFBP4	-0.681	0.306	-2.226	0.026	0.506	0.155	-3.189	0.001	0.278	0.922
IGFBP3	-0.995	0.328	-3.035	0.002	0.37	0.121	-5.2	0	0.195	0.703
IGFBP2	-0.051	0.298	-0.171	0.864	0.95	0.283	-0.176	0.861	0.53	1.704
IGFBP1	-0.096	0.296	-0.323	0.746	0.909	0.269	-0.339	0.734	0.509	1.622
IGFALS	0.005	0.296	0.017	0.986	1.005	0.298	0.017	0.986	0.563	1.796
IGF2R	-0.437	0.299	-1.459	0.145	0.646	0.193	-1.829	0.067	0.359	1.162
IGF2BP3	0.185	0.296	0.625	0.532	1.203	0.356	0.57	0.568	0.673	2.15
IGF2BP2	0.175	0.296	0.591	0.554	1.191	0.353	0.542	0.587	0.667	2.129
IGF2-AS	0.325	0.297	1.095	0.274	1.384	0.411	0.935	0.35	0.774	2.475
IGF1R	0.013	0.297	0.045	0.964	1.014	0.301	0.045	0.964	0.567	1.813
IGF1	-0.811	0.31	-2.617	0.009	0.444	0.138	-4.033	0	0.242	0.816
IGBP1	-0.591	0.306	-1.935	0.053	0.554	0.169	-2.638	0.008	0.304	1.008
IFT88	-0.095	0.296	-0.322	0.747	0.909	0.269	-0.338	0.736	0.509	1.623
IFT81	0.317	0.298	1.066	0.286	1.373	0.409	0.914	0.361	0.766	2.46
IFT74	0.134	0.299	0.448	0.654	1.143	0.342	0.419	0.675	0.636	2.053
IFT57	0.049	0.296	0.167	0.867	1.051	0.311	0.163	0.871	0.588	1.876
IFT52	0.102	0.298	0.341	0.733	1.107	0.33	0.324	0.746	0.617	1.985
IFT46	-0.921	0.322	-2.863	0.004	0.398	0.128	-4.699	0	0.212	0.748
IFT27	-0.265	0.298	-0.891	0.373	0.767	0.228	-1.02	0.308	0.428	1.375
IFT22	-0.17	0.299	-0.57	0.569	0.844	0.252	-0.621	0.534	0.47	1.514
IFT20	0.065	0.295	0.22	0.826	1.067	0.315	0.213	0.831	0.598	1.904
IFT140	-0.574	0.306	-1.879	0.06	0.563	0.172	-2.538	0.011	0.309	1.025
IFT122	-0.313	0.301	-1.04	0.298	0.731	0.22	-1.221	0.222	0.406	1.319
IFRD2	-0.349	0.298	-1.17	0.242	0.706	0.21	-1.399	0.162	0.394	1.266
IFRD1	0.05	0.297	0.169	0.866	1.051	0.312	0.165	0.869	0.587	1.883
IFNW1	0.38	0.3	1.269	0.204	1.463	0.438	1.056	0.291	0.813	2.631
IFNGR2	0.357	0.298	1.199	0.231	1.429	0.425	1.008	0.313	0.797	2.561
IFNGR1	-0.516	0.302	-1.706	0.088	0.597	0.18	-2.233	0.026	0.33	1.08
IFNG	0.382	0.302	1.264	0.206	1.465	0.443	1.05	0.294	0.81	2.651
IFNB1	0.637	0.306	2.082	0.037	1.891	0.579	1.54	0.124	1.038	3.445

IFNAR2	-0.55	0.303	-1.818	0.069	0.577	0.175	-2.425	0.015	0.319	1.044
IFNAR1	0.171	0.296	0.577	0.564	1.186	0.351	0.53	0.596	0.664	2.12
IFNA8	0.226	0.296	0.764	0.445	1.254	0.372	0.683	0.494	0.702	2.241
IFNA7	-0.029	0.295	-0.099	0.921	0.971	0.287	-0.1	0.92	0.544	1.733
IFNA5	-0.59	0.303	-1.946	0.052	0.554	0.168	-2.652	0.008	0.306	1.004
IFNA4	-0.315	0.3	-1.051	0.293	0.73	0.219	-1.235	0.217	0.405	1.314
IFNA21	-0.149	0.296	-0.505	0.614	0.861	0.255	-0.544	0.586	0.482	1.538
IFNA2	0.532	0.302	1.759	0.079	1.702	0.515	1.364	0.172	0.941	3.08
IFNA17	-0.089	0.296	-0.302	0.763	0.915	0.271	-0.316	0.752	0.512	1.634
IFNA16	0.117	0.296	0.397	0.691	1.124	0.332	0.375	0.708	0.63	2.007
IFNA14	0.519	0.302	1.716	0.086	1.68	0.508	1.339	0.181	0.929	3.038
IFNA10	0.295	0.296	0.995	0.32	1.343	0.398	0.862	0.389	0.751	2.401
IFNA1	-0.159	0.296	-0.536	0.592	0.853	0.252	-0.581	0.561	0.478	1.524
IFITM3	0.145	0.297	0.489	0.625	1.156	0.343	0.455	0.649	0.646	2.069
IFITM2	-0.082	0.295	-0.279	0.78	0.921	0.272	-0.291	0.771	0.516	1.643
IFITM1	0.298	0.298	1.002	0.316	1.348	0.401	0.866	0.386	0.752	2.415
IFIT5	-0.115	0.296	-0.388	0.698	0.892	0.263	-0.411	0.681	0.5	1.591
IFIT3	0.181	0.296	0.612	0.541	1.199	0.355	0.559	0.576	0.671	2.143
IFIT2	0.125	0.297	0.421	0.673	1.133	0.336	0.396	0.692	0.633	2.027
IFIT1	0.237	0.298	0.796	0.426	1.267	0.377	0.708	0.479	0.707	2.271
IFIH1	0.303	0.298	1.017	0.309	1.354	0.403	0.878	0.38	0.755	2.428
IFI6	0.088	0.296	0.299	0.765	1.092	0.323	0.286	0.775	0.612	1.95
IFI44L	0.097	0.296	0.326	0.744	1.101	0.326	0.311	0.756	0.617	1.966
IFI44	0.4	0.3	1.334	0.182	1.492	0.448	1.099	0.272	0.829	2.686
IFI35	0.256	0.297	0.86	0.39	1.291	0.384	0.759	0.448	0.721	2.311
IFI27	0.43	0.303	1.422	0.155	1.538	0.465	1.156	0.248	0.85	2.782
IFI16	-0.59	0.303	-1.949	0.051	0.554	0.168	-2.656	0.008	0.306	1.003
IFFO1	-0.422	0.3	-1.408	0.159	0.656	0.197	-1.751	0.08	0.365	1.18
IER5	0.447	0.302	1.478	0.139	1.563	0.472	1.192	0.233	0.865	2.827
IER3IP1	0.361	0.297	1.217	0.224	1.435	0.427	1.021	0.307	0.802	2.57
IER3	0.221	0.296	0.746	0.455	1.247	0.369	0.67	0.503	0.698	2.227
IER2	0.19	0.296	0.642	0.521	1.209	0.358	0.584	0.559	0.677	2.161
IDUA	-0.441	0.3	-1.471	0.141	0.644	0.193	-1.849	0.065	0.358	1.158
IDS	-0.059	0.296	-0.2	0.842	0.943	0.279	-0.206	0.837	0.528	1.682
IDO1	0.281	0.301	0.934	0.35	1.324	0.398	0.814	0.416	0.735	2.387
IDI2-AS1	0.185	0.296	0.627	0.531	1.204	0.356	0.572	0.567	0.674	2.148
IDI1	0.375	0.298	1.261	0.207	1.456	0.433	1.052	0.293	0.812	2.609
IDH3G	-0.236	0.3	-0.785	0.433	0.79	0.237	-0.885	0.376	0.439	1.423

IDH3B	0.091	0.295	0.308	0.758	1.095	0.324	0.295	0.768	0.614	1.954
IDH3A	0.136	0.295	0.462	0.644	1.146	0.339	0.432	0.666	0.642	2.045
IDH2	0.253	0.298	0.85	0.395	1.288	0.384	0.751	0.453	0.719	2.31
IDH1	0.18	0.298	0.603	0.546	1.197	0.356	0.552	0.581	0.668	2.145
IDE	-0.209	0.298	-0.701	0.483	0.812	0.242	-0.78	0.436	0.453	1.455
ID4	-0.372	0.298	-1.248	0.212	0.69	0.205	-1.511	0.131	0.385	1.236
ID3	-0.833	0.315	-2.647	0.008	0.435	0.137	-4.133	0	0.235	0.805
ID2B	0.243	0.296	0.821	0.412	1.275	0.378	0.729	0.466	0.714	2.279
ID2	-0.258	0.298	-0.868	0.386	0.772	0.23	-0.99	0.322	0.431	1.384
ID1	-0.44	0.3	-1.47	0.142	0.644	0.193	-1.847	0.065	0.358	1.158
ICOSLG	-0.072	0.299	-0.24	0.811	0.931	0.278	-0.248	0.804	0.518	1.672
ICOS	0.253	0.298	0.848	0.396	1.288	0.384	0.75	0.453	0.718	2.308
ICMT	0.056	0.295	0.191	0.848	1.058	0.312	0.186	0.852	0.593	1.887
ICE2	-0.473	0.306	-1.547	0.122	0.623	0.191	-1.978	0.048	0.342	1.135
ICE1	0.366	0.298	1.231	0.218	1.442	0.429	1.031	0.303	0.805	2.584
ICAM5	-0.21	0.298	-0.705	0.481	0.811	0.241	-0.784	0.433	0.453	1.453
ICAM4	-0.501	0.303	-1.654	0.098	0.606	0.183	-2.147	0.032	0.335	1.097
ICAM3	0.351	0.299	1.172	0.241	1.42	0.425	0.989	0.323	0.79	2.553
ICAM2	-0.679	0.306	-2.218	0.027	0.507	0.155	-3.175	0.001	0.278	0.924
ICAM1	-0.187	0.296	-0.63	0.529	0.83	0.246	-0.692	0.489	0.464	1.483
ICA1	-0.165	0.299	-0.553	0.58	0.848	0.253	-0.602	0.547	0.472	1.523
IBTK	-0.496	0.302	-1.641	0.101	0.609	0.184	-2.125	0.034	0.337	1.101
IBSP	0.194	0.295	0.656	0.512	1.214	0.359	0.597	0.551	0.68	2.166
IBA57	-0.07	0.295	-0.235	0.814	0.933	0.275	-0.244	0.807	0.523	1.664
IARS2	-0.003	0.296	-0.009	0.992	0.997	0.295	-0.009	0.992	0.558	1.781
IARS1	0.321	0.3	1.071	0.284	1.379	0.413	0.916	0.36	0.766	2.481
IAPP	-0.278	0.297	-0.938	0.348	0.757	0.225	-1.081	0.28	0.423	1.354
HYOU1	-0.43	0.3	-1.431	0.152	0.651	0.195	-1.788	0.074	0.361	1.172
HYMAI	-0.419	0.3	-1.4	0.162	0.657	0.197	-1.739	0.082	0.365	1.183
HYI	-0.223	0.296	-0.751	0.453	0.8	0.237	-0.841	0.4	0.448	1.431
HYAL4	-0.447	0.3	-1.491	0.136	0.639	0.192	-1.88	0.06	0.355	1.151
HYAL3	-0.127	0.297	-0.427	0.669	0.881	0.261	-0.455	0.649	0.492	1.576
HYAL2	-0.739	0.31	-2.383	0.017	0.478	0.148	-3.527	0	0.26	0.877
HYAL1	0.117	0.296	0.396	0.692	1.124	0.332	0.374	0.709	0.63	2.007
HUWE1	-0.354	0.3	-1.18	0.238	0.702	0.211	-1.415	0.157	0.39	1.264
HUS1	-0.122	0.296	-0.413	0.68	0.885	0.262	-0.439	0.661	0.495	1.581
HUNK	0.039	0.295	0.133	0.894	1.04	0.307	0.131	0.896	0.583	1.854
HTT	0.482	0.299	1.614	0.107	1.62	0.484	1.281	0.2	0.902	2.91

HTRA2	-0.082	0.295	-0.278	0.781	0.921	0.272	-0.289	0.772	0.516	1.644
HTRA1	-0.513	0.302	-1.696	0.09	0.599	0.181	-2.216	0.027	0.331	1.083
HTR7P1	-0.192	0.298	-0.646	0.519	0.825	0.246	-0.712	0.476	0.46	1.48
HTR7	0.528	0.301	1.755	0.079	1.696	0.51	1.364	0.173	0.94	3.057
HTR6	0.661	0.306	2.163	0.031	1.937	0.592	1.583	0.113	1.064	3.526
HTR5A	-0.118	0.297	-0.397	0.691	0.889	0.264	-0.422	0.673	0.497	1.59
HTR4	0.348	0.3	1.163	0.245	1.417	0.424	0.982	0.326	0.788	2.549
HTR3B	0.224	0.296	0.755	0.45	1.251	0.37	0.677	0.499	0.7	2.235
HTR3A	0.252	0.296	0.852	0.394	1.287	0.381	0.753	0.452	0.72	2.3
HTR2C	0.78	0.311	2.512	0.012	2.182	0.678	1.744	0.081	1.187	4.011
HTR2B	-0.591	0.306	-1.932	0.053	0.554	0.169	-2.634	0.008	0.304	1.009
HTR2A	0.058	0.297	0.195	0.845	1.06	0.315	0.19	0.85	0.592	1.897
HTR1F	0	0.295	0.001	0.999	1	0.295	0.001	0.999	0.561	1.785
HTR1E	0.12	0.296	0.407	0.684	1.128	0.334	0.384	0.701	0.632	2.014
HTR1D	0.334	0.3	1.115	0.265	1.397	0.419	0.948	0.343	0.776	2.513
HTR1B	-0.206	0.297	-0.694	0.488	0.814	0.241	-0.77	0.441	0.455	1.456
HTR1A	-0.485	0.3	-1.616	0.106	0.616	0.185	-2.08	0.038	0.342	1.109
HTN3	-0.253	0.296	-0.854	0.393	0.777	0.23	-0.971	0.331	0.435	1.388
HTN1	0.076	0.296	0.256	0.798	1.079	0.319	0.247	0.805	0.604	1.925
HTATSF1	1.232	0.346	3.56	0	3.427	1.186	2.047	0.041	1.739	6.752
HTATIP2	0.88	0.315	2.791	0.005	2.41	0.76	1.857	0.063	1.3	4.47
HSPH1	0.332	0.303	1.095	0.273	1.394	0.423	0.932	0.351	0.769	2.525
HSPG2	-0.391	0.297	-1.315	0.189	0.677	0.201	-1.609	0.108	0.378	1.211
HSPE1	0.347	0.298	1.167	0.243	1.415	0.421	0.986	0.324	0.79	2.536
HSPD1	0.543	0.307	1.77	0.077	1.721	0.528	1.366	0.172	0.943	3.14
HSPBP1	0.103	0.295	0.348	0.728	1.108	0.327	0.33	0.741	0.621	1.976
HSPBAP1	0.056	0.298	0.188	0.851	1.058	0.315	0.183	0.855	0.59	1.897
HSPB8	-0.096	0.296	-0.325	0.745	0.908	0.269	-0.342	0.733	0.508	1.623
HSPB7	-0.635	0.306	-2.076	0.038	0.53	0.162	-2.901	0.004	0.291	0.965
HSPB6	-0.113	0.296	-0.381	0.703	0.893	0.264	-0.404	0.687	0.5	1.595
HSPB3	0.07	0.295	0.237	0.813	1.072	0.317	0.229	0.819	0.601	1.913
HSPB2	0.015	0.295	0.05	0.96	1.015	0.3	0.05	0.96	0.569	1.81
HSPB11	-0.148	0.296	-0.501	0.617	0.862	0.255	-0.54	0.589	0.482	1.541
HSPB1	0.08	0.295	0.272	0.785	1.084	0.32	0.262	0.794	0.608	1.932
HSPA9	0.712	0.31	2.3	0.021	2.039	0.632	1.645	0.1	1.111	3.741
HSPA6	0.172	0.296	0.583	0.56	1.188	0.351	0.535	0.592	0.666	2.12
HSPA5	-0.215	0.296	-0.725	0.468	0.807	0.239	-0.809	0.419	0.451	1.442
HSPA4L	-0.152	0.296	-0.512	0.609	0.859	0.254	-0.553	0.58	0.481	1.535

HSPA4	-0.013	0.296	-0.043	0.965	0.987	0.292	-0.044	0.965	0.553	1.763
HSPA2	-0.487	0.302	-1.613	0.107	0.614	0.186	-2.078	0.038	0.34	1.111
HSPA1L	-0.318	0.299	-1.065	0.287	0.728	0.217	-1.253	0.21	0.405	1.306
HSPA14	0.66	0.303	2.176	0.03	1.935	0.587	1.593	0.111	1.068	3.507
HSPA13	0.201	0.296	0.68	0.497	1.223	0.361	0.616	0.538	0.685	2.182
HSPA12A	-0.242	0.297	-0.814	0.416	0.785	0.234	-0.921	0.357	0.438	1.406
HSP90B1	-0.677	0.31	-2.185	0.029	0.508	0.157	-3.124	0.002	0.277	0.933
HSP90AB1	0.174	0.297	0.586	0.558	1.19	0.354	0.538	0.591	0.665	2.132
HSP90AA1	0.679	0.31	2.192	0.028	1.972	0.611	1.591	0.112	1.075	3.618
HSF4	0.321	0.297	1.078	0.281	1.378	0.41	0.922	0.356	0.769	2.468
HSF2BP	0.383	0.3	1.278	0.201	1.467	0.439	1.062	0.288	0.815	2.639
HSF2	0.304	0.297	1.025	0.305	1.356	0.402	0.884	0.377	0.758	2.425
HSF1	0.299	0.3	0.996	0.319	1.348	0.405	0.861	0.389	0.749	2.428
HSDL2	0.01	0.295	0.034	0.973	1.01	0.298	0.034	0.973	0.567	1.801
HSD3B2	-0.324	0.3	-1.079	0.28	0.723	0.217	-1.275	0.202	0.402	1.302
HSD3B1	-0.048	0.296	-0.163	0.87	0.953	0.282	-0.167	0.867	0.534	1.701
HSD17B8	0.469	0.3	1.565	0.118	1.599	0.479	1.249	0.212	0.888	2.877
HSD17B7	0.213	0.296	0.721	0.471	1.238	0.367	0.649	0.516	0.693	2.212
HSD17B6	-0.29	0.3	-0.966	0.334	0.749	0.224	-1.12	0.263	0.416	1.347
HSD17B4	-0.457	0.307	-1.488	0.137	0.633	0.194	-1.887	0.059	0.347	1.156
HSD17B3	-0.128	0.296	-0.432	0.666	0.88	0.261	-0.461	0.645	0.492	1.572
HSD17B2	-0.291	0.296	-0.983	0.326	0.747	0.222	-1.141	0.254	0.418	1.336
HSD17B14	0.241	0.296	0.814	0.416	1.273	0.377	0.723	0.47	0.712	2.275
HSD17B12	0.079	0.296	0.267	0.79	1.082	0.32	0.257	0.798	0.606	1.932
HSD17B11	-0.471	0.3	-1.572	0.116	0.624	0.187	-2.008	0.045	0.347	1.123
HSD17B10	0.768	0.307	2.502	0.012	2.156	0.662	1.746	0.081	1.181	3.936
HSD17B1	0.625	0.306	2.044	0.041	1.868	0.571	1.52	0.129	1.026	3.4
HSD11B2	-0.13	0.296	-0.439	0.661	0.878	0.26	-0.469	0.639	0.492	1.569
HSD11B1	-0.338	0.298	-1.135	0.256	0.713	0.212	-1.351	0.177	0.398	1.279
HSBP1	0.687	0.304	2.261	0.024	1.987	0.603	1.636	0.102	1.096	3.603
HS6ST1	0.227	0.295	0.768	0.442	1.255	0.371	0.687	0.492	0.703	2.238
HS3ST3B1	0.462	0.3	1.541	0.123	1.587	0.475	1.234	0.217	0.882	2.855
HS3ST3A1	-0.612	0.306	-2.002	0.045	0.542	0.166	-2.762	0.006	0.298	0.987
HS3ST2	0.644	0.306	2.106	0.035	1.904	0.582	1.553	0.121	1.046	3.467
HS3ST1	0.41	0.3	1.368	0.171	1.507	0.452	1.122	0.262	0.837	2.714
HS2ST1	0.463	0.3	1.545	0.122	1.589	0.476	1.237	0.216	0.883	2.859
HS1BP3	0.019	0.296	0.065	0.948	1.019	0.301	0.064	0.949	0.571	1.82
HROB	0.269	0.296	0.908	0.364	1.309	0.388	0.796	0.426	0.732	2.339

HRK	0.188	0.296	0.635	0.526	1.207	0.357	0.579	0.563	0.675	2.156
HRH4	0.459	0.3	1.53	0.126	1.582	0.474	1.227	0.22	0.879	2.846
HRH3	0.277	0.3	0.924	0.355	1.32	0.396	0.807	0.42	0.733	2.377
HRH2	-0.19	0.296	-0.641	0.521	0.827	0.245	-0.706	0.48	0.463	1.478
HRH1	-0.132	0.295	-0.448	0.654	0.876	0.259	-0.479	0.632	0.491	1.563
HRG	-0.077	0.296	-0.259	0.796	0.926	0.274	-0.269	0.788	0.518	1.655
HRC	-0.431	0.298	-1.448	0.148	0.65	0.193	-1.81	0.07	0.363	1.165
HRAS	0.098	0.296	0.332	0.74	1.104	0.327	0.317	0.752	0.617	1.972
HR	0.474	0.302	1.567	0.117	1.606	0.485	1.248	0.212	0.888	2.903
HPX	0.065	0.295	0.22	0.826	1.067	0.315	0.213	0.831	0.598	1.904
HPSE2	0.85	0.316	2.691	0.007	2.34	0.739	1.812	0.07	1.26	4.346
HPSE	0.679	0.306	2.222	0.026	1.972	0.603	1.613	0.107	1.083	3.59
HPS6	0.503	0.302	1.664	0.096	1.654	0.5	1.307	0.191	0.914	2.991
HPS5	-0.394	0.301	-1.308	0.191	0.674	0.203	-1.603	0.109	0.374	1.217
HPS4	-0.224	0.296	-0.755	0.45	0.799	0.237	-0.846	0.397	0.447	1.429
HPS1	0.513	0.302	1.695	0.09	1.67	0.505	1.326	0.185	0.923	3.02
HPRT1	0.11	0.295	0.373	0.709	1.116	0.329	0.353	0.724	0.626	1.991
HPR	0.272	0.296	0.916	0.359	1.312	0.389	0.802	0.422	0.734	2.346
HPN	-0.525	0.311	-1.688	0.091	0.592	0.184	-2.219	0.026	0.322	1.088
HPGDS	-0.342	0.3	-1.139	0.255	0.711	0.213	-1.358	0.174	0.395	1.279
HPGD	-0.647	0.306	-2.112	0.035	0.524	0.16	-2.969	0.003	0.287	0.955
HPF1	0.303	0.297	1.022	0.307	1.354	0.402	0.882	0.378	0.757	2.422
HPD	-0.383	0.298	-1.284	0.199	0.682	0.203	-1.565	0.118	0.38	1.223
HPCAL4	0.062	0.295	0.209	0.835	1.063	0.314	0.202	0.84	0.596	1.896
HPCAL1	0.062	0.295	0.209	0.834	1.064	0.314	0.203	0.839	0.596	1.898
HPCA	-0.022	0.295	-0.073	0.941	0.979	0.289	-0.074	0.941	0.548	1.746
HP1BP3	-0.607	0.31	-1.959	0.05	0.545	0.169	-2.696	0.007	0.297	1
HP	-0.021	0.295	-0.072	0.942	0.979	0.289	-0.073	0.942	0.549	1.746
HOXD9	-0.413	0.3	-1.378	0.168	0.662	0.198	-1.706	0.088	0.368	1.191
HOXD4	-0.476	0.3	-1.589	0.112	0.621	0.186	-2.035	0.042	0.345	1.118
HOXD13	-0.106	0.296	-0.36	0.719	0.899	0.266	-0.38	0.704	0.504	1.605
HOXD12	-0.002	0.295	-0.005	0.996	0.998	0.295	-0.005	0.996	0.56	1.782
HOXD11	0.257	0.298	0.862	0.388	1.293	0.385	0.761	0.447	0.721	2.317
HOXD10	-0.039	0.295	-0.132	0.895	0.962	0.284	-0.134	0.893	0.539	1.716
HOXD1	-0.061	0.296	-0.207	0.836	0.941	0.279	-0.213	0.831	0.526	1.682
HOXC8	0.547	0.306	1.789	0.074	1.728	0.528	1.378	0.168	0.949	3.145
HOXC6	0.04	0.298	0.133	0.894	1.04	0.31	0.13	0.896	0.581	1.864
HOXC5	-0.002	0.295	-0.007	0.994	0.998	0.295	-0.007	0.994	0.56	1.78

HOXC4	0.406	0.303	1.341	0.18	1.501	0.455	1.102	0.271	0.829	2.719
HOXC13	0.541	0.302	1.791	0.073	1.719	0.52	1.383	0.167	0.95	3.108
HOXC11	0.23	0.298	0.77	0.441	1.258	0.375	0.688	0.491	0.701	2.258
HOXC10	-0.304	0.297	-1.023	0.306	0.738	0.219	-1.196	0.232	0.412	1.321
HOXB9	0.336	0.3	1.121	0.262	1.4	0.42	0.952	0.341	0.778	2.518
HOXB8	0.049	0.296	0.165	0.869	1.05	0.31	0.161	0.872	0.588	1.874
HOXB7	0.086	0.295	0.29	0.771	1.09	0.322	0.278	0.781	0.611	1.944
HOXB6	0.302	0.298	1.014	0.31	1.352	0.402	0.876	0.381	0.755	2.423
HOXB5	0.106	0.297	0.357	0.721	1.112	0.33	0.339	0.735	0.622	1.988
HOXB3	-0.201	0.296	-0.678	0.498	0.818	0.242	-0.751	0.453	0.458	1.462
HOXB2	0.206	0.296	0.697	0.486	1.229	0.364	0.629	0.529	0.688	2.196
HOXB13	-0.003	0.295	-0.01	0.992	0.997	0.295	-0.01	0.992	0.559	1.779
HOXB1	0.327	0.3	1.092	0.275	1.387	0.416	0.931	0.352	0.771	2.496
HOXA7	-0.091	0.296	-0.308	0.758	0.913	0.27	-0.323	0.747	0.511	1.631
HOXA6	0.297	0.297	1.001	0.317	1.346	0.399	0.866	0.387	0.752	2.408
HOXA5	-0.163	0.296	-0.55	0.582	0.85	0.251	-0.598	0.55	0.476	1.517
HOXA4	0.018	0.295	0.06	0.952	1.018	0.301	0.06	0.952	0.57	1.817
HOXA3	-0.118	0.299	-0.395	0.693	0.889	0.265	-0.419	0.675	0.495	1.596
HOXA2	0.239	0.298	0.804	0.421	1.271	0.378	0.715	0.474	0.709	2.277
HOXA11	-0.241	0.297	-0.81	0.418	0.786	0.234	-0.916	0.359	0.439	1.407
HOXA10	-0.043	0.295	-0.144	0.885	0.958	0.283	-0.147	0.883	0.537	1.71
HOXA1	-0.174	0.298	-0.584	0.559	0.84	0.251	-0.638	0.523	0.468	1.507
HOPX	-0.344	0.298	-1.155	0.248	0.709	0.211	-1.378	0.168	0.396	1.271
HOOK2	-0.175	0.296	-0.592	0.554	0.839	0.248	-0.648	0.517	0.47	1.499
HOOK1	0.502	0.3	1.672	0.095	1.652	0.496	1.315	0.189	0.917	2.975
HOMER3	-0.191	0.298	-0.642	0.521	0.826	0.246	-0.707	0.479	0.46	1.482
HOMER2	-0.09	0.296	-0.303	0.762	0.914	0.271	-0.317	0.752	0.511	1.634
HOMER1	-0.08	0.295	-0.269	0.788	0.924	0.273	-0.28	0.779	0.518	1.647
HNRNPUL2	-0.007	0.3	-0.022	0.982	0.993	0.298	-0.022	0.982	0.551	1.79
HNRNPUL1	0.305	0.298	1.026	0.305	1.357	0.404	0.884	0.377	0.757	2.433
HNRNPU	0.35	0.299	1.173	0.241	1.419	0.424	0.99	0.322	0.791	2.548
HNRNPR	-0.027	0.296	-0.091	0.927	0.973	0.289	-0.092	0.926	0.544	1.74
HNRNPM	-0.009	0.295	-0.029	0.977	0.991	0.293	-0.029	0.977	0.556	1.768
HNRNPL	0.652	0.31	2.104	0.035	1.92	0.595	1.545	0.122	1.046	3.525
HNRNPK	-0.132	0.295	-0.448	0.654	0.876	0.259	-0.48	0.632	0.491	1.563
HNRNPH3	0.411	0.3	1.372	0.17	1.508	0.452	1.125	0.261	0.838	2.714
HNRNPH1	0.394	0.3	1.314	0.189	1.483	0.445	1.086	0.277	0.824	2.669
HNRNPF	-0.367	0.303	-1.212	0.226	0.693	0.21	-1.464	0.143	0.382	1.255

HNRNPDL	0.571	0.306	1.869	0.062	1.771	0.541	1.424	0.155	0.973	3.224
HNRNPD	0.226	0.298	0.757	0.449	1.253	0.374	0.678	0.498	0.699	2.249
HNRNPC	0.275	0.297	0.927	0.354	1.317	0.391	0.811	0.418	0.736	2.357
HNRNPAB	0.385	0.298	1.295	0.195	1.47	0.437	1.075	0.283	0.82	2.634
HNRNPA3P1	0.027	0.295	0.091	0.927	1.027	0.303	0.09	0.928	0.576	1.832
HNRNPA3	-0.324	0.298	-1.088	0.277	0.723	0.215	-1.284	0.199	0.404	1.297
HNRNPA2B1	0.747	0.315	2.37	0.018	2.11	0.665	1.67	0.095	1.138	3.912
HNRNPA1	0.251	0.298	0.843	0.399	1.285	0.383	0.746	0.456	0.717	2.303
HNRNPA0	-0.203	0.298	-0.681	0.496	0.816	0.243	-0.755	0.45	0.455	1.464
HNMT	-0.712	0.31	-2.298	0.022	0.491	0.152	-3.351	0.001	0.267	0.9
HNF4G	-0.344	0.298	-1.155	0.248	0.709	0.211	-1.379	0.168	0.395	1.271
HNF4A	0.095	0.295	0.322	0.747	1.1	0.325	0.307	0.759	0.617	1.962
HNF1B	0.2	0.298	0.67	0.503	1.221	0.364	0.607	0.544	0.681	2.189
HNF1A	0.283	0.298	0.95	0.342	1.327	0.396	0.827	0.408	0.74	2.38
HMX1	0.028	0.295	0.096	0.923	1.029	0.304	0.095	0.925	0.577	1.835
HMOX2	-0.409	0.303	-1.351	0.177	0.664	0.201	-1.67	0.095	0.367	1.202
HMOX1	0.329	0.298	1.103	0.27	1.389	0.414	0.94	0.347	0.775	2.491
HMMR	0.353	0.3	1.177	0.239	1.423	0.427	0.991	0.321	0.791	2.561
HMHB1	0.479	0.302	1.585	0.113	1.614	0.488	1.259	0.208	0.893	2.918
HMGXB4	-0.141	0.296	-0.476	0.634	0.869	0.257	-0.511	0.609	0.486	1.552
HMGXB3	0.404	0.3	1.346	0.178	1.497	0.449	1.108	0.268	0.832	2.694
HMGN5	0.53	0.302	1.752	0.08	1.699	0.514	1.36	0.174	0.939	3.073
HMGN4	0.074	0.295	0.249	0.803	1.076	0.318	0.24	0.81	0.603	1.92
HMGN3	0.288	0.3	0.961	0.336	1.334	0.401	0.835	0.404	0.741	2.403
HMGN2	0.42	0.3	1.403	0.161	1.523	0.456	1.145	0.252	0.846	2.739
HMGN1	0.995	0.318	3.133	0.002	2.706	0.86	1.984	0.047	1.452	5.044
HMGCS2	-0.297	0.298	-0.996	0.319	0.743	0.221	-1.159	0.246	0.415	1.333
HMGCS1	1.044	0.321	3.251	0.001	2.841	0.913	2.017	0.044	1.514	5.332
HMGCR	-0.01	0.295	-0.035	0.972	0.99	0.292	-0.036	0.972	0.555	1.765
HMGCL	-0.042	0.298	-0.142	0.887	0.959	0.286	-0.145	0.885	0.535	1.719
HMGB3P1	0.551	0.302	1.821	0.069	1.735	0.525	1.4	0.161	0.959	3.138
HMGB3	0.682	0.306	2.231	0.026	1.978	0.605	1.617	0.106	1.086	3.602
HMGB2	0.547	0.302	1.809	0.07	1.728	0.522	1.394	0.163	0.956	3.125
HMGB1	0.243	0.298	0.815	0.415	1.275	0.38	0.724	0.469	0.711	2.287
HMGA2	0.006	0.295	0.02	0.984	1.006	0.297	0.02	0.984	0.564	1.794
HMGA1	0.397	0.3	1.325	0.185	1.488	0.446	1.093	0.274	0.826	2.678
HMG20B	-0.571	0.302	-1.889	0.059	0.565	0.171	-2.547	0.011	0.312	1.022
HMG20A	-0.177	0.297	-0.596	0.551	0.838	0.248	-0.652	0.514	0.469	1.498

HMCES	0.875	0.315	2.777	0.005	2.398	0.755	1.851	0.064	1.294	4.446
HMBS	0.246	0.296	0.831	0.406	1.279	0.379	0.737	0.461	0.716	2.286
HMBOX1	0.189	0.298	0.636	0.525	1.208	0.36	0.579	0.562	0.674	2.166
HLX	0.074	0.295	0.252	0.801	1.077	0.318	0.243	0.808	0.604	1.922
HLTF	0.977	0.321	3.047	0.002	2.656	0.851	1.945	0.052	1.417	4.978
HLF	-0.461	0.3	-1.539	0.124	0.631	0.189	-1.956	0.051	0.35	1.134
HLCS	-0.455	0.306	-1.487	0.137	0.635	0.194	-1.882	0.06	0.348	1.156
HLA-J	-0.435	0.3	-1.45	0.147	0.647	0.194	-1.817	0.069	0.359	1.165
HLA-G	-0.017	0.295	-0.059	0.953	0.983	0.29	-0.059	0.953	0.551	1.753
HLA-F-AS1	-0.043	0.295	-0.147	0.883	0.958	0.283	-0.15	0.881	0.537	1.708
HLA-F	-0.02	0.295	-0.069	0.945	0.98	0.29	-0.07	0.944	0.549	1.748
HLA-E	-0.234	0.296	-0.79	0.429	0.791	0.235	-0.89	0.373	0.443	1.415
HLA-DRB6	0.419	0.3	1.398	0.162	1.52	0.456	1.142	0.253	0.845	2.736
HLA-DRB4	-0.233	0.297	-0.784	0.433	0.793	0.235	-0.882	0.378	0.443	1.418
HLA-DRA	-0.402	0.3	-1.338	0.181	0.669	0.201	-1.647	0.1	0.371	1.205
HLA-DQB2	-0.577	0.303	-1.904	0.057	0.562	0.17	-2.576	0.01	0.31	1.017
HLA-DQB1	-0.809	0.31	-2.606	0.009	0.445	0.138	-4.012	0	0.242	0.818
HLA-DQA1	-0.39	0.298	-1.308	0.191	0.677	0.202	-1.6	0.11	0.378	1.214
HLA-DPB1	-0.258	0.296	-0.872	0.383	0.772	0.229	-0.995	0.32	0.432	1.38
HLA-DPA1	-0.985	0.321	-3.07	0.002	0.374	0.12	-5.228	0	0.199	0.701
HLA-DOB	-0.507	0.302	-1.675	0.094	0.603	0.182	-2.181	0.029	0.333	1.09
HLA-DOA	-0.352	0.298	-1.181	0.238	0.703	0.21	-1.415	0.157	0.392	1.261
HLA-DMB	-0.328	0.3	-1.094	0.274	0.72	0.216	-1.295	0.195	0.4	1.297
HLA-DMA	-0.627	0.306	-2.05	0.04	0.534	0.163	-2.851	0.004	0.293	0.973
HLA-C	-0.208	0.297	-0.702	0.482	0.812	0.241	-0.781	0.435	0.454	1.452
HLA-B	-0.026	0.296	-0.086	0.931	0.975	0.289	-0.087	0.93	0.546	1.742
HLA-A	-0.021	0.295	-0.071	0.943	0.979	0.289	-0.072	0.943	0.549	1.747
HKDC1	-1.019	0.328	-3.105	0.002	0.361	0.118	-5.397	0	0.19	0.687
HK3	0.3	0.3	0.999	0.318	1.35	0.405	0.863	0.388	0.749	2.432
HK2	0.244	0.297	0.821	0.412	1.276	0.379	0.728	0.466	0.713	2.284
HK1	-0.459	0.3	-1.527	0.127	0.632	0.19	-1.938	0.053	0.351	1.139
HJURP	0.636	0.306	2.081	0.037	1.889	0.577	1.54	0.124	1.038	3.438
HIVEP3	0.046	0.296	0.156	0.876	1.047	0.31	0.153	0.879	0.586	1.872
HIVEP2	-0.459	0.3	-1.531	0.126	0.632	0.189	-1.944	0.052	0.351	1.137
HIVEP1	0.196	0.298	0.656	0.512	1.216	0.362	0.596	0.551	0.678	2.18
HIRIP3	-0.077	0.295	-0.262	0.794	0.926	0.273	-0.272	0.786	0.519	1.651
HIRA	0.385	0.298	1.291	0.197	1.469	0.438	1.072	0.284	0.819	2.636
HIPK3	-0.85	0.315	-2.697	0.007	0.427	0.135	-4.252	0	0.23	0.793

HIPK2	-0.094	0.296	-0.319	0.75	0.91	0.269	-0.335	0.738	0.509	1.626
HIPK1	0.081	0.296	0.272	0.785	1.084	0.321	0.262	0.794	0.607	1.937
HIP1R	0.064	0.296	0.217	0.828	1.066	0.315	0.211	0.833	0.597	1.904
HIP1	-0.095	0.297	-0.321	0.748	0.909	0.27	-0.337	0.736	0.508	1.627
HINT1	0.154	0.297	0.519	0.604	1.166	0.346	0.481	0.631	0.652	2.086
HINFP	0.103	0.295	0.349	0.727	1.109	0.327	0.332	0.74	0.622	1.978
HILPDA	0.535	0.302	1.772	0.076	1.708	0.516	1.372	0.17	0.945	3.088
HIKESHI	0.142	0.295	0.479	0.632	1.152	0.34	0.447	0.655	0.646	2.056
HIGD2A	0.232	0.297	0.782	0.434	1.261	0.374	0.698	0.485	0.705	2.256
HIGD1B	0	0.296	0.002	0.999	1	0.297	0.002	0.999	0.56	1.789
HIGD1A	-0.115	0.297	-0.388	0.698	0.891	0.264	-0.411	0.681	0.498	1.594
HIF3A	0.28	0.297	0.944	0.345	1.323	0.393	0.823	0.411	0.74	2.367
HIF1AN	0.81	0.31	2.611	0.009	2.247	0.697	1.79	0.074	1.224	4.126
HIF1A	0.018	0.295	0.063	0.95	1.019	0.301	0.062	0.951	0.571	1.817
HIC2	0.446	0.3	1.483	0.138	1.561	0.469	1.197	0.231	0.866	2.813
HIC1	0.244	0.296	0.824	0.41	1.277	0.378	0.731	0.465	0.714	2.282
HIBCH	0.053	0.295	0.179	0.858	1.054	0.312	0.175	0.861	0.591	1.881
HHLA3	-0.074	0.297	-0.248	0.804	0.929	0.276	-0.257	0.797	0.519	1.663
HHLA2	-0.114	0.295	-0.385	0.701	0.893	0.264	-0.407	0.684	0.5	1.592
HHLA1	-0.209	0.297	-0.705	0.481	0.811	0.241	-0.784	0.433	0.454	1.451
HHIPL2	0.236	0.296	0.797	0.426	1.266	0.375	0.71	0.478	0.709	2.262
HHEX	-0.007	0.295	-0.025	0.98	0.993	0.293	-0.025	0.98	0.557	1.77
HHAT	-0.267	0.301	-0.888	0.375	0.766	0.23	-1.017	0.309	0.425	1.381
HGSNAT	-0.694	0.31	-2.239	0.025	0.499	0.155	-3.232	0.001	0.272	0.917
HGS	-0.029	0.295	-0.097	0.923	0.972	0.287	-0.098	0.922	0.545	1.733
HGH1	0.671	0.31	2.168	0.03	1.957	0.606	1.579	0.114	1.067	3.591
HGFAC	-0.205	0.297	-0.691	0.49	0.815	0.242	-0.767	0.443	0.456	1.457
HGF	-0.254	0.298	-0.854	0.393	0.776	0.231	-0.972	0.331	0.433	1.39
HGD	-0.405	0.299	-1.353	0.176	0.667	0.2	-1.667	0.095	0.371	1.199
HGC6.3	0.606	0.306	1.984	0.047	1.834	0.561	1.487	0.137	1.007	3.338
HFE	-0.216	0.297	-0.728	0.467	0.806	0.239	-0.813	0.416	0.451	1.441
HEYL	0.429	0.3	1.429	0.153	1.535	0.461	1.163	0.245	0.853	2.764
HEY2	0.103	0.295	0.35	0.726	1.109	0.328	0.332	0.74	0.622	1.978
HEY1	0.186	0.296	0.627	0.531	1.204	0.356	0.572	0.567	0.674	2.151
HEXIM1	-0.2	0.296	-0.675	0.5	0.819	0.243	-0.747	0.455	0.458	1.463
HEXB	-0.55	0.306	-1.8	0.072	0.577	0.176	-2.4	0.016	0.317	1.05
HEXA-AS1	-0.082	0.295	-0.279	0.78	0.921	0.272	-0.291	0.771	0.516	1.643
HEXA	-0.065	0.296	-0.219	0.826	0.937	0.277	-0.227	0.821	0.525	1.673

HESX1	0.088	0.296	0.295	0.768	1.091	0.323	0.283	0.777	0.611	1.951
HES2	0.162	0.296	0.546	0.585	1.176	0.348	0.504	0.614	0.658	2.102
HES1	0.145	0.296	0.488	0.625	1.156	0.342	0.455	0.649	0.647	2.064
HERPUD1	-0.231	0.296	-0.78	0.435	0.794	0.235	-0.877	0.38	0.444	1.418
HERC6	0.252	0.298	0.847	0.397	1.287	0.383	0.748	0.454	0.718	2.307
HERC5	0.598	0.306	1.956	0.05	1.818	0.556	1.472	0.141	0.999	3.311
HERC4	0.086	0.295	0.291	0.771	1.09	0.322	0.278	0.781	0.611	1.944
HERC2P3	-0.357	0.3	-1.191	0.234	0.7	0.21	-1.431	0.152	0.389	1.259
HERC2	0.094	0.296	0.318	0.751	1.098	0.325	0.303	0.762	0.615	1.961
HERC1	-0.32	0.3	-1.065	0.287	0.726	0.218	-1.255	0.21	0.403	1.308
HEPH	0.035	0.296	0.117	0.907	1.035	0.307	0.115	0.908	0.579	1.85
HEMK1	-0.192	0.296	-0.648	0.517	0.825	0.244	-0.714	0.475	0.462	1.475
HELZ	-0.359	0.297	-1.207	0.227	0.698	0.208	-1.453	0.146	0.39	1.251
HELLS	1.033	0.321	3.216	0.001	2.809	0.902	2.005	0.045	1.497	5.27
HEG1	-0.783	0.307	-2.555	0.011	0.457	0.14	-3.877	0	0.251	0.833
HECW1	0.02	0.296	0.067	0.946	1.02	0.302	0.067	0.947	0.571	1.823
HECTD4	-0.271	0.297	-0.913	0.361	0.763	0.226	-1.049	0.294	0.426	1.364
HECTD3	-0.438	0.3	-1.46	0.144	0.646	0.194	-1.832	0.067	0.359	1.162
HECA	-0.351	0.299	-1.174	0.241	0.704	0.211	-1.406	0.16	0.392	1.265
HEBP2	0.182	0.296	0.613	0.54	1.199	0.355	0.561	0.575	0.671	2.144
HEBP1	-0.832	0.321	-2.593	0.01	0.435	0.14	-4.046	0	0.232	0.816
HEATR6	0.196	0.296	0.661	0.509	1.216	0.36	0.6	0.548	0.681	2.173
HEATR3	0.131	0.296	0.442	0.659	1.14	0.338	0.414	0.679	0.638	2.037
HEATR1	0.596	0.31	1.92	0.055	1.815	0.563	1.446	0.148	0.988	3.334
HDLBP	-0.573	0.303	-1.892	0.059	0.564	0.171	-2.554	0.011	0.312	1.021
HDHD5	0.547	0.302	1.809	0.07	1.728	0.523	1.393	0.164	0.955	3.126
HDHD3	-0.23	0.298	-0.773	0.44	0.795	0.237	-0.869	0.385	0.443	1.424
HDGFL3	-0.663	0.316	-2.096	0.036	0.516	0.163	-2.973	0.003	0.277	0.958
HDGF	0.187	0.296	0.632	0.528	1.206	0.357	0.576	0.565	0.675	2.155
HDDC2	0.297	0.3	0.989	0.322	1.345	0.403	0.856	0.392	0.748	2.421
HDC	-0.091	0.295	-0.307	0.759	0.913	0.27	-0.321	0.748	0.512	1.63
HDAC9	0.267	0.297	0.9	0.368	1.306	0.388	0.79	0.429	0.73	2.337
HDAC7	-0.564	0.302	-1.865	0.062	0.569	0.172	-2.505	0.012	0.315	1.029
HDAC6	0.085	0.297	0.285	0.776	1.088	0.323	0.273	0.785	0.608	1.947
HDAC5	-0.353	0.298	-1.186	0.236	0.702	0.209	-1.423	0.155	0.392	1.259
HDAC4	-0.013	0.296	-0.044	0.965	0.987	0.293	-0.044	0.965	0.552	1.765
HDAC3	-0.279	0.297	-0.938	0.348	0.756	0.225	-1.083	0.279	0.422	1.355
HDAC2	0.448	0.302	1.482	0.138	1.565	0.473	1.194	0.232	0.865	2.832

HDAC11	-0.109	0.3	-0.361	0.718	0.897	0.269	-0.382	0.703	0.498	1.616
HDAC1	-0.101	0.297	-0.339	0.734	0.904	0.268	-0.357	0.721	0.505	1.618
HCRTR2	-0.071	0.297	-0.24	0.81	0.931	0.276	-0.249	0.803	0.521	1.665
HCRTR1	0.114	0.297	0.385	0.7	1.121	0.333	0.364	0.716	0.626	2.008
HCRT	-0.07	0.296	-0.238	0.812	0.932	0.275	-0.247	0.805	0.522	1.663
HCP5	-0.067	0.296	-0.227	0.82	0.935	0.277	-0.235	0.814	0.523	1.67
HCN4	0.033	0.296	0.111	0.911	1.033	0.305	0.109	0.913	0.579	1.845
HCN2	0.253	0.296	0.854	0.393	1.288	0.382	0.755	0.45	0.721	2.303
HCLS1	-0.531	0.303	-1.754	0.079	0.588	0.178	-2.314	0.021	0.325	1.065
HCK	-0.234	0.296	-0.791	0.429	0.791	0.234	-0.892	0.373	0.443	1.414
HCG9	-0.26	0.298	-0.874	0.382	0.771	0.229	-0.998	0.318	0.43	1.381
HCG4B	0.181	0.297	0.611	0.541	1.199	0.356	0.558	0.577	0.67	2.146
HCG4	0.143	0.295	0.483	0.629	1.153	0.341	0.45	0.653	0.646	2.058
HCG26	0.206	0.296	0.697	0.486	1.229	0.364	0.629	0.529	0.688	2.198
HCG18	0.039	0.296	0.132	0.895	1.04	0.307	0.129	0.897	0.583	1.855
HCFC2	0.416	0.299	1.393	0.164	1.516	0.453	1.14	0.254	0.844	2.721
HCFC1R1	-0.395	0.3	-1.318	0.187	0.673	0.202	-1.617	0.106	0.374	1.212
HCFC1	0.138	0.3	0.461	0.645	1.149	0.345	0.43	0.667	0.637	2.07
HCCS	0.818	0.31	2.642	0.008	2.267	0.702	1.804	0.071	1.235	4.16
HCAR3	-0.679	0.306	-2.216	0.027	0.507	0.155	-3.171	0.002	0.278	0.925
HBZ	0.177	0.296	0.597	0.55	1.193	0.353	0.547	0.584	0.668	2.132
HBS1L	0.113	0.296	0.382	0.702	1.12	0.331	0.362	0.718	0.627	1.999
HBQ1	0.301	0.298	1.012	0.312	1.351	0.402	0.874	0.382	0.754	2.422
HBP1	-0.472	0.302	-1.561	0.119	0.624	0.189	-1.994	0.046	0.345	1.128
HBEGF	-0.02	0.296	-0.067	0.946	0.98	0.29	-0.068	0.946	0.549	1.751
HBE1	0.084	0.297	0.283	0.778	1.087	0.323	0.271	0.786	0.608	1.946
HBD	0.251	0.297	0.846	0.398	1.286	0.382	0.748	0.455	0.718	2.302
HBBP1	-0.521	0.303	-1.722	0.085	0.594	0.18	-2.261	0.024	0.328	1.075
HBB	0.071	0.295	0.241	0.809	1.074	0.317	0.233	0.816	0.602	1.914
HAX1	-0.358	0.298	-1.201	0.23	0.699	0.208	-1.444	0.149	0.39	1.254
HAVCR1	0.019	0.295	0.065	0.948	1.019	0.301	0.065	0.949	0.572	1.818
HAUS7	0.445	0.3	1.486	0.137	1.561	0.467	1.199	0.231	0.868	2.807
HAUS6	0.254	0.297	0.854	0.393	1.289	0.384	0.755	0.451	0.72	2.31
HAUS5	0.203	0.299	0.679	0.497	1.225	0.367	0.615	0.539	0.682	2.203
HAUS3	0.669	0.306	2.189	0.029	1.953	0.597	1.596	0.111	1.073	3.557
HAUS2	0.614	0.31	1.981	0.048	1.848	0.573	1.48	0.139	1.006	3.393
HAT1	-0.079	0.296	-0.267	0.789	0.924	0.274	-0.278	0.781	0.517	1.652
HAS2	-0.947	0.321	-2.952	0.003	0.388	0.124	-4.92	0	0.207	0.727

HAS1	-0.406	0.3	-1.353	0.176	0.666	0.2	-1.668	0.095	0.37	1.2
HARS2	0.127	0.295	0.429	0.668	1.135	0.335	0.403	0.687	0.636	2.025
HARS1	-0.69	0.306	-2.252	0.024	0.502	0.154	-3.243	0.001	0.275	0.914
HAPLN2	0.074	0.297	0.248	0.804	1.077	0.319	0.24	0.811	0.602	1.926
HAPLN1	0.186	0.3	0.62	0.535	1.204	0.361	0.566	0.571	0.669	2.168
HAP1	0.184	0.296	0.621	0.535	1.202	0.356	0.567	0.571	0.673	2.147
HAO2	-0.696	0.306	-2.271	0.023	0.499	0.153	-3.281	0.001	0.274	0.909
HAO1	-0.251	0.297	-0.844	0.399	0.778	0.231	-0.96	0.337	0.435	1.393
HAND2-AS1	0.15	0.296	0.508	0.611	1.162	0.344	0.472	0.637	0.651	2.077
HAND2	-0.212	0.298	-0.713	0.476	0.809	0.241	-0.795	0.427	0.451	1.449
HAND1	0.05	0.295	0.17	0.865	1.051	0.31	0.166	0.868	0.589	1.876
HAMP	1.155	0.324	3.566	0	3.175	1.029	2.115	0.034	1.683	5.991
HAL	-0.045	0.297	-0.153	0.879	0.956	0.284	-0.156	0.876	0.533	1.712
HAGH	-0.149	0.299	-0.499	0.618	0.862	0.257	-0.538	0.591	0.48	1.547
HADHB	-0.25	0.3	-0.834	0.404	0.779	0.234	-0.948	0.343	0.432	1.402
HADHA	-0.461	0.302	-1.524	0.128	0.631	0.191	-1.936	0.053	0.349	1.141
HADH	0.515	0.303	1.702	0.089	1.673	0.506	1.33	0.184	0.925	3.028
HACD3	-0.504	0.306	-1.648	0.099	0.604	0.185	-2.142	0.032	0.332	1.1
HACD2	0.492	0.306	1.605	0.108	1.635	0.501	1.268	0.205	0.897	2.981
HACD1	0.223	0.296	0.754	0.451	1.25	0.37	0.676	0.499	0.7	2.234
HABP4	-0.286	0.302	-0.949	0.343	0.751	0.227	-1.099	0.272	0.416	1.357
HABP2	-0.039	0.295	-0.132	0.895	0.962	0.284	-0.135	0.893	0.539	1.715
HAAO	-0.212	0.296	-0.715	0.475	0.809	0.24	-0.796	0.426	0.453	1.446
H6PD	-1.34	0.338	-3.967	0	0.262	0.088	-8.347	0	0.135	0.508
H4C9	0.9	0.315	2.854	0.004	2.46	0.776	1.882	0.06	1.326	4.563
H4C8	0.006	0.296	0.02	0.984	1.006	0.298	0.02	0.984	0.563	1.796
H4C7	0.234	0.296	0.789	0.43	1.263	0.374	0.704	0.482	0.707	2.258
H4C5	0.05	0.295	0.17	0.865	1.051	0.31	0.166	0.868	0.59	1.875
H4C4	0.406	0.3	1.356	0.175	1.502	0.45	1.115	0.265	0.835	2.701
H4C13	-0.753	0.315	-2.393	0.017	0.471	0.148	-3.57	0	0.254	0.873
H4C1	-0.006	0.295	-0.022	0.982	0.994	0.293	-0.022	0.982	0.557	1.773
H3C8	0.502	0.302	1.662	0.097	1.653	0.5	1.306	0.192	0.914	2.99
H3C7	1.189	0.337	3.533	0	3.284	1.105	2.066	0.039	1.698	6.352
H3C6	-0.098	0.296	-0.33	0.742	0.907	0.268	-0.346	0.729	0.508	1.62
H3C3	0.184	0.296	0.62	0.535	1.202	0.356	0.566	0.571	0.672	2.147
H3C2	0.675	0.304	2.222	0.026	1.964	0.597	1.615	0.106	1.083	3.564
H3C11	0.27	0.298	0.909	0.363	1.311	0.39	0.796	0.426	0.731	2.348
H3C1	0.055	0.295	0.188	0.851	1.057	0.312	0.183	0.855	0.592	1.886

H3-4	-0.308	0.298	-1.035	0.301	0.735	0.219	-1.212	0.225	0.41	1.317
H3-3A	0.912	0.316	2.889	0.004	2.49	0.786	1.895	0.058	1.341	4.624
H2BS1	-0.03	0.295	-0.102	0.919	0.97	0.287	-0.103	0.918	0.544	1.731
H2BC9	0.273	0.296	0.922	0.356	1.314	0.389	0.807	0.42	0.735	2.348
H2BC6	0.326	0.298	1.094	0.274	1.385	0.412	0.934	0.35	0.773	2.481
H2BC5	0.295	0.296	0.996	0.319	1.343	0.398	0.862	0.389	0.751	2.402
H2BC3	0.004	0.295	0.012	0.99	1.004	0.296	0.012	0.99	0.563	1.789
H2BC21	-0.208	0.298	-0.7	0.484	0.812	0.242	-0.778	0.436	0.453	1.455
H2BC17	-0.138	0.296	-0.466	0.641	0.871	0.258	-0.5	0.617	0.488	1.556
H2BC15	0.272	0.296	0.919	0.358	1.313	0.389	0.804	0.421	0.735	2.347
H2BC14	0.145	0.295	0.491	0.623	1.156	0.342	0.457	0.648	0.648	2.063
H2BC13	-0.136	0.296	-0.46	0.646	0.873	0.259	-0.493	0.622	0.488	1.56
H2BC12	0.32	0.298	1.074	0.283	1.377	0.41	0.919	0.358	0.768	2.467
H2BC11	0.27	0.296	0.912	0.362	1.31	0.388	0.799	0.424	0.733	2.342
H2AZ2	0.743	0.315	2.358	0.018	2.102	0.662	1.664	0.096	1.133	3.896
H2AZ1	0.779	0.31	2.515	0.012	2.179	0.675	1.747	0.081	1.187	4
H2AX	0.5	0.3	1.666	0.096	1.648	0.494	1.312	0.19	0.916	2.966
H2AW	0.02	0.295	0.067	0.947	1.02	0.301	0.066	0.947	0.572	1.818
H2AP	-0.185	0.296	-0.626	0.531	0.831	0.246	-0.688	0.492	0.465	1.485
H2AJ	-0.258	0.298	-0.868	0.386	0.772	0.23	-0.99	0.322	0.431	1.385
H2AC8	-0.062	0.295	-0.208	0.835	0.94	0.278	-0.215	0.83	0.527	1.678
H2AC6	0.071	0.296	0.24	0.81	1.074	0.317	0.232	0.816	0.602	1.916
H2AC4	-0.172	0.296	-0.582	0.561	0.842	0.249	-0.635	0.525	0.471	1.504
H2AC18	0.241	0.296	0.813	0.416	1.272	0.377	0.722	0.47	0.712	2.274
H2AC17	0.344	0.298	1.155	0.248	1.41	0.42	0.977	0.328	0.787	2.526
H2AC16	0.242	0.297	0.815	0.415	1.274	0.378	0.724	0.469	0.712	2.279
H2AC15	0.367	0.298	1.232	0.218	1.443	0.429	1.031	0.302	0.805	2.586
H2AC14	0.51	0.303	1.683	0.092	1.665	0.504	1.319	0.187	0.92	3.013
H1-6	0.795	0.315	2.523	0.012	2.213	0.697	1.741	0.082	1.194	4.103
H1-5	0.735	0.307	2.398	0.016	2.086	0.639	1.698	0.089	1.144	3.803
H1-4	0.688	0.305	2.255	0.024	1.99	0.608	1.63	0.103	1.094	3.62
H1-3	0.339	0.298	1.139	0.255	1.404	0.418	0.966	0.334	0.783	2.517
H1-2	0.547	0.3	1.821	0.069	1.728	0.519	1.403	0.161	0.959	3.113
H1-10	0.15	0.296	0.508	0.612	1.162	0.344	0.472	0.637	0.651	2.076
H1-1	0.258	0.296	0.871	0.384	1.295	0.384	0.768	0.443	0.724	2.314
H1-0	-0.448	0.3	-1.494	0.135	0.639	0.192	-1.885	0.059	0.355	1.15
GZMM	-0.107	0.295	-0.362	0.717	0.899	0.265	-0.382	0.702	0.504	1.603
GZMK	-0.333	0.298	-1.115	0.265	0.717	0.214	-1.323	0.186	0.4	1.287

GZMH	0.163	0.296	0.549	0.583	1.177	0.349	0.507	0.612	0.659	2.103
GZMB	0.216	0.296	0.73	0.465	1.242	0.368	0.657	0.511	0.695	2.219
GZMA	-0.302	0.297	-1.016	0.309	0.74	0.22	-1.186	0.235	0.413	1.323
GYS2	0.46	0.3	1.536	0.125	1.584	0.475	1.231	0.218	0.881	2.85
GYS1	-0.056	0.295	-0.191	0.848	0.945	0.279	-0.197	0.844	0.53	1.686
GYPE	0.364	0.298	1.221	0.222	1.439	0.429	1.024	0.306	0.802	2.58
GYPC	-0.363	0.298	-1.217	0.224	0.696	0.207	-1.467	0.142	0.388	1.248
GYPB	0.382	0.3	1.273	0.203	1.465	0.439	1.058	0.29	0.814	2.636
GYPA	-0.033	0.297	-0.111	0.911	0.967	0.287	-0.113	0.91	0.541	1.732
GYG2	-0.179	0.295	-0.607	0.544	0.836	0.247	-0.665	0.506	0.468	1.492
GYG1	0.913	0.315	2.897	0.004	2.493	0.786	1.9	0.057	1.344	4.625
GVINP1	-0.683	0.306	-2.23	0.026	0.505	0.155	-3.199	0.001	0.277	0.921
GUSBP14	-0.563	0.306	-1.844	0.065	0.569	0.174	-2.476	0.013	0.313	1.036
GUSBP11	-0.521	0.303	-1.724	0.085	0.594	0.18	-2.262	0.024	0.328	1.074
GUSB	0.495	0.3	1.648	0.099	1.64	0.493	1.3	0.194	0.91	2.955
GULP1	-0.737	0.307	-2.402	0.016	0.478	0.147	-3.551	0	0.262	0.873
GUK1	0.06	0.296	0.202	0.84	1.062	0.314	0.196	0.844	0.595	1.895
GUF1	-0.502	0.306	-1.64	0.101	0.605	0.185	-2.13	0.033	0.332	1.103
GUCY2F	0.028	0.295	0.096	0.924	1.029	0.304	0.094	0.925	0.577	1.835
GUCY2D	0.398	0.304	1.312	0.19	1.489	0.452	1.082	0.279	0.821	2.7
GUCY2C	0.173	0.296	0.585	0.559	1.189	0.352	0.537	0.591	0.666	2.125
GUCY1B2	0.19	0.296	0.642	0.521	1.21	0.358	0.585	0.558	0.677	2.161
GUCY1B1	-0.582	0.303	-1.924	0.054	0.559	0.169	-2.611	0.009	0.309	1.011
GUCY1A2	0.204	0.296	0.69	0.49	1.227	0.363	0.624	0.533	0.687	2.191
GUCY1A1	-0.27	0.296	-0.912	0.362	0.763	0.226	-1.048	0.295	0.427	1.364
GUCA2B	0.55	0.303	1.814	0.07	1.733	0.525	1.395	0.163	0.957	3.139
GUCA1B	-0.222	0.297	-0.745	0.456	0.801	0.238	-0.835	0.404	0.447	1.435
GUCA1A	-0.027	0.295	-0.091	0.928	0.974	0.288	-0.092	0.927	0.546	1.737
GTSE1	0.674	0.31	2.177	0.029	1.962	0.608	1.583	0.113	1.069	3.601
GTPBP8	0.583	0.302	1.926	0.054	1.791	0.542	1.46	0.144	0.99	3.24
GTPBP6	0.351	0.298	1.178	0.239	1.421	0.424	0.993	0.321	0.792	2.548
GTPBP4	1.118	0.336	3.326	0.001	3.06	1.029	2.002	0.045	1.583	5.914
GTPBP3	0.896	0.321	2.794	0.005	2.45	0.786	1.845	0.065	1.307	4.593
GTPBP2	0.39	0.303	1.285	0.199	1.477	0.448	1.064	0.287	0.815	2.676
GTPBP1	0.105	0.295	0.355	0.722	1.111	0.328	0.337	0.736	0.622	1.982
GTF3C5	0.086	0.296	0.29	0.772	1.09	0.323	0.278	0.781	0.61	1.948
GTF3C4	0.356	0.3	1.187	0.235	1.427	0.428	0.998	0.318	0.793	2.569
GTF3C3	0.309	0.298	1.039	0.299	1.362	0.405	0.894	0.371	0.76	2.441

GTF3C2	0.03	0.296	0.103	0.918	1.031	0.305	0.101	0.919	0.578	1.84
GTF3C1	-0.198	0.296	-0.668	0.504	0.82	0.243	-0.739	0.46	0.459	1.466
GTF3A	0.347	0.3	1.157	0.247	1.414	0.424	0.978	0.328	0.786	2.545
GTF2IRD2B	-0.006	0.296	-0.02	0.984	0.994	0.294	-0.02	0.984	0.557	1.775
GTF2IRD1	0.261	0.297	0.88	0.379	1.298	0.385	0.774	0.439	0.726	2.322
GTF2I	-0.09	0.297	-0.302	0.763	0.914	0.271	-0.316	0.752	0.511	1.636
GTF2H5	-0.213	0.298	-0.715	0.474	0.808	0.241	-0.797	0.425	0.45	1.449
GTF2H4	0.98	0.321	3.052	0.002	2.665	0.856	1.945	0.052	1.42	5.002
GTF2H3	0.29	0.298	0.976	0.329	1.337	0.398	0.847	0.397	0.746	2.395
GTF2H1	0.479	0.3	1.596	0.11	1.614	0.484	1.268	0.205	0.897	2.905
GTF2F2	0.161	0.298	0.54	0.589	1.174	0.35	0.499	0.618	0.655	2.105
GTF2F1	-0.644	0.306	-2.105	0.035	0.525	0.161	-2.956	0.003	0.288	0.957
GTF2E2	-0.304	0.3	-1.011	0.312	0.738	0.222	-1.182	0.237	0.41	1.329
GTF2E1	0.541	0.302	1.789	0.074	1.717	0.519	1.382	0.167	0.95	3.106
GTF2B	-0.209	0.301	-0.694	0.488	0.811	0.244	-0.772	0.44	0.45	1.464
GTF2A2	0.363	0.298	1.216	0.224	1.437	0.429	1.02	0.308	0.801	2.579
GTF2A1	-0.258	0.298	-0.866	0.387	0.773	0.23	-0.988	0.323	0.431	1.385
GTDC1	-0.14	0.296	-0.473	0.636	0.869	0.257	-0.508	0.612	0.487	1.552
GSTZ1	0.019	0.297	0.063	0.95	1.019	0.303	0.062	0.95	0.569	1.825
GSTT4	0.172	0.296	0.583	0.56	1.188	0.352	0.535	0.593	0.665	2.123
GSTT2	0.191	0.296	0.646	0.518	1.211	0.359	0.588	0.557	0.678	2.163
GSTT1	0.172	0.296	0.583	0.56	1.188	0.352	0.536	0.592	0.665	2.122
GSTP1	0.24	0.3	0.8	0.424	1.272	0.382	0.711	0.477	0.706	2.291
GSTO1	0.502	0.3	1.675	0.094	1.652	0.495	1.317	0.188	0.918	2.972
GSTM5	-0.165	0.296	-0.555	0.579	0.848	0.251	-0.604	0.546	0.475	1.516
GSTM4	-0.087	0.295	-0.296	0.767	0.916	0.271	-0.309	0.757	0.514	1.635
GSTM3	0.179	0.296	0.603	0.547	1.196	0.354	0.552	0.581	0.669	2.137
GSTM2	-0.045	0.295	-0.151	0.88	0.956	0.282	-0.154	0.877	0.536	1.706
GSTM1	0.016	0.295	0.053	0.958	1.016	0.3	0.052	0.958	0.569	1.812
GSTK1	-0.758	0.315	-2.407	0.016	0.468	0.148	-3.602	0	0.253	0.869
GSTCD	0.474	0.303	1.564	0.118	1.606	0.487	1.246	0.213	0.887	2.908
GSTA4	0.458	0.303	1.512	0.13	1.58	0.478	1.214	0.225	0.873	2.859
GSTA3	0.467	0.3	1.555	0.12	1.594	0.478	1.243	0.214	0.886	2.87
GSTA1	0.344	0.298	1.157	0.247	1.411	0.42	0.979	0.328	0.787	2.528
GSS	-0.725	0.31	-2.339	0.019	0.484	0.15	-3.435	0.001	0.264	0.889
GSR	-0.186	0.296	-0.628	0.53	0.83	0.246	-0.69	0.49	0.465	1.484
GSPT2	-0.136	0.297	-0.46	0.646	0.872	0.259	-0.492	0.622	0.488	1.561
GSPT1	0.395	0.3	1.317	0.188	1.484	0.445	1.088	0.277	0.825	2.67

GSN-AS1	0.075	0.296	0.254	0.8	1.078	0.319	0.244	0.807	0.604	1.924
GSN	-0.39	0.298	-1.309	0.191	0.677	0.202	-1.601	0.109	0.377	1.214
GSK3B	1.11	0.328	3.384	0.001	3.034	0.995	2.044	0.041	1.595	5.771
GSK3A	0.051	0.299	0.172	0.863	1.053	0.315	0.168	0.867	0.586	1.891
GSG1	0.003	0.295	0.01	0.992	1.003	0.296	0.01	0.992	0.562	1.789
GSE1	-0.186	0.298	-0.622	0.534	0.831	0.248	-0.683	0.494	0.463	1.49
GSDME	0.158	0.296	0.533	0.594	1.171	0.347	0.493	0.622	0.655	2.092
GSDMD	0.34	0.299	1.139	0.255	1.406	0.42	0.966	0.334	0.783	2.525
GSDMB	0.445	0.303	1.47	0.142	1.56	0.472	1.187	0.235	0.862	2.824
GSC2	-0.049	0.296	-0.165	0.869	0.952	0.282	-0.169	0.866	0.534	1.7
GSAP	-0.468	0.306	-1.525	0.127	0.627	0.192	-1.945	0.052	0.344	1.142
GRWD1	-0.354	0.3	-1.183	0.237	0.702	0.21	-1.42	0.156	0.39	1.262
GRTP1	-0.084	0.296	-0.283	0.777	0.92	0.272	-0.296	0.768	0.515	1.641
GRSF1	0.319	0.297	1.076	0.282	1.376	0.408	0.921	0.357	0.769	2.46
GRPR	-0.378	0.3	-1.262	0.207	0.685	0.205	-1.534	0.125	0.381	1.233
GRPEL1	-0.249	0.297	-0.838	0.402	0.78	0.231	-0.952	0.341	0.436	1.395
GRP	-0.577	0.303	-1.904	0.057	0.562	0.17	-2.576	0.01	0.31	1.017
GRN	-0.15	0.295	-0.508	0.611	0.861	0.254	-0.548	0.583	0.482	1.535
GRM8	-0.077	0.296	-0.259	0.796	0.926	0.274	-0.269	0.788	0.519	1.654
GRM7	0.153	0.296	0.518	0.604	1.166	0.345	0.48	0.631	0.652	2.083
GRM6	0.328	0.301	1.092	0.275	1.389	0.418	0.931	0.352	0.77	2.504
GRM5	-0.016	0.295	-0.054	0.957	0.984	0.29	-0.055	0.956	0.552	1.755
GRM4	0.458	0.302	1.515	0.13	1.581	0.478	1.216	0.224	0.874	2.858
GRM3	-0.391	0.299	-1.306	0.192	0.677	0.202	-1.598	0.11	0.376	1.216
GRM2	-0.118	0.295	-0.401	0.688	0.888	0.262	-0.426	0.67	0.498	1.585
GRM1	0.187	0.298	0.627	0.531	1.206	0.36	0.572	0.567	0.672	2.164
GRK6	0.54	0.301	1.795	0.073	1.716	0.516	1.387	0.165	0.952	3.093
GRK5	-0.2	0.298	-0.671	0.502	0.819	0.244	-0.743	0.457	0.457	1.468
GRK4	-0.3	0.3	-1.001	0.317	0.741	0.222	-1.168	0.243	0.412	1.333
GRK3	0.035	0.295	0.119	0.905	1.036	0.306	0.117	0.907	0.581	1.847
GRK2	0.027	0.297	0.093	0.926	1.028	0.305	0.091	0.927	0.575	1.838
GRK1	0.248	0.298	0.832	0.405	1.281	0.381	0.737	0.461	0.715	2.296
GRIP2	0.231	0.298	0.775	0.438	1.26	0.376	0.692	0.489	0.702	2.26
GRIP1	0.011	0.295	0.037	0.971	1.011	0.298	0.036	0.971	0.567	1.803
GRINA	0.219	0.298	0.735	0.462	1.245	0.371	0.66	0.509	0.694	2.232
GRIN2D	0.498	0.303	1.647	0.1	1.646	0.498	1.297	0.195	0.91	2.979
GRIN2C	-0.311	0.298	-1.046	0.295	0.733	0.218	-1.227	0.22	0.409	1.312
GRIN2B	0.323	0.298	1.086	0.277	1.382	0.411	0.928	0.353	0.771	2.476

GRIN2A	-0.082	0.295	-0.278	0.781	0.921	0.272	-0.29	0.772	0.516	1.643
GRIN1	-0.256	0.297	-0.861	0.389	0.774	0.23	-0.982	0.326	0.432	1.387
GRIK5	-0.189	0.296	-0.637	0.524	0.828	0.245	-0.701	0.483	0.463	1.48
GRIK4	0.072	0.296	0.243	0.808	1.075	0.318	0.235	0.815	0.602	1.919
GRIK3	-0.039	0.296	-0.131	0.896	0.962	0.284	-0.134	0.894	0.539	1.717
GRIK2	0.446	0.3	1.489	0.137	1.562	0.468	1.201	0.23	0.868	2.81
GRIK1	0.152	0.297	0.51	0.61	1.164	0.346	0.474	0.636	0.65	2.083
GRID2	0.202	0.296	0.681	0.496	1.223	0.362	0.617	0.537	0.685	2.185
GRIA4	-0.238	0.3	-0.794	0.427	0.788	0.236	-0.897	0.37	0.438	1.419
GRIA3	-0.076	0.295	-0.258	0.796	0.927	0.274	-0.268	0.789	0.519	1.653
GRIA2	-0.101	0.295	-0.34	0.734	0.904	0.267	-0.358	0.72	0.507	1.613
GRIA1	0.126	0.295	0.426	0.67	1.134	0.335	0.4	0.689	0.636	2.023
GRHPR	0.177	0.296	0.599	0.549	1.194	0.354	0.549	0.583	0.668	2.135
GRHL2	0.751	0.31	2.425	0.015	2.119	0.656	1.705	0.088	1.155	3.889
GREM2	-0.409	0.3	-1.366	0.172	0.664	0.199	-1.688	0.091	0.369	1.195
GREM1	-0.228	0.296	-0.769	0.442	0.796	0.236	-0.864	0.387	0.445	1.423
GREB1L	-0.392	0.3	-1.308	0.191	0.676	0.202	-1.601	0.109	0.376	1.216
GREB1	0.07	0.296	0.237	0.813	1.072	0.317	0.228	0.819	0.601	1.914
GRB7	0.097	0.296	0.328	0.743	1.102	0.326	0.313	0.754	0.617	1.967
GRB2	0.545	0.3	1.813	0.07	1.724	0.518	1.398	0.162	0.957	3.106
GRB14	0.439	0.3	1.462	0.144	1.551	0.465	1.184	0.236	0.861	2.791
GRB10	0.615	0.306	2.012	0.044	1.849	0.565	1.503	0.133	1.016	3.366
GRAP2	0.789	0.311	2.541	0.011	2.201	0.684	1.757	0.079	1.198	4.046
GRAP	0.572	0.303	1.888	0.059	1.771	0.536	1.438	0.15	0.979	3.206
GRAMD4	-0.32	0.298	-1.073	0.283	0.726	0.217	-1.265	0.206	0.404	1.303
GRAMD2B	-0.075	0.296	-0.254	0.799	0.928	0.274	-0.264	0.792	0.52	1.656
GRAMD1C	-0.356	0.3	-1.186	0.236	0.701	0.21	-1.424	0.154	0.389	1.261
GRAMD1B	0.151	0.3	0.504	0.614	1.163	0.349	0.468	0.64	0.646	2.094
GPX7	-0.23	0.296	-0.776	0.438	0.795	0.235	-0.872	0.383	0.445	1.42
GPX5	-0.023	0.295	-0.078	0.938	0.977	0.289	-0.078	0.937	0.548	1.744
GPX4	-0.071	0.296	-0.239	0.811	0.932	0.275	-0.248	0.805	0.522	1.663
GPX3	-0.142	0.296	-0.479	0.632	0.868	0.257	-0.515	0.607	0.486	1.55
GPX2	0.132	0.297	0.445	0.656	1.141	0.339	0.417	0.677	0.638	2.042
GPX1	0.024	0.298	0.08	0.937	1.024	0.305	0.079	0.937	0.571	1.836
GPT	0.217	0.296	0.735	0.462	1.243	0.368	0.661	0.509	0.696	2.219
GPSM3	-0.29	0.298	-0.974	0.33	0.748	0.223	-1.13	0.258	0.418	1.341
GPSM2	0.78	0.307	2.544	0.011	2.182	0.669	1.766	0.077	1.196	3.98
GPS2	0.057	0.296	0.191	0.848	1.058	0.313	0.186	0.852	0.593	1.889

GPS1	0.205	0.296	0.693	0.488	1.228	0.364	0.627	0.531	0.687	2.194
GPRIN2	0.728	0.306	2.376	0.017	2.071	0.635	1.688	0.091	1.136	3.775
GPRC5D	-0.022	0.295	-0.075	0.94	0.978	0.289	-0.076	0.94	0.548	1.745
GPRC5C	0.25	0.297	0.841	0.4	1.283	0.381	0.745	0.457	0.718	2.296
GPRC5B	0.139	0.299	0.465	0.642	1.149	0.343	0.434	0.664	0.64	2.064
GPRC5A	0.562	0.303	1.853	0.064	1.754	0.532	1.418	0.156	0.968	3.178
GPRASP1	-0.218	0.296	-0.737	0.461	0.804	0.238	-0.823	0.411	0.451	1.436
GPR88	0.285	0.298	0.956	0.339	1.329	0.396	0.832	0.405	0.742	2.382
GPR87	0.227	0.296	0.767	0.443	1.255	0.372	0.686	0.493	0.702	2.242
GPR85	0.184	0.296	0.622	0.534	1.202	0.355	0.568	0.57	0.673	2.145
GPR75	-0.209	0.295	-0.707	0.479	0.811	0.24	-0.787	0.432	0.455	1.448
GPR68	0.21	0.296	0.709	0.478	1.234	0.365	0.64	0.522	0.69	2.205
GPR65	-0.118	0.295	-0.4	0.689	0.889	0.262	-0.424	0.671	0.498	1.585
GPR63	0.754	0.306	2.461	0.014	2.125	0.651	1.728	0.084	1.166	3.874
GPR6	0.317	0.298	1.063	0.288	1.373	0.409	0.911	0.362	0.766	2.461
GPR52	-0.306	0.298	-1.028	0.304	0.736	0.219	-1.203	0.229	0.411	1.32
GPR50	-0.274	0.298	-0.92	0.357	0.76	0.226	-1.059	0.29	0.424	1.362
GPR45	0.088	0.296	0.296	0.767	1.092	0.323	0.283	0.777	0.611	1.95
GPR4	-0.65	0.31	-2.1	0.036	0.522	0.162	-2.958	0.003	0.284	0.958
GPR39	-0.534	0.304	-1.76	0.078	0.586	0.178	-2.326	0.02	0.323	1.063
GPR37L1	-0.046	0.296	-0.156	0.876	0.955	0.282	-0.159	0.874	0.535	1.704
GPR37	-0.117	0.295	-0.395	0.693	0.89	0.263	-0.418	0.676	0.499	1.588
GPR35	-0.138	0.296	-0.467	0.64	0.871	0.258	-0.501	0.616	0.488	1.555
GPR32	0.113	0.295	0.383	0.701	1.12	0.331	0.362	0.717	0.628	1.998
GPR31	0.139	0.296	0.468	0.639	1.149	0.341	0.437	0.662	0.643	2.054
GPR3	0.145	0.296	0.489	0.625	1.156	0.342	0.455	0.649	0.647	2.065
GPR27	-0.143	0.296	-0.484	0.628	0.867	0.256	-0.52	0.603	0.485	1.548
GPR25	-0.036	0.298	-0.12	0.904	0.965	0.287	-0.122	0.903	0.538	1.73
GPR22	-0.209	0.298	-0.702	0.483	0.811	0.242	-0.781	0.435	0.453	1.454
GPR21	0.031	0.295	0.105	0.917	1.031	0.305	0.103	0.918	0.578	1.84
GPR20	-0.53	0.302	-1.752	0.08	0.589	0.178	-2.309	0.021	0.326	1.065
GPR19	0.413	0.3	1.38	0.168	1.512	0.453	1.13	0.258	0.841	2.72
GPR183	-0.226	0.296	-0.762	0.446	0.798	0.236	-0.855	0.392	0.446	1.426
GPR182	0.148	0.299	0.495	0.621	1.16	0.347	0.46	0.645	0.645	2.084
GPR18	-0.547	0.303	-1.805	0.071	0.579	0.175	-2.403	0.016	0.32	1.048
GPR176-DT	-0.282	0.297	-0.95	0.342	0.754	0.224	-1.098	0.272	0.421	1.35
GPR176	-0.38	0.3	-1.268	0.205	0.684	0.205	-1.542	0.123	0.38	1.23
GPR173	-0.489	0.3	-1.628	0.103	0.613	0.184	-2.1	0.036	0.34	1.105

GPR171	-0.105	0.296	-0.355	0.722	0.9	0.266	-0.375	0.708	0.504	1.608
GPR17	0.243	0.296	0.821	0.412	1.275	0.378	0.729	0.466	0.714	2.278
GPR162	-0.098	0.295	-0.332	0.74	0.907	0.268	-0.348	0.728	0.508	1.617
GPR161	0.406	0.3	1.355	0.175	1.501	0.45	1.114	0.265	0.834	2.7
GPR157	0.246	0.298	0.823	0.41	1.278	0.381	0.73	0.465	0.713	2.294
GPR153	-0.499	0.3	-1.664	0.096	0.607	0.182	-2.157	0.031	0.337	1.093
GPR15	0.286	0.3	0.952	0.341	1.331	0.399	0.828	0.408	0.739	2.396
GPR143	0.293	0.298	0.982	0.326	1.34	0.4	0.851	0.395	0.747	2.404
GPR137B	-0.11	0.296	-0.372	0.71	0.896	0.265	-0.394	0.694	0.502	1.599
GPR137	-0.766	0.315	-2.435	0.015	0.465	0.146	-3.661	0	0.251	0.861
GPR135	-0.244	0.298	-0.819	0.413	0.784	0.233	-0.927	0.354	0.437	1.404
GPR132	0.445	0.3	1.482	0.138	1.56	0.468	1.196	0.232	0.866	2.809
GPR12	0.706	0.31	2.279	0.023	2.026	0.627	1.635	0.102	1.104	3.717
GPR107	0.245	0.297	0.826	0.409	1.278	0.379	0.733	0.464	0.714	2.287
GPR1	-0.151	0.296	-0.51	0.61	0.86	0.255	-0.551	0.582	0.481	1.537
GPNMB	-0.598	0.306	-1.956	0.05	0.55	0.168	-2.677	0.007	0.302	1.001
GPN3	-0.072	0.296	-0.242	0.809	0.931	0.276	-0.25	0.802	0.521	1.664
GPN2	0.253	0.296	0.853	0.394	1.287	0.381	0.753	0.451	0.72	2.3
GPN1	0.252	0.296	0.851	0.395	1.287	0.382	0.752	0.452	0.72	2.301
GPM6B	-0.349	0.3	-1.167	0.243	0.705	0.211	-1.397	0.163	0.392	1.268
GPM6A	-0.262	0.298	-0.88	0.379	0.77	0.229	-1.006	0.314	0.429	1.379
GPLD1	-0.497	0.303	-1.643	0.1	0.608	0.184	-2.128	0.033	0.336	1.101
GPKOW	-0.092	0.3	-0.306	0.76	0.912	0.274	-0.32	0.749	0.506	1.644
GPI	0.28	0.296	0.947	0.344	1.324	0.392	0.826	0.409	0.741	2.365
GPHN	-0.652	0.31	-2.105	0.035	0.521	0.161	-2.969	0.003	0.284	0.956
GPER1	-0.514	0.306	-1.681	0.093	0.598	0.183	-2.197	0.028	0.329	1.089
GPD2	0.001	0.295	0.003	0.997	1.001	0.296	0.003	0.997	0.561	1.786
GPD1L	-0.867	0.321	-2.704	0.007	0.42	0.135	-4.304	0	0.224	0.788
GPD1	0.062	0.295	0.211	0.833	1.064	0.314	0.205	0.838	0.597	1.899
GPC5	0.158	0.295	0.535	0.593	1.171	0.346	0.495	0.621	0.656	2.089
GPC4	-0.304	0.298	-1.021	0.307	0.738	0.22	-1.193	0.233	0.412	1.323
GPC3	0.152	0.296	0.513	0.608	1.164	0.345	0.476	0.634	0.651	2.08
GPC1	0.237	0.298	0.796	0.426	1.267	0.377	0.709	0.478	0.707	2.271
GPBP1L1	-0.623	0.305	-2.045	0.041	0.536	0.163	-2.838	0.005	0.295	0.974
GPATCH8	-0.053	0.298	-0.179	0.858	0.948	0.282	-0.184	0.854	0.529	1.7
GPATCH4	0.11	0.3	0.367	0.714	1.116	0.335	0.348	0.728	0.62	2.01
GPATCH3	0.393	0.297	1.323	0.186	1.481	0.439	1.095	0.274	0.828	2.648
GPATCH2L	0.214	0.296	0.723	0.47	1.239	0.367	0.651	0.515	0.693	2.213

GPATCH2	0.091	0.295	0.309	0.758	1.095	0.324	0.295	0.768	0.614	1.955
GPATCH1	0.405	0.298	1.359	0.174	1.499	0.446	1.117	0.264	0.836	2.687
GPALPP1	0.67	0.307	2.184	0.029	1.954	0.599	1.592	0.111	1.071	3.564
GPAA1	0.504	0.303	1.664	0.096	1.655	0.501	1.307	0.191	0.914	2.997
GPA33	0.33	0.304	1.087	0.277	1.391	0.423	0.925	0.355	0.767	2.525
GP9	-0.272	0.297	-0.918	0.359	0.762	0.226	-1.055	0.292	0.426	1.362
GP6	0.276	0.298	0.927	0.354	1.318	0.393	0.81	0.418	0.735	2.363
GP5	0.232	0.296	0.784	0.433	1.261	0.374	0.699	0.484	0.706	2.255
GP2	-0.492	0.302	-1.629	0.103	0.611	0.185	-2.104	0.035	0.338	1.105
GP1BA	0.146	0.298	0.491	0.624	1.157	0.345	0.456	0.648	0.646	2.075
GOT2	-0.225	0.298	-0.754	0.451	0.799	0.238	-0.846	0.398	0.446	1.432
GOT1	0.309	0.297	1.041	0.298	1.362	0.404	0.896	0.37	0.762	2.435
GOSR2	-0.289	0.298	-0.972	0.331	0.749	0.223	-1.127	0.26	0.418	1.342
GOSR1	-0.41	0.302	-1.356	0.175	0.664	0.201	-1.677	0.094	0.367	1.2
GORASP2	-0.075	0.296	-0.254	0.8	0.928	0.274	-0.264	0.792	0.519	1.657
GORASP1	-0.509	0.303	-1.682	0.093	0.601	0.182	-2.192	0.028	0.332	1.088
GON4L	-0.549	0.303	-1.813	0.07	0.577	0.175	-2.416	0.016	0.319	1.045
GOLT1B	-0.017	0.295	-0.058	0.954	0.983	0.29	-0.059	0.953	0.551	1.754
GOLPH3L	-0.495	0.306	-1.616	0.106	0.61	0.187	-2.09	0.037	0.334	1.111
GOLPH3	0.036	0.295	0.123	0.902	1.037	0.306	0.121	0.904	0.582	1.849
GOLM1	-0.197	0.296	-0.668	0.504	0.821	0.243	-0.738	0.46	0.46	1.465
GOLIM4	0.312	0.298	1.049	0.294	1.366	0.407	0.901	0.368	0.762	2.449
GOLGB1	-0.582	0.303	-1.923	0.055	0.559	0.169	-2.609	0.009	0.308	1.011
GOLGA8N	-0.612	0.306	-2.003	0.045	0.542	0.166	-2.762	0.006	0.298	0.987
GOLGA8A	-0.33	0.298	-1.106	0.269	0.719	0.214	-1.31	0.19	0.401	1.29
GOLGA7	-0.251	0.298	-0.845	0.398	0.778	0.231	-0.961	0.337	0.434	1.393
GOLGA5	-0.205	0.296	-0.692	0.489	0.815	0.241	-0.768	0.442	0.456	1.456
GOLGA4	0.151	0.296	0.509	0.611	1.163	0.345	0.472	0.637	0.65	2.079
GOLGA3	-0.264	0.296	-0.89	0.374	0.768	0.228	-1.018	0.309	0.43	1.373
GOLGA2P5	-0.033	0.295	-0.111	0.911	0.968	0.286	-0.113	0.91	0.542	1.726
GOLGA2	0.378	0.299	1.264	0.206	1.46	0.437	1.053	0.292	0.812	2.623
GOLGA1	-0.154	0.297	-0.518	0.604	0.858	0.254	-0.56	0.575	0.48	1.534
GNS	-0.156	0.296	-0.527	0.598	0.856	0.253	-0.57	0.569	0.479	1.529
GNRHR	-0.071	0.295	-0.239	0.811	0.932	0.275	-0.248	0.804	0.522	1.662
GNRH2	0.279	0.298	0.935	0.35	1.322	0.394	0.816	0.414	0.737	2.37
GNRH1	-0.085	0.295	-0.288	0.774	0.919	0.271	-0.3	0.764	0.515	1.639
GNPTAB	-0.5	0.303	-1.653	0.098	0.607	0.183	-2.144	0.032	0.335	1.097
GNPDA1	-0.461	0.306	-1.507	0.132	0.631	0.193	-1.914	0.056	0.346	1.149

GNPAT	-0.566	0.306	-1.852	0.064	0.568	0.174	-2.49	0.013	0.312	1.034
GNMT	-0.483	0.3	-1.611	0.107	0.617	0.185	-2.071	0.038	0.343	1.11
GNLY	-0.028	0.296	-0.093	0.926	0.973	0.288	-0.095	0.925	0.545	1.737
GNL3L	0.111	0.296	0.374	0.708	1.117	0.331	0.354	0.723	0.625	1.997
GNL2	0.315	0.297	1.06	0.289	1.37	0.407	0.909	0.363	0.766	2.451
GNL1	0.428	0.3	1.43	0.153	1.535	0.46	1.163	0.245	0.853	2.762
GNGT1	0.42	0.3	1.399	0.162	1.521	0.456	1.143	0.253	0.845	2.738
GNG7	-0.309	0.3	-1.03	0.303	0.734	0.22	-1.206	0.228	0.408	1.322
GNG5	0.512	0.304	1.684	0.092	1.669	0.508	1.317	0.188	0.919	3.031
GNG4	0.226	0.296	0.763	0.445	1.254	0.371	0.683	0.494	0.702	2.241
GNG3	0.085	0.298	0.286	0.775	1.089	0.324	0.274	0.784	0.607	1.952
GNG13	0.625	0.306	2.04	0.041	1.868	0.572	1.517	0.129	1.025	3.404
GNG12	-1.207	0.336	-3.594	0	0.299	0.1	-6.98	0	0.155	0.578
GNG11	-0.696	0.306	-2.275	0.023	0.498	0.153	-3.288	0.001	0.274	0.908
GNE	-0.187	0.296	-0.63	0.529	0.83	0.246	-0.692	0.489	0.464	1.483
GNB5	-0.054	0.299	-0.181	0.856	0.947	0.283	-0.186	0.853	0.527	1.702
GNB3	0.542	0.3	1.808	0.071	1.72	0.516	1.396	0.163	0.955	3.095
GNB2	-0.228	0.298	-0.765	0.444	0.796	0.238	-0.859	0.39	0.444	1.429
GNB1L	0.014	0.295	0.047	0.963	1.014	0.3	0.047	0.963	0.568	1.809
GNB1	-0.555	0.306	-1.815	0.069	0.574	0.176	-2.427	0.015	0.315	1.045
GNAZ	0.491	0.302	1.623	0.105	1.634	0.494	1.283	0.2	0.903	2.956
GNAT3	0.082	0.296	0.277	0.782	1.085	0.321	0.266	0.79	0.608	1.938
GNAT2	-0.21	0.297	-0.707	0.479	0.811	0.241	-0.787	0.431	0.453	1.45
GNAT1	-0.102	0.296	-0.345	0.73	0.903	0.267	-0.363	0.716	0.506	1.612
GNAS	0.175	0.296	0.591	0.555	1.191	0.352	0.542	0.588	0.667	2.125
GNAQ	-0.632	0.306	-2.069	0.039	0.531	0.162	-2.886	0.004	0.292	0.967
GNAO1	-0.072	0.296	-0.242	0.809	0.931	0.276	-0.25	0.802	0.521	1.663
GNAL	-0.635	0.302	-2.099	0.036	0.53	0.16	-2.931	0.003	0.293	0.959
GNAI3	0.534	0.302	1.767	0.077	1.706	0.516	1.369	0.171	0.943	3.085
GNAI2	-1.043	0.328	-3.183	0.001	0.352	0.115	-5.609	0	0.185	0.67
GNAI1	-0.099	0.296	-0.336	0.737	0.905	0.268	-0.353	0.724	0.507	1.618
GNA15	0.081	0.295	0.276	0.783	1.085	0.32	0.265	0.791	0.608	1.935
GNA14	-0.237	0.298	-0.797	0.426	0.789	0.235	-0.899	0.368	0.44	1.414
GNA13	-0.507	0.306	-1.658	0.097	0.602	0.184	-2.16	0.031	0.331	1.097
GNA12	0.184	0.296	0.62	0.535	1.202	0.356	0.566	0.571	0.672	2.147
GNA11	0.005	0.295	0.016	0.987	1.005	0.297	0.016	0.987	0.563	1.792
GMPS	1.207	0.336	3.591	0	3.342	1.123	2.085	0.037	1.73	6.457
GMPR2	0.13	0.296	0.439	0.661	1.139	0.337	0.412	0.68	0.637	2.036

GMPR	0.465	0.302	1.538	0.124	1.592	0.481	1.23	0.219	0.88	2.878
GMPPA	-0.336	0.298	-1.128	0.259	0.715	0.213	-1.34	0.18	0.399	1.281
GMNN	0.24	0.298	0.806	0.421	1.271	0.379	0.716	0.474	0.709	2.28
GML	0.123	0.297	0.414	0.679	1.131	0.336	0.39	0.697	0.632	2.024
GMIP	0.002	0.296	0.008	0.994	1.002	0.296	0.008	0.994	0.561	1.789
GMFG	-0.169	0.296	-0.571	0.568	0.844	0.25	-0.622	0.534	0.472	1.509
GMFB	0.061	0.295	0.208	0.835	1.063	0.314	0.201	0.84	0.596	1.897
GMEB2	0.945	0.315	2.999	0.003	2.573	0.811	1.94	0.052	1.387	4.773
GMEB1	0.357	0.298	1.198	0.231	1.429	0.426	1.008	0.314	0.797	2.564
GMDS	-0.183	0.296	-0.619	0.536	0.833	0.247	-0.679	0.497	0.466	1.488
GM2A	-0.164	0.296	-0.556	0.578	0.848	0.251	-0.605	0.545	0.475	1.514
GLYR1	-0.1	0.295	-0.339	0.735	0.905	0.267	-0.356	0.722	0.507	1.614
GLYAT	-0.305	0.297	-1.024	0.306	0.737	0.219	-1.197	0.231	0.412	1.321
GLUL	-0.133	0.296	-0.45	0.653	0.875	0.259	-0.481	0.63	0.49	1.564
GLUD2	-0.483	0.306	-1.579	0.114	0.617	0.189	-2.029	0.042	0.339	1.124
GLUD1	-0.053	0.295	-0.18	0.857	0.948	0.28	-0.185	0.853	0.531	1.692
GLTP	0.216	0.296	0.731	0.465	1.242	0.368	0.657	0.511	0.695	2.219
GLT8D2	-0.704	0.306	-2.302	0.021	0.494	0.151	-3.342	0.001	0.272	0.901
GLT8D1	-0.477	0.306	-1.556	0.12	0.621	0.19	-1.993	0.046	0.341	1.132
GLS2	-0.261	0.3	-0.872	0.383	0.77	0.231	-0.997	0.319	0.428	1.386
GLS	-0.192	0.297	-0.646	0.518	0.825	0.245	-0.713	0.476	0.461	1.477
GLRX5	0.303	0.3	1.009	0.313	1.354	0.407	0.871	0.384	0.752	2.44
GLRX3	-0.011	0.296	-0.036	0.971	0.989	0.293	-0.037	0.971	0.553	1.769
GLRX2	0.594	0.303	1.959	0.05	1.811	0.549	1.477	0.14	1	3.282
GLRX	-0.105	0.295	-0.354	0.723	0.901	0.266	-0.374	0.709	0.505	1.607
GLRB	-0.859	0.321	-2.68	0.007	0.424	0.136	-4.246	0	0.226	0.794
GLRA3	0.335	0.298	1.126	0.26	1.398	0.416	0.957	0.339	0.78	2.506
GLRA2	0.197	0.296	0.665	0.506	1.218	0.361	0.603	0.546	0.681	2.176
GLRA1	0.2	0.296	0.677	0.499	1.222	0.362	0.613	0.54	0.684	2.183
GLP2R	-0.092	0.295	-0.31	0.756	0.913	0.269	-0.325	0.745	0.512	1.628
GLP1R	0.147	0.298	0.491	0.623	1.158	0.346	0.457	0.648	0.645	2.078
GLOD4	-0.031	0.295	-0.104	0.917	0.97	0.286	-0.105	0.916	0.544	1.73
GLO1	0.47	0.3	1.566	0.117	1.6	0.48	1.249	0.212	0.888	2.881
GLMN	0.127	0.295	0.43	0.667	1.135	0.335	0.404	0.687	0.636	2.026
GLIPR1	-0.758	0.31	-2.446	0.014	0.468	0.145	-3.66	0	0.255	0.86
GLI3	-0.431	0.3	-1.437	0.151	0.65	0.195	-1.796	0.073	0.361	1.17
GLI2	-0.217	0.296	-0.735	0.462	0.805	0.238	-0.821	0.411	0.451	1.436
GLI1	-0.118	0.295	-0.399	0.69	0.889	0.262	-0.424	0.672	0.498	1.585

GLG1	0.096	0.295	0.325	0.745	1.101	0.325	0.31	0.757	0.617	1.963
GLE1	-0.292	0.298	-0.98	0.327	0.746	0.223	-1.138	0.255	0.416	1.34
GLDC	-0.04	0.295	-0.134	0.893	0.961	0.284	-0.137	0.891	0.539	1.714
GLCE	0.137	0.297	0.46	0.646	1.146	0.34	0.43	0.667	0.641	2.051
GLB1L2	-0.004	0.295	-0.014	0.989	0.996	0.294	-0.014	0.989	0.558	1.776
GLB1L	-0.383	0.303	-1.262	0.207	0.682	0.207	-1.538	0.124	0.376	1.236
GLA	-0.349	0.3	-1.166	0.244	0.705	0.211	-1.395	0.163	0.392	1.269
GKN1	-0.145	0.297	-0.488	0.626	0.865	0.257	-0.525	0.6	0.483	1.549
GK3P	-0.565	0.306	-1.849	0.064	0.568	0.174	-2.486	0.013	0.312	1.034
GK2	0.262	0.298	0.88	0.379	1.3	0.388	0.774	0.439	0.724	2.333
GK	-0.175	0.297	-0.591	0.554	0.839	0.249	-0.646	0.518	0.469	1.501
GJD2	0.22	0.296	0.741	0.459	1.246	0.369	0.666	0.506	0.697	2.226
GJC2	0.525	0.303	1.734	0.083	1.691	0.512	1.349	0.177	0.934	3.062
GJC1	-0.212	0.298	-0.711	0.477	0.809	0.241	-0.792	0.428	0.452	1.45
GJB5	-0.109	0.296	-0.367	0.714	0.897	0.266	-0.388	0.698	0.502	1.603
GJB4	0.334	0.303	1.101	0.271	1.396	0.423	0.936	0.349	0.771	2.529
GJB3	-0.012	0.295	-0.04	0.968	0.988	0.292	-0.041	0.968	0.554	1.762
GJB1	-0.167	0.296	-0.562	0.574	0.847	0.251	-0.612	0.541	0.474	1.513
GJA8	-0.253	0.298	-0.85	0.395	0.777	0.231	-0.967	0.333	0.433	1.391
GJA5	-1.15	0.337	-3.415	0.001	0.317	0.107	-6.41	0	0.164	0.613
GJA4	-0.168	0.297	-0.564	0.573	0.846	0.251	-0.614	0.539	0.473	1.514
GJA3	0.424	0.3	1.416	0.157	1.529	0.458	1.154	0.248	0.85	2.75
GJA1	-1.469	0.359	-4.095	0	0.23	0.083	-9.327	0	0.114	0.465
GIT2	-0.108	0.295	-0.366	0.714	0.897	0.265	-0.387	0.699	0.503	1.601
GIT1	0.792	0.311	2.548	0.011	2.207	0.685	1.76	0.078	1.2	4.056
GIPR	0.177	0.296	0.599	0.549	1.194	0.354	0.549	0.583	0.668	2.133
GIPC2	-0.422	0.3	-1.409	0.159	0.656	0.197	-1.753	0.08	0.364	1.18
GIPC1	0.635	0.305	2.082	0.037	1.887	0.576	1.541	0.123	1.038	3.431
GIP	-0.139	0.295	-0.47	0.638	0.87	0.257	-0.504	0.614	0.488	1.553
GINS4	0.808	0.31	2.607	0.009	2.244	0.696	1.788	0.074	1.222	4.119
GINS3	0.489	0.302	1.617	0.106	1.631	0.493	1.279	0.201	0.902	2.949
GINS2	0.754	0.306	2.464	0.014	2.126	0.651	1.73	0.084	1.167	3.874
GINS1	0.574	0.302	1.9	0.057	1.776	0.537	1.446	0.148	0.982	3.212
GIN1	0.152	0.296	0.512	0.609	1.164	0.345	0.475	0.635	0.651	2.08
GIMAP6	-1.028	0.321	-3.198	0.001	0.358	0.115	-5.585	0	0.191	0.672
GIMAP4	-0.511	0.302	-1.689	0.091	0.6	0.181	-2.205	0.027	0.332	1.085
GIGYF2	-0.559	0.303	-1.846	0.065	0.572	0.173	-2.474	0.013	0.316	1.035
GID8	0.264	0.302	0.874	0.382	1.303	0.394	0.768	0.442	0.72	2.356

GID4	0.183	0.296	0.616	0.538	1.2	0.355	0.563	0.573	0.672	2.145
GHSR	-0.736	0.31	-2.372	0.018	0.479	0.149	-3.506	0	0.261	0.88
GHRHR	0.255	0.298	0.858	0.391	1.291	0.384	0.757	0.449	0.72	2.313
GHRH	0.058	0.295	0.197	0.844	1.06	0.313	0.191	0.849	0.594	1.891
GHR	-0.136	0.295	-0.461	0.645	0.873	0.258	-0.494	0.621	0.489	1.557
GHITM	0.313	0.296	1.057	0.291	1.368	0.405	0.907	0.364	0.765	2.444
GH2	0.176	0.296	0.595	0.552	1.193	0.353	0.546	0.585	0.667	2.132
GH1	0.491	0.3	1.637	0.102	1.634	0.49	1.293	0.196	0.908	2.942
GGTLC1	0.077	0.296	0.26	0.795	1.08	0.319	0.25	0.803	0.605	1.927
GGT5	-0.248	0.298	-0.832	0.406	0.781	0.233	-0.944	0.345	0.435	1.399
GGPS1	-0.295	0.298	-0.992	0.321	0.744	0.221	-1.154	0.248	0.415	1.334
GGNBP2	0.091	0.296	0.307	0.759	1.095	0.324	0.294	0.769	0.614	1.954
GGH	0.491	0.302	1.623	0.105	1.633	0.494	1.282	0.2	0.903	2.954
GGCX	0.145	0.296	0.49	0.624	1.156	0.343	0.456	0.648	0.647	2.066
GGCT	0.338	0.301	1.122	0.262	1.402	0.422	0.952	0.341	0.777	2.529
GGA3	0.139	0.297	0.468	0.64	1.149	0.341	0.437	0.662	0.642	2.055
GGA2	-0.171	0.296	-0.578	0.563	0.843	0.249	-0.631	0.528	0.472	1.505
GGA1	0.081	0.295	0.274	0.784	1.084	0.32	0.263	0.793	0.608	1.935
GFUS	0.086	0.297	0.289	0.773	1.09	0.323	0.277	0.782	0.609	1.949
GFRA4	0.304	0.297	1.023	0.306	1.355	0.402	0.882	0.378	0.757	2.425
GFRA3	0.065	0.296	0.22	0.826	1.067	0.316	0.213	0.831	0.598	1.907
GFRA2	0.44	0.302	1.455	0.146	1.553	0.47	1.177	0.239	0.858	2.809
GFRA1	0.436	0.3	1.456	0.145	1.547	0.464	1.18	0.238	0.86	2.783
GFPT2	-1.05	0.321	-3.266	0.001	0.35	0.112	-5.78	0	0.186	0.657
GFPT1	-0.154	0.296	-0.521	0.602	0.857	0.254	-0.564	0.573	0.479	1.531
GFOD2	0.027	0.296	0.09	0.928	1.027	0.303	0.089	0.929	0.575	1.833
GFOD1	0.367	0.3	1.225	0.22	1.444	0.433	1.026	0.305	0.802	2.597
GFM1	0.564	0.302	1.865	0.062	1.757	0.531	1.425	0.154	0.972	3.179
GFI1B	-0.937	0.316	-2.969	0.003	0.392	0.124	-4.919	0	0.211	0.727
GFI1	-0.112	0.296	-0.377	0.706	0.894	0.264	-0.399	0.69	0.501	1.596
GFER	-0.158	0.296	-0.534	0.593	0.854	0.253	-0.579	0.563	0.478	1.525
GFAP	0.111	0.296	0.374	0.709	1.117	0.331	0.354	0.724	0.625	1.997
GET3	-0.194	0.302	-0.643	0.52	0.823	0.249	-0.71	0.478	0.455	1.489
GET1	-0.139	0.298	-0.467	0.641	0.87	0.26	-0.501	0.617	0.485	1.561
GEMIN8	-0.603	0.306	-1.971	0.049	0.547	0.167	-2.705	0.007	0.301	0.997
GEMIN7	0.102	0.295	0.347	0.729	1.108	0.327	0.33	0.742	0.621	1.977
GEMIN6	0.261	0.298	0.877	0.38	1.298	0.387	0.772	0.44	0.724	2.327
GEMIN4	0.425	0.3	1.419	0.156	1.529	0.458	1.156	0.248	0.85	2.751

GEMIN2	-0.357	0.299	-1.195	0.232	0.7	0.209	-1.437	0.151	0.39	1.257
GEM	-0.142	0.296	-0.48	0.631	0.868	0.257	-0.515	0.606	0.486	1.549
GDPD5	0.431	0.3	1.437	0.151	1.538	0.461	1.167	0.243	0.855	2.768
GDPD3	-0.337	0.297	-1.134	0.257	0.714	0.212	-1.349	0.177	0.398	1.278
GDPD2	-0.037	0.295	-0.125	0.901	0.964	0.284	-0.127	0.899	0.541	1.719
GDNF	1.038	0.328	3.167	0.002	2.822	0.925	1.971	0.049	1.485	5.364
GDI2	-0.097	0.297	-0.328	0.743	0.907	0.269	-0.345	0.73	0.507	1.623
GDI1	-0.03	0.295	-0.102	0.918	0.97	0.286	-0.104	0.917	0.544	1.731
GDF9	0.083	0.296	0.279	0.78	1.086	0.321	0.268	0.789	0.608	1.938
GDF5	-0.301	0.3	-1.006	0.314	0.74	0.222	-1.174	0.24	0.411	1.331
GDF3	-0.179	0.296	-0.605	0.545	0.836	0.248	-0.663	0.508	0.468	1.494
GDF2	-0.767	0.31	-2.475	0.013	0.464	0.144	-3.722	0	0.253	0.852
GDF15	0.084	0.295	0.284	0.777	1.087	0.321	0.272	0.785	0.609	1.941
GDF11	0.19	0.296	0.642	0.521	1.21	0.358	0.585	0.559	0.677	2.161
GDF10	-1.083	0.319	-3.401	0.001	0.338	0.108	-6.135	0	0.181	0.632
GDE1	-0.791	0.315	-2.514	0.012	0.453	0.143	-3.831	0	0.245	0.84
GDAP2	0.05	0.295	0.168	0.866	1.051	0.31	0.164	0.87	0.589	1.874
GDAP1L1	0.663	0.303	2.187	0.029	1.941	0.589	1.598	0.11	1.071	3.517
GDAP1	-0.566	0.306	-1.852	0.064	0.568	0.174	-2.49	0.013	0.312	1.034
GCSH	0.359	0.298	1.205	0.228	1.432	0.426	1.012	0.311	0.799	2.566
GCNT4	0.091	0.295	0.308	0.758	1.095	0.323	0.294	0.768	0.614	1.954
GCNT3	0.278	0.298	0.932	0.351	1.32	0.394	0.814	0.416	0.736	2.368
GCNT2	0.228	0.296	0.771	0.441	1.256	0.372	0.689	0.491	0.703	2.245
GCNT1	0.184	0.298	0.619	0.536	1.203	0.358	0.566	0.572	0.671	2.155
GCN1	0.115	0.298	0.385	0.7	1.122	0.335	0.364	0.716	0.625	2.014
GCM2	0.278	0.298	0.933	0.351	1.32	0.393	0.815	0.415	0.737	2.366
GCM1	-0.195	0.296	-0.658	0.51	0.823	0.244	-0.727	0.467	0.46	1.47
GCLM	0.065	0.296	0.218	0.827	1.067	0.316	0.211	0.833	0.597	1.907
GCLC	0.221	0.295	0.749	0.454	1.248	0.369	0.672	0.502	0.699	2.226
GCKR	0.01	0.295	0.033	0.974	1.01	0.298	0.032	0.974	0.566	1.8
GCK	0.009	0.296	0.032	0.975	1.009	0.299	0.031	0.975	0.565	1.804
GCHFR	0.44	0.299	1.474	0.141	1.553	0.464	1.192	0.233	0.865	2.789
GCH1	0.145	0.295	0.491	0.623	1.156	0.341	0.457	0.647	0.648	2.062
GCGR	0.379	0.3	1.263	0.207	1.46	0.438	1.051	0.293	0.811	2.628
GCG	-0.024	0.295	-0.083	0.934	0.976	0.288	-0.084	0.933	0.547	1.74
GCFC2	0.324	0.297	1.091	0.275	1.383	0.411	0.932	0.351	0.773	2.475
GCDH	0.486	0.299	1.625	0.104	1.626	0.486	1.287	0.198	0.905	2.923
GCC2	-0.456	0.3	-1.519	0.129	0.634	0.19	-1.925	0.054	0.352	1.142

GCC1	-0.424	0.298	-1.422	0.155	0.654	0.195	-1.772	0.076	0.365	1.174
GCAT	-0.065	0.296	-0.219	0.826	0.937	0.277	-0.227	0.821	0.525	1.674
GCA	0.576	0.303	1.905	0.057	1.78	0.539	1.447	0.148	0.983	3.221
GC	-0.587	0.305	-1.921	0.055	0.556	0.17	-2.613	0.009	0.306	1.012
GBX2	-0.007	0.296	-0.022	0.982	0.993	0.294	-0.022	0.982	0.557	1.773
GBX1	-0.327	0.298	-1.098	0.272	0.721	0.215	-1.299	0.194	0.402	1.293
GBP2	-0.474	0.3	-1.579	0.114	0.623	0.187	-2.02	0.043	0.346	1.121
GBP1	0.359	0.3	1.195	0.232	1.431	0.429	1.004	0.315	0.795	2.577
GBF1	-0.222	0.296	-0.751	0.453	0.801	0.237	-0.841	0.401	0.448	1.431
GBE1	0.119	0.296	0.401	0.688	1.126	0.333	0.379	0.705	0.631	2.009
GBAP1	0.23	0.296	0.776	0.438	1.258	0.373	0.693	0.488	0.704	2.248
GBA3	0.304	0.297	1.025	0.305	1.356	0.403	0.884	0.377	0.758	2.427
GATM	-0.491	0.302	-1.623	0.105	0.612	0.185	-2.095	0.036	0.338	1.107
GATD3A	0.365	0.298	1.224	0.221	1.44	0.429	1.025	0.305	0.803	2.584
GATC	-0.164	0.296	-0.555	0.579	0.848	0.251	-0.603	0.546	0.475	1.517
GATB	0.088	0.295	0.297	0.767	1.092	0.323	0.284	0.776	0.612	1.948
GATAD2A	0.659	0.31	2.124	0.034	1.932	0.599	1.556	0.12	1.052	3.549
GATAD1	0.081	0.296	0.275	0.784	1.085	0.321	0.264	0.792	0.607	1.937
GATA6	-0.908	0.317	-2.867	0.004	0.404	0.128	-4.67	0	0.217	0.75
GATA4	0.109	0.295	0.371	0.711	1.116	0.329	0.351	0.726	0.625	1.99
GATA3	-0.213	0.298	-0.714	0.475	0.809	0.241	-0.796	0.426	0.451	1.449
GATA2	-0.144	0.296	-0.487	0.627	0.866	0.256	-0.523	0.601	0.485	1.547
GATA1	-0.412	0.3	-1.376	0.169	0.662	0.198	-1.703	0.089	0.368	1.191
GAST	0.082	0.295	0.278	0.781	1.085	0.32	0.267	0.79	0.609	1.935
GASK1B	-1.534	0.372	-4.119	0	0.216	0.08	-9.765	0	0.104	0.447
GAS8-AS1	0.133	0.296	0.451	0.652	1.143	0.338	0.422	0.673	0.64	2.039
GAS8	-0.082	0.295	-0.277	0.782	0.921	0.272	-0.289	0.773	0.517	1.644
GAS7	-0.292	0.296	-0.985	0.325	0.747	0.221	-1.143	0.253	0.418	1.335
GAS6	-0.124	0.296	-0.42	0.674	0.883	0.261	-0.447	0.655	0.495	1.576
GAS2L1	0.531	0.302	1.758	0.079	1.701	0.514	1.364	0.173	0.941	3.077
GAS2	0.17	0.296	0.574	0.566	1.185	0.351	0.528	0.597	0.663	2.118
GAS1	-0.271	0.296	-0.914	0.361	0.763	0.226	-1.049	0.294	0.427	1.364
GART	0.1	0.295	0.34	0.734	1.106	0.326	0.323	0.746	0.62	1.972
GARS1	0.213	0.296	0.72	0.471	1.238	0.367	0.649	0.517	0.693	2.212
GARRE1	-0.808	0.311	-2.601	0.009	0.446	0.138	-4.003	0	0.242	0.819
GARNL3	0.064	0.296	0.216	0.829	1.066	0.315	0.209	0.834	0.597	1.903
GAREM1	0.032	0.296	0.109	0.913	1.033	0.305	0.107	0.915	0.579	1.843
GAR1	1.106	0.336	3.292	0.001	3.022	1.015	1.992	0.046	1.564	5.837

GAPVD1	0.368	0.298	1.235	0.217	1.445	0.431	1.033	0.302	0.806	2.591
GAPDHS	0.265	0.298	0.892	0.372	1.304	0.388	0.784	0.433	0.728	2.337
GAPDH	-0.175	0.296	-0.591	0.555	0.839	0.249	-0.646	0.518	0.47	1.5
GAP43	0.293	0.298	0.986	0.324	1.341	0.399	0.855	0.393	0.748	2.403
GANAB	-0.43	0.3	-1.432	0.152	0.651	0.195	-1.789	0.074	0.362	1.171
GAN	0.518	0.302	1.715	0.086	1.679	0.508	1.338	0.181	0.929	3.037
GAMT	-0.074	0.297	-0.25	0.803	0.928	0.276	-0.259	0.795	0.519	1.662
GALT	0.931	0.321	2.905	0.004	2.538	0.813	1.89	0.059	1.354	4.757
GALR3	-0.056	0.297	-0.189	0.85	0.945	0.281	-0.194	0.846	0.528	1.693
GALR2	-0.031	0.295	-0.106	0.915	0.969	0.286	-0.108	0.914	0.543	1.729
GALR1	0.012	0.295	0.042	0.966	1.013	0.299	0.042	0.967	0.568	1.806
GALNT8	0.204	0.296	0.689	0.491	1.227	0.364	0.623	0.533	0.686	2.193
GALNT7	-0.307	0.298	-1.03	0.303	0.736	0.219	-1.206	0.228	0.411	1.319
GALNT6	-0.077	0.295	-0.261	0.794	0.926	0.273	-0.271	0.786	0.519	1.652
GALNT3	-0.458	0.3	-1.525	0.127	0.632	0.19	-1.935	0.053	0.351	1.14
GALNT2	0.136	0.296	0.461	0.645	1.146	0.339	0.431	0.667	0.642	2.045
GALNT14	-0.083	0.295	-0.28	0.78	0.921	0.272	-0.292	0.771	0.516	1.642
GALNT12	-0.506	0.301	-1.684	0.092	0.603	0.181	-2.193	0.028	0.334	1.086
GALNT11	-0.261	0.297	-0.877	0.381	0.771	0.229	-1.001	0.317	0.43	1.38
GALNT10	-0.399	0.3	-1.332	0.183	0.671	0.201	-1.638	0.102	0.373	1.207
GALNT1	-0.761	0.315	-2.419	0.016	0.467	0.147	-3.625	0	0.252	0.866
GALNS	0.094	0.295	0.318	0.75	1.098	0.324	0.304	0.761	0.616	1.96
GALK2	-0.385	0.3	-1.286	0.199	0.68	0.204	-1.569	0.117	0.378	1.224
GALK1	0.126	0.297	0.423	0.672	1.134	0.337	0.398	0.691	0.634	2.03
GALE	-0.226	0.298	-0.759	0.448	0.798	0.238	-0.851	0.395	0.445	1.43
GALC	-0.481	0.303	-1.59	0.112	0.618	0.187	-2.043	0.041	0.341	1.118
GAL3ST4	-0.188	0.296	-0.634	0.526	0.829	0.246	-0.697	0.486	0.464	1.481
GAL3ST1	-0.111	0.296	-0.375	0.708	0.895	0.265	-0.396	0.692	0.5	1.6
GAL	-0.084	0.295	-0.283	0.777	0.92	0.272	-0.296	0.768	0.515	1.641
GAK	0.019	0.296	0.065	0.949	1.019	0.301	0.064	0.949	0.571	1.82
GAGE1	-0.477	0.302	-1.577	0.115	0.621	0.188	-2.021	0.043	0.343	1.123
GADD45GIP1	0.411	0.298	1.381	0.167	1.509	0.45	1.132	0.258	0.841	2.707
GADD45G	-0.158	0.296	-0.534	0.593	0.854	0.253	-0.579	0.563	0.478	1.526
GADD45B	0.135	0.296	0.457	0.647	1.145	0.339	0.428	0.669	0.641	2.046
GADD45A	-0.552	0.31	-1.779	0.075	0.576	0.179	-2.375	0.018	0.313	1.058
GAD2	0.022	0.295	0.074	0.941	1.022	0.302	0.073	0.942	0.573	1.824
GAD1	-0.25	0.298	-0.839	0.401	0.779	0.232	-0.953	0.341	0.435	1.396
GABRR2	0.282	0.298	0.949	0.343	1.326	0.395	0.827	0.408	0.74	2.376

GABRR1	0.111	0.296	0.375	0.708	1.117	0.33	0.355	0.723	0.626	1.994
GABRQ	0.024	0.296	0.082	0.935	1.024	0.303	0.081	0.936	0.573	1.83
GABRP	-0.212	0.296	-0.715	0.474	0.809	0.239	-0.797	0.426	0.453	1.445
GABRG3	0.298	0.298	1.001	0.317	1.348	0.401	0.866	0.387	0.752	2.416
GABRG2	-0.184	0.297	-0.62	0.535	0.832	0.247	-0.681	0.496	0.465	1.488
GABRD	-0.011	0.295	-0.039	0.969	0.989	0.292	-0.039	0.969	0.554	1.763
GABRB3	-0.298	0.3	-0.993	0.321	0.742	0.223	-1.157	0.247	0.412	1.336
GABRB2	-0.33	0.298	-1.108	0.268	0.719	0.214	-1.313	0.189	0.401	1.289
GABRB1	0.147	0.296	0.495	0.621	1.158	0.343	0.46	0.645	0.648	2.071
GABRA6	-0.243	0.297	-0.819	0.413	0.784	0.233	-0.927	0.354	0.438	1.403
GABRA5	-0.029	0.296	-0.097	0.923	0.972	0.288	-0.099	0.921	0.543	1.737
GABRA4	-0.27	0.297	-0.911	0.362	0.763	0.226	-1.046	0.296	0.427	1.365
GABRA3	-0.046	0.295	-0.155	0.876	0.955	0.282	-0.159	0.874	0.536	1.703
GABRA2	0.751	0.31	2.422	0.015	2.12	0.657	1.703	0.089	1.154	3.893
GABRA1	-0.283	0.3	-0.945	0.344	0.753	0.226	-1.093	0.274	0.418	1.356
GABPB1-IT1	-0.058	0.295	-0.197	0.843	0.943	0.279	-0.203	0.839	0.529	1.683
GABPB1	-0.343	0.303	-1.131	0.258	0.709	0.215	-1.35	0.177	0.391	1.286
GABPA	0.638	0.306	2.087	0.037	1.893	0.579	1.542	0.123	1.04	3.449
GABBR2	0.379	0.3	1.262	0.207	1.46	0.438	1.05	0.294	0.811	2.629
GABBR1	0.267	0.3	0.89	0.374	1.306	0.392	0.781	0.435	0.725	2.354
GABARAPL3	0.388	0.298	1.301	0.193	1.474	0.44	1.078	0.281	0.822	2.645
GABARAPL2	0.186	0.295	0.631	0.528	1.205	0.356	0.575	0.565	0.675	2.149
GABARAPL1	-0.098	0.295	-0.331	0.74	0.907	0.268	-0.348	0.728	0.508	1.617
GABARAP	-0.698	0.315	-2.214	0.027	0.497	0.157	-3.203	0.001	0.268	0.923
GAB2	0.28	0.296	0.943	0.346	1.323	0.392	0.823	0.411	0.74	2.365
GAB1	-0.278	0.298	-0.934	0.35	0.757	0.226	-1.077	0.281	0.422	1.357
GAA	0.138	0.296	0.466	0.642	1.148	0.339	0.435	0.664	0.643	2.048
G6PD	0.366	0.298	1.23	0.219	1.442	0.429	1.03	0.303	0.805	2.584
G6PC3	0.273	0.297	0.92	0.358	1.314	0.39	0.805	0.421	0.735	2.349
G6PC2	0.219	0.299	0.733	0.463	1.245	0.372	0.659	0.51	0.693	2.234
G6PC	0.361	0.3	1.205	0.228	1.435	0.43	1.012	0.312	0.798	2.582
G3BP2	0.568	0.31	1.831	0.067	1.765	0.548	1.396	0.163	0.961	3.244
G3BP1	0.092	0.295	0.311	0.756	1.096	0.324	0.297	0.766	0.615	1.956
G0S2	0.46	0.302	1.521	0.128	1.584	0.479	1.22	0.223	0.876	2.864
FZR1	0.337	0.298	1.132	0.258	1.4	0.417	0.961	0.337	0.782	2.509
FZD9	0.314	0.298	1.056	0.291	1.369	0.407	0.906	0.365	0.764	2.453
FZD7	-0.267	0.296	-0.9	0.368	0.766	0.227	-1.032	0.302	0.428	1.369
FZD6	0.228	0.298	0.764	0.445	1.256	0.374	0.684	0.494	0.7	2.252

FZD5	0.668	0.31	2.156	0.031	1.95	0.604	1.573	0.116	1.063	3.578
FZD4	-0.119	0.297	-0.4	0.689	0.888	0.264	-0.425	0.671	0.496	1.589
FZD3	-0.419	0.3	-1.397	0.162	0.658	0.197	-1.735	0.083	0.365	1.184
FZD2	0.331	0.298	1.11	0.267	1.392	0.415	0.945	0.345	0.776	2.498
FZD10	-0.029	0.295	-0.1	0.92	0.971	0.287	-0.101	0.919	0.544	1.732
FZD1	-0.609	0.306	-1.993	0.046	0.544	0.166	-2.744	0.006	0.299	0.99
FYN	-0.331	0.298	-1.112	0.266	0.718	0.214	-1.318	0.187	0.401	1.287
FYCO1	-0.311	0.298	-1.045	0.296	0.732	0.218	-1.226	0.22	0.409	1.313
FYB1	0.08	0.295	0.271	0.786	1.083	0.32	0.261	0.794	0.607	1.933
FXYD7	-0.237	0.296	-0.799	0.424	0.789	0.234	-0.902	0.367	0.441	1.411
FXYD6	-0.217	0.296	-0.732	0.464	0.805	0.238	-0.818	0.414	0.45	1.439
FXYD5	-0.6	0.31	-1.933	0.053	0.549	0.17	-2.647	0.008	0.299	1.009
FXYD3	-0.156	0.296	-0.528	0.598	0.855	0.253	-0.571	0.568	0.479	1.528
FXYD2	0.425	0.302	1.406	0.16	1.529	0.462	1.145	0.252	0.846	2.764
FXYD1	-0.36	0.298	-1.21	0.226	0.698	0.208	-1.456	0.145	0.389	1.25
FXR2	0.06	0.295	0.202	0.84	1.061	0.314	0.196	0.845	0.595	1.894
FXR1	0.502	0.302	1.659	0.097	1.652	0.5	1.304	0.192	0.913	2.988
FXN	0.088	0.297	0.297	0.767	1.092	0.324	0.284	0.776	0.611	1.953
FUZ	-0.366	0.303	-1.208	0.227	0.693	0.21	-1.459	0.145	0.383	1.256
FUT9	0.065	0.297	0.219	0.827	1.067	0.317	0.212	0.832	0.596	1.909
FUT8	-0.857	0.315	-2.722	0.006	0.424	0.134	-4.308	0	0.229	0.787
FUT7	0.305	0.297	1.028	0.304	1.357	0.403	0.886	0.376	0.758	2.427
FUT6	-0.385	0.299	-1.286	0.199	0.681	0.204	-1.568	0.117	0.379	1.223
FUT5	-0.017	0.296	-0.057	0.955	0.983	0.291	-0.057	0.954	0.551	1.755
FUT4	-0.07	0.295	-0.235	0.814	0.933	0.276	-0.244	0.807	0.523	1.665
FUT3	-0.056	0.295	-0.189	0.85	0.946	0.279	-0.194	0.846	0.53	1.687
FUT2	0.209	0.298	0.702	0.483	1.232	0.367	0.634	0.526	0.688	2.209
FUT1	0.591	0.303	1.954	0.051	1.807	0.547	1.475	0.14	0.998	3.27
FUS	0.429	0.3	1.429	0.153	1.536	0.462	1.162	0.245	0.852	2.768
FURIN	0.079	0.296	0.265	0.791	1.082	0.32	0.255	0.799	0.606	1.932
FUCA1	-0.364	0.3	-1.213	0.225	0.695	0.208	-1.463	0.143	0.386	1.251
FUBP3	0.512	0.306	1.675	0.094	1.669	0.51	1.311	0.19	0.917	3.039
FUBP1	-0.144	0.296	-0.488	0.626	0.866	0.256	-0.525	0.6	0.484	1.546
FTSJ3	0.08	0.296	0.271	0.786	1.083	0.32	0.261	0.794	0.607	1.934
FTSJ1	0.049	0.296	0.167	0.867	1.051	0.311	0.163	0.871	0.588	1.876
FTO	-0.662	0.315	-2.101	0.036	0.516	0.163	-2.978	0.003	0.278	0.957
FTL	0.172	0.297	0.581	0.561	1.188	0.352	0.534	0.594	0.664	2.125
FTH1P5	-0.186	0.3	-0.62	0.535	0.83	0.249	-0.682	0.496	0.461	1.494

FTH1	-0.146	0.299	-0.487	0.626	0.864	0.258	-0.524	0.6	0.481	1.553
FTCD	-0.213	0.296	-0.719	0.472	0.809	0.239	-0.801	0.423	0.453	1.443
FSTL4	-0.028	0.295	-0.094	0.925	0.973	0.287	-0.095	0.924	0.545	1.735
FSTL3	-0.211	0.296	-0.712	0.477	0.81	0.24	-0.792	0.428	0.453	1.447
FSTL1	-0.934	0.315	-2.96	0.003	0.393	0.124	-4.896	0	0.212	0.729
FST	-0.923	0.321	-2.878	0.004	0.397	0.127	-4.73	0	0.212	0.745
FSHR	-0.083	0.296	-0.279	0.78	0.921	0.272	-0.291	0.771	0.516	1.644
FSHB	0.238	0.297	0.802	0.423	1.269	0.377	0.713	0.476	0.709	2.272
FSD1	-0.132	0.295	-0.448	0.654	0.876	0.259	-0.479	0.632	0.491	1.563
FSCN3	0.131	0.301	0.436	0.663	1.14	0.343	0.408	0.683	0.632	2.055
FSCN2	-0.264	0.298	-0.887	0.375	0.768	0.229	-1.016	0.31	0.429	1.376
FSCN1	-0.535	0.3	-1.779	0.075	0.586	0.176	-2.352	0.019	0.325	1.056
FRZB	-0.671	0.306	-2.196	0.028	0.511	0.156	-3.13	0.002	0.281	0.93
FRYL	-0.547	0.303	-1.809	0.07	0.579	0.175	-2.408	0.016	0.32	1.047
FRY	-0.576	0.31	-1.857	0.063	0.562	0.174	-2.511	0.012	0.306	1.032
FRS3	0.303	0.298	1.015	0.31	1.354	0.404	0.876	0.381	0.754	2.43
FRS2	0.321	0.297	1.081	0.28	1.378	0.409	0.924	0.355	0.77	2.467
FRRS1L	-0.02	0.296	-0.067	0.946	0.98	0.29	-0.068	0.946	0.549	1.752
FRMPD4	0.079	0.295	0.267	0.789	1.082	0.319	0.257	0.797	0.607	1.93
FRMPD1	0.247	0.298	0.83	0.406	1.28	0.381	0.736	0.462	0.714	2.295
FRMD8	-0.203	0.296	-0.687	0.492	0.816	0.242	-0.762	0.446	0.457	1.458
FRMD4B	-0.706	0.306	-2.307	0.021	0.494	0.151	-3.352	0.001	0.271	0.899
FRMD4A	-0.155	0.298	-0.521	0.602	0.856	0.255	-0.564	0.573	0.477	1.535
FRMD1	0.028	0.295	0.094	0.925	1.028	0.303	0.093	0.926	0.577	1.833
FRK	0.593	0.303	1.956	0.05	1.809	0.548	1.476	0.14	0.999	3.274
FRAT2	0.562	0.306	1.836	0.066	1.754	0.537	1.405	0.16	0.963	3.195
FRAT1	0.38	0.3	1.269	0.204	1.463	0.438	1.056	0.291	0.813	2.631
FRAS1	0.396	0.3	1.321	0.187	1.485	0.445	1.091	0.275	0.826	2.672
FPR3	0.833	0.315	2.644	0.008	2.299	0.724	1.795	0.073	1.24	4.262
FPR2	0.144	0.295	0.488	0.626	1.155	0.341	0.454	0.65	0.647	2.06
FPR1	0.286	0.298	0.959	0.338	1.331	0.396	0.834	0.404	0.742	2.386
FPGT	-0.077	0.297	-0.259	0.795	0.926	0.275	-0.27	0.787	0.517	1.658
FPGS	-0.169	0.296	-0.572	0.567	0.844	0.25	-0.623	0.533	0.472	1.508
FOXRED2	0.158	0.299	0.528	0.597	1.171	0.35	0.488	0.625	0.652	2.105
FOXP3	-0.351	0.299	-1.176	0.24	0.704	0.21	-1.409	0.159	0.392	1.264
FOXO4	-0.281	0.301	-0.934	0.35	0.755	0.227	-1.079	0.281	0.419	1.361
FOXO3	0.416	0.302	1.378	0.168	1.516	0.458	1.127	0.26	0.839	2.742
FOXO1	-0.23	0.296	-0.778	0.437	0.794	0.235	-0.874	0.382	0.445	1.419

FOXN3-AS2	0.268	0.298	0.899	0.369	1.307	0.389	0.789	0.43	0.729	2.342
FOXN3	0.257	0.298	0.863	0.388	1.293	0.385	0.761	0.447	0.722	2.316
FOXN2	0.84	0.311	2.702	0.007	2.317	0.721	1.828	0.068	1.259	4.262
FOXN1	-0.216	0.296	-0.731	0.465	0.805	0.239	-0.816	0.415	0.451	1.439
FOXM1	0.691	0.306	2.26	0.024	1.996	0.61	1.632	0.103	1.096	3.634
FOXL2	0.21	0.297	0.71	0.478	1.234	0.366	0.64	0.522	0.69	2.207
FOXL1	0.434	0.299	1.452	0.146	1.543	0.461	1.178	0.239	0.859	2.77
FOXK2	0.397	0.3	1.324	0.185	1.488	0.446	1.093	0.275	0.826	2.678
FOXJ3	0.263	0.296	0.887	0.375	1.3	0.385	0.78	0.435	0.728	2.323
FOXJ2	-0.342	0.3	-1.142	0.253	0.71	0.213	-1.362	0.173	0.395	1.277
FOXJ1	0.185	0.296	0.624	0.533	1.203	0.356	0.569	0.569	0.673	2.148
FOXI1	0.166	0.296	0.561	0.575	1.18	0.349	0.517	0.605	0.661	2.107
FOXH1	0.006	0.296	0.02	0.984	1.006	0.297	0.02	0.984	0.564	1.796
FOXG1	0.233	0.298	0.785	0.433	1.263	0.376	0.7	0.484	0.705	2.263
FOXF2	-0.503	0.3	-1.679	0.093	0.605	0.181	-2.182	0.029	0.336	1.088
FOXF1	-0.151	0.295	-0.512	0.608	0.86	0.254	-0.553	0.58	0.482	1.534
FOXE3	0.127	0.297	0.428	0.669	1.136	0.337	0.402	0.688	0.634	2.033
FOXE1	-0.389	0.3	-1.297	0.195	0.678	0.203	-1.585	0.113	0.377	1.22
FOXD3	0.212	0.298	0.712	0.476	1.236	0.368	0.642	0.521	0.69	2.216
FOXD2	0.233	0.297	0.786	0.432	1.263	0.375	0.701	0.483	0.706	2.261
FOXD1	-0.087	0.295	-0.296	0.767	0.916	0.271	-0.309	0.757	0.514	1.634
FOXC2	0	0.296	-0.001	0.999	1	0.296	-0.001	0.999	0.559	1.787
FOXC1	-0.384	0.298	-1.288	0.198	0.681	0.203	-1.57	0.116	0.38	1.222
FOXB1	-0.144	0.298	-0.485	0.628	0.866	0.258	-0.522	0.602	0.483	1.552
FOXA2	0.282	0.297	0.948	0.343	1.326	0.394	0.826	0.409	0.74	2.375
FOXA1	0.009	0.295	0.029	0.977	1.009	0.298	0.029	0.977	0.565	1.799
FOSL2	-0.11	0.296	-0.372	0.71	0.896	0.265	-0.393	0.694	0.501	1.6
FOSL1	0.317	0.297	1.068	0.285	1.373	0.408	0.915	0.36	0.767	2.458
FOSB	-0.28	0.3	-0.933	0.351	0.756	0.227	-1.076	0.282	0.42	1.361
FOS	-0.224	0.298	-0.753	0.451	0.799	0.238	-0.844	0.399	0.445	1.433
FOLR3	-0.346	0.298	-1.163	0.245	0.707	0.211	-1.39	0.165	0.394	1.268
FOLR2	-0.423	0.299	-1.416	0.157	0.655	0.196	-1.763	0.078	0.365	1.177
FOLR1	0.189	0.298	0.635	0.525	1.209	0.36	0.579	0.563	0.674	2.168
FOLH1B	0.266	0.298	0.895	0.371	1.305	0.388	0.785	0.432	0.728	2.338
FOLH1	0.17	0.296	0.574	0.566	1.185	0.351	0.528	0.598	0.663	2.118
FOCAD	0.286	0.296	0.965	0.335	1.331	0.395	0.839	0.401	0.745	2.38
FNTA	-0.023	0.297	-0.076	0.939	0.978	0.29	-0.077	0.939	0.547	1.749
FNDC8	0.633	0.303	2.09	0.037	1.884	0.571	1.548	0.122	1.04	3.412

FNDC4	-0.014	0.295	-0.048	0.962	0.986	0.291	-0.048	0.961	0.553	1.758
FNDC3B	0.071	0.295	0.24	0.811	1.073	0.317	0.231	0.817	0.602	1.915
FNDC3A	-0.696	0.306	-2.277	0.023	0.498	0.152	-3.291	0.001	0.274	0.907
FNDC11	-0.148	0.296	-0.499	0.618	0.863	0.255	-0.538	0.591	0.483	1.54
FNBP4	0.131	0.297	0.442	0.659	1.14	0.338	0.414	0.679	0.637	2.039
FNBP1L	0.272	0.298	0.913	0.361	1.312	0.391	0.799	0.424	0.732	2.351
FNBP1	-0.482	0.3	-1.608	0.108	0.617	0.185	-2.066	0.039	0.343	1.111
FN3KRP	0.408	0.3	1.362	0.173	1.504	0.451	1.119	0.263	0.836	2.707
FN3K	0.003	0.295	0.009	0.993	1.003	0.296	0.009	0.993	0.562	1.789
FN1	-0.145	0.295	-0.49	0.624	0.865	0.256	-0.528	0.598	0.485	1.543
FMR1	-0.085	0.297	-0.286	0.775	0.919	0.272	-0.298	0.766	0.514	1.643
FMOD	-0.5	0.3	-1.667	0.095	0.607	0.182	-2.163	0.031	0.337	1.092
FMO6P	0.06	0.295	0.205	0.838	1.062	0.314	0.199	0.842	0.596	1.894
FMO5	-0.459	0.302	-1.519	0.129	0.632	0.191	-1.928	0.054	0.349	1.143
FMO4	0.168	0.296	0.566	0.571	1.183	0.351	0.521	0.602	0.662	2.114
FMO3	-0.506	0.302	-1.675	0.094	0.603	0.182	-2.181	0.029	0.333	1.09
FMO2	0.285	0.298	0.959	0.338	1.33	0.396	0.834	0.404	0.742	2.384
FMO1	-0.087	0.296	-0.293	0.77	0.917	0.271	-0.306	0.76	0.514	1.637
FMNL1	-0.373	0.3	-1.246	0.213	0.688	0.206	-1.51	0.131	0.383	1.239
FLVCR2	0.09	0.295	0.305	0.76	1.094	0.323	0.292	0.77	0.613	1.953
FLT4	-0.22	0.298	-0.74	0.459	0.802	0.239	-0.828	0.408	0.447	1.438
FLT3LG	-0.341	0.298	-1.147	0.252	0.711	0.212	-1.366	0.172	0.397	1.274
FLT3	-0.474	0.302	-1.567	0.117	0.622	0.188	-2.005	0.045	0.344	1.126
FLT1	-0.044	0.295	-0.148	0.882	0.957	0.283	-0.152	0.879	0.537	1.707
FLRT3	0.027	0.295	0.092	0.927	1.027	0.303	0.09	0.928	0.576	1.833
FLRT1	-0.054	0.295	-0.184	0.854	0.947	0.28	-0.189	0.85	0.531	1.689
FLOT2	-0.396	0.3	-1.32	0.187	0.673	0.202	-1.619	0.105	0.374	1.211
FLOT1	-0.04	0.296	-0.135	0.892	0.961	0.284	-0.138	0.89	0.538	1.715
FLNC	0.44	0.303	1.452	0.146	1.552	0.47	1.175	0.24	0.858	2.81
FLNB	-0.733	0.31	-2.367	0.018	0.48	0.149	-3.492	0	0.262	0.882
FLNA	0.03	0.296	0.103	0.918	1.031	0.305	0.102	0.919	0.577	1.841
FLJ42627	0.427	0.307	1.392	0.164	1.533	0.47	1.133	0.257	0.84	2.797
FLJ20712	-0.024	0.295	-0.08	0.936	0.977	0.288	-0.081	0.935	0.547	1.742
FLJ13224	0.063	0.297	0.21	0.833	1.065	0.316	0.204	0.838	0.595	1.906
FLII	0.03	0.295	0.1	0.92	1.03	0.304	0.099	0.921	0.577	1.838
FLI1	-0.907	0.313	-2.898	0.004	0.404	0.126	-4.717	0	0.219	0.746
FLG	0.517	0.303	1.71	0.087	1.677	0.507	1.335	0.182	0.927	3.035
FLCN	0.151	0.3	0.503	0.615	1.163	0.349	0.467	0.641	0.646	2.093

FLAD1	0.066	0.295	0.225	0.822	1.069	0.315	0.217	0.828	0.599	1.905
FKTN	0.082	0.295	0.278	0.781	1.086	0.32	0.267	0.79	0.609	1.936
FKSG49	0.638	0.31	2.058	0.04	1.892	0.586	1.522	0.128	1.031	3.472
FKRP	-0.092	0.296	-0.311	0.756	0.912	0.27	-0.326	0.744	0.511	1.628
FKBPL	0.879	0.315	2.793	0.005	2.408	0.758	1.858	0.063	1.3	4.462
FKBP9	-0.488	0.3	-1.629	0.103	0.614	0.184	-2.1	0.036	0.341	1.104
FKBP8	-0.009	0.295	-0.029	0.977	0.991	0.293	-0.03	0.976	0.556	1.769
FKBP6	0.046	0.295	0.155	0.876	1.047	0.309	0.152	0.879	0.587	1.868
FKBP5	-0.751	0.315	-2.385	0.017	0.472	0.149	-3.554	0	0.255	0.875
FKBP4	-0.018	0.296	-0.061	0.951	0.982	0.29	-0.062	0.951	0.55	1.753
FKBP3	0.84	0.31	2.708	0.007	2.317	0.719	1.832	0.067	1.261	4.255
FKBP2	-0.282	0.301	-0.937	0.349	0.754	0.227	-1.083	0.279	0.418	1.361
FKBP1B	0.076	0.296	0.258	0.796	1.079	0.319	0.248	0.804	0.604	1.927
FKBP1A	-0.26	0.297	-0.875	0.382	0.771	0.229	-0.999	0.318	0.43	1.381
FKBP15	-0.531	0.302	-1.757	0.079	0.588	0.178	-2.319	0.02	0.325	1.063
FKBP14	-0.399	0.3	-1.331	0.183	0.671	0.201	-1.635	0.102	0.373	1.208
FKBP11	-0.622	0.306	-2.036	0.042	0.537	0.164	-2.824	0.005	0.295	0.977
FKBP10	0.461	0.3	1.537	0.124	1.585	0.475	1.232	0.218	0.881	2.853
FJX1	0.348	0.298	1.17	0.242	1.417	0.422	0.988	0.323	0.791	2.539
FIS1	-0.187	0.299	-0.626	0.531	0.829	0.248	-0.688	0.491	0.462	1.49
FIP1L1	0.484	0.301	1.609	0.108	1.623	0.488	1.276	0.202	0.9	2.926
FILIP1L	-0.959	0.316	-3.034	0.002	0.383	0.121	-5.092	0	0.206	0.712
FIG4	0.188	0.297	0.634	0.526	1.207	0.359	0.578	0.564	0.674	2.161
FICD	0.225	0.302	0.745	0.457	1.252	0.378	0.667	0.505	0.693	2.262
FIBP	0.354	0.297	1.189	0.234	1.424	0.424	1.002	0.316	0.795	2.551
FHOD3	-0.632	0.306	-2.064	0.039	0.532	0.163	-2.878	0.004	0.292	0.969
FHOD1	-0.193	0.298	-0.649	0.516	0.824	0.245	-0.716	0.474	0.46	1.477
FHL5	-0.592	0.306	-1.937	0.053	0.553	0.169	-2.643	0.008	0.304	1.007
FHL3	-0.194	0.299	-0.65	0.516	0.824	0.246	-0.717	0.473	0.459	1.479
FHL2	-0.238	0.298	-0.801	0.423	0.788	0.235	-0.905	0.366	0.44	1.412
FHL1	-0.167	0.295	-0.564	0.573	0.847	0.25	-0.613	0.54	0.474	1.511
FHIT	-0.025	0.295	-0.086	0.932	0.975	0.288	-0.087	0.931	0.546	1.74
FH	-0.242	0.298	-0.815	0.415	0.785	0.233	-0.922	0.356	0.438	1.406
FGR	-0.445	0.3	-1.483	0.138	0.641	0.192	-1.868	0.062	0.356	1.154
FGL2	-0.652	0.306	-2.131	0.033	0.521	0.159	-3.005	0.003	0.286	0.949
FGL1	0.339	0.3	1.132	0.258	1.404	0.42	0.96	0.337	0.78	2.525
FGGY	-0.386	0.302	-1.278	0.201	0.68	0.205	-1.56	0.119	0.376	1.229
FGG	0.275	0.296	0.929	0.353	1.317	0.39	0.812	0.417	0.737	2.353

FGFR4	0.389	0.303	1.286	0.198	1.476	0.447	1.066	0.287	0.816	2.671
FGFR3	0.134	0.296	0.452	0.651	1.143	0.338	0.423	0.672	0.64	2.042
FGFR2	-0.107	0.295	-0.364	0.716	0.898	0.265	-0.384	0.701	0.503	1.602
FGFR1	-0.344	0.298	-1.157	0.247	0.709	0.211	-1.381	0.167	0.395	1.27
FGFBP1	-0.136	0.297	-0.457	0.648	0.873	0.259	-0.49	0.624	0.488	1.563
FGF9	-0.128	0.295	-0.432	0.666	0.88	0.26	-0.461	0.645	0.493	1.571
FGF8	0.222	0.296	0.748	0.455	1.248	0.37	0.671	0.502	0.698	2.23
FGF7	-0.545	0.303	-1.8	0.072	0.58	0.176	-2.393	0.017	0.32	1.05
FGF6	0.496	0.3	1.65	0.099	1.641	0.493	1.301	0.193	0.911	2.957
FGF5	0.046	0.298	0.155	0.877	1.047	0.313	0.151	0.88	0.583	1.88
FGF4	-0.111	0.297	-0.374	0.708	0.895	0.266	-0.396	0.692	0.5	1.602
FGF3	0.108	0.296	0.364	0.716	1.114	0.33	0.345	0.73	0.623	1.99
FGF23	0.416	0.3	1.39	0.165	1.517	0.454	1.137	0.256	0.843	2.729
FGF22	-0.305	0.298	-1.025	0.305	0.737	0.219	-1.199	0.231	0.411	1.321
FGF21	0.101	0.296	0.343	0.732	1.107	0.327	0.326	0.744	0.62	1.976
FGF20	0.41	0.299	1.374	0.17	1.507	0.45	1.127	0.26	0.839	2.705
FGF2	-0.268	0.297	-0.901	0.367	0.765	0.227	-1.034	0.301	0.428	1.369
FGF18	0.255	0.298	0.855	0.392	1.291	0.385	0.755	0.45	0.719	2.316
FGF17	0.064	0.297	0.214	0.831	1.066	0.317	0.207	0.836	0.595	1.909
FGF16	0.211	0.296	0.712	0.476	1.235	0.366	0.642	0.521	0.691	2.206
FGF14	-0.49	0.302	-1.62	0.105	0.613	0.185	-2.09	0.037	0.339	1.108
FGF13	0.262	0.298	0.877	0.38	1.299	0.388	0.772	0.44	0.724	2.332
FGF12	0.135	0.295	0.456	0.648	1.144	0.338	0.427	0.669	0.641	2.041
FGF1	-0.124	0.296	-0.42	0.674	0.883	0.261	-0.448	0.654	0.494	1.577
FGD6	-0.228	0.296	-0.768	0.442	0.796	0.236	-0.863	0.388	0.446	1.423
FGD2	0.416	0.3	1.387	0.166	1.515	0.454	1.135	0.257	0.842	2.726
FGD1	0.518	0.302	1.713	0.087	1.678	0.507	1.337	0.181	0.928	3.035
FGB	0.208	0.297	0.7	0.484	1.231	0.365	0.632	0.527	0.688	2.202
FGA	0.411	0.3	1.37	0.171	1.509	0.453	1.123	0.261	0.838	2.717
FFAR2	0.098	0.295	0.331	0.741	1.103	0.325	0.315	0.753	0.618	1.966
FEZF2	0.085	0.295	0.288	0.774	1.089	0.321	0.276	0.783	0.61	1.942
FEZ2	-0.587	0.306	-1.92	0.055	0.556	0.17	-2.611	0.009	0.306	1.012
FEZ1	-0.508	0.303	-1.681	0.093	0.601	0.182	-2.19	0.028	0.332	1.088
FEV	0.211	0.296	0.713	0.476	1.235	0.366	0.642	0.521	0.691	2.207
FETUB	0.164	0.295	0.556	0.578	1.179	0.348	0.513	0.608	0.66	2.103
FES	0.016	0.297	0.055	0.956	1.017	0.302	0.055	0.956	0.568	1.819
FERMT2	0.032	0.295	0.107	0.915	1.032	0.305	0.105	0.916	0.578	1.842
FERMT1	-0.011	0.295	-0.038	0.97	0.989	0.292	-0.038	0.97	0.555	1.763

FER1L4	0.107	0.297	0.36	0.719	1.113	0.331	0.342	0.733	0.621	1.994
FER	0.29	0.297	0.978	0.328	1.336	0.396	0.849	0.396	0.747	2.39
FEN1	0.291	0.298	0.975	0.329	1.337	0.399	0.846	0.397	0.746	2.399
FEM1C	-0.141	0.296	-0.476	0.634	0.869	0.257	-0.511	0.609	0.487	1.551
FEM1B	-0.001	0.295	-0.004	0.997	0.999	0.295	-0.004	0.997	0.56	1.781
FECH	0.328	0.296	1.107	0.268	1.388	0.411	0.944	0.345	0.777	2.482
FDXR	0.316	0.297	1.062	0.288	1.371	0.408	0.911	0.362	0.766	2.456
FDX1	-0.564	0.306	-1.842	0.065	0.569	0.174	-2.474	0.013	0.312	1.037
FDPS	0.41	0.298	1.376	0.169	1.508	0.45	1.129	0.259	0.84	2.705
FDFT1	0.248	0.296	0.838	0.402	1.282	0.38	0.742	0.458	0.717	2.29
FCRL2	-0.592	0.303	-1.955	0.051	0.553	0.168	-2.668	0.008	0.305	1.001
FCN3	0.104	0.296	0.35	0.726	1.109	0.328	0.333	0.739	0.621	1.981
FCN2	-0.177	0.297	-0.597	0.55	0.838	0.248	-0.653	0.514	0.468	1.498
FCN1	-0.189	0.296	-0.638	0.523	0.828	0.245	-0.703	0.482	0.463	1.479
FCMR	-0.155	0.297	-0.521	0.602	0.857	0.254	-0.563	0.573	0.479	1.533
FCHSD2	-0.361	0.3	-1.204	0.229	0.697	0.209	-1.45	0.147	0.387	1.254
FCHO1	-0.386	0.298	-1.296	0.195	0.68	0.202	-1.581	0.114	0.379	1.219
FCGRT	-0.009	0.296	-0.029	0.977	0.991	0.293	-0.029	0.977	0.555	1.77
FCGR3B	0.044	0.295	0.149	0.881	1.045	0.308	0.146	0.884	0.586	1.863
FCGR2C	-0.369	0.3	-1.231	0.218	0.691	0.207	-1.489	0.137	0.384	1.244
FCGR2B	-0.214	0.298	-0.72	0.471	0.807	0.24	-0.803	0.422	0.45	1.446
FCGR2A	-0.269	0.3	-0.898	0.369	0.764	0.229	-1.031	0.303	0.424	1.375
FCGR1B	0.024	0.296	0.08	0.936	1.024	0.303	0.079	0.937	0.574	1.828
FCGR1A	-0.177	0.298	-0.594	0.552	0.838	0.25	-0.65	0.515	0.467	1.502
FCGBP	-0.49	0.306	-1.601	0.109	0.613	0.187	-2.066	0.039	0.336	1.116
FCF1	-0.013	0.297	-0.043	0.966	0.987	0.293	-0.043	0.966	0.552	1.767
FCER2	0.165	0.296	0.556	0.578	1.179	0.349	0.512	0.608	0.66	2.107
FCER1G	-0.065	0.295	-0.221	0.825	0.937	0.277	-0.229	0.819	0.525	1.671
FCER1A	-0.593	0.306	-1.94	0.052	0.552	0.169	-2.649	0.008	0.303	1.006
FCAR	0.124	0.295	0.419	0.675	1.132	0.334	0.394	0.693	0.634	2.019
FBXW7	-0.108	0.295	-0.364	0.716	0.898	0.265	-0.385	0.701	0.504	1.602
FBXW4P1	0.537	0.306	1.756	0.079	1.711	0.523	1.358	0.174	0.939	3.117
FBXW4	-0.56	0.306	-1.834	0.067	0.571	0.174	-2.459	0.014	0.314	1.039
FBXW2	-0.668	0.31	-2.156	0.031	0.513	0.159	-3.068	0.002	0.279	0.941
FBXW12	0.401	0.302	1.326	0.185	1.493	0.451	1.093	0.275	0.826	2.701
FBXW11	0.317	0.298	1.066	0.287	1.374	0.409	0.913	0.361	0.766	2.463
FBXO9	0.031	0.297	0.104	0.917	1.031	0.306	0.102	0.919	0.576	1.845
FBXO7	-0.199	0.297	-0.671	0.503	0.819	0.243	-0.742	0.458	0.458	1.466

FBXO5	0.66	0.306	2.158	0.031	1.934	0.591	1.58	0.114	1.062	3.521
FBXO46	0.613	0.311	1.974	0.048	1.846	0.573	1.476	0.14	1.004	3.394
FBXO42	-0.191	0.296	-0.646	0.518	0.826	0.245	-0.712	0.477	0.462	1.476
FBXO41	0.501	0.302	1.658	0.097	1.651	0.499	1.304	0.192	0.913	2.986
FBXO40	0.382	0.3	1.273	0.203	1.465	0.439	1.059	0.29	0.814	2.636
FBXO4	0.03	0.296	0.102	0.919	1.031	0.305	0.1	0.92	0.577	1.84
FBXO38	0.229	0.296	0.776	0.438	1.258	0.372	0.693	0.488	0.705	2.245
FBXO34	-0.064	0.296	-0.218	0.828	0.938	0.277	-0.225	0.822	0.525	1.675
FBXO31	0.445	0.3	1.487	0.137	1.561	0.468	1.2	0.23	0.868	2.808
FBXO3	-0.044	0.296	-0.15	0.881	0.957	0.283	-0.154	0.878	0.536	1.707
FBXO28	-0.405	0.3	-1.354	0.176	0.667	0.2	-1.669	0.095	0.371	1.199
FBXO24	0.473	0.3	1.574	0.116	1.605	0.482	1.254	0.21	0.89	2.891
FBXO22	0.425	0.3	1.419	0.156	1.53	0.458	1.156	0.248	0.85	2.752
FBXO21	-0.171	0.296	-0.577	0.564	0.843	0.25	-0.629	0.529	0.471	1.507
FBXO2	-0.047	0.297	-0.157	0.875	0.954	0.283	-0.161	0.872	0.534	1.707
FBXO17	0.043	0.296	0.145	0.885	1.044	0.309	0.142	0.887	0.585	1.863
FBXO11	-0.355	0.299	-1.19	0.234	0.701	0.209	-1.429	0.153	0.39	1.259
FBXL8	-0.189	0.298	-0.635	0.525	0.828	0.247	-0.699	0.485	0.461	1.484
FBXL7	-0.233	0.298	-0.783	0.434	0.792	0.236	-0.882	0.378	0.442	1.42
FBXL6	0.365	0.3	1.218	0.223	1.441	0.432	1.02	0.308	0.801	2.593
FBXL5	-0.488	0.306	-1.594	0.111	0.614	0.188	-2.054	0.04	0.337	1.118
FBXL4	0.658	0.306	2.154	0.031	1.932	0.59	1.578	0.115	1.061	3.517
FBXL2	0.116	0.296	0.391	0.696	1.123	0.333	0.369	0.712	0.628	2.007
FBXL18	-0.529	0.3	-1.764	0.078	0.589	0.177	-2.325	0.02	0.327	1.061
FBXL15	-0.218	0.298	-0.731	0.465	0.805	0.24	-0.816	0.414	0.449	1.442
FBXL14	-0.216	0.298	-0.723	0.47	0.806	0.241	-0.807	0.42	0.449	1.446
FBXL12	0.084	0.296	0.283	0.777	1.087	0.322	0.271	0.786	0.608	1.944
FBRS	0.146	0.295	0.496	0.62	1.158	0.342	0.461	0.645	0.649	2.066
FBP2	-0.338	0.299	-1.129	0.259	0.714	0.213	-1.343	0.179	0.397	1.282
FBP1	-0.09	0.297	-0.304	0.761	0.914	0.271	-0.318	0.75	0.511	1.634
FBN2	-0.426	0.298	-1.426	0.154	0.653	0.195	-1.777	0.076	0.364	1.173
FBN1	-0.453	0.3	-1.511	0.131	0.636	0.191	-1.911	0.056	0.353	1.144
FBLN5	-0.743	0.307	-2.422	0.015	0.476	0.146	-3.593	0	0.261	0.868
FBLN2	-0.686	0.31	-2.214	0.027	0.504	0.156	-3.181	0.001	0.274	0.924
FBLN1	-0.677	0.306	-2.213	0.027	0.508	0.155	-3.165	0.002	0.279	0.925
FBL	-0.33	0.298	-1.105	0.269	0.719	0.215	-1.309	0.19	0.401	1.29
FAXDC2	-0.252	0.297	-0.849	0.396	0.777	0.231	-0.966	0.334	0.434	1.391
FAU	0.477	0.302	1.578	0.115	1.611	0.487	1.255	0.21	0.891	2.913

FAT4	-0.733	0.307	-2.388	0.017	0.48	0.148	-3.523	0	0.263	0.877
FAT2	-0.001	0.295	-0.004	0.996	0.999	0.295	-0.004	0.996	0.56	1.781
FAT1	-0.131	0.297	-0.443	0.658	0.877	0.26	-0.473	0.636	0.49	1.568
FASTKD5	0.222	0.299	0.744	0.457	1.249	0.373	0.667	0.505	0.696	2.243
FASTKD3	-0.066	0.295	-0.224	0.822	0.936	0.276	-0.232	0.817	0.525	1.669
FASTKD2	0.252	0.296	0.852	0.394	1.287	0.381	0.753	0.452	0.72	2.3
FASTKD1	0.069	0.295	0.233	0.816	1.071	0.316	0.225	0.822	0.6	1.911
FASTK	0.081	0.296	0.274	0.784	1.085	0.321	0.263	0.792	0.607	1.938
FASN	-0.314	0.299	-1.051	0.293	0.731	0.218	-1.234	0.217	0.407	1.312
FASLG	-0.35	0.298	-1.173	0.241	0.705	0.21	-1.405	0.16	0.393	1.264
FAS	-0.604	0.303	-1.995	0.046	0.547	0.165	-2.74	0.006	0.302	0.989
FARSA	-0.02	0.298	-0.067	0.947	0.98	0.292	-0.067	0.946	0.547	1.756
FARS2	-0.359	0.298	-1.205	0.228	0.698	0.208	-1.45	0.147	0.389	1.252
FARP2	-0.197	0.296	-0.667	0.505	0.821	0.243	-0.737	0.461	0.459	1.467
FARP1	-0.216	0.298	-0.727	0.467	0.805	0.24	-0.811	0.417	0.449	1.444
FAR2	-0.467	0.302	-1.547	0.122	0.627	0.189	-1.972	0.049	0.346	1.133
FAP	-0.85	0.315	-2.7	0.007	0.427	0.135	-4.255	0	0.231	0.792
FANCL	0.614	0.306	2.006	0.045	1.848	0.566	1.499	0.134	1.014	3.367
FANCI	0.494	0.303	1.633	0.102	1.639	0.496	1.289	0.197	0.906	2.966
FANCG	0.805	0.31	2.597	0.009	2.237	0.693	1.784	0.074	1.218	4.107
FANCF	-0.14	0.299	-0.467	0.641	0.87	0.26	-0.501	0.617	0.484	1.563
FANCE	0.318	0.296	1.073	0.283	1.375	0.407	0.919	0.358	0.769	2.457
FANCC	0.599	0.31	1.929	0.054	1.819	0.565	1.451	0.147	0.99	3.342
FANCA	0.616	0.306	2.014	0.044	1.852	0.567	1.504	0.133	1.017	3.374
FAN1	-0.049	0.296	-0.165	0.869	0.952	0.282	-0.169	0.866	0.534	1.7
FAM98A	-0.07	0.296	-0.237	0.812	0.932	0.276	-0.246	0.806	0.522	1.664
FAM90A1	-0.303	0.298	-1.017	0.309	0.738	0.22	-1.188	0.235	0.412	1.325
FAM8A1	-0.148	0.295	-0.503	0.615	0.862	0.255	-0.542	0.588	0.483	1.538
FAM89B	0.032	0.295	0.107	0.915	1.032	0.305	0.106	0.916	0.579	1.84
FAM86C1P	0.031	0.295	0.106	0.916	1.032	0.305	0.104	0.917	0.578	1.841
FAM83E	0.093	0.295	0.315	0.753	1.097	0.324	0.301	0.764	0.615	1.958
FAM76A	0.757	0.315	2.404	0.016	2.132	0.671	1.686	0.092	1.15	3.952
FAM66D	-0.07	0.298	-0.237	0.813	0.932	0.277	-0.245	0.806	0.52	1.67
FAM53C	0.06	0.297	0.201	0.841	1.061	0.315	0.195	0.845	0.593	1.9
FAM53B	-0.066	0.295	-0.224	0.823	0.936	0.277	-0.232	0.817	0.524	1.67
FAM50B	0.026	0.295	0.089	0.929	1.026	0.303	0.087	0.93	0.576	1.83
FAM50A	0.509	0.299	1.701	0.089	1.663	0.498	1.333	0.182	0.926	2.99
FAM3C	-0.223	0.296	-0.753	0.452	0.8	0.237	-0.843	0.399	0.448	1.43

FAM3A	0.259	0.296	0.873	0.382	1.295	0.384	0.77	0.442	0.725	2.316
FAM32A	0.179	0.298	0.6	0.549	1.196	0.357	0.549	0.583	0.667	2.145
FAM30A	-0.705	0.306	-2.302	0.021	0.494	0.151	-3.344	0.001	0.271	0.901
FAM234B	-0.418	0.3	-1.394	0.163	0.658	0.197	-1.73	0.084	0.366	1.185
FAM224A	0.108	0.295	0.364	0.716	1.114	0.329	0.346	0.73	0.624	1.986
FAM222B	0.177	0.296	0.599	0.549	1.194	0.353	0.549	0.583	0.669	2.131
FAM216A	-0.006	0.295	-0.019	0.985	0.994	0.294	-0.019	0.985	0.557	1.774
FAM215A	-0.525	0.302	-1.74	0.082	0.591	0.179	-2.289	0.022	0.327	1.069
FAM214B	-0.321	0.3	-1.068	0.285	0.726	0.218	-1.259	0.208	0.403	1.307
FAM20B	-0.071	0.295	-0.239	0.811	0.932	0.275	-0.247	0.805	0.522	1.663
FAM205BP	0.515	0.302	1.702	0.089	1.673	0.506	1.331	0.183	0.925	3.026
FAM204A	-0.046	0.295	-0.155	0.876	0.955	0.282	-0.159	0.874	0.535	1.704
FAM193B	-0.131	0.297	-0.44	0.66	0.877	0.261	-0.47	0.638	0.49	1.571
FAM193A	0.066	0.296	0.224	0.823	1.068	0.316	0.217	0.828	0.599	1.907
FAM189B	0.411	0.298	1.378	0.168	1.509	0.45	1.13	0.258	0.841	2.707
FAM189A2	0.355	0.298	1.19	0.234	1.426	0.425	1.002	0.316	0.795	2.556
FAM189A1	-0.679	0.31	-2.193	0.028	0.507	0.157	-3.139	0.002	0.276	0.93
FAM186A	-0.118	0.297	-0.396	0.692	0.889	0.264	-0.42	0.674	0.497	1.59
FAM184A	0.115	0.295	0.39	0.696	1.122	0.331	0.369	0.712	0.629	2.001
FAM174C	-0.003	0.296	-0.012	0.991	0.997	0.295	-0.012	0.991	0.558	1.779
FAM174B	-0.696	0.315	-2.211	0.027	0.499	0.157	-3.195	0.001	0.269	0.924
FAM172A	-0.33	0.298	-1.107	0.268	0.719	0.214	-1.312	0.19	0.401	1.289
FAM171A1	-0.261	0.297	-0.881	0.378	0.77	0.228	-1.006	0.314	0.431	1.377
FAM169A	0.776	0.315	2.467	0.014	2.174	0.684	1.716	0.086	1.173	4.028
FAM168B	-0.201	0.297	-0.677	0.499	0.818	0.243	-0.749	0.454	0.457	1.463
FAM168A	-0.161	0.296	-0.544	0.587	0.851	0.252	-0.59	0.555	0.477	1.521
FAM163A	0.744	0.315	2.365	0.018	2.105	0.663	1.668	0.095	1.136	3.901
FAM162A	-0.108	0.297	-0.364	0.716	0.898	0.266	-0.385	0.7	0.502	1.605
FAM160B2	0.243	0.296	0.82	0.412	1.275	0.378	0.728	0.466	0.713	2.279
FAM155B	0.209	0.296	0.704	0.481	1.232	0.365	0.635	0.525	0.689	2.202
FAM155A	0.049	0.296	0.165	0.869	1.05	0.31	0.161	0.872	0.588	1.874
FAM153A	-0.153	0.296	-0.517	0.605	0.858	0.254	-0.559	0.576	0.48	1.534
FAM149B1	-0.127	0.296	-0.428	0.669	0.881	0.261	-0.456	0.648	0.493	1.574
FAM149A	-0.625	0.306	-2.044	0.041	0.535	0.164	-2.839	0.005	0.294	0.975
FAM13C	0.555	0.303	1.833	0.067	1.741	0.527	1.407	0.16	0.962	3.151
FAM13B	-0.164	0.295	-0.555	0.579	0.849	0.251	-0.603	0.546	0.476	1.514
FAM13A	0.561	0.302	1.856	0.064	1.752	0.53	1.42	0.156	0.969	3.17
FAM135A	0.345	0.298	1.159	0.246	1.412	0.42	0.98	0.327	0.788	2.531

FAM131B	0.382	0.3	1.273	0.203	1.464	0.439	1.058	0.29	0.814	2.635
FAM131A	0.528	0.3	1.762	0.078	1.696	0.509	1.369	0.171	0.942	3.053
FAM124B	0.006	0.295	0.022	0.982	1.007	0.297	0.022	0.983	0.564	1.796
FAM120C	0.459	0.301	1.527	0.127	1.582	0.476	1.225	0.221	0.878	2.852
FAM120A	-0.543	0.301	-1.808	0.071	0.581	0.175	-2.402	0.016	0.322	1.047
FAM118A	0.127	0.296	0.427	0.669	1.135	0.336	0.401	0.688	0.635	2.029
FAM117A	-0.286	0.296	-0.966	0.334	0.751	0.222	-1.118	0.263	0.42	1.342
FAM114A2	-0.152	0.297	-0.512	0.609	0.859	0.255	-0.553	0.58	0.48	1.536
FAM114A1	-0.155	0.299	-0.518	0.604	0.857	0.256	-0.56	0.575	0.477	1.538
FAM111A	0.398	0.3	1.329	0.184	1.489	0.447	1.096	0.273	0.828	2.681
FAM110D	0.076	0.296	0.256	0.798	1.079	0.319	0.246	0.805	0.604	1.926
FAM110B	-0.336	0.3	-1.12	0.263	0.715	0.214	-1.331	0.183	0.397	1.286
FAM107A	0.023	0.295	0.077	0.939	1.023	0.302	0.076	0.939	0.574	1.824
FAM106A	0.136	0.297	0.456	0.648	1.145	0.34	0.427	0.67	0.64	2.05
FAM102A	-0.389	0.3	-1.297	0.195	0.678	0.203	-1.585	0.113	0.377	1.22
FAIM2	-0.048	0.296	-0.162	0.871	0.953	0.282	-0.166	0.868	0.534	1.702
FAIM	0.792	0.307	2.575	0.01	2.207	0.678	1.779	0.075	1.208	4.031
FAHD2A	0.466	0.302	1.541	0.123	1.593	0.481	1.232	0.218	0.881	2.881
FAH	0.02	0.296	0.067	0.946	1.02	0.302	0.067	0.947	0.571	1.822
FAF2	0.441	0.298	1.478	0.139	1.554	0.464	1.195	0.232	0.866	2.789
FAF1	0.011	0.295	0.038	0.97	1.011	0.299	0.038	0.97	0.567	1.804
FADS3	0.322	0.298	1.081	0.28	1.38	0.411	0.925	0.355	0.77	2.473
FADS2	0.199	0.296	0.671	0.502	1.22	0.361	0.609	0.543	0.683	2.18
FADD	0.811	0.308	2.633	0.008	2.25	0.693	1.804	0.071	1.23	4.114
FABP7	0.072	0.297	0.242	0.808	1.075	0.32	0.234	0.815	0.6	1.925
FABP6	-0.142	0.296	-0.477	0.633	0.868	0.257	-0.513	0.608	0.486	1.552
FABP5	0.683	0.31	2.205	0.027	1.98	0.613	1.598	0.11	1.079	3.633
FABP4	0.046	0.295	0.154	0.877	1.047	0.309	0.151	0.88	0.587	1.868
FABP3	0.358	0.298	1.201	0.23	1.43	0.426	1.01	0.312	0.798	2.563
FABP2	-0.581	0.31	-1.876	0.061	0.559	0.173	-2.544	0.011	0.305	1.026
FABP1	0.304	0.298	1.02	0.308	1.355	0.403	0.88	0.379	0.756	2.428
FAAP24	0.139	0.296	0.47	0.639	1.149	0.34	0.438	0.661	0.644	2.052
FAAP100	0.531	0.302	1.758	0.079	1.701	0.514	1.364	0.173	0.941	3.076
FAAH	-0.466	0.307	-1.521	0.128	0.627	0.192	-1.938	0.053	0.344	1.144
FA2H	1.104	0.322	3.433	0.001	3.016	0.97	2.079	0.038	1.606	5.665
F9	-0.075	0.295	-0.253	0.8	0.928	0.274	-0.263	0.793	0.52	1.655
F8	-0.431	0.3	-1.436	0.151	0.65	0.195	-1.795	0.073	0.361	1.17
F7	0.412	0.299	1.378	0.168	1.51	0.452	1.129	0.259	0.84	2.714

F5	-0.164	0.296	-0.553	0.58	0.849	0.251	-0.601	0.548	0.475	1.517
F3	-0.326	0.298	-1.093	0.275	0.722	0.215	-1.292	0.196	0.403	1.295
F2RL3	0.116	0.295	0.393	0.694	1.123	0.332	0.371	0.711	0.629	2.004
F2RL2	-1.017	0.321	-3.166	0.002	0.362	0.116	-5.492	0	0.193	0.679
F2RL1	0.214	0.296	0.723	0.47	1.239	0.367	0.651	0.515	0.693	2.215
F2R	-0.61	0.302	-2.017	0.044	0.543	0.164	-2.779	0.005	0.3	0.983
F2	0.247	0.298	0.83	0.406	1.28	0.381	0.736	0.462	0.714	2.294
F13B	0.423	0.3	1.412	0.158	1.527	0.458	1.151	0.25	0.848	2.749
F13A1	-0.836	0.311	-2.689	0.007	0.433	0.135	-4.204	0	0.236	0.797
F12	0.026	0.296	0.09	0.929	1.027	0.303	0.088	0.93	0.575	1.833
F11R	0.731	0.31	2.357	0.018	2.076	0.643	1.673	0.094	1.131	3.811
F11	-0.112	0.296	-0.379	0.704	0.894	0.264	-0.401	0.688	0.501	1.596
F10	0.797	0.311	2.564	0.01	2.218	0.69	1.767	0.077	1.206	4.08
EZR	-0.108	0.296	-0.364	0.716	0.898	0.266	-0.384	0.701	0.502	1.604
EZH2	0.629	0.306	2.057	0.04	1.875	0.573	1.527	0.127	1.03	3.413
EZH1	-0.29	0.296	-0.977	0.328	0.748	0.222	-1.134	0.257	0.419	1.338
EYA4	-0.078	0.296	-0.265	0.791	0.925	0.274	-0.275	0.783	0.517	1.652
EYA3	-0.019	0.295	-0.065	0.948	0.981	0.29	-0.066	0.948	0.55	1.75
EYA2	0.033	0.295	0.112	0.911	1.034	0.305	0.11	0.912	0.58	1.843
EYA1	-0.029	0.295	-0.098	0.922	0.971	0.287	-0.099	0.921	0.545	1.732
EXTL3	0.777	0.31	2.507	0.012	2.176	0.675	1.743	0.081	1.185	3.995
EXTL2	0.108	0.296	0.364	0.716	1.114	0.33	0.345	0.73	0.623	1.991
EXTL1	0.502	0.3	1.671	0.095	1.652	0.496	1.314	0.189	0.917	2.975
EXT2	-0.041	0.296	-0.139	0.889	0.96	0.284	-0.142	0.887	0.537	1.714
EXT1	-0.04	0.295	-0.135	0.892	0.961	0.284	-0.138	0.89	0.539	1.713
EXPH5	-0.042	0.296	-0.143	0.886	0.959	0.283	-0.146	0.884	0.537	1.711
EXOSC9	0.806	0.31	2.601	0.009	2.24	0.694	1.786	0.074	1.22	4.112
EXOSC8	0.05	0.295	0.17	0.865	1.051	0.31	0.166	0.868	0.589	1.876
EXOSC7	0.334	0.298	1.121	0.262	1.396	0.416	0.953	0.34	0.779	2.503
EXOSC5	0.107	0.295	0.362	0.717	1.113	0.329	0.344	0.731	0.624	1.986
EXOSC4	0.65	0.306	2.127	0.033	1.916	0.586	1.564	0.118	1.052	3.487
EXOSC2	0.947	0.321	2.949	0.003	2.577	0.827	1.907	0.057	1.374	4.833
EXOSC10	-0.074	0.297	-0.251	0.802	0.928	0.275	-0.261	0.794	0.519	1.66
EXOSC1	0.129	0.295	0.438	0.661	1.138	0.336	0.411	0.681	0.638	2.03
EXOG	0.084	0.296	0.285	0.776	1.088	0.322	0.273	0.785	0.609	1.943
EXOC7	0.187	0.299	0.625	0.532	1.205	0.36	0.57	0.569	0.671	2.163
EXOC6B	0.388	0.299	1.299	0.194	1.474	0.44	1.077	0.282	0.821	2.645
EXOC5	-0.555	0.306	-1.815	0.069	0.574	0.175	-2.426	0.015	0.316	1.045

EXOC3	0.06	0.296	0.204	0.838	1.062	0.314	0.198	0.843	0.595	1.898
EXOC2	-0.306	0.298	-1.028	0.304	0.737	0.219	-1.202	0.229	0.411	1.32
EXOC1	-0.097	0.296	-0.328	0.743	0.908	0.269	-0.344	0.731	0.508	1.621
EXO5	0.25	0.297	0.841	0.4	1.284	0.382	0.744	0.457	0.717	2.301
EXO1	1.129	0.336	3.359	0.001	3.091	1.039	2.013	0.044	1.6	5.972
EXD3	1.301	0.337	3.861	0	3.674	1.238	2.16	0.031	1.898	7.111
EXD2	0.12	0.297	0.404	0.687	1.128	0.335	0.38	0.704	0.629	2.02
EWSR1	0.911	0.315	2.895	0.004	2.487	0.783	1.9	0.057	1.342	4.61
EVX1	0.686	0.31	2.215	0.027	1.987	0.616	1.603	0.109	1.082	3.647
EVPL	0.352	0.3	1.177	0.239	1.422	0.426	0.992	0.321	0.791	2.559
EVL	-0.712	0.31	-2.3	0.021	0.49	0.152	-3.355	0.001	0.267	0.9
EVI5	-0.505	0.302	-1.669	0.095	0.604	0.182	-2.171	0.03	0.334	1.092
EVI2B	-0.56	0.303	-1.851	0.064	0.571	0.173	-2.482	0.013	0.315	1.033
EVI2A	-0.744	0.307	-2.422	0.015	0.475	0.146	-3.595	0	0.26	0.868
EVC	-0.502	0.299	-1.677	0.093	0.605	0.181	-2.179	0.029	0.337	1.088
EVA1B	-0.334	0.3	-1.114	0.265	0.716	0.215	-1.323	0.186	0.398	1.289
ETV7	0.262	0.298	0.879	0.379	1.299	0.387	0.774	0.439	0.725	2.328
ETV6	0.127	0.295	0.43	0.667	1.136	0.336	0.404	0.686	0.636	2.026
ETV5	-0.318	0.3	-1.061	0.289	0.728	0.218	-1.248	0.212	0.405	1.309
ETV4	0.073	0.295	0.248	0.804	1.076	0.318	0.239	0.811	0.603	1.92
ETV3	0.673	0.31	2.172	0.03	1.96	0.607	1.581	0.114	1.068	3.598
ETV2	0.218	0.296	0.737	0.461	1.244	0.368	0.662	0.508	0.696	2.222
ETV1	-0.26	0.298	-0.873	0.383	0.771	0.23	-0.997	0.319	0.43	1.382
ETS2	-0.463	0.3	-1.54	0.123	0.63	0.189	-1.959	0.05	0.349	1.134
ETS1	-0.23	0.296	-0.776	0.438	0.795	0.236	-0.872	0.383	0.444	1.42
ETNPPL	0.085	0.295	0.288	0.774	1.089	0.322	0.276	0.783	0.61	1.942
ETNK2	0.056	0.295	0.19	0.85	1.058	0.312	0.184	0.854	0.593	1.887
ETNK1	-0.135	0.296	-0.454	0.65	0.874	0.259	-0.486	0.627	0.489	1.562
ETHE1	-0.39	0.3	-1.3	0.194	0.677	0.203	-1.59	0.112	0.376	1.219
ETFDH	-0.228	0.298	-0.767	0.443	0.796	0.237	-0.862	0.389	0.444	1.426
ETFB	0.56	0.302	1.853	0.064	1.751	0.53	1.419	0.156	0.968	3.168
ETFA	0.645	0.305	2.116	0.034	1.907	0.582	1.559	0.119	1.049	3.467
ETF1	-0.125	0.295	-0.424	0.672	0.882	0.261	-0.451	0.652	0.495	1.574
ETAA1	0.247	0.298	0.829	0.407	1.28	0.381	0.734	0.463	0.714	2.294
ESYT1	-0.021	0.295	-0.071	0.944	0.979	0.289	-0.072	0.943	0.549	1.746
ESRRG	0.497	0.303	1.641	0.101	1.643	0.497	1.294	0.196	0.908	2.974
ESRRB	-0.608	0.306	-1.985	0.047	0.544	0.167	-2.733	0.006	0.298	0.992
ESRRA	0.452	0.302	1.496	0.135	1.572	0.475	1.203	0.229	0.869	2.844

ESRP1	0.302	0.298	1.015	0.31	1.353	0.403	0.876	0.381	0.755	2.424
ESR2	-0.131	0.297	-0.439	0.661	0.878	0.261	-0.469	0.639	0.49	1.572
ESR1	-0.315	0.3	-1.05	0.294	0.73	0.219	-1.233	0.217	0.406	1.314
ESPL1	0.865	0.321	2.698	0.007	2.376	0.762	1.805	0.071	1.267	4.456
ESM1	0.268	0.296	0.903	0.367	1.307	0.387	0.792	0.428	0.731	2.336
ESF1	0.045	0.295	0.152	0.879	1.046	0.309	0.149	0.882	0.586	1.866
ESD	-0.225	0.298	-0.755	0.45	0.799	0.238	-0.847	0.397	0.446	1.431
ERVMER34-1	0.218	0.296	0.737	0.461	1.244	0.368	0.662	0.508	0.696	2.223
ERVK3-2	-0.232	0.298	-0.781	0.435	0.793	0.236	-0.879	0.38	0.442	1.42
ERVH-6	0.058	0.295	0.197	0.844	1.06	0.313	0.192	0.848	0.594	1.891
ERVH-4	-0.116	0.295	-0.394	0.693	0.89	0.263	-0.418	0.676	0.499	1.588
ERV9-1	0.65	0.31	2.099	0.036	1.916	0.594	1.543	0.123	1.044	3.517
ERV3-2	-0.306	0.298	-1.027	0.304	0.737	0.219	-1.202	0.229	0.411	1.32
ERP44	0.387	0.3	1.289	0.197	1.472	0.442	1.069	0.285	0.818	2.651
ERP29	-0.323	0.3	-1.077	0.282	0.724	0.217	-1.271	0.204	0.402	1.304
ERO1B	-0.102	0.295	-0.346	0.73	0.903	0.267	-0.364	0.716	0.506	1.611
ERO1A	-0.269	0.3	-0.894	0.371	0.764	0.23	-1.026	0.305	0.424	1.377
ERN2	0.001	0.295	0.005	0.996	1.001	0.295	0.005	0.996	0.562	1.786
ERN1	0.331	0.298	1.111	0.267	1.392	0.415	0.946	0.344	0.776	2.497
ERMP1	-0.34	0.3	-1.134	0.257	0.712	0.213	-1.351	0.177	0.396	1.281
ERMAP	-0.282	0.3	-0.939	0.348	0.754	0.226	-1.085	0.278	0.419	1.358
ERLIN2	-0.131	0.296	-0.441	0.659	0.877	0.26	-0.472	0.637	0.491	1.568
ERLIN1	-0.135	0.298	-0.452	0.651	0.874	0.26	-0.484	0.628	0.488	1.567
ERICH1	-0.277	0.298	-0.928	0.353	0.758	0.226	-1.069	0.285	0.423	1.36
ERI3	0.3	0.298	1.006	0.315	1.349	0.402	0.869	0.385	0.753	2.419
ERI2	0.535	0.302	1.769	0.077	1.707	0.516	1.37	0.171	0.944	3.087
ERH	0.112	0.298	0.374	0.708	1.118	0.333	0.354	0.723	0.623	2.005
ERGIC3	-0.886	0.312	-2.835	0.005	0.412	0.129	-4.56	0	0.224	0.761
ERGIC2	0.873	0.311	2.802	0.005	2.393	0.745	1.869	0.062	1.3	4.406
ERG28	-0.086	0.297	-0.289	0.773	0.918	0.272	-0.301	0.763	0.513	1.642
ERG	-0.345	0.298	-1.158	0.247	0.708	0.211	-1.382	0.167	0.395	1.27
ERF	0.168	0.295	0.568	0.57	1.183	0.349	0.523	0.601	0.663	2.11
EREG	0.088	0.295	0.297	0.767	1.092	0.322	0.284	0.776	0.612	1.947
ERCC8	0.13	0.295	0.44	0.66	1.139	0.336	0.412	0.68	0.639	2.03
ERCC6L	0.89	0.315	2.825	0.005	2.435	0.767	1.871	0.061	1.313	4.516
ERCC6	-0.089	0.296	-0.3	0.764	0.915	0.271	-0.313	0.754	0.512	1.635
ERCC5	0.352	0.302	1.166	0.244	1.423	0.43	0.983	0.326	0.787	2.573
ERCC4	0.639	0.306	2.09	0.037	1.895	0.58	1.544	0.122	1.041	3.451

ERCC3	0.087	0.297	0.292	0.77	1.091	0.324	0.28	0.78	0.609	1.952
ERCC2	-0.482	0.306	-1.574	0.115	0.617	0.189	-2.023	0.043	0.339	1.125
ERCC1	-0.72	0.315	-2.286	0.022	0.487	0.153	-3.349	0.001	0.262	0.902
ERC2-IT1	0.451	0.3	1.505	0.132	1.57	0.471	1.211	0.226	0.873	2.825
ERC2	-0.047	0.296	-0.158	0.875	0.954	0.283	-0.162	0.872	0.534	1.706
ERC1	0.628	0.306	2.052	0.04	1.873	0.573	1.524	0.128	1.028	3.413
ERBIN	-0.192	0.301	-0.638	0.524	0.825	0.249	-0.703	0.482	0.457	1.489
ERBB4	-0.719	0.315	-2.282	0.022	0.487	0.153	-3.34	0.001	0.263	0.903
ERBB3	-0.588	0.303	-1.945	0.052	0.555	0.168	-2.648	0.008	0.307	1.005
ERBB2	-0.215	0.296	-0.725	0.469	0.807	0.239	-0.809	0.419	0.451	1.442
ERAP2	-0.655	0.306	-2.142	0.032	0.52	0.159	-3.025	0.002	0.285	0.946
ERAP1	-0.014	0.296	-0.049	0.961	0.986	0.292	-0.049	0.961	0.552	1.761
ERAL1	0.08	0.297	0.27	0.787	1.083	0.322	0.259	0.796	0.605	1.939
EPYC	-0.71	0.31	-2.288	0.022	0.492	0.153	-3.332	0.001	0.268	0.903
EPX	-0.128	0.296	-0.434	0.664	0.879	0.26	-0.464	0.643	0.493	1.57
EPS8L3	-0.254	0.296	-0.858	0.391	0.775	0.23	-0.978	0.328	0.434	1.386
EPS8L2	0.06	0.295	0.203	0.839	1.062	0.313	0.197	0.844	0.595	1.893
EPS8L1	-0.104	0.295	-0.352	0.725	0.901	0.266	-0.371	0.711	0.505	1.608
EPS8	0.107	0.295	0.363	0.717	1.113	0.329	0.344	0.731	0.624	1.985
EPS15L1	0.783	0.31	2.524	0.012	2.188	0.679	1.75	0.08	1.191	4.018
EPS15	-0.24	0.298	-0.807	0.42	0.786	0.234	-0.912	0.362	0.439	1.409
EPRS1	0.122	0.296	0.413	0.68	1.13	0.334	0.389	0.698	0.633	2.018
EPPK1	0.04	0.299	0.133	0.894	1.041	0.311	0.131	0.896	0.579	1.871
EPOR	-0.543	0.302	-1.795	0.073	0.581	0.176	-2.383	0.017	0.321	1.051
EPO	0.199	0.296	0.673	0.501	1.22	0.361	0.611	0.542	0.683	2.18
EPN3	0.504	0.301	1.677	0.093	1.656	0.498	1.317	0.188	0.918	2.985
EPN1	0.408	0.3	1.359	0.174	1.504	0.451	1.116	0.264	0.835	2.708
EPM2AIP1	-0.086	0.296	-0.289	0.773	0.918	0.272	-0.302	0.763	0.514	1.641
EPM2A	0.038	0.295	0.13	0.897	1.039	0.307	0.127	0.899	0.583	1.853
EPHX3	0.291	0.297	0.983	0.326	1.338	0.397	0.852	0.394	0.748	2.394
EPHX2	-0.815	0.321	-2.541	0.011	0.442	0.142	-3.927	0	0.236	0.83
EPHX1	-0.21	0.298	-0.704	0.481	0.811	0.242	-0.783	0.433	0.452	1.454
EPHB6	-0.421	0.298	-1.409	0.159	0.657	0.196	-1.752	0.08	0.366	1.179
EPHB4	0.128	0.295	0.434	0.664	1.137	0.336	0.408	0.684	0.637	2.028
EPHB3	0.699	0.31	2.255	0.024	2.013	0.624	1.622	0.105	1.096	3.696
EPHB2	0.125	0.296	0.424	0.672	1.133	0.335	0.398	0.69	0.635	2.023
EPHB1	0.077	0.295	0.261	0.794	1.08	0.319	0.251	0.802	0.605	1.927
EPHA7	-0.087	0.296	-0.294	0.769	0.917	0.271	-0.307	0.759	0.514	1.636

EPHA5	0.429	0.306	1.399	0.162	1.535	0.47	1.138	0.255	0.842	2.799
EPHA4	0.105	0.297	0.353	0.724	1.11	0.329	0.335	0.738	0.621	1.985
EPHA3	-0.736	0.31	-2.376	0.018	0.479	0.148	-3.511	0	0.261	0.879
EPHA2	-0.227	0.298	-0.76	0.447	0.797	0.238	-0.853	0.394	0.445	1.43
EPHA1	-0.616	0.303	-2.034	0.042	0.54	0.164	-2.812	0.005	0.299	0.978
EPCAM	0.918	0.321	2.861	0.004	2.504	0.804	1.872	0.061	1.335	4.697
EPB42	0.316	0.3	1.055	0.291	1.372	0.411	0.905	0.366	0.762	2.468
EPB41L5	-0.158	0.296	-0.533	0.594	0.854	0.253	-0.578	0.563	0.478	1.526
EPB41L4B	-0.409	0.3	-1.365	0.172	0.664	0.199	-1.686	0.092	0.369	1.195
EPB41L4A-DT	-0.106	0.296	-0.359	0.719	0.899	0.266	-0.379	0.705	0.504	1.605
EPB41L4A	-0.188	0.298	-0.632	0.528	0.828	0.247	-0.695	0.487	0.462	1.486
EPB41L3	-0.668	0.31	-2.154	0.031	0.513	0.159	-3.064	0.002	0.279	0.942
EPB41L2	-0.882	0.315	-2.799	0.005	0.414	0.13	-4.494	0	0.223	0.768
EPB41L1	0.204	0.296	0.687	0.492	1.226	0.363	0.622	0.534	0.686	2.19
EPB41	0.191	0.296	0.645	0.519	1.21	0.359	0.587	0.557	0.677	2.164
EPAS1	-0.569	0.301	-1.895	0.058	0.566	0.17	-2.553	0.011	0.314	1.02
EP400	-1.037	0.323	-3.209	0.001	0.355	0.115	-5.633	0	0.188	0.668
EP300	-0.626	0.306	-2.047	0.041	0.535	0.163	-2.845	0.004	0.294	0.974
EOLA2	0.108	0.295	0.364	0.716	1.114	0.329	0.345	0.73	0.624	1.987
EOLA1	0.029	0.295	0.097	0.923	1.029	0.304	0.095	0.924	0.577	1.836
EOGT	0.019	0.297	0.063	0.949	1.019	0.302	0.063	0.95	0.57	1.823
ENY2	1.115	0.328	3.402	0.001	3.048	0.999	2.051	0.04	1.604	5.793
ENTR1	0.586	0.303	1.938	0.053	1.798	0.544	1.466	0.143	0.993	3.253
ENTPD7	-0.147	0.296	-0.497	0.619	0.863	0.256	-0.535	0.592	0.483	1.542
ENTPD6	0.354	0.298	1.19	0.234	1.425	0.424	1.002	0.316	0.795	2.555
ENTPD5	-0.216	0.296	-0.731	0.465	0.805	0.238	-0.816	0.415	0.451	1.439
ENTPD4	-0.559	0.306	-1.828	0.068	0.572	0.175	-2.448	0.014	0.314	1.041
ENTPD3	-0.134	0.296	-0.451	0.652	0.875	0.259	-0.483	0.629	0.489	1.564
ENTPD2	0.113	0.296	0.382	0.703	1.12	0.332	0.361	0.718	0.626	2.002
ENTPD1-AS1	0.387	0.298	1.297	0.195	1.472	0.439	1.076	0.282	0.821	2.64
ENTPD1	-0.704	0.306	-2.301	0.021	0.495	0.151	-3.34	0.001	0.272	0.901
ENSA	-0.208	0.298	-0.698	0.485	0.812	0.242	-0.776	0.438	0.453	1.456
ENPP4	-0.043	0.295	-0.146	0.884	0.958	0.283	-0.149	0.881	0.537	1.709
ENPP3	0.164	0.297	0.554	0.58	1.179	0.35	0.51	0.61	0.659	2.109
ENPP2	-0.639	0.304	-2.103	0.035	0.528	0.16	-2.945	0.003	0.291	0.957
ENPP1	0.075	0.296	0.255	0.799	1.078	0.319	0.245	0.806	0.604	1.926
ENPEP	0.182	0.295	0.615	0.539	1.199	0.354	0.562	0.574	0.672	2.14
ENOX2	-0.024	0.295	-0.081	0.936	0.976	0.288	-0.082	0.935	0.548	1.741

ENOX1	0.049	0.295	0.165	0.869	1.05	0.31	0.161	0.872	0.589	1.872
ENOSF1	0.02	0.296	0.068	0.945	1.02	0.302	0.068	0.946	0.572	1.822
ENOPH1	0.296	0.298	0.996	0.319	1.345	0.4	0.862	0.389	0.751	2.41
ENO3	0.222	0.296	0.751	0.453	1.249	0.369	0.674	0.501	0.699	2.229
ENO2	0.022	0.297	0.074	0.941	1.022	0.303	0.073	0.942	0.572	1.828
ENO1	0.097	0.297	0.326	0.744	1.102	0.328	0.311	0.756	0.615	1.974
ENGASE	0.143	0.299	0.478	0.633	1.154	0.345	0.446	0.656	0.642	2.073
ENG	-0.296	0.296	-0.998	0.318	0.744	0.22	-1.162	0.245	0.416	1.33
ENDOU	-0.511	0.303	-1.688	0.091	0.6	0.182	-2.203	0.028	0.332	1.086
ENDOG	0.436	0.298	1.463	0.143	1.547	0.461	1.186	0.236	0.862	2.776
ENDOD1	-0.361	0.298	-1.209	0.227	0.697	0.208	-1.456	0.145	0.389	1.251
ENC1	0.426	0.302	1.41	0.159	1.531	0.463	1.148	0.251	0.847	2.769
ENAH	-0.339	0.298	-1.138	0.255	0.713	0.212	-1.355	0.176	0.397	1.277
EN2	0.072	0.295	0.245	0.806	1.075	0.317	0.237	0.813	0.603	1.917
EN1	0.01	0.295	0.032	0.974	1.01	0.298	0.032	0.974	0.566	1.801
EMX2	-0.347	0.298	-1.166	0.244	0.707	0.21	-1.394	0.163	0.394	1.267
EMX1	0.033	0.295	0.112	0.911	1.033	0.305	0.11	0.913	0.58	1.843
EMSY	0.637	0.304	2.097	0.036	1.891	0.575	1.551	0.121	1.043	3.43
EMP3	-0.407	0.3	-1.358	0.174	0.666	0.199	-1.676	0.094	0.37	1.198
EMP2	0.193	0.296	0.652	0.514	1.213	0.36	0.593	0.553	0.679	2.169
EMP1	-0.093	0.295	-0.316	0.752	0.911	0.269	-0.331	0.741	0.511	1.625
EML4	-0.773	0.309	-2.503	0.012	0.461	0.143	-3.777	0	0.252	0.846
EML3	-0.391	0.3	-1.306	0.192	0.676	0.203	-1.599	0.11	0.376	1.216
EML2	0.029	0.295	0.097	0.922	1.029	0.304	0.096	0.924	0.577	1.835
EML1	-0.602	0.304	-1.98	0.048	0.548	0.166	-2.716	0.007	0.302	0.994
EMILIN2	0.195	0.299	0.653	0.514	1.215	0.363	0.593	0.553	0.677	2.183
EMILIN1	-0.54	0.301	-1.795	0.073	0.583	0.175	-2.381	0.017	0.323	1.051
EMID1	0.895	0.315	2.841	0.004	2.448	0.771	1.877	0.06	1.32	4.539
EMG1	0.261	0.298	0.878	0.38	1.299	0.387	0.772	0.44	0.725	2.327
EMD	0.649	0.303	2.141	0.032	1.914	0.58	1.575	0.115	1.056	3.467
EMCN	-0.558	0.302	-1.845	0.065	0.572	0.173	-2.47	0.014	0.316	1.036
EMC9	0.869	0.31	2.803	0.005	2.386	0.74	1.872	0.061	1.299	4.382
EMC8	0.977	0.315	3.099	0.002	2.657	0.838	1.978	0.048	1.432	4.93
EMC7	-0.142	0.297	-0.478	0.633	0.868	0.258	-0.513	0.608	0.485	1.553
EMC6	1.047	0.317	3.304	0.001	2.849	0.903	2.048	0.041	1.531	5.302
EMC3	0.001	0.295	0.002	0.998	1.001	0.296	0.002	0.998	0.561	1.785
EMC2	0.489	0.302	1.619	0.106	1.631	0.493	1.28	0.201	0.902	2.951
EMC10	0.12	0.296	0.406	0.685	1.128	0.334	0.382	0.702	0.631	2.014

EMC1	0.049	0.296	0.165	0.869	1.05	0.31	0.161	0.872	0.588	1.875
ELSPBP1	-0.043	0.296	-0.144	0.886	0.958	0.283	-0.147	0.883	0.537	1.71
ELP6	0.299	0.298	1.006	0.315	1.349	0.401	0.869	0.385	0.753	2.417
ELP5	-0.67	0.31	-2.163	0.031	0.512	0.159	-3.08	0.002	0.279	0.939
ELP4	0.374	0.298	1.257	0.209	1.454	0.433	1.048	0.294	0.811	2.605
ELP3	-0.409	0.303	-1.352	0.176	0.664	0.201	-1.67	0.095	0.367	1.202
ELP1	0.154	0.295	0.52	0.603	1.166	0.345	0.482	0.63	0.653	2.081
ELOVL6	0.481	0.303	1.59	0.112	1.618	0.489	1.262	0.207	0.894	2.927
ELOVL5	-0.501	0.306	-1.636	0.102	0.606	0.185	-2.124	0.034	0.333	1.104
ELOVL4	-0.3	0.3	-0.999	0.318	0.741	0.222	-1.165	0.244	0.412	1.334
ELOVL2	-0.144	0.296	-0.486	0.627	0.866	0.257	-0.523	0.601	0.484	1.548
ELOC	0.626	0.306	2.049	0.04	1.87	0.572	1.523	0.128	1.027	3.404
ELOB	0.371	0.298	1.243	0.214	1.449	0.432	1.039	0.299	0.807	2.601
ELOA2	0.555	0.306	1.818	0.069	1.743	0.532	1.395	0.163	0.958	3.172
ELOA-AS1	-0.32	0.3	-1.066	0.286	0.726	0.218	-1.256	0.209	0.403	1.308
ELOA	-0.287	0.298	-0.964	0.335	0.751	0.223	-1.117	0.264	0.419	1.345
ELN	-0.723	0.31	-2.331	0.02	0.485	0.15	-3.419	0.001	0.264	0.891
ELMO3	0.616	0.303	2.031	0.042	1.851	0.561	1.516	0.129	1.022	3.354
ELMO2	0.231	0.298	0.774	0.439	1.259	0.375	0.691	0.489	0.702	2.258
ELMO1	-0.506	0.3	-1.687	0.092	0.603	0.181	-2.196	0.028	0.335	1.085
ELL3	0.382	0.297	1.287	0.198	1.465	0.434	1.07	0.285	0.819	2.62
ELL2	-0.147	0.296	-0.496	0.62	0.863	0.256	-0.534	0.593	0.483	1.543
ELL	-0.468	0.302	-1.549	0.121	0.626	0.189	-1.976	0.048	0.346	1.132
ELK4	0.071	0.297	0.239	0.811	1.073	0.318	0.231	0.818	0.6	1.92
ELK3	-0.81	0.311	-2.61	0.009	0.445	0.138	-4.022	0	0.242	0.817
ELK2AP	-0.08	0.296	-0.272	0.786	0.923	0.273	-0.283	0.777	0.517	1.647
ELK1	0.251	0.296	0.848	0.397	1.286	0.381	0.75	0.453	0.719	2.297
ELF5	0.064	0.296	0.217	0.828	1.066	0.315	0.21	0.834	0.597	1.903
ELF4	0.229	0.297	0.773	0.44	1.258	0.373	0.691	0.49	0.703	2.25
ELF3	0.136	0.295	0.462	0.644	1.146	0.339	0.432	0.666	0.642	2.045
ELF2	0.245	0.296	0.826	0.409	1.277	0.378	0.732	0.464	0.715	2.282
ELF1	-0.3	0.298	-1.005	0.315	0.741	0.221	-1.172	0.241	0.413	1.329
ELAVL4	0.027	0.295	0.09	0.928	1.027	0.303	0.089	0.929	0.576	1.831
ELAVL3	0.732	0.307	2.381	0.017	2.079	0.639	1.689	0.091	1.138	3.796
ELAVL2	0.271	0.298	0.911	0.362	1.312	0.39	0.798	0.425	0.732	2.35
ELAVL1	0.939	0.321	2.927	0.003	2.557	0.82	1.899	0.058	1.364	4.794
ELAPOR1	-0.621	0.31	-2.003	0.045	0.537	0.167	-2.776	0.006	0.293	0.987
ELANE	0.635	0.306	2.079	0.038	1.887	0.577	1.539	0.124	1.037	3.436

ELAC2	-0.079	0.295	-0.267	0.789	0.924	0.273	-0.278	0.781	0.518	1.649
ELAC1	-0.139	0.296	-0.469	0.639	0.87	0.258	-0.503	0.615	0.487	1.556
EIPR1	0.248	0.299	0.83	0.406	1.281	0.382	0.735	0.462	0.714	2.3
EIF6	0.238	0.296	0.803	0.422	1.268	0.376	0.715	0.475	0.71	2.266
EIF5B	0.05	0.297	0.167	0.867	1.051	0.312	0.163	0.87	0.587	1.881
EIF5A2	0.378	0.3	1.262	0.207	1.46	0.437	1.051	0.293	0.811	2.625
EIF5A	0.028	0.296	0.095	0.924	1.029	0.304	0.094	0.925	0.576	1.836
EIF5	0.69	0.306	2.253	0.024	1.994	0.611	1.627	0.104	1.094	3.636
EIF4H	-0.128	0.299	-0.429	0.668	0.88	0.263	-0.458	0.647	0.49	1.58
EIF4G3	-0.338	0.3	-1.126	0.26	0.713	0.214	-1.339	0.181	0.396	1.284
EIF4G2	0.004	0.297	0.014	0.989	1.004	0.298	0.014	0.989	0.561	1.796
EIF4G1	0.336	0.3	1.12	0.263	1.399	0.419	0.951	0.342	0.777	2.518
EIF4ENIF1	-0.186	0.298	-0.624	0.532	0.83	0.247	-0.686	0.493	0.463	1.488
EIF4EBP2	-0.139	0.296	-0.47	0.639	0.87	0.258	-0.504	0.614	0.487	1.554
EIF4EBP1	0.25	0.296	0.847	0.397	1.284	0.38	0.749	0.454	0.72	2.293
EIF4E2	0.626	0.303	2.065	0.039	1.87	0.567	1.535	0.125	1.032	3.389
EIF4E	0.472	0.3	1.576	0.115	1.604	0.481	1.256	0.209	0.891	2.886
EIF4B	-0.738	0.31	-2.378	0.017	0.478	0.148	-3.518	0	0.26	0.878
EIF4A3	0.502	0.3	1.674	0.094	1.653	0.496	1.316	0.188	0.918	2.976
EIF3M	-0.05	0.298	-0.167	0.868	0.952	0.283	-0.171	0.864	0.531	1.706
EIF3L	-0.596	0.303	-1.97	0.049	0.551	0.167	-2.695	0.007	0.304	0.997
EIF3K	-0.447	0.302	-1.479	0.139	0.639	0.193	-1.866	0.062	0.354	1.156
EIF3J	0.112	0.296	0.378	0.705	1.118	0.331	0.358	0.72	0.626	1.997
EIF3I	-0.109	0.296	-0.367	0.714	0.897	0.266	-0.388	0.698	0.502	1.603
EIF3H	-0.084	0.296	-0.283	0.777	0.92	0.272	-0.295	0.768	0.515	1.643
EIF3G	-0.287	0.298	-0.964	0.335	0.751	0.223	-1.117	0.264	0.419	1.345
EIF3F	0.168	0.296	0.567	0.571	1.183	0.351	0.522	0.602	0.662	2.114
EIF3E	0.171	0.296	0.576	0.565	1.186	0.351	0.529	0.596	0.664	2.119
EIF3D	-0.429	0.3	-1.429	0.153	0.651	0.195	-1.784	0.074	0.362	1.172
EIF3B	0.307	0.301	1.02	0.308	1.359	0.409	0.878	0.38	0.754	2.45
EIF3A	-0.268	0.297	-0.902	0.367	0.765	0.227	-1.035	0.301	0.427	1.369
EIF2S3	0.021	0.295	0.071	0.944	1.021	0.301	0.07	0.944	0.573	1.82
EIF2S2	0.215	0.296	0.727	0.468	1.24	0.367	0.654	0.513	0.694	2.217
EIF2S1	0.597	0.303	1.973	0.049	1.817	0.55	1.485	0.137	1.004	3.288
EIF2D	0.311	0.298	1.045	0.296	1.365	0.407	0.898	0.369	0.762	2.447
EIF2B5	0.725	0.31	2.34	0.019	2.064	0.639	1.665	0.096	1.125	3.789
EIF2B4	-0.542	0.306	-1.775	0.076	0.581	0.178	-2.356	0.018	0.319	1.058
EIF2B3	-0.399	0.302	-1.319	0.187	0.671	0.203	-1.621	0.105	0.371	1.214

EIF2B2	0.244	0.296	0.823	0.411	1.276	0.378	0.73	0.465	0.714	2.281
EIF2B1	-0.342	0.3	-1.141	0.254	0.71	0.213	-1.361	0.174	0.395	1.278
EIF2AK3	-0.469	0.3	-1.565	0.118	0.626	0.188	-1.996	0.046	0.348	1.126
EIF2AK2	-0.197	0.296	-0.664	0.507	0.822	0.243	-0.733	0.463	0.46	1.468
EIF2AK1	-0.234	0.298	-0.786	0.432	0.792	0.236	-0.885	0.376	0.442	1.418
EIF1B	0.275	0.297	0.926	0.355	1.317	0.392	0.809	0.418	0.735	2.359
EIF1AY	0.135	0.296	0.457	0.648	1.145	0.338	0.427	0.669	0.641	2.043
EIF1AX	0.392	0.301	1.305	0.192	1.481	0.445	1.079	0.281	0.821	2.67
EIF1	0.591	0.303	1.953	0.051	1.806	0.547	1.474	0.14	0.998	3.27
EID1	-0.338	0.3	-1.126	0.26	0.713	0.214	-1.34	0.18	0.396	1.284
EI24	0.398	0.3	1.324	0.186	1.488	0.447	1.092	0.275	0.826	2.682
EHMT2	-0.444	0.3	-1.48	0.139	0.641	0.192	-1.863	0.062	0.356	1.155
EHMT1	-0.032	0.296	-0.108	0.914	0.969	0.287	-0.11	0.913	0.542	1.731
EHHADH	-0.041	0.296	-0.139	0.889	0.96	0.284	-0.142	0.887	0.537	1.714
EHF	0.217	0.296	0.731	0.465	1.242	0.368	0.657	0.511	0.695	2.219
EHD4	-0.056	0.295	-0.19	0.849	0.945	0.279	-0.196	0.845	0.53	1.687
EHD3	-0.528	0.3	-1.759	0.079	0.59	0.177	-2.317	0.021	0.328	1.062
EHD2	-0.483	0.302	-1.598	0.11	0.617	0.186	-2.054	0.04	0.341	1.116
EHD1	0.299	0.298	1.003	0.316	1.348	0.402	0.867	0.386	0.752	2.418
EHBP1L1	-0.528	0.303	-1.745	0.081	0.59	0.179	-2.299	0.022	0.326	1.067
EHBP1	0.159	0.295	0.538	0.591	1.172	0.346	0.498	0.619	0.657	2.092
EGR4	0.034	0.295	0.114	0.909	1.034	0.305	0.112	0.91	0.58	1.844
EGR3	-0.286	0.3	-0.954	0.34	0.751	0.225	-1.104	0.27	0.418	1.352
EGR2	-0.099	0.295	-0.336	0.737	0.906	0.267	-0.353	0.724	0.508	1.616
EGR1	-0.058	0.295	-0.197	0.844	0.944	0.279	-0.202	0.84	0.529	1.683
EGOT	-0.27	0.3	-0.898	0.369	0.764	0.229	-1.031	0.303	0.424	1.375
EGLN3	-0.142	0.297	-0.477	0.634	0.868	0.258	-0.512	0.609	0.485	1.554
EGLN1	0.264	0.298	0.884	0.377	1.302	0.388	0.777	0.437	0.725	2.336
EGFR	0.202	0.298	0.676	0.499	1.223	0.364	0.613	0.54	0.682	2.193
EGFL7	-0.061	0.295	-0.206	0.837	0.941	0.278	-0.212	0.832	0.528	1.679
EGFL6	-0.11	0.296	-0.37	0.711	0.896	0.266	-0.391	0.695	0.501	1.602
EGF	-0.278	0.298	-0.932	0.351	0.758	0.226	-1.075	0.283	0.423	1.358
EFS	0.07	0.295	0.238	0.812	1.073	0.317	0.229	0.819	0.601	1.913
EFR3B	0.183	0.298	0.615	0.538	1.201	0.358	0.562	0.574	0.67	2.154
EFR3A	0.098	0.297	0.33	0.741	1.103	0.328	0.314	0.753	0.616	1.975
EFNB3	-0.054	0.296	-0.183	0.855	0.947	0.28	-0.188	0.851	0.531	1.691
EFNB2	0.011	0.295	0.039	0.969	1.012	0.299	0.039	0.969	0.567	1.805
EFNB1	0.52	0.306	1.701	0.089	1.682	0.514	1.327	0.185	0.924	3.063

EFNA5	0.194	0.298	0.652	0.514	1.214	0.361	0.593	0.553	0.678	2.176
EFNA4	-0.077	0.295	-0.259	0.795	0.926	0.273	-0.269	0.788	0.519	1.652
EFNA3	-0.154	0.296	-0.519	0.604	0.857	0.254	-0.561	0.575	0.48	1.533
EFNA2	0.54	0.303	1.782	0.075	1.715	0.519	1.377	0.168	0.948	3.105
EFNA1	0.208	0.297	0.7	0.484	1.231	0.365	0.632	0.527	0.688	2.201
EFL1	-0.052	0.297	-0.176	0.861	0.949	0.282	-0.18	0.857	0.53	1.7
EFHD2	-0.546	0.303	-1.803	0.071	0.579	0.175	-2.398	0.016	0.32	1.049
EFHD1	-0.315	0.3	-1.052	0.293	0.73	0.219	-1.237	0.216	0.406	1.312
EFHC2	0.318	0.298	1.068	0.286	1.374	0.409	0.915	0.36	0.767	2.462
EFHC1	-0.113	0.295	-0.383	0.701	0.893	0.264	-0.406	0.685	0.5	1.593
EFEMP2	-0.321	0.298	-1.08	0.28	0.725	0.216	-1.273	0.203	0.405	1.3
EFEMP1	-0.187	0.296	-0.63	0.529	0.83	0.246	-0.693	0.489	0.464	1.483
EFCC1	-0.774	0.31	-2.498	0.012	0.461	0.143	-3.771	0	0.251	0.846
EFCAB6	-0.523	0.306	-1.711	0.087	0.593	0.181	-2.249	0.025	0.325	1.079
EFCAB2	0.057	0.297	0.191	0.849	1.058	0.314	0.185	0.853	0.592	1.893
EFCAB14	-0.467	0.302	-1.547	0.122	0.627	0.189	-1.972	0.049	0.347	1.133
EFCAB11	0.23	0.296	0.778	0.436	1.259	0.372	0.695	0.487	0.705	2.248
EFCAB1	0.338	0.298	1.136	0.256	1.403	0.418	0.964	0.335	0.782	2.515
EEF2KMT	-0.134	0.297	-0.45	0.652	0.875	0.259	-0.482	0.63	0.489	1.565
EEF2	-0.502	0.3	-1.671	0.095	0.605	0.182	-2.17	0.03	0.336	1.091
EEF1E1	0.874	0.311	2.811	0.005	2.396	0.745	1.874	0.061	1.303	4.408
EEF1D	0.033	0.295	0.113	0.91	1.034	0.305	0.111	0.911	0.58	1.844
EEF1AKNMT	0.738	0.31	2.378	0.017	2.091	0.649	1.682	0.093	1.138	3.841
EEF1AKMT3	-0.787	0.315	-2.501	0.012	0.455	0.143	-3.804	0	0.246	0.843
EEF1AKMT2	-0.127	0.299	-0.426	0.67	0.88	0.263	-0.455	0.649	0.49	1.581
EEF1A2	0.271	0.296	0.915	0.36	1.311	0.389	0.801	0.423	0.734	2.344
EEF1A1	-0.265	0.299	-0.889	0.374	0.767	0.229	-1.018	0.309	0.427	1.377
EED	0.342	0.298	1.15	0.25	1.408	0.419	0.974	0.33	0.786	2.524
EEA1	0.574	0.302	1.899	0.058	1.776	0.537	1.445	0.149	0.982	3.212
EDRF1	0.431	0.3	1.438	0.15	1.539	0.461	1.168	0.243	0.855	2.767
EDNRB	-0.137	0.295	-0.464	0.642	0.872	0.258	-0.498	0.619	0.489	1.555
EDNRA	-0.621	0.306	-2.03	0.042	0.537	0.164	-2.815	0.005	0.295	0.979
EDN3	0.147	0.295	0.497	0.619	1.158	0.342	0.463	0.644	0.649	2.067
EDN2	-0.526	0.306	-1.717	0.086	0.591	0.181	-2.258	0.024	0.324	1.077
EDN1	-0.071	0.296	-0.239	0.811	0.932	0.275	-0.248	0.804	0.522	1.663
EDIL3	-0.013	0.296	-0.043	0.965	0.987	0.292	-0.044	0.965	0.553	1.763
EDF1	-0.583	0.306	-1.904	0.057	0.558	0.171	-2.585	0.01	0.307	1.017
EDEM3	-0.093	0.295	-0.316	0.752	0.911	0.269	-0.331	0.741	0.51	1.625

EDEM2	0.03	0.295	0.1	0.92	1.03	0.304	0.099	0.921	0.577	1.837
EDEM1	-0.295	0.297	-0.993	0.321	0.745	0.221	-1.155	0.248	0.416	1.333
EDDM3B	0.353	0.298	1.186	0.236	1.424	0.424	0.999	0.318	0.794	2.553
EDDM3A	-0.214	0.296	-0.722	0.47	0.807	0.239	-0.806	0.42	0.452	1.443
EDC4	0.274	0.298	0.918	0.358	1.315	0.392	0.803	0.422	0.733	2.358
EDC3	-0.339	0.303	-1.12	0.263	0.712	0.216	-1.334	0.182	0.393	1.29
EDAR	-0.116	0.296	-0.392	0.695	0.89	0.264	-0.416	0.678	0.498	1.591
EDA2R	-0.443	0.3	-1.477	0.14	0.642	0.193	-1.858	0.063	0.357	1.156
EDA	0.631	0.31	2.036	0.042	1.88	0.583	1.51	0.131	1.024	3.452
ECT2	0.963	0.321	3.002	0.003	2.618	0.84	1.928	0.054	1.397	4.909
ECSIT	-0.493	0.306	-1.612	0.107	0.611	0.187	-2.084	0.037	0.335	1.112
ECRP	0.159	0.296	0.536	0.592	1.172	0.347	0.495	0.62	0.656	2.093
ECPAS	0.229	0.298	0.767	0.443	1.257	0.375	0.686	0.493	0.7	2.257
ECM2	-0.484	0.3	-1.613	0.107	0.616	0.185	-2.074	0.038	0.342	1.11
ECM1	-0.312	0.3	-1.04	0.299	0.732	0.22	-1.22	0.222	0.407	1.318
ECI2	-0.493	0.306	-1.612	0.107	0.611	0.187	-2.084	0.037	0.335	1.112
ECI1	-0.253	0.3	-0.844	0.398	0.776	0.233	-0.961	0.337	0.431	1.398
ECHS1	0.312	0.296	1.053	0.293	1.366	0.405	0.904	0.366	0.764	2.443
ECHDC3	0.893	0.316	2.831	0.005	2.443	0.771	1.872	0.061	1.316	4.535
ECHDC2	-0.206	0.298	-0.692	0.489	0.814	0.242	-0.769	0.442	0.454	1.458
ECHDC1	-0.017	0.296	-0.056	0.955	0.984	0.291	-0.056	0.955	0.551	1.756
ECH1	0.394	0.3	1.314	0.189	1.482	0.444	1.086	0.277	0.824	2.667
ECEL1	0.136	0.295	0.46	0.645	1.146	0.339	0.431	0.667	0.642	2.045
ECE2	-0.281	0.298	-0.943	0.345	0.755	0.225	-1.089	0.276	0.421	1.353
ECE1	0.04	0.296	0.136	0.892	1.041	0.308	0.133	0.894	0.583	1.859
ECD	-0.273	0.3	-0.909	0.363	0.761	0.229	-1.046	0.296	0.422	1.371
EBP	0.397	0.298	1.331	0.183	1.487	0.443	1.098	0.272	0.829	2.667
EBLN2	0.121	0.296	0.408	0.683	1.129	0.335	0.384	0.701	0.631	2.018
EBI3	-0.412	0.304	-1.358	0.175	0.662	0.201	-1.68	0.093	0.365	1.201
EBF2	-0.304	0.297	-1.024	0.306	0.738	0.219	-1.197	0.231	0.412	1.32
EBAG9	0.444	0.3	1.482	0.138	1.559	0.467	1.196	0.232	0.866	2.805
EAPP	-0.279	0.302	-0.924	0.356	0.756	0.229	-1.065	0.287	0.418	1.368
EAF2	-0.323	0.298	-1.085	0.278	0.724	0.216	-1.28	0.201	0.404	1.298
E4F1	0.088	0.295	0.298	0.765	1.092	0.323	0.286	0.775	0.612	1.949
E2F8	0.598	0.306	1.958	0.05	1.819	0.556	1.473	0.141	0.999	3.311
E2F6	0.201	0.296	0.68	0.496	1.223	0.362	0.616	0.538	0.685	2.184
E2F5	0.516	0.303	1.705	0.088	1.675	0.507	1.332	0.183	0.926	3.032
E2F4	0.214	0.298	0.717	0.473	1.239	0.369	0.646	0.518	0.69	2.222

E2F3	1.108	0.336	3.296	0.001	3.028	1.018	1.992	0.046	1.567	5.852
E2F2	0.205	0.296	0.691	0.49	1.227	0.364	0.625	0.532	0.686	2.194
E2F1	0.539	0.307	1.758	0.079	1.715	0.526	1.359	0.174	0.94	3.129
DZIP3	-0.278	0.298	-0.932	0.351	0.758	0.226	-1.074	0.283	0.422	1.358
DZIP1	-0.439	0.3	-1.463	0.143	0.645	0.193	-1.836	0.066	0.358	1.161
DZANK1	0.011	0.295	0.036	0.971	1.011	0.298	0.036	0.971	0.567	1.803
DYSF	-0.32	0.299	-1.069	0.285	0.726	0.217	-1.26	0.208	0.404	1.305
DYRK4	0.253	0.297	0.852	0.394	1.288	0.382	0.753	0.452	0.72	2.304
DYRK3	0.557	0.301	1.853	0.064	1.746	0.525	1.421	0.155	0.968	3.147
DYRK2	0.616	0.306	2.014	0.044	1.852	0.566	1.504	0.133	1.017	3.372
DYRK1B	0.07	0.295	0.237	0.813	1.072	0.317	0.228	0.819	0.601	1.913
DYRK1A	0.1	0.296	0.338	0.735	1.105	0.327	0.322	0.748	0.619	1.974
DYNLT3	-0.202	0.301	-0.673	0.501	0.817	0.246	-0.746	0.456	0.453	1.473
DYNLT1	0.132	0.296	0.444	0.657	1.141	0.338	0.416	0.677	0.638	2.039
DYNLRB1	-0.083	0.295	-0.282	0.778	0.92	0.272	-0.294	0.769	0.516	1.642
DYNLL1	0.224	0.296	0.755	0.45	1.251	0.371	0.676	0.499	0.7	2.235
DYNC2LI1	0.229	0.297	0.771	0.441	1.257	0.374	0.689	0.491	0.702	2.251
DYNC2I1	0.044	0.297	0.15	0.881	1.045	0.31	0.146	0.884	0.584	1.87
DYNC2H1	-0.28	0.298	-0.941	0.347	0.756	0.225	-1.086	0.277	0.422	1.354
DYNC1LI2	0.579	0.303	1.912	0.056	1.785	0.541	1.451	0.147	0.986	3.232
DYNC1LI1	-0.203	0.303	-0.672	0.502	0.816	0.247	-0.745	0.456	0.451	1.476
DYNC1I2	-0.287	0.298	-0.965	0.335	0.75	0.224	-1.117	0.264	0.418	1.345
DYNC1I1	0.179	0.296	0.606	0.545	1.196	0.354	0.554	0.579	0.67	2.138
DYNC1H1	-0.175	0.296	-0.591	0.554	0.839	0.249	-0.646	0.518	0.47	1.5
DYM	0.17	0.295	0.576	0.565	1.185	0.35	0.53	0.596	0.664	2.115
DXO	0.176	0.296	0.595	0.552	1.193	0.353	0.545	0.585	0.667	2.131
DVL3	0.134	0.296	0.452	0.651	1.143	0.339	0.423	0.672	0.64	2.044
DVL2	-0.113	0.296	-0.383	0.702	0.893	0.264	-0.406	0.685	0.5	1.595
DVL1	-0.026	0.295	-0.088	0.93	0.974	0.288	-0.09	0.929	0.546	1.739
DUX1	-0.028	0.295	-0.095	0.924	0.972	0.287	-0.096	0.923	0.545	1.735
DUT	0.895	0.315	2.839	0.005	2.447	0.771	1.876	0.061	1.319	4.538
DUSP9	0.064	0.295	0.216	0.829	1.066	0.315	0.209	0.835	0.597	1.901
DUSP7	0.115	0.295	0.39	0.696	1.122	0.331	0.369	0.712	0.629	2.002
DUSP6	-0.078	0.295	-0.264	0.791	0.925	0.273	-0.275	0.783	0.519	1.65
DUSP5	-0.233	0.3	-0.776	0.437	0.792	0.238	-0.874	0.382	0.44	1.427
DUSP4	-0.295	0.3	-0.983	0.326	0.745	0.223	-1.143	0.253	0.414	1.341
DUSP3	-0.085	0.296	-0.287	0.774	0.919	0.272	-0.299	0.765	0.515	1.64
DUSP26	0.241	0.297	0.81	0.418	1.272	0.378	0.72	0.471	0.711	2.277

DUSP22	0.417	0.3	1.391	0.164	1.517	0.455	1.138	0.255	0.843	2.73
DUSP21	0.502	0.302	1.661	0.097	1.652	0.499	1.306	0.192	0.913	2.987
DUSP2	0.213	0.296	0.72	0.472	1.238	0.367	0.648	0.517	0.693	2.212
DUSP14	0.762	0.31	2.456	0.014	2.143	0.665	1.719	0.086	1.166	3.936
DUSP13	-0.148	0.296	-0.499	0.618	0.863	0.255	-0.538	0.591	0.483	1.541
DUSP12	-0.053	0.295	-0.178	0.858	0.949	0.28	-0.183	0.855	0.532	1.692
DUSP11	-0.122	0.296	-0.411	0.681	0.885	0.262	-0.437	0.662	0.495	1.583
DUSP10	-0.684	0.31	-2.206	0.027	0.504	0.156	-3.167	0.002	0.275	0.927
DUSP1	0.116	0.296	0.391	0.696	1.123	0.332	0.369	0.712	0.629	2.005
DUS4L	-0.065	0.295	-0.219	0.827	0.937	0.277	-0.226	0.821	0.526	1.671
DUS2	0.619	0.306	2.026	0.043	1.857	0.568	1.51	0.131	1.02	3.382
DUS1L	0.411	0.3	1.372	0.17	1.508	0.452	1.125	0.261	0.839	2.714
DUOX2	-0.309	0.298	-1.038	0.299	0.734	0.219	-1.216	0.224	0.41	1.316
DUOX1	-0.21	0.296	-0.709	0.478	0.811	0.24	-0.789	0.43	0.454	1.448
DTYMK	0.276	0.297	0.932	0.351	1.318	0.391	0.814	0.415	0.737	2.358
DTX4	-0.468	0.3	-1.561	0.119	0.626	0.188	-1.991	0.047	0.348	1.127
DTX3	-0.59	0.306	-1.931	0.053	0.554	0.169	-2.632	0.008	0.304	1.009
DTX2P1-UPK3BI	0.517	0.302	1.712	0.087	1.677	0.507	1.337	0.181	0.928	3.032
DTX2	0.183	0.296	0.618	0.537	1.201	0.356	0.565	0.572	0.672	2.146
DTWD1	-0.108	0.296	-0.364	0.716	0.898	0.265	-0.385	0.701	0.503	1.603
DTNB-AS1	-0.324	0.298	-1.089	0.276	0.723	0.215	-1.286	0.198	0.403	1.296
DTNB	-0.12	0.296	-0.406	0.685	0.887	0.263	-0.431	0.666	0.496	1.585
DTNA	0.279	0.298	0.939	0.348	1.322	0.394	0.819	0.413	0.738	2.37
DTL	0.702	0.31	2.265	0.023	2.018	0.626	1.628	0.104	1.099	3.705
DSTYK	0.24	0.298	0.805	0.421	1.271	0.378	0.716	0.474	0.709	2.277
DSTNP2	0.093	0.297	0.313	0.754	1.097	0.325	0.299	0.765	0.614	1.962
DSTN	-0.327	0.298	-1.099	0.272	0.721	0.215	-1.3	0.193	0.402	1.292
DST	-0.583	0.301	-1.937	0.053	0.558	0.168	-2.63	0.009	0.309	1.007
DSPP	0.496	0.3	1.654	0.098	1.643	0.493	1.304	0.192	0.912	2.959
DSP	0.287	0.301	0.954	0.34	1.332	0.4	0.829	0.407	0.739	2.4
DSN1	0.749	0.31	2.417	0.016	2.116	0.656	1.701	0.089	1.152	3.885
DSG3	-0.379	0.3	-1.264	0.206	0.685	0.205	-1.537	0.124	0.381	1.232
DSG2	-0.017	0.296	-0.059	0.953	0.983	0.291	-0.059	0.953	0.55	1.754
DSG1	0.237	0.296	0.799	0.424	1.267	0.376	0.711	0.477	0.709	2.265
DSE	-0.622	0.303	-2.053	0.04	0.537	0.163	-2.848	0.004	0.296	0.972
DSCR4	0.116	0.295	0.393	0.694	1.123	0.331	0.371	0.71	0.63	2.002
DSCC1	0.507	0.3	1.691	0.091	1.66	0.497	1.327	0.185	0.923	2.987
DSCAM	0.623	0.31	2.012	0.044	1.865	0.578	1.497	0.134	1.016	3.422

DSC3	-0.143	0.296	-0.483	0.629	0.867	0.257	-0.52	0.603	0.485	1.548
DSC2	0.146	0.297	0.494	0.621	1.158	0.344	0.459	0.646	0.647	2.071
DSC1	-0.319	0.298	-1.07	0.285	0.727	0.217	-1.26	0.208	0.405	1.304
DRP2	0.068	0.295	0.231	0.818	1.07	0.316	0.223	0.824	0.6	1.91
DROSHA	0.765	0.315	2.426	0.015	2.149	0.678	1.696	0.09	1.158	3.987
DRICH1	0.113	0.295	0.383	0.702	1.12	0.331	0.362	0.717	0.628	1.998
DRG2	-0.216	0.298	-0.727	0.467	0.805	0.24	-0.812	0.417	0.449	1.443
DRG1	0.214	0.296	0.722	0.47	1.238	0.367	0.65	0.516	0.693	2.213
DRD5	0.463	0.3	1.544	0.123	1.589	0.476	1.236	0.216	0.883	2.859
DRD4	-0.187	0.296	-0.631	0.528	0.829	0.246	-0.694	0.487	0.464	1.482
DRD3	-0.438	0.3	-1.463	0.144	0.645	0.193	-1.835	0.066	0.359	1.161
DRD2	0.015	0.299	0.05	0.96	1.015	0.304	0.05	0.96	0.565	1.825
DRD1	0.338	0.298	1.135	0.256	1.402	0.417	0.963	0.335	0.782	2.512
DRC3	-0.233	0.298	-0.784	0.433	0.792	0.236	-0.883	0.378	0.442	1.419
DRAP1	0.371	0.298	1.245	0.213	1.449	0.432	1.04	0.298	0.808	2.598
DRAM1	-0.388	0.299	-1.298	0.194	0.679	0.203	-1.585	0.113	0.378	1.219
DR1	-0.086	0.295	-0.29	0.772	0.918	0.271	-0.303	0.762	0.514	1.638
DPYSL4	-0.043	0.297	-0.145	0.885	0.958	0.284	-0.148	0.882	0.536	1.713
DPYSL3	-0.608	0.303	-2.01	0.044	0.544	0.165	-2.766	0.006	0.301	0.985
DPYSL2	-0.326	0.298	-1.097	0.273	0.721	0.215	-1.298	0.194	0.403	1.293
DPYS	0.263	0.298	0.884	0.377	1.301	0.387	0.777	0.437	0.726	2.332
DPYD	-0.566	0.303	-1.869	0.062	0.568	0.172	-2.513	0.012	0.314	1.028
DPY19L4	0.09	0.295	0.305	0.761	1.094	0.323	0.291	0.771	0.614	1.95
DPY19L2P2	-0.258	0.297	-0.869	0.385	0.773	0.229	-0.991	0.322	0.432	1.382
DPY19L1P1	-0.332	0.298	-1.116	0.265	0.717	0.214	-1.323	0.186	0.4	1.286
DPY19L1	-0.213	0.297	-0.717	0.473	0.809	0.24	-0.799	0.424	0.452	1.446
DPT	-1.026	0.321	-3.197	0.001	0.358	0.115	-5.579	0	0.191	0.672
DPPA4	0.028	0.296	0.094	0.925	1.028	0.304	0.092	0.926	0.576	1.836
DPP8	-0.471	0.302	-1.557	0.119	0.625	0.189	-1.989	0.047	0.345	1.13
DPP6	-0.017	0.295	-0.058	0.954	0.983	0.29	-0.059	0.953	0.551	1.753
DPP4	-0.311	0.297	-1.047	0.295	0.732	0.218	-1.229	0.219	0.409	1.312
DPP3	0.376	0.298	1.264	0.206	1.457	0.434	1.054	0.292	0.813	2.611
DPM3	-0.009	0.297	-0.03	0.976	0.991	0.294	-0.03	0.976	0.554	1.773
DPM2	0.287	0.296	0.968	0.333	1.332	0.395	0.841	0.4	0.745	2.381
DPM1	0.722	0.31	2.331	0.02	2.059	0.638	1.66	0.097	1.122	3.778
DPH5	-0.392	0.3	-1.307	0.191	0.676	0.203	-1.6	0.11	0.376	1.216
DPH2	0.265	0.296	0.896	0.37	1.304	0.386	0.787	0.431	0.73	2.331
DPF3	-0.362	0.299	-1.21	0.226	0.697	0.208	-1.458	0.145	0.388	1.251

DPF2	0.31	0.298	1.043	0.297	1.364	0.406	0.897	0.37	0.761	2.444
DPF1	-0.285	0.3	-0.952	0.341	0.752	0.225	-1.102	0.271	0.418	1.353
DPEP3	-0.157	0.296	-0.53	0.596	0.855	0.253	-0.573	0.566	0.478	1.527
DPEP2	-0.467	0.3	-1.558	0.119	0.627	0.188	-1.985	0.047	0.348	1.128
DPEP1	-0.252	0.297	-0.849	0.396	0.777	0.231	-0.965	0.334	0.434	1.391
DPAGT1	-0.426	0.3	-1.421	0.155	0.653	0.196	-1.772	0.076	0.363	1.175
DOT1L	-0.419	0.3	-1.399	0.162	0.657	0.197	-1.738	0.082	0.365	1.183
DOP1B	-0.266	0.296	-0.899	0.369	0.766	0.227	-1.03	0.303	0.429	1.369
DOP1A	0.584	0.306	1.91	0.056	1.793	0.548	1.447	0.148	0.985	3.262
DONSON	0.402	0.298	1.349	0.177	1.494	0.445	1.111	0.267	0.834	2.678
DOLPP1	-0.106	0.296	-0.358	0.72	0.899	0.266	-0.378	0.705	0.504	1.606
DOLK	0.042	0.297	0.14	0.888	1.043	0.31	0.138	0.891	0.582	1.866
DOK5	-0.442	0.3	-1.475	0.14	0.642	0.193	-1.855	0.064	0.357	1.157
DOK4	-0.374	0.303	-1.234	0.217	0.688	0.208	-1.497	0.134	0.38	1.246
DOK3	0.055	0.296	0.186	0.852	1.057	0.313	0.181	0.856	0.592	1.887
DOK2	0.341	0.3	1.135	0.256	1.406	0.422	0.962	0.336	0.781	2.531
DOK1	-0.401	0.304	-1.32	0.187	0.67	0.203	-1.624	0.104	0.369	1.215
DOHH	0.99	0.319	3.106	0.002	2.691	0.858	1.972	0.049	1.441	5.027
DOCK9	0.007	0.295	0.025	0.98	1.007	0.298	0.025	0.98	0.565	1.797
DOCK6	-0.489	0.3	-1.627	0.104	0.614	0.184	-2.098	0.036	0.341	1.105
DOCK5	0.118	0.297	0.396	0.692	1.125	0.334	0.374	0.709	0.629	2.013
DOCK4	-0.006	0.298	-0.02	0.984	0.994	0.296	-0.02	0.984	0.555	1.781
DOCK3	0.401	0.303	1.325	0.185	1.494	0.452	1.091	0.275	0.825	2.704
DOCK2	-0.021	0.295	-0.07	0.944	0.98	0.289	-0.07	0.944	0.549	1.748
DOCK10	-0.996	0.328	-3.039	0.002	0.369	0.121	-5.21	0	0.194	0.702
DOCK1	-0.494	0.306	-1.614	0.107	0.61	0.187	-2.088	0.037	0.335	1.112
DOC2B	0.461	0.302	1.527	0.127	1.586	0.479	1.223	0.221	0.877	2.868
DOC2A	-0.436	0.3	-1.456	0.145	0.647	0.194	-1.825	0.068	0.359	1.163
DNTTIP2	0.017	0.295	0.059	0.953	1.018	0.301	0.059	0.953	0.57	1.816
DNTT	0.359	0.3	1.196	0.232	1.432	0.429	1.005	0.315	0.795	2.577
DNPH1	0.559	0.306	1.829	0.067	1.749	0.535	1.401	0.161	0.961	3.185
DNPEP	0.285	0.296	0.961	0.337	1.329	0.394	0.836	0.403	0.744	2.376
DNMT3L	0.087	0.295	0.295	0.768	1.091	0.322	0.283	0.777	0.612	1.946
DNMT3B	0.747	0.315	2.369	0.018	2.11	0.665	1.669	0.095	1.137	3.914
DNMT3A	0.542	0.306	1.771	0.077	1.72	0.526	1.367	0.172	0.944	3.134
DNMT1	-0.005	0.296	-0.018	0.986	0.995	0.295	-0.018	0.986	0.557	1.778
DNMBP	-0.653	0.31	-2.109	0.035	0.52	0.161	-2.976	0.003	0.284	0.955
DNM3	-0.211	0.296	-0.715	0.474	0.809	0.239	-0.797	0.426	0.453	1.445

DNM2	-0.073	0.295	-0.249	0.804	0.929	0.274	-0.258	0.796	0.521	1.658
DNM1L	0.346	0.298	1.163	0.245	1.414	0.421	0.983	0.325	0.789	2.534
DNM1	-0.348	0.3	-1.162	0.245	0.706	0.212	-1.39	0.165	0.392	1.27
DND1	0.227	0.299	0.757	0.449	1.254	0.375	0.677	0.498	0.698	2.255
DNASE2B	0.224	0.298	0.753	0.452	1.251	0.373	0.674	0.5	0.698	2.243
DNASE2	-0.472	0.301	-1.571	0.116	0.624	0.187	-2.009	0.045	0.346	1.124
DNASE1L3	-0.591	0.303	-1.952	0.051	0.554	0.168	-2.661	0.008	0.306	1.003
DNASE1L2	-0.224	0.296	-0.755	0.45	0.8	0.237	-0.846	0.398	0.447	1.43
DNASE1L1	-0.26	0.298	-0.872	0.383	0.771	0.23	-0.996	0.319	0.43	1.382
DNASE1	0.472	0.3	1.575	0.115	1.604	0.481	1.255	0.209	0.891	2.887
DNALI1	-0.091	0.296	-0.306	0.759	0.913	0.27	-0.321	0.749	0.512	1.631
DNAL4	0.155	0.296	0.526	0.599	1.168	0.346	0.487	0.626	0.654	2.086
DNAJC9	0.899	0.315	2.856	0.004	2.456	0.773	1.884	0.06	1.326	4.551
DNAJC8	0.269	0.298	0.905	0.365	1.309	0.39	0.794	0.427	0.731	2.346
DNAJC7	0.229	0.296	0.774	0.439	1.258	0.372	0.692	0.489	0.704	2.247
DNAJC6	0.368	0.298	1.234	0.217	1.444	0.43	1.032	0.302	0.805	2.59
DNAJC4	-0.847	0.315	-2.69	0.007	0.429	0.135	-4.233	0	0.231	0.795
DNAJC3	0.786	0.31	2.532	0.011	2.194	0.681	1.754	0.079	1.194	4.03
DNAJC28	0.055	0.297	0.185	0.854	1.056	0.313	0.18	0.857	0.591	1.889
DNAJC24	-0.025	0.295	-0.084	0.933	0.975	0.288	-0.085	0.932	0.547	1.74
DNAJC22	0.19	0.296	0.642	0.521	1.209	0.358	0.584	0.559	0.677	2.161
DNAJC2	0.238	0.296	0.803	0.422	1.269	0.376	0.715	0.475	0.71	2.267
DNAJC17	0.6	0.306	1.964	0.05	1.823	0.557	1.477	0.14	1.001	3.319
DNAJC16	-0.41	0.3	-1.368	0.171	0.664	0.199	-1.691	0.091	0.369	1.194
DNAJC15	0.428	0.3	1.425	0.154	1.534	0.46	1.16	0.246	0.852	2.761
DNAJC13	0.264	0.298	0.889	0.374	1.303	0.388	0.781	0.435	0.727	2.334
DNAJC12	-0.752	0.315	-2.389	0.017	0.471	0.148	-3.562	0	0.254	0.874
DNAJC11	-0.441	0.301	-1.467	0.142	0.643	0.193	-1.844	0.065	0.357	1.16
DNAJC10	-0.212	0.296	-0.714	0.475	0.809	0.24	-0.796	0.426	0.453	1.446
DNAJC1	-0.059	0.297	-0.199	0.843	0.943	0.28	-0.205	0.838	0.527	1.687
DNAJB9	-0.5	0.302	-1.653	0.098	0.607	0.183	-2.144	0.032	0.335	1.097
DNAJB6	-0.759	0.315	-2.412	0.016	0.468	0.147	-3.611	0	0.253	0.867
DNAJB5	0.276	0.296	0.93	0.353	1.317	0.39	0.813	0.416	0.737	2.355
DNAJB4	-0.528	0.303	-1.747	0.081	0.59	0.178	-2.302	0.021	0.326	1.067
DNAJB2	0.138	0.295	0.467	0.64	1.148	0.339	0.436	0.663	0.644	2.048
DNAJB14	0.133	0.295	0.45	0.653	1.142	0.337	0.421	0.674	0.64	2.037
DNAJB12	-0.213	0.297	-0.717	0.473	0.808	0.24	-0.799	0.424	0.451	1.447
DNAJB1	0.052	0.295	0.175	0.861	1.053	0.311	0.171	0.864	0.591	1.878

DNAJA4	-0.194	0.297	-0.653	0.514	0.824	0.245	-0.72	0.471	0.46	1.475
DNAJA3	0.222	0.296	0.751	0.453	1.249	0.37	0.673	0.501	0.699	2.232
DNAJA2	0.333	0.3	1.108	0.268	1.394	0.419	0.942	0.346	0.774	2.511
DNAJA1	0.2	0.296	0.677	0.499	1.222	0.362	0.613	0.54	0.684	2.184
DNAI4	-0.454	0.302	-1.503	0.133	0.635	0.192	-1.903	0.057	0.351	1.148
DNAI2	-0.245	0.298	-0.823	0.411	0.782	0.233	-0.932	0.351	0.436	1.404
DNAI1	-0.048	0.296	-0.163	0.871	0.953	0.282	-0.167	0.867	0.534	1.701
DNAH9	0.007	0.295	0.025	0.98	1.007	0.297	0.025	0.98	0.565	1.796
DNAH7	0.052	0.295	0.177	0.859	1.054	0.311	0.173	0.863	0.591	1.88
DNAH6	0.282	0.297	0.949	0.342	1.325	0.393	0.828	0.408	0.741	2.37
DNAH3	-0.218	0.296	-0.737	0.461	0.804	0.238	-0.824	0.41	0.45	1.437
DNAH2	-0.332	0.298	-1.114	0.265	0.717	0.214	-1.321	0.186	0.4	1.287
DNAH17	0.422	0.302	1.394	0.163	1.524	0.461	1.138	0.255	0.843	2.757
DNAAF5	0.365	0.3	1.217	0.224	1.44	0.432	1.02	0.308	0.8	2.592
DNAAF2	-0.05	0.295	-0.168	0.866	0.952	0.281	-0.173	0.863	0.533	1.697
DNAAF1	-0.002	0.296	-0.005	0.996	0.998	0.295	-0.005	0.996	0.559	1.783
DNA2	0.348	0.3	1.161	0.246	1.417	0.425	0.981	0.327	0.787	2.55
DMXL2	-0.512	0.302	-1.693	0.09	0.599	0.181	-2.211	0.027	0.331	1.084
DMXL1	-0.377	0.302	-1.247	0.212	0.686	0.207	-1.514	0.13	0.379	1.241
DMWD	0.123	0.3	0.409	0.682	1.131	0.339	0.385	0.7	0.628	2.034
DMTN	0.134	0.296	0.452	0.651	1.143	0.339	0.423	0.672	0.64	2.044
DMTF1	-0.105	0.297	-0.354	0.723	0.9	0.267	-0.373	0.709	0.504	1.61
DMRT1	0.135	0.295	0.456	0.648	1.144	0.338	0.427	0.67	0.641	2.042
DMPK	-0.354	0.298	-1.186	0.236	0.702	0.209	-1.423	0.155	0.392	1.259
DMP1	-0.384	0.3	-1.28	0.201	0.681	0.204	-1.56	0.119	0.379	1.226
DMD	-0.188	0.296	-0.637	0.524	0.828	0.245	-0.701	0.483	0.464	1.479
DMC1	-0.423	0.3	-1.411	0.158	0.655	0.196	-1.756	0.079	0.364	1.179
DMBT1	0.492	0.302	1.629	0.103	1.636	0.495	1.286	0.198	0.905	2.959
DMAC2L	-0.241	0.297	-0.813	0.416	0.786	0.233	-0.919	0.358	0.439	1.406
DMAC2	-0.414	0.3	-1.38	0.168	0.661	0.198	-1.71	0.087	0.367	1.19
DLX6	0.083	0.296	0.282	0.778	1.087	0.321	0.271	0.787	0.609	1.94
DLX5	0.01	0.295	0.033	0.973	1.01	0.298	0.033	0.974	0.566	1.801
DLX4	0.327	0.298	1.1	0.271	1.387	0.413	0.938	0.348	0.774	2.487
DLX2	-0.384	0.298	-1.289	0.198	0.681	0.203	-1.571	0.116	0.38	1.221
DLL3	-0.003	0.295	-0.011	0.991	0.997	0.294	-0.011	0.991	0.559	1.778
DLK2	0.571	0.306	1.863	0.062	1.769	0.542	1.42	0.156	0.971	3.224
DLK1	0.234	0.298	0.786	0.432	1.264	0.376	0.701	0.483	0.705	2.264
DLGAP5	0.86	0.315	2.733	0.006	2.364	0.744	1.833	0.067	1.275	4.381

DLGAP4	-0.105	0.295	-0.357	0.721	0.9	0.266	-0.376	0.707	0.505	1.605
DLGAP2	-0.618	0.304	-2.03	0.042	0.539	0.164	-2.809	0.005	0.297	0.979
DLGAP1	0.537	0.302	1.775	0.076	1.71	0.517	1.374	0.17	0.946	3.094
DLG5	-0.105	0.296	-0.356	0.722	0.9	0.267	-0.375	0.708	0.503	1.609
DLG4	-0.027	0.295	-0.09	0.928	0.974	0.288	-0.091	0.927	0.546	1.737
DLG3	-0.226	0.298	-0.759	0.448	0.798	0.237	-0.851	0.395	0.445	1.43
DLG2	0.139	0.295	0.471	0.638	1.149	0.339	0.44	0.66	0.644	2.05
DLG1	0.178	0.295	0.604	0.546	1.195	0.353	0.553	0.58	0.67	2.133
DLEU2	0.164	0.295	0.554	0.58	1.178	0.348	0.511	0.609	0.66	2.101
DLEU1	0.499	0.302	1.652	0.098	1.648	0.498	1.301	0.193	0.911	2.979
DLEC1	-0.41	0.298	-1.374	0.169	0.664	0.198	-1.699	0.089	0.37	1.191
DLD	-0.188	0.298	-0.631	0.528	0.829	0.247	-0.694	0.488	0.462	1.486
DLC1	-0.567	0.302	-1.876	0.061	0.567	0.172	-2.524	0.012	0.313	1.026
DLAT	-0.617	0.304	-2.034	0.042	0.539	0.164	-2.813	0.005	0.297	0.978
DKKL1	0.144	0.296	0.487	0.626	1.155	0.342	0.454	0.65	0.646	2.065
DKK4	-0.522	0.303	-1.726	0.084	0.593	0.18	-2.266	0.023	0.328	1.073
DKK3	-0.918	0.316	-2.909	0.004	0.399	0.126	-4.768	0	0.215	0.741
DKK2	-0.946	0.321	-2.948	0.003	0.388	0.125	-4.909	0	0.207	0.728
DKK1	-0.296	0.298	-0.994	0.32	0.743	0.222	-1.157	0.247	0.414	1.334
DKFZP586I1420	-0.397	0.299	-1.325	0.185	0.673	0.201	-1.626	0.104	0.374	1.21
DKFZP434A062	0.102	0.295	0.346	0.729	1.108	0.327	0.329	0.742	0.621	1.977
DIXDC1	-0.519	0.302	-1.718	0.086	0.595	0.18	-2.253	0.024	0.329	1.076
DISC1	0.198	0.296	0.668	0.504	1.219	0.361	0.606	0.545	0.682	2.177
DIS3	-0.399	0.3	-1.332	0.183	0.671	0.201	-1.636	0.102	0.373	1.207
DIRAS3	-0.492	0.306	-1.607	0.108	0.611	0.187	-2.076	0.038	0.335	1.114
DIRAS2	0.388	0.303	1.28	0.201	1.474	0.447	1.061	0.289	0.814	2.669
DIPK2B	0.094	0.295	0.317	0.752	1.098	0.324	0.302	0.762	0.615	1.959
DIPK1A	-0.357	0.3	-1.191	0.234	0.7	0.21	-1.431	0.152	0.389	1.259
DIP2C	0.12	0.295	0.407	0.684	1.128	0.333	0.383	0.702	0.632	2.012
DIP2A	0.139	0.297	0.47	0.638	1.15	0.341	0.439	0.661	0.643	2.056
DIO3	-0.547	0.303	-1.807	0.071	0.579	0.175	-2.404	0.016	0.32	1.048
DIO2	-0.523	0.3	-1.74	0.082	0.593	0.178	-2.286	0.022	0.329	1.068
DIO1	-0.192	0.298	-0.643	0.52	0.825	0.246	-0.709	0.478	0.46	1.481
DIMT1	-0.175	0.299	-0.587	0.557	0.839	0.251	-0.642	0.521	0.467	1.507
DIDO1	0.539	0.306	1.762	0.078	1.714	0.525	1.362	0.173	0.941	3.123
DICER1	-0.125	0.296	-0.423	0.672	0.882	0.261	-0.451	0.652	0.494	1.576
DIAPH3	0.142	0.296	0.481	0.631	1.153	0.341	0.448	0.654	0.646	2.058
DIAPH2-AS1	-0.051	0.295	-0.173	0.863	0.95	0.281	-0.177	0.859	0.533	1.696

DIAPH2	-0.135	0.295	-0.456	0.649	0.874	0.258	-0.488	0.626	0.49	1.56
DIAPH1	0.279	0.3	0.931	0.352	1.322	0.397	0.812	0.417	0.734	2.38
DIABLO	0.243	0.296	0.821	0.411	1.276	0.378	0.729	0.466	0.714	2.281
DHX9	0.446	0.3	1.489	0.136	1.562	0.468	1.201	0.23	0.868	2.811
DHX8	-0.014	0.298	-0.048	0.962	0.986	0.294	-0.048	0.962	0.55	1.768
DHX58	0.016	0.297	0.055	0.956	1.017	0.302	0.055	0.956	0.568	1.819
DHX57	-0.049	0.296	-0.167	0.868	0.952	0.282	-0.171	0.864	0.533	1.7
DHX40	-0.435	0.302	-1.439	0.15	0.647	0.196	-1.802	0.072	0.358	1.171
DHX38	-0.278	0.298	-0.934	0.35	0.757	0.226	-1.077	0.281	0.422	1.357
DHX35	-0.201	0.298	-0.676	0.499	0.818	0.243	-0.749	0.454	0.456	1.465
DHX34	0.444	0.3	1.481	0.139	1.558	0.467	1.196	0.232	0.866	2.804
DHX32	-0.264	0.298	-0.887	0.375	0.768	0.229	-1.015	0.31	0.428	1.376
DHX30	0.333	0.297	1.12	0.263	1.395	0.415	0.952	0.341	0.779	2.5
DHX29	-0.551	0.306	-1.802	0.072	0.577	0.176	-2.403	0.016	0.317	1.05
DHX16	0.179	0.296	0.606	0.545	1.196	0.354	0.555	0.579	0.67	2.135
DHX15	0.72	0.307	2.349	0.019	2.054	0.63	1.674	0.094	1.127	3.747
DHTKD1	1.212	0.336	3.606	0	3.362	1.131	2.089	0.037	1.739	6.499
DHRS9	0.129	0.295	0.438	0.662	1.138	0.336	0.41	0.681	0.638	2.03
DHRS7B	-0.144	0.296	-0.485	0.627	0.866	0.257	-0.522	0.602	0.485	1.548
DHRS7	-0.141	0.297	-0.475	0.635	0.868	0.258	-0.51	0.61	0.485	1.555
DHRS3	0.228	0.296	0.768	0.442	1.256	0.372	0.687	0.492	0.702	2.245
DHRS2	-0.174	0.296	-0.588	0.556	0.84	0.249	-0.643	0.52	0.47	1.501
DHRS12	-0.417	0.303	-1.379	0.168	0.659	0.199	-1.711	0.087	0.364	1.192
DHRS11	0.312	0.297	1.052	0.293	1.366	0.405	0.904	0.366	0.764	2.443
DHRS1	0.264	0.297	0.89	0.373	1.302	0.386	0.782	0.434	0.728	2.33
DHPS	-0.437	0.306	-1.426	0.154	0.646	0.198	-1.788	0.074	0.354	1.178
DHODH	0.354	0.3	1.181	0.237	1.425	0.427	0.995	0.32	0.792	2.564
DHFRP3	0.098	0.295	0.332	0.74	1.103	0.326	0.316	0.752	0.618	1.967
DHFR	0.932	0.315	2.959	0.003	2.54	0.8	1.925	0.054	1.37	4.709
DHDDS	0.302	0.297	1.017	0.309	1.352	0.402	0.878	0.38	0.756	2.42
DHCR7	0.213	0.296	0.718	0.473	1.237	0.367	0.647	0.518	0.692	2.212
DHCR24	0.094	0.295	0.317	0.751	1.098	0.324	0.303	0.762	0.616	1.959
DGUOK	0.719	0.31	2.321	0.02	2.053	0.636	1.655	0.098	1.118	3.768
DGLUCY	-0.241	0.298	-0.808	0.419	0.786	0.234	-0.914	0.361	0.438	1.409
DGKZ	-0.292	0.297	-0.981	0.327	0.747	0.222	-1.139	0.255	0.417	1.338
DGKQ	-0.117	0.295	-0.394	0.693	0.89	0.263	-0.418	0.676	0.499	1.588
DGKI	-0.555	0.303	-1.828	0.067	0.574	0.174	-2.444	0.015	0.317	1.041
DGKH	-0.005	0.295	-0.016	0.987	0.995	0.294	-0.016	0.987	0.558	1.775

DGKG	0.026	0.295	0.087	0.93	1.026	0.303	0.086	0.931	0.575	1.831
DGKE	-0.341	0.3	-1.14	0.254	0.711	0.213	-1.359	0.174	0.395	1.278
DGKD	-0.119	0.295	-0.404	0.686	0.887	0.262	-0.429	0.668	0.497	1.583
DGKB	0.154	0.296	0.519	0.604	1.166	0.345	0.481	0.63	0.653	2.084
DGKA	-0.787	0.31	-2.538	0.011	0.455	0.141	-3.86	0	0.248	0.836
DGCR6L	-0.404	0.299	-1.349	0.177	0.668	0.2	-1.662	0.096	0.372	1.2
DGCR5	0.538	0.303	1.777	0.075	1.712	0.518	1.375	0.169	0.946	3.097
DGCR2	-0.25	0.298	-0.84	0.401	0.779	0.232	-0.954	0.34	0.434	1.396
DGCR11	0.073	0.295	0.247	0.805	1.076	0.317	0.238	0.812	0.603	1.918
DGAT1	0.477	0.302	1.579	0.114	1.612	0.487	1.255	0.209	0.891	2.914
DFFB	-0.415	0.302	-1.374	0.17	0.66	0.2	-1.703	0.089	0.365	1.194
DFFA	0.459	0.3	1.527	0.127	1.582	0.475	1.225	0.221	0.878	2.85
DEXI	-0.271	0.298	-0.911	0.362	0.762	0.227	-1.047	0.295	0.425	1.366
DET1	-0.144	0.296	-0.487	0.626	0.866	0.256	-0.524	0.6	0.485	1.546
DESI2	0.01	0.296	0.035	0.972	1.01	0.299	0.035	0.972	0.566	1.805
DESI1	-0.248	0.298	-0.833	0.405	0.78	0.232	-0.946	0.344	0.435	1.399
DES	0.393	0.298	1.319	0.187	1.481	0.441	1.091	0.275	0.826	2.654
DERL2	0.137	0.296	0.463	0.643	1.147	0.34	0.433	0.665	0.642	2.051
DERL1	0.292	0.296	0.984	0.325	1.339	0.397	0.854	0.393	0.749	2.393
DERA	0.001	0.296	0.005	0.996	1.001	0.296	0.005	0.996	0.561	1.787
DEPTOR	-0.134	0.296	-0.451	0.652	0.875	0.259	-0.483	0.629	0.489	1.564
DEPP1	-0.113	0.296	-0.38	0.704	0.893	0.265	-0.403	0.687	0.5	1.597
DEPDC5	0.192	0.296	0.647	0.518	1.211	0.358	0.589	0.556	0.678	2.163
DEPDC1	0.723	0.31	2.332	0.02	2.06	0.639	1.66	0.097	1.122	3.783
DENR	-0.079	0.295	-0.268	0.789	0.924	0.273	-0.279	0.78	0.518	1.648
DENND6B	-0.225	0.298	-0.756	0.45	0.799	0.238	-0.848	0.397	0.446	1.431
DENND5B	0.274	0.297	0.925	0.355	1.316	0.39	0.809	0.419	0.736	2.352
DENND5A	0.171	0.296	0.579	0.563	1.187	0.351	0.532	0.595	0.665	2.118
DENND4C	-0.522	0.303	-1.724	0.085	0.593	0.18	-2.265	0.024	0.328	1.074
DENND4B	-0.109	0.296	-0.369	0.712	0.897	0.265	-0.39	0.696	0.502	1.6
DENND4A	-0.375	0.303	-1.238	0.216	0.688	0.208	-1.502	0.133	0.38	1.244
DENND3	0.026	0.296	0.087	0.93	1.026	0.304	0.086	0.931	0.574	1.834
DENND2D	-0.084	0.298	-0.281	0.779	0.92	0.274	-0.293	0.77	0.513	1.648
DENND2B	-0.107	0.296	-0.361	0.718	0.898	0.266	-0.381	0.703	0.503	1.606
DENND2A	-0.097	0.295	-0.328	0.743	0.908	0.268	-0.344	0.731	0.509	1.62
DENND1C	-0.474	0.3	-1.581	0.114	0.623	0.187	-2.022	0.043	0.346	1.12
DENND1B	-0.17	0.296	-0.575	0.566	0.844	0.25	-0.626	0.531	0.472	1.507
DENND1A	0.692	0.31	2.234	0.025	1.998	0.619	1.612	0.107	1.089	3.665

DELEC1	0.127	0.297	0.427	0.669	1.135	0.338	0.401	0.688	0.634	2.034
DELE1	0.013	0.295	0.043	0.966	1.013	0.299	0.042	0.966	0.568	1.807
DEK	0.248	0.296	0.837	0.403	1.281	0.38	0.741	0.459	0.717	2.29
DEGS1	0.063	0.296	0.214	0.831	1.065	0.316	0.207	0.836	0.596	1.904
DEFB126	0.05	0.296	0.17	0.865	1.052	0.311	0.166	0.868	0.589	1.877
DEFB1	-0.087	0.295	-0.296	0.767	0.916	0.27	-0.31	0.757	0.514	1.634
DEFA6	-0.32	0.297	-1.075	0.282	0.726	0.216	-1.267	0.205	0.406	1.301
DEFA5	0.401	0.3	1.339	0.181	1.494	0.448	1.103	0.27	0.83	2.687
DEFA4	-0.226	0.297	-0.763	0.445	0.797	0.237	-0.856	0.392	0.446	1.426
DEF8	0.222	0.296	0.75	0.453	1.249	0.37	0.673	0.501	0.699	2.231
DEF6	-0.286	0.297	-0.966	0.334	0.751	0.223	-1.118	0.263	0.42	1.343
DEDD	-0.294	0.298	-0.987	0.324	0.745	0.222	-1.147	0.251	0.416	1.336
DECR2	0.488	0.3	1.626	0.104	1.63	0.489	1.286	0.198	0.905	2.936
DECR1	0.574	0.301	1.906	0.057	1.775	0.534	1.451	0.147	0.984	3.2
DEAF1	-0.405	0.3	-1.35	0.177	0.667	0.2	-1.664	0.096	0.371	1.2
DDX60	-0.298	0.298	-1.001	0.317	0.742	0.221	-1.166	0.244	0.414	1.331
DDX6	0.669	0.307	2.184	0.029	1.953	0.599	1.592	0.111	1.071	3.561
DDX58	0.181	0.296	0.613	0.54	1.199	0.355	0.56	0.575	0.671	2.142
DDX56	0.662	0.306	2.165	0.03	1.938	0.592	1.583	0.113	1.065	3.528
DDX54	-0.175	0.298	-0.586	0.558	0.84	0.25	-0.64	0.522	0.468	1.507
DDX52	0.455	0.303	1.503	0.133	1.577	0.478	1.207	0.227	0.871	2.855
DDX51	-0.057	0.295	-0.192	0.847	0.945	0.279	-0.198	0.843	0.53	1.686
DDX50	-0.02	0.296	-0.067	0.947	0.98	0.29	-0.068	0.946	0.549	1.752
DDX49	0.474	0.302	1.568	0.117	1.606	0.485	1.249	0.212	0.888	2.904
DDX46	0.197	0.296	0.664	0.507	1.217	0.361	0.603	0.547	0.681	2.175
DDX43	-0.107	0.297	-0.36	0.719	0.899	0.267	-0.379	0.704	0.502	1.608
DDX42	0.725	0.315	2.305	0.021	2.065	0.65	1.64	0.101	1.115	3.826
DDX41	0.264	0.296	0.89	0.373	1.302	0.386	0.783	0.434	0.728	2.327
DDX4	0.239	0.296	0.807	0.42	1.27	0.376	0.718	0.473	0.711	2.27
DDX3Y	-0.642	0.306	-2.096	0.036	0.526	0.161	-2.94	0.003	0.288	0.959
DDX3X	-0.462	0.3	-1.537	0.124	0.63	0.189	-1.954	0.051	0.35	1.135
DDX39A	0.333	0.301	1.107	0.268	1.396	0.42	0.942	0.346	0.774	2.517
DDX31	0.242	0.296	0.816	0.415	1.274	0.377	0.725	0.469	0.712	2.277
DDX28	0.464	0.3	1.549	0.121	1.591	0.477	1.239	0.215	0.884	2.862
DDX27	0.425	0.303	1.403	0.161	1.529	0.463	1.143	0.253	0.845	2.767
DDX25	0.241	0.296	0.815	0.415	1.273	0.377	0.724	0.469	0.712	2.275
DDX24	0.266	0.298	0.892	0.372	1.304	0.388	0.784	0.433	0.728	2.337
DDX23	-0.184	0.296	-0.62	0.535	0.832	0.247	-0.68	0.496	0.465	1.488

DDX21	0.074	0.298	0.247	0.805	1.076	0.321	0.238	0.812	0.6	1.93
DDX19A	0.412	0.3	1.375	0.169	1.51	0.452	1.127	0.26	0.839	2.715
DDX18	0.677	0.31	2.185	0.029	1.968	0.609	1.588	0.112	1.072	3.611
DDX17	-0.149	0.296	-0.502	0.616	0.862	0.255	-0.542	0.588	0.482	1.541
DDX11	-0.245	0.298	-0.824	0.41	0.783	0.233	-0.934	0.35	0.437	1.402
DDX10	0.268	0.297	0.904	0.366	1.307	0.388	0.793	0.428	0.731	2.338
DDX1	-0.603	0.304	-1.985	0.047	0.547	0.166	-2.724	0.006	0.302	0.992
DDRGK1	-0.242	0.296	-0.818	0.413	0.785	0.233	-0.926	0.355	0.439	1.403
DDR2	-0.644	0.304	-2.121	0.034	0.525	0.159	-2.977	0.003	0.29	0.952
DDR1-DT	0.449	0.3	1.498	0.134	1.567	0.469	1.207	0.228	0.871	2.818
DDOST	-0.613	0.306	-2.007	0.045	0.542	0.166	-2.769	0.006	0.298	0.986
DDO	-0.067	0.297	-0.224	0.823	0.935	0.278	-0.232	0.817	0.522	1.676
DDN	0.396	0.3	1.323	0.186	1.486	0.445	1.092	0.275	0.826	2.674
DDIT4	0.359	0.298	1.206	0.228	1.432	0.426	1.013	0.311	0.799	2.566
DDIT3	0.187	0.298	0.627	0.53	1.206	0.36	0.572	0.567	0.672	2.163
DDHD2	-0.255	0.298	-0.855	0.392	0.775	0.231	-0.974	0.33	0.432	1.39
DDC	0.272	0.298	0.913	0.361	1.312	0.39	0.8	0.424	0.732	2.351
DDB2	0.014	0.3	0.047	0.963	1.014	0.304	0.047	0.963	0.563	1.826
DDB1	-0.122	0.296	-0.414	0.679	0.885	0.262	-0.44	0.66	0.495	1.581
DDAH2	0.716	0.307	2.332	0.02	2.046	0.628	1.666	0.096	1.121	3.734
DDAH1	-0.206	0.3	-0.685	0.493	0.814	0.244	-0.761	0.447	0.452	1.466
DDA1	0.272	0.297	0.916	0.36	1.312	0.389	0.802	0.423	0.734	2.346
DCXR	-0.208	0.296	-0.701	0.483	0.812	0.241	-0.78	0.436	0.454	1.452
DCX	-0.115	0.295	-0.388	0.698	0.892	0.263	-0.411	0.681	0.5	1.591
DCUN1D4	0.694	0.306	2.264	0.024	2.001	0.613	1.633	0.103	1.098	3.649
DCUN1D2	0.259	0.297	0.873	0.383	1.296	0.385	0.769	0.442	0.724	2.321
DCUN1D1	0.671	0.303	2.214	0.027	1.955	0.592	1.613	0.107	1.08	3.541
DCTPP1	-0.181	0.298	-0.609	0.542	0.834	0.248	-0.668	0.504	0.465	1.495
DCTN6	-0.002	0.297	-0.006	0.995	0.998	0.297	-0.006	0.995	0.557	1.788
DCTN5	0.811	0.315	2.573	0.01	2.249	0.709	1.763	0.078	1.213	4.17
DCTN4	-1.176	0.337	-3.491	0	0.308	0.104	-6.654	0	0.159	0.597
DCTN3	0.205	0.296	0.691	0.49	1.227	0.363	0.625	0.532	0.687	2.192
DCTN2	-0.283	0.3	-0.945	0.345	0.753	0.226	-1.092	0.275	0.419	1.355
DCTN1	-0.144	0.296	-0.488	0.625	0.865	0.256	-0.525	0.599	0.485	1.545
DCTD	-1.271	0.346	-3.67	0	0.281	0.097	-7.405	0	0.142	0.553
DCT	-0.182	0.296	-0.615	0.538	0.833	0.247	-0.675	0.5	0.467	1.489
DCSTAMP	0.514	0.302	1.698	0.089	1.671	0.505	1.328	0.184	0.924	3.023
DCPS	-0.001	0.295	-0.004	0.997	0.999	0.295	-0.004	0.997	0.56	1.781

DCP2	-0.215	0.296	-0.726	0.468	0.806	0.239	-0.81	0.418	0.451	1.442
DCP1A	-0.668	0.31	-2.158	0.031	0.513	0.159	-3.07	0.002	0.279	0.941
DCN	-0.864	0.315	-2.744	0.006	0.422	0.133	-4.358	0	0.228	0.781
DCLRE1C	1.13	0.329	3.438	0.001	3.097	1.018	2.06	0.039	1.626	5.899
DCLRE1B	0.598	0.303	1.971	0.049	1.818	0.551	1.484	0.138	1.003	3.293
DCLRE1A	-0.12	0.296	-0.405	0.685	0.887	0.263	-0.431	0.667	0.496	1.585
DCLK2	0.739	0.31	2.387	0.017	2.095	0.649	1.687	0.092	1.141	3.843
DCLK1	0.064	0.295	0.217	0.829	1.066	0.315	0.21	0.834	0.597	1.902
DCK	-0.073	0.296	-0.246	0.805	0.93	0.275	-0.256	0.798	0.52	1.661
DCHS2	0.135	0.295	0.456	0.649	1.144	0.338	0.426	0.67	0.641	2.041
DCHS1	0.002	0.295	0.007	0.995	1.002	0.296	0.007	0.995	0.562	1.787
DCC	-0.256	0.298	-0.861	0.389	0.774	0.23	-0.981	0.327	0.432	1.387
DCBLD2	0.35	0.298	1.174	0.24	1.418	0.422	0.991	0.322	0.791	2.542
DCAKD	-0.043	0.297	-0.145	0.885	0.958	0.284	-0.148	0.882	0.535	1.714
DCAF8	-0.335	0.298	-1.127	0.26	0.715	0.213	-1.339	0.181	0.399	1.281
DCAF7	0.569	0.306	1.862	0.063	1.766	0.54	1.42	0.156	0.971	3.215
DCAF6	0.118	0.296	0.4	0.689	1.126	0.333	0.377	0.706	0.631	2.009
DCAF4	0.023	0.296	0.077	0.939	1.023	0.303	0.076	0.939	0.573	1.827
DCAF17	0.742	0.306	2.424	0.015	2.1	0.643	1.711	0.087	1.153	3.826
DCAF16	-0.159	0.296	-0.539	0.59	0.853	0.252	-0.584	0.559	0.478	1.522
DCAF15	-0.673	0.31	-2.169	0.03	0.51	0.158	-3.093	0.002	0.278	0.937
DCAF13	0.233	0.296	0.787	0.431	1.263	0.374	0.702	0.483	0.707	2.256
DCAF11	0.288	0.298	0.966	0.334	1.333	0.397	0.839	0.401	0.744	2.391
DCAF10	0.121	0.296	0.41	0.682	1.129	0.334	0.386	0.699	0.632	2.016
DCAF1	0.361	0.297	1.215	0.224	1.435	0.426	1.02	0.308	0.801	2.569
DBT	0.158	0.299	0.529	0.597	1.171	0.35	0.489	0.625	0.652	2.103
DBR1	0.133	0.295	0.449	0.653	1.142	0.337	0.421	0.674	0.64	2.036
DBP	-0.544	0.312	-1.747	0.081	0.58	0.181	-2.322	0.02	0.315	1.069
DBNDD1	0.142	0.296	0.481	0.63	1.153	0.341	0.449	0.654	0.646	2.059
DBN1	0.663	0.306	2.17	0.03	1.941	0.593	1.586	0.113	1.066	3.534
DBI	0.202	0.296	0.683	0.495	1.224	0.363	0.618	0.537	0.685	2.188
DBH	-0.194	0.296	-0.656	0.512	0.823	0.244	-0.724	0.469	0.461	1.472
DBF4B	0.225	0.299	0.752	0.452	1.252	0.375	0.673	0.501	0.697	2.251
DBF4	0.28	0.296	0.944	0.345	1.323	0.392	0.824	0.41	0.74	2.363
DAZL	0.113	0.295	0.383	0.701	1.12	0.331	0.363	0.717	0.628	1.998
DAZAP2	-0.854	0.315	-2.713	0.007	0.426	0.134	-4.285	0	0.23	0.789
DAZAP1	0.602	0.304	1.981	0.048	1.826	0.555	1.488	0.137	1.006	3.312
DAXX	-0.334	0.298	-1.122	0.262	0.716	0.213	-1.332	0.183	0.399	1.284

DARS2	0.354	0.298	1.189	0.235	1.424	0.424	1.001	0.317	0.795	2.552
DARS1	0.166	0.295	0.562	0.574	1.181	0.349	0.518	0.605	0.662	2.106
DAPP1	0.13	0.299	0.435	0.663	1.139	0.341	0.408	0.683	0.634	2.048
DAPK2	0.677	0.303	2.23	0.026	1.967	0.597	1.62	0.105	1.085	3.566
DAPK1	0.523	0.306	1.709	0.087	1.687	0.516	1.331	0.183	0.926	3.073
DAP3	-0.209	0.296	-0.704	0.482	0.812	0.241	-0.782	0.434	0.454	1.451
DAP	-0.177	0.296	-0.599	0.549	0.837	0.248	-0.656	0.512	0.469	1.496
DAO	0.928	0.321	2.894	0.004	2.528	0.81	1.886	0.059	1.349	4.739
DALRD3	-0.348	0.303	-1.146	0.252	0.706	0.214	-1.37	0.171	0.39	1.28
DAGLA	-0.074	0.295	-0.252	0.801	0.928	0.274	-0.262	0.794	0.52	1.656
DAG1	-0.305	0.297	-1.028	0.304	0.737	0.219	-1.201	0.23	0.412	1.319
DAD1	0.381	0.298	1.278	0.201	1.464	0.437	1.062	0.288	0.816	2.628
DACT1	-0.481	0.3	-1.602	0.109	0.618	0.186	-2.057	0.04	0.343	1.113
DACH1	0.275	0.299	0.921	0.357	1.316	0.393	0.805	0.421	0.733	2.364
DAB2	-0.786	0.308	-2.554	0.011	0.456	0.14	-3.88	0	0.249	0.833
DAB1	-0.373	0.3	-1.245	0.213	0.689	0.206	-1.509	0.131	0.383	1.239
DAAM2	-0.141	0.295	-0.477	0.634	0.869	0.257	-0.512	0.609	0.487	1.55
DAAM1	0.549	0.302	1.815	0.069	1.732	0.524	1.397	0.162	0.957	3.133
CZIB	-0.01	0.295	-0.033	0.974	0.99	0.292	-0.033	0.974	0.556	1.766
CYTL1	-0.989	0.328	-3.019	0.003	0.372	0.122	-5.155	0	0.196	0.707
CYTIP	-0.454	0.303	-1.501	0.133	0.635	0.192	-1.9	0.057	0.351	1.149
CYTH4	-0.01	0.295	-0.034	0.973	0.99	0.293	-0.034	0.973	0.555	1.767
CYTH3	0.327	0.299	1.091	0.275	1.387	0.415	0.931	0.352	0.771	2.494
CYTH2	-0.349	0.303	-1.151	0.25	0.705	0.214	-1.377	0.168	0.389	1.278
CYTH1	0.307	0.298	1.031	0.302	1.359	0.404	0.888	0.374	0.759	2.436
CYSLTR2	-0.5	0.302	-1.654	0.098	0.606	0.183	-2.147	0.032	0.335	1.097
CYSLTR1	0.418	0.3	1.395	0.163	1.519	0.455	1.14	0.254	0.844	2.732
CYRIB	0.181	0.297	0.61	0.542	1.198	0.356	0.558	0.577	0.67	2.144
CYRIA	0.329	0.3	1.095	0.274	1.389	0.417	0.933	0.351	0.771	2.504
CYREN	0.187	0.296	0.632	0.528	1.206	0.357	0.576	0.565	0.675	2.156
CYP7B1	0.4	0.298	1.345	0.179	1.492	0.444	1.108	0.268	0.833	2.675
CYP7A1	-0.135	0.296	-0.456	0.649	0.874	0.258	-0.488	0.626	0.49	1.56
CYP4F8	0.648	0.306	2.118	0.034	1.912	0.585	1.559	0.119	1.049	3.483
CYP4F3	-0.493	0.302	-1.631	0.103	0.611	0.185	-2.108	0.035	0.338	1.105
CYP4F2	0.19	0.295	0.644	0.519	1.21	0.357	0.587	0.557	0.678	2.158
CYP4F12	-0.606	0.31	-1.955	0.051	0.546	0.169	-2.688	0.007	0.297	1.001
CYP4F11	-0.179	0.299	-0.599	0.549	0.836	0.25	-0.657	0.511	0.465	1.503
CYP4B1	0.291	0.299	0.973	0.331	1.338	0.4	0.844	0.399	0.744	2.403

CYP4A11	-0.081	0.296	-0.275	0.783	0.922	0.273	-0.287	0.774	0.516	1.645
CYP46A1	-0.156	0.296	-0.527	0.598	0.855	0.253	-0.571	0.568	0.479	1.529
CYP3A7-CYP3A5	0.108	0.295	0.367	0.713	1.115	0.329	0.348	0.728	0.625	1.989
CYP3A5	0.103	0.295	0.35	0.726	1.109	0.328	0.333	0.739	0.622	1.978
CYP3A43	-0.574	0.303	-1.893	0.058	0.563	0.171	-2.557	0.011	0.311	1.021
CYP3A4	0.394	0.302	1.303	0.193	1.483	0.448	1.077	0.282	0.82	2.682
CYP39A1	-0.271	0.3	-0.906	0.365	0.762	0.228	-1.041	0.298	0.424	1.371
CYP2W1	0.256	0.299	0.858	0.391	1.292	0.386	0.757	0.449	0.72	2.319
CYP2R1	0.275	0.296	0.93	0.352	1.317	0.39	0.813	0.416	0.737	2.354
CYP2J2	0.575	0.303	1.9	0.057	1.778	0.539	1.444	0.149	0.982	3.22
CYP2F1	0.127	0.295	0.43	0.667	1.135	0.335	0.403	0.687	0.636	2.025
CYP2E1	0.397	0.304	1.306	0.191	1.487	0.451	1.078	0.281	0.82	2.696
CYP2D6	-0.339	0.298	-1.139	0.255	0.712	0.212	-1.356	0.175	0.398	1.277
CYP2C9	0.49	0.302	1.621	0.105	1.632	0.494	1.281	0.2	0.903	2.952
CYP2C8	-0.292	0.298	-0.981	0.326	0.747	0.222	-1.139	0.255	0.417	1.338
CYP2C19	0.119	0.295	0.402	0.687	1.126	0.333	0.379	0.704	0.631	2.009
CYP2C18	-0.102	0.296	-0.345	0.73	0.903	0.267	-0.363	0.716	0.506	1.612
CYP2B7P	-0.239	0.298	-0.804	0.422	0.787	0.234	-0.908	0.364	0.439	1.411
CYP2B6	-0.332	0.3	-1.107	0.268	0.718	0.215	-1.312	0.189	0.399	1.291
CYP2A7P1	-0.574	0.303	-1.895	0.058	0.563	0.171	-2.56	0.01	0.311	1.02
CYP2A7	-0.333	0.298	-1.117	0.264	0.717	0.214	-1.325	0.185	0.399	1.286
CYP2A6	0.121	0.297	0.406	0.685	1.128	0.335	0.383	0.702	0.631	2.018
CYP2A13	-0.097	0.296	-0.328	0.743	0.908	0.268	-0.345	0.73	0.508	1.62
CYP27B1	-0.265	0.297	-0.889	0.374	0.768	0.228	-1.018	0.309	0.428	1.375
CYP27A1	-0.143	0.295	-0.484	0.629	0.867	0.256	-0.52	0.603	0.486	1.547
CYP26B1	-0.211	0.296	-0.713	0.476	0.81	0.24	-0.794	0.427	0.453	1.447
CYP26A1	-0.265	0.298	-0.89	0.374	0.767	0.228	-1.019	0.308	0.428	1.375
CYP24A1	0.405	0.3	1.349	0.177	1.499	0.45	1.109	0.267	0.832	2.698
CYP20A1	-0.473	0.301	-1.573	0.116	0.623	0.187	-2.011	0.044	0.346	1.123
CYP1B1	-0.473	0.299	-1.582	0.114	0.623	0.186	-2.023	0.043	0.347	1.12
CYP1A2	0.245	0.297	0.826	0.409	1.278	0.379	0.732	0.464	0.714	2.285
CYP1A1	0.063	0.296	0.213	0.831	1.065	0.316	0.207	0.836	0.596	1.904
CYP19A1	0.264	0.298	0.886	0.375	1.302	0.388	0.779	0.436	0.726	2.336
CYP17A1	-0.208	0.296	-0.702	0.483	0.812	0.241	-0.781	0.435	0.454	1.452
CYP11B2	0.026	0.295	0.089	0.929	1.027	0.303	0.088	0.93	0.575	1.832
CYP11B1	0.175	0.296	0.59	0.555	1.191	0.353	0.541	0.588	0.666	2.129
CYP11A1	-0.195	0.296	-0.66	0.51	0.823	0.243	-0.728	0.466	0.461	1.469
CYLD	-0.314	0.298	-1.056	0.291	0.73	0.217	-1.241	0.215	0.408	1.309

CYLC2	0.074	0.295	0.251	0.802	1.077	0.318	0.242	0.809	0.604	1.921
CYLC1	-0.25	0.298	-0.84	0.401	0.778	0.232	-0.954	0.34	0.434	1.397
CYHR1	0.013	0.296	0.043	0.966	1.013	0.3	0.043	0.966	0.567	1.81
CYFIP2	0.126	0.296	0.425	0.671	1.134	0.335	0.4	0.689	0.635	2.025
CYFIP1	0.107	0.295	0.362	0.718	1.113	0.328	0.343	0.732	0.624	1.984
CYCS	0.442	0.303	1.46	0.144	1.556	0.471	1.18	0.238	0.859	2.817
CYC1	0.245	0.296	0.827	0.408	1.277	0.378	0.734	0.463	0.715	2.282
CYBRD1	-0.29	0.298	-0.974	0.33	0.748	0.223	-1.129	0.259	0.418	1.341
CYBC1	0.092	0.295	0.31	0.756	1.096	0.324	0.297	0.767	0.614	1.956
CYBB	0.135	0.296	0.455	0.649	1.144	0.338	0.426	0.67	0.641	2.042
CYBA	0.166	0.296	0.56	0.575	1.181	0.35	0.516	0.606	0.66	2.111
CYB5R4	0.159	0.296	0.536	0.592	1.172	0.347	0.495	0.62	0.656	2.094
CYB5R3	0.017	0.295	0.058	0.954	1.017	0.3	0.057	0.954	0.57	1.815
CYB5R2	0.104	0.295	0.351	0.725	1.109	0.328	0.334	0.739	0.622	1.979
CYB5R1	-0.485	0.306	-1.587	0.113	0.616	0.188	-2.042	0.041	0.338	1.121
CYB5B	0.697	0.303	2.3	0.021	2.008	0.609	1.656	0.098	1.109	3.638
CYB5A	0.422	0.299	1.414	0.157	1.526	0.456	1.154	0.249	0.85	2.74
CYB561D2	-0.474	0.303	-1.566	0.117	0.623	0.188	-2.004	0.045	0.344	1.126
CYB561	0.236	0.296	0.797	0.425	1.266	0.375	0.71	0.478	0.709	2.264
CXXC4	0.343	0.298	1.153	0.249	1.409	0.42	0.976	0.329	0.786	2.526
CXXC1	-0.526	0.302	-1.743	0.081	0.591	0.178	-2.293	0.022	0.327	1.068
CXorf56	0.878	0.316	2.782	0.005	2.406	0.759	1.852	0.064	1.296	4.466
CXCR6	-0.128	0.296	-0.434	0.664	0.88	0.26	-0.463	0.643	0.493	1.57
CXCR5	0.073	0.295	0.247	0.805	1.076	0.318	0.238	0.812	0.603	1.919
CXCR4	-0.035	0.296	-0.12	0.905	0.965	0.285	-0.122	0.903	0.541	1.723
CXCR3	0.135	0.296	0.457	0.648	1.145	0.339	0.427	0.669	0.64	2.047
CXCR2	-0.107	0.296	-0.361	0.718	0.899	0.266	-0.381	0.703	0.503	1.605
CXCR1	-0.041	0.298	-0.138	0.89	0.96	0.286	-0.141	0.888	0.535	1.722
CXCL9	0.332	0.3	1.107	0.268	1.394	0.418	0.942	0.346	0.774	2.511
CXCL8	0.219	0.295	0.74	0.459	1.245	0.368	0.665	0.506	0.697	2.221
CXCL6	0.05	0.295	0.17	0.865	1.051	0.31	0.165	0.869	0.59	1.875
CXCL5	0.216	0.296	0.73	0.465	1.241	0.368	0.657	0.511	0.695	2.218
CXCL3	0.277	0.296	0.934	0.35	1.319	0.391	0.816	0.415	0.738	2.357
CXCL2	-0.48	0.303	-1.586	0.113	0.619	0.187	-2.036	0.042	0.342	1.12
CXCL14	-0.392	0.3	-1.308	0.191	0.676	0.202	-1.601	0.109	0.376	1.216
CXCL13	-0.59	0.303	-1.948	0.051	0.554	0.168	-2.655	0.008	0.306	1.004
CXCL12	-1.001	0.321	-3.12	0.002	0.368	0.118	-5.364	0	0.196	0.689
CXCL11	0.139	0.297	0.468	0.639	1.149	0.341	0.437	0.662	0.642	2.056

CXCL10	0.322	0.298	1.082	0.279	1.381	0.411	0.925	0.355	0.77	2.475
CXCL1	-0.28	0.297	-0.942	0.346	0.756	0.225	-1.087	0.277	0.422	1.353
CXADR	0.438	0.3	1.463	0.143	1.55	0.465	1.185	0.236	0.862	2.789
CX3CR1	-1.015	0.328	-3.096	0.002	0.362	0.119	-5.366	0	0.191	0.689
CX3CL1	-0.035	0.296	-0.119	0.905	0.965	0.286	-0.121	0.904	0.54	1.725
CWH43	0.341	0.298	1.144	0.253	1.406	0.418	0.97	0.332	0.784	2.519
CWF19L1	-0.216	0.3	-0.719	0.472	0.806	0.242	-0.803	0.422	0.447	1.451
CWC25	0.139	0.298	0.468	0.64	1.15	0.342	0.437	0.662	0.641	2.061
CUZD1	-0.107	0.297	-0.362	0.717	0.898	0.266	-0.382	0.702	0.502	1.606
CUX2	-0.217	0.296	-0.732	0.464	0.805	0.239	-0.817	0.414	0.45	1.439
CUX1	0.079	0.295	0.267	0.79	1.082	0.319	0.257	0.797	0.607	1.93
CUTC	0.409	0.298	1.371	0.17	1.505	0.448	1.126	0.26	0.839	2.698
CUTA	0.283	0.297	0.955	0.34	1.328	0.394	0.832	0.406	0.742	2.375
CUL9	0.297	0.297	0.999	0.318	1.345	0.399	0.864	0.387	0.752	2.407
CUL7	0.254	0.298	0.853	0.394	1.289	0.384	0.753	0.451	0.719	2.313
CUL5	-0.447	0.301	-1.488	0.137	0.639	0.192	-1.876	0.061	0.355	1.153
CUL4B	-0.595	0.306	-1.947	0.052	0.552	0.169	-2.66	0.008	0.303	1.004
CUL4A	0.186	0.296	0.627	0.531	1.204	0.356	0.572	0.567	0.674	2.151
CUL3	-0.272	0.298	-0.913	0.361	0.762	0.227	-1.049	0.294	0.425	1.365
CUL2	0.714	0.31	2.305	0.021	2.042	0.632	1.648	0.099	1.113	3.746
CUL1	-0.42	0.302	-1.391	0.164	0.657	0.199	-1.728	0.084	0.363	1.188
CUEDC2	0.273	0.296	0.922	0.356	1.314	0.39	0.807	0.42	0.735	2.35
CUEDC1	0.576	0.303	1.903	0.057	1.78	0.539	1.446	0.148	0.983	3.223
CUBN	-0.153	0.296	-0.516	0.606	0.858	0.254	-0.557	0.577	0.481	1.533
CTXND1	-0.103	0.296	-0.347	0.728	0.902	0.267	-0.366	0.714	0.505	1.613
CTTN	0.473	0.302	1.566	0.117	1.605	0.485	1.248	0.212	0.888	2.902
CTSZ	0.671	0.306	2.193	0.028	1.955	0.598	1.598	0.11	1.074	3.561
CTSW	-0.729	0.31	-2.352	0.019	0.482	0.149	-3.463	0.001	0.263	0.885
CTSV	0.713	0.306	2.33	0.02	2.039	0.624	1.666	0.096	1.12	3.714
CTSS	-0.146	0.296	-0.494	0.622	0.864	0.256	-0.531	0.595	0.484	1.543
CTSO	-0.542	0.306	-1.772	0.076	0.582	0.178	-2.351	0.019	0.32	1.059
CTSL	0.589	0.306	1.927	0.054	1.802	0.551	1.456	0.145	0.99	3.28
CTSK	-0.596	0.302	-1.97	0.049	0.551	0.167	-2.693	0.007	0.305	0.997
CTSH	-0.096	0.296	-0.325	0.746	0.908	0.269	-0.341	0.733	0.509	1.623
CTSG	0.039	0.295	0.132	0.895	1.04	0.307	0.129	0.897	0.583	1.855
CTSF	-0.397	0.302	-1.312	0.189	0.672	0.203	-1.611	0.107	0.372	1.216
CTSE	0.241	0.296	0.813	0.416	1.272	0.377	0.723	0.47	0.712	2.273
CTSD	0.135	0.295	0.456	0.648	1.144	0.338	0.427	0.669	0.641	2.042

CTSC	0.324	0.298	1.09	0.276	1.383	0.412	0.931	0.352	0.772	2.479
CTSB	0.134	0.297	0.452	0.651	1.144	0.339	0.423	0.672	0.639	2.045
CTSA	0.02	0.296	0.068	0.946	1.02	0.302	0.067	0.946	0.571	1.822
CTRL	-0.117	0.296	-0.394	0.693	0.89	0.263	-0.418	0.676	0.498	1.59
CTRC	-0.185	0.298	-0.622	0.534	0.831	0.247	-0.684	0.494	0.464	1.489
CTRB2	-0.25	0.297	-0.844	0.399	0.779	0.231	-0.959	0.338	0.435	1.392
CTR9	0.005	0.296	0.018	0.986	1.005	0.297	0.018	0.986	0.563	1.794
CTPS2	0.849	0.315	2.696	0.007	2.337	0.736	1.817	0.069	1.261	4.332
CTPS1	0.151	0.295	0.511	0.61	1.163	0.343	0.474	0.635	0.652	2.075
CTNS	-0.391	0.299	-1.307	0.191	0.676	0.202	-1.6	0.11	0.377	1.215
CTNND2	-0.05	0.296	-0.17	0.865	0.951	0.281	-0.174	0.862	0.532	1.698
CTNNBL1	-0.158	0.296	-0.534	0.593	0.854	0.253	-0.579	0.563	0.478	1.525
CTNNBIP1	0.383	0.299	1.283	0.2	1.467	0.438	1.066	0.287	0.817	2.633
CTNNB1	0.001	0.295	0.002	0.998	1.001	0.296	0.002	0.998	0.561	1.785
CTNNAL1	-0.213	0.296	-0.72	0.472	0.808	0.239	-0.802	0.423	0.453	1.443
CTNNA3	-0.12	0.295	-0.406	0.685	0.887	0.262	-0.431	0.666	0.497	1.582
CTNNA2	-0.185	0.296	-0.624	0.532	0.831	0.246	-0.686	0.493	0.465	1.486
CTNNA1	0.056	0.295	0.19	0.849	1.058	0.312	0.185	0.853	0.593	1.886
CTLA4	0.094	0.295	0.318	0.751	1.098	0.324	0.303	0.762	0.616	1.96
CTIF	-0.558	0.3	-1.855	0.064	0.573	0.172	-2.484	0.013	0.318	1.032
CTH	0.056	0.295	0.191	0.848	1.058	0.313	0.186	0.853	0.593	1.888
CTF1	-0.579	0.306	-1.893	0.058	0.561	0.171	-2.564	0.01	0.308	1.021
CTDSPL	-1.118	0.336	-3.328	0.001	0.327	0.11	-6.128	0	0.169	0.632
CTDSP2	-0.524	0.302	-1.736	0.083	0.592	0.179	-2.281	0.023	0.328	1.07
CTDSP1	-0.368	0.297	-1.239	0.215	0.692	0.206	-1.498	0.134	0.386	1.239
CTDP1	-0.346	0.303	-1.14	0.254	0.708	0.215	-1.362	0.173	0.39	1.282
CTDNEP1	-0.119	0.296	-0.403	0.687	0.888	0.263	-0.428	0.669	0.497	1.585
CTCF	-0.244	0.298	-0.818	0.413	0.784	0.233	-0.926	0.354	0.437	1.405
CTC1	-0.108	0.295	-0.364	0.716	0.898	0.265	-0.384	0.701	0.503	1.602
CTC-338M12.4	0.239	0.296	0.808	0.419	1.27	0.376	0.718	0.473	0.711	2.27
CTBS	-0.105	0.296	-0.354	0.723	0.9	0.267	-0.373	0.709	0.504	1.61
CTBP2	0.059	0.295	0.198	0.843	1.06	0.313	0.193	0.847	0.594	1.891
CTBP1	-0.403	0.299	-1.349	0.177	0.668	0.2	-1.661	0.097	0.372	1.2
CTAGE11P	0.085	0.296	0.289	0.773	1.089	0.322	0.277	0.782	0.61	1.944
CTAGE1	-0.028	0.295	-0.096	0.924	0.972	0.287	-0.097	0.923	0.545	1.734
CTAG2	0.392	0.298	1.317	0.188	1.48	0.441	1.089	0.276	0.826	2.655
CT62	-0.295	0.303	-0.974	0.33	0.745	0.225	-1.133	0.257	0.411	1.348
CT55	0.131	0.296	0.441	0.659	1.139	0.338	0.413	0.68	0.638	2.036

CSTF3	0.423	0.303	1.395	0.163	1.527	0.463	1.137	0.255	0.842	2.767
CSTF2T	0.217	0.296	0.735	0.463	1.243	0.368	0.66	0.509	0.696	2.219
CSTF2	-0.108	0.295	-0.365	0.715	0.898	0.265	-0.386	0.7	0.503	1.601
CSTF1	0.584	0.306	1.909	0.056	1.793	0.548	1.446	0.148	0.984	3.264
CSTB	0.236	0.296	0.798	0.425	1.267	0.375	0.711	0.477	0.709	2.264
CSTA	-0.511	0.301	-1.695	0.09	0.6	0.181	-2.212	0.027	0.333	1.083
CST8	0.021	0.297	0.069	0.945	1.021	0.304	0.069	0.945	0.57	1.828
CST7	-0.419	0.298	-1.404	0.16	0.658	0.196	-1.743	0.081	0.367	1.181
CST6	-0.111	0.295	-0.377	0.706	0.895	0.264	-0.399	0.69	0.502	1.596
CST5	0.151	0.296	0.511	0.609	1.164	0.345	0.474	0.635	0.651	2.08
CST4	-0.162	0.297	-0.548	0.584	0.85	0.252	-0.595	0.552	0.475	1.52
CST3	-0.516	0.306	-1.686	0.092	0.597	0.183	-2.207	0.027	0.328	1.087
CST2	-0.504	0.307	-1.644	0.1	0.604	0.185	-2.138	0.033	0.331	1.102
CST1	-0.227	0.298	-0.763	0.445	0.797	0.237	-0.857	0.391	0.444	1.429
CSRP3	-0.001	0.295	-0.003	0.997	0.999	0.295	-0.003	0.997	0.56	1.781
CSRP2	0.238	0.298	0.799	0.424	1.268	0.377	0.711	0.477	0.708	2.272
CSRP1	0.159	0.296	0.536	0.592	1.172	0.347	0.496	0.62	0.656	2.094
CSRNP3	0.249	0.298	0.835	0.404	1.283	0.382	0.739	0.46	0.715	2.301
CSRNP2	-0.167	0.298	-0.561	0.575	0.846	0.252	-0.61	0.542	0.472	1.517
CSPP1	0.318	0.298	1.068	0.286	1.374	0.409	0.915	0.36	0.767	2.463
CSPG5	0.248	0.296	0.837	0.402	1.281	0.38	0.742	0.458	0.717	2.29
CSPG4P1Y	0.864	0.315	2.744	0.006	2.373	0.747	1.837	0.066	1.28	4.4
CSPG4	0.032	0.295	0.11	0.913	1.033	0.305	0.108	0.914	0.579	1.841
CSNK2B	0.27	0.298	0.906	0.365	1.309	0.39	0.794	0.427	0.731	2.346
CSNK2A2	0.506	0.3	1.688	0.091	1.659	0.497	1.325	0.185	0.922	2.986
CSNK2A1	0.218	0.296	0.735	0.462	1.243	0.368	0.661	0.509	0.696	2.221
CSNK1G3	-0.167	0.297	-0.563	0.573	0.846	0.251	-0.613	0.54	0.473	1.513
CSNK1G2	0.312	0.298	1.048	0.295	1.367	0.407	0.9	0.368	0.762	2.452
CSNK1G1	0.048	0.296	0.161	0.872	1.049	0.31	0.157	0.875	0.588	1.872
CSNK1E	0.64	0.306	2.095	0.036	1.897	0.58	1.547	0.122	1.042	3.453
CSNK1D	-0.107	0.295	-0.361	0.718	0.899	0.265	-0.381	0.703	0.504	1.603
CSNK1A1	-0.052	0.295	-0.176	0.86	0.949	0.28	-0.18	0.857	0.532	1.694
CSN3	0.147	0.296	0.498	0.619	1.159	0.343	0.463	0.643	0.649	2.068
CSN2	0.12	0.297	0.404	0.686	1.127	0.335	0.381	0.704	0.63	2.017
CSN1S1	-0.212	0.296	-0.716	0.474	0.809	0.24	-0.798	0.425	0.452	1.446
CSK	0.25	0.299	0.836	0.403	1.284	0.383	0.74	0.459	0.715	2.305
CSHL1	-0.746	0.315	-2.368	0.018	0.474	0.149	-3.518	0	0.256	0.879
CSH1	-0.686	0.31	-2.215	0.027	0.503	0.156	-3.183	0.001	0.274	0.924

CSGALNACT2	-0.009	0.295	-0.031	0.975	0.991	0.292	-0.031	0.975	0.556	1.766
CSGALNACT1	-0.229	0.296	-0.775	0.438	0.795	0.235	-0.871	0.384	0.445	1.42
CSF3R	-0.326	0.298	-1.094	0.274	0.722	0.215	-1.294	0.196	0.403	1.294
CSF3	-0.204	0.296	-0.691	0.49	0.815	0.241	-0.766	0.444	0.457	1.455
CSF2RB	-0.402	0.299	-1.347	0.178	0.669	0.2	-1.658	0.097	0.373	1.201
CSF2RA	-0.896	0.316	-2.836	0.005	0.408	0.129	-4.589	0	0.22	0.758
CSF2	0.177	0.296	0.6	0.549	1.194	0.353	0.549	0.583	0.669	2.131
CSF1R	-0.737	0.31	-2.376	0.018	0.478	0.148	-3.513	0	0.26	0.879
CSF1	-0.352	0.304	-1.158	0.247	0.703	0.214	-1.388	0.165	0.388	1.276
CSE1L	0.093	0.296	0.312	0.755	1.097	0.325	0.298	0.765	0.614	1.96
CSDE1	-0.748	0.31	-2.415	0.016	0.473	0.147	-3.594	0	0.258	0.868
CSDC2	-0.687	0.306	-2.247	0.025	0.503	0.154	-3.231	0.001	0.276	0.916
CSAD	-0.166	0.296	-0.56	0.575	0.847	0.25	-0.609	0.542	0.475	1.512
CS	-0.026	0.295	-0.088	0.93	0.974	0.288	-0.089	0.929	0.546	1.738
CRYZL1	0.13	0.298	0.438	0.661	1.139	0.339	0.411	0.681	0.636	2.041
CRYZ	-0.269	0.3	-0.895	0.371	0.764	0.229	-1.027	0.305	0.425	1.376
CRYM	0.227	0.298	0.764	0.445	1.255	0.374	0.683	0.495	0.7	2.249
CRYL1	0.525	0.302	1.736	0.083	1.69	0.511	1.35	0.177	0.934	3.057
CRYGD	-0.266	0.298	-0.895	0.371	0.766	0.228	-1.026	0.305	0.427	1.373
CRYGC	-0.384	0.3	-1.283	0.2	0.681	0.204	-1.564	0.118	0.378	1.225
CRYGB	-0.019	0.295	-0.063	0.95	0.982	0.29	-0.064	0.949	0.551	1.75
CRYGA	-0.139	0.296	-0.468	0.64	0.871	0.258	-0.502	0.616	0.487	1.556
CRYBG3	-0.103	0.295	-0.348	0.728	0.902	0.266	-0.366	0.714	0.506	1.61
CRYBG2	0.014	0.296	0.047	0.963	1.014	0.3	0.046	0.963	0.568	1.811
CRYBG1	-0.066	0.295	-0.223	0.824	0.936	0.276	-0.23	0.818	0.525	1.67
CRYBB3	-0.02	0.296	-0.068	0.946	0.98	0.29	-0.069	0.945	0.549	1.751
CRYBB2P1	0.077	0.296	0.259	0.796	1.08	0.319	0.249	0.803	0.605	1.927
CRYBB2	-0.19	0.296	-0.642	0.521	0.827	0.245	-0.707	0.479	0.463	1.477
CRYBB1	0.108	0.295	0.364	0.716	1.114	0.329	0.345	0.73	0.624	1.986
CRYBA4	0.083	0.297	0.281	0.779	1.087	0.323	0.269	0.788	0.607	1.945
CRYBA2	-0.073	0.295	-0.247	0.805	0.93	0.275	-0.256	0.798	0.521	1.659
CRYBA1	0.944	0.321	2.946	0.003	2.571	0.824	1.906	0.057	1.372	4.819
CRYAB	-0.422	0.299	-1.413	0.158	0.656	0.196	-1.757	0.079	0.365	1.177
CRYAA	-0.336	0.298	-1.127	0.26	0.715	0.213	-1.339	0.181	0.399	1.282
CRY2	-0.129	0.295	-0.438	0.661	0.879	0.26	-0.468	0.64	0.492	1.567
CRY1	-0.13	0.295	-0.44	0.66	0.878	0.259	-0.47	0.639	0.492	1.567
CRX	-0.203	0.298	-0.68	0.496	0.817	0.243	-0.754	0.451	0.455	1.464
CRTC3	-0.706	0.306	-2.307	0.021	0.493	0.151	-3.353	0.001	0.271	0.899

CRTC1	0.327	0.298	1.099	0.272	1.387	0.413	0.937	0.349	0.774	2.487
CRTAP	-0.233	0.298	-0.781	0.435	0.793	0.236	-0.879	0.379	0.442	1.421
CRTAM	-0.189	0.296	-0.64	0.522	0.827	0.245	-0.705	0.481	0.464	1.477
CRTAC1	0.068	0.296	0.231	0.817	1.071	0.317	0.223	0.823	0.6	1.911
CRP	-0.013	0.296	-0.044	0.965	0.987	0.292	-0.044	0.965	0.553	1.762
CROT	0.052	0.296	0.177	0.86	1.054	0.312	0.172	0.863	0.589	1.884
CROCCP3	0.15	0.296	0.505	0.613	1.162	0.344	0.469	0.639	0.65	2.076
CROCCP2	-0.151	0.296	-0.509	0.611	0.86	0.255	-0.55	0.582	0.481	1.537
CROCC	-0.52	0.302	-1.722	0.085	0.594	0.18	-2.258	0.024	0.329	1.075
CRNN	0.347	0.298	1.163	0.245	1.414	0.421	0.983	0.326	0.789	2.536
CRNKL1	-0.674	0.306	-2.199	0.028	0.51	0.156	-3.139	0.002	0.28	0.929
CRMP1	0.058	0.298	0.195	0.845	1.06	0.316	0.19	0.85	0.591	1.9
CRLF3	-0.24	0.297	-0.808	0.419	0.787	0.233	-0.914	0.361	0.44	1.407
CRLF2	-0.035	0.3	-0.116	0.907	0.966	0.29	-0.119	0.906	0.537	1.738
CRLF1	0.33	0.298	1.109	0.268	1.391	0.415	0.944	0.345	0.776	2.495
CRKL	-0.123	0.296	-0.415	0.678	0.884	0.262	-0.441	0.659	0.495	1.581
CRK	-0.782	0.315	-2.485	0.013	0.458	0.144	-3.768	0	0.247	0.848
CRISPLD2	-0.683	0.306	-2.234	0.025	0.505	0.154	-3.205	0.001	0.277	0.92
CRISP3	-0.41	0.3	-1.366	0.172	0.664	0.199	-1.688	0.091	0.369	1.195
CRISP2	0.051	0.295	0.173	0.862	1.053	0.311	0.169	0.866	0.59	1.878
CRISP1	0.13	0.297	0.438	0.661	1.139	0.338	0.411	0.681	0.637	2.037
CRIPT	0.089	0.296	0.302	0.762	1.094	0.324	0.289	0.772	0.612	1.953
CRIP2	0.051	0.296	0.172	0.864	1.052	0.311	0.168	0.867	0.589	1.878
CRIP1	0.051	0.295	0.171	0.864	1.052	0.311	0.167	0.867	0.589	1.877
CRIM1	-0.656	0.31	-2.116	0.034	0.519	0.161	-2.99	0.003	0.283	0.953
CRHR2	-0.656	0.306	-2.145	0.032	0.519	0.159	-3.031	0.002	0.285	0.945
CRHR1	0.432	0.303	1.427	0.154	1.54	0.466	1.159	0.247	0.851	2.787
CRHBP	0.192	0.299	0.642	0.521	1.211	0.362	0.585	0.559	0.675	2.175
CRH	-0.371	0.3	-1.239	0.215	0.69	0.207	-1.5	0.134	0.383	1.241
CREM	-0.54	0.304	-1.778	0.075	0.583	0.177	-2.357	0.018	0.321	1.057
CRELD2	-0.207	0.298	-0.695	0.487	0.813	0.242	-0.772	0.44	0.453	1.458
CRELD1	-0.641	0.31	-2.068	0.039	0.527	0.163	-2.897	0.004	0.287	0.967
CREG1	-0.135	0.297	-0.456	0.648	0.873	0.259	-0.488	0.625	0.488	1.562
CREBZF	0.303	0.298	1.019	0.308	1.355	0.403	0.879	0.379	0.756	2.428
CREBL2	-0.575	0.306	-1.88	0.06	0.563	0.172	-2.541	0.011	0.309	1.025
CREBBP	0.255	0.298	0.858	0.391	1.291	0.384	0.757	0.449	0.72	2.313
CREB3L2	-0.552	0.301	-1.835	0.066	0.576	0.173	-2.45	0.014	0.319	1.038
CREB3L1	-0.497	0.3	-1.657	0.098	0.608	0.182	-2.146	0.032	0.338	1.095

CREB3	0.022	0.295	0.075	0.94	1.022	0.302	0.074	0.941	0.573	1.823
CREB1	0.147	0.296	0.498	0.619	1.159	0.343	0.463	0.644	0.649	2.069
CRCT1	-0.021	0.295	-0.071	0.944	0.979	0.289	-0.071	0.943	0.549	1.746
CRCP	0.569	0.306	1.861	0.063	1.766	0.54	1.419	0.156	0.97	3.216
CRBN	-0.551	0.306	-1.801	0.072	0.577	0.176	-2.402	0.016	0.317	1.05
CRB1	-0.411	0.3	-1.373	0.17	0.663	0.199	-1.699	0.089	0.368	1.192
CRAT	0.218	0.298	0.731	0.465	1.243	0.37	0.657	0.511	0.694	2.228
CRADD	-0.324	0.303	-1.066	0.286	0.724	0.22	-1.259	0.208	0.399	1.312
CRACDL	-0.05	0.296	-0.17	0.865	0.951	0.281	-0.174	0.862	0.533	1.698
CRABP2	0.039	0.296	0.131	0.895	1.04	0.308	0.129	0.897	0.582	1.857
CRABP1	0.103	0.295	0.35	0.726	1.109	0.327	0.333	0.739	0.622	1.978
CR2	-0.395	0.299	-1.322	0.186	0.674	0.201	-1.621	0.105	0.375	1.21
CR1	0.442	0.303	1.46	0.144	1.555	0.471	1.18	0.238	0.859	2.815
CPVL	-0.243	0.297	-0.818	0.413	0.784	0.233	-0.926	0.354	0.439	1.403
CPTP	0.542	0.302	1.794	0.073	1.719	0.52	1.385	0.166	0.951	3.109
CPT2	-0.47	0.306	-1.535	0.125	0.625	0.191	-1.961	0.05	0.343	1.139
CPT1A	0.162	0.295	0.549	0.583	1.176	0.347	0.507	0.612	0.659	2.098
CPSF7	0.65	0.317	2.053	0.04	1.915	0.606	1.51	0.131	1.03	3.562
CPSF6	0.767	0.315	2.434	0.015	2.152	0.678	1.7	0.089	1.161	3.991
CPSF4	0.273	0.299	0.913	0.361	1.314	0.392	0.799	0.424	0.731	2.359
CPSF1	-0.574	0.31	-1.852	0.064	0.563	0.175	-2.503	0.012	0.307	1.034
CPS1-IT1	0.732	0.31	2.362	0.018	2.079	0.644	1.675	0.094	1.133	3.815
CPS1	0.121	0.295	0.41	0.682	1.129	0.333	0.386	0.699	0.633	2.014
CPQ	-0.696	0.31	-2.246	0.025	0.499	0.154	-3.246	0.001	0.272	0.915
CPPED1	0.423	0.298	1.419	0.156	1.527	0.456	1.157	0.247	0.851	2.741
CPOX	-0.295	0.298	-0.993	0.321	0.744	0.221	-1.155	0.248	0.415	1.333
CPNE7	0.681	0.306	2.225	0.026	1.977	0.605	1.613	0.107	1.085	3.603
CPNE6	0.064	0.296	0.216	0.829	1.066	0.316	0.209	0.835	0.597	1.904
CPNE3	-0.472	0.303	-1.56	0.119	0.624	0.189	-1.994	0.046	0.345	1.129
CPNE1	-0.455	0.302	-1.504	0.133	0.635	0.192	-1.904	0.057	0.351	1.148
CPN2	0.209	0.296	0.707	0.48	1.233	0.365	0.638	0.524	0.69	2.204
CPN1	0.415	0.3	1.382	0.167	1.514	0.454	1.132	0.258	0.841	2.725
CPM	-0.261	0.298	-0.878	0.38	0.77	0.229	-1.004	0.316	0.43	1.38
CPLX3	0.62	0.306	2.027	0.043	1.858	0.568	1.51	0.131	1.021	3.384
CPLX2	0.152	0.295	0.513	0.608	1.164	0.344	0.476	0.634	0.652	2.076
CPLANE2	0.245	0.298	0.823	0.41	1.278	0.381	0.73	0.465	0.713	2.293
CPLANE1	0.055	0.295	0.185	0.853	1.056	0.312	0.18	0.857	0.592	1.884
CPED1	-0.386	0.3	-1.289	0.198	0.68	0.204	-1.573	0.116	0.378	1.223

CPEB3	-0.569	0.306	-1.858	0.063	0.566	0.173	-2.503	0.012	0.311	1.032
CPEB1	0.552	0.303	1.823	0.068	1.736	0.526	1.401	0.161	0.959	3.142
CPE	-0.47	0.3	-1.568	0.117	0.625	0.187	-2.002	0.045	0.347	1.125
CPD	-0.182	0.297	-0.613	0.54	0.834	0.247	-0.672	0.502	0.466	1.492
CPB2	-0.165	0.296	-0.555	0.579	0.848	0.251	-0.604	0.546	0.475	1.516
CPB1	0.066	0.295	0.223	0.824	1.068	0.315	0.216	0.829	0.599	1.904
CPA4	-0.182	0.297	-0.612	0.54	0.834	0.247	-0.672	0.502	0.466	1.491
CPA3	-0.492	0.307	-1.604	0.109	0.612	0.187	-2.071	0.038	0.335	1.115
CPA2	0.071	0.297	0.24	0.81	1.074	0.319	0.232	0.817	0.6	1.922
CPA1	0.234	0.297	0.789	0.43	1.264	0.375	0.703	0.482	0.706	2.26
CP	0.172	0.295	0.584	0.559	1.188	0.351	0.536	0.592	0.666	2.12
COX8A	0.012	0.295	0.04	0.968	1.012	0.299	0.04	0.968	0.567	1.805
COX7C	0.25	0.298	0.836	0.403	1.283	0.383	0.74	0.459	0.715	2.303
COX7B	0.504	0.303	1.664	0.096	1.655	0.501	1.308	0.191	0.914	2.994
COX7A2L	0.11	0.295	0.371	0.711	1.116	0.33	0.351	0.725	0.625	1.991
COX7A2	0.57	0.303	1.884	0.06	1.768	0.535	1.436	0.151	0.977	3.2
COX7A1	-0.287	0.298	-0.964	0.335	0.751	0.223	-1.117	0.264	0.419	1.345
COX6C	-0.016	0.296	-0.054	0.957	0.984	0.291	-0.054	0.957	0.552	1.756
COX6B1	0.739	0.306	2.414	0.016	2.094	0.641	1.706	0.088	1.149	3.817
COX6A2	0.152	0.295	0.514	0.607	1.164	0.344	0.477	0.634	0.652	2.077
COX6A1	0.409	0.298	1.373	0.17	1.506	0.449	1.127	0.26	0.84	2.7
COX5B	0.547	0.306	1.791	0.073	1.729	0.528	1.38	0.168	0.95	3.146
COX5A	0.728	0.31	2.35	0.019	2.07	0.641	1.67	0.095	1.128	3.799
COX4I1	0.669	0.306	2.185	0.029	1.952	0.597	1.593	0.111	1.071	3.556
COX15	-0.244	0.297	-0.823	0.41	0.783	0.233	-0.933	0.351	0.438	1.401
COX11	0.133	0.296	0.449	0.653	1.142	0.338	0.421	0.674	0.64	2.038
COX10	0.161	0.295	0.545	0.586	1.175	0.347	0.503	0.615	0.658	2.096
CORT	0.475	0.3	1.584	0.113	1.609	0.483	1.261	0.207	0.893	2.897
CORO7	0.44	0.298	1.477	0.14	1.553	0.463	1.195	0.232	0.866	2.786
CORO2B	-0.437	0.298	-1.465	0.143	0.646	0.193	-1.837	0.066	0.36	1.159
CORO2A	-0.297	0.3	-0.99	0.322	0.743	0.223	-1.153	0.249	0.413	1.338
CORO1C	0.397	0.298	1.331	0.183	1.487	0.443	1.099	0.272	0.829	2.667
CORO1B	-0.51	0.302	-1.689	0.091	0.6	0.181	-2.203	0.028	0.332	1.085
CORO1A	-0.588	0.303	-1.939	0.052	0.556	0.168	-2.639	0.008	0.307	1.006
CORIN	-0.526	0.306	-1.722	0.085	0.591	0.181	-2.266	0.023	0.325	1.075
COQ9	0.348	0.298	1.169	0.242	1.416	0.421	0.987	0.323	0.79	2.537
COQ8B	0.194	0.296	0.658	0.511	1.215	0.359	0.598	0.55	0.68	2.169
COQ8A	0.164	0.295	0.555	0.579	1.178	0.348	0.512	0.609	0.66	2.102

COQ7	0.56	0.303	1.851	0.064	1.751	0.53	1.417	0.156	0.968	3.169
COQ6	-0.202	0.298	-0.679	0.497	0.817	0.243	-0.752	0.452	0.456	1.464
COQ4	-0.14	0.3	-0.465	0.642	0.87	0.261	-0.499	0.618	0.483	1.566
COQ3	0.637	0.306	2.084	0.037	1.891	0.578	1.541	0.123	1.039	3.442
COQ2	0.046	0.295	0.157	0.875	1.048	0.31	0.154	0.878	0.587	1.869
COQ10B	0.41	0.298	1.376	0.169	1.506	0.448	1.129	0.259	0.84	2.7
COPZ2	-0.484	0.302	-1.603	0.109	0.616	0.186	-2.062	0.039	0.341	1.114
COPZ1	-0.457	0.302	-1.513	0.13	0.633	0.191	-1.918	0.055	0.35	1.145
COPS8	0.306	0.298	1.028	0.304	1.358	0.404	0.886	0.376	0.758	2.433
COPS7B	-0.523	0.303	-1.728	0.084	0.593	0.179	-2.269	0.023	0.328	1.073
COPS7A	-0.324	0.298	-1.089	0.276	0.723	0.215	-1.286	0.198	0.404	1.296
COPS6	-0.001	0.297	-0.004	0.997	0.999	0.297	-0.004	0.997	0.558	1.788
COPS5	0.15	0.295	0.508	0.612	1.162	0.343	0.472	0.637	0.651	2.073
COPS4	0.424	0.298	1.423	0.155	1.528	0.456	1.16	0.246	0.852	2.741
COPS3	0.536	0.302	1.775	0.076	1.71	0.517	1.374	0.17	0.946	3.092
COPS2	-0.126	0.298	-0.422	0.673	0.882	0.263	-0.45	0.653	0.492	1.582
COPG1	0.134	0.298	0.45	0.653	1.143	0.341	0.421	0.674	0.638	2.05
COPE	-0.208	0.296	-0.702	0.483	0.812	0.241	-0.78	0.435	0.455	1.451
COPB2	0.04	0.295	0.135	0.893	1.041	0.307	0.132	0.895	0.583	1.857
COPB1	0.51	0.302	1.685	0.092	1.665	0.503	1.321	0.187	0.92	3.011
COPA	-0.286	0.298	-0.962	0.336	0.751	0.224	-1.114	0.265	0.419	1.346
COMT	-0.1	0.296	-0.337	0.736	0.905	0.268	-0.354	0.723	0.507	1.616
COMP	-0.585	0.303	-1.933	0.053	0.557	0.169	-2.627	0.009	0.308	1.008
COMMD9	-0.461	0.306	-1.507	0.132	0.631	0.193	-1.915	0.055	0.346	1.148
COMMD8	0.171	0.3	0.57	0.569	1.186	0.356	0.524	0.6	0.659	2.135
COMMD4	0.003	0.295	0.01	0.992	1.003	0.296	0.01	0.992	0.562	1.79
COMMD3	-0.241	0.301	-0.8	0.424	0.786	0.237	-0.904	0.366	0.435	1.419
COMMD10	-0.312	0.298	-1.05	0.294	0.732	0.218	-1.232	0.218	0.408	1.311
COLQ	-0.383	0.298	-1.285	0.199	0.682	0.203	-1.566	0.117	0.38	1.223
COLGALT2	-0.077	0.302	-0.254	0.8	0.926	0.279	-0.264	0.792	0.513	1.673
COLGALT1	0.904	0.321	2.819	0.005	2.469	0.792	1.856	0.064	1.317	4.628
COLEC12	-0.674	0.31	-2.176	0.03	0.51	0.158	-3.107	0.002	0.278	0.935
COLEC11	0.079	0.297	0.265	0.791	1.082	0.321	0.255	0.799	0.605	1.935
COLEC10	-0.368	0.299	-1.229	0.219	0.692	0.207	-1.486	0.137	0.385	1.245
COL9A3	0.024	0.296	0.082	0.934	1.025	0.304	0.081	0.935	0.573	1.831
COL9A2	0.52	0.302	1.719	0.086	1.682	0.508	1.341	0.18	0.93	3.042
COL9A1	-0.237	0.296	-0.799	0.424	0.789	0.234	-0.901	0.367	0.441	1.411
COL8A2	-0.574	0.302	-1.898	0.058	0.563	0.17	-2.563	0.01	0.312	1.019

COL8A1	-0.486	0.302	-1.608	0.108	0.615	0.186	-2.07	0.038	0.34	1.112
COL7A1	-0.061	0.295	-0.206	0.836	0.941	0.278	-0.213	0.831	0.528	1.678
COL6A3	-0.084	0.295	-0.284	0.776	0.92	0.271	-0.296	0.767	0.516	1.64
COL6A2	-0.807	0.31	-2.601	0.009	0.446	0.138	-4	0	0.243	0.82
COL6A1	-0.422	0.298	-1.417	0.157	0.656	0.195	-1.762	0.078	0.366	1.176
COL5A3	-0.004	0.295	-0.015	0.988	0.996	0.294	-0.015	0.988	0.558	1.776
COL5A2	-0.494	0.3	-1.647	0.1	0.61	0.183	-2.129	0.033	0.339	1.098
COL5A1	-0.543	0.302	-1.797	0.072	0.581	0.176	-2.386	0.017	0.321	1.051
COL4A6	-0.107	0.297	-0.359	0.72	0.899	0.267	-0.379	0.705	0.502	1.608
COL4A5	-0.311	0.3	-1.037	0.3	0.732	0.22	-1.216	0.224	0.406	1.32
COL4A4	-0.067	0.296	-0.226	0.821	0.935	0.276	-0.234	0.815	0.524	1.669
COL4A3	0.228	0.302	0.753	0.452	1.255	0.379	0.673	0.501	0.694	2.27
COL4A2	-0.217	0.297	-0.731	0.465	0.805	0.239	-0.817	0.414	0.449	1.441
COL4A1	-0.354	0.298	-1.19	0.234	0.702	0.209	-1.428	0.153	0.391	1.258
COL3A1	-0.385	0.298	-1.293	0.196	0.681	0.203	-1.576	0.115	0.38	1.22
COL2A1	-0.107	0.296	-0.362	0.717	0.898	0.266	-0.382	0.702	0.503	1.604
COL21A1	-0.277	0.296	-0.937	0.349	0.758	0.224	-1.079	0.28	0.424	1.354
COL1A2	-0.352	0.298	-1.184	0.236	0.703	0.209	-1.419	0.156	0.392	1.26
COL1A1	-0.474	0.302	-1.568	0.117	0.623	0.188	-2.006	0.045	0.344	1.126
COL19A1	-0.21	0.296	-0.709	0.478	0.81	0.24	-0.789	0.43	0.453	1.449
COL18A1	-0.152	0.295	-0.513	0.608	0.859	0.254	-0.554	0.579	0.482	1.533
COL17A1	-0.576	0.302	-1.905	0.057	0.562	0.17	-2.575	0.01	0.311	1.017
COL16A1	-0.596	0.306	-1.949	0.051	0.551	0.168	-2.666	0.008	0.302	1.003
COL15A1	-0.644	0.304	-2.12	0.034	0.525	0.16	-2.977	0.003	0.29	0.952
COL14A1	-0.608	0.303	-2.011	0.044	0.544	0.165	-2.768	0.006	0.301	0.985
COL13A1	-0.142	0.296	-0.479	0.632	0.868	0.257	-0.515	0.607	0.486	1.55
COL11A2	0.056	0.295	0.189	0.85	1.057	0.312	0.184	0.854	0.593	1.886
COL11A1	-0.176	0.296	-0.594	0.552	0.839	0.248	-0.65	0.516	0.469	1.498
COL10A1	-0.424	0.3	-1.414	0.157	0.655	0.196	-1.761	0.078	0.364	1.178
COIL	0.138	0.297	0.463	0.643	1.148	0.341	0.433	0.665	0.641	2.055
COG7	-0.487	0.302	-1.61	0.107	0.615	0.186	-2.074	0.038	0.34	1.112
COG5	-0.235	0.298	-0.791	0.429	0.79	0.235	-0.892	0.372	0.441	1.416
COG4	0.84	0.315	2.668	0.008	2.316	0.729	1.805	0.071	1.25	4.292
COG2	-0.424	0.3	-1.416	0.157	0.654	0.196	-1.764	0.078	0.364	1.177
COCH	-0.19	0.296	-0.642	0.521	0.827	0.245	-0.707	0.479	0.463	1.476
COBLL1	0.005	0.295	0.016	0.988	1.005	0.297	0.015	0.988	0.563	1.792
COBL	0.162	0.296	0.548	0.584	1.176	0.348	0.506	0.613	0.658	2.102
COASY	0.259	0.296	0.872	0.383	1.295	0.384	0.769	0.442	0.724	2.315

COA7	0.829	0.31	2.67	0.008	2.291	0.711	1.815	0.07	1.247	4.211
COA4	0.254	0.296	0.859	0.391	1.29	0.382	0.758	0.448	0.721	2.306
COA3	-0.137	0.297	-0.462	0.644	0.872	0.259	-0.495	0.621	0.487	1.56
COA1	0.95	0.316	3.012	0.003	2.586	0.816	1.944	0.052	1.394	4.8
CNTRL	0.325	0.297	1.093	0.275	1.384	0.412	0.933	0.351	0.773	2.48
CNTNAP3P2	0.334	0.296	1.127	0.26	1.396	0.414	0.958	0.338	0.781	2.495
CNTNAP2	-0.217	0.297	-0.73	0.465	0.805	0.239	-0.815	0.415	0.45	1.441
CNTNAP1	0.112	0.297	0.379	0.705	1.119	0.332	0.359	0.72	0.626	2.001
CNTN6	0.221	0.296	0.746	0.456	1.247	0.369	0.669	0.503	0.698	2.229
CNTN5	0.286	0.3	0.952	0.341	1.331	0.4	0.828	0.408	0.739	2.397
CNTN2	-0.072	0.296	-0.242	0.809	0.931	0.276	-0.25	0.802	0.521	1.663
CNTN1	-0.367	0.298	-1.234	0.217	0.693	0.206	-1.491	0.136	0.386	1.241
CNTLN	-0.19	0.296	-0.64	0.522	0.827	0.245	-0.705	0.481	0.463	1.478
CNTFR	-0.045	0.295	-0.154	0.878	0.956	0.282	-0.158	0.875	0.536	1.704
CNTF	0.408	0.297	1.372	0.17	1.504	0.447	1.126	0.26	0.839	2.693
CNR2	-0.313	0.298	-1.052	0.293	0.731	0.218	-1.235	0.217	0.408	1.31
CNR1	-0.701	0.307	-2.285	0.022	0.496	0.152	-3.311	0.001	0.272	0.905
CNPY4	0.249	0.296	0.843	0.399	1.283	0.38	0.746	0.456	0.719	2.292
CNPY3	0.331	0.298	1.111	0.267	1.392	0.415	0.946	0.344	0.776	2.497
CNPY2	0.08	0.296	0.272	0.786	1.084	0.32	0.261	0.794	0.607	1.934
CNPPD1	0.348	0.3	1.163	0.245	1.417	0.424	0.982	0.326	0.788	2.549
CNP	0.593	0.31	1.913	0.056	1.81	0.561	1.443	0.149	0.985	3.323
CNOT9	-0.093	0.296	-0.314	0.753	0.911	0.269	-0.329	0.742	0.511	1.626
CNOT8	-0.523	0.306	-1.711	0.087	0.593	0.181	-2.248	0.025	0.326	1.079
CNOT7	0.515	0.301	1.711	0.087	1.674	0.504	1.337	0.181	0.928	3.02
CNOT6	0.182	0.296	0.614	0.539	1.199	0.355	0.561	0.575	0.671	2.141
CNOT4	0.114	0.296	0.384	0.701	1.121	0.332	0.363	0.717	0.627	2.003
CNOT3	0.026	0.295	0.089	0.929	1.027	0.303	0.088	0.93	0.575	1.831
CNOT2	0.218	0.296	0.735	0.462	1.243	0.368	0.661	0.509	0.696	2.223
CNOT1	0.309	0.298	1.037	0.3	1.362	0.405	0.892	0.372	0.76	2.441
CNNM4	0.057	0.295	0.194	0.846	1.059	0.313	0.189	0.85	0.594	1.889
CNNM3	-0.331	0.298	-1.112	0.266	0.718	0.214	-1.318	0.187	0.401	1.287
CNNM2	-0.44	0.302	-1.455	0.146	0.644	0.195	-1.827	0.068	0.356	1.165
CNNM1	0.03	0.295	0.101	0.919	1.03	0.304	0.1	0.921	0.578	1.837
CNN3	-0.093	0.295	-0.315	0.753	0.911	0.269	-0.33	0.742	0.511	1.626
CNN2	-0.586	0.302	-1.939	0.053	0.556	0.168	-2.636	0.008	0.308	1.006
CNN1	-0.452	0.298	-1.515	0.13	0.636	0.19	-1.915	0.055	0.355	1.142
CNMD	0.289	0.298	0.972	0.331	1.335	0.397	0.844	0.399	0.745	2.393

CNKSR2	0.104	0.297	0.35	0.726	1.11	0.33	0.332	0.74	0.62	1.986
CNKSR1	0.581	0.303	1.915	0.055	1.787	0.542	1.453	0.146	0.986	3.238
CNIH4	0.414	0.3	1.383	0.167	1.513	0.453	1.133	0.257	0.841	2.722
CNIH3	-0.207	0.295	-0.702	0.483	0.813	0.24	-0.78	0.435	0.455	1.45
CNIH1	0.347	0.3	1.157	0.247	1.414	0.424	0.978	0.328	0.786	2.545
CNGB3	-0.175	0.296	-0.591	0.555	0.84	0.248	-0.645	0.519	0.471	1.499
CNGB1	-0.174	0.295	-0.589	0.556	0.84	0.248	-0.643	0.52	0.471	1.499
CNGA3	-0.087	0.295	-0.293	0.769	0.917	0.271	-0.306	0.76	0.514	1.636
CNGA1	0.285	0.298	0.956	0.339	1.329	0.396	0.832	0.405	0.742	2.383
CNDP2	0.201	0.296	0.68	0.497	1.223	0.362	0.616	0.538	0.684	2.185
CNBP	-0.197	0.296	-0.665	0.506	0.821	0.243	-0.735	0.462	0.459	1.468
CMTR1	-0.126	0.296	-0.427	0.669	0.881	0.261	-0.455	0.649	0.493	1.574
CMTM6	-0.29	0.3	-0.967	0.334	0.748	0.225	-1.122	0.262	0.415	1.347
CMPK1	-0.33	0.298	-1.106	0.269	0.719	0.214	-1.31	0.19	0.401	1.29
CMKLR1	0.099	0.295	0.336	0.737	1.104	0.326	0.32	0.749	0.619	1.97
CMC4	0.569	0.304	1.871	0.061	1.766	0.537	1.427	0.154	0.973	3.204
CMC2	1.068	0.328	3.259	0.001	2.909	0.953	2.003	0.045	1.531	5.529
CMAS	0.124	0.296	0.42	0.675	1.132	0.335	0.395	0.693	0.634	2.021
CMAHP	-0.483	0.301	-1.606	0.108	0.617	0.185	-2.065	0.039	0.342	1.112
CMA1	-0.083	0.296	-0.281	0.778	0.92	0.272	-0.294	0.769	0.515	1.643
CLUL1	-0.075	0.295	-0.254	0.8	0.928	0.274	-0.263	0.792	0.52	1.656
CLUHP3	0.128	0.296	0.431	0.667	1.136	0.337	0.404	0.686	0.636	2.03
CLUH	0.581	0.306	1.902	0.057	1.788	0.546	1.442	0.149	0.982	3.255
CLUAP1	-0.52	0.302	-1.72	0.085	0.595	0.18	-2.256	0.024	0.329	1.075
CLU	-0.95	0.321	-2.964	0.003	0.387	0.124	-4.949	0	0.206	0.725
CLTCL1	-0.288	0.298	-0.968	0.333	0.75	0.223	-1.122	0.262	0.418	1.344
CLTC	-0.154	0.296	-0.521	0.602	0.857	0.254	-0.564	0.573	0.48	1.531
CLTB	-0.064	0.297	-0.217	0.828	0.938	0.278	-0.224	0.823	0.524	1.678
CLTA	0.083	0.298	0.279	0.78	1.087	0.324	0.268	0.789	0.606	1.948
CLSTN3	0.399	0.3	1.331	0.183	1.49	0.446	1.098	0.272	0.828	2.68
CLSTN2	-0.058	0.302	-0.193	0.847	0.943	0.285	-0.199	0.842	0.522	1.704
CLSTN1	-0.505	0.306	-1.65	0.099	0.604	0.185	-2.146	0.032	0.332	1.1
CLSPN	0.234	0.296	0.789	0.43	1.263	0.374	0.704	0.482	0.707	2.257
CLPX	0.42	0.298	1.41	0.159	1.522	0.454	1.151	0.25	0.849	2.73
CLPTM1	-0.526	0.306	-1.721	0.085	0.591	0.181	-2.264	0.024	0.325	1.076
CLPS	0.114	0.295	0.385	0.7	1.12	0.331	0.364	0.716	0.628	1.999
CLPP	0.166	0.296	0.562	0.574	1.181	0.35	0.518	0.604	0.661	2.11
CLPB	0.641	0.306	2.095	0.036	1.899	0.581	1.547	0.122	1.042	3.459

CLP1	0.718	0.31	2.318	0.02	2.05	0.635	1.654	0.098	1.117	3.762
CLOCK	-0.187	0.296	-0.631	0.528	0.83	0.246	-0.693	0.488	0.464	1.482
CLNS1A	-0.063	0.295	-0.213	0.831	0.939	0.277	-0.22	0.826	0.526	1.675
CLN8	-0.349	0.299	-1.167	0.243	0.705	0.211	-1.397	0.162	0.392	1.268
CLN6	0.422	0.3	1.409	0.159	1.525	0.457	1.149	0.25	0.848	2.744
CLN5	-0.259	0.297	-0.871	0.384	0.772	0.229	-0.995	0.32	0.431	1.381
CLN3	-0.427	0.3	-1.423	0.155	0.653	0.196	-1.775	0.076	0.363	1.174
CLMN	-0.704	0.306	-2.303	0.021	0.495	0.151	-3.342	0.001	0.272	0.901
CLK4	-0.166	0.295	-0.561	0.575	0.847	0.25	-0.61	0.542	0.475	1.512
CLK3	-0.324	0.298	-1.085	0.278	0.724	0.216	-1.281	0.2	0.403	1.298
CLK2	-0.155	0.295	-0.524	0.6	0.857	0.253	-0.567	0.571	0.48	1.528
CLK1	-0.403	0.299	-1.35	0.177	0.668	0.2	-1.663	0.096	0.372	1.2
CLIP4	0.046	0.295	0.154	0.877	1.047	0.309	0.151	0.88	0.587	1.867
CLIP3	0.057	0.298	0.19	0.849	1.058	0.315	0.185	0.853	0.591	1.896
CLIP2	-0.205	0.297	-0.693	0.489	0.814	0.242	-0.769	0.442	0.455	1.456
CLIP1	-0.327	0.299	-1.096	0.273	0.721	0.215	-1.296	0.195	0.402	1.294
CLINT1	-0.307	0.298	-1.033	0.301	0.735	0.219	-1.21	0.226	0.41	1.318
CLIC5	-0.169	0.299	-0.564	0.573	0.845	0.253	-0.615	0.539	0.47	1.518
CLIC4	-0.308	0.298	-1.033	0.302	0.735	0.219	-1.21	0.226	0.41	1.318
CLIC3	-0.089	0.295	-0.302	0.763	0.915	0.27	-0.316	0.752	0.513	1.632
CLIC2	-0.125	0.295	-0.423	0.672	0.883	0.261	-0.451	0.652	0.495	1.575
CLIC1	0.662	0.306	2.16	0.031	1.938	0.594	1.58	0.114	1.063	3.533
CLGN	-0.197	0.298	-0.659	0.51	0.822	0.245	-0.729	0.466	0.458	1.473
CLEC7A	0.177	0.296	0.599	0.549	1.194	0.353	0.549	0.583	0.669	2.131
CLEC5A	0.24	0.296	0.809	0.418	1.271	0.377	0.719	0.472	0.711	2.272
CLEC4M	-0.084	0.295	-0.285	0.776	0.919	0.272	-0.297	0.766	0.515	1.64
CLEC4E	0.169	0.295	0.57	0.568	1.184	0.35	0.525	0.6	0.663	2.112
CLEC4A	-0.316	0.3	-1.055	0.291	0.729	0.218	-1.241	0.215	0.405	1.311
CLEC2D	-0.34	0.298	-1.143	0.253	0.712	0.212	-1.362	0.173	0.397	1.275
CLEC2B	-0.896	0.311	-2.882	0.004	0.408	0.127	-4.663	0	0.222	0.751
CLEC1B	-0.2	0.296	-0.674	0.5	0.819	0.243	-0.746	0.456	0.458	1.464
CLEC1A	0.012	0.295	0.04	0.968	1.012	0.298	0.04	0.968	0.568	1.804
CLEC16A	0.114	0.297	0.384	0.701	1.121	0.332	0.363	0.717	0.627	2.004
CLEC11A	-0.294	0.297	-0.989	0.323	0.745	0.222	-1.15	0.25	0.416	1.335
CLEC10A	-0.637	0.306	-2.085	0.037	0.529	0.162	-2.916	0.004	0.29	0.962
CLDND1	-0.501	0.302	-1.657	0.097	0.606	0.183	-2.152	0.031	0.335	1.096
CLDN9	0.192	0.296	0.648	0.517	1.211	0.358	0.59	0.555	0.678	2.163
CLDN8	-0.489	0.301	-1.628	0.104	0.613	0.184	-2.099	0.036	0.34	1.105

CLDN7	0.258	0.296	0.872	0.383	1.295	0.383	0.768	0.442	0.725	2.313
CLDN6	-0.114	0.296	-0.386	0.7	0.892	0.264	-0.409	0.683	0.499	1.594
CLDN5	-0.066	0.295	-0.224	0.822	0.936	0.277	-0.232	0.817	0.524	1.67
CLDN4	0.055	0.295	0.185	0.853	1.056	0.312	0.18	0.857	0.592	1.883
CLDN3	0.175	0.296	0.589	0.556	1.191	0.353	0.54	0.589	0.666	2.129
CLDN18	0.187	0.296	0.63	0.529	1.205	0.357	0.575	0.565	0.674	2.154
CLDN17	-0.112	0.298	-0.375	0.707	0.894	0.266	-0.397	0.691	0.499	1.603
CLDN16	0.024	0.296	0.083	0.934	1.025	0.303	0.082	0.935	0.574	1.829
CLDN15	0.432	0.3	1.442	0.149	1.541	0.462	1.171	0.242	0.856	2.773
CLDN14	0.385	0.298	1.293	0.196	1.47	0.438	1.073	0.283	0.82	2.635
CLDN11	-0.706	0.309	-2.285	0.022	0.494	0.152	-3.319	0.001	0.27	0.905
CLDN10	0.082	0.295	0.279	0.78	1.086	0.321	0.268	0.789	0.609	1.937
CLDN1	0.283	0.296	0.956	0.339	1.327	0.393	0.832	0.405	0.743	2.373
CLCNKB	0.252	0.296	0.852	0.394	1.287	0.381	0.753	0.451	0.72	2.3
CLCN7	-0.193	0.296	-0.653	0.514	0.824	0.244	-0.72	0.471	0.461	1.472
CLCN6	-0.149	0.296	-0.503	0.615	0.862	0.255	-0.542	0.588	0.482	1.54
CLCN5	0.207	0.298	0.694	0.488	1.23	0.367	0.627	0.531	0.686	2.206
CLCN4	0.264	0.298	0.888	0.375	1.303	0.388	0.78	0.435	0.727	2.335
CLCN3	-0.143	0.296	-0.482	0.63	0.867	0.257	-0.518	0.604	0.485	1.549
CLCN2	0.868	0.31	2.797	0.005	2.381	0.739	1.87	0.061	1.297	4.374
CLCN1	0.195	0.298	0.653	0.514	1.215	0.363	0.593	0.553	0.677	2.181
CLCF1	0.201	0.296	0.679	0.497	1.223	0.362	0.615	0.539	0.684	2.186
CLCC1	0.303	0.3	1.011	0.312	1.354	0.406	0.872	0.383	0.753	2.436
CLCA4	-0.233	0.297	-0.786	0.432	0.792	0.235	-0.885	0.376	0.442	1.417
CLCA3P	1.108	0.329	3.37	0.001	3.029	0.996	2.037	0.042	1.59	5.77
CLCA2	-0.253	0.297	-0.852	0.394	0.777	0.23	-0.969	0.333	0.434	1.389
CLCA1	0.53	0.306	1.732	0.083	1.699	0.519	1.345	0.179	0.933	3.093
CLC	-0.369	0.299	-1.232	0.218	0.692	0.207	-1.49	0.136	0.385	1.243
CLBA1	0.234	0.297	0.788	0.431	1.263	0.375	0.702	0.482	0.706	2.259
CLASRP	-0.221	0.297	-0.743	0.457	0.802	0.238	-0.832	0.405	0.448	1.435
CLASP2	-0.464	0.302	-1.534	0.125	0.629	0.19	-1.952	0.051	0.348	1.137
CLASP1	0.455	0.302	1.507	0.132	1.577	0.477	1.21	0.226	0.872	2.851
CKS2	1.351	0.346	3.905	0	3.862	1.337	2.142	0.032	1.96	7.61
CKS1B	0.426	0.298	1.43	0.153	1.531	0.456	1.164	0.244	0.854	2.744
CKMT2	-0.149	0.295	-0.505	0.614	0.861	0.255	-0.545	0.586	0.483	1.537
CKM	0.084	0.297	0.284	0.776	1.088	0.323	0.272	0.785	0.608	1.948
CKLF	0.576	0.302	1.904	0.057	1.778	0.538	1.448	0.148	0.983	3.216
CKB	-0.255	0.296	-0.862	0.389	0.775	0.229	-0.982	0.326	0.434	1.384

CKAP5	0.632	0.31	2.04	0.041	1.882	0.583	1.512	0.131	1.025	3.454
CKAP4	-0.203	0.296	-0.686	0.493	0.816	0.242	-0.76	0.447	0.457	1.459
CKAP2	0.758	0.31	2.449	0.014	2.135	0.661	1.716	0.086	1.163	3.918
CIZ1	-0.308	0.298	-1.035	0.301	0.735	0.219	-1.212	0.225	0.41	1.317
CITED2	-0.751	0.307	-2.444	0.015	0.472	0.145	-3.642	0	0.258	0.862
CITED1	0.194	0.296	0.657	0.511	1.214	0.359	0.597	0.55	0.68	2.167
CISH	-0.082	0.295	-0.276	0.783	0.922	0.272	-0.288	0.774	0.517	1.645
CISD1	0.402	0.298	1.348	0.178	1.495	0.446	1.11	0.267	0.833	2.683
CIRBP	-0.424	0.302	-1.402	0.161	0.655	0.198	-1.746	0.081	0.362	1.184
CIR1	0.048	0.296	0.162	0.871	1.049	0.31	0.159	0.874	0.588	1.873
CINP	0.369	0.3	1.233	0.218	1.447	0.434	1.031	0.303	0.804	2.603
CILP	-0.493	0.3	-1.647	0.1	0.611	0.183	-2.129	0.033	0.339	1.098
CILK1	-0.031	0.296	-0.103	0.918	0.97	0.287	-0.105	0.916	0.543	1.732
CIITA	-0.735	0.31	-2.369	0.018	0.479	0.149	-3.499	0	0.261	0.881
CIDEC	0.135	0.296	0.456	0.648	1.145	0.339	0.427	0.67	0.64	2.046
CIDEB	-0.321	0.3	-1.072	0.284	0.725	0.217	-1.264	0.206	0.403	1.305
CIDEA	-0.071	0.296	-0.241	0.81	0.931	0.276	-0.25	0.803	0.521	1.665
CIC	-0.238	0.297	-0.803	0.422	0.788	0.234	-0.907	0.365	0.441	1.41
CIB2	0.219	0.298	0.734	0.463	1.244	0.371	0.66	0.51	0.694	2.231
CIB1	-0.179	0.299	-0.599	0.549	0.836	0.25	-0.656	0.512	0.466	1.501
CIAPIN1	0.479	0.3	1.597	0.11	1.614	0.484	1.269	0.204	0.897	2.903
CIAO3	-0.11	0.296	-0.372	0.71	0.896	0.265	-0.393	0.694	0.501	1.601
CIAO2B	0.457	0.3	1.523	0.128	1.579	0.474	1.222	0.222	0.877	2.843
CIAO1	-0.259	0.298	-0.871	0.384	0.772	0.23	-0.994	0.32	0.43	1.383
CHUK	0.386	0.298	1.296	0.195	1.471	0.438	1.075	0.282	0.821	2.636
CHTOP	-0.327	0.298	-1.099	0.272	0.721	0.215	-1.299	0.194	0.403	1.292
CHSY1	0.066	0.296	0.223	0.824	1.068	0.316	0.216	0.829	0.599	1.906
CHST8	0.531	0.301	1.763	0.078	1.701	0.513	1.367	0.171	0.942	3.071
CHST7	-0.218	0.296	-0.735	0.463	0.804	0.238	-0.821	0.412	0.45	1.438
CHST5	-0.024	0.296	-0.081	0.935	0.976	0.289	-0.082	0.935	0.546	1.745
CHST4	0.044	0.295	0.149	0.881	1.045	0.309	0.146	0.884	0.586	1.865
CHST3	-0.165	0.296	-0.559	0.576	0.848	0.251	-0.608	0.543	0.474	1.514
CHST2	-0.404	0.298	-1.353	0.176	0.668	0.199	-1.667	0.095	0.372	1.198
CHST15	-0.639	0.31	-2.062	0.039	0.528	0.164	-2.887	0.004	0.287	0.969
CHST12	0.132	0.296	0.446	0.656	1.141	0.337	0.417	0.676	0.639	2.036
CHST11	0.313	0.298	1.05	0.294	1.367	0.407	0.902	0.367	0.763	2.45
CHST10	0.119	0.295	0.404	0.687	1.127	0.333	0.38	0.704	0.632	2.01
CHST1	-0.062	0.295	-0.21	0.834	0.94	0.278	-0.216	0.829	0.527	1.677

CHRNG	0.328	0.298	1.1	0.271	1.388	0.413	0.938	0.348	0.774	2.488
CHRNE	-0.231	0.298	-0.776	0.438	0.794	0.236	-0.873	0.383	0.443	1.423
CHRND	0.188	0.297	0.635	0.526	1.207	0.358	0.579	0.563	0.675	2.159
CHRNB4	-0.058	0.295	-0.198	0.843	0.943	0.279	-0.204	0.839	0.529	1.683
CHRNB3	-0.204	0.296	-0.691	0.49	0.815	0.241	-0.766	0.444	0.457	1.455
CHRNB2	0.236	0.296	0.796	0.426	1.266	0.375	0.709	0.478	0.708	2.261
CHRNB1	0.046	0.298	0.153	0.878	1.047	0.312	0.15	0.881	0.584	1.877
CHRNA9	-0.069	0.295	-0.232	0.816	0.934	0.276	-0.24	0.81	0.523	1.666
CHRNA6	-0.561	0.306	-1.834	0.067	0.571	0.175	-2.46	0.014	0.313	1.039
CHRNA5	0.318	0.298	1.07	0.285	1.375	0.409	0.916	0.36	0.767	2.463
CHRNA4	-0.211	0.296	-0.714	0.475	0.809	0.24	-0.795	0.427	0.453	1.446
CHRNA3	-0.41	0.3	-1.368	0.171	0.663	0.199	-1.691	0.091	0.368	1.194
CHRNA2	0.125	0.297	0.419	0.675	1.133	0.337	0.394	0.694	0.632	2.028
CHRNA10	0.39	0.3	1.302	0.193	1.477	0.443	1.078	0.281	0.821	2.657
CHRNA1	0.228	0.296	0.769	0.442	1.256	0.372	0.688	0.491	0.703	2.245
CHRM5	0.081	0.295	0.274	0.784	1.084	0.32	0.263	0.792	0.608	1.935
CHRM4	0.299	0.3	0.996	0.319	1.348	0.404	0.861	0.389	0.749	2.426
CHRM3	-0.478	0.301	-1.591	0.112	0.62	0.186	-2.041	0.041	0.344	1.117
CHRM2	0.034	0.296	0.116	0.908	1.035	0.306	0.114	0.91	0.58	1.847
CHRDL1	-0.247	0.296	-0.833	0.405	0.781	0.231	-0.945	0.345	0.437	1.396
CHRD	0.224	0.296	0.755	0.451	1.251	0.371	0.676	0.499	0.7	2.236
CHPT1	-0.304	0.303	-1.004	0.315	0.738	0.223	-1.174	0.24	0.408	1.335
CHPF2	-0.386	0.298	-1.296	0.195	0.68	0.203	-1.581	0.114	0.379	1.219
CHPF	-0.173	0.296	-0.584	0.559	0.841	0.249	-0.637	0.524	0.47	1.504
CHP2	-0.246	0.296	-0.829	0.407	0.782	0.232	-0.94	0.347	0.438	1.398
CHP1	-0.525	0.306	-1.718	0.086	0.592	0.181	-2.26	0.024	0.325	1.077
CHORDC1	0.281	0.301	0.934	0.35	1.325	0.399	0.814	0.416	0.734	2.39
CHODL	0.627	0.304	2.063	0.039	1.873	0.569	1.532	0.125	1.032	3.398
CHN2	0.06	0.295	0.203	0.839	1.062	0.313	0.197	0.844	0.595	1.894
CHN1	-0.291	0.298	-0.975	0.33	0.748	0.223	-1.131	0.258	0.417	1.341
CHMP7	-0.353	0.304	-1.16	0.246	0.703	0.214	-1.391	0.164	0.388	1.275
CHMP6	0.372	0.298	1.25	0.211	1.451	0.433	1.043	0.297	0.809	2.603
CHMP5	-0.071	0.297	-0.238	0.812	0.932	0.277	-0.247	0.805	0.52	1.668
CHMP2B	-0.367	0.303	-1.21	0.226	0.693	0.21	-1.461	0.144	0.382	1.256
CHMP2A	0.32	0.298	1.074	0.283	1.377	0.41	0.919	0.358	0.768	2.467
CHMP1B	-0.243	0.298	-0.816	0.415	0.784	0.233	-0.923	0.356	0.438	1.406
CHMP1A	0.509	0.3	1.698	0.089	1.663	0.498	1.331	0.183	0.925	2.992
CHML	0.57	0.306	1.865	0.062	1.769	0.541	1.421	0.155	0.971	3.221

CHM	-0.194	0.299	-0.651	0.515	0.823	0.246	-0.718	0.473	0.458	1.479
CHL1	-0.938	0.321	-2.922	0.003	0.391	0.126	-4.843	0	0.209	0.734
CHKB	-0.612	0.304	-2.015	0.044	0.542	0.165	-2.779	0.005	0.299	0.983
CHKA	-0.272	0.298	-0.915	0.36	0.762	0.227	-1.052	0.293	0.425	1.365
CHIT1	-0.423	0.3	-1.41	0.159	0.655	0.197	-1.755	0.079	0.364	1.18
CHIC2	0.52	0.302	1.721	0.085	1.682	0.509	1.342	0.18	0.93	3.042
CHIA	-0.17	0.297	-0.571	0.568	0.844	0.251	-0.623	0.534	0.471	1.511
CHI3L2	-0.219	0.296	-0.74	0.459	0.803	0.238	-0.828	0.408	0.449	1.435
CHI3L1	0.085	0.295	0.289	0.772	1.089	0.322	0.277	0.782	0.61	1.943
CHGB	-0.49	0.3	-1.632	0.103	0.613	0.184	-2.106	0.035	0.34	1.103
CHGA	0.317	0.298	1.067	0.286	1.374	0.409	0.914	0.361	0.767	2.461
CHFR	0.089	0.297	0.298	0.765	1.093	0.325	0.286	0.775	0.61	1.956
CHERP	-0.035	0.296	-0.119	0.905	0.965	0.285	-0.121	0.903	0.541	1.723
CHEK2	0.143	0.297	0.481	0.631	1.153	0.342	0.448	0.654	0.645	2.063
CHEK1	0.401	0.3	1.338	0.181	1.494	0.448	1.102	0.27	0.83	2.689
CHD9	-0.284	0.297	-0.955	0.34	0.753	0.224	-1.104	0.269	0.42	1.349
CHD8	-0.29	0.296	-0.979	0.328	0.748	0.222	-1.136	0.256	0.419	1.337
CHD7	0.236	0.298	0.792	0.428	1.266	0.377	0.706	0.48	0.706	2.268
CHD5	0.076	0.297	0.257	0.797	1.079	0.32	0.247	0.805	0.603	1.93
CHD4	-0.494	0.302	-1.634	0.102	0.61	0.185	-2.113	0.035	0.337	1.104
CHD3	-0.349	0.297	-1.177	0.239	0.705	0.209	-1.408	0.159	0.394	1.262
CHD2	-0.017	0.296	-0.058	0.954	0.983	0.291	-0.058	0.954	0.55	1.756
CHD1L	-0.253	0.297	-0.851	0.395	0.777	0.231	-0.968	0.333	0.434	1.391
CHD1	0.406	0.299	1.358	0.175	1.5	0.448	1.116	0.264	0.835	2.695
CHCHD7	0.031	0.295	0.105	0.917	1.031	0.304	0.103	0.918	0.578	1.839
CHCHD3	0.334	0.298	1.122	0.262	1.397	0.416	0.954	0.34	0.779	2.503
CHCHD2	0.934	0.315	2.963	0.003	2.545	0.802	1.926	0.054	1.372	4.72
CHAT	-0.621	0.306	-2.029	0.042	0.537	0.164	-2.813	0.005	0.295	0.979
CHAF1B	0.55	0.3	1.833	0.067	1.734	0.521	1.41	0.159	0.963	3.123
CHAF1A	0.997	0.321	3.107	0.002	2.709	0.869	1.967	0.049	1.445	5.079
CHAD	-0.234	0.299	-0.784	0.433	0.791	0.236	-0.883	0.377	0.441	1.421
CHAC1	0.265	0.298	0.891	0.373	1.304	0.388	0.782	0.434	0.727	2.336
CH25H	-0.948	0.316	-2.999	0.003	0.387	0.123	-5	0	0.209	0.72
CGRRF1	0.035	0.296	0.117	0.907	1.035	0.307	0.115	0.908	0.579	1.85
CGREF1	-0.335	0.3	-1.117	0.264	0.715	0.215	-1.328	0.184	0.397	1.288
CGGBP1	0.781	0.31	2.521	0.012	2.184	0.677	1.749	0.08	1.19	4.009
CGA	0.224	0.296	0.757	0.449	1.251	0.371	0.678	0.498	0.7	2.236
CFTR	-0.451	0.299	-1.508	0.131	0.637	0.19	-1.906	0.057	0.355	1.145

CFP	-0.286	0.298	-0.959	0.338	0.751	0.224	-1.11	0.267	0.419	1.348
CFLAR	0.236	0.295	0.8	0.424	1.266	0.374	0.712	0.476	0.71	2.26
CFL1	0.431	0.3	1.437	0.151	1.538	0.461	1.168	0.243	0.855	2.769
CFI	-0.514	0.302	-1.699	0.089	0.598	0.181	-2.221	0.026	0.331	1.082
CFHR5	-0.607	0.31	-1.96	0.05	0.545	0.169	-2.696	0.007	0.297	1
CFHR4	0.34	0.3	1.136	0.256	1.405	0.421	0.963	0.335	0.781	2.528
CFHR2	0.481	0.3	1.601	0.109	1.617	0.486	1.271	0.204	0.898	2.914
CFH	-0.802	0.31	-2.583	0.01	0.449	0.139	-3.962	0	0.244	0.824
CFDP1	0.993	0.317	3.135	0.002	2.699	0.855	1.987	0.047	1.451	5.022
CFD	-0.337	0.298	-1.131	0.258	0.714	0.213	-1.345	0.179	0.398	1.28
CFB	-0.181	0.296	-0.611	0.541	0.835	0.247	-0.67	0.503	0.467	1.491
CFAP94	-0.561	0.306	-1.835	0.067	0.571	0.174	-2.461	0.014	0.313	1.039
CFAP74	-0.471	0.302	-1.562	0.118	0.624	0.188	-1.995	0.046	0.346	1.127
CFAP70	-0.019	0.295	-0.063	0.949	0.981	0.29	-0.064	0.949	0.55	1.751
CFAP69	-0.63	0.31	-2.03	0.042	0.533	0.165	-2.828	0.005	0.29	0.978
CFAP46	0.197	0.298	0.661	0.509	1.217	0.362	0.6	0.549	0.679	2.181
CFAP45	-0.676	0.31	-2.182	0.029	0.509	0.158	-3.117	0.002	0.277	0.934
CFAP44	-0.103	0.295	-0.349	0.727	0.902	0.266	-0.368	0.713	0.506	1.609
CFAP43	0.014	0.296	0.047	0.963	1.014	0.3	0.047	0.963	0.568	1.81
CFAP410	0.239	0.297	0.806	0.42	1.27	0.377	0.717	0.474	0.71	2.271
CFAP298	0.228	0.299	0.762	0.446	1.256	0.375	0.681	0.496	0.699	2.255
CFAP20	0.65	0.303	2.146	0.032	1.915	0.58	1.578	0.114	1.058	3.467
CETP	-0.398	0.298	-1.333	0.182	0.672	0.2	-1.637	0.102	0.375	1.205
CETN3	-0.023	0.296	-0.078	0.938	0.977	0.289	-0.079	0.937	0.548	1.744
CETN2	0.35	0.298	1.175	0.24	1.419	0.423	0.991	0.322	0.791	2.546
CETN1	0.123	0.297	0.415	0.678	1.131	0.336	0.39	0.696	0.632	2.026
CES3	0.181	0.296	0.612	0.541	1.199	0.355	0.56	0.576	0.671	2.142
CES2	0.643	0.31	2.076	0.038	1.903	0.589	1.531	0.126	1.037	3.492
CES1P1	0.499	0.306	1.631	0.103	1.647	0.503	1.284	0.199	0.904	2.998
CES1	0.024	0.295	0.081	0.936	1.024	0.302	0.08	0.936	0.574	1.827
CERT1	0.018	0.295	0.061	0.951	1.018	0.301	0.061	0.952	0.571	1.816
CERS6	-0.398	0.302	-1.317	0.188	0.672	0.203	-1.618	0.106	0.371	1.214
CERS4	0.042	0.295	0.142	0.887	1.043	0.308	0.14	0.889	0.585	1.86
CERS2	-0.004	0.298	-0.013	0.99	0.996	0.296	-0.013	0.99	0.556	1.785
CERNA1	-0.282	0.298	-0.948	0.343	0.754	0.224	-1.096	0.273	0.421	1.351
CERK	0.458	0.302	1.513	0.13	1.58	0.478	1.214	0.225	0.874	2.859
CER1	-0.01	0.299	-0.032	0.974	0.99	0.296	-0.032	0.974	0.551	1.781
CEPT1	0.994	0.322	3.091	0.002	2.702	0.869	1.959	0.05	1.439	5.075

CEP97	0.636	0.306	2.078	0.038	1.888	0.578	1.538	0.124	1.037	3.439
CEP85	0.025	0.295	0.084	0.933	1.025	0.303	0.083	0.934	0.574	1.829
CEP83	-0.179	0.296	-0.605	0.545	0.836	0.248	-0.662	0.508	0.468	1.494
CEP76	0.191	0.3	0.635	0.525	1.21	0.363	0.578	0.563	0.672	2.178
CEP72	0.042	0.296	0.144	0.886	1.043	0.309	0.141	0.888	0.584	1.864
CEP70	0.731	0.306	2.386	0.017	2.077	0.636	1.693	0.091	1.139	3.787
CEP68	-0.043	0.295	-0.146	0.884	0.958	0.283	-0.149	0.881	0.537	1.708
CEP63	-0.044	0.296	-0.149	0.882	0.957	0.283	-0.152	0.879	0.536	1.708
CEP57	-0.034	0.295	-0.117	0.907	0.966	0.285	-0.119	0.906	0.542	1.723
CEP55	0.66	0.306	2.16	0.031	1.935	0.591	1.581	0.114	1.063	3.523
CEP43	0.001	0.296	0.002	0.998	1.001	0.296	0.002	0.998	0.56	1.788
CEP41	-0.061	0.296	-0.206	0.837	0.941	0.278	-0.212	0.832	0.527	1.68
CEP350	-0.049	0.295	-0.166	0.868	0.952	0.281	-0.17	0.865	0.534	1.699
CEP295	0.327	0.298	1.098	0.272	1.386	0.413	0.937	0.349	0.774	2.484
CEP290	-0.538	0.307	-1.756	0.079	0.584	0.179	-2.326	0.02	0.32	1.065
CEP250	0.409	0.3	1.365	0.172	1.505	0.451	1.121	0.262	0.837	2.708
CEP192	-0.043	0.295	-0.147	0.883	0.958	0.283	-0.15	0.881	0.537	1.709
CEP170B	0.731	0.31	2.358	0.018	2.076	0.643	1.673	0.094	1.131	3.81
CEP164	0.223	0.296	0.754	0.451	1.25	0.37	0.676	0.499	0.7	2.233
CEP162	0.128	0.296	0.433	0.665	1.136	0.336	0.406	0.685	0.637	2.028
CEP152	0.51	0.302	1.687	0.092	1.666	0.504	1.321	0.186	0.921	3.014
CEP135	-0.141	0.296	-0.476	0.634	0.869	0.257	-0.511	0.609	0.486	1.552
CEP131	0.317	0.301	1.056	0.291	1.373	0.413	0.905	0.366	0.762	2.475
CEP112	-0.124	0.295	-0.421	0.673	0.883	0.261	-0.449	0.654	0.495	1.575
CEP104	0.726	0.305	2.378	0.017	2.067	0.631	1.691	0.091	1.136	3.759
CENPX	0.404	0.299	1.353	0.176	1.498	0.447	1.113	0.266	0.834	2.689
CENPU	0.806	0.31	2.601	0.009	2.238	0.693	1.786	0.074	1.22	4.108
CENPT	-0.277	0.298	-0.929	0.353	0.758	0.226	-1.07	0.284	0.423	1.359
CENPQ	0.624	0.302	2.062	0.039	1.865	0.564	1.535	0.125	1.031	3.374
CENPO	0.291	0.297	0.983	0.326	1.338	0.397	0.852	0.394	0.748	2.393
CENPN	0.501	0.3	1.672	0.095	1.65	0.495	1.315	0.188	0.917	2.969
CENPM	0.713	0.306	2.332	0.02	2.04	0.624	1.668	0.095	1.121	3.714
CENPJ	0.799	0.315	2.537	0.011	2.223	0.7	1.747	0.081	1.199	4.12
CENPI	0.47	0.303	1.554	0.12	1.601	0.485	1.239	0.215	0.884	2.897
CENPF	0.497	0.307	1.619	0.106	1.644	0.505	1.275	0.202	0.9	3.001
CENPE	0.819	0.31	2.645	0.008	2.269	0.703	1.805	0.071	1.236	4.164
CENPC	0.542	0.302	1.792	0.073	1.719	0.52	1.384	0.166	0.951	3.109
CENPBD1P1	0.216	0.296	0.728	0.467	1.241	0.367	0.655	0.513	0.694	2.216

CENPB	-0.023	0.296	-0.077	0.939	0.978	0.289	-0.078	0.938	0.548	1.745
CENPA	0.614	0.311	1.975	0.048	1.848	0.575	1.476	0.14	1.005	3.399
CEND1	0.708	0.31	2.282	0.023	2.029	0.629	1.635	0.102	1.105	3.727
CEMP1	0.623	0.304	2.047	0.041	1.865	0.567	1.524	0.128	1.027	3.386
CEMIP2	-0.231	0.296	-0.78	0.436	0.794	0.235	-0.877	0.38	0.444	1.418
CEMIP	0.138	0.296	0.468	0.64	1.148	0.34	0.437	0.662	0.643	2.051
CELSR3	0.141	0.295	0.479	0.632	1.152	0.34	0.447	0.655	0.646	2.055
CELSR2	-0.284	0.3	-0.946	0.344	0.753	0.226	-1.094	0.274	0.418	1.355
CELSR1	-0.225	0.301	-0.748	0.454	0.799	0.24	-0.839	0.401	0.443	1.44
CELP	0.029	0.296	0.097	0.923	1.029	0.304	0.095	0.924	0.576	1.837
CELF3	0.177	0.296	0.599	0.549	1.194	0.353	0.549	0.583	0.669	2.131
CELF2	-0.718	0.304	-2.361	0.018	0.488	0.148	-3.454	0.001	0.269	0.885
CELF1	-0.072	0.296	-0.243	0.808	0.931	0.275	-0.252	0.801	0.521	1.662
CELA3B	0.867	0.316	2.743	0.006	2.379	0.751	1.835	0.067	1.281	4.418
CELA3A	0.159	0.298	0.533	0.594	1.172	0.349	0.493	0.622	0.654	2.1
CELA2B	-0.222	0.296	-0.75	0.453	0.801	0.237	-0.84	0.401	0.448	1.431
CEL	0.157	0.295	0.53	0.596	1.169	0.346	0.49	0.624	0.655	2.087
CEBPZ	0.57	0.303	1.881	0.06	1.769	0.536	1.433	0.152	0.976	3.204
CEBPG	0.942	0.317	2.97	0.003	2.564	0.813	1.924	0.054	1.378	4.772
CEBPE	-0.048	0.295	-0.163	0.871	0.953	0.282	-0.167	0.868	0.534	1.7
CEBPD	-0.014	0.296	-0.049	0.961	0.986	0.291	-0.049	0.961	0.552	1.76
CEBPB	0.385	0.3	1.284	0.199	1.47	0.441	1.066	0.287	0.817	2.645
CEBPA-DT	1.05	0.328	3.205	0.001	2.858	0.936	1.984	0.047	1.504	5.432
CEBPA	-0.247	0.297	-0.833	0.405	0.781	0.232	-0.945	0.345	0.437	1.397
CEACAM8	0.039	0.295	0.132	0.895	1.04	0.307	0.13	0.897	0.583	1.854
CEACAM7	0.401	0.3	1.337	0.181	1.493	0.448	1.101	0.271	0.829	2.687
CEACAM6	-0.364	0.298	-1.22	0.222	0.695	0.207	-1.471	0.141	0.388	1.247
CEACAM5	0.301	0.298	1.011	0.312	1.351	0.402	0.873	0.383	0.754	2.42
CEACAM4	0.326	0.298	1.095	0.274	1.386	0.413	0.934	0.35	0.773	2.484
CEACAM3	-0.069	0.295	-0.232	0.816	0.934	0.276	-0.24	0.81	0.523	1.666
CEACAM21	0.271	0.298	0.91	0.363	1.311	0.39	0.797	0.425	0.732	2.35
CEACAM1	-0.027	0.295	-0.093	0.926	0.973	0.287	-0.094	0.925	0.545	1.736
CDYL	-0.163	0.298	-0.546	0.585	0.85	0.253	-0.593	0.553	0.473	1.525
CDY1	0.046	0.295	0.155	0.877	1.047	0.309	0.152	0.879	0.587	1.867
CDX4	0.008	0.295	0.026	0.98	1.008	0.297	0.026	0.98	0.565	1.797
CDX2	0.522	0.303	1.722	0.085	1.685	0.511	1.342	0.18	0.931	3.052
CDX1	-0.428	0.302	-1.415	0.157	0.652	0.197	-1.766	0.077	0.36	1.179
CDV3	0.175	0.296	0.59	0.555	1.191	0.353	0.541	0.589	0.666	2.129

CDT1	0.606	0.306	1.982	0.048	1.833	0.56	1.486	0.137	1.007	3.337
CDSN	0.218	0.296	0.735	0.462	1.243	0.368	0.66	0.509	0.696	2.221
CDS2	0.291	0.297	0.98	0.327	1.338	0.397	0.851	0.395	0.748	2.393
CDS1	0.387	0.298	1.299	0.194	1.472	0.438	1.077	0.281	0.821	2.639
CDRT1	0.29	0.298	0.975	0.33	1.337	0.398	0.846	0.398	0.746	2.397
CDR2L	0.158	0.295	0.536	0.592	1.171	0.346	0.495	0.62	0.657	2.09
CDR1	-0.163	0.296	-0.55	0.582	0.85	0.252	-0.598	0.55	0.475	1.518
CDON	-0.581	0.31	-1.874	0.061	0.559	0.173	-2.541	0.011	0.305	1.027
CDO1	-0.418	0.3	-1.394	0.163	0.659	0.197	-1.73	0.084	0.366	1.185
CDKN3	0.339	0.298	1.138	0.255	1.403	0.418	0.965	0.335	0.783	2.515
CDKN2D	0.075	0.296	0.255	0.799	1.078	0.319	0.246	0.806	0.604	1.927
CDKN2C	0.313	0.298	1.05	0.294	1.367	0.408	0.901	0.367	0.762	2.452
CDKN2B	-0.424	0.3	-1.412	0.158	0.655	0.196	-1.758	0.079	0.363	1.179
CDKN2AIP	-0.164	0.302	-0.543	0.587	0.849	0.256	-0.59	0.555	0.469	1.534
CDKN2A-DT	0.319	0.298	1.071	0.284	1.376	0.41	0.917	0.359	0.767	2.466
CDKN2A	-0.016	0.295	-0.054	0.957	0.984	0.29	-0.054	0.957	0.552	1.755
CDKN1C	-0.557	0.302	-1.843	0.065	0.573	0.173	-2.467	0.014	0.317	1.036
CDKN1B	-0.553	0.306	-1.809	0.07	0.575	0.176	-2.415	0.016	0.316	1.047
CDKN1A	0.445	0.3	1.485	0.138	1.56	0.467	1.198	0.231	0.867	2.807
CDKL5	0.122	0.296	0.413	0.679	1.13	0.334	0.389	0.697	0.633	2.017
CDKL3	-0.12	0.297	-0.403	0.687	0.887	0.263	-0.429	0.668	0.496	1.587
CDKL2	-0.353	0.299	-1.183	0.237	0.702	0.21	-1.419	0.156	0.391	1.261
CDKL1	-0.142	0.295	-0.48	0.631	0.868	0.256	-0.516	0.606	0.486	1.548
CDKAL1	0.394	0.299	1.318	0.188	1.483	0.443	1.089	0.276	0.825	2.665
CDK9	-0.136	0.296	-0.459	0.647	0.873	0.259	-0.491	0.623	0.488	1.561
CDK8	0.471	0.302	1.56	0.119	1.602	0.484	1.244	0.214	0.886	2.898
CDK7	-0.009	0.299	-0.03	0.976	0.991	0.296	-0.031	0.976	0.552	1.78
CDK6	0.068	0.297	0.23	0.818	1.071	0.318	0.222	0.824	0.598	1.915
CDK5RAP3	-0.247	0.296	-0.834	0.405	0.781	0.231	-0.945	0.344	0.437	1.396
CDK5RAP2	0.043	0.297	0.145	0.885	1.044	0.31	0.142	0.887	0.583	1.869
CDK5RAP1	0.21	0.296	0.709	0.478	1.234	0.365	0.639	0.523	0.69	2.204
CDK5R2	0.674	0.306	2.204	0.028	1.961	0.6	1.603	0.109	1.077	3.571
CDK5R1	0.369	0.298	1.239	0.215	1.446	0.431	1.036	0.3	0.807	2.593
CDK5	-0.071	0.3	-0.237	0.813	0.931	0.279	-0.246	0.806	0.517	1.676
CDK4	0.002	0.295	0.005	0.996	1.002	0.295	0.005	0.996	0.562	1.786
CDK2AP2	0.242	0.296	0.817	0.414	1.274	0.378	0.726	0.468	0.713	2.277
CDK2AP1	0.386	0.3	1.288	0.198	1.471	0.441	1.069	0.285	0.818	2.646
CDK20	-0.169	0.296	-0.569	0.569	0.845	0.25	-0.62	0.535	0.472	1.51

CDK2	0.441	0.308	1.434	0.152	1.555	0.479	1.159	0.246	0.85	2.842
CDK19	0.182	0.296	0.614	0.539	1.199	0.355	0.561	0.575	0.671	2.143
CDK18	0.157	0.295	0.532	0.595	1.17	0.346	0.492	0.622	0.656	2.088
CDK17	-0.285	0.3	-0.949	0.343	0.752	0.226	-1.098	0.272	0.418	1.354
CDK16	-0.046	0.295	-0.157	0.875	0.955	0.282	-0.161	0.872	0.535	1.703
CDK14	-0.613	0.304	-2.015	0.044	0.542	0.165	-2.78	0.005	0.299	0.983
CDK13	0.264	0.298	0.888	0.375	1.302	0.388	0.78	0.435	0.727	2.334
CDK12	0.142	0.297	0.479	0.632	1.153	0.342	0.446	0.655	0.644	2.061
CDK10	-0.928	0.318	-2.914	0.004	0.395	0.126	-4.802	0	0.212	0.738
CDK1	0.535	0.302	1.771	0.077	1.708	0.516	1.371	0.17	0.944	3.089
CDIPT	0.239	0.297	0.804	0.421	1.27	0.377	0.715	0.475	0.709	2.274
CDIP1	0.218	0.297	0.733	0.463	1.243	0.369	0.659	0.51	0.695	2.224
CDHR5	0.442	0.3	1.475	0.14	1.556	0.466	1.192	0.233	0.865	2.8
CDHR2	0.137	0.297	0.461	0.645	1.147	0.341	0.431	0.667	0.641	2.053
CDHR1	0.443	0.3	1.477	0.14	1.557	0.467	1.193	0.233	0.865	2.802
CDH9	-0.284	0.298	-0.954	0.34	0.753	0.224	-1.103	0.27	0.42	1.349
CDH8	0.4	0.3	1.335	0.182	1.492	0.447	1.1	0.271	0.829	2.683
CDH7	-0.342	0.3	-1.142	0.254	0.71	0.213	-1.362	0.173	0.395	1.278
CDH6	-0.093	0.297	-0.315	0.753	0.911	0.27	-0.33	0.741	0.509	1.629
CDH5	-0.912	0.321	-2.843	0.004	0.402	0.129	-4.642	0	0.214	0.753
CDH4	0.553	0.306	1.808	0.071	1.738	0.532	1.389	0.165	0.954	3.166
CDH3	0.045	0.295	0.151	0.88	1.046	0.309	0.148	0.882	0.586	1.865
CDH22	-0.039	0.295	-0.131	0.896	0.962	0.284	-0.134	0.894	0.54	1.715
CDH20	0.041	0.295	0.141	0.888	1.042	0.307	0.138	0.89	0.585	1.858
CDH2	-0.006	0.295	-0.019	0.985	0.994	0.294	-0.019	0.985	0.558	1.774
CDH19	-0.239	0.297	-0.805	0.421	0.788	0.234	-0.91	0.363	0.44	1.408
CDH18	-0.09	0.295	-0.304	0.761	0.914	0.27	-0.318	0.751	0.512	1.631
CDH17	-0.256	0.296	-0.864	0.388	0.774	0.229	-0.984	0.325	0.433	1.384
CDH16	0.024	0.296	0.08	0.936	1.024	0.303	0.079	0.937	0.573	1.829
CDH15	-0.189	0.297	-0.638	0.523	0.827	0.246	-0.702	0.482	0.463	1.48
CDH13	-0.537	0.306	-1.756	0.079	0.585	0.179	-2.324	0.02	0.321	1.064
CDH12	0.216	0.296	0.73	0.465	1.241	0.367	0.657	0.511	0.695	2.217
CDH11	-0.494	0.3	-1.648	0.099	0.61	0.183	-2.131	0.033	0.339	1.098
CDH10	0.478	0.302	1.582	0.114	1.613	0.487	1.258	0.209	0.892	2.916
CDH1	0.334	0.298	1.122	0.262	1.396	0.416	0.954	0.34	0.779	2.502
CDCP1	0.353	0.298	1.183	0.237	1.423	0.424	0.997	0.319	0.793	2.551
CDCA8	0.46	0.3	1.534	0.125	1.584	0.474	1.23	0.219	0.88	2.849
CDCA4	0.531	0.302	1.755	0.079	1.701	0.514	1.362	0.173	0.94	3.077

CDCA3	1.006	0.321	3.135	0.002	2.734	0.877	1.977	0.048	1.458	5.129
CDC73	0.542	0.302	1.792	0.073	1.72	0.52	1.383	0.167	0.95	3.111
CDC7	0.302	0.299	1.012	0.312	1.353	0.404	0.873	0.383	0.753	2.43
CDC6	0.852	0.315	2.707	0.007	2.345	0.738	1.822	0.068	1.265	4.347
CDC5L	0.41	0.298	1.377	0.168	1.507	0.448	1.13	0.259	0.841	2.7
CDC45	0.535	0.302	1.769	0.077	1.707	0.516	1.37	0.171	0.944	3.088
CDC42SE1	-0.475	0.299	-1.591	0.112	0.622	0.186	-2.036	0.042	0.346	1.117
CDC42EP4	0.144	0.295	0.489	0.625	1.155	0.341	0.455	0.649	0.648	2.061
CDC42EP3	-0.331	0.298	-1.111	0.267	0.719	0.214	-1.316	0.188	0.401	1.288
CDC42EP2	0.153	0.296	0.519	0.604	1.166	0.345	0.481	0.631	0.653	2.081
CDC42EP1	-0.226	0.296	-0.763	0.446	0.798	0.236	-0.856	0.392	0.446	1.426
CDC42BPB	-0.069	0.295	-0.235	0.815	0.933	0.276	-0.243	0.808	0.523	1.664
CDC42BPA	-0.207	0.297	-0.697	0.486	0.813	0.242	-0.775	0.439	0.454	1.456
CDC42	-0.103	0.297	-0.345	0.73	0.903	0.268	-0.364	0.716	0.504	1.615
CDC40	0.048	0.295	0.162	0.871	1.049	0.31	0.159	0.874	0.588	1.872
CDC37L1	-0.781	0.315	-2.481	0.013	0.458	0.144	-3.759	0	0.247	0.849
CDC37	-0.32	0.298	-1.073	0.283	0.726	0.216	-1.265	0.206	0.405	1.302
CDC34	0.719	0.305	2.358	0.018	2.052	0.626	1.681	0.093	1.129	3.732
CDC27	0.378	0.298	1.27	0.204	1.46	0.435	1.057	0.29	0.814	2.616
CDC25C	0.838	0.308	2.719	0.007	2.312	0.713	1.841	0.066	1.264	4.231
CDC25B	0.349	0.3	1.165	0.244	1.418	0.425	0.983	0.326	0.788	2.554
CDC25A	0.002	0.295	0.007	0.994	1.002	0.296	0.007	0.994	0.562	1.787
CDC23	0.118	0.295	0.401	0.689	1.126	0.333	0.378	0.706	0.631	2.009
CDC20	0.699	0.31	2.254	0.024	2.011	0.624	1.622	0.105	1.095	3.693
CDC16	0.129	0.296	0.435	0.663	1.137	0.336	0.408	0.683	0.637	2.03
CDC14B	-0.147	0.295	-0.499	0.618	0.863	0.255	-0.537	0.591	0.484	1.54
CDC14A	-0.34	0.303	-1.123	0.261	0.712	0.216	-1.338	0.181	0.393	1.288
CDC123	0.192	0.296	0.649	0.516	1.212	0.359	0.59	0.555	0.678	2.165
CDADC1	0.082	0.295	0.278	0.781	1.085	0.321	0.267	0.79	0.608	1.937
CDA	-0.106	0.298	-0.356	0.722	0.899	0.268	-0.375	0.707	0.501	1.614
CD99	-0.37	0.298	-1.244	0.214	0.691	0.206	-1.505	0.132	0.385	1.238
CD96	-0.08	0.295	-0.27	0.788	0.924	0.273	-0.281	0.779	0.518	1.647
CD93	-0.374	0.298	-1.256	0.209	0.688	0.205	-1.523	0.128	0.384	1.233
CD9	0.314	0.297	1.055	0.292	1.368	0.407	0.905	0.365	0.764	2.45
CD8B	-0.41	0.298	-1.374	0.17	0.664	0.198	-1.698	0.09	0.37	1.191
CD8A	-0.162	0.296	-0.546	0.585	0.851	0.252	-0.592	0.554	0.476	1.521
CD86	0.141	0.295	0.476	0.634	1.151	0.34	0.444	0.657	0.645	2.054
CD84	0.318	0.298	1.069	0.285	1.375	0.41	0.915	0.36	0.767	2.466

CD83	0.165	0.296	0.556	0.578	1.179	0.35	0.513	0.608	0.66	2.109
CD82	0.049	0.295	0.165	0.869	1.05	0.31	0.161	0.872	0.588	1.873
CD81	0.177	0.296	0.598	0.55	1.194	0.354	0.548	0.584	0.668	2.133
CD80	0.053	0.296	0.178	0.859	1.054	0.312	0.173	0.863	0.59	1.882
CD79B	0.107	0.297	0.36	0.719	1.113	0.33	0.341	0.733	0.622	1.99
CD79A	-0.29	0.297	-0.977	0.328	0.748	0.222	-1.134	0.257	0.418	1.339
CD74	-0.594	0.306	-1.94	0.052	0.552	0.169	-2.65	0.008	0.303	1.006
CD72	-0.049	0.296	-0.166	0.868	0.952	0.281	-0.17	0.865	0.533	1.699
CD70	0.269	0.296	0.906	0.365	1.308	0.388	0.795	0.427	0.732	2.339
CD7	-0.085	0.297	-0.287	0.774	0.918	0.273	-0.3	0.765	0.513	1.643
CD69	-0.671	0.306	-2.19	0.029	0.511	0.157	-3.121	0.002	0.281	0.932
CD63	-0.421	0.3	-1.406	0.16	0.656	0.197	-1.749	0.08	0.365	1.181
CD6	0.006	0.296	0.02	0.984	1.006	0.298	0.02	0.984	0.563	1.796
CD5L	0.414	0.303	1.368	0.171	1.513	0.458	1.12	0.263	0.836	2.739
CD59	-0.25	0.298	-0.841	0.4	0.779	0.232	-0.956	0.339	0.435	1.395
CD58	-0.147	0.296	-0.498	0.619	0.863	0.255	-0.536	0.592	0.483	1.541
CD55	-0.304	0.3	-1.015	0.31	0.738	0.221	-1.187	0.235	0.41	1.327
CD53	-0.5	0.303	-1.651	0.099	0.606	0.184	-2.143	0.032	0.335	1.098
CD52	-0.365	0.298	-1.227	0.22	0.694	0.207	-1.481	0.139	0.387	1.244
CD5	-0.131	0.296	-0.444	0.657	0.877	0.259	-0.474	0.635	0.491	1.566
CD48	-0.463	0.3	-1.54	0.124	0.63	0.189	-1.958	0.05	0.349	1.135
CD47	0.156	0.296	0.525	0.599	1.168	0.346	0.486	0.627	0.654	2.089
CD46	0.094	0.295	0.32	0.749	1.099	0.325	0.305	0.76	0.616	1.961
CD44	-0.846	0.315	-2.685	0.007	0.429	0.135	-4.222	0	0.231	0.796
CD40LG	-0.04	0.295	-0.135	0.893	0.961	0.284	-0.138	0.891	0.539	1.714
CD40	-0.245	0.299	-0.819	0.413	0.783	0.234	-0.928	0.354	0.435	1.407
CD4	-0.554	0.302	-1.834	0.067	0.575	0.174	-2.449	0.014	0.318	1.039
CD3G	-0.153	0.296	-0.517	0.605	0.858	0.254	-0.559	0.576	0.481	1.532
CD3E	-0.296	0.298	-0.991	0.322	0.744	0.222	-1.153	0.249	0.415	1.335
CD3D	-0.267	0.297	-0.902	0.367	0.765	0.227	-1.034	0.301	0.428	1.369
CD38	-0.005	0.296	-0.018	0.985	0.995	0.295	-0.018	0.985	0.556	1.778
CD37	-1.019	0.328	-3.105	0.002	0.361	0.118	-5.394	0	0.19	0.687
CD36	0.351	0.3	1.171	0.242	1.42	0.426	0.987	0.323	0.789	2.555
CD34	-0.338	0.297	-1.136	0.256	0.713	0.212	-1.351	0.177	0.398	1.278
CD33	0.432	0.3	1.441	0.15	1.54	0.461	1.17	0.242	0.856	2.77
CD320	0.144	0.295	0.487	0.626	1.155	0.341	0.454	0.65	0.647	2.06
CD300C	-0.301	0.298	-1.01	0.313	0.74	0.221	-1.178	0.239	0.412	1.328
CD300A	0.098	0.296	0.331	0.741	1.103	0.326	0.315	0.753	0.618	1.969

CD2BP2	-0.218	0.298	-0.732	0.464	0.804	0.239	-0.818	0.413	0.449	1.441
CD2AP	-0.025	0.296	-0.085	0.932	0.975	0.288	-0.086	0.931	0.546	1.74
CD28	-0.166	0.296	-0.56	0.576	0.847	0.251	-0.609	0.543	0.474	1.514
CD27	-0.372	0.298	-1.249	0.212	0.689	0.205	-1.513	0.13	0.384	1.236
CD248	-0.272	0.296	-0.917	0.359	0.762	0.226	-1.054	0.292	0.426	1.362
CD247	-0.12	0.296	-0.406	0.685	0.887	0.262	-0.432	0.666	0.497	1.583
CD244	-0.428	0.298	-1.437	0.151	0.652	0.194	-1.793	0.073	0.363	1.169
CD24	0.02	0.295	0.066	0.947	1.02	0.301	0.066	0.948	0.572	1.819
CD226	-0.042	0.296	-0.14	0.889	0.959	0.284	-0.143	0.886	0.537	1.715
CD22	0.31	0.297	1.045	0.296	1.364	0.405	0.898	0.369	0.762	2.441
CD209	-0.082	0.296	-0.278	0.781	0.921	0.273	-0.29	0.772	0.515	1.646
CD207	0.304	0.298	1.018	0.309	1.355	0.404	0.878	0.38	0.755	2.43
CD200	-0.965	0.316	-3.056	0.002	0.381	0.12	-5.145	0	0.205	0.707
CD2	-0.065	0.296	-0.219	0.826	0.937	0.277	-0.227	0.821	0.525	1.673
CD1E	-0.311	0.297	-1.048	0.295	0.733	0.217	-1.229	0.219	0.41	1.311
CD1D	-0.092	0.296	-0.311	0.756	0.912	0.27	-0.325	0.745	0.511	1.63
CD1C	-1.33	0.346	-3.843	0	0.265	0.092	-8.034	0	0.134	0.521
CD1B	-0.075	0.295	-0.255	0.799	0.928	0.274	-0.265	0.791	0.52	1.655
CD1A	-0.007	0.295	-0.024	0.981	0.993	0.293	-0.024	0.981	0.557	1.771
CD19	-0.706	0.306	-2.305	0.021	0.494	0.151	-3.348	0.001	0.271	0.9
CD180	-0.103	0.295	-0.347	0.729	0.903	0.267	-0.365	0.715	0.506	1.611
CD177	-0.244	0.296	-0.822	0.411	0.784	0.232	-0.931	0.352	0.439	1.401
CD164	-0.113	0.296	-0.382	0.702	0.893	0.264	-0.405	0.686	0.5	1.594
CD163	0.226	0.296	0.761	0.447	1.253	0.371	0.681	0.496	0.701	2.239
CD160	0.166	0.298	0.558	0.577	1.181	0.352	0.514	0.607	0.659	2.117
CD151	-0.41	0.302	-1.355	0.175	0.664	0.201	-1.674	0.094	0.367	1.201
CD14	-0.095	0.296	-0.322	0.747	0.909	0.269	-0.338	0.735	0.508	1.625
CD101	-0.024	0.296	-0.08	0.936	0.977	0.289	-0.081	0.935	0.547	1.745
CCZ1B	0.407	0.302	1.346	0.178	1.503	0.455	1.106	0.269	0.831	2.718
CCZ1	0.519	0.306	1.699	0.089	1.68	0.513	1.325	0.185	0.923	3.059
CCT8L2	0.339	0.3	1.13	0.259	1.403	0.42	0.958	0.338	0.78	2.524
CCT8	0.29	0.296	0.977	0.328	1.336	0.396	0.849	0.396	0.747	2.389
CCT7	0.545	0.302	1.803	0.071	1.725	0.522	1.39	0.165	0.954	3.121
CCT6B	-0.207	0.298	-0.696	0.487	0.813	0.242	-0.773	0.44	0.453	1.458
CCT6A	0.358	0.3	1.193	0.233	1.43	0.429	1.003	0.316	0.795	2.573
CCT5	0.528	0.3	1.76	0.078	1.696	0.509	1.368	0.171	0.942	3.053
CCT4	0.592	0.31	1.908	0.056	1.807	0.56	1.44	0.15	0.984	3.317
CCT3	0.091	0.295	0.308	0.758	1.095	0.323	0.294	0.769	0.614	1.952

CCT2	0.322	0.297	1.084	0.278	1.38	0.409	0.927	0.354	0.771	2.468
CCSER2	-0.608	0.303	-2.003	0.045	0.545	0.165	-2.755	0.006	0.301	0.987
CCS	0.274	0.297	0.926	0.355	1.316	0.39	0.81	0.418	0.736	2.353
CCRL2	0.16	0.296	0.541	0.589	1.173	0.347	0.5	0.617	0.657	2.094
CCR9	0.09	0.295	0.305	0.76	1.094	0.323	0.292	0.771	0.613	1.952
CCR8	-0.098	0.298	-0.33	0.741	0.906	0.27	-0.347	0.729	0.505	1.626
CCR7	0.272	0.299	0.912	0.362	1.313	0.392	0.798	0.425	0.731	2.358
CCR6	-0.533	0.3	-1.773	0.076	0.587	0.176	-2.342	0.019	0.326	1.058
CCR5	0.06	0.295	0.205	0.838	1.062	0.314	0.199	0.843	0.595	1.896
CCR4	0.438	0.303	1.445	0.148	1.549	0.469	1.171	0.242	0.856	2.805
CCR3	0.114	0.296	0.387	0.699	1.121	0.332	0.365	0.715	0.628	2.003
CCR2	-0.273	0.297	-0.919	0.358	0.761	0.226	-1.057	0.291	0.425	1.362
CCR10	-0.443	0.3	-1.476	0.14	0.642	0.193	-1.857	0.063	0.357	1.156
CCR1	0.654	0.306	2.137	0.033	1.923	0.589	1.568	0.117	1.056	3.504
CCPG1	-0.243	0.3	-0.811	0.418	0.784	0.235	-0.918	0.359	0.435	1.412
CCP110	0.085	0.295	0.289	0.772	1.089	0.322	0.277	0.782	0.611	1.943
CCNT2	-0.327	0.298	-1.098	0.272	0.721	0.215	-1.298	0.194	0.402	1.293
CCNT1	0.388	0.298	1.302	0.193	1.474	0.44	1.079	0.281	0.822	2.645
CCNP	-0.328	0.298	-1.1	0.271	0.721	0.215	-1.302	0.193	0.402	1.292
CCNO	0.194	0.296	0.654	0.513	1.214	0.359	0.595	0.552	0.68	2.167
CCNL2	-0.87	0.313	-2.778	0.005	0.419	0.131	-4.428	0	0.227	0.774
CCNL1	-0.055	0.297	-0.186	0.852	0.946	0.281	-0.191	0.848	0.528	1.695
CCNK	0.067	0.295	0.227	0.82	1.069	0.316	0.22	0.826	0.599	1.908
CCNJL	0.072	0.296	0.245	0.807	1.075	0.319	0.236	0.814	0.601	1.922
CCNJ	0.964	0.321	3.003	0.003	2.623	0.842	1.927	0.054	1.398	4.922
CCNI	-0.381	0.3	-1.273	0.203	0.683	0.205	-1.549	0.121	0.38	1.229
CCNH	0.179	0.296	0.606	0.545	1.196	0.354	0.554	0.579	0.67	2.138
CCNG2	-0.332	0.299	-1.109	0.267	0.718	0.215	-1.315	0.188	0.399	1.29
CCNG1	-0.751	0.315	-2.386	0.017	0.472	0.149	-3.556	0	0.254	0.875
CCNF	0.447	0.3	1.491	0.136	1.564	0.469	1.203	0.229	0.869	2.815
CCNE2	0.67	0.306	2.191	0.028	1.953	0.597	1.597	0.11	1.073	3.556
CCNE1	0.565	0.302	1.868	0.062	1.759	0.532	1.427	0.153	0.973	3.181
CCND3	-0.064	0.295	-0.215	0.83	0.938	0.277	-0.222	0.824	0.526	1.674
CCND2	-0.999	0.317	-3.153	0.002	0.368	0.117	-5.416	0	0.198	0.685
CCND1	-0.342	0.303	-1.129	0.259	0.711	0.215	-1.346	0.178	0.393	1.286
CCNC	0.136	0.297	0.46	0.646	1.146	0.34	0.43	0.667	0.641	2.05
CCNB2	0.874	0.315	2.778	0.005	2.397	0.754	1.852	0.064	1.294	4.442
CCNB1IP1	0.226	0.298	0.758	0.448	1.254	0.374	0.679	0.497	0.699	2.25

CCNB1	0.2	0.295	0.677	0.498	1.221	0.361	0.614	0.539	0.685	2.179
CCNA2	0.593	0.306	1.937	0.053	1.809	0.554	1.462	0.144	0.993	3.297
CCNA1	0.198	0.296	0.668	0.504	1.219	0.361	0.606	0.544	0.682	2.178
CCN6	0.319	0.304	1.051	0.293	1.376	0.418	0.9	0.368	0.759	2.495
CCN5	-0.466	0.302	-1.54	0.123	0.628	0.19	-1.962	0.05	0.347	1.135
CCN4	-1.392	0.346	-4.018	0	0.249	0.086	-8.726	0	0.126	0.49
CCN3	-0.48	0.3	-1.601	0.109	0.619	0.185	-2.054	0.04	0.344	1.114
CCN2	-0.57	0.306	-1.864	0.062	0.566	0.173	-2.513	0.012	0.311	1.03
CCN1	-0.003	0.296	-0.011	0.991	0.997	0.295	-0.011	0.991	0.558	1.781
CCL8	0.663	0.31	2.138	0.033	1.941	0.602	1.563	0.118	1.057	3.564
CCL7	0.027	0.295	0.092	0.927	1.027	0.303	0.09	0.928	0.576	1.833
CCL5	0.064	0.296	0.217	0.828	1.066	0.315	0.21	0.833	0.597	1.903
CCL4	-0.074	0.296	-0.249	0.803	0.929	0.275	-0.258	0.796	0.521	1.658
CCL27	-0.281	0.298	-0.943	0.346	0.755	0.225	-1.089	0.276	0.421	1.354
CCL25	0.414	0.3	1.383	0.167	1.513	0.453	1.132	0.258	0.841	2.722
CCL24	0.408	0.303	1.346	0.178	1.503	0.455	1.106	0.269	0.83	2.721
CCL23	-0.548	0.303	-1.812	0.07	0.578	0.175	-2.413	0.016	0.319	1.046
CCL22	-0.558	0.303	-1.842	0.066	0.573	0.173	-2.466	0.014	0.316	1.036
CCL21	-0.237	0.296	-0.8	0.424	0.789	0.234	-0.903	0.367	0.442	1.41
CCL20	-0.128	0.296	-0.431	0.666	0.88	0.261	-0.46	0.646	0.492	1.573
CCL2	0.031	0.296	0.104	0.917	1.031	0.305	0.103	0.918	0.578	1.841
CCL19	-0.275	0.297	-0.928	0.353	0.759	0.225	-1.069	0.285	0.425	1.358
CCL18	0.184	0.296	0.621	0.535	1.202	0.356	0.567	0.571	0.673	2.148
CCL17	0.553	0.302	1.828	0.067	1.738	0.526	1.405	0.16	0.961	3.145
CCL16	-0.553	0.303	-1.827	0.068	0.575	0.174	-2.441	0.015	0.318	1.041
CCL13	-0.477	0.3	-1.588	0.112	0.621	0.186	-2.035	0.042	0.345	1.118
CCL11	-0.416	0.3	-1.39	0.165	0.659	0.198	-1.724	0.085	0.367	1.186
CCL1	-0.219	0.298	-0.737	0.461	0.803	0.239	-0.825	0.41	0.448	1.439
CCKBR	0.307	0.298	1.032	0.302	1.36	0.405	0.889	0.374	0.759	2.438
CCKAR	0.138	0.296	0.468	0.639	1.149	0.339	0.437	0.662	0.643	2.05
CCK	-0.325	0.302	-1.078	0.281	0.722	0.218	-1.274	0.203	0.4	1.305
CCIN	-0.136	0.296	-0.46	0.645	0.873	0.258	-0.493	0.622	0.488	1.559
CCHCR1	0.291	0.298	0.977	0.329	1.338	0.398	0.847	0.397	0.746	2.398
CCDC93	0.142	0.298	0.475	0.635	1.152	0.344	0.443	0.658	0.642	2.068
CCDC92	-0.391	0.3	-1.305	0.192	0.676	0.203	-1.597	0.11	0.376	1.217
CCDC91	-0.437	0.306	-1.428	0.153	0.646	0.198	-1.791	0.073	0.354	1.177
CCDC90B	-0.036	0.296	-0.123	0.902	0.964	0.285	-0.125	0.901	0.54	1.722
CCDC9	0.223	0.299	0.746	0.456	1.25	0.374	0.668	0.504	0.695	2.248

CCDC88C	0.313	0.297	1.054	0.292	1.368	0.407	0.905	0.366	0.764	2.451
CCDC88A	0.462	0.303	1.525	0.127	1.587	0.48	1.221	0.222	0.877	2.872
CCDC87	-0.027	0.295	-0.09	0.928	0.974	0.288	-0.091	0.927	0.546	1.737
CCDC86	0.7	0.306	2.289	0.022	2.014	0.616	1.646	0.1	1.106	3.668
CCDC85C	0.531	0.302	1.757	0.079	1.701	0.515	1.363	0.173	0.94	3.077
CCDC85B	-0.011	0.296	-0.036	0.971	0.989	0.293	-0.037	0.971	0.554	1.767
CCDC82	-0.09	0.295	-0.303	0.762	0.914	0.27	-0.317	0.751	0.512	1.631
CCDC81	-0.131	0.296	-0.443	0.658	0.877	0.26	-0.473	0.636	0.491	1.567
CCDC71	0.14	0.295	0.475	0.635	1.151	0.34	0.443	0.658	0.645	2.053
CCDC70	0.357	0.298	1.198	0.231	1.429	0.426	1.007	0.314	0.797	2.564
CCDC7	-0.236	0.296	-0.798	0.425	0.789	0.234	-0.9	0.368	0.442	1.411
CCDC69	-0.525	0.306	-1.714	0.087	0.592	0.181	-2.254	0.024	0.325	1.078
CCDC68	-0.169	0.296	-0.571	0.568	0.844	0.25	-0.622	0.534	0.472	1.509
CCDC6	0.107	0.297	0.359	0.719	1.113	0.331	0.341	0.733	0.621	1.992
CCDC59	0.425	0.3	1.418	0.156	1.529	0.458	1.155	0.248	0.85	2.751
CCDC57	0.582	0.302	1.923	0.054	1.789	0.541	1.458	0.145	0.989	3.237
CCDC51	0.412	0.299	1.379	0.168	1.51	0.451	1.13	0.258	0.841	2.713
CCDC47	-0.153	0.297	-0.514	0.607	0.858	0.255	-0.556	0.578	0.48	1.536
CCDC40	-0.16	0.296	-0.54	0.589	0.852	0.252	-0.585	0.559	0.477	1.522
CCDC33	0.275	0.298	0.923	0.356	1.316	0.392	0.807	0.42	0.734	2.359
CCDC30	0.605	0.306	1.98	0.048	1.832	0.56	1.485	0.137	1.006	3.335
CCDC28B	0.313	0.298	1.052	0.293	1.368	0.407	0.903	0.366	0.763	2.452
CCDC28A	-0.207	0.298	-0.694	0.488	0.813	0.242	-0.771	0.441	0.453	1.459
CCDC25	-0.36	0.3	-1.201	0.23	0.698	0.209	-1.446	0.148	0.388	1.255
CCDC22	0.164	0.296	0.553	0.58	1.178	0.349	0.51	0.61	0.659	2.105
CCDC198	-0.224	0.298	-0.752	0.452	0.8	0.238	-0.842	0.4	0.446	1.433
CCDC186	-0.087	0.295	-0.295	0.768	0.916	0.271	-0.309	0.758	0.514	1.635
CCDC181	0.351	0.298	1.178	0.239	1.42	0.423	0.993	0.321	0.792	2.545
CCDC177	-0.126	0.298	-0.424	0.672	0.881	0.263	-0.452	0.651	0.491	1.582
CCDC170	-0.474	0.306	-1.546	0.122	0.623	0.191	-1.977	0.048	0.342	1.135
CCDC15	0.178	0.296	0.603	0.547	1.195	0.354	0.552	0.581	0.669	2.136
CCDC144A	0.249	0.296	0.842	0.4	1.283	0.38	0.745	0.456	0.718	2.294
CCDC134	0.509	0.301	1.691	0.091	1.663	0.5	1.325	0.185	0.922	2.998
CCDC130	-0.108	0.297	-0.365	0.715	0.897	0.267	-0.385	0.7	0.501	1.606
CCDC121	-0.211	0.297	-0.713	0.476	0.809	0.24	-0.794	0.427	0.453	1.447
CCDC106	-0.252	0.301	-0.835	0.404	0.778	0.234	-0.949	0.343	0.431	1.404
CCDC102B	-0.208	0.296	-0.703	0.482	0.812	0.241	-0.781	0.435	0.454	1.451
CCAR2	0.079	0.296	0.267	0.789	1.082	0.32	0.257	0.797	0.606	1.931

CC2D1A	0.306	0.298	1.027	0.304	1.358	0.405	0.885	0.376	0.757	2.438
CBY1	-0.109	0.295	-0.369	0.712	0.897	0.265	-0.39	0.697	0.503	1.6
CBX8	-0.273	0.298	-0.917	0.359	0.761	0.227	-1.054	0.292	0.425	1.364
CBX7	-0.41	0.3	-1.369	0.171	0.664	0.199	-1.693	0.091	0.369	1.194
CBX6	-0.231	0.296	-0.781	0.435	0.794	0.235	-0.878	0.38	0.444	1.418
CBX5	-0.735	0.31	-2.373	0.018	0.479	0.149	-3.506	0	0.261	0.88
CBX4	-0.189	0.296	-0.639	0.523	0.828	0.245	-0.703	0.482	0.463	1.479
CBX3	0.461	0.3	1.538	0.124	1.586	0.475	1.232	0.218	0.881	2.853
CBX2	0.34	0.298	1.144	0.253	1.406	0.418	0.97	0.332	0.784	2.519
CBX1	0.846	0.315	2.689	0.007	2.331	0.734	1.814	0.07	1.258	4.319
CBS	-0.019	0.295	-0.065	0.949	0.981	0.29	-0.065	0.948	0.55	1.75
CBR4	-0.356	0.298	-1.194	0.233	0.701	0.209	-1.434	0.152	0.391	1.257
CBR3	0.195	0.298	0.656	0.512	1.216	0.362	0.596	0.551	0.678	2.181
CBR1	0.09	0.297	0.303	0.762	1.094	0.324	0.29	0.772	0.612	1.957
CBLN1	0.236	0.298	0.792	0.428	1.266	0.378	0.706	0.48	0.706	2.272
CBLL1	-0.041	0.295	-0.139	0.89	0.96	0.283	-0.142	0.887	0.538	1.712
CBLIF	0.143	0.295	0.485	0.628	1.154	0.341	0.452	0.651	0.647	2.058
CBLC	-0.244	0.3	-0.813	0.416	0.783	0.235	-0.921	0.357	0.435	1.411
CBLB	-0.035	0.295	-0.118	0.906	0.966	0.285	-0.12	0.904	0.542	1.722
CBL	0.294	0.301	0.977	0.329	1.342	0.404	0.846	0.397	0.744	2.423
CBFB	0.325	0.298	1.093	0.274	1.384	0.412	0.933	0.351	0.773	2.481
CBFA2T3	-0.002	0.296	-0.008	0.994	0.998	0.296	-0.008	0.993	0.558	1.784
CBFA2T2	-0.225	0.297	-0.759	0.448	0.798	0.237	-0.851	0.395	0.446	1.428
CBARP	0.153	0.295	0.519	0.604	1.166	0.344	0.481	0.63	0.653	2.079
CAVIN3	-0.201	0.296	-0.68	0.497	0.818	0.242	-0.753	0.452	0.457	1.461
CAVIN2	-0.327	0.3	-1.093	0.275	0.721	0.216	-1.293	0.196	0.401	1.297
CAVIN1	-0.949	0.316	-3.003	0.003	0.387	0.122	-5.01	0	0.208	0.719
CAV3	-0.28	0.298	-0.938	0.348	0.756	0.225	-1.083	0.279	0.422	1.356
CAV2	-0.87	0.311	-2.798	0.005	0.419	0.13	-4.462	0	0.228	0.77
CAV1	-0.458	0.3	-1.525	0.127	0.633	0.19	-1.934	0.053	0.351	1.14
CATSPERG	0.266	0.298	0.894	0.371	1.305	0.389	0.785	0.432	0.728	2.341
CATSPERB	-0.392	0.3	-1.307	0.191	0.675	0.203	-1.601	0.109	0.375	1.216
CATR1	0.521	0.302	1.723	0.085	1.684	0.509	1.343	0.179	0.931	3.047
CAT	-0.166	0.295	-0.561	0.575	0.847	0.25	-0.61	0.542	0.475	1.512
CASZ1	0.252	0.298	0.848	0.397	1.287	0.383	0.749	0.454	0.718	2.307
CAST	-0.94	0.321	-2.931	0.003	0.391	0.125	-4.864	0	0.208	0.732
CASS4	0.07	0.295	0.239	0.811	1.073	0.317	0.231	0.818	0.602	1.913
CASR	-0.103	0.296	-0.35	0.726	0.902	0.267	-0.369	0.712	0.505	1.609

CASQ2	-0.41	0.3	-1.367	0.171	0.664	0.199	-1.69	0.091	0.369	1.194
CASQ1	-0.376	0.298	-1.261	0.207	0.687	0.205	-1.531	0.126	0.383	1.231
CASP9	-0.843	0.312	-2.702	0.007	0.431	0.134	-4.241	0	0.234	0.793
CASP8AP2	0.549	0.302	1.816	0.069	1.732	0.524	1.397	0.162	0.957	3.132
CASP8	-0.298	0.296	-1.005	0.315	0.742	0.22	-1.171	0.242	0.415	1.327
CASP7	0.356	0.3	1.189	0.234	1.428	0.428	1	0.317	0.794	2.57
CASP6	-0.126	0.296	-0.424	0.671	0.882	0.261	-0.452	0.651	0.494	1.576
CASP5	-0.345	0.3	-1.151	0.25	0.708	0.212	-1.375	0.169	0.394	1.274
CASP4LP	0.459	0.3	1.53	0.126	1.582	0.474	1.227	0.22	0.879	2.848
CASP4	0.106	0.296	0.359	0.72	1.112	0.329	0.341	0.733	0.623	1.985
CASP3	0.489	0.302	1.617	0.106	1.631	0.493	1.279	0.201	0.902	2.95
CASP2	-0.259	0.299	-0.866	0.386	0.772	0.231	-0.989	0.323	0.43	1.386
CASP10	0.538	0.302	1.78	0.075	1.713	0.518	1.377	0.169	0.947	3.098
CASP1	-0.173	0.296	-0.583	0.56	0.841	0.249	-0.637	0.524	0.47	1.504
CASKIN2	-0.21	0.296	-0.708	0.479	0.811	0.24	-0.788	0.431	0.454	1.449
CASK	-0.659	0.307	-2.146	0.032	0.517	0.159	-3.038	0.002	0.283	0.944
CASD1	-0.532	0.306	-1.738	0.082	0.587	0.18	-2.294	0.022	0.322	1.07
CARTPT	0.133	0.296	0.451	0.652	1.143	0.338	0.422	0.673	0.64	2.039
CARS2	0.489	0.3	1.628	0.104	1.631	0.49	1.287	0.198	0.905	2.939
CARS1	0.85	0.311	2.737	0.006	2.34	0.727	1.844	0.065	1.273	4.301
CARMIL1	0.101	0.296	0.341	0.733	1.106	0.328	0.325	0.745	0.619	1.977
CARHSP1	0.237	0.296	0.798	0.425	1.267	0.375	0.711	0.477	0.709	2.264
CARF	-0.334	0.3	-1.113	0.266	0.716	0.215	-1.322	0.186	0.398	1.289
CARD8	-0.308	0.298	-1.033	0.302	0.735	0.219	-1.21	0.226	0.41	1.318
CARD14	-0.175	0.296	-0.592	0.554	0.839	0.248	-0.647	0.517	0.47	1.498
CARD10	-0.207	0.296	-0.698	0.485	0.813	0.241	-0.775	0.438	0.455	1.453
CAPZB	-0.741	0.306	-2.419	0.016	0.477	0.146	-3.584	0	0.261	0.869
CAPZA2	-0.297	0.3	-0.992	0.321	0.743	0.223	-1.155	0.248	0.413	1.336
CAPZA1	0.418	0.302	1.383	0.167	1.519	0.459	1.131	0.258	0.84	2.746
CAPRIN2	-0.196	0.297	-0.659	0.51	0.822	0.244	-0.729	0.466	0.459	1.472
CAPRIN1	-0.188	0.296	-0.634	0.526	0.829	0.246	-0.697	0.486	0.464	1.481
CAPNS1	-0.411	0.302	-1.36	0.174	0.663	0.2	-1.682	0.093	0.366	1.199
CAPN9	-0.235	0.296	-0.795	0.427	0.79	0.234	-0.896	0.37	0.442	1.412
CAPN7	-0.78	0.315	-2.475	0.013	0.459	0.144	-3.749	0	0.247	0.85
CAPN6	-0.043	0.295	-0.147	0.883	0.958	0.283	-0.15	0.881	0.537	1.707
CAPN5	-0.398	0.3	-1.328	0.184	0.671	0.201	-1.631	0.103	0.373	1.209
CAPN3	0.174	0.302	0.577	0.564	1.19	0.359	0.529	0.596	0.659	2.149
CAPN2	0.424	0.3	1.414	0.157	1.528	0.458	1.153	0.249	0.849	2.751

CAPN15	0.122	0.295	0.413	0.68	1.13	0.334	0.389	0.698	0.633	2.016
CAPN11	-0.03	0.296	-0.102	0.919	0.97	0.288	-0.103	0.918	0.543	1.735
CAPN10	-0.377	0.3	-1.259	0.208	0.686	0.205	-1.529	0.126	0.381	1.234
CAPN1	0.088	0.298	0.295	0.768	1.092	0.325	0.282	0.778	0.609	1.956
CAPG	0.645	0.304	2.123	0.034	1.906	0.579	1.564	0.118	1.051	3.458
CAP2	0.022	0.295	0.073	0.942	1.022	0.302	0.072	0.942	0.573	1.823
CAP1	-0.279	0.298	-0.936	0.349	0.757	0.225	-1.08	0.28	0.422	1.356
CANX	-0.337	0.298	-1.131	0.258	0.714	0.213	-1.345	0.179	0.398	1.281
CANT1	0.706	0.31	2.277	0.023	2.026	0.629	1.633	0.103	1.103	3.722
CAND2	0.442	0.299	1.479	0.139	1.556	0.465	1.195	0.232	0.866	2.796
CAND1	0.401	0.298	1.345	0.179	1.493	0.445	1.108	0.268	0.833	2.676
CAMTA2	0.015	0.295	0.051	0.959	1.015	0.299	0.051	0.959	0.569	1.81
CAMTA1	-0.473	0.302	-1.564	0.118	0.623	0.188	-2	0.046	0.345	1.127
CAMSAP2	0.31	0.3	1.035	0.301	1.364	0.409	0.89	0.374	0.758	2.455
CAMSAP1	-0.03	0.296	-0.101	0.919	0.97	0.287	-0.103	0.918	0.544	1.732
CAMP	0.492	0.302	1.627	0.104	1.635	0.494	1.285	0.199	0.904	2.956
CAMLG	-0.281	0.3	-0.936	0.349	0.755	0.227	-1.08	0.28	0.42	1.36
CAMKV	-0.03	0.295	-0.102	0.919	0.97	0.287	-0.104	0.917	0.544	1.731
CAMKMT	-0.424	0.302	-1.403	0.16	0.654	0.198	-1.748	0.08	0.362	1.183
CAMKK2	0.539	0.306	1.758	0.079	1.714	0.525	1.359	0.174	0.94	3.126
CAMK4	0.013	0.295	0.043	0.966	1.013	0.299	0.042	0.966	0.568	1.806
CAMK2N1	-0.183	0.296	-0.616	0.538	0.833	0.247	-0.676	0.499	0.466	1.489
CAMK2G	-0.229	0.3	-0.763	0.445	0.795	0.239	-0.857	0.391	0.442	1.432
CAMK2B	0.229	0.298	0.767	0.443	1.257	0.375	0.685	0.493	0.701	2.254
CAMK2A	0.246	0.298	0.825	0.409	1.278	0.38	0.732	0.464	0.713	2.29
CAMK1G	-0.07	0.295	-0.238	0.812	0.932	0.275	-0.247	0.805	0.522	1.663
CAMK1D	0.291	0.3	0.971	0.332	1.338	0.402	0.842	0.4	0.743	2.409
CAMK1	-0.375	0.3	-1.25	0.211	0.687	0.206	-1.516	0.129	0.382	1.237
CALY	-0.134	0.295	-0.454	0.65	0.874	0.258	-0.486	0.627	0.49	1.56
CALU	-0.272	0.296	-0.917	0.359	0.762	0.226	-1.053	0.292	0.426	1.362
CALR	0.089	0.297	0.3	0.764	1.093	0.325	0.287	0.774	0.611	1.956
CALML5	0.23	0.298	0.774	0.439	1.259	0.375	0.691	0.49	0.702	2.256
CALML4	0.204	0.296	0.689	0.491	1.227	0.363	0.624	0.533	0.686	2.192
CALML3	0.043	0.295	0.145	0.885	1.044	0.308	0.142	0.887	0.585	1.862
CALM1	-0.199	0.296	-0.67	0.503	0.82	0.243	-0.741	0.459	0.459	1.466
CALHM2	-0.899	0.316	-2.849	0.004	0.407	0.128	-4.618	0	0.219	0.755
CALD1	-0.635	0.304	-2.088	0.037	0.53	0.161	-2.917	0.004	0.292	0.962
CALCRL	-0.3	0.297	-1.013	0.311	0.74	0.22	-1.182	0.237	0.414	1.324

CALCR	-0.075	0.296	-0.254	0.799	0.928	0.274	-0.264	0.792	0.52	1.656
CALCOCO2	-0.035	0.295	-0.118	0.906	0.966	0.285	-0.12	0.905	0.542	1.723
CALCOCO1	-0.786	0.311	-2.526	0.012	0.456	0.142	-3.839	0	0.248	0.839
CALCB	0.007	0.295	0.024	0.981	1.007	0.298	0.024	0.981	0.564	1.797
CALCA	0.093	0.299	0.312	0.755	1.098	0.328	0.298	0.766	0.611	1.97
CALB2	0.268	0.298	0.901	0.367	1.308	0.389	0.791	0.429	0.73	2.344
CALB1	0.128	0.295	0.433	0.665	1.136	0.336	0.406	0.685	0.637	2.027
CADPS2	-0.652	0.306	-2.132	0.033	0.521	0.159	-3.005	0.003	0.286	0.949
CADPS	0.227	0.298	0.76	0.447	1.255	0.374	0.68	0.497	0.699	2.252
CADM4	0.363	0.298	1.217	0.224	1.438	0.429	1.021	0.307	0.801	2.579
CADM3-AS1	0.165	0.295	0.557	0.577	1.179	0.348	0.514	0.607	0.661	2.103
CADM3	-0.458	0.301	-1.521	0.128	0.633	0.19	-1.929	0.054	0.351	1.141
CADM1	-0.376	0.298	-1.263	0.207	0.687	0.204	-1.533	0.125	0.383	1.231
CAD	-0.337	0.297	-1.135	0.256	0.714	0.212	-1.349	0.177	0.399	1.278
CACYBP	0.225	0.298	0.756	0.45	1.252	0.373	0.677	0.499	0.699	2.245
CACTIN	-0.019	0.295	-0.063	0.95	0.982	0.289	-0.064	0.949	0.551	1.75
CACNG5	0.421	0.3	1.405	0.16	1.524	0.457	1.147	0.252	0.847	2.742
CACNG4	-0.194	0.296	-0.655	0.512	0.824	0.244	-0.723	0.47	0.461	1.471
CACNG3	-0.112	0.296	-0.379	0.705	0.894	0.265	-0.401	0.689	0.5	1.598
CACNG2	-0.052	0.295	-0.176	0.86	0.949	0.28	-0.18	0.857	0.532	1.694
CACNG1	0.096	0.295	0.327	0.744	1.101	0.325	0.312	0.755	0.617	1.964
CACNB4	-0.202	0.297	-0.681	0.496	0.817	0.243	-0.754	0.451	0.457	1.462
CACNB3	0.681	0.304	2.24	0.025	1.976	0.601	1.624	0.104	1.089	3.586
CACNB2	-0.125	0.296	-0.422	0.673	0.883	0.261	-0.449	0.653	0.495	1.576
CACNB1	0.121	0.297	0.407	0.684	1.128	0.335	0.383	0.702	0.631	2.018
CACNA2D3	-0.405	0.3	-1.349	0.177	0.667	0.2	-1.663	0.096	0.371	1.201
CACNA2D2	-0.057	0.297	-0.191	0.849	0.945	0.281	-0.196	0.844	0.528	1.692
CACNA2D1	0.25	0.298	0.839	0.401	1.284	0.382	0.742	0.458	0.716	2.302
CACNA1S	0.235	0.297	0.793	0.428	1.265	0.375	0.706	0.48	0.707	2.262
CACNA1I	0.059	0.295	0.2	0.842	1.061	0.313	0.194	0.846	0.595	1.893
CACNA1H	-0.439	0.3	-1.46	0.144	0.645	0.194	-1.832	0.067	0.358	1.162
CACNA1G	0.224	0.299	0.751	0.453	1.251	0.374	0.672	0.501	0.697	2.246
CACNA1F	-0.014	0.295	-0.047	0.963	0.986	0.291	-0.047	0.962	0.553	1.758
CACNA1E	-0.15	0.295	-0.509	0.61	0.86	0.254	-0.55	0.582	0.482	1.535
CACNA1D	-0.369	0.3	-1.23	0.219	0.691	0.208	-1.488	0.137	0.384	1.245
CACNA1C	-0.595	0.306	-1.946	0.052	0.552	0.169	-2.659	0.008	0.303	1.004
CACNA1B	0.368	0.298	1.236	0.216	1.445	0.43	1.034	0.301	0.806	2.59
CACNA1A	-0.198	0.296	-0.668	0.504	0.821	0.243	-0.739	0.46	0.46	1.465

CACFD1	0.312	0.298	1.048	0.295	1.366	0.407	0.9	0.368	0.762	2.449
CABYR	-0.44	0.311	-1.412	0.158	0.644	0.201	-1.773	0.076	0.35	1.186
CABP5	0.144	0.296	0.486	0.627	1.155	0.342	0.453	0.651	0.646	2.065
CABP2	0.217	0.297	0.731	0.465	1.242	0.369	0.657	0.511	0.695	2.222
CABP1	-0.087	0.297	-0.295	0.768	0.916	0.272	-0.308	0.758	0.512	1.639
CABIN1	-0.813	0.311	-2.61	0.009	0.444	0.138	-4.027	0	0.241	0.817
CAB39L	-0.718	0.315	-2.281	0.023	0.488	0.154	-3.337	0.001	0.263	0.904
CAB39	-0.459	0.3	-1.531	0.126	0.632	0.189	-1.942	0.052	0.351	1.137
CAAP1	0.227	0.297	0.763	0.445	1.255	0.373	0.683	0.495	0.701	2.246
CA9	-0.088	0.296	-0.298	0.765	0.915	0.271	-0.312	0.755	0.513	1.635
CA8	-0.179	0.296	-0.604	0.546	0.836	0.248	-0.662	0.508	0.468	1.494
CA7	-0.151	0.295	-0.511	0.609	0.86	0.254	-0.551	0.581	0.482	1.534
CA6	0.043	0.296	0.144	0.885	1.044	0.308	0.141	0.888	0.585	1.863
CA5BP1	0.105	0.296	0.355	0.722	1.111	0.329	0.337	0.736	0.622	1.985
CA5B	0.069	0.295	0.234	0.815	1.072	0.316	0.227	0.821	0.601	1.911
CA5A	0.065	0.295	0.222	0.825	1.068	0.315	0.215	0.83	0.599	1.904
CA4	0.224	0.296	0.756	0.45	1.251	0.371	0.677	0.498	0.7	2.236
CA3	-0.022	0.296	-0.075	0.94	0.978	0.289	-0.076	0.94	0.548	1.746
CA2	0.028	0.295	0.096	0.924	1.029	0.304	0.095	0.925	0.577	1.835
CA14	0.366	0.3	1.222	0.222	1.442	0.432	1.023	0.306	0.801	2.596
CA12	0.065	0.297	0.22	0.826	1.067	0.317	0.213	0.831	0.597	1.909
CA11	0.113	0.296	0.38	0.704	1.119	0.331	0.36	0.719	0.627	1.998
CA10	-0.197	0.296	-0.667	0.505	0.821	0.243	-0.737	0.461	0.459	1.467
CA1	0.497	0.3	1.659	0.097	1.644	0.493	1.307	0.191	0.914	2.959
C9orf78	-0.479	0.302	-1.584	0.113	0.619	0.187	-2.032	0.042	0.342	1.12
C9orf40	0.191	0.295	0.647	0.517	1.211	0.358	0.589	0.556	0.679	2.16
C9orf16	-0.022	0.296	-0.075	0.941	0.978	0.29	-0.075	0.94	0.548	1.747
C9orf116	-0.243	0.298	-0.815	0.415	0.784	0.234	-0.923	0.356	0.438	1.406
C9	-0.168	0.298	-0.565	0.572	0.845	0.252	-0.615	0.539	0.471	1.516
C8orf44	0.353	0.303	1.166	0.244	1.423	0.431	0.982	0.326	0.786	2.576
C8orf33	0.635	0.306	2.079	0.038	1.887	0.577	1.539	0.124	1.037	3.435
C8orf17	-0.271	0.298	-0.911	0.362	0.762	0.227	-1.047	0.295	0.426	1.366
C8G	0.45	0.3	1.5	0.134	1.568	0.471	1.208	0.227	0.871	2.824
C8B	0.049	0.296	0.164	0.87	1.05	0.31	0.16	0.873	0.588	1.874
C8A	0.141	0.296	0.476	0.634	1.151	0.34	0.444	0.657	0.645	2.055
C7orf69	0.622	0.305	2.041	0.041	1.862	0.567	1.52	0.128	1.025	3.382
C7orf26	0.081	0.296	0.273	0.785	1.084	0.321	0.262	0.793	0.607	1.936
C7	-0.784	0.31	-2.531	0.011	0.457	0.141	-3.842	0	0.249	0.838

C6orf62	0.423	0.308	1.375	0.169	1.526	0.47	1.121	0.262	0.835	2.789
C6orf47	0.423	0.306	1.382	0.167	1.527	0.467	1.127	0.26	0.838	2.782
C6orf15	0.719	0.306	2.35	0.019	2.053	0.628	1.676	0.094	1.127	3.74
C6orf120	-0.109	0.296	-0.37	0.712	0.896	0.265	-0.391	0.696	0.502	1.6
C6	-0.267	0.296	-0.9	0.368	0.766	0.227	-1.031	0.302	0.428	1.369
C5orf66	0.16	0.296	0.54	0.589	1.173	0.347	0.499	0.618	0.657	2.097
C5orf22	0.487	0.3	1.623	0.104	1.627	0.488	1.285	0.199	0.904	2.93
C5orf15	-0.129	0.296	-0.435	0.664	0.879	0.261	-0.464	0.643	0.492	1.572
C5AR2	0.047	0.297	0.159	0.874	1.048	0.311	0.155	0.877	0.586	1.877
C5AR1	0.037	0.295	0.125	0.901	1.037	0.306	0.122	0.903	0.582	1.85
C5	-0.191	0.296	-0.644	0.519	0.826	0.245	-0.71	0.478	0.462	1.477
C4orf19	0.493	0.303	1.628	0.104	1.637	0.495	1.285	0.199	0.904	2.962
C4BPB	0.306	0.298	1.03	0.303	1.359	0.404	0.887	0.375	0.758	2.434
C4BPA	0.119	0.298	0.399	0.69	1.126	0.336	0.376	0.707	0.628	2.021
C3orf52	0.247	0.298	0.829	0.407	1.28	0.381	0.735	0.462	0.714	2.293
C3orf36	-0.355	0.3	-1.183	0.237	0.701	0.21	-1.419	0.156	0.39	1.263
C3orf18	-0.474	0.306	-1.55	0.121	0.622	0.191	-1.983	0.047	0.341	1.134
C3orf14	0.303	0.3	1.011	0.312	1.354	0.406	0.872	0.383	0.753	2.435
C3AR1	-0.337	0.3	-1.125	0.261	0.714	0.214	-1.338	0.181	0.396	1.285
C3	-1.005	0.321	-3.126	0.002	0.366	0.118	-5.386	0	0.195	0.687
C2orf83	0.174	0.297	0.585	0.559	1.19	0.353	0.537	0.591	0.665	2.129
C2orf72	0.042	0.297	0.141	0.888	1.043	0.31	0.138	0.891	0.582	1.867
C2orf68	-0.39	0.3	-1.302	0.193	0.677	0.203	-1.592	0.111	0.376	1.218
C2orf49	0.499	0.301	1.656	0.098	1.647	0.496	1.304	0.192	0.913	2.971
C2orf42	-0.294	0.3	-0.98	0.327	0.745	0.223	-1.14	0.254	0.414	1.341
C2orf27A	0.45	0.302	1.488	0.137	1.568	0.474	1.198	0.231	0.867	2.836
C2CD5	-0.328	0.303	-1.085	0.278	0.72	0.218	-1.284	0.199	0.398	1.303
C2CD3	0.51	0.306	1.668	0.095	1.665	0.509	1.306	0.191	0.914	3.033
C2CD2L	-0.393	0.299	-1.313	0.189	0.675	0.202	-1.609	0.108	0.375	1.214
C2CD2	0.391	0.3	1.305	0.192	1.479	0.444	1.08	0.28	0.822	2.662
C22orf46	-0.386	0.298	-1.296	0.195	0.68	0.202	-1.581	0.114	0.379	1.219
C22orf31	0.284	0.297	0.956	0.339	1.328	0.394	0.832	0.405	0.742	2.376
C22orf24	-0.209	0.297	-0.704	0.482	0.811	0.241	-0.783	0.434	0.453	1.453
C21orf91	0.387	0.298	1.297	0.195	1.472	0.439	1.076	0.282	0.821	2.641
C21orf62	-0.153	0.297	-0.516	0.606	0.858	0.254	-0.557	0.577	0.48	1.535
C20orf27	-0.207	0.298	-0.696	0.487	0.813	0.242	-0.773	0.44	0.453	1.457
C2	-0.049	0.296	-0.165	0.869	0.952	0.282	-0.169	0.866	0.534	1.7
C1S	-0.815	0.31	-2.625	0.009	0.443	0.137	-4.055	0	0.241	0.813

C1RL	-0.006	0.297	-0.021	0.983	0.994	0.295	-0.021	0.983	0.556	1.777
C1R	-0.853	0.311	-2.745	0.006	0.426	0.132	-4.334	0	0.232	0.784
C1QTNF3	-0.524	0.302	-1.733	0.083	0.592	0.179	-2.279	0.023	0.327	1.071
C1QTNF1	-0.14	0.296	-0.472	0.637	0.869	0.258	-0.507	0.612	0.486	1.554
C1QL1	0.814	0.31	2.623	0.009	2.257	0.7	1.795	0.073	1.228	4.147
C1QBP	-0.21	0.298	-0.706	0.48	0.811	0.241	-0.786	0.432	0.452	1.452
C1QB	0.234	0.296	0.79	0.43	1.264	0.374	0.704	0.481	0.707	2.258
C1QA	0.222	0.296	0.75	0.453	1.248	0.369	0.673	0.501	0.699	2.228
C1orf68	0.651	0.31	2.101	0.036	1.918	0.594	1.544	0.123	1.045	3.521
C1orf56	-0.143	0.296	-0.484	0.629	0.866	0.257	-0.52	0.603	0.485	1.549
C1orf54	-0.655	0.306	-2.141	0.032	0.519	0.159	-3.025	0.002	0.285	0.946
C1orf50	0.381	0.298	1.278	0.201	1.464	0.437	1.063	0.288	0.816	2.627
C1orf35	0.737	0.31	2.38	0.017	2.09	0.647	1.684	0.092	1.139	3.834
C1orf216	-0.152	0.296	-0.512	0.609	0.859	0.255	-0.553	0.58	0.481	1.536
C1orf21	-0.403	0.302	-1.332	0.183	0.669	0.202	-1.64	0.101	0.37	1.209
C1orf174	0.568	0.306	1.859	0.063	1.764	0.539	1.418	0.156	0.97	3.211
C1orf159	-0.273	0.298	-0.914	0.361	0.761	0.227	-1.051	0.293	0.424	1.366
C1orf116	0.524	0.303	1.732	0.083	1.689	0.511	1.348	0.178	0.933	3.057
C1orf115	-0.229	0.296	-0.772	0.44	0.796	0.235	-0.868	0.385	0.446	1.421
C1orf112	0.183	0.295	0.621	0.534	1.201	0.355	0.568	0.57	0.673	2.143
C1orf109	0.116	0.297	0.391	0.696	1.123	0.333	0.369	0.712	0.628	2.008
C1orf105	0.18	0.296	0.607	0.544	1.197	0.355	0.556	0.578	0.67	2.141
C1GALT1C1	0.007	0.295	0.025	0.98	1.007	0.298	0.025	0.98	0.565	1.798
C1GALT1	0.983	0.321	3.067	0.002	2.674	0.857	1.952	0.051	1.426	5.012
C1D	0.231	0.296	0.78	0.435	1.26	0.373	0.696	0.486	0.705	2.249
C19orf73	0.323	0.298	1.084	0.278	1.381	0.411	0.927	0.354	0.77	2.476
C19orf54	0.107	0.296	0.36	0.719	1.112	0.329	0.342	0.733	0.623	1.987
C19orf53	-0.292	0.298	-0.98	0.327	0.747	0.222	-1.138	0.255	0.417	1.339
C18orf25	0.428	0.303	1.414	0.157	1.535	0.465	1.15	0.25	0.847	2.78
C17orf80	-0.223	0.298	-0.747	0.455	0.8	0.238	-0.837	0.402	0.447	1.435
C17orf75	0.491	0.3	1.635	0.102	1.634	0.491	1.292	0.196	0.907	2.944
C16orf72	0.385	0.3	1.281	0.2	1.47	0.442	1.064	0.288	0.816	2.648
C16orf71	-0.619	0.303	-2.042	0.041	0.538	0.163	-2.827	0.005	0.297	0.976
C16orf70	-0.03	0.295	-0.1	0.92	0.971	0.287	-0.102	0.919	0.544	1.732
C15orf39	-0.001	0.296	-0.003	0.998	0.999	0.296	-0.003	0.998	0.559	1.786
C14orf93	0.038	0.296	0.127	0.899	1.038	0.308	0.125	0.901	0.581	1.856
C14orf132	-0.012	0.295	-0.042	0.966	0.988	0.292	-0.042	0.966	0.554	1.762
C12orf43	-0.249	0.3	-0.83	0.407	0.78	0.234	-0.942	0.346	0.433	1.403

C12orf4	0.372	0.298	1.248	0.212	1.451	0.433	1.042	0.297	0.809	2.604
C12orf29	-0.661	0.316	-2.092	0.036	0.516	0.163	-2.964	0.003	0.278	0.959
C11orf95	0.828	0.321	2.579	0.01	2.288	0.734	1.755	0.079	1.22	4.29
C11orf80	0.322	0.296	1.086	0.277	1.38	0.409	0.929	0.353	0.772	2.467
C11orf71	0.011	0.295	0.038	0.97	1.011	0.298	0.038	0.97	0.567	1.803
C11orf68	-0.179	0.296	-0.604	0.546	0.836	0.247	-0.662	0.508	0.469	1.493
C11orf58	0.061	0.296	0.205	0.838	1.062	0.314	0.199	0.842	0.595	1.896
C11orf49	0.571	0.302	1.893	0.058	1.77	0.534	1.442	0.149	0.98	3.196
C11orf24	0.01	0.295	0.033	0.973	1.01	0.298	0.033	0.973	0.566	1.802
C11orf21	-0.289	0.297	-0.974	0.33	0.749	0.222	-1.129	0.259	0.419	1.34
C11orf16	-0.13	0.295	-0.441	0.659	0.878	0.259	-0.471	0.638	0.492	1.566
C11orf1	-0.026	0.298	-0.088	0.93	0.974	0.291	-0.089	0.929	0.543	1.749
C10orf95	0.075	0.298	0.252	0.801	1.078	0.322	0.243	0.808	0.601	1.935
C10orf88	0.43	0.3	1.434	0.152	1.537	0.46	1.166	0.244	0.854	2.764
BZW2	0.761	0.31	2.455	0.014	2.139	0.663	1.719	0.086	1.166	3.927
BZW1	-0.702	0.307	-2.289	0.022	0.495	0.152	-3.32	0.001	0.271	0.904
BYSL	0.746	0.31	2.408	0.016	2.109	0.653	1.697	0.09	1.149	3.87
BUD31	0.232	0.299	0.777	0.437	1.262	0.377	0.694	0.488	0.702	2.266
BUD23	0.351	0.297	1.18	0.238	1.42	0.422	0.995	0.32	0.793	2.543
BUB3	-0.185	0.298	-0.621	0.535	0.831	0.247	-0.682	0.495	0.464	1.489
BUB1B	0.545	0.3	1.818	0.069	1.724	0.517	1.402	0.161	0.958	3.103
BUB1	0.795	0.315	2.526	0.012	2.215	0.697	1.742	0.081	1.195	4.106
BTRC	-0.299	0.297	-1.006	0.315	0.741	0.221	-1.172	0.241	0.414	1.328
BTNL8	-0.005	0.295	-0.016	0.987	0.995	0.294	-0.017	0.987	0.558	1.774
BTNL3	0.629	0.31	2.026	0.043	1.875	0.582	1.504	0.133	1.021	3.446
BTNL2	0.272	0.297	0.914	0.361	1.313	0.39	0.801	0.423	0.733	2.351
BTN3A3	-0.512	0.3	-1.706	0.088	0.599	0.18	-2.228	0.026	0.333	1.079
BTN3A2	-0.618	0.303	-2.04	0.041	0.539	0.163	-2.824	0.005	0.298	0.976
BTN3A1	-0.048	0.296	-0.164	0.87	0.953	0.282	-0.168	0.867	0.534	1.701
BTN2A3P	0.12	0.296	0.407	0.684	1.128	0.333	0.383	0.701	0.632	2.013
BTN2A2	0.534	0.303	1.764	0.078	1.707	0.517	1.367	0.172	0.942	3.09
BTN2A1	0.278	0.3	0.927	0.354	1.321	0.397	0.809	0.418	0.733	2.38
BTN1A1	-0.162	0.296	-0.547	0.584	0.851	0.252	-0.594	0.553	0.476	1.519
BTK	-0.284	0.298	-0.954	0.34	0.753	0.224	-1.103	0.27	0.42	1.349
BTG4	0.46	0.3	1.533	0.125	1.584	0.475	1.229	0.219	0.88	2.851
BTG3	0.143	0.295	0.485	0.628	1.154	0.341	0.452	0.652	0.647	2.058
BTG2	-0.591	0.303	-1.95	0.051	0.554	0.168	-2.658	0.008	0.306	1.003
BTG1	-0.35	0.297	-1.177	0.239	0.705	0.209	-1.409	0.159	0.394	1.262

BTF3P11	0.523	0.303	1.726	0.084	1.687	0.511	1.344	0.179	0.932	3.054
BTF3	-0.592	0.317	-1.864	0.062	0.553	0.176	-2.543	0.011	0.297	1.031
BTD	-0.885	0.321	-2.758	0.006	0.413	0.132	-4.435	0	0.22	0.774
BTC	0.024	0.295	0.083	0.934	1.025	0.302	0.082	0.935	0.575	1.827
BTBD7	0.076	0.296	0.256	0.798	1.079	0.319	0.247	0.805	0.604	1.927
BTBD3	0.381	0.3	1.267	0.205	1.463	0.439	1.054	0.292	0.812	2.636
BTBD2	-0.269	0.301	-0.894	0.371	0.764	0.23	-1.026	0.305	0.423	1.379
BTBD18	-0.033	0.295	-0.113	0.91	0.967	0.286	-0.115	0.908	0.542	1.725
BTBD1	-0.598	0.311	-1.922	0.055	0.55	0.171	-2.63	0.009	0.299	1.012
BTAF1	-0.483	0.301	-1.607	0.108	0.617	0.185	-2.065	0.039	0.342	1.112
BST2	0.136	0.296	0.459	0.646	1.146	0.339	0.43	0.668	0.642	2.045
BST1	-0.305	0.303	-1.004	0.315	0.737	0.224	-1.174	0.24	0.407	1.336
BSPRY	-0.406	0.303	-1.34	0.18	0.666	0.202	-1.654	0.098	0.368	1.207
BSN	0.077	0.295	0.261	0.794	1.08	0.319	0.251	0.802	0.606	1.925
BSG	-0.405	0.302	-1.34	0.18	0.667	0.202	-1.651	0.099	0.369	1.206
BSDC1	-0.464	0.301	-1.545	0.122	0.628	0.189	-1.966	0.049	0.349	1.133
BRWD1	0.497	0.301	1.653	0.098	1.643	0.494	1.303	0.193	0.912	2.961
BRSK2	0.394	0.3	1.314	0.189	1.482	0.444	1.086	0.277	0.824	2.666
BRS3	-0.216	0.296	-0.729	0.466	0.806	0.239	-0.814	0.416	0.451	1.44
BRPF1	0.472	0.303	1.56	0.119	1.604	0.486	1.243	0.214	0.886	2.903
BRMS1	0.075	0.295	0.253	0.8	1.078	0.318	0.244	0.807	0.604	1.922
BRME1	0.81	0.315	2.573	0.01	2.247	0.707	1.763	0.078	1.213	4.164
BRIX1	0.663	0.304	2.184	0.029	1.941	0.59	1.596	0.11	1.07	3.52
BRIP1	0.282	0.298	0.949	0.343	1.326	0.395	0.827	0.408	0.74	2.376
BRINP3	0.646	0.31	2.085	0.037	1.908	0.591	1.536	0.125	1.04	3.502
BRINP2	-0.486	0.302	-1.608	0.108	0.615	0.186	-2.071	0.038	0.34	1.112
BRINP1	0.24	0.298	0.807	0.42	1.271	0.378	0.717	0.473	0.71	2.278
BRF2	-0.245	0.3	-0.817	0.414	0.783	0.235	-0.925	0.355	0.435	1.409
BRF1	-0.183	0.296	-0.617	0.537	0.833	0.247	-0.677	0.498	0.466	1.489
BRDT	0.151	0.296	0.509	0.611	1.163	0.344	0.473	0.636	0.651	2.077
BRD9	0.015	0.296	0.051	0.959	1.015	0.3	0.051	0.959	0.569	1.812
BRD8	-0.199	0.296	-0.672	0.502	0.82	0.243	-0.743	0.457	0.459	1.465
BRD7P3	0.538	0.304	1.773	0.076	1.713	0.52	1.371	0.17	0.945	3.106
BRD7	0.851	0.315	2.702	0.007	2.342	0.738	1.819	0.069	1.263	4.343
BRD4	0.025	0.295	0.085	0.932	1.026	0.303	0.084	0.933	0.575	1.83
BRD3OS	0.193	0.296	0.653	0.514	1.213	0.36	0.593	0.553	0.679	2.169
BRD3	-0.114	0.295	-0.386	0.7	0.892	0.264	-0.409	0.683	0.5	1.592
BRD2	0.634	0.31	2.044	0.041	1.885	0.585	1.514	0.13	1.027	3.462

BRD1	-0.353	0.298	-1.184	0.236	0.702	0.209	-1.421	0.155	0.391	1.26
BRCC3	0.287	0.298	0.965	0.335	1.333	0.397	0.839	0.402	0.744	2.388
BRCA2	0.501	0.302	1.656	0.098	1.65	0.499	1.303	0.193	0.912	2.985
BRCA1	0.249	0.296	0.84	0.401	1.283	0.38	0.744	0.457	0.718	2.291
BRAP	-0.419	0.307	-1.363	0.173	0.658	0.202	-1.692	0.091	0.36	1.201
BPY2	0.19	0.295	0.642	0.521	1.209	0.357	0.585	0.559	0.677	2.157
BPTF	-0.206	0.297	-0.692	0.489	0.814	0.242	-0.768	0.442	0.455	1.458
BPNT2	0.025	0.295	0.085	0.932	1.026	0.303	0.084	0.933	0.575	1.828
BPNT1	-0.195	0.296	-0.657	0.511	0.823	0.244	-0.726	0.468	0.461	1.471
BPIFA1	-0.174	0.296	-0.588	0.557	0.84	0.249	-0.642	0.521	0.47	1.501
BPHL	0.008	0.295	0.027	0.979	1.008	0.298	0.027	0.979	0.565	1.798
BPGM	-0.323	0.298	-1.085	0.278	0.724	0.216	-1.281	0.2	0.404	1.298
BPESC1	0.077	0.296	0.261	0.794	1.08	0.319	0.252	0.801	0.605	1.928
BORCS8	0.671	0.304	2.208	0.027	1.956	0.594	1.608	0.108	1.078	3.548
BORCS6	-0.129	0.299	-0.431	0.666	0.879	0.263	-0.46	0.645	0.489	1.58
BORA	0.715	0.31	2.309	0.021	2.045	0.633	1.649	0.099	1.114	3.753
BOLA1	0.146	0.295	0.496	0.62	1.158	0.342	0.461	0.645	0.649	2.066
BNIP3L	-0.674	0.31	-2.178	0.029	0.509	0.158	-3.109	0.002	0.278	0.935
BNIP3	0.502	0.3	1.674	0.094	1.652	0.495	1.316	0.188	0.918	2.974
BNIP2	0.478	0.3	1.595	0.111	1.613	0.484	1.268	0.205	0.896	2.904
BNIP1	-0.065	0.295	-0.22	0.826	0.937	0.277	-0.228	0.82	0.525	1.671
BNC2	-0.352	0.298	-1.183	0.237	0.703	0.209	-1.418	0.156	0.392	1.26
BNC1	-0.307	0.298	-1.033	0.302	0.735	0.219	-1.209	0.227	0.41	1.318
BMX	-0.247	0.297	-0.833	0.405	0.781	0.232	-0.945	0.344	0.436	1.397
BMS1	0.811	0.322	2.515	0.012	2.249	0.725	1.723	0.085	1.196	4.231
BMPR2	0.402	0.307	1.312	0.189	1.495	0.459	1.08	0.28	0.82	2.727
BMPR1B	0.013	0.296	0.044	0.965	1.013	0.3	0.044	0.965	0.568	1.809
BMPR1A	0.398	0.3	1.328	0.184	1.489	0.446	1.096	0.273	0.828	2.678
BMP8B	-0.382	0.3	-1.274	0.203	0.683	0.205	-1.551	0.121	0.379	1.228
BMP8A	0.201	0.298	0.674	0.5	1.223	0.364	0.611	0.541	0.682	2.192
BMP7	0.404	0.303	1.334	0.182	1.498	0.453	1.098	0.272	0.827	2.711
BMP6	-0.227	0.297	-0.766	0.444	0.797	0.236	-0.86	0.39	0.446	1.425
BMP5	-0.253	0.297	-0.853	0.394	0.776	0.23	-0.97	0.332	0.434	1.389
BMP4	-0.701	0.306	-2.291	0.022	0.496	0.152	-3.32	0.001	0.272	0.904
BMP3	-0.487	0.303	-1.611	0.107	0.614	0.186	-2.075	0.038	0.34	1.112
BMP2K	-0.091	0.296	-0.306	0.76	0.913	0.271	-0.32	0.749	0.511	1.633
BMP2	0.341	0.3	1.138	0.255	1.406	0.421	0.964	0.335	0.782	2.53
BMP15	-0.117	0.295	-0.394	0.693	0.89	0.263	-0.418	0.676	0.499	1.588

BMP10	0.185	0.296	0.626	0.531	1.204	0.357	0.571	0.568	0.673	2.151
BMP1	-0.136	0.296	-0.459	0.646	0.873	0.259	-0.492	0.623	0.488	1.56
BMERB1	-0.185	0.296	-0.624	0.532	0.831	0.246	-0.686	0.493	0.465	1.485
BLZF1	0.203	0.296	0.685	0.493	1.225	0.363	0.62	0.535	0.686	2.188
BLVRB	0.425	0.298	1.426	0.154	1.53	0.456	1.162	0.245	0.853	2.744
BLVRA	0.709	0.306	2.313	0.021	2.031	0.622	1.657	0.097	1.114	3.702
BLOC1S1	-0.149	0.299	-0.5	0.617	0.861	0.257	-0.54	0.589	0.48	1.546
BLNK	-0.154	0.295	-0.522	0.602	0.857	0.253	-0.565	0.572	0.48	1.529
BLMH	-0.263	0.298	-0.883	0.377	0.769	0.229	-1.01	0.313	0.429	1.378
BLM	0.436	0.303	1.439	0.15	1.547	0.469	1.166	0.244	0.854	2.801
BLK	-0.187	0.297	-0.63	0.529	0.829	0.246	-0.692	0.489	0.463	1.485
BLCAP	-0.085	0.296	-0.288	0.774	0.918	0.272	-0.3	0.764	0.514	1.641
BIRC7	0.506	0.3	1.688	0.091	1.659	0.497	1.325	0.185	0.922	2.985
BIRC5	0.685	0.306	2.243	0.025	1.985	0.606	1.623	0.105	1.09	3.612
BIRC3	0.02	0.296	0.069	0.945	1.021	0.302	0.068	0.946	0.572	1.821
BIRC2	0.13	0.295	0.439	0.661	1.138	0.336	0.412	0.681	0.638	2.031
BIN3-IT1	0.018	0.295	0.062	0.95	1.019	0.301	0.062	0.951	0.571	1.816
BIN3	-0.437	0.303	-1.443	0.149	0.646	0.196	-1.81	0.07	0.357	1.169
BIN2	-0.185	0.296	-0.626	0.532	0.831	0.246	-0.687	0.492	0.465	1.485
BIN1	-0.402	0.3	-1.342	0.18	0.669	0.2	-1.651	0.099	0.372	1.204
BIK	-0.243	0.298	-0.817	0.414	0.784	0.233	-0.924	0.355	0.438	1.405
BID	0.654	0.306	2.14	0.032	1.924	0.588	1.571	0.116	1.057	3.503
BICRAL	0.007	0.295	0.024	0.981	1.007	0.297	0.024	0.981	0.565	1.797
BICRA	-0.054	0.295	-0.183	0.854	0.947	0.28	-0.188	0.851	0.531	1.69
BICDL1	-0.609	0.307	-1.982	0.047	0.544	0.167	-2.729	0.006	0.298	0.993
BICD2	-0.382	0.299	-1.279	0.201	0.682	0.204	-1.558	0.119	0.38	1.226
BICD1	0.241	0.298	0.809	0.418	1.273	0.379	0.719	0.472	0.71	2.282
BICC1	-0.706	0.304	-2.319	0.02	0.494	0.15	-3.369	0.001	0.272	0.897
BHMT2	0.005	0.295	0.015	0.988	1.005	0.296	0.015	0.988	0.563	1.791
BHMT	-0.54	0.303	-1.784	0.074	0.583	0.176	-2.365	0.018	0.322	1.055
BHLHE41	-0.366	0.3	-1.22	0.222	0.694	0.208	-1.473	0.141	0.386	1.248
BHLHE40	-0.443	0.302	-1.464	0.143	0.642	0.194	-1.842	0.065	0.355	1.162
BHLHB9	0.328	0.299	1.098	0.272	1.388	0.415	0.936	0.349	0.773	2.493
BGN	-0.364	0.296	-1.228	0.22	0.695	0.206	-1.481	0.139	0.389	1.242
BFSP2	-0.081	0.295	-0.275	0.784	0.922	0.272	-0.286	0.775	0.517	1.645
BFSP1	0.567	0.303	1.873	0.061	1.763	0.534	1.43	0.153	0.974	3.191
BFAR	0.217	0.296	0.732	0.464	1.242	0.368	0.658	0.511	0.695	2.219
BEX4	0.26	0.297	0.876	0.381	1.298	0.386	0.771	0.441	0.724	2.324

BEX3	0.588	0.306	1.921	0.055	1.801	0.551	1.452	0.146	0.988	3.281
BEX1	0.253	0.296	0.853	0.394	1.288	0.382	0.754	0.451	0.72	2.301
BET1L	-0.548	0.31	-1.765	0.077	0.578	0.179	-2.351	0.019	0.315	1.062
BET1	-0.138	0.296	-0.468	0.639	0.871	0.257	-0.502	0.615	0.488	1.554
BEST2	0.309	0.298	1.037	0.3	1.362	0.405	0.893	0.372	0.76	2.441
BEST1	-0.417	0.303	-1.375	0.169	0.659	0.2	-1.706	0.088	0.364	1.194
BEND5	-0.336	0.3	-1.12	0.263	0.714	0.215	-1.332	0.183	0.396	1.287
BEGAIN	0.036	0.297	0.122	0.903	1.037	0.308	0.12	0.905	0.58	1.854
BECN1	-0.425	0.303	-1.402	0.161	0.654	0.198	-1.747	0.081	0.361	1.184
BEAN1	-0.518	0.303	-1.713	0.087	0.596	0.18	-2.244	0.025	0.329	1.078
BDNF	0.07	0.295	0.237	0.813	1.073	0.317	0.229	0.819	0.601	1.914
BDKRB2	-0.089	0.296	-0.3	0.764	0.915	0.271	-0.314	0.753	0.512	1.635
BDKRB1	0.671	0.306	2.191	0.028	1.955	0.598	1.597	0.11	1.073	3.562
BDH2	-0.391	0.3	-1.305	0.192	0.676	0.203	-1.596	0.11	0.376	1.217
BDH1	-0.129	0.298	-0.434	0.664	0.879	0.262	-0.463	0.643	0.49	1.575
BCS1L	0.064	0.299	0.216	0.829	1.067	0.318	0.209	0.834	0.594	1.915
BCR	0.166	0.299	0.556	0.578	1.181	0.353	0.512	0.609	0.657	2.123
BCORL1	0.692	0.31	2.232	0.026	1.997	0.619	1.611	0.107	1.088	3.666
BCOR	-0.062	0.295	-0.208	0.835	0.94	0.278	-0.215	0.83	0.527	1.678
BCO1	0.099	0.299	0.331	0.74	1.104	0.33	0.315	0.753	0.614	1.984
BCLAF1	-0.455	0.3	-1.519	0.129	0.634	0.19	-1.923	0.054	0.353	1.141
BCL9	-0.065	0.295	-0.221	0.825	0.937	0.277	-0.228	0.819	0.525	1.671
BCL7C	0.069	0.296	0.233	0.816	1.071	0.317	0.225	0.822	0.6	1.913
BCL7B	0.152	0.297	0.512	0.609	1.164	0.346	0.475	0.635	0.651	2.083
BCL7A	-0.032	0.295	-0.11	0.913	0.968	0.286	-0.112	0.911	0.543	1.727
BCL6	-0.281	0.298	-0.943	0.346	0.755	0.225	-1.089	0.276	0.421	1.354
BCL2L2	-0.08	0.296	-0.272	0.786	0.923	0.273	-0.283	0.777	0.517	1.648
BCL2L14	-0.34	0.299	-1.138	0.255	0.712	0.213	-1.355	0.175	0.396	1.278
BCL2L13	-0.231	0.297	-0.779	0.436	0.794	0.236	-0.876	0.381	0.444	1.42
BCL2L11	0.477	0.3	1.589	0.112	1.611	0.483	1.264	0.206	0.895	2.9
BCL2L10	0.019	0.296	0.066	0.948	1.02	0.302	0.065	0.948	0.571	1.822
BCL2L1	-0.246	0.296	-0.83	0.407	0.782	0.232	-0.941	0.347	0.437	1.398
BCL2A1	0.109	0.296	0.367	0.714	1.115	0.33	0.348	0.728	0.624	1.99
BCL2	-0.393	0.308	-1.279	0.201	0.675	0.208	-1.567	0.117	0.369	1.233
BCL11B	-0.218	0.296	-0.734	0.463	0.804	0.238	-0.821	0.412	0.45	1.438
BCL11A	0.323	0.3	1.077	0.282	1.381	0.414	0.92	0.357	0.767	2.485
BCL10	0.241	0.299	0.807	0.419	1.273	0.38	0.717	0.473	0.709	2.286
BCKDK	0.107	0.296	0.36	0.719	1.113	0.329	0.342	0.733	0.623	1.988

BCKDHB	0.256	0.297	0.864	0.388	1.292	0.383	0.762	0.446	0.722	2.31
BCKDHA	0.223	0.298	0.749	0.454	1.25	0.373	0.671	0.502	0.697	2.243
BCHE	-0.482	0.306	-1.576	0.115	0.617	0.189	-2.025	0.043	0.339	1.125
BCCIP	0.177	0.296	0.598	0.55	1.194	0.354	0.548	0.584	0.668	2.134
BCAT2	0.07	0.299	0.236	0.814	1.073	0.32	0.228	0.82	0.598	1.926
BCAT1	0.676	0.306	2.211	0.027	1.967	0.602	1.607	0.108	1.08	3.582
BCAS4	-0.114	0.296	-0.386	0.7	0.892	0.264	-0.409	0.683	0.499	1.594
BCAS3	0.043	0.295	0.147	0.883	1.044	0.308	0.144	0.886	0.585	1.863
BCAS2	0.305	0.297	1.026	0.305	1.357	0.404	0.884	0.377	0.757	2.431
BCAS1	0.523	0.303	1.725	0.084	1.687	0.511	1.344	0.179	0.931	3.055
BCAR3	0.031	0.298	0.104	0.917	1.031	0.307	0.102	0.919	0.576	1.848
BCAP31	0.197	0.295	0.666	0.505	1.217	0.36	0.605	0.545	0.682	2.172
BCAP29	0.057	0.296	0.191	0.849	1.058	0.314	0.186	0.853	0.592	1.892
BCAN	0.448	0.3	1.495	0.135	1.565	0.469	1.205	0.228	0.87	2.816
BCAM	-0.469	0.3	-1.567	0.117	0.625	0.187	-2	0.046	0.348	1.125
BBX	0.374	0.3	1.249	0.212	1.454	0.435	1.042	0.297	0.808	2.615
BBS9	0.177	0.297	0.596	0.551	1.193	0.354	0.546	0.585	0.667	2.134
BBS7	0.71	0.307	2.317	0.02	2.035	0.624	1.659	0.097	1.116	3.711
BBS4	-0.486	0.306	-1.588	0.112	0.615	0.188	-2.045	0.041	0.338	1.12
BBS10	-0.202	0.296	-0.68	0.496	0.817	0.242	-0.754	0.451	0.457	1.461
BBS1	-0.427	0.303	-1.412	0.158	0.652	0.197	-1.762	0.078	0.36	1.18
BBOX1	-0.082	0.295	-0.276	0.782	0.922	0.272	-0.288	0.773	0.517	1.644
BBOF1	-0.664	0.31	-2.142	0.032	0.515	0.16	-3.039	0.002	0.281	0.945
BBIP1	-0.991	0.321	-3.084	0.002	0.371	0.119	-5.273	0	0.198	0.697
BAZ2B	-0.564	0.303	-1.863	0.063	0.569	0.172	-2.502	0.012	0.315	1.03
BAZ2A	0.098	0.295	0.332	0.74	1.103	0.326	0.316	0.752	0.618	1.967
BAZ1B	0.718	0.307	2.337	0.019	2.05	0.63	1.667	0.095	1.123	3.743
BAZ1A	-0.542	0.301	-1.802	0.072	0.582	0.175	-2.391	0.017	0.322	1.049
BAX	0.575	0.301	1.91	0.056	1.778	0.535	1.453	0.146	0.985	3.208
BATF3	-0.56	0.306	-1.833	0.067	0.571	0.175	-2.457	0.014	0.314	1.04
BATF	-0.376	0.3	-1.256	0.209	0.686	0.206	-1.525	0.127	0.381	1.235
BASP1	-0.031	0.295	-0.104	0.917	0.97	0.286	-0.105	0.916	0.544	1.729
BARX2	0.814	0.311	2.619	0.009	2.256	0.701	1.792	0.073	1.227	4.149
BARX1	-0.482	0.302	-1.595	0.111	0.617	0.187	-2.05	0.04	0.341	1.116
BARD1	-0.14	0.298	-0.47	0.638	0.869	0.259	-0.504	0.614	0.485	1.558
BAP1	-0.126	0.296	-0.426	0.67	0.881	0.261	-0.455	0.649	0.493	1.575
BANP	0.53	0.303	1.752	0.08	1.699	0.514	1.36	0.174	0.939	3.075
BANK1	-0.445	0.3	-1.482	0.138	0.641	0.192	-1.866	0.062	0.356	1.154

BANF1	0.449	0.3	1.497	0.135	1.567	0.471	1.205	0.228	0.87	2.823
BAMBI	0.118	0.296	0.4	0.689	1.126	0.334	0.377	0.706	0.63	2.012
BAK1	0.353	0.298	1.186	0.236	1.424	0.424	0.999	0.318	0.794	2.552
BAIAP3	-0.566	0.306	-1.853	0.064	0.568	0.173	-2.493	0.013	0.312	1.033
BAIAP2L2	-0.035	0.295	-0.118	0.906	0.966	0.285	-0.12	0.904	0.541	1.723
BAIAP2	-0.07	0.295	-0.236	0.813	0.933	0.275	-0.244	0.807	0.523	1.664
BAHD1	-0.431	0.302	-1.428	0.153	0.65	0.196	-1.785	0.074	0.359	1.175
BAHCC1	-0.119	0.295	-0.402	0.688	0.888	0.262	-0.426	0.67	0.498	1.584
BAGE	-0.4	0.3	-1.333	0.183	0.671	0.201	-1.638	0.101	0.372	1.207
BAG6	0.483	0.301	1.605	0.108	1.621	0.488	1.273	0.203	0.899	2.925
BAG5	0.244	0.296	0.825	0.409	1.277	0.378	0.732	0.464	0.715	2.282
BAG4	0.17	0.295	0.576	0.565	1.185	0.35	0.529	0.597	0.664	2.114
BAG3	0.485	0.302	1.605	0.108	1.625	0.491	1.272	0.204	0.898	2.938
BAG2	-0.284	0.298	-0.954	0.34	0.753	0.224	-1.103	0.27	0.42	1.349
BAG1	-0.733	0.315	-2.329	0.02	0.48	0.151	-3.436	0.001	0.259	0.89
BAD	0.332	0.3	1.105	0.269	1.393	0.418	0.94	0.347	0.774	2.51
BACH2	-0.21	0.296	-0.71	0.478	0.811	0.24	-0.79	0.429	0.454	1.447
BACH1	-0.224	0.297	-0.754	0.451	0.8	0.237	-0.845	0.398	0.447	1.43
BACE2	0.067	0.295	0.227	0.82	1.069	0.316	0.22	0.826	0.6	1.908
BACE1	-0.536	0.306	-1.753	0.08	0.585	0.179	-2.319	0.02	0.322	1.065
BABAM2	-0.213	0.298	-0.715	0.474	0.808	0.241	-0.797	0.425	0.451	1.449
BABAM1	0.099	0.295	0.335	0.737	1.104	0.326	0.319	0.75	0.619	1.97
BAAT	0.271	0.296	0.915	0.36	1.311	0.388	0.802	0.423	0.734	2.342
BAALC	0.303	0.298	1.017	0.309	1.354	0.403	0.878	0.38	0.755	2.426
B9D2	0.088	0.296	0.296	0.767	1.092	0.323	0.284	0.777	0.611	1.95
B9D1	0.123	0.296	0.415	0.678	1.131	0.334	0.39	0.696	0.633	2.018
B4GAT1	-0.187	0.297	-0.631	0.528	0.829	0.246	-0.694	0.488	0.463	1.484
B4GALT7	0.601	0.303	1.983	0.047	1.824	0.553	1.491	0.136	1.007	3.303
B4GALT6	-0.318	0.298	-1.069	0.285	0.728	0.217	-1.258	0.208	0.406	1.304
B4GALT5	0.376	0.303	1.242	0.214	1.457	0.441	1.035	0.3	0.805	2.637
B4GALT4	0.83	0.311	2.671	0.008	2.293	0.712	1.815	0.07	1.247	4.214
B4GALT3	-0.354	0.3	-1.18	0.238	0.702	0.21	-1.416	0.157	0.39	1.263
B4GALT2	0.37	0.298	1.242	0.214	1.448	0.431	1.038	0.299	0.807	2.595
B4GALT1	-0.151	0.295	-0.511	0.61	0.86	0.254	-0.551	0.581	0.482	1.534
B4GALNT1	0.21	0.296	0.711	0.477	1.234	0.365	0.641	0.522	0.691	2.205
B3GNTL1	0.448	0.3	1.494	0.135	1.566	0.47	1.204	0.228	0.87	2.819
B3GNT4	0.058	0.295	0.195	0.845	1.059	0.313	0.19	0.849	0.594	1.89
B3GNT3	0.404	0.3	1.35	0.177	1.498	0.449	1.11	0.267	0.833	2.695

B3GNT2	-0.272	0.3	-0.905	0.365	0.762	0.229	-1.04	0.298	0.423	1.372
B3GAT3	0.222	0.296	0.752	0.452	1.249	0.369	0.674	0.5	0.7	2.23
B3GAT1	0.165	0.296	0.556	0.578	1.179	0.349	0.512	0.608	0.66	2.106
B3GALT5	0.54	0.302	1.786	0.074	1.716	0.519	1.38	0.168	0.949	3.104
B3GALT4	0.595	0.302	1.971	0.049	1.813	0.547	1.486	0.137	1.003	3.276
B3GALT2	-0.314	0.3	-1.048	0.295	0.731	0.219	-1.231	0.218	0.406	1.314
B3GALT1	0.858	0.314	2.732	0.006	2.358	0.74	1.834	0.067	1.274	4.363
B3GALNT1	0.103	0.296	0.348	0.728	1.108	0.328	0.33	0.741	0.62	1.981
B2M	0.015	0.295	0.05	0.96	1.015	0.299	0.049	0.961	0.569	1.809
AZU1	0.235	0.296	0.794	0.427	1.265	0.375	0.707	0.479	0.708	2.262
AZIN1	-0.043	0.295	-0.144	0.885	0.958	0.283	-0.147	0.883	0.537	1.71
AZI2	-0.049	0.295	-0.165	0.869	0.952	0.281	-0.169	0.866	0.534	1.698
AZGP1P1	-0.753	0.315	-2.391	0.017	0.471	0.148	-3.567	0	0.254	0.873
AZGP1	-0.529	0.303	-1.747	0.081	0.589	0.178	-2.303	0.021	0.326	1.066
AXL	-0.8	0.311	-2.575	0.01	0.449	0.14	-3.944	0	0.245	0.826
AXIN1	-0.381	0.3	-1.271	0.204	0.683	0.205	-1.547	0.122	0.38	1.23
AVPR2	-0.108	0.295	-0.366	0.715	0.898	0.265	-0.386	0.699	0.503	1.602
AVPR1B	0.092	0.295	0.311	0.756	1.096	0.324	0.297	0.767	0.614	1.955
AVPR1A	0.295	0.298	0.99	0.322	1.343	0.4	0.857	0.391	0.749	2.406
AVPI1	0.223	0.296	0.754	0.451	1.25	0.37	0.676	0.499	0.7	2.233
AVP	0.076	0.296	0.257	0.797	1.079	0.319	0.247	0.805	0.604	1.926
AVL9	0.36	0.298	1.21	0.226	1.434	0.427	1.016	0.31	0.8	2.569
AVIL	0.234	0.298	0.787	0.431	1.264	0.377	0.701	0.483	0.705	2.266
AVEN	0.156	0.296	0.528	0.598	1.169	0.346	0.489	0.625	0.655	2.087
AUTS2	0.233	0.296	0.786	0.432	1.262	0.374	0.701	0.483	0.706	2.256
AURKC	-0.027	0.297	-0.091	0.928	0.973	0.289	-0.092	0.927	0.544	1.741
AURKB	0.686	0.306	2.244	0.025	1.986	0.607	1.624	0.104	1.091	3.616
AURKAIP1	0.367	0.299	1.228	0.219	1.443	0.431	1.028	0.304	0.803	2.593
AURKA	0.82	0.315	2.605	0.009	2.271	0.715	1.778	0.075	1.225	4.208
AUP1	0.129	0.296	0.436	0.663	1.138	0.337	0.409	0.682	0.637	2.033
AUNIP	0.64	0.306	2.094	0.036	1.897	0.58	1.547	0.122	1.042	3.452
AUH	-0.489	0.307	-1.594	0.111	0.613	0.188	-2.055	0.04	0.336	1.119
ATXN8OS	0.31	0.298	1.041	0.298	1.363	0.406	0.896	0.371	0.761	2.442
ATXN7L3B	-0.293	0.297	-0.986	0.324	0.746	0.222	-1.146	0.252	0.416	1.336
ATXN7L1	0.845	0.315	2.684	0.007	2.328	0.733	1.812	0.07	1.256	4.314
ATXN7	-0.137	0.296	-0.464	0.643	0.872	0.258	-0.497	0.619	0.489	1.556
ATXN3L	0.267	0.297	0.899	0.368	1.306	0.387	0.789	0.43	0.73	2.335
ATXN3	0.626	0.304	2.06	0.039	1.87	0.568	1.531	0.126	1.031	3.393

ATXN2L	0.009	0.297	0.029	0.977	1.009	0.299	0.029	0.977	0.564	1.804
ATXN2	0.118	0.296	0.398	0.69	1.125	0.333	0.376	0.707	0.63	2.009
ATXN10	-0.133	0.296	-0.448	0.654	0.876	0.259	-0.48	0.632	0.491	1.564
ATXN1	-0.882	0.311	-2.834	0.005	0.414	0.129	-4.55	0	0.225	0.762
ATRX	-0.159	0.296	-0.537	0.591	0.853	0.253	-0.582	0.561	0.477	1.524
ATRNL1	0.448	0.3	1.494	0.135	1.565	0.469	1.205	0.228	0.87	2.816
ATRN	-0.1	0.295	-0.337	0.736	0.905	0.267	-0.355	0.723	0.507	1.615
ATRAID	-0.337	0.3	-1.124	0.261	0.714	0.214	-1.337	0.181	0.397	1.285
ATR	0.456	0.303	1.506	0.132	1.578	0.478	1.209	0.227	0.872	2.856
ATPAF2	-0.136	0.299	-0.455	0.649	0.873	0.261	-0.487	0.626	0.486	1.568
ATP9B	-0.313	0.3	-1.045	0.296	0.731	0.219	-1.227	0.22	0.406	1.316
ATP9A	0.334	0.298	1.122	0.262	1.397	0.416	0.954	0.34	0.779	2.506
ATP8B4	-0.171	0.296	-0.576	0.565	0.843	0.25	-0.628	0.53	0.472	1.507
ATP8B3	0.301	0.298	1.01	0.312	1.351	0.402	0.873	0.383	0.754	2.42
ATP8B2	0.128	0.296	0.431	0.666	1.136	0.337	0.405	0.685	0.636	2.031
ATP8B1	-0.413	0.3	-1.377	0.169	0.661	0.199	-1.705	0.088	0.367	1.191
ATP8A2	-0.218	0.296	-0.734	0.463	0.804	0.239	-0.82	0.412	0.45	1.438
ATP8A1	-0.621	0.304	-2.043	0.041	0.537	0.163	-2.832	0.005	0.296	0.975
ATP7B	-0.009	0.295	-0.03	0.976	0.991	0.293	-0.03	0.976	0.556	1.768
ATP7A	0.015	0.296	0.052	0.959	1.015	0.3	0.051	0.959	0.569	1.813
ATP6V1H	0.116	0.295	0.391	0.696	1.122	0.332	0.369	0.712	0.629	2.003
ATP6V1G2	0.591	0.306	1.932	0.053	1.805	0.552	1.459	0.145	0.991	3.286
ATP6V1G1	0.221	0.296	0.746	0.456	1.247	0.369	0.669	0.503	0.698	2.229
ATP6V1F	0.121	0.297	0.406	0.685	1.128	0.335	0.383	0.702	0.631	2.018
ATP6V1E1	-0.106	0.295	-0.358	0.72	0.9	0.266	-0.377	0.706	0.504	1.605
ATP6V1D	0.189	0.297	0.636	0.525	1.208	0.359	0.58	0.562	0.675	2.161
ATP6V1C1	0.053	0.296	0.179	0.858	1.054	0.312	0.174	0.862	0.59	1.883
ATP6V1B2	-0.035	0.297	-0.118	0.906	0.966	0.287	-0.12	0.905	0.539	1.728
ATP6V1B1	0.1	0.295	0.338	0.735	1.105	0.326	0.322	0.748	0.619	1.971
ATP6V1A	0.226	0.298	0.758	0.448	1.253	0.373	0.679	0.497	0.699	2.247
ATP6V0E2	0.128	0.295	0.432	0.666	1.136	0.336	0.406	0.685	0.637	2.027
ATP6V0E1	0.142	0.296	0.479	0.632	1.152	0.341	0.447	0.655	0.645	2.057
ATP6V0D1	0.141	0.295	0.476	0.634	1.151	0.34	0.444	0.657	0.645	2.053
ATP6V0C	-0.104	0.295	-0.352	0.725	0.901	0.266	-0.371	0.711	0.505	1.608
ATP6V0B	0.174	0.296	0.589	0.556	1.19	0.352	0.541	0.589	0.667	2.125
ATP6V0A4	-0.272	0.297	-0.917	0.359	0.762	0.226	-1.054	0.292	0.426	1.363
ATP6V0A2	0.427	0.3	1.426	0.154	1.533	0.46	1.16	0.246	0.852	2.759
ATP6V0A1	0.019	0.297	0.065	0.948	1.019	0.302	0.064	0.949	0.57	1.823

ATP6AP2	-0.168	0.297	-0.567	0.571	0.845	0.251	-0.617	0.537	0.472	1.513
ATP6AP1	-0.559	0.306	-1.828	0.068	0.572	0.175	-2.449	0.014	0.314	1.041
ATP5PO	0.172	0.296	0.582	0.561	1.188	0.352	0.534	0.593	0.665	2.123
ATP5PF	0.354	0.298	1.189	0.234	1.425	0.425	1.001	0.317	0.795	2.556
ATP5PD	0.437	0.3	1.459	0.145	1.548	0.464	1.182	0.237	0.861	2.785
ATP5PB	0.274	0.297	0.921	0.357	1.315	0.391	0.805	0.421	0.734	2.355
ATP5MPL	0.058	0.295	0.195	0.846	1.059	0.313	0.189	0.85	0.594	1.889
ATP5MG	-0.314	0.3	-1.048	0.295	0.731	0.219	-1.231	0.218	0.406	1.314
ATP5ME	0.026	0.295	0.088	0.93	1.026	0.303	0.087	0.93	0.576	1.831
ATP5MC3	0.281	0.298	0.945	0.345	1.325	0.394	0.824	0.41	0.739	2.374
ATP5MC2	-0.17	0.297	-0.572	0.567	0.844	0.25	-0.624	0.533	0.472	1.509
ATP5MC1	0.523	0.302	1.729	0.084	1.687	0.51	1.347	0.178	0.933	3.05
ATP5IF1	0.564	0.301	1.87	0.062	1.757	0.53	1.429	0.153	0.973	3.172
ATP5F1E	0.533	0.302	1.764	0.078	1.704	0.515	1.367	0.172	0.942	3.082
ATP5F1D	-0.336	0.3	-1.122	0.262	0.714	0.214	-1.334	0.182	0.397	1.286
ATP5F1C	0.598	0.303	1.976	0.048	1.818	0.55	1.487	0.137	1.005	3.291
ATP5F1B	0.217	0.296	0.732	0.464	1.242	0.368	0.658	0.511	0.695	2.221
ATP5F1A	0.28	0.297	0.944	0.345	1.323	0.392	0.823	0.41	0.74	2.367
ATP4B	0.736	0.307	2.398	0.016	2.088	0.641	1.697	0.09	1.144	3.813
ATP4A	0.645	0.304	2.124	0.034	1.905	0.578	1.565	0.118	1.051	3.454
ATP2C2	-0.263	0.296	-0.887	0.375	0.769	0.228	-1.015	0.31	0.43	1.374
ATP2C1	0.407	0.302	1.346	0.178	1.502	0.454	1.105	0.269	0.83	2.718
ATP2B4	-0.833	0.311	-2.677	0.007	0.435	0.135	-4.179	0	0.236	0.8
ATP2B3	-0.232	0.298	-0.781	0.435	0.793	0.236	-0.879	0.38	0.442	1.421
ATP2B2	-0.11	0.296	-0.372	0.71	0.896	0.265	-0.393	0.694	0.502	1.6
ATP2B1	-0.162	0.296	-0.547	0.584	0.851	0.252	-0.594	0.553	0.476	1.519
ATP2A3	-0.178	0.296	-0.601	0.548	0.837	0.248	-0.658	0.511	0.468	1.496
ATP2A2	-0.238	0.297	-0.801	0.423	0.788	0.234	-0.905	0.366	0.44	1.411
ATP2A1	-0.025	0.295	-0.083	0.934	0.976	0.288	-0.084	0.933	0.547	1.741
ATP1B4	-0.014	0.295	-0.049	0.961	0.986	0.291	-0.049	0.961	0.553	1.757
ATP1B3	0.63	0.303	2.08	0.038	1.878	0.569	1.543	0.123	1.037	3.401
ATP1B2	0.295	0.3	0.984	0.325	1.343	0.403	0.852	0.394	0.746	2.417
ATP1B1	-0.319	0.3	-1.064	0.287	0.727	0.218	-1.254	0.21	0.404	1.308
ATP1A3	0.01	0.295	0.033	0.973	1.01	0.298	0.033	0.974	0.566	1.801
ATP1A2	-0.834	0.31	-2.691	0.007	0.434	0.135	-4.203	0	0.237	0.797
ATP1A1	-0.247	0.298	-0.83	0.407	0.781	0.232	-0.941	0.347	0.436	1.4
ATP13A3	0.282	0.297	0.948	0.343	1.326	0.394	0.826	0.409	0.74	2.376
ATP13A2	0.156	0.298	0.524	0.6	1.169	0.348	0.485	0.628	0.652	2.094

ATP13A1	0.042	0.296	0.142	0.887	1.043	0.309	0.139	0.89	0.584	1.864
ATP12A	0.057	0.295	0.192	0.848	1.058	0.312	0.186	0.852	0.593	1.887
ATP11B	0.48	0.306	1.57	0.116	1.616	0.494	1.247	0.212	0.888	2.942
ATP11A	-0.3	0.298	-1.005	0.315	0.741	0.221	-1.172	0.241	0.413	1.329
ATP10D	-0.348	0.298	-1.167	0.243	0.706	0.21	-1.396	0.163	0.394	1.267
ATP10B	-0.145	0.297	-0.489	0.625	0.865	0.257	-0.526	0.599	0.483	1.548
ATP10A	0.143	0.296	0.483	0.629	1.153	0.341	0.45	0.652	0.646	2.059
ATOX1	0.041	0.296	0.14	0.889	1.042	0.308	0.137	0.891	0.584	1.86
ATOH1	0.206	0.296	0.697	0.486	1.229	0.364	0.63	0.529	0.688	2.197
ATN1	0.325	0.3	1.083	0.279	1.384	0.415	0.925	0.355	0.769	2.493
ATMIN	0.295	0.296	0.997	0.319	1.344	0.398	0.863	0.388	0.752	2.402
ATM	-0.766	0.308	-2.487	0.013	0.465	0.143	-3.736	0	0.254	0.85
ATIC	-0.12	0.295	-0.406	0.685	0.887	0.262	-0.431	0.666	0.497	1.583
ATG9A	-0.349	0.299	-1.166	0.244	0.706	0.211	-1.395	0.163	0.393	1.268
ATG7	-0.177	0.296	-0.6	0.549	0.837	0.248	-0.656	0.512	0.469	1.495
ATG5	0.25	0.296	0.844	0.399	1.284	0.38	0.747	0.455	0.719	2.294
ATG4B	0.431	0.303	1.423	0.155	1.538	0.466	1.156	0.248	0.85	2.784
ATG4A	-0.255	0.3	-0.849	0.396	0.775	0.233	-0.967	0.333	0.431	1.395
ATG3	0.618	0.306	2.021	0.043	1.854	0.567	1.507	0.132	1.019	3.376
ATG2B	-0.191	0.296	-0.646	0.518	0.826	0.245	-0.712	0.477	0.462	1.476
ATG2A	0.698	0.306	2.281	0.023	2.009	0.615	1.642	0.101	1.103	3.66
ATG16L1	0.181	0.296	0.61	0.542	1.198	0.355	0.558	0.577	0.67	2.14
ATG14	-0.347	0.298	-1.164	0.244	0.707	0.211	-1.392	0.164	0.394	1.268
ATG13	-0.201	0.297	-0.677	0.498	0.818	0.243	-0.75	0.453	0.457	1.464
ATG12	0.452	0.3	1.507	0.132	1.572	0.472	1.212	0.225	0.873	2.831
ATG101	0.318	0.297	1.069	0.285	1.374	0.408	0.916	0.36	0.767	2.46
ATG10	0.051	0.295	0.172	0.863	1.052	0.31	0.168	0.867	0.59	1.876
ATF7IP2	-0.516	0.306	-1.687	0.092	0.597	0.182	-2.208	0.027	0.328	1.087
ATF7IP	0.929	0.316	2.935	0.003	2.532	0.801	1.912	0.056	1.362	4.707
ATF7	-0.192	0.296	-0.65	0.516	0.825	0.244	-0.716	0.474	0.462	1.474
ATF6B	0.195	0.296	0.657	0.511	1.215	0.36	0.597	0.55	0.68	2.17
ATF6	0.144	0.297	0.485	0.628	1.155	0.343	0.451	0.652	0.645	2.066
ATF5	-0.293	0.3	-0.978	0.328	0.746	0.223	-1.136	0.256	0.415	1.342
ATF4	0.311	0.298	1.046	0.296	1.365	0.407	0.899	0.369	0.762	2.447
ATF3	-0.046	0.295	-0.155	0.877	0.955	0.282	-0.159	0.874	0.535	1.704
ATF2	-0.096	0.295	-0.326	0.745	0.908	0.268	-0.342	0.732	0.509	1.621
ATF1	-0.354	0.298	-1.19	0.234	0.702	0.209	-1.428	0.153	0.392	1.258
ATAT1	0.638	0.306	2.087	0.037	1.893	0.578	1.543	0.123	1.04	3.445

ATAD5	0.822	0.315	2.608	0.009	2.274	0.716	1.778	0.075	1.226	4.217
ATAD2B	1.342	0.346	3.874	0	3.826	1.325	2.133	0.033	1.94	7.542
ATAD2	0.777	0.315	2.465	0.014	2.175	0.686	1.714	0.087	1.173	4.035
ASXL2	-0.439	0.299	-1.465	0.143	0.645	0.193	-1.84	0.066	0.359	1.16
ASXL1	-0.297	0.297	-1.002	0.317	0.743	0.221	-1.167	0.243	0.415	1.329
ASTN2	-0.1	0.295	-0.34	0.734	0.904	0.267	-0.358	0.721	0.507	1.614
ASTN1	0.215	0.301	0.714	0.475	1.24	0.373	0.642	0.521	0.687	2.236
ASTE1	0.101	0.296	0.341	0.733	1.106	0.327	0.324	0.746	0.619	1.976
ASS1	0.18	0.296	0.606	0.544	1.197	0.355	0.555	0.579	0.67	2.139
ASRGL1	0.379	0.298	1.27	0.204	1.46	0.435	1.058	0.29	0.814	2.619
ASPSCR1	-0.185	0.295	-0.627	0.53	0.831	0.245	-0.689	0.491	0.466	1.482
ASPN	-0.492	0.302	-1.629	0.103	0.611	0.185	-2.104	0.035	0.338	1.105
ASPM	0.503	0.303	1.663	0.096	1.654	0.5	1.307	0.191	0.914	2.992
ASPHD1	0.487	0.301	1.618	0.106	1.628	0.49	1.281	0.2	0.902	2.936
ASPH	-0.916	0.321	-2.854	0.004	0.4	0.128	-4.67	0	0.213	0.751
ASPA	-0.468	0.306	-1.528	0.127	0.626	0.192	-1.948	0.051	0.344	1.142
ASNSD1	0.241	0.299	0.806	0.42	1.272	0.38	0.716	0.474	0.708	2.285
ASNS	0.434	0.298	1.457	0.145	1.543	0.46	1.182	0.237	0.861	2.767
ASMTL-AS1	0.413	0.3	1.376	0.169	1.511	0.453	1.127	0.26	0.839	2.721
ASMTL	-0.058	0.298	-0.194	0.847	0.944	0.281	-0.199	0.842	0.526	1.693
ASMT	0.25	0.297	0.844	0.399	1.284	0.381	0.747	0.455	0.718	2.297
ASL	0.623	0.304	2.05	0.04	1.865	0.567	1.525	0.127	1.028	3.386
ASIP	0.006	0.296	0.021	0.983	1.006	0.298	0.021	0.984	0.563	1.798
ASIC4	0.368	0.299	1.228	0.219	1.444	0.433	1.027	0.304	0.803	2.598
ASIC2	0.426	0.3	1.421	0.155	1.531	0.459	1.157	0.247	0.851	2.755
ASIC1	0.659	0.306	2.155	0.031	1.932	0.591	1.578	0.114	1.061	3.518
ASH2L	-0.381	0.3	-1.272	0.203	0.683	0.205	-1.549	0.121	0.38	1.229
ASH1L	0.474	0.306	1.549	0.121	1.607	0.492	1.234	0.217	0.882	2.928
ASGR2	0.168	0.296	0.567	0.571	1.183	0.35	0.522	0.602	0.662	2.113
ASGR1	0.135	0.295	0.456	0.649	1.144	0.338	0.426	0.67	0.641	2.041
ASF1B	0.628	0.306	2.055	0.04	1.874	0.573	1.526	0.127	1.029	3.412
ASF1A	0.217	0.296	0.734	0.463	1.243	0.368	0.66	0.509	0.696	2.221
ASCL3	0.058	0.297	0.195	0.845	1.06	0.314	0.19	0.849	0.592	1.896
ASCL2	0.429	0.3	1.433	0.152	1.536	0.46	1.165	0.244	0.854	2.763
ASCL1	0.103	0.296	0.348	0.728	1.109	0.328	0.331	0.741	0.621	1.979
ASCC3	-0.253	0.297	-0.852	0.394	0.777	0.23	-0.97	0.332	0.434	1.389
ASCC2	-0.627	0.306	-2.05	0.04	0.534	0.163	-2.852	0.004	0.293	0.973
ASCC1	0.357	0.299	1.192	0.233	1.429	0.428	1.002	0.316	0.795	2.57

ASB9	0.566	0.306	1.852	0.064	1.761	0.538	1.415	0.157	0.968	3.206
ASB8	0.131	0.296	0.442	0.659	1.14	0.337	0.414	0.679	0.638	2.034
ASB7	-0.148	0.299	-0.495	0.62	0.862	0.258	-0.534	0.593	0.48	1.549
ASB6	0.349	0.3	1.164	0.245	1.417	0.425	0.982	0.326	0.788	2.55
ASB4	-0.162	0.296	-0.545	0.586	0.851	0.252	-0.592	0.554	0.476	1.521
ASB13	-0.355	0.3	-1.185	0.236	0.701	0.21	-1.423	0.155	0.39	1.261
ASB1	-0.577	0.304	-1.899	0.058	0.561	0.171	-2.57	0.01	0.309	1.019
ASAP3	-0.025	0.299	-0.083	0.934	0.976	0.292	-0.084	0.933	0.542	1.754
ASAP2	0.681	0.31	2.198	0.028	1.976	0.612	1.594	0.111	1.077	3.626
ASAP1-IT1	0.448	0.303	1.477	0.14	1.565	0.474	1.19	0.234	0.864	2.835
ASAP1	0.28	0.297	0.94	0.347	1.323	0.393	0.82	0.412	0.738	2.369
ASAH1	-0.686	0.31	-2.215	0.027	0.504	0.156	-3.183	0.001	0.274	0.924
ARVCF	0.249	0.298	0.836	0.403	1.282	0.382	0.74	0.459	0.716	2.298
ARTN	0.328	0.297	1.105	0.269	1.388	0.412	0.942	0.346	0.776	2.483
ART4	-0.177	0.295	-0.598	0.55	0.838	0.248	-0.654	0.513	0.47	1.495
ART3	0.097	0.296	0.328	0.743	1.102	0.327	0.313	0.754	0.616	1.971
ART1	-0.312	0.297	-1.051	0.293	0.732	0.217	-1.233	0.217	0.409	1.31
ARSL	-0.404	0.298	-1.357	0.175	0.667	0.199	-1.673	0.094	0.372	1.197
ARSJ	0.028	0.296	0.095	0.924	1.029	0.304	0.094	0.925	0.576	1.836
ARSF	-0.229	0.296	-0.772	0.44	0.795	0.236	-0.867	0.386	0.445	1.422
ARSD	-0.29	0.3	-0.968	0.333	0.748	0.224	-1.123	0.261	0.416	1.346
ARSB	-0.214	0.296	-0.724	0.469	0.807	0.239	-0.807	0.42	0.451	1.443
ARSA	-0.07	0.296	-0.235	0.814	0.933	0.276	-0.243	0.808	0.523	1.665
ARRB2	-0.227	0.296	-0.765	0.445	0.797	0.236	-0.858	0.391	0.446	1.425
ARRB1	-0.213	0.296	-0.719	0.472	0.808	0.239	-0.801	0.423	0.452	1.444
ARR3	0.158	0.297	0.532	0.595	1.171	0.348	0.492	0.623	0.654	2.096
ARPP21	0.289	0.296	0.974	0.33	1.335	0.396	0.846	0.398	0.747	2.387
ARPP19	-0.343	0.3	-1.143	0.253	0.71	0.213	-1.364	0.173	0.395	1.277
ARPIN	0.316	0.3	1.055	0.291	1.372	0.411	0.905	0.366	0.763	2.467
ARPC5L	0.857	0.31	2.762	0.006	2.357	0.732	1.855	0.064	1.283	4.33
ARPC5	-0.146	0.296	-0.492	0.623	0.864	0.256	-0.53	0.596	0.484	1.544
ARPC4	-0.266	0.298	-0.892	0.372	0.767	0.228	-1.022	0.307	0.428	1.374
ARPC3	-0.52	0.302	-1.718	0.086	0.595	0.18	-2.253	0.024	0.329	1.076
ARPC2	0.154	0.295	0.52	0.603	1.166	0.345	0.482	0.63	0.653	2.081
ARPC1B	-0.036	0.295	-0.121	0.904	0.965	0.285	-0.123	0.902	0.541	1.721
ARPC1A	-0.291	0.3	-0.971	0.332	0.748	0.224	-1.127	0.26	0.416	1.345
ARNTL2	-0.45	0.3	-1.5	0.133	0.637	0.191	-1.895	0.058	0.354	1.148
ARNTL	-0.142	0.297	-0.477	0.633	0.868	0.257	-0.513	0.608	0.485	1.552

ARNT2	0.026	0.297	0.087	0.93	1.026	0.304	0.086	0.931	0.574	1.836
ARNT	0.23	0.297	0.777	0.437	1.259	0.373	0.694	0.488	0.704	2.252
ARMT1	-0.203	0.298	-0.683	0.495	0.816	0.243	-0.757	0.449	0.455	1.463
ARMH3	-0.181	0.296	-0.613	0.54	0.834	0.247	-0.672	0.502	0.467	1.49
ARMCX6	0.506	0.301	1.683	0.092	1.659	0.499	1.321	0.187	0.92	2.992
ARMCX5	0.133	0.297	0.447	0.655	1.142	0.339	0.418	0.676	0.638	2.044
ARMCX4	0.645	0.303	2.131	0.033	1.907	0.577	1.57	0.116	1.053	3.451
ARMCX3	0.079	0.295	0.268	0.789	1.082	0.32	0.257	0.797	0.607	1.931
ARMCX2	-0.672	0.31	-2.17	0.03	0.511	0.158	-3.094	0.002	0.278	0.937
ARMCX1	-0.414	0.3	-1.38	0.167	0.661	0.198	-1.71	0.087	0.367	1.19
ARMC9	-0.059	0.296	-0.199	0.842	0.943	0.279	-0.205	0.838	0.528	1.683
ARMC8	-0.035	0.295	-0.118	0.906	0.966	0.285	-0.12	0.904	0.541	1.723
ARMC7	0.219	0.296	0.739	0.46	1.245	0.368	0.664	0.507	0.697	2.223
ARMC6	0.671	0.31	2.165	0.03	1.956	0.606	1.577	0.115	1.065	3.589
ARMC4	-0.188	0.296	-0.633	0.527	0.829	0.246	-0.696	0.486	0.464	1.482
ARMC1	0.782	0.31	2.525	0.012	2.186	0.677	1.752	0.08	1.191	4.013
ARL8B	-0.756	0.315	-2.402	0.016	0.469	0.148	-3.59	0	0.253	0.87
ARL6IP5	-0.781	0.31	-2.52	0.012	0.458	0.142	-3.819	0	0.25	0.841
ARL6IP4	-0.222	0.301	-0.737	0.461	0.801	0.241	-0.826	0.409	0.444	1.445
ARL6IP1	0.853	0.315	2.71	0.007	2.348	0.739	1.823	0.068	1.266	4.352
ARL4D	0.338	0.3	1.126	0.26	1.402	0.42	0.955	0.339	0.779	2.522
ARL4C	-0.071	0.295	-0.24	0.81	0.931	0.275	-0.249	0.803	0.522	1.662
ARL4A	0.403	0.302	1.334	0.182	1.497	0.453	1.098	0.272	0.828	2.707
ARL3	0.028	0.299	0.095	0.924	1.029	0.307	0.094	0.925	0.573	1.848
ARL2BP	-0.39	0.299	-1.305	0.192	0.677	0.202	-1.597	0.11	0.377	1.216
ARL2	0.41	0.298	1.374	0.17	1.507	0.449	1.127	0.26	0.84	2.703
ARL15	0.339	0.298	1.137	0.256	1.403	0.418	0.964	0.335	0.783	2.515
ARL14	-0.332	0.298	-1.116	0.264	0.717	0.214	-1.324	0.186	0.4	1.286
ARL1	0.249	0.296	0.84	0.401	1.282	0.38	0.744	0.457	0.718	2.291
ARIH2	-0.171	0.296	-0.577	0.564	0.843	0.25	-0.629	0.529	0.472	1.507
ARIH1	-0.554	0.306	-1.81	0.07	0.575	0.176	-2.418	0.016	0.316	1.047
ARID5B	-0.429	0.297	-1.442	0.149	0.651	0.194	-1.8	0.072	0.364	1.167
ARID5A	0.062	0.296	0.208	0.835	1.064	0.314	0.202	0.84	0.596	1.899
ARID4B	-0.218	0.296	-0.737	0.461	0.804	0.238	-0.823	0.41	0.45	1.436
ARID4A	-0.502	0.306	-1.643	0.1	0.605	0.185	-2.134	0.033	0.332	1.102
ARID3B	-0.205	0.295	-0.694	0.488	0.815	0.241	-0.77	0.441	0.457	1.454
ARID3A	-0.155	0.296	-0.523	0.601	0.857	0.253	-0.566	0.572	0.48	1.53
ARID1A	-0.598	0.303	-1.974	0.048	0.55	0.167	-2.701	0.007	0.304	0.996

ARHGEF9	0.367	0.3	1.224	0.221	1.443	0.432	1.025	0.305	0.802	2.597
ARHGEF7	0.758	0.31	2.446	0.014	2.135	0.662	1.714	0.086	1.163	3.92
ARHGEF6	0.209	0.296	0.707	0.479	1.233	0.365	0.638	0.523	0.69	2.202
ARHGEF5	0.225	0.296	0.762	0.446	1.253	0.371	0.682	0.495	0.702	2.237
ARHGEF40	0.126	0.295	0.427	0.67	1.134	0.335	0.401	0.688	0.636	2.024
ARHGEF4	0.122	0.296	0.412	0.68	1.13	0.335	0.388	0.698	0.632	2.02
ARHGEF38	0.143	0.295	0.485	0.627	1.154	0.341	0.452	0.651	0.647	2.059
ARHGEF3	-0.471	0.3	-1.572	0.116	0.624	0.187	-2.008	0.045	0.347	1.123
ARHGEF28	-0.177	0.296	-0.598	0.55	0.838	0.248	-0.654	0.513	0.469	1.497
ARHGEF26	-0.387	0.303	-1.279	0.201	0.679	0.206	-1.561	0.118	0.375	1.229
ARHGEF2	0.084	0.295	0.283	0.777	1.087	0.321	0.272	0.786	0.609	1.94
ARHGEF18	-0.174	0.296	-0.589	0.556	0.84	0.248	-0.644	0.52	0.471	1.499
ARHGEF17	-0.076	0.295	-0.256	0.798	0.927	0.274	-0.266	0.79	0.52	1.654
ARHGEF16	-0.078	0.297	-0.261	0.794	0.925	0.275	-0.272	0.786	0.517	1.657
ARHGEF15	0.165	0.298	0.552	0.581	1.179	0.351	0.509	0.61	0.657	2.114
ARHGEF12	-0.098	0.296	-0.333	0.739	0.906	0.268	-0.35	0.726	0.508	1.618
ARHGEF11	-0.175	0.296	-0.592	0.554	0.839	0.248	-0.647	0.517	0.47	1.499
ARHGEF10L	0.335	0.298	1.126	0.26	1.398	0.416	0.957	0.339	0.78	2.506
ARHGEF10	-0.425	0.3	-1.418	0.156	0.654	0.196	-1.767	0.077	0.364	1.176
ARHGDIG	0.15	0.296	0.507	0.612	1.162	0.345	0.471	0.638	0.65	2.078
ARHGDIB	-0.323	0.3	-1.075	0.282	0.724	0.217	-1.269	0.205	0.402	1.304
ARHGDIA	-0.203	0.296	-0.684	0.494	0.816	0.242	-0.759	0.448	0.457	1.459
ARHGAP6	-0.234	0.297	-0.79	0.429	0.791	0.235	-0.89	0.373	0.442	1.415
ARHGAP5	-0.747	0.31	-2.409	0.016	0.474	0.147	-3.583	0	0.258	0.87
ARHGAP45	0.02	0.296	0.066	0.947	1.02	0.301	0.065	0.948	0.571	1.82
ARHGAP44	-0.008	0.296	-0.026	0.979	0.992	0.294	-0.026	0.979	0.555	1.774
ARHGAP4	-0.475	0.302	-1.572	0.116	0.622	0.188	-2.013	0.044	0.344	1.124
ARHGAP35	0.158	0.295	0.536	0.592	1.171	0.346	0.496	0.62	0.657	2.09
ARHGAP33	0.663	0.303	2.185	0.029	1.94	0.588	1.598	0.11	1.071	3.514
ARHGAP32	-0.325	0.298	-1.093	0.274	0.722	0.215	-1.292	0.196	0.403	1.294
ARHGAP29	-0.072	0.295	-0.243	0.808	0.931	0.275	-0.252	0.801	0.522	1.66
ARHGAP28	-0.18	0.296	-0.608	0.543	0.835	0.247	-0.667	0.505	0.467	1.492
ARHGAP26	-0.267	0.298	-0.898	0.369	0.766	0.228	-1.029	0.303	0.427	1.372
ARHGAP25	-0.459	0.301	-1.525	0.127	0.632	0.19	-1.935	0.053	0.351	1.14
ARHGAP24	0.069	0.295	0.234	0.815	1.072	0.317	0.226	0.821	0.601	1.912
ARHGAP22	-0.127	0.296	-0.428	0.669	0.881	0.261	-0.456	0.648	0.494	1.573
ARHGAP19	-0.508	0.3	-1.696	0.09	0.602	0.18	-2.21	0.027	0.334	1.082
ARHGAP17	0.509	0.303	1.682	0.093	1.664	0.504	1.318	0.188	0.919	3.013

ARHGAP12	-0.2	0.296	-0.676	0.499	0.819	0.242	-0.748	0.454	0.458	1.463
ARHGAP11A	0.691	0.31	2.229	0.026	1.995	0.618	1.61	0.107	1.087	3.661
ARHGAP10	0.111	0.295	0.377	0.706	1.118	0.33	0.357	0.721	0.627	1.994
ARHGAP1	-0.036	0.295	-0.121	0.903	0.965	0.285	-0.124	0.902	0.541	1.72
ARGLU1	0.152	0.298	0.511	0.609	1.164	0.347	0.474	0.636	0.649	2.088
ARG2	0.84	0.315	2.667	0.008	2.315	0.729	1.804	0.071	1.249	4.292
ARG1	-0.444	0.298	-1.487	0.137	0.642	0.191	-1.872	0.061	0.357	1.151
ARFRP1	0.351	0.301	1.165	0.244	1.42	0.428	0.983	0.326	0.787	2.563
ARFIP2	-0.581	0.303	-1.914	0.056	0.559	0.17	-2.596	0.009	0.309	1.014
ARFIP1	0.437	0.299	1.463	0.144	1.548	0.462	1.185	0.236	0.862	2.779
ARFGEF2	-0.402	0.299	-1.346	0.178	0.669	0.2	-1.657	0.098	0.373	1.201
ARFGEF1	-0.245	0.298	-0.824	0.41	0.782	0.233	-0.934	0.35	0.436	1.403
ARFGAP3	-0.212	0.298	-0.712	0.477	0.809	0.241	-0.793	0.428	0.451	1.451
ARFGAP2	-0.627	0.31	-2.024	0.043	0.534	0.165	-2.814	0.005	0.291	0.98
ARFGAP1	0.484	0.306	1.583	0.113	1.623	0.497	1.255	0.21	0.891	2.956
ARF6	0.663	0.306	2.166	0.03	1.94	0.593	1.584	0.113	1.065	3.533
ARF5	0.398	0.3	1.325	0.185	1.489	0.447	1.093	0.275	0.826	2.683
ARF4	-0.364	0.298	-1.224	0.221	0.695	0.207	-1.476	0.14	0.388	1.245
ARF3	-0.625	0.304	-2.057	0.04	0.535	0.163	-2.857	0.004	0.295	0.971
AREL1	-0.41	0.299	-1.372	0.17	0.663	0.198	-1.697	0.09	0.369	1.192
AREG	-0.523	0.306	-1.708	0.088	0.593	0.181	-2.244	0.025	0.325	1.08
ARCN1	-0.502	0.302	-1.66	0.097	0.605	0.183	-2.155	0.031	0.335	1.095
ARC	0.008	0.296	0.027	0.978	1.008	0.298	0.027	0.978	0.564	1.801
ARAP3	-0.005	0.295	-0.017	0.986	0.995	0.293	-0.017	0.986	0.558	1.774
ARAP1	0.233	0.296	0.786	0.432	1.262	0.374	0.701	0.483	0.706	2.255
ARAF	-0.117	0.295	-0.396	0.692	0.89	0.263	-0.42	0.675	0.499	1.587
AR	-0.011	0.295	-0.036	0.971	0.989	0.292	-0.036	0.971	0.555	1.764
AQR	-0.462	0.302	-1.53	0.126	0.63	0.19	-1.945	0.052	0.348	1.139
AQP9	0.735	0.31	2.372	0.018	2.085	0.646	1.68	0.093	1.136	3.826
AQP8	0.375	0.304	1.235	0.217	1.455	0.442	1.03	0.303	0.802	2.64
AQP6	0.508	0.302	1.68	0.093	1.662	0.502	1.318	0.188	0.919	3.006
AQP5	-0.086	0.295	-0.291	0.771	0.918	0.271	-0.304	0.761	0.514	1.637
AQP4	0.302	0.296	1.021	0.307	1.353	0.401	0.881	0.378	0.757	2.418
AQP3	-0.328	0.299	-1.095	0.273	0.72	0.216	-1.296	0.195	0.401	1.296
AQP2	0.554	0.306	1.809	0.07	1.74	0.533	1.389	0.165	0.955	3.171
AQP1	-0.41	0.3	-1.368	0.171	0.664	0.199	-1.691	0.091	0.369	1.194
APTX	0.36	0.298	1.208	0.227	1.433	0.427	1.015	0.31	0.799	2.568
APRT	0.363	0.298	1.218	0.223	1.437	0.428	1.022	0.307	0.802	2.577

APPL2	-1.153	0.33	-3.493	0	0.316	0.104	-6.567	0	0.165	0.603
APPL1	-0.229	0.299	-0.766	0.444	0.795	0.238	-0.861	0.389	0.442	1.43
APPBP2	0.116	0.295	0.391	0.696	1.122	0.332	0.369	0.712	0.629	2.003
APP	-0.297	0.298	-0.998	0.318	0.743	0.221	-1.162	0.245	0.414	1.332
APOOL	0.421	0.299	1.408	0.159	1.523	0.456	1.149	0.25	0.848	2.737
APOO	0.017	0.296	0.056	0.955	1.017	0.301	0.056	0.956	0.57	1.815
APOM	0.399	0.298	1.34	0.18	1.491	0.444	1.105	0.269	0.831	2.674
APOLD1	0.219	0.297	0.736	0.461	1.245	0.37	0.661	0.508	0.695	2.229
APOL6	0.182	0.297	0.614	0.539	1.2	0.356	0.561	0.574	0.671	2.148
APOL5	0.031	0.296	0.105	0.916	1.032	0.305	0.103	0.918	0.577	1.843
APOL3	-0.219	0.297	-0.738	0.46	0.803	0.238	-0.825	0.409	0.449	1.437
APOL2	-0.034	0.295	-0.116	0.907	0.966	0.285	-0.119	0.906	0.541	1.724
APOL1	-0.477	0.3	-1.587	0.112	0.621	0.186	-2.034	0.042	0.345	1.118
APOH	-0.029	0.295	-0.099	0.921	0.971	0.287	-0.1	0.92	0.545	1.732
APOF	0.566	0.306	1.852	0.064	1.762	0.539	1.414	0.157	0.968	3.208
APOE	0.363	0.3	1.21	0.226	1.437	0.431	1.015	0.31	0.799	2.587
APOD	0.034	0.297	0.113	0.91	1.034	0.307	0.111	0.911	0.578	1.849
APOC3	0.756	0.31	2.439	0.015	2.131	0.661	1.711	0.087	1.16	3.913
APOC1	0.459	0.3	1.527	0.127	1.582	0.475	1.225	0.221	0.878	2.849
APOBR	-0.272	0.298	-0.914	0.361	0.762	0.227	-1.05	0.294	0.425	1.365
APOBEC3G	-0.263	0.298	-0.884	0.377	0.768	0.229	-1.011	0.312	0.428	1.378
APOBEC3F	0.166	0.301	0.552	0.581	1.181	0.355	0.508	0.611	0.655	2.13
APOBEC3C	-0.119	0.295	-0.404	0.686	0.888	0.262	-0.429	0.668	0.498	1.583
APOBEC3B	0.996	0.321	3.104	0.002	2.706	0.868	1.966	0.049	1.443	5.075
APOBEC2	-0.009	0.295	-0.031	0.975	0.991	0.292	-0.031	0.975	0.556	1.767
APOBEC1	-0.026	0.295	-0.088	0.929	0.974	0.288	-0.09	0.929	0.546	1.738
APOB	-0.061	0.295	-0.206	0.837	0.941	0.278	-0.213	0.832	0.527	1.679
APOA4	-0.004	0.296	-0.014	0.989	0.996	0.295	-0.014	0.988	0.557	1.78
APOA2	-0.016	0.295	-0.055	0.956	0.984	0.29	-0.056	0.955	0.552	1.754
APOA1	0.637	0.306	2.08	0.038	1.891	0.579	1.538	0.124	1.037	3.446
APMAP	-0.298	0.298	-1	0.317	0.743	0.221	-1.164	0.244	0.414	1.331
APLP2	-0.737	0.31	-2.377	0.017	0.479	0.148	-3.514	0	0.261	0.879
APLP1	0.129	0.297	0.434	0.664	1.138	0.338	0.407	0.684	0.636	2.036
APLNR	0.225	0.298	0.757	0.449	1.253	0.373	0.678	0.498	0.699	2.245
APIP	0.327	0.298	1.097	0.273	1.386	0.413	0.936	0.349	0.773	2.484
API5	0.217	0.299	0.727	0.467	1.242	0.371	0.653	0.514	0.692	2.23
APH1B	-0.186	0.298	-0.624	0.532	0.83	0.247	-0.686	0.493	0.463	1.489
APH1A	0.57	0.302	1.888	0.059	1.769	0.534	1.439	0.15	0.978	3.197

APEX2	0.271	0.301	0.901	0.367	1.312	0.395	0.789	0.43	0.727	2.368
APEX1	0.266	0.297	0.897	0.37	1.305	0.387	0.788	0.431	0.729	2.335
APEH	-0.106	0.297	-0.356	0.722	0.9	0.267	-0.376	0.707	0.503	1.609
APCS	0.109	0.296	0.369	0.712	1.115	0.33	0.349	0.727	0.625	1.991
APC2	0.33	0.296	1.114	0.265	1.391	0.412	0.949	0.343	0.778	2.488
APC	0.289	0.296	0.974	0.33	1.335	0.396	0.846	0.398	0.747	2.387
APBB2	-0.303	0.298	-1.016	0.309	0.739	0.22	-1.187	0.235	0.412	1.324
APBB1IP	-0.444	0.3	-1.482	0.138	0.641	0.192	-1.866	0.062	0.356	1.154
APBB1	-0.292	0.298	-0.979	0.327	0.747	0.222	-1.137	0.255	0.417	1.339
APBA3	0.054	0.298	0.183	0.855	1.056	0.314	0.178	0.859	0.589	1.892
APBA2	-0.118	0.296	-0.398	0.691	0.889	0.263	-0.422	0.673	0.498	1.588
APBA1	0.273	0.297	0.919	0.358	1.314	0.39	0.805	0.421	0.734	2.352
APAF1	0.464	0.3	1.548	0.122	1.59	0.476	1.239	0.215	0.884	2.861
AP5S1	-0.062	0.295	-0.21	0.833	0.94	0.277	-0.217	0.828	0.527	1.676
AP5M1	-0.044	0.295	-0.148	0.882	0.957	0.283	-0.151	0.88	0.537	1.708
AP4S1	-0.342	0.301	-1.137	0.255	0.71	0.214	-1.356	0.175	0.394	1.281
AP4M1	-0.005	0.297	-0.016	0.987	0.995	0.296	-0.016	0.987	0.556	1.783
AP4E1	-0.072	0.295	-0.243	0.808	0.931	0.275	-0.252	0.801	0.522	1.66
AP3S1	-0.162	0.296	-0.548	0.584	0.85	0.252	-0.595	0.552	0.476	1.519
AP3M2	-0.077	0.297	-0.259	0.795	0.926	0.275	-0.269	0.788	0.518	1.657
AP3D1	-0.01	0.296	-0.034	0.973	0.99	0.293	-0.034	0.973	0.555	1.767
AP3B2	0.057	0.295	0.193	0.847	1.059	0.313	0.187	0.852	0.593	1.888
AP3B1	-1.049	0.322	-3.252	0.001	0.35	0.113	-5.748	0	0.186	0.659
AP2S1	0.397	0.299	1.325	0.185	1.487	0.445	1.094	0.274	0.827	2.673
AP2M1	0.133	0.296	0.449	0.653	1.143	0.339	0.421	0.674	0.639	2.043
AP2B1	-0.151	0.296	-0.509	0.611	0.86	0.255	-0.549	0.583	0.481	1.537
AP2A2	-0.707	0.31	-2.284	0.022	0.493	0.153	-3.321	0.001	0.269	0.905
AP1S2	-0.008	0.295	-0.027	0.979	0.992	0.293	-0.027	0.978	0.556	1.769
AP1S1	0.312	0.3	1.041	0.298	1.366	0.41	0.894	0.371	0.759	2.459
AP1M2	0.069	0.296	0.233	0.816	1.071	0.317	0.225	0.822	0.6	1.914
AP1G2	0.185	0.296	0.623	0.533	1.203	0.356	0.569	0.569	0.673	2.149
AP1G1	0.526	0.302	1.74	0.082	1.692	0.511	1.353	0.176	0.936	3.059
AP1B1	0.709	0.306	2.319	0.02	2.033	0.622	1.661	0.097	1.116	3.702
AP1AR	-0.005	0.295	-0.018	0.985	0.995	0.294	-0.018	0.985	0.558	1.774
AOX1	-0.546	0.303	-1.803	0.071	0.579	0.175	-2.399	0.016	0.32	1.049
AOPEP	-0.304	0.297	-1.025	0.306	0.738	0.219	-1.197	0.231	0.413	1.32
AOC4P	0.631	0.306	2.063	0.039	1.879	0.574	1.53	0.126	1.032	3.421
AOC3	0.316	0.298	1.062	0.288	1.372	0.409	0.91	0.363	0.765	2.459

AOC2	0.23	0.298	0.771	0.441	1.258	0.375	0.689	0.491	0.702	2.256
AOC1	0.558	0.306	1.826	0.068	1.747	0.534	1.399	0.162	0.96	3.181
AOAH	-0.326	0.3	-1.086	0.277	0.722	0.217	-1.284	0.199	0.401	1.3
ANXA9	0.203	0.298	0.681	0.496	1.225	0.365	0.617	0.538	0.683	2.198
ANXA7	0.229	0.297	0.771	0.441	1.257	0.373	0.689	0.491	0.703	2.249
ANXA6	-0.093	0.296	-0.313	0.754	0.911	0.27	-0.328	0.743	0.51	1.628
ANXA5	0.036	0.296	0.12	0.904	1.036	0.306	0.118	0.906	0.58	1.85
ANXA4	-0.397	0.3	-1.325	0.185	0.672	0.201	-1.627	0.104	0.374	1.209
ANXA3	0.017	0.296	0.059	0.953	1.018	0.301	0.058	0.954	0.57	1.816
ANXA2P3	0.137	0.295	0.465	0.642	1.147	0.339	0.434	0.664	0.643	2.046
ANXA2P2	-0.115	0.296	-0.39	0.697	0.891	0.264	-0.413	0.679	0.499	1.592
ANXA2P1	0.399	0.299	1.338	0.181	1.491	0.445	1.103	0.27	0.831	2.677
ANXA2	0.35	0.296	1.179	0.238	1.418	0.421	0.995	0.32	0.793	2.536
ANXA13	-0.379	0.3	-1.264	0.206	0.685	0.205	-1.536	0.124	0.381	1.232
ANXA11	-0.097	0.295	-0.33	0.742	0.907	0.268	-0.346	0.729	0.508	1.619
ANXA10	-0.421	0.3	-1.403	0.161	0.656	0.197	-1.745	0.081	0.364	1.182
ANXA1	-0.816	0.31	-2.628	0.009	0.442	0.137	-4.062	0	0.241	0.813
ANTXR1	-0.375	0.3	-1.252	0.21	0.687	0.206	-1.52	0.129	0.382	1.236
ANTKMT	0.236	0.297	0.793	0.428	1.266	0.376	0.707	0.48	0.707	2.265
ANPEP	-0.35	0.3	-1.169	0.242	0.704	0.211	-1.4	0.161	0.392	1.267
ANP32E	0.582	0.303	1.92	0.055	1.789	0.542	1.456	0.146	0.988	3.241
ANP32D	-0.238	0.296	-0.804	0.422	0.788	0.234	-0.907	0.364	0.441	1.409
ANP32C	-0.584	0.316	-1.848	0.065	0.558	0.176	-2.51	0.012	0.3	1.036
ANP32B	0.19	0.296	0.642	0.521	1.209	0.358	0.584	0.559	0.677	2.161
ANP32A-IT1	-0.282	0.297	-0.95	0.342	0.754	0.224	-1.097	0.273	0.421	1.351
ANP32A	-0.172	0.296	-0.581	0.561	0.842	0.249	-0.634	0.526	0.471	1.504
ANOS1	0.005	0.295	0.018	0.986	1.005	0.297	0.018	0.986	0.564	1.793
ANO3	0.03	0.296	0.102	0.918	1.031	0.305	0.101	0.92	0.577	1.84
ANO2	0.192	0.296	0.647	0.517	1.211	0.358	0.589	0.556	0.678	2.163
ANO10	0.355	0.298	1.191	0.234	1.426	0.425	1.003	0.316	0.795	2.556
ANO1	-0.63	0.306	-2.061	0.039	0.533	0.163	-2.871	0.004	0.293	0.97
ANKZF1	-0.511	0.302	-1.69	0.091	0.6	0.181	-2.207	0.027	0.332	1.085
ANKS1B	-0.529	0.306	-1.731	0.083	0.589	0.18	-2.282	0.022	0.323	1.072
ANKS1A	0.427	0.3	1.425	0.154	1.533	0.46	1.16	0.246	0.852	2.759
ANKRD7	0.569	0.303	1.877	0.061	1.766	0.535	1.431	0.152	0.975	3.197
ANKRD6	-0.232	0.296	-0.783	0.434	0.793	0.235	-0.881	0.378	0.444	1.417
ANKRD55	0.273	0.297	0.919	0.358	1.314	0.391	0.804	0.421	0.734	2.355
ANKRD53	0.1	0.297	0.338	0.735	1.106	0.328	0.322	0.747	0.618	1.977

ANKRD49	-0.278	0.298	-0.933	0.351	0.757	0.225	-1.076	0.282	0.423	1.358
ANKRD46	0.24	0.296	0.811	0.417	1.271	0.377	0.721	0.471	0.712	2.272
ANKRD40CL	0.226	0.296	0.763	0.445	1.254	0.372	0.683	0.495	0.701	2.242
ANKRD40	0.41	0.3	1.368	0.171	1.507	0.452	1.123	0.262	0.838	2.712
ANKRD36BP2	-0.102	0.296	-0.345	0.73	0.903	0.268	-0.363	0.717	0.505	1.614
ANKRD36B	0.433	0.303	1.43	0.153	1.542	0.467	1.161	0.246	0.852	2.791
ANKRD36	0.377	0.298	1.265	0.206	1.458	0.435	1.054	0.292	0.813	2.615
ANKRD34C	0.092	0.296	0.31	0.756	1.096	0.324	0.296	0.767	0.614	1.957
ANKRD28	-0.249	0.298	-0.836	0.403	0.78	0.232	-0.95	0.342	0.435	1.397
ANKRD27	0.327	0.301	1.088	0.276	1.387	0.417	0.928	0.353	0.77	2.5
ANKRD26	0.301	0.299	1.005	0.315	1.351	0.405	0.868	0.386	0.751	2.43
ANKRD2	0.315	0.299	1.054	0.292	1.37	0.409	0.904	0.366	0.763	2.461
ANKRD17	0.418	0.303	1.379	0.168	1.519	0.46	1.127	0.26	0.839	2.749
ANKRD12	-0.463	0.302	-1.531	0.126	0.629	0.19	-1.947	0.052	0.348	1.139
ANKRD11	0.454	0.302	1.501	0.133	1.574	0.476	1.206	0.228	0.87	2.848
ANKRD10-IT1	0.081	0.297	0.272	0.786	1.084	0.322	0.261	0.794	0.606	1.941
ANKRD10	0.016	0.295	0.055	0.956	1.016	0.3	0.054	0.957	0.57	1.814
ANKRD1	-0.113	0.298	-0.38	0.704	0.893	0.266	-0.402	0.688	0.498	1.602
ANKRA2	-0.618	0.31	-1.995	0.046	0.539	0.167	-2.762	0.006	0.294	0.989
ANKMY2	-0.079	0.297	-0.266	0.79	0.924	0.274	-0.277	0.782	0.517	1.653
ANKMY1	-0.138	0.295	-0.468	0.64	0.871	0.257	-0.502	0.615	0.488	1.554
ANKLE2	-0.583	0.31	-1.881	0.06	0.558	0.173	-2.554	0.011	0.304	1.025
ANKH	-0.292	0.298	-0.981	0.327	0.747	0.222	-1.139	0.255	0.417	1.338
ANKFY1	0.076	0.295	0.258	0.796	1.079	0.318	0.249	0.804	0.605	1.924
ANKEF1	-0.072	0.297	-0.243	0.808	0.93	0.276	-0.252	0.801	0.52	1.665
ANK3	0.182	0.296	0.614	0.539	1.199	0.355	0.561	0.575	0.671	2.144
ANK2	-0.403	0.298	-1.354	0.176	0.668	0.199	-1.667	0.095	0.373	1.198
ANK1	0.142	0.297	0.478	0.632	1.152	0.342	0.446	0.656	0.644	2.061
ANGPTL8	0.084	0.296	0.285	0.776	1.088	0.322	0.273	0.784	0.609	1.942
ANGPTL7	0.129	0.295	0.437	0.662	1.138	0.336	0.41	0.682	0.638	2.03
ANGPTL4	0.787	0.31	2.535	0.011	2.196	0.681	1.755	0.079	1.195	4.034
ANGPTL3	0.221	0.296	0.75	0.454	1.248	0.369	0.672	0.501	0.699	2.227
ANGPTL2	-0.371	0.298	-1.247	0.212	0.69	0.205	-1.51	0.131	0.385	1.237
ANGPT4	0.212	0.298	0.713	0.476	1.237	0.368	0.643	0.52	0.69	2.217
ANGPT2	0.088	0.295	0.299	0.765	1.092	0.323	0.286	0.775	0.612	1.949
ANGPT1	0.2	0.298	0.67	0.503	1.221	0.364	0.608	0.543	0.681	2.19
ANGEL2	-0.199	0.296	-0.674	0.501	0.819	0.243	-0.745	0.456	0.458	1.464
ANGEL1	-0.811	0.31	-2.612	0.009	0.444	0.138	-4.026	0	0.242	0.817

ANG	-0.435	0.302	-1.439	0.15	0.647	0.196	-1.803	0.071	0.358	1.171
ANAPC5	-0.115	0.296	-0.389	0.697	0.891	0.264	-0.412	0.68	0.499	1.591
ANAPC2	-0.205	0.296	-0.692	0.489	0.815	0.241	-0.768	0.442	0.456	1.456
ANAPC15	0.478	0.302	1.581	0.114	1.612	0.487	1.257	0.209	0.892	2.915
ANAPC13	-0.026	0.295	-0.089	0.929	0.974	0.288	-0.09	0.928	0.546	1.738
ANAPC10	0.649	0.303	2.139	0.032	1.913	0.58	1.573	0.116	1.056	3.467
AMZ2	0.243	0.298	0.816	0.414	1.275	0.379	0.725	0.469	0.712	2.284
AMT	0.502	0.306	1.643	0.1	1.653	0.505	1.292	0.196	0.908	3.009
AMPH	-0.608	0.303	-2.005	0.045	0.545	0.165	-2.759	0.006	0.301	0.986
AMPD3	0.183	0.296	0.619	0.536	1.201	0.356	0.565	0.572	0.672	2.148
AMPD2	-0.024	0.296	-0.082	0.935	0.976	0.289	-0.083	0.934	0.547	1.743
AMPD1	-0.468	0.3	-1.561	0.118	0.626	0.188	-1.991	0.046	0.348	1.127
AMOTL2	-0.068	0.295	-0.23	0.818	0.934	0.276	-0.238	0.812	0.524	1.666
AMOT	-0.027	0.296	-0.09	0.928	0.974	0.288	-0.091	0.927	0.545	1.74
AMN	0.639	0.303	2.106	0.035	1.895	0.575	1.556	0.12	1.045	3.434
AMMECR1	0.17	0.296	0.574	0.566	1.185	0.351	0.528	0.598	0.663	2.118
AMIGO2	-0.545	0.303	-1.801	0.072	0.58	0.175	-2.395	0.017	0.32	1.049
AMHR2	-0.032	0.296	-0.108	0.914	0.969	0.287	-0.109	0.913	0.542	1.73
AMFR	-0.383	0.3	-1.279	0.201	0.682	0.204	-1.558	0.119	0.379	1.227
AMELY	0.438	0.3	1.46	0.144	1.549	0.465	1.182	0.237	0.861	2.789
AMELX	0.756	0.308	2.45	0.014	2.129	0.657	1.719	0.086	1.163	3.897
AMD1	0.192	0.299	0.641	0.522	1.211	0.362	0.583	0.56	0.674	2.176
AMBRA1	0.252	0.297	0.85	0.395	1.287	0.382	0.751	0.452	0.719	2.302
AMBP	-0.42	0.299	-1.407	0.159	0.657	0.196	-1.749	0.08	0.366	1.179
AMBN	-0.805	0.31	-2.594	0.009	0.447	0.139	-3.986	0	0.243	0.821
AMACR	0.541	0.301	1.798	0.072	1.718	0.517	1.388	0.165	0.952	3.099
ALX4	0.352	0.3	1.174	0.241	1.421	0.426	0.99	0.322	0.79	2.557
ALX3	0.037	0.297	0.124	0.901	1.038	0.308	0.122	0.903	0.58	1.857
ALX1	-0.065	0.295	-0.22	0.826	0.937	0.277	-0.228	0.82	0.525	1.671
ALS2CL	0.196	0.299	0.655	0.512	1.216	0.363	0.595	0.552	0.677	2.184
ALPP	0.226	0.297	0.762	0.446	1.254	0.372	0.682	0.495	0.701	2.243
ALPL	-0.141	0.296	-0.476	0.634	0.869	0.257	-0.511	0.609	0.486	1.552
ALPK3	-0.797	0.312	-2.558	0.011	0.451	0.14	-3.912	0	0.245	0.83
ALPK1	-0.193	0.295	-0.653	0.514	0.824	0.244	-0.721	0.471	0.462	1.471
ALPI	-0.013	0.295	-0.045	0.964	0.987	0.291	-0.045	0.964	0.553	1.76
ALPG	-0.112	0.296	-0.379	0.705	0.894	0.265	-0.401	0.688	0.5	1.598
ALOXE3	0.33	0.3	1.098	0.272	1.39	0.417	0.935	0.35	0.772	2.504
ALOX5AP	-0.223	0.298	-0.749	0.454	0.8	0.238	-0.839	0.401	0.446	1.434

ALOX5	0.095	0.295	0.322	0.747	1.1	0.325	0.307	0.759	0.617	1.962
ALOX15B	-0.25	0.296	-0.842	0.4	0.779	0.231	-0.956	0.339	0.436	1.393
ALOX15	0.705	0.315	2.24	0.025	2.024	0.637	1.607	0.108	1.092	3.752
ALOX12P2	-0.31	0.298	-1.04	0.298	0.733	0.219	-1.219	0.223	0.409	1.316
ALOX12B	0.331	0.297	1.113	0.266	1.392	0.414	0.948	0.343	0.777	2.492
ALOX12	-0.122	0.296	-0.411	0.681	0.885	0.262	-0.437	0.662	0.496	1.582
ALMS1	0.187	0.298	0.628	0.53	1.206	0.36	0.573	0.567	0.672	2.164
ALLC	-0.223	0.296	-0.754	0.451	0.8	0.237	-0.845	0.398	0.448	1.429
ALKBH4	-0.272	0.301	-0.903	0.366	0.762	0.229	-1.038	0.299	0.423	1.374
ALKBH1	0.099	0.296	0.333	0.739	1.104	0.327	0.317	0.751	0.618	1.972
ALK	0.547	0.306	1.789	0.074	1.728	0.528	1.378	0.168	0.949	3.144
ALG9	-0.074	0.297	-0.248	0.804	0.929	0.276	-0.257	0.797	0.52	1.661
ALG8	-0.169	0.296	-0.569	0.569	0.845	0.25	-0.62	0.536	0.473	1.51
ALG6	-0.104	0.296	-0.35	0.726	0.902	0.266	-0.369	0.712	0.505	1.609
ALG5	0.003	0.295	0.009	0.992	1.003	0.296	0.009	0.992	0.562	1.788
ALG3	1.04	0.328	3.176	0.001	2.83	0.927	1.974	0.048	1.489	5.377
ALG13	-0.616	0.31	-1.989	0.047	0.54	0.167	-2.75	0.006	0.294	0.991
ALG12	0.215	0.296	0.726	0.468	1.24	0.367	0.653	0.514	0.694	2.214
ALDOC	0.356	0.303	1.178	0.239	1.428	0.432	0.991	0.322	0.789	2.584
ALDOB	0.026	0.296	0.089	0.929	1.027	0.304	0.088	0.93	0.575	1.834
ALDOAP2	0.701	0.307	2.281	0.023	2.015	0.619	1.64	0.101	1.104	3.679
ALDOA	0.156	0.295	0.528	0.598	1.169	0.345	0.489	0.625	0.655	2.084
ALDH9A1	-0.207	0.298	-0.695	0.487	0.813	0.242	-0.773	0.44	0.453	1.457
ALDH8A1	0.34	0.3	1.133	0.257	1.405	0.422	0.96	0.337	0.78	2.533
ALDH7A1	0.33	0.298	1.109	0.267	1.391	0.414	0.944	0.345	0.776	2.495
ALDH6A1	0.046	0.296	0.156	0.876	1.047	0.31	0.153	0.879	0.587	1.87
ALDH5A1	0.088	0.296	0.297	0.766	1.092	0.323	0.284	0.776	0.612	1.949
ALDH4A1	-0.638	0.306	-2.084	0.037	0.528	0.162	-2.916	0.004	0.29	0.963
ALDH3B2	0.362	0.3	1.207	0.227	1.437	0.431	1.013	0.311	0.798	2.587
ALDH3B1	0.801	0.315	2.546	0.011	2.229	0.702	1.751	0.08	1.202	4.131
ALDH3A2	0.162	0.296	0.548	0.583	1.176	0.348	0.506	0.613	0.659	2.1
ALDH3A1	0.322	0.297	1.087	0.277	1.38	0.409	0.929	0.353	0.772	2.469
ALDH2	-0.183	0.297	-0.616	0.538	0.833	0.247	-0.676	0.499	0.466	1.49
ALDH1L1	0.055	0.295	0.185	0.853	1.056	0.312	0.18	0.857	0.592	1.885
ALDH1B1	-0.11	0.295	-0.371	0.711	0.896	0.265	-0.392	0.695	0.502	1.599
ALDH1A3	-0.311	0.298	-1.045	0.296	0.733	0.218	-1.226	0.22	0.409	1.313
ALDH1A2	0.589	0.305	1.932	0.053	1.802	0.549	1.46	0.144	0.991	3.274
ALDH1A1	-0.887	0.316	-2.809	0.005	0.412	0.13	-4.521	0	0.222	0.765

ALDH18A1	-0.396	0.3	-1.322	0.186	0.673	0.202	-1.622	0.105	0.374	1.211
ALCAM	-0.357	0.3	-1.19	0.234	0.7	0.21	-1.43	0.153	0.389	1.26
ALB	-0.473	0.302	-1.565	0.118	0.623	0.188	-2.002	0.045	0.345	1.127
ALAS2	0.642	0.307	2.093	0.036	1.9	0.583	1.545	0.122	1.042	3.465
ALAS1	0.24	0.297	0.809	0.418	1.271	0.377	0.719	0.472	0.711	2.275
ALAD	-0.496	0.306	-1.618	0.106	0.609	0.187	-2.095	0.036	0.334	1.11
AKTIP	0.125	0.296	0.424	0.672	1.133	0.335	0.398	0.691	0.635	2.023
AKT3	-0.359	0.298	-1.207	0.228	0.698	0.208	-1.452	0.147	0.39	1.251
AKT2	-0.229	0.296	-0.774	0.439	0.795	0.235	-0.869	0.385	0.445	1.421
AKT1	-0.189	0.296	-0.638	0.523	0.828	0.245	-0.703	0.482	0.464	1.479
AKR7A3	0.293	0.297	0.985	0.324	1.34	0.398	0.854	0.393	0.749	2.399
AKR7A2	0.08	0.297	0.27	0.787	1.083	0.321	0.26	0.795	0.606	1.937
AKR1D1	-0.089	0.295	-0.3	0.764	0.915	0.27	-0.314	0.754	0.513	1.633
AKR1C4	-0.019	0.295	-0.064	0.949	0.981	0.289	-0.065	0.948	0.55	1.749
AKR1C3	-0.318	0.298	-1.07	0.285	0.727	0.216	-1.26	0.208	0.406	1.303
AKR1C2	0.158	0.296	0.533	0.594	1.171	0.347	0.493	0.622	0.655	2.093
AKR1C1	0.471	0.302	1.557	0.119	1.601	0.484	1.242	0.214	0.885	2.895
AKR1B10	-0.287	0.298	-0.963	0.335	0.751	0.224	-1.115	0.265	0.419	1.346
AKR1B1	-0.484	0.3	-1.611	0.107	0.616	0.185	-2.072	0.038	0.342	1.11
AKR1A1	-0.436	0.307	-1.421	0.155	0.646	0.198	-1.782	0.075	0.354	1.18
AKIRIN2	0.074	0.295	0.252	0.801	1.077	0.318	0.242	0.808	0.604	1.922
AKIRIN1	0.039	0.295	0.133	0.894	1.04	0.307	0.13	0.896	0.583	1.854
AKIP1	0.121	0.295	0.41	0.682	1.129	0.333	0.386	0.7	0.633	2.014
AKAP9	-0.195	0.297	-0.657	0.511	0.823	0.244	-0.726	0.468	0.46	1.472
AKAP8L	0.224	0.297	0.756	0.45	1.251	0.371	0.677	0.498	0.7	2.237
AKAP8	0.346	0.3	1.155	0.248	1.414	0.424	0.976	0.329	0.786	2.544
AKAP7	-0.522	0.302	-1.727	0.084	0.593	0.179	-2.269	0.023	0.328	1.073
AKAP6	-0.007	0.296	-0.023	0.981	0.993	0.294	-0.023	0.981	0.556	1.774
AKAP5	0.114	0.296	0.383	0.702	1.12	0.332	0.362	0.717	0.627	2.002
AKAP4	0.934	0.328	2.85	0.004	2.544	0.833	1.852	0.064	1.338	4.834
AKAP3	-0.325	0.299	-1.087	0.277	0.723	0.216	-1.284	0.199	0.402	1.298
AKAP17A	0.12	0.299	0.4	0.689	1.127	0.337	0.377	0.706	0.627	2.025
AKAP13	-0.685	0.306	-2.239	0.025	0.504	0.154	-3.216	0.001	0.277	0.918
AKAP12	-0.551	0.301	-1.832	0.067	0.577	0.173	-2.443	0.015	0.32	1.039
AKAP11	-0.893	0.311	-2.872	0.004	0.409	0.127	-4.639	0	0.222	0.753
AKAP10	0.374	0.3	1.25	0.211	1.454	0.436	1.043	0.297	0.808	2.616
AKAP1	0.124	0.3	0.414	0.679	1.132	0.34	0.389	0.697	0.628	2.04
AK6	0.287	0.297	0.967	0.334	1.332	0.395	0.841	0.401	0.745	2.384

AK5	-0.887	0.321	-2.763	0.006	0.412	0.132	-4.448	0	0.219	0.773
AK4	0.167	0.296	0.565	0.572	1.182	0.35	0.521	0.603	0.662	2.112
AK2	-0.241	0.3	-0.803	0.422	0.786	0.236	-0.908	0.364	0.436	1.416
AK1	0.198	0.297	0.667	0.505	1.219	0.362	0.605	0.545	0.681	2.184
AJAP1	0.033	0.295	0.112	0.911	1.034	0.305	0.11	0.912	0.58	1.843
AIRE	-0.114	0.296	-0.387	0.699	0.892	0.264	-0.41	0.682	0.499	1.593
AIPL1	0.15	0.296	0.507	0.612	1.162	0.344	0.471	0.638	0.651	2.075
AIP	0.345	0.3	1.15	0.25	1.412	0.423	0.973	0.331	0.785	2.54
AIMP2	0.417	0.3	1.391	0.164	1.517	0.455	1.138	0.255	0.843	2.73
AIMP1	0.038	0.295	0.13	0.896	1.039	0.307	0.128	0.898	0.583	1.853
AIM2	-0.287	0.297	-0.964	0.335	0.751	0.223	-1.117	0.264	0.419	1.345
AIFM1	0.142	0.296	0.479	0.632	1.152	0.34	0.447	0.655	0.646	2.056
AIF1	-0.44	0.302	-1.454	0.146	0.644	0.195	-1.826	0.068	0.356	1.165
AIDA	-0.492	0.302	-1.626	0.104	0.612	0.185	-2.101	0.036	0.338	1.106
AICDA	-0.049	0.295	-0.165	0.869	0.952	0.281	-0.169	0.865	0.534	1.699
AHSP	0.43	0.3	1.434	0.151	1.538	0.461	1.166	0.244	0.854	2.768
AHSG	-0.661	0.306	-2.161	0.031	0.516	0.158	-3.064	0.002	0.283	0.94
AHSA1	0.579	0.306	1.894	0.058	1.784	0.545	1.438	0.15	0.98	3.247
AHR	-0.486	0.3	-1.619	0.105	0.615	0.185	-2.085	0.037	0.341	1.108
AHNAK2	-0.623	0.306	-2.039	0.041	0.536	0.164	-2.83	0.005	0.295	0.976
AHNAK	-0.374	0.3	-1.248	0.212	0.688	0.206	-1.514	0.13	0.382	1.238
AHI1	-0.164	0.296	-0.555	0.579	0.848	0.251	-0.604	0.546	0.475	1.515
AHDC1	-0.481	0.306	-1.573	0.116	0.618	0.189	-2.02	0.043	0.339	1.126
AHCYL2	-0.543	0.306	-1.777	0.076	0.581	0.178	-2.361	0.018	0.319	1.057
AHCYL1	0.191	0.296	0.645	0.519	1.211	0.359	0.587	0.557	0.677	2.164
AHCY	-0.278	0.297	-0.937	0.349	0.757	0.225	-1.08	0.28	0.423	1.355
AHCTF1	0.645	0.306	2.11	0.035	1.906	0.582	1.555	0.12	1.047	3.469
AGXT	-0.478	0.302	-1.584	0.113	0.62	0.187	-2.031	0.042	0.343	1.12
AGTR2	0.33	0.3	1.1	0.271	1.391	0.417	0.937	0.349	0.773	2.502
AGTR1	-0.282	0.3	-0.939	0.348	0.754	0.227	-1.085	0.278	0.419	1.359
AGTPBP1	0.768	0.31	2.478	0.013	2.155	0.668	1.73	0.084	1.174	3.956
AGT	-0.029	0.295	-0.099	0.921	0.971	0.287	-0.101	0.92	0.544	1.733
AGRP	-0.364	0.299	-1.218	0.223	0.695	0.208	-1.47	0.142	0.387	1.248
AGRN	-0.114	0.295	-0.385	0.7	0.893	0.264	-0.408	0.683	0.5	1.592
AGR2	0.287	0.297	0.968	0.333	1.333	0.395	0.841	0.4	0.745	2.384
AGPS	0.046	0.295	0.156	0.876	1.047	0.309	0.153	0.879	0.587	1.867
AGPAT5	-0.133	0.295	-0.449	0.653	0.876	0.259	-0.481	0.631	0.491	1.562
AGPAT4	0.22	0.296	0.744	0.457	1.247	0.369	0.668	0.504	0.697	2.228

AGPAT3	0.184	0.295	0.624	0.533	1.202	0.355	0.57	0.569	0.674	2.146
AGPAT2	0.035	0.298	0.119	0.905	1.036	0.308	0.117	0.907	0.578	1.857
AGPAT1	0.304	0.3	1.012	0.312	1.355	0.407	0.873	0.383	0.752	2.441
AGO4	0.264	0.297	0.888	0.374	1.302	0.387	0.781	0.435	0.727	2.331
AGO3	1.003	0.321	3.119	0.002	2.726	0.876	1.969	0.049	1.452	5.118
AGO1	-0.124	0.296	-0.418	0.676	0.884	0.261	-0.445	0.656	0.495	1.577
AGMAT	-0.195	0.298	-0.655	0.512	0.823	0.245	-0.723	0.469	0.459	1.475
AGL	-0.125	0.296	-0.424	0.671	0.882	0.261	-0.452	0.651	0.494	1.574
AGK	0.379	0.297	1.277	0.202	1.461	0.434	1.063	0.288	0.816	2.615
AGGF1	-0.4	0.303	-1.324	0.186	0.67	0.203	-1.628	0.104	0.37	1.212
AGFG2	-0.024	0.296	-0.081	0.935	0.976	0.289	-0.082	0.934	0.547	1.744
AGFG1	-0.11	0.296	-0.371	0.71	0.896	0.265	-0.393	0.695	0.502	1.6
AGER	0.542	0.302	1.793	0.073	1.72	0.52	1.384	0.166	0.951	3.11
AGBL5	0.143	0.295	0.485	0.627	1.154	0.341	0.452	0.651	0.647	2.059
AGBL3	0.175	0.296	0.59	0.555	1.191	0.353	0.541	0.588	0.666	2.128
AGBL2	0.288	0.298	0.965	0.334	1.333	0.397	0.839	0.402	0.743	2.391
AGAP2	0.509	0.302	1.685	0.092	1.664	0.503	1.32	0.187	0.92	3.009
AGAP1	-0.387	0.3	-1.292	0.197	0.679	0.203	-1.577	0.115	0.378	1.222
AGA	-0.327	0.3	-1.091	0.275	0.721	0.216	-1.291	0.197	0.401	1.297
AFTPH	0.017	0.295	0.059	0.953	1.018	0.3	0.059	0.953	0.571	1.814
AFP	0.004	0.296	0.012	0.99	1.004	0.297	0.012	0.99	0.562	1.792
AFM	0.012	0.296	0.04	0.968	1.012	0.299	0.039	0.969	0.567	1.807
AFG3L2	-0.158	0.297	-0.531	0.595	0.854	0.254	-0.576	0.565	0.477	1.528
AFF4	0.439	0.3	1.464	0.143	1.551	0.465	1.185	0.236	0.862	2.789
AFF3	-0.394	0.307	-1.284	0.199	0.674	0.207	-1.574	0.116	0.369	1.231
AFF2	-0.162	0.296	-0.548	0.584	0.85	0.252	-0.595	0.552	0.476	1.519
AFF1	-0.103	0.295	-0.349	0.727	0.902	0.266	-0.368	0.713	0.506	1.609
AFDN-DT	-0.378	0.298	-1.27	0.204	0.685	0.204	-1.543	0.123	0.382	1.228
AFDN	0.332	0.299	1.112	0.266	1.394	0.416	0.946	0.344	0.776	2.503
AFAP1	0.336	0.299	1.125	0.261	1.399	0.418	0.955	0.339	0.779	2.512
AEN	-0.262	0.3	-0.873	0.382	0.77	0.231	-0.998	0.318	0.428	1.385
AEBP1	-0.764	0.31	-2.467	0.014	0.466	0.144	-3.703	0	0.254	0.855
ADTRP	-0.121	0.295	-0.411	0.681	0.886	0.262	-0.437	0.662	0.496	1.58
ADSS2	-0.068	0.295	-0.229	0.819	0.935	0.276	-0.236	0.813	0.524	1.668
ADSL	0.579	0.306	1.893	0.058	1.784	0.546	1.437	0.151	0.98	3.249
ADRM1	0.543	0.302	1.796	0.072	1.721	0.52	1.386	0.166	0.952	3.112
ADRB3	0.587	0.311	1.888	0.059	1.798	0.559	1.428	0.153	0.978	3.306
ADRB2	-0.801	0.31	-2.582	0.01	0.449	0.139	-3.957	0	0.245	0.825

ADRB1	0.455	0.3	1.518	0.129	1.576	0.473	1.22	0.223	0.876	2.837
ADRA2C	-0.32	0.298	-1.077	0.282	0.726	0.216	-1.269	0.204	0.405	1.301
ADRA2B	-0.208	0.297	-0.7	0.484	0.812	0.241	-0.778	0.436	0.454	1.453
ADRA2A	-1.129	0.336	-3.359	0.001	0.323	0.109	-6.225	0	0.167	0.625
ADRA1D	-0.362	0.298	-1.217	0.224	0.696	0.207	-1.466	0.143	0.388	1.248
ADRA1B	0.027	0.296	0.092	0.927	1.028	0.304	0.091	0.928	0.576	1.834
ADRA1A	-0.039	0.295	-0.133	0.894	0.961	0.284	-0.136	0.892	0.539	1.715
ADPRM	-0.229	0.298	-0.77	0.441	0.795	0.237	-0.865	0.387	0.443	1.426
ADPRH	-0.452	0.3	-1.507	0.132	0.636	0.191	-1.906	0.057	0.353	1.146
ADPGK	-0.289	0.298	-0.971	0.332	0.749	0.223	-1.125	0.26	0.418	1.342
ADORA3	-0.332	0.3	-1.107	0.268	0.718	0.215	-1.313	0.189	0.399	1.291
ADORA2B	0.247	0.296	0.832	0.405	1.28	0.379	0.737	0.461	0.716	2.287
ADORA1	0.053	0.296	0.18	0.858	1.054	0.312	0.175	0.861	0.591	1.882
ADO	0.104	0.297	0.35	0.726	1.11	0.33	0.333	0.739	0.62	1.986
ADNP2	-0.3	0.3	-0.999	0.318	0.741	0.222	-1.165	0.244	0.412	1.334
ADNP	0.165	0.296	0.557	0.578	1.179	0.349	0.513	0.608	0.66	2.108
ADM2	0.145	0.295	0.491	0.624	1.156	0.341	0.457	0.648	0.648	2.062
ADM	0.958	0.321	2.987	0.003	2.606	0.836	1.922	0.055	1.39	4.886
ADK	0.392	0.298	1.316	0.188	1.479	0.44	1.089	0.276	0.826	2.651
ADIRF	-0.077	0.296	-0.262	0.793	0.926	0.274	-0.272	0.785	0.519	1.652
ADIPOR2	-0.213	0.298	-0.714	0.475	0.808	0.241	-0.796	0.426	0.451	1.449
ADIPOR1	-0.098	0.296	-0.331	0.741	0.907	0.268	-0.348	0.728	0.508	1.619
ADIPOQ	-0.227	0.296	-0.767	0.443	0.797	0.236	-0.861	0.389	0.446	1.424
ADI1	0.223	0.298	0.748	0.454	1.249	0.372	0.671	0.503	0.697	2.239
ADH7	0.342	0.298	1.149	0.251	1.407	0.419	0.973	0.331	0.786	2.522
ADH6	0.206	0.296	0.696	0.486	1.229	0.364	0.629	0.529	0.688	2.196
ADH5	0.957	0.321	2.984	0.003	2.605	0.836	1.92	0.055	1.389	4.886
ADH1C	0.043	0.295	0.144	0.885	1.043	0.308	0.141	0.888	0.585	1.861
ADH1B	-0.159	0.295	-0.537	0.591	0.853	0.252	-0.582	0.561	0.478	1.523
ADH1A	0.198	0.296	0.667	0.505	1.219	0.361	0.606	0.545	0.682	2.179
ADGRV1	0.053	0.295	0.18	0.857	1.055	0.312	0.175	0.861	0.591	1.882
ADGRL4	-0.214	0.296	-0.723	0.47	0.807	0.239	-0.806	0.42	0.452	1.443
ADGRL3	0.29	0.296	0.978	0.328	1.336	0.396	0.849	0.396	0.748	2.387
ADGRL2	-0.407	0.298	-1.368	0.171	0.665	0.198	-1.689	0.091	0.371	1.193
ADGRL1	0.397	0.3	1.324	0.185	1.487	0.446	1.093	0.274	0.827	2.677
ADGRG6	-0.191	0.298	-0.64	0.522	0.826	0.246	-0.705	0.481	0.461	1.481
ADGRG3	-0.196	0.297	-0.662	0.508	0.822	0.244	-0.732	0.464	0.459	1.469
ADGRG2	0.364	0.303	1.202	0.229	1.439	0.435	1.008	0.314	0.795	2.603

ADGRG1	0.511	0.302	1.691	0.091	1.667	0.504	1.324	0.185	0.922	3.014
ADGRF5	-0.314	0.297	-1.056	0.291	0.731	0.217	-1.24	0.215	0.408	1.308
ADGRF1	0.648	0.306	2.118	0.034	1.912	0.585	1.559	0.119	1.05	3.483
ADGRE5	0.721	0.31	2.327	0.02	2.056	0.637	1.658	0.097	1.12	3.773
ADGRE3	0.03	0.295	0.1	0.92	1.03	0.304	0.099	0.921	0.577	1.837
ADGRE2	-0.278	0.298	-0.935	0.35	0.757	0.225	-1.078	0.281	0.422	1.357
ADGRE1	-0.205	0.296	-0.692	0.489	0.814	0.241	-0.769	0.442	0.456	1.456
ADGRD2	-0.022	0.295	-0.076	0.94	0.978	0.289	-0.076	0.939	0.548	1.744
ADGRB3	0.455	0.3	1.519	0.129	1.577	0.473	1.22	0.222	0.876	2.838
ADGRB2	-0.064	0.296	-0.218	0.828	0.938	0.278	-0.225	0.822	0.525	1.675
ADGRB1	0.369	0.297	1.242	0.214	1.447	0.43	1.039	0.299	0.808	2.592
ADGRA3	0.83	0.315	2.637	0.008	2.293	0.722	1.792	0.073	1.238	4.249
ADGRA2	-1.166	0.329	-3.548	0	0.312	0.102	-6.721	0	0.164	0.593
ADGB	0.089	0.295	0.302	0.763	1.093	0.323	0.289	0.773	0.613	1.951
ADD3-AS1	0.532	0.302	1.758	0.079	1.702	0.515	1.364	0.173	0.941	3.079
ADD3	-0.199	0.295	-0.674	0.501	0.82	0.242	-0.745	0.456	0.459	1.462
ADD2	-0.369	0.299	-1.238	0.216	0.691	0.206	-1.497	0.134	0.385	1.241
ADD1	-0.467	0.302	-1.545	0.122	0.627	0.189	-1.969	0.049	0.347	1.134
ADCYAP1R1	0.005	0.295	0.016	0.987	1.005	0.296	0.016	0.987	0.563	1.791
ADCYAP1	0.262	0.299	0.878	0.38	1.3	0.388	0.772	0.44	0.724	2.334
ADCY9	-0.01	0.296	-0.034	0.973	0.99	0.293	-0.034	0.973	0.554	1.768
ADCY8	0.296	0.296	0.999	0.318	1.344	0.398	0.864	0.387	0.752	2.403
ADCY7	0.151	0.296	0.51	0.61	1.163	0.345	0.474	0.636	0.651	2.08
ADCY6	-0.503	0.302	-1.664	0.096	0.605	0.183	-2.162	0.031	0.335	1.094
ADCY3	-0.732	0.311	-2.358	0.018	0.481	0.149	-3.477	0.001	0.262	0.884
ADCY2	0.079	0.295	0.268	0.789	1.082	0.32	0.258	0.797	0.607	1.931
ADCY10	-0.012	0.295	-0.04	0.968	0.988	0.292	-0.04	0.968	0.554	1.763
ADCY1	0.34	0.298	1.143	0.253	1.405	0.418	0.969	0.333	0.784	2.517
ADCK2	0.553	0.3	1.839	0.066	1.738	0.522	1.413	0.158	0.964	3.131
ADAT1	-0.16	0.297	-0.54	0.589	0.852	0.253	-0.586	0.558	0.476	1.524
ADARB2	-0.474	0.3	-1.579	0.114	0.623	0.187	-2.02	0.043	0.346	1.121
ADARB1	-0.33	0.298	-1.109	0.268	0.719	0.214	-1.313	0.189	0.401	1.288
ADAR	-0.034	0.296	-0.115	0.908	0.966	0.286	-0.117	0.907	0.541	1.726
ADAP2	0.418	0.3	1.397	0.163	1.519	0.455	1.141	0.254	0.845	2.733
ADAP1	0.033	0.295	0.113	0.91	1.034	0.305	0.111	0.912	0.58	1.844
ADAMTSL4	-0.081	0.295	-0.274	0.784	0.922	0.272	-0.286	0.775	0.517	1.645
ADAMTSL3	-0.177	0.296	-0.597	0.55	0.838	0.248	-0.653	0.513	0.469	1.497
ADAMTSL2	0.472	0.301	1.566	0.117	1.603	0.483	1.249	0.212	0.888	2.892

ADAMTS9	-0.5	0.302	-1.658	0.097	0.606	0.183	-2.152	0.031	0.336	1.095
ADAMTS8	0.113	0.295	0.381	0.703	1.119	0.33	0.36	0.719	0.627	1.996
ADAMTS7	0.428	0.3	1.43	0.153	1.535	0.46	1.163	0.245	0.853	2.761
ADAMTS6	0.211	0.298	0.707	0.48	1.235	0.368	0.637	0.524	0.688	2.215
ADAMTS5	-0.116	0.296	-0.391	0.696	0.891	0.264	-0.414	0.679	0.498	1.592
ADAMTS3	-0.381	0.3	-1.273	0.203	0.683	0.205	-1.55	0.121	0.38	1.228
ADAMTS20	0.313	0.298	1.053	0.292	1.368	0.407	0.904	0.366	0.764	2.451
ADAMTS2	-0.305	0.296	-1.029	0.304	0.737	0.218	-1.203	0.229	0.412	1.318
ADAMTS13	0.021	0.296	0.072	0.943	1.021	0.303	0.071	0.943	0.572	1.825
ADAMTS12	0.021	0.296	0.07	0.945	1.021	0.302	0.069	0.945	0.572	1.823
ADAMTS1	0.429	0.3	1.43	0.153	1.535	0.46	1.163	0.245	0.853	2.763
ADAMDEC1	0.043	0.295	0.147	0.883	1.044	0.308	0.144	0.886	0.586	1.862
ADAM9	-0.014	0.295	-0.046	0.963	0.987	0.291	-0.046	0.963	0.553	1.76
ADAM8	0.44	0.299	1.474	0.141	1.553	0.464	1.192	0.233	0.865	2.791
ADAM7	0.248	0.296	0.836	0.403	1.281	0.38	0.74	0.459	0.717	2.29
ADAM5	-0.412	0.299	-1.377	0.169	0.662	0.198	-1.703	0.088	0.369	1.191
ADAM3A	0.251	0.298	0.843	0.399	1.285	0.383	0.746	0.456	0.717	2.304
ADAM30	-0.105	0.296	-0.354	0.724	0.901	0.266	-0.373	0.709	0.505	1.608
ADAM28	-0.672	0.31	-2.169	0.03	0.511	0.158	-3.092	0.002	0.278	0.937
ADAM23	-0.034	0.295	-0.116	0.908	0.966	0.285	-0.118	0.906	0.542	1.724
ADAM22	0.126	0.296	0.426	0.67	1.135	0.336	0.4	0.689	0.635	2.029
ADAM21	0.001	0.296	0.002	0.998	1.001	0.296	0.002	0.998	0.56	1.788
ADAM20	-0.512	0.3	-1.706	0.088	0.599	0.18	-2.228	0.026	0.333	1.079
ADAM2	0.628	0.303	2.072	0.038	1.874	0.568	1.538	0.124	1.034	3.394
ADAM19	-0.467	0.3	-1.555	0.12	0.627	0.188	-1.982	0.047	0.348	1.129
ADAM18	0.168	0.296	0.569	0.57	1.183	0.35	0.523	0.601	0.662	2.114
ADAM17	-0.553	0.3	-1.841	0.066	0.575	0.173	-2.459	0.014	0.319	1.036
ADAM15	-0.502	0.306	-1.639	0.101	0.605	0.185	-2.128	0.033	0.332	1.103
ADAM12	-0.714	0.306	-2.335	0.02	0.489	0.15	-3.409	0.001	0.269	0.892
ADAM11	0.142	0.296	0.478	0.633	1.152	0.341	0.445	0.656	0.644	2.059
ADAM10	0.005	0.295	0.017	0.986	1.005	0.297	0.017	0.986	0.564	1.792
ADA2	-0.611	0.303	-2.014	0.044	0.543	0.165	-2.775	0.006	0.3	0.984
ADA	0.268	0.298	0.901	0.368	1.308	0.389	0.79	0.43	0.729	2.344
ACYP2	-0.45	0.302	-1.486	0.137	0.638	0.193	-1.877	0.061	0.353	1.154
ACYP1	0.278	0.296	0.938	0.348	1.32	0.391	0.819	0.413	0.739	2.358
ACVRL1	-0.483	0.3	-1.612	0.107	0.617	0.185	-2.072	0.038	0.343	1.11
ACVR2B-AS1	-0.42	0.298	-1.41	0.159	0.657	0.196	-1.753	0.08	0.366	1.178
ACVR2B	0.639	0.31	2.064	0.039	1.895	0.587	1.525	0.127	1.033	3.477

ACVR2A	-0.159	0.296	-0.537	0.591	0.853	0.252	-0.582	0.561	0.478	1.524
ACVR1B	-0.4	0.3	-1.336	0.181	0.67	0.201	-1.644	0.1	0.372	1.205
ACVR1	-0.523	0.301	-1.737	0.082	0.593	0.178	-2.282	0.023	0.329	1.069
ACTR8	0.122	0.297	0.41	0.682	1.129	0.335	0.386	0.699	0.631	2.02
ACTR6	0.082	0.296	0.279	0.781	1.086	0.321	0.267	0.789	0.608	1.938
ACTR5	0.394	0.3	1.316	0.188	1.484	0.445	1.088	0.277	0.825	2.669
ACTR3B	0.455	0.303	1.503	0.133	1.575	0.477	1.208	0.227	0.871	2.85
ACTR3	0.065	0.298	0.218	0.828	1.067	0.318	0.211	0.833	0.595	1.913
ACTR2	-0.092	0.295	-0.31	0.757	0.913	0.269	-0.325	0.746	0.512	1.628
ACTR1B	-0.652	0.306	-2.133	0.033	0.521	0.159	-3.008	0.003	0.286	0.948
ACTR1A	0.088	0.295	0.297	0.767	1.091	0.322	0.284	0.776	0.612	1.946
ACTR10	-0.033	0.297	-0.111	0.912	0.968	0.288	-0.113	0.91	0.54	1.733
ACTN4	-0.128	0.295	-0.432	0.666	0.88	0.26	-0.46	0.645	0.493	1.571
ACTN3	0.342	0.3	1.139	0.255	1.407	0.422	0.965	0.335	0.782	2.535
ACTN2	0.287	0.298	0.963	0.335	1.332	0.396	0.838	0.402	0.743	2.387
ACTN1	0.005	0.295	0.017	0.986	1.005	0.297	0.017	0.986	0.563	1.793
ACTL8	0.035	0.296	0.119	0.905	1.036	0.306	0.117	0.907	0.58	1.849
ACTL7B	0.352	0.3	1.173	0.241	1.422	0.426	0.989	0.323	0.79	2.559
ACTL7A	0.009	0.297	0.032	0.975	1.009	0.299	0.032	0.975	0.565	1.805
ACTL6B	0.607	0.304	1.997	0.046	1.835	0.558	1.497	0.134	1.011	3.329
ACTL6A	1.021	0.328	3.114	0.002	2.775	0.91	1.952	0.051	1.46	5.275
ACTG2	-0.506	0.302	-1.676	0.094	0.603	0.182	-2.182	0.029	0.333	1.09
ACTC1	-0.224	0.296	-0.756	0.45	0.8	0.237	-0.847	0.397	0.447	1.429
ACTB	0.012	0.296	0.041	0.967	1.012	0.299	0.041	0.968	0.567	1.807
ACTA2	-0.481	0.3	-1.602	0.109	0.618	0.185	-2.057	0.04	0.344	1.113
ACTA1	0.292	0.298	0.982	0.326	1.34	0.399	0.852	0.394	0.748	2.4
ACSS3	-0.103	0.295	-0.348	0.728	0.902	0.266	-0.366	0.714	0.506	1.61
ACSM5	-0.064	0.295	-0.216	0.829	0.938	0.277	-0.223	0.823	0.526	1.673
ACSM3	0.22	0.296	0.742	0.458	1.246	0.369	0.666	0.505	0.697	2.227
ACSM1	-0.042	0.296	-0.143	0.886	0.958	0.284	-0.146	0.884	0.536	1.713
ACSL6	-0.128	0.296	-0.431	0.667	0.88	0.261	-0.459	0.646	0.493	1.573
ACSL5	-0.397	0.298	-1.331	0.183	0.673	0.2	-1.634	0.102	0.375	1.206
ACSL4	-0.07	0.295	-0.238	0.812	0.932	0.275	-0.246	0.805	0.523	1.663
ACSL3	0.04	0.296	0.134	0.893	1.041	0.308	0.132	0.895	0.583	1.857
ACSL1	0.245	0.298	0.822	0.411	1.277	0.38	0.729	0.466	0.713	2.289
ACSF2	-0.281	0.298	-0.944	0.345	0.755	0.225	-1.089	0.276	0.421	1.353
ACSBG2	-0.451	0.3	-1.503	0.133	0.637	0.191	-1.899	0.058	0.354	1.147
ACSBG1	-0.09	0.296	-0.305	0.76	0.914	0.27	-0.319	0.75	0.512	1.631

ACRV1	-0.14	0.296	-0.472	0.637	0.87	0.257	-0.506	0.613	0.487	1.553
ACR	-0.333	0.298	-1.121	0.262	0.716	0.213	-1.33	0.183	0.4	1.284
ACP6	0.337	0.297	1.133	0.257	1.4	0.416	0.962	0.336	0.782	2.508
ACP5	0.062	0.295	0.211	0.833	1.064	0.314	0.204	0.838	0.596	1.899
ACP3	-0.122	0.296	-0.414	0.679	0.885	0.262	-0.44	0.66	0.496	1.579
ACP2	0.303	0.298	1.016	0.31	1.354	0.404	0.877	0.381	0.755	2.43
ACP1	-0.343	0.298	-1.152	0.249	0.71	0.211	-1.374	0.17	0.396	1.272
ACOXL	-0.042	0.296	-0.142	0.887	0.959	0.283	-0.145	0.884	0.537	1.711
ACOX3	-0.42	0.3	-1.401	0.161	0.657	0.197	-1.741	0.082	0.365	1.183
ACOX2	0.379	0.299	1.268	0.205	1.461	0.437	1.055	0.291	0.813	2.626
ACOX1	0.72	0.31	2.325	0.02	2.055	0.637	1.657	0.097	1.12	3.772
ACOT9	0.334	0.3	1.114	0.265	1.396	0.418	0.947	0.343	0.776	2.513
ACOT8	0.641	0.307	2.092	0.036	1.899	0.582	1.544	0.123	1.041	3.464
ACOT7	0.284	0.296	0.962	0.336	1.329	0.393	0.838	0.402	0.745	2.372
ACOT13	0.852	0.308	2.769	0.006	2.344	0.722	1.863	0.062	1.283	4.286
ACOT11	0.17	0.296	0.575	0.565	1.186	0.351	0.529	0.597	0.664	2.119
ACO2	-0.08	0.295	-0.271	0.786	0.923	0.273	-0.282	0.778	0.517	1.647
ACO1	0.19	0.295	0.642	0.521	1.209	0.357	0.585	0.559	0.678	2.157
ACLY	0.666	0.31	2.15	0.032	1.947	0.603	1.57	0.116	1.061	3.573
ACKR4	-0.516	0.302	-1.706	0.088	0.597	0.181	-2.233	0.026	0.33	1.08
ACKR3	0.256	0.298	0.86	0.39	1.292	0.384	0.758	0.448	0.721	2.315
ACKR2	-0.394	0.3	-1.314	0.189	0.674	0.202	-1.61	0.107	0.374	1.214
ACKR1	-0.46	0.3	-1.534	0.125	0.632	0.189	-1.947	0.052	0.351	1.136
ACIN1	-0.392	0.3	-1.307	0.191	0.676	0.203	-1.601	0.109	0.376	1.216
ACHE	0.251	0.301	0.833	0.405	1.285	0.387	0.736	0.461	0.712	2.318
ACE2	-0.147	0.298	-0.494	0.621	0.863	0.257	-0.532	0.595	0.482	1.547
ACE	0.147	0.295	0.498	0.618	1.159	0.342	0.463	0.643	0.649	2.067
ACD	0.314	0.297	1.059	0.29	1.369	0.406	0.909	0.364	0.765	2.448
ACBD4	0.106	0.296	0.358	0.72	1.112	0.329	0.34	0.734	0.623	1.985
ACBD3	0.054	0.296	0.182	0.856	1.055	0.312	0.177	0.86	0.591	1.883
ACAT2	0.022	0.295	0.074	0.941	1.022	0.302	0.073	0.942	0.573	1.824
ACAT1	0.009	0.295	0.032	0.974	1.009	0.298	0.032	0.975	0.566	1.8
ACAP2	0.041	0.295	0.14	0.888	1.042	0.308	0.137	0.891	0.584	1.859
ACAP1	-0.195	0.295	-0.659	0.51	0.823	0.243	-0.728	0.467	0.461	1.469
ACAN	-0.466	0.3	-1.554	0.12	0.627	0.188	-1.98	0.048	0.348	1.13
ACADVL	-0.445	0.3	-1.482	0.138	0.641	0.192	-1.866	0.062	0.356	1.154
ACADSB	-0.205	0.298	-0.689	0.491	0.814	0.243	-0.765	0.445	0.454	1.461
ACADS	-0.233	0.298	-0.784	0.433	0.792	0.236	-0.883	0.377	0.442	1.419

ACADM	-0.171	0.297	-0.576	0.565	0.843	0.25	-0.628	0.53	0.471	1.509
ACADL	-0.267	0.296	-0.9	0.368	0.766	0.227	-1.032	0.302	0.428	1.369
ACAD8	-0.799	0.311	-2.573	0.01	0.45	0.14	-3.941	0	0.245	0.826
ACAD10	0.268	0.297	0.901	0.367	1.307	0.388	0.791	0.429	0.73	2.34
ACACB	-0.493	0.303	-1.629	0.103	0.611	0.185	-2.105	0.035	0.337	1.106
ACACA	0.245	0.299	0.82	0.412	1.277	0.381	0.727	0.467	0.712	2.293
ACAA2	0.252	0.297	0.846	0.397	1.286	0.382	0.748	0.454	0.718	2.304
ACAA1	-0.458	0.303	-1.515	0.13	0.632	0.191	-1.922	0.055	0.349	1.144
ABTB2	0.392	0.3	1.306	0.192	1.479	0.444	1.081	0.28	0.822	2.663
ABT1	-0.316	0.298	-1.059	0.289	0.729	0.217	-1.246	0.213	0.407	1.308
ABRAXAS2	0.088	0.296	0.297	0.767	1.092	0.323	0.284	0.776	0.612	1.948
ABR	-0.635	0.306	-2.078	0.038	0.53	0.162	-2.903	0.004	0.291	0.964
ABO	0.22	0.296	0.742	0.458	1.246	0.369	0.666	0.505	0.697	2.228
ABLIM3	-0.184	0.298	-0.619	0.536	0.832	0.248	-0.68	0.497	0.464	1.491
ABLIM1	-0.487	0.302	-1.611	0.107	0.615	0.186	-2.075	0.038	0.34	1.111
ABL2	0.084	0.295	0.283	0.777	1.087	0.321	0.272	0.786	0.609	1.94
ABL1	-0.276	0.296	-0.932	0.351	0.759	0.225	-1.073	0.283	0.424	1.356
ABITRAM	0.329	0.298	1.104	0.269	1.389	0.413	0.941	0.347	0.775	2.489
ABI3BP	-0.043	0.297	-0.145	0.884	0.958	0.285	-0.149	0.882	0.535	1.715
ABI2	-0.26	0.298	-0.874	0.382	0.771	0.229	-0.999	0.318	0.43	1.381
ABI1	0.185	0.296	0.623	0.534	1.203	0.356	0.568	0.57	0.673	2.15
ABHD8	0.354	0.298	1.189	0.234	1.425	0.425	1.001	0.317	0.795	2.557
ABHD6	0.332	0.298	1.116	0.264	1.394	0.415	0.95	0.342	0.778	2.498
ABHD5	0.675	0.303	2.225	0.026	1.963	0.595	1.618	0.106	1.084	3.557
ABHD4	-0.38	0.3	-1.269	0.204	0.684	0.205	-1.544	0.123	0.38	1.23
ABHD3	-0.399	0.3	-1.332	0.183	0.671	0.201	-1.637	0.102	0.373	1.207
ABHD2	-0.187	0.296	-0.632	0.527	0.829	0.246	-0.695	0.487	0.464	1.482
ABHD18	-0.01	0.295	-0.035	0.972	0.99	0.292	-0.036	0.972	0.555	1.764
ABHD17B	-0.435	0.3	-1.448	0.148	0.647	0.194	-1.813	0.07	0.359	1.166
ABHD17A	-0.002	0.298	-0.008	0.994	0.998	0.297	-0.008	0.994	0.556	1.788
ABHD11	0.325	0.298	1.091	0.275	1.384	0.412	0.932	0.351	0.772	2.479
ABHD10	-0.613	0.303	-2.025	0.043	0.541	0.164	-2.795	0.005	0.299	0.981
ABCG5	0.101	0.296	0.342	0.733	1.106	0.328	0.325	0.745	0.619	1.977
ABCG4	0.423	0.3	1.414	0.158	1.527	0.458	1.152	0.249	0.849	2.748
ABCG2	-0.172	0.296	-0.582	0.561	0.842	0.249	-0.635	0.525	0.471	1.504
ABCG1	-0.552	0.306	-1.804	0.071	0.576	0.176	-2.407	0.016	0.316	1.049
ABCF3	0.355	0.3	1.185	0.236	1.426	0.427	0.998	0.318	0.793	2.566
ABCF2	-0.351	0.3	-1.172	0.241	0.704	0.211	-1.404	0.16	0.391	1.267

ABCF1	0.379	0.303	1.25	0.211	1.461	0.443	1.04	0.298	0.806	2.646
ABCE1	0.11	0.296	0.37	0.711	1.116	0.33	0.351	0.726	0.625	1.993
ABCD4	-0.468	0.3	-1.56	0.119	0.626	0.188	-1.989	0.047	0.348	1.128
ABCD3	0.053	0.297	0.177	0.86	1.054	0.313	0.172	0.863	0.589	1.887
ABCD2	-0.158	0.296	-0.533	0.594	0.854	0.253	-0.577	0.564	0.478	1.526
ABCD1	0.307	0.298	1.031	0.303	1.359	0.405	0.888	0.375	0.758	2.436
ABCC9	-0.538	0.306	-1.761	0.078	0.584	0.178	-2.333	0.02	0.321	1.063
ABCC8	-0.05	0.296	-0.169	0.865	0.951	0.281	-0.174	0.862	0.533	1.699
ABCC6	-0.205	0.296	-0.693	0.488	0.814	0.241	-0.769	0.442	0.456	1.456
ABCC5	0.892	0.321	2.781	0.005	2.439	0.782	1.84	0.066	1.301	4.571
ABCC4	-0.025	0.296	-0.084	0.933	0.976	0.288	-0.085	0.932	0.547	1.741
ABCC3	0.118	0.296	0.399	0.69	1.126	0.334	0.376	0.707	0.629	2.012
ABCC2	0.017	0.295	0.057	0.954	1.017	0.3	0.057	0.955	0.57	1.815
ABCC10	0.437	0.303	1.443	0.149	1.548	0.469	1.169	0.242	0.855	2.803
ABCC1	0.746	0.315	2.369	0.018	2.109	0.664	1.669	0.095	1.138	3.911
ABCB9	0.385	0.298	1.289	0.197	1.469	0.438	1.07	0.285	0.819	2.637
ABCB8	-0.707	0.315	-2.245	0.025	0.493	0.155	-3.264	0.001	0.266	0.914
ABCB7	0.345	0.299	1.155	0.248	1.412	0.422	0.976	0.329	0.786	2.537
ABCB6	0.408	0.3	1.36	0.174	1.503	0.45	1.117	0.264	0.835	2.705
ABCB4	-0.21	0.296	-0.709	0.478	0.811	0.24	-0.789	0.43	0.454	1.448
ABCB11	-0.672	0.311	-2.163	0.031	0.511	0.159	-3.084	0.002	0.278	0.939
ABCB1	-0.752	0.306	-2.453	0.014	0.472	0.145	-3.656	0	0.259	0.86
ABCA8	-0.601	0.302	-1.987	0.047	0.548	0.166	-2.724	0.006	0.303	0.992
ABCA7	-0.014	0.295	-0.047	0.962	0.986	0.291	-0.048	0.962	0.553	1.758
ABCA6	-0.416	0.298	-1.394	0.163	0.66	0.197	-1.729	0.084	0.368	1.184
ABCA5	-0.021	0.296	-0.072	0.942	0.979	0.289	-0.073	0.942	0.548	1.748
ABCA4	0.767	0.31	2.476	0.013	2.153	0.667	1.729	0.084	1.173	3.951
ABCA3	-0.034	0.297	-0.114	0.91	0.967	0.287	-0.116	0.908	0.54	1.73
ABCA2	-0.041	0.295	-0.141	0.888	0.959	0.283	-0.143	0.886	0.538	1.711
ABCA12	-0.323	0.299	-1.079	0.281	0.724	0.217	-1.273	0.203	0.403	1.301
ABCA11P	-0.44	0.3	-1.468	0.142	0.644	0.193	-1.844	0.065	0.358	1.159
ABCA1	-0.547	0.301	-1.814	0.07	0.579	0.174	-2.414	0.016	0.321	1.045
ABAT	-0.176	0.297	-0.595	0.552	0.838	0.249	-0.65	0.516	0.469	1.499
AATK	0.101	0.295	0.342	0.732	1.106	0.327	0.326	0.745	0.62	1.974
AATF	0.567	0.303	1.873	0.061	1.764	0.535	1.429	0.153	0.974	3.195
AASS	-0.305	0.298	-1.025	0.305	0.737	0.219	-1.198	0.231	0.411	1.321
AASDHPPT	-0.159	0.296	-0.537	0.591	0.853	0.253	-0.582	0.56	0.477	1.524
AARS1	-0.009	0.297	-0.03	0.976	0.991	0.295	-0.03	0.976	0.553	1.775

AAR2	0.212	0.298	0.711	0.477	1.236	0.369	0.641	0.522	0.689	2.218
AANAT	0.074	0.296	0.251	0.802	1.077	0.318	0.242	0.809	0.603	1.922
AAMP	-0.199	0.296	-0.671	0.502	0.82	0.243	-0.742	0.458	0.459	1.465
AAMDC	0.778	0.307	2.534	0.011	2.176	0.668	1.762	0.078	1.193	3.971
AAK1	0.32	0.3	1.066	0.286	1.377	0.413	0.912	0.362	0.765	2.479
AAGAB	0.101	0.296	0.34	0.734	1.106	0.328	0.323	0.747	0.619	1.977
AADAC	0.029	0.295	0.1	0.92	1.03	0.304	0.098	0.922	0.578	1.836
AACS	-0.358	0.3	-1.196	0.232	0.699	0.209	-1.438	0.15	0.388	1.257
AAAS	-0.064	0.297	-0.214	0.83	0.938	0.279	-0.221	0.825	0.524	1.679
A4GNT	0.204	0.296	0.687	0.492	1.226	0.363	0.622	0.534	0.686	2.191
A4GALT	0.451	0.3	1.504	0.133	1.57	0.471	1.21	0.226	0.872	2.827
A2M	-0.484	0.3	-1.613	0.107	0.616	0.185	-2.074	0.038	0.342	1.11
A1CF	0.114	0.297	0.383	0.701	1.121	0.333	0.362	0.717	0.626	2.005

Supplementary Table 2. Gene Ontology Pathway Enrichment Analysis of all metastases-related genes									
ONTOLOG	ID	Description	GeneRatio	BgRatio	pvalue	p.adjust	qvalue	geneID	Count
BP	GO:0044839	cell cycle G2/M phase transition	47/808	273/18866	2.54E-16	1.28E-12	1.13E-12	7480/1008	47
BP	GO:0140014	mitotic nuclear division	47/808	286/18866	1.63E-15	3.09E-12	2.73E-12	11130/110	47
BP	GO:0000086	G2/M transition of mitotic cell cycle	44/808	254/18866	1.84E-15	3.09E-12	2.73E-12	7480/1008	44
BP	GO:1901990	regulation of mitotic cell cycle phase transit	59/808	448/18866	1.30E-14	1.62E-11	1.43E-11	7480/1106	59
BP	GO:0048285	organelle fission	61/808	476/18866	1.61E-14	1.62E-11	1.43E-11	11130/755	61
BP	GO:1902749	regulation of cell cycle G2/M phase transiti	39/808	217/18866	2.16E-14	1.65E-11	1.46E-11	7480/2297	39
BP	GO:0000280	nuclear division	57/808	428/18866	2.29E-14	1.65E-11	1.46E-11	11130/755	57
BP	GO:0000070	mitotic sister chromatid segregation	33/808	161/18866	4.57E-14	2.88E-11	2.54E-11	11130/110	33
BP	GO:1901987	regulation of cell cycle phase transition	60/808	486/18866	1.41E-13	7.92E-11	7.00E-11	7480/1106	60
BP	GO:0010389	regulation of G2/M transition of mitotic cel	36/808	200/18866	2.09E-13	1.05E-10	9.29E-11	7480/2297	36
BP	GO:0007088	regulation of mitotic nuclear division	32/808	163/18866	3.89E-13	1.68E-10	1.48E-10	11065/931	32
BP	GO:0031145	anaphase-promoting complex-dependent	23/808	83/18866	3.99E-13	1.68E-10	1.48E-10	11065/731	23
BP	GO:0051783	regulation of nuclear division	34/808	188/18866	8.49E-13	3.29E-10	2.91E-10	7552/1106	34
BP	GO:0007059	chromosome segregation	46/808	334/18866	2.39E-12	8.58E-10	7.58E-10	11130/110	46
BP	GO:0051983	regulation of chromosome segregation	25/808	107/18866	2.55E-12	8.58E-10	7.58E-10	11065/931	25
BP	GO:0000819	sister chromatid segregation	34/808	196/18866	2.90E-12	9.13E-10	8.07E-10	11130/110	34
BP	GO:0010965	regulation of mitotic sister chromatid separ	19/808	61/18866	4.44E-12	1.32E-09	1.16E-09	11065/931	19
BP	GO:0045930	negative regulation of mitotic cell cycle	46/808	341/18866	4.99E-12	1.40E-09	1.24E-09	11130/931	46
BP	GO:0010948	negative regulation of cell cycle process	47/808	359/18866	8.55E-12	2.27E-09	2.00E-09	9319/5149	47
BP	GO:0051306	mitotic sister chromatid separation	19/808	64/18866	1.16E-11	2.91E-09	2.57E-09	11065/931	19
BP	GO:0033047	regulation of mitotic sister chromatid segre	20/808	72/18866	1.30E-11	3.12E-09	2.76E-09	11065/931	20
BP	GO:1902850	microtubule cytoskeleton organization invo	28/808	144/18866	1.47E-11	3.36E-09	2.97E-09	22974/104	28
BP	GO:1905818	regulation of chromosome separation	19/808	66/18866	2.11E-11	4.63E-09	4.09E-09	11065/931	19
BP	GO:1901988	negative regulation of cell cycle phase trans	39/808	270/18866	2.65E-11	5.57E-09	4.92E-09	9319/5149	39
BP	GO:1901991	negative regulation of mitotic cell cycle pha	37/808	251/18866	4.72E-11	9.51E-09	8.41E-09	9319/5149	37
BP	GO:0051304	chromosome separation	22/808	94/18866	5.07E-11	9.83E-09	8.69E-09	11065/931	22
BP	GO:0033044	regulation of chromosome organization	45/808	356/18866	7.85E-11	1.46E-08	1.29E-08	55100/597	45
BP	GO:0098813	nuclear chromosome segregation	38/808	272/18866	1.30E-10	2.34E-08	2.07E-08	11130/110	38
BP	GO:0007091	metaphase/anaphase transition of mitotic c	17/808	58/18866	1.76E-10	3.06E-08	2.70E-08	11065/931	17
BP	GO:0090068	positive regulation of cell cycle process	40/808	302/18866	2.19E-10	3.68E-08	3.25E-08	7552/7480	40
BP	GO:0007051	spindle organization	30/808	183/18866	2.35E-10	3.82E-08	3.38E-08	22974/104	30
BP	GO:0071103	DNA conformation change	44/808	355/18866	2.43E-10	3.82E-08	3.38E-08	7314/7153	44
BP	GO:0033045	regulation of sister chromatid segregation	20/808	84/18866	2.75E-10	4.20E-08	3.71E-08	11065/931	20
BP	GO:0044784	metaphase/anaphase transition of cell cycle	17/808	60/18866	3.18E-10	4.72E-08	4.17E-08	11065/931	17
BP	GO:2001251	negative regulation of chromosome organi	26/808	148/18866	8.02E-10	1.15E-07	1.02E-07	9319/7153	26
BP	GO:0030071	regulation of mitotic metaphase/anaphase	16/808	56/18866	9.24E-10	1.29E-07	1.14E-07	11065/931	16

BP	GO:1902099	regulation of metaphase/anaphase transitio	16/808	58/18866	1.64E-09	2.23E-07	1.97E-07	11065/931	16
BP	GO:0033046	negative regulation of sister chromatid seg	14/808	44/18866	2.17E-09	2.88E-07	2.54E-07	9319/5490	14
BP	GO:0051985	negative regulation of chromosome segreg	14/808	45/18866	3.03E-09	3.91E-07	3.45E-07	9319/5490	14
BP	GO:0010639	negative regulation of organelle organizati	46/808	416/18866	3.98E-09	5.02E-07	4.44E-07	7429/9319	46
BP	GO:2000816	negative regulation of mitotic sister chroma	13/808	39/18866	4.28E-09	5.27E-07	4.65E-07	9319/5490	13
BP	GO:0065004	protein-DNA complex assembly	33/808	243/18866	4.45E-09	5.34E-07	4.72E-07	7480/7314	33
BP	GO:0071824	protein-DNA complex subunit organization	36/808	282/18866	4.80E-09	5.62E-07	4.97E-07	7480/6944	36
BP	GO:0007052	mitotic spindle organization	22/808	118/18866	5.10E-09	5.79E-07	5.11E-07	22974/104	22
BP	GO:1902750	negative regulation of cell cycle G2/M phas	21/808	108/18866	5.17E-09	5.79E-07	5.11E-07	5885/5713	21
BP	GO:1905819	negative regulation of chromosome separa	13/808	40/18866	6.10E-09	6.68E-07	5.90E-07	9319/5490	13
BP	GO:0045787	positive regulation of cell cycle	44/808	396/18866	7.53E-09	8.07E-07	7.13E-07	7552/7480	44
BP	GO:1902036	regulation of hematopoietic stem cell differ	17/808	73/18866	8.61E-09	9.05E-07	7.99E-07	10413/571	17
BP	GO:0033048	negative regulation of mitotic sister chroma	13/808	42/18866	1.19E-08	1.23E-06	1.09E-06	9319/5490	13
BP	GO:0061418	regulation of transcription from RNA polym	17/808	77/18866	2.04E-08	2.05E-06	1.81E-06	7314/5713	17
BP	GO:0051784	negative regulation of nuclear division	15/808	60/18866	2.31E-08	2.28E-06	2.02E-06	9319/5490	15
BP	GO:0045839	negative regulation of mitotic nuclear divisi	14/808	52/18866	2.43E-08	2.35E-06	2.08E-06	9319/5490	14
BP	GO:0006521	regulation of cellular amino acid metabolic	15/808	63/18866	4.70E-08	4.47E-06	3.95E-06	5713/5708	15
BP	GO:0070498	interleukin-1-mediated signaling pathway	19/808	103/18866	6.73E-08	6.28E-06	5.55E-06	7444/7314	19
BP	GO:0006260	DNA replication	33/808	273/18866	7.92E-08	7.26E-06	6.42E-06	58525/597	33
BP	GO:0010972	negative regulation of G2/M transition of m	18/808	96/18866	1.15E-07	1.02E-05	8.99E-06	5885/5713	18
BP	GO:1901532	regulation of hematopoietic progenitor cell	17/808	86/18866	1.15E-07	1.02E-05	8.99E-06	10413/571	17
BP	GO:0060218	hematopoietic stem cell differentiation	17/808	87/18866	1.37E-07	1.19E-05	1.05E-05	10413/571	17
BP	GO:0006323	DNA packaging	30/808	240/18866	1.47E-07	1.25E-05	1.11E-05	7153/1104	30
BP	GO:0045841	negative regulation of mitotic metaphase/a	11/808	36/18866	1.87E-07	1.57E-05	1.39E-05	9319/5490	11
BP	GO:0000075	cell cycle checkpoint	28/808	219/18866	2.37E-07	1.96E-05	1.73E-05	11130/799	28
BP	GO:1902100	negative regulation of metaphase/anaphas	11/808	37/18866	2.56E-07	2.08E-05	1.84E-05	9319/5490	11
BP	GO:0006333	chromatin assembly or disassembly	28/808	220/18866	2.61E-07	2.09E-05	1.85E-05	79723/660	28
BP	GO:0033238	regulation of cellular amine metabolic proc	16/808	81/18866	2.73E-07	2.15E-05	1.90E-05	55737/571	16
BP	GO:0045814	negative regulation of gene expression, epi	20/808	126/18866	3.99E-07	3.09E-05	2.73E-05	55183/593	20
BP	GO:0043161	proteasome-mediated ubiquitin-dependen	42/808	424/18866	4.11E-07	3.14E-05	2.77E-05	11060/748	42
BP	GO:0034508	centromere complex assembly	13/808	56/18866	5.08E-07	3.82E-05	3.37E-05	5931/5532	13
BP	GO:2000736	regulation of stem cell differentiation	19/808	118/18866	6.18E-07	4.58E-05	4.05E-05	10413/677	19
BP	GO:0007093	mitotic cell cycle checkpoint	23/808	166/18866	6.77E-07	4.94E-05	4.37E-05	11130/931	23
BP	GO:0097549	chromatin organization involved in negativ	21/808	143/18866	7.79E-07	5.61E-05	4.96E-05	23378/551	21
BP	GO:0006334	nucleosome assembly	21/808	145/18866	9.84E-07	6.92E-05	6.11E-05	5931/5532	21
BP	GO:0031497	chromatin assembly	25/808	195/18866	9.88E-07	6.92E-05	6.11E-05	23378/593	25
BP	GO:0000910	cytokinesis	23/808	172/18866	1.27E-06	8.79E-05	7.77E-05	3925/1061	23
BP	GO:0043488	regulation of mRNA stability	24/808	186/18866	1.44E-06	9.83E-05	8.68E-05	54915/597	24

BP	GO:0034470	ncRNA processing	39/808	400/18866	1.58E-06	0.000106	9.36E-05	23160/270	39
BP	GO:0006310	DNA recombination	32/808	299/18866	1.91E-06	0.000125	0.00011	7552/7516	32
BP	GO:0010565	regulation of cellular ketone metabolic proc	24/808	189/18866	1.92E-06	0.000125	0.00011	23408/571	24
BP	GO:0006261	DNA-dependent DNA replication	21/808	151/18866	1.93E-06	0.000125	0.00011	58525/597	21
BP	GO:1903311	regulation of mRNA metabolic process	35/808	344/18866	2.06E-06	0.000131	0.000116	54915/597	35
BP	GO:0070507	regulation of microtubule cytoskeleton org	24/808	190/18866	2.12E-06	0.000133	0.000117	22974/104	24
BP	GO:0010498	proteasomal protein catabolic process	44/808	483/18866	2.14E-06	0.000133	0.000117	11060/748	44
BP	GO:0006338	chromatin remodeling	26/808	218/18866	2.40E-06	0.000146	0.000129	6944/7019	26
BP	GO:0036294	cellular response to decreased oxygen leve	26/808	218/18866	2.40E-06	0.000146	0.000129	7314/5710	26
BP	GO:0071453	cellular response to oxygen levels	27/808	235/18866	3.18E-06	0.000191	0.000168	7314/5710	27
BP	GO:0071456	cellular response to hypoxia	25/808	208/18866	3.25E-06	0.000192	0.00017	7314/5710	25
BP	GO:0034401	chromatin organization involved in regulati	21/808	156/18866	3.28E-06	0.000192	0.00017	23378/551	21
BP	GO:0043487	regulation of RNA stability	24/808	195/18866	3.36E-06	0.000194	0.000172	54915/597	24
BP	GO:0031055	chromatin remodeling at centromere	11/808	47/18866	3.52E-06	0.000202	0.000178	5931/5532	11
BP	GO:0034728	nucleosome organization	23/808	183/18866	3.74E-06	0.00021	0.000186	6944/6605	23
BP	GO:0043618	regulation of transcription from RNA polym	17/808	109/18866	3.75E-06	0.00021	0.000186	7314/5713	17
BP	GO:0006270	DNA replication initiation	10/808	39/18866	4.03E-06	0.000223	0.000197	23594/499	10
BP	GO:0045840	positive regulation of mitotic nuclear divisio	12/808	57/18866	4.20E-06	0.00023	0.000203	11065/703	12
BP	GO:0032465	regulation of cytokinesis	15/808	90/18866	6.00E-06	0.000325	0.000287	6755/2912	15
BP	GO:0000082	G1/S transition of mitotic cell cycle	30/808	287/18866	6.39E-06	0.000343	0.000303	7298/5149	30
BP	GO:0090263	positive regulation of canonical Wnt signali	20/808	150/18866	6.47E-06	0.000343	0.000303	10413/748	20
BP	GO:0140013	meiotic nuclear division	22/808	177/18866	7.25E-06	0.000381	0.000336	7552/7314	22
BP	GO:0043620	regulation of DNA-templated transcription	17/808	115/18866	7.88E-06	0.000405	0.000358	7314/5713	17
BP	GO:0051225	spindle assembly	17/808	115/18866	7.88E-06	0.000405	0.000358	22974/106	17
BP	GO:0006302	double-strand break repair	28/808	262/18866	8.51E-06	0.000433	0.000383	7516/5510	28
BP	GO:0051785	positive regulation of nuclear division	13/808	71/18866	8.62E-06	0.000434	0.000384	7552/1106	13
BP	GO:0031146	SCF-dependent proteasomal ubiquitin-dep	15/808	93/18866	9.09E-06	0.00044	0.000389	5713/5708	15
BP	GO:1903046	meiotic cell cycle process	23/808	193/18866	9.18E-06	0.00044	0.000389	7552/7314	23
BP	GO:0007094	mitotic spindle assembly checkpoint	9/808	34/18866	9.26E-06	0.00044	0.000389	9319/5490	9
BP	GO:0031577	spindle checkpoint	9/808	34/18866	9.26E-06	0.00044	0.000389	9319/5490	9
BP	GO:0071173	spindle assembly checkpoint	9/808	34/18866	9.26E-06	0.00044	0.000389	9319/5490	9
BP	GO:0071174	mitotic spindle checkpoint	9/808	34/18866	9.26E-06	0.00044	0.000389	9319/5490	9
BP	GO:0009314	response to radiation	40/808	447/18866	9.45E-06	0.000445	0.000393	10413/751	40
BP	GO:0061013	regulation of mRNA catabolic process	24/808	208/18866	1.03E-05	0.000479	0.000424	54915/597	24
BP	GO:1903706	regulation of hemopoiesis	43/808	498/18866	1.07E-05	0.000497	0.000439	10413/677	43
BP	GO:0044843	cell cycle G1/S phase transition	31/808	310/18866	1.11E-05	0.000506	0.000447	7298/5149	31
BP	GO:0040029	regulation of gene expression, epigenetic	24/808	209/18866	1.11E-05	0.000506	0.000447	5976/5518	24
BP	GO:0016072	rRNA metabolic process	26/808	238/18866	1.20E-05	0.000535	0.000473	23160/270	26

BP	GO:0040001	establishment of mitotic spindle localizatior	9/808	35/18866	1.20E-05	0.000535	0.000473	54908/534	9
BP	GO:0006336	DNA replication-independent nucleosome	11/808	53/18866	1.22E-05	0.000539	0.000476	5931/5532	11
BP	GO:0071347	cellular response to interleukin-1	22/808	183/18866	1.24E-05	0.000544	0.000481	7444/9100	22
BP	GO:0032886	regulation of microtubule-based process	26/808	240/18866	1.39E-05	0.000604	0.000533	22974/104	26
BP	GO:0051302	regulation of cell division	21/808	171/18866	1.40E-05	0.000604	0.000533	7039/6755	21
BP	GO:0034724	DNA replication-independent nucleosome	11/808	54/18866	1.47E-05	0.000624	0.000551	5931/5532	11
BP	GO:0006402	mRNA catabolic process	35/808	376/18866	1.47E-05	0.000624	0.000551	54915/597	35
BP	GO:0042770	signal transduction in response to DNA dar	18/808	133/18866	1.51E-05	0.000633	0.000559	9100/5149	18
BP	GO:0006364	rRNA processing	25/808	227/18866	1.53E-05	0.000633	0.00056	23160/270	25
BP	GO:0002244	hematopoietic progenitor cell differentiatio	21/808	172/18866	1.53E-05	0.000633	0.00056	10413/715	21
BP	GO:0002479	antigen processing and presentation of exo	13/808	75/18866	1.60E-05	0.000657	0.000581	5713/5708	13
BP	GO:0032200	telomere organization	21/808	174/18866	1.83E-05	0.000744	0.000658	5976/5518	21
BP	GO:0034660	ncRNA metabolic process	42/808	493/18866	1.87E-05	0.000755	0.000667	23160/270	42
BP	GO:0006342	chromatin silencing	13/808	77/18866	2.15E-05	0.000861	0.00076	55183/849	13
BP	GO:0061982	meiosis I cell cycle process	17/808	124/18866	2.18E-05	0.000864	0.000764	7552/7314	17
BP	GO:0006401	RNA catabolic process	37/808	415/18866	2.20E-05	0.000866	0.000765	54915/597	37
BP	GO:0022613	ribonucleoprotein complex biogenesis	41/808	482/18866	2.43E-05	0.000951	0.00084	23160/270	41
BP	GO:0010499	proteasomal ubiquitin-independent proteir	7/808	22/18866	2.50E-05	0.000968	0.000855	5695/5691	7
BP	GO:0009308	amine metabolic process	18/808	140/18866	3.05E-05	0.001174	0.001038	55737/681	18
BP	GO:0042590	antigen processing and presentation of exo	13/808	80/18866	3.28E-05	0.001252	0.001106	5713/5708	13
BP	GO:0072331	signal transduction by p53 class mediator	27/808	267/18866	3.30E-05	0.001252	0.001106	9100/7314	27
BP	GO:0032392	DNA geometric change	16/808	116/18866	3.50E-05	0.001307	0.001155	7314/7153	16
BP	GO:0045931	positive regulation of mitotic cell cycle	20/808	168/18866	3.51E-05	0.001307	0.001155	7480/1106	20
BP	GO:0016579	protein deubiquitination	28/808	283/18866	3.53E-05	0.001307	0.001155	9097/2193	28
BP	GO:0051321	meiotic cell cycle	26/808	254/18866	3.73E-05	0.001374	0.001214	7552/7516	26
BP	GO:0070555	response to interleukin-1	23/808	211/18866	3.89E-05	0.001421	0.001256	7444/9100	23
BP	GO:0044106	cellular amine metabolic process	17/808	131/18866	4.47E-05	0.001619	0.001431	55737/571	17
BP	GO:0045089	positive regulation of innate immune respo	23/808	213/18866	4.52E-05	0.001625	0.001436	3093/1045	23
BP	GO:0051052	regulation of DNA metabolic process	33/808	365/18866	4.59E-05	0.00164	0.001449	7644/7552	33
BP	GO:0007127	meiosis I	16/808	119/18866	4.80E-05	0.001705	0.001506	7552/7314	16
BP	GO:0000281	mitotic cytokinesis	12/808	72/18866	5.06E-05	0.001783	0.001576	3925/1061	12
BP	GO:0033209	tumor necrosis factor-mediated signaling p	20/808	173/18866	5.35E-05	0.001872	0.001655	3093/2295	20
BP	GO:0060071	Wnt signaling pathway, planar cell polarity	15/808	108/18866	5.61E-05	0.001951	0.001724	5713/5708	15
BP	GO:0006520	cellular amino acid metabolic process	31/808	340/18866	6.60E-05	0.002279	0.002014	6472/5713	31
BP	GO:0090175	regulation of establishment of planar polari	15/808	110/18866	6.96E-05	0.002382	0.002105	5713/5708	15
BP	GO:0034080	CENP-A containing nucleosome assembly	9/808	43/18866	7.04E-05	0.002382	0.002105	5931/5532	9
BP	GO:0061641	CENP-A containing chromatin organizatior	9/808	43/18866	7.04E-05	0.002382	0.002105	5931/5532	9
BP	GO:0042254	ribosome biogenesis	29/808	310/18866	7.14E-05	0.002391	0.002113	23160/270	29

BP	GO:0048863	stem cell differentiation	26/808	264/18866	7.16E-05	0.002391	0.002113	10413/694	26
BP	GO:0008608	attachment of spindle microtubules to kine	8/808	34/18866	7.36E-05	0.002435	0.002152	10615/291	8
BP	GO:0002478	antigen processing and presentation of exo	20/808	177/18866	7.39E-05	0.002435	0.002152	29127/571	20
BP	GO:0002833	positive regulation of response to biotic sti	25/808	251/18866	8.17E-05	0.002675	0.002364	3093/1045	25
BP	GO:0002831	regulation of response to biotic stimulus	35/808	409/18866	8.36E-05	0.002703	0.002389	3093/5710	35
BP	GO:0035567	non-canonical Wnt signaling pathway	18/808	151/18866	8.37E-05	0.002703	0.002389	5713/5708	18
BP	GO:0090305	nucleic acid phosphodiester bond hydrolys	29/808	313/18866	8.47E-05	0.002718	0.002402	7314/7247	29
BP	GO:0000727	double-strand break repair via break-induc	5/808	12/18866	8.77E-05	0.002796	0.002471	4175/4174	5
BP	GO:0044786	cell cycle DNA replication	11/808	65/18866	8.96E-05	0.00284	0.00251	58525/597	11
BP	GO:0061640	cytoskeleton-dependent cytokinesis	14/808	100/18866	9.02E-05	0.00284	0.00251	3925/1061	14
BP	GO:0002223	stimulatory C-type lectin receptor signaling	15/808	113/18866	9.52E-05	0.002979	0.002633	10454/571	15
BP	GO:0070646	protein modification by small protein remo	28/808	300/18866	9.83E-05	0.00306	0.002704	9097/2193	28
BP	GO:0051293	establishment of spindle localization	9/808	45/18866	0.000103	0.00317	0.002801	54908/534	9
BP	GO:0000460	maturation of 5.8S rRNA	8/808	36/18866	0.000114	0.003495	0.003089	51018/622	8
BP	GO:0043044	ATP-dependent chromatin remodeling	13/808	90/18866	0.000115	0.003523	0.003113	6944/6605	13
BP	GO:0030177	positive regulation of Wnt signaling pathwa	20/808	183/18866	0.000118	0.00357	0.003155	10413/748	20
BP	GO:0006220	pyrimidine nucleotide metabolic process	9/808	46/18866	0.000123	0.003702	0.003271	54963/729	9
BP	GO:0002220	innate immune response activating cell surf	15/808	116/18866	0.000129	0.003856	0.003407	10454/571	15
BP	GO:0019884	antigen processing and presentation of exo	20/808	185/18866	0.000137	0.004071	0.003597	29127/571	20
BP	GO:0051988	regulation of attachment of spindle microtu	5/808	13/18866	0.000137	0.004074	0.0036	10615/291	5
BP	GO:0002758	innate immune response-activating signal t	15/808	117/18866	0.000142	0.004176	0.003691	10454/571	15
BP	GO:0042180	cellular ketone metabolic process	25/808	260/18866	0.000143	0.004189	0.003701	23408/571	25
BP	GO:0036293	response to decreased oxygen levels	32/808	371/18866	0.000144	0.004189	0.003701	7314/5710	32
BP	GO:0030261	chromosome condensation	9/808	47/18866	0.000146	0.004229	0.003737	7153/1104	9
BP	GO:0002218	activation of innate immune response	17/808	144/18866	0.000147	0.004237	0.003744	10454/552	17
BP	GO:0062012	regulation of small molecule metabolic pro	37/808	456/18866	0.000158	0.004511	0.003986	57103/677	37
BP	GO:0015980	energy derivation by oxidation of organic c	26/808	278/18866	0.000167	0.004742	0.004191	57103/647	26
BP	GO:0090224	regulation of spindle organization	8/808	38/18866	0.00017	0.004824	0.004263	22974/104	8
BP	GO:0001666	response to hypoxia	31/808	359/18866	0.000178	0.005011	0.004428	7314/5710	31
BP	GO:0043486	histone exchange	10/808	59/18866	0.000184	0.00514	0.004542	6944/5931	10
BP	GO:0000723	telomere maintenance	18/808	161/18866	0.000191	0.005277	0.004663	5976/5518	18
BP	GO:0031570	DNA integrity checkpoint	18/808	161/18866	0.000191	0.005277	0.004663	79968/514	18
BP	GO:0097711	ciliary basal body-plasma membrane docki	13/808	95/18866	0.000201	0.005542	0.004897	10733/534	13
BP	GO:0031936	negative regulation of chromatin silencing	5/808	14/18866	0.000206	0.005649	0.004992	3010/3009	5
BP	GO:0070482	response to oxygen levels	33/808	396/18866	0.000216	0.005892	0.005206	7314/5710	33
BP	GO:0000132	establishment of mitotic spindle orientation	7/808	30/18866	0.000221	0.005986	0.00529	54908/534	7
BP	GO:0006303	double-strand break repair via nonhomolo	13/808	96/18866	0.000224	0.006032	0.00533	7336/7341	13
BP	GO:0042176	regulation of protein catabolic process	33/808	397/18866	0.000226	0.006072	0.005365	11060/748	33

BP	GO:0051653	spindle localization	9/808	50/18866	0.000239	0.006383	0.00564	54908/534	9
BP	GO:0042775	mitochondrial ATP synthesis coupled electr	13/808	97/18866	0.000248	0.006591	0.005825	6389/4726	13
BP	GO:0071459	protein localization to chromosome, centro	6/808	22/18866	0.000251	0.006613	0.005843	54908/104	6
BP	GO:0048002	antigen processing and presentation of pep	20/808	194/18866	0.000259	0.006801	0.00601	29127/571	20
BP	GO:0034502	protein localization to chromosome	12/808	85/18866	0.000261	0.006806	0.006014	54908/588	12
BP	GO:1901796	regulation of signal transduction by p53 cla	19/808	180/18866	0.000271	0.006977	0.006165	7314/5149	19
BP	GO:0001736	establishment of planar polarity	15/808	124/18866	0.000271	0.006977	0.006165	5713/5708	15
BP	GO:0007164	establishment of tissue polarity	15/808	124/18866	0.000271	0.006977	0.006165	5713/5708	15
BP	GO:0006221	pyrimidine nucleotide biosynthetic process	7/808	31/18866	0.000275	0.006977	0.006165	54963/729	7
BP	GO:0007143	female meiotic nuclear division	7/808	31/18866	0.000275	0.006977	0.006165	7314/9319	7
BP	GO:0042773	ATP synthesis coupled electron transport	13/808	98/18866	0.000275	0.006977	0.006165	6389/4726	13
BP	GO:0031349	positive regulation of defense response	32/808	385/18866	0.000281	0.007071	0.006249	3093/8717	32
BP	GO:0072395	signal transduction involved in cell cycle ch	11/808	74/18866	0.000295	0.007378	0.00652	51499/665	11
BP	GO:0007098	centrosome cycle	15/808	125/18866	0.000296	0.007378	0.00652	7516/6491	15
BP	GO:0072528	pyrimidine-containing compound biosynth	8/808	41/18866	0.000297	0.007378	0.00652	54963/729	8
BP	GO:0002474	antigen processing and presentation of pep	13/808	99/18866	0.000305	0.007533	0.006657	5713/5708	13
BP	GO:0006913	nucleocytoplasmic transport	30/808	354/18866	0.00031	0.007622	0.006736	5976/7307	30
BP	GO:0009147	pyrimidine nucleoside triphosphate metabo	6/808	23/18866	0.000327	0.007997	0.007066	54963/729	6
BP	GO:0060968	regulation of gene silencing	16/808	140/18866	0.000331	0.008065	0.007126	6774/8480	16
BP	GO:0051169	nuclear transport	30/808	357/18866	0.000358	0.008664	0.007656	5976/7307	30
BP	GO:0002066	columnar/cuboidal epithelial cell developm	10/808	64/18866	0.000365	0.008802	0.007778	286451/10	10
BP	GO:0051984	positive regulation of chromosome segrega	7/808	33/18866	0.000415	0.009912	0.008759	5885/9555	7
BP	GO:0090307	mitotic spindle assembly	10/808	65/18866	0.000415	0.009912	0.008759	22974/291	10
BP	GO:0046034	ATP metabolic process	27/808	311/18866	0.000418	0.009912	0.008759	57103/677	27
BP	GO:0060576	intestinal epithelial cell development	5/808	16/18866	0.000419	0.009912	0.008759	286451/10	5
BP	GO:0045333	cellular respiration	19/808	187/18866	0.000439	0.010308	0.009109	6472/6389	19
BP	GO:0022904	respiratory electron transport chain	14/808	116/18866	0.00044	0.010308	0.009109	6389/4726	14
BP	GO:0009266	response to temperature stimulus	22/808	233/18866	0.000452	0.010535	0.00931	7341/6647	22
BP	GO:0001738	morphogenesis of a polarized epithelium	16/808	145/18866	0.000492	0.011379	0.010055	23513/571	16
BP	GO:0009124	nucleoside monophosphate biosynthetic pr	8/808	44/18866	0.000492	0.011379	0.010055	54963/729	8
BP	GO:0000726	non-recombinational repair	13/808	104/18866	0.000496	0.011408	0.010081	7336/7341	13
BP	GO:0006367	transcription initiation from RNA polymeras	19/808	189/18866	0.000501	0.011469	0.010135	10413/748	19
BP	GO:0060236	regulation of mitotic spindle organization	7/808	34/18866	0.000503	0.011469	0.010135	22974/104	7
BP	GO:0050000	chromosome localization	11/808	79/18866	0.000525	0.011872	0.010491	54908/106	11
BP	GO:0051303	establishment of chromosome localization	11/808	79/18866	0.000525	0.011872	0.010491	54908/106	11
BP	GO:0008334	histone mRNA metabolic process	6/808	25/18866	0.000533	0.011991	0.010596	5976/1104	6
BP	GO:0050852	T cell receptor signaling pathway	20/808	206/18866	0.000566	0.012679	0.011204	8767/5713	20
BP	GO:0035999	tetrahydrofolate interconversion	4/808	10/18866	0.00057	0.012715	0.011235	7298/6472	4

BP	GO:0000083	regulation of transcription involved in G1/S	7/808	35/18866	0.000606	0.013389	0.011831	7298/6241	7
BP	GO:0009262	deoxyribonucleotide metabolic process	7/808	35/18866	0.000606	0.013389	0.011831	7298/6241	7
BP	GO:0006304	DNA modification	14/808	120/18866	0.000621	0.013674	0.012084	8493/4913	14
BP	GO:0006119	oxidative phosphorylation	16/808	149/18866	0.000665	0.014563	0.012868	6472/6389	16
BP	GO:0002065	columnar/cuboidal epithelial cell differentia	14/808	121/18866	0.000676	0.014739	0.013025	286451/10	14
BP	GO:0032508	DNA duplex unwinding	13/808	108/18866	0.000714	0.015502	0.013698	7314/7153	13
BP	GO:0051294	establishment of spindle orientation	7/808	36/18866	0.000724	0.01567	0.013847	54908/534	7
BP	GO:0031023	microtubule organizing center organization	15/808	136/18866	0.000732	0.015775	0.01394	7516/6491	15
BP	GO:2001020	regulation of response to DNA damage stir	21/808	226/18866	0.000742	0.015921	0.014069	79968/551	21
BP	GO:0000209	protein polyubiquitination	28/808	340/18866	0.00076	0.016223	0.014336	11060/733	28
BP	GO:0034501	protein localization to kinetochore	5/808	18/18866	0.000765	0.016243	0.014354	54908/104	5
BP	GO:0045088	regulation of innate immune response	26/808	307/18866	0.000767	0.016243	0.014354	3093/1045	26
BP	GO:1905330	regulation of morphogenesis of an epitheli	18/808	181/18866	0.000792	0.016692	0.01475	5713/5708	18
BP	GO:0045815	positive regulation of gene expression, epig	9/808	59/18866	0.000852	0.017835	0.01576	5437/5435	9
BP	GO:0000724	double-strand break repair via homologou	15/808	138/18866	0.000853	0.017835	0.01576	7516/5518	15
BP	GO:0060969	negative regulation of gene silencing	7/808	37/18866	0.000861	0.017928	0.015843	6774/3010	7
BP	GO:0051574	positive regulation of histone H3-K9 methy	4/808	11/18866	0.000866	0.017954	0.015865	55183/593	4
BP	GO:0038095	Fc-epsilon receptor signaling pathway	17/808	169/18866	0.000963	0.01984	0.017532	10454/571	17
BP	GO:0033260	nuclear DNA replication	9/808	60/18866	0.000964	0.01984	0.017532	58525/597	9
BP	GO:0072527	pyrimidine-containing compound metabol	11/808	85/18866	0.000983	0.02012	0.01778	54963/729	11
BP	GO:0000725	recombinational repair	15/808	140/18866	0.00099	0.02012	0.01778	7516/5518	15
BP	GO:0009148	pyrimidine nucleoside triphosphate biosynt	5/808	19/18866	0.001002	0.02012	0.01778	54963/729	5
BP	GO:0031935	regulation of chromatin silencing	5/808	19/18866	0.001002	0.02012	0.01778	3010/3009	5
BP	GO:0033189	response to vitamin A	5/808	19/18866	0.001002	0.02012	0.01778	7298/5914	5
BP	GO:0046653	tetrahydrofolate metabolic process	5/808	19/18866	0.001002	0.02012	0.01778	7298/6472	5
BP	GO:0006760	folic acid-containing compound metabolic	6/808	28/18866	0.001016	0.020175	0.017828	7298/6573	6
BP	GO:0071168	protein localization to chromatin	6/808	28/18866	0.001016	0.020175	0.017828	5885/5347	6
BP	GO:0051567	histone H3-K9 methylation	7/808	38/18866	0.001017	0.020175	0.017828	79723/551	7
BP	GO:0019882	antigen processing and presentation	21/808	232/18866	0.001039	0.02053	0.018141	54915/291	21
BP	GO:0072401	signal transduction involved in DNA integri	10/808	73/18866	0.001059	0.020777	0.01836	51499/665	10
BP	GO:0072422	signal transduction involved in DNA damag	10/808	73/18866	0.001059	0.020777	0.01836	51499/665	10
BP	GO:0008380	RNA splicing	36/808	487/18866	0.001082	0.021137	0.018678	59286/730	36
BP	GO:0051310	metaphase plate congression	9/808	61/18866	0.001089	0.021195	0.018729	54908/106	9
BP	GO:0140056	organelle localization by membrane tetheri	17/808	171/18866	0.001098	0.021277	0.018802	9217/6809	17
BP	GO:0007006	mitochondrial membrane organization	15/808	142/18866	0.001144	0.0221	0.019529	28978/883	15
BP	GO:0006305	DNA alkylation	10/808	74/18866	0.001179	0.022593	0.019964	8493/4609	10
BP	GO:0006306	DNA methylation	10/808	74/18866	0.001179	0.022593	0.019964	8493/4609	10
BP	GO:0009162	deoxyribonucleoside monophosphate meta	4/808	12/18866	0.001255	0.023772	0.021007	7298/7083	4

BP	GO:0021702	cerebellar Purkinje cell differentiation	4/808	12/18866	0.001255	0.023772	0.021007	91137/397	4
BP	GO:0071044	histone mRNA catabolic process	4/808	12/18866	0.001255	0.023772	0.021007	5976/1104	4
BP	GO:0046655	folic acid metabolic process	5/808	20/18866	0.001289	0.024338	0.021507	6573/6472	5
BP	GO:0010212	response to ionizing radiation	15/808	144/18866	0.001319	0.02481	0.021924	10413/751	15
BP	GO:1901293	nucleoside phosphate biosynthetic process	23/808	269/18866	0.001327	0.024868	0.021975	54963/729	23
BP	GO:2000058	regulation of ubiquitin-dependent protein	16/808	159/18866	0.001336	0.024945	0.022043	7480/9097	16
BP	GO:0032259	methylation	29/808	371/18866	0.001391	0.025849	0.022842	7552/7298	29
BP	GO:0034968	histone lysine methylation	13/808	116/18866	0.001395	0.025849	0.022842	7552/7972	13
BP	GO:1903829	positive regulation of cellular protein localiz	27/808	338/18866	0.001465	0.027039	0.023894	7429/5928	27
BP	GO:1902751	positive regulation of cell cycle G2/M phase	6/808	30/18866	0.001489	0.027191	0.024028	7480/4204	6
BP	GO:0000377	RNA splicing, via transesterification reactior	30/808	390/18866	0.001489	0.027191	0.024028	59286/730	30
BP	GO:0000398	mRNA splicing, via spliceosome	30/808	390/18866	0.001489	0.027191	0.024028	59286/730	30
BP	GO:0007007	inner mitochondrial membrane organizatio	8/808	52/18866	0.001546	0.028135	0.024862	8834/2651	8
BP	GO:0043624	cellular protein complex disassembly	20/808	224/18866	0.001595	0.02892	0.025555	7429/5976	20
BP	GO:0000375	RNA splicing, via transesterification reactior	30/808	393/18866	0.001675	0.030259	0.026739	59286/730	30
BP	GO:0045732	positive regulation of protein catabolic pro	20/808	225/18866	0.001682	0.030284	0.026761	11060/557	20
BP	GO:0045682	regulation of epidermis development	11/808	91/18866	0.00173	0.031036	0.027425	7480/8717	11
BP	GO:0021694	cerebellar Purkinje cell layer formation	4/808	13/18866	0.001751	0.031187	0.027559	91137/397	4
BP	GO:0043247	telomere maintenance in response to DNA	4/808	13/18866	0.001751	0.031187	0.027559	55183/207	4
BP	GO:0009394	2'-deoxyribonucleotide metabolic process	6/808	31/18866	0.00178	0.03159	0.027915	7298/4521	6
BP	GO:1902275	regulation of chromatin organization	18/808	195/18866	0.001852	0.032751	0.028941	55100/807	18
BP	GO:1903362	regulation of cellular protein catabolic proc	22/808	260/18866	0.001906	0.033593	0.029685	7480/5573	22
BP	GO:0022406	membrane docking	17/808	180/18866	0.001918	0.033648	0.029734	9217/6809	17
BP	GO:0046824	positive regulation of nucleocytoplasmic tra	9/808	66/18866	0.001923	0.033648	0.029734	10204/229	9
BP	GO:0051656	establishment of organelle localization	32/808	432/18866	0.001933	0.033719	0.029796	7314/8369	32
BP	GO:0090501	RNA phosphodiester bond hydrolysis	16/808	165/18866	0.001965	0.034145	0.030173	7247/7904	16
BP	GO:0051383	kinetochore organization	5/808	22/18866	0.00204	0.035208	0.031112	10403/978	5
BP	GO:0062033	positive regulation of mitotic sister chromat	5/808	22/18866	0.00204	0.035208	0.031112	9555/9700	5
BP	GO:0060828	regulation of canonical Wnt signaling pathw	24/808	295/18866	0.002059	0.035378	0.031262	10413/748	24
BP	GO:0045132	meiotic chromosome segregation	11/808	93/18866	0.002064	0.035378	0.031262	9319/7153	11
BP	GO:0030330	DNA damage response, signal transduction	12/808	107/18866	0.002087	0.035591	0.03145	9100/5149	12
BP	GO:0009408	response to heat	16/808	166/18866	0.00209	0.035591	0.03145	7341/6647	16
BP	GO:0032984	protein-containing complex disassembly	26/808	330/18866	0.002151	0.036373	0.032142	7429/5976	26
BP	GO:0031647	regulation of protein stability	24/808	296/18866	0.002153	0.036373	0.032142	7480/5852	24
BP	GO:2001252	positive regulation of chromosome organiz	17/808	182/18866	0.002158	0.036373	0.032142	55183/588	17
BP	GO:0036297	interstrand cross-link repair	8/808	55/18866	0.002236	0.037557	0.033188	7314/1063	8
BP	GO:0044774	mitotic DNA integrity checkpoint	12/808	108/18866	0.002259	0.037819	0.03342	51499/715	12
BP	GO:0006241	CTP biosynthetic process	4/808	14/18866	0.002369	0.039278	0.034709	54963/102	4

BP	GO:0007100	mitotic centrosome separation	4/808	14/18866	0.002369	0.039278	0.034709	5902/4751	4
BP	GO:0046036	CTP metabolic process	4/808	14/18866	0.002369	0.039278	0.034709	54963/102	4
BP	GO:0031123	RNA 3'-end processing	15/808	153/18866	0.002408	0.039631	0.035021	7307/1104	15
BP	GO:0006611	protein export from nucleus	17/808	184/18866	0.002422	0.039631	0.035021	5976/7307	17
BP	GO:0022900	electron transport chain	17/808	184/18866	0.002422	0.039631	0.035021	6389/4726	17
BP	GO:0090090	negative regulation of canonical Wnt signa	17/808	184/18866	0.002422	0.039631	0.035021	6657/5713	17
BP	GO:0051054	positive regulation of DNA metabolic proce	18/808	200/18866	0.002447	0.039907	0.035265	7552/5710	18
BP	GO:0045684	positive regulation of epidermis developme	7/808	44/18866	0.002472	0.04019	0.035514	7480/8717	7
BP	GO:0006352	DNA-templated transcription, initiation	21/808	249/18866	0.002484	0.040223	0.035544	10413/748	21
BP	GO:0019692	deoxyribose phosphate metabolic process	6/808	33/18866	0.00249	0.040223	0.035544	7298/4521	6
BP	GO:0051570	regulation of histone H3-K9 methylation	5/808	23/18866	0.002516	0.040508	0.035795	55183/593	5
BP	GO:0009165	nucleotide biosynthetic process	22/808	266/18866	0.002531	0.040624	0.035898	54963/729	22
BP	GO:1901989	positive regulation of cell cycle phase trans	12/808	110/18866	0.002637	0.042186	0.037278	7480/1106	12
BP	GO:0043414	macromolecule methylation	25/808	318/18866	0.002666	0.042525	0.037578	7552/9676	25
BP	GO:0034605	cellular response to heat	13/808	125/18866	0.002734	0.043472	0.038415	7341/5931	13
BP	GO:0072431	signal transduction involved in mitotic G1 D	8/808	57/18866	0.002815	0.044434	0.039264	51499/665	8
BP	GO:1902400	intracellular signal transduction involved in	8/808	57/18866	0.002815	0.044434	0.039264	51499/665	8
BP	GO:0051204	protein insertion into mitochondrial membr	7/808	45/18866	0.002821	0.044434	0.039264	26517/265	7
BP	GO:0050821	protein stabilization	17/808	187/18866	0.002868	0.045032	0.039794	7480/5852	17
BP	GO:0042558	pteridine-containing compound metabolic	6/808	34/18866	0.002916	0.045639	0.04033	7298/6573	6
BP	GO:0051168	nuclear export	18/808	204/18866	0.003031	0.04729	0.041789	5976/7307	18
BP	GO:0051004	regulation of lipoprotein lipase activity	5/808	24/18866	0.003067	0.047564	0.04203	4023/345/	5
BP	GO:1904666	regulation of ubiquitin protein ligase activit	5/808	24/18866	0.003067	0.047564	0.04203	11065/534	5
BP	GO:0009209	pyrimidine ribonucleoside triphosphate bio	4/808	15/18866	0.003122	0.047938	0.042361	54963/102	4
BP	GO:0051299	centrosome separation	4/808	15/18866	0.003122	0.047938	0.042361	5902/4751	4
BP	GO:0051315	attachment of mitotic spindle microtubules	4/808	15/18866	0.003122	0.047938	0.042361	10403/106	4
BP	GO:0044728	DNA methylation or demethylation	11/808	98/18866	0.003129	0.047938	0.042361	8493/4609	11
CC	GO:0098687	chromosomal region	56/829	350/19559	6.75E-18	3.91E-15	3.22E-15	11130/597	56
CC	GO:0000775	chromosome, centromeric region	38/829	196/19559	2.67E-15	7.73E-13	6.37E-13	11130/797	38
CC	GO:0005819	spindle	52/829	367/19559	1.82E-14	3.14E-12	2.58E-12	55689/229	52
CC	GO:0000779	condensed chromosome, centromeric regio	29/829	122/19559	2.17E-14	3.14E-12	2.58E-12	11130/549	29
CC	GO:0000793	condensed chromosome	37/829	222/19559	8.31E-13	9.62E-11	7.92E-11	11130/715	37
CC	GO:0000777	condensed chromosome kinetochore	25/829	106/19559	1.64E-12	1.58E-10	1.30E-10	11130/549	25
CC	GO:0000776	kinetochore	28/829	137/19559	3.27E-12	2.70E-10	2.23E-10	11130/549	28
CC	GO:0072686	mitotic spindle	24/829	133/19559	1.68E-09	1.22E-07	1.00E-07	55689/229	24
CC	GO:0000922	spindle pole	25/829	165/19559	3.10E-08	1.99E-06	1.64E-06	22974/104	25
CC	GO:0000502	proteasome complex	15/829	63/19559	4.11E-08	2.38E-06	1.96E-06	9097/5137	15
CC	GO:1905369	endopeptidase complex	15/829	69/19559	1.51E-07	7.92E-06	6.53E-06	9097/5137	15

CC	GO:0051233	spindle midzone	11/829	36/19559	1.69E-07	8.14E-06	6.70E-06	55183/291	11
CC	GO:1905368	peptidase complex	16/829	92/19559	1.45E-06	6.45E-05	5.31E-05	9097/5137	16
CC	GO:0000792	heterochromatin	14/829	78/19559	4.44E-06	0.000184	0.000151	79968/734	14
CC	GO:0000780	condensed nuclear chromosome, centrome	9/829	32/19559	4.92E-06	0.00019	0.000156	5885/5347	9
CC	GO:0031970	organelle envelope lumen	15/829	90/19559	5.31E-06	0.000192	0.000158	51499/167	15
CC	GO:0005758	mitochondrial intermembrane space	14/829	80/19559	6.04E-06	0.000206	0.00017	51499/167	14
CC	GO:0005839	proteasome core complex	7/829	20/19559	1.14E-05	0.000368	0.000303	5695/5691	7
CC	GO:0005743	mitochondrial inner membrane	42/829	489/19559	1.22E-05	0.000371	0.000305	7298/1045	42
CC	GO:0005876	spindle microtubule	12/829	66/19559	1.85E-05	0.000537	0.000442	220134/90	12
CC	GO:0000781	chromosome, telomeric region	20/829	164/19559	2.14E-05	0.00059	0.000486	5976/6741	20
CC	GO:0098798	mitochondrial protein complex	27/829	265/19559	2.43E-05	0.000639	0.000526	10452/265	27
CC	GO:0030496	midbody	21/829	182/19559	3.12E-05	0.000785	0.000646	58525/981	21
CC	GO:1902555	endoribonuclease complex	8/829	31/19559	3.33E-05	0.000804	0.000662	79042/107	8
CC	GO:0005874	microtubule	37/829	431/19559	4.01E-05	0.000929	0.000765	80086/229	37
CC	GO:0000778	condensed nuclear chromosome kinetocho	6/829	17/19559	4.71E-05	0.00105	0.000865	5347/1040	6
CC	GO:0000784	nuclear chromosome, telomeric region	16/829	124/19559	7.06E-05	0.001513	0.001246	5976/6741	16
CC	GO:0005759	mitochondrial matrix	38/829	473/19559	0.000129	0.002598	0.002139	7298/5480	38
CC	GO:1905348	endonuclease complex	8/829	37/19559	0.00013	0.002598	0.002139	79042/107	8
CC	GO:0005838	proteasome regulatory particle	6/829	21/19559	0.000179	0.003449	0.002841	5713/5708	6
CC	GO:0000940	condensed chromosome outer kinetochore	5/829	14/19559	0.000196	0.003555	0.002928	54908/220	5
CC	GO:1990023	mitotic spindle midzone	5/829	14/19559	0.000196	0.003555	0.002928	9055/1469	5
CC	GO:0101002	ficolin-1-rich granule	15/829	124/19559	0.000243	0.004143	0.003412	5713/5708	15
CC	GO:1904813	ficolin-1-rich granule lumen	15/829	124/19559	0.000243	0.004143	0.003412	5713/5708	15
CC	GO:0031616	spindle pole centrosome	5/829	15/19559	0.000284	0.004706	0.003876	10101/201	5
CC	GO:0044391	ribosomal subunit	19/829	187/19559	0.000387	0.005781	0.004761	6227/6205	19
CC	GO:0044815	DNA packaging complex	14/829	116/19559	0.000397	0.005781	0.004761	23397/641	14
CC	GO:0022624	proteasome accessory complex	6/829	24/19559	0.000398	0.005781	0.004761	5713/5708	6
CC	GO:0045120	pronucleus	5/829	16/19559	0.000399	0.005781	0.004761	55183/319	5
CC	GO:0032154	cleavage furrow	9/829	54/19559	0.000405	0.005781	0.004761	10617/589	9
CC	GO:0000794	condensed nuclear chromosome	13/829	103/19559	0.000409	0.005781	0.004761	5885/5810	13
CC	GO:0005635	nuclear envelope	36/829	473/19559	0.000535	0.007371	0.00607	7444/2319	36
CC	GO:0032153	cell division site	10/829	72/19559	0.000879	0.01183	0.009742	10617/108	10
CC	GO:0098803	respiratory chain complex	11/829	85/19559	0.000904	0.011902	0.009802	6389/4726	11
CC	GO:0016607	nuclear speck	31/829	401/19559	0.000985	0.012668	0.010433	6944/5589	31
CC	GO:0005721	pericentric heterochromatin	5/829	20/19559	0.001231	0.015496	0.012762	9555/3070	5
CC	GO:0000786	nucleosome	12/829	108/19559	0.002075	0.02556	0.021051	55506/955	12
CC	GO:0005720	nuclear heterochromatin	6/829	34/19559	0.002769	0.033407	0.027512	9555/3015	6
CC	GO:0005686	U2 snRNP	5/829	24/19559	0.002933	0.034655	0.02854	6637/6629	5

CC	GO:0005643	nuclear pore	10/829	85/19559	0.003134	0.036287	0.029885	7341/5902	10
CC	GO:0005681	spliceosomal complex	17/829	192/19559	0.003395	0.03854	0.03174	5976/7307	17
CC	GO:0070469	respirasome	11/829	101/19559	0.003661	0.040767	0.033574	6389/4726	11
CC	GO:0015934	large ribosomal subunit	12/829	116/19559	0.003768	0.041158	0.033896	6181/1168	12
CC	GO:0005746	mitochondrial respirasome	10/829	89/19559	0.004379	0.046954	0.038669	6389/4726	10
CC	GO:0005747	mitochondrial respiratory chain complex I	7/829	50/19559	0.004884	0.049609	0.040856	4726/4713	7
CC	GO:0030964	NADH dehydrogenase complex	7/829	50/19559	0.004884	0.049609	0.040856	4726/4713	7
CC	GO:0045271	respiratory chain complex I	7/829	50/19559	0.004884	0.049609	0.040856	4726/4713	7
MF	GO:0016887	ATPase activity	45/821	423/18352	6.84E-08	5.88E-05	5.45E-05	7516/5976	45
MF	GO:0031490	chromatin DNA binding	16/821	106/18352	1.89E-05	0.006715	0.006222	6774/6605	16
MF	GO:0004298	threonine-type endopeptidase activity	7/821	21/18352	2.34E-05	0.006715	0.006222	5695/5691	7
MF	GO:0031492	nucleosomal DNA binding	9/821	40/18352	5.38E-05	0.011176	0.010356	6605/9555	9
MF	GO:0140097	catalytic activity, acting on DNA	23/821	209/18352	6.50E-05	0.011176	0.010356	7153/5984	23
MF	GO:0004386	helicase activity	19/821	160/18352	0.0001	0.014376	0.01332	5976/5984	19
MF	GO:0043138	3'-5' DNA helicase activity	6/821	19/18352	0.000129	0.01589	0.014723	9401/4175	6
MF	GO:0008094	DNA-dependent ATPase activity	14/821	108/18352	0.000324	0.034231	0.031717	7516/7153	14
MF	GO:0070003	threonine-type peptidase activity	7/821	31/18352	0.000358	0.034231	0.031717	5695/5691	7
MF	GO:0003688	DNA replication origin binding	6/821	23/18352	0.000413	0.0355	0.032893	4998/4175	6
MF	GO:0004518	nuclease activity	21/821	210/18352	0.000495	0.035753	0.033127	7247/7904	21
MF	GO:0015631	tubulin binding	31/821	365/18352	0.000499	0.035753	0.033127	9217/2297	31
BP	GO:0001667	ameboidal-type cell migration	45/660	481/18866	2.14E-09	1.07E-05	8.70E-06	7048/7040	45
BP	GO:0030198	extracellular matrix organization	39/660	395/18866	5.86E-09	1.07E-05	8.70E-06	64856/717	39
BP	GO:0043062	extracellular structure organization	39/660	396/18866	6.29E-09	1.07E-05	8.70E-06	64856/717	39
BP	GO:0048015	phosphatidylinositol-mediated signaling	25/660	192/18866	1.58E-08	2.02E-05	1.64E-05	7337/7249	25
BP	GO:0040013	negative regulation of locomotion	38/660	397/18866	2.13E-08	2.05E-05	1.66E-05	54361/704	38
BP	GO:0048017	inositol lipid-mediated signaling	25/660	196/18866	2.40E-08	2.05E-05	1.66E-05	7337/7249	25
BP	GO:0061448	connective tissue development	29/660	262/18866	4.59E-08	3.32E-05	2.69E-05	9658/6935	29
BP	GO:0060348	bone development	25/660	204/18866	5.35E-08	3.32E-05	2.69E-05	7048/6424	25
BP	GO:0014068	positive regulation of phosphatidylinositol 3	16/660	88/18866	5.84E-08	3.32E-05	2.69E-05	7337/7989	16
BP	GO:0061041	regulation of wound healing	21/660	151/18866	7.06E-08	3.62E-05	2.93E-05	54361/709	21
BP	GO:0051271	negative regulation of cellular component r	37/660	400/18866	7.93E-08	3.69E-05	2.99E-05	54361/704	37
BP	GO:0014066	regulation of phosphatidylinositol 3-kinase	19/660	127/18866	9.09E-08	3.88E-05	3.14E-05	7337/7249	19
BP	GO:0001649	osteoblast differentiation	26/660	231/18866	1.63E-07	6.14E-05	4.97E-05	23051/543	26
BP	GO:0001503	ossification	37/660	412/18866	1.68E-07	6.14E-05	4.97E-05	23051/543	37
BP	GO:0007599	hemostasis	33/660	348/18866	2.26E-07	7.73E-05	6.26E-05	7099/7035	33
BP	GO:0010632	regulation of epithelial cell migration	30/660	301/18866	2.77E-07	8.71E-05	7.06E-05	7048/7040	30
BP	GO:0048771	tissue remodeling	22/660	178/18866	2.89E-07	8.71E-05	7.06E-05	7040/1051	22
BP	GO:0045669	positive regulation of osteoblast differentia	13/660	66/18866	3.87E-07	0.000109	8.85E-05	23051/543	13

BP	GO:0060485	mesenchyme development	29/660	290/18866	4.08E-07	0.000109	8.85E-05	54361/101	29
BP	GO:0014065	phosphatidylinositol 3-kinase signaling	20/660	154/18866	4.48E-07	0.000109	8.85E-05	7337/7249	20
BP	GO:0061045	negative regulation of wound healing	14/660	78/18866	4.63E-07	0.000109	8.85E-05	54361/703	14
BP	GO:1903034	regulation of response to wounding	22/660	183/18866	4.69E-07	0.000109	8.85E-05	54361/709	22
BP	GO:0007596	blood coagulation	32/660	343/18866	4.97E-07	0.000111	8.96E-05	7099/7035	32
BP	GO:0090287	regulation of cellular response to growth fa	30/660	310/18866	5.21E-07	0.000111	9.02E-05	23090/693	30
BP	GO:0003151	outflow tract morphogenesis	14/660	80/18866	6.40E-07	0.000128	0.000103	7048/1051	14
BP	GO:0010631	epithelial cell migration	33/660	365/18866	6.73E-07	0.000128	0.000103	7048/7040	33
BP	GO:2000146	negative regulation of cell motility	33/660	365/18866	6.73E-07	0.000128	0.000103	54361/704	33
BP	GO:0050817	coagulation	32/660	349/18866	7.29E-07	0.000133	0.000108	7099/7035	32
BP	GO:0090132	epithelium migration	33/660	368/18866	8.09E-07	0.000143	0.000116	7048/7040	33
BP	GO:0045778	positive regulation of ossification	15/660	94/18866	8.80E-07	0.000147	0.000119	23051/543	15
BP	GO:0071560	cellular response to transforming growth fa	26/660	252/18866	8.93E-07	0.000147	0.000119	7791/678/	26
BP	GO:0090130	tissue migration	33/660	374/18866	1.16E-06	0.000186	0.00015	7048/7040	33
BP	GO:0071559	response to transforming growth factor bet	26/660	258/18866	1.40E-06	0.000217	0.000175	7791/678/	26
BP	GO:0051216	cartilage development	22/660	197/18866	1.65E-06	0.000249	0.000202	6935/7048	22
BP	GO:0048762	mesenchymal cell differentiation	24/660	229/18866	1.77E-06	0.000259	0.00021	54361/101	24
BP	GO:0070661	leukocyte proliferation	29/660	313/18866	1.95E-06	0.000278	0.000225	7535/5436	29
BP	GO:0030336	negative regulation of cell migration	31/660	350/18866	2.26E-06	0.00031	0.000251	54361/704	31
BP	GO:0030098	lymphocyte differentiation	32/660	368/18866	2.30E-06	0.00031	0.000251	678/6935/	32
BP	GO:0014033	neural crest cell differentiation	14/660	89/18866	2.42E-06	0.000318	0.000258	6591/6469	14
BP	GO:0046651	lymphocyte proliferation	27/660	283/18866	2.53E-06	0.000323	0.000262	7535/5436	27
BP	GO:0002576	platelet degranulation	17/660	129/18866	2.64E-06	0.00033	0.000267	10490/707	17
BP	GO:0030203	glycosaminoglycan metabolic process	19/660	158/18866	2.80E-06	0.000342	0.000277	7040/5557	19
BP	GO:0032943	mononuclear cell proliferation	27/660	286/18866	3.08E-06	0.000367	0.000298	7535/5436	27
BP	GO:0045667	regulation of osteoblast differentiation	17/660	131/18866	3.27E-06	0.000381	0.000309	23051/543	17
BP	GO:0007178	transmembrane receptor protein serine/thr	31/660	359/18866	3.82E-06	0.000434	0.000352	7791/2309	31
BP	GO:1903035	negative regulation of response to woundir	14/660	93/18866	4.14E-06	0.000461	0.000373	54361/703	14
BP	GO:0071902	positive regulation of protein serine/threon	30/660	345/18866	4.75E-06	0.000517	0.000419	7099/7040	30
BP	GO:0030278	regulation of ossification	22/660	212/18866	5.56E-06	0.000594	0.000481	23051/543	22
BP	GO:0048863	stem cell differentiation	25/660	264/18866	6.74E-06	0.000691	0.000559	678/8626/	25
BP	GO:0048732	gland development	35/660	443/18866	6.74E-06	0.000691	0.000559	54361/799	35
BP	GO:0006027	glycosaminoglycan catabolic process	11/660	61/18866	7.51E-06	0.000755	0.000611	7040/5557	11
BP	GO:0045785	positive regulation of cell adhesion	34/660	428/18866	8.12E-06	0.000799	0.000648	7535/5436	34
BP	GO:0006022	aminoglycan metabolic process	19/660	172/18866	9.80E-06	0.000947	0.000768	7040/5557	19
BP	GO:0032102	negative regulation of response to external	34/660	433/18866	1.04E-05	0.000985	0.000798	54361/713	34
BP	GO:1902105	regulation of leukocyte differentiation	26/660	290/18866	1.18E-05	0.001089	0.000882	678/6935/	26
BP	GO:0071383	cellular response to steroid hormone stimu	21/660	206/18866	1.19E-05	0.001089	0.000882	678/7337/	21

BP	GO:0010634	positive regulation of epithelial cell migratio	19/660	176/18866	1.37E-05	0.001224	0.000991	7048/7040	19
BP	GO:0022407	regulation of cell-cell adhesion	34/660	439/18866	1.39E-05	0.001224	0.000991	7535/5436	34
BP	GO:0032970	regulation of actin filament-based process	32/660	405/18866	1.66E-05	0.001443	0.001169	54361/508	32
BP	GO:0002683	negative regulation of immune system proc	35/660	463/18866	1.74E-05	0.001449	0.001174	7292/2895	35
BP	GO:0045621	positive regulation of lymphocyte differenti	14/660	105/18866	1.74E-05	0.001449	0.001174	7535/7292	14
BP	GO:1901342	regulation of vasculature development	34/660	444/18866	1.75E-05	0.001449	0.001174	54361/704	34
BP	GO:0042326	negative regulation of phosphorylation	36/660	484/18866	1.87E-05	0.001523	0.001234	65125/724	36
BP	GO:0006026	aminoglycan catabolic process	11/660	67/18866	1.91E-05	0.001528	0.001238	7040/5557	11
BP	GO:0014032	neural crest cell development	12/660	80/18866	2.08E-05	0.001637	0.001326	6591/6469	12
BP	GO:0007162	negative regulation of cell adhesion	26/660	301/18866	2.27E-05	0.001746	0.001415	65125/729	26
BP	GO:0018108	peptidyl-tyrosine phosphorylation	30/660	374/18866	2.28E-05	0.001746	0.001415	7535/7132	30
BP	GO:0019216	regulation of lipid metabolic process	33/660	431/18866	2.33E-05	0.001757	0.001423	54361/713	33
BP	GO:1904375	regulation of protein localization to cell per	15/660	123/18866	2.60E-05	0.001933	0.001566	10490/704	15
BP	GO:0018212	peptidyl-tyrosine modification	30/660	377/18866	2.66E-05	0.001944	0.001575	7535/7132	30
BP	GO:1901653	cellular response to peptide	31/660	398/18866	2.96E-05	0.002134	0.001729	7249/7099	31
BP	GO:0150146	cell junction disassembly	6/660	19/18866	3.29E-05	0.002342	0.001897	6591/1098	6
BP	GO:1905314	semi-lunar valve development	8/660	37/18866	3.38E-05	0.002342	0.001897	7132/7040	8
BP	GO:0014031	mesenchymal cell development	12/660	84/18866	3.43E-05	0.002342	0.001897	6591/6469	12
BP	GO:0048864	stem cell development	12/660	84/18866	3.43E-05	0.002342	0.001897	6591/6469	12
BP	GO:0007159	leukocyte cell-cell adhesion	29/660	364/18866	3.53E-05	0.002378	0.001926	7535/6512	29
BP	GO:0048545	response to steroid hormone	28/660	346/18866	3.63E-05	0.002417	0.001958	678/7337/	28
BP	GO:0045765	regulation of angiogenesis	31/660	403/18866	3.75E-05	0.002435	0.001973	7048/6558	31
BP	GO:0048660	regulation of smooth muscle cell proliferati	18/660	173/18866	3.76E-05	0.002435	0.001973	7048/5159	18
BP	GO:0032963	collagen metabolic process	14/660	113/18866	4.02E-05	0.002545	0.002062	54361/704	14
BP	GO:1903510	mucopolysaccharide metabolic process	14/660	113/18866	4.02E-05	0.002545	0.002062	7040/5557	14
BP	GO:0050866	negative regulation of cell activation	20/660	207/18866	4.15E-05	0.00255	0.002066	7292/2320	20
BP	GO:0048009	insulin-like growth factor receptor signaling	8/660	38/18866	4.15E-05	0.00255	0.002066	7249/5295	8
BP	GO:0051051	negative regulation of transport	35/660	483/18866	4.18E-05	0.00255	0.002066	65125/713	35
BP	GO:0050727	regulation of inflammatory response	32/660	425/18866	4.28E-05	0.002576	0.002087	7791/1031	32
BP	GO:0010712	regulation of collagen metabolic process	9/660	49/18866	4.36E-05	0.002576	0.002087	54361/704	9
BP	GO:0048659	smooth muscle cell proliferation	18/660	175/18866	4.38E-05	0.002576	0.002087	7048/5159	18
BP	GO:0003170	heart valve development	10/660	61/18866	4.61E-05	0.002686	0.002176	7132/7048	10
BP	GO:0014706	striated muscle tissue development	30/660	389/18866	4.76E-05	0.002739	0.002219	7048/7040	30
BP	GO:0001894	tissue homeostasis	23/660	261/18866	4.88E-05	0.002752	0.00223	7099/2734	23
BP	GO:0003012	muscle system process	34/660	467/18866	4.89E-05	0.002752	0.00223	10911/713	34
BP	GO:0060537	muscle tissue development	31/660	409/18866	4.96E-05	0.002761	0.002237	7048/7040	31
BP	GO:0033002	muscle cell proliferation	22/660	244/18866	5.02E-05	0.002766	0.002241	7048/6469	22
BP	GO:1903076	regulation of protein localization to plasma	13/660	102/18866	5.59E-05	0.003046	0.002468	10490/704	13

BP	GO:1901136	carbohydrate derivative catabolic process	19/660	195/18866	5.71E-05	0.00308	0.002496	7040/5557	19
BP	GO:0071526	semaphorin-plexin signaling pathway	8/660	40/18866	6.13E-05	0.003273	0.002651	8482/9037	8
BP	GO:0050673	epithelial cell proliferation	33/660	453/18866	6.22E-05	0.003286	0.002662	8626/7099	33
BP	GO:0045619	regulation of lymphocyte differentiation	18/660	181/18866	6.82E-05	0.003563	0.002886	678/6935/	18
BP	GO:0003179	heart valve morphogenesis	9/660	52/18866	7.11E-05	0.003679	0.00298	7048/7040	9
BP	GO:0060411	cardiac septum morphogenesis	11/660	77/18866	7.24E-05	0.003696	0.002994	7048/1051	11
BP	GO:0032956	regulation of actin cytoskeleton organizatio	28/660	360/18866	7.29E-05	0.003696	0.002994	54361/508	28
BP	GO:0061037	negative regulation of cartilage developme	7/660	31/18866	7.85E-05	0.00394	0.003192	6591/5744	7
BP	GO:0032964	collagen biosynthetic process	9/660	53/18866	8.30E-05	0.004084	0.003309	54361/704	9
BP	GO:0014812	muscle cell migration	13/660	106/18866	8.37E-05	0.004084	0.003309	5327/5159	13
BP	GO:0046632	alpha-beta T cell differentiation	13/660	106/18866	8.37E-05	0.004084	0.003309	7535/7292	13
BP	GO:0045582	positive regulation of T cell differentiation	12/660	92/18866	8.54E-05	0.004121	0.003339	7535/7292	12
BP	GO:0030217	T cell differentiation	22/660	253/18866	8.61E-05	0.004121	0.003339	678/6935/	22
BP	GO:0007409	axonogenesis	34/660	482/18866	9.09E-05	0.004184	0.00339	8408/2290	34
BP	GO:0090092	regulation of transmembrane receptor prot	22/660	254/18866	9.12E-05	0.004184	0.00339	23090/693	22
BP	GO:0050670	regulation of lymphocyte proliferation	20/660	219/18866	9.14E-05	0.004184	0.00339	7535/7292	20
BP	GO:0010827	regulation of glucose transmembrane trans	11/660	79/18866	9.19E-05	0.004184	0.00339	6041/2343	11
BP	GO:0007179	transforming growth factor beta receptor si	19/660	202/18866	9.19E-05	0.004184	0.00339	7791/6935	19
BP	GO:0007265	Ras protein signal transduction	27/660	346/18866	9.23E-05	0.004184	0.00339	10155/712	27
BP	GO:0042110	T cell activation	34/660	483/18866	9.47E-05	0.004194	0.003397	678/6935/	34
BP	GO:0046777	protein autophosphorylation	21/660	237/18866	9.49E-05	0.004194	0.003397	7535/8408	21
BP	GO:0014909	smooth muscle cell migration	12/660	93/18866	9.50E-05	0.004194	0.003397	5327/5159	12
BP	GO:0003176	aortic valve development	7/660	32/18866	9.74E-05	0.004246	0.00344	7132/7040	7
BP	GO:0001933	negative regulation of protein phosphoryla	32/660	444/18866	9.78E-05	0.004246	0.00344	65125/724	32
BP	GO:1903037	regulation of leukocyte cell-cell adhesion	26/660	329/18866	0.000101	0.004342	0.003517	7535/6512	26
BP	GO:0032944	regulation of mononuclear cell proliferatior	20/660	221/18866	0.000104	0.00442	0.003581	7535/7292	20
BP	GO:0043401	steroid hormone mediated signaling pathw	15/660	139/18866	0.000108	0.004562	0.003696	7337/8626	15
BP	GO:0070977	bone maturation	6/660	23/18866	0.000109	0.004562	0.003696	6261/387/	6
BP	GO:0043434	response to peptide hormone	32/660	447/18866	0.000111	0.004615	0.003739	54832/724	32
BP	GO:0070663	regulation of leukocyte proliferation	21/660	240/18866	0.000113	0.004683	0.003794	7535/7292	21
BP	GO:0003007	heart morphogenesis	22/660	258/18866	0.000115	0.004697	0.003806	7048/7040	22
BP	GO:0016239	positive regulation of macroautophagy	10/660	68/18866	0.000119	0.004856	0.003934	54832/840	10
BP	GO:1903053	regulation of extracellular matrix organizati	8/660	44/18866	0.000125	0.00504	0.004083	7132/7040	8
BP	GO:0003018	vascular process in circulatory system	18/660	190/18866	0.000127	0.005095	0.004128	10911/704	18
BP	GO:1903707	negative regulation of hemopoiesis	16/660	158/18866	0.000138	0.005466	0.004428	7292/2895	16
BP	GO:0060396	growth hormone receptor signaling pathwa	6/660	24/18866	0.000141	0.00554	0.004488	6777/6776	6
BP	GO:0030111	regulation of Wnt signaling pathway	28/660	375/18866	0.000146	0.005712	0.004627	65125/799	28
BP	GO:1902107	positive regulation of leukocyte differentiat	16/660	159/18866	0.000148	0.005751	0.004659	7535/7292	16

BP	GO:0046578	regulation of Ras protein signal transductio	18/660	194/18866	0.000166	0.00638	0.005169	7126/8878	18
BP	GO:0046579	positive regulation of Ras protein signal tra	10/660	71/18866	0.000173	0.0066	0.005347	5159/1098	10
BP	GO:0003279	cardiac septum development	13/660	114/18866	0.000177	0.006702	0.00543	7048/1051	13
BP	GO:0071378	cellular response to growth hormone stimu	6/660	25/18866	0.00018	0.006763	0.005479	6777/6776	6
BP	GO:0043405	regulation of MAP kinase activity	26/660	342/18866	0.000188	0.006993	0.005665	7099/7040	26
BP	GO:0051056	regulation of small GTPase mediated signal	25/660	323/18866	0.000188	0.006993	0.005665	7249/7126	25
BP	GO:0061035	regulation of cartilage development	10/660	72/18866	0.000194	0.007096	0.005749	6591/860/	10
BP	GO:0042306	regulation of protein import into nucleus	9/660	59/18866	0.000195	0.007096	0.005749	10155/704	9
BP	GO:0048008	platelet-derived growth factor receptor sig	9/660	59/18866	0.000195	0.007096	0.005749	10628/532	9
BP	GO:0050772	positive regulation of axonogenesis	11/660	86/18866	0.000199	0.007192	0.005826	22906/848	11
BP	GO:0003206	cardiac chamber morphogenesis	14/660	131/18866	0.000202	0.007192	0.005826	7048/7040	14
BP	GO:0046638	positive regulation of alpha-beta T cell diffe	8/660	47/18866	0.000202	0.007192	0.005826	7535/7292	8
BP	GO:0048608	reproductive structure development	31/660	443/18866	0.000211	0.007469	0.006051	54361/733	31
BP	GO:0034405	response to fluid shear stress	7/660	36/18866	0.000214	0.007507	0.006081	5311/5159	7
BP	GO:0032967	positive regulation of collagen biosynthetic	6/660	26/18866	0.000227	0.007896	0.006396	54361/704	6
BP	GO:0042113	B cell activation	25/660	328/18866	0.000238	0.008252	0.006685	678/7535/	25
BP	GO:0009755	hormone-mediated signaling pathway	18/660	200/18866	0.000242	0.008267	0.006697	7337/8626	18
BP	GO:0031032	actomyosin structure organization	18/660	200/18866	0.000242	0.008267	0.006697	7791/5436	18
BP	GO:0061458	reproductive system development	31/660	447/18866	0.000247	0.008393	0.0068	54361/733	31
BP	GO:0045580	regulation of T cell differentiation	15/660	150/18866	0.000253	0.008481	0.006871	6935/7535	15
BP	GO:0046324	regulation of glucose import	9/660	61/18866	0.000253	0.008481	0.006871	6041/2343	9
BP	GO:0002695	negative regulation of leukocyte activation	17/660	184/18866	0.000262	0.008717	0.007062	7292/2320	17
BP	GO:0002460	adaptive immune response based on soma	27/660	370/18866	0.000276	0.009108	0.007378	7292/7099	27
BP	GO:0003148	outflow tract septum morphogenesis	6/660	27/18866	0.000283	0.009168	0.007427	7048/1051	6
BP	GO:0010714	positive regulation of collagen metabolic p	6/660	27/18866	0.000283	0.009168	0.007427	54361/704	6
BP	GO:0048799	animal organ maturation	6/660	27/18866	0.000283	0.009168	0.007427	6261/387/	6
BP	GO:1904589	regulation of protein import	9/660	62/18866	0.000287	0.009256	0.007498	10155/704	9
BP	GO:0050708	regulation of protein secretion	26/660	352/18866	0.000295	0.009436	0.007644	79971/729	26
BP	GO:0060828	regulation of canonical Wnt signaling pathv	23/660	295/18866	0.000303	0.009616	0.00779	65125/799	23
BP	GO:1902106	negative regulation of leukocyte differentia	12/660	105/18866	0.000304	0.009616	0.00779	7292/2895	12
BP	GO:0032496	response to lipopolysaccharide	25/660	334/18866	0.000314	0.009832	0.007965	7292/7099	25
BP	GO:0030195	negative regulation of blood coagulation	8/660	50/18866	0.000315	0.009832	0.007965	7035/710/	8
BP	GO:0046849	bone remodeling	11/660	91/18866	0.00033	0.010229	0.008287	7040/9770	11
BP	GO:0034446	substrate adhesion-dependent cell spreadi	12/660	106/18866	0.000332	0.010253	0.008306	6239/387/	12
BP	GO:0003180	aortic valve morphogenesis	6/660	28/18866	0.000349	0.010506	0.008511	7040/6591	6
BP	GO:0061437	renal system vasculature development	6/660	28/18866	0.000349	0.010506	0.008511	5311/5159	6
BP	GO:0061440	kidney vasculature development	6/660	28/18866	0.000349	0.010506	0.008511	5311/5159	6
BP	GO:0071880	adenylate cyclase-activating adrenergic rec	6/660	28/18866	0.000349	0.010506	0.008511	5350/2771	6

BP	GO:0019935	cyclic-nucleotide-mediated signaling	19/660	224/18866	0.000351	0.010506	0.008511	5744/5577	19
BP	GO:0060416	response to growth hormone	7/660	39/18866	0.00036	0.010598	0.008586	6777/6776	7
BP	GO:1903706	regulation of hemopoiesis	33/660	498/18866	0.00036	0.010598	0.008586	678/6935/	33
BP	GO:0043277	apoptotic cell clearance	8/660	51/18866	0.000362	0.010598	0.008586	80208/399	8
BP	GO:1900047	negative regulation of hemostasis	8/660	51/18866	0.000362	0.010598	0.008586	7035/710/	8
BP	GO:0051057	positive regulation of small GTPase mediate	10/660	78/18866	0.000378	0.011001	0.008912	5159/1098	10
BP	GO:0060070	canonical Wnt signaling pathway	25/660	339/18866	0.000392	0.011227	0.009095	54361/651	25
BP	GO:0002062	chondrocyte differentiation	12/660	108/18866	0.000395	0.011227	0.009095	7048/7040	12
BP	GO:0006024	glycosaminoglycan biosynthetic process	12/660	108/18866	0.000395	0.011227	0.009095	7040/1104	12
BP	GO:0031589	cell-substrate adhesion	26/660	359/18866	0.000399	0.011227	0.009095	7791/5436	26
BP	GO:0045063	T-helper 1 cell differentiation	5/660	19/18866	0.000399	0.011227	0.009095	7292/6421	5
BP	GO:0072074	kidney mesenchyme development	5/660	19/18866	0.000399	0.011227	0.009095	54361/646	5
BP	GO:0086023	adenylate cyclase-activating adrenergic rec	4/660	11/18866	0.000402	0.011263	0.009125	5350/2771	4
BP	GO:0003205	cardiac chamber development	16/660	174/18866	0.000415	0.011549	0.009356	7048/7040	16
BP	GO:0006809	nitric oxide biosynthetic process	10/660	79/18866	0.000419	0.011613	0.009408	7099/6542	10
BP	GO:0042307	positive regulation of protein import into n	7/660	40/18866	0.000423	0.011658	0.009445	10155/704	7
BP	GO:0009306	protein secretion	31/660	462/18866	0.000436	0.01194	0.009673	79971/729	31
BP	GO:0003014	renal system process	13/660	125/18866	0.00044	0.011962	0.00969	387/5577/	13
BP	GO:0007411	axon guidance	22/660	284/18866	0.000443	0.011962	0.00969	6709/6469	22
BP	GO:0030168	platelet activation	15/660	158/18866	0.000444	0.011962	0.00969	7099/5270	15
BP	GO:0035592	establishment of protein localization to extr	31/660	463/18866	0.000452	0.012124	0.009822	79971/729	31
BP	GO:0097485	neuron projection guidance	22/660	285/18866	0.000465	0.012404	0.010049	6709/6469	22
BP	GO:0090100	positive regulation of transmembrane recep	12/660	110/18866	0.000468	0.012418	0.01006	23090/704	12
BP	GO:0070371	ERK1 and ERK2 cascade	24/660	325/18866	0.000498	0.01316	0.010662	678/10318	24
BP	GO:0048738	cardiac muscle tissue development	19/660	231/18866	0.000514	0.013502	0.010938	7048/7040	19
BP	GO:0043931	ossification involved in bone maturation	5/660	20/18866	0.000517	0.013502	0.010938	6261/387/	5
BP	GO:0060249	anatomical structure homeostasis	31/660	469/18866	0.000561	0.014586	0.011817	3104/8035	31
BP	GO:0030193	regulation of blood coagulation	10/660	82/18866	0.000567	0.014672	0.011886	7099/7035	10
BP	GO:1904591	positive regulation of protein import	7/660	42/18866	0.000577	0.014842	0.012024	10155/704	7
BP	GO:0071692	protein localization to extracellular region	31/660	470/18866	0.000581	0.014883	0.012057	79971/729	31
BP	GO:0051549	positive regulation of keratinocyte migratio	4/660	12/18866	0.000587	0.014883	0.012057	10982/944	4
BP	GO:0070493	thrombin-activated receptor signaling path	4/660	12/18866	0.000587	0.014883	0.012057	4233/3248	4
BP	GO:0050819	negative regulation of coagulation	8/660	55/18866	0.000612	0.015454	0.01252	7035/710/	8
BP	GO:0071375	cellular response to peptide hormone stimu	24/660	330/18866	0.000619	0.015548	0.012596	7249/6777	24
BP	GO:1900046	regulation of hemostasis	10/660	83/18866	0.000625	0.015566	0.01261	7099/7035	10
BP	GO:0030316	osteoclast differentiation	11/660	98/18866	0.000626	0.015566	0.01261	7099/9770	11
BP	GO:1903039	positive regulation of leukocyte cell-cell ad	19/660	235/18866	0.000635	0.015701	0.012719	7535/7292	19
BP	GO:0032147	activation of protein kinase activity	24/660	331/18866	0.000646	0.015856	0.012846	65125/709	24

BP	GO:0010810	regulation of cell-substrate adhesion	18/660	217/18866	0.000647	0.015856	0.012846	54361/623	18
BP	GO:0003177	pulmonary valve development	5/660	21/18866	0.000659	0.016067	0.013017	7132/4846	5
BP	GO:0030521	androgen receptor signaling pathway	7/660	43/18866	0.000668	0.016222	0.013142	7337/387/	7
BP	GO:0046209	nitric oxide metabolic process	10/660	84/18866	0.000688	0.016581	0.013433	7099/6542	10
BP	GO:0019218	regulation of steroid metabolic process	13/660	131/18866	0.00069	0.016581	0.013433	54361/677	13
BP	GO:0001755	neural crest cell migration	8/660	56/18866	0.000693	0.016587	0.013438	6469/8482	8
BP	GO:0006023	aminoglycan biosynthetic process	12/660	115/18866	0.0007	0.016687	0.013519	7040/1104	12
BP	GO:0006644	phospholipid metabolic process	30/660	455/18866	0.000712	0.016895	0.013687	7040/6610	30
BP	GO:0019932	second-messenger-mediated signaling	30/660	456/18866	0.000738	0.017372	0.014074	7535/5164	30
BP	GO:0010594	regulation of endothelial cell migration	19/660	238/18866	0.00074	0.017372	0.014074	7040/6776	19
BP	GO:1903531	negative regulation of secretion by cell	15/660	166/18866	0.000746	0.017372	0.014074	7132/2320	15
BP	GO:0071875	adrenergic receptor signaling pathway	6/660	32/18866	0.000746	0.017372	0.014074	5350/2771	6
BP	GO:0015749	monosaccharide transmembrane transport	12/660	116/18866	0.000757	0.017544	0.014213	9962/6041	12
BP	GO:0010463	mesenchymal cell proliferation	7/660	44/18866	0.000771	0.01771	0.014348	6935/7048	7
BP	GO:0032965	regulation of collagen biosynthetic process	7/660	44/18866	0.000771	0.01771	0.014348	54361/704	7
BP	GO:0042531	positive regulation of tyrosine phosphoryla	9/660	71/18866	0.000798	0.018193	0.014739	7132/3717	9
BP	GO:0002237	response to molecule of bacterial origin	25/660	356/18866	0.000799	0.018193	0.014739	7292/7099	25
BP	GO:0070665	positive regulation of leukocyte proliferatio	14/660	150/18866	0.000805	0.018253	0.014787	7535/7292	14
BP	GO:0030282	bone mineralization	12/660	117/18866	0.000817	0.018441	0.014939	54361/704	12
BP	GO:0021537	telencephalon development	20/660	259/18866	0.000824	0.018441	0.014939	56949/796	20
BP	GO:0043568	positive regulation of insulin-like growth fa	4/660	13/18866	0.000824	0.018441	0.014939	3487/3486	4
BP	GO:0014910	regulation of smooth muscle cell migration	10/660	86/18866	0.000829	0.018443	0.014942	5159/8031	10
BP	GO:0051251	positive regulation of lymphocyte activatior	25/660	357/18866	0.000832	0.018443	0.014942	7535/7292	25
BP	GO:0022409	positive regulation of cell-cell adhesion	21/660	279/18866	0.000858	0.018881	0.015296	7535/7292	21
BP	GO:1903708	positive regulation of hemopoiesis	17/660	204/18866	0.000859	0.018881	0.015296	7535/7292	17
BP	GO:0007566	embryo implantation	8/660	58/18866	0.00088	0.019067	0.015446	10155/704	8
BP	GO:0034219	carbohydrate transmembrane transport	12/660	118/18866	0.000882	0.019067	0.015446	9962/6041	12
BP	GO:0010464	regulation of mesenchymal cell proliferatio	6/660	33/18866	0.000885	0.019067	0.015446	6935/7048	6
BP	GO:0001974	blood vessel remodeling	7/660	45/18866	0.000886	0.019067	0.015446	7040/1051	7
BP	GO:0010828	positive regulation of glucose transmembra	7/660	45/18866	0.000886	0.019067	0.015446	6041/2343	7
BP	GO:2001057	reactive nitrogen species metabolic process	10/660	87/18866	0.000908	0.019442	0.015751	7099/6542	10
BP	GO:0002285	lymphocyte activation involved in immune	16/660	187/18866	0.000911	0.019442	0.015751	7292/7099	16
BP	GO:0001837	epithelial to mesenchymal transition	14/660	152/18866	0.000917	0.019497	0.015795	54361/101	14
BP	GO:0070997	neuron death	25/660	360/18866	0.000937	0.019825	0.016061	8626/7099	25
BP	GO:0008630	intrinsic apoptotic signaling pathway in res	11/660	103/18866	0.000953	0.020078	0.016266	8626/7132	11
BP	GO:0002791	regulation of peptide secretion	26/660	381/18866	0.000964	0.020232	0.016391	79971/729	26
BP	GO:0010517	regulation of phospholipase activity	9/660	73/18866	0.000978	0.020408	0.016533	5330/5159	9
BP	GO:0050671	positive regulation of lymphocyte proliferat	13/660	136/18866	0.00098	0.020408	0.016533	7535/7292	13

BP	GO:0050818	regulation of coagulation	10/660	88/18866	0.000993	0.020555	0.016652	7099/7035	10
BP	GO:0050730	regulation of peptidyl-tyrosine phosphoryla	20/660	263/18866	0.000995	0.020555	0.016652	7132/7040	20
BP	GO:0006936	muscle contraction	25/660	362/18866	0.001013	0.02069	0.016762	10911/808	25
BP	GO:0010761	fibroblast migration	7/660	46/18866	0.001014	0.02069	0.016762	7040/7989	7
BP	GO:0010863	positive regulation of phospholipase C acti	7/660	46/18866	0.001014	0.02069	0.016762	5330/5159	7
BP	GO:1903077	negative regulation of protein localization t	5/660	23/18866	0.001028	0.020895	0.016928	7040/2343	5
BP	GO:0044706	multi-multicellular organism process	18/660	226/18866	0.001036	0.020979	0.016996	54361/101	18
BP	GO:0043406	positive regulation of MAP kinase activity	20/660	264/18866	0.001043	0.021023	0.017032	7099/7040	20
BP	GO:0032946	positive regulation of mononuclear cell pro	13/660	137/18866	0.001049	0.021073	0.017072	7535/7292	13
BP	GO:0051048	negative regulation of secretion	16/660	190/18866	0.001079	0.021588	0.017489	7132/2320	16
BP	GO:0030865	cortical cytoskeleton organization	8/660	60/18866	0.001105	0.021944	0.017778	23433/399	8
BP	GO:0010935	regulation of macrophage cytokine produc	4/660	14/18866	0.001122	0.021944	0.017778	7099/7040	4
BP	GO:0033604	negative regulation of catecholamine secre	4/660	14/18866	0.001122	0.021944	0.017778	23208/269	4
BP	GO:0035810	positive regulation of urine volume	4/660	14/18866	0.001122	0.021944	0.017778	4881/8692	4
BP	GO:0051547	regulation of keratinocyte migration	4/660	14/18866	0.001122	0.021944	0.017778	10982/944	4
BP	GO:0060397	growth hormone receptor signaling pathwa	4/660	14/18866	0.001122	0.021944	0.017778	6777/6776	4
BP	GO:0022408	negative regulation of cell-cell adhesion	16/660	191/18866	0.001141	0.022212	0.017994	65125/729	16
BP	GO:0016049	cell growth	31/660	490/18866	0.001146	0.022225	0.018005	8408/7048	31
BP	GO:0050919	negative chemotaxis	7/660	47/18866	0.001156	0.022343	0.018101	8482/9037	7
BP	GO:0043542	endothelial cell migration	21/660	286/18866	0.001173	0.022595	0.018305	7040/6776	21
BP	GO:0046427	positive regulation of receptor signaling pa	10/660	90/18866	0.001182	0.022608	0.018316	7132/3717	10
BP	GO:0002573	myeloid leukocyte differentiation	17/660	210/18866	0.001183	0.022608	0.018316	7099/7048	17
BP	GO:0046323	glucose import	9/660	75/18866	0.00119	0.022653	0.018352	6041/2343	9
BP	GO:0050920	regulation of chemotaxis	18/660	229/18866	0.001204	0.022757	0.018436	65125/704	18
BP	GO:0030038	contractile actin filament bundle assembly	11/660	106/18866	0.001208	0.022757	0.018436	7791/5436	11
BP	GO:0043149	stress fiber assembly	11/660	106/18866	0.001208	0.022757	0.018436	7791/5436	11
BP	GO:0010259	multicellular organism aging	6/660	35/18866	0.001223	0.022859	0.018518	8626/7799	6
BP	GO:0098751	bone cell development	6/660	35/18866	0.001223	0.022859	0.018518	5305/5048	6
BP	GO:0033673	negative regulation of kinase activity	20/660	268/18866	0.001252	0.023057	0.018679	65125/724	20
BP	GO:0010770	positive regulation of cell morphogenesis ir	14/660	157/18866	0.001256	0.023057	0.018679	22906/848	14
BP	GO:0043271	negative regulation of ion transport	14/660	157/18866	0.001256	0.023057	0.018679	65125/232	14
BP	GO:0051017	actin filament bundle assembly	14/660	157/18866	0.001256	0.023057	0.018679	7791/5436	14
BP	GO:0030325	adrenal gland development	5/660	24/18866	0.001262	0.023057	0.018679	54361/515	5
BP	GO:0032331	negative regulation of chondrocyte differer	5/660	24/18866	0.001262	0.023057	0.018679	6591/5744	5
BP	GO:0097191	extrinsic apoptotic signaling pathway	18/660	230/18866	0.001265	0.023057	0.018679	7185/7132	18
BP	GO:0034103	regulation of tissue remodeling	10/660	91/18866	0.001287	0.023297	0.018874	7040/4054	10
BP	GO:0060021	roof of mouth development	10/660	91/18866	0.001287	0.023297	0.018874	7048/6591	10
BP	GO:0014911	positive regulation of smooth muscle cell m	7/660	48/18866	0.001313	0.023518	0.019052	5159/8031	7

BP	GO:0048701	embryonic cranial skeleton morphogenesis	7/660	48/18866	0.001313	0.023518	0.019052	7048/860/	7
BP	GO:1900274	regulation of phospholipase C activity	7/660	48/18866	0.001313	0.023518	0.019052	5330/5159	7
BP	GO:0035051	cardiocyte differentiation	14/660	158/18866	0.001335	0.023828	0.019303	7040/1051	14
BP	GO:0042098	T cell proliferation	16/660	195/18866	0.001417	0.025116	0.020347	7535/5436	16
BP	GO:0050731	positive regulation of peptidyl-tyrosine pho	16/660	195/18866	0.001417	0.025116	0.020347	7132/7040	16
BP	GO:0030500	regulation of bone mineralization	9/660	77/18866	0.001437	0.025375	0.020557	54361/704	9
BP	GO:0050870	positive regulation of T cell activation	17/660	214/18866	0.001452	0.025561	0.020708	7535/7292	17
BP	GO:0030879	mammary gland development	13/660	142/18866	0.001458	0.025572	0.020717	54361/704	13
BP	GO:0045346	regulation of MHC class II biosynthetic proc	4/660	15/18866	0.001489	0.025846	0.020938	7099/5196	4
BP	GO:0060391	positive regulation of SMAD protein signal	4/660	15/18866	0.001489	0.025846	0.020938	7040/3717	4
BP	GO:0072075	metanephric mesenchyme development	4/660	15/18866	0.001489	0.025846	0.020938	54361/646	4
BP	GO:0007565	female pregnancy	16/660	196/18866	0.001494	0.02586	0.02095	54361/101	16
BP	GO:0050863	regulation of T cell activation	23/660	332/18866	0.00152	0.026134	0.021172	6935/7535	23
BP	GO:1904894	positive regulation of receptor signaling pa	10/660	93/18866	0.001521	0.026134	0.021172	7132/3717	10
BP	GO:0046635	positive regulation of alpha-beta T cell acti	8/660	63/18866	0.001526	0.026134	0.021172	7535/7292	8
BP	GO:1904376	negative regulation of protein localization t	5/660	25/18866	0.001532	0.026158	0.021191	7040/2343	5
BP	GO:0035296	regulation of tube diameter	13/660	143/18866	0.001554	0.026341	0.021339	10911/387	13
BP	GO:0097746	regulation of blood vessel diameter	13/660	143/18866	0.001554	0.026341	0.021339	10911/387	13
BP	GO:0018210	peptidyl-threonine modification	12/660	126/18866	0.001563	0.026341	0.021339	65125/840	12
BP	GO:0055007	cardiac muscle cell differentiation	12/660	126/18866	0.001563	0.026341	0.021339	7040/1061	12
BP	GO:0061572	actin filament bundle organization	14/660	161/18866	0.001597	0.026826	0.021732	7791/5436	14
BP	GO:0045444	fat cell differentiation	18/660	235/18866	0.00161	0.026867	0.021765	9658/678/	18
BP	GO:0050678	regulation of epithelial cell proliferation	26/660	395/18866	0.00161	0.026867	0.021765	8626/7099	26
BP	GO:1904659	glucose transmembrane transport	11/660	110/18866	0.001635	0.027164	0.022006	6041/2343	11
BP	GO:0030212	hyaluronan metabolic process	6/660	37/18866	0.001651	0.027164	0.022006	7040/5557	6
BP	GO:0048846	axon extension involved in axon guidance	6/660	37/18866	0.001651	0.027164	0.022006	8482/9037	6
BP	GO:1902284	neuron projection extension involved in ne	6/660	37/18866	0.001651	0.027164	0.022006	8482/9037	6
BP	GO:0035150	regulation of tube size	13/660	144/18866	0.001655	0.027164	0.022006	10911/387	13
BP	GO:0010718	positive regulation of epithelial to mesench	7/660	50/18866	0.001677	0.027448	0.022237	7048/7040	7
BP	GO:0045453	bone resorption	8/660	64/18866	0.001691	0.027499	0.022278	4054/7970	8
BP	GO:0046637	regulation of alpha-beta T cell differentiatio	8/660	64/18866	0.001691	0.027499	0.022278	7535/7292	8
BP	GO:0019933	cAMP-mediated signaling	16/660	199/18866	0.001748	0.028336	0.022956	5744/5577	16
BP	GO:0070167	regulation of biomineral tissue developmer	10/660	95/18866	0.001787	0.028732	0.023277	54361/704	10
BP	GO:0110149	regulation of biomineralization	10/660	95/18866	0.001787	0.028732	0.023277	54361/704	10
BP	GO:0031214	biomineral tissue development	14/660	163/18866	0.001795	0.028732	0.023277	54361/704	14
BP	GO:0110148	biomineralization	14/660	163/18866	0.001795	0.028732	0.023277	54361/704	14
BP	GO:1901888	regulation of cell junction assembly	16/660	200/18866	0.00184	0.029224	0.023675	54361/799	16
BP	GO:0043567	regulation of insulin-like growth factor rece	5/660	26/18866	0.001843	0.029224	0.023675	3489/3487	5

BP	GO:0072012	glomerulus vasculature development	5/660	26/18866	0.001843	0.029224	0.023675	5159/5156	5
BP	GO:0046631	alpha-beta T cell activation	13/660	146/18866	0.001873	0.029609	0.023987	7535/7292	13
BP	GO:0002312	B cell activation involved in immune respor	9/660	80/18866	0.001883	0.029674	0.02404	7292/7099	9
BP	GO:0071604	transforming growth factor beta productior	6/660	38/18866	0.001903	0.02969	0.024053	4060/4054	6
BP	GO:0050867	positive regulation of cell activation	27/660	421/18866	0.001919	0.02969	0.024053	7535/7292	27
BP	GO:0010934	macrophage cytokine production	4/660	16/18866	0.00193	0.02969	0.024053	7099/7040	4
BP	GO:0030214	hyaluronan catabolic process	4/660	16/18866	0.00193	0.02969	0.024053	7040/5557	4
BP	GO:0045342	MHC class II biosynthetic process	4/660	16/18866	0.00193	0.02969	0.024053	7099/5196	4
BP	GO:0072109	glomerular mesangium development	4/660	16/18866	0.00193	0.02969	0.024053	5159/8031	4
BP	GO:0086103	G protein-coupled receptor signaling pathv	4/660	16/18866	0.00193	0.02969	0.024053	5350/2771	4
BP	GO:1905153	regulation of membrane invagination	4/660	16/18866	0.00193	0.02969	0.024053	23208/110	4
BP	GO:0030177	positive regulation of Wnt signaling pathwa	15/660	183/18866	0.001991	0.030438	0.024658	65125/799	15
BP	GO:0007189	adenylate cyclase-activating G protein-cou	13/660	147/18866	0.001991	0.030438	0.024658	5744/5350	13
BP	GO:0052547	regulation of peptidase activity	29/660	466/18866	0.002082	0.031731	0.025706	8626/7078	29
BP	GO:0042100	B cell proliferation	10/660	97/18866	0.00209	0.031771	0.025739	7099/5585	10
BP	GO:0032330	regulation of chondrocyte differentiation	7/660	52/18866	0.002115	0.032058	0.025971	6591/860/	7
BP	GO:1901214	regulation of neuron death	22/660	321/18866	0.002177	0.032801	0.026573	7099/2911	22
BP	GO:0008645	hexose transmembrane transport	11/660	114/18866	0.002178	0.032801	0.026573	6041/2343	11
BP	GO:0046326	positive regulation of glucose import	6/660	39/18866	0.002184	0.032801	0.026573	6041/2343	6
BP	GO:0051953	negative regulation of amine transport	5/660	27/18866	0.002197	0.032911	0.026662	23208/269	5
BP	GO:0042129	regulation of T cell proliferation	14/660	167/18866	0.002252	0.033602	0.027222	7535/7292	14
BP	GO:0048010	vascular endothelial growth factor receptor	10/660	98/18866	0.002257	0.033602	0.027222	387/5296/	10
BP	GO:0050918	positive chemotaxis	8/660	67/18866	0.002274	0.033669	0.027276	7249/9037	8
BP	GO:0072678	T cell migration	8/660	67/18866	0.002274	0.033669	0.027276	7535/6512	8
BP	GO:0006650	glycerophospholipid metabolic process	23/660	343/18866	0.00231	0.034095	0.027621	57515/515	23
BP	GO:0003184	pulmonary valve morphogenesis	4/660	17/18866	0.002455	0.035629	0.028864	4846/182/	4
BP	GO:0006957	complement activation, alternative pathway	4/660	17/18866	0.002455	0.035629	0.028864	81494/307	4
BP	GO:0010715	regulation of extracellular matrix disassemb	4/660	17/18866	0.002455	0.035629	0.028864	7040/4035	4
BP	GO:0035855	megakaryocyte development	4/660	17/18866	0.002455	0.035629	0.028864	5305/4211	4
BP	GO:0046068	cGMP metabolic process	4/660	17/18866	0.002455	0.035629	0.028864	5152/5136	4
BP	GO:0008217	regulation of blood pressure	15/660	187/18866	0.002456	0.035629	0.028864	10911/299	15
BP	GO:0009187	cyclic nucleotide metabolic process	6/660	40/18866	0.002494	0.035774	0.028981	5744/5152	6
BP	GO:0030518	intracellular steroid hormone receptor signa	11/660	116/18866	0.002499	0.035774	0.028981	7337/8626	11
BP	GO:0045428	regulation of nitric oxide biosynthetic proce	8/660	68/18866	0.0025	0.035774	0.028981	7099/5740	8
BP	GO:1904377	positive regulation of protein localization to	8/660	68/18866	0.0025	0.035774	0.028981	8878/1107	8
BP	GO:1904888	cranial skeletal system development	8/660	68/18866	0.0025	0.035774	0.028981	8626/7048	8
BP	GO:0002449	lymphocyte mediated immunity	24/660	366/18866	0.002511	0.035826	0.029024	7292/7040	24
BP	GO:0008643	carbohydrate transport	13/660	151/18866	0.002524	0.035918	0.029098	11046/996	13

BP	GO:0051402	neuron apoptotic process	18/660	245/18866	0.002541	0.036059	0.029212	8626/387/	18
BP	GO:0002335	mature B cell differentiation	5/660	28/18866	0.002599	0.036777	0.029794	4242/9452	5
BP	GO:1901343	negative regulation of vasculature developm	16/660	207/18866	0.002607	0.03678	0.029797	54361/655	16
BP	GO:0030099	myeloid cell differentiation	27/660	431/18866	0.002663	0.037331	0.030243	7099/7048	27
BP	GO:0070372	regulation of ERK1 and ERK2 cascade	21/660	306/18866	0.002671	0.037331	0.030243	10318/709	21
BP	GO:0018107	peptidyl-threonine phosphorylation	11/660	117/18866	0.002674	0.037331	0.030243	65125/840	11
BP	GO:0002224	toll-like receptor signaling pathway	13/660	152/18866	0.002675	0.037331	0.030243	10318/709	13
BP	GO:0045766	positive regulation of angiogenesis	16/660	208/18866	0.002735	0.038069	0.03084	7048/9037	16
BP	GO:0031331	positive regulation of cellular catabolic proc	25/660	390/18866	0.00279	0.038631	0.031296	678/10444	25
BP	GO:0003158	endothelium development	12/660	135/18866	0.002798	0.038631	0.031296	6935/7132	12
BP	GO:0030856	regulation of epithelial cell differentiation	14/660	171/18866	0.0028	0.038631	0.031296	6935/8626	14
BP	GO:0042102	positive regulation of T cell proliferation	10/660	101/18866	0.002821	0.038631	0.031296	7535/7292	10
BP	GO:0042116	macrophage activation	10/660	101/18866	0.002821	0.038631	0.031296	7099/2320	10
BP	GO:0060191	regulation of lipase activity	10/660	101/18866	0.002821	0.038631	0.031296	387/5330/	10
BP	GO:1905209	positive regulation of cardiocyte differentia	6/660	41/18866	0.002837	0.038751	0.031393	7040/1051	6
BP	GO:2000736	regulation of stem cell differentiation	11/660	118/18866	0.002858	0.038931	0.031539	678/10512	11
BP	GO:0030900	forebrain development	25/660	391/18866	0.002885	0.039199	0.031756	56949/543	25
BP	GO:0034329	cell junction assembly	27/660	434/18866	0.00293	0.039702	0.032164	54361/799	27
BP	GO:0022604	regulation of cell morphogenesis	30/660	499/18866	0.00294	0.039735	0.032191	7337/2290	30
BP	GO:0051250	negative regulation of lymphocyte activatio	13/660	154/18866	0.002997	0.040318	0.032662	7292/6469	13
BP	GO:0008088	axo-dendritic transport	8/660	70/18866	0.003005	0.040318	0.032662	22906/802	8
BP	GO:0035265	organ growth	15/660	191/18866	0.003007	0.040318	0.032662	7337/7048	15
BP	GO:0060840	artery development	10/660	102/18866	0.003032	0.040526	0.032831	6469/5396	10
BP	GO:0001655	urogenital system development	22/660	330/18866	0.003051	0.040526	0.032831	54361/733	22
BP	GO:0045671	negative regulation of osteoclast differentia	5/660	29/18866	0.003052	0.040526	0.032831	7099/5295	5
BP	GO:0006491	N-glycan processing	4/660	18/18866	0.00307	0.040526	0.032831	7903/1125	4
BP	GO:0006883	cellular sodium ion homeostasis	4/660	18/18866	0.00307	0.040526	0.032831	64699/655	4
BP	GO:0010766	negative regulation of sodium ion transpor	4/660	18/18866	0.00307	0.040526	0.032831	65125/273	4
BP	GO:0042509	regulation of tyrosine phosphorylation of S	9/660	86/18866	0.003105	0.040773	0.033031	7132/3717	9
BP	GO:0045445	myoblast differentiation	9/660	86/18866	0.003105	0.040773	0.033031	7040/6469	9
BP	GO:0050679	positive regulation of epithelial cell prolifera	16/660	211/18866	0.003152	0.041231	0.033402	8626/7099	16
BP	GO:0030183	B cell differentiation	12/660	137/18866	0.003159	0.041231	0.033402	678/6777/	12
BP	GO:0006979	response to oxidative stress	28/660	458/18866	0.003164	0.041231	0.033402	11179/733	28
BP	GO:1902903	regulation of supramolecular fiber organiza	24/660	373/18866	0.003199	0.04159	0.033693	54361/508	24
BP	GO:0001709	cell fate determination	6/660	42/18866	0.003214	0.041674	0.033762	5396/4208	6
BP	GO:0048661	positive regulation of smooth muscle cell p	10/660	103/18866	0.003256	0.042108	0.034113	7048/5159	10
BP	GO:0001659	temperature homeostasis	14/660	174/18866	0.00328	0.042276	0.034249	9658/2309	14
BP	GO:0002292	T cell differentiation involved in immune res	8/660	71/18866	0.003285	0.042276	0.034249	7292/6421	8

BP	GO:1901215	negative regulation of neuron death	16/660	212/18866	0.003303	0.042335	0.034297	5176/387/	16
BP	GO:0048638	regulation of developmental growth	23/660	353/18866	0.003306	0.042335	0.034297	80351/704	23
BP	GO:0018209	peptidyl-serine modification	22/660	333/18866	0.003402	0.043448	0.035198	65125/840	22
BP	GO:2000181	negative regulation of blood vessel morpho	15/660	194/18866	0.003484	0.044393	0.035964	54361/655	15
BP	GO:0061082	myeloid leukocyte cytokine production	5/660	30/18866	0.003558	0.045224	0.036637	7099/7040	5
BP	GO:0045747	positive regulation of Notch signaling path	7/660	57/18866	0.003598	0.045506	0.036866	8626/4846	7
BP	GO:0061005	cell differentiation involved in kidney devel	7/660	57/18866	0.003598	0.045506	0.036866	54361/646	7
BP	GO:0045773	positive regulation of axon extension	6/660	43/18866	0.003627	0.045652	0.036984	8482/9037	6
BP	GO:1901879	regulation of protein depolymerization	9/660	88/18866	0.003628	0.045652	0.036984	50853/670	9
BP	GO:1905475	regulation of protein localization to membr	15/660	195/18866	0.003656	0.045902	0.037187	10490/862	15
BP	GO:0050769	positive regulation of neurogenesis	29/660	485/18866	0.003696	0.046107	0.037352	6935/2290	29
BP	GO:0010811	positive regulation of cell-substrate adhesi	11/660	122/18866	0.0037	0.046107	0.037352	54361/623	11
BP	GO:0043244	regulation of protein-containing complex d	11/660	122/18866	0.0037	0.046107	0.037352	50853/670	11
BP	GO:0050729	positive regulation of inflammatory respons	13/660	158/18866	0.003738	0.046457	0.037636	10318/729	13
BP	GO:0032011	ARF protein signal transduction	4/660	19/18866	0.003782	0.046457	0.037636	23362/109	4
BP	GO:0032012	regulation of ARF protein signal transductio	4/660	19/18866	0.003782	0.046457	0.037636	23362/109	4
BP	GO:0051546	keratinocyte migration	4/660	19/18866	0.003782	0.046457	0.037636	10982/944	4
BP	GO:0060438	trachea development	4/660	19/18866	0.003782	0.046457	0.037636	7048/6469	4
BP	GO:0060973	cell migration involved in heart developme	4/660	19/18866	0.003782	0.046457	0.037636	6591/1051	4
BP	GO:1904018	positive regulation of vasculature developm	17/660	235/18866	0.003863	0.047332	0.038345	7048/9037	17
BP	GO:0007260	tyrosine phosphorylation of STAT protein	9/660	89/18866	0.003914	0.047728	0.038666	7132/3717	9
BP	GO:0055006	cardiac cell development	9/660	89/18866	0.003914	0.047728	0.038666	10611/515	9
BP	GO:0048675	axon extension	11/660	123/18866	0.003938	0.047916	0.038818	8408/8020	11
BP	GO:0051348	negative regulation of transferase activity	20/660	296/18866	0.003969	0.048173	0.039027	65125/724	20
BP	GO:2000379	positive regulation of reactive oxygen speci	10/660	106/18866	0.004006	0.048506	0.039296	7099/7048	10
BP	GO:0051897	positive regulation of protein kinase B signa	14/660	178/18866	0.004021	0.048579	0.039356	7040/9037	14
BP	GO:0090257	regulation of muscle system process	18/660	256/18866	0.004049	0.048748	0.039492	7132/387/	18
BP	GO:0042088	T-helper 1 type immune response	6/660	44/18866	0.004078	0.048748	0.039492	7292/7099	6
BP	GO:0085029	extracellular matrix assembly	6/660	44/18866	0.004078	0.048748	0.039492	7040/2898	6
BP	GO:2000404	regulation of T cell migration	6/660	44/18866	0.004078	0.048748	0.039492	65125/273	6
BP	GO:0060325	face morphogenesis	5/660	31/18866	0.004122	0.048748	0.039492	5156/5117	5
BP	GO:1905476	negative regulation of protein localization t	5/660	31/18866	0.004122	0.048748	0.039492	7040/2343	5
BP	GO:0002698	negative regulation of immune effector pro	11/660	124/18866	0.004189	0.048748	0.039492	7292/7040	11
BP	GO:0010633	negative regulation of epithelial cell migrat	11/660	124/18866	0.004189	0.048748	0.039492	7040/5176	11
BP	GO:0062197	cellular response to chemical stress	23/660	360/18866	0.004201	0.048748	0.039492	11179/709	23
BP	GO:1903036	positive regulation of response to woundin	8/660	74/18866	0.004249	0.048748	0.039492	7099/6239	8
BP	GO:0001865	NK T cell differentiation	3/660	10/18866	0.004254	0.048748	0.039492	7048/3702	3
BP	GO:0003149	membranous septum morphogenesis	3/660	10/18866	0.004254	0.048748	0.039492	7048/8321	3

BP	GO:0006307	DNA dealkylation involved in DNA repair	3/660	10/18866	0.004254	0.048748	0.039492	4350/7906	3
BP	GO:0031943	regulation of glucocorticoid metabolic proc	3/660	10/18866	0.004254	0.048748	0.039492	54361/956	3
BP	GO:0034651	cortisol biosynthetic process	3/660	10/18866	0.004254	0.048748	0.039492	54361/956	3
BP	GO:0045348	positive regulation of MHC class II biosynth	3/660	10/18866	0.004254	0.048748	0.039492	7099/3717	3
BP	GO:0045625	regulation of T-helper 1 cell differentiation	3/660	10/18866	0.004254	0.048748	0.039492	7292/3566	3
BP	GO:0048251	elastic fiber assembly	3/660	10/18866	0.004254	0.048748	0.039492	4239/4054	3
BP	GO:0060513	prostatic bud formation	3/660	10/18866	0.004254	0.048748	0.039492	8626/6469	3
BP	GO:0071104	response to interleukin-9	3/660	10/18866	0.004254	0.048748	0.039492	6777/6776	3
BP	GO:0071670	smooth muscle cell chemotaxis	3/660	10/18866	0.004254	0.048748	0.039492	5159/8031	3
BP	GO:1902645	tertiary alcohol biosynthetic process	3/660	10/18866	0.004254	0.048748	0.039492	54361/956	3
BP	GO:2000786	positive regulation of autophagosome asse	3/660	10/18866	0.004254	0.048748	0.039492	8408/5305	3
BP	GO:0055017	cardiac muscle tissue growth	10/660	107/18866	0.004284	0.048982	0.039682	7048/1061	10
BP	GO:0007568	aging	21/660	319/18866	0.004331	0.049404	0.040024	11179/862	21
BP	GO:0016050	vesicle organization	22/660	340/18866	0.004351	0.049528	0.040124	23325/104	22
BP	GO:0001819	positive regulation of cytokine production	27/660	447/18866	0.004363	0.049546	0.040138	7292/7099	27
BP	GO:0043030	regulation of macrophage activation	7/660	59/18866	0.004373	0.049555	0.040146	7099/2320	7
CC	GO:0062023	collagen-containing extracellular matrix	50/682	427/19559	5.90E-14	3.15E-11	2.71E-11	64856/644	50
CC	GO:0045121	membrane raft	34/682	329/19559	1.70E-08	3.27E-06	2.81E-06	7535/7132	34
CC	GO:0098857	membrane microdomain	34/682	330/19559	1.84E-08	3.27E-06	2.81E-06	7535/7132	34
CC	GO:0098589	membrane region	34/682	343/19559	4.77E-08	6.38E-06	5.49E-06	7535/7132	34
CC	GO:0005925	focal adhesion	37/682	415/19559	1.88E-07	2.02E-05	1.73E-05	7791/2613	37
CC	GO:0030055	cell-substrate junction	37/682	423/19559	3.03E-07	2.70E-05	2.32E-05	7791/2613	37
CC	GO:0005901	caveola	14/682	82/19559	8.44E-07	6.45E-05	5.55E-05	7048/7035	14
CC	GO:0044853	plasma membrane raft	16/682	113/19559	1.91E-06	0.000128	0.00011	7048/7035	16
CC	GO:0005788	endoplasmic reticulum lumen	28/682	308/19559	4.00E-06	0.000238	0.000205	54361/648	28
CC	GO:0031091	platelet alpha granule	13/682	91/19559	1.58E-05	0.000844	0.000726	10490/704	13
CC	GO:0005774	vacuolar membrane	32/682	427/19559	4.43E-05	0.002157	0.001855	10490/233	32
CC	GO:0031093	platelet alpha granule lumen	10/682	67/19559	0.000102	0.004569	0.003928	10490/704	10
CC	GO:0030017	sarcomere	19/682	207/19559	0.000122	0.004794	0.004122	23345/887	19
CC	GO:0005765	lysosomal membrane	28/682	375/19559	0.000139	0.004794	0.004122	10490/233	28
CC	GO:0098852	lytic vacuole membrane	28/682	375/19559	0.000139	0.004794	0.004122	10490/233	28
CC	GO:0030016	myofibril	20/682	227/19559	0.000143	0.004794	0.004122	23345/887	20
CC	GO:0009897	external side of plasma membrane	30/682	417/19559	0.000157	0.004947	0.004253	7099/7048	30
CC	GO:0042383	sarcolemma	14/682	135/19559	0.000268	0.007589	0.006525	8082/6641	14
CC	GO:0043292	contractile fiber	20/682	238/19559	0.00027	0.007589	0.006525	23345/887	20
CC	GO:0031252	cell leading edge	29/682	421/19559	0.000413	0.011052	0.009503	79971/745	29
CC	GO:0031941	filamentous actin	6/682	34/19559	0.001027	0.025799	0.022182	6525/5311	6
CC	GO:0031089	platelet dense granule lumen	4/682	14/19559	0.001109	0.025799	0.022182	7078/3699	4

CC	GO:0033643	host cell part	4/682	14/19559	0.001109	0.025799	0.022182	5297/5196	4
CC	GO:0031256	leading edge membrane	15/682	175/19559	0.00124	0.02764	0.023765	79971/555	15
CC	GO:0030139	endocytic vesicle	22/682	313/19559	0.00153	0.03198	0.027497	7791/5436	22
CC	GO:0001726	ruffle	15/682	179/19559	0.001554	0.03198	0.027497	7456/7099	15
CC	GO:0030018	Z disc	12/682	128/19559	0.001743	0.03454	0.029698	6261/4660	12
CC	GO:0005911	cell-cell junction	30/682	493/19559	0.002356	0.04373	0.0376	7791/7535	30
CC	GO:0001725	stress fiber	8/682	68/19559	0.002452	0.04373	0.0376	7791/9260	8
CC	GO:0097517	contractile actin filament bundle	8/682	68/19559	0.002452	0.04373	0.0376	7791/9260	8
CC	GO:0045177	apical part of cell	27/682	433/19559	0.002725	0.046653	0.040112	27347/655	27
CC	GO:0005884	actin filament	11/682	118/19559	0.00279	0.046653	0.040112	7456/6525	11
MF	GO:0005201	extracellular matrix structural constituent	24/649	169/18352	6.64E-09	5.39E-06	4.96E-06	64856/104	24
MF	GO:0005539	glycosaminoglycan binding	25/649	232/18352	7.80E-07	0.000317	0.000291	7048/5557	25
MF	GO:0004714	transmembrane receptor protein tyrosine k	12/649	61/18352	1.23E-06	0.000333	0.000306	5157/5159	12
MF	GO:0019199	transmembrane receptor protein kinase act	13/649	80/18352	4.28E-06	0.000868	0.000798	7048/5157	13
MF	GO:0004713	protein tyrosine kinase activity	17/649	135/18352	5.70E-06	0.000926	0.000851	7535/5157	17
MF	GO:0005520	insulin-like growth factor binding	7/649	29/18352	5.30E-05	0.007168	0.006589	3685/3490	7
MF	GO:0019838	growth factor binding	15/649	136/18352	9.51E-05	0.011029	0.010137	7048/5159	15
MF	GO:0005161	platelet-derived growth factor receptor bin	5/649	15/18352	0.000122	0.012357	0.011357	5159/5156	5
MF	GO:1901681	sulfur compound binding	22/649	262/18352	0.000166	0.013555	0.012459	5270/9037	22
MF	GO:0005518	collagen binding	10/649	70/18352	0.000167	0.013555	0.012459	5118/4060	10
MF	GO:0008083	growth factor activity	16/649	162/18352	0.000208	0.015329	0.014089	7040/8031	16
MF	GO:0043394	proteoglycan binding	7/649	36/18352	0.000228	0.015461	0.014211	9037/4035	7
MF	GO:0008081	phosphoric diester hydrolase activity	11/649	89/18352	0.000297	0.017377	0.015971	10924/661	11
MF	GO:0001968	fibronectin binding	6/649	27/18352	0.0003	0.017377	0.015971	131578/36	6
MF	GO:0097493	structural molecule activity conferring elast	4/649	11/18352	0.000419	0.022695	0.020859	3915/1051	4
MF	GO:0017154	semaphorin receptor activity	4/649	12/18352	0.000611	0.0292	0.026838	10154/536	4
MF	GO:0047555	3',5'-cyclic-GMP phosphodiesterase activity	4/649	12/18352	0.000611	0.0292	0.026838	5152/5137	4
MF	GO:0031994	insulin-like growth factor I binding	4/649	13/18352	0.000858	0.038725	0.035592	3685/3489	4
MF	GO:0008201	heparin binding	15/649	169/18352	0.000998	0.041712	0.038338	5270/6159	15
MF	GO:0030215	semaphorin receptor binding	5/649	23/18352	0.001079	0.041712	0.038338	8482/9037	5
MF	GO:0050431	transforming growth factor beta binding	5/649	23/18352	0.001079	0.041712	0.038338	7048/4054	5
MF	GO:0004857	enzyme inhibitor activity	26/649	383/18352	0.001208	0.043343	0.039837	65125/106	26
MF	GO:0048018	receptor ligand activity	31/649	487/18352	0.001228	0.043343	0.039837	54361/109	31
MF	GO:0030546	signaling receptor activator activity	31/649	492/18352	0.001443	0.048375	0.044462	54361/109	31
MF	GO:0019902	phosphatase binding	16/649	194/18352	0.001498	0.048375	0.044462	65125/724	16
MF	GO:0051371	muscle alpha-actinin binding	4/649	15/18352	0.001549	0.048375	0.044462	5311/9260	4

Supplementary Table 3. Kyoto encyclopedia of genes and genomes pathway enrichment analysis of all metastases-related genes in GSE11121								
ID	Description	GeneRatio	BgRatio	pvalue	p.adjust	qvalue	geneID	Count
hsa03050	Proteasome	15/423	46/8108	5.20E-09	1.60E-06	1.51E-06	SEM1/PSMD7/PSMD	15
hsa04110	Cell cycle	22/423	124/8108	3.51E-07	5.40E-05	5.09E-05	RBL1/RAD21/PTTG1/	22
hsa05012	Parkinson disease	32/423	249/8108	1.79E-06	0.000173	0.000163	VDAC3/UBB/TXN/SL	32
hsa05014	Amyotrophic lateral sclerosis	41/423	365/8108	2.24E-06	0.000173	0.000163	VAPB/TOMM40/SRS	41
hsa05010	Alzheimer disease	41/423	369/8108	2.96E-06	0.000183	0.000172	WNT10B/VDAC3/SL	41
hsa05020	Prion disease	33/423	273/8108	4.81E-06	0.000247	0.000233	VDAC3/SOD1/SLC25	33
hsa05022	Pathways of neurodegeneration - multiple diseases	48/423	476/8108	6.42E-06	0.000283	0.000267	WNT10B/VDAC3/VA	48
hsa05016	Huntington disease	35/423	306/8108	8.61E-06	0.000331	0.000313	VDAC3/TFAM/SOD1/	35
hsa05017	Spinocerebellar ataxia	19/423	143/8108	0.000149	0.004653	0.004389	VDAC3/SLC25A5/SE	19
hsa04932	Non-alcoholic fatty liver disease	20/423	155/8108	0.000151	0.004653	0.004389	SDHA/NDUFS6/NDU	20
hsa04610	Complement and coagulation cascades	15/359	85/8108	3.90E-06	0.001116	0.000875	TFPI/SERPING1/SERP	15
hsa04151	PI3K-Akt signaling pathway	34/359	354/8108	1.49E-05	0.002125	0.001666	TSC2/TLR4/TCL1A/P	34
hsa04510	Focal adhesion	23/359	201/8108	2.55E-05	0.002428	0.001903	ZYX/RHOA/PPP1R12	23
hsa05202	Transcriptional misregulation in cancer	22/359	192/8108	3.74E-05	0.002676	0.002098	ZEB1/TRAF1/TGFBR2	22
hsa04935	Growth hormone synthesis, secretion and action	16/359	119/8108	6.48E-05	0.003206	0.002514	STAT5B/STAT5A/PLC	16
hsa04659	Th17 cell differentiation	15/359	107/8108	6.73E-05	0.003206	0.002514	ZAP70/TGFBR2/TGFB	15
hsa05205	Proteoglycans in cancer	22/359	205/8108	0.000101	0.004132	0.003239	WNT4/TLR4/TIMP3/T	22
hsa04630	JAK-STAT signaling pathway	18/359	162/8108	0.00028	0.010003	0.007842	STAT5B/STAT5A/PIK	18
hsa05215	Prostate cancer	13/359	97/8108	0.000322	0.010245	0.008032	ZEB1/PLAT/PIK3R2/P	13
hsa05145	Toxoplasmosis	14/359	112/8108	0.0004	0.011432	0.008962	TNFRSF1A/TLR4/TGF	14
hsa05321	Inflammatory bowel disease	10/359	65/8108	0.000518	0.013475	0.010564	TLR4/TGFB1/RORC/	10
hsa05164	Influenza A	18/359	172/8108	0.000582	0.013875	0.010877	TPSB2/TPSAB1/TNFR	18
hsa01521	EGFR tyrosine kinase inhibitor resistance	11/359	79/8108	0.000663	0.01426	0.011179	PIK3R2/PIK3R1/PDG	11
hsa04658	Th1 and Th2 cell differentiation	12/359	92/8108	0.000698	0.01426	0.011179	ZAP70/STAT5B/STAT	12
hsa04360	Axon guidance	18/359	182/8108	0.001133	0.021593	0.016928	WNT4/SHH/SEMA7A	18
hsa04115	p53 signaling pathway	10/359	73/8108	0.001314	0.022661	0.017766	ZMAT3/TSC2/SESN1/	10
hsa04640	Hematopoietic cell lineage	12/359	99/8108	0.001347	0.022661	0.017766	MS4A1/ITGA5/IL4R/H	12
hsa05142	Chagas disease	12/359	102/8108	0.001748	0.027774	0.021773	TNFRSF1A/TLR4/TGF	12
hsa04020	Calcium signaling pathway	21/359	240/8108	0.002159	0.032341	0.025354	RYR1/PLN/PLCB2/PD	21
hsa05163	Human cytomegalovirus infection	20/359	225/8108	0.002272	0.032341	0.025354	TSC2/TNFRSF1A/TBK	20
hsa04015	Rap1 signaling pathway	19/359	210/8108	0.002375	0.032341	0.025354	RHOA/PLCB2/PIK3R2	19
hsa04371	Apelin signaling pathway	14/359	138/8108	0.0031	0.0403	0.031593	RYR1/PRKAB1/PLCB2	14

Supplementary Table 4. GO analysis of genes in the turquoise module									
ONTOLOG	ID	Description	GeneRatio	BgRatio	pvalue	p.adjust	qvalue	geneID	Count
BP	GO:0000070	mitotic sister chromatid segregation	34/545	161/18866	4.74E-20	2.11E-16	1.85E-16	11130/110	34
BP	GO:0140014	mitotic nuclear division	43/545	286/18866	4.91E-19	1.09E-15	9.57E-16	11130/110	43
BP	GO:0007059	chromosome segregation	46/545	334/18866	1.00E-18	1.49E-15	1.30E-15	11130/110	46
BP	GO:0000819	sister chromatid segregation	35/545	196/18866	3.94E-18	4.39E-15	3.84E-15	11130/110	35
BP	GO:0051983	regulation of chromosome segregation	26/545	107/18866	3.14E-17	2.80E-14	2.45E-14	11065/931	26
BP	GO:1902850	microtubule cytoskeleton organization involved in m	29/545	144/18866	1.07E-16	7.97E-14	6.98E-14	22974/104	29
BP	GO:0000280	nuclear division	49/545	428/18866	1.62E-16	1.03E-13	9.05E-14	11130/110	49
BP	GO:0010965	regulation of mitotic sister chromatid separation	20/545	61/18866	2.41E-16	1.34E-13	1.17E-13	11065/931	20
BP	GO:0010948	negative regulation of cell cycle process	44/545	359/18866	4.96E-16	2.46E-13	2.15E-13	9319/5490	44
BP	GO:0048285	organelle fission	51/545	476/18866	6.02E-16	2.46E-13	2.15E-13	11130/557	51
BP	GO:0033047	regulation of mitotic sister chromatid segregation	21/545	72/18866	6.35E-16	2.46E-13	2.15E-13	11065/931	21
BP	GO:0051306	mitotic sister chromatid separation	20/545	64/18866	6.99E-16	2.46E-13	2.15E-13	11065/931	20
BP	GO:0098813	nuclear chromosome segregation	38/545	272/18866	7.16E-16	2.46E-13	2.15E-13	11130/110	38
BP	GO:1901990	regulation of mitotic cell cycle phase transition	49/545	448/18866	1.02E-15	3.25E-13	2.85E-13	11065/931	49
BP	GO:1905818	regulation of chromosome separation	20/545	66/18866	1.37E-15	4.08E-13	3.57E-13	11065/931	20
BP	GO:0045930	negative regulation of mitotic cell cycle	41/545	341/18866	9.99E-15	2.78E-12	2.44E-12	11130/931	41
BP	GO:0033045	regulation of sister chromatid segregation	21/545	84/18866	1.95E-14	5.08E-12	4.44E-12	11065/931	21
BP	GO:0051304	chromosome separation	22/545	94/18866	2.05E-14	5.08E-12	4.44E-12	11065/931	22
BP	GO:0007091	metaphase/anaphase transition of mitotic cell cycle	18/545	58/18866	2.29E-14	5.38E-12	4.71E-12	11065/931	18
BP	GO:1901987	regulation of cell cycle phase transition	49/545	486/18866	2.50E-14	5.51E-12	4.82E-12	11065/931	49
BP	GO:0007088	regulation of mitotic nuclear division	28/545	163/18866	2.59E-14	5.51E-12	4.82E-12	11065/931	28
BP	GO:0033046	negative regulation of sister chromatid segregation	16/545	44/18866	3.73E-14	7.57E-12	6.62E-12	9319/5490	16
BP	GO:0044784	metaphase/anaphase transition of cell cycle	18/545	60/18866	4.47E-14	8.66E-12	7.58E-12	11065/931	18
BP	GO:0051985	negative regulation of chromosome segregation	16/545	45/18866	5.64E-14	1.05E-11	9.17E-12	9319/5490	16
BP	GO:1901991	negative regulation of mitotic cell cycle phase transiti	34/545	251/18866	6.28E-14	1.12E-11	9.79E-12	9319/5490	34
BP	GO:1901988	negative regulation of cell cycle phase transition	35/545	270/18866	1.01E-13	1.73E-11	1.52E-11	9319/5490	35
BP	GO:0051783	regulation of nuclear division	29/545	188/18866	1.58E-13	2.62E-11	2.29E-11	11065/931	29
BP	GO:0030071	regulation of mitotic metaphase/anaphase transition	17/545	56/18866	1.83E-13	2.92E-11	2.55E-11	11065/931	17
BP	GO:0033048	negative regulation of mitotic sister chromatid segreg	15/545	42/18866	3.24E-13	4.97E-11	4.35E-11	9319/5490	15
BP	GO:1902099	regulation of metaphase/anaphase transition of cell c	17/545	58/18866	3.50E-13	5.21E-11	4.56E-11	11065/931	17
BP	GO:0000086	G2/M transition of mitotic cell cycle	33/545	254/18866	4.89E-13	7.04E-11	6.16E-11	22974/687	33
BP	GO:0007051	spindle organization	28/545	183/18866	5.14E-13	7.16E-11	6.27E-11	22974/104	28
BP	GO:0044839	cell cycle G2/M phase transition	34/545	273/18866	7.37E-13	9.95E-11	8.71E-11	22974/687	34
BP	GO:2000816	negative regulation of mitotic sister chromatid separa	14/545	39/18866	1.86E-12	2.43E-10	2.13E-10	9319/5490	14
BP	GO:0031145	anaphase-promoting complex-dependent catabolic	19/545	83/18866	1.91E-12	2.43E-10	2.13E-10	11065/923	19
BP	GO:1905819	negative regulation of chromosome separation	14/545	40/18866	2.78E-12	3.44E-10	3.01E-10	9319/5490	14

BP	GO:0007052	mitotic spindle organization	22/545	118/18866	2.86E-12	3.45E-10	3.02E-10	22974/104	22
BP	GO:0010389	regulation of G2/M transition of mitotic cell cycle	28/545	200/18866	4.75E-12	5.58E-10	4.88E-10	22974/588	28
BP	GO:1902749	regulation of cell cycle G2/M phase transition	29/545	217/18866	6.40E-12	7.32E-10	6.40E-10	22974/588	29
BP	GO:0010639	negative regulation of organelle organization	41/545	416/18866	7.42E-12	8.27E-10	7.24E-10	9319/2297	41
BP	GO:0051784	negative regulation of nuclear division	16/545	60/18866	8.74E-12	9.51E-10	8.32E-10	9319/5490	16
BP	GO:0045839	negative regulation of mitotic nuclear division	15/545	52/18866	1.13E-11	1.19E-09	1.05E-09	9319/5490	15
BP	GO:0045841	negative regulation of mitotic metaphase/anaphase t	12/545	36/18866	1.99E-10	2.07E-08	1.81E-08	9319/5490	12
BP	GO:1902750	negative regulation of cell cycle G2/M phase transitic	19/545	108/18866	2.52E-10	2.55E-08	2.23E-08	5885/5713	19
BP	GO:0070507	regulation of microtubule cytoskeleton organization	25/545	190/18866	2.60E-10	2.57E-08	2.25E-08	22974/104	25
BP	GO:1902100	negative regulation of metaphase/anaphase transitio	12/545	37/18866	2.87E-10	2.78E-08	2.44E-08	9319/5490	12
BP	GO:0032886	regulation of microtubule-based process	27/545	240/18866	1.75E-09	1.64E-07	1.44E-07	22974/104	27
BP	GO:2001251	negative regulation of chromosome organization	21/545	148/18866	1.77E-09	1.64E-07	1.44E-07	9319/7153	21
BP	GO:0010972	negative regulation of G2/M transition of mitotic cell	17/545	96/18866	2.00E-09	1.82E-07	1.59E-07	5885/5713	17
BP	GO:0033044	regulation of chromosome organization	33/545	356/18866	4.13E-09	3.68E-07	3.22E-07	11065/931	33
BP	GO:0000075	cell cycle checkpoint	24/545	219/18866	2.35E-08	2.06E-06	1.80E-06	11130/931	24
BP	GO:0007094	mitotic spindle assembly checkpoint	10/545	34/18866	2.62E-08	2.12E-06	1.86E-06	9319/5490	10
BP	GO:0031577	spindle checkpoint	10/545	34/18866	2.62E-08	2.12E-06	1.86E-06	9319/5490	10
BP	GO:0071173	spindle assembly checkpoint	10/545	34/18866	2.62E-08	2.12E-06	1.86E-06	9319/5490	10
BP	GO:0071174	mitotic spindle checkpoint	10/545	34/18866	2.62E-08	2.12E-06	1.86E-06	9319/5490	10
BP	GO:0006260	DNA replication	27/545	273/18866	2.82E-08	2.25E-06	1.97E-06	6241/5984	27
BP	GO:0040001	establishment of mitotic spindle localization	10/545	35/18866	3.57E-08	2.79E-06	2.44E-06	54908/534	10
BP	GO:0007093	mitotic cell cycle checkpoint	20/545	166/18866	7.26E-08	5.58E-06	4.89E-06	11130/931	20
BP	GO:0006261	DNA-dependent DNA replication	19/545	151/18866	7.66E-08	5.79E-06	5.06E-06	5984/5983	19
BP	GO:0006270	DNA replication initiation	10/545	39/18866	1.11E-07	8.28E-06	7.25E-06	23594/499	10
BP	GO:0000910	cytokinesis	20/545	172/18866	1.31E-07	9.60E-06	8.40E-06	3925/1080	20
BP	GO:1902036	regulation of hematopoietic stem cell differentiation	13/545	73/18866	1.49E-07	1.07E-05	9.40E-06	5713/5708	13
BP	GO:0051225	spindle assembly	16/545	115/18866	2.02E-07	1.43E-05	1.25E-05	22974/106	16
BP	GO:0061418	regulation of transcription from RNA polymerase II p	13/545	77/18866	2.86E-07	1.97E-05	1.73E-05	5713/5708	13
BP	GO:2000736	regulation of stem cell differentiation	16/545	118/18866	2.90E-07	1.97E-05	1.73E-05	5713/5708	16
BP	GO:0000082	G1/S transition of mitotic cell cycle	26/545	287/18866	2.92E-07	1.97E-05	1.73E-05	7298/6241	26
BP	GO:0008608	attachment of spindle microtubules to kinetochore	9/545	34/18866	3.61E-07	2.40E-05	2.10E-05	10615/291	9
BP	GO:0051293	establishment of spindle localization	10/545	45/18866	4.79E-07	3.14E-05	2.75E-05	54908/534	10
BP	GO:0071103	DNA conformation change	29/545	355/18866	5.40E-07	3.49E-05	3.05E-05	7153/1104	29
BP	GO:1903046	meiotic cell cycle process	20/545	193/18866	8.54E-07	5.44E-05	4.76E-05	9319/7153	20
BP	GO:0048002	antigen processing and presentation of peptide antic	20/545	194/18866	9.27E-07	5.80E-05	5.07E-05	29127/571	20
BP	GO:0002478	antigen processing and presentation of exogenous p	19/545	177/18866	9.36E-07	5.80E-05	5.07E-05	29127/571	19
BP	GO:1901532	regulation of hematopoietic progenitor cell differenti	13/545	86/18866	1.07E-06	6.50E-05	5.69E-05	5713/5708	13
BP	GO:0061640	cytoskeleton-dependent cytokinesis	14/545	100/18866	1.08E-06	6.50E-05	5.69E-05	3925/1080	14

BP	GO:0019882	antigen processing and presentation	22/545	232/18866	1.11E-06	6.62E-05	5.79E-05	54915/291	22
BP	GO:0060218	hematopoietic stem cell differentiation	13/545	87/18866	1.23E-06	7.19E-05	6.29E-05	5713/5708	13
BP	GO:0044843	cell cycle G1/S phase transition	26/545	310/18866	1.28E-06	7.39E-05	6.47E-05	7298/6241	26
BP	GO:0051653	spindle localization	10/545	50/18866	1.35E-06	7.74E-05	6.77E-05	54908/534	10
BP	GO:0000132	establishment of mitotic spindle orientation	8/545	30/18866	1.54E-06	8.68E-05	7.60E-05	54908/534	8
BP	GO:0045787	positive regulation of cell cycle	30/545	396/18866	1.67E-06	9.19E-05	8.04E-05	11065/106	30
BP	GO:0006521	regulation of cellular amino acid metabolic process	11/545	63/18866	1.67E-06	9.19E-05	8.04E-05	5713/5708	11
BP	GO:0019884	antigen processing and presentation of exogenous a	19/545	185/18866	1.83E-06	9.96E-05	8.71E-05	29127/571	19
BP	GO:0010499	proteasomal ubiquitin-independent protein catabolic	7/545	22/18866	1.89E-06	0.000101	8.88E-05	5695/5691	7
BP	GO:0043488	regulation of mRNA stability	19/545	186/18866	1.99E-06	0.000105	9.22E-05	54915/110	19
BP	GO:0051302	regulation of cell division	18/545	171/18866	2.40E-06	0.000126	0.00011	5933/2912	18
BP	GO:0090068	positive regulation of cell cycle process	25/545	302/18866	2.58E-06	0.000134	0.000117	11065/106	25
BP	GO:0071456	cellular response to hypoxia	20/545	208/18866	2.76E-06	0.000141	0.000124	57103/571	20
BP	GO:1903311	regulation of mRNA metabolic process	27/545	344/18866	2.85E-06	0.000144	0.000126	54915/110	27
BP	GO:0043618	regulation of transcription from RNA polymerase II p	14/545	109/18866	3.09E-06	0.000155	0.000135	5713/5708	14
BP	GO:0033238	regulation of cellular amine metabolic process	12/545	81/18866	3.45E-06	0.000171	0.00015	55737/571	12
BP	GO:0140013	meiotic nuclear division	18/545	177/18866	3.92E-06	0.000192	0.000168	9319/7153	18
BP	GO:0043487	regulation of RNA stability	19/545	195/18866	4.01E-06	0.000194	0.00017	54915/110	19
BP	GO:0051321	meiotic cell cycle	22/545	254/18866	4.97E-06	0.000237	0.000208	9319/7153	22
BP	GO:0071453	cellular response to oxygen levels	21/545	235/18866	5.00E-06	0.000237	0.000208	57103/571	21
BP	GO:0036294	cellular response to decreased oxygen levels	20/545	218/18866	5.64E-06	0.000265	0.000232	57103/571	20
BP	GO:0034502	protein localization to chromosome	12/545	85/18866	5.78E-06	0.000268	0.000235	54908/588	12
BP	GO:0043620	regulation of DNA-templated transcription in respon	14/545	115/18866	5.85E-06	0.000269	0.000235	5713/5708	14
BP	GO:0000281	mitotic cytokinesis	11/545	72/18866	6.48E-06	0.000295	0.000258	3925/2912	11
BP	GO:0051294	establishment of spindle orientation	8/545	36/18866	6.83E-06	0.000307	0.000269	54908/534	8
BP	GO:0042770	signal transduction in response to DNA damage	15/545	133/18866	7.09E-06	0.000316	0.000277	9100/5695	15
BP	GO:0070498	interleukin-1-mediated signaling pathway	13/545	103/18866	8.41E-06	0.000371	0.000325	7444/8767	13
BP	GO:0051310	metaphase plate congression	10/545	61/18866	8.94E-06	0.000391	0.000342	54908/106	10
BP	GO:0002479	antigen processing and presentation of exogenous p	11/545	75/18866	9.72E-06	0.000421	0.000368	5713/5708	11
BP	GO:0061013	regulation of mRNA catabolic process	19/545	208/18866	1.02E-05	0.000439	0.000384	54915/110	19
BP	GO:0032465	regulation of cytokinesis	12/545	90/18866	1.06E-05	0.000446	0.00039	29127/905	12
BP	GO:0002244	hematopoietic progenitor cell differentiation	17/545	172/18866	1.06E-05	0.000446	0.00039	7153/5713	17
BP	GO:0072331	signal transduction by p53 class mediator	22/545	267/18866	1.10E-05	0.000459	0.000402	9100/2297	22
BP	GO:0000727	double-strand break repair via break-induced replica	5/545	12/18866	1.32E-05	0.000546	0.000478	4175/4174	5
BP	GO:0001736	establishment of planar polarity	14/545	124/18866	1.41E-05	0.000566	0.000495	5713/5708	14
BP	GO:0007164	establishment of tissue polarity	14/545	124/18866	1.41E-05	0.000566	0.000495	5713/5708	14
BP	GO:0061982	meiosis I cell cycle process	14/545	124/18866	1.41E-05	0.000566	0.000495	9319/7153	14
BP	GO:0030010	establishment of cell polarity	15/545	141/18866	1.45E-05	0.000576	0.000504	54908/235	15

BP	GO:0090307	mitotic spindle assembly	10/545	65/18866	1.60E-05	0.000626	0.000548	22974/291	10
BP	GO:0050000	chromosome localization	11/545	79/18866	1.62E-05	0.000626	0.000548	54908/106	11
BP	GO:0051303	establishment of chromosome localization	11/545	79/18866	1.62E-05	0.000626	0.000548	54908/106	11
BP	GO:0043161	proteasome-mediated ubiquitin-dependent protein	29/545	424/18866	1.77E-05	0.00068	0.000595	11060/110	29
BP	GO:0042590	antigen processing and presentation of exogenous p	11/545	80/18866	1.82E-05	0.000695	0.000608	5713/5708	11
BP	GO:1901796	regulation of signal transduction by p53 class mediat	17/545	180/18866	1.93E-05	0.000729	0.000638	22974/687	17
BP	GO:0001738	morphogenesis of a polarized epithelium	15/545	145/18866	2.02E-05	0.000752	0.000658	23513/571	15
BP	GO:0000209	protein polyubiquitination	25/545	340/18866	2.02E-05	0.000752	0.000658	11060/733	25
BP	GO:0051988	regulation of attachment of spindle microtubules to k	5/545	13/18866	2.10E-05	0.000773	0.000676	10615/291	5
BP	GO:0044106	cellular amine metabolic process	14/545	131/18866	2.64E-05	0.000963	0.000843	55737/571	14
BP	GO:0051261	protein depolymerization	13/545	115/18866	2.80E-05	0.001016	0.000889	22974/392	13
BP	GO:0071459	protein localization to chromosome, centromeric reg	6/545	22/18866	2.84E-05	0.001022	0.000894	54908/104	6
BP	GO:0034508	centromere complex assembly	9/545	56/18866	2.96E-05	0.001057	0.000925	54069/553	9
BP	GO:0045931	positive regulation of mitotic cell cycle	16/545	168/18866	3.04E-05	0.001076	0.000941	11065/590	16
BP	GO:0032392	DNA geometric change	13/545	116/18866	3.07E-05	0.001079	0.000944	7153/5984	13
BP	GO:0031109	microtubule polymerization or depolymerization	13/545	117/18866	3.37E-05	0.001173	0.001027	22974/392	13
BP	GO:0006304	DNA modification	13/545	120/18866	4.41E-05	0.001523	0.001333	8493/4609	13
BP	GO:0007019	microtubule depolymerization	8/545	46/18866	4.57E-05	0.001566	0.001371	22974/392	8
BP	GO:0051315	attachment of mitotic spindle microtubules to kineto	5/545	15/18866	4.66E-05	0.001587	0.001389	10403/106	5
BP	GO:0001666	response to hypoxia	25/545	359/18866	4.97E-05	0.00168	0.00147	57103/571	25
BP	GO:0007080	mitotic metaphase plate congression	8/545	47/18866	5.37E-05	0.001789	0.001565	54908/104	8
BP	GO:0000083	regulation of transcription involved in G1/S transition	7/545	35/18866	5.38E-05	0.001789	0.001565	7298/6241	7
BP	GO:0009308	amine metabolic process	14/545	140/18866	5.53E-05	0.001827	0.001599	55737/571	14
BP	GO:0006310	DNA recombination	22/545	299/18866	6.23E-05	0.002042	0.001787	9319/7153	22
BP	GO:0051656	establishment of organelle localization	28/545	432/18866	6.36E-05	0.002071	0.001812	83696/549	28
BP	GO:0060071	Wnt signaling pathway, planar cell polarity pathway	12/545	108/18866	6.71E-05	0.002169	0.001898	5713/5708	12
BP	GO:0007163	establishment or maintenance of cell polarity	18/545	220/18866	7.50E-05	0.002395	0.002096	54908/235	18
BP	GO:0010212	response to ionizing radiation	14/545	144/18866	7.53E-05	0.002395	0.002096	57103/843	14
BP	GO:0031146	SCF-dependent proteasomal ubiquitin-dependent p	11/545	93/18866	7.58E-05	0.002395	0.002096	5713/5708	11
BP	GO:0090175	regulation of establishment of planar polarity	12/545	110/18866	8.04E-05	0.002497	0.002185	5713/5708	12
BP	GO:0006302	double-strand break repair	20/545	262/18866	8.04E-05	0.002497	0.002185	7336/9319	20
BP	GO:0016579	protein deubiquitination	21/545	283/18866	8.06E-05	0.002497	0.002185	9100/6472	21
BP	GO:0036293	response to decreased oxygen levels	25/545	371/18866	8.44E-05	0.002595	0.002271	57103/571	25
BP	GO:0048863	stem cell differentiation	20/545	264/18866	8.93E-05	0.002727	0.002386	6949/6664	20
BP	GO:0090224	regulation of spindle organization	7/545	38/18866	9.36E-05	0.002822	0.002469	22974/104	7
BP	GO:0070482	response to oxygen levels	26/545	396/18866	9.37E-05	0.002822	0.002469	57103/571	26
BP	GO:0044786	cell cycle DNA replication	9/545	65/18866	9.93E-05	0.002972	0.0026	5984/5983	9
BP	GO:2001020	regulation of response to DNA damage stimulus	18/545	226/18866	0.000106	0.003144	0.002752	7336/5710	18

BP	GO:0009314	response to radiation	28/545	447/18866	0.000114	0.003368	0.002947	7295/5710	28
BP	GO:0090263	positive regulation of canonical Wnt signaling pathwa	14/545	150/18866	0.000117	0.003433	0.003004	55737/571	14
BP	GO:0034501	protein localization to kinetochore	5/545	18/18866	0.000124	0.003609	0.003158	54908/104	5
BP	GO:0010565	regulation of cellular ketone metabolic process	16/545	189/18866	0.000125	0.003617	0.003165	5713/5708	16
BP	GO:0002474	antigen processing and presentation of peptide antig	11/545	99/18866	0.000134	0.003856	0.003374	5713/5708	11
BP	GO:0006520	cellular amino acid metabolic process	23/545	340/18866	0.000152	0.004335	0.003793	6472/5713	23
BP	GO:0072528	pyrimidine-containing compound biosynthetic proce	7/545	41/18866	0.000155	0.004394	0.003845	54963/729	7
BP	GO:0007127	meiosis I	12/545	119/18866	0.000172	0.00484	0.004235	9319/7153	12
BP	GO:0010498	proteasomal protein catabolic process	29/545	483/18866	0.000179	0.005023	0.004395	11060/110	29
BP	GO:0070646	protein modification by small protein removal	21/545	300/18866	0.000182	0.00508	0.004445	9100/6472	21
BP	GO:0051262	protein tetramerization	10/545	87/18866	0.000203	0.005614	0.004913	7083/6472	10
BP	GO:0071824	protein-DNA complex subunit organization	20/545	282/18866	0.000217	0.005972	0.005226	6882/4609	20
BP	GO:0045840	positive regulation of mitotic nuclear division	8/545	57/18866	0.000219	0.00599	0.005242	11065/590	8
BP	GO:0006323	DNA packaging	18/545	240/18866	0.000224	0.006041	0.005286	7153/1104	18
BP	GO:0006221	pyrimidine nucleotide biosynthetic process	6/545	31/18866	0.000225	0.006041	0.005286	54963/729	6
BP	GO:0007143	female meiotic nuclear division	6/545	31/18866	0.000225	0.006041	0.005286	9319/7153	6
BP	GO:0000723	telomere maintenance	14/545	161/18866	0.000247	0.00655	0.005731	55183/598	14
BP	GO:0031570	DNA integrity checkpoint	14/545	161/18866	0.000247	0.00655	0.005731	7153/5810	14
BP	GO:0065004	protein-DNA complex assembly	18/545	243/18866	0.000261	0.006894	0.006033	6882/5406	18
BP	GO:0006913	nucleocytoplasmic transport	23/545	354/18866	0.000272	0.007138	0.006246	7307/7295	23
BP	GO:0032508	DNA duplex unwinding	11/545	108/18866	0.000291	0.007579	0.006632	7153/5984	11
BP	GO:0051169	nuclear transport	23/545	357/18866	0.000307	0.007956	0.006962	7307/7295	23
BP	GO:0051984	positive regulation of chromosome segregation	6/545	33/18866	0.000322	0.008302	0.007265	5885/9700	6
BP	GO:0006220	pyrimidine nucleotide metabolic process	7/545	46/18866	0.000325	0.008334	0.007293	54963/729	7
BP	GO:0009896	positive regulation of catabolic process	27/545	454/18866	0.000345	0.008763	0.007668	54915/110	27
BP	GO:0051383	kinetochore organization	5/545	22/18866	0.000346	0.008763	0.007668	10403/978	5
BP	GO:0045132	meiotic chromosome segregation	10/545	93/18866	0.000351	0.00884	0.007736	9319/7153	10
BP	GO:0046785	microtubule polymerization	9/545	77/18866	0.000369	0.009244	0.008089	22974/392	9
BP	GO:0031055	chromatin remodeling at centromere	7/545	47/18866	0.000373	0.009285	0.008125	54069/553	7
BP	GO:0060236	regulation of mitotic spindle organization	6/545	34/18866	0.000382	0.009455	0.008274	22974/104	6
BP	GO:0002223	stimulatory C-type lectin receptor signaling pathway	11/545	113/18866	0.000431	0.010604	0.009279	5713/5708	11
BP	GO:0070555	response to interleukin-1	16/545	211/18866	0.000437	0.010693	0.009357	7444/9100	16
BP	GO:0009262	deoxyribonucleotide metabolic process	6/545	35/18866	0.00045	0.010954	0.009585	7298/6241	6
BP	GO:1904666	regulation of ubiquitin protein ligase activity	5/545	24/18866	0.000532	0.012784	0.011187	11065/534	5
BP	GO:0044728	DNA methylation or demethylation	10/545	98/18866	0.000535	0.012784	0.011187	8493/4609	10
BP	GO:0048732	gland development	26/545	443/18866	0.000538	0.012784	0.011187	7298/2351	26
BP	GO:0002220	innate immune response activating cell surface recep	11/545	116/18866	0.000539	0.012784	0.011187	5713/5708	11
BP	GO:0043242	negative regulation of protein-containing complex d	9/545	81/18866	0.000539	0.012784	0.011187	22974/971	9

BP	GO:0032200	telomere organization	14/545	174/18866	0.000544	0.012843	0.011239	55183/598	14
BP	GO:0002758	innate immune response-activating signal transducti	11/545	117/18866	0.000579	0.013597	0.011898	5713/5708	11
BP	GO:0042180	cellular ketone metabolic process	18/545	260/18866	0.000588	0.013714	0.012001	5713/5708	18
BP	GO:1902904	negative regulation of supramolecular fiber organiza	13/545	156/18866	0.000611	0.01419	0.012417	22974/916	13
BP	GO:0006338	chromatin remodeling	16/545	218/18866	0.000624	0.014409	0.012609	7019/4609	16
BP	GO:0032984	protein-containing complex disassembly	21/545	330/18866	0.000649	0.014855	0.012999	22974/392	21
BP	GO:0036010	protein localization to endosome	5/545	25/18866	0.00065	0.014855	0.012999	55737/235	5
BP	GO:0000724	double-strand break repair via homologous recombi	12/545	138/18866	0.000672	0.015281	0.013372	55183/940	12
BP	GO:0051054	positive regulation of DNA metabolic process	15/545	200/18866	0.000731	0.01654	0.014474	57103/551	15
BP	GO:0000079	regulation of cyclin-dependent protein serine/threon	10/545	102/18866	0.000735	0.016541	0.014475	5347/2356	10
BP	GO:0000725	recombinational repair	12/545	140/18866	0.000764	0.017047	0.014917	55183/940	12
BP	GO:0031110	regulation of microtubule polymerization or depolym	9/545	85/18866	0.000769	0.017047	0.014917	22974/392	9
BP	GO:0072527	pyrimidine-containing compound metabolic process	9/545	85/18866	0.000769	0.017047	0.014917	54963/729	9
BP	GO:0006336	DNA replication-independent nucleosome assembly	7/545	53/18866	0.000787	0.017373	0.015203	54069/553	7
BP	GO:1905330	regulation of morphogenesis of an epithelium	14/545	181/18866	0.000805	0.017668	0.015461	5713/5708	14
BP	GO:0031111	negative regulation of microtubule polymerization or	6/545	39/18866	0.00082	0.017914	0.015676	22974/392	6
BP	GO:0043244	regulation of protein-containing complex disassemb	11/545	122/18866	0.000824	0.017916	0.015678	22974/971	11
BP	GO:0043624	cellular protein complex disassembly	16/545	224/18866	0.000835	0.018077	0.015819	22974/392	16
BP	GO:0045732	positive regulation of protein catabolic process	16/545	225/18866	0.000876	0.018864	0.016507	11060/557	16
BP	GO:0034724	DNA replication-independent nucleosome organizat	7/545	54/18866	0.000882	0.01891	0.016548	54069/553	7
BP	GO:0030177	positive regulation of Wnt signaling pathway	14/545	183/18866	0.000896	0.019015	0.016639	55737/571	14
BP	GO:0071347	cellular response to interleukin-1	14/545	183/18866	0.000896	0.019015	0.016639	7444/9100	14
BP	GO:0051494	negative regulation of cytoskeleton organization	13/545	163/18866	0.000922	0.019437	0.017009	22974/916	13
BP	GO:0015949	nucleobase-containing small molecule interconversio	5/545	27/18866	0.000941	0.019437	0.017009	7298/6241	5
BP	GO:0090090	negative regulation of canonical Wnt signaling pathw	14/545	184/18866	0.000944	0.019437	0.017009	5713/5708	14
BP	GO:0006268	DNA unwinding involved in DNA replication	4/545	16/18866	0.00095	0.019437	0.017009	9401/4175	4
BP	GO:0007076	mitotic chromosome condensation	4/545	16/18866	0.00095	0.019437	0.017009	11044/512	4
BP	GO:0009263	deoxyribonucleotide biosynthetic process	4/545	16/18866	0.00095	0.019437	0.017009	7298/6241	4
BP	GO:0060576	intestinal epithelial cell development	4/545	16/18866	0.00095	0.019437	0.017009	7298/5710	4
BP	GO:0099116	tRNA 5'-end processing	4/545	16/18866	0.00095	0.019437	0.017009	6741/1079	4
BP	GO:0002218	activation of innate immune response	12/545	144/18866	0.000979	0.019737	0.017271	5713/5708	12
BP	GO:0014068	positive regulation of phosphatidylinositol 3-kinase s	9/545	88/18866	0.000988	0.019737	0.017271	5747/5467	9
BP	GO:1901879	regulation of protein depolymerization	9/545	88/18866	0.000988	0.019737	0.017271	22974/265	9
BP	GO:0051785	positive regulation of nuclear division	8/545	71/18866	0.000991	0.019737	0.017271	11065/590	8
BP	GO:1901880	negative regulation of protein depolymerization	8/545	71/18866	0.000991	0.019737	0.017271	22974/265	8
BP	GO:1904029	regulation of cyclin-dependent protein kinase activity	10/545	106/18866	0.000992	0.019737	0.017271	5347/2356	10
BP	GO:0051052	regulation of DNA metabolic process	22/545	365/18866	0.000998	0.019764	0.017295	7644/7336	22
BP	GO:0008637	apoptotic mitochondrial changes	11/545	125/18866	0.001008	0.019878	0.017395	55737/292	11

BP	GO:0071168	protein localization to chromatin	5/545	28/18866	0.001119	0.021979	0.019233	5885/5347	5
BP	GO:0014066	regulation of phosphatidylinositol 3-kinase signaling	11/545	127/18866	0.001148	0.022449	0.019645	5747/5467	11
BP	GO:0043044	ATP-dependent chromatin remodeling	9/545	90/18866	0.001161	0.022608	0.019784	54069/553	9
BP	GO:2000027	regulation of animal organ morphogenesis	17/545	254/18866	0.001198	0.023213	0.020313	5713/5708	17
BP	GO:0006305	DNA alkylation	8/545	74/18866	0.001302	0.025026	0.021899	8493/4609	8
BP	GO:0006306	DNA methylation	8/545	74/18866	0.001302	0.025026	0.021899	8493/4609	8
BP	GO:0042176	regulation of protein catabolic process	23/545	397/18866	0.001309	0.025041	0.021912	11060/557	23
BP	GO:1901989	positive regulation of cell cycle phase transition	10/545	110/18866	0.001318	0.025113	0.021976	11065/262	10
BP	GO:0045089	positive regulation of innate immune response	15/545	213/18866	0.001383	0.026138	0.022873	5713/5708	15
BP	GO:0034080	CENP-A containing nucleosome assembly	6/545	43/18866	0.00139	0.026138	0.022873	54069/553	6
BP	GO:0061641	CENP-A containing chromatin organization	6/545	43/18866	0.00139	0.026138	0.022873	54069/553	6
BP	GO:0051170	import into nucleus	13/545	171/18866	0.001429	0.026766	0.023422	6637/1020	13
BP	GO:0035567	non-canonical Wnt signaling pathway	12/545	151/18866	0.001476	0.027535	0.024095	5713/5708	12
BP	GO:0051289	protein homotetramerization	7/545	59/18866	0.001502	0.027893	0.024408	7083/6472	7
BP	GO:0051782	negative regulation of cell division	4/545	18/18866	0.001526	0.028236	0.024709	23594/460	4
BP	GO:0051642	centrosome localization	5/545	30/18866	0.001548	0.028508	0.024946	4085/2989	5
BP	GO:0009124	nucleoside monophosphate biosynthetic process	6/545	44/18866	0.00157	0.028809	0.02521	54963/729	6
BP	GO:0033209	tumor necrosis factor-mediated signaling pathway	13/545	173/18866	0.001587	0.028991	0.025369	5713/5708	13
BP	GO:0033260	nuclear DNA replication	7/545	60/18866	0.001658	0.030177	0.026406	5984/5983	7
BP	GO:0097711	ciliary basal body-plasma membrane docking	9/545	95/18866	0.001702	0.030848	0.026994	10733/534	9
BP	GO:0071897	DNA biosynthetic process	14/545	196/18866	0.001726	0.031157	0.027264	9100/7298	14
BP	GO:0062012	regulation of small molecule metabolic process	25/545	456/18866	0.001766	0.031742	0.027776	57103/111	25
BP	GO:0009394	2'-deoxyribonucleotide metabolic process	5/545	31/18866	0.001802	0.03211	0.028098	7298/4521	5
BP	GO:0061842	microtubule organizing center localization	5/545	31/18866	0.001802	0.03211	0.028098	4085/2989	5
BP	GO:0022613	ribonucleoprotein complex biogenesis	26/545	482/18866	0.001808	0.03211	0.028098	96764/663	26
BP	GO:0030178	negative regulation of Wnt signaling pathway	15/545	219/18866	0.001818	0.032164	0.028145	5713/5708	15
BP	GO:0006271	DNA strand elongation involved in DNA replication	4/545	19/18866	0.00189	0.033037	0.02891	5984/5983	4
BP	GO:0033189	response to vitamin A	4/545	19/18866	0.00189	0.033037	0.02891	7298/5467	4
BP	GO:0046653	tetrahydrofolate metabolic process	4/545	19/18866	0.00189	0.033037	0.02891	7298/6472	4
BP	GO:0002831	regulation of response to biotic stimulus	23/545	409/18866	0.001923	0.033489	0.029305	57103/876	23
BP	GO:0031023	microtubule organizing center organization	11/545	136/18866	0.001994	0.034591	0.03027	6491/5902	11
BP	GO:0045143	homologous chromosome segregation	7/545	62/18866	0.00201	0.034731	0.030392	9319/5885	7
BP	GO:0008380	RNA splicing	26/545	487/18866	0.002084	0.035622	0.031172	7307/9676	26
BP	GO:0017038	protein import	14/545	200/18866	0.002084	0.035622	0.031172	10452/914	14
BP	GO:0097421	liver regeneration	5/545	32/18866	0.002086	0.035622	0.031172	7298/5781	5
BP	GO:0007062	sister chromatid cohesion	7/545	63/18866	0.002206	0.037537	0.032848	11044/588	7
BP	GO:0000377	RNA splicing, via transesterification reactions with bu	22/545	390/18866	0.002299	0.038674	0.033842	7307/9676	22
BP	GO:0000398	mRNA splicing, via spliceosome	22/545	390/18866	0.002299	0.038674	0.033842	7307/9676	22

BP	GO:0031331	positive regulation of cellular catabolic process	22/545	390/18866	0.002299	0.038674	0.033842	54915/557	22
BP	GO:0032495	response to muramyl dipeptide	4/545	20/18866	0.002309	0.038694	0.033859	8767/1432	4
BP	GO:0019692	deoxyribose phosphate metabolic process	5/545	33/18866	0.0024	0.040079	0.035072	7298/4521	5
BP	GO:0002066	columnar/cuboidal epithelial cell development	7/545	64/18866	0.002417	0.040199	0.035177	7298/5710	7
BP	GO:0009113	purine nucleobase biosynthetic process	3/545	10/18866	0.002472	0.040666	0.035586	6472/5631	3
BP	GO:0035999	tetrahydrofolate interconversion	3/545	10/18866	0.002472	0.040666	0.035586	7298/6472	3
BP	GO:0051231	spindle elongation	3/545	10/18866	0.002472	0.040666	0.035586	29127/905	3
BP	GO:2001252	positive regulation of chromosome organization	13/545	182/18866	0.002486	0.040752	0.035661	55183/588	13
BP	GO:0000375	RNA splicing, via transesterification reactions	22/545	393/18866	0.002524	0.041224	0.036074	7307/9676	22
BP	GO:0001889	liver development	11/545	141/18866	0.002649	0.0431	0.037715	7298/5469	11
BP	GO:0002065	columnar/cuboidal epithelial cell differentiation	10/545	121/18866	0.002686	0.043547	0.038106	7298/5710	10
BP	GO:0002833	positive regulation of response to biotic stimulus	16/545	251/18866	0.002697	0.043568	0.038125	8767/5713	16
BP	GO:2001021	negative regulation of response to DNA damage stim	8/545	83/18866	0.002721	0.043789	0.038318	55183/262	8
BP	GO:0050852	T cell receptor signaling pathway	14/545	206/18866	0.002734	0.043841	0.038364	8767/5713	14
BP	GO:0042558	pteridine-containing compound metabolic process	5/545	34/18866	0.002748	0.043916	0.03843	7298/6472	5
BP	GO:0034471	ncRNA 5'-end processing	4/545	21/18866	0.002788	0.044382	0.038838	6741/1079	4
BP	GO:1903706	regulation of hemopoiesis	26/545	498/18866	0.002819	0.044729	0.039141	9092/8767	26
BP	GO:0002495	antigen processing and presentation of peptide antig	9/545	103/18866	0.00297	0.046957	0.04109	29127/379	9
BP	GO:0061008	hepaticobiliary system development	11/545	144/18866	0.003119	0.048985	0.042865	7298/5469	11
BP	GO:0034470	ncRNA processing	22/545	400/18866	0.003125	0.048985	0.042865	11044/674	22
BP	GO:0010259	multicellular organism aging	5/545	35/18866	0.003132	0.048985	0.042865	8438/4726	5
BP	GO:0002504	antigen processing and presentation of peptide or po	9/545	104/18866	0.003171	0.049423	0.043249	29127/379	9
BP	GO:0006402	mRNA catabolic process	21/545	376/18866	0.003213	0.049915	0.043679	54915/110	21
CC	GO:0005819	spindle	52/559	367/19559	8.57E-22	4.85E-19	4.17E-19	55689/229	52
CC	GO:0098687	chromosomal region	48/559	350/19559	1.38E-19	3.90E-17	3.35E-17	11130/674	48
CC	GO:0000775	chromosome, centromeric region	33/559	196/19559	1.70E-16	2.67E-14	2.29E-14	11130/549	33
CC	GO:0000776	kinetochore	28/559	137/19559	1.89E-16	2.67E-14	2.29E-14	11130/549	28
CC	GO:0000779	condensed chromosome, centromeric region	26/559	122/19559	7.77E-16	8.79E-14	7.56E-14	11130/549	26
CC	GO:0072686	mitotic spindle	26/559	133/19559	7.06E-15	6.66E-13	5.72E-13	55689/229	26
CC	GO:0000777	condensed chromosome kinetochore	22/559	106/19559	2.36E-13	1.91E-11	1.64E-11	11130/549	22
CC	GO:0000793	condensed chromosome	31/559	222/19559	2.77E-13	1.96E-11	1.68E-11	11130/715	31
CC	GO:0000922	spindle pole	26/559	165/19559	1.40E-12	8.80E-11	7.56E-11	22974/104	26
CC	GO:0051233	spindle midzone	12/559	36/19559	1.77E-10	1.00E-08	8.60E-09	55183/291	12
CC	GO:0005876	spindle microtubule	13/559	66/19559	3.77E-08	1.94E-06	1.67E-06	220134/90	13
CC	GO:0030496	midbody	21/559	182/19559	6.15E-08	2.90E-06	2.49E-06	10615/798	21
CC	GO:0000502	proteasome complex	12/559	63/19559	1.87E-07	8.13E-06	6.98E-06	5713/5708	12
CC	GO:0005874	microtubule	33/559	431/19559	3.15E-07	1.27E-05	1.09E-05	80086/229	33
CC	GO:1905369	endopeptidase complex	12/559	69/19559	5.27E-07	1.99E-05	1.71E-05	5713/5708	12

CC	GO:0005839	proteasome core complex	7/559	20/19559	8.41E-07	2.98E-05	2.56E-05	5695/5691	7
CC	GO:1905368	peptidase complex	13/559	92/19559	2.09E-06	6.96E-05	5.98E-05	5713/5708	13
CC	GO:0000780	condensed nuclear chromosome, centromeric region	8/559	32/19559	2.43E-06	7.64E-05	6.57E-05	5885/5347	8
CC	GO:0000781	chromosome, telomeric region	16/559	164/19559	1.99E-05	0.000592	0.000509	6741/5518	16
CC	GO:0000940	condensed chromosome outer kinetochore	5/559	14/19559	3.03E-05	0.000816	0.000701	54908/220	5
CC	GO:1990023	mitotic spindle midzone	5/559	14/19559	3.03E-05	0.000816	0.000701	9055/1469	5
CC	GO:0032153	cell division site	10/559	72/19559	3.66E-05	0.000942	0.000809	10801/589	10
CC	GO:0000778	condensed nuclear chromosome kinetochore	5/559	17/19559	8.71E-05	0.002145	0.001843	5347/1040	5
CC	GO:0005635	nuclear envelope	29/559	473/19559	0.000105	0.00248	0.002131	7444/1104	29
CC	GO:0032154	cleavage furrow	8/559	54/19559	0.000138	0.003133	0.002692	5898/2912	8
CC	GO:0000794	condensed nuclear chromosome	11/559	103/19559	0.000175	0.003623	0.003113	5885/5810	11
CC	GO:0097431	mitotic spindle pole	6/559	30/19559	0.000176	0.003623	0.003113	10615/848	6
CC	GO:0000235	astral microtubule	4/559	11/19559	0.000186	0.003623	0.003113	22919/146	4
CC	GO:0005818	aster	4/559	11/19559	0.000186	0.003623	0.003113	22919/146	4
CC	GO:0000784	nuclear chromosome, telomeric region	12/559	124/19559	0.00023	0.004335	0.003724	6741/5591	12
CC	GO:0005686	U2 snRNP	5/559	24/19559	0.000507	0.009262	0.007958	6637/6629	5
CC	GO:0005871	kinesin complex	7/559	51/19559	0.000584	0.010324	0.00887	3833/3797	7
CC	GO:0005643	nuclear pore	9/559	85/19559	0.000713	0.012235	0.010512	5902/8480	9
CC	GO:0001650	fibrillar center	11/559	129/19559	0.001199	0.019966	0.017155	6949/6629	11
CC	GO:0005681	spliceosomal complex	14/559	192/19559	0.001288	0.020833	0.0179	7307/6637	14
CC	GO:0005684	U2-type spliceosomal complex	9/559	93/19559	0.001363	0.021433	0.018415	6637/6631	9
CC	GO:0120114	Sm-like protein family complex	10/559	114/19559	0.001598	0.024452	0.02101	96764/663	10
CC	GO:0090543	Flemming body	5/559	31/19559	0.00172	0.025617	0.02201	5898/2912	5
CC	GO:0005875	microtubule associated complex	12/559	159/19559	0.002094	0.03039	0.026111	3833/3797	12
CC	GO:0030532	small nuclear ribonucleoprotein complex	9/559	102/19559	0.002591	0.035308	0.030337	96764/663	9
CC	GO:1990752	microtubule end	5/559	34/19559	0.002625	0.035308	0.030337	10615/229	5
CC	GO:0061695	transferase complex, transferring phosphorus-contai	16/559	253/19559	0.002633	0.035308	0.030337	6873/6882	16
CC	GO:0005838	proteasome regulatory particle	4/559	21/19559	0.002682	0.035308	0.030337	5713/5708	4
CC	GO:0016607	nuclear speck	22/559	401/19559	0.002843	0.036478	0.031343	7307/1091	22
CC	GO:0071005	U2-type precatalytic spliceosome	6/559	50/19559	0.0029	0.036478	0.031343	6637/6629	6
CC	GO:0001725	stress fiber	7/559	68/19559	0.003224	0.038061	0.032702	10801/574	7
CC	GO:0097517	contractile actin filament bundle	7/559	68/19559	0.003224	0.038061	0.032702	10801/574	7
CC	GO:0019774	proteasome core complex, beta-subunit complex	3/559	11/19559	0.003228	0.038061	0.032702	5695/5691	3
CC	GO:0035371	microtubule plus-end	4/559	23/19559	0.003794	0.043355	0.037252	10615/229	4
CC	GO:0071011	precatalytic spliceosome	6/559	53/19559	0.0039	0.043355	0.037252	6637/6629	6
CC	GO:0005657	replication fork	7/559	71/19559	0.004111	0.043355	0.037252	5984/5983	7
CC	GO:0042555	MCM complex	3/559	12/19559	0.004213	0.043355	0.037252	4175/4174	3
CC	GO:0044615	nuclear pore nuclear basket	3/559	12/19559	0.004213	0.043355	0.037252	4085/5694	3

CC	GO:0071564	npBAF complex	3/559	12/19559	0.004213	0.043355	0.037252	86/6595/8	3
CC	GO:0072687	meiotic spindle	3/559	12/19559	0.004213	0.043355	0.037252	26271/798	3
CC	GO:0022624	proteasome accessory complex	4/559	24/19559	0.004451	0.044985	0.038652	5713/5708	4
MF	GO:0015631	tubulin binding	34/547	365/18352	4.59E-09	3.35E-06	3.15E-06	9217/2297	34
MF	GO:0008017	microtubule binding	27/547	265/18352	2.85E-08	1.04E-05	9.77E-06	9217/2297	27
MF	GO:0043021	ribonucleoprotein complex binding	17/547	133/18352	4.54E-07	9.44E-05	8.86E-05	54915/512	17
MF	GO:0016887	ATPase activity	33/547	423/18352	5.17E-07	9.44E-05	8.86E-05	7153/5984	33
MF	GO:0004298	threonine-type endopeptidase activity	7/547	21/18352	1.63E-06	0.000237	0.000223	5695/5691	7
MF	GO:0043138	3'-5' DNA helicase activity	6/547	19/18352	1.33E-05	0.001617	0.001518	9401/4175	6
MF	GO:0140097	catalytic activity, acting on DNA	19/547	209/18352	1.69E-05	0.001762	0.001654	7153/5984	19
MF	GO:0070003	threonine-type peptidase activity	7/547	31/18352	2.84E-05	0.00259	0.002431	5695/5691	7
MF	GO:0003688	DNA replication origin binding	6/547	23/18352	4.47E-05	0.003624	0.003402	4998/4175	6
MF	GO:0008094	DNA-dependent ATPase activity	12/547	108/18352	9.02E-05	0.006584	0.006181	7153/5984	12
MF	GO:0017116	single-stranded DNA helicase activity	5/547	18/18352	0.000143	0.009516	0.008933	5984/5983	5
MF	GO:0004386	helicase activity	14/547	160/18352	0.000317	0.019285	0.018103	5984/5983	14
MF	GO:0045296	cadherin binding	22/547	332/18352	0.000427	0.023486	0.022046	9217/1080	22
MF	GO:1990825	sequence-specific mRNA binding	4/547	13/18352	0.00045	0.023486	0.022046	7298/6741	4
MF	GO:0003678	DNA helicase activity	9/547	78/18352	0.000509	0.024795	0.023275	5984/5983	9
MF	GO:0000900	translation repressor activity, mRNA regulatory eleme	4/547	16/18352	0.001068	0.048716	0.04573	7298/6472	4
MF	GO:0003713	transcription coactivator activity	18/547	267/18352	0.001138	0.048851	0.045857	10915/688	18

Supplementary Table 5. KEGG analysis of genes in the turquoise module

ID	Description	GeneRatio	BgRatio	pvalue	p.adjust	qvalue	geneID	Count
hsa04110	Cell cycle	22/281	124/8108	1.92E-10	5.11E-08	4.77E-08	RBL1/RAD21/PTTG1/PRKDC/PLK1/O	22
hsa03050	Proteasome	13/281	46/8108	2.88E-09	3.83E-07	3.58E-07	SEM1/PSMD7/PSMD2/PSMD12/PSM	13
hsa05014	Amyotrophic lateral sclerosis	30/281	365/8108	8.54E-06	0.000758	0.000708	VAPB/TOMM40/SRSF3/SEM1/SDHA/	30
hsa05017	Spinocerebellar ataxia	15/281	143/8108	0.000121	0.008076	0.007542	SLC25A5/SEM1/PSMD7/PSMD2/PSM	15
hsa05020	Prion disease	22/281	273/8108	0.00019	0.010114	0.009445	SLC25A5/SEM1/SDHA/PSMD7/PSMD	22
hsa05016	Huntington disease	23/281	306/8108	0.000376	0.014764	0.013788	TFAM/SLC25A5/SEM1/SDHA/PSMD7	23
hsa05012	Parkinson disease	20/281	249/8108	0.000389	0.014764	0.013788	TXN/SLC25A5/SEM1/SDHA/PSMD7/	20
hsa04114	Oocyte meiosis	13/281	129/8108	0.000507	0.01686	0.015746	PTTG1/PLK1/MAPK14/MAD2L1/FBX(	13
hsa05010	Alzheimer disease	25/281	369/8108	0.000983	0.029065	0.027145	SLC25A5/SEM1/SDHA/PSMD7/PSMD	25
hsa03040	Spliceosome	13/281	147/8108	0.001721	0.045778	0.042752	U2AF1/TCERG1/SRSF3/SNRPG/SNRP	13

Supplementary Table 6. GO analysis of genes in the blue module									
ONTOLOG	ID	Description	GeneRatio	BgRatio	pvalue	p.adjust	qvalue	geneID	Count
BP	GO:0030198	extracellular matrix organization	27/256	395/18866	5.62E-12	1.12E-08	9.30E-09	7177/7132	27
BP	GO:0043062	extracellular structure organization	27/256	396/18866	5.96E-12	1.12E-08	9.30E-09	7177/7132	27
BP	GO:1903053	regulation of extracellular matrix organizatio	8/256	44/18866	1.20E-07	0.00015	0.000125	7132/7040	8
BP	GO:0007162	negative regulation of cell adhesion	18/256	301/18866	1.68E-07	0.000158	0.000131	7001/384/	18
BP	GO:1903077	negative regulation of protein localization to	6/256	23/18866	4.90E-07	0.000312	0.000259	8500/7040	6
BP	GO:0007178	transmembrane receptor protein serine/thre	19/256	359/18866	4.98E-07	0.000312	0.000259	7314/1045	19
BP	GO:1904376	negative regulation of protein localization to	6/256	25/18866	8.40E-07	0.000452	0.000374	8500/7040	6
BP	GO:0022408	negative regulation of cell-cell adhesion	13/256	191/18866	2.18E-06	0.001027	0.000851	7001/384/	13
BP	GO:0032963	collagen metabolic process	10/256	113/18866	3.24E-06	0.001227	0.001017	265/7040/	10
BP	GO:1905476	negative regulation of protein localization to	6/256	31/18866	3.26E-06	0.001227	0.001017	8500/7040	6
BP	GO:0030111	regulation of Wnt signaling pathway	18/256	375/18866	3.99E-06	0.001364	0.001131	8629/6512	18
BP	GO:0071902	positive regulation of protein serine/threoni	17/256	345/18866	5.26E-06	0.001615	0.001339	7314/7039	17
BP	GO:0090287	regulation of cellular response to growth fac	16/256	310/18866	5.58E-06	0.001615	0.001339	7314/2309	16
BP	GO:1904375	regulation of protein localization to cell peri	10/256	123/18866	6.95E-06	0.001772	0.001469	8500/7040	10
BP	GO:0032964	collagen biosynthetic process	7/256	53/18866	7.06E-06	0.001772	0.001469	265/7040/	7
BP	GO:1905314	semi-lunar valve development	6/256	37/18866	9.62E-06	0.002164	0.001794	7132/7040	6
BP	GO:0060840	artery development	9/256	102/18866	1.03E-05	0.002164	0.001794	10454/646	9
BP	GO:1903076	regulation of protein localization to plasma	9/256	102/18866	1.03E-05	0.002164	0.001794	8500/7040	9
BP	GO:0003007	heart morphogenesis	14/256	258/18866	1.24E-05	0.002419	0.002006	10454/704	14
BP	GO:0003151	outflow tract morphogenesis	8/256	80/18866	1.29E-05	0.002419	0.002006	55742/882	8
BP	GO:0061448	connective tissue development	14/256	262/18866	1.47E-05	0.002632	0.002181	7314/265/	14
BP	GO:0006024	glycosaminoglycan biosynthetic process	9/256	108/18866	1.65E-05	0.00272	0.002254	9956/7040	9
BP	GO:0051216	cartilage development	12/256	197/18866	1.66E-05	0.00272	0.002254	265/6935/	12
BP	GO:1903038	negative regulation of leukocyte cell-cell ad	10/256	137/18866	1.80E-05	0.002822	0.002339	7001/384/	10
BP	GO:0032967	positive regulation of collagen biosynthetic	5/256	26/18866	2.30E-05	0.00347	0.002876	265/7040/	5
BP	GO:0050866	negative regulation of cell activation	12/256	207/18866	2.72E-05	0.003761	0.003117	7001/384/	12
BP	GO:0006023	aminoglycan biosynthetic process	9/256	115/18866	2.73E-05	0.003761	0.003117	9956/7040	9
BP	GO:0010714	positive regulation of collagen metabolic pr	5/256	27/18866	2.80E-05	0.003761	0.003117	265/7040/	5
BP	GO:0060021	roof of mouth development	8/256	91/18866	3.32E-05	0.004199	0.00348	2049/6591	8
BP	GO:0050868	negative regulation of T cell activation	9/256	118/18866	3.35E-05	0.004199	0.00348	7001/384/	9
BP	GO:0045581	negative regulation of T cell differentiation	6/256	46/18866	3.50E-05	0.004248	0.003522	7001/7292	6
BP	GO:0048732	gland development	18/256	443/18866	3.76E-05	0.00442	0.003664	29842/664	18
BP	GO:0060485	mesenchyme development	14/256	290/18866	4.50E-05	0.005134	0.004256	10505/265	14
BP	GO:0090092	regulation of transmembrane receptor prote	13/256	254/18866	4.64E-05	0.005137	0.004258	7314/2309	13
BP	GO:0051250	negative regulation of lymphocyte activatior	10/256	154/18866	4.92E-05	0.005279	0.004376	7001/384/	10
BP	GO:0010712	regulation of collagen metabolic process	6/256	49/18866	5.05E-05	0.005279	0.004376	265/7040/	6

BP	GO:0060828	regulation of canonical Wnt signaling pathw	14/256	295/18866	5.42E-05	0.005509	0.004566	8629/6512	14
BP	GO:0060070	canonical Wnt signaling pathway	15/256	339/18866	6.52E-05	0.006169	0.005114	8629/6512	15
BP	GO:0003176	aortic valve development	5/256	32/18866	6.60E-05	0.006169	0.005114	7132/7040	5
BP	GO:0010566	regulation of ketone biosynthetic process	4/256	17/18866	6.86E-05	0.006169	0.005114	23408/118	4
BP	GO:0010715	regulation of extracellular matrix disassembl	4/256	17/18866	6.86E-05	0.006169	0.005114	7040/4035	4
BP	GO:0043406	positive regulation of MAP kinase activity	13/256	264/18866	6.88E-05	0.006169	0.005114	7314/7039	13
BP	GO:0035924	cellular response to vascular endothelial gro	7/256	75/18866	7.06E-05	0.006179	0.005122	5159/5156	7
BP	GO:0048762	mesenchymal cell differentiation	12/256	229/18866	7.25E-05	0.006202	0.005141	10505/265	12
BP	GO:0001649	osteoblast differentiation	12/256	231/18866	7.88E-05	0.00659	0.005462	265/6591/	12
BP	GO:1902106	negative regulation of leukocyte differentiat	8/256	105/18866	9.24E-05	0.007542	0.006252	7001/7292	8
BP	GO:0045620	negative regulation of lymphocyte differenti	6/256	55/18866	9.78E-05	0.007542	0.006252	7001/7292	6
BP	GO:0014812	muscle cell migration	8/256	106/18866	9.88E-05	0.007542	0.006252	5159/5574	8
BP	GO:0034446	substrate adhesion-dependent cell spreadin	8/256	106/18866	9.88E-05	0.007542	0.006252	2049/5574	8
BP	GO:0071772	response to BMP	10/256	168/18866	0.000102	0.007542	0.006252	23090/704	10
BP	GO:0071773	cellular response to BMP stimulus	10/256	168/18866	0.000102	0.007542	0.006252	23090/704	10
BP	GO:0045063	T-helper 1 cell differentiation	4/256	19/18866	0.000109	0.007764	0.006436	7292/5117	4
BP	GO:0072074	kidney mesenchyme development	4/256	19/18866	0.000109	0.007764	0.006436	6469/5311	4
BP	GO:0031589	cell-substrate adhesion	15/256	359/18866	0.000123	0.008385	0.006951	8500/2049	15
BP	GO:0007568	aging	14/256	319/18866	0.000124	0.008385	0.006951	29842/664	14
BP	GO:0022617	extracellular matrix disassembly	7/256	82/18866	0.000125	0.008385	0.006951	7177/7040	7
BP	GO:0001503	ossification	16/256	412/18866	0.000171	0.01103	0.009143	265/7040/	16
BP	GO:0007431	salivary gland development	5/256	39/18866	0.000175	0.01103	0.009143	29842/118	5
BP	GO:0003170	heart valve development	6/256	61/18866	0.000175	0.01103	0.009143	7132/7040	6
BP	GO:1902105	regulation of leukocyte differentiation	13/256	290/18866	0.000176	0.01103	0.009143	6647/7001	13
BP	GO:0032147	activation of protein kinase activity	14/256	331/18866	0.000183	0.011251	0.009326	7314/7039	14
BP	GO:1903828	negative regulation of cellular protein locali	8/256	116/18866	0.000185	0.011251	0.009326	8500/9555	8
BP	GO:0045619	regulation of lymphocyte differentiation	10/256	181/18866	0.000188	0.011258	0.009332	6647/7001	10
BP	GO:0046636	negative regulation of alpha-beta T cell acti	5/256	40/18866	0.000197	0.011614	0.009627	384/7292/	5
BP	GO:0030177	positive regulation of Wnt signaling pathway	10/256	183/18866	0.000206	0.011915	0.009877	8629/6512	10
BP	GO:0002683	negative regulation of immune system proc	17/256	463/18866	0.000209	0.011915	0.009877	7001/3313	17
BP	GO:0045580	regulation of T cell differentiation	9/256	150/18866	0.000213	0.011917	0.009878	6647/7001	9
BP	GO:0002695	negative regulation of leukocyte activation	10/256	184/18866	0.000215	0.011917	0.009878	7001/384/	10
BP	GO:0043405	regulation of MAP kinase activity	14/256	342/18866	0.000255	0.013921	0.011539	7314/7039	14
BP	GO:0030509	BMP signaling pathway	9/256	155/18866	0.000272	0.014239	0.011803	23090/704	9
BP	GO:0014909	smooth muscle cell migration	7/256	93/18866	0.000274	0.014239	0.011803	5159/5574	7
BP	GO:0030510	regulation of BMP signaling pathway	7/256	93/18866	0.000274	0.014239	0.011803	23090/642	7
BP	GO:0045625	regulation of T-helper 1 cell differentiation	3/256	10/18866	0.000276	0.014239	0.011803	7292/3566	3
BP	GO:0048745	smooth muscle tissue development	4/256	24/18866	0.000284	0.014454	0.011981	6469/5311	4

BP	GO:0032965	regulation of collagen biosynthetic process	5/256	44/18866	0.000312	0.015351	0.012724	265/7040/	5
BP	GO:0030203	glycosaminoglycan metabolic process	9/256	158/18866	0.000314	0.015351	0.012724	9956/7040	9
BP	GO:1903707	negative regulation of hemopoiesis	9/256	158/18866	0.000314	0.015351	0.012724	7001/3313	9
BP	GO:0001667	ameboidal-type cell migration	17/256	481/18866	0.000325	0.015702	0.013015	10505/704	17
BP	GO:0046639	negative regulation of alpha-beta T cell diff	4/256	25/18866	0.000335	0.015947	0.013218	7292/6469	4
BP	GO:0022407	regulation of cell-cell adhesion	16/256	439/18866	0.000347	0.016347	0.01355	7001/2049	16
BP	GO:0045628	regulation of T-helper 2 cell differentiation	3/256	11/18866	0.000376	0.017255	0.014303	7292/3566	3
BP	GO:0090030	regulation of steroid hormone biosynthetic	3/256	11/18866	0.000376	0.017255	0.014303	1181/9563	3
BP	GO:0048608	reproductive structure development	16/256	443/18866	0.000384	0.017406	0.014428	7314/6647	16
BP	GO:0046777	protein autophosphorylation	11/256	237/18866	0.000411	0.018419	0.015268	4296/2285	11
BP	GO:0061458	reproductive system development	16/256	447/18866	0.000423	0.018751	0.015543	7314/6647	16
BP	GO:0003148	outflow tract septum morphogenesis	4/256	27/18866	0.000455	0.019666	0.016301	55742/882	4
BP	GO:0033081	regulation of T cell differentiation in thymus	4/256	27/18866	0.000455	0.019666	0.016301	6647/6935	4
BP	GO:0003180	aortic valve morphogenesis	4/256	28/18866	0.000525	0.021944	0.01819	7040/6591	4
BP	GO:0061437	renal system vasculature development	4/256	28/18866	0.000525	0.021944	0.01819	5311/5159	4
BP	GO:0061440	kidney vasculature development	4/256	28/18866	0.000525	0.021944	0.01819	5311/5159	4
BP	GO:0048844	artery morphogenesis	6/256	75/18866	0.000542	0.022421	0.018584	5396/5311	6
BP	GO:0010718	positive regulation of epithelial to mesenchy	5/256	50/18866	0.000569	0.0233	0.019313	7040/2898	5
BP	GO:0006022	aminoglycan metabolic process	9/256	172/18866	0.000584	0.023619	0.019578	9956/7040	9
BP	GO:0018108	peptidyl-tyrosine phosphorylation	14/256	374/18866	0.000623	0.023985	0.019881	7039/2285	14
BP	GO:0060411	cardiac septum morphogenesis	6/256	77/18866	0.000624	0.023985	0.019881	55742/882	6
BP	GO:0035272	exocrine system development	5/256	51/18866	0.000624	0.023985	0.019881	29842/118	5
BP	GO:0038084	vascular endothelial growth factor signaling	5/256	51/18866	0.000624	0.023985	0.019881	5159/5156	5
BP	GO:0043277	apoptotic cell clearance	5/256	51/18866	0.000624	0.023985	0.019881	4035/3685	5
BP	GO:0034309	primary alcohol biosynthetic process	3/256	13/18866	0.000639	0.02428	0.020126	1181/9563	3
BP	GO:0018212	peptidyl-tyrosine modification	14/256	377/18866	0.000674	0.02536	0.021021	7039/2285	14
BP	GO:0003179	heart valve morphogenesis	5/256	52/18866	0.000683	0.025467	0.02111	7040/6591	5
BP	GO:0007492	endoderm development	6/256	79/18866	0.000715	0.026141	0.021668	9555/3915	6
BP	GO:0010827	regulation of glucose transmembrane transp	6/256	79/18866	0.000715	0.026141	0.021668	2171/2343	6
BP	GO:0045661	regulation of myoblast differentiation	5/256	53/18866	0.000746	0.02701	0.022389	55662/704	5
BP	GO:0014032	neural crest cell development	6/256	80/18866	0.000765	0.02722	0.022563	10505/659	6
BP	GO:0002828	regulation of type 2 immune response	4/256	31/18866	0.000781	0.02722	0.022563	384/7292/	4
BP	GO:0061037	negative regulation of cartilage developmer	4/256	31/18866	0.000781	0.02722	0.022563	6591/1513	4
BP	GO:2000515	negative regulation of CD4-positive, alpha-	4/256	31/18866	0.000781	0.02722	0.022563	384/7292/	4
BP	GO:0035791	platelet-derived growth factor receptor-bet	3/256	14/18866	0.000805	0.027533	0.022822	5157/5159	3
BP	GO:1903054	negative regulation of extracellular matrix o	3/256	14/18866	0.000805	0.027533	0.022822	7132/7040	3
BP	GO:0007596	blood coagulation	13/256	343/18866	0.000865	0.029316	0.0243	7001/6469	13
BP	GO:0030199	collagen fibril organization	5/256	55/18866	0.000885	0.029743	0.024654	4060/4016	5

BP	GO:1903034	regulation of response to wounding	9/256	183/18866	0.000908	0.030261	0.025083	7001/6558	9
BP	GO:0001570	vasculogenesis	6/256	83/18866	0.000929	0.030447	0.025238	55662/704	6
BP	GO:0003279	cardiac septum development	7/256	114/18866	0.000936	0.030447	0.025238	10454/557	7
BP	GO:0051051	negative regulation of transport	16/256	483/18866	0.000969	0.030447	0.025238	28978/404	16
BP	GO:0007599	hemostasis	13/256	348/18866	0.000986	0.030447	0.025238	7001/6469	13
BP	GO:0014031	mesenchymal cell development	6/256	84/18866	0.000989	0.030447	0.025238	10505/659	6
BP	GO:0048864	stem cell development	6/256	84/18866	0.000989	0.030447	0.025238	10505/659	6
BP	GO:0010464	regulation of mesenchymal cell proliferation	4/256	33/18866	0.000994	0.030447	0.025238	6935/6469	4
BP	GO:0045064	T-helper 2 cell differentiation	3/256	15/18866	0.000996	0.030447	0.025238	7292/3566	3
BP	GO:0060391	positive regulation of SMAD protein signal t	3/256	15/18866	0.000996	0.030447	0.025238	7040/1601	3
BP	GO:0072075	metanephric mesenchyme development	3/256	15/18866	0.000996	0.030447	0.025238	6469/5311	3
BP	GO:0048863	stem cell differentiation	11/256	264/18866	0.001003	0.030447	0.025238	79918/105	11
BP	GO:0050817	coagulation	13/256	349/18866	0.001013	0.030489	0.025272	7001/6469	13
BP	GO:0061041	regulation of wound healing	8/256	151/18866	0.00108	0.032257	0.026738	7001/6558	8
BP	GO:0040013	negative regulation of locomotion	14/256	397/18866	0.001108	0.032374	0.026835	10505/704	14
BP	GO:0043276	anoikis	4/256	34/18866	0.001115	0.032374	0.026835	23604/659	4
BP	GO:0014910	regulation of smooth muscle cell migration	6/256	86/18866	0.001118	0.032374	0.026835	5159/4035	6
BP	GO:0045445	myoblast differentiation	6/256	86/18866	0.001118	0.032374	0.026835	55662/704	6
BP	GO:0001837	epithelial to mesenchymal transition	8/256	152/18866	0.001127	0.032374	0.026835	265/7040/	8
BP	GO:0051271	negative regulation of cellular component m	14/256	400/18866	0.00119	0.033925	0.028121	10505/704	14
BP	GO:1901201	regulation of extracellular matrix assembly	3/256	16/18866	0.001213	0.034239	0.028381	7040/2898	3
BP	GO:0048008	platelet-derived growth factor receptor sign	5/256	59/18866	0.001219	0.034239	0.028381	5157/5159	5
BP	GO:0014068	positive regulation of phosphatidylinositol 3	6/256	88/18866	0.00126	0.035145	0.029132	79899/556	6
BP	GO:0035904	aorta development	5/256	60/18866	0.001315	0.0364	0.030173	10454/531	5
BP	GO:0014033	neural crest cell differentiation	6/256	89/18866	0.001337	0.036523	0.030274	10505/659	6
BP	GO:0070997	neuron death	13/256	360/18866	0.001339	0.036523	0.030274	7314/6647	13
BP	GO:0071634	regulation of transforming growth factor be	4/256	36/18866	0.001387	0.037449	0.031042	4060/2615	4
BP	GO:0050673	epithelial cell proliferation	15/256	453/18866	0.001393	0.037449	0.031042	7039/7040	15
BP	GO:0042098	T cell proliferation	9/256	195/18866	0.001417	0.037551	0.031126	7001/384/	9
BP	GO:1905475	regulation of protein localization to membra	9/256	195/18866	0.001417	0.037551	0.031126	8500/7040	9
BP	GO:0045623	negative regulation of T-helper cell differen	3/256	17/18866	0.001459	0.038071	0.031558	7292/3566	3
BP	GO:2000811	negative regulation of anoikis	3/256	17/18866	0.001459	0.038071	0.031558	6591/4170	3
BP	GO:0060537	muscle tissue development	14/256	409/18866	0.001467	0.038071	0.031558	140465/70	14
BP	GO:0007159	leukocyte cell-cell adhesion	13/256	364/18866	0.001478	0.038096	0.031578	7001/384/	13
BP	GO:0042475	odontogenesis of dentin-containing tooth	6/256	91/18866	0.001499	0.038384	0.031817	265/6469/	6
BP	GO:2000146	negative regulation of cell motility	13/256	365/18866	0.001514	0.038508	0.03192	7040/6469	13
BP	GO:0060428	lung epithelium development	4/256	37/18866	0.001538	0.038859	0.03221	6469/5733	4
BP	GO:0035270	endocrine system development	7/256	125/18866	0.0016	0.040149	0.033279	6469/5159	7

BP	GO:0072659	protein localization to plasma membrane	11/256	281/18866	0.001652	0.041176	0.034131	8500/7132	11
BP	GO:0055007	cardiac muscle cell differentiation	7/256	126/18866	0.001675	0.041223	0.03417	7040/5159	7
BP	GO:0031032	actomyosin structure organization	9/256	200/18866	0.001687	0.041223	0.03417	8500/7791	9
BP	GO:2000377	regulation of reactive oxygen species metab	9/256	200/18866	0.001687	0.041223	0.03417	6647/2340	9
BP	GO:0071604	transforming growth factor beta production	4/256	38/18866	0.001701	0.041309	0.034241	4060/2615	4
BP	GO:0046885	regulation of hormone biosynthetic process	3/256	18/18866	0.001733	0.041811	0.034658	1181/9563	3
BP	GO:0014066	regulation of phosphatidylinositol 3-kinase s	7/256	127/18866	0.001753	0.041864	0.034701	5270/7989	7
BP	GO:0042130	negative regulation of T cell proliferation	5/256	64/18866	0.001757	0.041864	0.034701	384/6469/	5
BP	GO:0045778	positive regulation of ossification	6/256	94/18866	0.001771	0.041915	0.034743	7040/9260	6
BP	GO:0007179	transforming growth factor beta receptor sig	9/256	202/18866	0.001806	0.042474	0.035207	7314/1045	9
BP	GO:1903037	regulation of leukocyte cell-cell adhesion	12/256	329/18866	0.00187	0.043577	0.036121	7001/384/	12
BP	GO:0042092	type 2 immune response	4/256	39/18866	0.001876	0.043577	0.036121	384/7292/	4
BP	GO:0002576	platelet degranulation	7/256	129/18866	0.001916	0.043785	0.036294	6647/7078	7
BP	GO:0001655	urogenital system development	12/256	330/18866	0.001918	0.043785	0.036294	2049/384/	12
BP	GO:0060348	bone development	9/256	204/18866	0.001931	0.043785	0.036294	6424/5603	9
BP	GO:0060541	respiratory system development	9/256	204/18866	0.001931	0.043785	0.036294	10454/646	9
BP	GO:0045669	positive regulation of osteoblast differentiat	5/256	66/18866	0.002015	0.044584	0.036956	9260/3611	5
BP	GO:0050863	regulation of T cell activation	12/256	332/18866	0.002016	0.044584	0.036956	6647/7001	12
BP	GO:0055003	cardiac myofibril assembly	3/256	19/18866	0.002037	0.044584	0.036956	5159/5156	3
BP	GO:0060438	trachea development	3/256	19/18866	0.002037	0.044584	0.036956	6469/5117	3
BP	GO:0060973	cell migration involved in heart developmen	3/256	19/18866	0.002037	0.044584	0.036956	6591/5159	3
BP	GO:0150146	cell junction disassembly	3/256	19/18866	0.002037	0.044584	0.036956	6591/9448	3
BP	GO:0072210	metanephric nephron development	4/256	40/18866	0.002062	0.044863	0.037187	5311/5159	4
BP	GO:0003206	cardiac chamber morphogenesis	7/256	131/18866	0.002091	0.045241	0.0375	7040/5574	7
BP	GO:0046890	regulation of lipid biosynthetic process	9/256	207/18866	0.002132	0.045862	0.038015	6647/2171	9
BP	GO:0034614	cellular response to reactive oxygen species	8/256	170/18866	0.002287	0.048911	0.040543	6647/7001	8
CC	GO:0062023	collagen-containing extracellular matrix	28/263	427/19559	4.99E-12	1.96E-09	1.81E-09	265/64499	28
CC	GO:0005925	focal adhesion	21/263	415/19559	2.26E-07	4.05E-05	3.74E-05	8500/3313	21
CC	GO:0030055	cell-substrate junction	21/263	423/19559	3.09E-07	4.05E-05	3.74E-05	8500/3313	21
CC	GO:0005788	endoplasmic reticulum lumen	14/263	308/19559	7.85E-05	0.007711	0.007125	4043/265/	14
CC	GO:0005775	vacuolar lumen	10/263	173/19559	0.000121	0.009537	0.008813	2992/2171	10
CC	GO:0005884	actin filament	8/263	118/19559	0.000197	0.012871	0.011894	7456/6525	8
CC	GO:0043292	contractile fiber	11/263	238/19559	0.000396	0.022237	0.020549	140465/23	11
CC	GO:0031252	cell leading edge	15/263	421/19559	0.000613	0.030095	0.02781	50855/644	15
CC	GO:0001726	ruffle	9/263	179/19559	0.00073	0.031883	0.029462	50855/644	9
CC	GO:0030016	myofibril	10/263	227/19559	0.001042	0.038544	0.035618	23345/887	10
CC	GO:0031941	filamentous actin	4/263	34/19559	0.001079	0.038544	0.035618	6525/5311	4
CC	GO:0031091	platelet alpha granule	6/263	91/19559	0.001433	0.046939	0.043375	7040/710/	6

MF	GO:0005201	extracellular matrix structural constituent	14/252	169/18352	9.13E-08	5.14E-05	4.85E-05	265/10418	14
MF	GO:1901681	sulfur compound binding	15/252	262/18352	3.61E-06	0.000961	0.000908	80339/404	15
MF	GO:0005518	collagen binding	8/252	70/18352	5.12E-06	0.000961	0.000908	5118/4060	8
MF	GO:0004714	transmembrane receptor protein tyrosine ki	7/252	61/18352	1.96E-05	0.002291	0.002163	2049/5157	7
MF	GO:0005539	glycosaminoglycan binding	13/252	232/18352	2.03E-05	0.002291	0.002163	4043/6469	13
MF	GO:0001968	fibronectin binding	5/252	27/18352	2.96E-05	0.002776	0.002621	131578/36	5
MF	GO:0005161	platelet-derived growth factor receptor binc	4/252	15/18352	4.21E-05	0.003383	0.003194	5159/5156	4
MF	GO:0019199	transmembrane receptor protein kinase acti	7/252	80/18352	0.000115	0.007979	0.007533	2049/5157	7
MF	GO:0050840	extracellular matrix binding	6/252	57/18352	0.000128	0.007979	0.007533	6469/1315	6
MF	GO:0097493	structural molecule activity conferring elastic	3/252	11/18352	0.000389	0.021906	0.020684	3915/2199	3
MF	GO:0008201	heparin binding	9/252	169/18352	0.000558	0.026249	0.024784	4043/5270	9
MF	GO:0004713	protein tyrosine kinase activity	8/252	135/18352	0.000559	0.026249	0.024784	2049/8445	8

Supplementary Table 7. GO analysis of genes in the brown module									
ONTOLOG	ID	Description	GeneRatio	BgRatio	pvalue	p.adjust	qvalue	geneID	Count
BP	GO:0009266	response to temperature stimulus	13/240	233/18866	9.50E-06	0.01781	0.016203	5931/1001	13
BP	GO:0070493	thrombin-activated receptor signaling path	4/240	12/18866	1.17E-05	0.01781	0.016203	4233/3248	4
BP	GO:0045765	regulation of angiogenesis	17/240	403/18866	1.71E-05	0.01781	0.016203	5170/1001	17
BP	GO:0090132	epithelium migration	16/240	368/18866	2.10E-05	0.01781	0.016203	5170/1001	16
BP	GO:0090130	tissue migration	16/240	374/18866	2.56E-05	0.01781	0.016203	5170/1001	16
BP	GO:0019935	cyclic-nucleotide-mediated signaling	12/240	224/18866	3.14E-05	0.018211	0.016568	2835/997/	12
BP	GO:0007565	female pregnancy	11/240	196/18866	4.40E-05	0.019558	0.017794	6573/5671	11
BP	GO:0007007	inner mitochondrial membrane organizatio	6/240	52/18866	4.97E-05	0.019558	0.017794	8834/2651	6
BP	GO:0019933	cAMP-mediated signaling	11/240	199/18866	5.05E-05	0.019558	0.017794	2835/997/	11
BP	GO:1901342	regulation of vasculature development	17/240	444/18866	5.76E-05	0.020086	0.018273	5170/1001	17
BP	GO:0010631	epithelial cell migration	15/240	365/18866	7.21E-05	0.022849	0.020787	5170/1001	15
BP	GO:0010634	positive regulation of epithelial cell migrati	10/240	176/18866	8.83E-05	0.025637	0.023324	5170/1001	10
BP	GO:0043931	ossification involved in bone maturation	4/240	20/18866	0.000105	0.028078	0.025545	3952/387/	4
BP	GO:0007189	adenylate cyclase-activating G protein-cou	9/240	147/18866	0.000113	0.028078	0.025545	2835/5744	9
BP	GO:0010632	regulation of epithelial cell migration	13/240	301/18866	0.000134	0.031152	0.028341	5170/1001	13
BP	GO:0001667	ameboidal-type cell migration	17/240	481/18866	0.000152	0.031371	0.028541	5170/1001	17
BP	GO:0044706	multi-multicellular organism process	11/240	226/18866	0.000157	0.031371	0.028541	6573/5671	11
BP	GO:0098760	response to interleukin-7	5/240	42/18866	0.000185	0.031371	0.028541	8968/8358	5
BP	GO:0098761	cellular response to interleukin-7	5/240	42/18866	0.000185	0.031371	0.028541	8968/8358	5
BP	GO:0070977	bone maturation	4/240	23/18866	0.000187	0.031371	0.028541	3952/387/	4
BP	GO:0016049	cell growth	17/240	490/18866	0.000189	0.031371	0.028541	5931/1020	17
BP	GO:0019932	second-messenger-mediated signaling	16/240	456/18866	0.000257	0.037435	0.034057	5170/2835	16
BP	GO:0098727	maintenance of cell number	9/240	164/18866	0.000257	0.037435	0.034057	23160/694	9
BP	GO:0051204	protein insertion into mitochondrial memb	5/240	45/18866	0.000258	0.037435	0.034057	26517/265	5
BP	GO:0009408	response to heat	9/240	166/18866	0.000282	0.039259	0.035716	5931/1001	9
BP	GO:0043542	endothelial cell migration	12/240	286/18866	0.000315	0.040924	0.037231	5170/1001	12
BP	GO:0046677	response to antibiotic	5/240	47/18866	0.000317	0.040924	0.037231	51319/332	5
BP	GO:0001558	regulation of cell growth	15/240	420/18866	0.000332	0.041338	0.037607	5931/1020	15
BP	GO:0140056	organelle localization by membrane tetheri	9/240	171/18866	0.000351	0.041367	0.037634	6809/9342	9
BP	GO:0048799	animal organ maturation	4/240	27/18866	0.000356	0.041367	0.037634	3952/387/	4
BP	GO:0090151	establishment of protein localization to mit	5/240	49/18866	0.000386	0.043405	0.039488	26517/265	5
BP	GO:0071560	cellular response to transforming growth fa	11/240	252/18866	0.0004	0.04359	0.039656	5170/1075	11
CC	GO:0005901	caveola	8/247	82/19559	9.20E-06	0.003579	0.003264	7048/7035	8
CC	GO:0044853	plasma membrane raft	8/247	113/19559	9.44E-05	0.018368	0.01675	7048/7035	8
CC	GO:0045121	membrane raft	13/247	329/19559	0.000299	0.02975	0.027129	8772/7048	13
CC	GO:0098857	membrane microdomain	13/247	330/19559	0.000308	0.02975	0.027129	8772/7048	13

CC	GO:0098589	membrane region	13/247	343/19559	0.000444	0.02975	0.027129	8772/7048	13
CC	GO:0000792	heterochromatin	6/247	78/19559	0.000459	0.02975	0.027129	79968/420	6

Supplementary Table 8. GO analysis of genes in the yellow module									
ONTOLOG	ID	Description	GeneRatio	BgRatio	pvalue	p.adjust	qvalue	geneID	Count
BP	GO:0002429	immune response-activating cell surface rece	18/87	481/18866	5.74E-12	5.22E-09	4.65E-09	2359/784/	18
BP	GO:0002757	immune response-activating signal transduct	18/87	481/18866	5.74E-12	5.22E-09	4.65E-09	2359/784/	18
BP	GO:0050851	antigen receptor-mediated signaling pathway	15/87	325/18866	2.18E-11	1.32E-08	1.17E-08	784/7535/	15
BP	GO:0050852	T cell receptor signaling pathway	10/87	206/18866	3.73E-08	1.69E-05	1.51E-05	784/7535/	10
BP	GO:0042113	B cell activation	10/87	328/18866	2.69E-06	0.000904	0.000804	7535/7099	10
BP	GO:0050853	B cell receptor signaling pathway	7/87	135/18866	2.98E-06	0.000904	0.000804	23180/507	7
BP	GO:0030316	osteoclast differentiation	6/87	98/18866	6.05E-06	0.001572	0.001399	11025/123	6
BP	GO:0030098	lymphocyte differentiation	10/87	368/18866	7.44E-06	0.001575	0.001402	7535/6421	10
BP	GO:0002460	adaptive immune response based on somatic	10/87	370/18866	7.80E-06	0.001575	0.001402	7099/6421	10
BP	GO:0045348	positive regulation of MHC class II biosynthet	3/87	10/18866	1.11E-05	0.002019	0.001797	7099/3717	3
BP	GO:0070661	leukocyte proliferation	9/87	313/18866	1.40E-05	0.002169	0.001931	6355/7535	9
BP	GO:0045670	regulation of osteoclast differentiation	5/87	67/18866	1.43E-05	0.002169	0.001931	11025/123	5
BP	GO:0002285	lymphocyte activation involved in immune res	7/87	187/18866	2.52E-05	0.003518	0.003131	7099/6421	7
BP	GO:0030183	B cell differentiation	6/87	137/18866	4.10E-05	0.005018	0.004466	931/4242/	6
BP	GO:0045346	regulation of MHC class II biosynthetic proces	3/87	15/18866	4.14E-05	0.005018	0.004466	7099/3717	3
BP	GO:0002449	lymphocyte mediated immunity	9/87	366/18866	4.78E-05	0.005426	0.004829	23180/382	9
BP	GO:0045342	MHC class II biosynthetic process	3/87	16/18866	5.08E-05	0.005432	0.004834	7099/3717	3
BP	GO:1902105	regulation of leukocyte differentiation	8/87	290/18866	5.78E-05	0.005839	0.005196	11025/123	8
BP	GO:0042100	B cell proliferation	5/87	97/18866	8.57E-05	0.008204	0.007301	7099/931/	5
BP	GO:0019882	antigen processing and presentation	7/87	232/18866	9.85E-05	0.008953	0.007967	23180/311	7
BP	GO:0006968	cellular defense response	4/87	54/18866	0.000112	0.009455	0.008414	7707/2364	4
BP	GO:0007204	positive regulation of cytosolic calcium ion co	8/87	322/18866	0.00012	0.009455	0.008414	8698/2359	8
BP	GO:0070663	regulation of leukocyte proliferation	7/87	240/18866	0.000122	0.009455	0.008414	6355/7535	7
BP	GO:1902106	negative regulation of leukocyte differentiatio	5/87	105/18866	0.000125	0.009455	0.008414	11025/289	5
BP	GO:0030099	myeloid cell differentiation	9/87	431/18866	0.000166	0.012043	0.010717	11025/123	9
BP	GO:0071346	cellular response to interferon-gamma	6/87	182/18866	0.000196	0.013721	0.012211	6355/7099	6
BP	GO:0051480	regulation of cytosolic calcium ion concentrat	8/87	357/18866	0.000242	0.016294	0.014501	8698/2359	8
BP	GO:0006874	cellular calcium ion homeostasis	9/87	456/18866	0.000252	0.016343	0.014545	8698/2359	9
BP	GO:0002761	regulation of myeloid leukocyte differentiatio	5/87	123/18866	0.000262	0.016409	0.014603	11025/123	5
BP	GO:0002335	mature B cell differentiation	3/87	28/18866	0.000285	0.0173	0.015396	4242/5561	3
BP	GO:0055074	calcium ion homeostasis	9/87	468/18866	0.000305	0.017869	0.015902	8698/2359	9
BP	GO:0045671	negative regulation of osteoclast differentiatio	3/87	29/18866	0.000317	0.01803	0.016045	11025/709	3
BP	GO:0046651	lymphocyte proliferation	7/87	283/18866	0.000333	0.018356	0.016336	7535/7099	7
BP	GO:0034341	response to interferon-gamma	6/87	202/18866	0.000344	0.018381	0.016358	6355/7099	6
BP	GO:0032943	mononuclear cell proliferation	7/87	286/18866	0.000355	0.018442	0.016412	7535/7099	7
BP	GO:0002573	myeloid leukocyte differentiation	6/87	210/18866	0.000423	0.021344	0.018995	11025/123	6

BP	GO:0072503	cellular divalent inorganic cation homeostasis	9/87	492/18866	0.000439	0.02155	0.019178	8698/2359	9
BP	GO:0002312	B cell activation involved in immune response	4/87	80/18866	0.000511	0.02444	0.02175	7099/4242	4
BP	GO:0002831	regulation of response to biotic stimulus	8/87	409/18866	0.000598	0.027868	0.024801	10133/511	8
BP	GO:0046777	protein autophosphorylation	6/87	237/18866	0.000798	0.035958	0.032	7535/9770	6
BP	GO:1903707	negative regulation of hemopoiesis	5/87	158/18866	0.000822	0.035958	0.032	11025/289	5
BP	GO:0060333	interferon-gamma-mediated signaling pathw	4/87	91/18866	0.000831	0.035958	0.032	3717/3119	4
BP	GO:0071674	mononuclear cell migration	4/87	97/18866	0.001055	0.044588	0.03968	7941/1230	4
CC	GO:0009897	external side of plasma membrane	10/93	417/19559	2.91E-05	0.005126	0.004516	1230/1666	10
CC	GO:0042613	MHC class II protein complex	3/93	16/19559	5.57E-05	0.005126	0.004516	3119/3113	3
CC	GO:0045121	membrane raft	8/93	329/19559	0.000173	0.007054	0.006214	7535/2318	8
CC	GO:0098857	membrane microdomain	8/93	330/19559	0.000177	0.007054	0.006214	7535/2318	8
CC	GO:0042611	MHC protein complex	3/93	25/19559	0.000222	0.007054	0.006214	3119/3113	3
CC	GO:0098589	membrane region	8/93	343/19559	0.00023	0.007054	0.006214	7535/2318	8

Supplementary Table 9. KEGG analysis of genes in the yellow module

ID	Description	GeneRatio	BgRatio	pvalue	p.adjust	qvalue	geneID	Count
hsa04640	Hematopoietic cell lineage	8/53	99/8108	2.09E-07	3.14E-05	2.58E-05	MS4A1/HLA-DQB1/H	8
hsa05145	Toxoplasmosis	8/53	112/8108	5.45E-07	4.09E-05	3.36E-05	TLR4/LY96/JAK2/HLA	8
hsa04612	Antigen processing and presentation	6/53	78/8108	1.05E-05	0.000523	0.000429	KLRD1/KLRC3/HLA-D	6
hsa04064	NF-kappa B signaling pathway	6/53	104/8108	5.43E-05	0.001592	0.001307	ZAP70/TRAF1/TLR4/L	6
hsa05321	Inflammatory bowel disease	5/53	65/8108	6.04E-05	0.001592	0.001307	TLR4/IL21R/HLA-DQE	5
hsa04659	Th17 cell differentiation	6/53	107/8108	6.37E-05	0.001592	0.001307	ZAP70/JAK2/IL21R/HI	6
hsa05140	Leishmaniasis	5/53	77/8108	0.000136	0.002842	0.002333	TLR4/JAK2/HLA-DQB	5
hsa05332	Graft-versus-host disease	4/53	42/8108	0.000152	0.002842	0.002333	KLRD1/HLA-DQB1/H	4
hsa04658	Th1 and Th2 cell differentiation	5/53	92/8108	0.000315	0.004967	0.004078	ZAP70/JAK2/HLA-DQ	5
hsa05323	Rheumatoid arthritis	5/53	93/8108	0.000331	0.004967	0.004078	TLR4/LTB/HLA-DQB1	5
hsa05164	Influenza A	6/53	172/8108	0.000839	0.011444	0.009396	TLR4/JAK2/HLA-DQB	6
hsa05310	Asthma	3/53	31/8108	0.001042	0.012272	0.010076	HLA-DQB1/HLA-DPA	3
hsa05152	Tuberculosis	6/53	180/8108	0.001064	0.012272	0.010076	TLR4/JAK2/HLA-DQB	6
hsa05330	Allograft rejection	3/53	38/8108	0.001893	0.018931	0.015543	HLA-DQB1/HLA-DPA	3
hsa05340	Primary immunodeficiency	3/53	38/8108	0.001893	0.018931	0.015543	ZAP70/CIITA/CD19	3
hsa04940	Type I diabetes mellitus	3/53	43/8108	0.002706	0.025373	0.020833	HLA-DQB1/HLA-DPA	3
hsa05150	Staphylococcus aureus infection	4/53	96/8108	0.003468	0.030602	0.025126	FPR3/HLA-DQB1/HLA	4
hsa04672	Intestinal immune network for IgA production	3/53	49/8108	0.003931	0.032755	0.026894	HLA-DQB1/HLA-DPA	3
hsa05320	Autoimmune thyroid disease	3/53	53/8108	0.004907	0.038739	0.031806	HLA-DQB1/HLA-DPA	3
